# The Complete Genome Sequence and Analysis of the Epsilonproteobacterium *Arcobacter butzleri*

**DOI:** 10.1371/journal.pone.0001358

**Published:** 2007-12-26

**Authors:** William G. Miller, Craig T. Parker, Marc Rubenfield, George L. Mendz, Marc M. S. M. Wösten, David W. Ussery, John F. Stolz, Tim T. Binnewies, Peter F. Hallin, Guilin Wang, Joel A. Malek, Andrea Rogosin, Larry H. Stanker, Robert E. Mandrell

**Affiliations:** 1 Produce Safety and Microbiology Research Unit, Agricultural Research Service, U.S. Department of Agriculture, Albany, California, United States of America; 2 Agencourt Bioscience Corporation, Beverly, Massachusetts, United States of America; 3 School of Medicine, Sydney, The University of Notre Dame Australia, Broadway, New South Wales, Australia; 4 Department of Infectious Diseases and Immunology, Utrecht University, Utrecht, The Netherlands; 5 Center for Biological Sequence Analysis, Technical University of Denmark, Lyngby, Denmark; 6 Department of Biological Sciences, Duquesne University, Pittsburgh, Pennsylvania, United States of America; 7 Foodborne Contaminants Research Unit, Agricultural Research Service, U.S. Department of Agriculture, Albany, California, United States of America; Pasteur Institute, France

## Abstract

**Background:**

*Arcobacter butzleri* is a member of the epsilon subdivision of the Proteobacteria and a close taxonomic relative of established pathogens, such as *Campylobacter jejuni* and *Helicobacter pylori*. Here we present the complete genome sequence of the human clinical isolate, *A. butzleri* strain RM4018.

**Methodology/Principal Findings:**

*Arcobacter butzleri* is a member of the *Campylobacteraceae*, but the majority of its proteome is most similar to those of *Sulfuromonas denitrificans* and *Wolinella succinogenes*, both members of the *Helicobacteraceae*, and those of the deep-sea vent Epsilonproteobacteria *Sulfurovum* and *Nitratiruptor*. In addition, many of the genes and pathways described here, e.g. those involved in signal transduction and sulfur metabolism, have been identified previously within the epsilon subdivision only in *S. denitrificans*, *W. succinogenes*, *Sulfurovum*, and/or *Nitratiruptor*, or are unique to the subdivision. In addition, the analyses indicated also that a substantial proportion of the *A. butzleri* genome is devoted to growth and survival under diverse environmental conditions, with a large number of respiration-associated proteins, signal transduction and chemotaxis proteins and proteins involved in DNA repair and adaptation. To investigate the genomic diversity of *A. butzleri* strains, we constructed an *A. butzleri* DNA microarray comprising 2238 genes from strain RM4018. Comparative genomic indexing analysis of 12 additional *A. butzleri* strains identified both the core genes of *A. butzleri* and intraspecies hypervariable regions, where <70% of the genes were present in at least two strains.

**Conclusion/Significance:**

The presence of pathways and loci associated often with non-host-associated organisms, as well as genes associated with virulence, suggests that *A. butzleri* is a free-living, water-borne organism that might be classified rightfully as an emerging pathogen. The genome sequence and analyses presented in this study are an important first step in understanding the physiology and genetics of this organism, which constitutes a bridge between the environment and mammalian hosts.

## Introduction

The epsilon subdivision of the Gram-negative Proteobacteria comprises multiple genera contained within three major families: *Campylobacteraceae*, *Helicobacteraceae* and *Nautiliaceae*. The majority of well-characterized species in this subdivision are members of genera within the first two families, including *Campylobacter*, *Arcobacter* and *Sulfurospirillum* in the *Campylobacteraceae*, and *Helicobacter* and *Wolinella* in the *Helicobacteraceae*. Many of these species are pathogenic, e.g. *Campylobacter jejuni* [1] and *Helicobacter pylori* [2], and/or are associated with a particular host or hosts, e.g. *Campylobacter upsaliensis* [3] and *Helicobacter mustelae* [4]; however, several species are free-living, e.g. *Sulfurospirillum* spp. [5], and they are not considered to be pathogenic.

The genus *Arcobacter* is an unusual taxon within the epsilon subdivision in that it contains both pathogenic and free-living species found in a wide range of environments. Currently, *Arcobacter* contains four recognized species: *A. butzleri* [6], *A. cryaerophilus* [7], *A. skirrowii* [8] and *A. nitrofigilis* [9]. *A. butzleri*, *A. cryaerophilus* and *A. skirrowii* have been isolated from animals and humans [10], while *A. nitrofigilis* is a nitrogen-fixing bacterium isolated originally from *Spartina aterniflora* roots in an estuarine marsh [9]. In addition to these established *Arcobacter* species, three new species have been described recently: 1) the obligate halophile *A. halophilus* sp. nov., isolated from a Hawaiian hypersaline lagoon [11], 2) *A. cibarius* sp. nov., isolated from broiler carcasses [12] and 3) *Candidatus A. sulfidicus*, a sulfide-oxidizing marine organism that produces filamentous sulfur [13]. In addition, several potential, novel *Arcobacter* species, based so far on only 16S rDNA sequence data, have been identified in: the flora of deep-sea hydrothermal vents [14], hydrocarbon-contaminated seawater [15], a low-salinity petroleum reservoir [16], infected or dead coral surfaces [17], deep-sea sediments [18], tube worms [19], anaerobic sludge [20], and a circulated dairy wastewater lagoon [21]. These studies demonstrate clearly that the genus is associated strongly with fresh-water and marine environments. In fact, although *A. butzleri*, *A. cryaerophilus* and *A. skirrowii* have been isolated often from animals or food sources, they have been isolated frequently also from water or water systems [22]–[30].

*Arcobacter butzleri* is the best characterized of all Arcobacters. *A. butzleri* cells are small, spiral and motile [10], similar morphologically to the taxonomically–related *Campylobacter*. Nonetheless, notable differences exist between *A. butzleri* and *Campylobacter* spp. Classified initially as an “aerotolerant *Campylobacter*”, along with *A. cryaerophilus* [10], *A. butzleri* is able to grow aerobically, at variance with most Campylobacters which are microaerophilic. However, *A. butzleri* grows also under microaerobic and anaerobic conditions [10]; thus, this bacterium can grow at all oxygen concentrations. Additionally, *Campylobacter* spp. grow generally between 37°C and 42°C [31], whereas *A. butzleri* is more psychrophilic with a temperature range between 15°C and 37°C, although some strains can grow at 42°C [10]. Furthermore, *A. butzleri* is more halotolerant than most *Campylobacter* spp., with some strains able to grow at 3.5% NaCl [10].

While *A. butzleri* is isolated often from aqueous environments, it is isolated also from multiple animals and food sources. It has been found in pigs [32] and ground pork [33], [34], chicken carcasses [35], [36] and other poultry [37], as well as in beef [38], [39], lamb [34] and the feces of other animals [40]. *A. butzleri* has also been isolated increasingly from human diarrheal stool samples [41]–[47]. The clinical symptomatology described for *A. butzleri* typically includes diarrhea and recurrent abdominal cramps [10], although *A. butzleri*-related bacteremia has also been reported [48], [49]. Prouzet-Mauléon et al. [46] reported an isolation frequency of 1% from human clinical stool samples. Additionally, Vandenberg et al. [47] reported an isolation frequency of 3.5% from diarrheic stool samples. Although co-infection with other enteric pathogens was reported by Prouzet-Mauléon and Vandenberg, in the majority of samples (14/15 clinical stool samples and 55/67 patients, respectively) no other enteric pathogen was detected. Houf et al. reported that Arcobacters were isolated from 1.4% (7/500) of asymptomatic human stool samples [50]. However, all seven isolates were typed as *A. cryaerophilus*; *A. butzleri* was not isolated. Similarly, Vandenberg et al. [47] reported also that *A. butzleri* was isolated more frequently from diarrheic stool samples than from non-diarrheic stool samples. Thus, the isolation of *A. butzleri* from diarrheic stool samples is likely to be relevant clinically and is probably not due to the organism being merely a human commensal. Therefore, these data suggest strongly that *A. butzleri* is an emerging pathogen [10], where transmission, as with *C. jejuni*, occurs probably through consumption of contaminated food or water. The low level of incidence reported in human clinical samples is most likely an underestimate, due to sub-optimal isolation and/or detection methods [46].

Relatively little is known about *A. butzleri,* compared to other members of the epsilon subdivision, but the wealth of genomic information from other epsilonproteobacterial taxa provides a solid foundation to compare and contrast *A. butzleri* to its taxonomic relatives. The genomes of multiple species of Epsilonproteobacteria have been sequenced; these include: four strains of *C. jejuni* subsp. *jejuni* ([51]–[54]: strains NCTC 11168, RM1221, 81-176 and 81116, respectively); *C. jejuni* subsp. *doylei* strain 269.97 (CP000768.1); *Campylobacter coli* strain RM2228, *Campylobacter lari* strain RM2100 and *C. upsaliensis* strain RM3195 [52]; *Campylobacter fetus* subsp. *fetus* strain 82-40 (CP000487.1); *Campylobacter curvus* strain 525.92 (CP000767.1); *Campylobacter concisus* strain 13826 (CP000792.1); *Campylobacter hominis* strain ATCC BAA-381 (CP000776.1); *Sulfuromonas denitrificans* strain ATCC 33889 (formerly *Thiomicrospira denitrificans* [55]: CP000153); *Wolinella succinogenes* strain DSM 1740 [56]; *Helicobacter hepaticus* strain ATCC 51449 [57]; three strains of *H. pylori* ([58]-[60]: 26695, J99 and HPAG1, respectively); *Helicobacter acinonychis* strain Sheeba [61]; and the deep-sea vent taxa *Nitratiruptor* sp. and *Sulfurovum* sp. (strains SB155-2 and NBC37-1, respectively [62]). This study presents the genomic sequence of a human clinical isolate, *A. butzleri* strain RM4018, a derivative of the type strain ATCC 49616. The genomic data revealed multiple differences between *A. butzleri* and other members of the *Campylobacteraceae*, as well as pathways and systems vital for its survival in diverse environments.

## Results and Discussion

### General features

The genome of *Arcobacter butzleri* strain RM4018 contains 2,341,251 bp; as such it is the second largest characterized epsilonproteobacterial genome to date, smaller than the genome of *Sulfurovum* strain NBC37-1 (2,562,277 bp) but larger than both the genomes of *S. denitrificans* strain ATCC 33889 (2,201,561 bp) and *W. succinogenes* strain DSM 1740 (2,110,355 bp). The G+C content of the RM4018 genome (27%) is remarkably low. A summary of the features of the strain RM4018 genome is provided in Table 1. A diagrammatic representation of the RM4018 genome is presented in Figure S1.

**Table 1:** Features of the *Arcobacter butzleri* RM4018 genome

General features	Number or % of total
Chromosome size (bp)	2,341,251
G+C content	27.05%
CDS numbers^a^	2259
Assigned function	1011 (45%)
Pseudogenes	5
General function	505 (22%)
Conserved hypothetical/hypothetical	743 (33%)
Prophage	1
Genetic islands	3
Ribosomal RNA operons	5
Plasmids	0
IS elements	0
Poly GC tracts	0
**Gene classes**	
Chemotaxis proteins	46
Che/Mot proteins	11
Methyl-accepting chemotaxis proteins	29
Redox-sensing PAS domain proteins	3
Cyclic diguanylic acid proteins	25
Restriction/modification systems	
Type I	0^b^
Type II/IIS	0
Type III	0
Transcriptional regulators	
Regulatory proteins	36
Non-ECF family σ factors	1
ECF family σ/anti-σ factor pairs	7
Two-component systems	
Response regulator	42
Sensor histidine kinase	37
**Taxon-specific genes**	
* A. butzleri* proteins found:	
Within *ε*-Proteobacterial taxa	1754 (77.6%)
* Campylobacter* only	98 (4.3%)
* Helicobacter* only	10 (0.4%)
* S. denitrificans* or *W. succinogenes* only	133 (5.9%)
* Sulfurovum* or *Nitratiruptor* only	56 (24.8%)
Only in non *ε*-Proteobacterial taxa	190 (8.4%)
Unique *A. butzleri* proteins	315 (13.9%)

a Total does not include pseudogenes.

b One *hsdM* pseudogene and an *hsdS*/*hsdM* pair are present but no complete Type I R/M system (including *hsdR*) is present.

Consistent with its size, the RM4018 genome is predicted to encode 2259 coding sequences (CDSs). Based on pairwise BLASTP comparisons of proteins predicted to be encoded by these CDSs against proteins in the NCBI non-redundant (nr) database (release 10/13/2007), and on the presence of various Pfam and PROSITE motifs, 1011 (45%) of the predicted proteins were assigned a specific function, 505 (22%) were attributed only a general function, and 743 (33%) were considered proteins of unknown function (Table 1). A complete list of the CDSs predicted to be present within the genome of strain RM4018 and their annotation is presented in the supplementary Table S1. A breakdown of the CDSs by function is presented in supplementary Table S2.

### Relationship of *A. butzleri* to other taxa

*Arcobacter butzleri* is a member of the family *Campylobacteraceae* which includes also the genera *Campylobacter* and *Sulfurospirillum*. Given the close taxonomic relationship between *Arcobacter* and *Campylobacter*, it is noteworthy that 17.2% and 12.4% of the RM4018 proteins have their best match in proteins encoded by *S. denitrificans* and *W. succinogenes*, respectively, both members of the *Helicobacteraceae* (Table 2). Moreover, approximately 25% of the RM4018 proteins have their best match in proteins encoded by *Sulfurovum* or *Nitratiruptor*, deep-sea vent Epsilonproteobacteria isolated from a sulfide mound off the coast of Japan [62].The percentage of *S. denitrificans* homologs with best matches is greater than that of the eight sequenced *Campylobacteraceae* species combined (13.1%). These differences are reduced somewhat by comparing the top five matches and not just the best match; however, even using these parameters, *S. denitrificans* (9.6%) remains the closest related organism (Table 2). Among the Campylobacters, *C. fetus* (3.1% best matches) is the closest to *A. butzleri*, although it is possible that other Campylobacters, whose genomes are as yet un-sequenced, may have higher degrees of similarity to *A. butzleri* than *C. fetus*.

**Table 2:** Similarity of predicted *A. butzleri* proteins to proteins from other taxa.

Taxon	Best match^a^		Within best 5 matches^b^
	#	%	%
*ε-Proteobacteria*	1390	71.50	63.43
* Campylobacteraceae*	258	13.27	22.54
* Campylobacter coli*	19	0.98	1.48
* Campylobacter concisus*	47	2.42	3.88
* Campylobacter curvus*	50	2.57	4.73
* Campylobacter fetus*	60	3.09	4.24
* Campylobacter hominis*	19	0.98	1.90
* Campylobacter jejuni*	35	1.80	3.55
* Campylobacter lari*	15	0.77	1.59
* Campylobacter upsaliensis*	10	0.51	1.00
Other	3	0.15	0.17
* Helicobacteraceae*	603	31.02	21.21
* Helicobacter acinonychis*	4	0.21	0.59
* Helicobacter hepaticus*	21	1.08	1.98
* Helicobacter pylori*	3	0.15	0.75
* Sulfuromonas denitrificans*	335	17.23	9.56
* Wolinella succinogenes*	240	12.35	8.31
Other	0	0.00	0.02
* Nautiliaceae*	47	2.42	3.33
Unclassified	482	24.79	16.34
* Nitratiruptor sp.*	240	12.35	7.98
* Sulfurovum sp.*	235	12.09	7.96
Other	7	0.36	0.41
Other *Proteobacteria*	395	20.32	24.53
α-*Proteobacteria*	30	1.54	2.47
* β-Proteobacteria*	47	2.42	3.11
* Burkholderiales*	34	1.75	2.15
Other	13	0.67	0.96
* γ-Proteobacteria*	242	12.45	14.30
* Alteromonadales*	60	3.09	4.06
* Enterobacteriales*	19	0.98	1.60
* Oceanospirillales*	36	1.85	1.58
* Pseudomonadales*	34	1.75	2.27
* Thiotrichales*	22	1.13	0.75
* Vibrionales*	26	1.34	1.68
Other	45	2.31	2.35
* δ-Proteobacteria*	68	3.50	4.23
* Desulfuromonadales*	46	2.37	2.64
Other	22	1.13	1.59
*Actinobacteria*	3	0.15	0.35
*Aquificae*	5	0.26	0.38
*Bacteroidetes/Chlorobi*	55	2.83	3.82
* Chlorobiales*	14	0.72	1.22
Other	41	2.11	2.60
*Chlamydiae/Verrucomicrobia*	1	0.05	0.25
*Chloroflexi*	3	0.15	0.25
*Cyanobacteria*	11	0.57	0.94
*Deinococcus-Thermus*	2	0.10	0.06
*Fibrobacteres/Acidobacteria*	2	0.10	0.17
*Firmicutes*	48	2.47	3.19
* Bacillales*	14	0.72	1.28
* Clostridiales*	30	1.54	1.61
Other	4	0.21	0.30
*Fusobacterium*	4	0.21	0.29
*Nitrospirae*	0	0.00	0.05
*Planctomycetes*	3	0.15	0.35
*Spirochaetes*	7	0.36	0.48
*Thermotogae*	1	0.05	0.13
Other Bacteria	0	0.00	0.04
*Archaea*	7	0.36	0.68
* Crenarchaeota*	1	0.05	0.06
* Euryarchaeota*	5	0.26	0.55
Other	1	0.05	0.07
Phage/Plasmid	1	0.05	0.29
*Eukaryota*	6	0.31	0.33

Top matches are derived from Table S3. Predicted *Arcobacter butzleri* proteins were compared to proteins in the NCBI non-redundant (nr) database by BLASTP. Matches with an Expect (*E*) value of >1×10^−5^, an identity of <25%, and an alignment length across either the query or match sequences of <75% were excluded. The top five matches (where applicable) were identified at each locus (Table S3) and classified by taxon. Individual orders are listed if they constitute >1% of the total.

a A total of 1944 loci contained matches to proteins in the nr database, according to the criteria listed above. Percentage values for the defined taxa do not include the 315 loci (13.9% of the 2259 identified loci) for which no homologs were identified.

b Multiple strains of the same species (e.g. *C. jejuni* RM1221 and *C. jejuni* NCTC 11168) at a given locus were counted once. Percentage values for the defined taxa do not include the 315 loci for which no homologs were identified.

**Figure 1:**
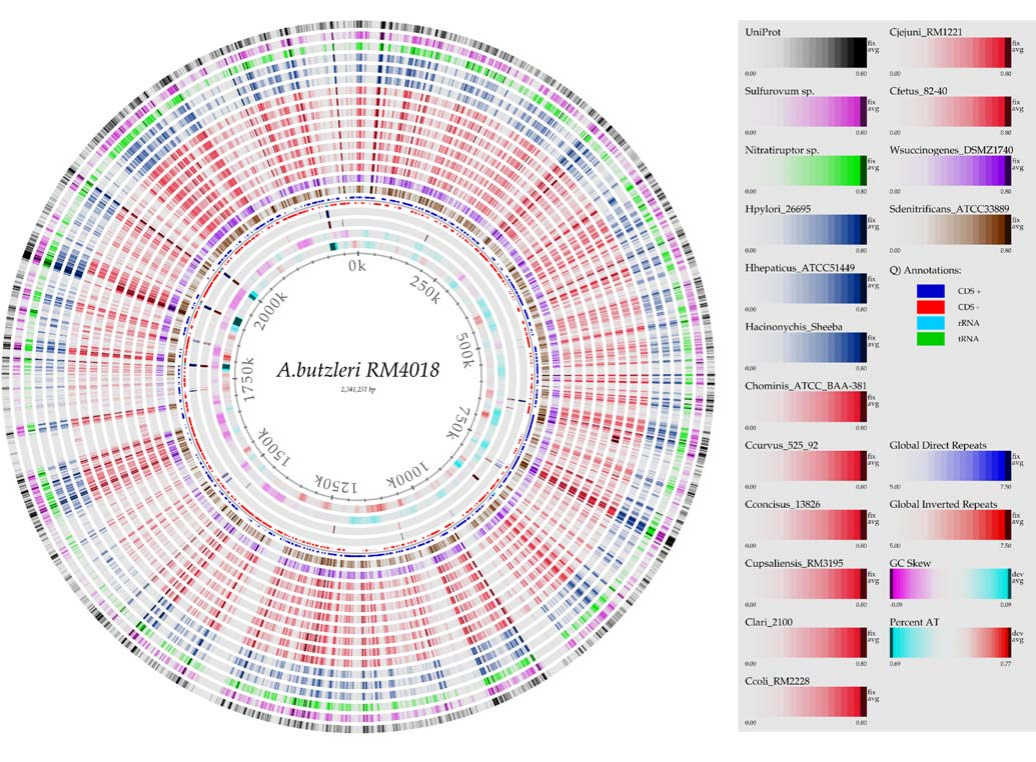
Genome BLAST atlas of the *A. butzleri* strain RM4018. *Arcobacter butzleri* strain RM4018 is the reference genome and is compared to a set of 15 other epsilonproteobacterial genomes, including different *Campylobacter* (rings 7–14 from center) and *Helicobacter* (rings 15–17 from center) strains, as well as the UniProt database (outermost ring in black). A web-based “zoomable atlas” can be found at [147].

In the entire *A. butzleri* strain RM4018 proteome (unique and non-unique), 61.5% (1390/2259) of the proteins have their best matches in proteins encoded by the epsilonproteobacterial taxa, and 79.0% (1785/2259) are similar to Proteobacterial proteins. Most of the matches to non-Epsilonproteobacteria are found within the gamma subdivision (Table 2), in genera such as *Marinobacter* (*Alteromonadales*), *Oceanospirillum* (*Oceanospirillales*) and *Pseudomonas* (*Pseudomonadales*). Other phyla with a moderate number of protein matches include Firmicutes (2.5%), especially *Clostridiales* (1.5%), and Bacteroidetes/Chlorobi (2.8%). Among the 2259 predicted CDSs of strain RM4018, 315 (13.9%) had no homolog within the nr database using a minimum expect (*E*) value of 1×10^−5^, a minimum identity of 25% and a minimum alignment length of 75% (Figure 1, Table 2, Table S3).

Although approximately 30% of the *A. butzleri* RM4018 proteins with homologs within the nr database have their best matches to non-epsilonproteobacterial proteins, the approximately 550 genes encoding these proteins are not distributed randomly through the RM4018 genome, but rather clustered with respect to position and protein function (Table S2, Table S3). Interestingly, many of these clusters contain proteins involved in transport, and several contain discrete loci associated with a single function, such as the urease and quinohemoprotein loci described below. The genes contained in these loci are found often in the same order as in other taxa, suggesting lateral transfer. However, the G+C content of these gene clusters is not significantly different from the genome as a whole, suggesting that if these clusters were acquired from other non-*Arcobacter* taxa the acquisition was not recent.

Generally, strain RM4018 proteins involved in “housekeeping” functions (e.g. amino acid biosynthesis, fatty acid biosynthesis and protein synthesis) have homologs well-conserved among the other Epsilonproteobacteria (Table 3). Nevertheless, a number of other major functional categories are more divergent. For example, 19% (13/70) of the strain RM4018 proteins involved in DNA replication and repair have either no homologs or homologs of low similarity (≤ 35% identity) among the other epsilonproteobacterial taxa; this proportion increases to 37% (26/70) if proteins with homologs only within the *Campylobacteraceae* are included (Table 3). Other divergent functional categories include: sulfur/nitrogen metabolism (34%), transcriptional regulators/σ-factors (47%), signal transduction (49%), cell envelope (37%), chemotaxis (70%) and antibiotic resistance (36%) (Table 3). These divergent functional categories will be discussed in further detail.

**Table 3:** Divergence within the major functional categories

	Divergent genes^a^		
Functional category	ε-proteobacteria	*Campylobacteraceae*	*Helicobacteraceae*
Glycolysis/TCA cycle/gluconeogenesis	5/34 (15%)	5/34 (15%)	6/34 (18%)
Respiration/electron transport	10/90 (11%)	24/90 (27%)	20/90 (22%)
Sulfur/nitrogen metabolism^b^	10/29 (34%)	22/29 (76%)	12/29 (41%)
Amino acid biosynthesis	3/85 (4%)	11/85 (13%)	3/85 (4%)
Purine/pyrimidine biosynthesis	2/29 (7%)	5/29 (17%)	2/29 (7%)
Biosynthesis of cofactors^c^	6/86 (7%)	16/86 (19%)	10/86 (12%)
Fatty acid biosynthesis	0/17 (0%)	1/17 (6%)	0/17 (0%)
Transcriptional regulators/σ factors	24/51 (47%)	41/51 (80%)	29/51 (57%)
Signal transduction	51/104 (49%)	77/104 (74%)	58/104 (56%)
Protein translation/modification	2/143 (1%)	9/143 (6%)	5/143 (3%)
DNA replication/repair	13/70 (19%)	26/70 (37%)	16/70 (23%)
RNA synthesis	1/17 (6%)	5/17 (29%)	1/17 (6%)
Macromolecule degradation	4/45 (9%)	13/45 (29%)	7/45 (16%)
Cell envelope^d^	70/190 (37%)	91/190 (48%)	85/190 (45%)
Transport/secretion	47/191 (25%)	88/191 (46%)	71/191 (37%)
Chemotaxis	32/46 (70%)	38/46 (83%)	33/46 (72%)
Antibiotic resistance	10/28 (36%)	20/28 (71%)	10/28 (36%)
General function	458/782 (59%)	597/782 (76%)	527/782 (67%)

a *Arcobacter butzleri* strain RM4018 proteins that have either no homologs or homologs of low similarity (≤ 35% identity) within the given taxa/taxon. Categories and values are derived from Table S2.

b Includes [Fe-S] centers.

c Includes biosynthesis of prosthetic groups and carriers.

d LOS, flagella and membrane proteins.

e Includes conserved hypothetical and hypothetical proteins. Does not include general function proteins assigned to the other functional categories.

### Methyl-directed mismatch repair and the absence of polynucleotide G:C tracts

Members of the Epsilonproteobacteria lack multiple genes in the methyl-directed mismatch repair system (MMR). The MMR system depends upon the presence of three main functions: 1) the MutSLH endonuclease complex, 2) a methylation system to identify parental vs. daughter strands and 3) multiple 5′→3′ and 3′→5′ single-strand DNA exonucleases, e.g. RecJ, ExoI and ExoVII [63]. The *A. butzleri* RM4018 genome is not predicted to encode MutL or MutH. However, it is predicted to encode a MutS2 family MutS protein, which is distinguished from MutS1 by the presence of a C-terminal Smr domain. It has been proposed that the Smr domain has a MutH-like nicking endonuclease function [64]. Thus, the *A. butzleri* MutS2 protein may contain both MutS and MutH domains, obviating the need for a MutL scaffold protein. *A. butzleri* strain RM4018 is not predicted to encode a Dam DNA-adenine methyltransferase (data not shown) used commonly in MMR systems, but the presence of a Dcm DNA-cytosine methyltransferase was demonstrated experimentally in strain RM4018 (data not shown); this Dcm function may be used by the *A. butzleri* MMR system to distinguish between parental and daughter strands. Although the annotated epsilonproteobacterial genomes contain the 5′→3′ exonuclease RecJ, they do not contain the 3′→5′ exonucleases ExoI or ExoVII. A 32-fold increase in +1 frameshifts and an 11-fold increase in -1 frameshifts were observed in an *E. coli* ExoI^−^ ExoVII^−^ mutant [65]; therefore, one possible outcome of such a defect in MMR systems would be the formation and extension of hypervariable G:C tracts, such as those identified in all characterized *Campylobacter* and *Helicobacter* genomes. A major distinguishing characteristic of the *A. butzleri* RM4018 genome is the lack of such G:C tracts, a feature shared by the *S. denitrificans* and *W. succinogenes* genomes. A comparison of these three genomes revealed the presence of two to four members of the DnaQ superfamily, to which the 3′→5′ exonuclease ExoX belongs [66]. Thus, one or more of these DnaQ homologs may provide the missing 3′→5′ exonuclease function, and it is conceivable that the absence of hypervariable G:C tracts in *A. butzleri* strain RM4018 and the presence of these tracts in *Campylobacter* and *Helicobacter* may be due to the presence or absence of a functional MMR system, respectively.

### Sulfur assimilation, oxidation and the biosynthesis of sulfur-containing amino acids

**Figure 2:**
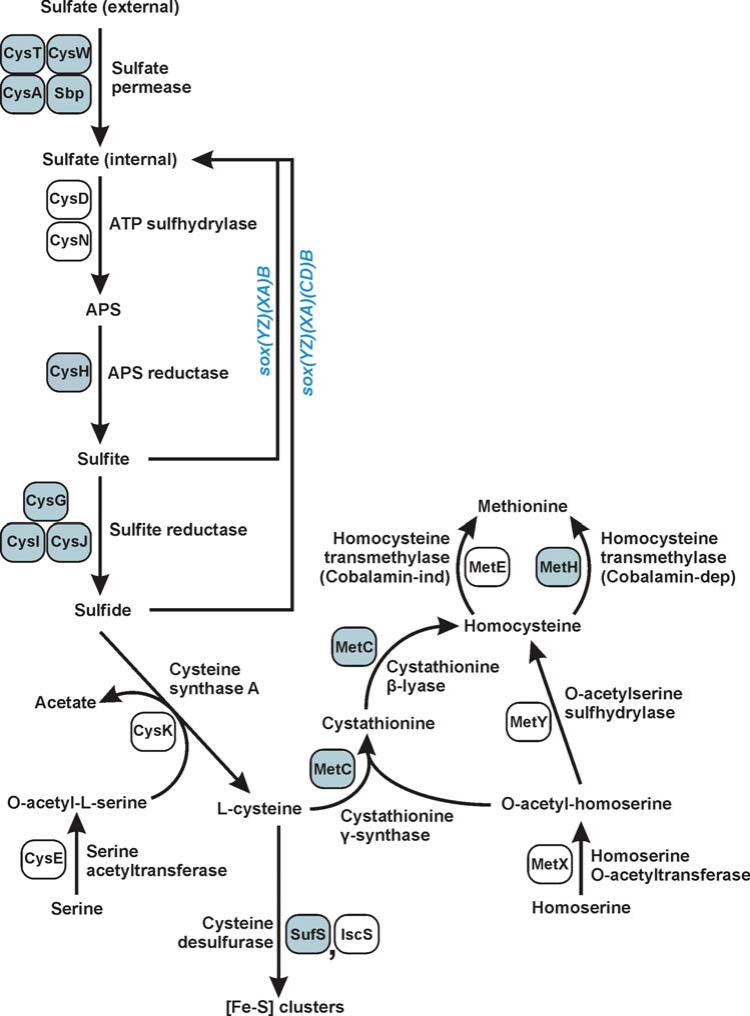
Sulfur assimilation and biosynthesis of the sulfur-containing amino acids. Genes/proteins in strain RM4018 unique within *Campylobacteraceae* or proteins with *Campylobacteraceae* orthologs of low similarity are labeled/shaded in blue.

*Arcobacter butzleri* strain RM4018 contains a number of genes required for sulfur uptake and assimilation (Figure 2). These genes include those encoding the sulfate ABC transporter CysATW, the sulfate binding protein Sbp, the ATP sulfhydrylase CysDN, the adenosine phosphosulfate (APS) reductase CysH, the sulfite reductase proteins CysI and CysJ and the siroheme synthase CysG. CysD and CysN have been identified also in *C. coli*, but the position of these genes, along with the APS kinase-encoding gene *cysC* and the 3′(2′),5′-bisphosphate nucleotidase-encoding gene *cysQ*, is in the capsular locus, suggesting that these genes in *C. coli* are involved in the formation or modification of the capsule and not sulfur assimilation per se.

Intracellular sulfate is reduced to sulfite in bacteria by one of two pathways. Both pathways first convert sulfate to APS via the ATP sulfhydrylase CysDN [67]. The first pathway converts APS to PAPS (phosphoadenosine phosphosulfate) using the kinase CysC, and then reduces PAPS to sulfite using the PAPS reductase CysH [67]; PAPS toxicity in some taxa is decreased through the conversion of PAPS to APS via CysQ [68], [69]. The second pathway, identified originally in plants and subsequently in taxa such as *P. aeruginosa* [70] and *M. tuberculosis* [71], reduces APS to sulfite directly using the APS reductase CysH. The presence of conserved two-cysteine motifs in strain RM4018 CysH, characteristic of bacterial APS reductases [70], [71], and the absence of both CysC and CysQ homologs, suggests that sulfate reduction to sulfite in this strain does not use a PAPS intermediate.

The sulfite generated by strain RM4018 CysH has two potential fates: reduction to sulfide and assimilation into the sulfur-containing amino acids L-cysteine and L-methionine (Figure 2), or oxidation to sulfate, facilitated by a cytochrome *c* multienzyme complex encoded by the *sox* genes (*ab0563*-*ab0570*). Initial data indicated that sulfite is oxidized by strain RM4018 (data not shown), and also that it can grow in minimal media without added cysteine or methionine (data not shown). Hence, both metabolic fates are possible, and the genetic switch modulating oxidation or reduction of sulfite remains to be identified. Homologs of the *sox* genes have been identified in multiple taxa, including *Paracoccus pantotrophus* [72], [73], *Chlorobium tepidum* [74] and *Rhodovulum sulfidophilum* [75]. The Sox clusters of some organisms can contain as many as 15 genes, but seven genes (*soxXYZABCD*) are essential for sulfur oxidation [76]. These seven genes encode four proteins: the heterodimeric *c*-type cytochrome SoxXA, the heterodimeric sulfur-binding protein SoxYZ, the heterotetrameric SoxCD sulfur dehydrogenase and the thiol sulfate esterase SoxB [76]. Sulfite, sulfide, sulfur and thiosulfate are possible substrates for the Sox complex; thiosulfate and sulfide oxidation requires the entire complex, while sulfite oxidation does not require SoxCD [76]. The presence of SoxCD in strain RM4018 would suggest that thiosulfate is oxidized by this organism. However, other *sox* genes important for thiosulfate oxidation, e.g. *soxV* [77], [78], are absent in this strain, as well as the Sbp-related thiosulfate-binding protein CysP. Thus, it is possible that only sulfite and sulfide are oxidized in strain RM4018. Sox proteins are present also in the related epsilonproteobacterial taxa *Sulfurovum*, *Nitratiruptor* and *S. denitrificans* (Table S2). However, there are multiple differences between the sulfur oxidation systems of these related organisms and those of strain RM4018: 1) the *sox* genes in strain RM4018 form a single cluster instead of two clusters, 2) the strain RM4018 *sox* cluster contains only one copy of *soxY* and *soxZ* and 3) no significant similarity exists between the SoxXA protein of strain RM4018 and the SoxXA proteins of *Sulfurovum*, *Nitratiruptor* and *S. denitrificans.* The *sox* cluster of strain RM4018 was not detected in 12 additional *A. butzleri* strains (see below). However, a cluster of *sox* genes similar to that of strain RM4018 has been identified in *A. halophilus* (data not shown). Thus, it appears that strain RM4018 may have acquired the *sox* cluster through lateral gene transfer from another *Arcobacter* species and that the sulfur oxidation machinery of *Arcobacter* and, e.g., *Sulfurovum* are likely to be distinct evolutionarily.

The genome of strain RM4018 contains all of the genes necessary for the biosynthesis of L-cysteine, L-methionine and iron-sulfur clusters (Figure 2). Consistent with the proposed absence of thiosulfate metabolism, the bacterium has only a gene encoding the CysK cysteine synthase A and not the CysM cysteine synthase B that utilizes thiosulfate instead of sulfide to produce S-sulfocysteine [67]. Strain RM4018 also contains both the cobalamin-dependent and cobalamin-independent homocysteine transmethylases MetH and MetE.

### The central metabolism of *A. butzleri*

**Figure 3:**
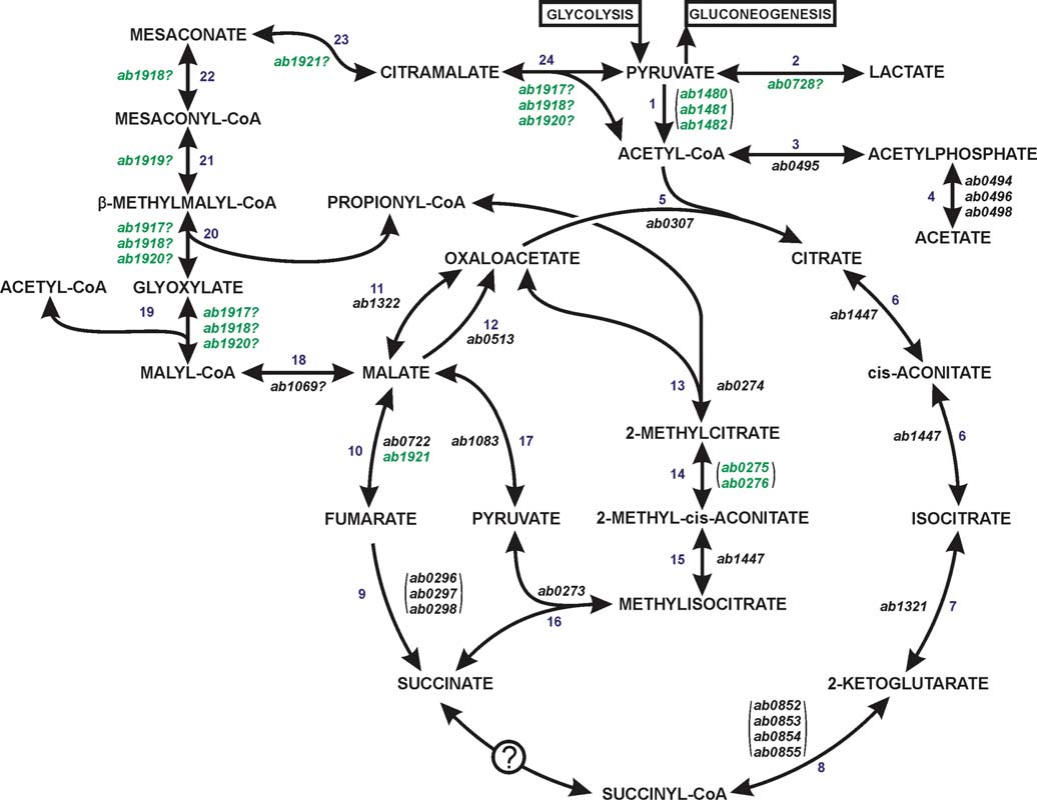
Predicted TCA, methylcitrate and citramalate cycles of *A. butzleri* strain RM4018. Genes unique within the *Campylobacteraceae* are labeled in green. Genes in parentheses encode multi-subunit proteins or a protein and its cognate accessory protein. 1: pyruvate dehydrogenase; 2: L-lactate dehydrogenase; 3: phosphate transacetylase; 4: acetate kinase; 5: citrate synthase; 6: aconitase; 7: isocitrate dehydrogenase; 8: 2-oxoglutarate:acceptor oxidoreductase; 9: fumarate reductase; 10: fumarase; 11: malate dehydrogenase; 12: malate:quinone oxidoreductase; 13: 2-methylcitrate synthase; 14: 2-methylcitrate dehydratase; 15: aconitase; 16: 2-methylisocitrate lyase; 17: malic enzyme; 18: malyl-CoA hydrolase; 19: malyl-CoA lyase; 20: β-methylmalyl-CoA lyase; 21: mesaconyl-CoA hydratase; 22: mesaconyl-CoA synthetase; 23: citramalate hydrolase; 24: citramalate lyase.

The general function of the citric acid/tricarboxylic acid (TCA) cycle is to oxidize organic tri- and di-carboxylic acids to provide energy and biosynthetic precursors for metabolism. *Arcobacter butzleri* strain RM4018 encodes several proteins homologous to other epsilonproteobacterial TCA cycle enzymes (Figure 3), e.g. isocitrate dehydrogenase (AB1321), 2-oxoglutarate dehydrogenase (AB0852-AB0855), malate dehydrogenase (AB1322) and citrate synthase (AB0307). However, *A. butzleri* strain RM4018 is predicted putatively to encode two aconitate hydratases and two fumarate dehydratases. Additionally, enzymes that catalyze two TCA cycle steps are absent apparently in strain RM4018.

The genes *ab0275* and *ab1447* encode proteins homologous to the *E. coli* aconitate hydratases AcnA and AcnB, respectively. *Arcobacter butzleri* AcnB shares between 62% and 76% identity with other epsilonproteobacterial aconitases. *Arcobacter butzleri* AcnD, encoded by *ab0275*, does not have significant similarity with any of the known epsilonproteobacterial proteins, but has an 82% similarity with the PrpD 2-methylisocitrate dehydratase of *Alkaliliminicola ehrlichei* as well as 68% similarity with *E. coli* AcnA. In addition, the proximity of *ab0275* to genes that encode the methylcitrate pathway enzymes PrpB and PrpC suggests that in strain RM4018 AcnD is the aconitase of the methylcitrate pathway and AcnB is the aconitase of the TCA cycle. Also, the absence of the 2-methylcitrate dehydratase PrpD and the presence of *acnD* next to *ab0276*, which encodes the AcnD-associated protein PrpF, suggests that *A. butzleri* strain RM4018 contains the alternate AcnD/PrpF methylcitrate pathway [79].

*Escherichia coli* can express three fumarases: A, B and C. The first two contain iron-sulfur clusters and are unstable aerobically; fumarase C is stable in the presence of oxygen. The genome of strain RM4018 is predicted to encode two fumarases: AB0722 and AB1921. AB0722 has 74% similarity to *E. coli* fumarase C and would be predicted to be active under aerobic conditions. AB1921 has 48% similarity to *E. coli* fumarases A and B and would require microaerobic or anaerobic conditions. AB1921 also has 55–64% similarities with two proteins encoded by the obligate microaerophile *W. succinogenes* and two encoded by *H. hepaticus.* Interestingly, the sequence similarities with the *W. succinogenes* proteins WS1766 and WS1767, and the *H. hepaticus* proteins HH1702 and HH1793 correspond to the first 282 and the last 185 residues of AB1921, respectively, suggesting that the two polypeptides in *W. succinogenes* or *H. hepaticus* are fused into one protein in *A. butzleri.*

No genes encoding the SucCD succinyl-CoA synthetase or the SdhABCD succinate dehydrogenase have been identified in the *A. butzleri* genome, but the gene cluster *ab0296-ab0298* encodes proteins with high similarities (71–85%) to the fumarate reductase FrdABC of *C. jejuni, H. hepaticus, H. pylori,* and *W. succinogenes,* which catalyzes the reaction in the reductive direction converting fumarate to succinate. Some bacteria such as *E. coli* and *C. jejuni* have genes encoding both Sdh and Frd, and others, e.g. *H. pylori* and *W. succinogenes*, encode only Frd. The similar structures of Sdh and Frd preclude predicting, solely from sequence analyses, whether an enzyme is one or the other. Regulation of transcriptional levels by the oxygen content in the atmosphere permits differentiation between both enzymes; obligate aerobes encode Sdh and anaerobes encode Frd. Initial experiments with *A. butzleri* indicated the presence of fumarate reduction and no succinate oxidation, and this activity increased several-fold in bacteria grown under anaerobic conditions relative to bacteria grown under aerobic conditions (data not shown). These results provided evidence supporting the identification of *A. butzleri* FrdABC (*ab0296*-*ab0298*) as a fumarate reductase (Figure 3).

The genome of strain RM4018 may contain also a pathway, encoded by the gene cluster *ab1917*-*ab1921*, which resembles segments of the 3-hydroxypropionate cycle [80], [81] and the citramalate cycle [82], [83], and interconverts glyoxylate and propionyl-CoA with pyruvate and acetyl-CoA (Figure 3). AB1917 and AB1920 are similar to the CitE citrate lyase. In addition, AB1918 is an acetyl-CoA synthetase with a CaiC domain in residues 50-550, and a CitE domain in residues 550-825. CitE domains have strong similarity to malyl-CoA lyases and have lesser homology to malate synthetase domains. Thus, any of these three proteins could function as a malyl-CoA lyase. AB1919 has a ∼73% similarity to enol-CoA hydratase which catalyzes reversible reactions interconverting 2-enoyl-CoA compounds, such as mesaconyl-CoA, and 3-hydroxyacyl-CoA compounds, such as β-methylmalate-CoA. If the acetyl-CoA synthetase encoded by *ab1918* and the anaerobic fumarate hydratase encoded by *ab1921* were broad specificity enzymes, the former could catalyze the synthesis of mesaconyl-CoA from mesaconate, and the latter the interconversion of mesaconate and citramalate. Finally, citramalate could be synthesized from pyruvate and acetyl-CoA by one of the CitE enzymes. The gene *ab1069* encodes an acyl-CoA thioester hydrolase which can catalyze the synthesis of malyl-CoA from malate or the reverse reaction. One of the three citrate lyases mentioned could then convert malyl-CoA to glyoxylate and acetyl-CoA and glyoxylate (Figure 3).

*Arcobacter butzleri* does not grow on acetate, citrate, propionate or acetate with propionate, and it grows on fumarate, lactate, malate and pyruvate (data not shown). These data suggested that the methylcitrate pathway would function to produce oxaloacetate for the TCA cycle and propionyl-CoA. The latter metabolite together with glyoxylate synthesized from malate via malyl-CoA would be converted to acetyl-CoA and pyruvate. Acetyl-CoA could be synthesized also from pyruvate via the pyruvate dehydrogenase complex encoded by *ab1480-ab1482.* Alternatively, the methylcitrate pathway could have a regulatory role converting any excess propionyl-CoA to succinate and pyruvate. Through the activity of a malic enzyme encoded by *ab1083,* pyruvate can be carboxylated to malate and used to replenish the TCA cycle.

### Anaerobic and aerobic respiration

**Figure 4:**
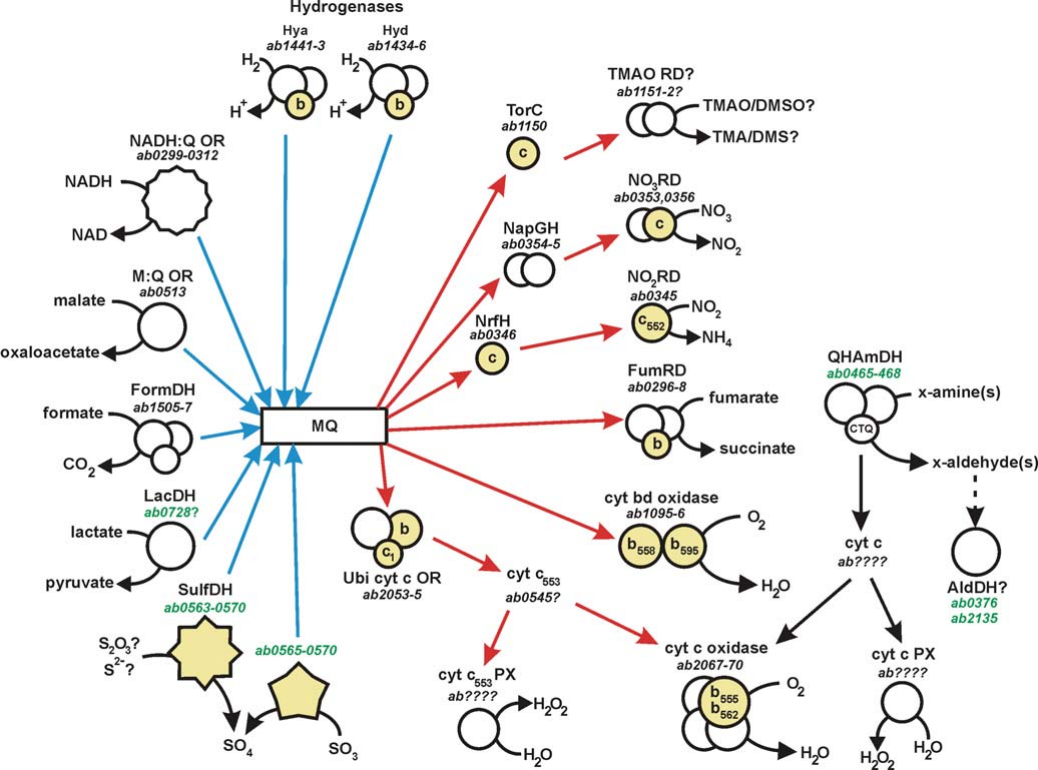
Respiratory pathways in *A. butzleri* strain RM4018. Transfer of electrons to the menaquinone (MQ) pool is represented by blue arrows; transfer of electrons from the menaquinone pool is represented by red arrows. *b*- and *c*-type cytochromes are shaded yellow. Genes unique within the *Campylobacteraceae* are labeled in green. NADH:Q OR: NADH:quinone oxidoreductase; M:Q OR: malate:quinone oxidoreductase; FormDH: formate dehydrogenase; LacDH: L-lactate dehydrogenase; SulfDH: sulfur dehydrogenase; Ubi cyt *c* OR: Ubiquinol cytochrome c oxidoreductase; TMAO RD: TMAO reductase; NO_3_RD: nitrate reductase; NO_2_RD: nitrite reductase; FumRD: fumarate reductase; QHAmDH: quinohemoprotein amine dehydrogenase; AldDH: aldehyde dehydrogenase; PX: peroxidase; CTQ: cysteine tryptophylquinone.

*A. butzleri* has a full complement of genes for aerobic/microaerobic respiration including those encoding NADH:quinone oxidoreductase, ubiquinol cytochrome *c* oxidase, ferredoxin, cytochrome *bd* oxidase, cytochrome *c* oxidase (*cbb*3-type), and F1/F0 ATPase (Figure 4), but it also has limited ability for anaerobic respiration. Potential electron donors, in addition to NADH, are hydrogen, malate and formate. One large gene cluster encodes three FeNi hydrogenases with one uptake hydrogenase (*hupSL*) and two membrane associated proteins encoded by *hydABCDF* and *hyaABCD*, the latter two predicted to be anchored to the membrane by the *b*-type cytochromes HydC and HyaC, respectively. There are two formate dehydrogenases, one selenocysteine homolog and one cysteine homolog, suggesting that selenium may be important in their regulation. Also present is a malate:quinone oxidoreductase (Mqo) and a putative lactate dehydrogenase (AB0728), suggesting that lactate may be a potential electron donor. Electrons may be transferred also to the menaquinone pool through the Sox system described above, the number of electrons depending on the substrate oxidized, 2 *e* ^−^ for sulfite and 8 *e* ^−^ for sulfide.

Fumarate, nitrate and nitrite are used by strain RM4018 as electron acceptors. Fumarate can be reduced to succinate by the fumarate reductase FrdABC. Nitrate can be reduced to ammonia via a periplasmic nitrate reductase NapAB and a pentaheme nitrite reductase NrfAH. The *nap* operon has the same gene number and order as that seen in *Campylobacter* species. It is possible also that trimethylamine–oxide (TMAO) and/or dimethylsulfoxide (DMSO) may serve as alternative electron acceptors. Analysis of the strain RM4018 genome indicates the presence of the pentaheme *c*-type cytochrome TorC encoded by *ab1150*. The CDS *ab1151* has been annotated as *bisC*, but the BisC family includes also other anaerobic dehydrogenases, such as the TMAO and DMSO reductases TorA and DmsA, respectively. The genes *torC* and *torA* are co-transcribed usually with *torD*, but no TorD homolog was detected in the strain RM4018 genome. Other electron acceptors may be present: CDS *ab1360* encodes a putative cytochrome *b*-type nitric oxide reductase, and *ab1987* encodes a putative nitrite/nitric oxide reductase. Additional investigations will be necessary to determine the function of these putative electron acceptors.

In addition to the respiratory proteins described, *A. butzleri* strain RM4018 is predicted to encode a quinohemoprotein amine dehydrogenase (QHAmDH), the presence of which is novel in the epsilon subdivision. QHAmDH is a heterotrimeric (αβγ) protein that deaminates oxidatively a variety of aliphatic and aromatic amines, e.g. n-butylamine and benzylamine [84], and is unusual in that the small (γ) subunit contains an intrinsic quinone cofactor [in *A. butzleri*, cysteine tryptophylquinone (CTQ)], formed by the covalent linkage of a cysteine residue to an oxidized tryptophan residue [85]. The presence of this intrinsic quinone cofactor permits the transfer of electrons directly to the *cbb*3 oxidase through either a cytochrome *c* [86] or blue-copper protein (e.g. azurin [87]) intermediate (Figure 4). The QHAmDH locus of strain RM4018 encodes also a radical SAM (S-Ado-Met) family protein (AB0466). A radical SAM protein (ORF2) is found also in the QHAmDH locus of *Paracoccus denitrificans* and has been proposed to play an important role in the formation of the CTQ cofactor [88]. Amino acid motifs conserved in ORF2 are 100% identical to those in AB0466, as well as to the relevant amino acids in the γ subunit AB0467, suggesting that the strain RM4018 QHAmDH locus encodes a functional dehydrogenase. M9 minimal media amended with 0.5% n-butylamine-HCl (v/v), benzylamine-HCl (w/v) or methylamine-HCl (w/v) as a sole carbon source does not support the growth of strain RM4018 (data not shown). However, substrate specificity has been demonstrated among the amine-utilizing taxa [89]; therefore, it is likely that RM4018 utilizes an as yet unidentified aliphatic or aromatic amine. Finally, two aldehyde dehydrogenases, encoded putatively by *ab0376* and *ab2135*, also novel in the Epsilonproteobacteria, were identified in strain RM4018 (Figure 4). Thus, it is possible that the aldehydes generated from the QHAmDH are oxidized further.

### Urease

*Arcobacter butzleri* strain RM4018 contains six genes (*ab0808-ab0813*) involved in the degradation of urea. In bacteria this catabolism involves generally three sets of genes: a nickel-containing urease (composed of α, β and γ subunits), urease accessory proteins which deliver the nickel to the urease and a nickel uptake system. The urease of strain RM4018 is functional (data not shown), as determined by a phenol red/urea assay [90], although the level of activity is not as high as that found in other urease-producing taxa (e.g. UPTC *C. lari*). As in *Helicobacter*, the urease α and β subunits are fused, and the strain RM4018 urease subunits show high homology to *Helicobacter* urease subunits. However, differences exist with the urease loci of both *Helicobacter* and UPTC *C. lari*. First, the gene order of the locus itself, *ureD(AB)CEFG*, is similar to the gene order of the urease loci in *Klebsiella*, *Proteus*, *E. coli* O157:H7 and *Vibrio,* but not the *Helicobacter* locus *ure(AB)IEFGH*. Second, although the urease enzyme itself is similar to the *Helicobacter* urease, the accessory proteins UreD, UreE and UreF, had greater similarity to those identified in *Bacillus, Lactobacillus* and *Psychromonas* (Table S3); additionally, the nickel-binding protein UreE has a histidine-rich C terminus, found in multiple UreE proteins, but not in those from *Helicobacter* or *C. lari*. Also, unlike *Helicobacter*, no obvious nickel uptake system, such as the *H. hepaticus nikABDE* or *H. pylori nixA*, was found in strain RM4018. A putative nickel transporter, AB1752, was identified, but it is unclear whether it is specific for nickel, or is a heavy-metal-ion transporter. Finally, although the *A. butzleri* urease may serve to degrade exogenous urea, it may also degrade endogenous urea, formed during putrescine biosynthesis, specifically during the conversion of agmatine to putrescine by SpeB (AB1578).

### Surface structures

SDS-PAGE analysis suggested that *A. butzleri* strain RM4018 can express lipooligosaccharide (LOS). To date, little is known about the roles of these molecules in *A. butzleri*. They generate great attention in bacterial pathogens since LOS/lipopolysaccharide (LPS) are major inducers of proinflammatory responses, are immunodominant antigens, and play a role in host cell interactions. The LOS biosynthesis locus of strain RM4018 (*ab1805*-*ab1833*) showed a similar organization to those of *Campylobacter* [51], [52], [91], [92], and is thus dissimilar from the loci of *Helicobacter* and *Wolinella*. At both ends of the locus are genes involved in the addition of heptose to the oligosaccharides and the encoded proteins are similar to those of other Epsilonproteobacteria (Table S2). Within the locus, genes are found whose products have functions related to LOS/LPS biosynthesis, including several glycosyltransferases, but the proteins encoded have greater similarity to proteins outside of the Epsilonproteobacteria (Table S3). This LOS/LPS biosynthesis region is conserved among 12 unrelated *A. butzleri* strains, based on comparative genomic indexing (described in detail later), which likely distinguishes *A. butzleri* from *C. jejuni*, where the LOS biosynthesis region is an intraspecies hypervariability region [93]. The gene conservation of this region in *A. butzleri* resembles more closely the conservation of the LPS core biosynthesis region occurring among many of the *Salmonella enterica* serovars [94].

Many bacteria synthesize structurally diverse polysaccharide polymers, O-antigen and capsule that are major antigenic determinants. It is possible that *A. butzleri* strain RM4018 produces O-antigen, since there is a locus (*ab0661*-*ab0697*) that encodes several additional glycosyltransferases. This region has two copies of *wbpG*, *hisH*, and *hisF*, found also in the *Pseudomonas aeruginosa* B-band O-antigen locus [95], and many of the other encoded proteins have greater similarity to proteins from bacteria outside of the Epsilonproteobacteria (Table S2). Although this region could be involved putatively in capsular formation, the absence of conserved *kps* capsular genes in this region, combined with the presence of O-antigen-related genes, suggests that *A. butzleri* strain RM4018 produces O-antigen and not capsule; however, further investigations will be necessary to determine the nature of the *A. butzleri* cell-surface structures. Like many O-antigen biosynthesis regions, the *A. butzleri* region appears to represent an intraspecies hypervariability region with the RM4018 region, present in only 1 of the 12 *A. butzleri* strains examined using comparative genomic indexing.

**Figure 5:**
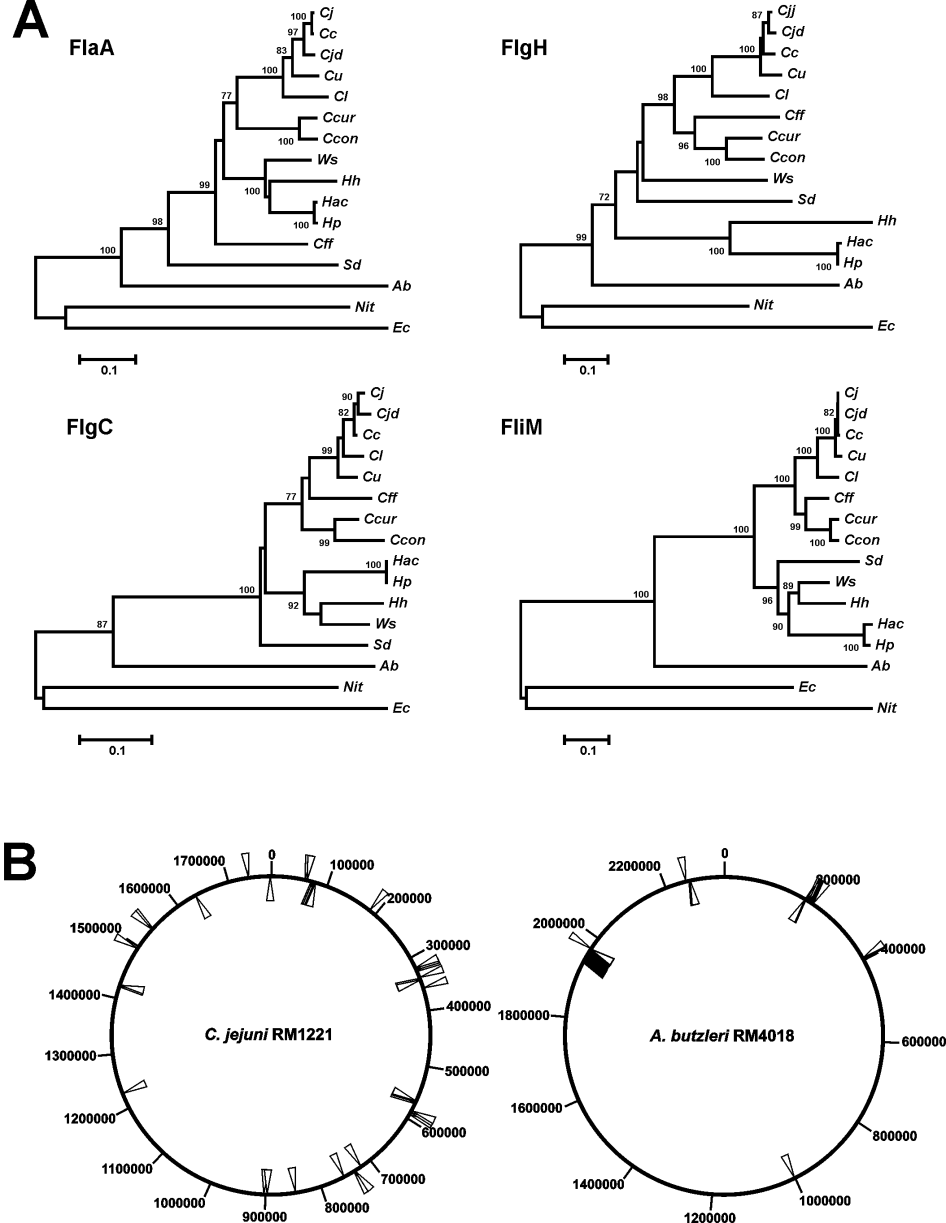
Phylogenetic analysis of four representative flagellar proteins and flagellar clustering in strain RM4018. A. Each dendrogram was constructed using the neighbor-joining algorithm and the Kimura two-parameter distance estimation method. Bootstrap values of >75%, generated from 500 replicates, are shown at the nodes. The scale bar represents substitutions per site. *Cjj*: *Campylobacter jejuni* subsp. *jejuni*; *Cjd*: *C. jejuni* subsp. *doylei*; *Cc*: *C. coli*; *Cu*: *C. upsaliensis*; *Cl*: *C. lari*; *Cff*: *C. fetus* subsp. *fetus*; *Ccur*: *C. curvus*; *Ccon*: *C. concisus*; *Ab*: *A. butzleri; Ws*: *W. succinogenes; Sd*: *S. denitrificans; Hh*: *H. hepaticus; Hac*: *H. acinonychis; Hp*: *H. pylori; Nit*: *Nitratiruptor sp.; Ec*: *Escherichia coli.* B. Location of the flagellar genes of *C. jejuni* strain RM1221 and *A. butzleri* strain RM4018.

*Arcobacter butzleri* strain RM4018 is a motile bacterium that synthesizes a polar flagellum. Many of the flagellar apparatus proteins encoded by strain RM4018 have homologs in other epsilonproteobacterial taxa. However, phylogenetic analysis of selected flagellar proteins suggests that the flagellar apparatus of strain RM4018 has an evolutionary history distinct from those of *Campylobacter* and *Helicobacter* (Figure 5A). This distinct history is supported also by predicted differences between strain RM4018 and *Campylobacter/Helicobacter* in flagellar gene regulation (see below). Additionally, the flagellar genes of strain RM4018 are highly clustered, compared to the flagellar genes of *Campylobacter jejuni* (Figure 5B). The primary cluster in strain RM4018 contains 20 flagellar genes (*ab1931*-*ab1961*) with the other two flagellar clusters containing eight (*ab0197*-*ab0208*) and three (*ab2238*-*ab2244*) genes. The flagellar genes of the related organism *Nitratiruptor* are also highly clustered with the primary cluster containing 36 flagellar and chemotaxis genes. Significantly, the *Nitratiruptor* flagellar proteins also appear to be distinct phylogenetically from those of both *A. butzleri* and *Campylobacter/Helicobacter*. Moreover, the primary flagellar cluster of *Nitratiruptor* has an atypical G+C content, suggesting acquisition through horizontal gene transfer [62]. Although the G+C content of the strain RM4018 flagellar genes is not atypical, it is possible that the flagellar genes of this organism were acquired via a similar mechanism.

### Prophage and genomic islands

The genome of the *A. butzleri* strain RM4018 is predicted to contain a prophage. The size of this prophage is approximately 38 kb and spans genes *ab1655*-*ab1706*. BLASTP comparison of the predicted phage proteins to proteins from other bacteriophage indicates that this prophage is a member of the mutator (Mu) bacteriophage family. The size of the prophage is similar to other Mu-like bacteriophage, and it contains proteins similar to the Mu transposition proteins A and B in addition to coat, baseplate, and tail proteins. Mu-like bacteriophage have been found in multiple bacterial taxa, including *E. coli, Neisseria meningitidis*, *Deinococcus radiodurans*, *Haemophilus influenzae*, *Burkholderia cenocepacia* [96], [97] and notably *C. jejuni* (CMLP1: strain RM1221[52], [93]). Indeed, 26 of the 50 predicted RM4018 Mu-like phage proteins are similar to those encoded by CMLP1 (Table S2). Hence, it is proposed to name this bacteriophage AMLP1 (Arcobacter Mu-like phage). Mu-like bacteriophage have been identified in other epsilonproteobacterial taxa, including *Campylobacter* (11 species), *Arcobacter* (3 species), and *Helicobacter bilis* ([93]; Miller and Mendoza, unpublished data). Some of these Mu-like phage are similar to CMLP1, but many show marked variation in gene content and gene sequence, indicating that CMLP1 and AMLP1 are members of a diverse Mu bacteriophage family common to Proteobacteria.

The genome of strain RM4018 also contains three small genomic islands, termed ABGI1, ABGI2 and ABGI3 (for *Arcobacter butzleri* genomic island). ABGI1 is 26,918 bp (bp 1,324,568-1,351,485) and contains 29 genes (*ab1330*-*ab1358*). ABGI2 is 15,973 bp (bp 1,703,320-1,719,292) and contains 13 genes (*ab1721*-*ab1733*). ABGI3 is 4,907 bp (bp 2,103,976-2,108,882) and contains eight genes (*ab2090*-*ab2097*). Additionally, ABGI1-3 are bordered by direct repeats of 21, 21 and 25 bp, respectively. The presence of genomic islands within the epsilon subdivision is not unusual. They have been identified in *C. jejuni* (CJIE2 and CJIE3; [52]), *H. hepaticus* (HHGI; [57]) and *H. pylori* (*cag*PAI; [58]). CJIE2 and CJIE3 are bounded by direct repeats, contain an integrase gene at one end, and have integrated into the 3′ end of a tRNA [52], [93]. Consistent with the *C. jejuni* islands, all three RM4018 genomic islands contain terminal integrase genes and ABGI1 has inserted into the 3′ end of a leucinyl-tRNA; however, ABGI2 and ABGI3 did not integrate into tRNAs, although ABGI3 is in close proximity to a cysteinyl-tRNA. CJIE3 has been proposed to be an integrated plasmid, based on the similarity of this element to the *C. coli* strain RM2228 megaplasmid [52], [93]; in contrast, none of the protein functions encoded by elements ABGI1-3 suggested a plasmid origin. Interestingly, ABGI2 does encode proteins similar to the Type I restriction enzymes HsdS and HsdM. Thus, the only Type I, II, or III restriction/modification enzymes present in strain RM4018 are encoded by a genomic island. The role of ABGI3 is unknown.

### Antibiotic resistance

*Arcobacter butzleri* strain RM4018 was resistant to 42 of the 65 antibiotics tested (see supplementary Table S4). This level of resistance is remarkably high, considering that the multi-drug-resistant *C. coli* strain RM2228 was resistant to only 33 of the same 65 antibiotics ([52] and Table S4). Strain RM4018 was resistant to all macrolides and sulfonamides tested and to all of the β-lactam antibiotics, with the exception of the β-lactam cephalosporin ceftazidime. Strain RM4018 had resistance to some quinolones, i.e. nalidixic acid and oxolinic acid, and also to chloramphenicol and 5-fluorouracil (5FU). The pattern of antibiotic resistance in strain RM4018 is consistent, in part, with the resistances of 39 *A. butzleri* strains tested against a smaller set of 23 antibiotics by Atabay and Aydin [98], with the exception that strain RM4018 was resistant to chloramphenicol and nalidixic acid. No plasmids were detected in strain RM4018; therefore all resistance mechanisms would be chromosomal in nature.

In many cases, the antibiotic resistance observed for strain RM4018 was due to the presence or absence of genes characterized previously in terms of antibiotic resistance. For example, chloramphenicol resistance is due most likely to the presence of a *cat* gene (*ab0785*), which encodes a chloramphenicol O-acetyltransferase. β-lactam resistance is due probably to the three putative β-lactamases (AB0578, AB1306 and AB1486) present in the RM4018 genome; β-lactam resistance may be enhanced also by the presence of the *lrgAB* operon (*ab0179*, *ab0180*) which modulates penicillin tolerance in *Staphylococcus* [99], [100]. Uracil phosphoribosyltransferase, encoded by the *upp* gene, catalyzes the first step in the pathway that leads to the production of the toxic analog 5-fluorodeoxyuridine monophosphate; mutations in the *upp* gene have been shown to lead to increased 5FU resistance [101], [102]. Thus, the absence of *upp* in *A. butzleri* strain RM4018 results presumably in high 5FU resistance.

Although strain RM4018 is resistant to some quinolones, mutations implicated previously in *Campylobacter* [103] and *Arcobacter* [104] quinolone resistance at Thr-86, Asp-90 and Ala-70 in the DNA gyrase subunit GyrA are not present in strain RM4018. It is probably not a coincidence that strain RM4018 is susceptible to hydrophilic quinolones (e.g. ciprofloxacin and norfloxacin) and resistant to hydrophobic ones (e.g. nalidixic acid and oxolinic acid). These data suggest that the mechanism of quinolone resistance in strain RM4018 is not at the level of the gyrase, but rather of uptake. Decreased quinolone uptake is associated with either increased impermeability or the activity of efflux pumps [105]. Hydrophobic quinolones alone can transit across the phospholipid bilayer but all quinolones can enter the cell through porins [105]. It is possible that the phospholipid bilayer of strain RM4018 has reduced permeability towards hydrophobic quinolones in conjunction with modifications in the porins to permit passage of only hydrophilic quinolones. A more likely scenario is the presence of a hydrophobic quinolone-specific efflux pump. Examples of pumps with specificity towards one class of quinolone are known; for example, the NorA protein of *Staphylococcus aureus* has been shown to be involved in the specific efflux of hydrophilic quinolones [106].

### Virulence determinants

Putative virulence determinants have been identified in *Campylobacter*, but little is known about potential virulence factors in *A. butzleri.* Since campylobacterioses and reported *A. butzleri*-related illnesses have similar clinical outcomes [10], it might be expected that some *C. jejuni* virulence factors would be found in *Arcobacter*. In fact, some virulence determinants identified in *C. jejuni*, have homologs within *A. butzleri*. For example, the fibronectin binding proteins CadF and Cj1349 have homologs in strain RM4018 (AB0483 and AB0070, respectively). Moreover, homologs of the invasin protein CiaB, the virulence factor MviN, the phospholipase PldA and the TlyA hemolysin are present in strain RM4018 (AB1555, AB0876, AB0859 and AB1846, respectively). However, it has not been determined if these putative virulence determinants are functional or if their function and role in *A. butzleri* biology is similar to the function of their *Campylobacter* homologs.

On the other hand, several *Campylobacter* virulence-associated genes are not present in the RM4018 genome. Most notably, the genes encoding the cytolethal distending toxin CDT (*cdtABC*) are absent from strain RM4018. CDT is an exotoxin which irreversibly blocks eukaryotic cells in the G_1_ or G_2_ phase [107]; *cdtABC* genes have been identified in *Helicobacter hepaticus* and several characterized *Campylobacter* genomes. The absence of *cdtABC* in strain RM4018 correlated well with a study by Johnson and Murano [108] which was unable to detect *cdt* genes in *Arcobacter* by PCR. *A. butzleri* strain RM4018 also contains no PEB1 or JlpA adhesin homologs [109], [110].

Analysis of the strain RM4018 genome identified two additional putative virulence determinants: *irgA* (*ab0729*) and *hecAB* (*ab0941*-*ab0940*). The *irgA* gene in *V. cholerae* encodes an iron-regulated outer membrane protein [111], and an IrgA homolog has been demonstrated to play a role in the pathogenesis of urinary tract infections by uropathogenic *E. coli* [112]. Adjacent to *irgA* is *ab0730*, which encodes a putative IroE homolog. IroE is a siderophore esterase found also in uropathogenic *E. coli* [113]. The other novel virulence determinant, HecA, is a member of the filamentous hemagglutinin (FHA) family, and *hecB* encodes a related hemolysin activation protein. FHA proteins are distributed widely among both plant and animal pathogens, e.g. HecA in *Erwinia crysthanthemi* contributes to both attachment and aggregation and is involved in epidermal cell killing [114]. Consistent with this distribution, RM4018 HecA homologs occur in both plant- (*Pseudomonas syringae*, *Ralstonia solanacearum*) and animal-pathogens (*Burkholderia cepacia*, *Acinetobacter spp*. and uropathogenic *E. coli*).

### Interacting with the environment: chemotaxis and signal transduction

Microbial life is characterized by continuous interactions between bacteria and their environment. The ability of microorganisms to monitor environmental parameters is a prerequisite for survival. Hence, bacteria have evolved different mechanisms such as sensory histidine kinases, methyl-accepting chemotaxis proteins, sigma(σ)/anti-sigma factor pairs, and adenylate and diguanylate cyclases to monitor and rapidly adapt to changes in their environment.

*Arcobacter butzleri* is well equipped with a large number of these systems, allowing it to survive in diverse ecological niches. Prime bacterial mechanisms of environmental adaptation and gene regulation are the two-component systems (2CS), consisting of a membrane-bound sensor histidine protein kinase (HPK) and a cytoplasmic response regulator (RR). *A. butzleri* has one of the highest densities of 2CS genes per megabase, with a total of 78 genes of this type (Table S2). Analysis of the genome sequence revealed 36 HPK genes, 41 RR genes, and one hybrid HPK/RR gene. On the genome, 31 pairs of HPK and RR genes are present that likely form functional 2CS involved in responses to environmental changes. In three instances, a more complex regulation may exist, where an additional sensor or response regulator gene is adjacent to the pair, namely AB0416, AB0417, AB0418; AB0453, AB0454, AB0455; and AB0795, AB0796, AB0797.

A fully functional chemotaxis system modulated by the 2CS CheA-CheY is present in *A. butzleri* strain RM4018. Its genome encodes a large number of putative chemotaxis proteins, many of which are novel in the epsilon subdivision. *A. butzleri* is predicted to have the CheA, CheY, CheV, CheW, CheR and CheB core enzymes, although most of these proteins have low similarity to their epsilonproteobacterial orthologs, and some have key differences in terms of domain composition. For instance, CheA in *Campylobacter* spp. is fused to a CheY-like response regulator (RR) domain [115] which is absent from *A. butzleri* CheA. Also, the methylesterase CheB in *A. butzleri* has both the C-terminal methylesterase and N-terminal RR domains whereas the *C. jejuni* CheB protein contains only the methylesterase domain [115]. The presence of this CheB RR domain may support adaptation in *A. butzleri*, allowing the cell to “sense” increasing concentrations of attractants. In addition to these core chemotaxis proteins, strain RM4018 is predicted to encode the CheC phosphatase, the CheD glutamine amidase, involved in maturation of the methyl-accepting chemotaxis proteins (MCPs), and two additional CheY proteins. CheC hydrolyzes phosphorylated CheY and is employed by multiple taxa as an alternative to CheZ in signal removal [116]. The additional CheY proteins also could modulate signal removal by serving as a “phosphate sink” [116]. In the chemotaxis network, the receptor and kinase functions are separated in order to allow the cell to execute an integrated response to multiple stimuli [117]. The elaborate sensing capabilities of *A. butzleri* are reflected also in its high number (N = 25) of chemotaxis-specific receptors (MCPs). Some MCPs, e.g. AB1764, contain Cache (Ca ^2+^ channels and chemotaxis) domains which have been identified in multiple bacterial taxa [118], and are similar to those found in animal voltage-gated Ca^2+^ channels. In addition, the periplasmic sensor region of AB1496 contains a putative nitrate/nitrite sensory domain, suggesting that nitrate and/or nitrite serves as an attractant for *A. butzleri*.

The limited similarity between the remaining non-chemotaxis-associated 2CS of *A. butzleri* and those of other bacteria do not allow functional prediction. However, six histidine kinases possess PAS domains, which are involved often in sensing changes in cellular energy levels, oxygen levels, or redox potential. Remarkable is the apparent lack of genes encoding homologs of the NtrC/NtrB family of two-component proteins that act together with the σ factor RpoN. Genes encoding RpoN as well as the flagellar sigma factor FliA are also missing in the *A. butzleri* genome. This indicates that the transcriptional regulation of the *A. butzleri* polar flagella machinery is completely different from that of other Epsilonproteobacteria where the 2CS FlgS/FlgR together with RpoN and FliA regulate the formation of the basal-body hook and filament complex [119], [120]. Regulation of *A. butzleri* flagella synthesis may involve a completely novel system, since also absent from the genome are genes encoding homologs of the lateral master flagella transcription factors, such as FlhC, FlhD of *E. coli* [121] or members of the LuxR-type of transcription regulators like *Sinorhizobium meliloti* VisNR [122].

There are no predicted alternate sigma factors in the *A. butzleri* genome. Instead, and unlike the genomes of *Campylobacter* spp. or *Helicobacter* spp., it contains seven ECF-family (extracytoplasmic function) σ/anti-σ factor pairs. Genes encoding these pairs are in tandem. Anti-σ factors are usually membrane-bound and prevent their cognate σ factor from associating with core RNA polymerase [123]. To date, only the *W. succinogenes* proteins WS1422 and WS1423 have been identified as an ECF-family σ factor pair within the epsilon subdivision. It is not known what genes are regulated in *A. butzleri* by these ECF-family σ factors. In other bacterial taxa, these ECF-family σ factors regulate functions such as alginate biosynthesis, iron-citrate transport, pyoverdine biosynthesis and cytochrome expression [123], and have been associated with virulence in *S. enterica*, *P. aeruginosa* and *M. tuberculosis* [124].

Finally, *A. butzleri* strain RM4018 encodes a class of proteins involved in formation and turnover of the “second messenger” bis-(3′-5′)-cyclic dimeric guanosine monophosphate (cyclic-di-GMP). In bacteria cyclic-di-GMP is synthesized by diguanylate cyclases containing a conserved GGDEF motif (GGDEF(DUF1) domain proteins) and degraded into diguanylate (pGpG) by phosphodiesterase proteins containing domains enriched in glutamic acid, alanine and leucine (EAL(DUF2) domain proteins) [125], [126]. The genome of strain RM4018 encodes 25 proteins, either membrane-associated or cytoplasmic/periplasmic, containing GGDEF and/or EAL domains (Figure S2b). In other bacteria, GGDEF/EAL domain proteins have been implicated in biofilm formation [127], [128], motility [129], [130] and virulence [131].

It is likely that many of the signal transduction proteins described above interact across group boundaries, e.g. GGDEF proteins with chemotaxis proteins. Thus, taken together, the large repertoire of 2C systems, chemotaxis proteins, ECF sigma factors and cyclic-di-GMP factors suggest the presence of a truly complex signal transduction network.

### Genomic comparisons with other *A. butzleri* strains

The genomic diversity of 13 *A. butzleri* strains, of both human and animal origin, was examined by microarray-based comparative genomic indexing (CGI) analysis. A list of the strains used in the CGI analysis is presented in Table 4. The CGI analysis allowed the assessment of CDS content for each *A. butzleri* strain relative to the *A. butzleri* DNA microarray, which comprises 2238 CDS from strain RM4018. Genomic DNA from strain RM4018 was used as a reference DNA and competitively hybridized with genomic DNA from each of the other *A. butzleri* strains. The GENCOM software described previously [132], [133] was used to assign the CDS as present, absent or multicopy for each *A. butzleri* strain. The complete CGI data sets as trinary scores (present = 1; absent = 0; and multicopy = 2) are available in supplementary Table S5. It was observed that 74.9% (1676 of 2238) of the CDS represented on the microarray were present in all *A. butzleri* strains, and served to define approximately the core genes of *A. butzleri* (supplementary Table S6).

**Table 4:** Strains used in the comparative genomic indexing analysis.

Strain	Description	Location	Source
RM4018	ATCC 49616, type strain	USA (Calif.)	Human diarrheal stool
RM1588	NADC 5276	USA	Chicken
RM1591	NADC 5377	USA	Turkey carcass
RM4128	29.97	South Africa	Human stool
RM4462	NADC 3553	USA (Tex.)	Human stool
RM4467	NADC 3566	USA	Primate rectal swab
RM4596	NADC 5262	USA (Iowa)	Turkey
RM4843	NADC 5278	USA (Iowa)	Chicken carcass
RM4850	NADC 6830	USA (Iowa)	Horse
RM5516	NADC 3156	USA (Iowa)	Pig
RM5538	CDC D2725	USA (Mass.)	Human stool
RM5541	CDC D2901	USA (Colo.)	Human stool
RM5544	CDC D2778	Thailand	Human stool

All test strains selected had unique MLST sequence types (data not shown).

To gain more information concerning how genomic diversity may affect the physiology of these *A. butzleri* strains, the *A. butzleri* CDS were grouped into defined functional categories. Genes in each functional category for the *A. butzleri* RM4018 genome were analyzed against the CGI data set with GeneSpring software. The results show that all *A. butzleri* strains in this study possessed every gene in our data set assigned to the functional categories: polyamine biosynthesis; purines, pyrimidines, nucleosides and nucleotides biosynthesis; aminoacyl tRNA synthetases and tRNA modification; protein translation and modification; and protein and peptide secretion (Table 5). The results demonstrated also that more than 95% of the genes in our data set assigned to the following functional categories were present for all strains: energy metabolism; amino acid biosynthesis; and ribosomal protein synthesis, modification and maturation (Table 5). In contrast, functional categories with less than 70% of the genes present in at least two strains were: central intermediary metabolism; transcriptional regulation; surface polysaccharides, lipopolysaccharides, and antigens; surface structures; phage-related functions and prophage; and hypothetical proteins (Table 5). There were also 42 CDS that were found only in strain RM4018 (Table S7), including the Sox cluster of seven genes (*soxXYZABCD*), suggesting that this system was acquired by RM4018. Some genes within the bacteriophage AMLP1 were present in the other 12 *A. butzleri* strains, but this prophage was not present in its entirety in any of the other 12 *A. butzleri* strains. The same applies to the small genomic islands ABGI1 and ABGI2. Finally, urea degradation does not appear to be a core function of *A. butzleri,* considering that only six of the 12 strains possessed the gene clusters *ab0802*-*ab0806* and *ab0808-ab0813*.

**Table 5:** Assignment of absent CDS to functional categories of *A. butzleri*.

	Genes in data set	Absent genes (%)^a^
**I. Small molecule metabolism**		
I.A Degradation	12	8.3
I.B Energy metabolism	129	3.8
I.C Central intermediary metabolism^b^	37	40.5
I.D Amino acid biosynthesis	85	3.5
I.E Polyamine biosynthesis	4	0
I.F Purines, pyrimidines, nucleosides and nucleotides	41	0
I.G Biosynthesis of cofactors, prosthetic groups and carriers	96	7.3
I.H Fatty acid biosynthesis	17	11.8
**II. Broad regulatory functions**	12	8.3
II.A Transcriptional regulation	51	33.3
II.B Signal transduction	104	23.1
**III. Macromolecule metabolism**		
III.A Synthesis and modification of macromolecules	240	7.1
III.A.2-3 Ribosome and ribosomal protein synthesis, modification and maturation	63	4.8
III.A.5 Aminoacyl tRNA synthetases and tRNA modification	53	0
III.A.7 DNA replication, restriction/modification, repair, and recombination	69	13
III.A.8 Protein translation and modification	27	0
III.A.9 RNA synthesis, and RNA modification, and DNA transcription	18	5.6
III.A.11 Phospholipids	11	27.2
III.B Degradation of macromolecules	45	8.9
III.C Cell envelope		
III.C.1 Membrane proteins, lipoproteins, and porins	93	16.1
III.C.2 Surface polysaccharides, lipopolysaccharides, and antigens	57	36.8
III.C.3 Surface structures	41	34.1
III.C.4 Murein sacculus and peptidoglycan	21	9.5
**IV. Cell processes**		
IV.A Transport/binding proteins	169	24.9
IV.B Chaperones	16	12.5
IV.C Cell division	14	7.1
IV.D Chemotaxis and mobility	46	21.7
IV.E Protein and peptide secretion	21	0
IV.G Detoxification	9	11.1
IV.I Pathogenicity	12	25
IV.J DNA uptake/competence	8	12.5
**V. Other**		
V.A Phage-related functions and prophage	54	96.3
V.D Drug/analog sensitivity and antibiotic resistance	28	17.9
V.F Adaptations and atypical conditions	9	11.1
**VI. Miscellaneous proteins, general function proteins, and hypothetical proteins**		
VI.A Miscellaneous/General function	143	16.1
VI.B Domain of unknown function (DUF) proteins	61	9.8
VI.C Conserved hypothetical proteins-no conserved domains	303	19.9
VI.D Hypothetical proteins	262	39.7

a Genes absent in at least 2 of 12 additional strains of *A. butzleri*.

b Primarily *sox* and *ure* genes.

### Conclusions

The human pathogen *A. butzleri* is a member of the family *Campylobacteraceae* in the epsilon subdivision of the Proteobacteria, and it was found, based on 16S rDNA sequence similarity, to be most closely related to the Campylobacters. However, based on complete genome analysis, *A. butzleri* appeared more closely related to non-*Campylobacteraceae* taxa, specifically *S. denitrificans, W. succinogenes, Sulfurovum* and *Nitratiruptor*. This unexpected finding may be less surprising considering the limited number of completed epsilonproteobacterial genomes. Considering that to date the Epsilonproteobacteria genome sequences annotated belong in their majority to pathogens, further refinements of the structure of the epsilonproteobacterial taxon are likely to emerge, as additional genomes of this subdivision are sequenced, especially those of other *Arcobacter* species, and of related genera such as *Sulfurospirillum*.

Even though *A. butzleri* has a large number of *S. denitrificans*, *W. succinogenes*, *Sulfurovum* and *Nitratiruptor* orthologs, strain RM4018 has several features in common with *Campylobacter*, such as the inability to utilize sugars as carbon sources and the presence of defined LOS/LPS loci. Interesting differences between *A. butzleri* and the Campylobacters are the absence of Type I, II or III restriction enzymes and the absence of polynucleotide G:C tracts. These tracts in *Campylobacter* are found often in LOS and capsular loci, and changes in the lengths of these tracts could modify the surface structure of the organism, perhaps altering the serotype. Therefore, the absence of these tracts and the resulting absence of contingency genes may disadvantage *A. butzleri* as a pathogen. Alternatively, the absence of restriction enzymes may facilitate the uptake and incorporation of phage, genomic islands, and novel genes/loci, as seen in strain RM4018, thus providing the genomic plasticity that is associated often with virulence.

Notwithstanding its relationships to *Campylobacter* and *Helicobacter*, *A. butzleri* is probably an environmental organism that may cause disease through either water-mediated food contamination or ingestion of *A. butzleri*-contaminated water, and whose biology is not associated tightly with any particular host or hosts. *A. butzleri* is more psychrophilic than *Campylobacter* spp. and may not proliferate in some hosts, especially avian hosts, to the same extent as, e.g. *C. jejuni*, resulting in *A. butzleri*-related food-borne illnesses occurring less frequently. The enhanced ability to survive in the environment, compared to such taxa as *Campylobacter*, is due most likely to the genes and systems described in this study, such as the increased number of respiratory enzymes, chemotaxis proteins and two-component systems. *A. butzleri* has a number of genes that confer increased environmental survival and the ability to grow under a wider range of atmospheric conditions and lower temperatures. An example of the first would be the photoreactivating lyase *phr*, which reduces DNA damage induced by UV radiation, a condition encountered frequently by environmental organisms. Examples of the second would include: 1) more oxygen-stable enzymes (e.g. pyruvate dehydrogenase), 2) a larger repertoire of TCA cycle enzymes active over a greater range of oxygen concentrations, 3) novel cold shock and stress proteins and 4) proteins such as the fatty acid cis/trans isomerase, Cti, which modulate membrane fluidity.

Although *A. butzleri* has been isolated from clinical diarrheal stool samples, the association of this organism with human illness is not as defined as the association of *C. jejuni* and human gastroenteritis. Obviously, the presence of *Campylobacter* virulence determinant homologs in strain RM4018 is not predictive of the pathogenicity of this strain; however, it is consistent with such a designation. It is likely, if *A. butzleri* is indeed pathogenic, that additional uncharacterized virulence determinant genes are present within the strain RM4018 genome. Therefore, additional investigations, using in part the genomic information presented here, will be required to elucidate further the mechanisms supporting the pathogenicity of *A. butzleri*. Indeed, the genome sequence and analyses presented in this study are an important first step in understanding the physiology and genetics of this organism, which constitutes a bridge between the environment and mammalian hosts.

## Materials and Methods

### Growth conditions and chemicals

All *Arcobacter* strains were cultured routinely at 28°C on Brain Heart Infusion agar (Becton Dickinson, Sparks, MD) amended with 5% (v/v) laked horse blood (Hema Resource & Supply, Aurora, OR). The incubation atmosphere for all strains was 5% H_2_, 10% CO_2_, and 85% N_2_. PCR enzymes and reagents were purchased from New England Biolabs (Beverly, MA) or Epicentre (Madison, WI). All chemicals were purchased from Sigma-Aldrich Chemicals (St. Louis, MO) or Fisher Scientific (Pittsburgh, PA). DNA sequencing chemicals and capillaries were purchased from Applied Biosystems (Foster City, CA).

### Genomic DNA preparation and library construction

A sample of the *Arcobacter butzleri* type strain ATCC 49616 was analyzed by two methods: 1) an *Arcobacter* multiplex speciation PCR [134] and 2) amplification and sequencing of the 16S rDNA locus, using the primers 16ARCO-1 (5′ ACA ATG GAC GAA AGT CTG AT 3′) and 16ARCO-2 (5′ CGC AAT CGG TAT TCC TTC 3′). Results of these tests indicated that the type strain isolate was a mixed culture containing an uncharacterized *A. cryaerophilus* strain. Therefore, a phosphate-buffered saline suspension of the type strain isolate was sonicated and dilution plated. Eight well-isolated colonies were picked and tested using the above two methods. Both tests confirmed that the DNA from each of the eight colonies was from a pure culture of *A. butzleri* and one purified isolate was given the new designation of RM4018. Moreover, the multilocus sequence type of strain RM4018 was identical to the sequence types of multiple isolates of strain ATCC 49616, obtained from independent laboratories (W. Miller, manuscript in preparation).

*Arcobacter* genomic DNA was prepared as described previously [135]. The genomic DNA was checked for high molecular weight using pulsed field gel electrophoresis, quantified using a PicoGreen assay (Invitrogen, Carlsbad, CA), and then sheared physically to the desired average sizes of approximately 4.0 kb and 40.0 kb, for the small- and large-fragment libraries, respectively. The 3–4 kb plasmid insert library was constructed in the pAGEN proprietary vector system (Agencourt). A large-insert fosmid library was constructed in the CopyControl pCC1FOS vector system (Epicentre, Madison, WI). Libraries were not amplified and were kept as ligation mixtures.

Initial quality control of each library included quantification of: the titer of each one, the frequency of recombinant/insert-less clones and the average insert size (using a subset of 20 recombinant clones). Prior to high throughput sequencing, 384 clones were picked and sequenced bi-directionally. A BLAST analysis was performed on the 768 sequencing reads to verify the integrity of the library. Sequence data was further reviewed to provide more precise information on the percentage of recombinant clones.

### Template preparation

Solid Phase Reversible Immobilization (SPRI) [136], [137] technology was used to purify the templates. SPRI technology uses carboxylate-coated, iron-core, paramagnetic particles to capture DNA of a desired fragment length based on tuned buffering conditions. Once the desired DNA is captured on the particles, they can be concentrated magnetically and separated so that contaminants can be washed away.

High-copy plasmid templates (insert size range 800 bp–4 kb) were purified using a streamlined SprintPrep SPRI protocol. Plasmid DNA was harvested directly from lysed bacterial cultures by trapping both plasmid and genomic DNA with the functionalized bead particles and selectively eluting only the plasmid DNA. Briefly, the purification procedure involves addition of alkaline lysis buffer (containing RNase A) to the bacterial culture, addition of an alcohol-based precipitation reagent including paramagnetic particles, separation of the magnetic particles using custom ring-based magnetic separator plates, 5× washing of the beads with 70% EtOH and elution of the plasmid DNA with water. The fosmid templates were purified using a SPRI protocol optimized to accommodate larger growth volumes and increased sample recovery needed for the low copy nature of these vectors.

### Polymerase chain reactions

Standard amplifications were performed on a Tetrad thermocycler (Bio-Rad, Hercules, CA) with the following settings: 30 s at 94°C; 30 s at 53°C; 2 min at 72°C (30 cycles). Each amplification mixture contained 50 ng genomic DNA, 1× PCR buffer (Epicentre), 1× PCR enhancer (Epicentre), 2.5 mM MgCl_2_, 250 µM each dNTP, 50 pmol each primer, and 1 U polymerase (New England Biolabs). Amplicons were purified on a BioRobot 8000 Workstation (Qiagen, Santa Clarita, CA). Sequencing and PCR oligonucleotides were purchased from Qiagen or MWG-Biotech (High Point, NC).

### DNA sequencing

DNA templates from shotgun clones were sequenced in a 384-well format. Cycle sequencing reactions were performed on a 96-well or 384-well Tetrad thermocycler (Bio-Rad, Hercules, CA) using the ABI PRISM BigDye terminator cycle sequencing kit (version 3.1) and standard protocols. Shotgun template extension products were purified using the CleanSeq dye-terminator removal kit (Agencourt) and amplicon extension products were purified using DyeEx 96 well plates (Qiagen) according to the manufacturer's protocols. DNA sequencing was performed on an ABI PRISM 3130XL or ABI PRISM 3730 Genetic Analyzer (Applied Biosystems) using the POP-7 polymer and ABI PRISM Genetic Analyzer Data Collection and ABI PRISM Genetic Analyzer Sequencing Analysis software. Shotgun reads were processed using Phred base calling software [138], [139] and monitored constantly against quality metrics using the Phred Q20. The quality scores for each run were monitored through a proprietary Galaxy LIMS system (Agencourt). Any substantial deviation from the normal range was investigated immediately.

### Contig assembly

Shotgun reads were assembled using the Paracel GenomeAssembler (Paracel, Pasadena, CA) with default program parameters and quality scores. To assist in the final assembly and to monitor read quality, Paracel-derived contig sequences and their associated reads were entered into SeqMan Pro (v7.0, DNAstar, Madison, WI). Within each contig, all reads were then trimmed for quality at the 5′ and 3′ ends. Those reads that did not meet minimum quality requirements were discarded. A large number of sequence and contig gaps were present after assembly of the initial shotgun reads. Arrangement of these contigs into a final single contig used both standard PCR and combinatorial long and standard PCR to bridge the contig gaps; due to low sequence similarity and minimal synteny, existing epsilonproteobacterial genome sequences could not be used as a scaffold for the *A. butzleri* genome. All gaps, both sequence and contig, present in the assembly were closed by PCR using primers designed with Primer Premier (v 5.0, Premier Biosoft, Palo Alto, CA). The *A. butzleri* strain RM4018 genome contains five nearly identical ribosomal RNA (rRNA) loci of ∼6 kb. Each locus was amplified independently using unique flanking primer sets and long PCR. rRNA region amplicons were sequenced, using rRNA primers common to all five loci, and assembled. The assembled rRNA loci were then integrated into the larger genome assembly manually. Other large repeat regions were analyzed similarly.

The final assembly contained 27,377 reads: 12,990 small insert reads (average insert size ≅ 4200 bp), 7,364 large insert reads (average insert size ≅31,650 bp) and 7,023 PCR-based reads. The final assembly contained also contiguous sequences (>2× coverage/nt) on both strands for an average coverage of 5.5×; ambiguous bases were re-sequenced at least twice. Validation of the final assembly was achieved by comparison of mock in silico digestion patterns with recently published PFGE patterns using the type strain of *A. butzleri* [140]. Although the PFGE band sizes were on average ∼10% larger than those predicted in silico, there was an almost 100% correlation between the two banding patterns.

### Genome analysis

Open reading frames were predicted using ORFscan (Rational Genomics, South San Francisco, CA) with the parameters set to include all three potential start codons (ATG, GTG and TTG) and a coding sequence (CDS) cut-off of 70 amino acids. Spurious CDSs (e.g. CDSs contained within larger CDSs) were deleted manually. Initial annotation of the putative CDSs was accomplished by comparing the predicted proteins to the nr database using BLASTP. The list of putative CDSs was then used to create a preliminary GenBank-formatted (.gbk) file that was entered into Artemis [141], [142]. Annotation within Artemis included the fusion of split CDSs into pseudogenes and the identification of small (usually ribosomal protein) genes overlooked in the initial ORFscan analysis. The start codon of each putative CDS was curated manually, either through visual inspection within Artemis of the ribosome binding site, or through BLAST comparison of each CDS to its epsilonproteobacterial homologs, where present; CDSs with dubious ribosome binding sites, lengths shorter than 200 bp and a low BLASTP score (*E*>10^−10^) were removed from the final list of CDSs. tRNAs were annotated using tRNAscan-SE [143], [144]. 16S and 23S rRNA loci were identified through BLASTN comparison to *Campylobacter* 16S and 23S loci; 5S rRNA loci were identified through BLASTN comparison to *Helicobacter* 5S loci.

Final annotation was performed using 1) BLASTP comparison to proteins in the NCBI nr database, 2) CDS identification, COG function prediction, cellular location prediction, and identification of Pfam domains and PROSITE motifs using the BASys Bacterial Annotation System [145] and 3) additional Pfam domain identification using the Sanger Centre Pfam search engine [146]. Four levels of gene assignment were used for each CDS: 1) CDSs predicted to encode proteins with high similarity across multiple taxa to proteins of known function (e.g. TrpF, RpsL, PurE) were assigned that function, 2) CDSs predicted to encode proteins with only a general function, but similar to members of various Pfam families or superfamilies, or containing various PROSITE motifs, were annotated to reflect the tentative general function (e.g. topoisomerase, ABC transporter, glycosyl transferase), 3) CDSs predicted to encode proteins with no defined function or motifs but similar to other non-defined proteins (*E*<10^−10^) across multiple bacterial taxa were designated as ‘conserved hypothetical’, and 4) CDSs predicted to encode proteins with no defined function and either unique to this genome or with a low similarity (*E*>10^−10^) to proteins from other taxa were designated as ‘hypothetical’. The complete nucleotide sequence and annotation of the *A. butzleri* strain RM4018 has been deposited at GenBank under the accession number CP000361.

### Construction of the *A. butzleri* DNA microarray

DNA fragments of individual ORFs within *A. butzleri* strain RM4018 were amplified using primers designed with ArrayDesigner 3.0 (Premier Biosoft, Palo Alto, CA) and purchased from MWG Biotech Inc. (High Point, NC). Each PCR reaction (total reaction volume, 100 µl) consisted of 1× MasterAmp *Taq* PCR buffer, 1× MasterAmp *Taq* Enhancer, 2.5 mM MgCl_2_, 200 µM each dNTP, forward and reverse primers at 0.2 µM each, 0.5 U of MasterAmp *Taq* DNA polymerase (Epicentre), and approximately 50 ng of genomic DNA from *A. butzleri* strain RM4018. Thermal cycling was performed using a Tetrad thermal cycler (Bio-Rad, Hercules, CA) with the following amplification parameters: 30 cycles of 25 s at 94°C, 25 s at 52°C, and 2 min at 72°C and a final extension at 72°C for 5 min. PCR products were analyzed by gel electrophoresis in a 1% (w/v) agarose gel in 1× Tris-borate-EDTA buffer and stained with ethidium bromide. The DNA bands were examined under UV illumination. We amplified successfully a total of 2238 PCR products from *A. butzleri* strain RM4018. These PCR products were purified on a Qiagen 8000 robot using a Qiaquick 96-well Biorobot kit (Qiagen, Valencia, CA), dried, and resuspended to an average concentration of 0.1–0.2 µg µl^−1^ in 20 µl of 50% dimethyl sulfoxide (DMSO) containing 0.3× Saline-Sodium Citrate (SSC). All of the PCR probes were then spotted in duplicate on UltraGAPS slides (Corning) using an OmniGrid Accent (GeneMachines, Ann Arbor, MI) producing a final array that contained a total of 4476 features. The DNA was UV cross-linked to the microarray slides with a Stratalinker at 300 mJ (Stratagene, La Jolla, CA).

### Preparation and fluorescent labeling of genomic DNA

Genomic DNA from *A. butzleri* was isolated as described previously [92]. For each microarray hybridization reaction, genomic DNA from the *A. butzleri* reference strain RM4018 and a test strain of *A. butzleri* were labeled fluorescently with indodicarbocyanine (Cy5)-dUTP and indocarbocyanine (Cy3)-dUTP, respectively. An aliquot (2 µg) of DNA was mixed with 5 µl 10× NEBlot labeling buffer containing random sequence octamer oligonucleotides (NEB, Beverly, MA.) and water to a final volume of 41 µl. This mixture was heated to 95°C for 5 min and then cooled for 5 min at 4°C. After this time, the remainder of the labeling reaction components were added: 5 µl of 10× dNTP labeling mix (1.2 mM each dATP, dGTP, dCTP; 0.5mM dTTP in 10 mM Tris pH 8.0; 1 mM EDTA), 3 µl of Cy3 dUTP or Cy5 dUTP (GE Biosciences, Piscataway, NJ) and 1 µl of Klenow fragment. The labeling reactions were incubated overnight at 37°C. Labeled DNA was purified from unincorporated label using Qiaquick PCR Cleanup kits. Labeling efficiencies were determined using a NanoDrop ND-1000 (NanoDrop, Wilmington, DE) and efficiently-labeled samples were dried by vacuum.

### Microarray hybridization

Labeled reference and test DNAs were combined in 45 µl Pronto! cDNA hybridization solution (Corning, Corning, NY) and heated to 95°C for 5 min. Then, 15 µl of the hybridization mixture was put onto a microarray slide and sealed with a cover slip. The microarray slide was placed in a hybridization chamber (Corning) and incubated at 42°C for 18 h. Following hybridization, the slides were washed twice in 2× SSC, 0.1% sodium dodecyl sulfate at 42°C for 10 min, followed by twice in 1× SSC at room temperature for 10 min, and finally twice in 0.2× SSC at room temperature for 5 min. The microarray slides were dried by centrifugation at 300×*g* for 10 min before scanning. At least two hybridization reactions were performed for each test strain.

### Microarray data analysis

Microarrays were scanned using an Axon GenePix 4000B microarray laser scanner (Axon Instruments, Inc. Union City, CA) as described previously [93]. Features (spots) and the local background intensities were detected and quantified with GenePix 4.0 software (Axon Instruments, Inc.). The data were filtered so that spots with a reference signal lower than the background plus 2 standard deviations of the background were discarded. Signal intensities were corrected by subtracting the local background, and then the Cy5/Cy3 ratios were calculated. The method for comparative genomic indexing (CGI) analysis utilized the GENCOM software described previously [132], [133] to assign present and absent CDS of *A. butzleri* strains when compared to reference strain RM4018. The Cy5 and Cy3 signal intensities were corrected by subtracting the local background before submission into the GENCOM program. GENCOM analysis was performed for each hybridization and the present/absent assignments for each CDS as determined by GENCOM were converted into scores (present = 1; absent = 0). If the CDS was assigned absent for the reference strain, this indicates that the test strain hybridized better than the reference. Considering all the CDS are from the reference strains, this may indicate that the CDS is in multicopy in the test strain. The CDS assigned absent for the reference strain, were given a score of 2 to indicate that the CDS may be in multicopy. The trinary CDS scores for all strains were analyzed further with GeneSpring microarray analysis software version 7.3 (Agilent Technologies, Redwood City, CA) and subjected to average-linkage hierarchical clustering with the standard correlation and bootstrapping. Microarray data have been deposited in the NCBI GEO repository (http://www.ncbi.nlm.nih.gov/geo/) with the accession number GSE8725.

## Supporting Information

Figure S1 Diagram of the *A. butzleri* strain RM4018 genome. Genes and features are drawn to scale. Genes are colored according to role category. tRNA and rRNA loci are included. Prophage and genetic islands are shaded in grey.
(0.17 MB PDF)

Figure S2 Selected *A. butzleri* RM4018 signal transduction proteins. Diagrammatic representation of the Non-Che chemotaxis proteins and GGDEF domain diguanylate cyclases/EAL domain c-di-GMP phosphodiesterases predicted to be encoded by strain RM4018. Relevant motifs and transmembrane helices are indicated and drawn to scale.
(0.03 MB PDF)

Table S1 *Arcobacter butzleri* predicted open reading frames and their functional annotation. Complete list of *A. butzleri* strain RM4018 genes, tRNA loci and rRNA loci. Start and end coordinates are provided for each feature along with its functional annotation.
(1.43 MB PDF)

Table S2 Functional prediction of *Arcobacter butzleri* genes: similarity to proteins from other epsilonproteobacteria. *A. butzleri* strain RM4018 genes are sorted by functional category. The % amino acid identity of each protein to homologs in other epsilonproteobacteria is listed.
(3.33 MB PDF)

Table S3 Similarity of *Arcobacter butzleri* proteins to proteins from other taxa. The five best homologs (where applicable) for each protein in strain RM4018 are listed, as well as the Expect (E) value, % identity and % similarity.
(4.71 MB PDF)

Table S4 Antibiotic resistance profiles of strain RM4018 and selected Campylobacters. Comparison of the antibiotic resistance profiles of *A. butzleri* strain RM4018, *C. jejuni* strain NCTC 11168, *C. coli* strain RM2228, *C. lari* strain RM2100 and *C. upsaliensis* strain RM3195.
(0.02 MB PDF)

Table S5 Trinary CGI data as determined with GENCOM. Trinary *A. butzleri* DNA microarray data for the reference strain RM4018 and the 12 additional *A. butzleri* tester strains.
(0.91 MB XLS)

Table S6 CDS contained in all *A. butzleri* strains examined. Core set of 1676 genes identified in RM4018 and all 12 additional *A. butzleri* strains by comparative genomic indexing. The functional annotation of each gene is provided.
(0.51 MB PDF)

Table S7 CDS present only in *A. butzleri* strain RM4018. Set of 42 genes identified by comparative genomic indexing of 13 *A. butzleri* strains to be present only in strain RM4018. The functional annotation of each gene is provided.
(0.02 MB PDF)
