# Supplementary figures and images for: The Complete Genome Sequence and Analysis of the Epsilonproteobacterium Arcobacter butzleri
Source: PLoS One. 2007 Dec 26;2(12):e1358. doi: 10.1371/journal.pone.0001358 (PMC2147049; doi:10.1371/journal.pone.0001358)

Arcobacter butzleri RM4018 (2341251 bp)

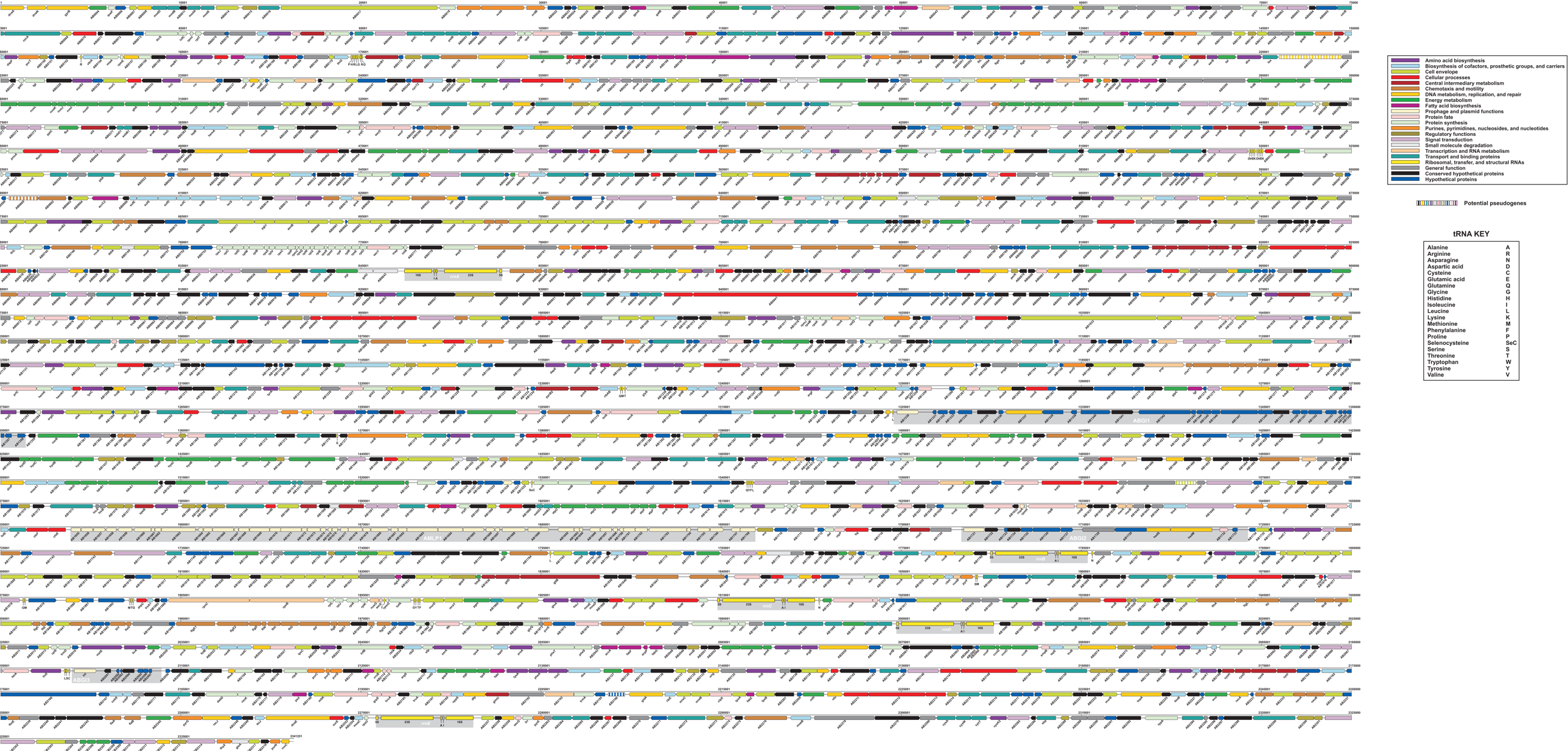

Supplement: Figure S1 — Diagram of the A. butzleri strain RM4018 genome. Genes and features are drawn to scale. Genes are colored according to role category. tRNA and rRNA loci are included. Prophage and genetic islands are shaded in grey. (0.17 MB PDF) [file pone.0001358.s001.pdf]
