# Supplementary material for: The Complete Genome Sequence and Analysis of the Epsilonproteobacterium Arcobacter butzleri
Source: PLoS One. 2007 Dec 26;2(12):e1358. doi: 10.1371/journal.pone.0001358 (PMC2147049; doi:10.1371/journal.pone.0001358)
Supplement: Figure S2 — Selected A. butzleri RM4018 signal transduction proteins. Diagrammatic representation of the Non-Che chemotaxis proteins and GGDEF domain diguanylate cyclases/EAL domain c-di-GMP phosphodiesterases predicted to be encoded by strain RM4018. Relevant motifs and transmembrane helices are indicated and drawn to scale. (0.03 MB PDF) [file pone.0001358.s002.pdf]

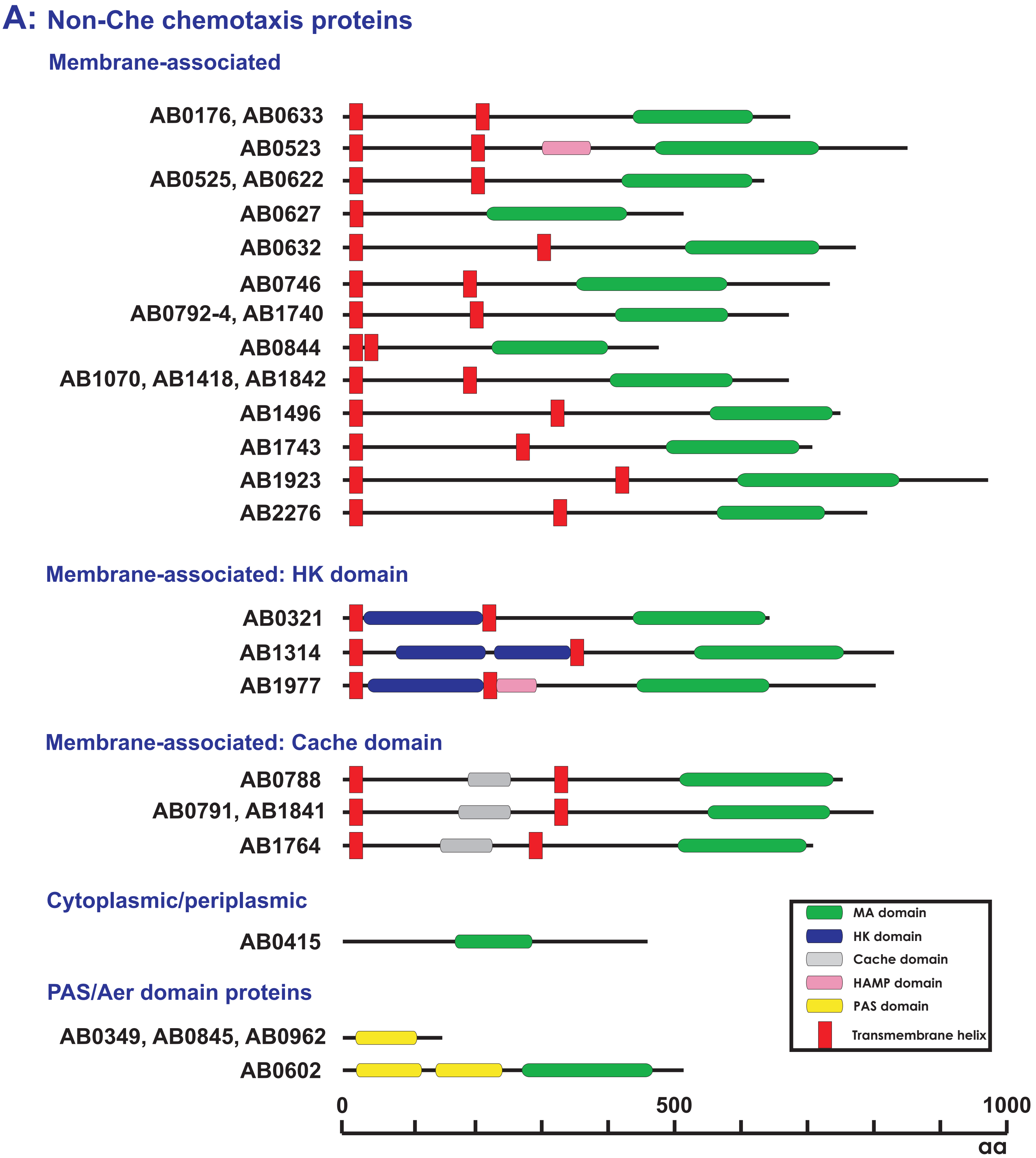
A: Non-Che chemotaxis proteins
Membrane-associated
AB0176, AB0633
AB0523
AB0525, AB0622
AB0627
AB0632
AB0746
AB0792-4, AB1740
AB0844
AB1070, AB1418, AB1842
AB1496
AB1743
AB1923
AB2276
Membrane-associated: HK domain
AB0321
AB1314
AB1977
Membrane-associated: Cache domain
AB0788
AB0791, AB1841
AB1764
Cytoplasmic/periplasmic
AB0415
PAS/Aer domain proteins
AB0349, AB0845, AB0962
AB0602
MA domain
HK domain
Cache domain
HAMP domain
PAS domain
Transmembrane helix
0
500
1000
aa

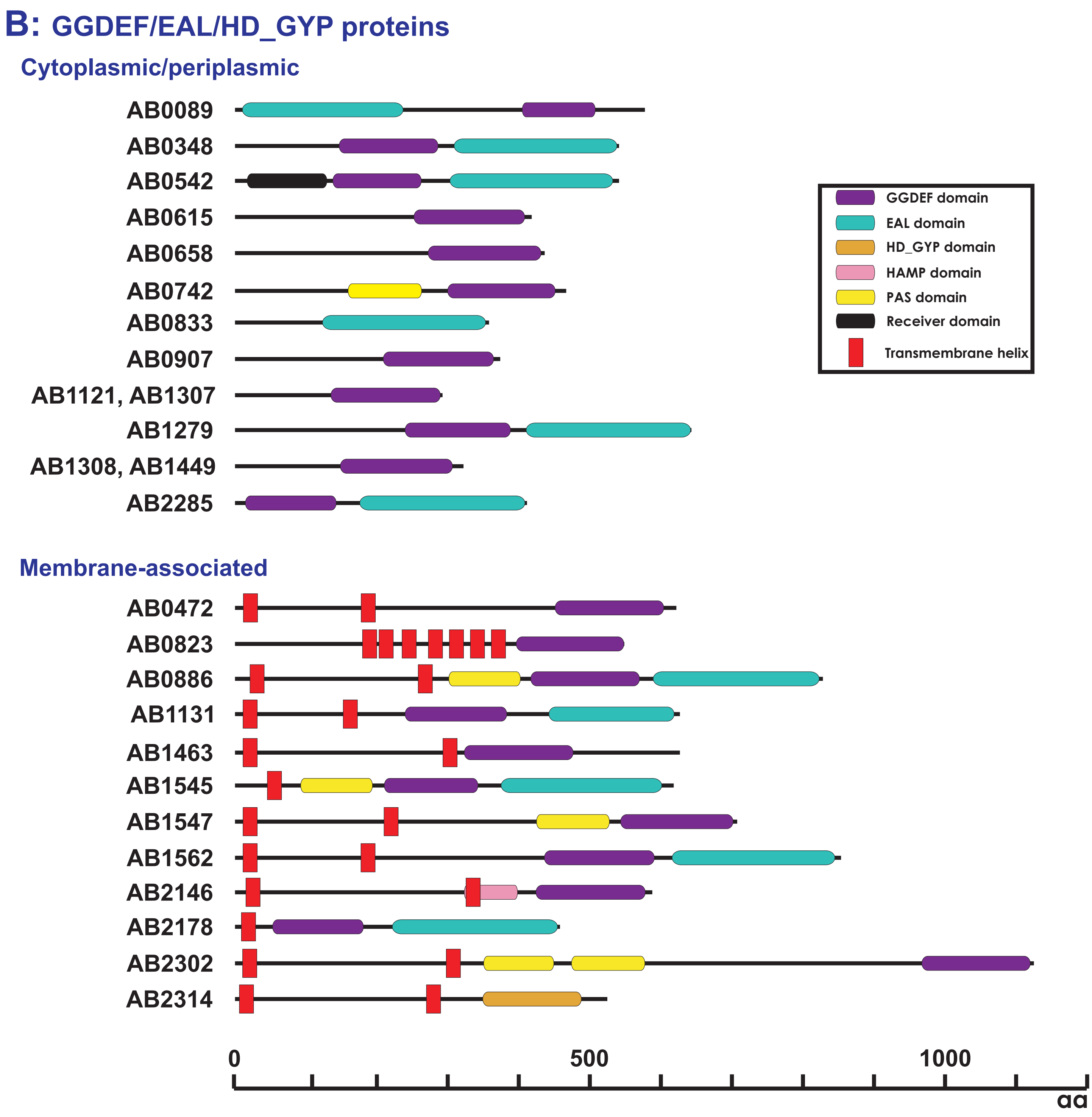

B: GGDEF/EAL/HD_GYP proteins
Cytoplasmic/periplasmic
AB0089
AB0348
AB0542
AB0615
AB0658
AB0742
AB0833
AB0907
AB1121, AB1307
AB1279
AB1308, AB1449
AB2285
Membrane-associated
AB0472
AB0823
AB0886
AB1131
AB1463
AB1545
AB1547
AB1562
AB2146
AB2178
AB2302
AB2314
GGDEF domain
EAL domain
HD_GYP domain
HAMP domain
PAS domain
Receiver domain
Transmembrane helix
0
500
1000
aa
