# Supplementary material for: The Complete Genome Sequence and Analysis of the Epsilonproteobacterium Arcobacter butzleri
Source: PLoS One. 2007 Dec 26;2(12):e1358. doi: 10.1371/journal.pone.0001358 (PMC2147049; doi:10.1371/journal.pone.0001358)
Supplement: Table S1 — Arcobacter butzleri predicted open reading frames and their functional annotation. Complete list of A. butzleri strain RM4018 genes, tRNA loci and rRNA loci. Start and end coordinates are provided for each feature along with its functional annotation. (1.43 MB PDF) [file pone.0001358.s003.pdf]

Table S1. ***Arcobacter butzleri*** **predicted open reading frames and their functional annotation**

ORF	Start	End	Len (AA)	Strand	Gene	Functional annotation
AB0001	1	1314	438	+	*dnaA*	Chromosomal replication initiator protein DnaA
AB0002	1475	2542	356	+	*dnaN*	DNA polymerase III, beta subunit
AB0003	2567	4885	773	+	*gyrB*	DNA gyrase, subunit B
AB0004	4900	5868	323	-		Probable thioredoxin reductase
AB0005	5984	6364	127	+	*queF*	7-cyano-7-deazaguanine reductase
AB0006	6377	6961	195	+		Hypothetical protein
AB0007	7173	7577	135	+		Conserved hypothetical protein, putative integral membrane protein
AB0008	7576	8379	268	-		Conserved hypothetical protein, possible methyltransferase
AB0009	8384	9235	284	-	*modD*	Molybdenum ABC transporter, ATP-binding protein
AB0010	9225	9929	235	-	*modB*	Molybdenum ABC transporter, permease protein
AB0011	9925	10323	133	-		Conserved hypothetical protein
AB0012	10323	11066	248	-	*modA*	Molybdenum ABC transporter, periplasmic molybdate-binding protein
AB0013	11079	11858	260	-	*modE*	Molybdenum-binding protein, N-terminal:molybdenum-pterin binding domain
AB0014	11985	13226	414	+		Conserved hypothetical membrane protein
AB0015	13402	14226	275	-		Transcriptional regulator, LysR family
AB0016	14324	15499	392	+		Conserved hypothetical protein, putative MFS permease
AB0017	15585	24278	2898	+		Hypothetical membrane protein
AB0018	24343	25242	300	-		Ribosomal large subunit pseudouridine synthase
AB0019	25356	26681	442	+	*purB*	Adenylosuccinate lyase
AB0020	26695	29076	794	+	*nrdA*	Ribonucleoside-diphosphate reductase, alpha chain
AB0021	29209	30225	339	+	*nrdB*	Ribonucleoside-diphosphate reductase, beta chain
AB0022	30386	31246	287	+		Hypothetical protein
AB0023	31307	31717	137	+		Conserved hypothetical protein
AB0024	31737	31985	83	+		Hypothetical protein
AB0025	32050	32997	316	+		Hypothetical protein, OmpA domain
AB0026	33004	33474	157	+		Conserved hypothetical protein
AB0027	33486	34469	328	-		Zinc-containing alcohol dehydrogenase superfamily protein
AB0028	34511	34915	135	+		Conserved hypothetical protein (DUF24 domain protein)
AB0029	34923	35816	298	-	*gpsA*	Glycerol 3-phosphate dehydrogenase
AB0030	35821	37251	477	-	*gatB*	Glutamyl-tRNA(Gln) amidotransferase, subunit B
AB0031	37264	38085	274	-		Conserved hypothetical protein
AB0032	38165	40999	945	-		Oxidoreductase, FAD-binding/iron-sulfur cluster-binding protein
AB0033	41121	42758	546	-	*lctP*	L-lactate permease
AB0034	42852	43493	214	-		Two-component response regulator

AB0035	43498	45687	730	-		Two-component sensor histidine kinase
AB0036	45715	46299	195	-		Conserved hypothetical protein (DUF162 domain protein)
AB0037	46295	47671	459	-		Iron-sulfur cluster binding protein, putative
AB0038	47671	48417	249	-		Fe-S oxidoreductase
AB0039	48513	48953	147	-		dCMP deaminase, putative
AB0040	49110	49580	157	+	*accB*	Acetyl-CoA carboxylase, biotin carboxyl carrier protein
AB0041	49593	50942	450	+	*accC1*	Acetyl CoA carboxylase, biotin carboxylase subunit
AB0042	51167	52714	516	+		ATP-dependent RNA helicase, DEAD box family
AB0044	52925	54517	531	+		Phosphate permease, putative
AB0045	54761	55492	244	+		Conserved hypothetical protein, putative permease
AB0046	55500	57284	595	+	*asnB1*	Asparagine synthetase (glutamine-hydrolyzing)
AB0047	57414	57848	145	-		Hypothetical protein
AB0048	58148	59545	466	-		PhoH family protein
AB0049	59541	60089	183	-		Conserved hypothetical integral membrane protein
AB0050	60062	61243	394	-		ATPase, AAA family protein
AB0051	61230	62054	275	-	*rluB*	Ribosomal large subunit pseudouridine synthase B
AB0052	62054	63013	320	-		Carbohydrate isomerase, KpsF/GutQ family
AB0053	63029	64948	640	-		Conserved hypothetical protein, predicted hydrolase of the metallo-beta-lactamase superfamily
AB0054	64935	65729	265	-	*ksgA*	Dimethyladenosine transferase
AB0055	65806	66564	253	+	*hisF1*	Imidazoleglycerol phosphate synthase, cyclase subunit
AB0056	66569	67249	227	+		Conserved hypothetical protein
AB0057	67249	67782	178	+		Conserved hypothetical protein, possible purine nucleoside phosphorylase
AB0058	67796	68875	360	+		Conserved hypothetical protein, radical SAM enzyme, Cfr family
AB0060	68967	70373	469	+	*gltX1*	Glutamyl-tRNA synthetase
AB0061	70400	70693	98	+	*rbpA*	RNA-binding region RNP-1 (RNA recognition motif)
AB0062	70806	71462	219	+		Two-component response regulator
AB0063	71440	72588	383	+		Two-component sensor histidine kinase
AB0064	72655	73035	127	+		Conserved hypothetical protein
AB0065	73038	74222	395	+		Hypothetical protein
AB0066	74222	75223	334	+	*czcB*	Cation efflux system, membrane fusion protein
AB0067	75233	78337	1035	+	*czcA*	Heavy metal efflux pump
AB0068	78389	78949	187	+		Conserved hypothetical periplasmic protein
AB0070	78986	80311	442	-		Fibronectin/fibrinogen-binding protein, putative
AB0071	80405	80719	105	+		Conserved hypothetical protein
AB0072	80789	82390	534	+		Conserved hypothetical protein
AB0073	82423	82935	171	-		Hypothetical protein

AB0074	83047	84852	602	+	*thrS*	Threonyl-tRNA synthetase
AB0075	84852	85376	175	+	*infC*	Translation initiation factor IF-3
AB0076	85518	85712	65	+	*rpmI*	50S ribosomal protein L35
AB0077	85814	86167	118	+	*rplT*	50S ribosomal protein L20
AB0078	86246	87439	398	-		Rhodanese-like sulfur transferase
AB0079	87426	87659	78	-		Hypothetical protein
AB0080	87672	89138	489	-		Conserved hypothetical protein, putative sulfide:quinone reductase
AB0081	89222	89821	200	-	*mobA*	Molybdopterin-guanine dinucleotide biosynthesis protein
AB0082	89824	91101	426	-	*leuC*	3-isopropylmalate dehydratase, large subunit
AB0083	91173	91634	154	-	*lspA*	Lipoprotein signal peptidase
AB0084	91634	92965	444	-	*glmM*	Phosphoglucosamine mutase
AB0085	93049	93306	86	+	*rpsT*	30S ribosomal protein S20
AB0086	93313	94377	355	+	*prfA*	Peptide chain release factor 1
AB0087	94401	94586	62	+		Hypothetical protein
AB0088	94665	95717	351	+	*pstS*	Phosphate ABC transporter, periplasmic phosphate-binding protein
AB0089	95790	97517	576	+		EAL/GGDEF domain protein
AB0090	97552	98505	318	+	*pstC*	Phosphate ABC transporter, permease protein
AB0091	98505	99686	394	+	*pstA*	Phosphate ABC transporter, permease protein
AB0092	99705	100472	256	+	*pstB*	Phosphate transporter, ATP-binding protein
AB0093	100475	101140	222	+		Phosphate transport system regulatory protein PhoU, putative
AB0094	101167	101568	134	+		Hypothetical protein
AB0095	101561	102235	225	+		Two-component response regulator
AB0096	102232	103614	461	+		Two-component sensor histidine kinase
AB0097	103693	104109	139	+	*rplM*	50S ribosomal protein L13
AB0098	104118	104504	129	+	*rpsI*	30S ribosomal protein S9
AB0099	104560	106107	516	+	*appA*	Oligopeptide ABC transporter, periplasmic substrate-binding protein
AB0100	106120	106764	215	+		Conserved hypothetical protein, HAD-superfamily hydrolase
AB0102	107081	108049	323	+		Conserved hypothetical protein, putative tricarboxylic transport protein TctC
AB0103	108089	108571	161	+		Conserved hypothetical protein, putative tricarboxylic transport protein TctB
AB0104	108571	110085	505	+		Conserved hypothetical protein, putative tricarboxylic transport protein TctA
AB0105	110116	110793	226	+		Two-component response regulator
AB0106	110786	112963	726	+		Two-component sensor histidine kinase
AB0107	113010	113660	217	+	*cynT1*	Carbonic anhydrase
AB0108	113656	114738	361	+		Conserved hypothetical protein, putative ammonia monooxygenase
AB0109	114721	115296	192	-		Hypothetical protein
AB0110	115296	116201	302	-		ABC transporter, periplasmic substrate-binding protein, putative
AB0111	116204	116938	245	-	*iamA*	ABC transporter, ATP-binding protein

AB0112	116942	118051	370	-	*iamB*	ABC transporter, permease protein
AB0113	118133	119041	303	-		Hypothetical protein
AB0114	119037	120617	527	-		Hypothetical protein
AB0115	120758	121270	171	+	*luxS*	Autoinducer-2 production protein LuxS
AB0116	121275	121712	146	+		Hypothetical protein
AB0117	121697	122464	256	+		Conserved hypothetical protein (DUF752 domain protein)
AB0118	122506	123324	273	+	*dacA*	Serine-type D-Ala-D-Ala carboxypeptidase
AB0119	123342	123839	166	-		Conserved hypothetical protein
AB0120	123957	124394	146	+		Putative membrane protein, DoxX family
AB0121	124424	127900	1159	-	*metH*	5-methyltetrahydrofolate--homocysteine methyltransferase
AB0122	128095	129012	306	+	*ilvE*	Branched-chain amino-acid aminotransferase
AB0123	129060	130130	357	+		Conserved hypothetical protein, Band 7 family protein
AB0124	130174	130458	95	+		Hypothetical protein
AB0125	130461	131138	226	+	*hisI*	Phosphoribosyl-AMP cyclohydrolase/ phosphoribosyl-ATP pyrophosphohydrolase
AB0126	131234	131488	85	+		Hypothetical protein
AB0127	131530	132708	393	-	*mrp*	ATP/GTP-binding protein
AB0128	132778	134127	450	-	*thiC*	Thiamine biosynthesis protein ThiC
AB0129	134273	135112	280	+		Conserved hypothetical protein
AB0130	135154	136275	374	+	*ispDF*	2-C-methyl-D-erythritol 4-phosphate cytidylyl transferase/synthase
AB0131	136263	137162	300	+		Two-component response regulator
AB0132	137176	137649	158	+	*pgpA*	Phosphatidylglycerophosphatase A
AB0133	137669	138379	237	+		Hypothetical protein
AB0134	138463	139578	372	-	*carA*	Carbamoylphosphate synthase, small subunit
AB0135	139581	140129	183	-		Conserved hypothetical protein (DUF507 domain protein)
AB0136	140137	141384	416	-	*purA*	Adenylosuccinate synthetase
AB0137	141389	142231	281	-		Conserved hypothetical protein, putative tRNA synthetase
AB0138	142234	143343	370	-		Putative aminotransferase
AB0139	143489	144115	209	+		Conserved hypothetical periplasmic protein
AB0140	144274	144723	150	+		Transcriptional regulator, MarR family
AB0141	144782	145330	183	+		Conserved hypothetical periplasmic protein
AB0142	145357	146130	258	+		Conserved hypothetical protein, LigB family protein
AB0143	146130	146825	232	+	*pirA*	Pirin
AB0144	146885	147910	342	+	*gapB*	Glyceraldehyde-3-phosphate dehydrogenase B
AB0145	147956	148948	331	-	*purM*	Phosphoribosylaminoimidazole synthetase
AB0146	149049	149594	182	+	*speE*	Spermidine synthase
AB0147	149641	150228	196	+	*coaE*	Dephospho-CoA kinase
AB0148	150228	150965	246	+	*dapF*	Diaminopimelate epimerase

AB0149	150993	152159	389	+	*purT*	Phosphoribosylglycinamide formyltransferase 2
AB0150	152171	152638	156	+		Transcriptional regulator, MarR family
AB0151	152646	152951	102	-		Hypothetical protein
AB0152	153038	154255	406	-		Conserved hypothetical protein
	**154428**	**154501**		**-**		**tRNA-Arg-1 (anticodon: TCG)**
AB0153	154547	155392	282	-	*ubiA*	4-hydroxybenzoate octaprenyltranferase
AB0154	155484	155684	67	+		Hypothetical protein
AB0155	155684	156556	291	+	*miaA*	tRNA delta(2)-isopentenylpyrophosphate transferase
AB0156	156640	156840	67	+	*rpmE*	50S ribosomal protein L31
AB0157	156856	157674	273	+		Conserved hypothetical protein, putative methyltransferase
AB0158	157674	158360	229	+		tRNA methyltransferase, TrmH family
AB0159	158376	159182	269	+		Conserved hypothetical protein
AB0160	159189	160400	404	+	*aspB1*	Aspartate aminotransferase, aminotransferase, classes I and II
AB0161	160528	161787	420	+	*hom*	Homoserine dehydrogenase
AB0162	161886	162659	258	+	*traT*	TraT complement resistance protein precursor
AB0163	162796	163551	252	+	*cdsA*	CDP-diglyceride synthetase CdsA
AB0164	163551	164615	355	+	*dxr*	1-deoxy-D-xylulose 5-phosphate reductoisomerase
AB0165	164608	164898	97	+		Hypothetical protein
AB0166	164894	165457	188	+		Conserved hypothetical protein (DUF1234 domain protein)
AB0167	165463	166461	333	+	*gcp*	O-sialoglycoprotein endopeptidase
AB0168	166465	166809	115	+		Hypothetical protein
AB0169	166844	168226	461	+		Conserved hypothetical protein, putative carbohydrate kinase
AB0170	168226	168534	103	+		Conserved hypothetical periplasmic protein
AB0171	168534	169310	259	+	*thiG*	Thiazole biosynthesis protein ThiG
	**169427**	**169501**		**+**		**tRNA-Pro (anticodon: TGG)**
	**169523**	**169596**		**+**		**tRNA-His (anticodon: GTG)**
	**169617**	**169690**		**+**		**tRNA-Arg-2 (anticodon: TCT)**
	**169698**	**169779**		**+**		**tRNA-Leu-5 (anticodon: TAG)**
	**169790**	**169863**		**+**		**tRNA-Gly-1 (anticodon: TCC)**
	**169935**	**170008**		**+**		**tRNA-Arg-3 (anticodon: TCT)**
	**170018**	**170091**		**+**		**tRNA-Gly-2 (anticodon: TCC)**
AB0172	170237	172066	610	-	*pycB1*	Pyruvate/oxaloacetate carboxyltransferase
AB0173	172076	172339	88	-		Hypothetical protein
AB0174	172487	173359	291	+		Transcriptional regulator, AraC family
AB0175	173409	174386	326	+		Calcium:sodium antiporter
AB0176	174413	176431	673	-		Methyl-accepting chemotaxis protein
AB0177	176536	179307	924	+		DNA/RNA helicase (DEAD/DEAH BOX family)

AB0178	179388	181304	639	+	*tkt*	Transketolase
AB0179	181425	181775	117	+	*lrgA*	LrgA family protein
AB0180	181768	182487	240	+	*lrgB*	LrgB-like protein
AB0181	182505	182855	117	+		Conserved hypothetical protein
AB0183	182876	183277	134	-		Conserved hypothetical protein (DUF350 domain protein)
AB0184	183344	184786	481	-	*dctA*	C4-dicarboxylate transport protein
AB0185	184871	185506	212	+		Hypothetical protein
AB0186	185509	185880	124	+		Hypothetical protein
AB0187	185873	186520	216	+		CDP-diacylglycerol--glycerol-3-phosphate 3-phosphatidyltransferase-related protein
AB0188	186523	187452	310	+		CDP-diglyceride synthetase/phosphatidate cytidylyltransferase
AB0189	187457	188080	208	+		1-acyl-sn-glycerol-3-phosphate acyltransferase
AB0190	188095	191574	1160	+	*aas*	2-acylglycerophosphoethanolamine acyltransferase / acyl-acyl carrier protein synthetase
AB0191	191585	194113	843	-	*glnD*	Protein-P-II uridylyltransferase
AB0192	194113	194862	250	-		Conserved hypothetical protein
AB0193	194927	195793	289	-		Conserved hypothetical protein
AB0194	195866	197797	644	-		Hypothetical protein, ankyrin repeat family
AB0195	197999	198334	112	+	*glnB1*	Nitrogen regulatory protein PII
AB0196	198338	199360	341	+	*pyrC*	Dihydroorotase
AB0197	199384	200487	368	-	*fliM*	Flagellar motor switch protein FliM
AB0198	200494	201540	349	-	*flgI*	Flagellar P-ring protein FlgI
AB0199	201645	202934	430	+		Conserved hypothetical protein
AB0200	202940	203197	86	+	*fliQ*	Flagellar basal body protein FliQ
AB0201	203202	203603	134	+		Hypothetical protein
AB0202	203615	204103	163	+		Hypothetical protein
AB0203	204108	204632	175	+	*fliL*	Flagellar basal body-associated protein
AB0204	204635	205060	142	+		Hypothetical protein
AB0205	205060	205761	234	+	*flgH*	Flagellar basal body L-ring protein FlgH
AB0206	205818	207725	636	+	*flgK*	Flagellar hook-associated protein FlgK
AB0207	207749	209083	445	+	*fliD*	Flagellar hook-associated protein 2
AB0208	209158	209511	118	+	*fliS*	Flagellar protein FliS
AB0209	209490	209756	89	+		Hypothetical protein
AB0210	209751	210728	326	-	*tilS*	tRNA(Ile)-lysidine synthase
AB0211	210734	212071	446	-	*yliG*	MiaB-like tRNA modifying enzyme
AB0212	212142	212957	272	-	*panC*	Pantoate--beta-alanine ligase
AB0213	212980	213936	319	-		Oligopeptide ABC transporter, permease protein
AB0214	213966	214319	118	+	*acpS*	Holo-(acyl-carrier-protein) synthase
AB0215	214360	215583	408	-	*gltS*	Sodium:glutamate symporter

AB0216	215610	215987	126	-		Conserved hypothetical protein, putative Holliday junction resolvase
AB0217	215987	216895	303	-	*metF*	5,10-methylenetetrahydrofolate reductase
AB0218	216976	217599	208	+	*serB*	3-phosphoserine phosphatase
AB0219	217605	218594	330	+	*tal*	Transaldolase
AB0220	218665	220014	450	+	*dhs*	3-deoxy-D-arabinoheptulosonate 7-phosphate synthase (DAHP synthetase class II)
AB0221	220083	220922	280	+	*tsx*	Nucleoside-specific channel-forming protein
AB0222	220994	224496		+		Type I restriction-modification system, M subunit, putative (pseudogene)
AB0224	224501	225493	331	+		Conserved hypothetical DNA binding protein
AB0225	225494	225832	113	-		Conserved hypothetical protein
AB0226	225986	226273	96	-	*gatC*	Glutamyl-tRNA(Gln) amidotransferase, subunit C
AB0227	226320	227438	373	-	*tgt*	Queuine tRNA-ribosyltransferase
AB0228	227540	228979	480	+		Conserved hypothetical protein
AB0229	228992	230119	376	+	*aspB2*	Aspartate aminotransferase, aminotransferase, classes I and II
AB0230	230115	230696	194	+		Hypothetical protein
AB0231	230699	232003	435	+	*murC*	UDP-N-acetylmuramate--alanine ligase
AB0232	232018	232791	258	-	*dprA*	SMF protein, DNA processing chain A
AB0233	232837	233913	359	-		Conserved hypothetical protein
AB0234	233958	234977	340	-	*ilvC*	Ketol-acid reductoisomerase
AB0235	235102	236952	617	+	*rnr*	Ribonuclease R
AB0236	236948	237322	125	-		Hypothetical protein (DUF180 domain protein)
AB0237	237387	238364	326	+		Conserved hypothetical protein
AB0238	238455	238808	118	+	*rpsF*	30S ribosomal protein S6
AB0239	238832	239341	170	+	*ssb*	Single-strand DNA binding protein
AB0240	239359	239622	88	+	*rpsR*	30S ribosomal protein S18
AB0241	239698	239895	66	+		Hypothetical protein
AB0242	239896	241296	467	-	*cysS*	Cysteinyl-tRNA synthetase
AB0243	241304	242476	391	-	*nusA*	Transcription termination factor NusA
AB0244	242630	242869	80	+		Conserved hypothetical protein
AB0245	242894	244201	436	+	*miaB*	tRNA-methylthiotransferase
AB0246	244207	244818	204	+		Putative lipoprotein
AB0247	244832	246397	522	+		Hypothetical protein
AB0248	246416	247474	353	-		Conserved hypothetical protein
AB0249	247483	247797	105	-		Hypothetical protein
AB0250	247805	248440	212	-	*cynT2*	Carbonic anhydrase
AB0251	248430	249533	368	-	*bioF*	8-amino-7-oxononanoate synthase
AB0252	249714	250043	110	-		Conserved hypothetical protein
AB0253	250051	250248	66	-		Conserved hypothetical protein

AB0254	250313	251035	241	-		OmpA domain protein
AB0255	251161	252633	491	+	*pyk*	Pyruvate kinase
AB0256	252658	253845	396	-	*argD1*	N-acetylornithine aminotransferase
AB0257	253908	256226	773	-	*cti*	Fatty acid cis/trans isomerase
AB0258	256322	257206	295	+	*pyrB*	Aspartate carbamoyltransferase
AB0259	257206	258162	319	+	*pabB*	Para-aminobenzoate synthase, glutamine amidotransferase component I
AB0260	258228	258782	185	+		Isochorismatase hydrolase
AB0261	258790	259458	223	+		Two-component response regulator
AB0262	259478	260053	192	+		Conserved hypothetical protein, putative para-aminobenzoate synthase component I
AB0263	260056	260607	184	-	*phnA*	Phosphonoacetate hydrolase
AB0264	260665	261153	163	+		Conserved hypothetical protein, predicted metal-dependent hydrolase
AB0265	261177	261923	249	+		Conserved hypothetical membrane protein, predicted permease
AB0266	261999	262562	188	+		Conserved hypothetical membrane protein, HPP family
AB0267	262571	263773	401	-		Hypothetical periplasmic protein
AB0268	263887	265083	399	+		Conserved hypothetical protein
AB0269	265093	266079	329	+		Conserved hypothetical protein, probable periplasmic protein
AB0270	266079	266198	40	+		Hypothetical protein
AB0271	266188	267117	310	-		D-isomer specific 2-hydroxyacid dehydrogenase, NAD-binding
AB0272	267136	268110	325	-		Conserved hypothetical protein
AB0273	268288	269172	295	+	*prpB*	Carboxyphosphoenolpyruvate phosphomutase PrpB
AB0274	269232	270374	381	+	*prpC*	2-methylcitrate synthase/citrate synthase 2
AB0275	270479	273067	863	+	*acnA*	Aconitate hydratase 1
AB0276	273160	274332	391	+		Conserved hypothetical protein (DUF453 domain protein)
AB0277	274409	274897	163	+		Hypothetical protein
AB0278	274932	275471	180	-		Isochorismatase hydrolase
AB0279	275534	277279	582	-		Hypothetical membrane protein
AB0280	277308	278243	312	-	*hemC*	Porphobilinogen deaminase
AB0281	278262	279089	276	-		Conserved hypothetical protein, putative DSBA oxidoreductase
AB0282	279154	281082	643	-	*dnaX*	DNA polymerase III, gamma and tau subunits
AB0283	281082	281864	261	-	*murI*	Glutamate racemase
AB0284	282092	283441	450	-	*gdhA*	NADP-specific glutamate dehydrogenase
AB0285	283584	284903	440	-	*rho*	Transcription termination factor Rho
AB0286	285066	285659	198	-	*tsaA*	Alkyl hydroperoxide reductase/ Thiol specific antioxidant
AB0287	285865	286134	90	+	*fdxA*	Ferredoxin
AB0288	286249	286659	137	+	*ndk*	Nucleoside diphosphate kinase
AB0289	286690	287064	125	+		Conserved hypothetical protein
AB0290	287079	287228	50	+	*rpmF*	50S ribosomal protein L32

AB0291	287231	288229	333	+	*plsX*	Fatty acid/phospholipid synthesis protein
AB0292	288259	289257	333	+	*fabH*	3-oxoacyl-(acyl carrier protein) synthase III
AB0293	289280	289711	144	-		Conserved hypothetical protein
AB0294	289716	291737	674	-		Conserved hypothetical protein, predicted ATP/GTP-binding protein
AB0295	291740	294088	783	-		Conserved hypothetical protein, predicted ATP/GTP-binding protein
AB0296	294191	294919	243	-	*frdB*	Fumarate reductase, iron-sulfur protein
AB0297	294922	296904	661	-	*frdA*	Fumarate reductase, flavoprotein subunit
AB0298	296907	297704	266	-	*frdC*	Fumarate reductase, cytochrome b subunit
AB0299	297905	299422	506	-	*nuoN*	NADH-quinone oxidoreductase, N subunit
AB0300	299422	300906	495	-	*nuoM*	NADH-quinone oxidoreductase, M subunit
AB0301	300911	302794	628	-	*nuoL*	NADH-quinone oxidoreductase, L subunit
AB0302	302799	303107	103	-	*nuoK*	NADH-quinone oxidoreductase, K subunit
AB0303	303114	303632	173	-	*nuoJ*	NADH-quinone oxidoreductase, J subunit
AB0304	303635	304132	166	-	*nuoI*	NADH-quinone oxidoreductase, I subunit
AB0305	304137	305408	424	-	*nuoH*	NADH-quinone oxidoreductase, H subunit
AB0306	305408	306841	478	-	*nuoG*	NADH-quinone oxidoreductase, G subunit
AB0307	306881	308164	428	-	*gltA*	Citrate synthase
AB0308	308192	309439	416	-	*nuoF*	NADH-quinone oxidoreductase, F subunit
AB0309	309432	309914	161	-	*nuoE*	NADH-quinone oxidoreductase, E subunit
AB0310	309917	311548	544	-	*nuoCD*	NADH-quinone oxidoreductase, C/D subunit
AB0311	311544	312056	171	-	*nuoB*	NADH-quinone oxidoreductase, B subunit
AB0312	312050	312409	120	-	*nuoA*	NADH-quinone oxidoreductase, A subunit
AB0313	312545	313708	388	-		Aminotransferase/L-cysteine desulfhydrase
AB0314	313793	315601	603	+		Carboxylyase-related protein
AB0315	315629	316093	155	-		Conserved hypothetical membrane protein (DUF1212 domain protein)
AB0316	316098	316868	257	-		Conserved hypothetical membrane protein (DUF1212 domain protein)
AB0317	316929	317258	110	+		Conserved hypothetical protein
AB0318	317230	317871	214	+		Transcriptional activator, putative, Baf family
AB0319	317852	318457	202	+	*hisG*	ATP phosphoribosyltransferase
AB0320	318460	319149	230	+		Conserved hypothetical protein, putative methyltransferase
AB0321	319275	321200	642	+		Methyl-accepting chemotaxis protein
AB0322	321178	322047	290	+		Conserved hypothetical protein
AB0323	322146	322748	201	+		Conserved hypothetical protein
AB0324	322772	323734	321	-	*trpS*	Tryptophanyl-tRNA synthetase
AB0325	323777	325879	701	-		TonB-dependent receptor protein
AB0326	326004	327164	387	-		Conserved hypothetical membrane protein
AB0328	327251	328423	391	-		MFS family, AmpG related permease

AB0329	328430	329416	329	-		O-methyltransferase
AB0330	329422	329643	74	-		Conserved hypothetical protein
AB0331	329655	331721	689	-		TonB-dependent receptor protein
AB0332	331763	332689	309	+		Transcriptional regulator
AB0333	332684	333220	179	-		Conserved hypothetical membrane protein (DUF204 domain protein)
AB0334	333217	334389	391	-		Major facilitator superfamily transporter
AB0335	334389	335594	402	-		Major facilitator superfamily transporter
AB0336	335594	337372	593	-		ABC transporter, ATP-binding/permease protein
AB0337	337368	339158	597	-		ABC transporter, ATP-binding/permease protein
AB0338	339264	341348	695	-		TonB-dependent receptor protein
AB0339	341462	342421	320	+		Transcriptional regulator, AraC family
AB0340	342454	344481	676	-		TonB-dependent receptor protein
AB0341	344649	344945	99	+		Hypothetical protein
AB0342	344960	346012	351	+		Cation efflux system, membrane protein
AB0343	346070	347305	412	+	*serS*	Seryl-tRNA synthetase
AB0344	347583	350705	1041	-	*nrfI*	Cytochrome c biogenesis protein
AB0345	350758	352245	496	-	*nrfA*	Cytochrome c552 nitrite reductase catalytic subunit NrfA
AB0346	352262	352789	176	-	*nrfH*	Cytochrome c nitrite reductase, small subunit NrfH
AB0347	352917	353300	128	-		Hypothetical protein
AB0348	353310	354923	538	-		EAL/GGDEF/PAS domain protein
AB0349	355018	355458	147	-		Methyl-accepting chemotaxis protein
AB0350	355463	355843	127	-	*napD*	Putative periplasmic nitrate reductase assembly protein NapD
AB0351	355848	356804	319	-	*napL*	Putative periplasmic protein
AB0352	356816	357289	158	-	*napF*	Ferredoxin-type protein NapF
AB0353	357292	357894	201	-	*napB*	Periplasmic nitrate reductase, small subunit, cytochrome c-type protein NapB
AB0354	357899	358702	268	-	*napH*	Methylamine utilization ferredoxin-type protein NapH
AB0355	358702	359511	270	-	*napG*	Fe-S ferredoxin-type protein NapG
AB0356	359532	362339	936	-	*napA*	Periplasmic nitrate reductase, large subunit
AB0357	362606	363595	330	+	*dctP*	C4-dicarboxylate-binding periplasmic protein
AB0358	363612	364154	181	+	*dctQ*	C4-dicarboxylate transport system, permease small subunit
AB0359	364160	365437	426	+	*dctM*	C4-dicarboxylate transport protein
AB0360	365470	367029	520	-		Two-component sensor histidine kinase
AB0361	367096	367791	232	+		Two-component response regulator
AB0362	367816	368295	160	-	*menG*	S-adenosylmethionine:2-demethylmenaquinone methyltransferase
AB0363	368454	369182	243	-	*cysG*	Uroporphyrin-III C-methyltransferase
AB0364	369241	370239	333	-		Conserved hypothetical protein
AB0365	370239	370901	221	-		Putative transcriptional regulator

AB0366	370987	371901	305	+		Glycosyltransferase, putative
AB0367	371920	372888	323	+	*moaA*	Molybdopterin biosynthesis protein A
AB0368	372988	373257	90	+	*rpsO*	30S ribosomal protein S15
AB0369	373389	373784	132	+		Transcriptional regulator, BadM/Rrf2 family
AB0370	373787	374821	345	+		Conserved hypothetical protein
AB0371	374835	375263	143	+		CoA-binding domain protein
AB0372	375266	376540	425	-		Two-component sensor histidine kinase
AB0373	376568	377245	226	-		Two-component response regulator
AB0374	377442	378248	269	+	*ppiC*	Peptidyl-prolyl cis-trans isomerase
AB0375	378262	379326	355	+	*fba*	Fructose-bisphosphate aldolase
AB0376	379481	380974	498	+	*ald*	Aldehyde dehydrogenase
AB0377	381031	381417	129	+		Conserved hypothetical protein (DUF779 domain protein)
AB0378	381424	381810	129	-	*exbD1*	Biopolymer transport protein ExbD
AB0379	381813	382283	157	-	*exbB1*	Biopolymer transport protein ExbB
AB0380	382302	383207	303	+		Conserved hypothetical protein
AB0381	383297	383770	158	+	*aroK*	Shikimate kinase
AB0382	383775	385064	430	+	*hisD*	Histidinol dehydrogenase
AB0383	385077	385376	100	+		Conserved hypothetical protein
AB0384	385390	386286	299	+	*ispB*	Octaprenyl-diphosphate synthase
AB0385	386289	387584	432	+	*hemA*	Glutamyl-tRNA reductase
AB0386	387604	389307	568	+	*proS*	Prolyl-tRNA synthetase
AB0387	389322	390209	296	+	*mscS*	Mechanosensitive ion channel
AB0388	390424	391395	324	+		tRNA nucleotidyltransferase/poly(A) polymerase
AB0389	391455	391892	146	+		Conserved hypothetical protein
AB0390	391879	393675	599	+		Conserved hypothetical protein
AB0391	393691	394920	410	-	*lnt*	Apolipoprotein N-acyltransferase
AB0392	394949	395209	87	+	*yajC*	Preprotein translocase subunit
AB0393	395279	396841	521	+	*secD*	Protein-export membrane protein SecD
AB0394	396844	397809	322	+	*secF*	Protein-export membrane protein SecF
AB0395	397881	398057	59	+		Hypothetical protein
AB0396	398056	398457	134	-		Hypothetical protein
AB0397	398466	399218	251	-	*motB*	Flagellar motor component MotB
AB0398	399225	399980	252	-	*motA*	Flagellar motor component MotA
AB0399	400128	400469	114	+		Conserved hypothetical protein
AB0400	400472	402934	821	+	*leuS*	Leucyl-tRNA synthetase
AB0401	402940	403461	174	+		Conserved hypothetical protein
AB0402	403467	404636	390	+	*folC*	Folylpolyglutamate synthase/dihydrofolate synthase

AB0403	404626	406743	706	+		Probable helicase
AB0404	406750	408450	567	-		Phospholipid/glycerol acyltransferase, possible hemolysin
AB0405	408556	409260	235	+		Conserved hypothetical protein
AB0406	409284	409658	125	-		Hypothetical protein
AB0407	409789	410091	101	+		Hypothetical protein
AB0408	410094	413078	995	+	*mfd*	Transcription-repair coupling factor
AB0409	413093	413608	172	+		Putative acetyltransferase
AB0410	413611	414549	313	-		Conserved hypothetical protein
AB0411	414654	415097	148	+		Hypothetical periplasmic protein
AB0412	415134	415811	226	+	*ompR*	Two-component response regulator
AB0413	415811	417043	411	+		Two-component sensor histidine kinase
AB0414	417092	418195	368	+		Conserved hypothetical protein
AB0415	418249	419658	470	+		Methyl-accepting chemotaxis protein
AB0416	419654	420028	125	+		Two-component response regulator
AB0417	420032	422071	680	+		Two-component sensor histidine kinase
AB0418	422071	424491	807	+		Two-component sensor histidine kinase
AB0419	424495	425304	270	+		Conserved hypothetical protein
AB0420	425354	426145	264	+		Hypothetical protein
AB0421	426145	426390	82	+		Hypothetical protein
AB0422	426531	427238	236	+	*ubiE*	Ubiquinone\menaquinone biosynthesis methlytransferase
AB0423	427266	428450	395	+	*xseA*	Exodeoxyribonuclease VII, large subunit
AB0425	428527	429003	159	+	*cheW*	Chemotaxis protein CheW
AB0426	429037	429567	177	-		Peptidyl-prolyl cis-trans isomerase-like protein
AB0427	429721	430101	127	+	*panD*	Aspartate 1-decarboxylase
AB0428	430097	430414	106	+		Conserved hypothetical protein (DUF149 domain protein)
AB0429	430422	431279	286	+	*ispA*	Geranyltranstransferase
AB0430	431419	431673	85	+	*groES*	10 kDa chaperonin
AB0431	431676	433307	544	+	*groEL*	60 kDa chaperonin
AB0432	433663	435036	458	-		Two-component sensor histidine kinase
AB0433	435029	435703	225	-		Two-component response regulator
AB0434	435703	436431	243	-		Ser/Thr protein phosphatase family protein
AB0435	436431	436883	151	-		Conserved hypothetical protein
AB0436	436890	437411	174	-		Hypothetical periplasmic protein
AB0437	437407	438369	321	-		Hypothetical protein
AB0438	438360	439928	523	-		Hypothetical protein
AB0439	439915	440535	207	-		ABC transporter, ATP-binding protein
AB0440	440535	441614	360	-		ABC transporter, permease protein

AB0442	441709	442293	195	-		Hypothetical protein
AB0443	442328	443098	257	+	*suhB*	Inositol-1-monophosphatase
AB0444	443151	444950	600	+	*glmS*	Glucosamine-fructose-6-phosphate aminotransferase
AB0445	445082	446338	419	+	*metK*	S-adenosylmethionine synthetase
AB0446	446443	447303	287	+	*accD*	Acetyl-CoA carboxylase, carboxyltransferase, beta subunit
AB0447	447310	447918	203	+	*thiE*	Thiamine-phosphate pyrophosphorylase
AB0448	447887	448363	159	+		Conserved hypothetical protein (DUF163 domain protein)
AB0449	448426	449430	335	+		Conserved hypothetical protein
AB0450	449434	450360	309	+		Probable tRNA-dihydrouridine synthase
AB0451	450445	451260	272	+	*prmA*	Ribosomal protein L11 methyltransferase
AB0452	451268	453250	661	+	*ftsH1*	Cell division protein FtsH
AB0453	453372	455195	608	+		Two-component response regulator
AB0454	455185	455589	135	+		Two-component response regulator
AB0455	455594	458164	857	+		Two-component sensor histidine kinase
AB0456	458444	459994	517	+	*leuA1*	2-isopropylmalate synthase
AB0457	460037	460366	110	+		Conserved hypothetical protein
AB0458	460366	460782	139	+		Conserved hypothetical protein
AB0459	460764	463739	992	+	*mutS1*	Mismatch repair ATPase
AB0460	463785	466808	1008	-		Outer membrane component of efflux system
AB0461	466813	467502	230	-		Putative membrane fusion component of efflux system
AB0462	467507	468724	406	-		Outer membrane component of efflux system
AB0463	468738	469331	198	-		Conserved hypothetical protein
AB0464	469425	470234	270	-		Conserved hypothetical protein
AB0465	470416	472020	535	+		Quinohemoprotein amine dehydrogenase, 60 kDa (α) subunit
AB0466	472033	473403	457	+		Quinohemoprotein amine dehydrogenase, putative quinone cofactor formation protein
AB0467	473417	473734	106	+		Quinohemoprotein amine dehydrogenase, 9 kDa (γ) subunit
AB0468	473755	474885	377	+		Quinohemoprotein amine dehydrogenase, 40 kDa (β) subunit
AB0469	474903	476537	545	+		ABC transporter, ATP-binding protein
AB0470	476533	477159	209	+		Conserved hypothetical protein
AB0471	477159	478481	441	+		Conserved hypothetical protein
AB0472	478496	480352	619	-		GGDEF domain protein
AB0473	480426	481130	235	-	*hisA*	Phosphoribosylformimino-5-aminoimidazole carboxamide ribotide isomerase
AB0474	481145	481993	283	-		Conserved hypothetical protein (DUF1731 domain protein)
AB0475	482017	482622	202	-	*hisH1*	Glutamine amidotransferase HisH
AB0476	482668	483237	190	-	*ctsW*	Transformation system protein
AB0477	483387	483791	135	+		Conserved hypothetical protein
AB0479	483939	485723	595	-	*lepA*	GTP-binding protein LepA

AB0480	485898	486161	88	+		Conserved hypothetical protein (DUF156 domain protein)
AB0481	486176	488692	839	+		Heavy-metal transporting P-type ATPase
AB0483	488764	489777	338	-	*cadF*	Outer membrane fibronectin-binding protein
AB0484	489860	490789	310	-	*prsA*	Ribose-phosphate pyrophosphokinase
AB0485	491053	492231	393	+		Conserved hypothetical protein
AB0486	492283	493317	345	-	*trmU1*	tRNA (5-methylaminomethyl-2-thiouridylate)-methyltransferase
AB0487	493332	494432	367	-	*trmU2*	tRNA (5-methylaminomethyl-2-thiouridylate)-methyltransferase
AB0488	494483	494965	161	-	*folK*	2-amino-4-hydroxy-6- hydroxymethyldihydropteridine pyrophosphokinase
AB0489	494999	496021	341	-	*pepQ*	Prolidase (Xaa-Pro dipeptidase) (pepQ)
AB0490	496099	496590	164	+	*aroQ*	3-dehydroquinate dehydratase
AB0491	496541	497818	426	+		Amidohydrolase family protein
AB0492	497814	498695	294	+	*pspA*	Protease IV (PspA)
AB0493	498784	499443	220	-		Hypothetical protein
AB0494	499683	500894	404	-	*ackA1*	Acetate kinase
AB0495	500924	501916	331	-	*pta*	Phosphate acetyltransferase
AB0496	502163	503350	396	-	*ackA2*	Acetate kinase
AB0497	503457	504392	312	-		Cation efflux protein
AB0498	504453	505640	396	-	*ackA3*	Acetate kinase
AB0499	505664	506269	202	-	*dnaQ1*	DNA polymerase III, epsilon subunit
AB0500	506265	508103	613	-		Conserved hypothetical protein (DUF294 domain protein)
AB0501	508157	509821	555	-		Sodium:solute symporter family protein
AB0502	509824	510135	104	-		Conserved hypothetical protein (DUF485 domain protein)
AB0504	510365	512011	549	-		Sodium:solute symporter family protein
AB0505	512014	512379	122	-		Conserved hypothetical protein (DUF485 domain protein)
AB0506	512454	513062	203	-	*dnaQ2*	DNA polymerase III, epsilon subunit
AB0507	513065	514882	606	-		Cyclic nucleotide-binding domain protein
AB0508	514905	516671	589	-		Sodium:solute symporter (Ssf family)
AB0509	516680	516931	84	-		Conserved hypothetical protein
AB0510	517010	517669	220	-		Two-component response regulator
AB0511	517653	519233	527	-		Two-component sensor histidine kinase
	**519313**	**519386**		**-**		**tRNA-Asp-1 (anticodon: GTC)**
	**519410**	**519482**		**-**		**tRNA-Val-1 (anticodon: TAC)**
	**519505**	**519576**		**-**		**tRNA-Glu-1 (anticodon: TTC)**
	**519602**	**519674**		**-**		**tRNA-Lys-1 (anticodon: TTT)**
	**519695**	**519768**		**-**		**tRNA-Asp-2 (anticodon: GTC)**
	**519835**	**519907**		**-**		**tRNA-Val-2 (anticodon: TAC)**
	**519930**	**520001**		**-**		**tRNA-Glu-2 (anticodon: TTC)**

	**520027**	**520099**		**-**		**tRNA-Lys-2 (anticodon: TTT)**
AB0512	520166	520669	168	-		CinA-like protein
AB0513	520769	522124	452	-	*mqo*	Malate:quinone oxidoreductase
AB0514	522392	525148	919	+	*ileS*	Isoleucyl-tRNA synthetase
AB0516	525275	525550	92	+		Conserved hypothetical NifU-like protein
AB0517	525556	526149	198	+		Conserved hypothetical protein
AB0518	526130	527416	429	+	*murE*	UDP-N-acetylmuramoylalanyl-D-glutamyl-2, 6-diaminopimelate ligase
AB0520	527751	528725	325	+		C4-dicarboxylate transporter/malic acid transport protein
AB0521	528768	529784	339	+	*tas*	Oxidoreductase Tas, aldo/keto reductase family
AB0522	529857	531341	495	+	*pitA*	Phosphate transporter family protein
AB0523	531631	534171	847	+		Methyl-accepting chemotaxis protein
AB0524	534240	534638	133	-		Hypothetical protein
AB0525	534784	536682	633	-		Methyl-accepting chemotaxis protein
AB0526	536838	537197	120	+		Conserved hypothetical protein
AB0527	537210	537677	156	+		Conserved hypothetical protein
AB0528	537720	538937	406	+		Peptidase, M48 family
AB0529	538937	540133	399	+		DNA recombination protein RmuC homolog
AB0530	540152	541048	299	-		Integral membrane domain protein (DUF6 domain protein)
AB0531	541051	542268	406	-	*moeA2*	Molybdenum cofactor biosynthesis protein A
AB0532	542282	542476	65	-		Hypothetical protein
AB0533	542584	543366	261	+		Conserved hypothetical protein
AB0534	543366	543863	166	+		Conserved hypothetical protein (DUF523 domain protein)
AB0535	543872	544108	79	-		Hypothetical protein
AB0536	544095	544589	165	-		Hypothetical protein
AB0537	544832	545626	265	+	*rpsB*	30S ribosomal protein S2
AB0538	545629	546672	348	+	*tsf*	Translation elongation factor EF-Ts
AB0539	546721	547398	226	+		ABC transporter, ATP-binding protein
AB0540	547398	548216	273	+		Hypothetical protein
AB0541	548216	548830	205	+	*gmk*	Guanylate kinase
AB0542	548837	550459	541	-		EAL/GGDEF domain protein
AB0543	550556	552991	812	+		Cation-transporting ATPase, P-type
AB0544	553011	553235	75	+		Hypothetical protein
AB0545	553271	553558	96	-		Conserved hypothetical protein, possible cytochrome c-553
AB0546	553758	554357	200	+		Oligopeptide ABC transporter, ATP-binding protein
AB0547	554347	555273	309	+	*hemH*	Ferrochelatase
AB0548	555307	557412	702	-	*feoB1*	Ferrous iron transport protein B
AB0549	557405	557629	75	-		Hypothetical protein

AB0550	557827	558570	248	+		Conserved hypothetical protein (DUF218 domain protein)
AB0551	558630	559367	246	+		ABC transporter, ATP-binding protein
AB0552	559384	560181	266	+		ABC transporter, permease protein
AB0553	560208	561029	274	+		Conserved hypothetical protein
AB0554	561113	561649	179	+	*tpx*	Thiol peroxidase
AB0555	561764	562489	242	+		Conserved hypothetical secreted protein
AB0556	562515	563489	325	-		Putative efflux protein, bile acid:sodium symporter family
AB0558	563572	564759	396	+		Hypothetical protein
AB0559	565013	565936	308	+		Oxidoreductase, molybdopterin binding
AB0560	565939	566502	188	+		Conserved hypothetical membrane protein
AB0561	566755	568797	681	+	*rep*	ATP-dependent DNA helicase, UvrD/Rep family
AB0562	568847	570001	385	+		Probable sodium/hydrogen antiporter
AB0563	570246	571577	444	+	*soxC*	Sulfur oxidation protein SoxCD, sulfur dehydrogenase subunit
AB0564	571549	572730	394	+	*soxD*	Sulfur oxidation protein SoxCD, diheme cytochrome c subunit
AB0565	572748	573098	117	+	*soxX*	Sulfur oxidation protein SoxXA, monoheme cytochrome c subunit
AB0566	573117	573566	150	+	*soxY*	Sulfur oxidation protein SoxYZ, sulfur covalently binding protein
AB0567	573627	573929	101	+	*soxZ*	Sulfur oxidation protein SoxYZ, sulfur compound chelating protein
AB0568	573970	574881	304	+	*soxA*	Sulfur oxidation protein SoxXA, diheme cytochrome c subunit
AB0569	574898	575314	139	+		Hypothetical protein
AB0570	575449	577212	588	+	*soxB*	Sulfur oxidation protein, sulfate thiol esterase
AB0571	577302	578093	264	+		Hypothetical protein
AB0572	578093	578524	144	+		Conserved hypothetical protein
AB0573	578531	578968	146	+		Conserved hypothetical protein
AB0574	578989	579777	263	+		Hypothetical protein
AB0576	579814	580377	188	-	*pfpI*	Peptidase, ThiJ/PfpI family protein
AB0578	580455	581369	305	-		Conserved hypothetical protein, beta-lactamase-like protein
AB0579	581459	582508	350	+		Conserved hypothetical protein, HD domain protein
AB0580	582538	583209	224	-		Rhodanese-like protein
AB0581	583395	586007	871	+	*valS*	Valyl-tRNA synthetase
AB0582	586038	586853	272	+		Hypothetical protein
AB0583	587017	587208	64	+		Conserved hypothetical protein
AB0584	587252	588103	284	-		Hypothetical protein
AB0585	588113	589423	437	-		Major facilitator superfamily transporter
AB0586	589501	590097	199	+		HAM1 protein homolog
AB0587	590093	590410	106	-		Hypothetical protein
AB0588	590410	590748	113	-		Hypothetical protein
AB0589	590741	591025	95	-		Hypothetical protein

AB0590	591015	591776	254	-	*proC*	Pyrroline-5-carboxylate reductase
AB0591	591846	592523	226	+		Conserved hypothetical membrane protein
AB0592	592569	594983	805	+	*lon*	ATP-dependent protease La
AB0593	594990	595550	187	+		Rhomboid-like protein
AB0594	595550	596005	152	+		Protein tyrosine phosphatase
AB0595	596048	597334	429	+		Conserved hypothetical protein
AB0596	597340	599019	560	+		Hypothetical TPR repeat protein
AB0597	599078	599506	143	-		Transcriptional regulator, MarR family
AB0598	599645	599965	107	+		Hypothetical protein
AB0599	600035	600280	82	-		Hypothetical protein
AB0600	600425	600805		+		Methyl-accepting chemotaxis protein (pseudogene)
AB0602	602086	603621	512	-		Methyl-accepting chemotaxis protein
AB0603	603754	603975	74	+		Conserved hypothetical protein
AB0604	604006	604875	290	+		Membrane protein, putative (DUF6 domain protein)
AB0605	605072	606520	483	-	*accC2*	Acetyl CoA carboxylase, biotin carboxylase subunit
AB0606	606772	607122	117	-		Conserved hypothetical protein (DUF59 domain protein)
AB0607	607135	607542	136	-	*sufE*	Putative suf regulatory protein
AB0608	607547	608728	394	-	*sufS*	Selenocysteine lyase/Cysteine desulfurase
AB0609	608724	609752	343	-	*sufD*	Fe-S assembly protein
AB0610	609742	610509	256	-	*sufC*	Fe-S assembly ABC transporter, ATP-binding protein
AB0611	610516	611982	489	-	*sufB*	Fe-S assembly ABC transporter, permease protein
AB0612	612179	613372	398	+	*iscS*	Cysteine desulfurase/aminotransferase (IscS/NifS)
AB0613	613406	614383	326	+	*iscU*	NifU-like protein
AB0614	614517	615137	207	+		Conserved hypothetical protein
AB0615	615145	616389	415	-		GGDEF domain protein
AB0616	616464	617861	466	+	*argH*	Argininosuccinate lyase
AB0617	617887	618792	302	-	*cheV*	Chemotaxis signal transduction protein CheV
AB0618	618846	619502	219	-		MoeB/ThiF family protein
AB0619	619565	620047	161	+	*greA*	Transcription elongation factor GreA
AB0620	620063	620758	232	+		Conserved hypothetical protein
AB0621	620773	622743	657	+	*uvrB*	Excinuclease ABC, subunit B
AB0622	622837	624717	627	+		Methyl-accepting chemotaxis protein
AB0623	624730	625371	214	-	*nth*	Endonuclease III
AB0624	625371	625778	136	-		Hypothetical protein
AB0625	625939	627918	660	+		TonB-dependent receptor protein
AB0626	628024	630177	718	+		TonB-dependent receptor protein
AB0627	630235	631623	463	-		Methyl-accepting chemotaxis protein

AB0628	631719	632813	365	-		Hypothetical protein
AB0629	633356	633538	61	+		Hypothetical protein
AB0630	633654	634343	230	+		Two-component response regulator
AB0631	634351	637329	993	+		Two-component sensor histidine kinase
AB0632	637521	639821	767	+		Methyl-accepting chemotaxis protein
AB0633	639938	641941	668	+		Methyl-accepting chemotaxis protein
AB0634	641972	643198	409	-		Conserved hypothetical protein
AB0636	643463	643915	151	-	*rpiB*	Ribose 5-phosphate isomerase
AB0637	643918	644697	260	-	*lepP*	Signal peptidase I
AB0638	644694	645554	287	-	*folD*	5,10-methylene-tetrahydrofolate dehydrogenase/5,10-methylene-tetrahydrofolate cyclohydrolase
AB0639	645702	646235	178	+	*rplY*	50S ribosomal protein L25
AB0640	646247	646795	183	+	*pth*	Peptidyl-tRNA hydrolase
AB0641	646801	647865	355	+		Conserved hypothetical membrane protein
AB0642	647882	648325	148	+		Hypothetical protein
AB0643	648294	649751	486	-	*amiA*	N-acetylmuramoyl-L-alanine amidase
AB0644	649760	650788	343	-	*npd*	2-nitropropane dioxygenase
AB0645	650852	652057	402	-	*tyrS*	Tyrosyl-tRNA synthetase
AB0646	652079	654220	714	-	*spoT*	ppGpp synthetase/guanosine-3',5'-bis(diphosphate) 3'-pyrophosphohydrolase
AB0647	654257	654472	72	-	*rpoZ*	DNA-directed RNA polymerase, omega chain
AB0648	654498	655202	235	-	*pyrH*	Uridylate kinase
AB0649	655262	656326	355	-		Conserved hypothetical protein
AB0650	656326	657258	311	-	*pdxA*	Pyridoxal phosphate biosynthetic protein A
AB0651	657254	658036	261	-	*pdxJ*	Pyridoxal phosphate biosynthesis protein
AB0652	658049	659461	471	-	*trpE*	Anthranilate synthase, component I
AB0653	659475	660500	342	-		Conserved hypothetical protein
AB0654	660529	661788	420	-	*glyA1*	Serine hydroxymethyltransferase
AB0655	661805	663316	504	-	*lysS*	Lysyl-tRNA synthetase
AB0656	663332	664033	234	-		Colicin V production protein
AB0657	664132	665190	353	+	*ispG*	4-hydroxy-3-methylbut-2-en-1-yl diphosphate synthase
AB0658	665193	666491	433	+		GGDEF domain protein
AB0659	666557	667996	480	+	*dnaB*	Replicative DNA helicase
AB0660	668065	669228	388	+	*ugd*	UDP-glucose 6-dehydrogenase
AB0661	669242	670330	363	+		NAD-dependent epimerase/dehydratase family protein
AB0662	670330	671571	414	+		UDP-hexose dehydrogenase
AB0663	671577	672542	322	+		Conserved hypothetical protein
AB0664	672550	673125	192	+		Conserved hypothetical protein

AB0665	673131	674216	362	+		DegT/DnrJ/EryC1/StrS aminotransferase
AB0666	674219	675394	392	+		FAD-dependent oxidoreductase
AB0667	675363	676367	335	+		NAD-dependent epimerase/dehydratase family protein
AB0668	676375	677562	396	+		DegT/DnrJ/EryC1/StrS aminotransferase
AB0669	677567	679561	665	+	*asnB2*	Asparagine synthetase
AB0670	679515	680072	186	+		Conserved hypothetical protein
AB0671	680075	681244	390	+	*wbpG1*	Putative LPS biosynthesis protein WbpG
AB0672	681244	681858	205	+	*hisH2*	Glutamine amidotransferase HisH
AB0673	681864	682622	253	+	*hisF2*	Imidazoleglycerol phosphate synthase, cyclase subunit
AB0674	682622	684133	504	+		Conserved hypothetical protein
AB0675	684136	685308	391	+		Hypothetical protein
AB0676	685396	687093	566	+		ABC transporter, ATP-binding/permease protein
AB0677	687100	689103	668	+		Putative O-antigen acyltransferase
AB0678	689103	690230	376	+	*pglJ*	Glycosyltransferase
AB0679	690221	692116	632	+	*asnB*	Asparagine synthetase
AB0680	692112	693215	368	+		Glycosyltransferase
AB0681	693277	694044	256	+		Conserved hypothetical membrane protein
AB0682	694138	694269	44	+		Hypothetical protein
AB0683	694296	695531	412	+		O-antigen translocase
AB0684	695534	696658	375	+	*wbpG2*	Putative LPS biosynthesis protein WbpG
AB0685	696658	697188	177	+		Conserved hypothetical protein
AB0686	697188	698330	381	+		Conserved hypothetical protein
AB0687	698326	699525	400	+		Conserved hypothetical protein
AB0688	699534	700694	387	+		Glycosyltransferase
AB0689	700687	701829	381	+	*wbpG3*	Putative LPS biosynthesis protein WbpG
AB0690	701841	702443	201	+	*hisH3*	Glutamine amidotransferase HisH
AB0691	702443	703210	256	+	*hisF3*	Imidazoleglycerol phosphate synthase, cyclase subunit
AB0692	703213	704319	369	+		Glycosyltransferase
AB0693	704319	705386	356	+		Putative hexose epimerase
AB0694	705382	706377	332	+		Glycosyltransferase
AB0695	706377	707336	320	+		Glycosyltransferase
AB0696	707339	707908	190	+		Putative hexose epimerase
AB0697	707921	709660	580	+	*pglF*	Sugar epimerase/dehydratase
AB0698	709725	710120	132	+		Hypothetical protein
AB0699	710130	710981	284	-	*ligA*	ATP-dependent DNA ligase
AB0700	711046	711600	185	+	*apt*	Adenine phosphoribosyltransferase
AB0701	711620	712825	402	+	*trpB1*	Tryptophan synthase, beta chain

AB0702	712846	713544	233	+		Conserved hypothetical membrane protein
AB0703	713537	714946	470	+	*pepA*	Aminopeptidase
AB0705	714980	715660	227	-		TonB-dependent receptor protein
AB0706	715671	716045	125	-	*exbD2*	Biopolymer transport protein ExbD
AB0707	716029	716454	142	-	*exbB2*	Biopolymer transport protein ExbB
AB0708	716612	717823	404	+		Conserved hypothetical membrane protein
AB0709	717833	719092	420	+		HlyD family secretion protein
AB0710	719082	719786	235	+		ABC transporter, ATP-binding protein
AB0711	719789	720997	403	+		ABC transporter, permease protein
AB0712	721029	721505	159	-		Hypothetical protein
AB0714	721993	724101	703	-		Heavy metal translocating P-type ATPase
AB0715	724106	724465	120	-		Conserved hypothetical protein
AB0716	724469	724816	116	-		Conserved hypothetical protein
AB0717	724819	725070	84	-		Hypothetical protein
AB0718	725077	725760	228	-		Conserved hypothetical protein
AB0719	725760	726071	104	-		Conserved hypothetical protein
AB0720	726135	726599	155	-	*fur1*	Ferric uptake regulation protein
AB0721	726750	727139	130	+		Hypothetical protein
AB0722	727165	728559	465	+	*fumC*	Fumarate hydratase, class II
AB0723	728584	728826	81	+		Hypothetical protein
AB0724	728870	730720	617	-		Two-component sensor histidine kinase
AB0725	730707	731558	284	-		Two-component response regulator
AB0726	731681	733825	715	+	*bfrE*	Probable TonB-dependent receptor
AB0727	733912	734589	226	+		Conserved hypothetical protein
AB0728	734589	735662	358	+		FMN-dependent alpha-hydroxy acid dehydrogenase, putative L-lactate dehydrogenase
AB0729	735880	737967	696	+	*irgA*	Iron-regulated outer membrane virulence protein homolog
AB0730	738049	738915	289	+		Conserved hypothetical protein, possible IroE protein
AB0731	738931	740208	426	+		Putative SAM-dependent methyltransferase
AB0732	740310	740972	221	+		Conserved hypothetical protein
AB0733	740987	742228	414	+	*cysJ*	Sulfite reductase, flavoprotein component
AB0734	742319	742513	65	-		Conserved hypothetical protein (DUF466 domain protein)
AB0735	742519	744615	699	-	*cstA*	Carbon starvation protein A
AB0736	744810	745910	367	+		GTP-binding protein, putative
AB0737	745923	746744	274	+		Conserved hypothetical protein
AB0738	746745	747359	205	-		Conserved hypothetical protein
AB0739	747362	747613	84	-		Conserved hypothetical protein
AB0740	747730	748041	104	+		Conserved hypothetical protein (DUF1255 domain protein)

AB0741	748087	748785	233	-		Conserved hypothetical protein
AB0742	748832	750220	463	-		GGDEF/PAS domain protein
AB0743	750338	751156	273	+	*thiL*	Thiamine monophosphate kinase
AB0744	751293	752354	354	+	*truD*	tRNA pseudouridine synthase D
AB0745	752410	752760	117	+		Hypothetical protein
AB0746	752792	754981	730	-		Methyl-accepting chemotaxis protein
AB0747	755090	755644	185	+	*ruvA*	Holliday junction DNA helicase RuvA
AB0748	755666	756706	347	+	*ddlA*	D-alanine--D-alanine ligase
AB0749	756712	757428	239	+		2-hydroxy-6-oxohepta-2,4-dienoate hydrolase
AB0750	757418	758851	478	+	*murF*	UDP-N-acetylmuramoylalanyl-D-glutamyl-2,6-diaminopimelate ligase
AB0751	758865	759350	162	+		HIT family protein
AB0752	759461	760450	330	+		Hypothetical protein
AB0753	760564	761781	406	+		Hypothetical protein
AB0754	761991	762302	104	+	*rpsJ*	30S ribosomal protein S10
AB0755	762324	762896	191	+	*rplC*	50S ribosomal protein L3
AB0756	762896	763504	203	+	*rplD*	50S ribosomal protein L4
AB0757	763509	763787	93	+	*rplW*	50S ribosomal protein L23
AB0758	763801	764625	275	+	*rplB*	50S ribosomal protein L2
AB0759	764638	764910	91	+	*rpsS*	30S ribosomal protein S19
AB0760	764916	765245	110	+	*rplV*	50S ribosomal protein L22
AB0761	765248	765949	234	+	*rpsC*	30S ribosomal protein S3
AB0762	765955	766377	141	+	*rplP*	50S ribosomal protein L16
AB0763	766367	766555	63	+	*rpmC*	50S ribosomal protein L29
AB0764	766569	766817	83	+	*rpsQ*	30S ribosomal protein S17
AB0765	766820	767185	122	+	*rplN*	50S ribosomal protein L14
AB0766	767188	767424	79	+	*rplX*	50S ribosomal protein L24
AB0767	767420	767974	185	+	*rplE*	50S ribosomal protein L5
AB0768	767977	768159	61	+	*rpsN*	30S ribosomal protein S14
AB0769	768175	768570	132	+	*rpsH*	30S ribosomal protein S8
AB0770	768583	769116	178	+	*rplF*	50S ribosomal protein L6
AB0771	769129	769485	119	+	*rplR*	50S ribosomal protein L18
AB0772	769498	769935	146	+	*rpsE*	30S ribosomal protein S5
AB0773	769942	770337	132	+	*rplO*	50S ribosomal protein L15
AB0774	770343	771602	420	+	*secY*	Preprotein translocase, SecY subunit
AB0775	771607	772368	254	+	*map*	Methionine aminopeptidase
AB0776	772381	772596	72	+	*infA*	Translation initiation factor IF-1
AB0777	772688	773353	222	+		Conserved hypothetical protein

AB0778	773353	774543	397	+		Conserved hypothetical protein
AB0779	774590	776356	589	+	*aspS*	Aspartyl-tRNA synthetase
AB0780	776500	779082	861	+		CheW-like chemotaxis protein
AB0781	779146	780900	585	+		Radical SAM domain protein
AB0782	780942	781580	213	-	*adk1*	Adenylate kinase
AB0783	781710	782276	189	+	*adk2*	Adenylate kinase
AB0784	782279	783043	255	+		Molybdopterin binding domain protein
AB0785	783072	783713	214	+	*cat*	Chloramphenicol O-acetyltransferase
AB0786	783841	784980	380	+		Zinc-containing NADP-dependent alcohol dehydrogenase
AB0787	785021	786145	375	+		Iron-containing NADP-dependent alcohol dehydrogenase
AB0788	786200	788458	753	-		Methyl-accepting chemotaxis protein
AB0789	788586	789605	340	-	*alr*	Alanine racemase
AB0790	789611	791458	616	-	*uvrC*	Excinuclease ABC, subunit C
AB0791	791614	794007	798	+		Methyl-accepting chemotaxis protein
AB0792	794444	796444	667	+		Methyl-accepting chemotaxis protein
AB0793	796489	798480	664	+		Methyl-accepting chemotaxis protein
AB0794	798834	800855	674	+		Methyl-accepting chemotaxis protein
AB0795	800869	801528	220	-		Two-component response regulator
AB0796	801528	802589	354	-		Two component system histidine kinase/response regulator fusion protein
AB0797	802655	804949	765	+		Two-component sensor histidine kinase
AB0798	804932	805339	136	-		MutT/nudix family protein
AB0799	805419	806186	256	-		Hypothetical protein
AB0800	806182	806541	120	-		Hypothetical protein
AB0801	806537	806890	118	-		Hypothetical protein
AB0802	806999	808270	424	+		Urea/short-chain amide ABC transporter, periplasmic urea/short-chain amide-binding protein
AB0803	808330	809931	534	+		Urea/short-chain amide ABC transporter, permease protein
AB0804	809938	811041	368	+		ABC transporter, permease protein
AB0805	811046	811831	262	+		ABC transporter, ATP-binding protein
AB0806	811964	812656	231	+		ABC transporter, ATP-binding protein
AB0807	812786	813772	329	+		Hypothetical protein
AB0808	813947	814696	250	+	*ureD*	Urease accessory protein UreD
AB0809	814709	815383	225	+	*ureAB*	Fusion of urease beta and gamma subunits
AB0810	815388	817085	566	+	*ureC*	Urease, alpha subunit
AB0811	817098	817541	148	+	*ureE*	Urease accessory protein
AB0812	817489	818229	247	+	*ureF*	Urease complex component
AB0813	818368	818952	195	+	*ureG*	Urease accessory protein UreG

AB0814	819155	819715	187	-		Conserved hypothetical protein
AB0815	819827	820417	197	+		Transcriptional reguator, TetR family
AB0816	820433	823558	1042	-		Hydrophobe/amphiphile efflux-1 family protein
AB0817	823564	824619	352	-		Multidrug efflux RND membrane fusion protein
AB0818	824619	825851	411	-		Outer membrane efflux protein
AB0819	825933	826433	167	+		Putative transcriptional regulator (MarR family)
AB0820	826517	826732	72	+		Hypothetical protein
AB0821	826756	826941	62	-		Conserved hypothetical protein
AB0822	826957	827073	39	-		Hypothetical protein
AB0823	827157	828809	551	+		GGDEF domain protein
AB0824	828812	829597	262	-	*xth*	Exodeoxyribonuclease
AB0825	829600	829845	82	-		Hypothetical protein
AB0826	829944	830663	240	+		Conserved hypothetical protein (DUF81 domain protein)
AB0827	830710	831003	98	-		Conserved hypothetical protein
AB0828	831010	831531	174	-		Outer membrane lipoprotein
AB0829	831694	832926	411	+		Conserved hypothetical protein
AB0830	832926	833654	243	+		Conserved hypothetical protein
AB0831	833650	834978	443	-		ABC transporter protein
AB0832	835206	835550	115	-		Conserved hypothetical protein
AB0833	835480	836547	356	-		EAL domain protein
AB0834	836615	837319	235	-		Two-component response regulator
AB0835	837319	838311	331	-		Two-component sensor histidine kinase
AB0836	838342	839754	471	-		Carotenoid isomerase, putative
AB0837	839744	841093	450	-	*phrB*	Deoxyribodipyrimidine photolyase
AB0838	841093	842043	317	-		Conserved hypothetical protein (DUF523/1722 domain protein)
AB0839	842172	843080	303	-		Auxin efflux carrier protein, putative
AB0840	843085	843570	162	-		Conserved hypothetical protein (DUF386 domain protein)
AB0841	843582	844811	410	-	*pgi*	Glucose-6-phosphate isomerase
AB0842	844814	845668	285	-	*galU*	UTP--glucose-1-phosphate uridylyltransferase
AB0843	845673	847046	458	-		Phosphohexosemutase
	**847461**	**848972**		**+**	***rrnA***	**16S ribosomal RNA**
	**849083**	**849156**		**+**		**tRNA-Ile-1 (anticodon: GAT)**
	**849214**	**849286**		**+**		**tRNA-Ala-1 (anticodon: TGC)**
	**849680**	**852577**		**+**	***rrnA***	**23S ribosomal RNA**
	**852738**	**852857**		**+**	***rrnA***	**5S ribosomal RNA**
AB0844	853283	854680	466	+		Methyl-accepting chemotaxis protein
AB0845	854693	855094	134	+		Methyl-accepting chemotaxis protein

AB0846	855124	855333	70	-		Probable tautomerase
AB0847	855424	856251	276	+		Transcriptional regulator, LysR family
AB0848	856254	857042	263	-	*trpC*	Indole-3-glycerol phosphate synthase
AB0849	857042	858346	435	-		Conserved hypothetical protein
AB0850	858325	858705	127	-		Conserved hypothetical protein
AB0851	858701	859414	238	-		Conserved hypothetical protein
AB0852	859644	859964	107	+	*oorD*	OorD subunit of 2-oxoglutarate:acceptor oxidoreductase
AB0853	859976	861103	376	+	*oorA*	OorA subunit of 2-oxoglutarate:acceptor oxidoreductase
AB0854	861108	861938	277	+	*oorB*	OorB subunit of 2-oxoglutarate:acceptor oxidoreductase
AB0855	861949	862530	194	+	*oorC*	OorC subunit of 2-oxoglutarate:acceptor oxidoreductase
AB0856	862560	863375	272	-	*dnaQ3*	DNA polymerase III, epsilon subunit
AB0857	863365	863955	197	-	*trpF*	N-(5'phosphoribosyl)anthranilate isomerase
AB0858	863955	864593	213	-	*rpe*	Ribulose-phosphate 3-epimerase
AB0859	864701	865708	336	+	*pldA*	Outer membrane phospholipase A
AB0860	865724	866143	140	-		Conserved hypothetical protein
AB0861	866149	866727	193	-		Conserved hypothetical protein
AB0862	866742	867239	166	-		Conserved hypothetical protein (DUF520 domain protein)
AB0863	867292	868044	251	-		Conserved hypothetical protein
AB0864	868048	868428	127	-		Conserved hypothetical membrane protein (DUF423)
AB0865	868444	868887	148	-		Putative nucleotide phosphoribosyltransferase
AB0866	869002	869643	214	+		Conserved hypothetical protein
AB0867	869652	869849	66	-		Hypothetical protein
AB0868	869856	870533	226	-		Conserved hypothetical protein
AB0869	870548	871603	352	-		Membrane-associated zinc metalloprotease, putative
AB0870	871616	872158	181	-	*pgsA*	CDP-1,2-diacyl-sn-glycero-3-phosphate phosphatidyltransferase
AB0871	872161	872937	259	-		Oxidoreductase, short chain dehydrogenase/reductase family
AB0872	872940	873824	295	-	*dapA*	Dihydrodipicolinate synthase
AB0873	873851	875182	444	-		Putative zinc protease
AB0874	875185	876240	352	-	*pyrD*	Dihydroorotate dehydrogenase
AB0875	876287	877993	569	-	*msbA*	Multidrug resistance protein MsbA
AB0876	878054	879352	433	-	*mviN*	Virulence factor MviN protein
AB0877	879355	880932	526	-		Endonuclease/exonuclease/phosphatase
AB0878	881116	881517	134	+	*perR*	Peroxide stress regulator
AB0879	881548	881967	140	-		Conserved hypothetical protein
AB0880	882058	882459	134	+		Conserved hypothetical protein
AB0881	882471	883094	208	-		Peptidase, S51 family
AB0882	883113	884459	449	-	*radA*	DNA repair protein RadA

AB0883	884577	885137	187	+		Conserved hypothetical protein
AB0885	885240	886154	305	+		Cytochrome c peroxidase
AB0886	886135	888612	826	+		EAL/GGDEF/PAS domain protein
AB0887	888620	889459	280	-		Hypothetical protein
AB0888	889480	890406	309	-	*ftsY*	Signal recognition particle-docking GTPase FtsY
AB0889	890422	890970	183	-		Putative lipoprotein thiredoxin
AB0890	891032	891565	178	+		Putative 5-formyltetrahydrofolate cyclo-ligase
AB0891	891581	893122	514	+		HD/HDIG/KH domain protein
AB0892	893144	893734	197	+		Putative methyltransferase
AB0893	893697	894539	281	+		ABC transporter, permease protein
AB0894	894582	895010	143	+		Hypothetical protein
AB0895	895013	895345	111	-		Hypothetical protein
AB0896	895423	895560	46	+		Hypothetical protein
AB0897	895563	895787	75	+		Hypothetical protein
AB0898	895888	896706	273	-		Oxidoreductase, short-chain dehydrogenase/reductase family
AB0899	896778	897557	260	+		Conserved hypothetical protein
AB0900	897584	898891	436	+		Hypothetical protein
AB0901	898912	899985	358	+		Cytochrome c family protein
AB0903	900004	900945	314	+		Putative methyl-accepting chemotaxis protein
AB0904	900945	901604	220	+		Two-component response regulator
AB0905	901594	902739	382	+		Two-component sensor histidine kinase
AB0906	902800	903642	281	+		Molybdenum transport system protein ModD, putative
AB0907	903662	904774	371	-		GGDEF domain protein
AB0908	904799	905239	147	-		GatB/YqeY family protein
AB0909	905281	907227	649	-		ABC transporter, ATP-binding protein
AB0910	907385	907990	202	+		Conserved hypothetical protein
AB0911	908011	908601	197	-		Conserved hypothetical protein
AB0912	908686	909384	233	-		Conserved hypothetical protein
AB0913	909769	911067	433	-		Conserved hypothetical protein
AB0914	911158	911502	115	-		Hypothetical protein
AB0915	911514	912020	169	-		Hypothetical protein
AB0916	912108	913808	567	+		Conserved hypothetical protein
AB0917	914027	914365	113	-		Conserved hypothetical protein
AB0918	914446	914805	120	-		Hypothetical protein
AB0919	914824	915606	261	-		Hypothetical protein
AB0920	915957	916547	197	-		Hypothetical protein
AB0921	916585	918117	511	-	*guaA*	GMP synthase

AB0922	918185	919633	483	-	*nadB*	L-aspartate oxidase
AB0923	919732	920145	138	+		Conserved hypothetical protein
AB0924	920135	920725	197	-		Conserved hypothetical protein (DUF45 domain protein)
AB0925	920807	921970	388	+	*aspB3*	Aspartate aminotransferase, aminotransferase, classes I and II
AB0926	921977	922867	297	+		Cation efflux protein
AB0927	922929	925259	777	+		Conserved hypothetical protein
AB0928	925249	927417	723	+	*cyaA*	Adenylate/guanylate cyclase
AB0929	927420	928730	437	-		Conserved hypothetical protein
AB0930	928742	929929	396	-		Conserved hypothetical protein
AB0931	930114	931298	395	+		Conserved hypothetical protein
AB0932	931348	932259	304	+		Conserved hypothetical protein
AB0933	932302	933546	415	+		Major facilitator superfamily protein, putative oxalate:formate antiporter
AB0934	933647	934369	241	+		Conserved hypothetical protein
AB0935	934374	934973	200	+	*cbiM*	Cobalamin (Vitamin B12) biosynthesis protein
AB0936	934973	935446	158	+		Hypothetical protein
AB0938	935446	936087	214	+		Cobalt ABC transporter, permease protein, putative
AB0939	936087	936734	216	+		Cobalt ABC transporter, ATP-binding protein, putative
AB0940	936838	938463	542	+		Hemolysin activation protein HecB, putative
AB0941	938478	947576	3033	+		Adhesin/haemagglutinin, HecA family
AB0942	947591	948457	289	+		Hypothetical protein
AB0943	948482	949261	260	+		Hypothetical protein
AB0944	949266	951641	792	+		Hypothetical protein
AB0945	951646	952371	242	+		Hypothetical protein
AB0946	952410	952655	82	+		Hypothetical protein
AB0947	952661	953374	238	+		Hypothetical protein
AB0948	953396	954049	218	+		Conserved hypothetical protein
AB0949	954128	954685	186	+		Hypothetical protein
AB0950	954750	955133	128	-		Hypothetical protein
AB0951	955147	955962	272	-		Conserved hypothetical protein
AB0953	956185	956934	250	-		Conserved hypothetical protein
AB0954	957100	958227	376	+		Conserved hypothetical protein
AB0955	958265	959347	361	+		Putative permease
AB0956	959381	959941	187	+		Hypothetical protein
AB0957	959953	960354	134	+		Hypothetical protein
AB0958	960376	962736	787	-		Conserved hypothetical protein
AB0959	962866	963219	118	+		Hypothetical protein
AB0960	963273	966083	937	-	*uvrA*	Excinuclease ABC, subunit A

AB0961	966188	966736	183	-		Conserved hypothetical protein
AB0962	967016	967423	136	+		Methyl-accepting chemotaxis protein
AB0963	967478	969232	585	-	*ggt*	Gamma-glutamyltranspeptidase
AB0964	969419	970015	199	-		Conserved hypothetical protein (DUF81 domain protein)
AB0966	970159	970890	244	-	*cysE*	Serine acetyltransferase
AB0967	970883	972691	603	-	*speA*	Arginine decarboxylase
AB0968	972695	973933	413	-	*hisS*	Histidyl-tRNA synthetase
AB0969	973926	974492	189	-	*tmk*	Thymidylate kinase
AB0970	974486	974977	164	-	*coaD*	Phosphopantetheine adenylyltransferase
AB0971	974985	975536	184	-	*ubiD*	Phenylacrylic acid decarboxylase
AB0972	975747	976370	208	+		Site-specific recombinase, resolvase family
AB0973	976447	976890	148	+	*rplI*	50S ribosomal protein L9
AB0974	976899	977432	178	+	*hslV*	Heat shock protein HslVU, ATP-dependent protease subunit HslV
AB0975	977443	978768	442	+	*hslU*	Heat shock protein HslVU, ATP-dependent protease subunit HslU
AB0976	978824	979126	101	+		Hypothetical protein
AB0977	979287	979928	214	+		TatD-related deoxyribonuclease
AB0978	979967	981223	419	+		Putative lytic murein transglycosylase
AB0979	981210	982151	314	+	*rlpA*	Rare lipoprotein A
AB0980	982164	982733	190	+	*hisB*	Imidazoleglycerol-phosphate dehydratase
AB0981	982733	983227	165	+		HAD-superfamily hydrolase subfamily IIIA:Phosphatase
AB0982	983221	983760	180	+		Hypothetical protein
AB0983	983763	984254	164	+		OstA family protein
AB0984	984257	984856	200	+		Putative ATP /GTP binding protein
AB0985	984849	985307	153	+		Acetyltransferase, GNAT Family
AB0986	985376	985849	158	+		Sigma factor, ECF family
AB0987	985853	986818	322	+		Sigma factor regulatory protein, FecR/PupR family
AB0988	986960	989314	785	+		TonB-dependent receptor protein
AB0989	989365	990318	318	-	*argC*	N-acetyl-gamma-glutamyl-phosphate reductase
AB0990	990377	991738	454	-		Transcriptional regulator, GntR family
AB0991	991829	992191	121	+		FMN-binding protein
AB0992	992207	992803	199	+		Putative antibiotic resistance protein
AB0993	992817	994469	551	-		AcrB/AcrD/AcrF family protein
AB0994	994469	996028	520	-		AcrB/AcrD/AcrF family protein
AB0995	996018	996740	241	-		AcrA/AcrE family protein
AB0996	996740	998089	450	-		Outer membrane efflux protein
AB0997	998221	1000161	647	-		Response regulator receiver:Metal-dependent phosphohydrolase, HD subdomain
AB0998	1000193	1000915	241	-	*fliP*	Flagellar biosynthetic protein FliP

AB0999	1000994	1002772	593	-	*pbpC*	Penicillin-binding protein
AB1000	1002772	1003197	142	-		Hypothetical protein
AB1001	1003209	1004480	424	-		Conserved hypothetical protein
AB1002	1004476	1005285	270	-	*ftsX*	Cell division protein FtsX
AB1003	1005275	1005934	220	-	*ftsE*	Cell division ATP-binding protein FtsE
AB1004	1005934	1007124	397	-		Conserved hypothetical protein
AB1005	1007135	1008394	420	-		Fibronectin type III domain protein
AB1006	1008345	1009328	328	-	*rluD*	Ribosomal large subunit pseudouridine synthase D
AB1007	1009348	1010454	369	+	*mrdB*	Rod shape-determining protein RodA
AB1008	1010484	1011260	259	-		Conserved hypothetical membrane protein
AB1009	1011293	1012570	426	+	*hemL*	Glutamate-1-semialdehyde 2,1-aminomutase
AB1010	1012587	1012898	104	+		Conserved hypothetical protein (DUF1255 domain protein)
AB1011	1012912	1013217	102	+		Conserved hypothetical protein
AB1012	1013159	1013752	198	+		Conserved hypothetical protein
AB1013	1013767	1014450	228	-		Conserved hypothetical protein
AB1014	1014456	1016333	626	-	*rpoD*	RNA polymerase sigma 70 factor
AB1015	1016501	1016998	166	+	*leuD*	3-isopropylmalate dehydratase, small subunit
AB1016	1017002	1018066	355	+	*leuB*	3-isopropylmalate dehydrogenase
AB1017	1018091	1019017	309	+		Exopolyphosphatase-related protein
AB1018	1019034	1019390	119	-		HIT family protein
AB1019	1019615	1019725	37	+	*rpmJ*	50S ribosomal protein L36
AB1020	1019734	1020099	122	+	*rpsM*	30S ribosomal protein S13
AB1021	1020113	1020502	130	+	*rpsK*	30S ribosomal protein S11
AB1022	1020516	1021139	208	+	*rpsD*	30S ribosomal protein S4
AB1023	1021160	1022155	332	+	*rpoA*	DNA-directed RNA polymerase, alpha chain
AB1024	1022173	1022520	116	+	*rplQ*	50S ribosomal protein L17
AB1025	1022677	1024035	453	+	*gatA*	Glutamyl-tRNA(Gln) amidotransferase, subunit A
AB1026	1024058	1025500	481	+	*guaB*	Inosine-5-monophosphate dehydrogenase
AB1027	1025653	1026102	150	+		Conserved hypothetical protein
AB1028	1026132	1026626	165	-		Hypothetical protein
AB1029	1026683	1028254	524	-		Two-component sensor histidine kinase
AB1030	1028268	1028939	224	-		Two-component response regulator
AB1031	1029118	1030914	599	+		Outer membrane efflux protein, putative
AB1032	1030927	1031637	237	+		Conserved hypothetical protein
AB1033	1031657	1039207	2517	+		Hypothetical membrane protein
AB1034	1039230	1040573	448	+		Putative membrane protein
AB1035	1040576	1042693	706	+		Peptidase, M50 family

AB1036	1042729	1043208	160	-		DnaJ domain protein
AB1037	1043296	1043559	88	+		DnaJ domain protein
AB1038	1043575	1044666	364	+		Glycosyl hydrolase
AB1039	1044676	1046580	635	-		Two-component sensor histidine kinase
AB1040	1046586	1047254	223	-		Two-component response regulator
AB1041	1047376	1047885	170	-		Hypothetical protein
AB1042	1048008	1048793	262	-		ABC transporter, ATP-binding protein
AB1044	1048932	1049408	159	+		Sigma factor, ECF family
AB1045	1049408	1050343	312	+		Sigma factor regulatory protein, FecR/PupR family
AB1046	1050422	1051474	351	+		NADP-dependent alcohol dehydrogenase
AB1047	1051503	1052120	206	+		Conserved hypothetical protein
AB1048	1052123	1052713	197	-		Cyclic nucleotide-binding protein
AB1049	1052826	1053704	293	+		Conserved hypothetical membrane protein (DUF6 domain protein)
AB1050	1053714	1054076	121	-		Conserved hypothetical protein (DUF24 domain protein)
AB1051	1054174	1054710	179	+		Conserved hypothetical protein
AB1052	1054739	1056091	451	-		Putative sodium:sulfate symporter
AB1053	1056128	1056442	105	-	*sugE*	Suppresses groEL, may be chaperone
AB1054	1056578	1057456	293	+		Conserved hypothetical protein, possible transporter
AB1055	1057474	1058277	268	+		Transcriptional regulator, AraC family
AB1056	1058345	1058866	174	+		Conserved hypothetical protein
AB1057	1058875	1059741	289	-		Transcriptional regulator, LysR family
AB1058	1059841	1060860	340	+		Putative integral membrane protein
AB1059	1060885	1061622	246	-		Conserved hypothetical periplasmic protein
AB1060	1061712	1062611	300	-		Auxin efflux carrier protein
AB1061	1062618	1062986	123	-		Conserved hypothetical protein (DUF24 domain protein)
AB1062	1063121	1063699	193	+		Flavodoxin-like fold domain protein, putative NADPH-quinone reductase
AB1063	1063712	1064005	98	+		Conserved hypothetical protein
AB1064	1064021	1065043	341	+		NADH:flavin oxidoreductase/NADH oxidase
AB1065	1065064	1065957	298	+		Conserved hypothetical integral membrane protein (DUF6 domain protein)
AB1066	1065988	1066620	211	+		NAD(P)H-flavin nitroreductase
AB1067	1066736	1068226	497	+		Hypothetical protein
AB1068	1068311	1069906	532	+		ABC transporter, ATP-binding protein
AB1069	1069967	1070440	158	-		Acyl-CoA thioester hydrolase family protein
AB1070	1070533	1072491	653	-		Methyl-accepting chemotaxis protein
AB1071	1072590	1074533	648	-	*lig*	DNA ligase
AB1072	1074552	1075727	392	-		Glutathionylspermidine synthase family protein
AB1073	1075734	1076372	213	-		Conserved hypothetical lipoprotein

AB1074	1076506	1077948	481	+	*dgt*	Deoxyguanosinetriphosphate triphosphohydrolase
AB1076	1078224	1079297	358	+	*mloA*	Conserved hypothetical protein, MloA homolog
AB1077	1079392	1080660	423	+		Conserved hypothetical protein
AB1080	1081125	1081916	264	-	*aroE*	Shikimate 5-dehydrogenase
AB1081	1081922	1082530	203	-		Methyltransferase, putative
AB1082	1082591	1083256	222	+		Conserved hypothetical protein (DUF152 domain protein)
AB1083	1083354	1084610	419	+	*maeA*	NAD-dependent malic enzyme
AB1084	1084681	1085511	277	+	*purU*	Formyltetrahydrofolate deformylase
AB1085	1085516	1085977	154	+		RNA methylase, SpoU family
AB1086	1085976	1086200	75	-		Hypothetical protein
AB1087	1086190	1087950	587	-		Sodium:phosphate cotransporter
AB1088	1088081	1088458	126	+		Hypothetical protein
AB1089	1088464	1088802	113	+		Hypothetical protein
AB1090	1088806	1089465	220	+		Two-component response regulator
AB1091	1089458	1090309	284	+		Two-component sensor histidine kinase
AB1092	1090356	1091282	309	+		Putative protease
AB1093	1091263	1091700	146	+		Conserved hypothetical protein
AB1094	1091737	1092114	126	-		Hypothetical protein
AB1095	1092228	1093355	376	-	*cydB*	Cytochrome bd oxidase, subunit II
AB1096	1093360	1094883	508	-	*cydA*	Cytochrome bd oxidase, subunit I
AB1097	1094902	1095105	68	-		Conserved hypothetical protein
AB1098	1095267	1095692	142	-		Conserved hypothetical protein
AB1099	1095718	1096875	386	-		Conserved hypothetical protein
AB1100	1096981	1097520	180	-		TPR repeat protein, SEL1 subfamily
AB1101	1097735	1097953	73	+		Hypothetical protein
AB1102	1097956	1098621	222	-	*aat*	Leucyl/phenylalanyl-tRNA--protein transferase
AB1103	1098680	1099363	228	+	*rsuA*	Ribosomal small subunit pseudouridine synthase A
AB1104	1099366	1099989	208	-		HAD-superfamily hydrolase
AB1105	1100305	1100805	167	+		Conserved hypothetical protein
AB1106	1100808	1101515	236	+		Conserved hypothetical protein
AB1107	1101546	1101728	61	+		Hypothetical protein
AB1108	1101751	1103667	639	+		TonB-dependent receptor protein
AB1109	1103672	1104499	276	+		Putative iron compound ABC transporter, periplasmic substrate-binding protein
AB1110	1104480	1105178	233	+		Putative iron compound ABC transporter, ATP-binding protein
AB1111	1105178	1106137	320	+		Putative iron compound ABC transporter, permease protein
AB1112	1106137	1106625	163	-		Phosphohistidine phosphatase
AB1113	1106641	1108407	589	-		ABC transporter, ATP-binding protein

AB1114	1108503	1108748	82	+		Hypothetical protein
AB1115	1108741	1109403	221	+		Two-component response regulator
AB1116	1109363	1110547	395	+		Two-component sensor histidine kinase
AB1117	1110544	1110882	113	+		Conserved hypothetical protein
AB1118	1110956	1111411	152	+		Hypothetical protein
AB1119	1111445	1112257	271	-		Conserved hypothetical protein
AB1120	1112315	1112827	171	+		Hypothetical protein
AB1121	1112843	1113715	291	-		GGDEF domain protein
AB1122	1113909	1114220	104	+		Hypothetical protein
AB1123	1114233	1115285	351	-		Putative ATP-dependent RNA helicase RhlE
AB1124	1115668	1117044	459	+		Two-component sensor histidine kinase
AB1125	1117044	1117730	229	+		Two-component response regulator
AB1126	1118036	1120597	854	+		Hypothetical outer membrane protein
AB1127	1120626	1122785	720	+		ABC transporter, transmembrane region
AB1128	1122781	1124247	489	+		HlyD family secretion protein
AB1129	1124257	1124847	197	+		Two-component response regulator
AB1130	1124920	1125516	199	+		Conserved hypothetical protein
AB1131	1125516	1127387	624	+		EAL/GGDEF/HAMP domain protein
AB1132	1127464	1128495	344	+	*aroB*	3-dehydroquinate synthase
AB1133	1128500	1130236	579	+		Conserved hypothetical integral membrane protein
AB1134	1130243	1131508	422	+		MiaB-like tRNA modifying enzyme
AB1135	1131492	1132961	490	+	*ftsH2*	Cell division protein FtsH
AB1136	1132951	1133466	172	+		Conserved hypothetical protein
AB1137	1133466	1133999	178	+	*mog*	Molybdenum cofactor biosynthesis protein Mog
AB1138	1134020	1134631	204	-	*ribB*	3,4-dihydroxy-2-butanone 4-phosphate synthase
AB1139	1134991	1135572	194	+		Conserved hypothetical protein
AB1140	1135585	1136106	174	+		Conserved hypothetical protein
AB1141	1136127	1136363	79	+		Conserved hypothetical protein
AB1142	1136363	1139692	1110	+		Hypothetical protein
AB1143	1139700	1140110	137	+		Conserved hypothetical protein
AB1144	1140130	1140372	81	-		Conserved hypothetical protein
AB1145	1140468	1140707	80	+		Hypothetical protein
AB1146	1140762	1141238	159	+		Conserved hypothetical protein
AB1147	1141351	1142835	495	+		ABC transporter, ATP-binding protein
AB1149	1142902	1143345	148	+		Conserved hypothetical protein
AB1150	1143508	1144098	197	+	*torC*	Cytochrome c-type protein TorC
AB1151	1144098	1146578	827	+	*bisC*	Biotin sulfoxide reductase

AB1152	1146610	1147428	273	-		Alpha/beta hydrolase fold protein
AB1153	1147505	1148095	197	+		Transcriptional regulator, TetR family
AB1154	1148145	1150343	733	+	*mutS2*	Mismatch repair ATPase
AB1155	1150339	1151673	445	-		Conserved hypothetical protein, putative efflux protein
AB1156	1151767	1152318	184	-		Hypothetical protein
AB1157	1152408	1155164	919	-		Conserved hypothetical protein (DUF748 domain protein)
AB1158	1155252	1156349	366	+	*dapE*	Succinyl-diaminopimelate desuccinylase
AB1159	1156461	1157777	439	+	*amtB*	Ammonium transporter
AB1160	1157800	1158135	112	+	*glnB2*	Nitrogen regulatory protein PII
AB1161	1158301	1158855	185	+	*pssA*	CDP-diacylglycerol--serine O-phosphatidyltransferase
AB1162	1158866	1159783	306	+		Ppx/GppA phosphatase family protein
AB1163	1159786	1160571	262	-		Hypothetical protein
AB1164	1160732	1162426	565	+	*ilvI*	Acetolactate synthase, large subunit
AB1165	1162429	1162923	165	+	*ilvH*	Acetolactate synthase, small subunit
AB1166	1162945	1163889	315	+	*lpxD*	UDP-3-O-[3-hydroxymyristoyl] glucosamine N-acyltransferase
AB1167	1164050	1166107	686	+	*ftsK*	Cell division protein FtsK
AB1168	1166175	1166858	228	+		Two-component response regulator
AB1169	1166977	1168170	398	+		Two-component sensor histidine kinase
AB1170	1168235	1169098	288	+		Conserved hypothetical protein
AB1171	1169171	1171015	615	+		Two-component sensor histidine kinase
AB1172	1171011	1171697	229	+		Two-component response regulator
AB1173	1171772	1172530	253	+		Hypothetical protein
AB1174	1172599	1173261	221	+		Two-component response regulator
AB1175	1173293	1173910	206	+		Hypothetical protein
AB1176	1174011	1174709	233	+		Hypothetical protein
AB1177	1174743	1175351	203	+		Hypothetical protein
AB1178	1175440	1175874	145	-		Conserved hypothetical protein
AB1179	1176074	1176361	96	+		Hypothetical protein
AB1180	1176351	1179050	900	+		Conserved hypothetical protein, putative DNA helicase
AB1181	1179144	1179239	32	+		Hypothetical protein
AB1182	1179437	1179976	180	+		Conserved hypothetical protein
AB1183	1180064	1181518	485	+	*engA*	GTP-binding protein
AB1184	1181548	1183119	524	+		Sulfate permease family protein
AB1185	1183119	1183745	209	+	*hemD*	Putative uroporphyrinogen III cosynthase HemD
AB1186	1183807	1184016	70	+		Hypothetical protein
AB1187	1183988	1185247	420	+	*purD*	Phosphoribosylamine-glycine ligase
AB1188	1185257	1185691	145	+		Conserved hypothetical protein

AB1189	1185687	1187861	725	+		Conserved hypothetical membrane protein
AB1190	1187864	1188514	217	+		Conserved hypothetical protein
AB1191	1188524	1190698	725	+	*pnp*	Polyribonucleotide nucleotidyltransferase
AB1192	1190831	1191196	122	+	*cheY1*	Chemotaxis protein CheY
AB1193	1191212	1193266	685	+	*cheA*	Chemotaxis protein CheA
AB1194	1193275	1194114	280	+	*cheR*	Chemotaxis protein methyltransferase
AB1195	1194114	1194800	229	+	*cheD*	Chemotaxis protein CheD
AB1196	1194836	1195912	359	+	*cheB*	Protein-glutamate methylesterase CheB
AB1197	1195916	1196506	197	+		Conserved hypothetical protein
AB1198	1196519	1197670	384	+	*fsr*	Fosmidomycin resistance protein
AB1199	1197782	1198831	350	+	*xerD*	Integrase/recombinase XerD
AB1200	1198836	1199177	114	+		Conserved hypothetical protein
AB1201	1199189	1199464	92	-		Hypothetical protein
AB1202	1199486	1199959	158	-		Hypothetical protein
AB1203	1200015	1201982	656	-	*prlC*	Oligopeptidase A
AB1204	1201988	1202818	277	-	*hemK*	Modification methylase
AB1205	1202864	1203934	357	-	*hemN1*	Oxygen-independent coproporphyrinogen III oxidase
AB1206	1203990	1204484	165	+	*nudH*	(Di)nucleoside polyphosphate hydrolase
AB1207	1204505	1205713	403	+	*lysC*	Aspartokinase
AB1208	1205719	1206267	183	+		Conserved hypothetical protein
AB1209	1206267	1206899	211	+	*holB*	Putative DNA polymerase III delta prime subunit HolB
AB1210	1206914	1208050	379	+	*folP*	Dihydropteroate synthase
AB1211	1208115	1208639	175	-		Conserved hypothetical protein
AB1212	1208639	1209862	408	-	*cfa*	Cyclopropane-fatty-acyl-phospholipid synthase
AB1213	1209868	1210617	250	-		Conserved hypothetical protein (DUF1365 domain protein)
AB1214	1210622	1211857	412	-		Conserved hypothetical protein
AB1215	1211857	1212342	162	-		Conserved hypothetical lipoprotein
AB1216	1212403	1213662	420	-		Sugar transporter, putative
AB1217	1213706	1214461	252	-		Oxidoreductase, short chain dehydrogenase/reductase family
AB1218	1214564	1215397	278	-	*tyrA*	Prephenate dehydrogenase
AB1219	1215469	1217772	768	+		Outer membrane surface antigen protein
AB1220	1217775	1218845	357	+		SAM domain protein
AB1221	1218852	1220141	430	+		Peptidase, M16 family
AB1222	1220147	1221445	433	+	*gltX2*	Glutamyl-tRNA synthetase
AB1223	1221445	1221714	90	+		Conserved hypothetical membrane protein
AB1225	1221727	1223382	552	+	*slt*	Soluble lytic murein transglycosylase (slt)
AB1226	1223357	1223851	165	-		Hypothetical protein

AB1227	1223949	1224443	165	+	*mobB*	Molybdopterin-guanine dinucleotide biosynthesis protein
AB1228	1224450	1225289	280	+	*fbp*	Fructose-1,6-biphosphatase
AB1229	1225285	1225488	68	+		Conserved hypothetical protein
AB1230	1225506	1227455	650	+	*metS*	Methionyl-tRNA synthetase
AB1231	1227458	1228453	332	+		Conserved hypothetical protein
AB1232	1228462	1229115	218	-		Peptidase, S24 family
AB1233	1229225	1229317	31	+		Hypothetical protein
AB1234	1229441	1229713	91	+		Hypothetical protein
AB1235	1229723	1231552	610	+	*pycB2*	Pyruvate/oxaloacetate carboxyltransferase
AB1236	1231629	1233203	525	+	*pckA*	Phosphoenolpyruvate carboxykinase
AB1237	1233246	1234274	343	-	*ribAB*	GTP cyclohydrolase II / 3,4-dihydroxy-2-butanone 4-phosphate synthase
	**1234393**	**1234464**		**+**		**tRNA-Gln-1 (anticodon: TTG)**
	**1234492**	**1234565**		**+**		**tRNA-Met-1 (anticodon: CAT)**
	**1234645**	**1234718**		**+**		**tRNA-Thr-1 (anticodon: TGT)**
AB1238	1234737	1236749	671	-	*glyS*	Glycyl-tRNA synthetase, beta chain
AB1239	1236745	1236900	52	-		Hypothetical protein
AB1240	1236932	1237312	127	-		Conserved hypothetical protein
AB1241	1237322	1237531	70	-		Conserved hypothetical protein
AB1242	1237531	1238112	194	-	*gidB*	Glucose inhibited division protein B
AB1243	1238115	1238687	191	-	*ribA*	GTP cyclohydrolase II
AB1244	1238749	1239720	324	+	*hemB*	Delta-aminolevulinic acid dehydratase
AB1245	1239732	1240808	359	+		Two-component sensor histidine kinase
AB1246	1240808	1241467	220	+		Two-component response regulator
AB1247	1241450	1242151	234	-		Radical SAM domain protein
AB1248	1242105	1242266	54	-		Conserved hypothetical protein
AB1249	1242247	1244358	704	-	*nrdD*	Oxygen-sensitive ribonucleoside-triphosphate reductase
AB1250	1244572	1245498	309	+	*argF*	Ornithine carbamoyltransferase
AB1251	1245502	1246866	455	+	*hemN2*	Oxygen-independent coproporphyrinogen III oxidase
AB1252	1246918	1247385	156	-		Hypothetical protein
AB1253	1247389	1247598	70	-		Conserved hypothetical protein
AB1254	1247610	1248551	314	-	*lpxK*	Lipid A biosynthesis protein LpxK
AB1255	1248561	1249694	378	-		DegT/DnrJ/EryC1/StrS aminotransferase
AB1256	1249694	1250455	254	-	*nadE*	NH(3)-dependent NAD+ synthetase
AB1257	1250486	1250695	70	-		Hypothetical protein
AB1258	1250706	1250927	74	-		Hypothetical protein
AB1259	1250931	1251683	251	-		DnaJ domain protein
AB1260	1252683	1252994	104	+		Hypothetical protein

AB1261	1253269	1253622	118	+		Conserved hypothetical membrane protein
AB1262	1253625	1254362	246	+	*tatC*	Sec-independent protein secretion pathway component TatC
AB1263	1254369	1255388	340	+	*queA*	S-adenosylmethionine:tRNA ribosyltransferase-isomerase
AB1264	1255402	1256142	247	+	*dnaQ4*	DNA polymerase III, epsilon subunit
AB1265	1256146	1256898	251	+		Conserved hypothetical protein
AB1266	1256893	1258125	411	-	*comE*	Competence locus E
AB1267	1258157	1258630	158	+		Hypothetical protein
AB1268	1258645	1260654	670	-		Conserved hypothetical protein
AB1269	1260668	1262899	744	-		Hypothetical protein
AB1270	1262889	1263461	191	-		Hypothetical protein
AB1271	1263533	1264522	330	+		Conserved hypothetical protein
AB1272	1264624	1265919	432	+	*glpC*	Anaerobic glycerol-3-phosphate dehydrogenase, subunit C (glpC)
AB1273	1265927	1266742	272	+	*lgt*	Phosphatidylglycerol-prolipoprotein diacylglyceryl transferase
AB1274	1266749	1266943	65	-		Hypothetical protein
AB1275	1266980	1267669	230	-		Two-component response regulator
AB1276	1267744	1270431	896	-	*polA*	DNA polymerase I
AB1277	1270509	1271039	177	+		Acetyltransferase
AB1278	1271065	1271793	243	+	*kdsB*	3-deoxy-manno-octulosonate cytidylyltransferase
AB1279	1271800	1273731	644	+		EAL/GGDEF domain protein
AB1280	1273749	1274468	240	-		ABC transporter, ATP-binding protein
AB1281	1274464	1274877	138	-		Conserved hypothetical protein
AB1282	1274891	1275871	327	-	*trpD*	Anthranilate phosphoribosyltransferase
AB1283	1276083	1276958	292	+		Conserved hypothetical protein
AB1284	1276974	1277219	82	-		S4 domain protein
AB1285	1277301	1278539	413	+	*argG*	Argininosuccinate synthase
AB1286	1278589	1280022	478	-	*dltA*	D-alanine activating enzyme
AB1287	1280022	1281155	378	-	*dltB*	D-alanyl transfer protein
AB1288	1281158	1281295	46	-		Hypothetical protein
AB1289	1281309	1282433	375	-	*dltD*	Poly D-alanine transfer protein
AB1290	1282436	1282660	75	-	*dltC*	D-alanyl carrier protein
AB1291	1282806	1283303	166	+		Conserved hypothetical protein
AB1292	1283686	1285179	498	+		Sulfate permease
AB1293	1285185	1286021	279	+	*uspA*	Universal stress protein
AB1295	1286691	1287338	216	-	*ktrA*	TRK system potassium uptake protein TrkA, putative
AB1296	1287338	1288663	442	-	*ktrB*	TRK system potassium uptake protein TrkB, putative
AB1297	1288673	1289356	228	-	*kdpE*	Two component system transcriptional regulatory protein
AB1298	1289356	1290381	342	-	*kdpD*	Two-component regulatory protein sensor kinase KdpD

AB1299	1290568	1291491	308	-	*dut*	Deoxyuridinetriphosphatase
AB1300	1291686	1292255	190	-	*recR*	Recombination protein RecR
AB1301	1292355	1293470	372	+	*dnaJ*	Co-chaperone and heat shock protein DnaJ
AB1302	1293505	1294719	405	+	*trpB2*	Tryptophan synthase, beta chain
AB1303	1294733	1295320	196	+		Hypothetical protein
AB1304	1295327	1295983	219	-		Hypothetical protein
AB1305	1295987	1296802	272	-		Conserved hypothetical protein (DUF455 domain protein)
AB1306	1296857	1297447	197	+		Beta-lactamase-like protein
AB1307	1297450	1298349	300	+		GGDEF domain protein
AB1308	1298360	1299325	322	+		GGDEF domain protein
AB1309	1299334	1300245	304	-		Conserved hypothetical protein
AB1310	1300314	1300808	165	+		Hypothetical protein
AB1311	1300915	1303659	915	+		Cytochrome c biogenesis protein
AB1312	1303701	1304984	428	+	*pepD*	Aminoacyl-histidine dipeptidase PepD
AB1313	1304992	1305285	98	-		Hypothetical protein
AB1314	1305351	1307831	827	-		Methyl-accepting chemotaxis protein
AB1315	1308062	1308313	84	+		Hypothetical protein
AB1316	1308306	1308833	176	-	*lolA*	Outer membrane lipoprotein carrier protein LolA
AB1317	1308890	1311505	872	+	*secA*	Protein translocase, SecA subunit
AB1318	1311510	1312712	401	+		Conserved hypothetical integral membrane protein
AB1319	1312734	1315649	972	-		Hypothetical protein
AB1320	1315570	1316688	373	+		4-amino-4-deoxychorismate lyase PabC
AB1321	1316810	1318999	730	+	*icd*	Isocitrate dehydrogenase
AB1322	1319102	1320043	314	+	*mdh*	Malate dehydrogenase
AB1323	1320146	1320421	92	+		Conserved hypothetical protein, putative cytochrome c
AB1324	1320449	1320850	134	-		Hypothetical protein
AB1325	1320937	1322211	425	+		Conserved hypothetical protein
AB1326	1322238	1322513	92	-		Hypothetical protein
AB1327	1322509	1323408	300	-	*mraW*	S-adenosyl-methyltransferase
AB1328	1323468	1324043	192	+		Conserved hypothetical protein
AB1329	1324160	1324432	91	+	*hup*	DNA-binding protein HU
	**1324502**	**1324582**		**+**		**tRNA-Leu-1 (anticodon: CAA)**
AB1330	1324763	1325977	405	+		Putative phage integrase
AB1331	1326731	1326940	70	-		Hypothetical protein
AB1332	1327013	1327564	184	-		Hypothetical protein
AB1333	1327652	1327987	112	-		Hypothetical protein
AB1334	1328136	1328930	265	-		Hypothetical protein

AB1335	1329234	1330313	360	+		Conserved hypothetical protein
AB1336	1330422	1330691	90	+		Hypothetical protein
AB1337	1330796	1332832	679	-		DEAD/DEAH box helicase domain protein
AB1338	1332828	1333994	389	-		Hypothetical protein
AB1339	1334421	1334747	109	-		Hypothetical protein
AB1340	1334980	1336620	547	+		Hypothetical protein
AB1341	1336697	1337890	398	+		Conserved hypothetical protein
AB1342	1338172	1341051	960	-		Hypothetical protein
AB1343	1341056	1341355	100	-		Hypothetical protein
AB1344	1341381	1341731	117	-		Hypothetical protein
AB1345	1341706	1342188	161	-		Hypothetical protein
AB1346	1342193	1342699	169	-		Hypothetical protein
AB1347	1342704	1344776	691	-		Hypothetical protein
AB1348	1344781	1345620	280	-		Hypothetical protein
AB1349	1345624	1346544	307	-		Conserved hypothetical protein
AB1350	1346691	1347140	150	-		Hypothetical protein
AB1351	1347155	1347553	133	-		Hypothetical protein
AB1352	1347566	1348354	263	-		Hypothetical protein
AB1353	1348537	1349172	212	-		Hypothetical protein
AB1354	1349253	1349483	77	-		Hypothetical protein
AB1355	1349498	1349716	73	-		Hypothetical protein
AB1356	1349716	1350192	159	-		Hypothetical protein
AB1357	1350234	1350605	124	-		Hypothetical protein
AB1358	1350938	1351387	150	+		Hypothetical protein
AB1359	1351529	1351933	135	-		Conserved hypothetical protein
AB1360	1352079	1354310	744	+	*norB*	Nitric oxide reductase, cytochrome b subunit
AB1361	1354348	1354914	189	-		Hypothetical protein
AB1362	1354914	1356080	389	-		D-amino acid oxidase domain protein
AB1363	1356080	1356391	104	-		Conserved hypothetical protein
AB1364	1356506	1356871	122	+		Response regulator receiver domain protein, CheY-like
AB1365	1356874	1357488	205	+		Inhibitor of MCP methylation, CheC
AB1366	1357488	1358978	497	+		Two-component sensor histidine kinase
AB1367	1359058	1359924	289	+	*cbpA*	Curved DNA-binding protein CbpA
AB1368	1359939	1360316	126	+		Transcriptional regulator (MerR family), putative heat shock regulator HspR
AB1369	1360392	1361381	330	+		HlyD-family secretion protein
AB1370	1361371	1363068	566	+		ABC transporter, ATP-binding protein
AB1371	1363072	1364157	362	+		ABC transporter, permease protein

AB1372	1364160	1365248	363	+		ABC transporter, permease protein
AB1373	1365255	1366331	359	-		Conserved hypothetical protein (DUF475 domain protein)
AB1374	1366423	1367712	430	-	*brnQ*	Branched-chain amino acid transport system II carrier protein
AB1376	1367766	1368620	285	-		Conserved hypothetical protein
	**1368659**	**1368731**		**-**		**tRNA-Glu-3 (anticodon: TTC)**
AB1377	1368804	1369244	147	-		Conserved hypothetical protein
AB1378	1369247	1372489	1081	-	*carB*	Carbamoylphosphate synthase, large subunit
AB1379	1372604	1373419	272	+		Polysaccharide deacetylase
AB1380	1373440	1374732	431	-	*nhaA1*	Sodium:hydrogen antiporter
AB1381	1374758	1374952	65	-		Hypothetical protein
AB1382	1374952	1376058	369	-	*metX*	Homoserine O-acetyltransferase
AB1383	1376172	1378526	785	-		TonB-dependent receptor protein
AB1384	1378839	1379099	87	+		Hypothetical protein
AB1385	1379137	1380267	377	-	*ftsZ*	Cell division protein FtsZ
AB1386	1380270	1381658	463	-	*ftsA*	Cell division protein FtsA
AB1387	1381666	1383120	485	-		Conserved hypothetical membrane protein
AB1388	1383186	1385510	775	-	*recD*	Exodeoxyribonuclease V, alpha subunit
AB1389	1385513	1385875	121	-		Conserved hypothetical protein
AB1391	1385875	1386222	116	-		Arsenate reductase
AB1392	1386340	1387161	274	-		Hypothetical protein
AB1393	1387221	1387508	96	-		Conserved hypothetical protein
AB1394	1387498	1387725	76	-		Conserved hypothetical protein
AB1395	1387896	1389584	563	+		Conserved hypothetical membrane protein
AB1396	1389590	1389871	94	+		FlhB domain protein
AB1397	1389888	1391621	578	-		ABC transporter, ATP-binding protein
AB1398	1391727	1392233	169	+		Conserved hypothetical protein (DUF1706 domain protein)
AB1399	1392276	1393460	395	-	*dapD*	2,3,4,5-tetrahydropyridine-2-carboxylate N-succinyltransferase DapD
AB1400	1393565	1395565	667	-		HAD-superfamily hydrolase
AB1401	1395870	1396238	123	+		Hypothetical protein
AB1403	1396295	1398139	615	-	*priA*	Primosomal protein N'
AB1404	1398142	1398534	131	-		Hypothetical protein
AB1405	1398572	1398781	70	-		Hypothetical protein
AB1406	1398781	1398972	64	-		Hypothetical protein
AB1407	1399025	1399435	137	-		Hypothetical protein
AB1408	1399512	1399880	123	-	*hypA*	Hydrogenase expression/formation protein HypA
AB1409	1399987	1400982	332	-	*hypE*	Hydrogenase expression/formation protein HypE
AB1410	1400986	1401339	118	-		Conserved hypothetical protein

AB1411	1401352	1402197	282	-		3-hydroxyisobutyrate dehydrogenase family protein
AB1412	1402197	1403705	503	-		McrBC endonuclease McrB, putative
AB1413	1403701	1404861	387	-		McrBC catalytic subunit McrC, putative
AB1414	1404873	1405994	374	-	*hypD*	Hydrogenase expression/formation protein HypD
AB1415	1406011	1406289	93	-	*hypC*	Hydrogenase expression/formation protein HypC
AB1416	1406418	1407236	273	-	*hypB*	Hydrogenase expression/formation protein HypB
AB1417	1407348	1407986	213	+		Conserved hypothetical protein
AB1418	1408077	1410047	657	+		Methyl-accepting chemotaxis protein
AB1419	1410071	1410910	280	-		Conserved hypothetical membrane protein (DUF161)
AB1420	1410928	1412160	411	-		HI0933-like protein
AB1421	1412237	1413340	368	+		Conserved hypothetical membrane protein (DUF808)
AB1422	1413490	1414068	193	-		Hypothetical protein
AB1423	1414104	1414880	259	-		Conserved hypothetical protein
AB1424	1414990	1415691	234	-		Conserved hypothetical membrane protein (DUF481)
AB1425	1415828	1416127	100	+		Conserved hypothetical protein
AB1426	1416162	1418066	635	-		Hypothetical protein
AB1427	1418154	1419617	488	-	*thiI*	Thiamine biosynthesis protein ThiI
AB1428	1419705	1419959	85	+		Hypothetical protein
AB1429	1419975	1420454	160	-		Hypothetical protein
AB1430	1420483	1421739	419	-		Conserved hypothetical protein (DUF1504 domain protein)
AB1431	1422132	1424378	749	-	*hypF*	Transcriptional regulatory protein HypF
AB1432	1424412	1426028	539	-		Conserved hypothetical protein
AB1433	1426015	1426590	192	-	*hydD*	Ni/Fe hydrogenase, expression/formation protein
AB1434	1426575	1427237	221	-	*hydC*	Ni/Fe hydrogenase, cytochrome b subunit
AB1435	1427262	1428989	576	-	*hydB*	Ni/Fe-hydrogenase, large subunit
AB1436	1428982	1430157	392	-	*hydA*	Ni/Fe-hydrogenase, small subunit
AB1437	1430398	1431354	319	-		Sigma factor regulatory protein, FecR/PupR family
AB1438	1431360	1431833	158	-		Sigma factor, ECF family
AB1439	1431936	1433483	516	-		Conserved hypothetical protein
AB1440	1433452	1434039	196	-	*hyaD*	Ni/Fe hydrogenase, expression/formation protein
AB1441	1434131	1434856	242	-	*hyaC*	Ni/Fe hydrogenase, cytochrome b subunit
AB1442	1434837	1436576	580	-	*hyaB*	Ni/Fe-hydrogenase, large subunit
AB1443	1436579	1437811	411	-	*hyaA*	Ni/Fe-hydrogenase, small subunit
AB1444	1438048	1439394	449	-	*hupL*	Ni/Fe-dependent hydrogenase, large subunit
AB1445	1439400	1440290	297	-	*hupS*	Ni/Fe-dependent hydrogenase, small subunit
AB1446	1440283	1440744	154	-		Transcriptional regulator, TetR family
AB1447	1440985	1443558	858	-	*acnB*	Aconitate hydratase 2

AB1448	1443817	1444254	438	+		Thioesterase/acetyltransferase, putative
AB1449	1444250	1445242	331	-		GGDEF/PAS domain protein
AB1450	1445464	1446207	248	-		Conserved hypothetical protein (DUF28 domain protein)
AB1451	1446307	1447047	247	+		VacJ-like lipoprotein
AB1452	1447054	1447641	196	+		Putative periplasmic protein
AB1453	1447644	1450127	828	+		Conserved hypothetical integral membrane protein
AB1454	1450130	1450690	187	+		Lysine decarboxylase-like protein
AB1455	1450870	1451502	211	+		HAD-superfamily hydrolase
AB1456	1451505	1452068	188	-		Putative lipoprotein thiredoxin
AB1457	1452084	1452728	215	-	*dsbA*	DsbA-like thioredoxin domain protein
AB1458	1452721	1453128	136	-	*dsbB*	Disulfide bond formation protein, DsbB family
AB1459	1453217	1455739	841	+		Glycosyltransferase
AB1460	1455844	1456314	157	+		Sigma factor, ECF family
AB1461	1456317	1457153	279	+		Sigma factor regulatory protein, FecR/PupR family
AB1462	1457255	1459567	771	+		TonB-dependent receptor protein
AB1463	1459749	1461185	479	+		GGDEF domain protein
AB1464	1461133	1462080	316	+		Conserved hypothetical protein (DUF125 domain protein)
AB1465	1462106	1464088	661	-	*betT*	High-affinity choline transport
AB1466	1464225	1465838	538	-	*kefB*	Glutathione-regulated potassium-efflux system protein KefB
AB1467	1465956	1466258	101	+		Conserved hypothetical protein
AB1468	1466335	1467594	420	+	*glyA2*	Serine hydroxymethyltransferase
AB1469	1467624	1468448	275	+	*ada*	O6-methylguanine-DNA methyltransferase
AB1470	1468452	1469162	237	+		Endonuclease III
AB1471	1469148	1469624	159	-		Ribonuclease H-like protein
AB1472	1469664	1469954	97	-		Conserved hypothetical protein
AB1473	1469954	1470157	68	-		Hypothetical protein
AB1474	1470267	1470737	157	-		Pyridoxamine 5'-phosphate oxidase-related, FMN-binding
AB1475	1470917	1472083	389	+	*benE*	Benzoate membrane transport protein
AB1476	1472131	1473300	390	-	*argD2*	N-acetylornithine aminotransferase
AB1477	1473316	1474014	233	-		Conserved hypothetical protein
AB1478	1474098	1474970	291	-	*lipA*	Lipoic acid synthetase
AB1479	1474985	1475686	234	-		Biotin/lipoate A/B protein ligase family protein
AB1480	1475844	1478513	890	+	*aceE*	Pyruvate dehydrogenase E1 component
AB1481	1478527	1480179	551	+	*aceF*	Dihydrolipoamide acetyltransferase
AB1482	1480192	1481622	477	+	*lpdA*	Dihydrolipoamide dehydrogenase
AB1483	1481727	1482389	221	+		Glutamine amidotransferase, class I
AB1484	1482506	1484842	779	-		TonB-dependent receptor protein

AB1485	1485115	1485960	282	-		Aldose 1-epimerase family protein, LacX
AB1486	1485960	1486718	253	-		Beta-lactamase, putative
AB1487	1486772	1488076	435	-	*rhlE*	ATP-dependent RNA helicase RhlE
AB1488	1488347	1489537	397	+		Conserved hypothetical protein
AB1489	1489715	1490692	326	+		DNA (Cytosine-5-)-methyltransferase
AB1490	1490703	1491707	335	+		Conserved hypothetical protein
AB1492	1491864	1492169	102	-		Conserved hypothetical protein, putative transcriptional regulator
AB1493	1492319	1493941	541	+		Conserved hypothetical protein
AB1494	1493979	1494353	125	+		Conserved hypothetical protein (DUF302 domain protein)
AB1495	1494359	1494829	157	+		Conserved hypothetical protein
AB1496	1494861	1497098	746	-		Methyl-accepting chemotaxis protein
AB1497	1497253	1498056	268	+	*rnhA*	Ribonuclease HI
AB1498	1498111	1499076	322	+		HMGL family protein
AB1499	1499110	1499409	100	-		Conserved hypothetical protein
AB1500	1499452	1499859	136	-		Conserved hypothetical protein
AB1501	1499988	1501250	421	-	*dinP*	DNA polymerase IV
AB1502	1501328	1502560	411	-	*moeA1*	Molybdenum cofactor biosynthesis protein A
AB1503	1502573	1503553	327	-		Cysteine desulfurase, NifS homolog
AB1504	1503549	1504379	277	-	*fdhD*	FdhD/NarQ protein required for formate dehydrogenase activity
AB1505	1504379	1505335	319	-	*fdhC*	Putative FdhC protein
AB1506	1505347	1505937	197	-	*fdhB1*	Formate dehydrogenase, iron-sulfur subunit FdhB
AB1507	1505951	1508749	933	-	*fdhA1*	Formate dehydrogenase, large subunit FdhA (Selenocysteine containing)
AB1509	1508778	1508999	74	-		Conserved hypothetical protein
AB1510	1509085	1509693	203	-		Conserved hypothetical protein
AB1511	1509689	1511359	557	-		4Fe-4S ferredoxin, iron-sulfur binding
AB1512	1511506	1512657	384	+	*livJ*	Leucine/isoleucine/valine-binding protein
AB1513	1512657	1514225	523	+		Two-component sensor histidine kinase
AB1514	1514236	1514892	219	+		Two-component response regulator
AB1515	1514888	1516138	417	+		Mn2+ and Fe2+ transporter, NRAMP family
AB1516	1516145	1516423	93	-		Heavy-metal transporting ATPase
AB1517	1516430	1516753	108	-		Heavy-metal transport protein, MerT homolog
AB1518	1516753	1517844	364	-		Transglutaminase family protein
AB1519	1518051	1518992	314	-		Putative FdhC protein
AB1520	1519004	1519594	197	-	*fdhB2*	Formate dehydrogenase, iron-sulfur subunit FdhB
AB1521	1519608	1522403	932	-	*fdhA2*	Formate dehydrogenase, large subunit FdhA (Cysteine containing)
AB1522	1522428	1522649	74	-		Formate dehydrogenase subunit E, putative
AB1523	1523138	1524172	345	+	*selD*	Selenide, water dikinase

AB1524	1524220	1524771	184	-		Hypothetical protein
AB1525	1524949	1525524	192	+		Hypothetical protein
AB1526	1525578	1526204	209	+		Conserved hypothetical protein
AB1527	1526219	1526479	87	+		Conserved hypothetical protein
AB1528	1526484	1526855	124	+		Hypothetical protein
AB1529	1526869	1527918	350	+		Hypothetical protein
AB1530	1527931	1528413	161	+		Phage-related lysozyme
AB1531	1528477	1528848	124	-		Hypothetical protein
AB1532	1528910	1529317	136	-		Sel1-like repeat protein
	**1529459**	**1529553**		**-**		**tRNA-SeC (anticodon: TCA)**
AB1533	1529610	1530962	451	+	*selA*	L-seryl-tRNA selenium transferase
AB1534	1530965	1532791	609	+	*selB*	Selenocysteine-specific elongation factor
AB1535	1532798	1534309	504	+		Putative DNA helicase
AB1536	1534309	1535244	312	+		Conserved hypothetical protein
AB1537	1535244	1537304	687	+		Hypothetical protein
AB1538	1537354	1538277	308	+		Conserved hypothetical protein
AB1539	1538365	1540533	723	+		Hypothetical protein
AB1540	1540576	1541319	248	+		Conserved hypothetical protein
	**1541409**	**1541480**		**+**		**tRNA-Gln-2 (anticodon: TTG)**
	**1541505**	**1541586**		**+**		**tRNA-Tyr-1 (anticodon: GTA)**
	**1541607**	**1541679**		**+**		**tRNA-Phe-1 (anticodon: GAA)**
	**1541707**	**1541790**		**+**		**tRNA-Leu-3 (anticodon: TAA)**
AB1541	1541812	1543707	632	-		Two-component sensor histidine kinase
AB1542	1543848	1544354	169	+		Putative FMN reductase
AB1543	1544415	1545788	458	+		PhoH family protein
AB1544	1545860	1547473	538	+		Conserved hypothetical protein
AB1545	1547482	1549326	615	-		EAL/PAS/GGDEF domain protein
AB1546	1549302	1549937	212	+	*pdxH*	Pyridoxamine 5'-phosphate oxidase
AB1547	1549949	1552063	705	-		GGDEF/PAS domain protein
AB1548	1552104	1553465	454	-	*dbpA*	ATP-dependent RNA helicase DbpA
AB1549	1553471	1554001	177	-		Conserved hypothetical integral membrane protein
AB1550	1554096	1554950	285	+		Conserved hypothetical protein
AB1551	1554977	1555558	194	-		Hypothetical protein
AB1552	1555698	1557605	636	-	*htpG*	Heat shock protein 90 HtpG
AB1553	1557824	1560028	735	+	*katG*	Catalase/peroxidase HPI
AB1555	1560142	1562022	627	+	*ciaB*	CiaB protein
AB1557	1562041	1562508	156	-		Ankyrin repeat protein

AB1558	1562533	1565184	884	-	*pqiB*	Paraquat-inducible protein B
AB1559	1565177	1566391		-	*pqiA*	Paraquat-inducible protein A (pseudogene)
AB1561	1566462	1566893	144	+		Protozoan/cyanobacterial globin homolog
AB1562	1566969	1569521	851	+		EAL/GGDEF domain protein
AB1563	1569538	1570017	160	-		Hypothetical protein
AB1564	1570121	1571059	313	+		Putative integral membrane protein (DUF6 domain protein)
AB1565	1571400	1571864	155	-		Conserved hypothetical membrane protein (DUF6)
AB1566	1571993	1572577	195	+		Conserved hypothetical protein
AB1567	1572585	1573655	357	-		Putative DNA alkylation repair enzyme
AB1568	1573667	1574524	286	-		Putative DNA-binding protein
AB1569	1574595	1575956	454	-		Hypothetical protein
AB1570	1575970	1576611	214	-		Transcriptional regulator, ThiJ/PfpI family
AB1572	1576789	1577445	219	+		Conserved hypothetical protein
AB1573	1577731	1580109	793	-		TonB-dependent receptor protein
AB1574	1580277	1580498	74	+	*hicA*	Hif-contiguous protein A
AB1575	1580525	1580866	114	+	*hicB*	Hif-contiguous protein B
AB1576	1580877	1581848	324	-		Sigma factor regulatory protein, FecR/PupR family
AB1577	1581852	1582322	157	-		Sigma factor, ECF family
AB1578	1582459	1583517	353	+	*speB*	Arginase/agmatinase/formiminoglutamate hydrolase, arginase family
AB1579	1583546	1584844	433	-	*leuA2*	2-isopropylmalate synthase
AB1580	1585028	1585810	261	-		Transcriptional regulator, AraC family
AB1581	1585823	1586149	109	-		Transcriptional regulator, PemK family
AB1582	1586146	1586403	86	-		Hypothetical protein
AB1583	1586475	1587425	317	-		Transcriptional regulator, AraC family
AB1584	1587544	1589658	705	+		TonB-dependent receptor protein
AB1585	1589690	1590262	191	-	*sodB*	Superoxide dismutase
AB1586	1590343	1591548	402	-		Conserved hypothetical membrane protein
AB1587	1591623	1592540	306	-	*ppa*	Inorganic pyrophosphatase, manganese-dependent
AB1588	1592663	1593259	199	-		Conserved hypothetical protein (DUF1121 domain protein)
AB1589	1593268	1593804	179	-		Conserved hypothetical protein, putative asparaginase
AB1590	1593758	1595161	468	-	*aspA*	Aspartate ammonia-lyase
AB1591	1595230	1595907	226	-		Two-component response regulator
AB1592	1595913	1597034	374	-		Two-component sensor histidine kinase
AB1593	1597109	1598542	478	-		Sodium:alanine symporter
AB1594	1598678	1599370	231	-	*pfs*	5'-methylthioadenosine\S-adenosylhomocysteine nucleosidase
AB1595	1599366	1600292	309	-	*fabD*	Malonyl coenzyme A-(acyl carrier protein) transacylase
AB1596	1600430	1601014	195	-	*slyD*	Peptidyl-prolyl cis-trans isomerase

AB1597	1601039	1601980	314	-		Conserved hypothetical protein
AB1598	1601985	1602506	174	-		OmpA/MotB precursor
AB1599	1602670	1603959	430	-	*tolB*	Colicin tolerance-like protein (tolB)
AB1600	1603967	1604695	243	-		Conserved hypothetical protein
AB1601	1604704	1605090	129	-	*exbD3*	Biopolymer transport protein ExbD
AB1602	1605098	1605655	186	-	*exbB3*	Biopolymer transport protein ExbB
AB1603	1605665	1606039	125	-	*atpC*	ATP synthase F1 sector, epsilon subunit
AB1605	1606066	1607457	464	-	*atpD*	ATP synthase F1 sector, beta subunit
AB1606	1607480	1608364	295	-	*atpG*	ATP synthase F1 sector, gamma subunit
AB1607	1608378	1609892	505	-	*atpA*	ATP synthase F1 sector, alpha subunit
AB1608	1609917	1610444	176	-	*atpH*	ATP synthase F1 sector, delta subunit
AB1609	1610447	1610956	170	-	*atpF*	ATP synthase F0 sector, subunit B
AB1610	1610973	1611392	140	-	*atpF'*	ATP synthase F0 sector, B' subunit
AB1611	1611514	1612365	284	-	*parB*	Transcriptional regulator involved in chromosome partitioning ParB
AB1612	1612389	1613162	258	-	*parA*	ATPases involved in chromosome partitioning ParA
AB1613	1613162	1613794	211	-	*birA*	Biotin--acetyl-CoA-carboxylase ligase
AB1614	1613891	1614589	233	-		Hypothetical protein
AB1615	1614821	1615711	297	+		Auxin efflux carrier protein
AB1616	1615722	1616639	306	-	*fmt*	10-formyltetrahydrofolate:L-methionyl-tRNA(fMet) N-formyltransferase
AB1617	1616652	1617413	254	-	*proB*	Glutamate 5-kinase
AB1618	1617413	1618501	363	-	*obg*	GTP-binding protein
AB1619	1618634	1618888	85	-	*rpmA*	50S ribosomal protein L27
AB1620	1618916	1619230	105	-	*rplU*	50S ribosomal protein L21
AB1621	1619367	1620998	544	-	*dnaG*	DNA primase
AB1622	1621080	1622126	349	+		Conserved hypothetical protein
AB1623	1622177	1622848	224	+	*rnc*	Ribonuclease III
AB1624	1622855	1623928	358	+	*aroC*	Chorismate synthase
AB1625	1623921	1624670	250	+		Conserved hypothetical protein
AB1626	1624751	1625599	283	-		Peptidase, M48 family
AB1627	1625656	1626339	228	-		Conserved hypothetical protein
AB1628	1626436	1626912	159	+		Phosphoglycerate/bisphosphoglycerate mutase, putative
AB1629	1626934	1627344	137	-		Hypothetical protein
AB1630	1627349	1628686	446	-	*trmE*	tRNA modification GTPase
AB1631	1628692	1629564	291	-		Conserved hypothetical protein
AB1632	1629588	1631186	533	-	*oxaA*	Inner membrane protein, 60 kDa
AB1633	1631182	1631511	110	-		Conserved hypothetical protein (DUF37 domain protein)
AB1633.5	1631511	1631843		-	*rnpA*	Ribonucleoside P protein component RnpA (pseudogene)

AB1634	1631834	1631965	44	-	*rpmH*	50S ribosomal protein L34
AB1635	1632043	1634619	859	-	*clpB*	ATP-dependent Clp protease, ATP-binding subunit
AB1636	1634738	1635313	192	+	*rnhB*	Ribonuclease HII
AB1637	1635321	1635632	104	-		Hypothetical protein (DUF77 domain protein)
AB1638	1635645	1636535	297	-		Conserved hypothetical protein (DUF344 domain protein)
AB1639	1636655	1636993	113	+		Conserved hypothetical protein
AB1640	1636996	1637487	164	-	*msrA*	Peptide methionine sulfoxide reductase
AB1641	1637493	1637849	119	-	*msrB*	Peptide methionine sulfoxide reductase
AB1642	1637864	1638475	204	-		Fumarylacetoacetate (FAA) hydrolase
AB1643	1638538	1638876	113	+		Conserved hypothetical protein (DUF1291 domain protein)
AB1644	1638879	1639532	218	-		Phosphohydrolase (MUTT/NUDIX family protein)
AB1645	1639536	1640801	422	-	*bioA*	7,8-diaminopelargonic acid synthetase
AB1646	1640852	1643446	865	-		Two-component sensor histidine kinase
AB1647	1643565	1645094	510	-	*purH*	Phosphoribosylaminoimidazolecarboxamide formyltransferase / IMP cyclohydrolase
AB1648	1645297	1647507	737	-	*purL*	Phosphoribosylformylglycinamidine synthase II
AB1649	1647575	1648543	323	-		Conserved hypothetical protein
AB1650	1648614	1649801	396	+		Peptidase, M23/M37 family
AB1651	1649849	1650418	190	-	*folE*	GTP cyclohydrolase I
AB1652	1650444	1651394	317	-	*corA*	Magnesium and cobalt transport protein
AB1653	1651413	1652600	396	-	*ctsF*	Campylobacter transformation system protein CtsF
AB1654	1652605	1653603	333	-	*ctsE*	Campylobacter transformation system protein CtsE
AB1655	1653878	1654447	190	-		Hypothetical protein
AB1656	1654462	1655106	215	-		Conserved hypothetical protein
AB1657	1655167	1655817	217	+		Conserved hypothetical protein
AB1658	1655851	1656423	191	-		Hypothetical protein
AB1659	1656511	1657290	260	-		N6 adenine-specific DNA methyltransferase, D12 class
AB1660	1657401	1658345	315	-		Phage tail protein D
AB1661	1658342	1658545	68	-		Hypothetical protein
AB1662	1658545	1658910	122	-		Hypothetical protein
AB1663	1658913	1660814	634	-		Phage tail tape measure protein, TP901 family, putative
AB1664	1660963	1661235	91	-		Hypothetical protein
AB1665	1661252	1661740	163	-		Phage major tail tube protein
AB1666	1661745	1662926	394	-		Phage tail sheath protein
AB1667	1662957	1663199	81	-		Hypothetical protein
AB1668	1663192	1663794	201	-		Tail fiber assembly protein
AB1669	1663810	1664916	369	-		Hypothetical protein
AB1670	1664916	1665551	212	-		Tail protein I, putative

AB1671	1665526	1666698	391	-		Baseplate assembly protein J, putative
AB1672	1666698	1667012	105	-		Hypothetical protein, putative baseplate assembly protein W
AB1673	1667015	1667743	243	-		Baseplate assembly protein V, putative
AB1674	1667743	1668057	105	-		Hypothetical protein
AB1675	1668158	1668382	75	-		Hypothetical protein
AB1676	1668378	1668701	108	-		Hypothetical protein
AB1677	1668877	1669314	146	+		Hypothetical protein
AB1678	1669310	1670107	266	+		Mu-like prophage I protein, putative
AB1679	1670123	1670557	145	+		Hypothetical protein
AB1680	1670570	1671550	327	+		Conserved hypothetical protein
AB1681	1671676	1672074	133	+		Conserved hypothetical protein
AB1682	1672077	1672580	168	+		Conserved hypothetical protein
AB1683	1672570	1674288	573	+		Phage uncharacterized protein
AB1684	1674292	1675680	463	+		Phage uncharacterized protein
AB1685	1675676	1676848	391	+		Mu-like prophage F protein, putative
AB1686	1676951	1677433	161	+		Mu-like prophage G protein, putative
AB1687	1677431	1677703	91	-		Conserved hypothetical protein
AB1688	1677853	1679136	428	+		Hypothetical protein
AB1690	1679284	1680453	390	+		Conserved hypothetical protein
AB1692	1680928	1681830	301	-		DnaB-like helicase-like protein
AB1693	1681826	1682128	101	-		Hypothetical protein
AB1694	1682112	1682321	70	-		Hypothetical protein
AB1696	1682694	1683281	196	-		Hypothetical protein
AB1697	1683271	1683924	218	-		Phage uncharacterized protein
AB1698	1683990	1684238	83	-		Hypothetical protein
AB1700	1684345	1684581	79	-		Hypothetical protein
AB1701	1684554	1685216	221	-		Hypothetical protein
AB1702	1685209	1685904	232	-		Prophage Mu, DNA transposition protein B
AB1703	1685979	1688081	701	-		Prophage Mu, DNA transposition protein A
AB1704	1688086	1688310	75	-		Hypothetical protein
AB1705	1688706	1689380	225	+		Phage repressor protein, putative
AB1706	1689400	1690146	249	+		Hypothetical protein
AB1707	1690557	1691051	165	-		Hypothetical protein
AB1708	1691035	1691910	292	-		Hypothetical protein
AB1709	1691986	1692867	294	-	*era*	GTP-binding protein Era homolog
AB1710	1692946	1693653	236	-		Hypothetical protein
AB1711	1693663	1695165	501	-		Mg chelatase-related protein

AB1712	1695366	1695737	124	+		Hypothetical protein
AB1713	1695748	1696260	171	-	*def*	Polypeptide deformylase
AB1714	1696272	1696853	194	-	*clpP*	ATP-dependent Clp protease, proteolytic subunit
AB1715	1696871	1698169	433	-	*tig*	Trigger factor
AB1716	1698320	1699453	378	-		Conserved hypothetical protein
AB1718	1699457	1700410	318	-		Conserved hypothetical protein
AB1719	1700437	1701579	381	-	*nspC*	Carboxynorspermidine decarboxylase
AB1720	1701586	1702785	400	-		Saccharopine dehydrogenase (L-lysine-forming)
AB1721	1703478	1704635	386	+		Site-specific recombinase, phage integrase family
AB1722	1704660	1705385	242	+		Conserved hypothetical protein
AB1723	1705737	1705985	83	+		Hypothetical protein
AB1724	1705912	1706655	248	+		Conserved hypothetical protein
AB1725	1706658	1707332	225	+		Hypothetical protein
AB1726	1707337	1708869	511	+		Hypothetical protein
AB1727	1708884	1709546	221	+		Hypothetical protein
AB1728	1710033	1711844	604	-		ATPase, putative
AB1729	1711844	1713613	590	-		Hypothetical protein
AB1730	1713606	1714949	448	-	*hsdS*	Type I restriction-modification system specificity determinant
AB1731	1714949	1717261	771	-	*hsdM*	Type I restriction-modification system, M subunit, putative
AB1732	1717694	1718155	154	+		Conserved hypothetical protein, putatve DnaJ domain protein
AB1733	1718375	1718671	99	-		Hypothetical protein
AB1734	1719300	1719665	122	-		Hypothetical protein
AB1735	1719682	1720416	245	-		Hypothetical protein, ErfK/YbiS/YcfS/YnhG family
AB1736	1720416	1720577	54	-		Hypothetical protein
AB1737	1720707	1721855	383	+	*metC1*	Cystathionine gamma-synthase
AB1738	1721860	1723353	498	+	*metC2*	Cystathionine gamma-synthase
AB1739	1723372	1724034	221	-		DNA-binding response regulator
AB1740	1724123	1726126	668	+		Methyl-accepting chemotaxis protein
AB1741	1726149	1726739	197	+		Hypothetical protein
AB1742	1726759	1728951	731	+		Response regulator receiver:Metal-dependent phosphohydrolase, HD subdomain
AB1743	1729094	1731205	704	+		Methyl-accepting chemotaxis protein
AB1744	1731313	1734162	950	+		Two-component sensor histidine kinase
AB1745	1734189	1735061	291	-		Auxin efflux carrier protein
AB1746	1735264	1736130	289	-		Hypothetical protein
AB1747	1736112	1736582	157	-		Hypothetical protein
AB1748	1736585	1737907	441	-		Hypothetical protein
AB1749	1737907	1739304	466	-		Conserved hypothetical protein, putative cytochrome

AB1750	1739389	1740051	221	-		Two-component response regulator
AB1751	1740161	1740712	184	+		Acetyltransferase, GNAT family
AB1752	1740715	1742154	480	-		Putative nickel transporter
AB1753	1742154	1743077	308	-		Putative cation ABC transporter, periplasmic-binding protein
AB1754	1743200	1743571	124	+		Transcriptional regulator, Fur family
AB1755	1743564	1744751	396	+	*nhaA2*	Sodium:hydrogen antiporter
AB1756	1744770	1745903	378	-		Diheme cytochrome c peroxidase
AB1757	1745906	1746808	301	-		Sterol desaturase-related protein
AB1758	1746819	1747760	314	-		Hypothetical protein
AB1759	1747760	1749157	466	-		Conserved hypothetical protein (DUF1111 domain protein)
AB1760	1749126	1750478	451	-		Conserved hypothetical protein
AB1761	1750579	1752654	692	-		Conserved hypothetical protein
AB1763	1752826	1753137	104	+	*atpE*	ATP synthase F0 sector, C subunit
AB1764	1753279	1755399	707	+		Methyl-accepting chemotaxis protein
	**1755477**	**1755558**		**+**		**tRNA-Leu-2 (anticodon: GAG)**
AB1765	1755664	1756704	347	-		Conserved hypothetical transmembrane protein
AB1766	1756794	1757594	267	-		Ion transport protein
AB1767	1757657	1758037	127	-		Hypothetical protein
AB1768	1758050	1758361	104	-		Hypothetical protein
AB1769	1758647	1758862	72	+		Conserved hypothetical protein
AB1770	1758877	1759629	251	+	*thiD*	Phosphomethylpyrimidine kinase
AB1771	1759707	1760942	412	+	*proA*	Gamma-glutamyl phosphate reductase
AB1772	1760948	1762834	629	+		Glycosyl hydrolase, putative
AB1773	1762868	1763512	215	-		Hypothetical protein
AB1774	1763645	1764955	437	+		Ferredoxin-like protein
AB1775	1764963	1766279	439	-		Two-component sensor histidine kinase
AB1776	1766272	1766949	226	-		Two-component response regulator
AB1777	1766992	1767507	172	+	*ogt*	Putative methylated-DNA-protein-cysteine methyltransferase
AB1778	1767500	1768906	469	+		Phosphohexosemutase
AB1779	1768884	1769606	241	+		Conserved hypothetical protein, putative nitrilase/cyanide hydratase
AB1780	1769686	1770180	165	+		Conserved hypothetical protein
AB1781	1770421	1770756	112	+		Conserved hypothetical protein
AB1782	1770753	1771508	252	+		Conserved hypothetical protein
AB1783	1771524	1772018	165	+	*fldA*	Flavodoxin
AB1784	1772048	1772494	149	-	*fur2*	Ferric uptake regulation protein
AB1785	1772583	1772954	124	-		Hypothetical protein
AB1786	1773032	1773766	245	-	*ate*	Putative arginyl-tRNA--protein transferase

AB1787	1773772	1774509	246	-	*trpA*	Tryptophan synthase, alpha chain
AB1788	1774566	1775960	465	-		Voltage-gated chloride channel family protein
AB1789	1776133	1776939	269	+	*panB*	3-methyl-2-oxobutanoate hydroxymethyltransferase
AB1790	1776946	1777980	345	+	*ruvB*	Holliday junction DNA helicase RuvB
AB1791	1777980	1779023	348	+	*amaA*	Acid membrane antigen A
AB1792	1779026	1779757	244	-		Conserved hypothetical protein
	**1779945**	**1780064**		**-**	***rrnB***	**5S ribosomal RNA**
	**1780226**	**1783123**		**-**	***rrnB***	**23S ribosomal RNA**
	**1783518**	**1783590**		**-**		**tRNA-Ala-2 (anticodon: TGC)**
	**1783648**	**1783721**		**-**		**tRNA-Ile-2 (anticodon: GAT)**
	**1783830**	**1785341**		**-**	***rrnB***	**16S ribosomal RNA**
	**1785565**	**1785636**		**-**		**tRNA-Asn-1 (anticodon: GTT)**
AB1795	1785712	1786635	308	-		Radical SAM domain protein
AB1796	1786716	1787753	346	-	*hemE*	Uroporphyrinogen decarboxylase
AB1797	1787769	1788278	170	-		Putative integral membrane protein
AB1798	1788281	1789315	345	-	*asd*	Aspartate-semialdehyde dehydrogenase
AB1799	1789321	1791900	860	-	*gyrA*	DNA gyrase, subunit A
AB1800	1792042	1792254	71	-		Conserved hypothetical protein
AB1801	1792341	1793519	393	-	*argJ*	Ornithine acetyltransferase / N-acetylglutamate synthase
AB1802	1793524	1794648	375	-		Potassium channel protein, putative
AB1803	1794675	1794860	62	-	*rpmB*	50S ribosomal protein L28
AB1804	1794929	1795792	288	-		Hypothetical protein
AB1805	1796039	1796404	122	+		Putative heptose phosphatase
AB1806	1796416	1797420	335	+	*waaD*	ADP-L-glycero-D-manno-heptose-6-epimerase
AB1807	1797424	1798857	478	+	*waaE*	ADP-heptose synthase
AB1808	1798841	1799407	189	+	*gmhA*	D-sedoheptulose 7-phosphate isomerase
AB1809	1799500	1801395	632	+		Putative sulfatase
AB1810	1801420	1802355	312	-	*waaF*	Lipopolysaccharide heptosyltransferase II
AB1811	1802351	1803409	353	-		Conserved hypothetical protein
AB1812	1803405	1804322	306	-		Putative heptosyltransferase
AB1813	1804325	1805071	249	-		Conserved hypothetical protein
AB1814	1805206	1806849	548	+		Conserved hypothetical membrane protein
AB1815	1806873	1807427	185	-		Conserved hypothetical protein
AB1816	1807439	1808329	297	-		Hypothetical protein
AB1817	1808341	1809795	485	-		Putative acetyltransferase
AB1818	1809804	1810928	375	-		Putative glycosyltransferase
AB1819	1810894	1811640	249	-		Putative glycosyltransferase

AB1820	1811720	1812787	356	+		Conserved hypothetical protein
AB1821	1812813	1813823	337	+		Probable glycosyltransferase
AB1822	1813839	1814426	196	-		Conserved hypothetical protein
AB1823	1814426	1815547	374	-		Glycosyltransferase
AB1824	1815547	1816881	445	-		Putative O-antigen polymerase
AB1825	1816896	1817966	357	-		Aminotransferase, DegT/DnrJ/EryC1/StrS family
AB1826	1817971	1818996	342	-		dTDP-glucose 4,6-dehydratase
AB1827	1818989	1819867	293	-		Glucose-1-phosphate thymidylyltransferase
AB1829	1819870	1821870	667	-		Phosphoglycerol transferase
AB1830	1821887	1822237	117	-	*dgkA*	Diacylglycerol kinase
AB1831	1822242	1823012	257	-		Conserved hypothetical protein
AB1832	1823012	1823923	304	-	*waaM*	Lipid A biosynthesis lauroyl acyltransferase
AB1833	1823919	1824920	334	-	*waaC*	Lipopolysaccharide heptosyltransferase I
AB1834	1824920	1826386	489	-	*gppA*	Guanosine pentaphosphate phosphohydrolase (gppA)
AB1835	1826389	1826640	84	-	*fdxB*	Ferredoxin
AB1836	1826687	1827433	249	-		Inositol monophosphatase family protein
AB1837	1827439	1828812	458	-	*gltD*	Glutamate synthase, small chain
AB1838	1828821	1833257	1479	-	*gltB*	Glutamate synthase, large chain
AB1839	1833455	1834246	264	-		Conserved hypothetical protein
AB1840	1834253	1835035	261	-		Conserved hypothetical protein
AB1841	1835234	1837615	794	-		Methyl-accepting chemotaxis protein
AB1842	1838232	1840184	651	-		Methyl-accepting chemotaxis protein
AB1843	1840200	1840367	56	-		Hypothetical protein
AB1844	1840721	1842154	478	-	*gspD*	General secretion pathway protein D
AB1845	1842187	1842756	190	-		Conserved hypothetical protein
AB1846	1842783	1843478	232	+	*tlyA*	Hemolysin A
AB1847	1843450	1844292	281	+	*ribF*	Riboflavin kinase/FAD synthase RibF
AB1848	1844327	1845031	235	+		Methyltransferase, putative
AB1849	1845047	1845550	168	+	*bcp*	Bacterioferritin comigratory protein, alkyl hydroperoxide reductase/thiol specific antioxidant
AB1850	1845565	1846566	334	+		Hypothetical protein
AB1851	1846569	1847954	462	+	*glcD*	Glycolate oxidase
AB1852	1847965	1848747	261	+	*rbn*	tRNA-processing ribonuclease BN
AB1853	1848750	1849313	188	-		Hypothetical protein
AB1854	1849319	1850584	422	-	*murA*	UDP-N-acetylglucosamine 1-carboxyvinyltransferase
AB1855	1850702	1851583	294	+		Conserved hypothetical membrane protein (DUF6)
AB1856	1851599	1852399	267	+	*kdsA*	3-deoxy-D-manno-octulosonic acid 8-phosphate synthase

AB1857	1852436	1852903	156	+	*ribH*	Riboflavin synthase, beta subunit
AB1858	1852909	1853301	131	+	*nusB*	Transcription termination factor NusB
AB1859	1853306	1854001	232	+	*pyrF*	Orotidine 5'-phosphate decarboxylase
	**1854100**	**1854187**		**+**		**tRNA-Ser-1 (anticodon: GCT)**
	**1854195**	**1854268**		**+**		**tRNA-Met-2 (anticodon: CAT)**
AB1860	1854561	1855400	280	+		ABC transporter, ATP-binding protein
AB1861	1855403	1857013	537	+		Hypothetical protein
AB1862	1857030	1857251	74	+	*feoA*	Ferrous iron transport protein A
AB1863	1857251	1859704	818	+	*feoB2*	Ferrous iron transport protein B
AB1864	1859751	1860050	100	+		Conserved hypothetical protein
AB1865	1860077	1860565	163	-		Conserved hypothetical protein
AB1866	1860579	1862159	527	-		Conserved hypothetical protein
AB1867	1862171	1862473	101	-		Conserved hypothetical protein
AB1868	1862605	1864725	707	-		Heavy metal-(Cd/Co/Hg/Pb/Zn)-translocating P-type ATPase
AB1869	1864733	1865071	113	-		Transcriptional regulator, ArsR family
AB1870	1865189	1867312	708	-		TonB-dependent receptor protein
AB1871	1867525	1868076	184	+		Hypothetical protein
AB1872	1868079	1871156	1026	+		AcrB/AcrD/AcrF family protein
AB1873	1871162	1871857	232	+		Conserved hypothetical protein
AB1874	1871860	1873014	385	+		Conserved hypothetical protein
AB1875	1873224	1873439	72	+	*cspA*	Cold-shock protein, DNA-binding
AB1876	1873476	1873613	46	+		Hypothetical protein
AB1877	1873643	1874335	231	-		Two-component response regulator
AB1878	1874396	1876096	567	+		Two-component sensor histidine kinase
	**1876219**	**1876292**		**+**		**tRNA-Gly-3 (anticodon: TCC)**
	**1876329**	**1876402**		**+**		**tRNA-Met-3 (anticodon: CAT)**
AB1879	1876559	1878091	511	+		Hypothetical protein
AB1880	1878625	1879416	264	+		Replication protein
AB1881	1879445	1880161	239	-		Hypothetical protein
AB1882	1880425	1881891	489	+		Hypothetical protein
	**1882130**	**1882203**		**+**		**tRNA-Met-4 (anticodon: CAT)**
	**1882217**	**1882290**		**+**		**tRNA-Thr-2 (anticodon: TGT)**
	**1882311**	**1882382**		**+**		**tRNA-Gln-3 (anticodon: TTG)**
AB1883	1882550	1883143	198	+	*ahpC*	Alkyl hydroperoxide reductase/ Thiol specific antioxidant
AB1884	1883158	1883472	105	+	*trxA1*	Thioredoxin
AB1885	1883531	1883815	95	-		Conserved hypothetical protein
AB1886	1883861	1884241	127	-		Hypothetical protein

AB1887	1884280	1888812	1511	-	*rpoC*	DNA-directed RNA polymerase, beta' chain
AB1888	1888805	1892950	1382	-	*rpoB*	DNA-directed RNA polymerase, beta chain
AB1889	1893110	1893478	123	-	*rplL*	50S ribosomal protein L7/L12
AB1890	1893533	1894018	162	-	*rplJ*	50S ribosomal protein L10
AB1891	1894182	1894877	232	-	*rplA*	50S ribosomal protein L1
AB1892	1894956	1895378	141	-	*rplK*	50S ribosomal protein L11
AB1893	1895476	1896000	175	-	*nusG*	Transcription termination factor NusG
AB1894	1896013	1896192	60	-	*secE*	Preprotein translocase, SecE subunit
	**1896275**	**1896347**		**-**		**tRNA-Trp (anticodon: CCA)**
AB1895	1896390	1896557	56	-	*rpmG*	50S ribosomal protein L33
AB1896	1896573	1897778	402	-	*tufA*	Elongation factor Tu
	**1897992**	**1898065**		**-**		**tRNA-Gly-4 (anticodon: TCC)**
	**1898078**	**1898159**		**-**		**tRNA-Tyr-2 (anticodon: GTA)**
	**1898220**	**1898293**		**-**		**tRNA-Thr-3 (anticodon: TGT)**
	**1898308**	**1898380**		**-**		**tRNA-Phe-2 (anticodon: GAA)**
AB1897	1898420	1899595	392	-	*murD*	UDP-N-acetylmuramoylalanine--D-glutamate ligase
AB1898	1899608	1900666	353	-	*mraY*	Phospho-N-acetylmuramoyl-pentapeptide transferase
AB1899	1900736	1902208	491	+	*pgm*	Phosphoglycerate mutase, 2,3-bisphosphoglycerate-independent
AB1900	1902211	1902897	229	+		ABC transporter, ATP-binding protein
AB1901	1902904	1904880	659	+	*pbpA*	Penicillin-binding protein 1A
AB1902	1905097	1906521	475	-	*glnA*	Glutamine synthetase
AB1903	1906622	1907410	263	-	*hisJ*	Histidinol-phosphate phosphatase
AB1904	1907410	1907844	145	-	*moaE*	Molybdopterin converting factor, subunit 2
AB1905	1907850	1908068	73	-	*moaD*	Molybdopterin converting factor, subunit 1
AB1906	1908138	1908794	219	+		Conserved hypothetical protein (DUF178 domain protein)
AB1907	1908794	1909561	256	+	*uppP*	Undecaprenyl diphosphatase, putative
AB1908	1909556	1910575	340	-	*murG*	UDP-N-acetylglucosamine--N-acetylmuramyl-(pentapeptide) pyrophosphoryl-undecaprenol N-acetylglucosamine transferase
AB1909	1910575	1911861	429	-	*pbpB*	Penicillin-binding protein
AB1910	1911890	1913749	620	+	*ftsW*	Cell division protein FtsW
AB1911	1913773	1914276	168	+	*ppi*	Peptidyl-prolyl cis-trans isomerase
	**1914787**	**1914906**		**-**	***rrnC***	**5S ribosomal RNA**
	**1915068**	**1917965**		**-**	***rrnC***	**23S ribosomal RNA**
	**1918360**	**1918432**		**-**		**tRNA-Ala-3 (anticodon: TGC)**
	**1918490**	**1918563**		**-**		**tRNA-Ile-3 (anticodon: GAT)**
	**1918672**	**1920183**		**-**	***rrnC***	**16S ribosomal RNA**
	**1920406**	**1920477**		**-**		**tRNA-Asn-2 (anticodon: GTT)**

AB1912	1920624	1921127	168	-		Cytochrome c
AB1913	1921199	1923409	737	-	*clpA*	ATP-dependent Clp protease, ATP-binding subunit
AB1914	1923414	1923710	99	-	*clpS*	ATP-dependent Clp protease adaptor protein ClpS
AB1915	1923788	1924423	212	+	*bioD*	Dethiobiotin synthetase
AB1916	1924446	1924607	54	-		Hypothetical protein
AB1917	1924710	1925840	377	+		CitE domain protein
AB1918	1925860	1928376	839	+		AMP-dependent synthetase and ligase/CitE domain protein
AB1919	1928389	1929444	352	+		MaoC family protein, putative enoyl-CoA hydratase
AB1920	1929480	1930460	327	+		CitE domain protein
AB1921	1930640	1932130	497	+	*fumA*	Fumarate hydratase, class I
AB1922	1932293	1933126	278	+		Conserved hypothetical protein
AB1923	1933143	1936052	970	-		Methyl-accepting chemotaxis protein
AB1924	1936236	1937483	416	-	*arsB*	Arsenical pump membrane protein
AB1925	1937480	1937788	103	-	*arsR*	Transcriptional regulator, ArsR family
AB1926	1937791	1938024	78	-		Putative redox-active disulfide protein
AB1927	1938032	1938994	321	-		Conserved hypothetical membrane protein (DUF318 domain protein)
AB1928	1938998	1939393	132	-	*arsC*	Arsenate reductase
AB1929	1939505	1940110	202	-		Conserved hypothetical protein
AB1930	1940194	1941375	394	-		Putative integral membrane protein
AB1931	1941412	1943520	703	-	*flhA*	Flagellar biosynthesis protein FlhA
AB1932	1943543	1944670	376	-	*flgL*	Flagellar hook-associated protein FlgL
AB1933	1944670	1945974	435	-	*fliI*	Flagellum-specific ATP synthase FliI
AB1934	1945974	1947971	666	-		Tetratricopeptide repeat domain protein
AB1935	1947978	1949024	349	-	*flhB*	Flagellar biosynthetic protein FlhB
AB1936	1949039	1949785	249	-	*fliR*	Flagellar biosynthetic protein FliR
AB1937	1949788	1951722	645	-		Hypothetical membrane protein
AB1938	1951738	1952199	154	-	*flgC*	Flagellar basal body rod protein FlgC
AB1939	1952217	1952534	106	-	*fliE*	Flagellar hook-basal body protein FliE
AB1940	1952550	1952930	127	-		Hypothetical protein
AB1941	1952997	1953818	274	-		ATP-binding protein
AB1942	1953824	1954972	383	-	*flhF*	Flagellar biosynthesis (GTP-binding) protein FlhF
AB1943	1954972	1955364	131	-		Hypothetical protein
AB1944	1955351	1955725	125	-		Hypothetical protein
AB1945	1955744	1956043	100	-		Hypothetical protein
AB1946	1956048	1956914	289	-	*fliY*	Flagellar motor switch protein FliY
AB1947	1956935	1957243	103	-	*flaG*	Polar flagellin
AB1948	1957294	1959381	696	-		TPR repeat protein

AB1949	1959347	1959670	108	-		Hypothetical protein
AB1950	1959727	1961895	723	-	*flgE1*	Flagellar hook protein FlgE
AB1951	1961992	1963740	583	-	*flgE2*	Flagellar hook protein FlgE
AB1952	1963763	1964440	226	-	*flgD*	Flagellar hook assembly protein FlgD
AB1953	1964455	1964739	95	-	*fliN*	Flagellar motor switch protein FliN
AB1954	1964792	1965514	241	-	*fliH*	Flagellar assembly protein FliH
AB1955	1965520	1966527	336	-	*fliG*	Flagellar motor switch protein FliG
AB1956	1966543	1968264	574	-	*fliF*	Flagellar M-ring protein FliF
AB1957	1968285	1968680	132	-	*flgB*	Flagellar basal body rod protein FlgB
AB1958	1968683	1969399	239	-	*flgG1*	Flagellar distal rod protein FlgG
AB1959	1969417	1970352	312	-		Conserved hypothetical protein
AB1960	1970370	1970738	123	-	*cheY2*	Chemotaxis protein CheY
AB1961	1970900	1971685	262	+	*flgG2*	Flagellar distal rod protein FlgG
AB1962	1971708	1972076	123	+		Hypothetical protein
AB1963	1972082	1973017	312	+		Hypothetical protein
AB1964	1973020	1973553	178	-	*rimM*	16S rRNA processing protein
AB1965	1973557	1973793	79	-		Conserved hypothetical protein
AB1966	1973810	1974034	75	-	*rpsP*	30S ribosomal protein S16
AB1967	1974118	1975467	450	-	*ffh*	Signal recognition particle protein
AB1968	1975621	1976373	251	-		Ribosomal large subunit pseudouridine synthase
AB1969	1976367	1977515	383	-	*kdtA*	3-deoxy-D-manno-octulosonic-acid transferase
AB1970	1977521	1978243	241	-		Conserved hypothetical protein (DUF164 domain protein)
AB1971	1978239	1978991	251	-		Conserved hypothetical protein
AB1972	1978997	1979875	293	-	*glyQ*	Glycyl-tRNA synthetase, alpha chain
AB1973	1979875	1980102	76	-		Glutaredoxin-like protein
AB1974	1980105	1980596	164	-	*purE*	Phosphoribosylaminoimidazole carboxylase, catalytic subunit
AB1975	1980640	1981881	414	-		Hypothetical protein
AB1976	1981907	1983193	429	-		Peptidase, U32 family
AB1977	1983307	1985706	800	-		Methyl-accepting chemotaxis protein
AB1978	1985764	1987293	510	-	*recN*	DNA repair protein RecN
AB1979	1987300	1988163	288	-		NAD(+) kinase
AB1980	1988360	1990465	702	-	*fus*	Translational elongation factor G
AB1981	1990478	1990942	155	-	*rpsG*	30S ribosomal protein S7
AB1982	1990962	1991342	127	-	*rpsL*	30S ribosomal protein S12
AB1983	1991564	1992322	253	+		Alpha/beta hydrolase
AB1984	1992399	1993541	381	+	*araJ*	Putative transport protein AraJ
AB1985	1993590	1995650	687	-		TonB-dependent receptor protein

AB1986	1995742	1996707	322	-		Putative sulfonate/nitrate transport system substrate-binding protein
AB1987	1996731	1997963	411	-		Nitrite/nitric oxide reductase-related protein NnrS
AB1988	1997963	1998709	249	-		ABC transporter, ATP-binding protein
AB1989	1998700	1999479	260	-		ABC transporter, permease protein
	**1999728**	**1999847**		**-**	***rrnD***	**5S ribosomal RNA**
	**2000009**	**2002906**		**-**	***rrnD***	**23S ribosomal RNA**
	**2003301**	**2003373**		**-**		**tRNA-Ala-4 (anticodon: TGC)**
	**2003431**	**2003504**		**-**		**tRNA-Ile-4 (anticodon: GAT)**
	**2003613**	**2005124**		**-**	***rrnD***	**16S ribosomal RNA**
AB1990	2005444	2006055	204	-		Conserved hypothetical protein
AB1991	2006055	2007260	402	-		Conserved hypothetical membrane protein
AB1992	2007271	2007837	189	-	*trpG*	Anthranilate synthase, component II
AB1993	2007996	2009000	335	+	*fbpA*	Putative iron-uptake ABC transporter, periplasmic iron-binding protein
AB1994	2008994	2010559	522	+	*fbpB*	Putative iron-uptake ABC transporter, permease protein
AB1996	2010559	2011488	310	+		ABC transporter, ATP-binding protein
AB1997	2011510	2012487	326	+		Transcriptional regulator, AraC family
AB1998	2012593	2014674	694	+		Ferrichrome-iron receptor
AB1999	2014689	2014988	100	+		Conserved hypothetical protein
AB2000	2015001	2016572	524	+		Conserved hypothetical protein
AB2001	2016559	2017059	167	+		Conserved hypothetical protein
AB2002	2017090	2018673	528	-	*nirA*	Ferredoxin-nitrite reductase
AB2003	2018884	2019972	363	+		Conserved hypothetical protein
AB2004	2020004	2020228	75	+		Hypothetical protein
AB2005	2020215	2020616	134	+		Conserved hypothetical protein
AB2006	2020695	2022800	702	+		S1 RNA binding domain protein
AB2007	2022816	2023169	118	-		Conserved hypothetical protein
AB2008	2023182	2023562	127	-		Conserved hypothetical protein
AB2009	2023606	2024376	257	-		MutT/nudix family protein
AB2010	2024392	2025267	292	-		Transcriptional regulator, AraC family
AB2011	2025397	2026506	370	+		Aminotransferase, classes I and II
AB2012	2026524	2027063	180	-		Hypothetical protein
AB2013	2027070	2027669	200	-		Conserved hypothetical integral membrane protein, DedA homolog
AB2014	2027774	2028127	118	-	*rplS*	50S ribosomal protein L19
AB2015	2028186	2028872	229	-	*trmD*	tRNA (guanine-N1)-methyltransferase
AB2016	2028979	2029326	116	+		Hypothetical protein
AB2017	2029494	2031758	755	+	*metE*	5-methyltetrahydropteroyltriglutamate--homocysteine methyltransferase
AB2018	2031867	2033069	401	+	*ilvA*	Threonine deaminase

AB2019	2033163	2033846	228	+	*atpB*	ATP synthase F0 sector, A subunit
AB2020	2033911	2034462	184	+		Thiamine monophosphate synthase
AB2021	2034544	2035842	433	-	*porA*	Major outer membrane protein
AB2022	2036137	2036445	103	-	*folB*	Dihydroneopterin aldolase
AB2023	2036445	2037065	207	-		Conserved hypothetical membrane protein (DUF205 domain protein)
AB2024	2037154	2038140	329	+	*nadA*	Quinolinate synthetase A protein
AB2025	2038140	2038958	273	+	*nadC*	Nicotinate-nucleotide pyrophosphorylase
AB2026	2039015	2039998	328	+		Exopolyphosphatase-related protein
AB2027	2039985	2041367	461	+		Peptidase, M23/M37 family
AB2028	2041374	2042276	301	+	*lpxC*	UDP-3-O-acyl-N-acetylglucosamine deacetylase
AB2029	2042286	2042735	150	+		Conserved hypothetical protein
AB2030	2042753	2043631	293	+	*thrB*	Homoserine kinase
AB2031	2043671	2043955	95	+		Hypothetical protein (DUF448 domain protein)
AB2032	2043933	2046602	890	+	*infB*	Translation initiation factor IF-2
AB2033	2046607	2046963	119	+	*rbfA*	Ribosome binding factor A
AB2034	2046963	2047388	142	+		Conserved hypothetical protein (DUF150 domain protein)
AB2035	2047406	2048407	334	+	*ribD*	Riboflavin biosynthesis protein RibD
AB2036	2048425	2048982	186	-	*efp*	Translation elongation factor EF-P
AB2037	2048999	2050582	528	-	*serA*	D-3-phosphoglycerate dehydrogenase
AB2038	2050596	2052245	550	-	*rpsA*	30S ribosomal protein S1
AB2039	2052315	2053151	279	-	*ispH*	4-hydroxy-3-methylbut-2-enyl diphosphate reductase
AB2040	2053233	2054507	425	-	*aroA*	3-phosphoshikimate 1-carboxyvinyltransferase
AB2041	2054524	2056845	774	-	*pheT*	Phenylalanyl-tRNA synthetase, beta subunit
AB2042	2056845	2057834	330	-	*pheS*	Phenylalanyl-tRNA synthetase, alpha subunit
AB2043	2057950	2058291	114	+		HIT family protein
AB2044	2058308	2059240	311	-	*accA*	Acetyl-CoA carboxylase, carboxyltransferase, alpha subunit
AB2045	2059301	2060557	419	-	*fabF*	Beta ketoacyl-(acyl carrier protein) synthase II
AB2046	2060677	2060904	76	-	*acpP*	Acyl carrier protein, putative
AB2047	2061004	2061744	247	-	*fabG*	3-oxoacyl-(acyl carrier protein) reductase
AB2048	2061805	2062566	254	-		Radical SAM domain protein
AB2049	2062562	2063083	174	-		Conserved hypothetical protein
AB2050	2063083	2063766	228	-	*exsB*	Transcriptional regulator, ExsB family
AB2051	2063878	2064279	134	+		Conserved hypothetical protein
AB2052	2064282	2064950	223	+		Conserved hypothetical protein (DUF558 domain protein)
AB2053	2065015	2066040	342	-	*petC*	Ubiquinol cytochrome c oxidoreductase, cytochrome c1 subunit
AB2054	2066040	2067287	416	-	*petB*	Ubiquinol cytochrome c oxidoreductase, cytochrome b subunit
AB2055	2067301	2067798	166	-	*petA*	Ubiquinol cytochrome c oxidoreductase, 2Fe-2S subunit

AB2056	2067959	2069437	493	-	*thrC*	Threonine synthase
AB2057	2069459	2070361	301	-	*argB*	Acetylglutamate kinase
AB2058	2070447	2073176	910	+		ATP-dependent DNA helicase, UvrD/REP family
AB2059	2073216	2073875	220	+		Hypothetical protein
AB2060	2073957	2075051	365	+	*prfB*	Peptide chain release factor 2
AB2061	2075054	2075431	126	-		Conserved hypothetical protein
AB2062	2075436	2077784	783	-		Conserved hypothetical protein
AB2063	2077777	2078331	185	-		Hypothetical protein
AB2064	2078338	2078964	209	-		Conserved hypothetical protein
AB2065	2078968	2079057	30	-		Hypothetical protein
AB2066	2079071	2079310	80	-		Hypothetical protein
AB2067	2079315	2080202	296	-	*ccoP*	Cytochrome c oxidase, cbb3-type, subunit III
AB2068	2080207	2080428	74	-	*ccoQ*	Cytochrome c oxidase, cbb3-type, subunit IV
AB2069	2080442	2081104	221	-	*ccoO*	Cytochrome c oxidase, cbb3-type, subunit II
AB2070	2081122	2082588	489	-	*ccoN*	Cytochrome c oxidase, cbb3-type, subunit I
AB2071	2082753	2083226	158	-	*smpB*	tmRNA-binding protein SmpB
AB2072	2083219	2083980	254	-	*ispE*	4-diphosphocytidyl-2C-methyl-D-erythritol kinase
AB2073	2083985	2084839	285	-	*truB*	tRNA pseudouridine synthase B
AB2074	2084859	2086910	684	-		ATP-dependent DNA helicase, UvrD/PcrA family
AB2075	2087027	2087920	298	+		Transcriptional regulator, LysR family
AB2076	2087943	2088902	320	-		Conserved hypothetical protein, radical SAM domain protein
AB2077	2088905	2090248	448	-	*purF*	Amidophosphoribosyltransferase
AB2078	2090263	2091033	257	-	*dapB*	Dihydrodipicolinate reductase
AB2079	2091050	2091976	309	-	*trxB*	Thioredoxin reductase
AB2080	2092196	2092510	105	-	*trxA2*	Thioredoxin
AB2081	2092690	2095242	851	+	*alaS*	Alanyl-tRNA synthetase
AB2082	2095276	2095989	238	-		Conserved hypothetical protein (DUF541 domain protein)
AB2083	2096022	2097152	377	-		Major facilitator superfamily transporter
AB2084	2097145	2097705	187	-		Hypothetical protein
AB2085	2097788	2098204	139	+		Conserved hypothetical protein
AB2086	2098211	2099431	407	-		Two-component sensor histidine kinase
AB2087	2099431	2100090	220	-		Two-component response regulator
AB2088	2100121	2101545	475	-	*htrA*	Periplasmic serine protease DO; heat shock protein HtrA
AB2089	2101663	2103348	562	-	*ilvD*	Dihydroxyacid dehydratase
	**2103541**	**2103624**		**+**		**tRNA-Leu-4 (anticodon: TAA)**
	**2103637**	**2103722**		**+**		**tRNA-Ser-2 (anticodon: TGA)**
	**2103791**	**2103863**		**+**		**tRNA-Cys (anticodon: GCA)**

AB2090	2104088	2105317	410	+	*int*	Phage integrase
AB2091	2105752	2106120	123	-		Conserved hypothetical protein
AB2092	2106415	2106576	54	-		Hypothetical protein
AB2093	2106625	2106738	38	-		Hypothetical protein
AB2094	2106741	2107421	227	-		Conserved hypothetical protein
AB2095	2107587	2107907	107	-		Hypothetical protein
AB2096	2107888	2108187	100	-		Hypothetical protein
AB2097	2108190	2108399	70	-		Hypothetical protein
AB2098	2109171	2109422	84	+		Hypothetical protein
AB2099	2109460	2110437	326	-		Conserved hypothetical protein
AB2100	2110421	2111089	223	-		ABC transporter, ATP-binding protein
AB2101	2111089	2112264	392	-		Conserved hypothetical protein
AB2103	2112651	2113019	123	+	*dksA*	DnaK suppressor protein DksA
AB2104	2113019	2114014	332	+		tRNA pseudouridine synthase
AB2105	2114054	2115034	327	-		Conserved hypothetical protein
AB2106	2115047	2115622	192	-		Conserved hypothetical protein
AB2107	2115740	2117047	436	+	*ctpA*	Carboxyl-terminal protease family protein
AB2108	2117062	2118012	317	+	*purC*	Phosphoribosylaminoimidazole-succinocarboxamide synthase
AB2109	2118072	2118314	81	+	*purS*	Phosphoribosylformylglycinamidine synthetase
AB2110	2118319	2118987	223	+	*purQ*	Phosphoribosylformylglycinamidine synthase I
AB2111	2119019	2120086	356	+		Conserved hypothetical protein
AB2112	2120076	2120759	228	+	*plsC*	1-acyl-sn-glycerol-3-phosphate acyltransferase PlsC
AB2113	2120734	2121135	134	+	*crcB*	Camphor resistance CrcB protein
AB2114	2121205	2121426	74	+		Twin-arginine translocation protein, TatA/E family
AB2115	2121450	2121683	78	+		Twin-arginine translocation protein, TatA/E family
AB2116	2121696	2123282	529	+	*argS*	Arginyl-tRNA synthetase
AB2117	2123303	2123626	108	-		Conserved hypothetical protein (DUF143 domain protein)
AB2118	2123626	2124171	182	-	*nadD*	Nicotinate (nicotinamide) nucleotide adenylyltransferase
AB2119	2124262	2125254	331	+	*gapA*	Glyceraldehyde 3-phosphate dehydrogenase A
AB2120	2125275	2126474	400	+	*pgk*	Phosphoglycerate kinase
AB2121	2126477	2127184	236	+	*tpiA*	Triosephosphate isomerase
AB2122	2127189	2128007	273	+	*fabI*	Enoyl-(acyl carrier protein) reductase
AB2123	2128106	2129311	402	+	*lysA*	Diaminopimelate decarboxylase
AB2124	2129326	2130402	359	+	*pheA*	Chorismate mutase\prephenate dehydratase
AB2125	2130417	2131517	367	+	*hisC*	Histidinol-phosphate aminotransferase
AB2126	2131532	2133334	601	+	*dxs*	1-deoxy-D-xylulose-5-phosphate synthase
AB2127	2133488	2134504	339	+		Cytochrome c551 peroxidase

AB2128	2134539	2135081	181	-		Septum formation protein Maf homolog
AB2129	2135164	2136111	316	+	*panE*	Ketopantoate reductase
AB2130	2136111	2137397	429	+		Conserved hypothetical protein
AB2131	2137462	2138046	195	-		Probable lipoprotein nlpC homolog precursor
AB2132	2138124	2138774	217	-		Putative transcriptional regulator, Crp/Fnr family
AB2133	2138858	2139202	115	+		Conserved hypothetical protein
AB2134	2139209	2139853	215	-	*ung*	Uracil-DNA glycosylase
AB2135	2139973	2141361	463	-		Aldehyde dehydrogenase family protein
AB2136	2141385	2143028	548	-	*ilvB*	Acetolactate synthase
AB2137	2143265	2143852	196	+	*lemA*	LemA protein
AB2138	2143855	2144472	206	+		Conserved hypothetical protein
AB2139	2144472	2145368	299	+		Conserved hypothetical membrane protein (DUF477)
AB2140	2145340	2146008	223	+	*gph*	Phosphoglycolate phosphatase
AB2141	2146100	2146534	145	+		DNA-binding ferritin-like protein (Dps/NapA)
AB2142	2146586	2148217	544	-		Conserved hypothetical protein
AB2143	2148221	2148595	125	-		Hypothetical protein
AB2144	2148823	2149911	363	+		Major facilitator superfamily transporter
AB2145	2149925	2150278	118	-		Hypothetical protein
AB2146	2150374	2152116	581	+		GGDEF/HAMP domain protein
AB2147	2152186	2153319	378	+		Multidrug efflux protein, HlyD family
AB2148	2153326	2156448	1041	+		Multidrug efflux protein, AcrB/AcrD/AcrF family
AB2149	2156444	2157841	466	+		RND efflux system, outer membrane lipoprotein
AB2150	2157978	2160305	776	-		TonB-dependent receptor protein
AB2151	2160460	2161413	318	-		Sigma factor regulatory protein, FecR/PupR family
AB2152	2161406	2161882	159	-		Sigma factor, ECF family
AB2153	2162027	2163133	369	-		Hypothetical protein
AB2154	2163136	2164362	409	-		HI0933-like protein
AB2155	2164376	2164690	105	-		Conserved hypothetical protein
AB2156	2164898	2166166	423	+	*metY*	O-acetylhomoserine sulfhydrylase
AB2157	2166176	2166586	137	+	*iscR*	Transcriptional regulator, BadM/Rrf2 family
AB2158	2166620	2167549	310	+	*cysK1*	Cysteine synthase
AB2159	2167586	2167813	76	+		Conserved hypothetical protein
AB2160	2167825	2168526	234	+	*cysH*	Adenosine phosphosulfate (APS) reductase
AB2161	2168546	2169454	303	+	*cysD*	ATP sulfurylase, small subunit
AB2162	2169459	2170865	469	+	*cysN*	ATP sulfurylase, large subunit
AB2163	2170878	2171966	363	+	*cysI*	Sulfite reductase, iron-sulfur subunit
AB2164	2171982	2173277	432	+		Hypothetical protein

AB2165	2173366	2174664	433	+		Aminotransferase, NifS-like protein
AB2166	2174714	2180311	1866	-		Hypothetical protein
AB2167	2180418	2181020	201	-		Hypothetical protein
AB2168	2181023	2181766	248	-	*cobS*	Cobalamin (Vitamin B12) synthase
AB2169	2181766	2182257	164	-	*cobP*	Cobinamide kinase / Cobinamide phosphate guanylyltransferase
AB2170	2182288	2182947	220	-		Lipolytic enzyme, GDSL domain
AB2171	2182952	2184016	355	-		Conserved hypothetical protein
AB2172	2184155	2185558	468	+		Sodium:sulfate symporter family protein
AB2173	2185594	2186337	248	-		Conserved hypothetical protein (DUF81 domain protein)
AB2174	2186384	2188138	585	-	*dsbD*	Thiol:disulfide interchange protein DsbD
AB2175	2188141	2189067	309	-	*rimK*	Ribosomal protein S6 modification protein
AB2176	2189082	2189501	140	-		Conserved hypothetical protein (DUF785 domain protein)
AB2177	2189554	2189775	74	-		Conserved hypothetical protein
AB2178	2189840	2191207	456	-		EAL/GGDEF domain protein
AB2179	2191185	2191985	267	-	*psd*	Phosphatidylserine decarboxylase
AB2180	2192091	2192330	80	+		Hypothetical protein
AB2181	2192347	2193432	362	-	*mltA*	Peptidoglycan N-acetylmuramoylhydrolase
AB2182	2193498	2195381	628	-	*dnaK*	DnaK-type molecular chaperone
AB2183	2195573	2196127	185	-	*grpE*	Heat shock protein GrpE
AB2184	2196140	2196928	263	-	*hrcA*	Putative heat shock regulator
AB2185	2197047	2197685	213	-		Conserved hypothetical protein
AB2186	2197692	2198411	240	-	*truA*	tRNA pseudouridine synthase A
AB2187	2198421	2199434	338	-		Conserved hypothetical membrane protein
AB2188	2199437	2200027	197	-		Peptidase, A24 family
AB2189	2199990	2200691	234	-	*uppS*	Undecaprenyl pyrophosphate synthetase
AB2190	2200681	2201901	407	-	*dfp*	DNA /pantothenate metabolism flavoprotein
AB2191	2201907	2203202	432	-	*glmU*	UDP-N-acetylglucosamine pyrophosphorylase
AB2192	2203208	2205142	645	-		Ankyrin repeat protein
AB2193	2205187	2206818	544	-		Probable ATP-dependent RNA helicase
AB2194	2206842	2207966	375	+	*trmA*	tRNA (uracil-5-)-methyltransferase
AB2195	2207983	2208561	193	-		Hypothetical protein
AB2196	2208692	2209660		-		Hypothetical protein (possible pseudogene)
AB2199	2209879	2211753	625	-	*gidA*	Glucose inhibited division protein A
AB2200	2211898	2212506	203	+	*ribE*	Riboflavin synthase, alpha subunit
AB2201	2212506	2213309	268	-	*mreC*	Rod shape-determining protein MreC
AB2202	2213317	2214357	347	-	*mreB*	Rod shape-determining protein MreB
AB2203	2214364	2215584	407	-	*clpX*	ATP-dependent Clp protease, ATP-binding subunit ClpX

AB2204	2215598	2216377	260	-	*lpxA*	UDP-N-acetylglucosamine acyltransferase
AB2205	2216390	2216830	147	-	*fabZ*	3-hydroxymyristoyl-(acyl carrier protein) dehydratase
AB2206	2216931	2217971	347	-	*lpxB*	Lipid A disaccharide synthase
AB2207	2217971	2219041	357	-		Conserved hypothetical protein (DUF208 domain protein)
AB2208	2219226	2219498	91	+		Hypothetical protein
AB2209	2219513	2220712	400	-	*ndh*	NADH dehydrogenase
AB2210	2220918	2221757	280	+	*nfo*	Endonuclease IV
AB2211	2221778	2223163	462	-		Outer membrane efflux lipoprotein
AB2212	2223159	2226323	1055	-		Multidrug efflux protein, Acr family
AB2213	2226323	2227474	384	-		Multidrug efflux protein, HlyD family
AB2214	2227583	2228119	179	+		Transcriptional regulator, TetR family
AB2215	2228197	2229384	396	+		Major facilitator superfamily transporter, Bcr/CflA subfamily
AB2216	2229395	2231017	541	-		Sulfate permease family protein
AB2217	2231079	2231723	215	-		Hypothetical protein
AB2218	2231727	2232347	207	-		Hypothetical protein
AB2219	2232419	2232814	132	+		Hypothetical protein
AB2220	2232814	2234544	577	+		AAA family ATPase
AB2221	2234557	2235345	263	+		Conserved hypothetical protein
AB2222	2235352	2236218	289	+		Conserved hypothetical protein (DUF191 domain protein)
AB2223	2236218	2236997	260	+	*murB*	UDP-N-acetylenolpyruvoylglucosamine reductase
AB2224	2237216	2239483	756	+	*topA*	DNA topoisomerase I
AB2225	2239486	2239995	170	+		Conserved hypothetical protein
AB2226	2239988	2240833	282	+	*bioB*	Biotin synthetase
AB2227	2240862	2241569	236	-		Conserved hypothetical protein
AB2228	2241569	2241811	81	-		Hypothetical protein
AB2229	2241818	2243095	426	-	*eno*	Enolase
AB2230	2243219	2244265	349	-	*recA*	DNA-dependent ATPase, RecA
AB2231	2244369	2244560	64	-		Hypothetical protein
AB2232	2244685	2245674	330	+	*pseB*	UDP GlcNAc dehydratase/reductase PseB, putative
AB2233	2245674	2246801	376	+		DegT/DnrJ/EryC1/StrS aminotransferase
AB2234	2246797	2247492	232	+	*neuA*	Acylneuraminate cytidylyltransferase
AB2235	2247492	2248514	341	+	*neuB*	N-acetylneuraminic acid synthetase
AB2236	2248514	2249533	340	+		Glycosyltransferase
AB2237	2249523	2250383	287	+		Hypothetical protein
AB2238	2250389	2251051	221	+	*flmE*	Flagellin modification protein FlmE
AB2239	2251036	2252154	373	+		ATP-grasp (A) domain protein
AB2240	2252154	2253023	290	+		Conserved hypothetical protein

AB2241	2253047	2253664	206	+		Conserved hypothetical protein
AB2242	2253682	2255709	676	+		Conserved hypothetical protein (DUF115 domain protein)
AB2243	2255762	2256682	307	-	*flaA*	Flagellin
AB2244	2256845	2257765	307	-	*flaB*	Flagellin
AB2245	2258098	2259477	460	+		4Fe-4S ferredoxin, iron-sulfur binding
AB2246	2259594	2261207	538	+	*pyrG*	CTP synthetase
AB2247	2261210	2262775	522	+	*recJ*	Single-stranded DNA-specific exonuclease
AB2248	2262776	2263375	200	-		Hypothetical protein
AB2249	2263383	2264009	209	-		Conserved hypothetical protein
AB2250	2264130	2264666	179	+	*thiJ*	4-methyl-5(beta-hydroxyethyl)-thiazole monophosphate synthesis protein ThiJ
AB2251	2264743	2268303	1187	+	*dnaE*	DNA polymerase III, alpha subunit
AB2252	2268281	2269066	262	+	*surE*	Stationary-phase survival protein SurE
AB2253	2269087	2269353	89	+		Hypothetical protein
AB2254	2269369	2269629	87	-		Conserved hypothetical protein
AB2255	2269635	2270102	156	-	*moaC*	Molybdenum cofactor biosynthesis protein C
AB2256	2270208	2270426	73	+	*rpsU*	30S ribosomal protein S21
	**2270931**	**2271050**		**-**	***rrnE***	**5S ribosomal RNA**
	**2271212**	**2274109**		**-**	***rrnE***	**23S ribosomal RNA**
	**2274504**	**2274576**		**-**		**tRNA-Ala-5 (anticodon: TGC)**
	**2274634**	**2274707**		**-**		**tRNA-Ile-5 (anticodon: GAT)**
	**2274816**	**2276327**		**-**	***rrnE***	**16S ribosomal RNA**
AB2257	2276717	2277415	233	+		Conserved hypothetical integral membrane protein
AB2258	2277452	2277856	135	+		Conserved hypothetical protein
AB2259	2277814	2278503	230	-		Conserved hypothetical protein, putative methyltransferase
AB2260	2278590	2278934	115	+	*secG*	Protein-export membrane protein SecG
AB2261	2278998	2279552	185	+	*frr*	Ribosome releasing factor
AB2262	2279552	2280163	204	+	*pyrE*	Orotate phosphoribosyltransferase
AB2263	2280166	2280642	159	+		Conserved hypothetical protein, RDD family
AB2264	2280645	2281724	360	+		Putative major facilitator superfamily transporter
AB2265	2281727	2282689	321	-		CorA-like Mg2+ transporter protein
AB2266	2282703	2283437	245	-		Conserved hypothetical protein (DUF328 domain protein)
AB2267	2283524	2283892	123	-		HHH domain protein, putative competence protein ComEA
AB2268	2283962	2284741	260	-		Conserved hypothetical protein
AB2270	2284914	2286029	372	+		Metallophosphoesterase
AB2271	2286091	2286981	297	-		Hypothetical protein
AB2272	2287009	2289111	701	-		TonB-dependent receptor protein
AB2273	2289208	2290167	320	+		Transcriptional regulator, AraC family

AB2274	2290177	2290587	137	-		Heat shock protein Hsp20
AB2275	2290752	2291189	146	-		Conserved hypothetical protein
AB2276	2291289	2293646	786	-		Methyl-accepting chemotaxis protein
AB2277	2293843	2294982	380	-	*moeB*	Molybdopterin biosynthesis protein
AB2278	2295191	2298556	1122	+		Conserved hypothetical protein, transglutaminase-like domain
AB2279	2298560	2301046	829	+		Conserved hypothetical protein (DUF403/404/407 domain protein)
AB2280	2301052	2301936	295	+		Conserved hypothetical protein, transglutaminase-like domain
AB2281	2301955	2302998	348	-	*cysA*	Sulfate ABC transporter (ATP-binding protein)
AB2282	2303017	2303874	286	-	*cysW*	Sulfate transport system permease protein
AB2283	2303874	2304725	284	-	*cysT*	Sulfate transport system permease protein
AB2284	2304920	2305849	310	-	*cysK2*	Cysteine synthase
AB2285	2305868	2307088	407	-		EAL domain protein
AB2286	2307198	2307464	89	-		Conserved hypothetical protein
AB2287	2307499	2308575	359	-	*sbp*	Sulfate-binding protein precursor
AB2288	2308853	2309308	152	-		Transcriptional regulator, BadM/Rrf2 family
AB2289	2309403	2310332	310	-		Conserved hypothetical protein, putative succinylglutamate desuccinylase/aspartoacylase
AB2290	2310322	2311278	319	-		Conserved hypothetical protein, putative succinylglutamate desuccinylase/aspartoacylase
AB2291	2311278	2311943	222	-		Hypothetical protein
AB2292	2311948	2313309	454	-		Conserved hypothetical protein (DUF404/407 domain protein)
AB2293	2313531	2315342	604	-	*typA*	GTP-binding elongation factor family protein
AB2294	2315612	2316805	398	+		Conserved hypothetical protein, putative transport system permease protein
AB2295	2316819	2317055	79	+		Conserved hypothetical protein, SirA-like protein
AB2296	2317083	2317568	162	-		Conserved hypothetical protein
AB2297	2317581	2317883	101	-		Hypothetical protein
AB2298	2317883	2319466	528	-		Conserved hypothetical protein
AB2299	2319519	2321864	782	-		TonB-dependent receptor protein
AB2300	2321966	2322910	315	-		Sigma factor regulatory protein, FecR/PupR family
AB2301	2322903	2323379	159	-		Sigma factor, ECF family
AB2302	2323547	2326912	1122	+		GGDEF/PAS domain protein
AB2303	2326992	2328614	541	+		Sulfatase
AB2304	2328595	2329308	238	+		PAP2 superfamily protein
AB2305	2329407	2329805	133	+		Cytochrome c-type protein, putative
AB2306	2329818	2330321	168	+		Diheme cytochrome c precursor, putative
AB2307	2330334	2331104	257	+		Cytochrome b, putative
AB2308	2331127	2331447	107	+		Hypothetical protein

AB2309	2331410	2331793	128	+		Hypothetical protein
AB2310	2331789	2332451	221	+		Two-component response regulator
AB2311	2332456	2333643	396	+		Two-component sensor histidine kinase
AB2312	2333644	2334390	249	-		Methyltransferase
AB2313	2334457	2335305	283	-		Transcriptional regulator, AraC family
AB2314	2335320	2336894	525	-		HD_GYP domain response regulator
AB2315	2337020	2337844	275	+	*thyX*	Thymidylate synthase ThyX
AB2316	2337847	2338761	305	+	*glsA*	Glutaminase A
AB2317	2338783	2339397	205	+		Conserved hypothetical membrane protein
AB2318	2339459	2339917	153	+		Conserved hypothetical protein
AB2319	2339935	2340510	192	-	*purN*	Phosphoribosylglycinamide formyltransferase
AB2320	2340561	2341022	154	-	*ruvC*	Crossover junction endodeoxyribonuclease RuvC
