# Supplementary material for: The Complete Genome Sequence and Analysis of the Epsilonproteobacterium Arcobacter butzleri
Source: PLoS One. 2007 Dec 26;2(12):e1358. doi: 10.1371/journal.pone.0001358 (PMC2147049; doi:10.1371/journal.pone.0001358)
Supplement: Table S2 — Functional prediction of Arcobacter butzleri genes: similarity to proteins from other epsilonproteobacteria. A. butzleri strain RM4018 genes are sorted by functional category. The % amino acid identity of each protein to homologs in other epsilonproteobacteria is listed. (3.33 MB PDF) [file pone.0001358.s004.pdf]

Table S2. Functional prediction of *Arcobacter butzleri* genes: similarity to proteins from other epsilonproteobacteria.

I. Small molecule metabolism

I.A Degradation

I.A.1 Carbon compounds

			Cj	Cc	Cl	Cu	Cf	Cv	Cn	Ch	Sv	Ni	Sd	Ws	Ha	Hh	Hp
ackA1	AB0494	Acetate kinase	43	45	44	42	43	44	45	44	45	--	39	--	43	52	43
ackA2	AB0496	Acetate kinase	45	47	45	45	43	47	49	42	45	--	45	--	45	51	43
ackA3	AB0498	Acetate kinase	43	46	46	44	45	44	46	41	45	--	45	--	43	51	42
acnD	AB0275	Aconitate hydratase 1	--	--	--	--	--	--	--	--	--	--	--	--	--	--	--
galU	AB0842	UTP--glucose-1-phosphate uridylyltransferase	67	68	66	68	68	68	69	68	76	73	76	76	69	70	69
glcD	AB1851	Glycolate oxidase	65	66	66	65	71	--	--	67	76	68	69	68	60	67	60
prpB	AB0273	Carboxyphosphoenolpyruvate phosphomutase PrpB	--	70	--	--	--	--	--	--	--	--	--	--	--	68	--
prpC	AB0274	2-methylcitrate synthase/citrate synthase 2	32	47	--	32	31	33	33	31	28	--	32	29	27	54	26
prpF	AB0276	Putative AcnD-accessory protein	--	--	--	--	--	--	--	--	--	--	--	--	--	--	--
pta	AB0495	Phosphate acetyltransferase	--	--	--	--	--	--	--	--	--	--	--	--	--	48	--
	AB0749	2-hydroxy-6-oxohepta-2,4-dienoate hydrolase	48	48	46	49	50	53	52	--	51	54	53	--	48	53	47
	AB0843	Phosphohexosemutase	47	46	47	47	52	51	52	50	60	65	60	55	51	55	52
	AB1778	Phosphohexosemutase	44	44	46	41	49	46	46	46	50	50	50	47	46	49	45

I.A.2 Amino acids

			Cj	Cc	Cl	Cu	Cf	Cv	Cn	Ch	Sv	Ni	Sd	Ws	Ha	Hh	Hp
glsA	AB2316	Glutaminase A	--	--	--	--	--	--	--	--	38	--	--	--	--	--	--
	AB1454	Lysine decarboxylase-like protein	25	26	27	--	34	25	--	29	--	--	--	26	--	--	--
	AB1589	Conserved hypothetical protein, putative asparaginase	--	--	--	--	--	--	--	--	44	47	43	41	--	--	--

I.A.5 Miscellaneous

			Cj	Cc	Cl	Cu	Cf	Cv	Cn	Ch	Sv	Ni	Sd	Ws	Ha	Hh	Hp
npd	AB0644	2-nitropropane dioxygenase	65	65	66	65	67	67	65	64	66	73	70	66	68	67	69

I.B Energy metabolism

I.B.1 Glycolysis

			Cj	Cc	Cl	Cu	Cf	Cv	Cn	Ch	Sv	Ni	Sd	Ws	Ha	Hh	Hp
eno	AB2229	Enolase	67	66	67	69	72	71	73	72	78	73	77	73	70	70	70
fba	AB0375	Fructose-bisphosphate aldolase	71	71	70	69	72	71	71	67	71	67	73	--	--	--	--
gapA	AB2119	Glyceraldehyde 3-phosphate dehydrogenase A	58	59	59	60	55	62	62	58	76	50	62	55	50	54	50
gapB	AB0144	Glyceraldehyde-3-phosphate dehydrogenase B	30	28	29	26	31	27	29	29	29	29	29	28	31	28	31
pgi	AB0841	Glucose-6-phosphate isomerase	42	41	42	38	41	44	46	42	52	48	60	41	--	43	--
pgk	AB2120	Phosphoglycerate kinase	67	68	68	64	65	66	65	65	64	66	68	50	49	53	49
pgm	AB1899	Phosphoglycerate mutase, 2,3-bisphosphoglycerate-independent	55	55	56	54	58	58	58	55	64	--	60	60	60	59	60
pyk	AB0255	Pyruvate kinase	54	55	54	54	55	52	50	54	55	44	--	--	--	--	--
tpiA	AB2121	Triosephosphate isomerase	45	43	44	42	44	42	45	44	51	50	56	52	43	45	43

I.B.2 Pyruvate dehydrogenase

			Cj	Cc	Cl	Cu	Cf	Cv	Cn	Ch	Sv	Ni	Sd	Ws	Ha	Hh	Hp
aceE	AB1480	Pyruvate dehydrogenase E1 component	--	--	--	--	--	--	--	--	--	--	--	--	--	--	--
aceF	AB1481	Dihydrolipoamide acetyltransferase	--	--	--	--	--	--	--	--	--	--	--	--	--	--	--
lpdA	AB1482	Dihydrolipoamide dehydrogenase	--	--	--	--	--	26	29	--	30	28	--	--	--	--	--

I.B.3 Tricarboxylic acid cycle

			Cj	Cc	Cl	Cu	Cf	Cv	Cn	Ch	Sv	Ni	Sd	Ws	Ha	Hh	Hp
acnB	AB1447	Aconitate hydratase 2	66	66	--	65	67	62	63	65	76	70	70	65	65	66	65
frdA	AB0297	Fumarate reductase, flavoprotein subunit	73	73	73	73	77	78	76	73	70	37	36	75	65	72	65
frdB	AB0296	Fumarate reductase, iron-sulfur protein	70	70	72	71	73	73	75	71	65	27	28	69	69	68	68
frdC	AB0298	Fumarate reductase, cytochrome *b* subunit	56	54	57	55	57	42	39	40	50	--	--	57	53	52	53
fumA	AB1921	Fumarate hydratase, class I	--	--	--	--	--	--	--	--	--	--	--	--	--	--	--
fumC	AB0722	Fumarate hydratase, class II	66	67	67	66	77	77	39	38	71	--	68	41	64	42	64
gltA	AB0307	Citrate synthase	58	57	--	57	58	59	60	57	64	--	61	58	56	57	55
icd	AB1321	Isocitrate dehydrogenase	54	53	--	53	58	52	49	55	55	54	57	--	--	53	--
mdh	AB1322	Malate dehydrogenase	34	36	38	35	39	38	36	37	49	48	51	47	--	39	--
mqo	AB0513	Malate:quinone oxidoreductase	57	56	57	55	63	56	57	65	61	--	61	--	46	25	45
oorA	AB0853	OorA subunit of 2-oxoglutarate:acceptor oxidoreductase	63	63	65	62	66	62	61	65	69	62	67	65	60	62	61
oorB	AB0854	OorB subunit of 2-oxoglutarate:acceptor oxidoreductase	71	72	72	71	75	67	72	73	73	73	75	72	71	72	69
oorC	AB0855	OorC subunit of 2-oxoglutarate:acceptor oxidoreductase	61	60	60	60	70	67	66	68	74	65	75	67	61	69	63
oorD	AB0852	OorD subunit of 2-oxoglutarate:acceptor oxidoreductase	66	67	70	67	63	66	63	56	63	64	64	65	61	65	60

I.B.5.b Pentose phosphate pathway - nonoxidative branch

			Cj	Cc	Cl	Cu	Cf	Cv	Cn	Ch	Sv	Ni	Sd	Ws	Ha	Hh	Hp
rpiB	AB0636	Ribose 5-phosphate isomerase	45	42	48	42	49	50	50	45	59	59	62	59	51	55	52
tal	AB0219	Transaldolase	45	42	43	41	50	46	48	45	52	50	51	53	47	49	49
tkt	AB0178	Transketolase	61	62	63	60	65	65	64	60	65	63	64	59	57	58	58

I.B.7 Respiration

			Cj	Cc	Cl	Cu	Cf	Cv	Cn	Ch	Sv	Ni	Sd	Ws	Ha	Hh	Hp
hupL	AB1444	Ni/Fe-dependent hydrogenase, large subunit	--	--	--	--	--	--	--	--	40	38	40	--	--	--	--
hupS	AB1445	Ni/Fe-dependent hydrogenase, small subunit	--	--	--	--	--	--	--	--	46	45	47	--	--	--	--
hydA	AB1436	Ni/Fe-hydrogenase, small subunit	77	76	75	72	74	75	74	72	52	53	76	76	66	73	66
hydB	AB1435	Ni/Fe-hydrogenase, large subunit	66	66	67	65	66	66	68	63	51	49	68	69	62	70	62
hydC	AB1434	Ni/Fe hydrogenase, cytochrome *b* subunit	55	55	57	54	57	58	57	57	--	--	50	52	46	44	46
hydD	AB1433	Ni/Fe hydrogenase, expression/formation protein	45	47	46	42	51	47	46	54	29	30	51	52	46	49	46
hypF	AB1431	Transcriptional regulatory protein HypF	40	40	40	38	41	42	42	--	49	44	50	45	36	38	35

I.B.7.a Respiration - aerobic

			Cj	Cc	Cl	Cu	Cf	Cv	Cn	Ch	Sv	Ni	Sd	Ws	Ha	Hh	Hp
hyaA	AB1443	Ni/Fe-hydrogenase, small subunit	52	52	50	50	49	50	50	48	77	76	50	53	48	50	48
hyaB	AB1442	Ni/Fe-hydrogenase, large subunit	50	50	50	50	49	49	50	49	72	74	51	51	52	53	52
hyaC	AB1441	Ni/Fe hydrogenase, cytochrome *b* subunit	--	--	26	--	26	27	--	27	68	68	25	--	--	--	--
hyaD	AB1440	Ni/Fe hydrogenase, expression/formation protein	--	27	27	26	28	26	26	26	41	62	30	34	31	32	32
ndh	AB2209	NADH dehydrogenase	--	--	25	--	--	--	--	25	--	36	40	--	--	26	--
nuoA	AB0312	NADH-quinone oxidoreductase, A subunit	36	37	30	31	50	41	35	43	38	40	--	40	--	38	--
nuoB	AB0311	NADH-quinone oxidoreductase, B subunit	51	51	53	51	72	50	49	48	53	50	52	55	52	50	51
nuoCD	AB0310	NADH-quinone oxidoreductase, C/D subunit	--	--	--	--	55	--	--	--	--	--	--	--	--	--	--
nuoE	AB0309	NADH-quinone oxidoreductase, E subunit	--	--	--	--	32	--	--	--	--	--	--	--	--	--	--
nuoF	AB0308	NADH-quinone oxidoreductase, F subunit	--	--	--	--	55	--	--	--	--	--	--	--	--	--	--
nuoG	AB0306	NADH-quinone oxidoreductase, G subunit	--	--	--	--	45	--	--	--	--	--	--	--	--	--	--
nuoH	AB0305	NADH-quinone oxidoreductase, H subunit	--	--	--	--	53	--	33	33	--	33	--	--	34	33	34
nuoI	AB0304	NADH-quinone oxidoreductase, I subunit	--	--	--	--	60	--	--	--	--	--	--	--	--	--	--
nuoJ	AB0303	NADH-quinone oxidoreductase, J subunit	31	31	30	27	41	30	30	28	27	30	26	29	29	--	29
nuoK	AB0302	NADH-quinone oxidoreductase, K subunit	30	31	36	33	52	30	32	33	34	32	29	37	42	30	42

nuoL	AB0301	NADH-quinone oxidoreductase, L subunit	40	39	38	39	48	37	37	37	37	37	38	38	37	38	38
nuoM	AB0300	NADH-quinone oxidoreductase, M subunit	32	33	31	31	42	36	35	33	35	35	36	35	37	35	36
nuoN	AB0299	NADH-quinone oxidoreductase, N subunit	26	27	27	25	32	30	29	27	28	28	28	27	26	30	27

**I.B.7.b**	**Respiration - anaerobic**		**Cj**	**Cc**	**Cl**	**Cu**	**Cf**	**Cv**	**Cn**	**Ch**	**Sv**	**Ni**	**Sd**	**Ws**	**Ha**	**Hh**	**Hp**
fdhA1	AB1507	Formate dehydrogenase, large subunit FdhA (SeC containing)	53	52	50	49	49	50	56	51	--	--	79	58	--	55	--
fdhA2	AB1521	Formate dehydrogenase, large subunit FdhA (Cys containing)	--	--	--	--	--	--	--	--	--	--	--	56	--	--	--
fdhB1	AB1506	Formate dehydrogenase, iron-sulfur subunit FdhB	66	68	70	64	72	67	70	75	--	32	87	71	--	70	--
fdhB2	AB1520	Formate dehydrogenase, iron-sulfur subunit FdhB	66	68	70	64	72	67	70	75	--	32	87	71	--	70	--
fdhC	AB1505	Putative FdhC protein	--	--	--	--	--	--	--	--	--	--	68	--	--	--	--
fdhD	AB1504	FdhD/NarQ protein required for formate dehydrogenase activity	38	37	--	36	--	--	36	--	--	--	67	56	--	49	--
glpC	AB1272	Anaerobic glycerol-3-phosphate dehydrogenase, subunit C (glpC)	54	55	56	53	60	--	--	56	65	64	63	60	48	52	48
hypA	AB1408	Hydrogenase expression/formation protein HypA	50	50	49	47	50	54	51	49	57	54	65	49	46	39	46
hypB	AB1416	Hydrogenase expression/formation protein HypB	49	49	52	50	55	53	56	53	75	68	72	51	48	50	48
hypC	AB1415	Hydrogenase expression/formation protein HypC	59	59	62	59	58	55	62	61	55	64	60	61	58	54	57
hypD	AB1414	Hydrogenase expression/formation protein HypD	56	56	56	55	57	55	55	55	69	64	69	59	54	56	53
hypE	AB1409	Hydrogenase expression/formation protein HypE	53	53	52	49	54	52	52	46	70	56	77	54	52	47	51
napA	AB0356	Periplasmic nitrate reductase, large subunit	68	68	70	68	70	71	70	71	78	71	79	72	--	67	--
napB	AB0353	Periplasmic nitrate reductase, small subunit, cytochrome *c* -type protein NapB	39	37	40	35	35	37	36	--	--	37	--	--	--	36	--
napD	AB0350	Putative periplasmic nitrate reductase assembly protein NapD	27	27	27	30	33	26	35	--	52	50	61	44	--	36	--
napF	AB0352	Ferredoxin-type protein NapF	--	--	--	28	41	38	37	35	45	46	57	43	--	--	--
napG	AB0355	Fe-S ferredoxin-type protein NapG	52	51	52	52	55	57	58	57	57	63	66	60	--	53	--
napH	AB0354	Methylamine utilization ferredoxin-type protein NapH	46	48	46	46	47	53	56	50	55	62	67	53	--	47	--
napL	AB0351	Putative periplasmic protein	30	34	30	29	--	27	29	29	39	28	44	33	--	--	--
nirA	AB2002	Ferredoxin-nitrite reductase	--	--	--	--	--	--	--	--	26	27	42	--	--	--	--
norB	AB1360	Nitric oxide reductase, cytochrome b subunit	--	--	--	--	--	30	32	--	--	--	--	--	--	--	--
nrfA	AB0345	Cytochrome *c552* nitrite reductase catalytic subunit NrfA	--	--	--	--	--	--	--	29	--	--	--	57	--	--	--
nrfH	AB0346	Cytochrome *c* nitrite reductase, small subunit NrfH	36	37	32	38	35	27	--	32	--	--	--	54	--	33	--
nrfI	AB0344	Cytochrome *c* biogenesis protein	37	37	39	37	40	40	40	39	36	40	37	--	--	--	39
torC	AB1150	Cytochrome *c* -type protein TorC	--	36	32	--	--	38	37	36	--	--	--	26	--	39	--
	AB0004	Probable thioredoxin reductase	49	50	47	49	51	49	49	50	50	54	46	50	46	49	45
	AB0032	Oxidoreductase, FAD-binding/iron-sulfur cluster-binding protein	40	40	--	39	--	--	--	--	--	--	--	--	42	39	41
	AB0037	Iron-sulfur cluster binding protein, putative	--	40	38	39	35	--	--	--	--	--	--	--	39	38	39
	AB1311	Cytochrome *c* biogenesis protein	--	--	43	--	--	--	38	39	--	--	--	46	40	39	41
	AB1519	Putative FdhC protein	--	--	--	--	--	--	--	--	--	--	68	--	--	--	--
	AB1522	Formate dehydrogenase subunit E, putative	33	38	--	33	41	42	49	41	--	--	70	--	--	--	--
	AB1987	Nitrite/nitric oxide reductase-related protein NnrS	--	--	--	--	--	--	--	--	--	46	50	41	--	--	--

**I.B.7.c**	**Electron transport**		**Cj**	**Cc**	**Cl**	**Cu**	**Cf**	**Cv**	**Cn**	**Ch**	**Sv**	**Ni**	**Sd**	**Ws**	**Ha**	**Hh**	**Hp**
ccoN	AB2070	Cytochrome *c* oxidase, *cbb3* -type, subunit I	79	78	78	78	78	73	77	76	74	77	72	71	65	68	66
ccoO	AB2069	Cytochrome *c* oxidase, *cbb3* -type, subunit II	72	75	74	74	73	70	68	68	75	71	70	73	65	58	65
ccoP	AB2067	Cytochrome *c* oxidase, *cbb3* -type, subunit III	44	44	43	43	42	43	46	39	51	51	45	44	36	40	36
ccoQ	AB2068	Cytochrome *c* oxidase, *cbb3* -type, subunit IV	--	--	39	38	--	34	37	32	35	--	--	38	33	--	32
cydA	AB1096	Cytochrome *bd* oxidase, subunit I	70	69	--	67	--	66	67	65	--	64	--	69	--	65	--
cydB	AB1095	Cytochrome *bd* oxidase, subunit II	62	62	--	64	--	60	61	55	--	53	--	58	--	60	--

fdxA	AB0287	Ferredoxin	70	70	69	41	74	76	65	66	71	74	73	--	40	75	--
fdxB	AB1835	Ferredoxin	63	60	65	63	69	--	--	65	80	78	75	78	73	75	70
fldA	AB1783	Flavodoxin	49	48	48	46	52	49	51	49	--	--	--	43	49	46	50
petA	AB2055	Ubiquinol cytochrome *c* oxidoreductase, 2Fe-2S subunit	59	60	61	65	64	59	61	60	47	56	53	57	51	51	49
petB	AB2054	Ubiquinol cytochrome *c* oxidoreductase, cytochrome b subunit	72	71	72	72	68	65	64	66	66	73	68	71	67	67	67
petC	AB2053	Ubiquinol cytochrome *c* oxidoreductase, cytochrome c1 subunit	39	40	39	43	46	45	45	41	38	40	39	45	40	--	41
	AB0465	Quinohemoprotein amine dehydrogenase, 60 kDa subunit	--	--	--	--	--	--	--	--	--	--	--	--	--	--	--
	AB0466	Quinohemoprotein amine dehydrogenase, putative SAM-radical dependent activating subunit	--	--	--	--	--	--	--	--	--	--	--	--	--	--	--
	AB0467	Quinohemoprotein amine dehydrogenase, 9 kDa subunit	--	--	--	--	--	--	--	--	--	--	--	--	--	--	--
	AB0468	Quinohemoprotein amine dehydrogenase, 40 kDa subunit	--	--	--	--	--	--	--	--	--	--	--	--	--	--	--
	AB0545	Conserved hypothetical protein, possible cytochrome c-553	--	--	40	--	35	42	43	32	46	47	48	44	38	46	43
	AB0885	Cytochrome *c* peroxidase	49	44	46	44	40	37	35	42	52	49	38	41	39	39	40
	AB0901	Cytochrome *c* family protein	32	--	--	--	--	44	--	--	--	--	--	47	--	--	--
	AB1323	Conserved hypothetical protein, putative cytochrome *c*	34	33	33	33	33	33	36	--	50	42	51	38	28	40	34
	AB1511	4Fe-4S ferredoxin, iron-sulfur binding	41	41	41	42	40	43	40	35	--	--	52	39	--	31	--
	AB1749	Conserved hypothetical protein, putative cytochrome	--	--	--	--	--	--	--	--	--	--	--	26	--	--	--
	AB1756	Diheme cytochrome *c* peroxidase	--	--	--	--	--	--	--	--	32	33	--	--	--	--	--
	AB1774	Ferredoxin-like protein	43	44	42	42	48	45	46	45	49	46	45	50	41	46	41
	AB1912	Cytochrome *c*	--	29	--	--	--	25	--	27	--	33	37	--	--	30	--
	AB2127	Cytochrome *c551* peroxidase	64	63	64	63	63	33	--	65	71	57	65	68	60	58	61
	AB2245	4Fe-4S ferredoxin, iron-sulfur binding	49	50	46	50	52	51	49	49	62	60	55	60	44	50	44
	AB2305	Cytochrome *c* -type protein, putative	--	--	--	--	59	--	--	--	--	--	--	--	--	--	--
	AB2306	Diheme cytochrome *c* precursor, putative	--	--	--	--	47	--	--	--	--	--	--	--	--	--	--
	AB2307	Cytochrome *b* , putative	--	--	--	--	--	--	--	--	--	--	--	--	--	--	--

I.B.9 ATP-proton motive force			Cj	Cc	Cl	Cu	Cf	Cv	Cn	Ch	Sv	Ni	Sd	Ws	Ha	Hh	Hp
atpA	AB1607	ATP synthase F1 sector, alpha subunit	77	77	78	77	77	77	77	75	76	77	78	78	71	76	71
atpB	AB2019	ATP synthase F0 sector, A subunit	63	63	58	61	60	60	56	52	60	58	54	59	54	58	54
atpC	AB1603	ATP synthase F1 sector, epsilon subunit	51	53	53	51	56	48	50	47	58	54	54	55	42	44	44
atpD	AB1605	ATP synthase F1 sector, beta subunit	83	82	83	82	82	83	83	83	81	84	82	83	79	82	79
atpE	AB1763	ATP synthase F0 sector, C subunit	73	73	81	71	78	76	75	61	77	75	81	--	65	66	65
atpF	AB1609	ATP synthase F0 sector, subunit B	35	39	35	37	37	34	38	36	35	40	38	44	31	37	30
atpF'	AB1610	ATP synthase F0 sector, B' subunit	36	35	37	34	38	39	38	36	44	39	41	44	26	36	29
atpG	AB1606	ATP synthase F1 sector, gamma subunit	52	52	52	52	54	54	53	53	55	57	53	57	52	53	51
atpH	AB1608	ATP synthase F1 sector, delta subunit	35	39	39	35	42	43	43	40	39	37	38	37	--	32	--

I.C Central intermediary metabolism

I.C.1 General			Cj	Cc	Cl	Cu	Cf	Cv	Cn	Ch	Sv	Ni	Sd	Ws	Ha	Hh	Hp
aldA	AB0376	Aldehyde dehydrogenase	31	31	--	--	33	47	47	48	27	29	26	--	--	--	--
aspA	AB1590	Aspartate ammonia-lyase	61	61	55	55	62	55	55	55	41	--	39	68	62	62	61
cynT1	AB0107	Carbonic anhydrase	37	37	40	38	41	38	37	37	49	50	44	38	34	38	38
cynT2	AB0250	Carbonic anhydrase	42	43	44	42	43	40	40	39	52	55	52	40	35	40	36
gltB	AB1838	Glutamate synthase, large chain	40	40	--	41	59	--	--	--	61	66	64	66	--	--	--
gltD	AB1837	Glutamate synthase, small chain	33	34	--	34	50	--	--	--	--	58	53	58	--	--	--
metF	AB0217	5,10-methylenetetrahydrofolate reductase	--	--	--	--	--	--	--	--	52	61	63	50	--	40	--

metK	AB0445	S-adenosylmethionine synthetase	76	75	76	75	76	77	79	75	54	54	77	42	39	41	39
ppa	AB1587	Inorganic pyrophosphatase, manganese-dependent	--	--	--	--	--	--	--	--	65	--	66	--	--	--	--
suhB	AB0443	Inositol-1-monophosphatase	--	30	--	--	27	25	28	29	29	48	26	--	--	--	--
	AB1836	Inositol monophosphatase family protein	39	40	--	--	--	--	--	--	38	39	--	38	--	40	--

I.C.2 Gluconeogenesis

			Cj	Cc	Cl	Cu	Cf	Cv	Cn	Ch	Sv	Ni	Sd	Ws	Ha	Hh	Hp
fbp	AB1228	Fructose-1,6-biphosphatase	51	51	51	52	57	55	53	57	60	61	63	58	43	55	45
maeA	AB1083	NAD-dependent malic enzyme	56	57	58	55	57	62	62	62	61	61	--	63	--	61	--
pckA	AB1236	Phosphoenolpyruvate carboxykinase	67	67	70	68	70	69	69	68	72	66	68	--	--	--	--
pycB1	AB0172	Pyruvate/oxaloacetate carboxyltransferase	66	66	68	64	68	69	69	68	73	72	68	--	--	--	--
pycB2	AB1235	Pyruvate/oxaloacetate carboxyltransferase	66	66	68	64	68	69	69	68	73	72	68	--	--	--	--

I.C.3 Sugar-nucleotides

			Cj	Cc	Cl	Cu	Cf	Cv	Cn	Ch	Sv	Ni	Sd	Ws	Ha	Hh	Hp
glmM	AB0084	Phosphoglucosamine mutase	64	64	64	63	66	68	68	63	67	68	65	66	61	65	62
glmU	AB2191	UDP-N-acetylglucosamine pyrophosphorylase	55	54	54	54	54	52	53	52	59	61	58	64	49	53	50

I.C.4 Amino-sugars

			Cj	Cc	Cl	Cu	Cf	Cv	Cn	Ch	Sv	Ni	Sd	Ws	Ha	Hh	Hp
glmS	AB0444	Glucosamine-fructose-6-phosphate aminotransferase	61	61	64	63	64	64	65	65	67	68	68	64	53	63	53

I.C.5 Sulfur metabolism

			Cj	Cc	Cl	Cu	Cf	Cv	Cn	Ch	Sv	Ni	Sd	Ws	Ha	Hh	Hp
cysD	AB2161	ATP sulfurylase, small subunit	--	74	--	--	--	--	--	--	78	--	88	37	--	--	--
cysH	AB2160	Adenosine phosphosulfate reductase	--	--	--	--	--	--	--	--	--	--	62	49	--	--	--
cysI	AB2163	Sulfite reductase, iron-sulfur subunit	--	--	--	--	--	--	--	--	--	--	--	--	--	--	--
cysJ	AB0733	Sulfite reductase, flavoprotein component	--	--	--	--	--	28	26	--	--	--	--	--	--	--	--
cysN	AB2162	ATP sulfurylase, large subunit	--	55	--	--	--	--	--	--	59	--	79	33	25	--	--
soxA	AB0568	Sulfur oxidation protein SoxXA, diheme cytochrome c subunit	--	--	--	--	--	--	--	--	--	--	--	--	--	--	--
soxB	AB0570	Sulfur oxidation protein, sulfate thiol esterase	--	--	--	--	--	--	--	--	39	42	39	--	--	--	--
soxC	AB0563	Sulfur oxidation protein SoxCD, sulfur dehydrogenase subunit	--	--	--	--	--	--	--	--	66	--	68	--	--	--	--
soxD	AB0564	Sulfur oxidation protein SoxCD, diheme cytochrome c subunit	--	--	--	--	--	--	--	--	47	--	46	--	--	--	--
soxX	AB0565	Sulfur oxidation protein SoxXA, monoheme cytochrome c subunit	--	--	--	--	--	--	--	--	--	--	--	--	--	--	--
soxY	AB0566	Sulfur oxidation protein SoxYZ, sulfur covalently binding protein	--	--	--	--	--	--	--	--	51	35	53	--	--	--	--
soxZ	AB0567	Sulfur oxidation protein SoxYZ, sulfur compound chelating protein	--	--	--	--	--	--	--	--	45	--	52	--	--	--	--

I.C.6 Nitrogen metabolism

			Cj	Cc	Cl	Cu	Cf	Cv	Cn	Ch	Sv	Ni	Sd	Ws	Ha	Hh	Hp
ureAB	AB0809	Fusion of urease beta and gamma subunits	--	--	--	--	--	--	--	--	--	--	--	--	55	54	53
ureC	AB0810	Urease, alpha subunit	--	--	--	--	--	--	--	--	--	--	--	--	63	65	63
ureD	AB0808	Urease accessory protein UreD	--	--	--	--	--	--	--	--	--	--	--	--	--	--	--
ureE	AB0811	Urease accessory protein	--	--	--	--	--	--	--	--	--	--	--	--	--	--	--
ureF	AB0812	Urease complex component	--	--	--	--	--	--	--	--	--	--	--	--	--	--	--
ureG	AB0813	Urease accessory protein UreG	--	--	--	--	--	--	--	--	--	--	--	--	54	60	55

I.D Amino acid biosynthesis

I.D.1 Glutamate family

			Cj	Cc	Cl	Cu	Cf	Cv	Cn	Ch	Sv	Ni	Sd	Ws	Ha	Hh	Hp
argB	AB2057	Acetylglutamate kinase	46	46	--	46	55	58	--	53	63	65	67	63	--	57	--
argC	AB0989	N-acetyl-gamma-glutamyl-phosphate reductase	26	26	--	--	26	--	--	27	27	28	26	--	--	--	--

argD1	AB0256	N-acetylornithine aminotransferase	37	37	25	34	57	54	--	54	66	63	61	59	27	56	26
argD2	AB1476	N-acetylornithine aminotransferase	37	36	--	35	56	55	--	56	53	54	55	51	--	50	--
argF	AB1250	Ornithine carbamoyltransferase	53	54	25	52	67	66	68	66	74	74	68	70	--	59	25
argG	AB1285	Argininosuccinate synthase	74	74	73	73	75	73	72	70	79	74	75	74	--	73	--
argH	AB0616	Argininosuccinate lyase	54	54	47	55	60	60	60	61	61	67	65	63	--	57	--
argJ	AB1801	Ornithine acetyltransferase / N-acetylglutamate synthase	--	--	--	--	59	57	--	54	52	60	59	55	--	53	--
gdhA	AB0284	NADP-specific glutamate dehydrogenase	--	--	62	--	65	66	64	65	65	--	79	68	66	67	65
glnA	AB1902	Glutamine synthetase	70	71	71	70	77	73	73	74	77	68	77	66	62	65	62
glnB1	AB0195	Nitrogen regulatory protein PII	--	--	--	--	52	--	--	--	71	81	83	74	--	--	--
glnB2	AB1160	Nitrogen regulatory protein PII	--	--	--	--	52	--	--	--	71	73	71	71	--	--	--
glnD	AB0191	Protein-P-II uridylyltransferase	--	--	--	--	30	28	29	--	41	45	39	36	--	--	--
proA	AB1771	Gamma-glutamyl phosphate reductase	44	44	--	--	49	41	--	50	65	62	61	59	--	48	--
proB	AB1617	Glutamate 5-kinase	51	52	--	--	49	--	--	53	55	61	55	56	--	50	--
proC	AB0590	Pyrroline-5-carboxylate reductase	42	42	39	41	42	--	--	40	51	50	50	46	36	34	36

**I.D.2 Aspartate family**			**Cj**	**Cc**	**Cl**	**Cu**	**Cf**	**Cv**	**Cn**	**Ch**	**Sv**	**Ni**	**Sd**	**Ws**	**Ha**	**Hh**	**Hp**
asd	AB1798	Aspartate-semialdehyde dehydrogenase	66	67	68	65	67	69	67	66	77	72	65	67	53	61	52
asnB1	AB0046	Asparagine synthetase (glutamine-hydrolyzing)	--	--	--	--	44	--	30	--	27	33	51	43	--	--	--
asnB2	AB0669	Asparagine synthetase	--	--	--	--	28	--	74	--	--	31	69	31	--	--	--
aspB1	AB0160	Aspartate aminotransferase, aminotransferase, classes I and II	68	68	69	68	68	70	70	68	69	70	67	69	27	64	27
aspB2	AB0229	Aspartate aminotransferase, aminotransferase, classes I and II	27	28	28	28	26	26	27	25	61	67	67	57	49	56	50
aspB3	AB0925	Aspartate aminotransferase, aminotransferase, classes I and II	62	63	56	57	58	58	58	52	65	66	67	58	61	59	60
dapA	AB0872	Dihydrodipicolinate synthase	61	62	62	62	62	62	64	62	65	64	65	61	56	59	57
dapB	AB2078	Dihydrodipicolinate reductase	57	56	57	56	61	60	61	56	63	63	64	60	45	58	44
dapD	AB1399	2,3,4,5-tetrahydropyridine-2-carboxylate N-succinyltransferase DapD	54	55	55	54	52	57	53	50	66	62	62	51	51	49	51
dapE	AB1158	Succinyl-diaminopimelate desuccinylase	54	54	56	55	57	55	54	52	58	60	64	54	48	47	47
dapF	AB0148	Diaminopimelate epimerase	53	53	52	49	53	53	50	49	47	58	54	52	45	41	45
hom	AB0161	Homoserine dehydrogenase	53	56	55	54	60	61	61	60	65	68	69	66	57	55	57
lysA	AB2123	Diaminopimelate decarboxylase	62	62	61	59	63	66	66	62	66	68	71	67	56	62	56
lysC	AB1207	Aspartokinase	65	65	65	64	65	64	65	64	67	70	72	68	60	64	59
metC1	AB1737	Cystathionine gamma-synthase	32	32	31	--	27	32	31	29	29	30	29	30	38	33	40
metC2	AB1738	Cystathionine gamma-synthase	--	--	--	--	--	--	--	--	--	--	--	--	--	--	--
metE	AB2017	5-methyltetrahydropteroyltriglutamate--homocysteine methyltransferase	47	47	--	46	64	66	66	--	66	58	66	52	--	59	--
metH	AB0121	5-methyltetrahydrofolate--homocysteine methyltransferase	--	--	--	--	--	--	--	--	63	62	65	55	--	--	--
metX	AB1382	Homoserine O-acetyltransferase	--	--	--	--	61	59	60	60	59	60	57	57	--	--	--
metY	AB2156	O-acetylhomoserine sulfhydrylase	55	56	28	--	64	60	60	58	62	64	59	57	33	58	34
thrB	AB2030	Homoserine kinase	56	56	56	55	49	53	53	50	61	64	62	57	51	52	52
thrC	AB2056	Threonine synthase	45	47	46	47	48	51	51	49	62	62	63	56	48	47	47

**I.D.3 Serine family**			**Cj**	**Cc**	**Cl**	**Cu**	**Cf**	**Cv**	**Cn**	**Ch**	**Sv**	**Ni**	**Sd**	**Ws**	**Ha**	**Hh**	**Hp**
cysE	AB0966	Serine acetyltransferase	--	58	54	--	56	--	--	59	58	60	57	58	--	58	--
cysK1	AB2158	Cysteine synthase	53	57	55	--	64	51	53	59	66	56	69	68	40	56	41
cysK2	AB2284	Cysteine synthase	53	57	55	--	64	51	53	59	66	56	69	68	40	56	41
glyA1	AB0654	Serine hydroxymethyltransferase	71	71	69	72	75	73	75	72	84	76	84	75	68	71	67

glyA2	AB1468	Serine hydroxymethyltransferase	70	70	68	72	75	73	75	71	84	76	85	74	68	71	67
serA	AB2037	D-3-phosphoglycerate dehydrogenase	60	61	60	58	58	60	62	57	64	65	62	62	52	59	52
serB	AB0218	3-phosphoserine phosphatase	56	55	56	56	52	58	57	55	66	70	72	63	52	--	52
	AB0271	D-isomer specific 2-hydroxyacid dehydrogenase, NAD-binding	53	55	48	51	58	53	55	55	51	59	54	47	44	47	44

**I.D.4 Aromatic amino acid family**			**Cj**	**Cc**	**Cl**	**Cu**	**Cf**	**Cv**	**Cn**	**Ch**	**Sv**	**Ni**	**Sd**	**Ws**	**Ha**	**Hh**	**Hp**
aroA	AB2040	3-phosphoshikimate 1-carboxyvinyltransferase	54	58	55	56	56	58	59	54	28	63	61	60	48	51	49
aroB	AB1132	3-dehydroquinate synthase	53	54	56	50	56	55	56	56	64	62	66	61	55	62	55
aroC	AB1624	Chorismate synthase	59	59	57	57	61	56	57	62	67	67	66	58	61	62	60
aroE	AB1080	Shikimate 5-dehydrogenase	46	47	46	47	45	47	48	45	51	52	54	43	40	44	41
aroK	AB0381	Shikimate kinase	39	40	44	39	56	56	55	59	54	61	60	35	32	35	34
aroQ	AB0490	3-dehydroquinate dehydratase	61	61	63	68	64	61	61	59	62	62	63	67	56	63	56
dhs	AB0220	3-deoxy-D-arabinoheptulosonate 7-phosphate synthase (DAHP synthetase class II)	59	59	61	60	61	61	61	59	70	68	64	66	59	64	60
pheA	AB2124	Chorismate mutase\prephenate dehydratase	50	48	50	48	50	51	48	51	62	61	58	54	--	52	--
trpA	AB1787	Tryptophan synthase, alpha chain	38	39	--	37	33	34	31	35	62	58	58	57	33	35	34
trpB1	AB0701	Tryptophan synthase, beta chain	58	58	56	57	58	58	60	57	74	75	79	74	58	51	59
trpB2	AB1302	Tryptophan synthase, beta chain	49	50	49	52	50	52	52	53	65	49	78	71	47	47	48
trpC	AB0848	Indole-3-glycerol phosphate synthase	64	64	--	64	62	61	62	59	64	63	57	63	--	--	--
trpD	AB1282	Anthranilate phosphoribosyltransferase	--	--	--	--	--	--	--	--	55	56	51	54	34	--	35
trpE	AB0652	Anthranilate synthase, component I	30	--	--	31	--	--	--	--	59	64	59	55	--	33	--
trpF	AB0857	N-(5'phosphoribosyl)anthranilate isomerase	28	31	--	28	31	31	30	28	51	55	51	46	--	--	--
trpG	AB1992	Anthranilate synthase, component II	46	43	--	29	--	--	--	--	70	75	75	69	37	46	37
tyrA	AB1218	Prephenate dehydrogenase	52	51	49	50	52	50	51	51	56	60	54	52	50	46	49

**I.D.5 Histidine family**			**Cj**	**Cc**	**Cl**	**Cu**	**Cf**	**Cv**	**Cn**	**Ch**	**Sv**	**Ni**	**Sd**	**Ws**	**Ha**	**Hh**	**Hp**
hisA	AB0473	Phosphoribosylformimino-5-aminoimidazole carboxamide ribotide isomerase	35	35	34	38	66	62	64	67	79	77	73	67	--	57	--
hisB	AB0980	Imidazoleglycerol-phosphate dehydratase	--	--	--	--	65	62	63	64	67	61	62	59	--	58	--
hisC	AB2125	Histidinol-phosphate aminotransferase	52	53	55	53	60	60	60	56	54	59	60	63	--	58	--
hisD	AB0382	Histidinol dehydrogenase	41	42	41	39	65	61	59	64	73	74	71	67	--	62	--
hisF1	AB0055	Imidazoleglycerol phosphate synthase, cyclase subunit	47	49	46	47	74	68	70	70	76	76	77	73	--	63	--
hisG	AB0319	ATP phosphoribosyltransferase	--	--	--	--	65	63	63	61	65	64	65	68	--	60	--
hisH1	AB0475	Glutamine amidotransferase HisH	44	43	42	42	57	58	56	53	61	64	65	59	--	47	--
hisIE	AB0125	Phosphoribosyl-AMP cyclohydrolase/ phosphoribosyl-ATP pyrophosphohydrolase	43	41	37	37	48	53	54	50	63	61	68	59	--	53	--
hisJ	AB1903	Histidinol-phosphate phosphatase	--	--	--	--	53	56	54	58	61	61	56	47	--	45	--

**I.D.6 Pyruvate family**			**Cj**	**Cc**	**Cl**	**Cu**	**Cf**	**Cv**	**Cn**	**Ch**	**Sv**	**Ni**	**Sd**	**Ws**	**Ha**	**Hh**	**Hp**
alr	AB0789	Alanine racemase	43	40	42	39	44	44	44	45	49	47	49	43	26	40	28

**I.D.7 Branched chain family**			**Cj**	**Cc**	**Cl**	**Cu**	**Cf**	**Cv**	**Cn**	**Ch**	**Sv**	**Ni**	**Sd**	**Ws**	**Ha**	**Hh**	**Hp**
ilvA	AB2018	Threonine deaminase	56	59	54	--	58	55	55	--	60	63	56	58	--	57	--
ilvB	AB2136	Acetolactate synthase	28	28	29	--	32	32	32	32	31	30	30	32	--	30	--
ilvC	AB0234	Ketol-acid reductoisomerase	70	70	69	66	74	76	75	73	81	76	81	73	49	74	48
ilvD	AB2089	Dihydroxyacid dehydratase	72	72	70	--	70	74	75	69	83	77	72	72	30	69	31

ilvE	AB0122	Branched-chain amino-acid aminotransferase	64	64	65	62	59	62	62	61	62	68	71	67	33	62	34
ilvH	AB1165	Acetolactate synthase, small subunit	52	54	54	--	59	54	50	52	54	57	55	52	--	52	--
ilvI	AB1164	Acetolactate synthase, large subunit	58	59	58	--	61	62	64	58	67	67	66	64	--	64	--
leuA1	AB0456	2-isopropylmalate synthase	49	48	--	--	68	66	65	66	69	69	25	66	--	26	--
leuA2	AB1579	2-isopropylmalate synthase	--	--	--	--	--	--	--	--	--	--	50	--	--	50	--
leuB	AB1016	3-isopropylmalate dehydrogenase	51	52	--	--	69	69	69	71	72	71	70	72	25	49	27
leuC	AB0082	3-isopropylmalate dehydratase, large subunit	34	34	--	--	75	73	75	73	78	78	80	76	--	52	--
leuD	AB1015	3-isopropylmalate dehydratase, small subunit	--	--	--	--	65	69	71	68	74	75	75	63	--	52	--

I.E Polyamine biosynthesis

			Cj	Cc	Cl	Cu	Cf	Cv	Cn	Ch	Sv	Ni	Sd	Ws	Ha	Hh	Hp
nspC	AB1719	Carboxynorspermidine decarboxylase	50	47	49	49	53	56	53	50	60	59	63	56	48	49	47
speA	AB0967	Arginine decarboxylase	60	60	60	60	60	58	58	59	59	63	58	61	59	59	59
speB	AB1578	Arginase/agmatinase/formiminoglutamate hydrolase, arginase family	--	--	--	--	--	--	--	--	--	--	--	--	--	--	--
speE	AB0146	Spermidine synthase	--	--	--	--	--	--	--	--	--	--	40	--	--	29	--

I.F Purines, pyrimidines, nucleosides and nucleotides

I.F.1 Purine ribonucleotide biosynthesis

			Cj	Cc	Cl	Cu	Cf	Cv	Cn	Ch	Sv	Ni	Sd	Ws	Ha	Hh	Hp
adk1	AB0782	Adenylate kinase	28	28	27	28	27	26	27	27	30	31	51	27	30	29	30
adk2	AB0783	Adenylate kinase	62	63	64	62	59	58	58	59	59	61	66	64	57	63	56
gmk	AB0541	Guanylate kinase	50	51	54	51	49	52	52	49	62	52	54	50	51	50	52
guaA	AB0921	GMP synthase	72	71	70	68	71	72	71	70	77	70	71	74	62	--	62
guaB	AB1026	Inosine-5-monophosphate dehydrogenase	71	70	71	70	69	68	68	68	74	77	75	74	70	71	70
ndk	AB0288	Nucleoside diphosphate kinase	82	82	82	83	84	84	83	79	85	79	85	84	75	82	74
prsA	AB0484	Ribose-phosphate pyrophosphokinase	70	69	71	71	69	68	69	66	74	71	72	72	66	66	65
purA	AB0136	Adenylosuccinate synthetase	68	68	68	67	69	70	70	66	67	66	76	61	53	54	54
purB	AB0019	Adenylosuccinate lyase	70	71	70	69	71	68	70	69	86	77	84	79	66	75	67
purC	AB2108	Phosphoribosylaminoimidazole-succinocarboxamide synthase	--	--	--	--	--	--	--	--	--	--	--	--	--	--	--
purD	AB1187	Phosphoribosylamine-glycine ligase	59	57	58	55	58	59	63	61	68	67	69	55	36	53	36
purE	AB1974	Phosphoribosylaminoimidazole carboxylase, catalytic subunit	71	70	65	64	68	72	70	67	73	75	73	73	--	67	--
purF	AB2077	Amidophosphoribosyltransferase	60	60	62	61	68	67	67	65	67	60	64	61	--	59	--
purH	AB1647	Phosphoribosylaminoimidazolecarboxamide formyltransferase / IMP cyclohydrolase	65	65	66	64	64	68	69	66	73	68	78	66	--	62	--
purL	AB1648	Phosphoribosylformylglycinamidine synthase II	60	61	61	60	63	65	64	64	70	70	72	67	--	61	--
purM	AB0145	Phosphoribosylaminoimidazole synthetase	64	64	64	65	65	66	68	62	72	69	71	62	--	61	--
purN	AB2319	Phosphoribosylglycinamide formyltransferase	27	29	26	26	27	26	29	29	31	32	56	27	--	29	--
purQ	AB2110	Phosphoribosylformylglycinamidine synthase I	59	59	56	57	63	63	64	66	68	65	69	61	--	59	--
purS	AB2109	Phosphoribosylformylglycinamidine synthetase	53	55	55	55	60	57	58	60	73	64	61	51	--	50	--
purT	AB0149	Phosphoribosylglycinamide formyltransferase 2	--	--	--	--	--	--	--	--	72	74	71	64	--	--	--
purU	AB1084	Formyltetrahydrofolate deformylase	56	58	--	59	59	60	--	56	63	58	57	59	50	55	49

I.F.2 Pyrimidine ribonucleotide biosynthesis

			Cj	Cc	Cl	Cu	Cf	Cv	Cn	Ch	Sv	Ni	Sd	Ws	Ha	Hh	Hp
carA	AB0134	Carbamoylphosphate synthase, small subunit	58	61	62	59	62	62	63	63	67	66	68	64	57	60	57
carB	AB1378	Carbamoylphosphate synthase, large subunit	67	66	67	66	68	69	70	68	73	73	74	73	60	67	63
pyrB	AB0258	Aspartate carbamoyltransferase	58	57	60	56	43	42	41	44	56	61	60	58	49	57	49
pyrC	AB0196	Dihydroorotase	58	60	59	57	--	--	--	--	67	65	61	49	46	45	46

pyrD	AB0874	Dihydroorotate dehydrogenase	54	53	57	52	57	53	54	55	60	56	58	56	50	48	50
pyrE	AB2262	Orotate phosphoribosyltransferase	65	64	63	64	62	65	66	64	66	68	72	69	61	61	61
pyrF	AB1859	Orotidine 5'-phosphate decarboxylase	62	61	57	56	60	60	61	59	68	67	65	55	53	49	52
thyX	AB2315	Thymidylate synthase thyX	--	--	--	--	--	--	--	--	--	--	--	--	--	--	--

**I.F.3 2'-deoxyribonucleotide biosynthesis**			**Cj**	**Cc**	**Cl**	**Cu**	**Cf**	**Cv**	**Cn**	**Ch**	**Sv**	**Ni**	**Sd**	**Ws**	**Ha**	**Hh**	**Hp**
dut	AB1299	Deoxyuridinetriphosphatase	--	--	--	--	--	--	--	--	--	--	--	--	--	--	--
nrdA	AB0020	Ribonucleoside-diphosphate reductase, alpha chain	70	69	69	70	69	69	72	68	70	72	71	69	66	67	66
nrdB	AB0021	Ribonucleoside-diphosphate reductase, beta chain	69	69	68	69	70	70	71	67	64	68	64	68	64	65	63
nrdD	AB1249	Anaerobic ribonucleoside-triphosphate reductase	--	--	--	--	70	72	72	68	75	73	79	74	--	--	--
nrdG	AB1247	Anaerobic ribonucleoside-triphosphate reductase activating protein	--	--	--	--	47	46	44	39	47	41	54	38	--	--	--
tmk	AB0969	Thymidylate kinase	46	44	46	43	42	45	47	45	49	51	53	57	54	44	47

**I.F.4 Salvage of nucleosides and nucleotides**			**Cj**	**Cc**	**Cl**	**Cu**	**Cf**	**Cv**	**Cn**	**Ch**	**Sv**	**Ni**	**Sd**	**Ws**	**Ha**	**Hh**	**Hp**
apt	AB0700	Adenine phosphoribosyltransferase	58	53	60	52	59	54	55	58	60	61	58	61	52	50	52
	AB0865	Putative nucleotide phosphoribosyltransferase	52	52	49	49	47	43	44	46	53	41	47	52	41	--	43

**I.F.5 Miscellaneous nucleoside/nucleotide reactions**			**Cj**	**Cc**	**Cl**	**Cu**	**Cf**	**Cv**	**Cn**	**Ch**	**Sv**	**Ni**	**Sd**	**Ws**	**Ha**	**Hh**	**Hp**
dgt	AB1074	Deoxyguanosinetriphosphate triphosphohydrolase	--	--	--	--	--	--	--	--	--	--	27	27	--	--	--
lepA	AB0479	GTP-binding protein LepA	73	73	72	71	74	75	75	71	82	76	73	74	71	70	71
mrp	AB0127	ATP/GTP-binding protein	57	58	58	55	60	58	60	60	64	65	59	60	52	60	52
pyrG	AB2246	CTP synthetase	66	66	67	67	66	68	66	67	66	70	69	70	60	66	61
pyrH	AB0648	Uridylate kinase	71	72	71	71	75	71	69	70	75	71	75	74	70	70	70

I.G Biosynthesis of cofactors, prosthetic groups and carriers

**I.G.1 Biotin**			**Cj**	**Cc**	**Cl**	**Cu**	**Cf**	**Cv**	**Cn**	**Ch**	**Sv**	**Ni**	**Sd**	**Ws**	**Ha**	**Hh**	**Hp**
bioA	AB1645	7,8-diaminopelargonic acid synthetase	33	33	34	32	33	--	--	26	36	36	35	32	33	32	32
bioB	AB2226	Biotin synthetase	56	56	55	57	55	54	54	39	56	64	58	54	46	51	47
bioD	AB1915	Dethiobiotin synthetase	30	30	34	--	32	--	--	--	53	51	47	39	35	--	33
bioF	AB0251	8-amino-7-oxononanoate synthase	33	32	31	30	29	--	--	--	56	53	55	51	47	30	46
birA	AB1613	Biotin--acetyl-CoA-carboxylase ligase	44	41	46	43	46	52	45	48	45	49	51	42	38	42	41
bisC	AB1151	Biotin sulfoxide reductase	--	62	62	--	41	62	60	64	--	--	--	45	--	63	43

**I.G.2 Folic acid**			**Cj**	**Cc**	**Cl**	**Cu**	**Cf**	**Cv**	**Cn**	**Ch**	**Sv**	**Ni**	**Sd**	**Ws**	**Ha**	**Hh**	**Hp**
folB	AB2022	Dihydroneopterin aldolase	42	42	38	43	34	36	34	43	45	41	54	38	42	31	41
folC	AB0402	Folylpolyglutamate synthase/dihydrofolate synthase	41	39	43	40	43	41	38	42	45	40	44	36	41	43	42
folD	AB0638	5,10-methylene-tetrahydrofolate dehydrogenase/5,10-methylene-tetrahydrofolate cyclohydrolase	58	58	61	55	59	62	61	62	65	63	62	62	49	58	49
folE	AB1651	GTP cyclohydrolase I	64	63	66	64	64	68	68	60	68	68	69	58	46	45	48
folK	AB0488	2-amino-4-hydroxy-6- hydroxymethyldihydropteridine pyrophosphokinase	47	45	43	43	48	44	44	45	47	46	50	44	39	37	39
folP	AB1210	Dihydropteroate synthase	48	45	49	46	46	49	47	44	58	57	58	49	41	43	41
pabB	AB0259	Para-aminobenzoate synthase, glutamine amidotransferase component I	--	--	--	--	47	45	--	--	55	54	52	47	--	--	--
	AB1320	4-amino-4-deoxychorismate lyase PabC	49	48	47	--	50	51	49	41	46	45	46	48	43	48	42

I.G.3 Lipoate

			Cj	Cc	Cl	Cu	Cf	Cv	Cn	Ch	Sv	Ni	Sd	Ws	Ha	Hh	Hp
lipA	AB1478	Lipoic acid synthetase	--	--	--	--	--	--	--	--	53	48	--	--	--	--	--

I.G.4 Molybdopterin

			Cj	Cc	Cl	Cu	Cf	Cv	Cn	Ch	Sv	Ni	Sd	Ws	Ha	Hh	Hp
moaA	AB0367	Molybdopterin biosynthesis protein A	61	62	60	59	57	63	64	52	63	68	62	52	--	50	53
moaC	AB2255	Molybdenum cofactor biosynthesis protein C	65	69	65	65	66	71	71	67	70	70	75	70	--	62	66
moaD	AB1905	Molybdopterin converting factor, subunit 1	62	62	56	58	58	56	56	60	52	53	64	62	--	46	49
moaE	AB1904	Molybdopterin converting factor, subunit 2	55	55	51	46	67	58	66	59	59	63	67	56	--	50	47
mobA	AB0081	Molybdopterin-guanine dinucleotide biosynthesis protein	39	40	43	39	35	40	41	37	42	40	41	40	--	34	35
mobB	AB1227	Molybdopterin-guanine dinucleotide biosynthesis protein	54	55	54	50	43	56	58	60	56	60	59	54	--	42	--
moeA1	AB1502	Molybdenum cofactor biosynthesis protein A	39	38	38	37	40	44	44	37	35	35	37	40	--	33	33
moeA2	AB0531	Molybdenum cofactor biosynthesis protein A	43	43	44	41	40	40	42	40	57	52	53	38	--	34	30
mog	AB1137	Molybdenum cofactor biosynthesis protein Mog	65	63	63	62	63	64	64	59	71	--	75	77	--	66	65

I.G.5 Pantothenate

			Cj	Cc	Cl	Cu	Cf	Cv	Cn	Ch	Sv	Ni	Sd	Ws	Ha	Hh	Hp
coaD	AB0970	Phosphopantetheine adenylyltransferase	60	61	60	58	56	60	61	59	64	59	59	56	53	62	53
coaE	AB0147	Dephospho-CoA kinase	43	43	45	40	43	44	51	49	50	60	52	46	48	47	46
panB	AB1789	3-methyl-2-oxobutanoate hydroxymethyltransferase	42	42	--	45	52	54	54	63	60	64	67	55	55	52	54
panC	AB0212	Pantoate--beta-alanine ligase	43	44	--	43	56	54	55	55	59	59	52	53	53	47	52
panD	AB0427	Aspartate 1-decarboxylase	50	50	--	59	67	62	61	66	66	76	72	70	66	--	67
panE	AB2129	Ketopantoate reductase	--	--	--	--	--	--	--	--	--	--	--	--	--	--	--

I.G.6 Pyridoxine

			Cj	Cc	Cl	Cu	Cf	Cv	Cn	Ch	Sv	Ni	Sd	Ws	Ha	Hh	Hp
pdxA	AB0650	Pyridoxal phosphate biosynthetic protein A	47	44	47	48	61	--	--	58	57	61	62	49	45	51	47
pdxH	AB1546	Pyridoxamine 5'-phosphate oxidase	--	--	--	--	--	--	--	--	--	--	--	--	--	--	--
pdxJ	AB0651	Pyridoxal phosphate biosynthesis protein	57	55	58	50	58	--	--	59	66	67	63	59	49	53	49
	AB1474	Pyridoxamine 5'-phosphate oxidase-related, FMN-binding	--	--	--	--	--	--	--	--	--	--	76	69	--	--	--

I.G.7 Pyridine nucleotide

			Cj	Cc	Cl	Cu	Cf	Cv	Cn	Ch	Sv	Ni	Sd	Ws	Ha	Hh	Hp
nadA	AB2024	Quinolinate synthetase A protein	--	--	--	--	55	--	--	--	68	67	72	64	54	56	54
nadB	AB0922	L-aspartate oxidase	28	--	--	26	--	28	27	--	51	54	52	52	--	51	--
nadC	AB2025	Nicotinate-nucleotide pyrophosphorylase	--	--	--	--	36	26	27	--	55	52	58	51	49	50	48
nadD	AB2118	Nicotinate (nicotinamide) nucleotide adenylyltransferase	45	45	43	47	46	--	--	--	40	49	41	--	36	--	37
nadE	AB1256	NH(3)-dependent NAD+ synthetase	48	49	45	48	48	51	48	50	--	--	53	53	49	42	50

I.G.8 Thiamine

			Cj	Cc	Cl	Cu	Cf	Cv	Cn	Ch	Sv	Ni	Sd	Ws	Ha	Hh	Hp
moeB	AB2277	Molybdopterin biosynthesis protein	--	--	--	--	--	--	--	--	--	--	--	--	--	--	--
thiC	AB0128	Thiamine biosynthesis protein ThiC	50	48	50	48	76	74	67	72	--	83	83	78	--	76	--
thiD	AB1770	Phosphomethylpyrimidine kinase	39	39	36	38	36	39	41	36	33	--	--	48	--	--	37
thiE	AB0447	Thiamine-phosphate pyrophosphorylase	37	--	--	35	--	26	--	--	--	42	53	--	--	--	--
thiG	AB0171	Thiazole biosynthesis protein ThiG	47	48	46	47	43	45	45	43	45	78	84	76	--	75	--
thiI	AB1427	Thiamine biosynthesis protein ThiI	--	--	--	--	--	--	--	--	64	--	--	--	--	--	--
thiJ	AB2250	4-methyl-5(beta-hydroxyethyl)-thiazole monophosphate synthesis protein ThiJ	42	43	41	39	51	48	49	49	48	45	56	38	--	45	--

thiL	AB0743	Thiamine monophosphate kinase	55	55	50	54	45	43	45	47	45	52	52	46	--	--	--
	AB0618	MoeB/ThiF family protein	42	43	43	45	45	46	45	38	44	50	--	46	42	42	44
	AB2020	Thiamine monophosphate synthase	--	--	--	--	39	--	--	--	--	40	51	38	--	33	--

**I.G.9 Riboflavin**			**Cj**	**Cc**	**Cl**	**Cu**	**Cf**	**Cv**	**Cn**	**Ch**	**Sv**	**Ni**	**Sd**	**Ws**	**Ha**	**Hh**	**Hp**
ribA	AB1243	GTP cyclohydrolase II	55	56	57	55	58	58	57	58	61	57	57	52	54	50	53
ribAB	AB1237	GTP cyclohydrolase II / 3,4-dihydroxy-2-butanone 4-phosphate synthase	49	48	51	48	52	51	54	48	55	59	61	52	50	47	50
ribB	AB1138	3,4-dihydroxy-2-butanone 4-phosphate synthase	--	--	--	--	--	--	--	--	--	--	--	--	--	--	--
ribD	AB2035	Riboflavin biosynthesis protein RibD	46	43	43	42	43	40	42	43	44	44	48	43	36	35	37
ribE	AB2200	Riboflavin synthase, alpha subunit	54	53	53	52	48	49	50	57	58	61	67	58	49	45	48
ribF	AB1847	Riboflavin kinase/FAD synthase RibF	40	40	43	37	48	49	50	49	49	48	50	44	44	42	43
ribH	AB1857	Riboflavin synthase, beta subunit	74	76	74	72	77	79	78	72	72	76	66	73	70	71	71

**I.G.10 Thioredoxin, glutaredoxin, and glutathione**			**Cj**	**Cc**	**Cl**	**Cu**	**Cf**	**Cv**	**Cn**	**Ch**	**Sv**	**Ni**	**Sd**	**Ws**	**Ha**	**Hh**	**Hp**
ggt	AB0963	Gamma-glutamyltranspeptidase	--	--	--	--	--	--	--	--	--	--	--	--	30	--	30
trxA1	AB1884	Thioredoxin	66	66	68	64	69	67	68	63	58	66	57	66	56	53	58
trxA2	AB2080	Thioredoxin	71	71	70	70	70	76	76	74	69	68	76	80	58	54	62
trxB	AB2079	Thioredoxin reductase	66	65	64	66	64	63	62	65	63	61	68	59	63	61	65

**I.G.11 Menaquinone and ubiquinone**			**Cj**	**Cc**	**Cl**	**Cu**	**Cf**	**Cv**	**Cn**	**Ch**	**Sv**	**Ni**	**Sd**	**Ws**	**Ha**	**Hh**	**Hp**
menG	AB0362	S-adenosylmethionine:2-demethylmenaquinone methyltransferase	--	--	--	--	--	--	--	--	47	--	--	--	--	--	--
ubiA	AB0153	4-hydroxybenzoate octaprenyltranferase	55	54	56	53	57	61	60	55	67	68	69	63	57	55	56
ubiE	AB0422	Ubiquinone\menaquinone biosynthesis methlytransferase	57	55	54	54	54	56	55	53	56	55	59	58	47	48	47
ubiD	AB0971	Phenylacrylic acid decarboxylase	43	43	40	43	45	47	52	52	52	50	54	52	49	49	51

**I.G.12 Heme and porphyrin**			**Cj**	**Cc**	**Cl**	**Cu**	**Cf**	**Cv**	**Cn**	**Ch**	**Sv**	**Ni**	**Sd**	**Ws**	**Ha**	**Hh**	**Hp**
cysG	AB0363	Uroporphyrin-III C-methyltransferase	--	--	--	--	--	--	--	--	70	69	71	--	--	--	--
hemA	AB0385	Glutamyl-tRNA reductase	45	42	44	42	45	43	43	43	39	45	45	46	37	41	36
hemB	AB1244	Delta-aminolevulinic acid dehydratase	70	71	69	67	73	75	74	74	71	75	73	74	60	65	60
hemC	AB0280	Porphobilinogen deaminase	55	55	56	53	54	55	54	56	50	58	58	53	50	52	50
hemD	AB1185	Putative uroporphyrinogen III cosynthase HemD	43	42	36	35	41	38	39	40	33	40	38	34	33	31	32
hemE	AB1796	Uroporphyrinogen decarboxylase	62	62	65	61	64	63	64	63	84	68	83	61	59	61	59
hemH	AB0547	Ferrochelatase	46	45	44	43	46	48	49	50	45	50	49	47	43	43	44
hemL	AB1009	Glutamate-1-semialdehyde 2,1-aminomutase	58	58	61	57	60	57	57	61	69	68	68	61	61	60	60
hemN1	AB1205	Oxygen-independent coproporphyrinogen III oxidase	46	42	44	44	49	44	46	49	47	48	52	41	45	41	44
hemN2	AB1251	Oxygen-independent coproporphyrinogen III oxidase	62	62	62	59	62	63	64	60	65	67	64	62	58	56	58

**I.G.13 Cobalamin**			**Cj**	**Cc**	**Cl**	**Cu**	**Cf**	**Cv**	**Cn**	**Ch**	**Sv**	**Ni**	**Sd**	**Ws**	**Ha**	**Hh**	**Hp**
cbiM	AB0935	Cobalamin (Vitamin B12) biosynthesis protein	--	--	--	--	43	41	--	44	--	--	31	45	--	--	--
cobP	AB2169	Cobinamide kinase / Cobinamide phosphate guanylyltransferase	--	--	--	--	--	--	--	--	--	--	38	--	--	--	--
cobS	AB2168	Cobalamin (Vitamin B12) synthase	--	--	--	--	--	--	--	--	--	--	25	--	--	--	--

**I.G.15 Polyisoprenoids**			**Cj**	**Cc**	**Cl**	**Cu**	**Cf**	**Cv**	**Cn**	**Ch**	**Sv**	**Ni**	**Sd**	**Ws**	**Ha**	**Hh**	**Hp**
dxr	AB0164	1-deoxy-D-xylulose 5-phosphate reductoisomerase	49	49	53	47	50	50	51	53	58	54	64	52	--	50	49

dxs	AB2126	1-deoxy-D-xylulose-5-phosphate synthase	61	60	60	60	62	60	60	61	65	65	64	62	58	63	59
ispA	AB0429	Geranyltranstransferase	58	59	56	59	54	51	51	50	59	60	57	55	50	49	50
ispB	AB0384	Octaprenyl-diphosphate synthase	55	55	54	51	56	53	56	54	53	54	52	52	44	50	45
ispDF	AB0130	2-C-methyl-D-erythritol 4-phosphate cytidylyl transferase/synthase	46	47	50	52	53	48	47	46	52	50	53	46	43	45	43
ispE	AB2072	4-diphosphocytidyl-2C-methyl-D-erythritol kinase	37	38	35	39	41	44	43	47	45	46	51	43	39	39	38
ispG	AB0657	4-hydroxy-3-methylbut-2-en-1-yl diphosphate synthase	63	65	64	64	66	67	68	66	77	75	77	69	66	67	66
ispH	AB2039	4-hydroxy-3-methylbut-2-enyl diphosphate reductase	60	61	57	57	61	59	61	58	66	64	59	58	57	57	57
uppS	AB2189	Undecaprenyl pyrophosphate synthetase	45	46	47	47	57	52	56	56	56	61	64	64	53	55	54

I.G.16 Iron-sulfur clusters

			Cj	Cc	Cl	Cu	Cf	Cv	Cn	Ch	Sv	Ni	Sd	Ws	Ha	Hh	Hp
iscR	AB2157	Transcriptional regulator, BadM/Rrf2 family	26	--	--	--	41	--	--	37	--	35	62	44	--	--	--
iscS	AB0612	Cysteine desulfurase/aminotransferase (IscS/NifS)	60	59	60	58	67	64	64	58	62	--	64	67	65	65	65
iscU	AB0613	NifU-like protein	69	70	70	68	69	74	73	71	74	--	72	70	67	70	67
sufB	AB0611	Fe-S assembly ABC transporter, permease protein	--	--	--	--	--	--	--	--	73	72	--	--	--	--	--
sufC	AB0610	Fe-S assembly ABC transporter, ATP-binding protein	27	26	29	25	29	27	27	28	55	53	30	27	26	27	29
sufD	AB0609	Fe-S assembly protein	--	--	--	--	--	--	--	--	27	--	--	--	--	--	--
sufE	AB0607	Putative suf regulatory protein	--	--	--	--	--	--	--	--	35	31	--	--	--	--	--
sufS	AB0608	Selenocysteine lyase/Cysteine desulfurase	26	26	26	27	26	27	27	27	--	--	26	26	--	26	--
	AB0516	Conserved hypothetical NifU-like protein	51	51	47	50	52	50	51	52	49	60	53	45	50	50	49
	AB1503	Cysteine desulfurase, NifS homolog	--	--	--	--	--	--	--	--	--	--	59	27	--	--	--
	AB2165	Aminotransferase, NifS-like protein	49	49	50	49	53	54	52	45	--	--	63	53	43	44	43

I.H Fatty acid biosynthesis

			Cj	Cc	Cl	Cu	Cf	Cv	Cn	Ch	Sv	Ni	Sd	Ws	Ha	Hh	Hp
aas	AB0190	2-acylglycerophosphoethanolamine acyltransferase / acyl-acyl carrier protein synthetase	42	42	41	--	--	42	41	43	44	--	--	--	--	42	--
accA	AB2044	Acetyl-CoA carboxylase, carboxyltransferase, alpha subunit	64	66	65	65	65	66	67	65	65	72	67	67	65	68	66
accB	AB0040	Acetyl-CoA carboxylase, biotin carboxyl carrier protein	49	46	46	46	47	49	49	47	--	55	--	50	41	47	39
accC1	AB0041	Acetyl CoA carboxylase, biotin carboxylase subunit	69	69	68	70	67	66	69	64	--	69	50	69	63	66	64
accC2	AB0605	Acetyl CoA carboxylase, biotin carboxylase subunit	51	51	51	50	49	49	51	47	--	49	78	51	47	52	47
accD	AB0446	Acetyl-CoA carboxylase, carboxyltransferase, beta subunit	68	68	67	68	69	68	73	66	72	74	72	71	63	67	65
acpP	AB2046	Acyl carrier protein, putative	70	70	72	64	66	70	67	66	82	73	80	76	65	72	63
acpS	AB0214	Holo-(acyl-carrier-protein) synthase	54	54	50	52	56	53	54	46	49	53	55	54	50	47	54
cdsA	AB0163	CDP-diglyceride synthetase CdsA	40	41	40	38	41	40	40	40	48	52	50	46	44	47	44
cfa	AB1212	Cyclopropane-fatty-acyl-phospholipid synthase	--	--	--	--	--	--	--	--	--	--	--	--	35	--	36
dgkA	AB1830	Diacylglycerol kinase	46	46	39	44	43	48	38	41	25	43	54	40	37	--	34
fabD	AB1595	Malonyl coenzyme A-(acyl carrier protein) transacylase	56	57	53	51	56	52	55	53	60	54	58	50	45	47	45
fabF	AB2045	Beta ketoacyl-(acyl carrier protein) synthase II	65	65	66	64	65	65	64	61	67	68	68	62	63	65	63
fabG	AB2047	3-oxoacyl-(acyl carrier protein) reductase	67	68	74	70	70	72	73	72	72	74	73	73	69	71	69
fabH	AB0292	3-oxoacyl-(acyl carrier protein) synthase III	60	61	56	58	58	61	60	56	67	65	65	61	59	57	59
fabI	AB2122	Enoyl-(acyl carrier protein) reductase	67	66	27	69	63	64	63	61	81	65	66	68	61	64	62
fabZ	AB2205	3-hydroxymyristoyl-(acyl carrier protein) dehydratase	67	67	68	69	64	66	66	61	66	69	62	59	56	51	56

II. Broad regulatory functions

			Cj	Cc	Cl	Cu	Cf	Cv	Cn	Ch	Sv	Ni	Sd	Ws	Ha	Hh	Hp
cstA	AB0735	Carbon starvation protein A	84	84	82	86	66	--	--	--	--	--	--	--	65	63	65
cyaA	AB0928	Adenylate/guanylate cyclase	--	--	--	--	--	--	--	--	43	--	49	45	--	--	--
era	AB1709	GTP-binding protein Era homolog	50	49	53	48	51	53	51	49	62	62	61	59	47	53	47

fur1	AB0720	Ferric uptake regulation protein	41	40	40	41	38	40	40	42	41	40	44	34	36	33	33
fur2	AB1784	Ferric uptake regulation protein	53	54	50	51	52	52	52	51	53	56	51	41	44	43	42
gppA	AB1834	Guanosine pentaphosphate phosphohydrolase (gppA)	44	44	41	45	39	43	44	41	39	47	43	44	45	46	44
lon	AB0592	ATP-dependent protease La	57	57	56	56	59	60	61	58	58	62	61	61	56	57	55
perR	AB0878	Peroxide stress regulator	34	33	29	42	36	38	38	26	44	40	28	42	--	40	--
spoT	AB0646	ppGpp synthetase/guanosine-3',5'-bis(diphosphate) 3'-pyrophosphohydrolase	46	46	44	45	45	47	46	47	50	50	46	51	41	47	41
	AB0507	Cyclic nucleotide-binding domain protein	--	--	--	--	--	--	--	--	37	37	42	37	--	--	--
	AB1048	Cyclic nucleotide-binding protein	--	--	--	--	--	--	--	--	--	--	--	--	--	--	--
	AB1162	Ppx/GppA phosphatase family protein	31	30	30	30	34	33	36	34	40	36	--	37	--	--	--

II.A Transcriptional regulation

II.A.1 Repressor/activator proteins

			Cj	Cc	Cl	Cu	Cf	Cv	Cn	Ch	Sv	Ni	Sd	Ws	Ha	Hh	Hp
arsR	AB1925	Transcriptional regulator, ArsR family	47	45	52	--	--	25	--	--	62	49	70	47	--	--	--
exsB	AB2050	Transcriptional regulator, ExsB family	61	61	59	59	60	63	60	57	70	68	67	33	37	--	37
	AB0015	Transcriptional regulator, LysR family	--	--	--	--	--	--	--	--	--	--	--	--	--	--	--
	AB0140	Transcriptional regulator, MarR family	--	--	--	--	--	--	--	--	--	--	--	--	--	--	--
	AB0150	Transcriptional regulator, MarR family	--	--	--	--	--	--	--	--	--	--	--	--	--	--	--
	AB0174	Transcriptional regulator, AraC family	--	--	--	--	--	--	--	--	--	--	71	47	--	--	--
	AB0318	Transcriptional activator, putative, Baf family	45	45	42	44	45	46	46	43	34	34	39	40	41	42	42
	AB0332	Transcriptional regulator	--	--	--	--	--	--	--	26	--	--	--	26	--	--	--
	AB0339	Transcriptional regulator, AraC family	--	--	--	--	30	--	25	--	--	--	--	30	--	--	--
	AB0365	Putative transcriptional regulator	26	25	--	--	35	32	29	29	30	30	61	42	--	29	--
	AB0369	Transcriptional regulator, BadM/Rrf2 family	54	53	53	47	53	51	51	51	50	49	51	--	--	--	--
	AB0597	Transcriptional regulator, MarR family	--	--	--	--	--	--	--	--	--	--	--	--	--	--	--
	AB0815	Transcriptional reguator, TetR family	--	--	--	--	--	--	--	--	--	33	--	30	--	--	--
	AB0819	Putative transcriptional regulator (MarR family)	--	--	--	--	--	--	--	--	--	--	45	--	--	--	--
	AB0847	Transcriptional regulator, LysR family	--	25	--	--	--	--	--	--	--	--	--	--	--	--	--
	AB0990	Transcriptional regulator, GntR family	--	--	--	--	--	--	--	--	45	--	--	--	--	--	--
	AB1055	Transcriptional regulator, AraC family	32	33	30	--	--	--	--	--	26	--	--	--	--	--	--
	AB1057	Transcriptional regulator, LysR family	34	33	39	--	38	27	--	--	39	34	--	31	--	--	--
	AB1153	Transcriptional regulator, TetR family	--	--	--	--	--	--	--	--	33	--	--	--	--	--	--
	AB1368	Transcriptional regulator (MerR family), putative heat shock regulator HspR	55	56	54	53	59	57	55	56	57	58	57	55	52	--	--
	AB1446	Transcriptional regulator, TetR family	--	--	--	--	--	--	--	--	56	54	54	--	--	--	--
	AB1492	Conserved hypothetical protein, putative transcriptional regulator	--	--	--	--	--	--	--	--	51	--	--	--	--	--	--
	AB1570	Transcriptional regulator, ThiJ/PfpI family	--	--	--	--	--	--	31	--	--	--	--	--	--	--	--
	AB1580	Transcriptional regulator, AraC family	--	--	--	--	--	--	--	--	--	--	--	27	--	--	--
	AB1581	Transcriptional regulator, PemK family	--	--	--	--	--	--	--	--	--	54	--	--	--	--	--
	AB1583	Transcriptional regulator, AraC family	--	--	--	--	28	--	--	--	--	--	--	27	--	--	--
	AB1754	Transcriptional regulator, Fur family	--	33	--	--	32	37	35	33	28	28	--	31	--	27	--
	AB1869	Transcriptional regulator, ArsR family	--	--	--	--	--	--	--	46	--	--	--	56	--	--	--
	AB1997	Transcriptional regulator, AraC family	--	--	--	--	--	27	27	--	--	--	--	47	--	--	--
	AB2010	Transcriptional regulator, AraC family	--	--	--	--	--	--	--	--	--	--	58	46	--	--	--
	AB2075	Transcriptional regulator, LysR family	--	--	--	--	31	--	--	--	51	52	55	53	--	--	--
	AB2132	Putative transcriptional regulator, Crp/Fnr family	36	34	37	--	41	41	40	38	36	44	37	44	--	28	--
	AB2214	Transcriptional regulator, TetR family	--	--	--	--	--	--	--	--	--	--	--	--	--	--	--

	AB2273	Transcriptional regulator, AraC family	--	--	--	--	30	--	--	--	--	--	--	30	--	--	--
	AB2288	Transcriptional regulator, BadM/Rrf2 family	27	25	28	25	--	--	--	27	30	--	--	--	--	--	--
	AB2313	Transcriptional regulator, AraC family	30	31	30	--	--	--	--	--	28	--	--	--	--	--	--
**II.A.2 Sigma factors (non-ECF family)**			**Cj**	**Cc**	**Cl**	**Cu**	**Cf**	**Cv**	**Cn**	**Ch**	**Sv**	**Ni**	**Sd**	**Ws**	**Ha**	**Hh**	**Hp**
rpoD	AB1014	RNA polymerase sigma 70 factor	67	68	68	68	69	67	69	66	67	69	67	68	63	67	63
**II.A.3 ECF family sigma/anti-sigma factors**			**Cj**	**Cc**	**Cl**	**Cu**	**Cf**	**Cv**	**Cn**	**Ch**	**Sv**	**Ni**	**Sd**	**Ws**	**Ha**	**Hh**	**Hp**
	AB0986	Sigma factor, ECF family	--	--	--	--	--	--	--	--	--	--	--	33	--	--	--
	AB0987	Sigma factor regulatory protein, FecR/PupR family	--	--	--	--	--	--	--	--	--	--	--	30	--	--	--
	AB1044	Sigma factor, ECF family	--	--	--	--	--	--	--	--	--	--	--	40	--	--	--
	AB1045	Sigma factor regulatory protein, FecR/PupR family	--	--	--	--	--	--	--	--	--	--	--	31	--	--	--
	AB1437	Sigma factor regulatory protein, FecR/PupR family	--	--	--	--	--	--	--	--	--	--	--	30	--	--	--
	AB1438	Sigma factor, ECF family	--	--	--	--	--	--	--	--	--	--	--	31	--	--	--
	AB1460	Sigma factor, ECF family	--	--	--	--	--	--	--	--	--	--	--	53	--	--	--
	AB1461	Sigma factor regulatory protein, FecR/PupR family	--	--	--	--	--	--	--	--	--	--	--	42	--	--	--
	AB1576	Sigma factor regulatory protein, FecR/PupR family	--	--	--	--	--	--	--	--	--	--	--	30	--	--	--
	AB1577	Sigma factor, ECF family	--	--	--	--	--	--	--	--	--	--	--	39	--	--	--
	AB2151	Sigma factor regulatory protein, FecR/PupR family	--	--	--	--	--	--	--	--	--	--	--	29	--	--	--
	AB2152	Sigma factor, ECF family	--	--	--	--	--	--	--	--	--	--	--	39	--	--	--
	AB2300	Sigma factor regulatory protein, FecR/PupR family	--	--	--	--	--	--	--	--	--	--	--	36	--	--	--
	AB2301	Sigma factor, ECF family	--	--	--	--	--	--	--	--	--	--	--	37	--	--	--
**II.B Signal transduction**																	
**II.B.1 Two-component systems**			**Cj**	**Cc**	**Cl**	**Cu**	**Cf**	**Cv**	**Cn**	**Ch**	**Sv**	**Ni**	**Sd**	**Ws**	**Ha**	**Hh**	**Hp**
kdpD	AB1298	Two-component regulatory protein sensor kinase KdpD	--	--	--	--	--	--	--	--	26	--	--	26	--	--	--
kdpE	AB1297	Two component system transcriptional regulatory protein	32	33	30	33	31	33	33	30	30	34	75	36	30	29	31
ompR	AB0412	Two-component response regulator	54	54	55	53	56	55	56	55	52	55	52	51	51	51	52
	AB0034	Two-component response regulator	34	35	36	32	31	32	34	32	35	35	36	42	33	33	33
	AB0035	Two-component sensor histidine kinase	--	--	--	--	--	--	--	--	--	--	--	33	--	--	--
	AB0062	Two-component response regulator	47	45	46	45	43	45	44	45	45	43	46	46	43	47	44
	AB0063	Two-component sensor histidine kinase	32	34	32	33	30	30	31	31	37	34	39	29	31	35	35
	AB0095	Two-component response regulator	41	42	38	32	47	27	30	54	50	54	54	53	28	26	29
	AB0096	Two-component sensor histidine kinase	27	28	30	--	--	--	--	35	--	36	38	35	--	--	--
	AB0105	Two-component response regulator	35	33	30	30	38	36	34	35	35	32	35	42	29	27	29
	AB0106	Two-component sensor histidine kinase	--	--	--	--	--	--	--	--	--	--	28	--	--	--	--
	AB0131	Two-component response regulator	40	39	38	37	41	39	39	--	41	41	42	39	34	35	33
	AB0261	Two-component response regulator	30	29	--	--	30	32	30	29	27	27	30	30	--	--	--
	AB0360	Two-component sensor histidine kinase	--	--	--	--	--	33	--	--	--	--	31	--	--	--	--
	AB0361	Two-component response regulator	31	32	28	26	34	31	31	32	36	29	33	36	28	--	29
	AB0372	Two-component sensor histidine kinase	--	--	--	--	41	40	41	42	--	42	40	39	--	--	--
	AB0373	Two-component response regulator	77	78	75	78	79	79	80	81	69	76	77	85	63	74	61
	AB0413	Two-component sensor histidine kinase	33	33	35	33	36	35	35	35	31	31	31	32	31	33	--
	AB0416	Two-component response regulator	29	29	29	--	31	29	29	32	34	28	58	30	26	--	27
	AB0417	Two-component sensor histidine kinase	--	--	--	--	--	--	--	--	--	--	26	--	--	--	--
	AB0418	Two-component sensor histidine kinase	--	--	--	--	--	--	--	--	--	--	--	--	--	--	--

AB0432	Two-component sensor histidine kinase	--	26	--	--	--	--	25	28	--	--	36	32	--	--	--
AB0433	Two-component response regulator	36	36	39	35	33	34	38	34	39	35	58	52	36	31	37
AB0453	Two-component response regulator	--	--	--	--	--	--	--	--	--	--	--	--	--	--	--
AB0454	Two-component response regulator	32	33	33	--	--	34	35	--	--	36	31	--	--	33	--
AB0455	Two-component sensor histidine kinase	--	--	--	--	--	--	--	--	--	--	--	--	--	--	--
AB0510	Two-component response regulator	35	35	36	32	33	37	35	35	48	55	54	51	31	35	30
AB0511	Two-component sensor histidine kinase	--	--	--	--	--	--	--	--	38	41	--	--	--	--	--
AB0630	Two-component response regulator	30	31	32	34	33	30	34	30	33	30	34	29	27	31	28
AB0631	Two-component sensor histidine kinase	--	--	--	--	--	--	--	--	--	--	--	--	--	--	--
AB0724	Two-component sensor histidine kinase	--	--	--	--	--	--	--	--	--	--	--	--	--	--	--
AB0725	Two-component response regulator	26	--	--	28	27	27	25	27	32	28	27	38	--	--	--
AB0795	Two-component response regulator	38	36	35	35	31	38	37	34	36	36	40	36	29	38	29
AB0796	Two component system histidine kinase/response regulator fusion protein	--	--	--	--	--	--	--	--	31	--	29	27	--	--	--
AB0797	Two-component sensor histidine kinase	--	--	--	--	--	--	--	--	--	--	--	--	--	--	--
AB0834	Two-component response regulator	--	--	--	--	--	25	--	--	27	--	45	--	--	26	--
AB0835	Two-component sensor histidine kinase	--	--	--	--	--	--	--	--	--	--	45	--	--	--	--
AB0904	Two-component response regulator	36	39	40	38	31	44	44	43	42	41	44	50	38	41	35
AB0905	Two-component sensor histidine kinase	--	--	26	--	--	36	--	26	32	36	35	40	--	--	--
AB0997	Response regulator receiver:Metal-dependent phosphohydrolase, HD subdomain	--	--	--	--	--	--	--	--	--	--	--	--	--	--	--
AB1029	Two-component sensor histidine kinase	--	--	--	--	--	--	--	--	25	--	--	--	--	--	--
AB1030	Two-component response regulator	27	28	25	25	28	32	27	28	27	29	47	36	28	--	28
AB1039	Two-component sensor histidine kinase	--	--	--	--	--	--	--	--	--	--	49	--	--	--	--
AB1040	Two-component response regulator	33	33	26	27	31	34	33	31	31	27	54	33	25	29	25
AB1090	Two-component response regulator	38	38	39	39	37	37	41	43	48	45	44	42	33	36	33
AB1091	Two-component sensor histidine kinase	--	--	--	--	25	--	--	29	37	43	34	--	--	--	--
AB1115	Two-component response regulator	47	50	50	48	45	46	46	43	59	60	54	43	38	47	40
AB1116	Two-component sensor histidine kinase	34	33	27	33	30	29	28	30	44	48	42	30	--	29	--
AB1124	Two-component sensor histidine kinase	31	--	--	--	33	30	--	33	--	--	33	--	--	--	--
AB1125	Two-component response regulator	32	33	29	26	29	38	35	32	42	33	36	34	29	28	29
AB1129	Two-component response regulator	--	--	--	--	--	28	31	--	--	--	--	--	--	--	--
AB1168	Two-component response regulator	49	49	52	53	56	53	53	53	42	48	52	54	52	52	53
AB1169	Two-component sensor histidine kinase	--	--	--	--	--	--	--	--	--	--	--	--	--	--	--
AB1171	Two-component sensor histidine kinase	--	--	--	--	--	--	--	--	31	34	--	--	--	--	--
AB1172	Two-component response regulator	30	28	26	28	28	29	27	30	38	38	36	31	29	26	27
AB1174	Two-component response regulator	28	29	29	28	30	30	26	27	36	37	29	36	29	26	30
AB1245	Two-component sensor histidine kinase	35	34	--	--	47	47	--	43	--	--	49	--	--	--	--
AB1246	Two-component response regulator	46	45	32	29	45	42	39	43	39	35	51	35	32	31	33
AB1275	Two-component response regulator	--	--	27	--	29	33	27	26	25	27	25	27	--	--	--
AB1366	Two-component sensor histidine kinase	--	--	--	--	--	--	28	--	--	--	30	--	--	--	--
AB1513	Two-component sensor histidine kinase	--	--	--	--	--	--	27	--	--	--	54	38	--	32	--
AB1514	Two-component response regulator	--	--	--	--	28	--	26	--	26	--	68	43	25	41	--
AB1541	Two-component sensor histidine kinase	--	--	--	--	--	--	--	--	--	--	--	--	--	--	--
AB1591	Two-component response regulator	36	37	25	28	35	33	32	37	33	32	39	41	26	27	27
AB1592	Two-component sensor histidine kinase	26	--	--	--	--	--	--	--	--	--	--	--	--	--	--
AB1646	Two-component sensor histidine kinase	--	--	--	--	--	--	--	--	--	--	35	--	--	--	--

		Cj	Cc	Cl	Cu	Cf	Cv	Cn	Ch	Sv	Ni	Sd	Ws	Ha	Hh	Hp
AB1739	DNA-binding response regulator	39	39	39	39	37	37	37	37	34	40	35	40	36	38	35
AB1742	Response regulator receiver:Metal-dependent phosphohydrolase, HD subdomain	--	--	--	--	--	--	--	--	--	--	--	--	--	--	--
AB1744	Two-component sensor histidine kinase	--	--	--	--	--	--	--	--	--	--	27	--	--	--	--
AB1750	Two-component response regulator	28	30	32	27	--	25	--	27	25	29	28	25	--	28	25
AB1775	Two-component sensor histidine kinase	--	--	--	--	--	--	--	--	26	--	--	--	--	29	--
AB1776	Two-component response regulator	35	30	29	29	33	32	33	31	35	30	37	39	--	26	--
AB1877	Two-component response regulator	33	33	25	25	36	34	32	34	37	32	38	42	--	--	--
AB1878	Two-component sensor histidine kinase	--	--	--	--	--	--	--	--	--	--	25	--	--	--	--
AB2086	Two-component sensor histidine kinase	33	33	33	34	34	33	34	35	43	34	26	29	30	29	--
AB2087	Two-component response regulator	54	55	52	55	55	54	54	52	60	50	49	50	47	48	47
AB2310	Two-component response regulator	39	39	41	39	50	40	41	39	43	43	38	39	38	37	38
AB2311	Two-component sensor histidine kinase	31	29	29	30	38	31	30	33	31	32	27	27	27	31	--

II.B.2 Cyclic diguanylic acid proteins

		Cj	Cc	Cl	Cu	Cf	Cv	Cn	Ch	Sv	Ni	Sd	Ws	Ha	Hh	Hp
AB0089	EAL/GGDEF domain protein	--	--	--	--	--	--	--	--	--	--	--	34	--	--	--
AB0348	EAL/GGDEF/PAS domain protein	--	--	--	--	--	--	--	--	--	33	27	35	--	--	--
AB0472	GGDEF domain protein	--	--	--	--	--	--	--	--	--	37	38	36	--	--	--
AB0542	EAL/GGDEF domain protein	--	--	--	--	--	--	--	--	--	30	29	25	--	--	--
AB0615	GGDEF domain protein	--	--	--	--	--	--	--	--	--	27	28	--	--	--	--
AB0658	GGDEF domain protein	--	--	--	--	--	26	26	--	25	--	42	--	--	--	--
AB0742	GGDEF/PAS domain protein	--	--	--	--	--	--	--	--	--	26	--	--	--	--	--
AB0823	GGDEF domain protein	--	--	--	--	--	--	--	--	--	--	--	--	--	--	--
AB0833	EAL domain protein	--	--	--	--	--	--	--	--	--	--	26	--	--	--	--
AB0886	EAL/GGDEF/PAS domain protein	--	--	--	--	--	25	--	--	32	32	--	--	--	--	--
AB0907	GGDEF domain protein	--	--	--	--	--	--	25	--	--	--	28	--	--	--	--
AB1121	GGDEF domain protein	--	--	--	--	--	--	--	--	--	--	26	--	--	--	--
AB1131	EAL/GGDEF/HAMP domain protein	--	--	--	--	--	27	27	--	--	--	--	--	--	--	--
AB1279	EAL/GGDEF domain protein	--	--	--	--	--	--	--	--	--	32	30	34	--	--	--
AB1307	GGDEF domain protein	--	--	--	--	--	--	--	--	--	--	--	--	--	--	--
AB1308	GGDEF domain protein	--	--	--	--	--	--	--	--	--	--	--	--	--	--	--
AB1449	GGDEF/PAS domain protein	--	--	--	--	--	--	--	--	--	--	28	--	--	--	--
AB1463	GGDEF domain protein	--	--	--	--	--	--	--	--	--	26	--	--	--	--	--
AB1545	EAL/PAS/GGDEF domain protein	--	--	--	--	--	--	--	--	--	--	28	39	--	--	--
AB1547	GGDEF/PAS domain protein	--	--	--	--	--	--	--	--	--	--	--	--	--	--	--
AB1562	EAL/GGDEF domain protein	--	--	--	--	--	--	--	--	--	46	58	--	--	--	--
AB2146	GGDEF/HAMP domain protein	--	--	--	--	--	--	--	--	--	--	27	--	--	--	--
AB2178	EAL/GGDEF domain protein	--	--	--	--	--	--	--	--	25	29	32	35	--	--	--
AB2285	EAL domain protein	--	--	--	--	--	--	--	--	--	27	31	--	--	--	--
AB2302	GGDEF/PAS domain protein	--	--	--	--	--	--	--	--	--	--	--	--	--	--	--

II.B.3 Other

		Cj	Cc	Cl	Cu	Cf	Cv	Cn	Ch	Sv	Ni	Sd	Ws	Ha	Hh	Hp
AB2314	HD_GYP domain response regulator	--	--	--	--	--	--	--	--	--	--	--	--	--	--	--

III. Macromolecule metabolism

III.A Synthesis and modification of macromolecules

III.A.2	Ribosomal protein synthesis and modification		Cj	Cc	Cl	Cu	Cf	Cv	Cn	Ch	Sv	Ni	Sd	Ws	Ha	Hh	Hp
prmA	AB0451	Ribosomal protein L11 methyltransferase	43	46	46	43	47	44	46	42	47	47	48	41	--	--	--
rplA	AB1891	50S ribosomal protein L1	69	69	69	67	66	70	68	65	71	70	67	72	66	66	67
rplB	AB0758	50S ribosomal protein L2	74	75	76	75	78	75	74	74	80	81	76	74	73	72	74
rplC	AB0755	50S ribosomal protein L3	66	66	64	68	65	59	60	62	68	65	73	71	62	66	62
rplD	AB0756	50S ribosomal protein L4	63	63	63	63	60	64	62	60	62	60	63	62	54	57	54
rplE	AB0767	50S ribosomal protein L5	72	71	70	69	71	71	72	67	67	69	65	67	64	70	64
rplF	AB0770	50S ribosomal protein L6	61	61	63	63	65	61	58	62	63	61	54	63	54	58	54
rplI	AB0973	50S ribosomal protein L9	55	55	54	51	52	54	54	51	53	61	53	57	53	49	54
rplJ	AB1890	50S ribosomal protein L10	53	54	52	50	53	53	52	52	50	46	56	53	43	52	43
rplK	AB1892	50S ribosomal protein L11	74	74	74	73	74	73	73	71	70	70	72	73	70	69	70
rplL	AB1889	50S ribosomal protein L7/L12	74	78	76	76	77	78	77	74	69	68	80	72	67	69	67
rplM	AB0097	50S ribosomal protein L13	72	72	71	--	69	70	71	65	72	68	68	71	70	70	71
rplN	AB0765	50S ribosomal protein L14	88	88	89	89	87	88	87	84	88	90	87	91	84	86	84
rplO	AB0773	50S ribosomal protein L15	71	70	66	70	71	69	68	66	71	63	63	64	58	58	58
rplP	AB0762	50S ribosomal protein L16	77	77	77	74	77	78	78	75	74	79	75	83	81	78	80
rplQ	AB1024	50S ribosomal protein L17	71	71	72	70	68	70	64	66	66	73	72	70	71	69	70
rplR	AB0771	50S ribosomal protein L18	56	56	58	52	55	59	59	58	58	62	53	67	57	65	55
rplS	AB2014	50S ribosomal protein L19	65	66	66	63	64	61	61	62	64	65	64	65	66	67	65
rplT	AB0077	50S ribosomal protein L20	81	80	81	79	81	81	81	76	78	78	80	79	74	73	72
rplU	AB1620	50S ribosomal protein L21	58	58	57	58	60	58	60	60	60	60	63	54	58	55	58
rplV	AB0760	50S ribosomal protein L22	64	64	63	68	65	65	65	59	70	70	74	67	62	--	65
rplW	AB0757	50S ribosomal protein L23	69	71	69	68	74	71	69	70	73	68	71	73	70	72	70
rplX	AB0766	50S ribosomal protein L24	74	74	75	78	74	69	76	73	71	76	66	69	62	66	60
rplY	AB0639	50S ribosomal protein L25	61	61	62	60	51	61	61	52	49	63	58	57	52	56	51
rpmA	AB1619	50S ribosomal protein L27	85	83	86	85	80	84	81	80	74	84	76	85	84	84	85
rpmB	AB1803	50S ribosomal protein L28	76	76	76	74	74	74	71	56	63	68	77	71	48	60	50
rpmC	AB0763	50S ribosomal protein L29	62	62	62	61	62	64	61	64	73	61	62	63	52	62	53
rpmE	AB0156	50S ribosomal protein L31	71	72	74	69	71	73	73	68	65	66	60	62	55	56	55
rpmF	AB0290	50S ribosomal protein L32	73	75	77	75	81	79	79	75	78	82	82	71	65	76	65
rpmG	AB1895	50S ribosomal protein L33	77	77	77	75	78	83	81	74	50	61	56	75	63	71	65
rpmH	AB1634	50S ribosomal protein L34	93	93	89	91	88	88	88	88	89	89	84	93	89	93	89
rpmI	AB0076	50S ribosomal protein L35	74	74	74	74	67	66	68	73	64	56	68	62	66	66	68
rpmJ	AB1019	50S ribosomal protein L36	81	81	81	81	84	84	84	--	86	81	86	84	86	86	78
rpsA	AB2038	30S ribosomal protein S1	53	55	53	54	56	57	56	55	53	52	51	56	47	49	47
rpsB	AB0537	30S ribosomal protein S2	71	71	73	71	72	69	71	69	75	71	70	76	70	70	69
rpsC	AB0761	30S ribosomal protein S3	73	73	75	72	76	77	77	71	75	77	79	74	72	70	72
rpsD	AB1022	30S ribosomal protein S4	76	75	77	75	79	77	77	77	81	80	79	78	75	77	75
rpsE	AB0772	30S ribosomal protein S5	71	71	71	71	74	73	73	72	78	78	79	77	78	77	79
rpsF	AB0238	30S ribosomal protein S6	53	54	54	50	54	53	53	51	50	52	50	58	57	58	56
rpsG	AB1981	30S ribosomal protein S7	69	69	69	70	69	71	71	69	64	71	72	71	69	73	69
rpsH	AB0769	30S ribosomal protein S8	62	64	66	62	64	61	62	61	63	59	64	63	55	64	56
rpsI	AB0098	30S ribosomal protein S9	67	67	67	72	67	68	67	64	67	63	68	72	69	73	69
rpsJ	AB0754	30S ribosomal protein S10	81	81	79	80	80	80	80	78	79	85	90	86	85	82	85
rpsK	AB1021	30S ribosomal protein S11	80	80	81	77	81	79	78	75	73	78	76	75	69	73	69
rpsL	AB1982	30S ribosomal protein S12	94	95	94	92	94	94	94	94	89	91	90	88	90	86	89
rpsM	AB1020	30S ribosomal protein S13	84	83	84	83	85	84	83	78	80	79	79	77	79	79	78

rpsN	AB0768	30S ribosomal protein S14	82	80	82	80	79	80	82	79	85	85	79	85	83	82	85
rpsO	AB0368	30S ribosomal protein S15	62	63	63	63	61	66	63	58	66	66	61	60	58	60	58
rpsP	AB1966	30S ribosomal protein S16	76	76	72	76	75	72	72	66	75	76	68	67	75	72	76
rpsQ	AB0764	30S ribosomal protein S17	73	72	76	69	69	71	67	63	79	71	76	62	60	56	63
rpsR	AB0240	30S ribosomal protein S18	69	69	71	70	72	70	70	71	66	65	59	66	65	64	68
rpsS	AB0759	30S ribosomal protein S19	80	80	79	79	80	79	80	74	81	84	83	80	80	82	80
rpsT	AB0085	30S ribosomal protein S20	64	65	64	63	64	63	62	63	60	57	59	62	65	60	64
rpsU	AB2256	30S ribosomal protein S21	87	87	87	86	87	89	83	86	69	74	67	80	76	77	77

**III.A.3 Ribosome maturation and modification**			**Cj**	**Cc**	**Cl**	**Cu**	**Cf**	**Cv**	**Cn**	**Ch**	**Sv**	**Ni**	**Sd**	**Ws**	**Ha**	**Hh**	**Hp**
ksgA	AB0054	Dimethyladenosine transferase	50	48	46	45	46	44	48	44	49	49	52	40	39	45	41
obg	AB1618	GTP-binding protein	57	57	58	57	62	61	62	62	57	63	57	61	52	52	54
rimK	AB2175	Ribosomal protein S6 modification protein	--	--	--	--	--	--	--	--	--	--	82	--	--	--	--
rimM	AB1964	16S rRNA processing protein	48	49	47	47	48	43	47	47	45	46	49	--	38	40	38
rluB	AB0051	Ribosomal large subunit pseudouridine synthase B	54	54	54	53	54	55	57	53	52	58	53	53	57	51	56
rluD	AB1006	Ribosomal large subunit pseudouridine synthase D	46	47	47	45	54	51	51	51	56	61	53	52	48	46	48
rsuA	AB1103	Ribosomal small subunit pseudouridine synthase A	--	--	27	--	26	--	--	26	--	--	64	--	--	--	--
	AB0018	Ribosomal large subunit pseudouridine synthase	41	40	40	40	38	37	41	41	40	42	43	39	39	35	36
	AB1968	Ribosomal large subunit pseudouridine synthase	46	49	49	46	53	48	48	48	52	59	52	50	47	47	46

**III.A.5 Aminoacyl tRNA synthetases and tRNA modification**			**Cj**	**Cc**	**Cl**	**Cu**	**Cf**	**Cv**	**Cn**	**Ch**	**Sv**	**Ni**	**Sd**	**Ws**	**Ha**	**Hh**	**Hp**
aat	AB1102	Leucyl/phenylalanyl-tRNA--protein transferase	36	36	40	36	43	--	--	--	44	47	47	43	--	--	--
alaS	AB2081	Alanyl-tRNA synthetase	61	61	60	59	60	61	60	60	62	62	62	61	55	57	53
argS	AB2116	Arginyl-tRNA synthetase	55	55	56	54	56	57	56	57	--	57	58	56	49	52	49
aspS	AB0779	Aspartyl-tRNA synthetase	67	68	68	67	67	66	67	66	77	65	69	66	62	66	62
cysS	AB0242	Cysteinyl-tRNA synthetase	49	51	55	53	59	57	56	57	63	64	58	58	58	54	59
fmt	AB1616	10-formyltetrahydrofolate:L-methionyl-tRNA(fMet) N-formyltransferase	52	52	53	51	47	48	46	50	47	56	53	52	49	41	49
gatA	AB1025	Glutamyl-tRNA(Gln) amidotransferase, subunit A	62	62	60	59	63	65	63	65	63	65	66	63	59	60	59
gatB	AB0030	Glutamyl-tRNA(Gln) amidotransferase, subunit B	68	67	68	67	68	69	69	66	67	72	70	70	63	69	62
gatC	AB0226	Glutamyl-tRNA(Gln) amidotransferase, subunit C	47	53	46	45	54	48	53	45	45	46	46	57	42	40	45
gltX1	AB0060	Glutamyl-tRNA synthetase	58	59	58	57	60	58	58	60	60	59	57	59	54	56	55
gltX2	AB1222	Glutamyl-tRNA synthetase	59	59	57	55	55	56	55	55	49	57	64	51	50	51	50
glyQ	AB1972	Glycyl-tRNA synthetase, alpha chain	71	73	74	72	73	74	74	70	84	82	83	77	69	75	70
glyS	AB1238	Glycyl-tRNA synthetase, beta chain	51	50	51	50	49	51	52	50	53	57	55	51	42	45	43
hisS	AB0968	Histidyl-tRNA synthetase	56	56	55	53	58	59	56	56	62	57	64	26	27	27	28
ileS	AB0514	Isoleucyl-tRNA synthetase	58	58	60	57	61	61	61	59	62	67	63	59	49	55	50
leuS	AB0400	Leucyl-tRNA synthetase	65	65	62	65	64	63	66	65	68	67	65	63	59	63	58
lysS	AB0655	Lysyl-tRNA synthetase	63	62	61	61	59	62	60	60	64	67	79	65	60	60	60
metS	AB1230	Methionyl-tRNA synthetase	54	56	59	54	61	60	61	59	67	67	65	65	52	55	52
miaA	AB0155	tRNA delta(2)-isopentenylpyrophosphate transferase	49	47	44	43	45	46	47	42	48	53	50	47	38	35	38
miaB	AB0245	tRNA-methylthiotransferase	58	57	57	56	57	57	57	55	61	64	60	56	53	53	55
pheS	AB2042	Phenylalanyl-tRNA synthetase, alpha subunit	66	67	67	67	67	67	67	64	71	69	66	65	56	61	58
pheT	AB2041	Phenylalanyl-tRNA synthetase, beta subunit	42	42	44	41	42	41	42	43	44	43	44	43	39	38	39
proS	AB0386	Prolyl-tRNA synthetase	60	61	59	58	58	61	61	61	61	58	67	59	51	54	50
pth	AB0640	Peptidyl-tRNA hydrolase	48	48	49	44	52	52	48	50	56	50	55	51	41	41	41

queA	AB1263	S-adenosylmethionine:tRNA ribosyltransferase-isomerase	57	57	58	56	57	55	53	59	63	63	56	49	49	47	49
queF	AB0005	7-cyano-7-deazaguanine reductase	66	64	65	61	64	--	--	--	80	63	62	59	--	--	--
rbn	AB1852	tRNA-processing ribonuclease BN	32	31	35	34	35	34	37	--	39	37	35	33	30	35	30
selA	AB1533	L-seryl-tRNA selenium transferase	46	47	46	45	50	49	50	51	--	--	63	50	33	47	32
selD	AB1523	Selenide, water dikinase	47	45	46	47	44	49	--	41	37	--	66	49	--	43	--
serS	AB0343	Seryl-tRNA synthetase	58	60	58	57	58	58	60	59	65	63	60	61	56	59	57
tgt	AB0227	Queuine tRNA-ribosyltransferase	70	71	72	69	73	67	68	68	72	70	72	65	59	63	59
thrS	AB0074	Threonyl-tRNA synthetase	68	68	70	66	65	63	65	65	66	68	64	62	61	63	60
tilS	AB0210	tRNA(Ile)-lysidine synthase	47	45	50	41	44	47	46	44	48	51	46	42	41	--	41
trmA	AB2194	tRNA (uracil-5-)-methyltransferase	44	44	48	44	46	44	48	43	49	48	50	44	--	39	--
trmD	AB2015	tRNA (guanine-N1)-methyltransferase	55	58	62	56	57	60	60	57	65	61	66	52	50	52	50
trmE	AB1630	tRNA modification GTPase	50	53	52	50	52	51	51	49	59	61	61	49	43	43	42
trmU1	AB0486	tRNA (5-methylaminomethyl-2-thiouridylate)-methyltransferase	55	55	53	54	47	49	49	44	51	53	55	46	43	45	43
trmU2	AB0487	tRNA (5-methylaminomethyl-2-thiouridylate)-methyltransferase	32	32	32	31	33	35	33	31	33	36	33	33	34	33	34
trpS	AB0324	Tryptophanyl-tRNA synthetase	61	61	60	62	62	62	60	59	63	37	59	37	36	37	38
truA	AB2186	tRNA pseudouridine synthase A	36	37	39	34	36	36	36	34	45	42	46	35	36	40	36
truB	AB2073	tRNA pseudouridine synthase B	54	53	54	50	53	49	49	49	56	51	48	46	--	47	--
truD	AB0744	tRNA pseudouridine synthase D	49	50	48	47	47	46	47	46	51	54	55	52	48	44	47
tyrS	AB0645	Tyrosyl-tRNA synthetase	62	64	64	63	64	62	65	60	64	68	69	65	59	62	60
valS	AB0581	Valyl-tRNA synthetase	63	64	65	64	63	65	62	63	68	67	70	64	58	58	58
yliG	AB0211	MiaB-like tRNA modifying enzyme	55	55	54	53	55	52	56	54	65	60	63	57	52	53	51
	AB0137	Conserved hypothetical protein, putative tRNA synthetase	--	--	--	--	47	47	45	46	44	44	48	43	--	39	--
	AB0158	tRNA methyltransferase, TrmH family	48	48	45	46	45	48	48	48	48	43	45	49	50	47	48
	AB0388	tRNA nucleotidyltransferase/poly(A) polymerase	--	--	--	--	--	--	--	--	--	--	--	--	--	--	--
	AB0450	Probable tRNA-dihydrouridine synthase	52	52	53	52	56	55	53	56	61	66	64	57	52	59	53
	AB1085	RNA methylase, SpoU family	53	53	57	54	52	51	50	50	52	47	55	52	--	49	--
	AB1134	MiaB-like tRNA modifying enzyme	53	54	54	55	54	55	54	49	63	67	61	59	54	57	54
	AB1284	S4 domain protein	59	56	56	53	49	57	57	53	48	56	56	58	45	51	46
	AB2104	tRNA pseudouridine synthase	34	34	35	34	32	33	32	33	39	44	48	37	32	33	33

**III.A.6 Nucleoproteins**			**Cj**	**Cc**	**Cl**	**Cu**	**Cf**	**Cv**	**Cn**	**Ch**	**Sv**	**Ni**	**Sd**	**Ws**	**Ha**	**Hh**	**Hp**
hup	AB1329	DNA-binding protein HU	68	69	66	68	60	69	68	62	52	67	43	69	46	53	48

**III.A.7 DNA replication, restriction/modification, repair, and recombination**			**Cj**	**Cc**	**Cl**	**Cu**	**Cf**	**Cv**	**Cn**	**Ch**	**Sv**	**Ni**	**Sd**	**Ws**	**Ha**	**Hh**	**Hp**
ada	AB1469	O6-methylguanine-DNA methyltransferase	--	--	--	--	--	40	--	--	--	--	88	60	--	--	--
dfp	AB2190	DNA /pantothenate metabolism flavoprotein	44	44	44	44	45	45	46	41	49	50	56	45	42	43	41
dinP	AB1501	DNA polymerase IV	--	--	--	--	34	--	--	--	44	44	42	--	--	--	--
dksA	AB2103	DnaK suppressor protein DksA	39	38	37	39	39	37	39	42	36	50	38	40	--	36	--
dnaA	AB0001	Chromosomal replication initiator protein DnaA	50	49	49	50	52	53	51	46	60	58	51	58	41	46	42
dnaB	AB0659	Replicative DNA helicase	47	46	43	44	48	47	46	44	--	54	57	57	44	46	45
dnaE	AB2251	DNA polymerase III, alpha subunit	60	59	59	57	58	63	62	54	75	67	64	65	56	56	55
dnaG	AB1621	DNA primase	41	39	36	39	41	44	41	39	53	53	52	49	42	51	42
dnaN	AB0002	DNA polymerase III, beta subunit	41	42	41	42	45	46	48	43	48	52	51	51	40	48	40
dnaQ1	AB0499	DNA polymerase III, epsilon subunit	--	--	--	--	--	--	--	--	56	48	54	52	--	--	--
dnaQ2	AB0506	DNA polymerase III, epsilon subunit	--	--	--	--	--	--	--	--	53	56	57	48	--	--	--
dnaQ3	AB0856	DNA polymerase III, epsilon subunit	46	48	43	43	45	43	46	44	42	46	46	--	42	40	41

dnaQ4	AB1264	DNA polymerase III, epsilon subunit	--	--	--	--	--	--	--	--	67	--	53	--	--	--	--
dnaX	AB0282	DNA polymerase III, gamma and tau subunits	46	--	--	--	--	--	--	--	46	--	48	--	--	--	39
gidA	AB2199	Glucose inhibited division protein A	64	63	63	62	64	64	65	59	68	67	64	63	59	58	59
gidB	AB1242	Glucose inhibited division protein B	37	33	35	36	34	35	36	37	48	43	41	42	42	37	42
gph	AB2140	Phosphoglycolate phosphatase	30	30	29	28	31	29	31	30	32	28	30	27	--	--	--
gyrA	AB1799	DNA gyrase, subunit A	66	68	68	68	67	67	68	66	71	71	67	71	64	67	64
gyrB	AB0003	DNA gyrase, subunit B	68	68	67	67	68	69	70	68	69	71	70	70	61	66	61
holB	AB1209	Putative DNA polymerase III delta prime subunit HolB	41	40	37	40	42	36	38	35	35	46	41	41	33	37	36
hsdM	AB1731	Type I restriction-modification system, M subunit, putative	--	--	--	29	--	--	--	--	--	--	--	--	--	--	--
hsdS	AB1730	Type I restriction-modification system specificity determinant	--	--	--	--	--	--	--	--	--	--	--	--	--	--	--
lig	AB1071	DNA ligase	55	53	54	54	56	55	57	54	65	55	59	55	49	55	49
ligA	AB0699	ATP-dependent DNA ligase	38	38	44	38	39	43	39	47	--	--	50	--	--	--	--
mfd	AB0408	Transcription-repair coupling factor	50	50	51	49	52	51	51	53	52	54	50	53	49	51	49
mutS1	AB0459	Mismatch repair ATPase	--	--	--	--	--	--	--	--	--	51	51	--	--	--	--
mutS2	AB1154	Mismatch repair ATPase	51	51	51	51	51	50	52	50	50	56	50	50	42	48	42
ogt	AB1777	Putative methylated-DNA-protein-cysteine methyltransferase	--	42	--	--	--	43	34	42	43	--	48	--	--	34	--
phrB	AB0837	Deoxyribodipyrimidine photolyase	--	--	--	--	--	--	--	--	--	--	47	41	--	--	--
polA	AB1276	DNA polymerase I	49	49	49	48	50	49	49	50	51	54	53	52	41	48	41
priA	AB1403	Primosomal protein N'	46	43	44	45	47	44	44	43	44	47	47	47	42	42	41
radA	AB0882	DNA repair protein RadA	64	65	65	64	64	64	62	60	67	64	67	65	60	64	60
recA	AB2230	DNA-dependent ATPase, RecA	75	76	73	74	74	75	76	76	72	75	76	75	76	73	76
recN	AB1978	DNA repair protein RecN	47	49	46	45	45	47	47	48	41	50	47	43	38	40	37
recR	AB1300	Recombination protein RecR	52	53	49	49	58	51	51	51	58	56	61	49	44	47	45
rep	AB0561	ATP-dependent DNA helicase, UvrD/Rep family	55	55	56	53	54	57	56	51	55	56	60	58	49	55	50
ruvA	AB0747	Holliday junction DNA helicase RuvA	46	47	49	45	43	48	49	46	64	60	57	54	44	47	43
ruvB	AB1790	Holliday junction DNA helicase RuvB	72	72	72	71	69	72	73	69	75	74	74	70	67	70	68
ruvC	AB2320	Crossover junction endodeoxyribonuclease RuvC	74	75	72	69	74	75	73	67	73	67	71	72	66	72	68
ssb	AB0239	Single-strand DNA binding protein	45	43	49	46	47	43	48	42	57	55	53	54	46	48	45
topA	AB2224	DNA topoisomerase I	48	47	49	47	47	47	48	46	46	47	47	45	43	44	43
ung	AB2134	Uracil-DNA glycosylase	49	50	50	50	49	54	53	50	--	--	50	48	40	44	40
uvrA	AB0960	Excinuclease ABC, subunit A	73	73	73	73	74	73	73	72	74	74	75	75	68	72	68
xerD	AB1199	Integrase/recombinase XerD	54	55	57	55	58	57	54	54	--	63	--	65	51	59	50
	AB0008	Conserved hypothetical protein, possible methyltransferase	--	--	--	--	44	39	29	--	--	--	39	41	--	--	--
	AB0157	Conserved hypothetical protein, putative methyltransferase	48	47	45	46	49	44	43	42	50	50	50	50	44	44	45
	AB0177	DNA/RNA helicase (DEAD/DEAH BOX family)	--	--	--	--	--	--	--	--	--	--	--	--	--	--	--
	AB0216	Conserved hypothetical protein, putative Holliday junction resolvase	48	46	45	46	48	50	48	49	66	59	50	48	45	39	44
	AB0320	Conserved hypothetical protein, putative methyltransferase	--	--	--	--	36	--	--	--	38	39	35	--	--	--	--
	AB0329	O-methyltransferase	--	--	--	--	--	--	--	--	--	--	--	--	--	--	--
	AB0403	Probable helicase	--	--	--	--	--	--	--	--	41	--	--	--	--	--	--
	AB0529	DNA recombination protein RmuC homolog	--	--	31	30	30	--	--	--	--	--	42	49	--	37	--
	AB0877	Endonuclease/exonuclease/phosphatase	--	--	--	--	30	28	27	30	36	--	--	--	--	--	--
	AB0892	Putative methyltransferase	47	46	48	43	43	39	43	44	44	48	41	41	40	40	39
	AB0972	Site-specific recombinase, resolvase family	--	31	27	--	--	--	--	--	--	26	--	32	--	--	--
	AB1081	Methyltransferase, putative	--	29	--	--	--	--	--	--	34	40	37	38	--	--	--
	AB1180	Conserved hypothetical protein, putative DNA helicase	--	--	--	37	--	--	--	--	--	--	--	47	--	42	--
	AB1337	DEAD/DEAH box helicase domain protein	--	--	--	--	--	--	--	--	--	--	--	--	--	25	--

	AB1412	McrBC endonuclease McrB, putative	32	--	25	--	--	--	--	--	--	--	--	--	--	--	--
	AB1413	McrBC catalytic subunit McrC, putative	29	--	--	--	--	--	--	--	--	--	--	--	--	--	--
	AB1470	Endonuclease III	33	--	--	--	--	--	--	--	--	--	58	--	--	--	--
	AB1489	DNA (Cytosine-5-)-methyltransferase	--	--	--	40	--	35	29	--	29	--	67	--	31	--	31
	AB1535	Putative DNA helicase	--	--	--	--	--	--	--	--	--	--	--	--	--	--	--
	AB1567	Putative DNA alkylation repair enzyme	--	--	--	--	--	--	--	--	--	--	--	--	--	--	--
	AB1848	Methyltransferase, putative	51	52	48	49	52	49	51	50	57	61	64	56	47	50	46
	AB1880	Replication protein	--	--	--	--	--	--	--	--	--	--	--	--	--	--	--
	AB2058	ATP-dependent DNA helicase, UvrD/REP family	33	33	34	33	34	33	33	34	36	35	37	35	29	31	31
	AB2074	ATP-dependent DNA helicase, UvrD/PcrA family	52	53	53	50	59	61	60	58	59	62	59	59	47	52	47
	AB2259	Conserved hypothetical protein, putative methyltransferase	25	--	28	--	--	--	--	--	40	43	45	32	32	--	32
	AB2312	Methyltransferase	--	--	--	--	--	--	--	--	--	--	--	--	--	--	--

**III.A.8**	**Protein translation and modification**		**Cj**	**Cc**	**Cl**	**Cu**	**Cf**	**Cv**	**Cn**	**Ch**	**Sv**	**Ni**	**Sd**	**Ws**	**Ha**	**Hh**	**Hp**
ate	AB1786	Putative arginyl-tRNA--protein transferase	48	47	42	45	43	--	--	--	46	47	45	53	--	46	--
ctpA	AB2107	Carboxyl-terminal protease family protein	58	57	54	55	60	60	60	56	55	57	57	52	56	51	56
def	AB1713	Polypeptide deformylase	61	57	56	61	59	59	56	58	60	68	54	56	55	52	56
dsbA	AB1457	DsbA-like thioredoxin domain protein	--	--	--	--	--	--	--	--	--	--	--	--	--	--	--
dsbD	AB2174	Thiol:disulfide interchange protein DsbD	44	42	44	37	46	41	44	42	45	47	48	--	--	--	--
efp	AB2036	Translation elongation factor EF-P	71	71	71	69	72	73	73	69	70	66	64	67	71	75	71
frr	AB2261	Ribosome releasing factor	65	63	64	62	59	63	62	59	70	60	60	68	65	63	65
fus	AB1980	Translational elongation factor G	76	76	77	76	75	75	76	76	73	74	75	74	72	72	72
hemK	AB1204	Modification methylase	41	41	40	42	42	46	50	48	42	41	44	42	42	39	42
infA	AB0776	Translation initiation factor IF-1	79	79	79	79	79	81	79	81	86	88	82	85	79	83	81
infB	AB2032	Translation initiation factor IF-2	50	50	50	50	50	48	50	48	52	53	50	50	44	47	45
infC	AB0075	Translation initiation factor IF-3	69	69	70	71	70	66	68	64	62	67	69	70	57	64	57
lnt	AB0391	Apolipoprotein N-acyltransferase	36	36	35	34	38	39	36	35	38	38	43	41	34	37	34
map	AB0775	Methionine aminopeptidase	66	66	67	65	62	62	63	64	65	70	69	66	62	57	62
ppi	AB1911	Peptidyl-prolyl cis-trans isomerase	59	59	59	61	62	59	61	57	58	61	69	60	58	60	60
ppiC	AB0374	Peptidyl-prolyl cis-trans isomerase	38	37	39	40	42	38	40	41	42	42	40	43	38	37	39
prfA	AB0086	Peptide chain release factor 1	71	70	70	66	68	69	67	68	66	68	72	67	68	68	67
prfB	AB2060	Peptide chain release factor 2	68	68	68	68	68	67	67	62	72	75	69	70	65	65	66
selB	AB1534	Selenocysteine-specific elongation factor	44	44	42	40	46	46	47	40	--	--	61	50	--	44	--
slyD	AB1596	Peptidyl-prolyl cis-trans isomerase	48	50	48	51	49	49	47	47	59	67	46	43	44	49	44
tsf	AB0538	Translation elongation factor EF-Ts	58	59	57	57	56	57	58	55	53	41	45	54	51	52	52
tufA	AB1896	Elongation factor Tu	78	78	80	79	81	80	80	77	80	80	81	79	79	78	79
typA	AB2293	GTP-binding elongation factor family protein	75	75	74	75	76	76	76	75	73	73	73	76	74	74	74
	AB0281	Conserved hypothetical protein, putative DSBA oxidoreductase	29	26	--	--	--	26	--	--	36	38	37	--	--	--	--
	AB0426	Peptidyl-prolyl cis-trans isomerase-like protein	41	40	40	42	44	46	44	45	58	--	57	60	--	--	45
	AB0594	Protein tyrosine phosphatase	35	34	33	31	--	--	--	--	--	--	--	54	--	51	--
	AB1479	Biotin/lipoate A/B protein ligase family protein	--	--	--	--	--	--	--	--	37	--	--	--	--	--	--

**III.A.9**	**RNA synthesis, and RNA modification, and DNA transcription**		**Cj**	**Cc**	**Cl**	**Cu**	**Cf**	**Cv**	**Cn**	**Ch**	**Sv**	**Ni**	**Sd**	**Ws**	**Ha**	**Hh**	**Hp**
dbpA	AB1548	ATP-dependent RNA helicase DbpA	--	--	--	--	--	--	--	--	45	40	48	--	--	37	--
greA	AB0619	Transcription elongation factor GreA	49	50	50	49	55	54	55	52	56	53	56	54	57	55	56
msrA	AB1640	Peptide methionine sulfoxide reductase	40	41	38	--	--	--	--	--	35	47	--	--	--	39	--

msrB	AB1641	Peptide methionine sulfoxide reductase	73	69	--	68	--	--	--	--	46	41	--	--	--	60	--
nusA	AB0243	Transcription termination factor NusA	49	53	51	50	49	52	53	49	48	53	49	51	42	49	41
nusB	AB1858	Transcription termination factor NusB	59	56	55	50	52	49	50	56	60	58	55	54	52	53	51
nusG	AB1893	Transcription termination factor NusG	67	67	69	66	72	68	65	64	64	66	60	66	63	61	64
pnp	AB1191	Polyribonucleotide nucleotidyltransferase	56	56	55	54	54	55	55	53	52	57	55	57	46	50	46
rhlE	AB1487	ATP-dependent RNA helicase RhlE	--	--	--	--	--	--	--	--	63	42	70	--	41	--	41
rho	AB0285	Transcription termination factor Rho	75	75	75	74	72	73	72	72	74	76	69	77	73	70	73
rpoA	AB1023	DNA-directed RNA polymerase, alpha chain	52	52	53	51	56	56	55	55	57	53	55	60	49	58	49
rpoB	AB1888	DNA-directed RNA polymerase, beta chain	72	72	72	70	72	71	71	70	73	72	70	--	--	--	--
rpoC	AB1887	DNA-directed RNA polymerase, beta' chain	71	71	70	70	71	72	73	69	70	74	71	--	--	--	--
rpoZ	AB0647	DNA-directed RNA polymerase, omega chain	46	44	48	46	50	45	44	48	47	57	46	52	39	44	39
	AB0042	ATP-dependent RNA helicase, DEAD box family	--	--	--	--	35	--	--	--	59	61	52	51	50	49	48
	AB1123	Putative ATP-dependent RNA helicase RhlE	--	--	--	--	--	--	--	--	40	--	44	--	--	--	--
	AB2193	Probable ATP-dependent RNA helicase	--	--	--	--	--	--	--	--	--	--	--	--	--	--	--

**III.A.11**	**Phospholipids**		**Cj**	**Cc**	**Cl**	**Cu**	**Cf**	**Cv**	**Cn**	**Ch**	**Sv**	**Ni**	**Sd**	**Ws**	**Ha**	**Hh**	**Hp**
gpsA	AB0029	Glycerol 3-phosphate dehydrogenase	63	62	60	59	65	64	63	64	60	59	64	56	53	52	53
lgt	AB1273	Phosphatidylglycerol-prolipoprotein diacylglyceryl transferase	57	58	58	55	55	55	55	55	31	58	53	61	45	47	45
pgpA	AB0132	Phosphatidylglycerophosphatase A	53	53	52	51	47	53	52	49	46	50	50	54	53	55	52
pgsA	AB0870	CDP-1,2-diacyl-sn-glycero-3-phosphate phosphatidyltransferase	58	57	58	58	64	66	65	63	63	59	62	52	46	49	44
plsC	AB2112	1-acyl-sn-glycerol-3-phosphate acyltransferase PlsC	34	34	38	36	40	38	40	43	32	39	39	39	37	34	37
plsX	AB0291	Fatty acid/phospholipid synthesis protein	59	61	60	58	62	65	67	59	60	63	58	56	54	53	55
psd	AB2179	Phosphatidylserine decarboxylase	43	47	43	42	43	45	48	43	49	52	50	41	45	46	44
pssA	AB1161	CDP-diacylglycerol--serine O-phosphatidyltransferase	28	--	--	--	--	--	--	--	51	--	56	--	--	--	--
	AB0187	CDP-diacylglycerol--glycerol-3-phosphate 3-phosphatidyltransferase-related protein	--	--	--	--	--	--	--	--	--	--	--	--	--	--	--
	AB0188	CDP-diglyceride synthetase/phosphatidate cytidylyltransferase	--	--	--	--	--	41	--	--	--	--	--	--	--	27	--
	AB0189	1-acyl-sn-glycerol-3-phosphate acyltransferase	--	--	--	--	--	29	--	--	--	--	28	--	--	--	--

III.B Degradation of macromolecules

**III.B.1**	**RNA**		**Cj**	**Cc**	**Cl**	**Cu**	**Cf**	**Cv**	**Cn**	**Ch**	**Sv**	**Ni**	**Sd**	**Ws**	**Ha**	**Hh**	**Hp**
rnc	AB1623	Ribonuclease III	58	55	61	54	56	57	56	59	67	62	67	65	53	62	55
rnhA	AB1497	Ribonuclease HI	--	--	--	--	--	--	--	--	--	--	--	--	--	--	--
rnhB	AB1636	Ribonuclease HII	52	53	51	53	44	49	51	54	54	53	59	53	45	33	45
rnr	AB0235	Ribonuclease R	39	39	37	36	38	39	38	39	40	41	38	39	36	34	35
	AB1471	Ribonuclease H-like protein	--	--	--	--	--	--	--	--	--	--	46	--	--	--	--
	AB2006	S1 RNA binding domain protein	--	--	--	--	--	--	--	--	54	--	56	--	--	--	--

**III.B.2**	**DNA**		**Cj**	**Cc**	**Cl**	**Cu**	**Cf**	**Cv**	**Cn**	**Ch**	**Sv**	**Ni**	**Sd**	**Ws**	**Ha**	**Hh**	**Hp**
nfo	AB2210	Endonuclease IV	--	--	--	--	64	63	63	65	69	71	78	66	--	--	--
nth	AB0623	Endonuclease III	60	59	62	61	60	61	60	62	66	63	65	58	57	58	56
recD	AB1388	Exodeoxyribonuclease V, alpha subunit	--	--	--	--	--	--	--	--	--	--	48	--	--	--	--
recJ	AB2247	Single-stranded DNA-specific exonuclease	45	46	43	45	45	44	45	45	41	43	44	43	38	39	40
uvrB	AB0621	Excinuclease ABC, subunit B	67	67	68	68	68	69	69	67	70	71	70	70	65	67	65
uvrC	AB0790	Excinuclease ABC, subunit C	48	48	51	47	49	47	49	48	52	56	56	56	51	45	52
xseA	AB0423	Exodeoxyribonuclease VII, large subunit	38	40	38	40	45	43	43	40	43	48	47	54	42	48	43

xth	AB0824	Exodeoxyribonuclease	53	53	52	51	54	51	54	52	61	29	--	--	41	41	40
	AB0977	TatD-related deoxyribonuclease	61	57	62	59	58	60	59	58	67	58	63	58	59	53	59
	AB2026	Exopolyphosphatase-related protein	--	--	--	--	--	--	--	--	39	41	26	34	--	--	--

III.B.3	Proteins, peptides, and glycopeptides		Cj	Cc	Cl	Cu	Cf	Cv	Cn	Ch	Sv	Ni	Sd	Ws	Ha	Hh	Hp
clpA	AB1913	ATP-dependent Clp protease ClpAP, ATP-binding subunit ClpA	49	49	48	45	45	--	--	41	55	58	55	57	44	51	44
clpB	AB1635	ATP-dependent Clp protease, ATP-binding subunit	64	64	64	64	68	65	66	66	74	73	69	70	65	67	64
clpP	AB1714	ATP-dependent Clp protease, proteolytic subunit	80	80	82	82	83	79	79	79	81	83	80	82	75	76	75
clpS	AB1914	ATP-dependent Clp protease ClpAP adaptor protein ClpS	53	49	51	60	54	--	--	54	52	65	56	52	42	52	34
clpX	AB2203	ATP-dependent Clp protease ClpXP, ATP-binding subunit ClpX	62	63	62	60	62	65	64	65	63	68	65	64	57	61	56
htrA	AB2088	Periplasmic serine protease DO; heat shock protein HtrA	47	46	49	46	45	49	49	50	--	51	47	49	49	48	51
pepA	AB0703	Aminopeptidase	49	50	49	49	49	53	54	51	52	55	53	51	48	51	50
pepD	AB1312	Aminoacyl-histidine dipeptidase PepD	37	37	39	37	39	38	37	37	43	39	--	33	--	34	--
pepQ	AB0489	Prolidase (Xaa-Pro dipeptidase) (pepQ)	--	--	51	--	51	52	53	51	54	53	--	48	48	53	48
pfpI	AB0576	Peptidase, ThiJ/PfpI family protein	--	--	--	--	--	--	--	--	--	--	--	--	--	--	--
prlC	AB1203	Oligopeptidase A	--	--	--	--	--	--	--	--	53	52	55	--	--	--	--
pspA	AB0492	Protease IV (PspA)	47	48	49	47	50	44	44	47	52	53	52	44	44	44	44
smpB	AB2071	tmRNA-binding protein SmpB	59	61	61	60	65	69	64	60	57	59	61	64	58	58	57
	AB0528	Peptidase, M48 family	42	45	43	--	46	47	44	45	47	52	50	42	44	48	43
	AB0869	Membrane-associated zinc metalloprotease, putative	47	47	47	46	46	48	48	46	53	53	54	54	39	51	39
	AB0873	Putative zinc protease	65	63	64	60	63	64	64	55	61	68	65	67	56	59	57
	AB0881	Peptidase, S51 family	--	--	--	--	--	--	40	--	--	--	--	63	--	--	--
	AB1035	Peptidase, M50 family	--	--	--	--	--	--	--	--	--	--	--	--	--	--	--
	AB1092	Putative protease	--	--	--	--	--	--	65	61	39	--	--	53	--	59	--
	AB1221	Peptidase, M16 family	42	42	39	40	45	43	43	41	48	49	49	48	46	47	45
	AB1232	Peptidase, S24 family	32	--	34	--	--	--	--	30	45	46	53	50	--	--	--
	AB1626	Peptidase, M48 family	--	--	--	--	55	57	57	59	61	30	--	30	32	--	32
	AB1650	Peptidase, M23/M37 family	44	44	46	44	49	43	45	45	38	42	45	48	42	39	44
	AB1976	Peptidase, U32 family	56	56	55	55	59	60	61	58	63	66	66	64	65	62	64
	AB2027	Peptidase, M23/M37 family	33	33	34	35	37	36	34	36	36	37	39	35	27	33	26
	AB2188	Peptidase, A24 family	--	--	--	--	--	--	--	--	--	--	--	--	--	--	--

III.B.4	Polysaccharides		Cj	Cc	Cl	Cu	Cf	Cv	Cn	Ch	Sv	Ni	Sd	Ws	Ha	Hh	Hp
	AB1038	Glycosyl hydrolase	--	--	--	--	35	35	34	--	46	--	56	--	--	--	--
	AB1379	Polysaccharide deacetylase	--	--	--	--	--	--	--	--	--	--	--	--	--	44	--
	AB1772	Glycosyl hydrolase, putative	--	--	--	--	--	--	--	--	--	--	53	--	--	--	--

III.C Cell envelope

III.C.1	Membrane proteins, lipoproteins, and porins		Cj	Cc	Cl	Cu	Cf	Cv	Cn	Ch	Sv	Ni	Sd	Ws	Ha	Hh	Hp
amaA	AB1791	Acid membrane antigen A	39	37	39	38	37	42	41	37	39	41	40	38	31	35	32
crcB	AB2113	Camphor resistance CrcB protein	48	45	45	--	43	--	43	40	46	43	48	39	35	--	33
porA	AB2021	Major outer membrane protein	--	27	--	26	--	26	--	26	--	--	--	30	--	--	--
rlpA	AB0979	Rare lipoprotein A	39	39	40	39	43	40	39	38	42	--	39	44	--	43	45
	AB0007	Conserved hypothetical protein, putative integral membrane protein	43	42	40	37	--	--	--	--	--	--	36	--	--	35	--
	AB0014	Conserved hypothetical membrane protein	--	--	--	--	--	--	--	--	53	53	--	--	--	--	--
	AB0017	Hypothetical membrane protein	--	--	--	--	--	--	--	--	--	--	--	--	--	--	--

AB0025	Hypothetical protein, OmpA domain	--	--	--	--	--	--	--	--	--	--	--	--	--	--	--
AB0049	Conserved hypothetical integral membrane protein	51	49	52	48	49	46	47	43	58	55	51	54	57	48	51
AB0068	Conserved hypothetical periplasmic protein	44	42	38	46	39	39	--	--	26	--	41	49	39	35	40
AB0108	Conserved hypothetical protein, putative ammonia monooxygenase	--	--	--	--	--	--	--	--	--	--	--	--	--	--	--
AB0139	Conserved hypothetical periplasmic protein	33	34	30	28	38	35	--	--	--	--	38	33	38	32	38
AB0141	Conserved hypothetical periplasmic protein	39	39	38	35	39	42	--	--	--	--	51	41	42	40	38
AB0170	Conserved hypothetical periplasmic protein	--	--	--	--	--	40	38	--	37	34	--	43	--	36	--
AB0194	Hypothetical protein, ankyrin repeat family	--	--	--	--	--	--	--	--	--	--	--	--	--	--	--
AB0246	Putative lipoprotein	44	43	46	43	46	44	--	45	43	46	47	46	40	35	--
AB0254	OmpA domain protein	--	--	--	--	--	--	--	--	51	--	--	--	--	--	--
AB0266	Conserved hypothetical membrane protein, HPP family	--	--	--	--	--	--	--	--	--	--	45	--	--	--	--
AB0267	Hypothetical periplasmic protein	--	--	--	--	--	--	--	--	--	--	--	--	--	--	--
AB0269	Conserved hypothetical protein, probable periplasmic protein	43	42	43	44	45	43	42	41	38	--	--	--	--	--	--
AB0279	Hypothetical membrane protein	--	--	--	--	--	--	--	--	--	--	--	--	--	--	--
AB0315	Conserved hypothetical membrane protein (DUF1212 domain protein)	27	29	30	28	--	28	--	26	--	--	--	--	--	--	--
AB0316	Conserved hypothetical membrane protein (DUF1212 domain protein)	--	--	--	--	--	--	--	--	--	--	--	--	--	--	--
AB0326	Conserved hypothetical membrane protein	--	--	--	--	--	--	--	--	--	--	--	--	--	--	--
AB0333	Conserved hypothetical membrane protein (DUF204 domain protein)	45	45	--	46	42	41	38	40	--	--	--	52	--	--	--
AB0411	Hypothetical periplasmic protein	--	--	--	--	--	--	--	--	--	--	--	--	--	--	--
AB0436	Hypothetical periplasmic protein	--	--	--	--	--	--	--	--	37	--	--	--	--	--	--
AB0530	Integral membrane domain protein (DUF6 domain protein)	33	34	--	34	--	--	--	--	--	--	33	48	--	--	--
AB0559	Oxidoreductase, molybdopterin binding	57	55	54	58	--	--	--	--	--	--	--	57	--	--	--
AB0560	Conserved hypothetical membrane protein	29	26	29	28	--	--	--	--	--	--	--	36	--	27	--
AB0591	Conserved hypothetical membrane protein	37	38	38	40	39	38	38	34	44	43	41	41	37	38	37
AB0593	Rhomboid-like protein	43	44	46	40	48	47	42	46	32	--	--	--	--	--	--
AB0604	Membrane protein, putative (DUF6 domain protein)	--	26	--	--	--	--	--	--	--	--	--	--	--	--	--
AB0641	Conserved hypothetical membrane protein	35	33	34	35	33	31	31	34	31	36	34	35	33	31	33
AB0681	Conserved hypothetical membrane protein	--	--	--	--	--	--	--	--	--	--	--	--	--	--	--
AB0702	Conserved hypothetical membrane protein	54	58	--	59	55	58	57	51	29	60	36	56	--	58	28
AB0708	Conserved hypothetical membrane protein	--	--	--	--	25	--	--	--	--	28	31	27	--	--	--
AB0828	Outer membrane lipoprotein	--	36	37	--	--	--	--	--	--	--	--	--	--	--	--
AB0864	Conserved hypothetical membrane protein (DUF423)	--	--	--	--	--	--	--	--	--	--	--	--	--	--	--
AB0889	Putative lipoprotein thiredoxin	42	44	36	40	38	36	42	28	31	33	31	48	--	--	--
AB1008	Conserved hypothetical membrane protein	--	--	--	28	25	--	--	26	--	33	31	--	30	25	--
AB1033	Hypothetical membrane protein	--	--	--	--	--	--	--	--	--	--	--	--	--	--	--
AB1034	Putative membrane protein	--	--	--	--	--	--	--	--	--	--	--	--	--	--	--
AB1049	Conserved hypothetical membrane protein (DUF6 domain protein)	--	--	--	26	--	--	--	--	--	--	--	31	--	--	--
AB1058	Putative integral membrane protein	37	39	45	--	44	46	42	--	48	49	50	41	--	40	--
AB1059	Conserved hypothetical periplasmic protein	--	--	--	--	--	--	--	--	--	--	26	--	--	--	--
AB1065	Conserved hypothetical integral membrane protein (DUF6 domain protein)	28	28	--	29	--	--	--	--	--	--	65	33	--	--	--
AB1073	Conserved hypothetical lipoprotein	52	51	49	49	48	54	48	--	56	--	35	49	47	51	48
AB1126	Hypothetical outer membrane protein	--	--	--	--	--	--	--	--	--	--	--	--	--	--	--

AB1133	Conserved hypothetical integral membrane protein	40	41	43	40	45	45	43	44	46	48	46	45	41	41	40
AB1152	Alpha/beta hydrolase fold protein	--	--	--	--	--	--	--	--	--	--	--	--	--	--	--
AB1189	Conserved hypothetical membrane protein	32	32	32	31	31	32	32	32	28	31	31	30	--	27	26
AB1215	Conserved hypothetical lipoprotein	--	37	26	--	--	--	--	--	--	--	--	--	--	--	--
AB1219	Outer membrane surface antigen protein	38	39	40	39	39	40	41	38	36	45	39	43	35	40	35
AB1223	Conserved hypothetical membrane protein	51	55	59	54	42	56	51	48	59	61	51	56	46	47	42
AB1261	Conserved hypothetical membrane protein	--	--	39	38	39	46	41	43	--	40	42	--	--	--	--
AB1318	Conserved hypothetical integral membrane protein	54	53	54	52	54	57	57	56	54	55	53	56	52	52	52
AB1387	Conserved hypothetical membrane protein	32	32	34	33	36	33	34	31	32	38	37	34	34	31	34
AB1395	Conserved hypothetical membrane protein	27	--	26	--	--	26	--	--	--	25	--	--	--	--	--
AB1419	Conserved hypothetical membrane protein (DUF161)	--	--	--	--	--	--	--	--	--	--	--	--	--	--	--
AB1421	Conserved hypothetical membrane protein (DUF808)	--	--	--	--	--	--	--	--	--	--	71	--	--	--	--
AB1424	Conserved hypothetical membrane protein (DUF481)	--	--	--	--	--	--	--	--	--	--	32	--	--	--	--
AB1452	Putative periplasmic protein	41	40	38	44	34	40	39	37	--	--	33	--	--	--	--
AB1453	Conserved hypothetical integral membrane protein	43	45	42	44	50	46	46	46	--	--	48	--	--	--	--
AB1456	Putative lipoprotein thioredoxin	35	33	30	36	34	31	--	27	27	27	29	39	--	--	--
AB1532	Sel1-like repeat protein	--	--	--	--	--	--	26	--	--	--	--	--	--	--	--
AB1549	Conserved hypothetical integral membrane protein	31	39	40	39	52	43	43	41	40	29	44	49	40	37	36
AB1564	Putative integral membrane protein (DUF6 domain protein)	--	25	--	--	26	26	28	26	--	--	--	--	--	--	--
AB1565	Conserved hypothetical membrane protein (DUF6)	--	--	--	--	--	33	39	36	--	--	--	36	--	--	--
AB1586	Conserved hypothetical membrane protein	--	--	--	--	--	--	--	--	--	--	41	--	--	--	--
AB1598	OmpA/MotB precursor	41	42	42	42	45	46	47	45	39	39	47	43	36	38	36
AB1735	Hypothetical protein, ErfK/YbiS/YcfS/YnhG family	--	--	--	--	--	--	--	--	--	--	--	--	--	--	--
AB1757	Sterol desaturase-related protein	--	--	--	--	--	--	--	--	--	--	--	--	--	--	--
AB1765	Conserved hypothetical transmembrane protein	--	--	--	--	38	--	38	34	35	--	--	--	--	--	--
AB1797	Putative integral membrane protein	55	55	56	56	55	53	50	--	70	62	65	56	--	--	--
AB1814	Conserved hypothetical membrane protein	--	--	--	--	--	--	--	25	--	33	--	--	--	--	--
AB1855	Conserved hypothetical membrane protein (DUF6)	47	46	48	48	57	51	48	48	58	61	54	40	38	37	37
AB1927	Conserved hypothetical membrane protein (DUF318 domain protein)	30	31	29	--	--	--	--	44	62	59	--	59	--	--	--
AB1930	Putative integral membrane protein	43	42	43	39	41	41	40	--	49	--	46	37	--	--	--
AB1937	Hypothetical membrane protein	--	--	--	--	--	--	--	--	--	--	--	--	--	--	--
AB1941	ATP-binding protein	33	34	34	33	33	31	31	--	--	36	31	32	36	32	36
AB1991	Conserved hypothetical membrane protein	40	41	41	43	42	39	41	39	33	41	42	37	31	35	31
AB2013	Conserved hypothetical integral membrane protein, DedA homolog	54	55	58	56	61	57	59	56	60	37	57	67	--	--	--
AB2023	Conserved hypothetical membrane protein (DUF205 domain protein)	56	56	58	57	52	51	57	54	58	60	61	58	44	48	44
AB2131	Probable lipoprotein nlpC homolog precursor	36	--	--	--	--	--	--	--	--	--	31	--	--	--	--
AB2139	Conserved hypothetical membrane protein (DUF477)	--	--	--	--	--	--	34	--	--	--	--	--	--	--	--
AB2149	RND efflux system, outer membrane lipoprotein	31	30	32	29	31	31	28	28	--	--	--	36	--	28	--
AB2187	Conserved hypothetical membrane protein	30	29	31	32	29	27	31	31	36	33	35	35	34	36	34
AB2220	AAA family ATPase	48	48	48	48	49	52	49	--	57	59	58	61	--	56	--
AB2257	Conserved hypothetical integral membrane protein	50	50	52	51	48	55	56	46	51	53	43	54	48	44	48
AB2303	Sulfatase	40	40	36	--	40	38	--	25	30	--	--	41	27	38	28
AB2317	Conserved hypothetical membrane protein	36	36	37	37	44	42	42	41	55	52	52	47	--	35	--

III.C.2 Surface polysaccharides, lipopolysaccharides, and antigens	Cj	Cc	Cl	Cu	Cf	Cv	Cn	Ch	Sv	Ni	Sd	Ws	Ha	Hh	Hp

asnB	AB0679	Asparagine synthetase	--	--	--	27	36	--	31	--	--	39	31	43	--	--	--
dltA	AB1286	D-alanine activating enzyme	30	30	29	29	28	--	--	--	--	--	--	--	--	--	--
dltB	AB1287	D-alanyl transfer protein	--	--	--	--	--	--	--	--	--	--	--	--	--	--	--
dltC	AB1290	D-alanyl carrier protein	--	--	--	--	--	--	--	--	--	--	--	--	--	--	--
dltD	AB1289	Poly D-alanine transfer protein	--	--	--	--	--	--	--	--	--	--	--	--	--	--	--
gmhA	AB1808	D-sedoheptulose 7-phosphate isomerase	62	63	59	52	67	68	64	--	--	63	55	66	63	64	63
kdsA	AB1856	3-deoxy-D-manno-octulosonic acid 8-phosphate synthase	61	62	59	58	66	66	64	63	65	58	61	59	59	61	58
kdsB	AB1278	3-deoxy-manno-octulosonate cytidylyltransferase	53	54	51	50	49	52	55	51	60	56	59	50	47	46	50
kdtA	AB1969	3-deoxy-D-manno-octulosonic-acid transferase	47	49	43	46	48	48	48	46	51	46	48	47	44	44	44
lpxA	AB2204	UDP-N-acetylglucosamine acyltransferase	50	51	50	52	52	49	50	52	67	61	67	54	48	53	48
lpxB	AB2206	Lipid A disaccharide synthase	48	46	46	45	48	47	47	49	55	53	60	50	48	46	49
lpxC	AB2028	UDP-3-O-acyl-N-acetylglucosamine deacetylase	55	55	56	55	57	54	52	54	54	63	63	61	56	63	56
lpxD	AB1166	UDP-3-O-[3-hydroxymyristoyl] glucosamine N-acyltransferase	50	50	47	49	50	47	48	49	53	58	55	49	41	46	41
lpxK	AB1254	Lipid A biosynthesis protein LpxK	40	44	43	42	43	43	48	42	46	43	47	45	46	46	45
pglF	AB0697	Sugar epimerase/dehydratase	43	43	45	42	46	44	46	43	44	47	--	47	--	--	--
pglJ	AB0678	Glycosyltransferase	38	41	41	38	39	38	37	40	29	--	29	41	--	--	--
ugd	AB0660	UDP-glucose 6-dehydrogenase	--	--	--	--	29	28	29	26	25	29	82	30	--	28	--
waaC	AB1833	Lipopolysaccharide heptosyltransferase I	41	40	43	39	43	40	41	--	--	26	54	39	38	36	38
waaD	AB1806	ADP-L-glycero-D-manno-heptose-6-epimerase	56	57	54	52	62	60	60	27	25	63	70	56	53	52	52
waaE	AB1807	ADP-heptose synthase	49	48	52	49	55	54	54	--	--	57	63	56	48	54	46
waaF	AB1810	Lipopolysaccharide heptosyltransferase II	45	47	47	46	51	46	50	--	--	--	44	37	37	--	37
waaM	AB1832	Lipid A biosynthesis lauroyl acyltransferase	26	29	31	33	34	32	32	26	--	26	34	35	28	27	28
wbpG1	AB0671	Putative LPS biosynthesis protein WbpG	--	--	--	--	--	--	--	--	--	--	--	--	--	--	--
wbpG2	AB0684	Putative LPS biosynthesis protein WbpG	--	--	--	--	--	--	--	--	--	--	--	--	--	--	--
wbpG3	AB0689	Putative LPS biosynthesis protein WbpG	25	25	--	--	--	--	--	--	--	--	--	--	--	--	--
	AB0366	Glycosyltransferase, putative	--	--	--	--	--	--	--	--	40	--	46	--	--	--	--
	AB0661	NAD-dependent epimerase/dehydratase family protein	25	26	26	25	66	65	68	--	--	67	61	75	--	26	25
	AB0662	UDP-hexose dehydrogenase	--	--	--	--	70	72	75	61	64	70	64	70	--	57	--
	AB0665	DegT/DnrJ/EryC1/StrS aminotransferase	49	48	53	28	32	58	75	28	74	61	56	70	29	55	29
	AB0667	NAD-dependent epimerase/dehydratase family protein	28	29	29	27	27	27	87	26	27	28	26	28	28	28	30
	AB0668	DegT/DnrJ/EryC1/StrS aminotransferase	29	29	27	26	31	27	76	29	30	31	66	27	--	30	--
	AB0677	Putative O-antigen acyltransferase	--	--	--	--	--	--	--	--	--	--	--	--	--	--	--
	AB0680	Glycosyltransferase	28	28	30	30	28	31	32	33	45	--	26	33	--	--	--
	AB0683	O-antigen translocase	--	--	--	--	--	--	--	--	38	--	39	--	--	--	--
	AB0688	Glycosyltransferase	--	--	--	--	--	--	27	--	--	--	--	--	--	--	--
	AB0692	Glycosyltransferase	--	--	--	--	--	--	--	--	--	--	--	27	--	--	--
	AB0693	Putative hexose epimerase	--	--	--	--	--	--	78	33	--	35	83	54	--	--	--
	AB0694	Glycosyltransferase	--	--	--	--	--	--	--	--	42	55	--	--	--	--	--
	AB0695	Glycosyltransferase	25	--	--	25	--	--	--	--	26	--	27	--	27	25	25
	AB0696	Putative hexose epimerase	--	--	--	31	--	44	--	--	42	--	--	--	--	--	--
	AB0981	HAD-superfamily hydrolase subfamily IIIA:Phosphatase	52	51	49	50	49	57	57	49	61	56	52	56	48	49	46
	AB1255	DegT/DnrJ/EryC1/StrS aminotransferase	29	29	30	27	49	49	49	25	29	53	47	50	25	27	--
	AB1459	Glycosyltransferase	--	--	--	--	--	--	--	--	--	--	59	--	--	--	--
	AB1805	Putative heptose phosphatase	--	--	--	--	--	--	--	--	--	--	--	--	--	--	--
	AB1809	Putative sulfatase	--	--	--	--	--	--	--	--	28	--	52	--	--	25	--
	AB1812	Putative heptosyltransferase	--	--	--	--	--	--	--	--	--	--	39	--	30	--	30
	AB1817	Putative acetyltransferase	35	37	--	34	59	62	--	--	61	--	--	--	52	28	53

	AB1818	Putative glycosyltransferase	--	26	27	25	27	26	29	29	--	30	26	26	--	--	--
	AB1819	Putative glycosyltransferase	--	--	--	--	32	32	32	27	--	--	29	27	--	--	--
	AB1821	Probable glycosyltransferase	26	30	25	28	33	30	--	28	28	--	--	--	--	--	--
	AB1823	Glycosyltransferase	--	--	--	--	--	--	--	--	--	--	--	--	--	--	--
	AB1824	Putative O-antigen polymerase	--	--	--	--	--	--	--	--	29	--	27	--	--	--	--
	AB1825	Aminotransferase, DegT/DnrJ/EryC1/StrS family	28	30	28	27	27	31	33	28	32	32	32	30	--	30	30
	AB1826	dTDP-glucose 4,6-dehydratase	33	32	29	30	--	65	26	--	26	28	28	26	26	61	--
	AB1827	Glucose-1-phosphate thymidylyltransferase	--	--	--	--	--	63	--	--	74	--	25	29	26	68	26
	AB2233	DegT/DnrJ/EryC1/StrS aminotransferase	41	41	41	38	41	42	31	28	31	31	31	37	33	34	32
	AB2236	Glycosyltransferase	--	--	--	--	--	--	--	--	--	--	--	--	--	--	--

III.C.3 Surface structures

			Cj	Cc	Cl	Cu	Cf	Cv	Cn	Ch	Sv	Ni	Sd	Ws	Ha	Hh	Hp
dsbB	AB1458	Disulfide bond formation protein, DsbB family	--	--	--	--	--	--	--	--	--	--	--	--	--	--	--
flaA	AB2243	Flagellin	--	--	--	--	--	--	--	--	--	--	34	--	--	--	--
flaB	AB2244	Flagellin	--	--	--	--	--	--	--	--	--	--	32	--	--	--	--
flaG	AB1947	Polar flagellin	--	--	31	--	--	--	--	--	--	--	--	--	--	--	--
flgB	AB1957	Flagellar basal body rod protein FlgB	32	35	31	35	37	39	35	--	--	32	38	32	36	30	36
flgC	AB1938	Flagellar basal body rod protein FlgC	44	44	44	44	46	44	43	--	--	38	44	43	42	43	42
flgD	AB1952	Flagellar hook assembly protein FlgD	--	--	--	--	--	--	--	--	--	--	--	28	--	--	--
flgE1	AB1950	Flagellar hook protein FlgE	--	--	--	--	--	--	--	--	--	--	--	26	--	--	--
flgE2	AB1951	Flagellar hook protein FlgE	28	28	--	25	--	26	26	--	--	27	--	--	--	--	--
flgG1	AB1958	Flagellar distal rod protein FlgG	30	29	33	30	30	31	30	--	--	33	33	32	35	33	35
flgG2	AB1961	Flagellar distal rod protein FlgG	56	56	54	55	59	59	60	--	--	53	54	59	56	58	56
flgH	AB0205	Flagellar basal body L-ring protein FlgH	37	37	37	36	38	37	35	--	--	34	36	34	31	32	31
flgI	AB0198	Flagellar P-ring protein FlgI	37	37	38	36	41	42	41	--	--	32	39	40	40	42	42
flgK	AB0206	Flagellar hook-associated protein FlgK	--	--	--	--	--	--	--	--	--	--	--	--	--	--	--
flgL	AB1932	Flagellar hook-associated protein FlgL	--	--	--	--	--	--	--	--	--	--	--	--	--	--	--
flhA	AB1931	Flagellar biosynthesis protein FlhA	49	49	50	47	50	50	50	--	--	40	53	49	48	44	48
flhB	AB1935	Flagellar biosynthetic protein FlhB	46	44	43	45	45	--	43	--	--	39	44	40	39	39	39
flhF	AB1942	Flagellar biosynthesis (GTP-binding) protein FlhF	--	--	36	33	37	34	36	--	31	28	38	--	--	--	--
fliD	AB0207	Flagellar hook-associated protein 2	--	--	--	--	--	--	26	--	--	--	--	--	--	--	--
fliE	AB1939	Flagellar hook-basal body protein FliE	39	37	40	40	43	32	34	--	--	36	--	40	36	37	36
fliF	AB1956	Flagellar M-ring protein FliF	29	29	31	29	32	32	32	--	--	--	30	30	32	29	31
fliG	AB1955	Flagellar motor switch protein FliG	40	40	40	41	40	43	44	--	--	28	42	43	39	42	39
fliH	AB1954	Flagellar assembly protein FliH	--	--	--	--	25	--	--	--	--	--	--	--	27	28	--
fliI	AB1933	Flagellum-specific ATP synthase FliI	58	58	56	58	60	59	58	29	30	37	60	61	54	53	54
fliL	AB0203	Flagellar basal body-associated protein	27	26	27	29	--	28	28	--	--	--	--	28	--	--	--
fliM	AB0197	Flagellar motor switch protein FliM	38	38	38	38	40	38	38	--	--	--	36	35	36	36	37
fliN	AB1953	Flagellar motor switch protein FliN	42	42	42	42	44	42	41	--	--	37	43	43	--	--	--
fliP	AB0998	Flagellar biosynthetic protein FliP	60	60	59	58	59	60	57	--	--	49	57	60	60	59	--
fliQ	AB0200	Flagellar basal body protein FliQ	44	44	44	43	45	43	41	--	--	36	41	43	43	43	41
fliR	AB1936	Flagellar biosynthetic protein FliR	28	27	28	29	26	27	28	--	--	--	27	29	--	27	--
fliS	AB0208	Flagellar protein FliS	41	41	39	42	42	48	51	--	--	34	43	50	46	47	45
fliY	AB1946	Flagellar motor switch protein FliY	28	27	28	27	28	29	29	--	--	--	29	33	28	--	29
flmE	AB2238	Flagellin modification protein FlmE	--	--	--	--	25	--	--	--	--	--	--	--	--	--	--
hisF2	AB0673	Imidazoleglycerol phosphate synthase, cyclase subunit	42	41	44	33	38	38	39	42	39	39	40	41	--	42	--
hisF3	AB0691	Imidazoleglycerol phosphate synthase, cyclase subunit	39	40	40	37	41	41	42	42	37	39	37	39	--	37	--

hisH2	AB0672	Glutamine amidotransferase HisH	45	45	43	38	41	41	45	41	44	48	48	40	--	37	--
hisH3	AB0690	Glutamine amidotransferase HisH	46	47	48	40	43	43	45	41	44	42	44	40	--	39	--
neuA	AB2234	Acylneuraminate cytidylyltransferase	48	47	47	48	42	49	32	--	--	--	36	52	37	51	--
neuB	AB2235	N-acetylneuraminic acid synthetase	56	56	56	53	55	62	37	--	--	--	36	58	53	53	53
pseB	AB2232	UDP GlcNAc dehydratase/reductase PseB, putative	74	73	74	74	73	75	--	32	--	--	--	71	65	69	66
	AB1396	FlhB domain protein	59	59	56	59	61	58	58	--	--	--	60	42	38	40	37

**III.C.4 Murein sacculus and peptidoglycan**			**Cj**	**Cc**	**Cl**	**Cu**	**Cf**	**Cv**	**Cn**	**Ch**	**Sv**	**Ni**	**Sd**	**Ws**	**Ha**	**Hh**	**Hp**
amiA	AB0643	N-acetylmuramoyl-L-alanine amidase	--	--	--	--	40	40	40	37	38	39	39	--	--	41	--
dacA	AB0118	Serine-type D-Ala-D-Ala carboxypeptidase	--	--	--	--	--	--	--	--	--	--	--	56	--	--	--
ddlA	AB0748	D-alanine--D-alanine ligase	51	51	51	51	49	52	50	53	57	53	49	57	49	54	51
mltA	AB2181	Peptidoglycan N-acetylmuramoylhydrolase	--	--	--	--	--	--	--	--	--	--	40	--	--	--	--
mraY	AB1898	Phospho-N-acetylmuramoyl-pentapeptide transferase	61	57	59	58	60	60	61	59	54	63	63	59	57	57	57
mrdB	AB1007	Rod shape-determining protein RodA	59	59	58	57	59	58	61	52	55	59	59	59	53	51	53
mreB	AB2202	Rod shape-determining protein MreB	69	69	70	70	68	69	68	68	67	69	71	71	65	67	65
mreC	AB2201	Rod shape-determining protein MreC	33	36	--	36	35	30	29	35	--	33	37	39	--	28	30
murA	AB1854	UDP-N-acetylglucosamine 1-carboxyvinyltransferase	65	65	66	63	66	68	68	66	67	66	68	64	59	63	61
murB	AB2223	UDP-N-acetylenolpyruvoylglucosamine reductase	47	47	46	47	46	47	42	46	50	50	49	48	44	45	43
murC	AB0231	UDP-N-acetylmuramate--alanine ligase	53	53	51	50	54	55	57	53	56	60	55	53	53	51	54
murD	AB1897	UDP-N-acetylmuramoylalanine--D-glutamate ligase	48	48	48	48	51	49	52	50	42	49	49	51	42	48	43
murE	AB0518	UDP-N-acetylmuramoylalanyl-D-glutamyl-2, 6-diaminopimelate ligase	48	47	47	45	49	46	47	48	47	47	51	48	47	47	47
murF	AB0750	UDP-N-acetylmuramoylalanyl-D-glutamyl-2,6-diaminopimelate ligase	44	45	44	43	46	47	49	46	46	47	51	47	43	45	41
murG	AB1908	UDP-N-acetylglucosamine--N-acetylmuramyl-(pentapeptide) pyrophosphoryl-undecaprenol N-acetylglucosamine transferase	51	49	48	47	47	51	52	49	45	47	54	47	45	45	45
murI	AB0283	Glutamate racemase	35	35	35	35	33	36	37	--	58	34	38	36	36	33	34
pbpA	AB1901	Penicillin-binding protein 1A	50	49	49	48	53	51	52	49	50	51	49	55	48	49	49
pbpB	AB1909	Penicillin-binding protein	49	49	47	47	53	55	52	48	52	51	53	52	45	49	46
pbpC	AB0999	Penicillin-binding protein	49	49	49	49	46	49	48	45	48	52	51	48	46	44	46
slt	AB1225	Soluble lytic murein transglycosylase (slt)	34	34	33	33	31	33	30	32	--	31	--	33	30	33	31
	AB0978	Putative lytic murein transglycosylase	28	28	30	27	28	28	28	--	26	31	30	29	26	25	26

IV. Cell processes

IV.A Transport/binding proteins

**IV.A.1 Amino acids and amines**			**Cj**	**Cc**	**Cl**	**Cu**	**Cf**	**Cv**	**Cn**	**Ch**	**Sv**	**Ni**	**Sd**	**Ws**	**Ha**	**Hh**	**Hp**
brnQ	AB1374	Branched-chain amino acid transport system II carrier protein	--	--	--	--	30	--	--	31	--	--	--	--	--	--	--
gltS	AB0215	Sodium:glutamate symporter	--	--	49	--	51	--	--	--	--	--	--	--	51	--	51
livJ	AB1512	Leucine/isoleucine/valine-binding protein	--	--	--	--	--	--	--	--	--	--	52	37	--	--	--
	AB0802	Urea/short-chain amide ABC transporter, periplasmic urea/short-chain amide-binding protein	--	--	--	--	--	--	--	--	--	--	--	--	--	--	--
	AB0803	Urea/short-chain amide ABC transporter, permease protein	--	--	--	--	--	--	--	--	--	--	--	--	--	--	--
	AB1593	Sodium:alanine symporter	40	39	41	40	39	37	42	--	35	--	33	36	37	42	38

**IV.A.2 Cations**			**Cj**	**Cc**	**Cl**	**Cu**	**Cf**	**Cv**	**Cn**	**Ch**	**Sv**	**Ni**	**Sd**	**Ws**	**Ha**	**Hh**	**Hp**
amtB	AB1159	Ammonium transporter	--	40	--	--	45	--	--	--	69	60	49	50	--	--	--

bfrE	AB0726	Probable TonB-dependent receptor	--	--	--	--	--	25	--	--	--	--	--	26	--	--	--
corA	AB1652	Magnesium and cobalt transport protein	39	39	--	40	--	--	--	--	--	--	--	43	33	40	33
czcA	AB0067	Heavy metal efflux pump	--	--	--	--	--	--	--	--	60	56	63	--	54	30	54
czcB	AB0066	Cation efflux system, membrane fusion protein	--	25	--	--	--	--	--	--	31	33	39	--	36	--	36
exbB1	AB0379	Biopolymer transport protein ExbB	28	28	34	28	32	30	29	--	33	--	30	30	29	33	28
exbB2	AB0707	Biopolymer transport protein ExbB	45	45	42	49	48	48	54	48	52	54	58	53	50	48	48
exbB3	AB1602	Biopolymer transport protein ExbB	46	48	48	47	46	46	--	46	52	46	45	45	43	41	41
exbD1	AB0378	Biopolymer transport protein ExbD	28	28	30	28	31	31	32	31	--	36	27	26	27	--	26
exbD2	AB0706	Biopolymer transport protein ExbD	43	42	41	35	46	42	41	39	34	43	42	41	44	44	44
exbD3	AB1601	Biopolymer transport protein ExbD	54	54	48	51	52	49	49	48	50	60	47	59	51	52	50
fbpA	AB1993	Putative iron-uptake ABC transporter, periplasmic iron-binding protein	47	47	44	48	49	--	--	--	--	--	--	--	--	--	--
fbpB	AB1994	Putative iron-uptake ABC transporter, permease protein	35	35	33	35	37	--	--	--	--	--	--	--	--	--	--
feoA	AB1862	Ferrous iron transport protein A	35	35	--	--	--	--	--	--	34	47	42	55	--	--	--
feoB1	AB0548	Ferrous iron transport protein B	--	51	54	52	--	--	--	54	64	50	61	59	47	49	47
feoB2	AB1863	Ferrous iron transport protein B	--	--	29	--	--	--	--	--	--	30	--	45	26	27	26
kefB	AB1466	Glutathione-regulated potassium-efflux system protein KefB	37	38	38	39	39	39	39	38	39	39	49	44	--	34	--
ktrA	AB1295	TRK system potassium uptake protein TrkA, putative	41	42	43	41	40	--	--	--	--	--	45	50	--	42	--
ktrB	AB1296	TRK system potassium uptake protein TrkB, putative	43	43	46	42	48	--	--	--	--	--	--	46	--	41	--
nhaA1	AB1380	Sodium:hydrogen antiporter	36	37	39	38	39	38	38	37	39	39	40	48	47	46	47
nhaA2	AB1755	Sodium:hydrogen antiporter	42	43	41	42	42	39	41	39	42	44	43	48	48	46	48
	AB0175	Calcium:sodium antiporter	--	--	--	--	--	--	--	--	33	32	81	39	--	--	--
	AB0325	TonB-dependent receptor protein	--	--	--	--	28	28	--	--	--	--	--	28	--	--	--
	AB0331	TonB-dependent receptor protein	--	--	--	--	--	--	--	--	26	--	--	43	--	--	--
	AB0338	TonB-dependent receptor protein	--	--	--	--	--	--	--	--	27	--	27	29	--	--	--
	AB0340	TonB-dependent receptor protein	--	--	--	--	--	25	--	--	--	--	--	33	--	--	--
	AB0342	Cation efflux system, membrane protein	46	45	--	45	46	35	37	--	27	29	--	--	--	--	--
	AB0481	Heavy-metal transporting P-type ATPase	32	32	30	31	39	37	38	32	43	32	44	37	39	34	39
	AB0497	Cation efflux protein	--	--	--	29	30	--	--	--	--	--	--	--	--	27	--
	AB0508	Sodium:solute symporter (Ssf family)	--	26	--	--	--	--	--	--	--	--	--	41	--	25	--
	AB0543	Cation-transporting ATPase, P-type	43	42	44	43	49	45	43	47	50	51	52	49	37	44	36
	AB0562	Probable sodium/hydrogen antiporter	35	34	36	38	39	37	36	35	34	42	43	43	38	40	37
	AB0625	TonB-dependent receptor protein	--	--	--	--	--	--	--	--	46	34	47	--	--	--	--
	AB0626	TonB-dependent receptor protein	--	--	--	--	--	31	--	--	--	--	--	--	--	30	--
	AB0705	TonB-dependent receptor protein	26	26	--	--	26	--	--	--	31	--	29	27	--	--	--
	AB0714	Heavy metal translocating P-type ATPase	31	--	31	--	59	60	59	53	29	--	31	57	28	--	28
	AB0926	Cation efflux protein	48	47	45	43	46	43	47	43	54	56	54	57	--	--	--
	AB0938	Cobalt ABC transporter, permease protein, putative	--	--	--	--	36	32	--	36	--	--	--	27	--	--	--
	AB0939	Cobalt ABC transporter, ATP-binding protein, putative	29	34	28	30	50	53	32	50	31	32	31	39	29	32	27
	AB0988	TonB-dependent receptor protein	--	--	--	--	--	27	28	--	--	--	--	--	--	--	--
	AB1108	TonB-dependent receptor protein	--	--	--	--	--	--	--	--	--	--	39	32	--	--	--
	AB1109	Putative iron compound ABC transporter, periplasmic substrate-binding protein	29	29	--	28	25	26	--	--	--	--	38	--	--	--	--
	AB1110	Putative iron compound ABC transporter, ATP-binding protein	31	32	34	35	32	30	31	30	28	28	38	26	26	28	28
	AB1111	Putative iron compound ABC transporter, permease protein	33	31	--	35	34	32	32	--	--	--	43	27	31	--	31
	AB1383	TonB-dependent receptor protein	--	--	--	--	37	40	--	--	--	--	--	44	--	--	--
	AB1462	TonB-dependent receptor protein	--	--	--	--	--	27	27	--	--	--	--	28	--	--	--

	AB1484	TonB-dependent receptor protein	--	--	--	--	37	40	--	--	--	--	--	45	--	--	--
	AB1515	Mn2+ and Fe2+ transporter, NRAMP family	--	--	--	--	--	--	--	--	--	--	--	--	--	--	--
	AB1516	Heavy-metal transporting ATPase	--	--	--	--	--	38	35	--	--	--	--	--	--	--	--
	AB1517	Heavy-metal transport protein, MerT homolog	--	--	--	--	--	37	41	--	--	--	--	--	--	--	--
	AB1573	TonB-dependent receptor protein	--	--	--	--	30	36	--	--	--	--	--	36	--	--	--
	AB1584	TonB-dependent receptor protein	--	--	--	--	--	--	--	--	--	--	27	29	--	--	--
	AB1752	Putative nickel transporter	--	28	--	--	28	29	31	29	--	--	--	26	--	--	--
	AB1753	Putative cation ABC transporter, periplasmic-binding protein	40	40	42	38	--	48	49	45	42	34	26	39	--	28	--
	AB1802	Potassium channel protein, putative	--	--	44	--	47	47	48	44	49	--	49	55	49	48	50
	AB1868	Heavy metal-(Cd/Co/Hg/Pb/Zn)-translocating P-type ATPase	29	30	27	28	31	28	29	29	36	27	29	45	40	36	41
	AB1870	TonB-dependent receptor protein	--	--	--	--	45	47	--	--	--	--	--	56	--	--	--
	AB1985	TonB-dependent receptor protein	--	26	25	--	--	27	26	--	--	--	39	37	--	26	--
	AB1998	Ferrichrome-iron receptor	--	--	--	--	25	27	--	--	--	--	--	54	--	--	--
	AB2150	TonB-dependent receptor protein	--	--	--	--	--	28	27	--	--	--	--	25	--	--	--
	AB2265	CorA-like Mg2+ transporter protein	--	--	--	--	--	--	--	--	--	--	--	--	--	--	--
	AB2272	TonB-dependent receptor protein	--	--	--	--	--	--	26	--	--	--	--	--	--	--	--
	AB2299	TonB-dependent receptor protein	--	--	--	--	29	32	--	--	--	--	--	34	--	--	--

IV.A.3 Carbohydrates, organic acids, and alcohols

			Cj	Cc	Cl	Cu	Cf	Cv	Cn	Ch	Sv	Ni	Sd	Ws	Ha	Hh	Hp
benE	AB1475	Benzoate membrane transport protein	--	--	--	--	--	--	--	--	--	--	--	--	--	--	--
dctA	AB0184	C4-dicarboxylate transport protein	59	57	--	58	64	63	62	26	--	28	25	69	--	--	--
dctM	AB0359	C4-dicarboxylate transport protein	--	--	--	--	--	68	67	29	--	--	30	70	--	66	--
dctP	AB0357	C4-dicarboxylate-binding periplasmic protein	--	--	--	--	--	61	66	--	--	--	--	59	--	56	--
dctQ	AB0358	C4-dicarboxylate transport system, permease small subunit	--	--	--	--	--	48	45	27	--	--	--	54	--	39	--
lctP	AB0033	L-lactate permease	52	52	52	51	51	46	--	--	--	--	--	--	57	49	57
	AB0102	Conserved hypothetical protein, putative tricarboxylic transport protein TctC	--	25	--	--	26	--	--	--	--	--	--	--	--	--	--
	AB0103	Conserved hypothetical protein, putative tricarboxylic transport protein TctB	--	--	--	--	--	--	--	--	--	--	--	--	--	--	--
	AB0104	Conserved hypothetical protein, putative tricarboxylic transport protein TctA	--	45	--	--	--	--	--	--	--	--	--	--	--	--	--
	AB0520	C4-dicarboxylate transporter/malic acid transport protein	--	--	--	--	31	46	--	--	--	--	58	50	--	29	--
	AB0933	Major facilitator superfamily protein, putative oxalate:formate antiporter	--	--	--	--	--	--	--	--	--	25	--	--	--	--	--
	AB1216	Sugar transporter, putative	--	--	--	--	--	--	--	--	--	--	--	--	--	--	--

IV.A.4 Nucleosides, purines, and pyrimidines

			Cj	Cc	Cl	Cu	Cf	Cv	Cn	Ch	Sv	Ni	Sd	Ws	Ha	Hh	Hp
tsx	AB0221	Nucleoside-specific channel-forming protein	--	--	--	--	--	--	--	--	--	--	--	--	--	--	--

IV.A.5 Anions

			Cj	Cc	Cl	Cu	Cf	Cv	Cn	Ch	Sv	Ni	Sd	Ws	Ha	Hh	Hp
cysA	AB2281	Sulfate ABC transporter (ATP-binding protein)	45	44	34	38	42	--	--	--	--	40	--	--	--	--	--
cysT	AB2283	Sulfate transport system permease protein	26	27	27	--	--	--	--	--	33	31	--	34	--	32	29
cysW	AB2282	Sulfate transport system permease protein	--	25	--	25	28	--	--	--	--	31	28	27	--	--	25
modA	AB0012	Molybdenum ABC transporter, periplasmic molybdate-binding protein	38	37	51	36	49	58	51	47	34	--	34	36	--	36	39
modB	AB0010	Molybdenum ABC transporter, permease protein	53	53	52	52	58	60	59	61	74	--	57	55	--	48	52

modD	AB0009	Molybdenum ABC transporter, ATP-binding protein	41	41	45	44	49	52	48	47	67	--	46	45	28	40	32
modE	AB0013	Molybdenum-binding protein, N-terminal:molybdenum-pterin binding domain	--	--	--	--	36	37	36	38	51	--	35	41	--	--	--
pitA	AB0522	Phosphate transporter family protein	--	--	--	--	--	--	--	--	--	--	--	--	--	--	--
pstA	AB0091	Phosphate ABC transporter, permease protein	50	50	49	--	--	--	--	--	--	--	--	62	--	--	--
pstB	AB0092	Phosphate transporter, ATP-binding protein	60	60	61	64	52	42	41	51	51	55	52	70	34	34	33
pstC	AB0090	Phosphate ABC transporter, permease protein	52	52	52	32	33	--	--	33	31	34	35	--	--	--	--
pstS	AB0088	Phosphate ABC transporter, periplasmic phosphate-binding protein	47	47	43	--	--	--	--	--	--	--	--	57	--	--	--
sbp	AB2287	Sulfate-binding protein precursor	--	--	--	--	--	--	--	--	--	--	--	--	--	--	--
	AB0044	Phosphate permease, putative	--	--	--	--	--	--	--	--	59	54	55	52	48	52	48
	AB0093	Phosphate transport system regulatory protein PhoU, putative	--	--	--	--	--	--	--	--	33	36	35	--	--	--	--
	AB0906	Molybdenum transport system protein ModD, putative	--	--	--	--	44	44	46	--	27	26	37	36	30	27	31
	AB1052	Putative sodium:sulfate symporter	--	--	--	--	--	--	--	--	--	--	--	--	--	--	--
	AB1087	Sodium:phosphate cotransporter	37	36	--	37	36	--	--	36	42	28	69	28	--	33	--
	AB1184	Sulfate permease family protein	--	--	--	--	--	--	--	--	30	43	34	31	--	--	--
	AB1292	Sulfate permease	--	--	--	--	--	--	--	--	42	42	79	27	--	--	44
	AB1788	Voltage-gated chloride channel family protein	--	--	--	--	--	--	--	--	58	58	--	--	--	--	--
	AB1986	Putative sulfonate/nitrate transport system substrate-binding protein	--	--	--	--	--	32	--	--	--	54	60	52	--	--	--
	AB2172	Sodium:sulfate symporter family protein	--	--	--	--	--	--	--	--	32	--	--	--	45	--	--
	AB2216	Sulfate permease family protein	--	--	--	--	--	--	--	--	26	32	38	40	--	--	--

**IV.A.6 Other**			**Cj**	**Cc**	**Cl**	**Cu**	**Cf**	**Cv**	**Cn**	**Ch**	**Sv**	**Ni**	**Sd**	**Ws**	**Ha**	**Hh**	**Hp**
araJ	AB1984	Putative transport protein AraJ	30	25	--	26	--	--	--	--	--	--	--	--	30	29	29
betT	AB1465	High-affinity choline transport	--	--	37	--	--	--	47	46	--	--	--	52	--	--	--
iamA	AB0111	ABC transporter, ATP-binding protein	46	47	53	43	46	49	43	45	45	38	43	50	47	48	47
iamB	AB0112	ABC transporter, permease protein	45	46	46	45	42	44	45	40	45	--	41	--	36	36	38
msbA	AB0875	Multidrug resistance protein MsbA	47	49	53	48	54	55	54	55	50	51	49	56	47	49	47
mscS	AB0387	Mechanosensitive ion channel	--	--	--	--	43	--	--	--	--	--	48	38	33	36	32
	AB0016	Conserved hypothetical protein, putative MFS permease	--	--	--	--	--	--	--	--	--	--	--	--	--	--	--
	AB0045	Conserved hypothetical protein, putative permease	--	--	--	--	--	--	--	--	--	44	42	40	--	--	--
	AB0110	ABC transporter, periplasmic substrate-binding protein, putative	36	34	29	35	--	--	--	--	32	--	32	--	--	--	--
	AB0265	Conserved hypothetical membrane protein, predicted permease	43	44	42	44	48	46	46	48	--	--	--	51	--	44	--
	AB0328	MFS family, AmpG related permease	--	--	--	--	--	--	--	--	--	--	--	--	--	--	--
	AB0334	Major facilitator superfamily transporter	--	--	--	--	--	--	26	--	--	--	--	54	--	--	--
	AB0335	Major facilitator superfamily transporter	--	--	--	--	--	--	--	--	--	--	--	39	--	--	--
	AB0336	ABC transporter, ATP-binding/permease protein	27	27	28	26	29	27	27	32	26	29	27	51	27	28	26
	AB0337	ABC transporter, ATP-binding/permease protein	29	29	27	--	26	28	27	35	27	25	30	57	26	26	--
	AB0439	ABC transporter, ATP-binding protein	39	40	41	40	43	39	38	42	50	44	45	38	39	39	39
	AB0440	ABC transporter, permease protein	--	--	--	--	--	--	--	--	48	--	--	--	--	--	--
	AB0469	ABC transporter, ATP-binding protein	26	25	25	26	26	25	--	25	--	--	--	--	--	--	--
	AB0501	Sodium:solute symporter family protein	--	58	--	--	57	--	--	--	68	65	65	65	25	58	--
	AB0504	Sodium:solute symporter family protein	--	60	--	--	56	--	--	--	65	64	63	63	--	59	--
	AB0539	ABC transporter, ATP-binding protein	59	59	60	61	63	67	68	61	56	58	64	57	54	58	55
	AB0551	ABC transporter, ATP-binding protein	46	46	49	44	46	52	55	53	59	41	40	50	33	46	33
	AB0552	ABC transporter, permease protein	50	47	50	47	58	58	60	49	63	48	31	54	--	45	--

	AB0556	Putative efflux protein, bile acid:sodium symporter family	--	--	--	--	--	--	--	--	--	--	--	--	--	--	--
	AB0585	Major facilitator superfamily transporter	49	50	51	49	50	47	47	48	49	51	52	52	45	47	46
	AB0676	ABC transporter, ATP-binding/permease protein	48	48	49	47	30	29	29	30	27	29	25	55	42	29	42
	AB0710	ABC transporter, ATP-binding protein	42	42	41	42	40	40	39	40	38	48	64	42	37	61	37
	AB0711	ABC transporter, permease protein	--	--	--	--	--	--	--	--	--	--	58	--	--	42	--
	AB0804	ABC transporter, permease protein	--	--	--	--	25	--	--	--	--	--	--	25	--	--	--
	AB0805	ABC transporter, ATP-binding protein	34	35	34	36	35	30	29	28	30	29	30	35	29	28	29
	AB0806	ABC transporter, ATP-binding protein	35	34	36	36	34	32	33	33	30	34	36	37	34	34	34
	AB0831	ABC transporter protein	--	--	--	--	--	--	--	--	54	56	64	53	--	--	--
	AB0839	Auxin efflux carrier protein, putative	--	--	--	--	49	--	--	--	--	--	--	36	--	--	--
	AB0893	ABC transporter, permease protein	31	30	26	27	29	33	--	--	29	66	63	30	30	--	30
	AB0909	ABC transporter, ATP-binding protein	51	51	49	50	53	51	52	52	59	60	58	59	30	32	30
	AB0955	Putative permease	--	--	--	--	32	--	32	29	30	--	--	--	--	--	--
	AB1042	ABC transporter, ATP-binding protein	26	26	28	27	27	29	31	27	32	30	26	30	27	26	26
	AB1054	Conserved hypothetical protein, possible transporter	--	--	--	--	--	--	--	--	--	--	--	30	--	--	--
	AB1060	Auxin efflux carrier protein	29	29	30	--	31	27	29	26	--	--	60	30	--	29	--
	AB1068	ABC transporter, ATP-binding protein	62	61	63	62	65	64	65	30	69	29	70	69	67	65	67
	AB1113	ABC transporter, ATP-binding protein	29	28	29	27	30	26	26	28	49	45	65	42	26	27	27
	AB1127	ABC transporter, transmembrane region	--	26	--	25	28	53	54	34	27	--	33	--	--	--	--
	AB1147	ABC transporter, ATP-binding protein	--	--	25	26	--	--	--	--	--	--	25	25	--	26	--
	AB1155	Conserved hypothetical protein, putative efflux protein	58	57	56	58	62	61	61	57	--	--	--	--	58	58	57
	AB1280	ABC transporter, ATP-binding protein	68	69	68	66	73	71	72	68	69	67	72	68	66	67	66
	AB1370	ABC transporter, ATP-binding protein	27	27	27	27	57	--	25	49	34	37	--	25	--	--	--
	AB1371	ABC transporter, permease protein	--	--	--	--	60	--	--	50	28	30	--	--	--	--	--
	AB1372	ABC transporter, permease protein	--	--	--	--	56	--	--	43	29	28	--	--	--	--	--
	AB1397	ABC transporter, ATP-binding protein	31	31	33	32	34	33	33	26	61	26	62	33	33	34	33
	AB1615	Auxin efflux carrier protein	28	26	--	--	31	28	30	29	--	--	26	31	--	29	--
	AB1745	Auxin efflux carrier protein	--	--	26	--	--	--	--	--	--	--	--	--	--	--	--
	AB1766	Ion transport protein	--	--	--	--	--	--	--	--	--	--	--	--	--	--	--
	AB1860	ABC transporter, ATP-binding protein	28	26	26	25	31	49	27	26	27	--	28	26	27	29	27
	AB1900	ABC transporter, ATP-binding protein	30	32	33	32	30	31	37	30	34	52	54	31	32	29	32
	AB1988	ABC transporter, ATP-binding protein	35	34	35	35	35	41	37	33	31	67	67	52	36	30	35
	AB1989	ABC transporter, permease protein	--	--	--	--	27	43	--	--	--	65	73	65	--	--	--
	AB1996	ABC transporter, ATP-binding protein	39	39	41	37	39	33	34	33	33	31	36	37	34	--	33
	AB2083	Major facilitator superfamily transporter	--	35	--	--	35	--	--	34	--	--	--	39	--	--	--
	AB2100	ABC transporter, ATP-binding protein	38	40	41	37	38	37	53	54	37	38	56	50	36	36	37
	AB2144	Major facilitator superfamily transporter	29	29	29	29	29	28	26	--	--	--	--	31	--	--	--
	AB2215	Major facilitator superfamily transporter, Bcr/CflA subfamily	28	28	27	29	31	30	30	--	--	--	--	28	--	25	--
	AB2264	Putative major facilitator superfamily transporter	--	--	--	--	--	--	--	--	49	52	47	42	--	--	--
	AB2294	Conserved hypothetical protein, putative transport system permease protein	48	49	50	48	47	--	--	--	--	--	73	--	--	62	--

IV.B Chaperones

			Cj	Cc	Cl	Cu	Cf	Cv	Cn	Ch	Sv	Ni	Sd	Ws	Ha	Hh	Hp
cbpA	AB1367	Curved DNA-binding protein CbpA	54	53	60	57	57	58	59	54	66	64	64	66	54	60	54
dnaJ	AB1301	Co-chaperone and heat shock protein DnaJ	56	56	56	53	58	--	56	52	58	58	62	60	52	57	53
dnaK	AB2182	DnaK-type molecular chaperone	71	71	70	72	71	73	73	70	74	72	76	74	71	73	71
groEL	AB0431	60 kDa chaperonin	77	77	78	77	78	77	78	77	76	77	76	76	75	75	75

groES	AB0430	10 kDa chaperonin	56	55	60	59	56	59	56	55	56	55	51	56	--	49	--
grpE	AB2183	Heat shock protein GrpE	44	45	45	48	43	48	50	41	46	50	51	52	46	44	46
hrcA	AB2184	Putative heat shock regulator	28	27	31	28	31	30	31	28	--	31	--	36	34	34	31
hslU	AB0975	Heat shock protein HslVU, ATP-dependent protease subunit HslU	65	65	64	65	65	65	66	62	61	66	65	67	63	62	63
hslV	AB0974	Heat shock protein HslVU, ATP-dependent protease subunit HslV	77	77	78	73	75	78	78	73	78	81	85	77	71	74	71
htpG	AB1552	Heat shock protein 90 HtpG	52	53	55	55	53	54	54	53	66	49	51	56	53	52	52
sugE	AB1053	Suppresses groEL, may be chaperone	37	35	36	36	36	34	34	--	--	--	--	--	--	37	--
	AB1036	DnaJ domain protein	--	--	--	--	--	--	--	--	--	--	--	--	--	--	--
	AB1037	DnaJ domain protein	--	--	--	--	--	--	--	--	39	42	42	--	--	--	--
	AB1259	DnaJ domain protein	30	30	--	31	34	38	35	37	--	--	39	36	--	25	25
	AB1732	Conserved hypothetical protein, putatve DnaJ domain protein	--	--	--	--	--	--	--	--	--	--	--	--	--	--	--
	AB2274	Heat shock protein Hsp20	--	--	--	--	--	--	--	47	59	48	60	53	--	--	--

**IV.C Cell division**			**Cj**	**Cc**	**Cl**	**Cu**	**Cf**	**Cv**	**Cn**	**Ch**	**Sv**	**Ni**	**Sd**	**Ws**	**Ha**	**Hh**	**Hp**
ftsA	AB1386	Cell division protein FtsA	36	35	33	35	36	40	41	34	40	37	36	43	32	38	32
ftsE	AB1003	Cell division ATP-binding protein FtsE	53	55	54	55	49	52	52	53	52	55	58	60	51	60	52
ftsH1	AB0452	Cell division protein FtsH	67	68	69	68	66	66	67	68	63	66	65	69	66	67	66
ftsH2	AB1135	Cell division protein FtsH	46	47	47	42	46	--	45	--	44	--	42	46	--	47	--
ftsK	AB1167	Cell division protein FtsK	--	--	--	--	59	59	59	57	55	60	58	--	--	--	--
ftsW	AB1910	Cell division protein FtsW	41	41	42	42	44	44	44	42	41	44	43	44	41	40	40
ftsX	AB1002	Cell division protein FtsX	29	31	26	28	27	25	28	29	--	29	26	27	27	27	28
ftsY	AB0888	Signal recognition particle-docking GTPase FtsY	57	58	56	57	56	61	59	58	58	63	59	55	49	57	49
ftsZ	AB1385	Cell division protein FtsZ	56	56	52	57	56	55	55	54	57	53	58	57	46	51	45
parA	AB1612	ATPases involved in chromosome partitioning ParA	66	66	65	66	66	67	69	63	69	73	73	71	58	66	58
parB	AB1611	Transcriptional regulator involved in chromosome partitioning ParB	49	47	47	47	50	48	49	50	52	49	51	50	52	57	52
tig	AB1715	Trigger factor	45	46	48	43	43	46	49	41	55	48	51	45	41	44	42
tolB	AB1599	Colicin tolerance-like protein (tolB)	41	39	40	38	44	43	42	44	41	44	39	45	41	40	40
	AB2128	Septum formation protein Maf homolog	37	38	42	39	45	46	50	46	53	55	60	54	39	--	40

**IV.D Chemotaxis and mobility**			**Cj**	**Cc**	**Cl**	**Cu**	**Cf**	**Cv**	**Cn**	**Ch**	**Sv**	**Ni**	**Sd**	**Ws**	**Ha**	**Hh**	**Hp**
cheA	AB1193	Chemotaxis protein CheA	31	31	31	31	31	30	31	--	--	33	31	31	34	32	33
cheB	AB1196	Protein-glutamate methylesterase CheB	--	--	--	--	--	--	--	--	--	34	38	--	--	--	--
cheD	AB1195	Chemotaxis protein CheD	--	--	--	--	--	--	--	--	--	--	--	--	--	--	--
cheR	AB1194	Chemotaxis protein methyltransferase	27	25	26	--	29	26	26	--	--	29	31	50	--	48	--
cheV	AB0617	Chemotaxis signal transduction protein CheV	26	26	25	--	--	26	27	--	--	26	28	29	29	27	26
cheW	AB0425	Chemotaxis protein CheW	26	26	--	25	30	26	27	--	--	--	34	30	--	--	--
cheY1	AB1192	Chemotaxis protein CheY	37	37	36	33	--	36	33	30	32	40	36	38	38	37	38
cheY2	AB1960	Chemotaxis protein CheY	66	65	66	63	--	66	66	30	27	51	61	61	66	63	65
motA	AB0398	Flagellar motor component MotA	55	55	56	55	56	52	--	--	--	29	27	26	54	51	54
motB	AB0397	Flagellar motor component MotB	37	38	37	35	37	35	--	--	--	26	28	27	35	38	35
	AB0176	Methyl-accepting chemotaxis protein	--	--	--	--	27	--	--	--	--	--	--	29	--	--	29
	AB0321	Methyl-accepting chemotaxis protein	--	--	--	29	--	--	27	--	--	--	--	28	--	--	--
	AB0349	Methyl-accepting chemotaxis protein	43	43	38	38	--	--	--	--	51	63	59	44	--	--	--
	AB0415	Methyl-accepting chemotaxis protein	--	27	31	28	30	--	--	--	--	--	44	25	32	33	32
	AB0523	Methyl-accepting chemotaxis protein	--	--	--	--	--	--	--	--	--	--	32	--	--	--	--
	AB0525	Methyl-accepting chemotaxis protein	--	28	--	--	29	--	--	--	--	--	29	33	--	27	--

	AB0602	Methyl-accepting chemotaxis protein	38	--	39	--	38	--	--	--	--	--	--	--	--	--	--
	AB0622	Methyl-accepting chemotaxis protein	32	--	--	--	30	--	--	--	--	--	--	32	--	--	--
	AB0627	Methyl-accepting chemotaxis protein	--	--	26	--	27	--	--	--	--	--	27	--	--	--	--
	AB0632	Methyl-accepting chemotaxis protein	27	--	--	--	28	25	29	--	--	--	27	--	--	--	--
	AB0633	Methyl-accepting chemotaxis protein	--	--	--	27	28	--	--	--	--	--	28	33	--	--	--
	AB0746	Methyl-accepting chemotaxis protein	--	--	--	--	--	--	--	--	--	--	--	--	--	--	--
	AB0780	CheW-like chemotaxis protein	--	--	--	--	--	--	--	--	--	--	--	33	--	--	--
	AB0788	Methyl-accepting chemotaxis protein	28	28	--	25	--	26	26	--	--	--	--	33	--	26	26
	AB0791	Methyl-accepting chemotaxis protein	30	29	28	27	27	28	28	--	--	--	--	32	26	27	27
	AB0792	Methyl-accepting chemotaxis protein	--	--	--	29	27	--	--	--	--	--	--	29	--	29	--
	AB0793	Methyl-accepting chemotaxis protein	29	--	--	--	29	--	29	--	--	--	--	31	--	--	--
	AB0794	Methyl-accepting chemotaxis protein	--	--	25	28	28	--	28	--	--	--	--	37	--	--	--
	AB0844	Methyl-accepting chemotaxis protein	--	29	--	--	--	--	--	--	--	--	--	30	--	--	--
	AB0845	Methyl-accepting chemotaxis protein	45	45	44	46	44	48	42	--	55	44	65	45	--	--	--
	AB0903	Putative methyl-accepting chemotaxis protein	--	--	--	--	--	28	27	--	--	--	--	32	--	--	--
	AB0962	Methyl-accepting chemotaxis protein	42	42	44	45	--	46	41	--	44	41	66	43	--	--	--
	AB1070	Methyl-accepting chemotaxis protein	--	--	--	--	28	--	--	--	--	--	--	29	--	--	--
	AB1314	Methyl-accepting chemotaxis protein	29	--	30	27	--	27	26	--	--	--	--	--	--	27	--
	AB1364	Response regulator receiver domain protein, CheY-like	30	31	30	30	26	28	28	26	34	34	50	26	29	26	27
	AB1365	Inhibitor of MCP methylation, CheC	--	--	--	--	--	--	--	--	--	--	36	--	--	--	--
	AB1418	Methyl-accepting chemotaxis protein	--	--	--	--	30	--	--	--	--	--	--	31	--	--	--
	AB1496	Methyl-accepting chemotaxis protein	28	27	--	--	26	--	27	--	--	29	31	33	--	28	26
	AB1740	Methyl-accepting chemotaxis protein	--	--	--	27	28	--	--	--	--	--	--	33	--	--	--
	AB1743	Methyl-accepting chemotaxis protein	28	--	--	--	27	--	--	--	--	--	28	--	--	--	--
	AB1764	Methyl-accepting chemotaxis protein	31	30	31	31	31	28	30	--	--	--	--	26	26	26	26
	AB1841	Methyl-accepting chemotaxis protein	28	28	29	28	27	26	29	--	--	--	--	29	--	27	--
	AB1842	Methyl-accepting chemotaxis protein	--	--	--	--	29	--	--	--	--	--	--	32	--	--	--
	AB1923	Methyl-accepting chemotaxis protein	--	--	--	--	--	--	--	--	--	--	--	--	--	--	--
	AB1977	Methyl-accepting chemotaxis protein	--	--	--	--	--	--	--	--	--	--	--	--	--	--	--
	AB2276	Methyl-accepting chemotaxis protein	28	--	28	27	28	26	27	--	--	--	25	31	26	29	25

IV.E Protein and peptide secretion			Cj	Cc	Cl	Cu	Cf	Cv	Cn	Ch	Sv	Ni	Sd	Ws	Ha	Hh	Hp
appA	AB0099	Oligopeptide ABC transporter, periplasmic substrate-binding protein	31	32	33	32	32	--	30	--	26	56	56	26	26	--	26
ffh	AB1967	Signal recognition particle protein	62	60	62	60	61	63	64	62	65	65	64	61	57	54	56
gspD	AB1844	General secretion pathway protein D	--	25	--	--	--	--	27	--	--	--	--	--	--	--	--
lepP	AB0637	Signal peptidase I	56	55	56	55	52	59	49	55	--	57	56	55	48	--	50
lolA	AB1316	Outer membrane lipoprotein carrier protein LolA	32	33	34	31	37	30	32	33	30	34	35	32	31	30	31
lspA	AB0083	Lipoprotein signal peptidase	44	42	47	43	47	46	44	46	43	44	48	50	39	45	38
oxaA	AB1632	Inner membrane protein, 60 kDa	50	49	47	49	53	50	50	47	45	52	45	48	43	44	43
secA	AB1317	Protein translocase, SecA subunit	64	65	65	66	65	66	66	66	63	66	65	66	64	65	64
secD	AB0393	Protein-export membrane protein SecD	57	56	60	57	62	61	63	59	59	64	65	59	55	50	54
secE	AB1894	Preprotein translocase, SecE subunit	61	63	66	66	53	68	63	59	51	58	57	63	53	58	54
secF	AB0394	Protein-export membrane protein SecF	54	54	53	52	53	52	52	50	57	56	52	48	47	50	46
secG	AB2260	Protein-export membrane protein SecG	50	53	53	53	58	62	62	50	62	53	58	62	--	--	--
secY	AB0774	Preprotein translocase, SecY subunit	74	76	79	75	80	79	79	73	71	79	75	76	70	70	70
tatC	AB1262	Sec-independent protein secretion pathway component TatC	56	56	59	59	65	63	64	60	--	69	56	65	54	52	54

yajC	AB0392	Preprotein translocase subunit	62	60	60	57	62	58	64	59	60	61	53	64	53	49	--
	AB0213	Oligopeptide ABC transporter, permease protein	29	29	29	30	32	30	28	--	28	65	62	31	29	30	28
	AB0546	Oligopeptide ABC transporter, ATP-binding protein	32	31	29	34	34	36	31	31	33	61	53	32	33	31	29
	AB0709	HlyD family secretion protein	26	26	26	28	--	27	27	26	--	29	42	--	--	36	--
	AB1128	HlyD family secretion protein	--	--	--	--	29	42	43	29	26	26	33	--	--	--	--
	AB1369	HlyD-family secretion protein	--	--	25	--	49	--	--	45	--	--	--	--	--	--	--
	AB2114	Twin-arginine translocation protein, TatA/E family	--	--	58	--	43	52	54	52	56	62	55	55	56	64	57
	AB2115	Twin-arginine translocation protein, TatA/E family	48	--	51	49	46	55	45	--	50	68	50	56	55	53	51

**IV.G Detoxification**			**Cj**	**Cc**	**Cl**	**Cu**	**Cf**	**Cv**	**Cn**	**Ch**	**Sv**	**Ni**	**Sd**	**Ws**	**Ha**	**Hh**	**Hp**
ahpC	AB1883	Alkyl hydroperoxide reductase/ Thiol specific antioxidant	73	74	76	74	71	75	74	67	81	75	78	77	66	73	67
bcp	AB1849	Bacterioferritin comigratory protein, alkyl hydroperoxide reductase/thiol specific antioxidant	52	53	53	54	--	53	51	47	62	54	60	52	47	46	49
katG	AB1553	Catalase/peroxidase HPI	--	--	--	--	--	--	--	--	--	--	--	--	--	--	--
sodB	AB1585	Superoxide dismutase	55	55	56	57	52	50	51	45	--	--	47	58	54	58	53
tpx	AB0554	Thiol peroxidase	58	56	54	56	56	64	55	58	61	61	58	64	--	57	43
tsaA	AB0286	Alkyl hydroperoxide reductase/ Thiol specific antioxidant	73	74	76	74	71	75	74	67	81	75	78	77	66	73	67
	AB1062	Flavodoxin-like fold domain protein, putative NADPH-quinone reductase	43	44	42	38	46	27	44	--	--	--	--	--	46	39	45
	AB1072	Glutathionylspermidine synthase family protein	65	66	67	63	65	66	69	--	68	--	62	61	59	60	59
	AB2141	DNA-binding ferritin-like protein (Dps/NapA)	53	52	49	49	44	51	51	46	--	--	--	55	51	55	51

**IV.I Pathogenicity**			**Cj**	**Cc**	**Cl**	**Cu**	**Cf**	**Cv**	**Cn**	**Ch**	**Sv**	**Ni**	**Sd**	**Ws**	**Ha**	**Hh**	**Hp**
cadF	AB0483	Outer membrane fibronectin-binding protein	39	38	39	38	42	42	42	40	--	--	40	41	--	--	--
ciaB	AB1555	CiaB protein	39	37	40	39	40	39	39	38	46	47	--	44	--	38	--
gcp	AB0167	O-sialoglycoprotein endopeptidase	56	55	55	51	54	54	55	56	60	64	66	59	57	51	56
irgA	AB0729	Iron-regulated outer membrane virulence protein homolog	30	31	30	--	30	37	34	--	--	--	25	25	--	34	--
mviN	AB0876	Virulence factor MviN protein	45	45	44	45	53	56	54	53	49	56	57	53	50	50	49
pldA	AB0859	Outer membrane phospholipase A	37	35	--	36	39	41	39	39	--	--	--	--	--	--	--
tlyA	AB1846	Hemolysin A	49	46	51	49	52	46	51	46	47	53	49	42	44	40	44
traT	AB0162	TraT complement resistance protein precursor	--	--	--	52	60	--	--	--	--	--	27	55	--	--	--
	AB0070	Fibronectin/fibrinogen-binding protein, putative	33	34	37	36	40	38	39	37	37	40	45	34	30	30	29
	AB0940	Hemolysin activation protein HecB, putative	--	--	--	--	25	--	26	--	--	--	--	31	--	--	--
	AB0941	Adhesin/haemagglutinin, HecA family	--	--	--	--	--	--	--	--	--	--	--	--	--	--	--
	AB1451	VacJ-like lipoprotein	45	46	37	43	47	54	54	45	--	--	50	--	--	--	--

**IV.J DNA uptake/competence**			**Cj**	**Cc**	**Cl**	**Cu**	**Cf**	**Cv**	**Cn**	**Ch**	**Sv**	**Ni**	**Sd**	**Ws**	**Ha**	**Hh**	**Hp**
comE	AB1266	Competence locus E	38	36	39	38	32	34	32	33	33	34	34	32	31	31	33
ctsE	AB1654	Campylobacter transformation system protein CtsE	--	--	--	--	--	--	--	--	--	--	--	--	--	--	--
ctsF	AB1653	Campylobacter transformation system protein CtsF	--	28	--	27	28	26	25	--	--	31	27	26	--	25	--
ctsW	AB0476	Transformation system protein	44	43	44	45	46	44	47	38	39	43	44	36	40	37	39
dprA	AB0232	SMF protein, DNA processing chain A	45	43	44	42	42	41	43	40	52	54	56	42	43	43	43
	AB0512	CinA-like protein	--	--	--	--	--	--	--	--	51	--	--	--	--	39	--
	AB0784	Molybdopterin binding domain protein	--	--	--	--	--	--	--	--	46	57	50	53	--	--	--
	AB2267	HHH domain protein, putative competence protein ComEA	--	--	--	--	39	37	34	--	37	--	--	--	--	--	--

V. Other

V.A Phage-related functions and prophage

			Cj	Cc	Cl	Cu	Cf	Cv	Cn	Ch	Sv	Ni	Sd	Ws	Ha	Hh	Hp
int	AB2090	Phage integrase	26	--	26	--	26	27	29	32	31	31	--	--	--	--	--
	AB1330	Putative phage integrase	32	--	33	--	28	39	32	31	41	31	--	--	--	--	--
	AB1530	Phage-related lysozyme	--	--	--	--	--	--	--	--	--	--	--	--	--	--	--
	AB1655	Hypothetical protein	--	--	--	--	--	--	--	--	--	--	--	--	--	--	--
	AB1656	Conserved hypothetical protein	43	--	40	--	--	--	--	--	40	42	35	42	--	--	--
	AB1657	Conserved hypothetical protein	--	--	--	--	--	--	--	--	--	--	--	--	--	--	--
	AB1658	Hypothetical protein	--	--	--	--	--	--	--	--	--	--	--	--	--	--	--
	AB1659	N6 adenine-specific DNA methyltransferase, D12 class	58	--	--	29	--	25	--	--	--	--	--	--	30	26	--
	AB1660	Phage tail protein D	33	--	--	--	--	--	--	--	--	--	--	--	--	--	--
	AB1661	Hypothetical protein	43	--	--	--	--	--	--	--	--	--	--	--	--	--	--
	AB1662	Hypothetical protein	29	--	--	--	--	--	--	--	--	--	--	--	--	--	--
	AB1663	Phage tail tape measure protein, TP901 family, putative	31	--	--	--	--	--	--	--	--	--	--	--	--	--	--
	AB1664	Hypothetical protein	--	--	--	--	--	--	--	--	--	--	--	--	--	--	--
	AB1665	Phage major tail tube protein	31	--	--	--	--	--	--	--	--	--	--	--	--	--	--
	AB1666	Phage tail sheath protein	26	--	--	--	--	--	--	--	--	--	--	--	--	--	--
	AB1667	Hypothetical protein	--	--	--	--	--	--	--	--	--	--	--	--	--	--	--
	AB1668	Tail fiber assembly protein	--	--	--	--	--	--	--	--	--	--	--	--	--	--	--
	AB1669	Hypothetical protein	--	--	--	--	--	--	--	--	--	--	--	--	--	--	--
	AB1670	Tail protein I, putative	33	--	--	--	--	--	--	--	--	--	--	--	--	--	--
	AB1671	Baseplate assembly protein J, putative	29	--	--	--	--	--	--	--	--	--	--	--	--	--	--
	AB1672	Hypothetical protein, putative baseplate assembly protein W	--	--	--	--	--	--	--	--	--	--	--	--	--	--	--
	AB1673	Baseplate assembly protein V, putative	38	--	--	--	--	--	--	--	--	--	--	--	--	--	--
	AB1674	Hypothetical protein	28	--	--	--	--	--	--	--	--	--	--	--	--	--	--
	AB1675	Hypothetical protein	40	--	--	--	--	42	37	45	--	--	--	--	33	--	--
	AB1676	Hypothetical protein	36	--	--	--	--	--	--	36	--	--	--	--	--	--	--
	AB1677	Hypothetical protein	26	--	--	--	--	--	--	--	--	--	--	--	--	--	--
	AB1678	Mu-like prophage I protein, putative	36	--	--	--	--	--	--	29	--	--	--	--	--	--	--
	AB1679	Hypothetical protein	--	--	--	--	--	--	--	--	--	--	--	--	--	--	--
	AB1680	Conserved hypothetical protein	31	--	--	--	--	--	--	--	--	--	--	--	--	--	--
	AB1681	Conserved hypothetical protein	--	--	--	--	--	--	--	--	--	--	--	--	--	--	--
	AB1682	Conserved hypothetical protein	33	--	--	--	--	--	--	--	--	--	--	--	--	--	--
	AB1683	Phage uncharacterized protein	45	--	--	--	--	--	--	28	--	--	--	--	--	--	--
	AB1684	Phage uncharacterized protein	35	--	--	--	--	--	--	--	--	--	--	--	--	--	--
	AB1685	Mu-like prophage F protein, putative	31	--	--	--	--	--	--	--	--	--	--	--	--	--	--
	AB1686	Mu-like prophage G protein, putative	29	--	--	--	--	--	--	27	--	--	--	--	--	--	--
	AB1687	Conserved hypothetical protein	41	--	--	--	--	--	--	--	--	--	--	--	--	--	--
	AB1688	Hypothetical protein	--	--	--	--	--	--	--	--	--	--	--	--	--	--	--
	AB1690	Conserved hypothetical protein	--	--	--	--	--	--	--	--	--	--	--	--	--	--	--
	AB1692	DnaB-like helicase-like protein	35	--	--	--	--	--	--	--	--	--	--	--	--	--	--
	AB1693	Hypothetical protein	--	--	--	--	--	--	--	--	--	--	--	--	--	--	--
	AB1694	Hypothetical protein	--	--	--	--	--	--	--	--	--	--	--	--	--	--	--
	AB1696	Hypothetical protein	--	--	--	--	--	50	--	44	--	42	--	--	--	--	--
	AB1697	Phage uncharacterized protein	--	--	--	--	--	--	--	--	--	--	--	--	--	--	--
	AB1698	Hypothetical protein	--	--	--	--	--	--	--	--	--	--	--	--	--	--	--
	AB1700	Hypothetical protein	--	--	--	--	--	--	--	--	--	--	--	--	--	--	--

	AB1701	Hypothetical protein	--	--	--	--	--	--	--	--	--	--	--	--	--	--	--
	AB1702	Prophage Mu, DNA transposition protein B	--	--	--	--	--	--	--	--	--	--	--	--	--	--	--
	AB1703	Prophage Mu, DNA transposition protein A	26	--	--	--	--	--	--	--	--	--	--	--	--	--	--
	AB1704	Hypothetical protein	--	--	--	--	--	--	--	--	--	--	--	--	--	--	--
	AB1705	Phage repressor protein, putative	26	--	--	--	--	--	--	33	--	--	27	--	--	28	--
	AB1706	Hypothetical protein	--	--	--	--	--	--	--	--	--	--	--	--	--	--	--
	AB1707	Hypothetical protein	--	--	--	--	--	--	--	--	--	--	--	--	--	--	--
	AB1708	Hypothetical protein	--	--	--	--	--	--	--	--	--	--	--	--	--	--	--
	AB1721	Site-specific recombinase, phage integrase family	--	--	--	--	--	37	--	--	42	49	38	--	--	--	--

V.B Colicin-related functions

			Cj	Cc	Cl	Cu	Cf	Cv	Cn	Ch	Sv	Ni	Sd	Ws	Ha	Hh	Hp
	AB0656	Colicin V production protein	40	38	38	32	33	36	37	31	--	39	40	38	29	27	28

V.D Drug/analog sensitivity and antibiotic resistance

			Cj	Cc	Cl	Cu	Cf	Cv	Cn	Ch	Sv	Ni	Sd	Ws	Ha	Hh	Hp
arsB	AB1924	Arsenical pump membrane protein	39	43	--	39	63	--	--	--	66	65	73	61	--	37	--
arsC	AB1928	Arsenate reductase	33	33	--	--	--	--	--	--	71	61	63	64	--	--	--
cat	AB0785	Chloramphenicol O-acetyltransferase	--	--	--	--	--	--	--	--	--	--	--	--	--	--	--
fsr	AB1198	Fosmidomycin resistance protein	26	--	25	--	--	25	29	--	--	--	--	--	--	--	--
lrgA	AB0179	LrgA family protein	--	--	--	--	--	--	--	--	--	--	--	--	--	--	--
lrgB	AB0180	LrgB-like protein	--	--	--	--	--	--	--	--	--	--	--	--	--	--	--
uppP	AB1907	Undecaprenyl diphosphatase, putative	48	50	49	47	47	32	31	46	61	--	63	50	--	--	--
	AB0460	Outer membrane component of efflux system	38	38	37	38	--	39	36	--	45	25	45	48	43	44	43
	AB0462	Outer membrane component of efflux system	--	--	27	27	--	--	--	--	26	--	--	29	27	27	28
	AB0578	Conserved hypothetical protein, beta-lactamase-like protein	--	--	--	--	--	--	--	--	49	34	52	--	--	--	--
	AB0816	Hydrophobe/amphiphile efflux-1 family protein	49	49	49	47	49	50	50	47	42	--	66	55	27	52	27
	AB0817	Multidrug efflux RND membrane fusion protein	29	32	32	35	30	30	28	34	27	26	43	32	--	32	--
	AB0818	Outer membrane efflux protein	--	--	28	28	--	26	27	--	30	--	40	--	--	26	26
	AB0992	Putative antibiotic resistance protein	--	--	--	--	--	27	--	29	42	--	--	44	--	38	--
	AB0993	AcrB/AcrD/AcrF family protein	--	--	--	--	--	--	--	--	49	48	49	--	--	--	--
	AB0994	AcrB/AcrD/AcrF family protein	--	--	--	--	--	--	--	--	50	51	55	--	--	--	--
	AB0995	AcrA/AcrE family protein	--	--	26	--	--	--	27	--	42	42	41	--	--	--	--
	AB0996	Outer membrane efflux protein	--	--	--	--	--	--	--	26	--	26	28	--	--	--	--
	AB1031	Outer membrane efflux protein, putative	--	--	--	--	--	--	--	--	--	--	--	--	--	--	--
	AB1306	Beta-lactamase-like protein	51	53	49	51	47	43	46	40	55	55	55	49	47	41	46
	AB1391	Arsenate reductase	--	--	--	--	--	--	--	--	59	--	53	30	--	--	--
	AB1486	Beta-lactamase, putative	38	--	38	--	--	--	--	--	--	--	37	--	--	--	--
	AB1872	AcrB/AcrD/AcrF family protein	--	--	--	--	--	--	--	--	41	38	42	41	--	--	--
	AB2147	Multidrug efflux protein, HlyD family	30	31	27	30	30	30	28	27	27	--	29	31	--	31	--
	AB2148	Multidrug efflux protein, AcrB/AcrD/AcrF family	39	39	41	40	39	40	39	39	37	--	41	44	28	39	28
	AB2211	Outer membrane efflux lipoprotein	28	27	29	27	29	28	30	29	27	--	--	34	--	--	--
	AB2212	Multidrug efflux protein, Acr family	39	39	38	38	38	36	37	37	38	--	40	43	28	39	27
	AB2213	Multidrug efflux protein, HlyD family	28	27	26	29	28	30	29	29	25	--	29	28	--	28	--

V.F Adaptations and atypical conditions

			Cj	Cc	Cl	Cu	Cf	Cv	Cn	Ch	Sv	Ni	Sd	Ws	Ha	Hh	Hp
cspA	AB1875	Cold-shock protein, DNA-binding	--	--	--	--	--	--	--	--	59	--	55	--	--	--	--
cti	AB0257	Fatty acid cis/trans isomerase	--	--	--	--	--	--	--	--	--	--	--	--	--	--	--

luxS	AB0115	Autoinducer-2 production protein LuxS	78	78	--	78	69	68	64	70	69	71	70	66	40	64	41
nudH	AB1206	(Di)nucleoside polyphosphate hydrolase	66	62	59	57	59	58	59	55	57	69	56	--	53	56	55
pfs	AB1594	5'-methylthioadenosine\S-adenosylhomocysteine nucleosidase	57	57	56	56	52	59	58	52	65	60	64	62	50	54	49
rbfA	AB2033	Ribosome binding factor A	46	46	43	41	44	44	44	36	42	45	40	46	39	46	40
rbpA	AB0061	RNA-binding region RNP-1 (RNA recognition motif)	--	--	--	--	62	--	--	--	--	58	--	--	46	56	45
surE	AB2252	Stationary-phase survival protein SurE	48	48	50	48	48	48	48	45	61	55	52	58	48	58	48
uspA	AB1293	Universal stress protein	--	--	43	--	--	--	--	30	--	--	--	--	--	--	--

VI. Miscellaenous proteins, general function proteins, and hypothetical proteins

VI.A Miscellaneous/General function

			Cj	Cc	Cl	Cu	Cf	Cv	Cn	Ch	Sv	Ni	Sd	Ws	Ha	Hh	Hp
engA	AB1183	GTP-binding protein	52	50	52	49	53	50	51	51	50	54	57	56	49	47	49
hicA	AB1574	Hif-contiguous protein A	--	--	--	--	--	--	--	--	--	--	--	--	--	--	--
hicB	AB1575	Hif-contiguous protein B	--	--	--	--	--	--	--	--	--	--	--	--	--	--	--
lemA	AB2137	LemA protein	--	--	--	--	--	31	54	37	36	--	--	--	--	--	--
mloA	AB1076	Conserved hypothetical protein, MloA homolog	57	58	--	--	--	--	--	51	25	--	--	--	--	55	--
mraW	AB1327	S-adenosyl-methyltransferase	54	53	50	54	50	55	55	54	54	57	59	55	52	51	51
phnA	AB0263	Phosphonoacetate hydrolase	--	--	--	--	--	--	--	--	--	--	61	--	--	--	--
pirA	AB0143	Pirin	--	--	--	--	--	--	--	--	--	--	--	--	--	--	--
pqiB	AB1558	Paraquat-inducible protein B	--	--	--	--	--	--	--	--	--	--	--	--	--	--	--
rpe	AB0858	Ribulose-phosphate 3-epimerase	61	61	62	58	69	66	68	67	77	72	76	69	60	62	61
tas	AB0521	Oxidoreductase Tas, aldo/keto reductase family	--	--	--	--	--	32	--	--	66	73	70	--	--	--	29
	AB0027	Zinc-containing alcohol dehydrogenase superfamily protein	--	--	--	--	--	--	--	--	--	--	--	--	--	--	--
	AB0038	Fe-S oxidoreductase	37	39	35	35	37	--	--	--	--	--	--	--	35	34	35
	AB0039	dCMP deaminase, putative	--	--	--	--	--	--	--	--	--	--	82	--	--	--	--
	AB0048	PhoH family protein	--	--	--	--	--	--	--	--	--	--	--	27	--	28	--
	AB0050	ATPase, AAA family protein	54	54	53	53	54	57	54	51	56	59	58	58	55	57	54
	AB0052	Carbohydrate isomerase, KpsF/GutQ family	49	50	51	48	59	59	62	65	63	64	62	60	53	57	53
	AB0053	Conserved hypothetical protein, predicted hydrolase of the metallo-beta-lactamase superfamily	59	61	62	62	63	62	65	59	57	67	63	68	58	62	61
	AB0057	Conserved hypothetical protein, possible purine nucleoside phosphorylase	50	51	45	49	46	--	36	46	59	61	66	49	50	43	50
	AB0058	Conserved hypothetical protein, radical SAM enzyme, Cfr family	60	60	60	--	60	60	61	60	65	63	65	64	59	60	59
	AB0078	Rhodanese-like sulfur transferase	--	--	--	--	--	--	--	--	--	--	--	39	--	--	--
	AB0080	Conserved hypothetical protein, putative sulfide:quinone reductase	--	--	--	--	--	--	--	--	60	40	52	47	--	--	--
	AB0100	Conserved hypothetical protein, HAD-superfamily hydrolase	44	44	47	47	49	45	44	43	48	41	54	47	--	--	--
	AB0120	Putative membrane protein, DoxX family	--	--	--	--	34	38	32	42	--	--	55	41	--	45	--
	AB0123	Conserved hypothetical protein, Band 7 family protein	54	53	53	54	56	56	52	54	52	56	52	59	52	47	52
	AB0138	Putative aminotransferase	--	--	--	--	--	--	--	--	64	59	63	67	49	58	49
	AB0142	Conserved hypothetical protein, LigB family protein	--	--	--	--	--	--	--	--	--	--	--	--	--	--	--
	AB0169	Conserved hypothetical protein, putative carbohydrate kinase	39	40	43	41	42	45	46	39	46	51	53	46	46	43	46
	AB0224	Conserved hypothetical DNA binding protein	--	--	--	--	--	--	--	--	--	--	--	--	--	--	--
	AB0260	Isochorismatase hydrolase	--	--	--	--	--	--	--	--	29	--	--	--	--	--	--
	AB0262	Conserved hypothetical protein, putative para-aminobenzoate synthase component I	--	--	--	--	45	42	--	--	40	41	48	36	--	--	--
	AB0264	Conserved hypothetical protein, predicted metal-dependent hydrolase	--	--	--	--	--	--	--	--	--	--	59	--	--	--	--

AB0278	Isochorismatase hydrolase	--	--	--	--	33	--	--	--	--	--	--	37	--	34	--
AB0294	Conserved hypothetical protein, predicted ATP/GTP-binding protein	27	29	26	27	27	29	28	27	--	--	--	35	--	26	--
AB0295	Conserved hypothetical protein, predicted ATP/GTP-binding protein	31	30	30	29	28	27	28	28	--	--	--	32	--	30	--
AB0313	Aminotransferase/L-cysteine desulfhydrase	28	27	26	--	--	36	37	28	39	29	41	35	26	27	26
AB0314	Carboxylyase-related protein	55	55	56	52	58	56	58	56	61	62	60	57	52	55	52
AB0371	CoA-binding domain protein	43	48	50	--	45	53	--	--	70	66	69	49	--	41	--
AB0404	Phospholipid/glycerol acyltransferase, possible hemolysin	--	--	--	--	--	--	--	--	--	--	--	47	--	--	--
AB0409	Putative acetyltransferase	--	--	--	--	--	--	--	--	32	--	--	--	--	--	--
AB0434	Ser/Thr protein phosphatase family protein	--	--	--	--	--	--	--	--	--	--	--	--	--	--	--
AB0461	Putative membrane fusion component of efflux system	30	29	33	32	--	35	34	--	35	--	34	35	36	38	37
AB0491	Amidohydrolase family protein	41	40	40	39	42	40	37	41	39	47	41	43	35	39	36
AB0579	Conserved hypothetical protein, HD domain protein	--	--	--	--	--	--	--	--	--	--	--	--	--	--	--
AB0580	Rhodanese-like protein	--	--	--	--	--	--	--	--	41	--	--	--	--	--	--
AB0586	HAM1 protein homolog	45	48	46	43	51	47	51	47	56	59	55	48	--	37	--
AB0596	Hypothetical TPR repeat protein	--	--	--	--	--	--	--	--	--	--	--	--	--	--	--
AB0666	FAD-dependent oxidoreductase	--	--	26	--	--	--	--	--	--	--	--	--	--	--	--
AB0728	FMN-dependent alpha-hydroxy acid dehydrogenase	--	--	--	--	--	30	--	--	--	--	--	--	--	--	--
AB0730	Conserved hypothetical protein, possible IroE protein	--	28	--	--	--	--	--	--	--	--	--	--	--	--	--
AB0731	Putative SAM-dependent methyltransferase	--	--	--	--	--	--	--	--	--	--	--	--	--	--	--
AB0736	GTP-binding protein, putative	69	70	67	68	70	71	70	69	71	76	70	69	64	66	64
AB0751	HIT family protein	53	53	56	55	50	57	54	53	--	56	51	46	44	44	43
AB0781	Radical SAM domain protein	--	--	--	--	--	--	--	--	57	--	--	--	--	--	--
AB0786	Zinc-containing NADP-dependent alcohol dehydrogenase	44	44	44	43	--	--	--	--	--	--	--	--	43	44	44
AB0787	Iron-containing NADP-dependent alcohol dehydrogenase	--	--	--	--	61	63	58	--	--	--	--	42	--	--	--
AB0798	MutT/nudix family protein	--	--	--	--	--	--	--	--	69	58	--	--	--	--	--
AB0836	Carotenoid isomerase, putative	--	--	--	--	--	--	--	--	--	--	40	--	--	--	--
AB0846	Probable tautomerase	33	54	35	--	50	51	41	--	--	--	--	52	55	--	44
AB0871	Oxidoreductase, short chain dehydrogenase/reductase family	74	75	74	73	71	74	74	72	73	71	69	67	70	70	70
AB0890	Putative 5-formyltetrahydrofolate cyclo-ligase	47	--	45	--	40	36	35	38	47	40	48	40	--	--	--
AB0891	HD/HDIG/KH domain protein	59	57	60	60	59	58	61	60	57	61	55	60	42	52	46
AB0898	Oxidoreductase, short-chain dehydrogenase/reductase family	27	26	--	--	--	26	--	--	53	49	--	--	32	--	30
AB0908	GatB/YqeY family protein	48	47	47	--	43	45	39	43	51	58	48	52	--	--	--
AB0983	OstA family protein	36	39	37	40	37	42	47	36	35	35	34	32	36	35	37
AB0984	Putative ATP /GTP binding protein	54	53	53	56	54	49	51	44	54	53	54	47	49	49	47
AB0985	Acetyltransferase, GNAT Family	--	--	--	--	53	50	--	52	55	54	52	51	--	54	--
AB0991	FMN-binding protein	--	--	--	--	--	--	--	--	--	--	--	--	--	--	--
AB1005	Fibronectin type III domain protein	34	34	35	35	39	--	--	38	--	41	34	36	31	31	31
AB1017	Exopolyphosphatase-related protein	--	--	--	--	--	--	--	--	--	--	--	--	--	--	--
AB1018	HIT family protein	58	58	53	60	51	52	49	58	63	61	50	--	--	--	--
AB1046	NADP-dependent alcohol dehydrogenase	--	--	--	--	--	--	--	--	--	--	--	--	--	--	--
AB1064	NADH:flavin oxidoreductase/NADH oxidase	--	--	--	--	50	55	53	--	--	--	--	--	--	--	--
AB1066	NAD(P)H-flavin nitroreductase	31	30	29	30	43	52	47	29	65	53	69	43	27	42	34
AB1069	Acyl-CoA thioester hydrolase family protein	--	--	--	--	--	--	--	--	--	--	--	61	57	60	57
AB1100	TPR repeat protein, SEL1 subfamily	--	33	30	--	--	30	--	--	--	--	--	--	29	29	--
AB1104	HAD-superfamily hydrolase	34	33	37	35	34	36	36	38	50	42	40	28	--	--	--

AB1112	Phosphohistidine phosphatase	38	33	35	32	39	37	32	38	43	30	--	--	--	--	--
AB1217	Oxidoreductase, short chain dehydrogenase/reductase family	30	32	30	30	27	26	26	--	--	--	32	26	--	26	25
AB1220	SAM domain protein	78	78	77	77	78	77	77	76	73	77	74	72	70	76	69
AB1277	Acetyltransferase	--	--	--	--	--	--	--	--	--	--	--	33	--	--	--
AB1362	D-amino acid oxidase domain protein	--	--	--	--	--	--	--	--	29	30	34	--	--	--	--
AB1400	HAD-superfamily hydrolase	--	--	--	--	--	--	--	--	--	--	--	--	--	--	--
AB1411	3-hydroxyisobutyrate dehydrogenase family protein	--	--	--	--	--	--	--	--	--	--	--	--	--	--	--
AB1420	HI0933-like protein	--	--	--	--	--	26	30	--	56	28	61	30	--	--	--
AB1448	Thioesterase/acetyltransferase, putative	--	--	--	--	--	--	--	--	--	--	--	--	--	--	--
AB1455	HAD-superfamily hydrolase	--	25	27	28	25	--	--	26	--	27	26	--	--	--	--
AB1483	Glutamine amidotransferase, class I	--	--	--	--	--	--	--	--	--	--	--	--	--	--	--
AB1485	Aldose 1-epimerase family protein, LacX	--	--	--	--	--	--	--	--	--	--	--	--	--	--	--
AB1498	HMGL family protein	--	--	--	30	--	--	--	--	29	--	--	--	--	--	--
AB1518	Transglutaminase family protein	--	--	--	--	--	45	43	--	--	--	--	--	--	--	--
AB1542	Putative FMN reductase	--	--	--	--	--	--	--	--	--	--	--	--	--	--	--
AB1543	PhoH family protein	--	--	--	--	--	--	--	--	--	--	--	29	--	29	--
AB1557	Ankyrin repeat protein	48	46	53	--	50	--	--	--	--	--	--	--	--	45	--
AB1561	Protozoan/cyanobacterial globin homolog	--	--	--	--	--	--	--	--	52	36	38	--	--	--	--
AB1568	Putative DNA-binding protein	--	--	--	--	--	--	--	--	52	--	--	--	--	--	--
AB1628	Phosphoglycerate/bisphosphoglycerate mutase, putative	34	34	28	34	--	30	30	34	33	27	--	--	--	27	--
AB1642	Fumarylacetoacetate (FAA) hydrolase	--	--	--	--	--	--	--	--	51	54	--	--	--	--	--
AB1644	Phosphohydrolase (MUTT/NUDIX family protein)	--	--	--	--	--	--	--	--	--	--	--	--	--	--	--
AB1711	Mg chelatase-related protein	52	50	52	51	52	53	52	51	53	--	54	47	52	50	52
AB1720	Saccharopine dehydrogenase (L-lysine-forming)	76	77	76	77	69	73	75	70	69	67	74	68	61	--	61
AB1728	ATPase, putative	--	--	--	--	--	--	--	--	--	--	--	--	--	--	--
AB1751	Acetyltransferase, GNAT family	--	--	--	--	34	44	40	35	--	--	--	34	--	--	--
AB1779	Conserved hypothetical protein, putative nitrilase/cyanide hydratase	--	27	--	--	37	35	31	29	43	49	42	51	--	50	27
AB1795	Radical SAM domain protein	45	45	45	45	45	45	49	48	54	49	48	46	44	41	43
AB1829	Phosphoglycerol transferase	--	--	--	--	--	--	--	--	--	--	--	--	--	28	--
AB1917	CitE domain protein	--	--	--	--	--	--	--	--	--	--	--	--	--	--	--
AB1918	AMP-dependent synthetase and ligase/CitE domain protein	--	--	--	--	--	--	--	--	--	--	--	--	--	--	--
AB1919	MaoC family protein, putative enoyl-CoA hydratase	--	--	--	--	--	--	--	--	--	--	--	--	--	--	--
AB1920	CitE domain protein	--	--	--	--	--	--	--	--	--	--	--	--	--	--	--
AB1926	Putative redox-active disulfide protein	--	--	--	--	--	--	--	--	67	65	--	75	--	--	--
AB1934	Tetratricopeptide repeat domain protein	--	--	--	--	--	--	--	--	--	--	--	--	--	--	--
AB1948	TPR repeat protein	--	--	--	--	--	--	--	--	--	--	--	--	--	--	--
AB1973	Glutaredoxin-like protein	--	--	--	--	--	--	--	--	49	49	--	--	--	--	--
AB1979	NAD(+) kinase	42	41	41	44	39	40	40	42	52	50	52	45	39	41	40
AB1983	Alpha/beta hydrolase	--	--	--	--	--	--	--	--	--	--	--	--	--	--	--
AB2009	MutT/nudix family protein	--	--	--	--	--	--	--	--	--	--	--	--	--	--	--
AB2011	Aminotransferase, classes I and II	--	--	--	--	--	--	--	--	--	--	--	43	--	25	--
AB2043	HIT family protein	49	50	59	52	58	56	59	55	64	62	55	57	50	52	50
AB2048	Radical SAM domain protein	37	38	45	42	40	35	37	38	39	41	40	--	39	--	39
AB2076	Conserved hypothetical protein, radical SAM domain protein	--	--	--	--	--	--	--	--	--	57	62	56	--	52	--
AB2135	Aldehyde dehydrogenase family protein	29	29	--	--	30	27	28	28	31	34	31	--	--	--	--
AB2154	HI0933-like protein	--	--	--	--	--	31	33	--	39	29	46	45	--	--	--

AB2170	Lipolytic enzyme, GDSL domain	--	--	--	--	--	--	--	--	--	--	--	--	--	--	--
AB2192	Ankyrin repeat protein	--	--	--	--	--	--	--	--	--	--	--	--	--	--	--
AB2239	ATP-grasp (A) domain protein	--	--	--	--	--	--	--	--	--	--	--	--	--	--	--
AB2263	Conserved hypothetical protein, RDD family	34	36	33	36	39	39	42	37	39	37	44	31	31	--	31
AB2270	Metallophosphoesterase	35	35	36	35	39	41	41	32	42	--	54	40	38	37	38
AB2278	Conserved hypothetical protein, transglutaminase-like domain	--	--	--	--	--	--	--	--	--	--	--	--	--	--	--
AB2280	Conserved hypothetical protein, transglutaminase-like domain	--	--	--	--	--	--	--	--	--	--	--	--	--	--	--
AB2289	Conserved hypothetical protein, putative succinylglutamate desuccinylase/aspartoacylase	--	--	--	--	--	--	--	--	--	--	--	--	--	--	--
AB2290	Conserved hypothetical protein, putative succinylglutamate	--	--	--	--	--	--	--	--	--	--	26	--	--	--	--
AB2295	Conserved hypothetical protein, SirA-like protein	64	64	59	66	59	--	--	42	--	--	63	--	--	69	--
AB2304	PAP2 superfamily protein	--	--	--	--	--	--	--	--	--	--	45	--	--	--	--

VI.B Domain of unknown function (DUF) proteins		Cj	Cc	Cl	Cu	Cf	Cv	Cn	Ch	Sv	Ni	Sd	Ws	Ha	Hh	Hp
AB0028	Conserved hypothetical protein (DUF24 domain protein)	42	--	--	--	--	--	--	--	--	--	--	--	--	--	--
AB0036	Conserved hypothetical protein (DUF162 domain protein)	--	--	--	--	27	--	--	--	--	--	--	--	31	--	30
AB0117	Conserved hypothetical protein (DUF752 domain protein)	--	--	--	--	--	--	--	--	46	48	49	37	--	31	--
AB0135	Conserved hypothetical protein (DUF507 domain protein)	46	48	47	42	48	54	50	44	48	46	47	45	41	44	40
AB0166	Conserved hypothetical protein (DUF1234 domain protein)	--	25	--	--	--	--	--	--	44	45	45	--	--	--	--
AB0183	Conserved hypothetical protein (DUF350 domain protein)	--	--	--	--	--	--	--	--	--	--	--	--	--	--	--
AB0236	Hypothetical protein (DUF180 domain protein)	--	--	25	--	27	31	33	--	--	--	29	28	26	--	26
AB0377	Conserved hypothetical protein (DUF779 domain protein)	--	--	--	--	--	--	--	--	--	--	--	--	--	--	--
AB0428	Conserved hypothetical protein (DUF149 domain protein)	50	48	47	47	48	46	47	52	55	48	47	53	46	55	48
AB0448	Conserved hypothetical protein (DUF163 domain protein)	45	43	41	38	48	50	49	45	48	47	50	50	42	39	41
AB0474	Conserved hypothetical protein (DUF1731 domain protein)	--	--	--	--	--	--	--	--	--	--	46	48	--	--	--
AB0480	Conserved hypothetical protein (DUF156 domain protein)	26	--	--	--	--	--	--	--	--	--	--	--	--	34	--
AB0500	Conserved hypothetical protein (DUF294 domain protein)	--	--	--	--	--	--	--	--	36	34	41	40	--	--	--
AB0502	Conserved hypothetical protein (DUF485 domain protein)	--	--	--	--	--	--	--	--	46	38	46	45	--	--	--
AB0505	Conserved hypothetical protein (DUF485 domain protein)	--	--	--	--	--	--	--	--	49	42	49	47	--	--	--
AB0534	Conserved hypothetical protein (DUF523 domain protein)	--	--	--	--	--	43	36	--	27	32	--	--	--	--	--
AB0550	Conserved hypothetical protein (DUF218 domain protein)	--	--	--	--	--	--	--	--	46	25	--	--	--	--	--
AB0606	Conserved hypothetical protein (DUF59 domain protein)	--	--	--	--	36	31	35	--	31	46	27	--	--	--	--
AB0734	Conserved hypothetical protein (DUF466 domain protein)	70	--	59	69	54	--	--	--	--	--	--	--	--	51	--
AB0740	Conserved hypothetical protein (DUF1255 domain protein)	--	--	--	--	--	--	--	--	48	--	51	49	--	--	--
AB0826	Conserved hypothetical protein (DUF81 domain protein)	--	--	--	--	25	--	--	--	--	--	--	--	--	--	--
AB0838	Conserved hypothetical protein (DUF523/1722 domain protein)	--	--	--	--	--	--	--	--	--	--	39	39	--	--	--
AB0840	Conserved hypothetical protein (DUF386 domain protein)	--	39	--	--	--	--	--	--	--	--	27	30	27	--	30
AB0862	Conserved hypothetical protein (DUF520 domain protein)	51	50	50	43	48	48	48	--	64	--	56	53	--	--	--
AB0924	Conserved hypothetical protein (DUF45 domain protein)	30	28	27	27	29	29	31	26	--	30	31	--	31	--	31
AB0964	Conserved hypothetical protein (DUF81 domain protein)	36	37	38	36	41	39	40	41	42	47	42	39	37	32	36
AB1010	Conserved hypothetical protein (DUF1255 domain protein)	--	--	--	--	--	--	--	--	54	--	61	57	--	--	--
AB1050	Conserved hypothetical protein (DUF24 domain protein)	39	--	34	--	41	38	--	--	--	--	--	42	--	39	--
AB1061	Conserved hypothetical protein (DUF24 domain protein)	40	--	37	--	40	39	--	--	--	--	--	44	--	--	--
AB1082	Conserved hypothetical protein (DUF152 domain protein)	35	34	35	36	39	37	35	34	35	39	44	30	--	34	--
AB1157	Conserved hypothetical protein (DUF748 domain protein)	--	--	--	--	27	--	--	27	--	--	--	--	--	--	--
AB1213	Conserved hypothetical protein (DUF1365 domain protein)	--	--	--	--	--	--	--	--	--	--	--	--	--	--	--
AB1305	Conserved hypothetical protein (DUF455 domain protein)	38	39	41	40	40	46	43	45	47	47	--	--	--	44	--

AB1373	Conserved hypothetical protein (DUF475 domain protein)	--	--	--	--	42	--	--	41	--	--	41	39	--	--	--
AB1398	Conserved hypothetical protein (DUF1706 domain protein)	--	--	--	--	--	--	--	--	--	--	--	--	--	26	--
AB1430	Conserved hypothetical protein (DUF1504 domain protein)	--	--	--	--	--	--	--	--	--	--	--	--	--	--	--
AB1450	Conserved hypothetical protein (DUF28 domain protein)	59	59	57	56	57	57	56	53	55	35	61	63	58	59	57
AB1464	Conserved hypothetical protein (DUF125 domain protein)	--	--	--	--	--	66	61	--	--	58	--	--	--	--	--
AB1494	Conserved hypothetical protein (DUF302 domain protein)	--	--	--	--	--	--	--	--	31	27	--	--	--	--	--
AB1588	Conserved hypothetical protein (DUF1121 domain protein)	--	--	--	--	--	--	--	54	--	--	--	31	--	--	--
AB1633	Conserved hypothetical protein (DUF37 domain protein)	42	--	45	39	--	47	50	44	43	51	47	41	38	--	37
AB1637	Hypothetical protein (DUF77 domain protein)	--	--	--	--	--	--	--	--	44	59	--	--	--	--	--
AB1638	Conserved hypothetical protein (DUF344 domain protein)	51	53	--	48	52	53	55	50	58	--	51	46	--	--	--
AB1643	Conserved hypothetical protein (DUF1291 domain protein)	--	--	--	--	--	--	--	--	--	--	--	--	--	--	--
AB1759	Conserved hypothetical protein (DUF1111 domain protein)	--	--	--	--	--	--	--	--	--	--	--	--	--	--	--
AB1906	Conserved hypothetical protein (DUF178 domain protein)	46	45	50	48	43	45	46	47	50	49	49	41	46	42	45
AB1970	Conserved hypothetical protein (DUF164 domain protein)	50	50	46	51	48	48	48	47	48	53	46	--	37	41	36
AB2031	Hypothetical protein (DUF448 domain protein)	38	37	--	--	37	36	36	--	--	--	40	--	--	--	--
AB2034	Conserved hypothetical protein (DUF150 domain protein)	44	38	41	42	46	46	49	45	56	51	42	44	40	39	39
AB2052	Conserved hypothetical protein (DUF558 domain protein)	38	40	38	38	44	44	43	44	45	46	42	38	37	38	38
AB2082	Conserved hypothetical protein (DUF541 domain protein)	48	47	--	--	42	--	--	--	--	--	--	47	--	--	--
AB2117	Conserved hypothetical protein (DUF143 domain protein)	50	50	56	53	60	--	--	--	49	53	55	--	52	54	52
AB2173	Conserved hypothetical protein (DUF81 domain protein)	--	--	--	--	--	--	--	38	--	--	--	--	--	--	--
AB2176	Conserved hypothetical protein (DUF785 domain protein)	--	--	--	--	--	--	--	--	--	--	73	--	--	--	--
AB2207	Conserved hypothetical protein (DUF208 domain protein)	43	47	45	44	44	43	43	38	52	54	53	40	39	38	40
AB2222	Conserved hypothetical protein (DUF191 domain protein)	64	64	68	65	66	64	63	67	63	69	70	66	64	63	66
AB2242	Conserved hypothetical protein (DUF115 domain protein)	--	--	--	--	--	--	--	--	--	--	27	--	--	--	--
AB2266	Conserved hypothetical protein (DUF328 domain protein)	51	51	55	49	57	53	50	51	51	49	48	35	--	37	--
AB2279	Conserved hypothetical protein (DUF403/404/407 domain protein)	--	--	--	--	--	--	--	--	--	--	--	--	--	--	--
AB2292	Conserved hypothetical protein (DUF404/407 domain protein)	--	--	--	--	--	--	--	--	--	--	--	--	--	--	--

**VI.C**	**Conserved hypothetical proteins - no conserved domains**	**Cj**	**Cc**	**Cl**	**Cu**	**Cf**	**Cv**	**Cn**	**Ch**	**Sv**	**Ni**	**Sd**	**Ws**	**Ha**	**Hh**	**Hp**
AB0011	Conserved hypothetical protein	25	27	--	26	32	32	29	30	45	--	26	--	--	--	--
AB0023	Conserved hypothetical protein	--	--	--	--	--	--	--	--	--	--	--	--	--	--	--
AB0026	Conserved hypothetical protein	--	--	--	--	--	--	--	--	--	--	--	--	--	--	--
AB0031	Conserved hypothetical protein	28	28	30	26	30	27	27	--	--	32	32	31	--	26	--
AB0056	Conserved hypothetical protein	--	--	--	--	--	--	--	--	--	--	25	--	--	--	--
AB0064	Conserved hypothetical protein	--	--	--	--	--	--	--	--	--	35	38	--	35	--	37
AB0071	Conserved hypothetical protein	--	--	--	--	--	--	--	--	51	51	35	42	--	--	--
AB0072	Conserved hypothetical protein	--	--	--	--	--	--	--	--	41	41	--	43	--	--	--
AB0119	Conserved hypothetical protein	45	45	44	45	49	48	45	42	--	29	--	--	--	--	--
AB0129	Conserved hypothetical protein	--	--	--	--	--	28	27	--	--	--	31	27	--	--	--
AB0152	Conserved hypothetical protein	--	--	--	--	--	--	--	--	--	--	--	--	--	--	--
AB0159	Conserved hypothetical protein	29	25	--	25	30	--	--	--	--	--	33	26	27	26	27
AB0181	Conserved hypothetical protein	--	--	--	--	--	--	--	--	--	--	--	--	--	--	--
AB0192	Conserved hypothetical protein	--	--	--	--	--	--	--	--	28	33	40	36	--	--	--
AB0193	Conserved hypothetical protein	31	--	--	35	30	30	30	30	31	28	33	--	--	--	--
AB0199	Conserved hypothetical protein	--	--	--	--	--	--	--	--	--	--	--	--	--	--	--
AB0225	Conserved hypothetical protein	31	31	29	35	--	--	--	--	49	60	--	47	--	38	--
AB0228	Conserved hypothetical protein	31	31	33	30	33	35	34	30	34	37	36	38	35	33	35

AB0233	Conserved hypothetical protein	35	37	38	36	30	--	--	34	31	38	37	--	--	--	--
AB0237	Conserved hypothetical protein	29	32	32	31	32	30	30	28	31	36	40	31	27	31	28
AB0244	Conserved hypothetical protein	60	59	--	61	64	63	61	54	51	58	51	59	34	45	34
AB0248	Conserved hypothetical protein	66	66	65	68	64	66	67	63	71	72	68	62	57	57	57
AB0252	Conserved hypothetical protein	45	42	44	40	42	44	42	44	42	42	51	41	48	43	45
AB0253	Conserved hypothetical protein	39	41	--	--	35	--	--	--	35	59	55	58	--	42	--
AB0268	Conserved hypothetical protein	35	35	36	35	37	31	33	34	31	--	--	--	--	--	--
AB0272	Conserved hypothetical protein	--	43	--	--	--	--	--	--	--	--	--	--	--	43	--
AB0289	Conserved hypothetical protein	35	35	34	38	37	35	36	33	32	42	33	34	30	29	33
AB0293	Conserved hypothetical protein	43	44	38	37	36	38	39	39	39	39	40	40	--	--	--
AB0317	Conserved hypothetical protein	--	--	--	--	--	31	--	28	41	36	40	--	--	--	--
AB0322	Conserved hypothetical protein	--	--	--	--	--	--	--	--	--	--	--	--	--	--	--
AB0323	Conserved hypothetical protein	--	--	--	--	--	--	--	--	51	46	56	--	--	--	--
AB0330	Conserved hypothetical protein	--	--	--	--	--	40	37	--	--	--	--	--	--	--	--
AB0364	Conserved hypothetical protein	--	--	--	--	--	--	--	--	--	--	48	--	--	--	--
AB0370	Conserved hypothetical protein	46	46	47	46	49	43	47	46	43	42	45	42	44	42	44
AB0380	Conserved hypothetical protein	44	44	--	42	46	50	48	44	55	51	38	--	--	--	--
AB0383	Conserved hypothetical protein	--	--	--	--	39	29	35	37	33	30	47	--	--	--	28
AB0389	Conserved hypothetical protein	--	--	--	--	--	--	--	--	--	--	50	--	--	43	--
AB0390	Conserved hypothetical protein	--	--	--	--	--	--	--	--	--	--	36	--	--	31	--
AB0399	Conserved hypothetical protein	--	--	63	--	61	66	69	54	63	38	66	69	54	55	54
AB0401	Conserved hypothetical protein	34	35	33	33	33	34	32	34	26	38	26	36	33	30	36
AB0405	Conserved hypothetical protein	--	--	--	--	--	--	--	--	--	--	49	--	--	--	--
AB0410	Conserved hypothetical protein	--	--	--	--	--	--	--	--	--	--	--	--	--	--	--
AB0414	Conserved hypothetical protein	--	--	--	--	--	39	42	--	47	43	46	42	--	--	--
AB0419	Conserved hypothetical protein	--	--	--	--	--	--	--	48	52	58	54	51	--	--	--
AB0435	Conserved hypothetical protein	--	--	--	--	--	--	--	--	37	--	31	--	--	--	--
AB0449	Conserved hypothetical protein	30	31	28	30	27	27	32	28	27	29	27	29	28	28	28
AB0457	Conserved hypothetical protein	--	--	--	--	--	--	--	--	40	43	40	--	--	--	--
AB0458	Conserved hypothetical protein	--	--	--	--	--	--	--	--	37	47	--	--	--	--	--
AB0463	Conserved hypothetical protein	26	--	--	--	--	--	--	--	--	--	--	40	--	--	--
AB0464	Conserved hypothetical protein	--	--	--	--	--	--	--	--	--	--	--	--	--	--	--
AB0470	Conserved hypothetical protein	--	--	--	--	--	--	--	--	--	--	--	--	--	--	--
AB0471	Conserved hypothetical protein	--	--	--	--	--	--	--	--	--	--	--	--	--	--	--
AB0477	Conserved hypothetical protein	--	--	--	--	--	--	--	--	--	--	--	--	--	--	--
AB0485	Conserved hypothetical protein	--	--	--	--	--	--	--	--	--	--	--	37	--	--	--
AB0509	Conserved hypothetical protein	--	--	--	--	--	--	--	--	--	--	--	51	--	--	--
AB0517	Conserved hypothetical protein	28	29	26	--	33	33	33	31	38	37	32	30	30	29	31
AB0526	Conserved hypothetical protein	47	50	50	53	--	47	50	41	46	53	44	46	45	43	40
AB0527	Conserved hypothetical protein	--	--	--	--	--	--	--	--	--	30	--	25	28	32	28
AB0533	Conserved hypothetical protein	57	57	60	56	63	66	66	56	60	59	62	56	56	54	56
AB0553	Conserved hypothetical protein	--	--	--	--	--	--	--	--	49	--	51	40	--	31	--
AB0555	Conserved hypothetical secreted protein	33	39	--	31	39	--	34	30	28	--	27	38	34	27	34
AB0572	Conserved hypothetical protein	--	--	--	--	--	--	--	--	37	--	40	--	--	--	--
AB0573	Conserved hypothetical protein	--	--	--	--	--	--	--	--	43	43	35	--	--	--	--
AB0583	Conserved hypothetical protein	--	--	--	--	--	--	--	--	69	--	65	--	--	--	--
AB0595	Conserved hypothetical protein	--	--	--	--	--	--	--	--	--	--	--	--	--	--	--

AB0603	Conserved hypothetical protein	--	--	--	--	--	44	48	--	--	--	--	42	--	--	--
AB0614	Conserved hypothetical protein	--	--	--	--	--	--	--	--	--	--	--	--	--	--	--
AB0620	Conserved hypothetical protein	37	--	43	38	41	40	38	39	45	43	38	30	29	--	30
AB0634	Conserved hypothetical protein	61	62	62	62	62	62	63	58	--	--	--	--	40	41	39
AB0649	Conserved hypothetical protein	32	31	32	33	31	30	33	34	27	34	30	33	27	31	27
AB0653	Conserved hypothetical protein	--	--	--	--	--	--	--	--	--	--	--	--	--	--	--
AB0663	Conserved hypothetical protein	--	--	--	--	--	--	85	--	78	--	79	70	42	56	41
AB0664	Conserved hypothetical protein	27	--	--	--	--	--	79	--	73	--	82	72	--	60	--
AB0670	Conserved hypothetical protein	--	--	--	--	--	--	--	--	--	--	--	--	--	--	--
AB0674	Conserved hypothetical protein	--	--	--	--	--	--	--	--	--	--	26	--	--	--	--
AB0685	Conserved hypothetical protein	--	--	--	--	--	--	--	--	--	--	--	--	--	--	--
AB0686	Conserved hypothetical protein	--	--	--	--	--	--	--	--	--	--	--	--	--	--	--
AB0687	Conserved hypothetical protein	--	--	--	--	--	--	--	--	--	--	--	--	--	--	--
AB0715	Conserved hypothetical protein	--	--	--	--	--	--	--	--	--	--	--	46	--	--	--
AB0716	Conserved hypothetical protein	--	--	--	--	--	--	--	--	--	--	--	55	--	--	--
AB0718	Conserved hypothetical protein	--	--	--	--	--	--	--	--	--	--	--	36	--	--	--
AB0719	Conserved hypothetical protein	--	--	--	--	41	50	51	36	--	--	--	43	--	--	--
AB0727	Conserved hypothetical protein	--	--	--	--	--	--	--	--	--	--	--	--	--	--	--
AB0732	Conserved hypothetical protein	--	--	--	--	--	--	--	--	--	--	--	33	--	--	--
AB0737	Conserved hypothetical protein	--	27	--	26	27	31	31	--	--	28	45	28	--	26	--
AB0738	Conserved hypothetical protein	50	50	47	49	41	45	50	48	41	43	49	48	44	42	43
AB0739	Conserved hypothetical protein	--	--	--	--	--	--	--	--	29	--	38	--	--	--	--
AB0741	Conserved hypothetical protein	--	--	--	--	--	--	--	--	--	--	--	40	--	--	--
AB0777	Conserved hypothetical protein	29	27	30	29	28	35	37	--	44	47	44	36	--	29	--
AB0778	Conserved hypothetical protein	28	28	32	28	31	--	--	--	--	--	30	31	--	--	--
AB0814	Conserved hypothetical protein	--	--	--	--	--	--	--	--	--	--	--	--	--	--	--
AB0821	Conserved hypothetical protein	40	--	--	--	44	--	32	--	--	30	81	52	--	--	--
AB0827	Conserved hypothetical protein	--	--	--	--	--	--	--	--	--	--	--	--	--	--	--
AB0829	Conserved hypothetical protein	--	--	--	--	--	--	--	--	--	--	--	53	--	--	--
AB0830	Conserved hypothetical protein	--	--	--	--	--	--	--	--	--	--	--	32	--	--	--
AB0832	Conserved hypothetical protein	--	--	--	--	--	--	--	--	45	46	56	53	--	--	--
AB0849	Conserved hypothetical protein	27	27	27	27	27	27	26	30	28	29	30	28	--	25	26
AB0850	Conserved hypothetical protein	46	45	50	46	43	46	46	--	54	49	48	44	39	--	39
AB0851	Conserved hypothetical protein	37	34	39	33	44	41	42	39	40	48	48	40	38	32	39
AB0860	Conserved hypothetical protein	28	32	34	31	38	38	--	36	--	--	--	--	33	--	34
AB0861	Conserved hypothetical protein	40	45	38	38	43	41	46	39	55	50	47	44	39	40	43
AB0863	Conserved hypothetical protein	--	--	--	--	--	--	--	--	--	--	28	--	--	--	--
AB0866	Conserved hypothetical protein	--	--	--	--	--	--	--	--	--	--	--	34	--	--	--
AB0868	Conserved hypothetical protein	51	51	49	52	50	50	48	48	60	58	60	61	53	56	54
AB0879	Conserved hypothetical protein	63	62	53	59	54	54	56	53	58	53	57	53	49	--	52
AB0880	Conserved hypothetical protein	--	--	--	--	--	--	--	--	--	--	--	--	--	--	--
AB0883	Conserved hypothetical protein	--	--	--	--	--	--	--	--	--	45	--	40	--	--	--
AB0899	Conserved hypothetical protein	50	51	55	50	49	45	50	47	56	49	54	47	--	42	--
AB0910	Conserved hypothetical protein	34	34	34	36	42	39	41	--	--	--	40	41	--	--	--
AB0911	Conserved hypothetical protein	32	32	--	--	--	--	--	--	--	--	--	38	--	--	--
AB0912	Conserved hypothetical protein	--	--	--	--	--	--	--	--	--	--	--	--	--	--	--
AB0913	Conserved hypothetical protein	39	38	39	35	39	40	42	--	--	30	50	49	37	--	37

AB0916	Conserved hypothetical protein	--	--	--	--	--	--	--	--	28	--	--	--	--	--	--
AB0917	Conserved hypothetical protein	--	--	--	--	--	--	--	--	--	--	--	--	--	--	--
AB0923	Conserved hypothetical protein	30	28	26	27	29	31	--	29	34	29	34	32	--	--	--
AB0927	Conserved hypothetical protein	--	--	--	--	--	--	--	--	--	--	43	--	--	--	--
AB0929	Conserved hypothetical protein	--	--	--	--	--	--	--	--	--	--	--	26	--	--	--
AB0930	Conserved hypothetical protein	--	--	--	--	--	--	--	--	--	--	--	28	--	--	--
AB0931	Conserved hypothetical protein	--	--	--	--	--	--	--	--	--	--	--	--	--	--	--
AB0932	Conserved hypothetical protein	--	--	--	--	--	--	--	--	--	--	51	--	--	--	--
AB0934	Conserved hypothetical protein	--	--	--	--	51	47	--	48	--	--	--	52	--	--	--
AB0948	Conserved hypothetical protein	--	--	--	--	--	--	--	--	--	--	--	--	--	--	--
AB0951	Conserved hypothetical protein	--	--	--	--	--	--	--	--	--	--	--	--	--	--	--
AB0953	Conserved hypothetical protein	--	--	--	--	--	50	34	--	--	--	--	--	--	--	--
AB0954	Conserved hypothetical protein	--	--	--	--	--	--	--	--	--	--	37	--	--	--	--
AB0958	Conserved hypothetical protein	--	--	--	--	41	51	52	47	44	--	--	--	--	--	--
AB0961	Conserved hypothetical protein	--	--	--	--	--	--	--	--	--	--	--	--	--	--	--
AB1001	Conserved hypothetical protein	31	31	31	32	34	34	34	30	30	27	33	32	29	30	30
AB1004	Conserved hypothetical protein	39	35	37	36	40	36	37	37	45	45	46	42	37	36	38
AB1011	Conserved hypothetical protein	--	--	--	--	49	--	47	53	49	53	57	47	54	--	56
AB1012	Conserved hypothetical protein	--	--	25	--	--	--	--	--	33	35	--	27	--	--	--
AB1013	Conserved hypothetical protein	--	--	--	--	--	--	--	--	34	33	42	32	--	--	--
AB1027	Conserved hypothetical protein	--	--	--	--	--	--	38	--	--	--	--	--	--	--	--
AB1032	Conserved hypothetical protein	--	--	--	--	--	--	--	--	--	--	--	--	--	--	--
AB1047	Conserved hypothetical protein	--	--	--	--	--	--	--	--	--	--	--	--	--	--	--
AB1051	Conserved hypothetical protein	--	39	--	--	40	36	32	--	--	--	--	43	--	--	--
AB1056	Conserved hypothetical protein	--	--	--	--	--	--	--	--	61	38	--	--	--	--	--
AB1063	Conserved hypothetical protein	--	--	--	--	--	--	--	--	--	--	31	--	--	--	--
AB1077	Conserved hypothetical protein	--	--	--	--	--	--	--	--	--	--	--	--	--	--	--
AB1093	Conserved hypothetical protein	--	--	--	--	--	30	35	42	--	--	--	26	--	34	--
AB1097	Conserved hypothetical protein	--	56	--	56	--	49	--	45	--	39	--	47	--	49	--
AB1098	Conserved hypothetical protein	--	--	--	--	--	--	--	--	--	--	--	--	--	--	--
AB1099	Conserved hypothetical protein	--	--	--	--	--	--	--	--	--	--	--	53	--	--	--
AB1105	Conserved hypothetical protein	--	--	--	--	--	--	34	--	--	--	51	40	--	--	--
AB1106	Conserved hypothetical protein	34	31	39	35	37	35	35	35	56	51	60	56	34	39	36
AB1117	Conserved hypothetical protein	--	--	--	--	--	--	--	--	47	30	33	--	--	--	--
AB1119	Conserved hypothetical protein	--	--	--	--	--	--	--	--	--	--	--	--	--	--	--
AB1130	Conserved hypothetical protein	--	--	--	--	--	46	41	--	--	--	--	--	--	--	--
AB1136	Conserved hypothetical protein	--	--	--	--	--	--	--	--	51	47	48	43	43	--	43
AB1139	Conserved hypothetical protein	--	--	--	--	--	--	--	--	68	--	--	--	--	--	--
AB1140	Conserved hypothetical protein	--	--	--	--	--	--	--	--	--	--	--	--	--	--	--
AB1141	Conserved hypothetical protein	--	--	--	--	--	--	--	--	--	--	--	--	--	--	--
AB1143	Conserved hypothetical protein	--	--	--	--	--	--	--	--	--	--	--	--	--	--	--
AB1144	Conserved hypothetical protein	--	41	--	--	36	--	--	--	44	41	36	31	--	49	--
AB1146	Conserved hypothetical protein	--	--	--	--	--	--	--	--	--	--	--	--	--	--	--
AB1149	Conserved hypothetical protein	--	--	--	--	--	33	37	--	--	--	--	--	--	--	--
AB1170	Conserved hypothetical protein	--	--	--	--	--	--	--	--	--	--	--	--	--	--	--
AB1178	Conserved hypothetical protein	--	--	--	--	--	--	--	--	31	43	33	--	--	--	--
AB1182	Conserved hypothetical protein	--	--	56	--	58	53	52	51	49	47	44	49	--	--	--

AB1188	Conserved hypothetical protein	34	34	34	37	42	43	35	37	30	--	36	36	--	35	--
AB1190	Conserved hypothetical protein	--	--	35	--	42	39	37	35	35	26	40	41	34	36	33
AB1197	Conserved hypothetical protein	--	--	--	--	--	--	--	--	--	--	--	--	--	--	--
AB1200	Conserved hypothetical protein	--	--	--	--	--	--	--	--	--	--	39	--	--	--	--
AB1208	Conserved hypothetical protein	41	39	39	39	42	46	47	31	42	43	40	47	34	36	33
AB1211	Conserved hypothetical protein	--	--	--	--	--	--	--	--	--	--	--	--	--	--	--
AB1214	Conserved hypothetical protein	--	--	--	--	--	--	--	--	--	--	--	--	--	--	--
AB1229	Conserved hypothetical protein	48	52	58	--	--	47	49	--	49	50	55	--	42	38	40
AB1231	Conserved hypothetical protein	28	26	28	--	29	26	27	27	27	29	30	27	--	25	--
AB1240	Conserved hypothetical protein	37	26	30	32	--	39	38	30	34	38	33	31	34	33	33
AB1241	Conserved hypothetical protein	--	--	--	--	38	31	33	37	38	51	50	38	36	40	--
AB1248	Conserved hypothetical protein	--	--	--	--	69	73	73	--	69	67	--	--	--	--	--
AB1253	Conserved hypothetical protein	--	--	--	--	--	--	--	--	--	--	--	--	--	--	--
AB1265	Conserved hypothetical protein	--	--	--	--	--	--	--	--	--	--	--	--	--	--	--
AB1268	Conserved hypothetical protein	--	--	--	--	--	--	--	--	46	--	46	--	--	--	--
AB1271	Conserved hypothetical protein	--	--	--	--	--	--	--	--	--	--	25	--	--	--	--
AB1281	Conserved hypothetical protein	44	43	43	45	47	44	45	40	41	43	50	41	47	48	47
AB1283	Conserved hypothetical protein	--	--	--	--	25	27	26	27	33	26	40	--	--	--	--
AB1291	Conserved hypothetical protein	50	46	--	49	--	--	--	50	61	61	61	49	--	--	--
AB1309	Conserved hypothetical protein	57	60	54	57	61	61	55	56	62	55	59	60	53	45	54
AB1325	Conserved hypothetical protein	33	--	--	35	--	--	--	30	42	--	35	32	31	32	33
AB1328	Conserved hypothetical protein	--	--	44	--	49	47	45	47	46	--	50	50	39	43	39
AB1335	Conserved hypothetical protein	--	--	--	--	--	--	--	--	--	--	--	--	--	--	--
AB1341	Conserved hypothetical protein	--	--	--	--	--	--	--	--	--	--	--	--	--	--	--
AB1349	Conserved hypothetical protein	--	--	--	--	--	--	--	--	--	--	--	--	--	--	--
AB1359	Conserved hypothetical protein	28	29	28	32	26	--	25	30	--	30	--	--	--	--	--
AB1363	Conserved hypothetical protein	--	--	--	--	--	--	--	--	--	--	52	--	--	--	--
AB1376	Conserved hypothetical protein	--	--	--	--	--	--	--	37	--	--	--	--	--	--	--
AB1377	Conserved hypothetical protein	48	45	48	44	46	42	44	41	36	48	38	43	47	40	44
AB1389	Conserved hypothetical protein	43	42	40	38	35	38	38	44	29	30	32	--	--	--	--
AB1393	Conserved hypothetical protein	--	--	--	--	--	--	--	--	--	--	40	--	--	--	--
AB1394	Conserved hypothetical protein	--	--	--	--	--	--	--	--	--	--	--	--	--	--	--
AB1410	Conserved hypothetical protein	--	--	--	--	--	--	--	--	--	--	--	--	--	--	--
AB1417	Conserved hypothetical protein	--	--	--	--	--	--	--	--	29	37	31	--	--	--	--
AB1423	Conserved hypothetical protein	--	--	--	--	--	--	--	--	--	--	--	--	--	--	--
AB1425	Conserved hypothetical protein	--	--	--	--	--	--	--	--	--	--	54	--	--	--	--
AB1432	Conserved hypothetical protein	--	--	26	27	27	25	26	25	--	--	30	--	--	--	--
AB1439	Conserved hypothetical protein	--	--	--	27	--	--	--	--	--	--	--	--	--	--	--
AB1467	Conserved hypothetical protein	--	--	--	--	--	--	--	--	42	--	--	--	--	--	--
AB1472	Conserved hypothetical protein	--	--	--	--	--	--	--	--	--	--	64	--	--	--	--
AB1477	Conserved hypothetical protein	35	--	34	32	--	--	34	--	--	--	29	44	--	--	--
AB1488	Conserved hypothetical protein	--	--	--	--	--	--	34	--	42	--	--	--	--	--	--
AB1490	Conserved hypothetical protein	--	--	--	--	--	--	--	--	--	--	35	--	--	--	--
AB1493	Conserved hypothetical protein	--	--	--	--	--	--	--	--	--	--	60	36	--	--	--
AB1495	Conserved hypothetical protein	--	--	--	--	--	--	--	--	--	--	--	--	--	--	--
AB1499	Conserved hypothetical protein	--	--	--	--	--	--	--	--	--	--	--	--	--	--	--
AB1500	Conserved hypothetical protein	--	--	--	--	--	--	--	--	--	--	--	--	--	--	--

AB1509	Conserved hypothetical protein	--	--	--	--	33	37	45	--	--	--	66	--	--	--	--
AB1510	Conserved hypothetical protein	30	31	--	29	31	29	31	31	--	--	58	29	--	--	--
AB1526	Conserved hypothetical protein	--	--	--	--	--	--	--	--	--	--	--	--	--	--	--
AB1527	Conserved hypothetical protein	--	--	--	--	--	--	--	--	--	--	--	--	--	--	--
AB1536	Conserved hypothetical protein	--	--	--	--	--	--	--	--	--	--	--	--	--	--	--
AB1538	Conserved hypothetical protein	--	--	--	--	--	--	--	--	--	--	--	--	--	--	--
AB1540	Conserved hypothetical protein	--	--	--	--	--	--	--	--	--	--	34	--	--	--	--
AB1544	Conserved hypothetical protein	--	--	--	--	--	--	--	--	--	--	--	--	--	--	--
AB1550	Conserved hypothetical protein	--	--	--	--	--	33	--	--	--	35	--	--	--	--	--
AB1566	Conserved hypothetical protein	28	--	--	--	27	--	--	--	--	29	--	--	26	29	27
AB1572	Conserved hypothetical protein	--	--	--	--	--	--	--	--	--	--	--	--	--	--	--
AB1597	Conserved hypothetical protein	31	33	28	29	30	32	30	27	33	33	30	30	27	30	29
AB1600	Conserved hypothetical protein	26	--	--	26	--	--	--	--	--	27	27	32	25	25	--
AB1622	Conserved hypothetical protein	30	31	31	27	31	30	30	32	29	33	37	36	29	30	28
AB1625	Conserved hypothetical protein	--	--	--	--	--	--	--	--	--	--	--	44	--	40	--
AB1627	Conserved hypothetical protein	--	--	--	--	--	--	--	--	--	--	--	--	--	--	--
AB1631	Conserved hypothetical protein	41	40	37	40	42	36	36	41	42	49	40	43	40	43	39
AB1639	Conserved hypothetical protein	--	--	--	--	37	33	34	38	48	44	46	--	--	--	--
AB1649	Conserved hypothetical protein	38	39	40	42	38	37	38	--	--	--	41	40	38	41	38
AB1716	Conserved hypothetical protein	--	--	--	--	--	--	--	--	--	--	--	--	--	--	--
AB1718	Conserved hypothetical protein	66	62	--	66	65	65	63	57	62	--	64	50	--	48	--
AB1722	Conserved hypothetical protein	--	--	--	--	--	--	--	--	--	--	--	--	--	--	--
AB1724	Conserved hypothetical protein	--	--	--	--	--	--	--	--	--	--	44	--	--	--	--
AB1760	Conserved hypothetical protein	--	--	--	--	--	--	--	--	--	--	--	--	--	--	--
AB1761	Conserved hypothetical protein	--	--	--	25	--	27	--	26	--	--	27	28	--	27	--
AB1769	Conserved hypothetical protein	--	--	--	--	--	36	35	42	39	--	34	44	--	--	--
AB1780	Conserved hypothetical protein	--	--	--	--	--	--	--	--	--	--	--	--	--	--	--
AB1781	Conserved hypothetical protein	62	61	63	60	60	57	60	58	--	--	56	56	--	--	--
AB1782	Conserved hypothetical protein	25	--	27	--	27	--	27	--	--	--	--	--	--	--	--
AB1792	Conserved hypothetical protein	--	--	--	--	--	--	--	--	55	--	50	--	--	--	--
AB1800	Conserved hypothetical protein	43	43	51	--	53	47	47	--	50	34	49	52	50	44	49
AB1811	Conserved hypothetical protein	--	--	--	--	--	--	--	--	--	--	47	--	--	--	--
AB1813	Conserved hypothetical protein	--	--	--	--	--	--	--	--	--	--	48	--	--	--	--
AB1815	Conserved hypothetical protein	--	--	--	--	--	--	--	--	--	--	--	--	--	--	--
AB1820	Conserved hypothetical protein	26	25	--	--	25	25	--	--	--	--	25	--	--	--	--
AB1822	Conserved hypothetical protein	--	--	--	--	--	--	--	--	--	--	--	--	--	--	--
AB1831	Conserved hypothetical protein	--	--	--	--	--	--	30	--	--	--	--	--	--	--	--
AB1839	Conserved hypothetical protein	32	30	29	30	27	29	28	30	--	29	43	--	26	--	27
AB1840	Conserved hypothetical protein	--	37	33	34	40	--	--	--	47	--	47	33	--	31	31
AB1845	Conserved hypothetical protein	52	51	51	42	45	42	43	45	50	--	--	48	38	42	39
AB1864	Conserved hypothetical protein	--	--	--	--	--	--	--	--	--	--	--	52	--	--	--
AB1865	Conserved hypothetical protein	--	--	--	--	--	--	--	--	--	--	--	57	--	--	--
AB1866	Conserved hypothetical protein	--	--	--	--	--	--	--	--	--	--	--	63	--	--	--
AB1867	Conserved hypothetical protein	--	--	--	--	--	--	--	--	--	--	--	57	--	--	--
AB1873	Conserved hypothetical protein	--	--	--	--	--	--	--	--	32	30	31	32	--	--	--
AB1874	Conserved hypothetical protein	--	--	--	--	--	--	--	--	--	--	29	--	--	--	--
AB1885	Conserved hypothetical protein	--	--	--	--	--	--	--	--	--	--	--	--	--	--	--

AB1922	Conserved hypothetical protein	--	--	--	--	41	--	--	--	48	--	45	--	40	--	40
AB1929	Conserved hypothetical protein	--	--	--	--	--	--	--	--	38	44	--	--	--	36	--
AB1959	Conserved hypothetical protein	--	--	--	--	28	--	32	--	--	--	32	--	29	--	--
AB1965	Conserved hypothetical protein	46	50	51	47	50	51	51	49	51	51	54	53	--	43	--
AB1971	Conserved hypothetical protein	52	51	47	49	47	48	49	47	48	50	49	--	41	41	45
AB1990	Conserved hypothetical protein	28	--	--	29	--	27	--	--	29	27	32	--	--	--	--
AB1999	Conserved hypothetical protein	--	--	--	--	--	--	--	--	--	--	--	51	--	--	--
AB2000	Conserved hypothetical protein	--	--	--	--	--	--	--	--	--	--	--	44	--	--	--
AB2001	Conserved hypothetical protein	--	--	--	--	--	--	--	--	--	--	--	43	--	--	--
AB2003	Conserved hypothetical protein	--	--	--	--	--	--	--	--	--	--	57	--	--	--	--
AB2005	Conserved hypothetical protein	--	--	--	--	--	--	--	--	--	--	--	--	--	--	--
AB2007	Conserved hypothetical protein	--	--	--	--	--	--	--	--	--	--	57	--	--	--	--
AB2008	Conserved hypothetical protein	51	51	50	--	43	51	50	56	53	55	57	53	48	55	47
AB2029	Conserved hypothetical protein	39	37	41	38	41	41	36	39	40	41	45	--	32	34	35
AB2049	Conserved hypothetical protein	--	--	--	--	--	--	--	--	--	--	--	61	--	--	--
AB2051	Conserved hypothetical protein	--	25	--	--	--	--	--	25	--	32	--	29	27	25	26
AB2061	Conserved hypothetical protein	--	--	--	--	--	50	--	--	--	--	--	--	--	--	--
AB2062	Conserved hypothetical protein	33	31	30	31	32	29	31	30	30	31	35	29	30	28	29
AB2064	Conserved hypothetical protein	28	25	29	32	29	31	31	31	29	35	28	26	--	--	--
AB2085	Conserved hypothetical protein	--	--	--	--	--	--	--	--	60	--	56	--	--	--	--
AB2091	Conserved hypothetical protein	--	--	--	--	--	--	--	--	--	--	--	--	--	--	--
AB2094	Conserved hypothetical protein	--	--	--	--	31	--	31	28	--	--	--	29	--	--	--
AB2099	Conserved hypothetical protein	--	--	--	--	--	--	41	38	--	--	42	44	--	--	--
AB2101	Conserved hypothetical protein	--	--	--	--	--	--	33	36	--	--	38	30	--	--	--
AB2105	Conserved hypothetical protein	28	30	26	28	32	32	33	27	30	29	29	31	--	26	--
AB2106	Conserved hypothetical protein	38	36	36	33	33	33	35	28	32	30	30	30	27	25	26
AB2111	Conserved hypothetical protein	28	31	30	27	32	30	30	30	32	29	32	27	27	--	27
AB2130	Conserved hypothetical protein	--	--	--	--	--	--	--	--	--	--	--	--	--	--	--
AB2133	Conserved hypothetical protein	--	--	--	--	41	42	--	32	--	--	--	44	--	--	--
AB2138	Conserved hypothetical protein	--	--	--	--	--	--	--	--	--	--	--	--	--	--	--
AB2142	Conserved hypothetical protein	--	--	--	--	--	--	--	--	--	--	--	--	--	--	--
AB2155	Conserved hypothetical protein	--	--	--	--	--	--	--	--	--	34	40	--	--	--	--
AB2159	Conserved hypothetical protein	--	--	--	--	--	--	--	--	--	--	72	--	--	--	--
AB2171	Conserved hypothetical protein	--	--	--	--	--	--	--	--	--	--	53	--	--	--	--
AB2177	Conserved hypothetical protein	--	--	51	--	54	--	--	--	46	--	51	39	--	--	--
AB2185	Conserved hypothetical protein	--	--	--	--	--	39	37	--	--	--	--	--	--	--	--
AB2221	Conserved hypothetical protein	--	30	31	--	--	27	31	34	--	--	--	--	--	29	--
AB2225	Conserved hypothetical protein	--	--	--	--	--	42	--	44	--	44	45	--	--	--	--
AB2227	Conserved hypothetical protein	--	--	--	--	--	--	--	--	--	--	28	--	--	--	--
AB2240	Conserved hypothetical protein	--	--	--	--	--	--	--	--	--	--	--	--	--	--	--
AB2241	Conserved hypothetical protein	--	--	--	--	--	--	--	--	--	--	--	--	--	--	--
AB2249	Conserved hypothetical protein	--	--	--	--	--	--	--	--	--	--	--	--	--	--	--
AB2254	Conserved hypothetical protein	35	34	35	32	41	41	45	32	32	--	49	38	29	30	34
AB2258	Conserved hypothetical protein	--	--	--	--	--	59	61	--	--	61	70	62	--	53	--
AB2268	Conserved hypothetical protein	--	--	--	--	--	30	27	--	--	--	--	--	--	--	--
AB2275	Conserved hypothetical protein	--	--	--	--	--	--	--	--	--	--	--	--	--	--	--
AB2286	Conserved hypothetical protein	--	--	--	--	--	--	--	--	--	--	--	--	--	--	--

AB2296	Conserved hypothetical protein	--	--	--	--	--	--	--	--	--	--	--	59	--	--	--
AB2298	Conserved hypothetical protein	--	--	--	--	--	--	--	--	--	--	--	50	--	--	--
AB2318	Conserved hypothetical protein	--	--	--	--	--	--	--	--	39	--	31	--	--	--	--

VI.D Hypothetical proteins

		Cj	Cc	Cl	Cu	Cf	Cv	Cn	Ch	Sv	Ni	Sd	Ws	Ha	Hh	Hp
AB0006	Hypothetical protein	--	--	--	--	25	--	--	--	26	--	--	--	--	--	--
AB0022	Hypothetical protein	--	--	--	--	--	--	--	--	--	--	--	--	--	--	--
AB0024	Hypothetical protein	--	--	--	--	--	--	--	--	--	--	--	--	--	--	--
AB0047	Hypothetical protein	--	--	--	--	--	--	--	--	--	--	--	--	--	--	--
AB0065	Hypothetical protein	--	--	--	--	--	--	--	--	--	--	--	--	--	--	--
AB0073	Hypothetical protein	--	--	--	--	--	--	--	--	--	--	--	--	--	--	--
AB0079	Hypothetical protein	--	--	--	--	--	--	--	--	51	38	--	--	--	--	--
AB0087	Hypothetical protein	--	--	--	--	--	--	--	--	--	--	--	--	--	--	--
AB0094	Hypothetical protein	--	--	--	--	--	--	--	--	--	--	--	--	--	--	--
AB0109	Hypothetical protein	--	--	--	27	--	--	--	25	--	--	26	--	--	--	--
AB0113	Hypothetical protein	--	--	--	--	--	--	--	--	30	--	--	--	--	--	--
AB0114	Hypothetical protein	--	--	--	--	--	--	--	--	31	--	--	--	--	--	--
AB0116	Hypothetical protein	--	--	--	--	--	--	--	--	--	--	--	--	--	--	--
AB0124	Hypothetical protein	--	--	--	--	--	--	--	--	--	--	--	--	--	--	--
AB0126	Hypothetical protein	--	--	--	--	--	--	--	--	--	--	--	--	--	--	--
AB0133	Hypothetical protein	--	--	--	--	--	--	--	--	--	--	--	--	--	--	--
AB0151	Hypothetical protein	--	--	--	--	--	--	--	--	--	--	--	--	--	--	--
AB0154	Hypothetical protein	--	--	--	--	--	--	--	--	--	--	--	--	--	--	--
AB0165	Hypothetical protein	--	--	--	--	--	--	--	--	--	--	--	--	--	--	--
AB0168	Hypothetical protein	--	--	--	--	--	--	--	--	37	--	47	--	--	--	--
AB0173	Hypothetical protein	--	--	--	--	--	--	--	--	--	--	--	--	--	--	--
AB0185	Hypothetical protein	--	--	--	--	--	27	--	--	29	--	--	--	--	--	--
AB0186	Hypothetical protein	--	--	--	--	--	--	--	--	--	--	--	--	--	--	--
AB0201	Hypothetical protein	--	--	--	--	--	--	--	--	--	--	--	--	--	--	--
AB0202	Hypothetical protein	--	--	--	--	--	--	--	--	--	--	--	--	--	--	--
AB0204	Hypothetical protein	--	--	--	--	--	--	--	--	--	--	--	--	--	--	--
AB0209	Hypothetical protein	--	--	--	--	--	--	--	--	--	--	--	--	--	--	--
AB0230	Hypothetical protein	--	--	--	--	--	--	--	--	29	34	28	--	--	--	--
AB0241	Hypothetical protein	--	--	--	--	--	--	--	--	--	--	--	--	--	--	--
AB0247	Hypothetical protein	--	--	--	--	--	--	--	--	--	--	--	--	--	--	--
AB0249	Hypothetical protein	--	--	--	--	--	--	--	--	--	--	--	--	--	--	--
AB0270	Hypothetical protein	--	--	--	--	--	--	--	--	--	--	--	--	--	--	--
AB0277	Hypothetical protein	--	--	--	--	--	--	--	--	--	--	--	--	--	--	--
AB0341	Hypothetical protein	--	--	--	--	--	--	--	--	--	--	--	--	--	--	--
AB0347	Hypothetical protein	--	--	--	--	--	--	--	--	--	--	--	--	--	--	--
AB0395	Hypothetical protein	--	--	--	--	--	--	--	--	--	--	--	--	--	--	--
AB0396	Hypothetical protein	--	--	--	--	--	--	--	--	--	--	--	--	--	--	--
AB0406	Hypothetical protein	--	--	--	--	--	--	--	--	--	--	--	--	--	--	--
AB0407	Hypothetical protein	--	--	--	--	--	--	--	--	--	--	--	--	--	--	--
AB0420	Hypothetical protein	--	--	--	--	--	--	--	--	--	36	--	--	--	--	--
AB0421	Hypothetical protein	--	--	--	--	--	--	--	--	--	--	--	--	--	--	--
AB0437	Hypothetical protein	--	--	--	--	--	--	--	--	38	--	--	--	--	--	--

AB0438	Hypothetical protein	--	--	--	--	--	--	--	--	50	--	--	--	--	--	--
AB0442	Hypothetical protein	--	--	--	--	--	--	--	--	--	--	--	--	--	--	--
AB0493	Hypothetical protein	--	--	--	--	--	--	--	--	--	--	--	--	--	--	--
AB0524	Hypothetical protein	--	--	--	--	--	--	--	--	65	--	--	--	--	--	--
AB0532	Hypothetical protein	--	--	--	--	--	--	--	--	--	--	--	--	--	--	--
AB0535	Hypothetical protein	--	--	--	--	--	--	--	--	--	--	--	--	--	--	--
AB0536	Hypothetical protein	--	--	--	--	--	--	--	--	--	--	--	--	--	--	--
AB0540	Hypothetical protein	--	--	--	--	--	--	--	--	--	--	--	--	--	--	--
AB0544	Hypothetical protein	--	--	--	--	38	36	--	39	34	31	42	33	45	39	45
AB0549	Hypothetical protein	39	39	27	--	36	--	--	38	36	36	31	--	--	--	--
AB0558	Hypothetical protein	--	--	--	--	--	49	--	--	33	36	--	--	--	--	--
AB0569	Hypothetical protein	--	--	--	--	--	--	--	--	--	--	--	--	--	--	--
AB0571	Hypothetical protein	--	--	--	--	--	--	--	--	36	35	--	--	--	--	--
AB0574	Hypothetical protein	--	--	--	--	--	--	--	--	--	--	--	--	--	--	--
AB0582	Hypothetical protein	--	--	--	--	--	--	--	--	--	--	--	--	--	--	--
AB0584	Hypothetical protein	--	--	--	--	--	--	--	--	--	--	--	--	--	--	--
AB0587	Hypothetical protein	--	--	--	--	--	--	--	--	--	--	--	--	--	--	--
AB0588	Hypothetical protein	--	--	--	--	--	--	--	--	--	--	--	--	--	--	--
AB0589	Hypothetical protein	--	--	--	--	--	--	--	--	--	--	--	--	--	--	--
AB0598	Hypothetical protein	--	--	--	--	--	--	--	--	--	--	--	--	--	--	--
AB0599	Hypothetical protein	--	34	--	--	37	--	--	--	47	--	39	39	--	--	--
AB0624	Hypothetical protein	--	--	--	--	--	--	--	--	--	--	--	--	--	--	--
AB0628	Hypothetical protein	--	--	--	--	--	--	--	--	--	--	--	--	--	--	--
AB0629	Hypothetical protein	--	--	--	--	--	--	--	--	--	--	--	--	--	--	--
AB0642	Hypothetical protein	--	--	--	--	--	--	--	--	--	--	--	--	--	--	--
AB0675	Hypothetical protein	--	--	--	--	--	--	--	--	--	--	--	--	--	--	--
AB0682	Hypothetical protein	--	--	--	--	--	--	--	--	--	--	--	--	--	--	--
AB0698	Hypothetical protein	--	--	--	30	26	--	29	--	32	--	--	--	--	27	--
AB0712	Hypothetical protein	--	--	--	--	--	--	--	--	--	--	--	--	--	--	--
AB0717	Hypothetical protein	--	--	--	--	--	43	--	32	--	--	--	31	--	--	--
AB0721	Hypothetical protein	--	--	--	--	--	--	--	--	--	--	--	--	--	--	--
AB0723	Hypothetical protein	--	--	--	--	--	--	--	--	--	--	--	--	--	--	--
AB0745	Hypothetical protein	--	--	--	--	--	--	--	--	--	--	--	--	--	--	--
AB0752	Hypothetical protein	--	--	--	--	--	--	--	--	--	--	--	--	--	--	--
AB0753	Hypothetical protein	--	--	--	--	--	--	--	--	--	--	--	--	--	--	--
AB0799	Hypothetical protein	--	--	--	--	--	--	--	--	--	--	--	--	--	--	--
AB0800	Hypothetical protein	--	--	--	--	--	--	--	31	43	--	--	--	--	--	--
AB0801	Hypothetical protein	--	--	--	--	--	--	--	38	39	--	--	--	--	--	--
AB0807	Hypothetical protein	--	--	--	--	--	--	--	--	--	--	--	--	--	--	--
AB0820	Hypothetical protein	--	--	--	--	--	--	--	--	--	--	--	--	--	--	--
AB0822	Hypothetical protein	--	--	--	--	--	--	--	--	--	--	--	--	--	--	--
AB0825	Hypothetical protein	--	--	--	--	--	--	--	--	--	--	--	--	--	--	--
AB0867	Hypothetical protein	--	--	--	--	--	--	--	--	--	--	--	--	--	--	--
AB0887	Hypothetical protein	--	--	--	--	--	--	--	--	--	--	--	--	--	--	--
AB0894	Hypothetical protein	--	--	--	--	--	--	--	--	--	--	--	--	--	--	--
AB0895	Hypothetical protein	--	--	--	--	--	--	--	--	--	--	--	--	--	--	--
AB0896	Hypothetical protein	--	--	--	--	--	--	--	--	--	--	--	--	--	--	--

AB0897	Hypothetical protein	--	--	--	--	--	--	--	--	--	--	--	--	--	--	--
AB0900	Hypothetical protein	--	--	--	--	--	--	--	--	--	--	--	--	--	--	--
AB0914	Hypothetical protein	--	--	--	--	--	--	--	--	--	--	--	--	--	--	--
AB0915	Hypothetical protein	--	--	--	--	--	--	--	--	--	--	--	--	--	--	--
AB0918	Hypothetical protein	--	--	--	--	--	--	--	--	--	--	--	--	--	--	--
AB0919	Hypothetical protein	--	--	--	--	--	--	--	--	--	--	--	--	--	--	--
AB0920	Hypothetical protein	--	--	--	--	--	--	--	--	--	--	--	--	--	--	--
AB0936	Hypothetical protein	--	--	--	--	28	26	--	28	--	--	--	--	--	--	--
AB0942	Hypothetical protein	--	--	--	--	--	--	--	--	--	--	--	--	--	--	--
AB0943	Hypothetical protein	--	--	--	--	--	--	--	--	--	--	--	--	--	--	--
AB0944	Hypothetical protein	--	--	--	--	--	--	--	--	--	--	--	--	--	--	--
AB0945	Hypothetical protein	--	--	--	--	--	--	--	--	--	--	--	--	--	--	--
AB0946	Hypothetical protein	--	--	--	--	--	--	--	--	--	--	--	--	--	--	--
AB0947	Hypothetical protein	--	--	--	--	--	--	--	--	--	--	--	--	--	--	--
AB0949	Hypothetical protein	--	--	--	--	--	--	--	--	--	--	--	--	--	--	--
AB0950	Hypothetical protein	--	--	--	--	--	--	--	--	--	--	--	--	--	--	--
AB0956	Hypothetical protein	--	--	--	--	--	--	--	--	--	--	--	--	--	--	--
AB0957	Hypothetical protein	--	--	--	--	--	--	--	--	--	--	--	--	--	--	--
AB0959	Hypothetical protein	--	--	--	--	26	--	--	--	--	--	--	--	--	--	--
AB0976	Hypothetical protein	--	--	--	--	--	--	--	--	--	--	--	--	--	--	--
AB0982	Hypothetical protein	28	--	--	--	--	26	--	26	--	--	--	--	--	--	--
AB1000	Hypothetical protein	--	--	--	--	--	--	--	--	--	--	--	--	--	--	--
AB1028	Hypothetical protein	--	--	--	--	--	--	--	--	--	--	--	--	--	--	--
AB1041	Hypothetical protein	--	--	--	--	--	--	--	--	--	--	--	--	--	--	--
AB1067	Hypothetical protein	--	--	--	--	--	--	--	--	--	--	--	--	--	--	--
AB1086	Hypothetical protein	--	--	--	--	--	--	--	--	33	--	39	--	--	--	--
AB1088	Hypothetical protein	--	--	--	--	--	--	--	--	--	--	--	--	--	--	--
AB1089	Hypothetical protein	--	--	--	--	--	--	--	--	--	--	--	--	--	--	--
AB1094	Hypothetical protein	--	--	--	--	--	--	--	--	--	--	--	--	--	--	--
AB1101	Hypothetical protein	--	--	--	--	--	--	--	--	--	--	--	--	--	--	--
AB1107	Hypothetical protein	--	--	--	--	--	--	--	--	--	--	--	--	--	--	--
AB1114	Hypothetical protein	--	--	--	--	--	--	--	--	--	--	--	--	--	--	--
AB1118	Hypothetical protein	--	--	--	--	--	--	--	--	--	--	--	--	--	--	--
AB1120	Hypothetical protein	--	--	--	--	--	--	--	--	--	--	--	--	--	--	--
AB1122	Hypothetical protein	--	--	--	--	--	--	--	--	--	--	--	--	--	--	--
AB1142	Hypothetical protein	--	--	--	--	--	--	--	--	--	--	--	--	--	--	--
AB1145	Hypothetical protein	--	--	--	--	--	--	--	--	--	--	--	--	--	--	--
AB1156	Hypothetical protein	--	--	--	--	--	--	--	--	--	--	--	--	--	--	--
AB1163	Hypothetical protein	--	--	--	--	--	--	--	--	--	--	--	--	--	--	--
AB1173	Hypothetical protein	--	--	--	--	--	--	--	--	--	--	--	--	--	--	--
AB1175	Hypothetical protein	--	--	--	--	--	--	--	--	--	--	--	--	--	--	--
AB1176	Hypothetical protein	--	--	--	--	--	--	--	--	--	--	--	--	--	--	--
AB1177	Hypothetical protein	--	--	--	--	--	--	--	--	--	--	--	--	--	--	--
AB1179	Hypothetical protein	--	--	--	--	--	--	--	--	--	--	--	--	--	--	--
AB1181	Hypothetical protein	--	--	--	--	--	--	--	--	--	--	--	--	--	--	--
AB1186	Hypothetical protein	--	--	--	--	--	--	--	--	--	--	--	--	--	--	--
AB1201	Hypothetical protein	--	--	--	--	--	--	--	--	--	--	--	--	--	--	--

AB1202	Hypothetical protein	--	--	--	--	--	--	--	--	--	--	--	--	--	--	--
AB1226	Hypothetical protein	--	--	--	--	--	--	--	--	27	29	29	--	--	--	30
AB1233	Hypothetical protein	--	--	--	--	--	--	--	--	--	--	--	--	--	--	--
AB1234	Hypothetical protein	--	--	--	--	--	--	--	--	--	--	--	--	--	--	--
AB1239	Hypothetical protein	--	--	--	--	--	--	--	--	--	--	--	--	--	--	--
AB1252	Hypothetical protein	--	--	--	--	--	--	--	--	--	--	--	--	--	--	--
AB1257	Hypothetical protein	--	--	--	--	--	--	--	--	--	--	--	--	--	--	--
AB1258	Hypothetical protein	--	--	--	--	--	--	--	--	--	--	--	--	--	--	--
AB1260	Hypothetical protein	--	--	--	--	--	--	--	--	--	--	--	--	--	--	--
AB1267	Hypothetical protein	--	--	--	--	--	--	--	--	--	--	--	--	--	--	--
AB1269	Hypothetical protein	--	--	--	--	--	--	--	--	--	--	--	--	--	--	--
AB1270	Hypothetical protein	--	--	--	--	--	--	--	--	--	--	--	--	--	--	--
AB1274	Hypothetical protein	--	--	--	--	--	--	--	--	--	--	--	--	--	--	--
AB1288	Hypothetical protein	--	--	--	--	--	--	--	--	--	--	--	--	--	--	--
AB1303	Hypothetical protein	--	--	--	--	--	--	--	--	--	--	--	--	--	--	--
AB1304	Hypothetical protein	--	--	--	--	--	--	--	--	--	--	--	--	--	--	--
AB1310	Hypothetical protein	--	--	--	--	--	--	--	--	--	--	--	--	--	--	--
AB1313	Hypothetical protein	--	--	--	--	--	--	--	--	--	--	--	--	--	--	--
AB1315	Hypothetical protein	--	--	--	--	--	--	--	--	--	--	--	--	--	--	--
AB1319	Hypothetical protein	--	--	--	--	--	--	--	--	--	--	--	--	--	--	--
AB1324	Hypothetical protein	--	--	--	--	--	--	--	--	--	--	--	--	--	--	--
AB1326	Hypothetical protein	--	--	--	--	46	--	--	--	--	--	--	--	--	--	--
AB1331	Hypothetical protein	--	--	--	--	--	--	--	--	--	--	--	--	--	--	--
AB1332	Hypothetical protein	--	--	--	--	--	--	--	--	--	--	--	--	--	--	--
AB1333	Hypothetical protein	--	--	--	--	--	--	--	--	--	--	--	--	--	--	--
AB1334	Hypothetical protein	--	--	--	--	--	--	--	--	--	--	--	--	--	--	--
AB1336	Hypothetical protein	--	--	--	--	--	--	--	--	--	--	--	--	--	--	--
AB1338	Hypothetical protein	--	--	--	--	--	--	--	--	--	--	--	--	--	--	--
AB1339	Hypothetical protein	--	--	--	--	--	--	--	--	--	--	--	--	--	--	--
AB1340	Hypothetical protein	--	--	--	--	--	--	--	--	--	--	--	--	--	--	--
AB1342	Hypothetical protein	--	--	--	--	--	--	--	--	--	--	--	--	--	--	--
AB1343	Hypothetical protein	--	--	--	--	--	--	--	--	--	--	--	--	--	--	--
AB1344	Hypothetical protein	--	--	--	--	--	--	--	--	--	--	--	--	--	--	--
AB1345	Hypothetical protein	--	--	--	--	--	--	--	--	--	--	--	--	--	--	--
AB1346	Hypothetical protein	--	--	--	--	--	--	--	--	--	--	--	--	--	--	--
AB1347	Hypothetical protein	--	--	--	--	--	--	--	--	--	--	--	--	--	--	--
AB1348	Hypothetical protein	--	--	--	--	--	--	--	--	--	--	--	--	--	--	--
AB1350	Hypothetical protein	--	--	--	--	--	--	--	--	--	--	--	--	--	--	--
AB1351	Hypothetical protein	--	--	--	--	--	--	--	--	--	--	--	--	--	--	--
AB1352	Hypothetical protein	--	--	--	--	--	--	--	--	--	34	--	--	--	--	--
AB1353	Hypothetical protein	--	--	--	--	--	--	--	--	--	--	--	--	--	--	--
AB1354	Hypothetical protein	--	--	--	--	--	--	--	--	--	--	--	--	--	--	--
AB1355	Hypothetical protein	--	--	--	--	--	--	--	--	--	--	--	--	--	--	--
AB1356	Hypothetical protein	--	--	--	--	--	--	--	--	--	--	--	--	--	--	--
AB1357	Hypothetical protein	--	--	--	--	--	--	--	--	--	--	--	--	--	--	--
AB1358	Hypothetical protein	--	--	--	--	--	--	--	--	--	--	--	--	--	--	--
AB1361	Hypothetical protein	--	--	--	--	--	--	--	--	--	--	--	--	--	--	--

AB1381	Hypothetical protein	42	--	--	38	43	--	--	--	--	47	--	52	--	44	--
AB1384	Hypothetical protein	--	--	--	--	--	--	--	--	--	--	--	--	--	--	--
AB1392	Hypothetical protein	--	--	--	--	--	--	--	--	--	--	--	--	--	--	--
AB1401	Hypothetical protein	--	--	--	--	--	--	--	--	--	--	--	--	--	--	--
AB1404	Hypothetical protein	26	--	--	33	--	--	--	--	--	--	--	--	--	--	--
AB1405	Hypothetical protein	--	--	--	--	--	--	--	--	--	--	--	--	--	--	--
AB1406	Hypothetical protein	--	--	--	--	--	--	--	--	--	--	--	--	--	--	--
AB1407	Hypothetical protein	--	--	--	--	--	--	--	--	--	--	--	--	--	--	--
AB1422	Hypothetical protein	--	--	--	--	--	--	--	--	--	--	--	--	--	--	--
AB1426	Hypothetical protein	--	--	--	--	--	--	--	--	--	--	--	--	--	--	--
AB1428	Hypothetical protein	--	--	--	--	--	--	--	--	--	--	--	--	--	--	--
AB1429	Hypothetical protein	--	--	--	--	--	--	--	--	--	--	--	--	--	--	--
AB1473	Hypothetical protein	--	--	--	--	--	--	--	--	--	--	43	--	--	--	--
AB1524	Hypothetical protein	--	--	--	--	--	--	--	--	--	--	--	--	--	--	--
AB1525	Hypothetical protein	--	--	--	--	--	--	--	--	--	--	--	--	--	--	--
AB1528	Hypothetical protein	--	--	--	--	--	--	--	--	--	--	--	--	--	--	--
AB1529	Hypothetical protein	--	--	--	--	--	--	--	--	--	--	--	--	--	--	--
AB1531	Hypothetical protein	--	--	--	--	--	--	--	--	--	--	--	--	--	--	--
AB1537	Hypothetical protein	--	--	--	--	--	--	--	--	--	--	--	--	--	--	--
AB1539	Hypothetical protein	--	--	--	--	--	--	--	--	--	--	--	--	--	--	--
AB1551	Hypothetical protein	--	--	--	--	--	--	--	--	--	--	--	--	--	--	--
AB1563	Hypothetical protein	--	--	--	--	--	--	--	--	--	--	--	--	--	--	--
AB1569	Hypothetical protein	--	--	--	--	--	--	--	--	--	--	--	--	--	--	--
AB1582	Hypothetical protein	--	--	--	--	--	--	--	--	--	--	--	--	--	--	--
AB1614	Hypothetical protein	--	--	--	--	--	--	--	--	--	--	--	--	--	--	--
AB1629	Hypothetical protein	--	--	--	--	--	--	--	--	--	--	--	--	--	--	--
AB1710	Hypothetical protein	--	--	--	--	--	--	--	25	--	--	--	--	--	--	--
AB1712	Hypothetical protein	--	--	--	--	--	--	--	--	--	--	--	--	--	--	--
AB1723	Hypothetical protein	--	--	--	--	--	--	--	--	--	64	--	--	--	--	--
AB1725	Hypothetical protein	--	--	--	--	--	--	--	--	--	--	--	--	--	--	--
AB1726	Hypothetical protein	--	--	--	--	--	--	--	--	--	--	--	--	--	--	--
AB1727	Hypothetical protein	--	--	--	--	--	--	--	--	--	--	--	--	--	--	--
AB1729	Hypothetical protein	--	--	--	--	--	--	--	--	--	--	--	--	--	--	--
AB1733	Hypothetical protein	--	--	--	--	--	--	--	--	--	--	--	--	--	--	--
AB1734	Hypothetical protein	--	--	--	--	--	--	--	--	--	--	--	--	--	--	--
AB1736	Hypothetical protein	--	--	--	--	--	--	--	--	--	--	--	--	--	--	--
AB1741	Hypothetical protein	--	--	--	--	--	--	--	--	--	--	--	--	--	--	--
AB1746	Hypothetical protein	--	--	--	--	--	--	--	--	--	--	--	--	--	--	--
AB1747	Hypothetical protein	--	--	--	--	--	--	--	--	--	--	--	--	--	--	--
AB1748	Hypothetical protein	--	--	--	--	--	--	--	--	--	--	--	--	--	--	--
AB1758	Hypothetical protein	--	--	--	--	--	--	--	--	--	--	--	--	--	--	--
AB1767	Hypothetical protein	--	--	--	--	--	--	--	--	--	30	--	--	--	--	--
AB1768	Hypothetical protein	--	--	--	--	--	--	--	--	--	--	--	--	--	--	--
AB1773	Hypothetical protein	--	--	--	--	--	--	--	--	--	--	--	--	--	--	--
AB1785	Hypothetical protein	--	--	--	--	--	--	--	--	--	--	--	--	--	--	--
AB1804	Hypothetical protein	--	--	--	--	--	--	--	--	--	--	--	--	--	--	--
AB1816	Hypothetical protein	--	--	--	--	--	--	--	--	--	--	--	--	--	--	--

AB1843	Hypothetical protein	--	--	--	--	--	--	--	--	--	--	--	--	--	--	--
AB1850	Hypothetical protein	--	--	--	--	--	--	--	--	--	--	--	--	--	--	--
AB1853	Hypothetical protein	--	--	--	--	--	--	--	--	--	--	--	--	--	--	--
AB1861	Hypothetical protein	--	--	--	--	--	--	--	--	--	--	--	--	--	--	--
AB1871	Hypothetical protein	--	--	--	--	--	--	--	--	--	--	--	--	--	--	--
AB1876	Hypothetical protein	--	--	--	--	--	--	--	--	--	--	--	--	--	--	--
AB1879	Hypothetical protein	--	--	--	--	--	--	--	--	--	--	--	--	--	--	--
AB1881	Hypothetical protein	--	--	--	--	--	--	--	--	--	--	--	--	--	--	--
AB1882	Hypothetical protein	--	--	--	--	--	--	--	--	--	--	--	--	--	--	--
AB1886	Hypothetical protein	--	--	--	--	--	--	--	--	--	34	25	--	--	--	--
AB1916	Hypothetical protein	--	--	--	--	--	--	--	--	--	--	--	--	--	--	--
AB1940	Hypothetical protein	--	--	--	--	--	--	--	--	--	--	--	--	--	--	--
AB1943	Hypothetical protein	--	--	--	--	--	--	--	--	--	--	--	--	--	--	--
AB1944	Hypothetical protein	31	31	--	--	26	--	--	--	--	--	--	--	--	--	--
AB1945	Hypothetical protein	--	--	--	--	33	31	32	--	--	--	35	--	--	--	--
AB1949	Hypothetical protein	--	--	--	--	--	--	--	--	--	--	--	--	--	--	--
AB1962	Hypothetical protein	--	--	--	--	--	--	--	--	--	--	--	--	--	--	--
AB1963	Hypothetical protein	--	--	--	--	--	--	--	--	--	--	--	--	--	--	--
AB1975	Hypothetical protein	--	--	--	--	--	--	--	--	--	--	--	--	--	--	--
AB2004	Hypothetical protein	--	--	--	--	--	--	--	--	--	--	--	--	--	--	--
AB2012	Hypothetical protein	--	--	--	--	--	--	--	--	36	--	--	--	--	--	--
AB2016	Hypothetical protein	--	--	--	--	--	--	--	--	--	32	32	--	--	--	--
AB2059	Hypothetical protein	--	--	--	--	--	--	--	--	--	--	--	--	--	--	--
AB2063	Hypothetical protein	--	--	--	--	--	--	--	--	--	--	--	--	--	--	--
AB2065	Hypothetical protein	--	--	--	--	--	--	--	--	--	--	--	--	--	--	--
AB2066	Hypothetical protein	38	35	35	35	--	41	48	--	49	40	--	42	45	50	39
AB2084	Hypothetical protein	--	--	--	--	--	--	--	--	--	--	--	--	--	--	--
AB2092	Hypothetical protein	--	--	--	--	--	--	--	--	--	--	--	--	--	--	--
AB2093	Hypothetical protein	--	--	--	--	--	--	--	--	--	--	--	--	--	--	--
AB2095	Hypothetical protein	--	--	--	--	--	--	--	--	--	--	--	--	--	--	--
AB2096	Hypothetical protein	--	--	--	--	--	--	--	--	--	--	--	--	--	--	--
AB2097	Hypothetical protein	--	--	--	--	--	--	--	--	31	--	--	--	--	--	--
AB2098	Hypothetical protein	--	--	--	--	--	--	--	--	--	--	--	--	--	--	--
AB2143	Hypothetical protein	--	--	--	--	--	--	--	--	--	--	--	--	--	--	--
AB2145	Hypothetical protein	--	--	--	--	--	--	--	--	--	--	--	--	--	--	--
AB2153	Hypothetical protein	--	--	--	--	--	--	--	--	--	--	--	--	--	--	--
AB2164	Hypothetical protein	--	--	--	--	--	--	--	--	--	--	--	--	--	--	--
AB2166	Hypothetical protein	--	--	--	--	--	--	--	--	--	--	--	--	--	--	--
AB2167	Hypothetical protein	--	--	--	--	--	--	--	--	--	--	--	--	--	--	--
AB2180	Hypothetical protein	--	--	--	--	--	--	--	--	48	--	--	--	--	--	--
AB2195	Hypothetical protein	--	--	--	--	--	--	--	--	--	--	--	--	--	--	--
AB2208	Hypothetical protein	--	--	--	--	--	--	--	--	--	--	--	--	--	--	--
AB2217	Hypothetical protein	--	--	--	--	--	--	--	--	--	--	--	--	--	--	--
AB2218	Hypothetical protein	--	--	--	--	--	--	--	--	--	--	--	--	--	--	--
AB2219	Hypothetical protein	--	--	--	--	--	--	--	--	--	--	--	--	--	--	--
AB2228	Hypothetical protein	39	39	36	--	37	43	--	--	44	--	--	43	--	--	--
AB2231	Hypothetical protein	--	--	--	--	--	--	--	--	--	--	--	--	--	--	--

AB2237	Hypothetical protein	--	--	--	--	--	--	--	--	--	--	--	--	--	--	--
AB2248	Hypothetical protein	--	--	--	--	--	--	--	--	--	--	--	--	--	--	--
AB2253	Hypothetical protein	--	--	--	--	--	--	--	--	47	--	--	37	--	--	--
AB2271	Hypothetical protein	--	--	--	--	--	--	--	--	--	--	--	--	--	--	--
AB2291	Hypothetical protein	--	--	--	--	--	--	--	--	--	--	--	--	--	--	--
AB2297	Hypothetical protein	--	--	--	--	--	--	--	--	--	--	--	36	--	--	--
AB2308	Hypothetical protein	--	--	--	--	--	--	--	--	--	--	--	--	--	--	--
AB2309	Hypothetical protein	--	--	--	--	--	--	--	--	--	--	--	--	--	--	--

Predicted *Arcobacter* coding sequences were compared to the complete proteomes of *Campylobacter jejuni* strain RM1221 (Cj), *Campylobacter coli* strain RM2228 (Cc), *Campylobacter lari* strain RM2100 (Cl), *Campylobacter fetus* subsp. *fetus* strain 82-40 (Cf), *Campylobacter curvus* strain 525.92 (Cv), *Campylobacter concisus* strain 13826 (Cn), *Campylobacter hominis* strain ATCC BAA-381 (Ch), *Sulfurovum* sp. strain NBC37-1 (Sv), *Nitratiruptor* sp. strain SB155-2 (Ni), *Sulfuromonas denitrificans* strain ATCC 25259 (Sd), *Wolinella succinogenes* strain DSM 1740 (Ws), *Helicobacter acinonychis* strain Sheeba (Ha), *Helicobacter hepaticus* strain ATCC 51449 (Hh), *Helicobacter pylori* strain 26695 (Hp), or the draft proteome of *Campylobacter upsaliensis* RM3195 (Cu) by pairwise BLASTP comparisons. Numbers represent % amino acid identities to the most similar homolog; rDNA numbers represent % nucleic acid identities after comparison by BLASTN. Several large and small ribosomal subunit proteins were un-annotated in the *W. succinogenes, H. acinonychis* and *H. hepaticus* genomes; comparisons to these proteins were performed using TBLASTN and the sequence of each genome. Amino acid identities < 25% are represented by '---'.
