# Supplementary material for: The Complete Genome Sequence and Analysis of the Epsilonproteobacterium Arcobacter butzleri
Source: PLoS One. 2007 Dec 26;2(12):e1358. doi: 10.1371/journal.pone.0001358 (PMC2147049; doi:10.1371/journal.pone.0001358)
Supplement: Table S3 — Similarity of Arcobacter butzleri proteins to proteins from other taxa. The five best homologs (where applicable) for each protein in strain RM4018 are listed, as well as the Expect (E) value, % identity and % similarity. (4.71 MB PDF) [file pone.0001358.s005.pdf]

Table S3. Similarity of *Arcobacter butzleri* proteins to proteins from other taxa.

Locus	#	Gene	ID	Taxon		
AB0001	1	*dnaA*	gi|152991598|ref|YP_001357319.1|	Sulfurovum sp. NBC37-1	E-value = 2.00E-136	Identity = 60.24%
	2		gi|152989754|ref|YP_001355476.1|	Nitratiruptor sp. SB155-2	E-value = 3.00E-132	Identity = 57.93%
	3		gi|34556459|ref|NP_906274.1|	Wolinella succinogenes DSM 1740	E-value = 4.00E-132	Identity = 57.38%
	4		gi|154174998|ref|YP_001407305.1|	Campylobacter curvus 525.92	E-value = 2.00E-117	Identity = 52.76%
	5		gi|118474813|ref|YP_891211.1|	Campylobacter fetus subsp. fetus 82-40	E-value = 6.00E-113	Identity = 52.18%
AB0002	1	*dnaN*	gi|34556460|ref|NP_906275.1|	Wolinella succinogenes DSM 1740	E-value = 2.00E-86	Identity = 50.99%
	2		gi|152989755|ref|YP_001355477.1|	Nitratiruptor sp. SB155-2	E-value = 2.00E-81	Identity = 52.39%
	3		gi|152991599|ref|YP_001357320.1|	Sulfurovum sp. NBC37-1	E-value = 2.00E-81	Identity = 48.31%
	4		gi|78776203|ref|YP_392518.1|	Sulfuromonas denitrificans ATCC 33889	E-value = 4.00E-80	Identity = 51.27%
	5		gi|32266625|ref|NP_860657.1|	Helicobacter hepaticus ATCC 51449	E-value = 5.00E-76	Identity = 47.89%
AB0003	1	*gyrB*	gi|34556461|ref|NP_906276.1|	Wolinella succinogenes DSM 1740	E-value = 0	Identity = 69.99%
	2		gi|152991737|ref|YP_001357458.1|	Sulfurovum sp. NBC37-1	E-value = 0	Identity = 69.38%
	3		gi|152989756|ref|YP_001355478.1|	Nitratiruptor sp. SB155-2	E-value = 0	Identity = 71.35%
	4		gi|78776204|ref|YP_392519.1|	Sulfuromonas denitrificans ATCC 33889	E-value = 0	Identity = 69.78%
	5		gi|157165358|ref|YP_001465928.1|	Campylobacter concisus 13826	E-value = 0	Identity = 69.65%
AB0004	1		gi|152991475|ref|YP_001357197.1|	Nitratiruptor sp. SB155-2	E-value = 1.00E-83	Identity = 53.75%
	2		gi|157164867|ref|YP_001466727.1|	Campylobacter concisus 13826	E-value = 1.00E-79	Identity = 48.73%
	3		gi|32266652|ref|NP_860684.1|	Helicobacter hepaticus ATCC 51449	E-value = 1.00E-77	Identity = 48.62%
	4		gi|34556762|ref|NP_906577.1|	Wolinella succinogenes DSM 1740	E-value = 2.00E-77	Identity = 50.47%
	5		gi|118474851|ref|YP_891811.1|	Campylobacter fetus subsp. fetus 82-40	E-value = 6.00E-77	Identity = 50.95%
AB0005	1	*queF*	gi|152991735|ref|YP_001357456.1|	Sulfurovum sp. NBC37-1	E-value = 6.00E-47	Identity = 79.51%
	2		gi|149194735|ref|ZP_01871830.1|	Caminibacter mediatlanticus TB-2	E-value = 2.00E-42	Identity = 72.22%
	3		gi|78776205|ref|YP_392520.1|	Sulfuromonas denitrificans ATCC 33889	E-value = 1.00E-39	Identity = 62.40%
	4		gi|152989758|ref|YP_001355480.1|	Nitratiruptor sp. SB155-2	E-value = 4.00E-39	Identity = 63.20%
	5		gi|57241678|ref|ZP_00369623.1|	Campylobacter lari RM2100	E-value = 3.00E-38	Identity = 64.75%
AB0006	1			***** No matches found *****		
AB0007	1		gi|39995462|ref|NP_951413.1|	Geobacter sulfurreducens PCA	E-value = 1.00E-22	Identity = 43.94%
	2		gi|114776874|ref|ZP_01451917.1|	Mariprofundus ferrooxydans PV-1	E-value = 5.00E-22	Identity = 46.09%
	3		gi|118578932|ref|YP_900182.1|	Pelobacter propionicus DSM 2379	E-value = 4.00E-21	Identity = 41.73%
	4		gi|88861218|ref|ZP_01135851.1|	Pseudoalteromonas tunicata D2	E-value = 1.00E-20	Identity = 44.19%
	5		gi|119469676|ref|ZP_01612545.1|	Alteromonadales bacterium TW-7	E-value = 3.00E-20	Identity = 43.61%
AB0008	1		gi|154174057|ref|YP_001409001.1|	Campylobacter curvus 525.92	E-value = 3.00E-52	Identity = 39.46%
	2		gi|34557400|ref|NP_907215.1|	Wolinella succinogenes DSM 1740	E-value = 9.00E-52	Identity = 40.84%
	3		gi|118475227|ref|YP_891483.1|	Campylobacter fetus subsp. fetus 82-40	E-value = 1.00E-48	Identity = 44.06%
	4		gi|78777747|ref|YP_394062.1|	Sulfuromonas denitrificans ATCC 33889	E-value = 1.00E-48	Identity = 39.30%
	5		gi|34557502|ref|NP_907317.1|	Wolinella succinogenes DSM 1740	E-value = 9.00E-48	Identity = 39.25%
AB0009	1	*modD*	gi|152993679|ref|YP_001359400.1|	Sulfurovum sp. NBC37-1	E-value = 1.00E-101	Identity = 66.90%
	2		gi|149194352|ref|ZP_01871449.1|	Caminibacter mediatlanticus TB-2	E-value = 3.00E-78	Identity = 56.58%
	3		gi|154173712|ref|YP_001408984.1|	Campylobacter curvus 525.92	E-value = 3.00E-75	Identity = 52.30%
	4		gi|157164060|ref|YP_001467610.1|	Campylobacter concisus 13826	E-value = 1.00E-67	Identity = 48.23%
	5		gi|68551926|ref|ZP_00591319.1|	Prosthecochloris aestuarii DSM 271	E-value = 4.00E-67	Identity = 46.71%
AB0010	1	*modB*	gi|152993680|ref|YP_001359401.1|	Sulfurovum sp. NBC37-1	E-value = 4.00E-85	Identity = 73.01%
	2		gi|67938704|ref|ZP_00531225.1|	Chlorobium phaeobacteroides BS1	E-value = 1.00E-65	Identity = 58.90%
	3		gi|149194351|ref|ZP_01871448.1|	Caminibacter mediatlanticus TB-2	E-value = 2.00E-65	Identity = 64.71%
	4		gi|154148639|ref|YP_001405915.1|	Campylobacter hominis ATCC BAA-381	E-value = 2.00E-63	Identity = 61.16%

	5		gi|68551925|ref|ZP_00591318.1|	Prosthecochloris aestuarii DSM 271	E-value = 2.00E-61	Identity = 57.47%
AB0011	1		gi|152993681|ref|YP_001359402.1|	Sulfurovum sp. NBC37-1	E-value = 2.00E-20	Identity = 45.04%
	2		gi|68550335|ref|ZP_00589786.1|	Pelodictyon phaeoclathratiforme BU-1	E-value = 1.00E-12	Identity = 36.84%
	3		gi|119356277|ref|YP_910921.1|	Chlorobium phaeobacteroides DSM 266	E-value = 7.00E-12	Identity = 37.50%
	4		gi|67918868|ref|ZP_00512460.1|	Chlorobium limicola DSM 245	E-value = 6.00E-11	Identity = 34.38%
	5		gi|67938703|ref|ZP_00531224.1|	Chlorobium phaeobacteroides BS1	E-value = 4.00E-10	Identity = 31.01%
AB0012	1	*modA*	gi|154175064|ref|YP_001408986.1|	Campylobacter curvus 525.92	E-value = 4.00E-67	Identity = 57.81%
	2		gi|157164768|ref|YP_001467608.1|	Campylobacter concisus 13826	E-value = 3.00E-59	Identity = 50.85%
	3		gi|154149022|ref|YP_001405919.1|	Campylobacter hominis ATCC BAA-381	E-value = 2.00E-56	Identity = 49.34%
	4		gi|118474610|ref|YP_891564.1|	Campylobacter fetus subsp. fetus 82-40	E-value = 9.00E-56	Identity = 50.87%
	5		gi|57241524|ref|ZP_00369470.1|	Campylobacter lari RM2100	E-value = 6.00E-55	Identity = 50.61%
AB0013	1	*modE*	gi|152993683|ref|YP_001359404.1|	Sulfurovum sp. NBC37-1	E-value = 3.00E-54	Identity = 51.15%
	2		gi|67158371|ref|ZP_00419362.1|	Azotobacter vinelandii AvOP	E-value = 1.00E-44	Identity = 38.71%
	3		gi|152995007|ref|YP_001339842.1|	Marinomonas sp. MWYL1	E-value = 2.00E-43	Identity = 43.33%
	4		gi|94309512|ref|YP_582722.1|	Ralstonia metallidurans CH34	E-value = 2.00E-43	Identity = 42.68%
	5		gi|152981988|ref|YP_001355349.1|	Janthinobacterium sp. Marseille	E-value = 3.00E-42	Identity = 40.34%
AB0014	1		gi|152992875|ref|YP_001358596.1|	Sulfurovum sp. NBC37-1	E-value = 2.00E-105	Identity = 52.88%
	2		gi|152990675|ref|YP_001356397.1|	Nitratiruptor sp. SB155-2	E-value = 2.00E-86	Identity = 52.72%
	3		gi|149194629|ref|ZP_01871725.1|	Caminibacter mediatlanticus TB-2	E-value = 3.00E-67	Identity = 52.70%
	4		gi|34540274|ref|NP_904753.1|	Porphyromonas gingivalis W83	E-value = 3.00E-48	Identity = 35.63%
	5		gi|153809231|ref|ZP_01961899.1|	Bacteroides caccae ATCC 43185	E-value = 1.00E-47	Identity = 33.25%
AB0015	1		gi|30263453|ref|NP_845830.1|	Bacillus anthracis str. Ames	E-value = 2.00E-35	Identity = 35.82%
	2		gi|89206591|ref|ZP_01185147.1|	Bacillus weihenstephanensis KBAB4	E-value = 3.00E-35	Identity = 35.45%
	3		gi|47564625|ref|ZP_00235670.1|	Bacillus cereus G9241	E-value = 5.00E-35	Identity = 35.07%
	4		gi|52142032|ref|YP_084797.1|	Bacillus cereus E33L	E-value = 6.00E-35	Identity = 35.45%
	5		gi|118478757|ref|YP_895908.1|	Bacillus thuringiensis str. Al Hakam	E-value = 8.00E-35	Identity = 35.82%
AB0016	1		gi|119946284|ref|YP_943964.1|	Psychromonas ingrahamii 37	E-value = 5.00E-126	Identity = 67.53%
	2		gi|71280563|ref|YP_271535.1|	Colwellia psychrerythraea 34H	E-value = 8.00E-119	Identity = 62.44%
	3		gi|88799378|ref|ZP_01114956.1|	Reinekea sp. MED297	E-value = 3.00E-109	Identity = 57.96%
	4		gi|89899664|ref|YP_522135.1|	Rhodoferax ferrireducens T118	E-value = 3.00E-56	Identity = 35.70%
	5		gi|114562761|ref|YP_750274.1|	Shewanella frigidimarina NCIMB 400	E-value = 4.00E-54	Identity = 33.60%
AB0017	1			***** No matches found *****		
AB0018	1		gi|152989807|ref|YP_001355529.1|	Nitratiruptor sp. SB155-2	E-value = 2.00E-55	Identity = 42.33%
	2		gi|78778288|ref|YP_394603.1|	Sulfuromonas denitrificans ATCC 33889	E-value = 5.00E-54	Identity = 43.42%
	3		gi|149194782|ref|ZP_01871877.1|	Caminibacter mediatlanticus TB-2	E-value = 3.00E-53	Identity = 42.76%
	4		gi|157163990|ref|YP_001467817.1|	Campylobacter concisus 13826	E-value = 1.00E-51	Identity = 40.79%
	5		gi|154148734|ref|YP_001407253.1|	Campylobacter hominis ATCC BAA-381	E-value = 6.00E-50	Identity = 41.30%
AB0019	1	*purB*	gi|78778289|ref|YP_394604.1|	Sulfuromonas denitrificans ATCC 33889	E-value = 0	Identity = 84.39%
	2		gi|152993943|ref|YP_001359664.1|	Sulfurovum sp. NBC37-1	E-value = 0	Identity = 86.17%
	3		gi|34556845|ref|NP_906660.1|	Wolinella succinogenes DSM 1740	E-value = 0	Identity = 78.91%
	4		gi|149194784|ref|ZP_01871879.1|	Caminibacter mediatlanticus TB-2	E-value = 0	Identity = 78%
	5		gi|32266852|ref|NP_860884.1|	Helicobacter hepaticus ATCC 51449	E-value = 0	Identity = 74.83%
AB0020	1	*nrdA*	gi|152989805|ref|YP_001355527.1|	Nitratiruptor sp. SB155-2	E-value = 0	Identity = 72.54%
	2		gi|78778290|ref|YP_394605.1|	Sulfuromonas denitrificans ATCC 33889	E-value = 0	Identity = 70.62%
	3		gi|157165034|ref|YP_001467815.1|	Campylobacter concisus 13826	E-value = 0	Identity = 71.5%
	4		gi|152993944|ref|YP_001359665.1|	Sulfurovum sp. NBC37-1	E-value = 0	Identity = 70.28%
	5		gi|154174561|ref|YP_001407352.1|	Campylobacter curvus 525.92	E-value = 0	Identity = 69.44%
AB0021	1	*nrdB*	gi|157164305|ref|YP_001467763.1|	Campylobacter concisus 13826	E-value = 6.00E-134	Identity = 70.59%
	2		gi|118474076|ref|YP_891239.1|	Campylobacter fetus subsp. fetus 82-40	E-value = 1.00E-133	Identity = 70.29%

	3		gi|154174558|ref|YP_001409201.1|	Campylobacter curvus 525.92	E-value = 8.00E-133	Identity = 70.00%
	4		gi|57505748|ref|ZP_00371674.1|	Campylobacter upsaliensis RM3195	E-value = 2.00E-132	Identity = 69.12%
	5		gi|15791603|ref|NP_281426.1|	Campylobacter jejuni subsp. jejuni NCTC 11168	E-value = 3.00E-131	Identity = 69.12%
AB0022	1		gi|153215749|ref|ZP_01950118.1|	Vibrio cholerae 1587	E-value = 2.00E-20	Identity = 31.27%
	2		gi|153216893|ref|ZP_01950657.1|	Vibrio cholerae 1587	E-value = 1.00E-12	Identity = 27.47%
	3		gi|104782694|ref|YP_609192.1|	Pseudomonas entomophila L48	E-value = 6.00E-11	Identity = 25.00%
AB0023	1		gi|56420405|ref|YP_147723.1|	Geobacillus kaustophilus HTA426	E-value = 9.00E-30	Identity = 42.64%
	2		gi|77359159|ref|YP_338734.1|	Pseudoalteromonas haloplanktis TAC125	E-value = 1.00E-29	Identity = 46.88%
	3		gi|149374593|ref|ZP_01892367.1|	Marinobacter algicola DG893	E-value = 2.00E-29	Identity = 46.72%
	4		gi|138895409|ref|YP_001125862.1|	Geobacillus thermodenitrificans NG80-2	E-value = 3.00E-29	Identity = 41.86%
	5		gi|134105196|pdb|2OIW|A	Geobacillus stearothermophilus	E-value = 6.00E-28	Identity = 41.41%
AB0024	1			***** No matches found *****		
AB0025	1			***** No matches found *****		
AB0026	1		gi|84684106|ref|ZP_01012008.1|	Rhodobacterales bacterium HTCC2654	E-value = 3.00E-13	Identity = 26.15%
AB0027	1		gi|93006608|ref|YP_581045.1|	Psychrobacter cryohalolentis K5	E-value = 1.00E-125	Identity = 68.10%
	2		gi|95929023|ref|ZP_01311768.1|	Desulfuromonas acetoxidans DSM 684	E-value = 4.00E-92	Identity = 53.38%
	3		gi|119490232|ref|ZP_01622745.1|	Lyngbya sp. PCC 8106	E-value = 8.00E-92	Identity = 50.77%
	4		gi|88796036|ref|ZP_01111721.1|	Alteromonas macleodii 'Deep ecotype'	E-value = 3.00E-91	Identity = 50.00%
	5		gi|118580629|ref|YP_901879.1|	Pelobacter propionicus DSM 2379	E-value = 5.00E-89	Identity = 48.62%
AB0028	1		gi|1881336|dbj|BAA19363.1|	Bacillus subtilis	E-value = 1.00E-28	Identity = 52.38%
	2		gi|16077596|ref|NP_388410.1|	Bacillus subtilis subsp. subtilis str. 168	E-value = 1.00E-27	Identity = 54.95%
	3		gi|113940897|ref|ZP_01426715.1|	Herpetosiphon aurantiacus ATCC 23779	E-value = 4.00E-27	Identity = 49.51%
	4		gi|23128560|ref|ZP_00110403.1|	Nostoc punctiforme PCC 73102	E-value = 9.00E-26	Identity = 55.88%
	5		gi|68056146|ref|ZP_00540278.1|	Exiguobacterium sibiricum 255-15	E-value = 3.00E-25	Identity = 54.90%
AB0029	1	*gpsA*	gi|118474527|ref|YP_891950.1|	Campylobacter fetus subsp. fetus 82-40	E-value = 2.00E-102	Identity = 65.41%
	2		gi|78777528|ref|YP_393843.1|	Sulfuromonas denitrificans ATCC 33889	E-value = 2.00E-102	Identity = 64.07%
	3		gi|57168075|ref|ZP_00367214.1|	Campylobacter coli RM2228	E-value = 1.00E-98	Identity = 62.24%
	4		gi|154148876|ref|YP_001406504.1|	Campylobacter hominis ATCC BAA-381	E-value = 2.00E-98	Identity = 64.38%
	5		gi|86150853|ref|ZP_01069069.1|	Campylobacter jejuni subsp. jejuni 260.94	E-value = 2.00E-98	Identity = 63.01%
AB0030	1	*gatB*	gi|152990603|ref|YP_001356325.1|	Nitratiruptor sp. SB155-2	E-value = 0	Identity = 72.36%
	2		gi|34558292|ref|NP_908107.1|	Wolinella succinogenes DSM 1740	E-value = 0	Identity = 70.19%
	3		gi|78777531|ref|YP_393846.1|	Sulfuromonas denitrificans ATCC 33889	E-value = 0	Identity = 69.98%
	4		gi|32266295|ref|NP_860327.1|	Helicobacter hepaticus ATCC 51449	E-value = 0	Identity = 68.57%
	5		gi|154174942|ref|YP_001408363.1|	Campylobacter curvus 525.92	E-value = 0	Identity = 68.84%
AB0031	1		gi|34556608|ref|NP_906423.1|	Wolinella succinogenes DSM 1740	E-value = 9.00E-36	Identity = 31.16%
	2		gi|152991383|ref|YP_001357105.1|	Nitratiruptor sp. SB155-2	E-value = 6.00E-34	Identity = 32.49%
	3		gi|57241512|ref|ZP_00369458.1|	Campylobacter lari RM2100	E-value = 1.00E-33	Identity = 30.18%
	4		gi|149194720|ref|ZP_01871815.1|	Caminibacter mediatlanticus TB-2	E-value = 2.00E-29	Identity = 33.21%
	5		gi|118474339|ref|YP_892696.1|	Campylobacter fetus subsp. fetus 82-40	E-value = 3.00E-29	Identity = 29.84%
AB0032	1		gi|78358643|ref|YP_390092.1|	Desulfovibrio desulfuricans G20	E-value = 0	Identity = 53.79%
	2		gi|120603763|ref|YP_968163.1|	Desulfovibrio vulgaris subsp. vulgaris DP4	E-value = 0	Identity = 50.9%
	3		gi|46578669|ref|YP_009477.1|	Desulfovibrio vulgaris subsp. vulgaris str. Hildenborough	E-value = 0	Identity = 50.79%
	4		gi|152994114|ref|YP_001338949.1|	Marinomonas sp. MWYL1	E-value = 0	Identity = 50.37%
	5		gi|119945154|ref|YP_942834.1|	Psychromonas ingrahamii 37	E-value = 0	Identity = 50.16%
AB0033	1	*lctP*	gi|126359878|ref|ZP_01716869.1|	Pseudomonas putida GB-1	E-value = 0	Identity = 68.23%
	2		gi|104780003|ref|YP_606501.1|	Pseudomonas entomophila L48	E-value = 0	Identity = 68.23%
	3		gi|26991418|ref|NP_746843.1|	Pseudomonas putida KT2440	E-value = 0	Identity = 68.05%
	4		gi|119859830|ref|ZP_01641240.1|	Pseudomonas putida W619	E-value = 0	Identity = 68.59%
	5		gi|67158701|ref|ZP_00419562.1|	Azotobacter vinelandii AvOP	E-value = 0	Identity = 67.08%

AB0034	1		gi|34557072|ref|NP_906887.1|	Wolinella succinogenes DSM 1740	E-value = 8.00E-41	Identity = 41.63%
	2		gi|34556569|ref|NP_906384.1|	Wolinella succinogenes DSM 1740	E-value = 3.00E-33	Identity = 43.54%
	3		gi|152990276|ref|YP_001355998.1|	Nitratiruptor sp. SB155-2	E-value = 1.00E-28	Identity = 35.24%
	4		gi|78777655|ref|YP_393970.1|	Sulfuromonas denitrificans ATCC 33889	E-value = 3.00E-28	Identity = 36.45%
	5		gi|152992275|ref|YP_001357996.1|	Sulfurovum sp. NBC37-1	E-value = 7.00E-28	Identity = 35.48%
AB0035	1		gi|34557073|ref|NP_906888.1|	Wolinella succinogenes DSM 1740	E-value = 2.00E-103	Identity = 32.50%
	2		gi|51244877|ref|YP_064761.1|	Desulfotalea psychrophila LSv54	E-value = 3.00E-66	Identity = 26.29%
	3		gi|118746174|ref|ZP_01594125.1|	Geobacter lovleyi SZ	E-value = 3.00E-49	Identity = 25.83%
AB0036	1		gi|150005398|ref|YP_001300142.1|	Bacteroides vulgatus ATCC 8482	E-value = 3.00E-37	Identity = 43.88%
	2		gi|149174457|ref|ZP_01853083.1|	Planctomyces maris DSM 8797	E-value = 5.00E-37	Identity = 41.79%
	3		gi|87311926|ref|ZP_01094037.1|	Blastopirellula marina DSM 3645	E-value = 8.00E-37	Identity = 41.79%
	4		gi|156107658|gb|EDO09403.1|	Bacteroides ovatus ATCC 8483	E-value = 2.00E-36	Identity = 42.78%
	5		gi|53714877|ref|YP_100869.1|	Bacteroides fragilis YCH46	E-value = 3.00E-36	Identity = 43.52%
AB0037	1		gi|87311927|ref|ZP_01094038.1|	Blastopirellula marina DSM 3645	E-value = 6.00E-177	Identity = 63.31%
	2		gi|149174456|ref|ZP_01853082.1|	Planctomyces maris DSM 8797	E-value = 1.00E-172	Identity = 62.47%
	3		gi|32474928|ref|NP_867922.1|	Rhodopirellula baltica SH 1	E-value = 7.00E-164	Identity = 60.94%
	4		gi|86159398|ref|YP_466183.1|	Anaeromyxobacter dehalogenans 2CP-C	E-value = 2.00E-163	Identity = 61.30%
	5		gi|153004302|ref|YP_001378627.1|	Anaeromyxobacter sp. Fw109-5	E-value = 2.00E-158	Identity = 59.72%
AB0038	1		gi|154492498|ref|ZP_02032124.1|	Parabacteroides merdae ATCC 43184	E-value = 8.00E-76	Identity = 54.69%
	2		gi|149174455|ref|ZP_01853081.1|	Planctomyces maris DSM 8797	E-value = 2.00E-75	Identity = 51.85%
	3		gi|150005396|ref|YP_001300140.1|	Bacteroides vulgatus ATCC 8482	E-value = 4.00E-75	Identity = 55.28%
	4		gi|53714879|ref|YP_100871.1|	Bacteroides fragilis YCH46	E-value = 2.00E-73	Identity = 53.06%
	5		gi|29349863|ref|NP_813366.1|	Bacteroides thetaiotaomicron VPI-5482	E-value = 2.00E-73	Identity = 52.24%
AB0039	1		gi|78776348|ref|YP_392663.1|	Sulfuromonas denitrificans ATCC 33889	E-value = 3.00E-59	Identity = 81.82%
	2		gi|157311519|ref|YP_001469562.1|	Enterobacteria phage Phi1	E-value = 4.00E-28	Identity = 44.08%
	3		gi|33620697|ref|NP_891789.1|	Enterobacteria phage RB49	E-value = 4.00E-28	Identity = 43.42%
	4		gi|109290173|ref|YP_656422.1|	Aeromonas phage 25	E-value = 1.00E-27	Identity = 48.20%
	5		gi|34419354|ref|NP_899367.1|	Vibrio phage KVP40	E-value = 1.00E-26	Identity = 47.83%
AB0040	1	*accB*	gi|152991386|ref|YP_001357108.1|	Nitratiruptor sp. SB155-2	E-value = 2.00E-38	Identity = 54.78%
	2		gi|34557455|ref|NP_907270.1|	Wolinella succinogenes DSM 1740	E-value = 2.00E-30	Identity = 49.04%
	3		gi|149194766|ref|ZP_01871861.1|	Caminibacter mediatlanticus TB-2	E-value = 4.00E-30	Identity = 45.86%
	4		gi|57168201|ref|ZP_00367340.1|	Campylobacter coli RM2228	E-value = 2.00E-28	Identity = 46.20%
	5		gi|32265693|ref|NP_859725.1|	Helicobacter hepaticus ATCC 51449	E-value = 2.00E-27	Identity = 46.54%
AB0041	1	*accC1*	gi|34557456|ref|NP_907271.1|	Wolinella succinogenes DSM 1740	E-value = 2.00E-179	Identity = 68.64%
	2		gi|157164344|ref|YP_001467578.1|	Campylobacter concisus 13826	E-value = 1.00E-176	Identity = 68.55%
	3		gi|152991385|ref|YP_001357107.1|	Nitratiruptor sp. SB155-2	E-value = 5.00E-176	Identity = 69.00%
	4		gi|57241511|ref|ZP_00369457.1|	Campylobacter lari RM2100	E-value = 1.00E-175	Identity = 68.33%
	5		gi|149194767|ref|ZP_01871862.1|	Caminibacter mediatlanticus TB-2	E-value = 3.00E-175	Identity = 66.59%
AB0042	1		gi|152991450|ref|YP_001357172.1|	Nitratiruptor sp. SB155-2	E-value = 1.00E-162	Identity = 61.62%
	2		gi|152992584|ref|YP_001358305.1|	Sulfurovum sp. NBC37-1	E-value = 1.00E-154	Identity = 58.08%
	3		gi|149194827|ref|ZP_01871921.1|	Caminibacter mediatlanticus TB-2	E-value = 1.00E-149	Identity = 63.78%
	4		gi|78777961|ref|YP_394276.1|	Sulfuromonas denitrificans ATCC 33889	E-value = 2.00E-137	Identity = 50.57%
	5		gi|34557821|ref|NP_907636.1|	Wolinella succinogenes DSM 1740	E-value = 3.00E-131	Identity = 49.60%
AB0044	1		gi|152992712|ref|YP_001358433.1|	Sulfurovum sp. NBC37-1	E-value = 2.00E-151	Identity = 59.05%
	2		gi|78484888|ref|YP_390813.1|	Thiomicrospira crunogena XCL-2	E-value = 6.00E-142	Identity = 53.11%
	3		gi|118737207|ref|ZP_01585611.1|	Dinoroseobacter shibae DFL 12	E-value = 6.00E-136	Identity = 54.79%
	4		gi|89093329|ref|ZP_01166278.1|	Oceanospirillum sp. MED92	E-value = 2.00E-134	Identity = 52.31%
	5		gi|152990317|ref|YP_001356039.1|	Nitratiruptor sp. SB155-2	E-value = 4.00E-134	Identity = 54.21%
AB0045	1		gi|152991060|ref|YP_001356782.1|	Nitratiruptor sp. SB155-2	E-value = 6.00E-40	Identity = 44.21%

	2		gi|78776542|ref|YP_392857.1|	Sulfuromonas denitrificans ATCC 33889	E-value = 3.00E-35	Identity = 41.49%
	3		gi|34556551|ref|NP_906366.1|	Wolinella succinogenes DSM 1740	E-value = 4.00E-34	Identity = 39.67%
	4		gi|149194218|ref|ZP_01871315.1|	Caminibacter mediatlanticus TB-2	E-value = 4.00E-23	Identity = 39.66%
	5		gi|126654701|ref|ZP_01726235.1|	Cyanothece sp. CCY0110	E-value = 4.00E-09	Identity = 27.04%
AB0046	1	*asnB1*	gi|78776387|ref|YP_392702.1|	Sulfuromonas denitrificans ATCC 33889	E-value = 1.00E-159	Identity = 51.17%
	2		gi|34558461|ref|NP_908276.1|	Wolinella succinogenes DSM 1740	E-value = 5.00E-126	Identity = 43.17%
	3		gi|118475321|ref|YP_892746.1|	Campylobacter fetus subsp. fetus 82-40	E-value = 3.00E-125	Identity = 44.09%
	4		gi|156719141|ref|ZP_02060788.1|	Hydrogenobaculum sp. Y04AAS1	E-value = 7.00E-115	Identity = 39.35%
	5		gi|149193735|ref|ZP_01870833.1|	Caminibacter mediatlanticus TB-2	E-value = 7.00E-94	Identity = 40.17%
AB0047	1			***** No matches found *****		
AB0048	1		gi|149194867|ref|ZP_01871961.1|	Caminibacter mediatlanticus TB-2	E-value = 3.00E-44	Identity = 36.29%
	2		gi|34558072|ref|NP_907887.1|	Wolinella succinogenes DSM 1740	E-value = 3.00E-35	Identity = 26.90%
	3		gi|32267318|ref|NP_861350.1|	Helicobacter hepaticus ATCC 51449	E-value = 8.00E-32	Identity = 28.37%
	4		gi|121541143|ref|ZP_01672899.1|	Candidatus Desulfococcus oleovorans Hxd3	E-value = 7.00E-31	Identity = 29.35%
	5		gi|34498271|ref|NP_902486.1|	Chromobacterium violaceum ATCC 12472	E-value = 6.00E-29	Identity = 27.16%
AB0049	1		gi|152993432|ref|YP_001359153.1|	Sulfurovum sp. NBC37-1	E-value = 2.00E-33	Identity = 58.33%
	2		gi|34557582|ref|NP_907397.1|	Wolinella succinogenes DSM 1740	E-value = 2.00E-33	Identity = 54.17%
	3		gi|34556816|ref|NP_906631.1|	Wolinella succinogenes DSM 1740	E-value = 5.00E-33	Identity = 47.53%
	4		gi|109948211|ref|YP_665439.1|	Helicobacter acinonychis str. Sheeba	E-value = 7.00E-33	Identity = 56.64%
	5		gi|152989953|ref|YP_001355675.1|	Nitratiruptor sp. SB155-2	E-value = 5.00E-32	Identity = 54.86%
AB0050	1		gi|152989818|ref|YP_001355540.1|	Nitratiruptor sp. SB155-2	E-value = 8.00E-133	Identity = 58.91%
	2		gi|32267139|ref|NP_861171.1|	Helicobacter hepaticus ATCC 51449	E-value = 7.00E-127	Identity = 57.28%
	3		gi|78776412|ref|YP_392727.1|	Sulfuromonas denitrificans ATCC 33889	E-value = 2.00E-124	Identity = 57.91%
	4		gi|152993934|ref|YP_001359655.1|	Sulfurovum sp. NBC37-1	E-value = 2.00E-121	Identity = 56.12%
	5		gi|34557620|ref|NP_907435.1|	Wolinella succinogenes DSM 1740	E-value = 5.00E-121	Identity = 58.29%
AB0051	1	*rluB*	gi|152989816|ref|YP_001355538.1|	Nitratiruptor sp. SB155-2	E-value = 1.00E-72	Identity = 57.77%
	2		gi|15646068|ref|NP_208250.1|	Helicobacter pylori 26695	E-value = 6.00E-71	Identity = 55.82%
	3		gi|109946748|ref|YP_663976.1|	Helicobacter acinonychis str. Sheeba	E-value = 2.00E-70	Identity = 56.63%
	4		gi|15612417|ref|NP_224070.1|	Helicobacter pylori J99	E-value = 3.00E-70	Identity = 55.42%
	5		gi|34558267|ref|NP_908082.1|	Wolinella succinogenes DSM 1740	E-value = 3.00E-70	Identity = 53.26%
AB0052	1		gi|152989815|ref|YP_001355537.1|	Nitratiruptor sp. SB155-2	E-value = 1.00E-110	Identity = 63.75%
	2		gi|152993936|ref|YP_001359657.1|	Sulfurovum sp. NBC37-1	E-value = 2.00E-107	Identity = 62.81%
	3		gi|149194802|ref|ZP_01871896.1|	Caminibacter mediatlanticus TB-2	E-value = 5.00E-106	Identity = 65.94%
	4		gi|78776414|ref|YP_392729.1|	Sulfuromonas denitrificans ATCC 33889	E-value = 1.00E-105	Identity = 62.07%
	5		gi|154148842|ref|YP_001407261.1|	Campylobacter hominis ATCC BAA-381	E-value = 1.00E-104	Identity = 65.06%
AB0053	1		gi|34556520|ref|NP_906335.1|	Wolinella succinogenes DSM 1740	E-value = 0	Identity = 68.07%
	2		gi|152989814|ref|YP_001355536.1|	Nitratiruptor sp. SB155-2	E-value = 0	Identity = 68.95%
	3		gi|78778283|ref|YP_394598.1|	Sulfuromonas denitrificans ATCC 33889	E-value = 0	Identity = 63.04%
	4		gi|149194710|ref|ZP_01871805.1|	Caminibacter mediatlanticus TB-2	E-value = 0	Identity = 61%
	5		gi|157165115|ref|YP_001467823.1|	Campylobacter concisus 13826	E-value = 0	Identity = 66.95%
AB0054	1	*ksgA*	gi|78778284|ref|YP_394599.1|	Sulfuromonas denitrificans ATCC 33889	E-value = 1.00E-64	Identity = 52.34%
	2		gi|152989813|ref|YP_001355535.1|	Nitratiruptor sp. SB155-2	E-value = 9.00E-61	Identity = 48.84%
	3		gi|152993938|ref|YP_001359659.1|	Sulfurovum sp. NBC37-1	E-value = 7.00E-60	Identity = 48.83%
	4		gi|57236897|ref|YP_179850.1|	Campylobacter jejuni subsp. jejuni RM1221	E-value = 4.00E-59	Identity = 49.81%
	5		gi|148926802|ref|ZP_01810481.1|	Campylobacter jejuni subsp. jejuni CG8486	E-value = 5.00E-59	Identity = 49.81%
AB0055	1	*hisF1*	gi|78778285|ref|YP_394600.1|	Sulfuromonas denitrificans ATCC 33889	E-value = 5.00E-106	Identity = 76.68%
	2		gi|152993939|ref|YP_001359660.1|	Sulfurovum sp. NBC37-1	E-value = 9.00E-106	Identity = 75.59%
	3		gi|152989811|ref|YP_001355533.1|	Nitratiruptor sp. SB155-2	E-value = 2.00E-103	Identity = 76.49%
	4		gi|34556518|ref|NP_906333.1|	Wolinella succinogenes DSM 1740	E-value = 7.00E-102	Identity = 72.83%

	5		gi|118474977|ref|YP_892921.1|	Campylobacter fetus subsp. fetus 82-40	E-value = 2.00E-100	Identity = 73.52%
AB0056	1			***** No matches found *****		
AB0057	1		gi|78778286|ref|YP_394601.1|	Sulfuromonas denitrificans ATCC 33889	E-value = 1.00E-57	Identity = 68.86%
	2		gi|152989810|ref|YP_001355532.1|	Nitratiruptor sp. SB155-2	E-value = 2.00E-53	Identity = 60.92%
	3		gi|149194714|ref|ZP_01871809.1|	Caminibacter mediatlanticus TB-2	E-value = 7.00E-51	Identity = 62.92%
	4		gi|152993940|ref|YP_001359661.1|	Sulfurovum sp. NBC37-1	E-value = 4.00E-50	Identity = 59.20%
	5		gi|34556517|ref|NP_906332.1|	Wolinella succinogenes DSM 1740	E-value = 8.00E-40	Identity = 48.59%
AB0058	1		gi|78778287|ref|YP_394602.1|	Sulfuromonas denitrificans ATCC 33889	E-value = 2.00E-133	Identity = 64.94%
	2		gi|152993941|ref|YP_001359662.1|	Sulfurovum sp. NBC37-1	E-value = 1.00E-128	Identity = 64.57%
	3		gi|34556516|ref|NP_906331.1|	Wolinella succinogenes DSM 1740	E-value = 6.00E-124	Identity = 64.27%
	4		gi|152989809|ref|YP_001355531.1|	Nitratiruptor sp. SB155-2	E-value = 8.00E-124	Identity = 63.04%
	5		gi|157164532|ref|YP_001467818.1|	Campylobacter concisus 13826	E-value = 4.00E-122	Identity = 60.51%
AB0060	1	*gltX1*	gi|154147872|ref|YP_001405724.1|	Campylobacter hominis ATCC BAA-381	E-value = 1.00E-157	Identity = 60.13%
	2		gi|118475134|ref|YP_892695.1|	Campylobacter fetus subsp. fetus 82-40	E-value = 6.00E-155	Identity = 59.78%
	3		gi|152991704|ref|YP_001357425.1|	Sulfurovum sp. NBC37-1	E-value = 8.00E-155	Identity = 59.74%
	4		gi|34558490|ref|NP_908305.1|	Wolinella succinogenes DSM 1740	E-value = 4.00E-153	Identity = 59.44%
	5		gi|86150731|ref|ZP_01068947.1|	Campylobacter jejuni subsp. jejuni 260.94	E-value = 2.00E-151	Identity = 58.79%
AB0061	1	*rbpA*	gi|146328793|ref|YP_001209360.1|	Dichelobacter nodosus VCS1703A	E-value = 6.00E-21	Identity = 61.18%
	2		gi|150385428|ref|ZP_01924054.1|	Victivallis vadensis ATCC BAA-548	E-value = 2.00E-20	Identity = 58.33%
	3		gi|78355840|ref|YP_387289.1|	Desulfovibrio desulfuricans G20	E-value = 3.00E-20	Identity = 59.52%
	4		gi|68552619|ref|ZP_00592006.1|	Prosthecochloris aestuarii DSM 271	E-value = 5.00E-20	Identity = 55.68%
	5		gi|145219698|ref|YP_001130407.1|	Prosthecochloris vibrioformis DSM 265	E-value = 8.00E-20	Identity = 59.77%
AB0062	1		gi|78777082|ref|YP_393397.1|	Sulfuromonas denitrificans ATCC 33889	E-value = 4.00E-47	Identity = 45.87%
	2		gi|152992275|ref|YP_001357996.1|	Sulfurovum sp. NBC37-1	E-value = 6.00E-45	Identity = 44.70%
	3		gi|57242734|ref|ZP_00370671.1|	Campylobacter upsaliensis RM3195	E-value = 6.00E-42	Identity = 45.16%
	4		gi|34557806|ref|NP_907621.1|	Wolinella succinogenes DSM 1740	E-value = 1.00E-41	Identity = 45.83%
	5		gi|154174037|ref|YP_001408153.1|	Campylobacter curvus 525.92	E-value = 2.00E-40	Identity = 44.91%
AB0063	1		gi|152993451|ref|YP_001359172.1|	Sulfurovum sp. NBC37-1	E-value = 6.00E-66	Identity = 36.57%
	2		gi|152992979|ref|YP_001358700.1|	Sulfurovum sp. NBC37-1	E-value = 4.00E-50	Identity = 34.03%
	3		gi|152992381|ref|YP_001358102.1|	Sulfurovum sp. NBC37-1	E-value = 9.00E-48	Identity = 31.84%
	4		gi|32267156|ref|NP_861188.1|	Helicobacter hepaticus ATCC 51449	E-value = 2.00E-46	Identity = 34.64%
	5		gi|15645976|ref|NP_208156.1|	Helicobacter pylori 26695	E-value = 1.00E-45	Identity = 33.77%
AB0064	1		gi|78777078|ref|YP_393393.1|	Sulfuromonas denitrificans ATCC 33889	E-value = 4.00E-15	Identity = 37.80%
	2		gi|108563696|ref|YP_628012.1|	Helicobacter pylori HPAG1	E-value = 2.00E-13	Identity = 37.01%
	3		gi|15645939|ref|NP_208118.1|	Helicobacter pylori 26695	E-value = 4.00E-13	Identity = 36.22%
	4		gi|15612311|ref|NP_223964.1|	Helicobacter pylori J99	E-value = 7.00E-13	Identity = 36.22%
	5		gi|109948131|ref|YP_665359.1|	Helicobacter acinonychis str. Sheeba	E-value = 1.00E-11	Identity = 34.43%
AB0065	1			***** No matches found *****		
AB0066	1	*czcB*	gi|78777080|ref|YP_393395.1|	Sulfuromonas denitrificans ATCC 33889	E-value = 1.00E-45	Identity = 39.02%
	2		gi|109948129|ref|YP_665357.1|	Helicobacter acinonychis str. Sheeba	E-value = 8.00E-40	Identity = 36.30%
	3		gi|15645941|ref|NP_208120.1|	Helicobacter pylori 26695	E-value = 8.00E-40	Identity = 36.63%
	4		gi|15612313|ref|NP_223966.1|	Helicobacter pylori J99	E-value = 2.00E-39	Identity = 36.30%
	5		gi|108563698|ref|YP_628014.1|	Helicobacter pylori HPAG1	E-value = 7.00E-39	Identity = 35.97%
AB0067	1	*czcA*	gi|78777081|ref|YP_393396.1|	Sulfuromonas denitrificans ATCC 33889	E-value = 0	Identity = 62.9%
	2		gi|152992282|ref|YP_001358003.1|	Sulfurovum sp. NBC37-1	E-value = 0	Identity = 59.67%
	3		gi|152990648|ref|YP_001356370.1|	Nitratiruptor sp. SB155-2	E-value = 0	Identity = 55.81%
	4		gi|15612314|ref|NP_223967.1|	Helicobacter pylori J99	E-value = 0	Identity = 54.48%
	5		gi|108563699|ref|YP_628015.1|	Helicobacter pylori HPAG1	E-value = 0	Identity = 54.58%
AB0068	1		gi|34558489|ref|NP_908304.1|	Wolinella succinogenes DSM 1740	E-value = 6.00E-39	Identity = 48.90%

	2	gi|51894086|ref|YP_076777.1|	Symbiobacterium thermophilum IAM 14863	E-value = 2.00E-33	Identity = 47.34%
	3	gi|108757273|ref|YP_632528.1|	Myxococcus xanthus DK 1622	E-value = 3.00E-31	Identity = 42.29%
	4	gi|146299493|ref|YP_001194084.1|	Flavobacterium johnsoniae UW101	E-value = 1.00E-30	Identity = 45.20%
	5	gi|115376667|ref|ZP_01463896.1|	Stigmatella aurantiaca DW4/3-1	E-value = 1.00E-30	Identity = 41.28%
AB0070	1	gi|78776324|ref|YP_392639.1|	Sulfuromonas denitrificans ATCC 33889	E-value = 4.00E-87	Identity = 45.12%
	2	gi|152991742|ref|YP_001357463.1|	Sulfurovum sp. NBC37-1	E-value = 2.00E-70	Identity = 36.82%
	3	gi|157163885|ref|YP_001467601.1|	Campylobacter concisus 13826	E-value = 4.00E-68	Identity = 38.51%
	4	gi|118475280|ref|YP_891399.1|	Campylobacter fetus subsp. fetus 82-40	E-value = 7.00E-67	Identity = 39.59%
	5	gi|154174165|ref|YP_001409000.1|	Campylobacter curvus 525.92	E-value = 4.00E-66	Identity = 37.61%
AB0071	1	gi|152991034|ref|YP_001356756.1|	Nitratiruptor sp. SB155-2	E-value = 1.00E-21	Identity = 57.78%
	2	gi|152992279|ref|YP_001358000.1|	Sulfurovum sp. NBC37-1	E-value = 2.00E-17	Identity = 53.93%
	3	gi|34557977|ref|NP_907792.1|	Wolinella succinogenes DSM 1740	E-value = 2.00E-13	Identity = 41.96%
	4	gi|152993243|ref|YP_001358964.1|	Sulfurovum sp. NBC37-1	E-value = 2.00E-08	Identity = 34.91%
	5	gi|152992278|ref|YP_001357999.1|	Sulfurovum sp. NBC37-1	E-value = 9.00E-08	Identity = 36.70%
AB0072	1	gi|34557043|ref|NP_906858.1|	Wolinella succinogenes DSM 1740	E-value = 8.00E-113	Identity = 42.81%
	2	gi|152991575|ref|YP_001357297.1|	Nitratiruptor sp. SB155-2	E-value = 5.00E-96	Identity = 40.64%
	3	gi|152991640|ref|YP_001357361.1|	Sulfurovum sp. NBC37-1	E-value = 1.00E-81	Identity = 41.43%
	4	gi|149194149|ref|ZP_01871247.1|	Caminibacter mediatlanticus TB-2	E-value = 2.00E-78	Identity = 39.55%
	5	gi|110600022|ref|ZP_01388252.1|	Geobacter sp. FRC-32	E-value = 4.00E-30	Identity = 28.63%
AB0073	1		***** No matches found *****		
AB0074	1 *thrS*	gi|157414515|ref|YP_001481771.1|	Campylobacter jejuni subsp. jejuni 81116	E-value = 0	Identity = 68.01%
	2	gi|15791593|ref|NP_281416.1|	Campylobacter jejuni subsp. jejuni NCTC 11168	E-value = 0	Identity = 68.01%
	3	gi|57168347|ref|ZP_00367481.1|	Campylobacter coli RM2228	E-value = 0	Identity = 67.85%
	4	gi|86149734|ref|ZP_01067964.1|	Campylobacter jejuni subsp. jejuni CF93-6	E-value = 0	Identity = 68.01%
	5	gi|121612820|ref|YP_999926.1|	Campylobacter jejuni subsp. jejuni 81-176	E-value = 0	Identity = 68.01%
AB0075	1 *infC*	gi|78776268|ref|YP_392583.1|	Sulfuromonas denitrificans ATCC 33889	E-value = 4.00E-55	Identity = 68.82%
	2	gi|152989861|ref|YP_001355583.1|	Nitratiruptor sp. SB155-2	E-value = 7.00E-52	Identity = 67.25%
	3	gi|118474606|ref|YP_892821.1|	Campylobacter fetus subsp. fetus 82-40	E-value = 4.00E-51	Identity = 69.54%
	4	gi|15791594|ref|NP_281417.1|	Campylobacter jejuni subsp. jejuni NCTC 11168	E-value = 9.00E-51	Identity = 68.97%
	5	gi|86153689|ref|ZP_01071892.1|	Campylobacter jejuni subsp. jejuni HB93-13	E-value = 1.00E-50	Identity = 68.39%
AB0076	1 *rpmI*	gi|154149059|ref|YP_001406081.1|	Campylobacter hominis ATCC BAA-381	E-value = 1.00E-08	Identity = 73.44%
	2	gi|54036292|sp|Q7VJ07|RL35_HELHP	Helicobacter hepaticus	E-value = 1.00E-07	Identity = 66.15%
	3	gi|149194091|ref|ZP_01871189.1|	Caminibacter mediatlanticus TB-2	E-value = 2.00E-07	Identity = 71.43%
	4	gi|15611185|ref|NP_222836.1|	Helicobacter pylori J99	E-value = 3.00E-07	Identity = 67.69%
	5	gi|157165502|ref|YP_001465939.1|	Campylobacter concisus 13826	E-value = 5.00E-07	Identity = 67.74%
AB0077	1 *rplT*	gi|34557221|ref|NP_907036.1|	Wolinella succinogenes DSM 1740	E-value = 7.00E-35	Identity = 78.63%
	2	gi|149194092|ref|ZP_01871190.1|	Caminibacter mediatlanticus TB-2	E-value = 4.00E-34	Identity = 80.87%
	3	gi|157163866|ref|YP_001465938.1|	Campylobacter concisus 13826	E-value = 5.00E-33	Identity = 80.51%
	4	gi|152989863|ref|YP_001355585.1|	Nitratiruptor sp. SB155-2	E-value = 5.00E-33	Identity = 78.26%
	5	gi|154174891|ref|YP_001407370.1|	Campylobacter curvus 525.92	E-value = 7.00E-33	Identity = 81.36%
AB0078	1	gi|34557042|ref|NP_906857.1|	Wolinella succinogenes DSM 1740	E-value = 1.00E-73	Identity = 40.17%
	2	gi|78355726|ref|YP_387175.1|	Desulfovibrio desulfuricans G20	E-value = 2.00E-42	Identity = 32.75%
	3	gi|71908505|ref|YP_286092.1|	Dechloromonas aromatica RCB	E-value = 1.00E-37	Identity = 31.99%
	4	gi|94263912|ref|ZP_01287716.1|	delta proteobacterium MLMS-1	E-value = 5.00E-37	Identity = 28.83%
	5	gi|94267329|ref|ZP_01290848.1|	delta proteobacterium MLMS-1	E-value = 6.00E-37	Identity = 28.83%
AB0079	1	gi|152992922|ref|YP_001358643.1|	Sulfurovum sp. NBC37-1	E-value = 2.00E-11	Identity = 50.68%
AB0080	1	gi|152991643|ref|YP_001357364.1|	Sulfurovum sp. NBC37-1	E-value = 4.00E-172	Identity = 59.96%
	2	gi|78778276|ref|YP_394591.1|	Sulfuromonas denitrificans ATCC 33889	E-value = 6.00E-132	Identity = 52.13%
	3	gi|34556669|ref|NP_906484.1|	Wolinella succinogenes DSM 1740	E-value = 3.00E-117	Identity = 46.93%

	4		gi|152989908|ref|YP_001355630.1|	Nitratiruptor sp. SB155-2	E-value = 2.00E-90	Identity = 40.20%
	5		gi|152992921|ref|YP_001358642.1|	Sulfurovum sp. NBC37-1	E-value = 4.00E-89	Identity = 40.16%
AB0081	1	*mobA*	gi|152991614|ref|YP_001357335.1|	Sulfurovum sp. NBC37-1	E-value = 3.00E-32	Identity = 42.02%
	2		gi|78778213|ref|YP_394528.1|	Sulfuromonas denitrificans ATCC 33889	E-value = 7.00E-30	Identity = 40.53%
	3		gi|154174777|ref|YP_001409011.1|	Campylobacter curvus 525.92	E-value = 1.00E-28	Identity = 40.32%
	4		gi|157164201|ref|YP_001467596.1|	Campylobacter concisus 13826	E-value = 6.00E-28	Identity = 41.01%
	5		gi|34558294|ref|NP_908109.1|	Wolinella succinogenes DSM 1740	E-value = 1.00E-27	Identity = 40.00%
AB0082	1	*leuC*	gi|78778211|ref|YP_394526.1|	Sulfuromonas denitrificans ATCC 33889	E-value = 0	Identity = 79.95%
	2		gi|152991743|ref|YP_001357464.1|	Sulfurovum sp. NBC37-1	E-value = 0	Identity = 77.7%
	3		gi|34556978|ref|NP_906793.1|	Wolinella succinogenes DSM 1740	E-value = 0	Identity = 76.29%
	4		gi|152991410|ref|YP_001357132.1|	Nitratiruptor sp. SB155-2	E-value = 0	Identity = 77.7%
	5		gi|118474772|ref|YP_891400.1|	Campylobacter fetus subsp. fetus 82-40	E-value = 3.00E-180	Identity = 74.88%
AB0083	1	*lspA*	gi|34557217|ref|NP_907032.1|	Wolinella succinogenes DSM 1740	E-value = 5.00E-26	Identity = 50.00%
	2		gi|154174466|ref|YP_001407401.1|	Campylobacter curvus 525.92	E-value = 7.00E-23	Identity = 46.10%
	3		gi|157165011|ref|YP_001465965.1|	Campylobacter concisus 13826	E-value = 2.00E-22	Identity = 43.62%
	4		gi|57242726|ref|ZP_00370663.1|	Campylobacter upsaliensis RM3195	E-value = 2.00E-22	Identity = 43.42%
	5		gi|157414657|ref|YP_001481913.1|	Campylobacter jejuni subsp. jejuni 81116	E-value = 9.00E-22	Identity = 41.84%
AB0084	1	*glmM*	gi|152991661|ref|YP_001357382.1|	Sulfurovum sp. NBC37-1	E-value = 3.00E-171	Identity = 66.67%
	2		gi|152989858|ref|YP_001355580.1|	Nitratiruptor sp. SB155-2	E-value = 4.00E-168	Identity = 67.57%
	3		gi|157164280|ref|YP_001465966.1|	Campylobacter concisus 13826	E-value = 9.00E-168	Identity = 68.02%
	4		gi|154173619|ref|YP_001407402.1|	Campylobacter curvus 525.92	E-value = 1.00E-163	Identity = 67.79%
	5		gi|34557216|ref|NP_907031.1|	Wolinella succinogenes DSM 1740	E-value = 3.00E-163	Identity = 65.99%
AB0085	1	*rpsT*	gi|118474807|ref|YP_892836.1|	Campylobacter fetus subsp. fetus 82-40	E-value = 2.00E-12	Identity = 63.95%
	2		gi|57238624|ref|YP_179755.1|	Campylobacter jejuni subsp. jejuni RM1221	E-value = 1.00E-11	Identity = 63.95%
	3		gi|153951103|ref|YP_001398892.1|	Campylobacter jejuni subsp. doylei 269.97	E-value = 1.00E-11	Identity = 63.95%
	4		gi|78778238|ref|YP_394553.1|	Sulfuromonas denitrificans ATCC 33889	E-value = 1.00E-11	Identity = 59.30%
	5		gi|57241668|ref|ZP_00369613.1|	Campylobacter lari RM2100	E-value = 2.00E-11	Identity = 63.95%
AB0086	1	*prfA*	gi|78778239|ref|YP_394554.1|	Sulfuromonas denitrificans ATCC 33889	E-value = 2.00E-136	Identity = 71.83%
	2		gi|154174699|ref|YP_001407404.1|	Campylobacter curvus 525.92	E-value = 2.00E-133	Identity = 69.01%
	3		gi|88596258|ref|ZP_01099495.1|	Campylobacter jejuni subsp. jejuni 84-25	E-value = 5.00E-132	Identity = 71.27%
	4		gi|15792917|ref|NP_282740.1|	Campylobacter jejuni subsp. jejuni NCTC 11168	E-value = 5.00E-132	Identity = 71.27%
	5		gi|86152274|ref|ZP_01070485.1|	Campylobacter jejuni subsp. jejuni 260.94	E-value = 6.00E-132	Identity = 71.27%
AB0087	1			***** No matches found *****		
AB0088	1	*pstS*	gi|92113600|ref|YP_573528.1|	Chromohalobacter salexigens DSM 3043	E-value = 2.00E-104	Identity = 58.54%
	2		gi|34557337|ref|NP_907152.1|	Wolinella succinogenes DSM 1740	E-value = 7.00E-100	Identity = 56.52%
	3		gi|67918870|ref|ZP_00512462.1|	Chlorobium limicola DSM 245	E-value = 2.00E-92	Identity = 51.30%
	4		gi|78188479|ref|YP_378817.1|	Chlorobium chlorochromatii CaD3	E-value = 1.00E-90	Identity = 51.95%
	5		gi|83591937|ref|YP_425689.1|	Rhodospirillum rubrum ATCC 11170	E-value = 1.00E-90	Identity = 54.77%
AB0089	1		gi|149194928|ref|ZP_01872021.1|	Caminibacter mediatlanticus TB-2	E-value = 8.00E-132	Identity = 49.03%
	2		gi|144898329|emb|CAM75193.1|	Magnetospirillum gryphiswaldense MSR-1	E-value = 1.00E-114	Identity = 38.15%
	3		gi|46201018|ref|ZP_00055892.2|	Magnetospirillum magnetotacticum MS-1	E-value = 2.00E-100	Identity = 34.21%
	4		gi|118588603|ref|ZP_01546011.1|	Stappia aggregata IAM 12614	E-value = 2.00E-95	Identity = 35.04%
	5		gi|116251042|ref|YP_766880.1|	Rhizobium leguminosarum bv. viciae 3841	E-value = 5.00E-87	Identity = 32.28%
AB0090	1	*pstC*	gi|34557336|ref|NP_907151.1|	Wolinella succinogenes DSM 1740	E-value = 7.00E-96	Identity = 64.29%
	2		gi|92113601|ref|YP_573529.1|	Chromohalobacter salexigens DSM 3043	E-value = 7.00E-92	Identity = 61.30%
	3		gi|78188478|ref|YP_378816.1|	Chlorobium chlorochromatii CaD3	E-value = 4.00E-87	Identity = 58.93%
	4		gi|67918871|ref|ZP_00512463.1|	Chlorobium limicola DSM 245	E-value = 1.00E-82	Identity = 58.18%
	5		gi|69938337|ref|ZP_00632834.1|	Paracoccus denitrificans PD1222	E-value = 4.00E-76	Identity = 54.18%
AB0091	1	*pstA*	gi|34557335|ref|NP_907150.1|	Wolinella succinogenes DSM 1740	E-value = 7.00E-134	Identity = 62.47%

	2		gi|92113602|ref|YP_573530.1|	Chromohalobacter salexigens DSM 3043	E-value = 1.00E-124	Identity = 58.62%
	3		gi|78188477|ref|YP_378815.1|	Chlorobium chlorochromatii CaD3	E-value = 6.00E-110	Identity = 51.86%
	4		gi|67918872|ref|ZP_00512464.1|	Chlorobium limicola DSM 245	E-value = 3.00E-100	Identity = 51.55%
	5		gi|67939612|ref|ZP_00532107.1|	Chlorobium phaeobacteroides BS1	E-value = 1.00E-99	Identity = 47.30%
AB0092	1	*pstB*	gi|34557334|ref|NP_907149.1|	Wolinella succinogenes DSM 1740	E-value = 2.00E-106	Identity = 69.53%
	2		gi|92113603|ref|YP_573531.1|	Chromohalobacter salexigens DSM 3043	E-value = 3.00E-100	Identity = 66.67%
	3		gi|15669201|ref|NP_248006.1|	Methanocaldococcus jannaschii DSM 2661	E-value = 3.00E-100	Identity = 69.32%
	4		gi|45358661|ref|NP_988218.1|	Methanococcus maripaludis S2	E-value = 2.00E-96	Identity = 68.13%
	5		gi|150401003|ref|YP_001324769.1|	Methanococcus aeolicus Nankai-3	E-value = 1.00E-95	Identity = 67.98%
AB0093	1		gi|152990330|ref|YP_001356052.1|	Nitratiruptor sp. SB155-2	E-value = 3.00E-28	Identity = 36.41%
	2		gi|78778082|ref|YP_394397.1|	Sulfuromonas denitrificans ATCC 33889	E-value = 3.00E-25	Identity = 35.16%
	3		gi|152993844|ref|YP_001359565.1|	Sulfurovum sp. NBC37-1	E-value = 2.00E-20	Identity = 32.58%
	4		gi|144900226|emb|CAM77090.1|	Magnetospirillum gryphiswaldense MSR-1	E-value = 3.00E-07	Identity = 26.15%
	5		gi|114327578|ref|YP_744735.1|	Granulibacter bethesdensis CGDNIH1	E-value = 7.00E-07	Identity = 26.22%
AB0094	1			***** No matches found *****		
AB0095	1		gi|78777756|ref|YP_394071.1|	Sulfuromonas denitrificans ATCC 33889	E-value = 1.00E-53	Identity = 54.30%
	2		gi|34557339|ref|NP_907154.1|	Wolinella succinogenes DSM 1740	E-value = 3.00E-50	Identity = 53.39%
	3		gi|154148866|ref|YP_001406002.1|	Campylobacter hominis ATCC BAA-381	E-value = 5.00E-49	Identity = 53.64%
	4		gi|152991494|ref|YP_001357216.1|	Nitratiruptor sp. SB155-2	E-value = 2.00E-48	Identity = 54.09%
	5		gi|152993849|ref|YP_001359570.1|	Sulfurovum sp. NBC37-1	E-value = 1.00E-47	Identity = 50.00%
AB0096	1		gi|152991493|ref|YP_001357215.1|	Nitratiruptor sp. SB155-2	E-value = 1.00E-69	Identity = 35.95%
	2		gi|78777755|ref|YP_394070.1|	Sulfuromonas denitrificans ATCC 33889	E-value = 4.00E-69	Identity = 37.86%
	3		gi|34557340|ref|NP_907155.1|	Wolinella succinogenes DSM 1740	E-value = 1.00E-58	Identity = 35.23%
	4		gi|154148145|ref|YP_001406001.1|	Campylobacter hominis ATCC BAA-381	E-value = 7.00E-51	Identity = 34.42%
	5		gi|106885381|ref|ZP_01352742.1|	Clostridium phytofermentans ISDg	E-value = 2.00E-33	Identity = 27.03%
AB0097	1	*rplM*	gi|34558418|ref|NP_908233.1|	Wolinella succinogenes DSM 1740	E-value = 1.00E-51	Identity = 70.80%
	2		gi|152991784|ref|YP_001357505.1|	Sulfurovum sp. NBC37-1	E-value = 2.00E-51	Identity = 72.26%
	3		gi|32265997|ref|NP_860029.1|	Helicobacter hepaticus ATCC 51449	E-value = 7.00E-51	Identity = 70.07%
	4		gi|15792795|ref|NP_282618.1|	Campylobacter jejuni subsp. jejuni NCTC 11168	E-value = 4.00E-49	Identity = 71.85%
	5		gi|78776293|ref|YP_392608.1|	Sulfuromonas denitrificans ATCC 33889	E-value = 4.00E-49	Identity = 68.12%
AB0098	1	*rpsI*	gi|34558417|ref|NP_908232.1|	Wolinella succinogenes DSM 1740	E-value = 2.00E-41	Identity = 72.09%
	2		gi|32265998|ref|NP_860030.1|	Helicobacter hepaticus ATCC 51449	E-value = 5.00E-41	Identity = 72.87%
	3		gi|157164941|ref|YP_001466194.1|	Campylobacter concisus 13826	E-value = 1.00E-39	Identity = 66.67%
	4		gi|148925648|ref|ZP_01809336.1|	Campylobacter jejuni subsp. jejuni CG8486	E-value = 3.00E-39	Identity = 67.44%
	5		gi|152991350|ref|YP_001357072.1|	Nitratiruptor sp. SB155-2	E-value = 3.00E-39	Identity = 62.79%
AB0099	1	*appA*	gi|152991348|ref|YP_001357070.1|	Nitratiruptor sp. SB155-2	E-value = 1.00E-165	Identity = 56.28%
	2		gi|78776295|ref|YP_392610.1|	Sulfuromonas denitrificans ATCC 33889	E-value = 4.00E-153	Identity = 55.51%
	3		gi|78222448|ref|YP_384195.1|	Geobacter metallireducens GS-15	E-value = 1.00E-108	Identity = 38.54%
	4		gi|39996533|ref|NP_952484.1|	Geobacter sulfurreducens PCA	E-value = 8.00E-108	Identity = 37.93%
	5		gi|118581066|ref|YP_902316.1|	Pelobacter propionicus DSM 2379	E-value = 4.00E-107	Identity = 39.67%
AB0100	1		gi|78776296|ref|YP_392611.1|	Sulfuromonas denitrificans ATCC 33889	E-value = 9.00E-55	Identity = 54.03%
	2		gi|149195078|ref|ZP_01872170.1|	Caminibacter mediatlanticus TB-2	E-value = 4.00E-50	Identity = 50.48%
	3		gi|152991793|ref|YP_001357514.1|	Sulfurovum sp. NBC37-1	E-value = 8.00E-49	Identity = 47.62%
	4		gi|118474263|ref|YP_891593.1|	Campylobacter fetus subsp. fetus 82-40	E-value = 5.00E-47	Identity = 48.80%
	5		gi|154175079|ref|YP_001407585.1|	Campylobacter curvus 525.92	E-value = 5.00E-46	Identity = 44.91%
AB0102	1		gi|149909488|ref|ZP_01898142.1|	Moritella sp. PE36	E-value = 9.00E-100	Identity = 56.23%
	2		gi|27366025|ref|NP_761553.1|	Vibrio vulnificus CMCP6	E-value = 1.00E-99	Identity = 57.19%
	3		gi|153833073|ref|ZP_01985740.1|	Vibrio harveyi HY01	E-value = 1.00E-98	Identity = 55.31%
	4		gi|89094282|ref|ZP_01167224.1|	Oceanospirillum sp. MED92	E-value = 3.00E-98	Identity = 57.19%

	5		gi|86145890|ref|ZP_01064218.1|	Vibrio sp. MED222	E-value = 1.00E-97	Identity = 55.00%
AB0103	1		gi|149909489|ref|ZP_01898143.1|	Moritella sp. PE36	E-value = 8.00E-11	Identity = 31.01%
	2		gi|126175631|ref|YP_001051780.1|	Shewanella baltica OS155	E-value = 1.00E-10	Identity = 30.26%
	3		gi|153802269|ref|ZP_01956855.1|	Vibrio cholerae MZO-3	E-value = 1.00E-10	Identity = 32.89%
	4		gi|116217501|ref|ZP_01483300.1|	Vibrio cholerae RC385	E-value = 1.00E-10	Identity = 32.89%
	5		gi|153833102|ref|ZP_01985769.1|	Vibrio harveyi HY01	E-value = 1.00E-10	Identity = 31.33%
AB0104	1		gi|89094284|ref|ZP_01167226.1|	Oceanospirillum sp. MED92	E-value = 4.00E-171	Identity = 73.07%
	2		gi|156974698|ref|YP_001445605.1|	Vibrio harveyi ATCC BAA-1116	E-value = 2.00E-167	Identity = 67.53%
	3		gi|149190001|ref|ZP_01868279.1|	Vibrio shilonii AK1	E-value = 2.00E-167	Identity = 67.87%
	4		gi|28898427|ref|NP_798032.1|	Vibrio parahaemolyticus RIMD 2210633	E-value = 2.00E-166	Identity = 67.13%
	5		gi|116186986|ref|ZP_01476877.1|	Vibrio sp. Ex25	E-value = 3.00E-166	Identity = 67.13%
AB0105	1		gi|34557780|ref|NP_907595.1|	Wolinella succinogenes DSM 1740	E-value = 2.00E-39	Identity = 41.63%
	2		gi|118475245|ref|YP_891579.1|	Campylobacter fetus subsp. fetus 82-40	E-value = 5.00E-32	Identity = 38.39%
	3		gi|34558806|gb|AAQ75151.1|	Alvinella pompejana epibiont 6C6	E-value = 1.00E-30	Identity = 40.27%
	4		gi|152993952|ref|YP_001359673.1|	Sulfurovum sp. NBC37-1	E-value = 2.00E-30	Identity = 34.80%
	5		gi|154173869|ref|YP_001407525.1|	Campylobacter curvus 525.92	E-value = 1.00E-29	Identity = 35.71%
AB0106	1		gi|78777177|ref|YP_393492.1|	Sulfuromonas denitrificans ATCC 33889	E-value = 8.00E-35	Identity = 28.89%
AB0107	1	*cynT1*	gi|152990078|ref|YP_001355800.1|	Nitratiruptor sp. SB155-2	E-value = 1.00E-55	Identity = 49.52%
	2		gi|152991985|ref|YP_001357706.1|	Sulfurovum sp. NBC37-1	E-value = 4.00E-54	Identity = 48.58%
	3		gi|152990058|ref|YP_001355780.1|	Nitratiruptor sp. SB155-2	E-value = 1.00E-52	Identity = 48.28%
	4		gi|78776363|ref|YP_392678.1|	Sulfuromonas denitrificans ATCC 33889	E-value = 4.00E-50	Identity = 43.75%
	5		gi|46446692|ref|YP_008057.1|	Candidatus Protochlamydia amoebophila UWE25	E-value = 1.00E-44	Identity = 39.72%
AB0108	1		gi|126727075|ref|ZP_01742913.1|	Rhodobacterales bacterium HTCC2150	E-value = 4.00E-38	Identity = 29.79%
	2		gi|114762688|ref|ZP_01442122.1|	Roseovarius sp. HTCC2601	E-value = 7.00E-38	Identity = 31.29%
	3		gi|118738749|ref|ZP_01586830.1|	Dinoroseobacter shibae DFL 12	E-value = 1.00E-36	Identity = 30.99%
	4		gi|89067648|ref|ZP_01155102.1|	Oceanicola granulosus HTCC2516	E-value = 2.00E-36	Identity = 30.91%
	5		gi|110679433|ref|YP_682440.1|	Roseobacter denitrificans OCh 114	E-value = 2.00E-36	Identity = 27.68%
AB0109	1		gi|153836554|ref|ZP_01989221.1|	Vibrio parahaemolyticus AQ3810	E-value = 1.00E-08	Identity = 26.11%
	2		gi|27363498|ref|NP_759026.1|	Vibrio vulnificus CMCP6	E-value = 1.00E-07	Identity = 25.00%
	3		gi|29654064|ref|NP_819756.1|	Coxiella burnetii RSA 493	E-value = 3.00E-07	Identity = 27.56%
AB0110	1		gi|152992946|ref|YP_001358667.1|	Sulfurovum sp. NBC37-1	E-value = 2.00E-32	Identity = 31.83%
	2		gi|121612777|ref|YP_001001291.1|	Campylobacter jejuni subsp. jejuni 81-176	E-value = 5.00E-30	Identity = 35.08%
	3		gi|15792953|ref|NP_282776.1|	Campylobacter jejuni subsp. jejuni NCTC 11168	E-value = 9.00E-30	Identity = 35.08%
	4		gi|57238659|ref|YP_179790.1|	Campylobacter jejuni subsp. jejuni RM1221	E-value = 1.00E-29	Identity = 34.75%
	5		gi|153951405|ref|YP_001398925.1|	Campylobacter jejuni subsp. doylei 269.97	E-value = 3.00E-29	Identity = 34.10%
AB0111	1	*iamA*	gi|57240652|ref|ZP_00368600.1|	Campylobacter lari RM2100	E-value = 6.00E-58	Identity = 52.81%
	2		gi|108563875|ref|YP_628191.1|	Helicobacter pylori HPAG1	E-value = 3.00E-55	Identity = 46.93%
	3		gi|15646074|ref|NP_208256.1|	Helicobacter pylori 26695	E-value = 5.00E-55	Identity = 46.93%
	4		gi|154174209|ref|YP_001408229.1|	Campylobacter curvus 525.92	E-value = 6.00E-55	Identity = 49.12%
	5		gi|15612423|ref|NP_224076.1|	Helicobacter pylori J99	E-value = 2.00E-54	Identity = 46.49%
AB0112	1	*iamB*	gi|152992948|ref|YP_001358669.1|	Sulfurovum sp. NBC37-1	E-value = 2.00E-83	Identity = 45.14%
	2		gi|119945392|ref|YP_943072.1|	Psychromonas ingrahamii 37	E-value = 5.00E-81	Identity = 42.97%
	3		gi|57240651|ref|ZP_00368599.1|	Campylobacter lari RM2100	E-value = 1.00E-73	Identity = 46.09%
	4		gi|153951693|ref|YP_001398923.1|	Campylobacter jejuni subsp. doylei 269.97	E-value = 8.00E-72	Identity = 45.58%
	5		gi|57168794|ref|ZP_00367925.1|	Campylobacter coli RM2228	E-value = 2.00E-71	Identity = 45.68%
AB0113	1		gi|150025607|ref|YP_001296433.1|	Flavobacterium psychrophilum JIP02/86	E-value = 1.00E-20	Identity = 29.55%
	2		gi|152993839|ref|YP_001359560.1|	Sulfurovum sp. NBC37-1	E-value = 2.00E-13	Identity = 29.65%
AB0114	1		gi|146300757|ref|YP_001195348.1|	Flavobacterium johnsoniae UW101	E-value = 6.00E-28	Identity = 26.72%
AB0115	1	*luxS*	gi|57168077|ref|ZP_00367216.1|	Campylobacter coli RM2228	E-value = 8.00E-71	Identity = 77.64%

	2		gi|148926201|ref|ZP_01809886.1|	Campylobacter jejuni subsp. jejuni CG8486	E-value = 1.00E-70	Identity = 78.26%
	3		gi|86150303|ref|ZP_01068529.1|	Campylobacter jejuni subsp. jejuni CF93-6	E-value = 2.00E-70	Identity = 77.64%
	4		gi|86152712|ref|ZP_01070917.1|	Campylobacter jejuni subsp. jejuni HB93-13	E-value = 2.00E-70	Identity = 78.26%
	5		gi|57238070|ref|YP_179319.1|	Campylobacter jejuni subsp. jejuni RM1221	E-value = 3.00E-70	Identity = 77.64%
AB0116	1			***** No matches found *****		
AB0117	1		gi|78778127|ref|YP_394442.1|	Sulfuromonas denitrificans ATCC 33889	E-value = 1.00E-58	Identity = 49.17%
	2		gi|152991800|ref|YP_001357521.1|	Sulfurovum sp. NBC37-1	E-value = 7.00E-54	Identity = 46.50%
	3		gi|152991340|ref|YP_001357062.1|	Nitratiruptor sp. SB155-2	E-value = 6.00E-49	Identity = 48.29%
	4		gi|149195377|ref|ZP_01872462.1|	Caminibacter mediatlanticus TB-2	E-value = 8.00E-43	Identity = 50.74%
	5		gi|34556885|ref|NP_906700.1|	Wolinella succinogenes DSM 1740	E-value = 2.00E-38	Identity = 36.99%
AB0118	1	*dacA*	gi|34557129|ref|NP_906944.1|	Wolinella succinogenes DSM 1740	E-value = 7.00E-68	Identity = 53.87%
	2		gi|67938599|ref|ZP_00531122.1|	Chlorobium phaeobacteroides BS1	E-value = 3.00E-57	Identity = 50.00%
	3		gi|68551674|ref|ZP_00591069.1|	Prosthecochloris aestuarii DSM 271	E-value = 7.00E-55	Identity = 45.98%
	4		gi|78186778|ref|YP_374821.1|	Pelodictyon luteolum DSM 273	E-value = 4.00E-51	Identity = 47.81%
	5		gi|119357228|ref|YP_911872.1|	Chlorobium phaeobacteroides DSM 266	E-value = 1.00E-46	Identity = 46.29%
AB0119	1		gi|154173815|ref|YP_001408415.1|	Campylobacter curvus 525.92	E-value = 3.00E-37	Identity = 47.20%
	2		gi|157165760|ref|YP_001466666.1|	Campylobacter concisus 13826	E-value = 9.00E-34	Identity = 44.10%
	3		gi|118475733|ref|YP_891754.1|	Campylobacter fetus subsp. fetus 82-40	E-value = 2.00E-32	Identity = 48.68%
	4		gi|57240588|ref|ZP_00368537.1|	Campylobacter lari RM2100	E-value = 4.00E-32	Identity = 44.23%
	5		gi|154148116|ref|YP_001406642.1|	Campylobacter hominis ATCC BAA-381	E-value = 3.00E-30	Identity = 42.31%
AB0120	1		gi|78777959|ref|YP_394274.1|	Sulfuromonas denitrificans ATCC 33889	E-value = 2.00E-29	Identity = 55.12%
	2		gi|68552281|ref|ZP_00591672.1|	Prosthecochloris aestuarii DSM 271	E-value = 8.00E-26	Identity = 52.00%
	3		gi|146283551|ref|YP_001173704.1|	Pseudomonas stutzeri A1501	E-value = 2.00E-25	Identity = 50.81%
	4		gi|50122075|ref|YP_051242.1|	Erwinia carotovora subsp. atroseptica SCRI1043	E-value = 3.00E-25	Identity = 50.41%
	5		gi|32266558|ref|NP_860590.1|	Helicobacter hepaticus ATCC 51449	E-value = 7.00E-24	Identity = 45.31%
AB0121	1	*metH*	gi|78778225|ref|YP_394540.1|	Sulfuromonas denitrificans ATCC 33889	E-value = 0	Identity = 65.27%
	2		gi|152993401|ref|YP_001359122.1|	Sulfurovum sp. NBC37-1	E-value = 0	Identity = 62.52%
	3		gi|152990298|ref|YP_001356020.1|	Nitratiruptor sp. SB155-2	E-value = 0	Identity = 61.47%
	4		gi|34557599|ref|NP_907414.1|	Wolinella succinogenes DSM 1740	E-value = 0	Identity = 54.67%
	5		gi|86609654|ref|YP_478416.1|	Synechococcus sp. JA-2-3B'a(2-13)	E-value = 0	Identity = 41.05%
AB0122	1	*ilvE*	gi|78778071|ref|YP_394386.1|	Sulfuromonas denitrificans ATCC 33889	E-value = 2.00E-126	Identity = 70.68%
	2		gi|149194823|ref|ZP_01871917.1|	Caminibacter mediatlanticus TB-2	E-value = 8.00E-125	Identity = 69.84%
	3		gi|34556545|ref|NP_906360.1|	Wolinella succinogenes DSM 1740	E-value = 5.00E-123	Identity = 66.89%
	4		gi|152991284|ref|YP_001357006.1|	Nitratiruptor sp. SB155-2	E-value = 1.00E-122	Identity = 67.87%
	5		gi|57241400|ref|ZP_00369346.1|	Campylobacter lari RM2100	E-value = 2.00E-115	Identity = 64.59%
AB0123	1		gi|34556544|ref|NP_906359.1|	Wolinella succinogenes DSM 1740	E-value = 9.00E-101	Identity = 59.09%
	2		gi|152991285|ref|YP_001357007.1|	Nitratiruptor sp. SB155-2	E-value = 4.00E-99	Identity = 55.71%
	3		gi|149194824|ref|ZP_01871918.1|	Caminibacter mediatlanticus TB-2	E-value = 9.00E-92	Identity = 54.63%
	4		gi|154175268|ref|YP_001407529.1|	Campylobacter curvus 525.92	E-value = 6.00E-91	Identity = 55.98%
	5		gi|154148517|ref|YP_001406987.1|	Campylobacter hominis ATCC BAA-381	E-value = 2.00E-90	Identity = 54.17%
AB0124	1			***** No matches found *****		
AB0125	1	*hisI*	gi|78778069|ref|YP_394384.1|	Sulfuromonas denitrificans ATCC 33889	E-value = 1.00E-78	Identity = 68.16%
	2		gi|152991833|ref|YP_001357554.1|	Sulfurovum sp. NBC37-1	E-value = 3.00E-70	Identity = 63.35%
	3		gi|34556543|ref|NP_906358.1|	Wolinella succinogenes DSM 1740	E-value = 2.00E-69	Identity = 59.19%
	4		gi|152991286|ref|YP_001357008.1|	Nitratiruptor sp. SB155-2	E-value = 1.00E-68	Identity = 61.09%
	5		gi|149194825|ref|ZP_01871919.1|	Caminibacter mediatlanticus TB-2	E-value = 2.00E-67	Identity = 63.30%
AB0126	1			***** No matches found *****		
AB0127	1	*mrp*	gi|152990346|ref|YP_001356068.1|	Nitratiruptor sp. SB155-2	E-value = 9.00E-133	Identity = 66.49%
	2		gi|152992119|ref|YP_001357840.1|	Sulfurovum sp. NBC37-1	E-value = 1.00E-128	Identity = 63.86%

	3		gi|57504882|ref|ZP_00370857.1|	Campylobacter coli RM2228	E-value = 7.00E-117	Identity = 57.92%
	4		gi|153952410|ref|YP_001398887.1|	Campylobacter jejuni subsp. doylei 269.97	E-value = 8.00E-117	Identity = 58.20%
	5		gi|86152336|ref|ZP_01070547.1|	Campylobacter jejuni subsp. jejuni 260.94	E-value = 1.00E-116	Identity = 57.92%
AB0128	1	*thiC*	gi|152990345|ref|YP_001356067.1|	Nitratiruptor sp. SB155-2	E-value = 0	Identity = 82.54%
	2		gi|78777683|ref|YP_393998.1|	Sulfuromonas denitrificans ATCC 33889	E-value = 0	Identity = 83.14%
	3		gi|32267080|ref|NP_861112.1|	Helicobacter hepaticus ATCC 51449	E-value = 0	Identity = 76.24%
	4		gi|34558232|ref|NP_908047.1|	Wolinella succinogenes DSM 1740	E-value = 0	Identity = 77.32%
	5		gi|118475288|ref|YP_891612.1|	Campylobacter fetus subsp. fetus 82-40	E-value = 0	Identity = 75.8%
AB0129	1		gi|78777397|ref|YP_393712.1|	Sulfuromonas denitrificans ATCC 33889	E-value = 6.00E-32	Identity = 31.30%
	2		gi|78776743|ref|YP_393058.1|	Sulfuromonas denitrificans ATCC 33889	E-value = 9.00E-29	Identity = 31.32%
	3		gi|154175137|ref|YP_001407999.1|	Campylobacter curvus 525.92	E-value = 7.00E-27	Identity = 27.57%
	4		gi|34558171|ref|NP_907986.1|	Wolinella succinogenes DSM 1740	E-value = 2.00E-25	Identity = 26.89%
	5		gi|157165151|ref|YP_001467161.1|	Campylobacter concisus 13826	E-value = 1.00E-23	Identity = 27.11%
AB0130	1	*ispDF*	gi|152992118|ref|YP_001357839.1|	Sulfurovum sp. NBC37-1	E-value = 9.00E-113	Identity = 52.14%
	2		gi|149194421|ref|ZP_01871518.1|	Caminibacter mediatlanticus TB-2	E-value = 3.00E-109	Identity = 55.68%
	3		gi|118474192|ref|YP_891611.1|	Campylobacter fetus subsp. fetus 82-40	E-value = 9.00E-109	Identity = 53.49%
	4		gi|78777684|ref|YP_393999.1|	Sulfuromonas denitrificans ATCC 33889	E-value = 3.00E-108	Identity = 52.97%
	5		gi|152990344|ref|YP_001356066.1|	Nitratiruptor sp. SB155-2	E-value = 7.00E-107	Identity = 50.40%
AB0131	1		gi|78777685|ref|YP_394000.1|	Sulfuromonas denitrificans ATCC 33889	E-value = 1.00E-51	Identity = 41.67%
	2		gi|152992117|ref|YP_001357838.1|	Sulfurovum sp. NBC37-1	E-value = 1.00E-50	Identity = 41.41%
	3		gi|154175524|ref|YP_001408624.1|	Campylobacter curvus 525.92	E-value = 4.00E-49	Identity = 39.39%
	4		gi|152990343|ref|YP_001356065.1|	Nitratiruptor sp. SB155-2	E-value = 7.00E-48	Identity = 41.41%
	5		gi|157164723|ref|YP_001466559.1|	Campylobacter concisus 13826	E-value = 2.00E-47	Identity = 38.72%
AB0132	1	*pgpA*	gi|78777687|ref|YP_394002.1|	Sulfuromonas denitrificans ATCC 33889	E-value = 9.00E-34	Identity = 50.00%
	2		gi|32267083|ref|NP_861115.1|	Helicobacter hepaticus ATCC 51449	E-value = 8.00E-33	Identity = 55.33%
	3		gi|34558228|ref|NP_908043.1|	Wolinella succinogenes DSM 1740	E-value = 5.00E-32	Identity = 53.80%
	4		gi|57504878|ref|ZP_00370853.1|	Campylobacter coli RM2228	E-value = 1.00E-31	Identity = 52.56%
	5		gi|57505829|ref|ZP_00371754.1|	Campylobacter upsaliensis RM3195	E-value = 5.00E-31	Identity = 51.28%
AB0133	1		gi|106895541|ref|ZP_01362630.1|	Clostridium sp. OhILAs	E-value = 7.00E-16	Identity = 36.13%
AB0134	1	*carA*	gi|78776281|ref|YP_392596.1|	Sulfuromonas denitrificans ATCC 33889	E-value = 6.00E-145	Identity = 67.56%
	2		gi|152991772|ref|YP_001357493.1|	Sulfurovum sp. NBC37-1	E-value = 2.00E-144	Identity = 67.02%
	3		gi|34558335|ref|NP_908150.1|	Wolinella succinogenes DSM 1740	E-value = 2.00E-136	Identity = 63.98%
	4		gi|149194998|ref|ZP_01872091.1|	Caminibacter mediatlanticus TB-2	E-value = 1.00E-135	Identity = 66.84%
	5		gi|152991364|ref|YP_001357086.1|	Nitratiruptor sp. SB155-2	E-value = 1.00E-135	Identity = 65.68%
AB0135	1		gi|154173949|ref|YP_001407568.1|	Campylobacter curvus 525.92	E-value = 1.00E-47	Identity = 54.10%
	2		gi|157165281|ref|YP_001466178.1|	Campylobacter concisus 13826	E-value = 2.00E-45	Identity = 49.73%
	3		gi|118474830|ref|YP_891575.1|	Campylobacter fetus subsp. fetus 82-40	E-value = 1.00E-43	Identity = 47.54%
	4		gi|57169079|ref|ZP_00368206.1|	Campylobacter coli RM2228	E-value = 3.00E-39	Identity = 48.35%
	5		gi|152991771|ref|YP_001357492.1|	Sulfurovum sp. NBC37-1	E-value = 3.00E-38	Identity = 47.54%
AB0136	1	*purA*	gi|78776276|ref|YP_392591.1|	Sulfuromonas denitrificans ATCC 33889	E-value = 0	Identity = 75.85%
	2		gi|118475039|ref|YP_891572.1|	Campylobacter fetus subsp. fetus 82-40	E-value = 3.00E-176	Identity = 68.60%
	3		gi|157165191|ref|YP_001467552.1|	Campylobacter concisus 13826	E-value = 2.00E-175	Identity = 69.57%
	4		gi|57240899|ref|ZP_00368847.1|	Campylobacter lari RM2100	E-value = 1.00E-173	Identity = 68.28%
	5		gi|157415722|ref|YP_001482978.1|	Campylobacter jejuni subsp. jejuni 81116	E-value = 3.00E-173	Identity = 68.28%
AB0137	1		gi|78776275|ref|YP_392590.1|	Sulfuromonas denitrificans ATCC 33889	E-value = 4.00E-72	Identity = 48.75%
	2		gi|152991769|ref|YP_001357490.1|	Sulfurovum sp. NBC37-1	E-value = 2.00E-69	Identity = 44.69%
	3		gi|154175309|ref|YP_001408901.1|	Campylobacter curvus 525.92	E-value = 1.00E-68	Identity = 46.74%
	4		gi|152991367|ref|YP_001357089.1|	Nitratiruptor sp. SB155-2	E-value = 3.00E-68	Identity = 44.13%
	5		gi|34558342|ref|NP_908157.1|	Wolinella succinogenes DSM 1740	E-value = 2.00E-67	Identity = 43.37%

AB0138	1		gi|34556482|ref|NP_906297.1|	Wolinella succinogenes DSM 1740	E-value = 1.00E-137	Identity = 67.03%
	2		gi|152991768|ref|YP_001357489.1|	Sulfurovum sp. NBC37-1	E-value = 5.00E-125	Identity = 63.88%
	3		gi|78776274|ref|YP_392589.1|	Sulfuromonas denitrificans ATCC 33889	E-value = 3.00E-121	Identity = 63.49%
	4		gi|32266408|ref|NP_860440.1|	Helicobacter hepaticus ATCC 51449	E-value = 2.00E-116	Identity = 58.04%
	5		gi|152991368|ref|YP_001357090.1|	Nitratiruptor sp. SB155-2	E-value = 1.00E-115	Identity = 58.86%
AB0139	1		gi|78778226|ref|YP_394541.1|	Sulfuromonas denitrificans ATCC 33889	E-value = 9.00E-26	Identity = 37.97%
	2		gi|42524478|ref|NP_969858.1|	Bdellovibrio bacteriovorus HD100	E-value = 4.00E-22	Identity = 34.57%
	3		gi|51894086|ref|YP_076777.1|	Symbiobacterium thermophilum IAM 14863	E-value = 3.00E-21	Identity = 33.53%
	4		gi|21223888|ref|NP_629667.1|	Streptomyces coelicolor A3(2)	E-value = 1.00E-20	Identity = 35.47%
	5		gi|34558489|ref|NP_908304.1|	Wolinella succinogenes DSM 1740	E-value = 4.00E-20	Identity = 32.40%
AB0140	1		gi|150017899|ref|YP_001310153.1|	Clostridium beijerinckii NCIMB 8052	E-value = 4.00E-20	Identity = 39.58%
	2		gi|125718200|ref|YP_001035333.1|	Streptococcus sanguinis SK36	E-value = 1.00E-17	Identity = 37.12%
	3		gi|157151017|ref|YP_001450580.1|	Streptococcus gordonii str. Challis substr. CH1	E-value = 4.00E-16	Identity = 37.21%
	4		gi|15675758|ref|NP_269932.1|	Streptococcus pyogenes M1 GAS	E-value = 7.00E-16	Identity = 36.36%
	5		gi|50915025|ref|YP_060997.1|	Streptococcus pyogenes MGAS10394	E-value = 8.00E-16	Identity = 36.36%
AB0141	1		gi|78778226|ref|YP_394541.1|	Sulfuromonas denitrificans ATCC 33889	E-value = 3.00E-40	Identity = 51.34%
	2		gi|42524478|ref|NP_969858.1|	Bdellovibrio bacteriovorus HD100	E-value = 2.00E-34	Identity = 41.97%
	3		gi|51894086|ref|YP_076777.1|	Symbiobacterium thermophilum IAM 14863	E-value = 3.00E-33	Identity = 50.60%
	4		gi|120405483|ref|YP_955312.1|	Mycobacterium vanbaalenii PYR-1	E-value = 3.00E-31	Identity = 47.16%
	5		gi|149277643|ref|ZP_01883784.1|	Pedobacter sp. BAL39	E-value = 3.00E-31	Identity = 49.07%
AB0142	1		gi|15598035|ref|NP_251529.1|	Pseudomonas aeruginosa PAO1	E-value = 3.00E-35	Identity = 35.81%
	2		gi|116050843|ref|YP_790334.1|	Pseudomonas aeruginosa UCBPP-PA14	E-value = 3.00E-35	Identity = 36.24%
	3		gi|148549052|ref|YP_001269154.1|	Pseudomonas putida F1	E-value = 8.00E-35	Identity = 34.20%
	4		gi|152986274|ref|YP_001347686.1|	Pseudomonas aeruginosa PA7	E-value = 3.00E-34	Identity = 33.46%
	5		gi|26988599|ref|NP_744024.1|	Pseudomonas putida KT2440	E-value = 4.00E-34	Identity = 34.20%
AB0143	1	*pirA*	gi|106886119|ref|ZP_01353466.1|	Clostridium phytofermentans ISDg	E-value = 6.00E-67	Identity = 57.94%
	2		gi|106894568|ref|ZP_01361686.1|	Clostridium sp. OhILAs	E-value = 2.00E-66	Identity = 55.98%
	3		gi|153940961|ref|YP_001390207.1|	Clostridium botulinum F str. Langeland	E-value = 3.00E-66	Identity = 57.69%
	4		gi|150017898|ref|YP_001310152.1|	Clostridium beijerinckii NCIMB 8052	E-value = 4.00E-66	Identity = 58.12%
	5		gi|148378852|ref|YP_001253393.1|	Clostridium botulinum A str. ATCC 3502	E-value = 3.00E-65	Identity = 56.84%
AB0144	1	*gapB*	gi|84517644|ref|ZP_01004993.1|	Prochlorococcus marinus str. MIT 9211	E-value = 3.00E-56	Identity = 37.13%
	2		gi|145954648|ref|ZP_01803653.1|	Clostridium difficile QCD-32g58	E-value = 2.00E-42	Identity = 33.72%
	3		gi|126699376|ref|YP_001088273.1|	Clostridium difficile 630	E-value = 3.00E-42	Identity = 33.72%
	4		gi|152976965|ref|YP_001376482.1|	Bacillus cereus subsp. cytotoxis NVH 391-98	E-value = 6.00E-41	Identity = 32.45%
	5		gi|94501439|ref|ZP_01307958.1|	Oceanobacter sp. RED65	E-value = 1.00E-40	Identity = 31.88%
AB0145	1	*purM*	gi|152993932|ref|YP_001359653.1|	Sulfurovum sp. NBC37-1	E-value = 2.00E-131	Identity = 71.90%
	2		gi|78778244|ref|YP_394559.1|	Sulfuromonas denitrificans ATCC 33889	E-value = 6.00E-131	Identity = 71.30%
	3		gi|152989851|ref|YP_001355573.1|	Nitratiruptor sp. SB155-2	E-value = 5.00E-129	Identity = 68.69%
	4		gi|149194992|ref|ZP_01872085.1|	Caminibacter mediatlanticus TB-2	E-value = 7.00E-129	Identity = 72.00%
	5		gi|157164168|ref|YP_001465934.1|	Campylobacter concisus 13826	E-value = 1.00E-117	Identity = 68.01%
AB0146	1	*speE*	gi|78778242|ref|YP_394557.1|	Sulfuromonas denitrificans ATCC 33889	E-value = 3.00E-25	Identity = 40.88%
	2		gi|32267198|ref|NP_861230.1|	Helicobacter hepaticus ATCC 51449	E-value = 2.00E-11	Identity = 29.05%
	3		gi|34558427|ref|NP_908242.1|	Wolinella succinogenes DSM 1740	E-value = 2.00E-11	Identity = 30.39%
	4		gi|149194745|ref|ZP_01871840.1|	Caminibacter mediatlanticus TB-2	E-value = 4.00E-06	Identity = 27.65%
AB0147	1	*coaE*	gi|152989853|ref|YP_001355575.1|	Nitratiruptor sp. SB155-2	E-value = 5.00E-53	Identity = 59.79%
	2		gi|78778241|ref|YP_394556.1|	Sulfuromonas denitrificans ATCC 33889	E-value = 5.00E-42	Identity = 51.58%
	3		gi|152993981|ref|YP_001359702.1|	Sulfurovum sp. NBC37-1	E-value = 2.00E-40	Identity = 50.00%
	4		gi|15611837|ref|NP_223488.1|	Helicobacter pylori J99	E-value = 6.00E-39	Identity = 46.15%
	5		gi|34558428|ref|NP_908243.1|	Wolinella succinogenes DSM 1740	E-value = 9.00E-39	Identity = 46.03%

AB0148	1	*dapF*	gi|152989854|ref|YP_001355576.1|	Nitratiruptor sp. SB155-2	E-value = 4.00E-71	Identity = 58.02%
	2		gi|78778240|ref|YP_394555.1|	Sulfuromonas denitrificans ATCC 33889	E-value = 6.00E-70	Identity = 54.12%
	3		gi|149194724|ref|ZP_01871819.1|	Caminibacter mediatlanticus TB-2	E-value = 2.00E-67	Identity = 56.20%
	4		gi|118475771|ref|YP_892886.1|	Campylobacter fetus subsp. fetus 82-40	E-value = 3.00E-65	Identity = 53.44%
	5		gi|157163987|ref|YP_001465937.1|	Campylobacter concisus 13826	E-value = 7.00E-64	Identity = 50.41%
AB0149	1	*purT*	gi|152989880|ref|YP_001355602.1|	Nitratiruptor sp. SB155-2	E-value = 8.00E-164	Identity = 74.23%
	2		gi|152991751|ref|YP_001357472.1|	Sulfurovum sp. NBC37-1	E-value = 2.00E-160	Identity = 71.91%
	3		gi|78776234|ref|YP_392549.1|	Sulfuromonas denitrificans ATCC 33889	E-value = 3.00E-158	Identity = 70.62%
	4		gi|149194863|ref|ZP_01871957.1|	Caminibacter mediatlanticus TB-2	E-value = 5.00E-145	Identity = 66.75%
	5		gi|34557962|ref|NP_907777.1|	Wolinella succinogenes DSM 1740	E-value = 2.00E-137	Identity = 63.97%
AB0150	1		gi|146297912|ref|YP_001192503.1|	Flavobacterium johnsoniae UW101	E-value = 2.00E-35	Identity = 50.00%
	2		gi|66805591|ref|XP_636517.1|	Dictyostelium discoideum AX4	E-value = 2.00E-32	Identity = 50.98%
	3		gi|88806543|ref|ZP_01122060.1|	Robiginitalea biformata HTCC2501	E-value = 1.00E-31	Identity = 44.30%
	4		gi|86133536|ref|ZP_01052118.1|	Tenacibaculum sp. MED152	E-value = 3.00E-24	Identity = 43.95%
	5		gi|88803244|ref|ZP_01118770.1|	Polaribacter irgensii 23-P	E-value = 1.00E-23	Identity = 42.48%
AB0151	1		gi|124545257|ref|ZP_01704462.1|	Shewanella putrefaciens 200	E-value = 7.00E-07	Identity = 35.16%
AB0152	1		gi|58616391|ref|YP_195521.1|	Azoarcus sp. EbN1	E-value = 1.00E-66	Identity = 31.70%
	2		gi|124262593|ref|YP_001023063.1|	Methylibium petroleiphilum PM1	E-value = 4.00E-56	Identity = 31.20%
	3		gi|91791171|ref|YP_552121.1|	Polaromonas sp. JS666	E-value = 9.00E-56	Identity = 29.13%
	4		gi|120554230|ref|YP_958581.1|	Marinobacter aquaeolei VT8	E-value = 7.00E-52	Identity = 27.95%
	5		gi|156933964|ref|YP_001437880.1|	Enterobacter sakazakii ATCC BAA-894	E-value = 2.00E-51	Identity = 31.00%
AB0153	1	*ubiA*	gi|78778005|ref|YP_394320.1|	Sulfuromonas denitrificans ATCC 33889	E-value = 2.00E-91	Identity = 68.33%
	2		gi|152990053|ref|YP_001355775.1|	Nitratiruptor sp. SB155-2	E-value = 5.00E-90	Identity = 67.51%
	3		gi|152993791|ref|YP_001359512.1|	Sulfurovum sp. NBC37-1	E-value = 1.00E-88	Identity = 66.55%
	4		gi|154175423|ref|YP_001407493.1|	Campylobacter curvus 525.92	E-value = 1.00E-82	Identity = 61.07%
	5		gi|157165573|ref|YP_001466048.1|	Campylobacter concisus 13826	E-value = 6.00E-79	Identity = 59.64%
AB0154	1			***** No matches found *****		
AB0155	1	*miaA*	gi|152990052|ref|YP_001355774.1|	Nitratiruptor sp. SB155-2	E-value = 1.00E-69	Identity = 52.54%
	2		gi|78778006|ref|YP_394321.1|	Sulfuromonas denitrificans ATCC 33889	E-value = 4.00E-66	Identity = 49.66%
	3		gi|152993793|ref|YP_001359514.1|	Sulfurovum sp. NBC37-1	E-value = 2.00E-65	Identity = 48.47%
	4		gi|157164731|ref|YP_001466050.1|	Campylobacter concisus 13826	E-value = 1.00E-63	Identity = 46.88%
	5		gi|154174124|ref|YP_001407491.1|	Campylobacter curvus 525.92	E-value = 6.00E-62	Identity = 45.52%
AB0156	1	*rpmE*	gi|118474643|ref|YP_891489.1|	Campylobacter fetus subsp. fetus 82-40	E-value = 6.00E-18	Identity = 70.77%
	2		gi|57168672|ref|ZP_00367804.1|	Campylobacter coli RM2228	E-value = 1.00E-17	Identity = 72.31%
	3		gi|57241384|ref|ZP_00369330.1|	Campylobacter lari RM2100	E-value = 1.00E-17	Identity = 73.85%
	4		gi|153951925|ref|YP_001397413.1|	Campylobacter jejuni subsp. doylei 269.97	E-value = 2.00E-17	Identity = 70.77%
	5		gi|15791543|ref|NP_281366.1|	Campylobacter jejuni subsp. jejuni NCTC 11168	E-value = 2.00E-17	Identity = 70.77%
AB0157	1		gi|149195113|ref|ZP_01872205.1|	Caminibacter mediatlanticus TB-2	E-value = 2.00E-73	Identity = 58.43%
	2		gi|152991817|ref|YP_001357538.1|	Sulfurovum sp. NBC37-1	E-value = 1.00E-71	Identity = 50.00%
	3		gi|34556872|ref|NP_906687.1|	Wolinella succinogenes DSM 1740	E-value = 1.00E-68	Identity = 49.82%
	4		gi|152991300|ref|YP_001357022.1|	Nitratiruptor sp. SB155-2	E-value = 3.00E-66	Identity = 50.38%
	5		gi|78778103|ref|YP_394418.1|	Sulfuromonas denitrificans ATCC 33889	E-value = 5.00E-66	Identity = 49.63%
AB0158	1		gi|34556873|ref|NP_906688.1|	Wolinella succinogenes DSM 1740	E-value = 2.00E-51	Identity = 48.26%
	2		gi|154174304|ref|YP_001407505.1|	Campylobacter curvus 525.92	E-value = 7.00E-51	Identity = 47.98%
	3		gi|152991818|ref|YP_001357539.1|	Sulfurovum sp. NBC37-1	E-value = 8.00E-51	Identity = 48.47%
	4		gi|157164272|ref|YP_001466038.1|	Campylobacter concisus 13826	E-value = 2.00E-50	Identity = 48.43%
	5		gi|109947425|ref|YP_664653.1|	Helicobacter acinonychis str. Sheeba	E-value = 1.00E-49	Identity = 49.78%
AB0159	1		gi|78778100|ref|YP_394415.1|	Sulfuromonas denitrificans ATCC 33889	E-value = 4.00E-32	Identity = 32.95%
	2		gi|34556875|ref|NP_906690.1|	Wolinella succinogenes DSM 1740	E-value = 2.00E-26	Identity = 26.07%

	3		gi|118475591|ref|YP_891493.1|	Campylobacter fetus subsp. fetus 82-40	E-value = 4.00E-24	Identity = 30.92%
	4		gi|109947423|ref|YP_664651.1|	Helicobacter acinonychis str. Sheeba	E-value = 4.00E-22	Identity = 26.62%
	5		gi|15645180|ref|NP_207350.1|	Helicobacter pylori 26695	E-value = 4.00E-21	Identity = 26.24%
AB0160	1	*aspB1*	gi|157165123|ref|YP_001466035.1|	Campylobacter concisus 13826	E-value = 2.00E-169	Identity = 70.47%
	2		gi|154174215|ref|YP_001407508.1|	Campylobacter curvus 525.92	E-value = 4.00E-168	Identity = 69.15%
	3		gi|34556876|ref|NP_906691.1|	Wolinella succinogenes DSM 1740	E-value = 8.00E-168	Identity = 68.98%
	4		gi|152991296|ref|YP_001357018.1|	Nitratiruptor sp. SB155-2	E-value = 7.00E-166	Identity = 70.05%
	5		gi|57241389|ref|ZP_00369335.1|	Campylobacter lari RM2100	E-value = 9.00E-165	Identity = 69.25%
AB0161	1	*hom*	gi|152991295|ref|YP_001357017.1|	Nitratiruptor sp. SB155-2	E-value = 3.00E-151	Identity = 68.02%
	2		gi|152991821|ref|YP_001357542.1|	Sulfurovum sp. NBC37-1	E-value = 9.00E-140	Identity = 64.71%
	3		gi|34556877|ref|NP_906692.1|	Wolinella succinogenes DSM 1740	E-value = 9.00E-138	Identity = 65.71%
	4		gi|78778098|ref|YP_394413.1|	Sulfuromonas denitrificans ATCC 33889	E-value = 7.00E-135	Identity = 68.97%
	5		gi|157163938|ref|YP_001466034.1|	Campylobacter concisus 13826	E-value = 2.00E-132	Identity = 60.66%
AB0162	1	*traT*	gi|34557173|ref|NP_906988.1|	Wolinella succinogenes DSM 1740	E-value = 3.00E-63	Identity = 53.10%
	2		gi|118475085|ref|YP_891499.1|	Campylobacter fetus subsp. fetus 82-40	E-value = 6.00E-62	Identity = 60.98%
	3		gi|153952157|ref|YP_001398078.1|	Campylobacter jejuni subsp. doylei 269.97	E-value = 4.00E-56	Identity = 57.39%
	4		gi|57242301|ref|ZP_00370240.1|	Campylobacter upsaliensis RM3195	E-value = 3.00E-48	Identity = 51.95%
	5		gi|71559043|ref|YP_271770.1|	Salmonella enterica	E-value = 8.00E-21	Identity = 30.04%
AB0163	1	*cdsA*	gi|152991408|ref|YP_001357130.1|	Nitratiruptor sp. SB155-2	E-value = 9.00E-60	Identity = 52.46%
	2		gi|152991741|ref|YP_001357462.1|	Sulfurovum sp. NBC37-1	E-value = 3.00E-54	Identity = 47.62%
	3		gi|34557211|ref|NP_907026.1|	Wolinella succinogenes DSM 1740	E-value = 5.00E-52	Identity = 47.28%
	4		gi|78776326|ref|YP_392641.1|	Sulfuromonas denitrificans ATCC 33889	E-value = 2.00E-51	Identity = 50.00%
	5		gi|32266022|ref|NP_860054.1|	Helicobacter hepaticus ATCC 51449	E-value = 2.00E-45	Identity = 46.91%
AB0164	1	*dxr*	gi|78776327|ref|YP_392642.1|	Sulfuromonas denitrificans ATCC 33889	E-value = 5.00E-124	Identity = 63.64%
	2		gi|149195077|ref|ZP_01872169.1|	Caminibacter mediatlanticus TB-2	E-value = 1.00E-120	Identity = 63.43%
	3		gi|152991740|ref|YP_001357461.1|	Sulfurovum sp. NBC37-1	E-value = 9.00E-114	Identity = 57.95%
	4		gi|34557210|ref|NP_907025.1|	Wolinella succinogenes DSM 1740	E-value = 2.00E-102	Identity = 52.39%
	5		gi|152991407|ref|YP_001357129.1|	Nitratiruptor sp. SB155-2	E-value = 3.00E-102	Identity = 54.21%
AB0165	1			***** No matches found *****		
AB0166	1		gi|78776345|ref|YP_392660.1|	Sulfuromonas denitrificans ATCC 33889	E-value = 5.00E-36	Identity = 45.14%
	2		gi|152991739|ref|YP_001357460.1|	Sulfurovum sp. NBC37-1	E-value = 3.00E-35	Identity = 46.63%
	3		gi|152991405|ref|YP_001357127.1|	Nitratiruptor sp. SB155-2	E-value = 4.00E-35	Identity = 45.14%
	4		gi|134097949|ref|YP_001103610.1|	Saccharopolyspora erythraea NRRL 2338	E-value = 4.00E-19	Identity = 34.27%
	5		gi|29831465|ref|NP_826099.1|	Streptomyces avermitilis MA-4680	E-value = 7.00E-16	Identity = 30.68%
AB0167	1	*gcp*	gi|78776332|ref|YP_392647.1|	Sulfuromonas denitrificans ATCC 33889	E-value = 2.00E-118	Identity = 65.95%
	2		gi|152991404|ref|YP_001357126.1|	Nitratiruptor sp. SB155-2	E-value = 2.00E-114	Identity = 63.89%
	3		gi|152991738|ref|YP_001357459.1|	Sulfurovum sp. NBC37-1	E-value = 9.00E-111	Identity = 60.24%
	4		gi|149195384|ref|ZP_01872469.1|	Caminibacter mediatlanticus TB-2	E-value = 3.00E-109	Identity = 61.03%
	5		gi|34557921|ref|NP_907736.1|	Wolinella succinogenes DSM 1740	E-value = 5.00E-101	Identity = 58.66%
AB0168	1		gi|78777706|ref|YP_394021.1|	Sulfuromonas denitrificans ATCC 33889	E-value = 1.00E-11	Identity = 46.67%
	2		gi|149372843|ref|ZP_01891864.1|	unidentified eubacterium SCB49	E-value = 1.00E-05	Identity = 34.83%
AB0169	1		gi|149910686|ref|ZP_01899322.1|	Moritella sp. PE36	E-value = 2.00E-146	Identity = 58.94%
	2		gi|78776417|ref|YP_392732.1|	Sulfuromonas denitrificans ATCC 33889	E-value = 2.00E-131	Identity = 52.92%
	3		gi|152989919|ref|YP_001355641.1|	Nitratiruptor sp. SB155-2	E-value = 2.00E-126	Identity = 50.65%
	4		gi|149194663|ref|ZP_01871758.1|	Caminibacter mediatlanticus TB-2	E-value = 5.00E-121	Identity = 50.11%
	5		gi|152991753|ref|YP_001357474.1|	Sulfurovum sp. NBC37-1	E-value = 3.00E-112	Identity = 46.30%
AB0170	1		gi|46199293|ref|YP_004960.1|	Thermus thermophilus HB27	E-value = 2.00E-16	Identity = 40.40%
	2		gi|21674425|ref|NP_662490.1|	Chlorobium tepidum TLS	E-value = 3.00E-16	Identity = 44.68%
	3		gi|55981325|ref|YP_144622.1|	Thermus thermophilus HB8	E-value = 4.00E-16	Identity = 39.39%

	4		gi|116750719|ref|YP_847406.1|	Syntrophobacter fumaroxidans MPOB	E-value = 5.00E-16	Identity = 37.37%
	5		gi|119773679|ref|YP_926419.1|	Shewanella amazonensis SB2B	E-value = 7.00E-16	Identity = 40.00%
AB0171	1	*thiG*	gi|78776418|ref|YP_392733.1|	Sulfuromonas denitrificans ATCC 33889	E-value = 6.00E-116	Identity = 83.78%
	2		gi|149194944|ref|ZP_01872037.1|	Caminibacter mediatlanticus TB-2	E-value = 6.00E-112	Identity = 80.69%
	3		gi|152989923|ref|YP_001355645.1|	Nitratiruptor sp. SB155-2	E-value = 3.00E-110	Identity = 77.99%
	4		gi|34557342|ref|NP_907157.1|	Wolinella succinogenes DSM 1740	E-value = 3.00E-105	Identity = 75.58%
	5		gi|32267350|ref|NP_861382.1|	Helicobacter hepaticus ATCC 51449	E-value = 3.00E-104	Identity = 75.29%
AB0172	1	*pycB1*	gi|152991046|ref|YP_001356768.1|	Nitratiruptor sp. SB155-2	E-value = 0	Identity = 72.49%
	2		gi|152993020|ref|YP_001358741.1|	Sulfurovum sp. NBC37-1	E-value = 0	Identity = 72.41%
	3		gi|149194559|ref|ZP_01871655.1|	Caminibacter mediatlanticus TB-2	E-value = 0	Identity = 69.46%
	4		gi|154174885|ref|YP_001407832.1|	Campylobacter curvus 525.92	E-value = 0	Identity = 69.44%
	5		gi|157165713|ref|YP_001466387.1|	Campylobacter concisus 13826	E-value = 0	Identity = 69.46%
AB0173	1			***** No matches found *****		
AB0174	1		gi|78776820|ref|YP_393135.1|	Sulfuromonas denitrificans ATCC 33889	E-value = 4.00E-115	Identity = 71.48%
	2		gi|34557184|ref|NP_906999.1|	Wolinella succinogenes DSM 1740	E-value = 5.00E-75	Identity = 47.08%
	3		gi|28900255|ref|NP_799910.1|	Vibrio parahaemolyticus RIMD 2210633	E-value = 5.00E-41	Identity = 31.71%
	4		gi|156977683|ref|YP_001448589.1|	Vibrio harveyi ATCC BAA-1116	E-value = 8.00E-41	Identity = 31.36%
	5		gi|116187268|ref|ZP_01477157.1|	Vibrio sp. Ex25	E-value = 8.00E-41	Identity = 31.71%
AB0175	1		gi|78777566|ref|YP_393881.1|	Sulfuromonas denitrificans ATCC 33889	E-value = 9.00E-114	Identity = 80.67%
	2		gi|83646426|ref|YP_434861.1|	Hahella chejuensis KCTC 2396	E-value = 1.00E-88	Identity = 62.04%
	3		gi|27366938|ref|NP_762465.1|	Vibrio vulnificus CMCP6	E-value = 2.00E-84	Identity = 64.47%
	4		gi|37676715|ref|NP_937111.1|	Vibrio vulnificus YJ016	E-value = 4.00E-84	Identity = 64.15%
	5		gi|117619777|ref|YP_855810.1|	Aeromonas hydrophila subsp. hydrophila ATCC 7966	E-value = 2.00E-81	Identity = 61.32%
AB0176	1		gi|34558440|ref|NP_908255.1|	Wolinella succinogenes DSM 1740	E-value = 6.00E-52	Identity = 28.73%
	2		gi|34558144|ref|NP_907959.1|	Wolinella succinogenes DSM 1740	E-value = 1.00E-39	Identity = 28.07%
AB0177	1		gi|118443735|ref|YP_878084.1|	Clostridium novyi NT	E-value = 0	Identity = 51.98%
	2		gi|148380042|ref|YP_001254583.1|	Clostridium botulinum A str. ATCC 3502	E-value = 0	Identity = 50.38%
	3		gi|90412540|ref|ZP_01220543.1|	Photobacterium profundum 3TCK	E-value = 0	Identity = 44.85%
	4		gi|68055380|ref|ZP_00539526.1|	Exiguobacterium sibiricum 255-15	E-value = 2.00E-150	Identity = 36.03%
	5		gi|148642750|ref|YP_001273263.1|	Methanobrevibacter smithii ATCC 35061	E-value = 2.00E-150	Identity = 38.58%
AB0178	1	*tkt*	gi|152991762|ref|YP_001357483.1|	Sulfurovum sp. NBC37-1	E-value = 0	Identity = 65.34%
	2		gi|152990007|ref|YP_001355729.1|	Nitratiruptor sp. SB155-2	E-value = 0	Identity = 63.34%
	3		gi|78776539|ref|YP_392854.1|	Sulfuromonas denitrificans ATCC 33889	E-value = 0	Identity = 63.86%
	4		gi|118475100|ref|YP_891279.1|	Campylobacter fetus subsp. fetus 82-40	E-value = 0	Identity = 64.74%
	5		gi|157165524|ref|YP_001467680.1|	Campylobacter concisus 13826	E-value = 0	Identity = 63.71%
AB0179	1	*lrgA*	gi|110599991|ref|ZP_01388221.1|	Geobacter sp. FRC-32	E-value = 7.00E-12	Identity = 36.28%
	2		gi|91783125|ref|YP_558331.1|	Burkholderia xenovorans LB400	E-value = 1.00E-11	Identity = 40.37%
	3		gi|92113916|ref|YP_573844.1|	Chromohalobacter salexigens DSM 3043	E-value = 2.00E-11	Identity = 38.26%
	4		gi|144897729|emb|CAM74593.1|	Magnetospirillum gryphiswaldense MSR-1	E-value = 3.00E-11	Identity = 45.92%
	5		gi|118743706|ref|ZP_01591708.1|	Geobacter lovleyi SZ	E-value = 1.00E-10	Identity = 37.14%
AB0180	1	*lrgB*	gi|78222419|ref|YP_384166.1|	Geobacter metallireducens GS-15	E-value = 2.00E-42	Identity = 41.33%
	2		gi|78486277|ref|YP_392202.1|	Thiomicrospira crunogena XCL-2	E-value = 4.00E-40	Identity = 43.23%
	3		gi|71908719|ref|YP_286306.1|	Dechloromonas aromatica RCB	E-value = 5.00E-39	Identity = 43.12%
	4		gi|120555974|ref|YP_960325.1|	Marinobacter aquaeolei VT8	E-value = 5.00E-39	Identity = 38.70%
	5		gi|126669005|ref|ZP_01739942.1|	Marinobacter sp. ELB17	E-value = 7.00E-39	Identity = 38.05%
AB0181	1		gi|84319020|ref|ZP_00967427.1|	Pseudomonas aeruginosa C3719	E-value = 3.00E-23	Identity = 42.99%
	2		gi|15599359|ref|NP_252853.1|	Pseudomonas aeruginosa PAO1	E-value = 3.00E-23	Identity = 42.99%
	3		gi|49080280|gb|AAT49999.1|	synthetic construct [Pseudomonas aeruginosa]	E-value = 3.00E-23	Identity = 42.99%
	4		gi|114773707|ref|ZP_01450738.1|	alpha proteobacterium HTCC2255	E-value = 3.00E-18	Identity = 41.67%

	5		gi|22034319|gb|AAL01561.1|	Escherichia fergusonii	E-value = 4.00E-16	Identity = 44.23%
AB0183	1		gi|88795630|ref|ZP_01111324.1|	Alteromonas macleodii 'Deep ecotype'	E-value = 5.00E-17	Identity = 41.22%
	2		gi|109897546|ref|YP_660801.1|	Pseudoalteromonas atlantica T6c	E-value = 9.00E-17	Identity = 41.73%
	3		gi|90409818|ref|ZP_01217835.1|	Photobacterium profundum 3TCK	E-value = 6.00E-16	Identity = 40.94%
	4		gi|54301732|ref|YP_131725.1|	Photobacterium profundum SS9	E-value = 8.00E-16	Identity = 40.16%
	5		gi|114771451|ref|ZP_01448866.1|	alpha proteobacterium HTCC2255	E-value = 2.00E-15	Identity = 40.48%
AB0184	1	*dctA*	gi|34556831|ref|NP_906646.1|	Wolinella succinogenes DSM 1740	E-value = 2.00E-153	Identity = 69.43%
	2		gi|118474683|ref|YP_891627.1|	Campylobacter fetus subsp. fetus 82-40	E-value = 1.00E-140	Identity = 63.87%
	3		gi|118474592|ref|YP_891628.1|	Campylobacter fetus subsp. fetus 82-40	E-value = 8.00E-139	Identity = 62.47%
	4		gi|154174743|ref|YP_001407837.1|	Campylobacter curvus 525.92	E-value = 3.00E-136	Identity = 63.29%
	5		gi|57168071|ref|ZP_00367210.1|	Campylobacter coli RM2228	E-value = 8.00E-130	Identity = 57.27%
AB0185	1		gi|152992756|ref|YP_001358477.1|	Sulfurovum sp. NBC37-1	E-value = 5.00E-14	Identity = 28.77%
	2		gi|126208873|ref|YP_001054098.1|	Actinobacillus pleuropneumoniae L20	E-value = 6.00E-07	Identity = 25.98%
	3		gi|154174239|ref|YP_001407562.1|	Campylobacter curvus 525.92	E-value = 2.00E-06	Identity = 28.64%
AB0186	1			***** No matches found *****		
AB0187	1		gi|153217038|ref|ZP_01950802.1|	Vibrio cholerae 1587	E-value = 4.00E-44	Identity = 50.00%
	2		gi|150423018|gb|EDN14967.1|	Vibrio cholerae AM-19226	E-value = 4.00E-44	Identity = 50.00%
	3		gi|153826391|ref|ZP_01979058.1|	Vibrio cholerae MZO-2	E-value = 6.00E-44	Identity = 50.00%
	4		gi|153829252|ref|ZP_01981919.1|	Vibrio cholerae 623-39	E-value = 1.00E-43	Identity = 49.53%
	5		gi|116189753|ref|ZP_01479500.1|	Vibrio cholerae MO10	E-value = 3.00E-43	Identity = 49.07%
AB0188	1		gi|147673088|ref|YP_001217468.1|	Vibrio cholerae O395	E-value = 1.00E-100	Identity = 63.84%
	2		gi|153217034|ref|ZP_01950798.1|	Vibrio cholerae 1587	E-value = 1.00E-99	Identity = 64.36%
	3		gi|153826388|ref|ZP_01979055.1|	Vibrio cholerae MZO-2	E-value = 1.00E-99	Identity = 64.03%
	4		gi|116220560|ref|ZP_01485989.1|	Vibrio cholerae V51	E-value = 2.00E-99	Identity = 64.03%
	5		gi|15641938|ref|NP_231570.1|	Vibrio cholerae O1 biovar eltor str. N16961	E-value = 4.00E-99	Identity = 63.70%
AB0189	1		gi|87118984|ref|ZP_01074882.1|	Marinomonas sp. MED121	E-value = 3.00E-63	Identity = 55.94%
	2		gi|15641939|ref|NP_231571.1|	Vibrio cholerae O1 biovar eltor str. N16961	E-value = 3.00E-58	Identity = 58.70%
	3		gi|153217033|ref|ZP_01950797.1|	Vibrio cholerae 1587	E-value = 4.00E-58	Identity = 58.70%
	4		gi|153829243|ref|ZP_01981910.1|	Vibrio cholerae 623-39	E-value = 5.00E-58	Identity = 58.70%
	5		gi|153826397|ref|ZP_01979064.1|	Vibrio cholerae MZO-2	E-value = 7.00E-58	Identity = 58.15%
AB0190	1	*aas*	gi|152992755|ref|YP_001358476.1|	Sulfurovum sp. NBC37-1	E-value = 0	Identity = 44.34%
	2		gi|89092511|ref|ZP_01165464.1|	Oceanospirillum sp. MED92	E-value = 0	Identity = 43.46%
	3		gi|90023171|ref|YP_528998.1|	Saccharophagus degradans 2-40	E-value = 0	Identity = 44.26%
	4		gi|57240331|ref|ZP_00368280.1|	Campylobacter lari RM2100	E-value = 0	Identity = 41.1%
	5		gi|32265805|ref|NP_859837.1|	Helicobacter hepaticus ATCC 51449	E-value = 0	Identity = 42.26%
AB0191	1	*glnD*	gi|152991173|ref|YP_001356895.1|	Nitratiruptor sp. SB155-2	E-value = 0	Identity = 44.09%
	2		gi|152992133|ref|YP_001357854.1|	Sulfurovum sp. NBC37-1	E-value = 0	Identity = 41.33%
	3		gi|78777679|ref|YP_393994.1|	Sulfuromonas denitrificans ATCC 33889	E-value = 2.00E-169	Identity = 38.53%
	4		gi|149194245|ref|ZP_01871342.1|	Caminibacter mediatlanticus TB-2	E-value = 1.00E-157	Identity = 42.40%
	5		gi|34556479|ref|NP_906294.1|	Wolinella succinogenes DSM 1740	E-value = 9.00E-152	Identity = 35.93%
AB0192	1		gi|78777591|ref|YP_393906.1|	Sulfuromonas denitrificans ATCC 33889	E-value = 4.00E-32	Identity = 40.25%
	2		gi|34556947|ref|NP_906762.1|	Wolinella succinogenes DSM 1740	E-value = 1.00E-27	Identity = 35.83%
	3		gi|152990239|ref|YP_001355961.1|	Nitratiruptor sp. SB155-2	E-value = 2.00E-22	Identity = 33.60%
	4		gi|152993222|ref|YP_001358943.1|	Sulfurovum sp. NBC37-1	E-value = 7.00E-19	Identity = 28.46%
	5		gi|149195054|ref|ZP_01872146.1|	Caminibacter mediatlanticus TB-2	E-value = 1.00E-11	Identity = 29.80%
AB0193	1		gi|78485116|ref|YP_391041.1|	Thiomicrospira crunogena XCL-2	E-value = 7.00E-48	Identity = 45.21%
	2		gi|149195055|ref|ZP_01872147.1|	Caminibacter mediatlanticus TB-2	E-value = 5.00E-39	Identity = 46.43%
	3		gi|78777654|ref|YP_393969.1|	Sulfuromonas denitrificans ATCC 33889	E-value = 5.00E-22	Identity = 32.97%
	4		gi|154173756|ref|YP_001407719.1|	Campylobacter curvus 525.92	E-value = 7.00E-21	Identity = 29.59%

	5		gi|57506021|ref|ZP_00371944.1|	Campylobacter upsaliensis RM3195	E-value = 7.00E-20	Identity = 34.82%
AB0194	1		gi|123449116|ref|XP_001313280.1|	Trichomonas vaginalis G3	E-value = 5.00E-24	Identity = 26.13%
	2		gi|123471510|ref|XP_001318954.1|	Trichomonas vaginalis G3	E-value = 1.00E-21	Identity = 25.49%
	3		gi|123413741|ref|XP_001304339.1|	Trichomonas vaginalis G3	E-value = 3.00E-21	Identity = 25.33%
AB0195	1	*glnB1*	gi|152990242|ref|YP_001355964.1|	Nitratiruptor sp. SB155-2	E-value = 5.00E-39	Identity = 81.25%
	2		gi|78776841|ref|YP_393156.1|	Sulfuromonas denitrificans ATCC 33889	E-value = 9.00E-39	Identity = 83.04%
	3		gi|149911060|ref|ZP_01899688.1|	Moritella sp. PE36	E-value = 6.00E-36	Identity = 71.43%
	4		gi|152991172|ref|YP_001356894.1|	Nitratiruptor sp. SB155-2	E-value = 7.00E-36	Identity = 75.00%
	5		gi|114321697|ref|YP_743380.1|	Alkalilimnicola ehrlichei MLHE-1	E-value = 8.00E-36	Identity = 68.75%
AB0196	1	*pyrC*	gi|152991986|ref|YP_001357707.1|	Sulfurovum sp. NBC37-1	E-value = 2.00E-129	Identity = 66.96%
	2		gi|152991169|ref|YP_001356891.1|	Nitratiruptor sp. SB155-2	E-value = 7.00E-118	Identity = 64.65%
	3		gi|78776823|ref|YP_393138.1|	Sulfuromonas denitrificans ATCC 33889	E-value = 1.00E-112	Identity = 60.90%
	4		gi|57168382|ref|ZP_00367516.1|	Campylobacter coli RM2228	E-value = 2.00E-108	Identity = 59.76%
	5		gi|86151298|ref|ZP_01069513.1|	Campylobacter jejuni subsp. jejuni 260.94	E-value = 5.00E-108	Identity = 58.98%
AB0197	1	*fliM*	gi|157164910|ref|YP_001467212.1|	Campylobacter concisus 13826	E-value = 6.00E-61	Identity = 38.40%
	2		gi|118475490|ref|YP_892646.1|	Campylobacter fetus subsp. fetus 82-40	E-value = 2.00E-60	Identity = 39.94%
	3		gi|154175307|ref|YP_001408769.1|	Campylobacter curvus 525.92	E-value = 5.00E-60	Identity = 38.11%
	4		gi|149194333|ref|ZP_01871430.1|	Caminibacter mediatlanticus TB-2	E-value = 2.00E-58	Identity = 38.31%
	5		gi|57168742|ref|ZP_00367873.1|	Campylobacter coli RM2228	E-value = 7.00E-57	Identity = 38.00%
AB0198	1	*flgI*	gi|32265641|ref|NP_859673.1|	Helicobacter hepaticus ATCC 51449	E-value = 7.00E-63	Identity = 42.20%
	2		gi|15644874|ref|NP_207044.1|	Helicobacter pylori 26695	E-value = 9.00E-62	Identity = 41.79%
	3		gi|108562674|ref|YP_626990.1|	Helicobacter pylori HPAG1	E-value = 2.00E-61	Identity = 41.21%
	4		gi|109947877|ref|YP_665105.1|	Helicobacter acinonychis str. Sheeba	E-value = 7.00E-61	Identity = 40.23%
	5		gi|15611301|ref|NP_222952.1|	Helicobacter pylori J99	E-value = 2.00E-60	Identity = 39.77%
AB0199	1			***** No matches found *****		
AB0200	1	*fliQ*	gi|149194202|ref|ZP_01871300.1|	Caminibacter mediatlanticus TB-2	E-value = 3.00E-08	Identity = 46.99%
	2		gi|118474914|ref|YP_891408.1|	Campylobacter fetus subsp. fetus 82-40	E-value = 2.00E-07	Identity = 44.58%
	3		gi|17548595|ref|NP_521935.1|	Ralstonia solanacearum GMI1000	E-value = 2.00E-07	Identity = 34.67%
	4		gi|78778199|ref|YP_394514.1|	Sulfuromonas denitrificans ATCC 33889	E-value = 2.00E-07	Identity = 41.46%
	5		gi|42524693|ref|NP_970073.1|	Bdellovibrio bacteriovorus HD100	E-value = 3.00E-07	Identity = 36.71%
AB0201	1			***** No matches found *****		
AB0202	1			***** No matches found *****		
AB0203	1	*fliL*	gi|157165347|ref|YP_001466934.1|	Campylobacter concisus 13826	E-value = 2.00E-09	Identity = 27.17%
	2		gi|117926843|ref|YP_867460.1|	Magnetococcus sp. MC-1	E-value = 2.00E-08	Identity = 29.11%
	3		gi|83720504|ref|YP_440586.1|	Burkholderia thailandensis E264	E-value = 3.00E-08	Identity = 30.23%
	4		gi|157376192|ref|YP_001474792.1|	Shewanella sediminis HAW-EB3	E-value = 1.00E-06	Identity = 26.35%
	5		gi|118072029|ref|ZP_01540221.1|	Shewanella woodyi ATCC 51908	E-value = 4.00E-06	Identity = 27.01%
AB0204	1			***** No matches found *****		
AB0205	1	*flgH*	gi|57242591|ref|ZP_00370528.1|	Campylobacter upsaliensis RM3195	E-value = 7.00E-34	Identity = 36.32%
	2		gi|78776932|ref|YP_393247.1|	Sulfuromonas denitrificans ATCC 33889	E-value = 8.00E-34	Identity = 35.83%
	3		gi|154174210|ref|YP_001408222.1|	Campylobacter curvus 525.92	E-value = 1.00E-33	Identity = 36.71%
	4		gi|86153207|ref|ZP_01071411.1|	Campylobacter jejuni subsp. jejuni HB93-13	E-value = 8.00E-33	Identity = 36.29%
	5		gi|121612803|ref|YP_001000383.1|	Campylobacter jejuni subsp. jejuni 81-176	E-value = 1.00E-32	Identity = 36.29%
AB0206	1	*flgK*		***** No matches found *****		
AB0207	1	*fliD*	gi|92114155|ref|YP_574083.1|	Chromohalobacter salexigens DSM 3043	E-value = 2.00E-14	Identity = 25.50%
	2		gi|113948270|ref|ZP_01433931.1|	Shewanella baltica OS195	E-value = 5.00E-14	Identity = 26.19%
	3		gi|153001459|ref|YP_001367140.1|	Shewanella baltica OS185	E-value = 6.00E-14	Identity = 26.19%
	4		gi|67154580|ref|ZP_00416325.1|	Azotobacter vinelandii AvOP	E-value = 1.00E-13	Identity = 26.37%
	5		gi|157161395|ref|YP_001458713.1|	Escherichia coli HS	E-value = 1.00E-13	Identity = 27.47%

AB0208	1	*fliS*	gi|149194067|ref|ZP_01871165.1|	Caminibacter mediatlanticus TB-2	E-value = 5.00E-21	Identity = 50.00%
	2		gi|34556599|ref|NP_906414.1|	Wolinella succinogenes DSM 1740	E-value = 2.00E-20	Identity = 49.54%
	3		gi|154175496|ref|YP_001408377.1|	Campylobacter curvus 525.92	E-value = 6.00E-18	Identity = 47.12%
	4		gi|86151684|ref|ZP_01069898.1|	Campylobacter jejuni subsp. jejuni 260.94	E-value = 9.00E-18	Identity = 42.73%
	5		gi|109947240|ref|YP_664468.1|	Helicobacter acinonychis str. Sheeba	E-value = 9.00E-18	Identity = 45.87%
AB0209	1			***** No matches found *****		
AB0210	1	*tilS*	gi|152991221|ref|YP_001356943.1|	Nitratiruptor sp. SB155-2	E-value = 1.00E-70	Identity = 50.64%
	2		gi|154175317|ref|YP_001408589.1|	Campylobacter curvus 525.92	E-value = 2.00E-69	Identity = 46.52%
	3		gi|152993772|ref|YP_001359493.1|	Sulfurovum sp. NBC37-1	E-value = 4.00E-66	Identity = 48.15%
	4		gi|157165572|ref|YP_001467030.1|	Campylobacter concisus 13826	E-value = 3.00E-65	Identity = 46.42%
	5		gi|78777932|ref|YP_394247.1|	Sulfuromonas denitrificans ATCC 33889	E-value = 1.00E-64	Identity = 46.15%
AB0211	1	*yliG*	gi|152993771|ref|YP_001359492.1|	Sulfurovum sp. NBC37-1	E-value = 7.00E-160	Identity = 64.61%
	2		gi|78777931|ref|YP_394246.1|	Sulfuromonas denitrificans ATCC 33889	E-value = 1.00E-156	Identity = 63.43%
	3		gi|152991220|ref|YP_001356942.1|	Nitratiruptor sp. SB155-2	E-value = 1.00E-147	Identity = 60.46%
	4		gi|34558076|ref|NP_907891.1|	Wolinella succinogenes DSM 1740	E-value = 5.00E-136	Identity = 57.67%
	5		gi|121613708|ref|YP_001001103.1|	Campylobacter jejuni subsp. jejuni 81-176	E-value = 2.00E-134	Identity = 56.09%
AB0212	1	*panC*	gi|152991914|ref|YP_001357635.1|	Sulfurovum sp. NBC37-1	E-value = 5.00E-89	Identity = 58.76%
	2		gi|152991275|ref|YP_001356997.1|	Nitratiruptor sp. SB155-2	E-value = 5.00E-88	Identity = 58.61%
	3		gi|118474313|ref|YP_892511.1|	Campylobacter fetus subsp. fetus 82-40	E-value = 1.00E-82	Identity = 56.04%
	4		gi|149194097|ref|ZP_01871195.1|	Caminibacter mediatlanticus TB-2	E-value = 2.00E-82	Identity = 58.74%
	5		gi|157165494|ref|YP_001467028.1|	Campylobacter concisus 13826	E-value = 6.00E-81	Identity = 54.95%
AB0213	1		gi|152991228|ref|YP_001356950.1|	Nitratiruptor sp. SB155-2	E-value = 1.00E-98	Identity = 64.86%
	2		gi|78777943|ref|YP_394258.1|	Sulfuromonas denitrificans ATCC 33889	E-value = 1.00E-95	Identity = 62.42%
	3		gi|39996534|ref|NP_952485.1|	Geobacter sulfurreducens PCA	E-value = 3.00E-66	Identity = 46.33%
	4		gi|78222449|ref|YP_384196.1|	Geobacter metallireducens GS-15	E-value = 1.00E-63	Identity = 43.13%
	5		gi|118580430|ref|YP_901680.1|	Pelobacter propionicus DSM 2379	E-value = 4.00E-61	Identity = 43.79%
AB0214	1	*acpS*	gi|15792727|ref|NP_282550.1|	Campylobacter jejuni subsp. jejuni NCTC 11168	E-value = 7.00E-26	Identity = 54.87%
	2		gi|153951701|ref|YP_001398710.1|	Campylobacter jejuni subsp. doylei 269.97	E-value = 2.00E-25	Identity = 53.98%
	3		gi|34558259|ref|NP_908074.1|	Wolinella succinogenes DSM 1740	E-value = 2.00E-25	Identity = 54.05%
	4		gi|57238446|ref|YP_179577.1|	Campylobacter jejuni subsp. jejuni RM1221	E-value = 3.00E-25	Identity = 53.98%
	5		gi|157415642|ref|YP_001482898.1|	Campylobacter jejuni subsp. jejuni 81116	E-value = 3.00E-25	Identity = 53.98%
AB0215	1	*gltS*	gi|50084018|ref|YP_045528.1|	Acinetobacter sp. ADP1	E-value = 4.00E-154	Identity = 76.34%
	2		gi|126642266|ref|YP_001085250.1|	Acinetobacter baumannii ATCC 17978	E-value = 2.00E-125	Identity = 76.11%
	3		gi|118051817|ref|ZP_01520365.1|	Comamonas testosteroni KF-1	E-value = 7.00E-125	Identity = 63.02%
	4		gi|121633963|ref|YP_974208.1|	Neisseria meningitidis FAM18	E-value = 1.00E-124	Identity = 62.59%
	5		gi|15676017|ref|NP_273147.1|	Neisseria meningitidis MC58	E-value = 1.00E-124	Identity = 62.34%
AB0216	1		gi|152992044|ref|YP_001357765.1|	Sulfurovum sp. NBC37-1	E-value = 3.00E-39	Identity = 65.87%
	2		gi|152990631|ref|YP_001356353.1|	Nitratiruptor sp. SB155-2	E-value = 6.00E-34	Identity = 58.87%
	3		gi|78776727|ref|YP_393042.1|	Sulfuromonas denitrificans ATCC 33889	E-value = 1.00E-28	Identity = 50.39%
	4		gi|149193947|ref|ZP_01871045.1|	Caminibacter mediatlanticus TB-2	E-value = 8.00E-28	Identity = 54.10%
	5		gi|154174481|ref|YP_001408003.1|	Campylobacter curvus 525.92	E-value = 1.00E-22	Identity = 49.60%
AB0217	1	*metF*	gi|152991052|ref|YP_001356774.1|	Nitratiruptor sp. SB155-2	E-value = 9.00E-106	Identity = 61.06%
	2		gi|78777863|ref|YP_394178.1|	Sulfuromonas denitrificans ATCC 33889	E-value = 3.00E-103	Identity = 62.58%
	3		gi|149195267|ref|ZP_01872356.1|	Caminibacter mediatlanticus TB-2	E-value = 8.00E-91	Identity = 60.07%
	4		gi|34557786|ref|NP_907601.1|	Wolinella succinogenes DSM 1740	E-value = 3.00E-82	Identity = 49.67%
	5		gi|152992236|ref|YP_001357957.1|	Sulfurovum sp. NBC37-1	E-value = 2.00E-81	Identity = 51.97%
AB0218	1	*serB*	gi|78777862|ref|YP_394177.1|	Sulfuromonas denitrificans ATCC 33889	E-value = 5.00E-75	Identity = 72.33%
	2		gi|149194448|ref|ZP_01871545.1|	Caminibacter mediatlanticus TB-2	E-value = 2.00E-72	Identity = 72.82%
	3		gi|152991051|ref|YP_001356773.1|	Nitratiruptor sp. SB155-2	E-value = 3.00E-72	Identity = 70.05%

	4		gi|152992237|ref|YP_001357958.1|	Sulfurovum sp. NBC37-1	E-value = 2.00E-67	Identity = 65.37%
	5		gi|34558365|ref|NP_908180.1|	Wolinella succinogenes DSM 1740	E-value = 1.00E-63	Identity = 63.11%
AB0219	1	*tal*	gi|34558364|ref|NP_908179.1|	Wolinella succinogenes DSM 1740	E-value = 3.00E-89	Identity = 52.62%
	2		gi|152993640|ref|YP_001359361.1|	Sulfurovum sp. NBC37-1	E-value = 5.00E-87	Identity = 52.13%
	3		gi|78776665|ref|YP_392980.1|	Sulfuromonas denitrificans ATCC 33889	E-value = 7.00E-82	Identity = 50.62%
	4		gi|152990131|ref|YP_001355853.1|	Nitratiruptor sp. SB155-2	E-value = 2.00E-78	Identity = 50.15%
	5		gi|82523962|emb|CAI78773.1|	uncultured epsilon proteobacterium	E-value = 5.00E-77	Identity = 48.46%
AB0220	1	*dhs*	gi|152992062|ref|YP_001357783.1|	Sulfurovum sp. NBC37-1	E-value = 0	Identity = 70.18%
	2		gi|152990928|ref|YP_001356650.1|	Nitratiruptor sp. SB155-2	E-value = 0	Identity = 67.86%
	3		gi|66046851|ref|YP_236692.1|	Pseudomonas syringae pv. syringae B728a	E-value = 0	Identity = 65.77%
	4		gi|70731605|ref|YP_261346.1|	Pseudomonas fluorescens Pf-5	E-value = 0	Identity = 66.22%
	5		gi|119945429|ref|YP_943109.1|	Psychromonas ingrahamii 37	E-value = 0	Identity = 68.15%
AB0221	1	*tsx*	gi|116053864|ref|YP_788301.1|	Pseudomonas aeruginosa UCBPP-PA14	E-value = 8.00E-73	Identity = 52.90%
	2		gi|83643131|ref|YP_431566.1|	Hahella chejuensis KCTC 2396	E-value = 5.00E-70	Identity = 56.40%
	3		gi|92112245|ref|YP_572173.1|	Chromohalobacter salexigens DSM 3043	E-value = 1.00E-52	Identity = 44.05%
	4		gi|42522254|ref|NP_967634.1|	Bdellovibrio bacteriovorus HD100	E-value = 3.00E-44	Identity = 39.71%
	5		gi|150423936|gb|EDN15876.1|	Vibrio cholerae AM-19226	E-value = 3.00E-42	Identity = 40.24%
AB0222	1		gi|153094431|gb|EDN75279.1|	Mannheimia haemolytica PHL213	E-value = 0	Identity = 67.11%
	2		gi|86150507|ref|ZP_01068732.1|	Campylobacter jejuni subsp. jejuni CF93-6	E-value = 0	Identity = 67.23%
	3		gi|153951579|ref|YP_001397840.1|	Campylobacter jejuni subsp. doylei 269.97	E-value = 0	Identity = 67.23%
	4		gi|157415308|ref|YP_001482564.1|	Campylobacter jejuni subsp. jejuni 81116	E-value = 0	Identity = 67.23%
	5		gi|148265621|ref|YP_001232327.1|	Geobacter uraniumreducens Rf4	E-value = 0	Identity = 55.95%
AB0225	1		gi|152990793|ref|YP_001356515.1|	Nitratiruptor sp. SB155-2	E-value = 6.00E-29	Identity = 60.18%
	2		gi|149194797|ref|ZP_01871891.1|	Caminibacter mediatlanticus TB-2	E-value = 1.00E-25	Identity = 56.25%
	3		gi|152992082|ref|YP_001357803.1|	Sulfurovum sp. NBC37-1	E-value = 6.00E-24	Identity = 48.67%
	4		gi|34557679|ref|NP_907494.1|	Wolinella succinogenes DSM 1740	E-value = 4.00E-22	Identity = 46.90%
	5		gi|32267261|ref|NP_861293.1|	Helicobacter hepaticus ATCC 51449	E-value = 4.00E-14	Identity = 38.18%
AB0226	1	*gatC*	gi|34556977|ref|NP_906792.1|	Wolinella succinogenes DSM 1740	E-value = 7.00E-21	Identity = 57.29%
	2		gi|118474805|ref|YP_892304.1|	Campylobacter fetus subsp. fetus 82-40	E-value = 9.00E-18	Identity = 54.17%
	3		gi|57168537|ref|ZP_00367670.1|	Campylobacter coli RM2228	E-value = 6.00E-17	Identity = 52.08%
	4		gi|152992083|ref|YP_001357804.1|	Sulfurovum sp. NBC37-1	E-value = 4.00E-16	Identity = 44.79%
	5		gi|157164525|ref|YP_001467458.1|	Campylobacter concisus 13826	E-value = 3.00E-15	Identity = 51.04%
AB0227	1	*tgt*	gi|152992601|ref|YP_001358322.1|	Sulfurovum sp. NBC37-1	E-value = 5.00E-162	Identity = 71.93%
	2		gi|78777105|ref|YP_393420.1|	Sulfuromonas denitrificans ATCC 33889	E-value = 5.00E-162	Identity = 72.31%
	3		gi|118474179|ref|YP_892148.1|	Campylobacter fetus subsp. fetus 82-40	E-value = 2.00E-158	Identity = 72.58%
	4		gi|57241059|ref|ZP_00369006.1|	Campylobacter lari RM2100	E-value = 2.00E-157	Identity = 71.58%
	5		gi|152990829|ref|YP_001356551.1|	Nitratiruptor sp. SB155-2	E-value = 9.00E-155	Identity = 70.16%
AB0228	1		gi|34557851|ref|NP_907666.1|	Wolinella succinogenes DSM 1740	E-value = 7.00E-86	Identity = 37.66%
	2		gi|78777217|ref|YP_393532.1|	Sulfuromonas denitrificans ATCC 33889	E-value = 1.00E-81	Identity = 36.48%
	3		gi|152990828|ref|YP_001356550.1|	Nitratiruptor sp. SB155-2	E-value = 2.00E-80	Identity = 37.05%
	4		gi|154173666|ref|YP_001408232.1|	Campylobacter curvus 525.92	E-value = 5.00E-67	Identity = 35.69%
	5		gi|152992602|ref|YP_001358323.1|	Sulfurovum sp. NBC37-1	E-value = 5.00E-67	Identity = 34.17%
AB0229	1	*aspB2*	gi|152991013|ref|YP_001356735.1|	Nitratiruptor sp. SB155-2	E-value = 4.00E-135	Identity = 66.85%
	2		gi|78776958|ref|YP_393273.1|	Sulfuromonas denitrificans ATCC 33889	E-value = 2.00E-129	Identity = 66.84%
	3		gi|152992004|ref|YP_001357725.1|	Sulfurovum sp. NBC37-1	E-value = 1.00E-128	Identity = 61.08%
	4		gi|34557822|ref|NP_907637.1|	Wolinella succinogenes DSM 1740	E-value = 8.00E-123	Identity = 56.76%
	5		gi|149193792|ref|ZP_01870890.1|	Caminibacter mediatlanticus TB-2	E-value = 5.00E-117	Identity = 62.13%
AB0230	1		gi|152991010|ref|YP_001356732.1|	Nitratiruptor sp. SB155-2	E-value = 9.00E-18	Identity = 34.05%
	2		gi|152992005|ref|YP_001357726.1|	Sulfurovum sp. NBC37-1	E-value = 3.00E-10	Identity = 28.80%

AB0231	1	*murC*	gi|152991009|ref|YP_001356731.1|	Nitratiruptor sp. SB155-2	E-value = 3.00E-141	Identity = 60.46%
	2		gi|149193790|ref|ZP_01870888.1|	Caminibacter mediatlanticus TB-2	E-value = 2.00E-124	Identity = 57.24%
	3		gi|78776956|ref|YP_393271.1|	Sulfuromonas denitrificans ATCC 33889	E-value = 2.00E-124	Identity = 54.82%
	4		gi|154175070|ref|YP_001407775.1|	Campylobacter curvus 525.92	E-value = 4.00E-124	Identity = 55.43%
	5		gi|34557823|ref|NP_907638.1|	Wolinella succinogenes DSM 1740	E-value = 6.00E-116	Identity = 52.87%
AB0232	1	*dprA*	gi|78777855|ref|YP_394170.1|	Sulfuromonas denitrificans ATCC 33889	E-value = 6.00E-76	Identity = 55.69%
	2		gi|152992041|ref|YP_001357762.1|	Sulfurovum sp. NBC37-1	E-value = 7.00E-70	Identity = 51.94%
	3		gi|152990902|ref|YP_001356624.1|	Nitratiruptor sp. SB155-2	E-value = 6.00E-62	Identity = 53.65%
	4		gi|148926621|ref|ZP_01810302.1|	Campylobacter jejuni subsp. jejuni CG8486	E-value = 5.00E-47	Identity = 44.73%
	5		gi|57236947|ref|YP_178748.1|	Campylobacter jejuni subsp. jejuni RM1221	E-value = 6.00E-47	Identity = 44.73%
AB0233	1		gi|78777856|ref|YP_394171.1|	Sulfuromonas denitrificans ATCC 33889	E-value = 1.00E-47	Identity = 36.99%
	2		gi|154148192|ref|YP_001405976.1|	Campylobacter hominis ATCC BAA-381	E-value = 6.00E-38	Identity = 33.71%
	3		gi|86151591|ref|ZP_01069805.1|	Campylobacter jejuni subsp. jejuni 260.94	E-value = 1.00E-37	Identity = 34.35%
	4		gi|121613073|ref|YP_001000334.1|	Campylobacter jejuni subsp. jejuni 81-176	E-value = 9.00E-37	Identity = 32.96%
	5		gi|88597070|ref|ZP_01100306.1|	Campylobacter jejuni subsp. jejuni 84-25	E-value = 1.00E-36	Identity = 34.42%
AB0234	1	*ilvC*	gi|78776740|ref|YP_393055.1|	Sulfuromonas denitrificans ATCC 33889	E-value = 1.00E-160	Identity = 81.47%
	2		gi|152992039|ref|YP_001357760.1|	Sulfurovum sp. NBC37-1	E-value = 5.00E-156	Identity = 81.07%
	3		gi|149193941|ref|ZP_01871039.1|	Caminibacter mediatlanticus TB-2	E-value = 1.00E-149	Identity = 78.57%
	4		gi|152990905|ref|YP_001356627.1|	Nitratiruptor sp. SB155-2	E-value = 1.00E-148	Identity = 76.18%
	5		gi|154174694|ref|YP_001408000.1|	Campylobacter curvus 525.92	E-value = 3.00E-148	Identity = 76.47%
AB0235	1	*rnr*	gi|152990906|ref|YP_001356628.1|	Nitratiruptor sp. SB155-2	E-value = 2.00E-107	Identity = 40.81%
	2		gi|78776742|ref|YP_393057.1|	Sulfuromonas denitrificans ATCC 33889	E-value = 1.00E-104	Identity = 37.79%
	3		gi|152992027|ref|YP_001357748.1|	Sulfurovum sp. NBC37-1	E-value = 2.00E-104	Identity = 40.26%
	4		gi|34558170|ref|NP_907985.1|	Wolinella succinogenes DSM 1740	E-value = 4.00E-97	Identity = 39.26%
	5		gi|118474787|ref|YP_892287.1|	Campylobacter fetus subsp. fetus 82-40	E-value = 2.00E-95	Identity = 37.81%
AB0236	1		gi|78776613|ref|YP_392928.1|	Sulfuromonas denitrificans ATCC 33889	E-value = 9.00E-06	Identity = 28.83%
AB0237	1		gi|78776741|ref|YP_393056.1|	Sulfuromonas denitrificans ATCC 33889	E-value = 1.00E-53	Identity = 40.50%
	2		gi|152990907|ref|YP_001356629.1|	Nitratiruptor sp. SB155-2	E-value = 2.00E-40	Identity = 36.90%
	3		gi|152992026|ref|YP_001357747.1|	Sulfurovum sp. NBC37-1	E-value = 6.00E-39	Identity = 31.46%
	4		gi|34558169|ref|NP_907984.1|	Wolinella succinogenes DSM 1740	E-value = 6.00E-33	Identity = 30.86%
	5		gi|118475683|ref|YP_892288.1|	Campylobacter fetus subsp. fetus 82-40	E-value = 2.00E-31	Identity = 32.09%
AB0238	1	*rpsF*	gi|34558168|ref|NP_907983.1|	Wolinella succinogenes DSM 1740	E-value = 4.00E-28	Identity = 58.41%
	2		gi|109946866|ref|YP_664094.1|	Helicobacter acinonychis str. Sheeba	E-value = 9.00E-26	Identity = 56.14%
	3		gi|32266035|ref|NP_860067.1|	Helicobacter hepaticus ATCC 51449	E-value = 1.00E-25	Identity = 58.04%
	4		gi|108563614|ref|YP_627930.1|	Helicobacter pylori HPAG1	E-value = 4.00E-25	Identity = 55.26%
	5		gi|15645860|ref|NP_208038.1|	Helicobacter pylori 26695	E-value = 5.00E-25	Identity = 55.26%
AB0239	1	*ssb*	gi|34558167|ref|NP_907982.1|	Wolinella succinogenes DSM 1740	E-value = 2.00E-42	Identity = 56.47%
	2		gi|152992023|ref|YP_001357744.1|	Sulfurovum sp. NBC37-1	E-value = 5.00E-41	Identity = 56.98%
	3		gi|152990909|ref|YP_001356631.1|	Nitratiruptor sp. SB155-2	E-value = 5.00E-40	Identity = 55.29%
	4		gi|32266036|ref|NP_860068.1|	Helicobacter hepaticus ATCC 51449	E-value = 1.00E-37	Identity = 48.28%
	5		gi|78777858|ref|YP_394173.1|	Sulfuromonas denitrificans ATCC 33889	E-value = 1.00E-35	Identity = 49.71%
AB0240	1	*rpsR*	gi|157164150|ref|YP_001467166.1|	Campylobacter concisus 13826	E-value = 3.00E-22	Identity = 69.88%
	2		gi|57240364|ref|ZP_00368313.1|	Campylobacter lari RM2100	E-value = 4.00E-22	Identity = 71.08%
	3		gi|118475552|ref|YP_892291.1|	Campylobacter fetus subsp. fetus 82-40	E-value = 6.00E-22	Identity = 72.29%
	4		gi|154174830|ref|YP_001407994.1|	Campylobacter curvus 525.92	E-value = 8.00E-22	Identity = 69.88%
	5		gi|57242167|ref|ZP_00370107.1|	Campylobacter upsaliensis RM3195	E-value = 1.00E-21	Identity = 69.88%
AB0241	1			***** No matches found *****		
AB0242	1	*cysS*	gi|152990845|ref|YP_001356567.1|	Nitratiruptor sp. SB155-2	E-value = 2.00E-167	Identity = 64.01%
	2		gi|152992414|ref|YP_001358135.1|	Sulfurovum sp. NBC37-1	E-value = 1.00E-162	Identity = 63.44%

	3		gi|149194602|ref|ZP_01871698.1|	Caminibacter mediatlanticus TB-2	E-value = 2.00E-160	Identity = 64.81%
	4		gi|34556870|ref|NP_906685.1|	Wolinella succinogenes DSM 1740	E-value = 4.00E-151	Identity = 58.28%
	5		gi|154175222|ref|YP_001408206.1|	Campylobacter curvus 525.92	E-value = 5.00E-150	Identity = 56.68%
AB0243	1	*nusA*	gi|152991227|ref|YP_001356949.1|	Nitratiruptor sp. SB155-2	E-value = 2.00E-97	Identity = 55.00%
	2		gi|34558085|ref|NP_907900.1|	Wolinella succinogenes DSM 1740	E-value = 2.00E-91	Identity = 51.15%
	3		gi|157165500|ref|YP_001467013.1|	Campylobacter concisus 13826	E-value = 9.00E-90	Identity = 54.22%
	4		gi|154175154|ref|YP_001408581.1|	Campylobacter curvus 525.92	E-value = 1.00E-89	Identity = 52.31%
	5		gi|78777942|ref|YP_394257.1|	Sulfuromonas denitrificans ATCC 33889	E-value = 7.00E-89	Identity = 49.49%
AB0244	1		gi|118475003|ref|YP_892519.1|	Campylobacter fetus subsp. fetus 82-40	E-value = 8.00E-18	Identity = 63.75%
	2		gi|154173644|ref|YP_001408582.1|	Campylobacter curvus 525.92	E-value = 1.00E-17	Identity = 62.50%
	3		gi|86153745|ref|ZP_01071948.1|	Campylobacter jejuni subsp. jejuni HB93-13	E-value = 7.00E-17	Identity = 58.75%
	4		gi|57168595|ref|ZP_00367728.1|	Campylobacter coli RM2228	E-value = 1.00E-16	Identity = 58.75%
	5		gi|57242308|ref|ZP_00370247.1|	Campylobacter upsaliensis RM3195	E-value = 1.00E-16	Identity = 61.25%
AB0245	1	*miaB*	gi|152991225|ref|YP_001356947.1|	Nitratiruptor sp. SB155-2	E-value = 5.00E-157	Identity = 63.57%
	2		gi|78777940|ref|YP_394255.1|	Sulfuromonas denitrificans ATCC 33889	E-value = 2.00E-149	Identity = 60.46%
	3		gi|86148942|ref|ZP_01067174.1|	Campylobacter jejuni subsp. jejuni CF93-6	E-value = 2.00E-143	Identity = 57.97%
	4		gi|86153933|ref|ZP_01072136.1|	Campylobacter jejuni subsp. jejuni HB93-13	E-value = 3.00E-143	Identity = 57.97%
	5		gi|15791822|ref|NP_281645.1|	Campylobacter jejuni subsp. jejuni NCTC 11168	E-value = 5.00E-143	Identity = 57.74%
AB0246	1		gi|78777939|ref|YP_394254.1|	Sulfuromonas denitrificans ATCC 33889	E-value = 2.00E-51	Identity = 47.26%
	2		gi|152991224|ref|YP_001356946.1|	Nitratiruptor sp. SB155-2	E-value = 4.00E-47	Identity = 45.88%
	3		gi|34558082|ref|NP_907897.1|	Wolinella succinogenes DSM 1740	E-value = 2.00E-45	Identity = 45.60%
	4		gi|154173622|ref|YP_001408584.1|	Campylobacter curvus 525.92	E-value = 1.00E-42	Identity = 44.28%
	5		gi|152993774|ref|YP_001359495.1|	Sulfurovum sp. NBC37-1	E-value = 2.00E-42	Identity = 42.57%
AB0247	1			***** No matches found *****		
AB0248	1		gi|152990397|ref|YP_001356119.1|	Nitratiruptor sp. SB155-2	E-value = 1.00E-152	Identity = 71.95%
	2		gi|152992134|ref|YP_001357855.1|	Sulfurovum sp. NBC37-1	E-value = 3.00E-146	Identity = 70.74%
	3		gi|78777672|ref|YP_393987.1|	Sulfuromonas denitrificans ATCC 33889	E-value = 5.00E-145	Identity = 67.71%
	4		gi|157164970|ref|YP_001466574.1|	Campylobacter concisus 13826	E-value = 2.00E-142	Identity = 67.42%
	5		gi|121613371|ref|YP_001001026.1|	Campylobacter jejuni subsp. jejuni 81-176	E-value = 2.00E-139	Identity = 67.90%
AB0249	1			***** No matches found *****		
AB0250	1	*cynT2*	gi|152990078|ref|YP_001355800.1|	Nitratiruptor sp. SB155-2	E-value = 1.00E-63	Identity = 55.24%
	2		gi|78776363|ref|YP_392678.1|	Sulfuromonas denitrificans ATCC 33889	E-value = 9.00E-63	Identity = 51.89%
	3		gi|152991985|ref|YP_001357706.1|	Sulfurovum sp. NBC37-1	E-value = 1.00E-61	Identity = 52.45%
	4		gi|152990058|ref|YP_001355780.1|	Nitratiruptor sp. SB155-2	E-value = 6.00E-59	Identity = 52.71%
	5		gi|46446692|ref|YP_008057.1|	Candidatus Protochlamydia amoebophila UWE25	E-value = 1.00E-56	Identity = 47.20%
AB0251	1	*bioF*	gi|152991945|ref|YP_001357666.1|	Sulfurovum sp. NBC37-1	E-value = 2.00E-107	Identity = 55.83%
	2		gi|78778088|ref|YP_394403.1|	Sulfuromonas denitrificans ATCC 33889	E-value = 9.00E-102	Identity = 55.06%
	3		gi|34558333|ref|NP_908148.1|	Wolinella succinogenes DSM 1740	E-value = 1.00E-93	Identity = 50.83%
	4		gi|152991265|ref|YP_001356987.1|	Nitratiruptor sp. SB155-2	E-value = 2.00E-92	Identity = 53.33%
	5		gi|108563003|ref|YP_627319.1|	Helicobacter pylori HPAG1	E-value = 1.00E-84	Identity = 46.32%
AB0252	1		gi|78778095|ref|YP_394410.1|	Sulfuromonas denitrificans ATCC 33889	E-value = 1.00E-19	Identity = 50.91%
	2		gi|152991827|ref|YP_001357548.1|	Sulfurovum sp. NBC37-1	E-value = 1.00E-16	Identity = 41.90%
	3		gi|32267250|ref|NP_861282.1|	Helicobacter hepaticus ATCC 51449	E-value = 1.00E-15	Identity = 43.12%
	4		gi|34556878|ref|NP_906693.1|	Wolinella succinogenes DSM 1740	E-value = 2.00E-15	Identity = 40.91%
	5		gi|109947298|ref|YP_664526.1|	Helicobacter acinonychis str. Sheeba	E-value = 2.00E-14	Identity = 47.66%
AB0253	1		gi|34558371|ref|NP_908186.1|	Wolinella succinogenes DSM 1740	E-value = 1.00E-12	Identity = 58.46%
	2		gi|152990806|ref|YP_001356528.1|	Nitratiruptor sp. SB155-2	E-value = 2.00E-12	Identity = 59.09%
	3		gi|78777036|ref|YP_393351.1|	Sulfuromonas denitrificans ATCC 33889	E-value = 2.00E-09	Identity = 54.55%
	4		gi|149193982|ref|ZP_01871080.1|	Caminibacter mediatlanticus TB-2	E-value = 2.00E-07	Identity = 53.03%

	5		gi|32266189|ref|NP_860221.1|	Helicobacter hepaticus ATCC 51449	E-value = 8.00E-07	Identity = 42.19%
AB0254	1		gi|83857307|ref|ZP_00950835.1|	Croceibacter atlanticus HTCC2559	E-value = 1.00E-20	Identity = 36.17%
	2		gi|56476024|ref|YP_157613.1|	Azoarcus sp. EbN1	E-value = 2.00E-20	Identity = 33.78%
	3		gi|146276251|ref|YP_001166410.1|	Rhodobacter sphaeroides ATCC 17025	E-value = 3.00E-20	Identity = 33.67%
	4		gi|77464614|ref|YP_354118.1|	Rhodobacter sphaeroides 2.4.1	E-value = 2.00E-19	Identity = 33.17%
	5		gi|69938372|ref|ZP_00632850.1|	Paracoccus denitrificans PD1222	E-value = 3.00E-19	Identity = 36.08%
AB0255	1	*pyk*	gi|123420152|ref|XP_001305702.1|	Trichomonas vaginalis G3	E-value = 9.00E-142	Identity = 55.56%
	2		gi|118475613|ref|YP_891806.1|	Campylobacter fetus subsp. fetus 82-40	E-value = 1.00E-141	Identity = 55.23%
	3		gi|152992032|ref|YP_001357753.1|	Sulfurovum sp. NBC37-1	E-value = 8.00E-139	Identity = 54.60%
	4		gi|57506237|ref|ZP_00372155.1|	Campylobacter upsaliensis RM3195	E-value = 2.00E-136	Identity = 53.44%
	5		gi|86151403|ref|ZP_01069618.1|	Campylobacter jejuni subsp. jejuni 260.94	E-value = 1.00E-134	Identity = 54.07%
AB0256	1	*argD1*	gi|152992186|ref|YP_001357907.1|	Sulfurovum sp. NBC37-1	E-value = 1.00E-145	Identity = 66.16%
	2		gi|34558829|gb|AAQ75173.1|	Alvinella pompejana epibiont 7G3	E-value = 1.00E-142	Identity = 63.36%
	3		gi|152990810|ref|YP_001356532.1|	Nitratiruptor sp. SB155-2	E-value = 1.00E-137	Identity = 63.20%
	4		gi|78777452|ref|YP_393767.1|	Sulfuromonas denitrificans ATCC 33889	E-value = 3.00E-132	Identity = 61.27%
	5		gi|34556738|ref|NP_906553.1|	Wolinella succinogenes DSM 1740	E-value = 1.00E-126	Identity = 59.18%
AB0257	1	*cti*	gi|118580480|ref|YP_901730.1|	Pelobacter propionicus DSM 2379	E-value = 0	Identity = 46.33%
	2		gi|118746541|ref|ZP_01594476.1|	Geobacter lovleyi SZ	E-value = 0	Identity = 46.95%
	3		gi|53804113|ref|YP_114244.1|	Methylococcus capsulatus str. Bath	E-value = 1.00E-134	Identity = 36.36%
	4		gi|91227908|ref|ZP_01262047.1|	Vibrio alginolyticus 12G01	E-value = 3.00E-133	Identity = 36.28%
	5		gi|118589292|ref|ZP_01546698.1|	Stappia aggregata IAM 12614	E-value = 7.00E-131	Identity = 37.00%
AB0258	1	*pyrB*	gi|57240463|ref|ZP_00368412.1|	Campylobacter lari RM2100	E-value = 2.00E-102	Identity = 60.41%
	2		gi|152990125|ref|YP_001355847.1|	Nitratiruptor sp. SB155-2	E-value = 4.00E-98	Identity = 61.30%
	3		gi|86150962|ref|ZP_01069178.1|	Campylobacter jejuni subsp. jejuni 260.94	E-value = 4.00E-97	Identity = 57.82%
	4		gi|157415359|ref|YP_001482615.1|	Campylobacter jejuni subsp. jejuni 81116	E-value = 4.00E-97	Identity = 58.50%
	5		gi|86152461|ref|ZP_01070666.1|	Campylobacter jejuni subsp. jejuni HB93-13	E-value = 4.00E-97	Identity = 58.50%
AB0259	1	*pabB*	gi|152993646|ref|YP_001359367.1|	Sulfurovum sp. NBC37-1	E-value = 2.00E-86	Identity = 54.49%
	2		gi|82523959|emb|CAI78770.1|	uncultured epsilon proteobacterium	E-value = 5.00E-81	Identity = 53.53%
	3		gi|152990124|ref|YP_001355846.1|	Nitratiruptor sp. SB155-2	E-value = 1.00E-79	Identity = 54.28%
	4		gi|78776663|ref|YP_392978.1|	Sulfuromonas denitrificans ATCC 33889	E-value = 9.00E-77	Identity = 51.60%
	5		gi|124006609|ref|ZP_01691441.1|	Microscilla marina ATCC 23134	E-value = 5.00E-73	Identity = 46.63%
AB0260	1		gi|156976003|ref|YP_001446909.1|	Vibrio harveyi ATCC BAA-1116	E-value = 3.00E-50	Identity = 52.72%
	2		gi|153832648|ref|ZP_01985315.1|	Vibrio harveyi HY01	E-value = 9.00E-50	Identity = 52.17%
	3		gi|148975786|ref|ZP_01812617.1|	Vibrionales bacterium SWAT-3	E-value = 7.00E-47	Identity = 51.09%
	4		gi|84387500|ref|ZP_00990518.1|	Vibrio splendidus 12B01	E-value = 1.00E-46	Identity = 52.20%
	5		gi|86145695|ref|ZP_01064024.1|	Vibrio sp. MED222	E-value = 2.00E-46	Identity = 51.10%
AB0261	1		gi|57238514|ref|YP_179645.1|	Campylobacter jejuni subsp. jejuni RM1221	E-value = 2.00E-17	Identity = 30.49%
	2		gi|153951036|ref|YP_001398791.1|	Campylobacter jejuni subsp. doylei 269.97	E-value = 2.00E-17	Identity = 29.28%
	3		gi|86152014|ref|ZP_01070227.1|	Campylobacter jejuni subsp. jejuni 260.94	E-value = 4.00E-17	Identity = 29.28%
	4		gi|86152639|ref|ZP_01070844.1|	Campylobacter jejuni subsp. jejuni HB93-13	E-value = 4.00E-17	Identity = 29.28%
	5		gi|154175183|ref|YP_001407477.1|	Campylobacter curvus 525.92	E-value = 5.00E-17	Identity = 31.51%
AB0262	1		gi|78776635|ref|YP_392950.1|	Sulfuromonas denitrificans ATCC 33889	E-value = 8.00E-36	Identity = 47.62%
	2		gi|152991212|ref|YP_001356934.1|	Nitratiruptor sp. SB155-2	E-value = 1.00E-30	Identity = 40.63%
	3		gi|53713529|ref|YP_099521.1|	Bacteroides fragilis YCH46	E-value = 1.00E-29	Identity = 38.50%
	4		gi|60681814|ref|YP_211958.1|	Bacteroides fragilis NCTC 9343	E-value = 2.00E-29	Identity = 38.50%
	5		gi|118475768|ref|YP_892260.1|	Campylobacter fetus subsp. fetus 82-40	E-value = 3.00E-29	Identity = 44.50%
AB0263	1	*phnA*	gi|78485453|ref|YP_391378.1|	Thiomicrospira crunogena XCL-2	E-value = 6.00E-59	Identity = 61.38%
	2		gi|119468124|ref|ZP_01611250.1|	Alteromonadales bacterium TW-7	E-value = 4.00E-56	Identity = 59.26%
	3		gi|124006643|ref|ZP_01691475.1|	Microscilla marina ATCC 23134	E-value = 2.00E-55	Identity = 60.10%

	4		gi|78776220|ref|YP_392535.1|	Sulfuromonas denitrificans ATCC 33889	E-value = 4.00E-55	Identity = 61.08%
	5		gi|119945955|ref|YP_943635.1|	Psychromonas ingrahamii 37	E-value = 7.00E-55	Identity = 56.02%
AB0264	1		gi|90021186|ref|YP_527013.1|	Saccharophagus degradans 2-40	E-value = 2.00E-49	Identity = 60.74%
	2		gi|123443885|ref|YP_001007856.1|	Yersinia enterocolitica subsp. enterocolitica 8081	E-value = 2.00E-49	Identity = 60.98%
	3		gi|77971985|ref|ZP_00827578.1|	Yersinia frederiksenii ATCC 33641	E-value = 2.00E-49	Identity = 60.37%
	4		gi|77976519|ref|ZP_00832004.1|	Yersinia intermedia ATCC 29909	E-value = 9.00E-49	Identity = 59.76%
	5		gi|148262333|ref|YP_001229039.1|	Geobacter uraniumreducens Rf4	E-value = 2.00E-48	Identity = 59.15%
AB0265	1		gi|123441619|ref|YP_001005604.1|	Yersinia enterocolitica subsp. enterocolitica 8081	E-value = 2.00E-62	Identity = 56.07%
	2		gi|77979078|ref|ZP_00834499.1|	Yersinia intermedia ATCC 29909	E-value = 4.00E-62	Identity = 55.65%
	3		gi|77960622|ref|ZP_00824485.1|	Yersinia mollaretii ATCC 43969	E-value = 4.00E-62	Identity = 56.07%
	4		gi|77956335|ref|ZP_00820444.1|	Yersinia bercovieri ATCC 43970	E-value = 8.00E-62	Identity = 55.23%
	5		gi|77975537|ref|ZP_00831072.1|	Yersinia frederiksenii ATCC 33641	E-value = 8.00E-62	Identity = 54.81%
AB0266	1		gi|51245076|ref|YP_064960.1|	Desulfotalea psychrophila LSv54	E-value = 3.00E-46	Identity = 60.53%
	2		gi|117923778|ref|YP_864395.1|	Magnetococcus sp. MC-1	E-value = 3.00E-44	Identity = 52.57%
	3		gi|110638398|ref|YP_678607.1|	Cytophaga hutchinsonii ATCC 33406	E-value = 6.00E-44	Identity = 53.80%
	4		gi|149275807|ref|ZP_01881952.1|	Pedobacter sp. BAL39	E-value = 4.00E-42	Identity = 52.33%
	5		gi|46581653|ref|YP_012461.1|	Desulfovibrio vulgaris subsp. vulgaris str. Hildenborough	E-value = 1.00E-40	Identity = 50.66%
AB0267	1			***** No matches found *****		
AB0268	1		gi|118474966|ref|YP_891706.1|	Campylobacter fetus subsp. fetus 82-40	E-value = 1.00E-62	Identity = 37.40%
	2		gi|57240322|ref|ZP_00368271.1|	Campylobacter lari RM2100	E-value = 6.00E-59	Identity = 36.17%
	3		gi|86151675|ref|ZP_01069889.1|	Campylobacter jejuni subsp. jejuni 260.94	E-value = 2.00E-55	Identity = 35.19%
	4		gi|157414880|ref|YP_001482136.1|	Campylobacter jejuni subsp. jejuni 81116	E-value = 2.00E-55	Identity = 35.19%
	5		gi|86153513|ref|ZP_01071717.1|	Campylobacter jejuni subsp. jejuni HB93-13	E-value = 2.00E-55	Identity = 35.19%
AB0269	1		gi|118474222|ref|YP_891707.1|	Campylobacter fetus subsp. fetus 82-40	E-value = 7.00E-49	Identity = 44.24%
	2		gi|157164388|ref|YP_001467102.1|	Campylobacter concisus 13826	E-value = 1.00E-48	Identity = 42.42%
	3		gi|154148493|ref|YP_001406772.1|	Campylobacter hominis ATCC BAA-381	E-value = 4.00E-48	Identity = 40.48%
	4		gi|57168163|ref|ZP_00367302.1|	Campylobacter coli RM2228	E-value = 2.00E-44	Identity = 40.81%
	5		gi|57505872|ref|ZP_00371797.1|	Campylobacter upsaliensis RM3195	E-value = 2.00E-44	Identity = 43.20%
AB0270	1			***** No matches found *****		
AB0271	1		gi|152989878|ref|YP_001355600.1|	Nitratiruptor sp. SB155-2	E-value = 3.00E-98	Identity = 59.09%
	2		gi|125972798|ref|YP_001036708.1|	Clostridium thermocellum ATCC 27405	E-value = 3.00E-94	Identity = 56.73%
	3		gi|118474629|ref|YP_892790.1|	Campylobacter fetus subsp. fetus 82-40	E-value = 2.00E-89	Identity = 57.56%
	4		gi|78776235|ref|YP_392550.1|	Sulfuromonas denitrificans ATCC 33889	E-value = 2.00E-87	Identity = 54.05%
	5		gi|154148917|ref|YP_001405667.1|	Campylobacter hominis ATCC BAA-381	E-value = 4.00E-87	Identity = 55.16%
AB0272	1		gi|32265894|ref|NP_859926.1|	Helicobacter hepaticus ATCC 51449	E-value = 4.00E-69	Identity = 42.35%
	2		gi|57168398|ref|ZP_00367532.1|	Campylobacter coli RM2228	E-value = 1.00E-64	Identity = 42.67%
AB0273	1	*prpB*	gi|57168400|ref|ZP_00367534.1|	Campylobacter coli RM2228	E-value = 1.00E-111	Identity = 70.34%
	2		gi|56460535|ref|YP_155816.1|	Idiomarina loihiensis L2TR	E-value = 1.00E-111	Identity = 69.42%
	3		gi|32265896|ref|NP_859928.1|	Helicobacter hepaticus ATCC 51449	E-value = 1.00E-110	Identity = 68.26%
	4		gi|85711677|ref|ZP_01042734.1|	Idiomarina baltica OS145	E-value = 4.00E-110	Identity = 68.38%
	5		gi|127512748|ref|YP_001093945.1|	Shewanella loihica PV-4	E-value = 9.00E-110	Identity = 68.26%
AB0274	1	*prpC*	gi|67153418|ref|ZP_00415163.1|	Azotobacter vinelandii AvOP	E-value = 8.00E-174	Identity = 74.47%
	2		gi|146307109|ref|YP_001187574.1|	Pseudomonas mendocina ymp	E-value = 5.00E-172	Identity = 73.82%
	3		gi|21230490|ref|NP_636407.1|	Xanthomonas campestris pv. campestris str. ATCC 33913	E-value = 8.00E-169	Identity = 73.07%
	4		gi|149926179|ref|ZP_01914441.1|	Limnobacter sp. MED105	E-value = 9.00E-169	Identity = 73.16%
	5		gi|58580516|ref|YP_199532.1|	Xanthomonas oryzae pv. oryzae KACC10331	E-value = 6.00E-168	Identity = 72.53%
AB0275	1	*acnD*	gi|114321750|ref|YP_743433.1|	Alkalilimnicola ehrlichei MLHE-1	E-value = 0	Identity = 68.56%
	2		gi|94500802|ref|ZP_01307331.1|	Oceanobacter sp. RED65	E-value = 0	Identity = 69.77%
	3		gi|127512750|ref|YP_001093947.1|	Shewanella loihica PV-4	E-value = 0	Identity = 69.53%

	4		gi|148548642|ref|YP_001268744.1|	Pseudomonas putida F1	E-value = 0	Identity = 69.37%
	5		gi|26989060|ref|NP_744485.1|	Pseudomonas putida KT2440	E-value = 0	Identity = 69.42%
AB0276	1	*prpF*	gi|146307111|ref|YP_001187576.1|	Pseudomonas mendocina ymp	E-value = 2.00E-149	Identity = 66.41%
	2		gi|146282397|ref|YP_001172550.1|	Pseudomonas stutzeri A1501	E-value = 8.00E-144	Identity = 63.61%
	3		gi|89093303|ref|ZP_01166253.1|	Oceanospirillum sp. MED92	E-value = 2.00E-141	Identity = 64.87%
	4		gi|120554583|ref|YP_958934.1|	Marinobacter aquaeolei VT8	E-value = 8.00E-141	Identity = 64.36%
	5		gi|156974676|ref|YP_001445583.1|	Vibrio harveyi ATCC BAA-1116	E-value = 1.00E-140	Identity = 63.85%
AB0277	1			***** No matches found *****		
AB0278	1		gi|71066487|ref|YP_265214.1|	Psychrobacter arcticus 273-4	E-value = 1.00E-42	Identity = 48.62%
	2		gi|93007049|ref|YP_581486.1|	Psychrobacter cryohalolentis K5	E-value = 1.00E-41	Identity = 47.51%
	3		gi|148653894|ref|YP_001280987.1|	Psychrobacter sp. PRwf-1	E-value = 4.00E-37	Identity = 46.41%
	4		gi|78188151|ref|YP_378489.1|	Chlorobium chlorochromatii CaD3	E-value = 2.00E-34	Identity = 43.68%
	5		gi|67917902|ref|ZP_00511505.1|	Chlorobium limicola DSM 245	E-value = 4.00E-32	Identity = 40.45%
AB0279	1			***** No matches found *****		
AB0280	1	*hemC*	gi|78777061|ref|YP_393376.1|	Sulfuromonas denitrificans ATCC 33889	E-value = 4.00E-93	Identity = 58.31%
	2		gi|152990765|ref|YP_001356487.1|	Nitratiruptor sp. SB155-2	E-value = 4.00E-92	Identity = 58.39%
	3		gi|57241238|ref|ZP_00369185.1|	Campylobacter lari RM2100	E-value = 4.00E-89	Identity = 56.35%
	4		gi|154174368|ref|YP_001408386.1|	Campylobacter curvus 525.92	E-value = 2.00E-88	Identity = 54.84%
	5		gi|154148130|ref|YP_001406461.1|	Campylobacter hominis ATCC BAA-381	E-value = 2.00E-87	Identity = 56.45%
AB0281	1		gi|152992230|ref|YP_001357951.1|	Sulfurovum sp. NBC37-1	E-value = 6.00E-42	Identity = 36.46%
	2		gi|152990766|ref|YP_001356488.1|	Nitratiruptor sp. SB155-2	E-value = 7.00E-41	Identity = 37.87%
	3		gi|78777060|ref|YP_393375.1|	Sulfuromonas denitrificans ATCC 33889	E-value = 1.00E-35	Identity = 37.60%
	4		gi|157415617|ref|YP_001482873.1|	Campylobacter jejuni subsp. jejuni 81116	E-value = 1.00E-06	Identity = 29.79%
	5		gi|91776495|ref|YP_546251.1|	Methylobacillus flagellatus KT	E-value = 7.00E-06	Identity = 25.79%
AB0282	1	*dnaX*	gi|152993623|ref|YP_001359344.1|	Sulfurovum sp. NBC37-1	E-value = 2.00E-131	Identity = 46.28%
	2		gi|78777754|ref|YP_394069.1|	Sulfuromonas denitrificans ATCC 33889	E-value = 5.00E-118	Identity = 51.02%
	3		gi|15645339|ref|NP_207511.1|	Helicobacter pylori 26695	E-value = 2.00E-103	Identity = 42.39%
AB0283	1	*murI*	gi|152993509|ref|YP_001359230.1|	Sulfurovum sp. NBC37-1	E-value = 9.00E-79	Identity = 58.23%
	2		gi|124266488|ref|YP_001020492.1|	Methylibium petroleiphilum PM1	E-value = 3.00E-51	Identity = 43.52%
	3		gi|152980748|ref|YP_001352569.1|	Janthinobacterium sp. Marseille	E-value = 5.00E-48	Identity = 42.22%
	4		gi|146283506|ref|YP_001173659.1|	Pseudomonas stutzeri A1501	E-value = 8.00E-48	Identity = 42.73%
	5		gi|134094154|ref|YP_001099229.1|	Herminiimonas arsenicoxydans	E-value = 3.00E-47	Identity = 42.67%
AB0284	1	*gdhA*	gi|78777707|ref|YP_394022.1|	Sulfuromonas denitrificans ATCC 33889	E-value = 0	Identity = 79.11%
	2		gi|109896617|ref|YP_659872.1|	Pseudoalteromonas atlantica T6c	E-value = 0	Identity = 68.67%
	3		gi|152995919|ref|YP_001340754.1|	Marinomonas sp. MWYL1	E-value = 0	Identity = 69.11%
	4		gi|34558218|ref|NP_908033.1|	Wolinella succinogenes DSM 1740	E-value = 0	Identity = 68.37%
	5		gi|77360335|ref|YP_339910.1|	Pseudoalteromonas haloplanktis TAC125	E-value = 2.00E-180	Identity = 69.33%
AB0285	1	*rho*	gi|152991197|ref|YP_001356919.1|	Nitratiruptor sp. SB155-2	E-value = 0	Identity = 76.43%
	2		gi|34557208|ref|NP_907023.1|	Wolinella succinogenes DSM 1740	E-value = 0	Identity = 76.81%
	3		gi|154173724|ref|YP_001408571.1|	Campylobacter curvus 525.92	E-value = 0	Identity = 73.46%
	4		gi|152993510|ref|YP_001359231.1|	Sulfurovum sp. NBC37-1	E-value = 2.00E-180	Identity = 74.24%
	5		gi|157165083|ref|YP_001467008.1|	Campylobacter concisus 13826	E-value = 1.00E-179	Identity = 72.11%
AB0286	1	*tsaA*	gi|152992346|ref|YP_001358067.1|	Sulfurovum sp. NBC37-1	E-value = 1.00E-91	Identity = 80.81%
	2		gi|78777972|ref|YP_394287.1|	Sulfuromonas denitrificans ATCC 33889	E-value = 6.00E-90	Identity = 78.28%
	3		gi|34558282|ref|NP_908097.1|	Wolinella succinogenes DSM 1740	E-value = 5.00E-88	Identity = 76.77%
	4		gi|57241472|ref|ZP_00369418.1|	Campylobacter lari RM2100	E-value = 5.00E-86	Identity = 76.26%
	5		gi|152990141|ref|YP_001355863.1|	Nitratiruptor sp. SB155-2	E-value = 5.00E-86	Identity = 75.38%
AB0287	1	*fdxA*	gi|152993630|ref|YP_001359351.1|	Sulfurovum sp. NBC37-1	E-value = 3.00E-22	Identity = 71.11%
	2		gi|149194084|ref|ZP_01871182.1|	Caminibacter mediatlanticus TB-2	E-value = 7.00E-22	Identity = 72.22%

	3		gi|118475634|ref|YP_891436.1|	Campylobacter fetus subsp. fetus 82-40	E-value = 2.00E-21	Identity = 73.75%
	4		gi|32266145|ref|NP_860177.1|	Helicobacter hepaticus ATCC 51449	E-value = 3.00E-21	Identity = 74.67%
	5		gi|57168471|ref|ZP_00367605.1|	Campylobacter coli RM2228	E-value = 5.00E-21	Identity = 69.88%
AB0288	1	*ndk*	gi|32266176|ref|NP_860208.1|	Helicobacter hepaticus ATCC 51449	E-value = 3.00E-55	Identity = 81.75%
	2		gi|157163948|ref|YP_001466313.1|	Campylobacter concisus 13826	E-value = 2.00E-54	Identity = 83.21%
	3		gi|154175138|ref|YP_001408843.1|	Campylobacter curvus 525.92	E-value = 1.00E-53	Identity = 83.94%
	4		gi|57506046|ref|ZP_00371969.1|	Campylobacter upsaliensis RM3195	E-value = 1.00E-53	Identity = 83.21%
	5		gi|118474185|ref|YP_891437.1|	Campylobacter fetus subsp. fetus 82-40	E-value = 2.00E-53	Identity = 83.94%
AB0289	1		gi|152990145|ref|YP_001355867.1|	Nitratiruptor sp. SB155-2	E-value = 7.00E-16	Identity = 42.02%
	2		gi|57506045|ref|ZP_00371968.1|	Campylobacter upsaliensis RM3195	E-value = 8.00E-12	Identity = 38.26%
	3		gi|152993628|ref|YP_001359349.1|	Sulfurovum sp. NBC37-1	E-value = 1.00E-11	Identity = 32.00%
	4		gi|118474642|ref|YP_891438.1|	Campylobacter fetus subsp. fetus 82-40	E-value = 5.00E-10	Identity = 37.39%
	5		gi|154174767|ref|YP_001408844.1|	Campylobacter curvus 525.92	E-value = 6.00E-10	Identity = 34.78%
AB0290	1	*rpmF*	gi|78777967|ref|YP_394282.1|	Sulfuromonas denitrificans ATCC 33889	E-value = 4.00E-09	Identity = 81.63%
	2		gi|152993627|ref|YP_001359348.1|	Sulfurovum sp. NBC37-1	E-value = 6.00E-09	Identity = 77.55%
	3		gi|118474248|ref|YP_891439.1|	Campylobacter fetus subsp. fetus 82-40	E-value = 7.00E-09	Identity = 81.25%
	4		gi|157164792|ref|YP_001466311.1|	Campylobacter concisus 13826	E-value = 1.00E-08	Identity = 79.17%
	5		gi|152990146|ref|YP_001355868.1|	Nitratiruptor sp. SB155-2	E-value = 1.00E-08	Identity = 81.63%
AB0291	1	*plsX*	gi|157164231|ref|YP_001466310.1|	Campylobacter concisus 13826	E-value = 1.00E-113	Identity = 66.57%
	2		gi|152990147|ref|YP_001355869.1|	Nitratiruptor sp. SB155-2	E-value = 3.00E-113	Identity = 63.44%
	3		gi|118474989|ref|YP_891440.1|	Campylobacter fetus subsp. fetus 82-40	E-value = 1.00E-109	Identity = 61.89%
	4		gi|154175013|ref|YP_001408845.1|	Campylobacter curvus 525.92	E-value = 4.00E-107	Identity = 64.49%
	5		gi|154148731|ref|YP_001407026.1|	Campylobacter hominis ATCC BAA-381	E-value = 3.00E-105	Identity = 58.61%
AB0292	1	*fabH*	gi|152993625|ref|YP_001359346.1|	Sulfurovum sp. NBC37-1	E-value = 3.00E-125	Identity = 66.77%
	2		gi|78777965|ref|YP_394280.1|	Sulfuromonas denitrificans ATCC 33889	E-value = 8.00E-122	Identity = 65.36%
	3		gi|152990148|ref|YP_001355870.1|	Nitratiruptor sp. SB155-2	E-value = 4.00E-121	Identity = 64.63%
	4		gi|34558276|ref|NP_908091.1|	Wolinella succinogenes DSM 1740	E-value = 4.00E-120	Identity = 61.47%
	5		gi|154173643|ref|YP_001408846.1|	Campylobacter curvus 525.92	E-value = 2.00E-109	Identity = 60.91%
AB0293	1		gi|152991213|ref|YP_001356935.1|	Nitratiruptor sp. SB155-2	E-value = 1.00E-21	Identity = 39.16%
	2		gi|78776636|ref|YP_392951.1|	Sulfuromonas denitrificans ATCC 33889	E-value = 4.00E-21	Identity = 39.87%
	3		gi|152991982|ref|YP_001357703.1|	Sulfurovum sp. NBC37-1	E-value = 1.00E-20	Identity = 39.01%
	4		gi|57237982|ref|YP_179231.1|	Campylobacter jejuni subsp. jejuni RM1221	E-value = 2.00E-20	Identity = 43.26%
	5		gi|34557424|ref|NP_907239.1|	Wolinella succinogenes DSM 1740	E-value = 2.00E-20	Identity = 39.61%
AB0294	1		gi|34557720|ref|NP_907535.1|	Wolinella succinogenes DSM 1740	E-value = 9.00E-95	Identity = 35.48%
	2		gi|154174332|ref|YP_001408714.1|	Campylobacter curvus 525.92	E-value = 3.00E-70	Identity = 29.17%
	3		gi|118474116|ref|YP_891638.1|	Campylobacter fetus subsp. fetus 82-40	E-value = 7.00E-61	Identity = 27.64%
	4		gi|157164882|ref|YP_001467249.1|	Campylobacter concisus 13826	E-value = 3.00E-57	Identity = 26.95%
	5		gi|15791779|ref|NP_281602.1|	Campylobacter jejuni subsp. jejuni NCTC 11168	E-value = 1.00E-55	Identity = 26.97%
AB0295	1		gi|34557719|ref|NP_907534.1|	Wolinella succinogenes DSM 1740	E-value = 3.00E-107	Identity = 34.89%
	2		gi|32266604|ref|NP_860636.1|	Helicobacter hepaticus ATCC 51449	E-value = 1.00E-85	Identity = 30.83%
	3		gi|157414706|ref|YP_001481962.1|	Campylobacter jejuni subsp. jejuni 81116	E-value = 4.00E-59	Identity = 30.25%
	4		gi|153951747|ref|YP_001398547.1|	Campylobacter jejuni subsp. doylei 269.97	E-value = 7.00E-58	Identity = 30.19%
	5		gi|15791778|ref|NP_281601.1|	Campylobacter jejuni subsp. jejuni NCTC 11168	E-value = 8.00E-58	Identity = 30.32%
AB0296	1	*frdB*	gi|157164335|ref|YP_001467268.1|	Campylobacter concisus 13826	E-value = 2.00E-109	Identity = 74.68%
	2		gi|118474349|ref|YP_891616.1|	Campylobacter fetus subsp. fetus 82-40	E-value = 3.00E-108	Identity = 73.42%
	3		gi|154175345|ref|YP_001408712.1|	Campylobacter curvus 525.92	E-value = 8.00E-108	Identity = 72.57%
	4		gi|57240866|ref|ZP_00368814.1|	Campylobacter lari RM2100	E-value = 2.00E-104	Identity = 71.73%
	5		gi|154148968|ref|YP_001406422.1|	Campylobacter hominis ATCC BAA-381	E-value = 5.00E-103	Identity = 70.82%
AB0297	1	*frdA*	gi|118475601|ref|YP_891617.1|	Campylobacter fetus subsp. fetus 82-40	E-value = 0	Identity = 76.93%

	2		gi|154174944|ref|YP_001408711.1|	Campylobacter curvus 525.92	E-value = 0	Identity = 77.54%
	3		gi|157165747|ref|YP_001467269.1|	Campylobacter concisus 13826	E-value = 0	Identity = 76.23%
	4		gi|34557227|ref|NP_907042.1|	Wolinella succinogenes DSM 1740	E-value = 0	Identity = 74.89%
	5		gi|154148872|ref|YP_001406421.1|	Campylobacter hominis ATCC BAA-381	E-value = 0	Identity = 73.14%
AB0298	1	*frdC*	gi|153952486|ref|YP_001398550.1|	Campylobacter jejuni subsp. doylei 269.97	E-value = 8.00E-77	Identity = 56.49%
	2		gi|15791775|ref|NP_281598.1|	Campylobacter jejuni subsp. jejuni NCTC 11168	E-value = 9.00E-77	Identity = 56.49%
	3		gi|90704622|emb|CAF31348.1|	Campylobacter jejuni	E-value = 2.00E-76	Identity = 56.11%
	4		gi|148926499|ref|ZP_01810182.1|	Campylobacter jejuni subsp. jejuni CG8486	E-value = 4.00E-76	Identity = 56.49%
	5		gi|57506240|ref|ZP_00372158.1|	Campylobacter upsaliensis RM3195	E-value = 5.00E-75	Identity = 55.34%
AB0299	1	*nuoN*	gi|118746651|ref|ZP_01594580.1|	Geobacter lovleyi SZ	E-value = 1.00E-63	Identity = 38.13%
	2		gi|145617426|ref|ZP_01773491.1|	Geobacter bemidjiensis Bem	E-value = 3.00E-61	Identity = 36.44%
	3		gi|39995460|ref|NP_951411.1|	Geobacter sulfurreducens PCA	E-value = 9.00E-61	Identity = 38.05%
	4		gi|148266241|ref|YP_001232947.1|	Geobacter uraniumreducens Rf4	E-value = 5.00E-60	Identity = 35.44%
	5		gi|91203934|emb|CAJ71587.1|	Candidatus Kuenenia stuttgartiensis	E-value = 2.00E-59	Identity = 37.30%
AB0300	1	*nuoM*	gi|118474847|ref|YP_891373.1|	Campylobacter fetus subsp. fetus 82-40	E-value = 3.00E-92	Identity = 42.33%
	2		gi|78224533|ref|YP_386280.1|	Geobacter metallireducens GS-15	E-value = 5.00E-88	Identity = 39.05%
	3		gi|148266242|ref|YP_001232948.1|	Geobacter uraniumreducens Rf4	E-value = 2.00E-87	Identity = 38.70%
	4		gi|118340557|gb|ABK80607.1|	uncultured Nitrospinaceae bacterium	E-value = 5.00E-87	Identity = 38.65%
	5		gi|110599304|ref|ZP_01387544.1|	Geobacter sp. FRC-32	E-value = 7.00E-85	Identity = 37.63%
AB0301	1	*nuoL*	gi|149280559|ref|ZP_01886675.1|	Pedobacter sp. BAL39	E-value = 2.00E-127	Identity = 46.40%
	2		gi|118475000|ref|YP_891372.1|	Campylobacter fetus subsp. fetus 82-40	E-value = 1.00E-123	Identity = 47.69%
	3		gi|91203930|emb|CAJ71583.1|	Candidatus Kuenenia stuttgartiensis	E-value = 6.00E-116	Identity = 42.48%
	4		gi|110637776|ref|YP_677983.1|	Cytophaga hutchinsonii ATCC 33406	E-value = 5.00E-113	Identity = 43.39%
	5		gi|24213586|ref|NP_711067.1|	Leptospira interrogans serovar Lai str. 56601	E-value = 2.00E-111	Identity = 41.16%
AB0302	1	*nuoK*	gi|145225070|ref|YP_001135748.1|	Mycobacterium gilvum PYR-GCK	E-value = 7.00E-12	Identity = 41.94%
	2		gi|15612256|ref|NP_223909.1|	Helicobacter pylori J99	E-value = 2.00E-11	Identity = 41.84%
	3		gi|51892734|ref|YP_075425.1|	Symbiobacterium thermophilum IAM 14863	E-value = 3.00E-11	Identity = 41.67%
	4		gi|118045956|ref|ZP_01514611.1|	Chloroflexus aggregans DSM 9485	E-value = 4.00E-11	Identity = 41.84%
	5		gi|41409309|ref|NP_962145.1|	Mycobacterium avium subsp. paratuberculosis K-10	E-value = 4.00E-11	Identity = 43.18%
AB0303	1	*nuoJ*	gi|118475160|ref|YP_891370.1|	Campylobacter fetus subsp. fetus 82-40	E-value = 3.00E-18	Identity = 41.21%
	2		gi|146298997|ref|YP_001193588.1|	Flavobacterium johnsoniae UW101	E-value = 1.00E-17	Identity = 40.72%
	3		gi|94985025|ref|YP_604389.1|	Deinococcus geothermalis DSM 11300	E-value = 4.00E-16	Identity = 38.41%
	4		gi|150026253|ref|YP_001297079.1|	Flavobacterium psychrophilum JIP02/86	E-value = 4.00E-16	Identity = 39.51%
	5		gi|110637778|ref|YP_677985.1|	Cytophaga hutchinsonii ATCC 33406	E-value = 2.00E-14	Identity = 36.14%
AB0304	1	*nuoI*	gi|118475180|ref|YP_891369.1|	Campylobacter fetus subsp. fetus 82-40	E-value = 3.00E-45	Identity = 59.87%
	2		gi|95928438|ref|ZP_01311186.1|	Desulfuromonas acetoxidans DSM 684	E-value = 5.00E-24	Identity = 46.62%
	3		gi|153891405|ref|ZP_02012421.1|	Opitutaceae bacterium TAV2	E-value = 9.00E-24	Identity = 41.25%
	4		gi|116329922|ref|YP_799640.1|	Leptospira borgpetersenii serovar Hardjo-bovis JB197	E-value = 1.00E-23	Identity = 39.38%
	5		gi|118746656|ref|ZP_01594585.1|	Geobacter lovleyi SZ	E-value = 2.00E-23	Identity = 47.06%
AB0305	1	*nuoH*	gi|118475393|ref|YP_891368.1|	Campylobacter fetus subsp. fetus 82-40	E-value = 9.00E-111	Identity = 52.35%
	2		gi|42525212|ref|NP_970592.1|	Bdellovibrio bacteriovorus HD100	E-value = 3.00E-60	Identity = 35.28%
	3		gi|118579074|ref|YP_900324.1|	Pelobacter propionicus DSM 2379	E-value = 5.00E-53	Identity = 36.47%
	4		gi|116619259|ref|YP_821415.1|	Solibacter usitatus Ellin6076	E-value = 2.00E-51	Identity = 35.55%
	5		gi|119714791|ref|YP_921756.1|	Nocardioides sp. JS614	E-value = 2.00E-49	Identity = 37.85%
AB0306	1	*nuoG*	gi|118475236|ref|YP_891367.1|	Campylobacter fetus subsp. fetus 82-40	E-value = 6.00E-93	Identity = 45.09%
	2		gi|42524472|ref|NP_969852.1|	Bdellovibrio bacteriovorus HD100	E-value = 1.00E-75	Identity = 36.63%
	3		gi|115378565|ref|ZP_01465720.1|	Stigmatella aurantiaca DW4/3-1	E-value = 3.00E-72	Identity = 33.55%
	4		gi|66219968|gb|AAY43001.1|	Rhodothermus marinus	E-value = 8.00E-71	Identity = 33.99%
	5		gi|153891409|ref|ZP_02012425.1|	Opitutaceae bacterium TAV2	E-value = 1.00E-67	Identity = 34.23%

AB0307	1	*gltA*	gi|152993967|ref|YP_001359688.1|	Sulfurovum sp. NBC37-1	E-value = 2.00E-165	Identity = 64.17%
	2		gi|78778294|ref|YP_394609.1|	Sulfuromonas denitrificans ATCC 33889	E-value = 6.00E-152	Identity = 61.12%
	3		gi|157164090|ref|YP_001467616.1|	Campylobacter concisus 13826	E-value = 3.00E-148	Identity = 59.95%
	4		gi|154175359|ref|YP_001408978.1|	Campylobacter curvus 525.92	E-value = 8.00E-146	Identity = 58.82%
	5		gi|118475504|ref|YP_891383.1|	Campylobacter fetus subsp. fetus 82-40	E-value = 2.00E-144	Identity = 58.25%
AB0308	1	*nuoF*	gi|118474520|ref|YP_891366.1|	Campylobacter fetus subsp. fetus 82-40	E-value = 4.00E-116	Identity = 55.21%
	2		gi|42524473|ref|NP_969853.1|	Bdellovibrio bacteriovorus HD100	E-value = 1.00E-109	Identity = 47.33%
	3		gi|66219967|gb|AAY43000.1|	Rhodothermus marinus	E-value = 2.00E-106	Identity = 46.44%
	4		gi|72163089|ref|YP_290746.1|	Thermobifida fusca YX	E-value = 5.00E-106	Identity = 45.99%
	5		gi|83814696|ref|YP_444543.1|	Salinibacter ruber DSM 13855	E-value = 8.00E-106	Identity = 46.17%
AB0309	1	*nuoE*	gi|42520577|ref|NP_966492.1|	Wolbachia endosymbiont of Drosophila melanogaster	E-value = 2.00E-36	Identity = 50.97%
	2		gi|58699915|ref|ZP_00374508.1|	Wolbachia endosymbiont of Drosophila ananassae	E-value = 3.00E-36	Identity = 50.97%
	3		gi|58584851|ref|YP_198424.1|	Wolbachia endosymbiont strain TRS of Brugia malayi	E-value = 6.00E-36	Identity = 50.00%
	4		gi|116329150|ref|YP_798870.1|	Leptospira borgpetersenii serovar Hardjo-bovis L550	E-value = 3.00E-35	Identity = 44.52%
	5		gi|24213591|ref|NP_711072.1|	Leptospira interrogans serovar Lai str. 56601	E-value = 6.00E-35	Identity = 44.59%
AB0310	1	*nuoCD*	gi|118474354|ref|YP_891364.1|	Campylobacter fetus subsp. fetus 82-40	E-value = 4.00E-175	Identity = 54.98%
	2		gi|42524475|ref|NP_969855.1|	Bdellovibrio bacteriovorus HD100	E-value = 1.00E-149	Identity = 49.34%
	3		gi|108758426|ref|YP_630946.1|	Myxococcus xanthus DK 1622	E-value = 8.00E-111	Identity = 48.56%
	4		gi|115378554|ref|ZP_01465709.1|	Stigmatella aurantiaca DW4/3-1	E-value = 1.00E-110	Identity = 48.60%
	5		gi|15606519|ref|NP_213899.1|	Aquifex aeolicus VF5	E-value = 5.00E-100	Identity = 37.14%
AB0311	1	*nuoB*	gi|118474832|ref|YP_891363.1|	Campylobacter fetus subsp. fetus 82-40	E-value = 3.00E-72	Identity = 71.60%
	2		gi|91203915|emb|CAJ71568.1|	Candidatus Kuenenia stuttgartiensis	E-value = 8.00E-56	Identity = 61.94%
	3		gi|113939599|ref|ZP_01425451.1|	Herpetosiphon aurantiacus ATCC 23779	E-value = 2.00E-53	Identity = 60.26%
	4		gi|146299005|ref|YP_001193596.1|	Flavobacterium johnsoniae UW101	E-value = 3.00E-53	Identity = 56.10%
	5		gi|153891414|ref|ZP_02012430.1|	Opitutaceae bacterium TAV2	E-value = 3.00E-53	Identity = 62.16%
AB0312	1	*nuoA*	gi|118474143|ref|YP_891362.1|	Campylobacter fetus subsp. fetus 82-40	E-value = 2.00E-19	Identity = 50.00%
	2		gi|146299006|ref|YP_001193597.1|	Flavobacterium johnsoniae UW101	E-value = 1.00E-17	Identity = 47.27%
	3		gi|150026263|ref|YP_001297089.1|	Flavobacterium psychrophilum JIP02/86	E-value = 9.00E-17	Identity = 49.07%
	4		gi|149280570|ref|ZP_01886686.1|	Pedobacter sp. BAL39	E-value = 4.00E-16	Identity = 47.27%
	5		gi|110599316|ref|ZP_01387556.1|	Geobacter sp. FRC-32	E-value = 6.00E-15	Identity = 45.45%
AB0313	1		gi|15615875|ref|NP_244179.1|	Bacillus halodurans C-125	E-value = 8.00E-91	Identity = 42.97%
	2		gi|56964652|ref|YP_176383.1|	Bacillus clausii KSM-K16	E-value = 5.00E-84	Identity = 42.05%
	3		gi|29377360|ref|NP_816514.1|	Enterococcus faecalis V583	E-value = 9.00E-83	Identity = 40.62%
	4		gi|154494442|ref|ZP_02033762.1|	Parabacteroides merdae ATCC 43184	E-value = 2.00E-82	Identity = 40.87%
	5		gi|145622251|ref|ZP_01778212.1|	Petrotoga mobilis SJ95	E-value = 9.00E-82	Identity = 41.39%
AB0314	1		gi|152992345|ref|YP_001358066.1|	Sulfurovum sp. NBC37-1	E-value = 0	Identity = 60.93%
	2		gi|78777062|ref|YP_393377.1|	Sulfuromonas denitrificans ATCC 33889	E-value = 0	Identity = 60.33%
	3		gi|34557868|ref|NP_907683.1|	Wolinella succinogenes DSM 1740	E-value = 0	Identity = 57.02%
	4		gi|152990593|ref|YP_001356315.1|	Nitratiruptor sp. SB155-2	E-value = 0	Identity = 62.1%
	5		gi|154175363|ref|YP_001408383.1|	Campylobacter curvus 525.92	E-value = 0	Identity = 56.02%
AB0315	1		gi|117618621|ref|YP_857867.1|	Aeromonas hydrophila subsp. hydrophila ATCC 7966	E-value = 8.00E-37	Identity = 54.84%
	2		gi|145297972|ref|YP_001140813.1|	Aeromonas salmonicida subsp. salmonicida A449	E-value = 4.00E-36	Identity = 54.19%
	3		gi|119945037|ref|YP_942717.1|	Psychromonas ingrahamii 37	E-value = 8.00E-34	Identity = 51.61%
	4		gi|90409110|ref|ZP_01217232.1|	Psychromonas sp. CNPT3	E-value = 2.00E-33	Identity = 51.61%
	5		gi|156973125|ref|YP_001444032.1|	Vibrio harveyi ATCC BAA-1116	E-value = 1.00E-30	Identity = 48.05%
AB0316	1		gi|68248722|ref|YP_247834.1|	Haemophilus influenzae 86-028NP	E-value = 3.00E-58	Identity = 45.31%
	2		gi|148827252|ref|YP_001292005.1|	Haemophilus influenzae PittGG	E-value = 2.00E-57	Identity = 45.67%
	3		gi|145629003|ref|ZP_01784802.1|	Haemophilus influenzae 22.1-21	E-value = 3.00E-57	Identity = 45.67%
	4		gi|145631513|ref|ZP_01787281.1|	Haemophilus influenzae 22.4-21	E-value = 3.00E-57	Identity = 45.67%

	5		gi|42632166|ref|ZP_00157704.1|	Haemophilus influenzae R2866	E-value = 3.00E-57	Identity = 45.67%
AB0317	1		gi|152992018|ref|YP_001357739.1|	Sulfurovum sp. NBC37-1	E-value = 5.00E-15	Identity = 40.95%
	2		gi|78777085|ref|YP_393400.1|	Sulfuromonas denitrificans ATCC 33889	E-value = 3.00E-13	Identity = 36.79%
	3		gi|152990915|ref|YP_001356637.1|	Nitratiruptor sp. SB155-2	E-value = 1.00E-10	Identity = 36.19%
AB0318	1		gi|32265649|ref|NP_859681.1|	Helicobacter hepaticus ATCC 51449	E-value = 5.00E-42	Identity = 42.44%
	2		gi|118474364|ref|YP_892308.1|	Campylobacter fetus subsp. fetus 82-40	E-value = 1.00E-41	Identity = 45.19%
	3		gi|157164936|ref|YP_001467454.1|	Campylobacter concisus 13826	E-value = 2.00E-40	Identity = 46.31%
	4		gi|34557201|ref|NP_907016.1|	Wolinella succinogenes DSM 1740	E-value = 1.00E-39	Identity = 39.90%
	5		gi|119390595|pdb|2NRH|A	Campylobacter jejuni	E-value = 5.00E-39	Identity = 44.93%
AB0319	1	*hisG*	gi|34557202|ref|NP_907017.1|	Wolinella succinogenes DSM 1740	E-value = 2.00E-67	Identity = 67.51%
	2		gi|152992020|ref|YP_001357741.1|	Sulfurovum sp. NBC37-1	E-value = 3.00E-66	Identity = 65.33%
	3		gi|154174655|ref|YP_001407781.1|	Campylobacter curvus 525.92	E-value = 5.00E-64	Identity = 63.32%
	4		gi|118475111|ref|YP_892309.1|	Campylobacter fetus subsp. fetus 82-40	E-value = 9.00E-64	Identity = 65.15%
	5		gi|152990913|ref|YP_001356635.1|	Nitratiruptor sp. SB155-2	E-value = 1.00E-62	Identity = 63.86%
AB0320	1		gi|152990912|ref|YP_001356634.1|	Nitratiruptor sp. SB155-2	E-value = 2.00E-35	Identity = 39.13%
	2		gi|152992021|ref|YP_001357742.1|	Sulfurovum sp. NBC37-1	E-value = 2.00E-33	Identity = 36.96%
	3		gi|149194289|ref|ZP_01871386.1|	Caminibacter mediatlanticus TB-2	E-value = 5.00E-30	Identity = 39.57%
	4		gi|78777088|ref|YP_393403.1|	Sulfuromonas denitrificans ATCC 33889	E-value = 1.00E-28	Identity = 35.09%
	5		gi|118474604|ref|YP_891805.1|	Campylobacter fetus subsp. fetus 82-40	E-value = 4.00E-27	Identity = 36.36%
AB0321	1		gi|57505672|ref|ZP_00371598.1|	Campylobacter upsaliensis RM3195	E-value = 1.00E-44	Identity = 28.80%
	2		gi|99080317|ref|YP_612471.1|	Silicibacter sp. TM1040	E-value = 3.00E-43	Identity = 27.29%
	3		gi|34558210|ref|NP_908025.1|	Wolinella succinogenes DSM 1740	E-value = 1.00E-41	Identity = 26.54%
	4		gi|116250723|ref|YP_766561.1|	Rhizobium leguminosarum bv. viciae 3841	E-value = 7.00E-41	Identity = 25.17%
	5		gi|154175470|ref|YP_001408196.1|	Campylobacter curvus 525.92	E-value = 2.00E-38	Identity = 27.53%
AB0322	1		gi|5714597|gb|AAD47989.1|	Pseudomonas sp. R9	E-value = 1.00E-31	Identity = 30.85%
	2		gi|146281368|ref|YP_001171521.1|	Pseudomonas stutzeri A1501	E-value = 6.00E-31	Identity = 26.62%
	3		gi|114881125|ref|YP_758666.1|	Pseudomonas aeruginosa	E-value = 1.00E-30	Identity = 30.74%
	4		gi|152112398|sp|Q8XB94|YJIK_ECO57	Escherichia coli O157:H7	E-value = 3.00E-29	Identity = 35.22%
	5		gi|148550258|ref|YP_001270360.1|	Pseudomonas putida F1	E-value = 1.00E-28	Identity = 31.93%
AB0323	1		gi|78776881|ref|YP_393196.1|	Sulfuromonas denitrificans ATCC 33889	E-value = 2.00E-53	Identity = 56.28%
	2		gi|152991549|ref|YP_001357271.1|	Nitratiruptor sp. SB155-2	E-value = 4.00E-43	Identity = 46.52%
	3		gi|56478901|ref|YP_160490.1|	Azoarcus sp. EbN1	E-value = 9.00E-43	Identity = 46.00%
	4		gi|152993857|ref|YP_001359578.1|	Sulfurovum sp. NBC37-1	E-value = 6.00E-42	Identity = 50.80%
	5		gi|30248823|ref|NP_840893.1|	Nitrosomonas europaea ATCC 19718	E-value = 2.00E-41	Identity = 44.51%
AB0324	1	*trpS*	gi|157164791|ref|YP_001466227.1|	Campylobacter concisus 13826	E-value = 8.00E-112	Identity = 60.31%
	2		gi|118475659|ref|YP_892487.1|	Campylobacter fetus subsp. fetus 82-40	E-value = 4.00E-111	Identity = 62.11%
	3		gi|57505890|ref|ZP_00371815.1|	Campylobacter upsaliensis RM3195	E-value = 4.00E-111	Identity = 62.19%
	4		gi|57168527|ref|ZP_00367660.1|	Campylobacter coli RM2228	E-value = 6.00E-109	Identity = 60.94%
	5		gi|157414683|ref|YP_001481939.1|	Campylobacter jejuni subsp. jejuni 81116	E-value = 8.00E-109	Identity = 61.88%
AB0325	1		gi|118729702|ref|ZP_01578219.1|	Delftia acidovorans SPH-1	E-value = 7.00E-161	Identity = 41.09%
	2		gi|91776737|ref|YP_546493.1|	Methylobacillus flagellatus KT	E-value = 5.00E-158	Identity = 43.42%
	3		gi|120612638|ref|YP_972316.1|	Acidovorax avenae subsp. citrulli AAC00-1	E-value = 1.00E-150	Identity = 42.99%
	4		gi|116694731|ref|YP_728942.1|	Ralstonia eutropha H16	E-value = 6.00E-149	Identity = 39.54%
	5		gi|83746751|ref|ZP_00943799.1|	Ralstonia solanacearum UW551	E-value = 6.00E-132	Identity = 40.40%
AB0326	1		gi|50121670|ref|YP_050837.1|	Erwinia carotovora subsp. atroseptica SCRI1043	E-value = 1.00E-111	Identity = 56.43%
	2		gi|28869771|ref|NP_792390.1|	Pseudomonas syringae pv. tomato str. DC3000	E-value = 3.00E-107	Identity = 53.81%
	3		gi|119858123|ref|ZP_01639548.1|	Pseudomonas putida W619	E-value = 4.00E-104	Identity = 52.59%
	4		gi|66045511|ref|YP_235352.1|	Pseudomonas syringae pv. syringae B728a	E-value = 4.00E-104	Identity = 53.33%
	5		gi|77459502|ref|YP_349009.1|	Pseudomonas fluorescens PfO-1	E-value = 1.00E-102	Identity = 52.80%

AB0328	1	gi|152994433|ref|YP_001339268.1|	Marinomonas sp. MWYL1	E-value = 4.00E-41	Identity = 31.32%
	2	gi|84994027|gb|ABC68353.1|	Pseudomonas putida	E-value = 8.00E-33	Identity = 29.55%
	3	gi|5070638|gb|AAD39224.1|AF149851_5	Pseudomonas stutzeri KC	E-value = 1.00E-31	Identity = 26.67%
	4	gi|154494179|ref|ZP_02033499.1|	Parabacteroides merdae ATCC 43184	E-value = 1.00E-30	Identity = 25.68%
	5	gi|120609354|ref|YP_969032.1|	Acidovorax avenae subsp. citrulli AAC00-1	E-value = 2.00E-27	Identity = 26.63%
AB0329	1	gi|6959523|gb|AAF33140.1|AF196567_16	Pseudomonas stutzeri	E-value = 6.00E-35	Identity = 26.36%
	2	gi|120609355|ref|YP_969033.1|	Acidovorax avenae subsp. citrulli AAC00-1	E-value = 1.00E-28	Identity = 25.53%
	3	gi|73669884|ref|YP_305899.1|	Methanosarcina barkeri str. Fusaro	E-value = 8.00E-28	Identity = 30.19%
	4	gi|152994432|ref|YP_001339267.1|	Marinomonas sp. MWYL1	E-value = 2.00E-27	Identity = 27.22%
	5	gi|50120479|ref|YP_049646.1|	Erwinia carotovora subsp. atroseptica SCRI1043	E-value = 5.00E-27	Identity = 25.00%
AB0330	1	gi|154173861|ref|YP_001407330.1|	Campylobacter curvus 525.92	E-value = 2.00E-05	Identity = 37.30%
	2	gi|149377009|ref|ZP_01894760.1|	Marinobacter algicola DG893	E-value = 9.00E-05	Identity = 39.71%
	3	gi|88859208|ref|YP_340087.1|	Pseudoalteromonas haloplanktis TAC125	E-value = 9.00E-05	Identity = 38.36%
AB0331	1	gi|34557929|ref|NP_907744.1|	Wolinella succinogenes DSM 1740	E-value = 1.00E-143	Identity = 43.31%
	2	gi|6959518|gb|AAF33135.1|AF196567_11	Pseudomonas stutzeri	E-value = 1.00E-112	Identity = 34.57%
	3	gi|28192385|gb|AAL65282.1|	Pseudomonas fluorescens	E-value = 1.00E-107	Identity = 33.33%
	4	gi|120609356|ref|YP_969034.1|	Acidovorax avenae subsp. citrulli AAC00-1	E-value = 5.00E-107	Identity = 32.60%
	5	gi|84994024|gb|ABC68350.1|	Pseudomonas putida	E-value = 3.00E-103	Identity = 31.42%
AB0332	1	gi|17230117|ref|NP_486665.1|	Nostoc sp. PCC 7120	E-value = 2.00E-23	Identity = 28.38%
	2	gi|34557928|ref|NP_907743.1|	Wolinella succinogenes DSM 1740	E-value = 3.00E-23	Identity = 27.16%
	3	gi|16329200|ref|NP_439928.1|	Synechocystis sp. PCC 6803	E-value = 3.00E-21	Identity = 25.83%
	4	gi|119513795|ref|ZP_01632771.1|	Nodularia spumigena CCY9414	E-value = 2.00E-20	Identity = 27.09%
	5	gi|84994025|gb|ABC68351.1|	Pseudomonas putida	E-value = 9.00E-19	Identity = 25.78%
AB0333	1	gi|114776572|ref|ZP_01451617.1|	Mariprofundus ferrooxydans PV-1	E-value = 5.00E-42	Identity = 60.00%
	2	gi|88857738|ref|ZP_01132381.1|	Pseudoalteromonas tunicata D2	E-value = 1.00E-35	Identity = 56.42%
	3	gi|71282107|ref|YP_269843.1|	Colwellia psychrerythraea 34H	E-value = 2.00E-35	Identity = 50.84%
	4	gi|114772347|ref|ZP_01449722.1|	alpha proteobacterium HTCC2255	E-value = 1.00E-34	Identity = 51.81%
	5	gi|90022928|ref|YP_528755.1|	Saccharophagus degradans 2-40	E-value = 2.00E-34	Identity = 54.24%
AB0334	1	gi|34557631|ref|NP_907446.1|	Wolinella succinogenes DSM 1740	E-value = 1.00E-101	Identity = 54.19%
	2	gi|70730623|ref|YP_260364.1|	Pseudomonas fluorescens Pf-5	E-value = 2.00E-69	Identity = 38.95%
	3	gi|86146498|ref|ZP_01064821.1|	Vibrio sp. MED222	E-value = 2.00E-68	Identity = 37.70%
	4	gi|104781204|ref|YP_607702.1|	Pseudomonas entomophila L48	E-value = 4.00E-64	Identity = 37.53%
	5	gi|70730865|ref|YP_260606.1|	Pseudomonas fluorescens Pf-5	E-value = 7.00E-62	Identity = 39.73%
AB0335	1	gi|34557630|ref|NP_907445.1|	Wolinella succinogenes DSM 1740	E-value = 8.00E-74	Identity = 40.00%
	2	gi|83593671|ref|YP_427423.1|	Rhodospirillum rubrum ATCC 11170	E-value = 7.00E-57	Identity = 34.41%
	3	gi|69935950|ref|ZP_00630837.1|	Paracoccus denitrificans PD1222	E-value = 8.00E-46	Identity = 32.11%
	4	gi|50085819|ref|YP_047329.1|	Acinetobacter sp. ADP1	E-value = 3.00E-41	Identity = 31.88%
	5	gi|17231517|ref|NP_488065.1|	Nostoc sp. PCC 7120	E-value = 3.00E-41	Identity = 30.75%
AB0336	1	gi|34557629|ref|NP_907444.1|	Wolinella succinogenes DSM 1740	E-value = 1.00E-179	Identity = 51.45%
	2	gi|117624159|ref|YP_853072.1|	Escherichia coli APEC O1	E-value = 6.00E-127	Identity = 39.11%
	3	gi|91211196|ref|YP_541182.1|	Escherichia coli UTI89	E-value = 1.00E-126	Identity = 39.11%
	4	gi|134048836|dbj|BAF49458.1|	Klebsiella pneumoniae	E-value = 1.00E-126	Identity = 39.11%
	5	gi|157145181|ref|YP_001452500.1|	Citrobacter koseri ATCC BAA-895	E-value = 2.00E-126	Identity = 39.29%
AB0337	1	gi|34557628|ref|NP_907443.1|	Wolinella succinogenes DSM 1740	E-value = 4.00E-178	Identity = 56.73%
	2	gi|119385741|ref|YP_916796.1|	Paracoccus denitrificans PD1222	E-value = 7.00E-150	Identity = 46.64%
	3	gi|92110320|emb|CAJ87588.1|	Escherichia coli	E-value = 2.00E-140	Identity = 45.25%
	4	gi|83748862|ref|ZP_00945874.1|	Ralstonia solanacearum UW551	E-value = 3.00E-140	Identity = 48.74%
	5	gi|16122161|ref|NP_405474.1|	Yersinia pestis CO92	E-value = 3.00E-140	Identity = 45.25%
AB0338	1	gi|34557929|ref|NP_907744.1|	Wolinella succinogenes DSM 1740	E-value = 5.00E-71	Identity = 29.04%

	2		gi|28192385|gb|AAL65282.1|	Pseudomonas fluorescens	E-value = 3.00E-67	Identity = 28.29%
	3		gi|17231518|ref|NP_488066.1|	Nostoc sp. PCC 7120	E-value = 2.00E-60	Identity = 29.03%
	4		gi|78356643|ref|YP_388092.1|	Desulfovibrio desulfuricans G20	E-value = 4.00E-60	Identity = 28.04%
	5		gi|75907894|ref|YP_322190.1|	Anabaena variabilis ATCC 29413	E-value = 8.00E-58	Identity = 28.59%
AB0339	1		gi|34557928|ref|NP_907743.1|	Wolinella succinogenes DSM 1740	E-value = 7.00E-31	Identity = 29.81%
	2		gi|118475050|ref|YP_892365.1|	Campylobacter fetus subsp. fetus 82-40	E-value = 1.00E-15	Identity = 28.57%
	3		gi|28211088|ref|NP_782032.1|	Clostridium tetani E88	E-value = 3.00E-15	Identity = 25.83%
	4		gi|116696381|ref|YP_841957.1|	Ralstonia eutropha H16	E-value = 2.00E-12	Identity = 26.22%
	5		gi|106894972|ref|ZP_01362082.1|	Clostridium sp. OhILAs	E-value = 2.00E-11	Identity = 29.39%
AB0340	1		gi|34557764|ref|NP_907579.1|	Wolinella succinogenes DSM 1740	E-value = 1.00E-91	Identity = 33.53%
	2		gi|95930822|ref|ZP_01313554.1|	Desulfuromonas acetoxidans DSM 684	E-value = 1.00E-75	Identity = 30.51%
	3		gi|121998685|ref|YP_001003472.1|	Halorhodospira halophila SL1	E-value = 4.00E-70	Identity = 29.86%
	4		gi|121997198|ref|YP_001001985.1|	Halorhodospira halophila SL1	E-value = 1.00E-68	Identity = 28.40%
	5		gi|121997196|ref|YP_001001983.1|	Halorhodospira halophila SL1	E-value = 2.00E-51	Identity = 26.18%
AB0341	1			***** No matches found *****		
AB0342	1		gi|118474270|ref|YP_891230.1|	Campylobacter fetus subsp. fetus 82-40	E-value = 8.00E-63	Identity = 47.27%
	2		gi|57238035|ref|YP_179284.1|	Campylobacter jejuni subsp. jejuni RM1221	E-value = 2.00E-62	Identity = 47.29%
	3		gi|148926168|ref|ZP_01809853.1|	Campylobacter jejuni subsp. jejuni CG8486	E-value = 2.00E-62	Identity = 47.29%
	4		gi|86152811|ref|ZP_01071016.1|	Campylobacter jejuni subsp. jejuni HB93-13	E-value = 2.00E-62	Identity = 47.65%
	5		gi|157415427|ref|YP_001482683.1|	Campylobacter jejuni subsp. jejuni 81116	E-value = 3.00E-62	Identity = 47.29%
AB0343	1	*serS*	gi|152993537|ref|YP_001359258.1|	Sulfurovum sp. NBC37-1	E-value = 1.00E-146	Identity = 64.73%
	2		gi|34557266|ref|NP_907081.1|	Wolinella succinogenes DSM 1740	E-value = 3.00E-139	Identity = 60.51%
	3		gi|157165746|ref|YP_001466225.1|	Campylobacter concisus 13826	E-value = 1.00E-137	Identity = 59.66%
	4		gi|152991122|ref|YP_001356844.1|	Nitratiruptor sp. SB155-2	E-value = 3.00E-136	Identity = 62.80%
	5		gi|78777864|ref|YP_394179.1|	Sulfuromonas denitrificans ATCC 33889	E-value = 2.00E-134	Identity = 59.90%
AB0344	1	*nrfI*	gi|152990931|ref|YP_001356653.1|	Nitratiruptor sp. SB155-2	E-value = 0	Identity = 40.36%
	2		gi|157164310|ref|YP_001466554.1|	Campylobacter concisus 13826	E-value = 0	Identity = 40.34%
	3		gi|118475397|ref|YP_891606.1|	Campylobacter fetus subsp. fetus 82-40	E-value = 0	Identity = 39.66%
	4		gi|154175361|ref|YP_001408628.1|	Campylobacter curvus 525.92	E-value = 0	Identity = 40.54%
	5		gi|154149248|ref|YP_001406884.1|	Campylobacter hominis ATCC BAA-381	E-value = 0	Identity = 39.41%
AB0345	1	*nrfA*	gi|95930440|ref|ZP_01313176.1|	Desulfuromonas acetoxidans DSM 684	E-value = 3.00E-172	Identity = 58.00%
	2		gi|51244196|ref|YP_064080.1|	Desulfotalea psychrophila LSv54	E-value = 2.00E-164	Identity = 55.49%
	3		gi|34557357|ref|NP_907172.1|	Wolinella succinogenes DSM 1740	E-value = 2.00E-158	Identity = 56.57%
	4		gi|5748660|emb|CAB53160.1|	Wolinella succinogenes	E-value = 4.00E-158	Identity = 56.37%
	5		gi|13096571|pdb|1FS7|A	Wolinella succinogenes	E-value = 2.00E-152	Identity = 57.08%
AB0346	1	*nrfH*	gi|17902506|emb|CAD19316.1|	Sulfurospirillum deleyianum	E-value = 1.00E-48	Identity = 56.00%
	2		gi|34557358|ref|NP_907173.1|	Wolinella succinogenes DSM 1740	E-value = 1.00E-47	Identity = 54.29%
	3		gi|51244195|ref|YP_064079.1|	Desulfotalea psychrophila LSv54	E-value = 1.00E-46	Identity = 54.02%
	4		gi|95930441|ref|ZP_01313177.1|	Desulfuromonas acetoxidans DSM 684	E-value = 3.00E-41	Identity = 47.70%
	5		gi|153889342|ref|ZP_02010456.1|	Opitutaceae bacterium TAV2	E-value = 2.00E-28	Identity = 41.88%
AB0347	1			***** No matches found *****		
AB0348	1		gi|34557884|ref|NP_907699.1|	Wolinella succinogenes DSM 1740	E-value = 5.00E-78	Identity = 34.98%
	2		gi|34557353|ref|NP_907168.1|	Wolinella succinogenes DSM 1740	E-value = 9.00E-76	Identity = 39.12%
	3		gi|85711301|ref|ZP_01042360.1|	Idiomarina baltica OS145	E-value = 3.00E-53	Identity = 33.26%
	4		gi|56460645|ref|YP_155926.1|	Idiomarina loihiensis L2TR	E-value = 7.00E-52	Identity = 31.93%
	5		gi|91203573|emb|CAJ71226.1|	Candidatus Kuenenia stuttgartiensis	E-value = 1.00E-49	Identity = 29.96%
AB0349	1		gi|78777717|ref|YP_394032.1|	Sulfuromonas denitrificans ATCC 33889	E-value = 5.00E-43	Identity = 59.18%
	2		gi|152991541|ref|YP_001357263.1|	Nitratiruptor sp. SB155-2	E-value = 3.00E-42	Identity = 62.50%
	3		gi|149194251|ref|ZP_01871348.1|	Caminibacter mediatlanticus TB-2	E-value = 1.00E-26	Identity = 46.51%

AB0350	1	*napD*	gi|78777718|ref|YP_394033.1|	Sulfuromonas denitrificans ATCC 33889	E-value = 4.00E-33	Identity = 60.47%
	2		gi|152991902|ref|YP_001357623.1|	Sulfurovum sp. NBC37-1	E-value = 1.00E-21	Identity = 52.14%
	3		gi|152991542|ref|YP_001357264.1|	Nitratiruptor sp. SB155-2	E-value = 2.00E-20	Identity = 50.44%
	4		gi|34557542|ref|NP_907357.1|	Wolinella succinogenes DSM 1740	E-value = 3.00E-12	Identity = 42.98%
	5		gi|32265662|ref|NP_859694.1|	Helicobacter hepaticus ATCC 51449	E-value = 4.00E-08	Identity = 35.71%
AB0351	1	*napL*	gi|78777716|ref|YP_394031.1|	Sulfuromonas denitrificans ATCC 33889	E-value = 2.00E-59	Identity = 45.08%
	2		gi|152991901|ref|YP_001357622.1|	Sulfurovum sp. NBC37-1	E-value = 2.00E-52	Identity = 38.72%
	3		gi|34557543|ref|NP_907358.1|	Wolinella succinogenes DSM 1740	E-value = 4.00E-45	Identity = 32.66%
	4		gi|152992264|ref|YP_001357985.1|	Sulfurovum sp. NBC37-1	E-value = 7.00E-42	Identity = 32.23%
	5		gi|152991543|ref|YP_001357265.1|	Nitratiruptor sp. SB155-2	E-value = 7.00E-34	Identity = 27.71%
AB0352	1	*napF*	gi|78777715|ref|YP_394030.1|	Sulfuromonas denitrificans ATCC 33889	E-value = 2.00E-39	Identity = 57.32%
	2		gi|152991544|ref|YP_001357266.1|	Nitratiruptor sp. SB155-2	E-value = 1.00E-27	Identity = 45.51%
	3		gi|34557544|ref|NP_907359.1|	Wolinella succinogenes DSM 1740	E-value = 4.00E-26	Identity = 42.68%
	4		gi|152991899|ref|YP_001357620.1|	Sulfurovum sp. NBC37-1	E-value = 2.00E-24	Identity = 44.52%
	5		gi|118474508|ref|YP_892315.1|	Campylobacter fetus subsp. fetus 82-40	E-value = 7.00E-23	Identity = 42.00%
AB0353	1	*napB*	gi|27529609|emb|CAD55550.1|	Wolinella succinogenes	E-value = 9.00E-32	Identity = 44.77%
	2		gi|152991545|ref|YP_001357267.1|	Nitratiruptor sp. SB155-2	E-value = 1.00E-29	Identity = 37.32%
	3		gi|15792121|ref|NP_281944.1|	Campylobacter jejuni subsp. jejuni NCTC 11168	E-value = 4.00E-29	Identity = 39.55%
	4		gi|121613406|ref|YP_001000472.1|	Campylobacter jejuni subsp. jejuni 81-176	E-value = 5.00E-29	Identity = 39.55%
	5		gi|86150158|ref|ZP_01068385.1|	Campylobacter jejuni subsp. jejuni CF93-6	E-value = 5.00E-29	Identity = 39.55%
AB0354	1	*napH*	gi|78777713|ref|YP_394028.1|	Sulfuromonas denitrificans ATCC 33889	E-value = 6.00E-106	Identity = 66.92%
	2		gi|152991546|ref|YP_001357268.1|	Nitratiruptor sp. SB155-2	E-value = 2.00E-97	Identity = 62.12%
	3		gi|152991897|ref|YP_001357618.1|	Sulfurovum sp. NBC37-1	E-value = 3.00E-83	Identity = 55.43%
	4		gi|149194346|ref|ZP_01871443.1|	Caminibacter mediatlanticus TB-2	E-value = 6.00E-76	Identity = 55.64%
	5		gi|34557546|ref|NP_907361.1|	Wolinella succinogenes DSM 1740	E-value = 1.00E-70	Identity = 52.85%
AB0355	1	*napG*	gi|78777712|ref|YP_394027.1|	Sulfuromonas denitrificans ATCC 33889	E-value = 8.00E-101	Identity = 67.86%
	2		gi|152991547|ref|YP_001357269.1|	Nitratiruptor sp. SB155-2	E-value = 2.00E-91	Identity = 62.55%
	3		gi|34557547|ref|NP_907362.1|	Wolinella succinogenes DSM 1740	E-value = 9.00E-89	Identity = 60.31%
	4		gi|152991896|ref|YP_001357617.1|	Sulfurovum sp. NBC37-1	E-value = 2.00E-86	Identity = 56.78%
	5		gi|154149165|ref|YP_001407229.1|	Campylobacter hominis ATCC BAA-381	E-value = 7.00E-79	Identity = 56.57%
AB0356	1	*napA*	gi|78777711|ref|YP_394026.1|	Sulfuromonas denitrificans ATCC 33889	E-value = 0	Identity = 79.06%
	2		gi|152991895|ref|YP_001357616.1|	Sulfurovum sp. NBC37-1	E-value = 0	Identity = 78.16%
	3		gi|22651592|gb|AAM21158.1|	Sulfurospirillum barnesii	E-value = 0	Identity = 76.5%
	4		gi|34557548|ref|NP_907363.1|	Wolinella succinogenes DSM 1740	E-value = 0	Identity = 71.75%
	5		gi|57240429|ref|ZP_00368378.1|	Campylobacter lari RM2100	E-value = 0	Identity = 69.98%
AB0357	1	*dctP*	gi|157163977|ref|YP_001467737.1|	Campylobacter concisus 13826	E-value = 5.00E-109	Identity = 66.25%
	2		gi|154173947|ref|YP_001409177.1|	Campylobacter curvus 525.92	E-value = 3.00E-105	Identity = 60.91%
	3		gi|34558158|ref|NP_907973.1|	Wolinella succinogenes DSM 1740	E-value = 7.00E-102	Identity = 59.27%
	4		gi|149194847|ref|ZP_01871941.1|	Caminibacter mediatlanticus TB-2	E-value = 1.00E-91	Identity = 54.29%
	5		gi|32267367|ref|NP_861399.1|	Helicobacter hepaticus ATCC 51449	E-value = 1.00E-90	Identity = 56.17%
AB0358	1	*dctQ*	gi|154175189|ref|YP_001409178.1|	Campylobacter curvus 525.92	E-value = 1.00E-40	Identity = 48.02%
	2		gi|34558159|ref|NP_907974.1|	Wolinella succinogenes DSM 1740	E-value = 2.00E-39	Identity = 54.27%
	3		gi|157164535|ref|YP_001467736.1|	Campylobacter concisus 13826	E-value = 4.00E-35	Identity = 45.03%
	4		gi|32267368|ref|NP_861400.1|	Helicobacter hepaticus ATCC 51449	E-value = 3.00E-26	Identity = 38.80%
	5		gi|149194848|ref|ZP_01871942.1|	Caminibacter mediatlanticus TB-2	E-value = 4.00E-19	Identity = 40.70%
AB0359	1	*dctM*	gi|34558160|ref|NP_907975.1|	Wolinella succinogenes DSM 1740	E-value = 8.00E-135	Identity = 70.42%
	2		gi|4226003|emb|CAA10758.1|	Wolinella succinogenes	E-value = 6.00E-132	Identity = 70.22%
	3		gi|154174636|ref|YP_001409179.1|	Campylobacter curvus 525.92	E-value = 8.00E-128	Identity = 67.84%
	4		gi|157164109|ref|YP_001467735.1|	Campylobacter concisus 13826	E-value = 4.00E-125	Identity = 67.13%

	5		gi|149194849|ref|ZP_01871943.1|	Caminibacter mediatlanticus TB-2	E-value = 2.00E-114	Identity = 64.17%
AB0360	1			***** No matches found *****		
AB0361	1		gi|34557780|ref|NP_907595.1|	Wolinella succinogenes DSM 1740	E-value = 4.00E-28	Identity = 36.41%
	2		gi|152993952|ref|YP_001359673.1|	Sulfurovum sp. NBC37-1	E-value = 9.00E-28	Identity = 35.71%
	3		gi|118475245|ref|YP_891579.1|	Campylobacter fetus subsp. fetus 82-40	E-value = 6.00E-27	Identity = 34.10%
	4		gi|34558806|gb|AAQ75151.1|	Alvinella pompejana epibiont 6C6	E-value = 3.00E-26	Identity = 36.89%
	5		gi|152993408|ref|YP_001359129.1|	Sulfurovum sp. NBC37-1	E-value = 4.00E-26	Identity = 33.48%
AB0362	1	*menG*	gi|119475751|ref|ZP_01616104.1|	marine gamma proteobacterium HTCC2143	E-value = 1.00E-40	Identity = 51.28%
	2		gi|89094648|ref|ZP_01167585.1|	Oceanospirillum sp. MED92	E-value = 2.00E-39	Identity = 50.63%
	3		gi|152992677|ref|YP_001358398.1|	Sulfurovum sp. NBC37-1	E-value = 1.00E-33	Identity = 46.79%
	4		gi|56477973|ref|YP_159562.1|	Azoarcus sp. EbN1	E-value = 3.00E-27	Identity = 39.75%
	5		gi|119897421|ref|YP_932634.1|	Azoarcus sp. BH72	E-value = 1.00E-26	Identity = 41.14%
AB0363	1	*cysG*	gi|78778183|ref|YP_394498.1|	Sulfuromonas denitrificans ATCC 33889	E-value = 3.00E-100	Identity = 71.49%
	2		gi|152991530|ref|YP_001357252.1|	Nitratiruptor sp. SB155-2	E-value = 6.00E-96	Identity = 69.01%
	3		gi|152991888|ref|YP_001357609.1|	Sulfurovum sp. NBC37-1	E-value = 2.00E-94	Identity = 69.83%
	4		gi|76667609|dbj|BAE45634.1|	Hydrogenobacter thermophilus	E-value = 2.00E-68	Identity = 54.04%
	5		gi|15605767|ref|NP_213144.1|	Aquifex aeolicus VF5	E-value = 7.00E-64	Identity = 51.46%
AB0364	1		gi|78777710|ref|YP_394025.1|	Sulfuromonas denitrificans ATCC 33889	E-value = 2.00E-61	Identity = 47.59%
	2		gi|78777495|ref|YP_393810.1|	Sulfuromonas denitrificans ATCC 33889	E-value = 2.00E-12	Identity = 28.84%
AB0365	1		gi|78777709|ref|YP_394024.1|	Sulfuromonas denitrificans ATCC 33889	E-value = 1.00E-56	Identity = 60.93%
	2		gi|78777494|ref|YP_393809.1|	Sulfuromonas denitrificans ATCC 33889	E-value = 1.00E-42	Identity = 47.93%
	3		gi|34557551|ref|NP_907366.1|	Wolinella succinogenes DSM 1740	E-value = 3.00E-36	Identity = 42.45%
	4		gi|118474814|ref|YP_891973.1|	Campylobacter fetus subsp. fetus 82-40	E-value = 3.00E-23	Identity = 34.60%
	5		gi|34557562|ref|NP_907377.1|	Wolinella succinogenes DSM 1740	E-value = 6.00E-21	Identity = 33.50%
AB0366	1		gi|78778174|ref|YP_394489.1|	Sulfuromonas denitrificans ATCC 33889	E-value = 3.00E-75	Identity = 46.23%
	2		gi|152991847|ref|YP_001357568.1|	Sulfurovum sp. NBC37-1	E-value = 1.00E-57	Identity = 40.74%
	3		gi|15605768|ref|NP_213145.1|	Aquifex aeolicus VF5	E-value = 2.00E-20	Identity = 27.61%
	4		gi|52787131|ref|YP_092960.1|	Bacillus licheniformis ATCC 14580	E-value = 2.00E-15	Identity = 28.51%
	5		gi|52081744|ref|YP_080535.1|	Bacillus licheniformis ATCC 14580	E-value = 1.00E-14	Identity = 28.45%
AB0367	1	*moaA*	gi|152991324|ref|YP_001357046.1|	Nitratiruptor sp. SB155-2	E-value = 3.00E-123	Identity = 67.59%
	2		gi|152991807|ref|YP_001357528.1|	Sulfurovum sp. NBC37-1	E-value = 3.00E-118	Identity = 62.69%
	3		gi|157163980|ref|YP_001466045.1|	Campylobacter concisus 13826	E-value = 1.00E-117	Identity = 64.09%
	4		gi|154174652|ref|YP_001407497.1|	Campylobacter curvus 525.92	E-value = 1.00E-117	Identity = 63.16%
	5		gi|78778109|ref|YP_394424.1|	Sulfuromonas denitrificans ATCC 33889	E-value = 4.00E-115	Identity = 61.92%
AB0368	1	*rpsO*	gi|152993457|ref|YP_001359178.1|	Sulfurovum sp. NBC37-1	E-value = 2.00E-20	Identity = 65.91%
	2		gi|152990868|ref|YP_001356590.1|	Nitratiruptor sp. SB155-2	E-value = 3.00E-20	Identity = 65.17%
	3		gi|157165733|ref|YP_001467136.1|	Campylobacter concisus 13826	E-value = 1.00E-19	Identity = 63.33%
	4		gi|154173820|ref|YP_001408024.1|	Campylobacter curvus 525.92	E-value = 1.00E-19	Identity = 65.56%
	5		gi|57240354|ref|ZP_00368303.1|	Campylobacter lari RM2100	E-value = 2.00E-19	Identity = 63.33%
AB0369	1		gi|152990870|ref|YP_001356592.1|	Nitratiruptor sp. SB155-2	E-value = 5.00E-31	Identity = 48.85%
	2		gi|78776924|ref|YP_393239.1|	Sulfuromonas denitrificans ATCC 33889	E-value = 3.00E-30	Identity = 51.15%
	3		gi|152993458|ref|YP_001359179.1|	Sulfurovum sp. NBC37-1	E-value = 6.00E-30	Identity = 50.38%
	4		gi|157165239|ref|YP_001467135.1|	Campylobacter concisus 13826	E-value = 3.00E-27	Identity = 51.15%
	5		gi|15792214|ref|NP_282037.1|	Campylobacter jejuni subsp. jejuni NCTC 11168	E-value = 3.00E-27	Identity = 54.20%
AB0370	1		gi|34557436|ref|NP_907251.1|	Wolinella succinogenes DSM 1740	E-value = 2.00E-77	Identity = 42.36%
	2		gi|118474400|ref|YP_892255.1|	Campylobacter fetus subsp. fetus 82-40	E-value = 2.00E-76	Identity = 49.55%
	3		gi|108562830|ref|YP_627146.1|	Helicobacter pylori HPAG1	E-value = 7.00E-74	Identity = 44.09%
	4		gi|15611450|ref|NP_223101.1|	Helicobacter pylori J99	E-value = 3.00E-73	Identity = 43.80%
	5		gi|57242642|ref|ZP_00370579.1|	Campylobacter upsaliensis RM3195	E-value = 8.00E-73	Identity = 46.29%

AB0371	1		gi|152990554|ref|YP_001356276.1|	Nitratiruptor sp. SB155-2	E-value = 6.00E-51	Identity = 66.20%
	2		gi|152993414|ref|YP_001359135.1|	Sulfurovum sp. NBC37-1	E-value = 7.00E-50	Identity = 69.50%
	3		gi|78777342|ref|YP_393657.1|	Sulfuromonas denitrificans ATCC 33889	E-value = 1.00E-46	Identity = 68.79%
	4		gi|150400634|ref|YP_001324400.1|	Methanococcus aeolicus Nankai-3	E-value = 3.00E-37	Identity = 59.71%
	5		gi|34556580|ref|NP_906395.1|	Wolinella succinogenes DSM 1740	E-value = 2.00E-31	Identity = 49.31%
AB0372	1		gi|152990159|ref|YP_001355881.1|	Nitratiruptor sp. SB155-2	E-value = 4.00E-78	Identity = 41.63%
	2		gi|34556741|ref|NP_906556.1|	Wolinella succinogenes DSM 1740	E-value = 7.00E-74	Identity = 38.76%
	3		gi|154173704|ref|YP_001408853.1|	Campylobacter curvus 525.92	E-value = 6.00E-68	Identity = 40.55%
	4		gi|78776862|ref|YP_393177.1|	Sulfuromonas denitrificans ATCC 33889	E-value = 7.00E-67	Identity = 40.48%
	5		gi|118474097|ref|YP_891447.1|	Campylobacter fetus subsp. fetus 82-40	E-value = 4.00E-64	Identity = 40.69%
AB0373	1		gi|34556742|ref|NP_906557.1|	Wolinella succinogenes DSM 1740	E-value = 6.00E-99	Identity = 84.55%
	2		gi|154173885|ref|YP_001408852.1|	Campylobacter curvus 525.92	E-value = 2.00E-98	Identity = 79.37%
	3		gi|157164291|ref|YP_001466303.1|	Campylobacter concisus 13826	E-value = 2.00E-97	Identity = 79.82%
	4		gi|118475303|ref|YP_891446.1|	Campylobacter fetus subsp. fetus 82-40	E-value = 3.00E-97	Identity = 79.37%
	5		gi|154148743|ref|YP_001406322.1|	Campylobacter hominis ATCC BAA-381	E-value = 7.00E-95	Identity = 80.72%
AB0374	1	*ppiC*	gi|34557645|ref|NP_907460.1|	Wolinella succinogenes DSM 1740	E-value = 2.00E-43	Identity = 41.70%
	2		gi|152990310|ref|YP_001356032.1|	Nitratiruptor sp. SB155-2	E-value = 3.00E-39	Identity = 40.30%
	3		gi|154148224|ref|YP_001407264.1|	Campylobacter hominis ATCC BAA-381	E-value = 3.00E-38	Identity = 40.66%
	4		gi|152993388|ref|YP_001359109.1|	Sulfurovum sp. NBC37-1	E-value = 6.00E-37	Identity = 41.70%
	5		gi|118474611|ref|YP_891704.1|	Campylobacter fetus subsp. fetus 82-40	E-value = 2.00E-36	Identity = 41.13%
AB0375	1	*fba*	gi|78776709|ref|YP_393024.1|	Sulfuromonas denitrificans ATCC 33889	E-value = 6.00E-150	Identity = 72.55%
	2		gi|118474472|ref|YP_891705.1|	Campylobacter fetus subsp. fetus 82-40	E-value = 3.00E-148	Identity = 72.19%
	3		gi|117619723|ref|YP_855324.1|	Aeromonas hydrophila subsp. hydrophila ATCC 7966	E-value = 5.00E-148	Identity = 70.82%
	4		gi|145300379|ref|YP_001143220.1|	Aeromonas salmonicida subsp. salmonicida A449	E-value = 3.00E-147	Identity = 70.54%
	5		gi|157165707|ref|YP_001467104.1|	Campylobacter concisus 13826	E-value = 1.00E-146	Identity = 71.35%
AB0376	1	*ald*	gi|149371938|ref|ZP_01891257.1|	unidentified eubacterium SCB49	E-value = 0	Identity = 68.48%
	2		gi|68054027|ref|ZP_00538192.1|	Exiguobacterium sibiricum 255-15	E-value = 0	Identity = 67.59%
	3		gi|124009805|ref|ZP_01694474.1|	Microscilla marina ATCC 23134	E-value = 0	Identity = 67%
	4		gi|21229579|ref|NP_635496.1|	Xanthomonas campestris pv. campestris str. ATCC 33913	E-value = 0	Identity = 66.73%
	5		gi|86140526|ref|ZP_01059085.1|	Flavobacterium sp. MED217	E-value = 0	Identity = 66.47%
AB0377	1		gi|108758321|ref|YP_633194.1|	Myxococcus xanthus DK 1622	E-value = 8.00E-31	Identity = 51.26%
	2		gi|77166366|ref|YP_344891.1|	Nitrosococcus oceani ATCC 19707	E-value = 3.00E-30	Identity = 50.81%
	3		gi|114327687|ref|YP_744844.1|	Granulibacter bethesdensis CGDNIH1	E-value = 1.00E-28	Identity = 45.04%
	4		gi|149371937|ref|ZP_01891256.1|	unidentified eubacterium SCB49	E-value = 7.00E-28	Identity = 44.88%
	5		gi|86140527|ref|ZP_01059086.1|	Flavobacterium sp. MED217	E-value = 5.00E-27	Identity = 46.55%
AB0378	1	*exbD1*	gi|77166132|ref|YP_344657.1|	Nitrosococcus oceani ATCC 19707	E-value = 5.00E-11	Identity = 34.92%
	2		gi|87308193|ref|ZP_01090335.1|	Blastopirellula marina DSM 3645	E-value = 4.00E-09	Identity = 34.59%
	3		gi|126463340|ref|YP_001044454.1|	Rhodobacter sphaeroides ATCC 17029	E-value = 1.00E-08	Identity = 32.20%
	4		gi|146279027|ref|YP_001169186.1|	Rhodobacter sphaeroides ATCC 17025	E-value = 2.00E-08	Identity = 32.20%
	5		gi|119478440|ref|ZP_01618425.1|	marine gamma proteobacterium HTCC2143	E-value = 5.00E-08	Identity = 31.50%
AB0379	1	*exbB1*	gi|153877900|ref|ZP_02004410.1|	Beggiatoa sp. PS	E-value = 6.00E-14	Identity = 32.64%
	2		gi|126667251|ref|ZP_01738224.1|	Marinobacter sp. ELB17	E-value = 9.00E-13	Identity = 31.05%
	3		gi|83645477|ref|YP_433912.1|	Hahella chejuensis KCTC 2396	E-value = 1.00E-11	Identity = 27.13%
	4		gi|150003064|ref|YP_001297808.1|	Bacteroides vulgatus ATCC 8482	E-value = 6.00E-11	Identity = 29.57%
	5		gi|77164132|ref|YP_342657.1|	Nitrosococcus oceani ATCC 19707	E-value = 2.00E-10	Identity = 30.83%
AB0380	1		gi|152993384|ref|YP_001359105.1|	Sulfurovum sp. NBC37-1	E-value = 9.00E-77	Identity = 54.64%
	2		gi|149910540|ref|ZP_01899179.1|	Moritella sp. PE36	E-value = 2.00E-75	Identity = 46.23%
	3		gi|152990314|ref|YP_001356036.1|	Nitratiruptor sp. SB155-2	E-value = 7.00E-71	Identity = 50.63%
	4		gi|127513075|ref|YP_001094272.1|	Shewanella loihica PV-4	E-value = 2.00E-69	Identity = 44.01%

	5		gi|153833374|ref|ZP_01986041.1|	Vibrio harveyi HY01	E-value = 7.00E-69	Identity = 44.85%
AB0381	1	*aroK*	gi|82523991|emb|CAI78802.1|	uncultured epsilon proteobacterium	E-value = 1.00E-40	Identity = 58.86%
	2		gi|154148531|ref|YP_001405673.1|	Campylobacter hominis ATCC BAA-381	E-value = 3.00E-38	Identity = 59.09%
	3		gi|78776695|ref|YP_393010.1|	Sulfuromonas denitrificans ATCC 33889	E-value = 1.00E-37	Identity = 60.13%
	4		gi|152990426|ref|YP_001356148.1|	Nitratiruptor sp. SB155-2	E-value = 1.00E-36	Identity = 61.39%
	5		gi|154173675|ref|YP_001407733.1|	Campylobacter curvus 525.92	E-value = 4.00E-34	Identity = 56.13%
AB0382	1	*hisD*	gi|152990315|ref|YP_001356037.1|	Nitratiruptor sp. SB155-2	E-value = 1.00E-176	Identity = 73.72%
	2		gi|152993383|ref|YP_001359104.1|	Sulfurovum sp. NBC37-1	E-value = 5.00E-176	Identity = 72.90%
	3		gi|78776697|ref|YP_393012.1|	Sulfuromonas denitrificans ATCC 33889	E-value = 8.00E-171	Identity = 71.16%
	4		gi|149193768|ref|ZP_01870866.1|	Caminibacter mediatlanticus TB-2	E-value = 3.00E-168	Identity = 70.63%
	5		gi|34557911|ref|NP_907726.1|	Wolinella succinogenes DSM 1740	E-value = 7.00E-163	Identity = 67.29%
AB0383	1		gi|78777054|ref|YP_393369.1|	Sulfuromonas denitrificans ATCC 33889	E-value = 5.00E-11	Identity = 46.24%
AB0384	1	*ispB*	gi|154148638|ref|YP_001406457.1|	Campylobacter hominis ATCC BAA-381	E-value = 4.00E-75	Identity = 54.03%
	2		gi|57167586|ref|ZP_00366726.1|	Campylobacter coli RM2228	E-value = 9.00E-74	Identity = 57.61%
	3		gi|153951500|ref|YP_001398431.1|	Campylobacter jejuni subsp. doylei 269.97	E-value = 4.00E-73	Identity = 54.70%
	4		gi|88596918|ref|ZP_01100154.1|	Campylobacter jejuni subsp. jejuni 84-25	E-value = 5.00E-73	Identity = 55.03%
	5		gi|15791902|ref|NP_281725.1|	Campylobacter jejuni subsp. jejuni NCTC 11168	E-value = 9.00E-73	Identity = 54.70%
AB0385	1	*hemA*	gi|34557906|ref|NP_907721.1|	Wolinella succinogenes DSM 1740	E-value = 9.00E-108	Identity = 46.64%
	2		gi|152990769|ref|YP_001356491.1|	Nitratiruptor sp. SB155-2	E-value = 2.00E-102	Identity = 45.09%
	3		gi|78777057|ref|YP_393372.1|	Sulfuromonas denitrificans ATCC 33889	E-value = 3.00E-95	Identity = 45.09%
	4		gi|121612724|ref|YP_001000242.1|	Campylobacter jejuni subsp. jejuni 81-176	E-value = 2.00E-94	Identity = 44.55%
	5		gi|15791903|ref|NP_281726.1|	Campylobacter jejuni subsp. jejuni NCTC 11168	E-value = 2.00E-94	Identity = 44.55%
AB0386	1	*proS*	gi|152992232|ref|YP_001357953.1|	Sulfurovum sp. NBC37-1	E-value = 0	Identity = 60.53%
	2		gi|154148423|ref|YP_001406459.1|	Campylobacter hominis ATCC BAA-381	E-value = 0	Identity = 61.42%
	3		gi|154175003|ref|YP_001408388.1|	Campylobacter curvus 525.92	E-value = 0	Identity = 60.6%
	4		gi|57167588|ref|ZP_00366728.1|	Campylobacter coli RM2228	E-value = 0	Identity = 62.21%
	5		gi|157165397|ref|YP_001466693.1|	Campylobacter concisus 13826	E-value = 0	Identity = 60.42%
AB0387	1	*mscS*	gi|89094052|ref|ZP_01166996.1|	Oceanospirillum sp. MED92	E-value = 4.00E-63	Identity = 48.46%
	2		gi|152997779|ref|YP_001342614.1|	Marinomonas sp. MWYL1	E-value = 3.00E-60	Identity = 45.74%
	3		gi|109899638|ref|YP_662893.1|	Pseudoalteromonas atlantica T6c	E-value = 9.00E-60	Identity = 49.19%
	4		gi|95930648|ref|ZP_01313382.1|	Desulfuromonas acetoxidans DSM 684	E-value = 3.00E-59	Identity = 43.73%
	5		gi|90415525|ref|ZP_01223459.1|	marine gamma proteobacterium HTCC2207	E-value = 5.00E-59	Identity = 46.80%
AB0388	1		gi|152992555|ref|YP_001358276.1|	Sulfurovum sp. NBC37-1	E-value = 8.00E-61	Identity = 38.01%
	2		gi|78776206|ref|YP_392521.1|	Sulfuromonas denitrificans ATCC 33889	E-value = 6.00E-45	Identity = 37.58%
	3		gi|118602242|ref|YP_903457.1|	Candidatus Ruthia magnifica str. Cm (Calyptogena magnifica)	E-value = 2.00E-19	Identity = 28.75%
	4		gi|57237634|ref|YP_178882.1|	Campylobacter jejuni subsp. jejuni RM1221	E-value = 3.00E-16	Identity = 31.56%
	5		gi|157415060|ref|YP_001482316.1|	Campylobacter jejuni subsp. jejuni 81116	E-value = 6.00E-16	Identity = 31.56%
AB0389	1		gi|78778191|ref|YP_394506.1|	Sulfuromonas denitrificans ATCC 33889	E-value = 1.00E-23	Identity = 49.65%
	2		gi|110601691|ref|ZP_01389866.1|	Geobacter sp. FRC-32	E-value = 7.00E-23	Identity = 52.85%
	3		gi|145620818|ref|ZP_01776834.1|	Geobacter bemidjiensis Bem	E-value = 3.00E-21	Identity = 50.40%
	4		gi|78224117|ref|YP_385864.1|	Geobacter metallireducens GS-15	E-value = 7.00E-21	Identity = 48.03%
	5		gi|32265838|ref|NP_859870.1|	Helicobacter hepaticus ATCC 51449	E-value = 9.00E-19	Identity = 43.45%
AB0390	1		gi|78778192|ref|YP_394507.1|	Sulfuromonas denitrificans ATCC 33889	E-value = 2.00E-87	Identity = 35.97%
	2		gi|78224116|ref|YP_385863.1|	Geobacter metallireducens GS-15	E-value = 6.00E-86	Identity = 33.85%
	3		gi|145620817|ref|ZP_01776833.1|	Geobacter bemidjiensis Bem	E-value = 2.00E-76	Identity = 35.67%
	4		gi|120556249|ref|YP_960600.1|	Marinobacter aquaeolei VT8	E-value = 2.00E-70	Identity = 31.50%
	5		gi|149375748|ref|ZP_01893516.1|	Marinobacter algicola DG893	E-value = 5.00E-70	Identity = 34.89%
AB0391	1	*lnt*	gi|78777774|ref|YP_394089.1|	Sulfuromonas denitrificans ATCC 33889	E-value = 1.00E-81	Identity = 43.78%
	2		gi|154175027|ref|YP_001408528.1|	Campylobacter curvus 525.92	E-value = 4.00E-71	Identity = 39.41%

	3		gi|152991988|ref|YP_001357709.1|	Sulfurovum sp. NBC37-1	E-value = 3.00E-70	Identity = 37.53%
	4		gi|34556534|ref|NP_906349.1|	Wolinella succinogenes DSM 1740	E-value = 4.00E-70	Identity = 41.16%
	5		gi|118474873|ref|YP_891901.1|	Campylobacter fetus subsp. fetus 82-40	E-value = 2.00E-67	Identity = 38.37%
AB0392	1	*yajC*	gi|34557614|ref|NP_907429.1|	Wolinella succinogenes DSM 1740	E-value = 2.00E-20	Identity = 63.53%
	2		gi|153952484|ref|YP_001397793.1|	Campylobacter jejuni subsp. doylei 269.97	E-value = 3.00E-20	Identity = 61.63%
	3		gi|157164205|ref|YP_001467051.1|	Campylobacter concisus 13826	E-value = 5.00E-20	Identity = 63.53%
	4		gi|118474680|ref|YP_891902.1|	Campylobacter fetus subsp. fetus 82-40	E-value = 6.00E-20	Identity = 61.63%
	5		gi|15792419|ref|NP_282242.1|	Campylobacter jejuni subsp. jejuni NCTC 11168	E-value = 9.00E-20	Identity = 61.63%
AB0393	1	*secD*	gi|152991208|ref|YP_001356930.1|	Nitratiruptor sp. SB155-2	E-value = 3.00E-173	Identity = 64.30%
	2		gi|78777772|ref|YP_394087.1|	Sulfuromonas denitrificans ATCC 33889	E-value = 2.00E-170	Identity = 64.48%
	3		gi|157164627|ref|YP_001467050.1|	Campylobacter concisus 13826	E-value = 3.00E-169	Identity = 62.99%
	4		gi|152991990|ref|YP_001357711.1|	Sulfurovum sp. NBC37-1	E-value = 5.00E-168	Identity = 59.08%
	5		gi|118475690|ref|YP_891903.1|	Campylobacter fetus subsp. fetus 82-40	E-value = 2.00E-167	Identity = 61.90%
AB0394	1	*secF*	gi|57167975|ref|ZP_00367114.1|	Campylobacter coli RM2228	E-value = 3.00E-87	Identity = 54.49%
	2		gi|57237974|ref|YP_179223.1|	Campylobacter jejuni subsp. jejuni RM1221	E-value = 2.00E-86	Identity = 54.49%
	3		gi|15792417|ref|NP_282240.1|	Campylobacter jejuni subsp. jejuni NCTC 11168	E-value = 2.00E-86	Identity = 54.18%
	4		gi|153951817|ref|YP_001397795.1|	Campylobacter jejuni subsp. doylei 269.97	E-value = 6.00E-86	Identity = 53.87%
	5		gi|154174926|ref|YP_001408525.1|	Campylobacter curvus 525.92	E-value = 9.00E-86	Identity = 52.01%
AB0395	1			***** No matches found *****		
AB0396	1			***** No matches found *****		
AB0397	1	*motB*	gi|15611819|ref|NP_223470.1|	Helicobacter pylori J99	E-value = 4.00E-33	Identity = 35.90%
	2		gi|15645435|ref|NP_207609.1|	Helicobacter pylori 26695	E-value = 5.00E-33	Identity = 35.90%
	3		gi|108563226|ref|YP_627542.1|	Helicobacter pylori HPAG1	E-value = 2.00E-32	Identity = 35.04%
	4		gi|109947291|ref|YP_664519.1|	Helicobacter acinonychis str. Sheeba	E-value = 4.00E-32	Identity = 34.62%
	5		gi|32266001|ref|NP_860033.1|	Helicobacter hepaticus ATCC 51449	E-value = 5.00E-32	Identity = 38.79%
AB0398	1	*motA*	gi|57168475|ref|ZP_00367609.1|	Campylobacter coli RM2228	E-value = 8.00E-69	Identity = 54.77%
	2		gi|153952523|ref|YP_001398612.1|	Campylobacter jejuni subsp. doylei 269.97	E-value = 2.00E-68	Identity = 55.19%
	3		gi|86151159|ref|ZP_01069374.1|	Campylobacter jejuni subsp. jejuni 260.94	E-value = 2.00E-68	Identity = 55.19%
	4		gi|57505392|ref|ZP_00371320.1|	Campylobacter upsaliensis RM3195	E-value = 2.00E-68	Identity = 54.77%
	5		gi|121612656|ref|YP_001000047.1|	Campylobacter jejuni subsp. jejuni 81-176	E-value = 3.00E-68	Identity = 54.77%
AB0399	1		gi|78777770|ref|YP_394085.1|	Sulfuromonas denitrificans ATCC 33889	E-value = 8.00E-28	Identity = 65.77%
	2		gi|157164293|ref|YP_001467048.1|	Campylobacter concisus 13826	E-value = 1.00E-26	Identity = 69.37%
	3		gi|149194836|ref|ZP_01871930.1|	Caminibacter mediatlanticus TB-2	E-value = 9.00E-26	Identity = 63.64%
	4		gi|152991992|ref|YP_001357713.1|	Sulfurovum sp. NBC37-1	E-value = 2.00E-25	Identity = 63.16%
	5		gi|154173752|ref|YP_001408524.1|	Campylobacter curvus 525.92	E-value = 4.00E-25	Identity = 65.77%
AB0400	1	*leuS*	gi|152993621|ref|YP_001359342.1|	Sulfurovum sp. NBC37-1	E-value = 0	Identity = 67.68%
	2		gi|152991206|ref|YP_001356928.1|	Nitratiruptor sp. SB155-2	E-value = 0	Identity = 66.87%
	3		gi|154174333|ref|YP_001408523.1|	Campylobacter curvus 525.92	E-value = 0	Identity = 63.29%
	4		gi|78777769|ref|YP_394084.1|	Sulfuromonas denitrificans ATCC 33889	E-value = 0	Identity = 64.69%
	5		gi|34557610|ref|NP_907425.1|	Wolinella succinogenes DSM 1740	E-value = 0	Identity = 63.3%
AB0401	1		gi|152991205|ref|YP_001356927.1|	Nitratiruptor sp. SB155-2	E-value = 4.00E-23	Identity = 37.82%
	2		gi|34557609|ref|NP_907424.1|	Wolinella succinogenes DSM 1740	E-value = 1.00E-22	Identity = 36.25%
	3		gi|57240454|ref|ZP_00368403.1|	Campylobacter lari RM2100	E-value = 2.00E-17	Identity = 32.94%
	4		gi|109946908|ref|YP_664136.1|	Helicobacter acinonychis str. Sheeba	E-value = 2.00E-17	Identity = 34.44%
	5		gi|57167973|ref|ZP_00367112.1|	Campylobacter coli RM2228	E-value = 1.00E-16	Identity = 34.59%
AB0402	1	*folC*	gi|149194840|ref|ZP_01871934.1|	Caminibacter mediatlanticus TB-2	E-value = 2.00E-80	Identity = 49.20%
	2		gi|78777766|ref|YP_394081.1|	Sulfuromonas denitrificans ATCC 33889	E-value = 2.00E-80	Identity = 44.36%
	3		gi|152993619|ref|YP_001359340.1|	Sulfurovum sp. NBC37-1	E-value = 5.00E-78	Identity = 44.50%
	4		gi|152991204|ref|YP_001356926.1|	Nitratiruptor sp. SB155-2	E-value = 2.00E-70	Identity = 40.05%

	5		gi|154175231|ref|YP_001408520.1|	Campylobacter curvus 525.92	E-value = 7.00E-70	Identity = 40.67%
AB0403	1		gi|152992179|ref|YP_001357900.1|	Sulfurovum sp. NBC37-1	E-value = 6.00E-153	Identity = 41.10%
	2		gi|118579744|ref|YP_900994.1|	Pelobacter propionicus DSM 2379	E-value = 7.00E-88	Identity = 30.15%
	3		gi|67939717|ref|ZP_00532208.1|	Chlorobium phaeobacteroides BS1	E-value = 3.00E-87	Identity = 31.27%
	4		gi|124522906|ref|ZP_01697290.1|	Bacillus coagulans 36D1	E-value = 9.00E-86	Identity = 31.26%
	5		gi|150004073|ref|YP_001298817.1|	Bacteroides vulgatus ATCC 8482	E-value = 4.00E-83	Identity = 29.82%
AB0404	1		gi|89092523|ref|ZP_01165476.1|	Oceanospirillum sp. MED92	E-value = 4.00E-164	Identity = 49.56%
	2		gi|114046627|ref|YP_737177.1|	Shewanella sp. MR-7	E-value = 1.00E-157	Identity = 47.06%
	3		gi|113969400|ref|YP_733193.1|	Shewanella sp. MR-4	E-value = 3.00E-157	Identity = 46.88%
	4		gi|117919509|ref|YP_868701.1|	Shewanella sp. ANA-3	E-value = 2.00E-156	Identity = 46.88%
	5		gi|87121771|ref|ZP_01077658.1|	Marinomonas sp. MED121	E-value = 3.00E-156	Identity = 47.62%
AB0405	1		gi|150425401|gb|EDN17177.1|	Vibrio cholerae AM-19226	E-value = 2.00E-60	Identity = 53.04%
	2		gi|116190895|ref|ZP_01480630.1|	Vibrio cholerae MO10	E-value = 4.00E-60	Identity = 52.61%
	3		gi|153212169|ref|ZP_01947964.1|	Vibrio cholerae 1587	E-value = 5.00E-60	Identity = 53.04%
	4		gi|15601750|ref|NP_233381.1|	Vibrio cholerae O1 biovar eltor str. N16961	E-value = 9.00E-60	Identity = 52.61%
	5		gi|116221832|ref|ZP_01487234.1|	Vibrio cholerae V51	E-value = 9.00E-60	Identity = 52.17%
AB0406	1			***** No matches found *****		
AB0407	1			***** No matches found *****		
AB0408	1	*mfd*	gi|34557604|ref|NP_907419.1|	Wolinella succinogenes DSM 1740	E-value = 0	Identity = 53.48%
	2		gi|152991203|ref|YP_001356925.1|	Nitratiruptor sp. SB155-2	E-value = 0	Identity = 54.41%
	3		gi|118474341|ref|YP_891912.1|	Campylobacter fetus subsp. fetus 82-40	E-value = 0	Identity = 52.26%
	4		gi|154175141|ref|YP_001408519.1|	Campylobacter curvus 525.92	E-value = 0	Identity = 50.91%
	5		gi|32265957|ref|NP_859989.1|	Helicobacter hepaticus ATCC 51449	E-value = 0	Identity = 50.61%
AB0409	1		gi|152965280|ref|YP_001361064.1|	Kineococcus radiotolerans SRS30216	E-value = 2.00E-23	Identity = 32.35%
	2		gi|84622941|ref|YP_450313.1|	Xanthomonas oryzae pv. oryzae MAFF 311018	E-value = 4.00E-21	Identity = 32.54%
	3		gi|146300323|ref|YP_001194914.1|	Flavobacterium johnsoniae UW101	E-value = 1.00E-20	Identity = 38.89%
	4		gi|21243666|ref|NP_643248.1|	Xanthomonas axonopodis pv. citri str. 306	E-value = 6.00E-20	Identity = 30.99%
	5		gi|78048639|ref|YP_364814.1|	Xanthomonas campestris pv. vesicatoria str. 85-10	E-value = 9.00E-20	Identity = 31.36%
AB0410	1		gi|91792122|ref|YP_561773.1|	Shewanella denitrificans OS217	E-value = 3.00E-34	Identity = 27.85%
	2		gi|127511624|ref|YP_001092821.1|	Shewanella loihica PV-4	E-value = 4.00E-34	Identity = 29.30%
	3		gi|118071530|ref|ZP_01539725.1|	Shewanella woodyi ATCC 51908	E-value = 1.00E-33	Identity = 29.77%
	4		gi|89072881|ref|ZP_01159438.1|	Photobacterium sp. SKA34	E-value = 4.00E-33	Identity = 31.89%
	5		gi|113969313|ref|YP_733106.1|	Shewanella sp. MR-4	E-value = 8.00E-33	Identity = 29.81%
AB0411	1			***** No matches found *****		
AB0412	1	*ompR*	gi|157164458|ref|YP_001466682.1|	Campylobacter concisus 13826	E-value = 7.00E-60	Identity = 55.56%
	2		gi|118474546|ref|YP_891938.1|	Campylobacter fetus subsp. fetus 82-40	E-value = 8.00E-59	Identity = 56.19%
	3		gi|57241251|ref|ZP_00369198.1|	Campylobacter lari RM2100	E-value = 2.00E-58	Identity = 55.31%
	4		gi|152991455|ref|YP_001357177.1|	Nitratiruptor sp. SB155-2	E-value = 3.00E-58	Identity = 55.36%
	5		gi|154174385|ref|YP_001408399.1|	Campylobacter curvus 525.92	E-value = 6.00E-58	Identity = 55.31%
AB0413	1		gi|32267106|ref|NP_861138.1|	Helicobacter hepaticus ATCC 51449	E-value = 9.00E-48	Identity = 33.01%
	2		gi|157163896|ref|YP_001466683.1|	Campylobacter concisus 13826	E-value = 2.00E-47	Identity = 34.87%
	3		gi|118474309|ref|YP_891937.1|	Campylobacter fetus subsp. fetus 82-40	E-value = 7.00E-46	Identity = 35.52%
	4		gi|154174937|ref|YP_001408398.1|	Campylobacter curvus 525.92	E-value = 1.00E-45	Identity = 34.89%
	5		gi|34558118|ref|NP_907933.1|	Wolinella succinogenes DSM 1740	E-value = 3.00E-42	Identity = 31.65%
AB0414	1		gi|78777930|ref|YP_394245.1|	Sulfuromonas denitrificans ATCC 33889	E-value = 4.00E-83	Identity = 46.47%
	2		gi|149194213|ref|ZP_01871311.1|	Caminibacter mediatlanticus TB-2	E-value = 2.00E-79	Identity = 50.28%
	3		gi|152992422|ref|YP_001358143.1|	Sulfurovum sp. NBC37-1	E-value = 1.00E-78	Identity = 46.59%
	4		gi|152990840|ref|YP_001356562.1|	Nitratiruptor sp. SB155-2	E-value = 5.00E-73	Identity = 42.59%
	5		gi|34557444|ref|NP_907259.1|	Wolinella succinogenes DSM 1740	E-value = 9.00E-71	Identity = 42.70%

AB0415	1		gi|78776484|ref|YP_392799.1|	Sulfuromonas denitrificans ATCC 33889	E-value = 2.00E-80	Identity = 43.55%
	2		gi|32266390|ref|NP_860422.1|	Helicobacter hepaticus ATCC 51449	E-value = 4.00E-49	Identity = 32.88%
	3		gi|108563004|ref|YP_627320.1|	Helicobacter pylori HPAG1	E-value = 1.00E-46	Identity = 32.35%
	4		gi|15645224|ref|NP_207394.1|	Helicobacter pylori 26695	E-value = 2.00E-46	Identity = 32.35%
	5		gi|1840146|gb|AAB47275.1|	Helicobacter pylori	E-value = 1.00E-45	Identity = 32.12%
AB0416	1		gi|78776483|ref|YP_392798.1|	Sulfuromonas denitrificans ATCC 33889	E-value = 1.00E-24	Identity = 57.50%
	2		gi|111075014|gb|ABH04866.1|	Heliobacillus mobilis	E-value = 2.00E-13	Identity = 36.44%
	3		gi|7767177|pdb|1DZ3|A	Geobacillus stearothermophilus	E-value = 4.00E-11	Identity = 40.00%
	4		gi|6730308|pdb|1DC7|A	Salmonella typhimurium	E-value = 7.00E-11	Identity = 34.95%
	5		gi|95930680|ref|ZP_01313414.1|	Desulfuromonas acetoxidans DSM 684	E-value = 7.00E-11	Identity = 37.40%
AB0417	1			***** No matches found *****		
AB0418	1			***** No matches found *****		
AB0419	1		gi|152991201|ref|YP_001356923.1|	Nitratiruptor sp. SB155-2	E-value = 3.00E-88	Identity = 57.68%
	2		gi|78777758|ref|YP_394073.1|	Sulfuromonas denitrificans ATCC 33889	E-value = 8.00E-81	Identity = 53.90%
	3		gi|34557603|ref|NP_907418.1|	Wolinella succinogenes DSM 1740	E-value = 2.00E-75	Identity = 50.98%
	4		gi|152993624|ref|YP_001359345.1|	Sulfurovum sp. NBC37-1	E-value = 1.00E-73	Identity = 52.61%
	5		gi|149194440|ref|ZP_01871537.1|	Caminibacter mediatlanticus TB-2	E-value = 2.00E-64	Identity = 51.57%
AB0420	1		gi|152990347|ref|YP_001356069.1|	Nitratiruptor sp. SB155-2	E-value = 5.00E-27	Identity = 36.23%
AB0421	1			***** No matches found *****		
AB0422	1	*ubiE*	gi|152990174|ref|YP_001355896.1|	Nitratiruptor sp. SB155-2	E-value = 2.00E-74	Identity = 55.32%
	2		gi|78776879|ref|YP_393194.1|	Sulfuromonas denitrificans ATCC 33889	E-value = 1.00E-73	Identity = 58.97%
	3		gi|34557586|ref|NP_907401.1|	Wolinella succinogenes DSM 1740	E-value = 4.00E-73	Identity = 58.30%
	4		gi|152993546|ref|YP_001359267.1|	Sulfurovum sp. NBC37-1	E-value = 6.00E-72	Identity = 56.41%
	5		gi|57237375|ref|YP_178388.1|	Campylobacter jejuni subsp. jejuni RM1221	E-value = 2.00E-68	Identity = 56.78%
AB0423	1	*xseA*	gi|34556985|ref|NP_906800.1|	Wolinella succinogenes DSM 1740	E-value = 4.00E-102	Identity = 53.57%
	2		gi|78776880|ref|YP_393195.1|	Sulfuromonas denitrificans ATCC 33889	E-value = 2.00E-98	Identity = 47.52%
	3		gi|152990176|ref|YP_001355898.1|	Nitratiruptor sp. SB155-2	E-value = 6.00E-97	Identity = 47.76%
	4		gi|32267231|ref|NP_861263.1|	Helicobacter hepaticus ATCC 51449	E-value = 2.00E-95	Identity = 47.63%
	5		gi|149194320|ref|ZP_01871417.1|	Caminibacter mediatlanticus TB-2	E-value = 2.00E-90	Identity = 47.91%
AB0425	1	*cheW*	gi|78222297|ref|YP_384044.1|	Geobacter metallireducens GS-15	E-value = 6.00E-23	Identity = 44.06%
	2		gi|145619181|ref|ZP_01775233.1|	Geobacter bemidjiensis Bem	E-value = 2.00E-22	Identity = 43.36%
	3		gi|30249817|ref|NP_841887.1|	Nitrosomonas europaea ATCC 19718	E-value = 3.00E-22	Identity = 43.36%
	4		gi|118040205|ref|ZP_01511601.1|	Burkholderia phytofirmans PsJN	E-value = 9.00E-22	Identity = 38.71%
	5		gi|114331166|ref|YP_747388.1|	Nitrosomonas eutropha C91	E-value = 1.00E-21	Identity = 43.36%
AB0426	1		gi|46446767|ref|YP_008132.1|	Candidatus Protochlamydia amoebophila UWE25	E-value = 2.00E-54	Identity = 65.82%
	2		gi|78776704|ref|YP_393019.1|	Sulfuromonas denitrificans ATCC 33889	E-value = 3.00E-49	Identity = 58.86%
	3		gi|71042629|pdb|2A2N|A	Homo sapiens	E-value = 2.00E-45	Identity = 58.71%
	4		gi|32352196|dbj|BAC78591.1|	Oryza sativa (japonica cultivar-group)	E-value = 2.00E-45	Identity = 58.00%
	5		gi|115477779|ref|NP_001062485.1|	Oryza sativa (japonica cultivar-group)	E-value = 2.00E-45	Identity = 58.00%
AB0427	1	*panD*	gi|152990004|ref|YP_001355726.1|	Nitratiruptor sp. SB155-2	E-value = 4.00E-42	Identity = 76.07%
	2		gi|34557994|ref|NP_907809.1|	Wolinella succinogenes DSM 1740	E-value = 2.00E-41	Identity = 70.25%
	3		gi|149195160|ref|ZP_01872251.1|	Caminibacter mediatlanticus TB-2	E-value = 3.00E-40	Identity = 72.58%
	4		gi|78776535|ref|YP_392850.1|	Sulfuromonas denitrificans ATCC 33889	E-value = 3.00E-40	Identity = 71.90%
	5		gi|152993855|ref|YP_001359576.1|	Sulfurovum sp. NBC37-1	E-value = 9.00E-37	Identity = 65.85%
AB0428	1		gi|12644595|sp|O34247|Y1681_WOLSU	Wolinella succinogenes	E-value = 2.00E-17	Identity = 53.26%
	2		gi|34557993|ref|NP_907808.1|	Wolinella succinogenes DSM 1740	E-value = 3.00E-17	Identity = 53.26%
	3		gi|152993854|ref|YP_001359575.1|	Sulfurovum sp. NBC37-1	E-value = 2.00E-16	Identity = 54.81%
	4		gi|118474266|ref|YP_891276.1|	Campylobacter fetus subsp. fetus 82-40	E-value = 4.00E-16	Identity = 48.08%
	5		gi|154175278|ref|YP_001409123.1|	Campylobacter curvus 525.92	E-value = 1.00E-15	Identity = 46.23%

AB0429	1	*ispA*	gi|152990006|ref|YP_001355728.1|	Nitratiruptor sp. SB155-2	E-value = 1.00E-89	Identity = 60.42%
	2		gi|152991761|ref|YP_001357482.1|	Sulfurovum sp. NBC37-1	E-value = 5.00E-87	Identity = 58.72%
	3		gi|57168796|ref|ZP_00367927.1|	Campylobacter coli RM2228	E-value = 8.00E-82	Identity = 58.87%
	4		gi|157415864|ref|YP_001483120.1|	Campylobacter jejuni subsp. jejuni 81116	E-value = 5.00E-81	Identity = 58.91%
	5		gi|86152300|ref|ZP_01070511.1|	Campylobacter jejuni subsp. jejuni 260.94	E-value = 7.00E-81	Identity = 59.27%
AB0430	1	*groES*	gi|109948170|ref|YP_665398.1|	Helicobacter acinonychis str. Sheeba	E-value = 8.00E-20	Identity = 53.93%
	2		gi|152993542|ref|YP_001359263.1|	Sulfurovum sp. NBC37-1	E-value = 8.00E-20	Identity = 56.47%
	3		gi|19338966|gb|AAL86899.1|AF479029_1	Helicobacter pylori	E-value = 1.00E-19	Identity = 55.06%
	4		gi|51035697|emb|CAH17456.1|	Helicobacter pylori	E-value = 2.00E-19	Identity = 52.81%
	5		gi|51035687|emb|CAH17451.1|	Helicobacter pylori	E-value = 2.00E-19	Identity = 52.81%
AB0431	1	*groEL*	gi|25452865|sp|Q93GW2|CH60_WOLRE	Campylobacter rectus	E-value = 0	Identity = 77.94%
	2		gi|118474493|ref|YP_891876.1|	Campylobacter fetus subsp. fetus 82-40	E-value = 0	Identity = 77.84%
	3		gi|157165097|ref|YP_001466610.1|	Campylobacter concisus 13826	E-value = 0	Identity = 77.9%
	4		gi|154173786|ref|YP_001408485.1|	Campylobacter curvus 525.92	E-value = 0	Identity = 76.97%
	5		gi|57240444|ref|ZP_00368393.1|	Campylobacter lari RM2100	E-value = 0	Identity = 77.82%
AB0432	1		gi|78776517|ref|YP_392832.1|	Sulfuromonas denitrificans ATCC 33889	E-value = 5.00E-53	Identity = 35.96%
	2		gi|148266304|ref|YP_001233010.1|	Geobacter uraniumreducens Rf4	E-value = 3.00E-43	Identity = 28.70%
	3		gi|39995560|ref|NP_951511.1|	Geobacter sulfurreducens PCA	E-value = 1.00E-42	Identity = 27.25%
	4		gi|145621868|ref|ZP_01777834.1|	Petrotoga mobilis SJ95	E-value = 1.00E-42	Identity = 31.69%
	5		gi|116749437|ref|YP_846124.1|	Syntrophobacter fumaroxidans MPOB	E-value = 1.00E-40	Identity = 26.16%
AB0433	1		gi|78776518|ref|YP_392833.1|	Sulfuromonas denitrificans ATCC 33889	E-value = 4.00E-62	Identity = 57.92%
	2		gi|114778428|ref|ZP_01453273.1|	Mariprofundus ferrooxydans PV-1	E-value = 3.00E-59	Identity = 50.67%
	3		gi|95928481|ref|ZP_01311228.1|	Desulfuromonas acetoxidans DSM 684	E-value = 2.00E-55	Identity = 49.78%
	4		gi|149190294|ref|ZP_01868568.1|	Vibrio shilonii AK1	E-value = 5.00E-55	Identity = 53.13%
	5		gi|77920671|ref|YP_358486.1|	Pelobacter carbinolicus DSM 2380	E-value = 9.00E-55	Identity = 47.75%
AB0434	1		gi|24215865|ref|NP_713346.1|	Leptospira interrogans serovar Lai str. 56601	E-value = 1.00E-22	Identity = 31.23%
	2		gi|157273426|gb|ABV27325.1|	Candidatus Chloracidobacterium thermophilum	E-value = 1.00E-17	Identity = 28.92%
	3		gi|34535523|dbj|BAC87349.1|	Homo sapiens	E-value = 2.00E-17	Identity = 28.63%
	4		gi|114585891|ref|XP_001168299.1|	Pan troglodytes	E-value = 2.00E-17	Identity = 28.63%
	5		gi|109052687|ref|XP_001098913.1|	Macaca mulatta	E-value = 3.00E-17	Identity = 28.63%
AB0435	1		gi|152992667|ref|YP_001358388.1|	Sulfurovum sp. NBC37-1	E-value = 7.00E-19	Identity = 36.88%
	2		gi|148547470|ref|YP_001267572.1|	Pseudomonas putida F1	E-value = 5.00E-13	Identity = 27.91%
	3		gi|26990237|ref|NP_745662.1|	Pseudomonas putida KT2440	E-value = 7.00E-13	Identity = 27.91%
	4		gi|119857144|ref|ZP_01638574.1|	Pseudomonas putida W619	E-value = 2.00E-10	Identity = 26.45%
	5		gi|84317142|ref|ZP_00965598.1|	Pseudomonas aeruginosa C3719	E-value = 3.00E-10	Identity = 27.73%
AB0436	1		gi|152992663|ref|YP_001358384.1|	Sulfurovum sp. NBC37-1	E-value = 6.00E-26	Identity = 37.91%
AB0437	1		gi|152992662|ref|YP_001358383.1|	Sulfurovum sp. NBC37-1	E-value = 4.00E-28	Identity = 40.00%
AB0438	1		gi|152992666|ref|YP_001358387.1|	Sulfurovum sp. NBC37-1	E-value = 1.00E-108	Identity = 48.72%
AB0439	1		gi|152992665|ref|YP_001358386.1|	Sulfurovum sp. NBC37-1	E-value = 5.00E-48	Identity = 50.00%
	2		gi|145622466|ref|ZP_01778425.1|	Petrotoga mobilis SJ95	E-value = 7.00E-44	Identity = 47.03%
	3		gi|154684709|ref|YP_001419870.1|	Bacillus amyloliquefaciens FZB42	E-value = 1.00E-43	Identity = 50.75%
	4		gi|152991413|ref|YP_001357135.1|	Nitratiruptor sp. SB155-2	E-value = 1.00E-42	Identity = 43.98%
	5		gi|153938448|ref|YP_001389610.1|	Clostridium botulinum F str. Langeland	E-value = 2.00E-41	Identity = 48.29%
AB0440	1		gi|152992664|ref|YP_001358385.1|	Sulfurovum sp. NBC37-1	E-value = 7.00E-83	Identity = 48.43%
	2		gi|150401758|ref|YP_001325524.1|	Methanococcus aeolicus Nankai-3	E-value = 5.00E-13	Identity = 25.45%
AB0442	1			***** No matches found *****		
AB0443	1	*suhB*	gi|149194588|ref|ZP_01871684.1|	Caminibacter mediatlanticus TB-2	E-value = 2.00E-72	Identity = 56.69%
	2		gi|152991453|ref|YP_001357175.1|	Nitratiruptor sp. SB155-2	E-value = 3.00E-62	Identity = 49.02%
	3		gi|120602452|ref|YP_966852.1|	Desulfovibrio vulgaris subsp. vulgaris DP4	E-value = 5.00E-47	Identity = 37.70%

	4		gi|46580090|ref|YP_010898.1|	Desulfovibrio vulgaris subsp. vulgaris str. Hildenborough	E-value = 1.00E-46	Identity = 37.70%
	5		gi|85860102|ref|YP_462304.1|	Syntrophus aciditrophicus SB	E-value = 1.00E-45	Identity = 37.60%
AB0444	1	*glmS*	gi|152990396|ref|YP_001356118.1|	Nitratiruptor sp. SB155-2	E-value = 0	Identity = 67.88%
	2		gi|78777680|ref|YP_393995.1|	Sulfuromonas denitrificans ATCC 33889	E-value = 0	Identity = 68.48%
	3		gi|152992132|ref|YP_001357853.1|	Sulfurovum sp. NBC37-1	E-value = 0	Identity = 67.22%
	4		gi|154149127|ref|YP_001406394.1|	Campylobacter hominis ATCC BAA-381	E-value = 0	Identity = 65.24%
	5		gi|157164153|ref|YP_001466572.1|	Campylobacter concisus 13826	E-value = 0	Identity = 64.74%
AB0445	1	*metK*	gi|157165275|ref|YP_001467053.1|	Campylobacter concisus 13826	E-value = 5.00E-179	Identity = 78.84%
	2		gi|154175411|ref|YP_001408530.1|	Campylobacter curvus 525.92	E-value = 9.00E-177	Identity = 77.08%
	3		gi|78777673|ref|YP_393988.1|	Sulfuromonas denitrificans ATCC 33889	E-value = 4.00E-176	Identity = 77.08%
	4		gi|118474936|ref|YP_891896.1|	Campylobacter fetus subsp. fetus 82-40	E-value = 9.00E-175	Identity = 76.01%
	5		gi|57237978|ref|YP_179227.1|	Campylobacter jejuni subsp. jejuni RM1221	E-value = 4.00E-173	Identity = 76.26%
AB0446	1	*accD*	gi|152990942|ref|YP_001356664.1|	Nitratiruptor sp. SB155-2	E-value = 1.00E-117	Identity = 74.09%
	2		gi|78777372|ref|YP_393687.1|	Sulfuromonas denitrificans ATCC 33889	E-value = 5.00E-117	Identity = 72.30%
	3		gi|34556605|ref|NP_906420.1|	Wolinella succinogenes DSM 1740	E-value = 6.00E-110	Identity = 71.05%
	4		gi|154174102|ref|YP_001408791.1|	Campylobacter curvus 525.92	E-value = 5.00E-108	Identity = 68.31%
	5		gi|57168702|ref|ZP_00367834.1|	Campylobacter coli RM2228	E-value = 8.00E-108	Identity = 67.62%
AB0447	1	*thiE*	gi|78777371|ref|YP_393686.1|	Sulfuromonas denitrificans ATCC 33889	E-value = 9.00E-39	Identity = 52.17%
	2		gi|149194760|ref|ZP_01871855.1|	Caminibacter mediatlanticus TB-2	E-value = 3.00E-32	Identity = 46.88%
	3		gi|152990941|ref|YP_001356663.1|	Nitratiruptor sp. SB155-2	E-value = 7.00E-32	Identity = 41.94%
	4		gi|76260809|ref|ZP_00768438.1|	Chloroflexus aurantiacus J-10-fl	E-value = 2.00E-11	Identity = 32.37%
	5		gi|118728152|ref|ZP_01576722.1|	Clostridium cellulolyticum H10	E-value = 8.00E-11	Identity = 31.25%
AB0448	1		gi|157165650|ref|YP_001466368.1|	Campylobacter concisus 13826	E-value = 7.00E-36	Identity = 49.01%
	2		gi|154174606|ref|YP_001408792.1|	Campylobacter curvus 525.92	E-value = 8.00E-35	Identity = 49.67%
	3		gi|78777370|ref|YP_393685.1|	Sulfuromonas denitrificans ATCC 33889	E-value = 1.00E-33	Identity = 50.00%
	4		gi|34556604|ref|NP_906419.1|	Wolinella succinogenes DSM 1740	E-value = 1.00E-31	Identity = 50.00%
	5		gi|152992912|ref|YP_001358633.1|	Sulfurovum sp. NBC37-1	E-value = 2.00E-31	Identity = 48.37%
AB0449	1		gi|152990939|ref|YP_001356661.1|	Nitratiruptor sp. SB155-2	E-value = 2.00E-33	Identity = 29.25%
	2		gi|78777368|ref|YP_393683.1|	Sulfuromonas denitrificans ATCC 33889	E-value = 1.00E-26	Identity = 26.79%
	3		gi|15791512|ref|NP_281335.1|	Campylobacter jejuni subsp. jejuni NCTC 11168	E-value = 2.00E-26	Identity = 29.55%
	4		gi|148926295|ref|ZP_01809979.1|	Campylobacter jejuni subsp. jejuni CG8486	E-value = 3.00E-26	Identity = 29.55%
	5		gi|86153822|ref|ZP_01072025.1|	Campylobacter jejuni subsp. jejuni HB93-13	E-value = 4.00E-26	Identity = 29.55%
AB0450	1		gi|152990938|ref|YP_001356660.1|	Nitratiruptor sp. SB155-2	E-value = 9.00E-116	Identity = 66.12%
	2		gi|78777367|ref|YP_393682.1|	Sulfuromonas denitrificans ATCC 33889	E-value = 8.00E-114	Identity = 64.08%
	3		gi|152992909|ref|YP_001358630.1|	Sulfurovum sp. NBC37-1	E-value = 6.00E-109	Identity = 60.97%
	4		gi|118475725|ref|YP_891451.1|	Campylobacter fetus subsp. fetus 82-40	E-value = 2.00E-97	Identity = 56.21%
	5		gi|34556610|ref|NP_906425.1|	Wolinella succinogenes DSM 1740	E-value = 2.00E-96	Identity = 57.10%
AB0451	1	*prmA*	gi|78777868|ref|YP_394183.1|	Sulfuromonas denitrificans ATCC 33889	E-value = 8.00E-66	Identity = 48.38%
	2		gi|152991152|ref|YP_001356874.1|	Nitratiruptor sp. SB155-2	E-value = 3.00E-64	Identity = 47.14%
	3		gi|149194983|ref|ZP_01872076.1|	Caminibacter mediatlanticus TB-2	E-value = 2.00E-63	Identity = 52.92%
	4		gi|157164415|ref|YP_001467284.1|	Campylobacter concisus 13826	E-value = 6.00E-61	Identity = 46.21%
	5		gi|118474775|ref|YP_892494.1|	Campylobacter fetus subsp. fetus 82-40	E-value = 2.00E-60	Identity = 46.35%
AB0452	1	*ftsH1*	gi|34557037|ref|NP_906852.1|	Wolinella succinogenes DSM 1740	E-value = 0	Identity = 68.78%
	2		gi|152991151|ref|YP_001356873.1|	Nitratiruptor sp. SB155-2	E-value = 0	Identity = 66.11%
	3		gi|121612703|ref|YP_001000794.1|	Campylobacter jejuni subsp. jejuni 81-176	E-value = 0	Identity = 67.6%
	4		gi|57237998|ref|YP_179247.1|	Campylobacter jejuni subsp. jejuni RM1221	E-value = 0	Identity = 67.6%
	5		gi|86150719|ref|ZP_01068935.1|	Campylobacter jejuni subsp. jejuni 260.94	E-value = 0	Identity = 67.6%
AB0453	1		gi|85860311|ref|YP_462513.1|	Syntrophus aciditrophicus SB	E-value = 8.00E-40	Identity = 25.63%
AB0454	1		gi|118743495|ref|ZP_01591498.1|	Geobacter lovleyi SZ	E-value = 4.00E-15	Identity = 35.65%

	2		gi|118743931|ref|ZP_01591930.1|	Geobacter lovleyi SZ	E-value = 4.00E-15	Identity = 38.84%
	3		gi|78221998|ref|YP_383745.1|	Geobacter metallireducens GS-15	E-value = 2.00E-12	Identity = 35.59%
	4		gi|39997787|ref|NP_953738.1|	Geobacter sulfurreducens PCA	E-value = 6.00E-12	Identity = 33.93%
	5		gi|148252134|ref|YP_001236719.1|	Bradyrhizobium sp. BTAi1	E-value = 8.00E-12	Identity = 33.61%
AB0455	1		gi|144897852|emb|CAM74716.1|	Magnetospirillum gryphiswaldense MSR-1	E-value = 1.00E-149	Identity = 35.35%
	2		gi|83311112|ref|YP_421376.1|	Magnetospirillum magneticum AMB-1	E-value = 1.00E-146	Identity = 35.27%
	3		gi|119900236|ref|YP_935449.1|	Azoarcus sp. BH72	E-value = 5.00E-138	Identity = 33.53%
	4		gi|71908921|ref|YP_286508.1|	Dechloromonas aromatica RCB	E-value = 1.00E-136	Identity = 34.13%
	5		gi|118581218|ref|YP_902468.1|	Pelobacter propionicus DSM 2379	E-value = 1.00E-114	Identity = 31.03%
AB0456	1	*leuA1*	gi|152993536|ref|YP_001359257.1|	Sulfurovum sp. NBC37-1	E-value = 0	Identity = 69.31%
	2		gi|152991149|ref|YP_001356871.1|	Nitratiruptor sp. SB155-2	E-value = 0	Identity = 68.82%
	3		gi|34557040|ref|NP_906855.1|	Wolinella succinogenes DSM 1740	E-value = 0	Identity = 66.21%
	4		gi|118474450|ref|YP_892490.1|	Campylobacter fetus subsp. fetus 82-40	E-value = 0	Identity = 67.72%
	5		gi|154149292|ref|YP_001406079.1|	Campylobacter hominis ATCC BAA-381	E-value = 0	Identity = 66.47%
AB0457	1		gi|152990985|ref|YP_001356707.1|	Nitratiruptor sp. SB155-2	E-value = 4.00E-17	Identity = 43.40%
	2		gi|78776543|ref|YP_392858.1|	Sulfuromonas denitrificans ATCC 33889	E-value = 2.00E-16	Identity = 40.38%
	3		gi|152993205|ref|YP_001358926.1|	Sulfurovum sp. NBC37-1	E-value = 4.00E-16	Identity = 40.00%
	4		gi|67941706|ref|ZP_00533752.1|	Chlorobium phaeobacteroides BS1	E-value = 4.00E-14	Identity = 33.33%
	5		gi|88797576|ref|ZP_01113165.1|	Reinekea sp. MED297	E-value = 2.00E-13	Identity = 39.00%
AB0458	1		gi|152991495|ref|YP_001357217.1|	Nitratiruptor sp. SB155-2	E-value = 2.00E-30	Identity = 47.10%
	2		gi|86142737|ref|ZP_01061176.1|	Flavobacterium sp. MED217	E-value = 2.00E-24	Identity = 42.76%
	3		gi|152992794|ref|YP_001358515.1|	Sulfurovum sp. NBC37-1	E-value = 9.00E-23	Identity = 43.28%
	4		gi|152992506|ref|YP_001358227.1|	Sulfurovum sp. NBC37-1	E-value = 2.00E-22	Identity = 36.51%
	5		gi|88804119|ref|ZP_01119639.1|	Robiginitalea biformata HTCC2501	E-value = 7.00E-22	Identity = 41.35%
AB0459	1	*mutS1*	gi|78777643|ref|YP_393958.1|	Sulfuromonas denitrificans ATCC 33889	E-value = 0	Identity = 51.12%
	2		gi|152990266|ref|YP_001355988.1|	Nitratiruptor sp. SB155-2	E-value = 0	Identity = 50.71%
	3		gi|55819234|ref|YP_142713.1|	Acanthamoeba polyphaga mimivirus	E-value = 1.00E-113	Identity = 30.42%
	4		gi|33087146|gb|AAP92777.1|	Leptogorgia chilensis	E-value = 7.00E-69	Identity = 27.13%
	5		gi|3914061|sp|O63852|MSHM_SARGL	Sarcophyton glaucum	E-value = 8.00E-69	Identity = 26.36%
AB0460	1		gi|34557635|ref|NP_907450.1|	Wolinella succinogenes DSM 1740	E-value = 0	Identity = 48.21%
	2		gi|32265721|ref|NP_859753.1|	Helicobacter hepaticus ATCC 51449	E-value = 0	Identity = 44.17%
	3		gi|152991871|ref|YP_001357592.1|	Sulfurovum sp. NBC37-1	E-value = 0	Identity = 44.76%
	4		gi|3068786|gb|AAC14433.1|	Helicobacter pylori	E-value = 0	Identity = 43.47%
	5		gi|109947899|ref|YP_665127.1|	Helicobacter acinonychis str. Sheeba	E-value = 0	Identity = 43.09%
AB0461	1		gi|32265722|ref|NP_859754.1|	Helicobacter hepaticus ATCC 51449	E-value = 2.00E-32	Identity = 38.18%
	2		gi|15611620|ref|NP_223271.1|	Helicobacter pylori J99	E-value = 7.00E-28	Identity = 37.14%
	3		gi|3068787|gb|AAC14434.1|	Helicobacter pylori	E-value = 9.00E-28	Identity = 37.14%
	4		gi|109947900|ref|YP_665128.1|	Helicobacter acinonychis str. Sheeba	E-value = 1.00E-27	Identity = 35.71%
	5		gi|108563012|ref|YP_627328.1|	Helicobacter pylori HPAG1	E-value = 1.00E-27	Identity = 37.14%
AB0462	1		gi|34557633|ref|NP_907448.1|	Wolinella succinogenes DSM 1740	E-value = 9.00E-31	Identity = 29.03%
	2		gi|3068785|gb|AAC14432.1|	Helicobacter pylori	E-value = 3.00E-25	Identity = 27.06%
	3		gi|15645230|ref|NP_207400.1|	Helicobacter pylori 26695	E-value = 8.00E-25	Identity = 28.13%
	4		gi|108563011|ref|YP_627327.1|	Helicobacter pylori HPAG1	E-value = 1.00E-24	Identity = 28.13%
	5		gi|15611619|ref|NP_223270.1|	Helicobacter pylori J99	E-value = 1.00E-24	Identity = 28.13%
AB0463	1		gi|34557295|ref|NP_907110.1|	Wolinella succinogenes DSM 1740	E-value = 9.00E-27	Identity = 40.31%
AB0464	1		gi|54302981|ref|YP_132974.1|	Photobacterium profundum SS9	E-value = 7.00E-14	Identity = 27.57%
	2		gi|54303357|ref|YP_133350.1|	Photobacterium profundum SS9	E-value = 8.00E-14	Identity = 27.57%
	3		gi|126700147|ref|YP_001089044.1|	Clostridium difficile 630	E-value = 3.00E-13	Identity = 26.56%
	4		gi|145952974|ref|ZP_01801982.1|	Clostridium difficile QCD-32g58	E-value = 3.00E-12	Identity = 26.74%

	5		gi|56963185|ref|YP_174916.1|	Bacillus clausii KSM-K16	E-value = 3.00E-09	Identity = 25.27%
AB0465	1		gi|89093842|ref|ZP_01166788.1|	Oceanospirillum sp. MED92	E-value = 2.00E-107	Identity = 38.35%
	2		gi|89094966|ref|ZP_01167896.1|	Oceanospirillum sp. MED92	E-value = 4.00E-107	Identity = 41.02%
	3		gi|56476668|ref|YP_158257.1|	Azoarcus sp. EbN1	E-value = 4.00E-104	Identity = 37.82%
	4		gi|119897530|ref|YP_932743.1|	Azoarcus sp. BH72	E-value = 2.00E-101	Identity = 37.97%
	5		gi|104782044|ref|YP_608542.1|	Pseudomonas entomophila L48	E-value = 6.00E-101	Identity = 39.11%
AB0466	1		gi|89093841|ref|ZP_01166787.1|	Oceanospirillum sp. MED92	E-value = 4.00E-131	Identity = 45.69%
	2		gi|89094965|ref|ZP_01167895.1|	Oceanospirillum sp. MED92	E-value = 2.00E-130	Identity = 46.38%
	3		gi|16950513|dbj|BAB72009.1|	Pseudomonas putida	E-value = 2.00E-126	Identity = 45.26%
	4		gi|119855927|ref|ZP_01637361.1|	Pseudomonas putida W619	E-value = 3.00E-126	Identity = 45.26%
	5		gi|148547529|ref|YP_001267631.1|	Pseudomonas putida F1	E-value = 5.00E-126	Identity = 45.26%
AB0467	1		gi|119897532|ref|YP_932745.1|	Azoarcus sp. BH72	E-value = 1.00E-32	Identity = 66.35%
	2		gi|119855926|ref|ZP_01637360.1|	Pseudomonas putida W619	E-value = 7.00E-32	Identity = 63.81%
	3		gi|126355720|ref|ZP_01712726.1|	Pseudomonas putida GB-1	E-value = 1.00E-31	Identity = 63.81%
	4		gi|104782042|ref|YP_608540.1|	Pseudomonas entomophila L48	E-value = 2.00E-31	Identity = 62.86%
	5		gi|26990173|ref|NP_745598.1|	Pseudomonas putida KT2440	E-value = 4.00E-31	Identity = 62.86%
AB0468	1		gi|104782041|ref|YP_608539.1|	Pseudomonas entomophila L48	E-value = 2.00E-73	Identity = 37.43%
	2		gi|70731473|ref|YP_261214.1|	Pseudomonas fluorescens Pf-5	E-value = 2.00E-72	Identity = 39.07%
	3		gi|148547531|ref|YP_001267633.1|	Pseudomonas putida F1	E-value = 1.00E-71	Identity = 38.66%
	4		gi|126355719|ref|ZP_01712725.1|	Pseudomonas putida GB-1	E-value = 1.00E-71	Identity = 38.95%
	5		gi|26990172|ref|NP_745597.1|	Pseudomonas putida KT2440	E-value = 1.00E-71	Identity = 38.66%
AB0469	1		gi|89094962|ref|ZP_01167892.1|	Oceanospirillum sp. MED92	E-value = 3.00E-67	Identity = 28.34%
	2		gi|77459205|ref|YP_348711.1|	Pseudomonas fluorescens PfO-1	E-value = 6.00E-61	Identity = 26.62%
	3		gi|70731490|ref|YP_261231.1|	Pseudomonas fluorescens Pf-5	E-value = 4.00E-58	Identity = 26.11%
	4		gi|104781894|ref|YP_608392.1|	Pseudomonas entomophila L48	E-value = 2.00E-55	Identity = 27.48%
	5		gi|148547523|ref|YP_001267625.1|	Pseudomonas putida F1	E-value = 2.00E-53	Identity = 27.20%
AB0470	1		gi|89094961|ref|ZP_01167891.1|	Oceanospirillum sp. MED92	E-value = 7.00E-15	Identity = 29.19%
	2		gi|119855932|ref|ZP_01637366.1|	Pseudomonas putida W619	E-value = 1.00E-10	Identity = 26.00%
	3		gi|126355726|ref|ZP_01712732.1|	Pseudomonas putida GB-1	E-value = 5.00E-10	Identity = 25.38%
	4		gi|104781893|ref|YP_608391.1|	Pseudomonas entomophila L48	E-value = 3.00E-09	Identity = 25.12%
	5		gi|77459196|ref|YP_348702.1|	Pseudomonas fluorescens PfO-1	E-value = 3.00E-07	Identity = 25.81%
AB0471	1		gi|89094960|ref|ZP_01167890.1|	Oceanospirillum sp. MED92	E-value = 1.00E-52	Identity = 32.68%
	2		gi|119855931|ref|ZP_01637365.1|	Pseudomonas putida W619	E-value = 1.00E-51	Identity = 33.00%
	3		gi|77459204|ref|YP_348710.1|	Pseudomonas fluorescens PfO-1	E-value = 4.00E-47	Identity = 33.09%
	4		gi|148547525|ref|YP_001267627.1|	Pseudomonas putida F1	E-value = 1.00E-45	Identity = 33.25%
	5		gi|126355725|ref|ZP_01712731.1|	Pseudomonas putida GB-1	E-value = 1.00E-45	Identity = 33.25%
AB0472	1		gi|78777681|ref|YP_393996.1|	Sulfuromonas denitrificans ATCC 33889	E-value = 2.00E-118	Identity = 38.35%
	2		gi|34556481|ref|NP_906296.1|	Wolinella succinogenes DSM 1740	E-value = 6.00E-104	Identity = 35.77%
	3		gi|152990872|ref|YP_001356594.1|	Nitratiruptor sp. SB155-2	E-value = 6.00E-100	Identity = 36.67%
	4		gi|83310566|ref|YP_420830.1|	Magnetospirillum magneticum AMB-1	E-value = 2.00E-95	Identity = 32.59%
	5		gi|144898358|emb|CAM75222.1|	Magnetospirillum gryphiswaldense MSR-1	E-value = 6.00E-94	Identity = 31.68%
AB0473	1	*hisA*	gi|152991153|ref|YP_001356875.1|	Nitratiruptor sp. SB155-2	E-value = 4.00E-98	Identity = 77.45%
	2		gi|152993531|ref|YP_001359252.1|	Sulfurovum sp. NBC37-1	E-value = 6.00E-97	Identity = 78.72%
	3		gi|78777870|ref|YP_394185.1|	Sulfuromonas denitrificans ATCC 33889	E-value = 4.00E-89	Identity = 72.53%
	4		gi|34557034|ref|NP_906849.1|	Wolinella succinogenes DSM 1740	E-value = 6.00E-80	Identity = 66.81%
	5		gi|154148634|ref|YP_001406507.1|	Campylobacter hominis ATCC BAA-381	E-value = 4.00E-78	Identity = 67.09%
AB0474	1		gi|34556546|ref|NP_906361.1|	Wolinella succinogenes DSM 1740	E-value = 1.00E-73	Identity = 48.23%
	2		gi|78778045|ref|YP_394360.1|	Sulfuromonas denitrificans ATCC 33889	E-value = 3.00E-65	Identity = 46.45%
	3		gi|157273524|gb|ABV27423.1|	Candidatus Chloracidobacterium thermophilum	E-value = 1.00E-57	Identity = 39.67%

	4		gi|114563726|ref|YP_751239.1|	Shewanella frigidimarina NCIMB 400	E-value = 5.00E-57	Identity = 42.18%
	5		gi|78485127|ref|YP_391052.1|	Thiomicrospira crunogena XCL-2	E-value = 1.00E-56	Identity = 39.65%
AB0475	1	*hisH1*	gi|149194980|ref|ZP_01872073.1|	Caminibacter mediatlanticus TB-2	E-value = 2.00E-66	Identity = 63.68%
	2		gi|152991154|ref|YP_001356876.1|	Nitratiruptor sp. SB155-2	E-value = 2.00E-66	Identity = 63.86%
	3		gi|78777871|ref|YP_394186.1|	Sulfuromonas denitrificans ATCC 33889	E-value = 4.00E-66	Identity = 65.00%
	4		gi|34557033|ref|NP_906848.1|	Wolinella succinogenes DSM 1740	E-value = 4.00E-65	Identity = 59.70%
	5		gi|152993530|ref|YP_001359251.1|	Sulfurovum sp. NBC37-1	E-value = 6.00E-63	Identity = 61.08%
AB0476	1	*ctsW*	gi|78777835|ref|YP_394150.1|	Sulfuromonas denitrificans ATCC 33889	E-value = 2.00E-35	Identity = 43.68%
	2		gi|15792355|ref|NP_282178.1|	Campylobacter jejuni subsp. jejuni NCTC 11168	E-value = 1.00E-34	Identity = 44.50%
	3		gi|152990186|ref|YP_001355908.1|	Nitratiruptor sp. SB155-2	E-value = 1.00E-34	Identity = 42.63%
	4		gi|57237915|ref|YP_179163.1|	Campylobacter jejuni subsp. jejuni RM1221	E-value = 2.00E-34	Identity = 43.98%
	5		gi|154173802|ref|YP_001408751.1|	Campylobacter curvus 525.92	E-value = 3.00E-34	Identity = 44.44%
AB0477	1		gi|109898163|ref|YP_661418.1|	Pseudoalteromonas atlantica T6c	E-value = 1.00E-15	Identity = 40.63%
	2		gi|120599736|ref|YP_964310.1|	Shewanella sp. W3-18-1	E-value = 5.00E-15	Identity = 39.10%
	3		gi|145618719|ref|ZP_01774776.1|	Geobacter bemidjiensis Bem	E-value = 8.00E-14	Identity = 41.88%
	4		gi|94502010|ref|ZP_01308517.1|	Oceanobacter sp. RED65	E-value = 1.00E-10	Identity = 35.61%
AB0479	1	*lepA*	gi|152992639|ref|YP_001358360.1|	Sulfurovum sp. NBC37-1	E-value = 0	Identity = 82.18%
	2		gi|152990185|ref|YP_001355907.1|	Nitratiruptor sp. SB155-2	E-value = 0	Identity = 76.43%
	3		gi|154173908|ref|YP_001408755.1|	Campylobacter curvus 525.92	E-value = 0	Identity = 75.08%
	4		gi|157164531|ref|YP_001466501.1|	Campylobacter concisus 13826	E-value = 0	Identity = 74.75%
	5		gi|34557971|ref|NP_907786.1|	Wolinella succinogenes DSM 1740	E-value = 0	Identity = 74.07%
AB0480	1		gi|15896889|ref|NP_350238.1|	Clostridium acetobutylicum ATCC 824	E-value = 2.00E-21	Identity = 68.18%
	2		gi|153953431|ref|YP_001394196.1|	Clostridium kluyveri DSM 555	E-value = 3.00E-21	Identity = 65.91%
	3		gi|148379283|ref|YP_001253824.1|	Clostridium botulinum A str. ATCC 3502	E-value = 2.00E-20	Identity = 64.77%
	4		gi|150019612|ref|YP_001311866.1|	Clostridium beijerinckii NCIMB 8052	E-value = 2.00E-19	Identity = 61.36%
	5		gi|110799183|ref|YP_694658.1|	Clostridium perfringens ATCC 13124	E-value = 2.00E-16	Identity = 61.80%
AB0481	1		gi|154496596|ref|ZP_02035292.1|	Bacteroides capillosus ATCC 29799	E-value = 0	Identity = 54.99%
	2		gi|156868268|gb|EDO61640.1|	Clostridium leptum DSM 753	E-value = 0	Identity = 54.79%
	3		gi|42525527|ref|NP_970625.1|	Treponema denticola ATCC 35405	E-value = 0	Identity = 52.53%
	4		gi|29374937|ref|NP_814090.1|	Enterococcus faecalis V583	E-value = 0	Identity = 52.37%
	5		gi|118444416|ref|YP_878393.1|	Clostridium novyi NT	E-value = 0	Identity = 53.7%
AB0483	1	*cadF*	gi|118474477|ref|YP_891591.1|	Campylobacter fetus subsp. fetus 82-40	E-value = 6.00E-61	Identity = 41.86%
	2		gi|154147928|ref|YP_001407122.1|	Campylobacter hominis ATCC BAA-381	E-value = 4.00E-58	Identity = 40.41%
	3		gi|154174678|ref|YP_001407584.1|	Campylobacter curvus 525.92	E-value = 8.00E-57	Identity = 40.29%
	4		gi|157164740|ref|YP_001466195.1|	Campylobacter concisus 13826	E-value = 2.00E-54	Identity = 41.64%
	5		gi|34556650|ref|NP_906465.1|	Wolinella succinogenes DSM 1740	E-value = 3.00E-54	Identity = 40.52%
AB0484	1	*prsA*	gi|152992638|ref|YP_001358359.1|	Sulfurovum sp. NBC37-1	E-value = 1.00E-129	Identity = 74.03%
	2		gi|34557951|ref|NP_907766.1|	Wolinella succinogenes DSM 1740	E-value = 4.00E-126	Identity = 72.40%
	3		gi|57242096|ref|ZP_00370036.1|	Campylobacter upsaliensis RM3195	E-value = 3.00E-122	Identity = 71.43%
	4		gi|118474648|ref|YP_892608.1|	Campylobacter fetus subsp. fetus 82-40	E-value = 3.00E-121	Identity = 68.93%
	5		gi|157164147|ref|YP_001467207.1|	Campylobacter concisus 13826	E-value = 4.00E-121	Identity = 69.26%
AB0485	1		gi|34557902|ref|NP_907717.1|	Wolinella succinogenes DSM 1740	E-value = 4.00E-51	Identity = 37.14%
	2		gi|94271809|ref|ZP_01292017.1|	delta proteobacterium MLMS-1	E-value = 5.00E-38	Identity = 32.37%
	3		gi|94264011|ref|ZP_01287812.1|	delta proteobacterium MLMS-1	E-value = 1.00E-37	Identity = 32.11%
AB0486	1	*trmU1*	gi|78776909|ref|YP_393224.1|	Sulfuromonas denitrificans ATCC 33889	E-value = 2.00E-101	Identity = 54.57%
	2		gi|152992636|ref|YP_001358357.1|	Sulfurovum sp. NBC37-1	E-value = 2.00E-99	Identity = 51.32%
	3		gi|121613044|ref|YP_999778.1|	Campylobacter jejuni subsp. jejuni 81-176	E-value = 7.00E-99	Identity = 55.16%
	4		gi|153951834|ref|YP_001397317.1|	Campylobacter jejuni subsp. doylei 269.97	E-value = 1.00E-98	Identity = 54.87%
	5		gi|157414371|ref|YP_001481627.1|	Campylobacter jejuni subsp. jejuni 81116	E-value = 2.00E-98	Identity = 55.16%

AB0487	1	*trmU2*	gi|124895824|gb|EAY69704.1|	Burkholderia dolosa AUO158	E-value = 1.00E-106	Identity = 53.19%
	2		gi|115350645|ref|YP_772484.1|	Burkholderia cepacia AMMD	E-value = 5.00E-106	Identity = 53.19%
	3		gi|84360823|ref|ZP_00985512.1|	Burkholderia dolosa AUO158	E-value = 2.00E-105	Identity = 53.35%
	4		gi|76810425|ref|YP_334767.1|	Burkholderia pseudomallei 1710b	E-value = 7.00E-105	Identity = 52.76%
	5		gi|99912870|ref|ZP_01319568.1|	Burkholderia pseudomallei 1655	E-value = 2.00E-104	Identity = 52.49%
AB0488	1	*folK*	gi|152992633|ref|YP_001358354.1|	Sulfurovum sp. NBC37-1	E-value = 9.00E-36	Identity = 46.84%
	2		gi|152990181|ref|YP_001355903.1|	Nitratiruptor sp. SB155-2	E-value = 1.00E-34	Identity = 46.20%
	3		gi|78776887|ref|YP_393202.1|	Sulfuromonas denitrificans ATCC 33889	E-value = 1.00E-32	Identity = 50.31%
	4		gi|154174244|ref|YP_001408774.1|	Campylobacter curvus 525.92	E-value = 1.00E-28	Identity = 44.37%
	5		gi|154148891|ref|YP_001406763.1|	Campylobacter hominis ATCC BAA-381	E-value = 2.00E-28	Identity = 44.52%
AB0489	1	*pepQ*	gi|152992632|ref|YP_001358353.1|	Sulfurovum sp. NBC37-1	E-value = 3.00E-99	Identity = 54.28%
	2		gi|152990180|ref|YP_001355902.1|	Nitratiruptor sp. SB155-2	E-value = 2.00E-94	Identity = 53.24%
	3		gi|34557960|ref|NP_907775.1|	Wolinella succinogenes DSM 1740	E-value = 1.00E-92	Identity = 48.39%
	4		gi|32266443|ref|NP_860475.1|	Helicobacter hepaticus ATCC 51449	E-value = 4.00E-92	Identity = 54.12%
	5		gi|157164005|ref|YP_001467218.1|	Campylobacter concisus 13826	E-value = 7.00E-90	Identity = 53.08%
AB0490	1	*aroQ*	gi|34557961|ref|NP_907776.1|	Wolinella succinogenes DSM 1740	E-value = 8.00E-53	Identity = 68.92%
	2		gi|57241948|ref|ZP_00369888.1|	Campylobacter upsaliensis RM3195	E-value = 1.00E-50	Identity = 68.49%
	3		gi|148926872|ref|ZP_01810550.1|	Campylobacter jejuni subsp. jejuni CG8486	E-value = 2.00E-50	Identity = 62.67%
	4		gi|57168748|ref|ZP_00367879.1|	Campylobacter coli RM2228	E-value = 5.00E-50	Identity = 64.38%
	5		gi|57240763|ref|ZP_00368711.1|	Campylobacter lari RM2100	E-value = 1.00E-49	Identity = 65.96%
AB0491	1		gi|152990178|ref|YP_001355900.1|	Nitratiruptor sp. SB155-2	E-value = 1.00E-90	Identity = 46.65%
	2		gi|149194325|ref|ZP_01871422.1|	Caminibacter mediatlanticus TB-2	E-value = 3.00E-83	Identity = 42.54%
	3		gi|78776885|ref|YP_393200.1|	Sulfuromonas denitrificans ATCC 33889	E-value = 1.00E-82	Identity = 41.32%
	4		gi|152992630|ref|YP_001358351.1|	Sulfurovum sp. NBC37-1	E-value = 1.00E-81	Identity = 38.73%
	5		gi|154174508|ref|YP_001408778.1|	Campylobacter curvus 525.92	E-value = 2.00E-80	Identity = 40.29%
AB0492	1	*pspA*	gi|78776884|ref|YP_393199.1|	Sulfuromonas denitrificans ATCC 33889	E-value = 5.00E-76	Identity = 51.92%
	2		gi|152992629|ref|YP_001358350.1|	Sulfurovum sp. NBC37-1	E-value = 2.00E-72	Identity = 56.97%
	3		gi|152990177|ref|YP_001355899.1|	Nitratiruptor sp. SB155-2	E-value = 8.00E-72	Identity = 52.58%
	4		gi|154148512|ref|YP_001406767.1|	Campylobacter hominis ATCC BAA-381	E-value = 7.00E-67	Identity = 46.37%
	5		gi|86151511|ref|ZP_01069726.1|	Campylobacter jejuni subsp. jejuni 260.94	E-value = 1.00E-65	Identity = 47.55%
AB0493	1			***** No matches found *****		
AB0494	1	*ackA1*	gi|154498124|ref|ZP_02036502.1|	Bacteroides capillosus ATCC 29799	E-value = 1.00E-116	Identity = 52.01%
	2		gi|19704506|ref|NP_604068.1|	Fusobacterium nucleatum subsp. nucleatum ATCC 25586	E-value = 6.00E-115	Identity = 52.02%
	3		gi|148323175|gb|EDK88425.1|	Fusobacterium nucleatum subsp. polymorphum ATCC 10953	E-value = 9.00E-115	Identity = 51.77%
	4		gi|156865731|gb|EDO59162.1|	Clostridium sp. L2-50	E-value = 3.00E-114	Identity = 51.39%
	5		gi|34762396|ref|ZP_00143397.1|	Fusobacterium nucleatum subsp. vincentii ATCC 49256	E-value = 8.00E-114	Identity = 50.76%
AB0495	1	*pta*	gi|19704507|ref|NP_604069.1|	Fusobacterium nucleatum subsp. nucleatum ATCC 25586	E-value = 1.00E-100	Identity = 57.75%
	2		gi|148323174|gb|EDK88424.1|	Fusobacterium nucleatum subsp. polymorphum ATCC 10953	E-value = 2.00E-99	Identity = 56.53%
	3		gi|34762395|ref|ZP_00143396.1|	Fusobacterium nucleatum subsp. vincentii ATCC 49256	E-value = 9.00E-99	Identity = 55.93%
	4		gi|89211635|ref|ZP_01189989.1|	Halothermothrix orenii H 168	E-value = 2.00E-93	Identity = 55.02%
	5		gi|78044760|ref|YP_360288.1|	Carboxydothermus hydrogenoformans Z-2901	E-value = 5.00E-93	Identity = 53.80%
AB0496	1	*ackA2*	gi|19704506|ref|NP_604068.1|	Fusobacterium nucleatum subsp. nucleatum ATCC 25586	E-value = 9.00E-124	Identity = 57.29%
	2		gi|148323175|gb|EDK88425.1|	Fusobacterium nucleatum subsp. polymorphum ATCC 10953	E-value = 1.00E-123	Identity = 57.04%
	3		gi|42526445|ref|NP_971543.1|	Treponema denticola ATCC 35405	E-value = 2.00E-122	Identity = 54.52%
	4		gi|34762396|ref|ZP_00143397.1|	Fusobacterium nucleatum subsp. vincentii ATCC 49256	E-value = 5.00E-122	Identity = 56.53%
	5		gi|78042887|ref|YP_360287.1|	Carboxydothermus hydrogenoformans Z-2901	E-value = 8.00E-118	Identity = 54.91%
AB0497	1		gi|91977554|ref|YP_570213.1|	Rhodopseudomonas palustris BisB5	E-value = 3.00E-94	Identity = 56.17%
	2		gi|15888334|ref|NP_354015.1|	Agrobacterium tumefaciens str. C58	E-value = 8.00E-94	Identity = 58.55%
	3		gi|153010261|ref|YP_001371475.1|	Ochrobactrum anthropi ATCC 49188	E-value = 2.00E-93	Identity = 54.40%

	4		gi|119877696|ref|ZP_01644684.1|	Stenotrophomonas maltophilia R551-3	E-value = 3.00E-92	Identity = 55.45%
	5		gi|21492770|ref|NP_659845.1|	Rhizobium etli CFN 42	E-value = 1.00E-90	Identity = 54.37%
AB0498	1	*ackA3*	gi|42526445|ref|NP_971543.1|	Treponema denticola ATCC 35405	E-value = 4.00E-124	Identity = 54.50%
	2		gi|19704506|ref|NP_604068.1|	Fusobacterium nucleatum subsp. nucleatum ATCC 25586	E-value = 4.00E-123	Identity = 54.52%
	3		gi|34762396|ref|ZP_00143397.1|	Fusobacterium nucleatum subsp. vincentii ATCC 49256	E-value = 4.00E-123	Identity = 55.03%
	4		gi|148323175|gb|EDK88425.1|	Fusobacterium nucleatum subsp. polymorphum ATCC 10953	E-value = 6.00E-123	Identity = 54.02%
	5		gi|146297045|ref|YP_001180816.1|	Caldicellulosiruptor saccharolyticus DSM 8903	E-value = 5.00E-122	Identity = 55.28%
AB0499	1	*dnaQ1*	gi|152992740|ref|YP_001358461.1|	Sulfurovum sp. NBC37-1	E-value = 2.00E-56	Identity = 55.50%
	2		gi|34557013|ref|NP_906828.1|	Wolinella succinogenes DSM 1740	E-value = 1.00E-53	Identity = 51.52%
	3		gi|78485464|ref|YP_391389.1|	Thiomicrospira crunogena XCL-2	E-value = 1.00E-50	Identity = 49.75%
	4		gi|78777649|ref|YP_393964.1|	Sulfuromonas denitrificans ATCC 33889	E-value = 3.00E-50	Identity = 54.36%
	5		gi|27379228|ref|NP_770757.1|	Bradyrhizobium japonicum USDA 110	E-value = 6.00E-47	Identity = 43.78%
AB0500	1		gi|34557014|ref|NP_906829.1|	Wolinella succinogenes DSM 1740	E-value = 3.00E-124	Identity = 40.39%
	2		gi|78777650|ref|YP_393965.1|	Sulfuromonas denitrificans ATCC 33889	E-value = 1.00E-113	Identity = 40.72%
	3		gi|27379227|ref|NP_770756.1|	Bradyrhizobium japonicum USDA 110	E-value = 1.00E-108	Identity = 33.77%
	4		gi|152992739|ref|YP_001358460.1|	Sulfurovum sp. NBC37-1	E-value = 8.00E-107	Identity = 36.33%
	5		gi|83591973|ref|YP_425725.1|	Rhodospirillum rubrum ATCC 11170	E-value = 2.00E-103	Identity = 32.45%
AB0501	1		gi|152990273|ref|YP_001355995.1|	Nitratiruptor sp. SB155-2	E-value = 2.00E-180	Identity = 65.23%
	2		gi|152992738|ref|YP_001358459.1|	Sulfurovum sp. NBC37-1	E-value = 9.00E-179	Identity = 67.50%
	3		gi|34557015|ref|NP_906830.1|	Wolinella succinogenes DSM 1740	E-value = 1.00E-177	Identity = 65.12%
	4		gi|148549182|ref|YP_001269284.1|	Pseudomonas putida F1	E-value = 5.00E-175	Identity = 64.72%
	5		gi|26988474|ref|NP_743899.1|	Pseudomonas putida KT2440	E-value = 5.00E-175	Identity = 63.80%
AB0502	1		gi|118053915|ref|ZP_01522457.1|	Comamonas testosteroni KF-1	E-value = 2.00E-20	Identity = 50.50%
	2		gi|94314701|ref|YP_587910.1|	Ralstonia metallidurans CH34	E-value = 6.00E-20	Identity = 45.00%
	3		gi|145588721|ref|YP_001155318.1|	Polynucleobacter sp. QLW-P1DMWA-1	E-value = 8.00E-20	Identity = 49.49%
	4		gi|89902541|ref|YP_525012.1|	Rhodoferax ferrireducens T118	E-value = 1.00E-19	Identity = 48.98%
	5		gi|119897268|ref|YP_932481.1|	Azoarcus sp. BH72	E-value = 4.00E-19	Identity = 47.47%
AB0504	1		gi|152992738|ref|YP_001358459.1|	Sulfurovum sp. NBC37-1	E-value = 1.00E-172	Identity = 65.22%
	2		gi|157148011|ref|YP_001455330.1|	Citrobacter koseri ATCC BAA-895	E-value = 3.00E-170	Identity = 63.65%
	3		gi|15598430|ref|NP_251924.1|	Pseudomonas aeruginosa PAO1	E-value = 7.00E-170	Identity = 64.64%
	4		gi|67155148|ref|ZP_00416776.1|	Azotobacter vinelandii AvOP	E-value = 2.00E-169	Identity = 66.79%
	5		gi|152990273|ref|YP_001355995.1|	Nitratiruptor sp. SB155-2	E-value = 2.00E-169	Identity = 64.47%
AB0505	1		gi|78777652|ref|YP_393967.1|	Sulfuromonas denitrificans ATCC 33889	E-value = 8.00E-20	Identity = 48.54%
	2		gi|34557016|ref|NP_906831.1|	Wolinella succinogenes DSM 1740	E-value = 3.00E-17	Identity = 46.53%
	3		gi|153870669|ref|ZP_02000019.1|	Beggiatoa sp. PS	E-value = 1.00E-16	Identity = 53.92%
	4		gi|69933594|ref|ZP_00628796.1|	Paracoccus denitrificans PD1222	E-value = 2.00E-16	Identity = 44.44%
	5		gi|145588721|ref|YP_001155318.1|	Polynucleobacter sp. QLW-P1DMWA-1	E-value = 6.00E-16	Identity = 46.46%
AB0506	1	*dnaQ2*	gi|78777649|ref|YP_393964.1|	Sulfuromonas denitrificans ATCC 33889	E-value = 8.00E-54	Identity = 56.57%
	2		gi|145588723|ref|YP_001155320.1|	Polynucleobacter sp. QLW-P1DMWA-1	E-value = 2.00E-51	Identity = 47.76%
	3		gi|152992740|ref|YP_001358461.1|	Sulfurovum sp. NBC37-1	E-value = 2.00E-51	Identity = 52.26%
	4		gi|152990271|ref|YP_001355993.1|	Nitratiruptor sp. SB155-2	E-value = 2.00E-50	Identity = 54.55%
	5		gi|78485464|ref|YP_391389.1|	Thiomicrospira crunogena XCL-2	E-value = 2.00E-49	Identity = 48.02%
AB0507	1		gi|78777650|ref|YP_393965.1|	Sulfuromonas denitrificans ATCC 33889	E-value = 8.00E-126	Identity = 42.36%
	2		gi|152992739|ref|YP_001358460.1|	Sulfurovum sp. NBC37-1	E-value = 6.00E-112	Identity = 37.42%
	3		gi|152990272|ref|YP_001355994.1|	Nitratiruptor sp. SB155-2	E-value = 4.00E-107	Identity = 37.48%
	4		gi|34557014|ref|NP_906829.1|	Wolinella succinogenes DSM 1740	E-value = 4.00E-107	Identity = 36.65%
	5		gi|89094458|ref|ZP_01167398.1|	Oceanospirillum sp. MED92	E-value = 5.00E-104	Identity = 35.26%
AB0508	1		gi|54310464|ref|YP_131484.1|	Photobacterium profundum SS9	E-value = 4.00E-130	Identity = 43.99%
	2		gi|119477496|ref|ZP_01617687.1|	marine gamma proteobacterium HTCC2143	E-value = 7.00E-130	Identity = 41.17%

	3		gi|90413369|ref|ZP_01221362.1|	Photobacterium profundum 3TCK	E-value = 2.00E-129	Identity = 43.47%
	4		gi|21674620|ref|NP_662685.1|	Chlorobium tepidum TLS	E-value = 5.00E-129	Identity = 43.41%
	5		gi|117924349|ref|YP_864966.1|	Magnetococcus sp. MC-1	E-value = 3.00E-128	Identity = 42.49%
AB0509	1		gi|88703630|ref|ZP_01101346.1|	gamma proteobacterium KT 71	E-value = 7.00E-19	Identity = 57.14%
	2		gi|149927004|ref|ZP_01915262.1|	Limnobacter sp. MED105	E-value = 6.00E-18	Identity = 57.83%
	3		gi|94499936|ref|ZP_01306471.1|	Oceanobacter sp. RED65	E-value = 1.00E-17	Identity = 53.01%
	4		gi|88797307|ref|ZP_01112897.1|	Reinekea sp. MED297	E-value = 2.00E-17	Identity = 54.88%
	5		gi|118755048|ref|ZP_01602841.1|	Shewanella pealeana ATCC 700345	E-value = 2.00E-17	Identity = 54.22%
AB0510	1		gi|152990276|ref|YP_001355998.1|	Nitratiruptor sp. SB155-2	E-value = 3.00E-56	Identity = 54.84%
	2		gi|78777655|ref|YP_393970.1|	Sulfuromonas denitrificans ATCC 33889	E-value = 3.00E-56	Identity = 54.38%
	3		gi|152992735|ref|YP_001358456.1|	Sulfurovum sp. NBC37-1	E-value = 2.00E-48	Identity = 48.15%
	4		gi|34557290|ref|NP_907105.1|	Wolinella succinogenes DSM 1740	E-value = 9.00E-48	Identity = 51.16%
	5		gi|34558101|ref|NP_907916.1|	Wolinella succinogenes DSM 1740	E-value = 1.00E-43	Identity = 44.19%
AB0511	1		gi|152990277|ref|YP_001355999.1|	Nitratiruptor sp. SB155-2	E-value = 4.00E-95	Identity = 40.64%
	2		gi|152992734|ref|YP_001358455.1|	Sulfurovum sp. NBC37-1	E-value = 1.00E-91	Identity = 37.03%
AB0512	1		gi|152993380|ref|YP_001359101.1|	Sulfurovum sp. NBC37-1	E-value = 7.00E-32	Identity = 50.94%
	2		gi|83719940|ref|YP_441733.1|	Burkholderia thailandensis E264	E-value = 4.00E-28	Identity = 42.86%
	3		gi|37525215|ref|NP_928559.1|	Photorhabdus luminescens subsp. laumondii TTO1	E-value = 4.00E-28	Identity = 46.36%
	4		gi|53720570|ref|YP_109556.1|	Burkholderia pseudomallei K96243	E-value = 7.00E-28	Identity = 41.83%
	5		gi|154493701|ref|ZP_02033021.1|	Parabacteroides merdae ATCC 43184	E-value = 8.00E-28	Identity = 45.33%
AB0513	1	*mqo*	gi|154148988|ref|YP_001407157.1|	Campylobacter hominis ATCC BAA-381	E-value = 4.00E-166	Identity = 64.75%
	2		gi|118475354|ref|YP_891804.1|	Campylobacter fetus subsp. fetus 82-40	E-value = 2.00E-156	Identity = 62.78%
	3		gi|152992028|ref|YP_001357749.1|	Sulfurovum sp. NBC37-1	E-value = 3.00E-154	Identity = 60.75%
	4		gi|78777703|ref|YP_394018.1|	Sulfuromonas denitrificans ATCC 33889	E-value = 4.00E-152	Identity = 60.71%
	5		gi|157164110|ref|YP_001467278.1|	Campylobacter concisus 13826	E-value = 8.00E-143	Identity = 57.02%
AB0514	1	*ileS*	gi|152991025|ref|YP_001356747.1|	Nitratiruptor sp. SB155-2	E-value = 0	Identity = 66.99%
	2		gi|78776979|ref|YP_393294.1|	Sulfuromonas denitrificans ATCC 33889	E-value = 0	Identity = 62.89%
	3		gi|152993379|ref|YP_001359100.1|	Sulfurovum sp. NBC37-1	E-value = 0	Identity = 61.55%
	4		gi|118474979|ref|YP_891786.1|	Campylobacter fetus subsp. fetus 82-40	E-value = 0	Identity = 60.89%
	5		gi|154173836|ref|YP_001407768.1|	Campylobacter curvus 525.92	E-value = 0	Identity = 60.86%
AB0516	1		gi|152990000|ref|YP_001355722.1|	Nitratiruptor sp. SB155-2	E-value = 1.00E-24	Identity = 60.44%
	2		gi|78776531|ref|YP_392846.1|	Sulfuromonas denitrificans ATCC 33889	E-value = 7.00E-23	Identity = 53.26%
	3		gi|149195166|ref|ZP_01872257.1|	Caminibacter mediatlanticus TB-2	E-value = 1.00E-21	Identity = 53.85%
	4		gi|157165751|ref|YP_001467687.1|	Campylobacter concisus 13826	E-value = 1.00E-20	Identity = 51.09%
	5		gi|152993863|ref|YP_001359584.1|	Sulfurovum sp. NBC37-1	E-value = 2.00E-20	Identity = 48.91%
AB0517	1		gi|152993862|ref|YP_001359583.1|	Sulfurovum sp. NBC37-1	E-value = 1.00E-24	Identity = 37.89%
	2		gi|152990001|ref|YP_001355723.1|	Nitratiruptor sp. SB155-2	E-value = 3.00E-21	Identity = 33.70%
	3		gi|34558001|ref|NP_907816.1|	Wolinella succinogenes DSM 1740	E-value = 1.00E-20	Identity = 30.39%
	4		gi|78776532|ref|YP_392847.1|	Sulfuromonas denitrificans ATCC 33889	E-value = 1.00E-17	Identity = 32.93%
	5		gi|32267365|ref|NP_861397.1|	Helicobacter hepaticus ATCC 51449	E-value = 2.00E-16	Identity = 28.65%
AB0518	1	*murE*	gi|78776533|ref|YP_392848.1|	Sulfuromonas denitrificans ATCC 33889	E-value = 2.00E-117	Identity = 50.93%
	2		gi|34558000|ref|NP_907815.1|	Wolinella succinogenes DSM 1740	E-value = 2.00E-109	Identity = 48.43%
	3		gi|152993861|ref|YP_001359582.1|	Sulfurovum sp. NBC37-1	E-value = 2.00E-107	Identity = 46.76%
	4		gi|118475066|ref|YP_891273.1|	Campylobacter fetus subsp. fetus 82-40	E-value = 3.00E-102	Identity = 48.60%
	5		gi|152990002|ref|YP_001355724.1|	Nitratiruptor sp. SB155-2	E-value = 4.00E-102	Identity = 46.52%
AB0520	1		gi|78776966|ref|YP_393281.1|	Sulfuromonas denitrificans ATCC 33889	E-value = 1.00E-72	Identity = 58.47%
	2		gi|34557051|ref|NP_906866.1|	Wolinella succinogenes DSM 1740	E-value = 9.00E-68	Identity = 50.16%
	3		gi|89093161|ref|ZP_01166111.1|	Oceanospirillum sp. MED92	E-value = 1.00E-56	Identity = 42.63%
	4		gi|156741941|ref|YP_001432070.1|	Roseiflexus castenholzii DSM 13941	E-value = 2.00E-54	Identity = 42.01%

	5		gi|120553315|ref|YP_957666.1|	Marinobacter aquaeolei VT8	E-value = 5.00E-53	Identity = 40.00%
AB0521	1	*tas*	gi|152989892|ref|YP_001355614.1|	Nitratiruptor sp. SB155-2	E-value = 8.00E-148	Identity = 73.45%
	2		gi|149194528|ref|ZP_01871624.1|	Caminibacter mediatlanticus TB-2	E-value = 9.00E-144	Identity = 72.86%
	3		gi|78777477|ref|YP_393792.1|	Sulfuromonas denitrificans ATCC 33889	E-value = 2.00E-140	Identity = 70.21%
	4		gi|152992743|ref|YP_001358464.1|	Sulfurovum sp. NBC37-1	E-value = 2.00E-135	Identity = 66.28%
	5		gi|24216672|ref|NP_714153.1|	Leptospira interrogans serovar Lai str. 56601	E-value = 1.00E-111	Identity = 58.33%
AB0522	1	*pitA*	gi|34496389|ref|NP_900604.1|	Chromobacterium violaceum ATCC 12472	E-value = 8.00E-110	Identity = 44.03%
	2		gi|50119010|ref|YP_048177.1|	Erwinia carotovora subsp. atroseptica SCRI1043	E-value = 3.00E-108	Identity = 43.35%
	3		gi|77457489|ref|YP_346994.1|	Pseudomonas fluorescens PfO-1	E-value = 3.00E-104	Identity = 45.17%
	4		gi|71736800|ref|YP_276152.1|	Pseudomonas syringae pv. phaseolicola 1448A	E-value = 6.00E-104	Identity = 46.22%
	5		gi|104780367|ref|YP_606865.1|	Pseudomonas entomophila L48	E-value = 1.00E-103	Identity = 45.04%
AB0523	1		gi|78484423|ref|YP_390348.1|	Thiomicrospira crunogena XCL-2	E-value = 4.00E-77	Identity = 27.74%
	2		gi|78776470|ref|YP_392785.1|	Sulfuromonas denitrificans ATCC 33889	E-value = 4.00E-75	Identity = 31.67%
	3		gi|34557844|ref|NP_907659.1|	Wolinella succinogenes DSM 1740	E-value = 2.00E-42	Identity = 27.55%
AB0524	1		gi|152992332|ref|YP_001358053.1|	Sulfurovum sp. NBC37-1	E-value = 7.00E-42	Identity = 64.57%
AB0525	1		gi|86360197|ref|YP_472086.1|	Rhizobium etli CFN 42	E-value = 7.00E-69	Identity = 34.55%
	2		gi|78047258|ref|YP_363433.1|	Xanthomonas campestris pv. vesicatoria str. 85-10	E-value = 2.00E-68	Identity = 31.61%
	3		gi|34557253|ref|NP_907068.1|	Wolinella succinogenes DSM 1740	E-value = 9.00E-65	Identity = 32.91%
	4		gi|116254843|ref|YP_770679.1|	Rhizobium leguminosarum bv. viciae 3841	E-value = 3.00E-63	Identity = 34.54%
	5		gi|78047496|ref|YP_363671.1|	Xanthomonas campestris pv. vesicatoria str. 85-10	E-value = 8.00E-45	Identity = 25.89%
AB0526	1		gi|152990434|ref|YP_001356156.1|	Nitratiruptor sp. SB155-2	E-value = 6.00E-26	Identity = 51.67%
	2		gi|15792294|ref|NP_282117.1|	Campylobacter jejuni subsp. jejuni NCTC 11168	E-value = 6.00E-24	Identity = 47.93%
	3		gi|86150971|ref|ZP_01069187.1|	Campylobacter jejuni subsp. jejuni 260.94	E-value = 1.00E-23	Identity = 47.93%
	4		gi|153951051|ref|YP_001397943.1|	Campylobacter jejuni subsp. doylei 269.97	E-value = 2.00E-23	Identity = 46.28%
	5		gi|57242367|ref|ZP_00370306.1|	Campylobacter upsaliensis RM3195	E-value = 3.00E-23	Identity = 52.68%
AB0527	1		gi|32266204|ref|NP_860236.1|	Helicobacter hepaticus ATCC 51449	E-value = 6.00E-14	Identity = 32.48%
	2		gi|152991241|ref|YP_001356963.1|	Nitratiruptor sp. SB155-2	E-value = 1.00E-13	Identity = 30.43%
	3		gi|34556636|ref|NP_906451.1|	Wolinella succinogenes DSM 1740	E-value = 9.00E-12	Identity = 25.30%
	4		gi|149195095|ref|ZP_01872187.1|	Caminibacter mediatlanticus TB-2	E-value = 1.00E-11	Identity = 35.58%
	5		gi|109947539|ref|YP_664767.1|	Helicobacter acinonychis str. Sheeba	E-value = 1.00E-11	Identity = 28.40%
AB0528	1		gi|152990782|ref|YP_001356504.1|	Nitratiruptor sp. SB155-2	E-value = 8.00E-109	Identity = 51.65%
	2		gi|78777518|ref|YP_393833.1|	Sulfuromonas denitrificans ATCC 33889	E-value = 4.00E-104	Identity = 49.87%
	3		gi|152992200|ref|YP_001357921.1|	Sulfurovum sp. NBC37-1	E-value = 3.00E-100	Identity = 47.49%
	4		gi|32266848|ref|NP_860880.1|	Helicobacter hepaticus ATCC 51449	E-value = 3.00E-90	Identity = 47.65%
	5		gi|154174982|ref|YP_001408446.1|	Campylobacter curvus 525.92	E-value = 8.00E-90	Identity = 47.16%
AB0529	1		gi|77919222|ref|YP_357037.1|	Pelobacter carbinolicus DSM 2380	E-value = 2.00E-91	Identity = 50.28%
	2		gi|56460805|ref|YP_156086.1|	Idiomarina loihiensis L2TR	E-value = 4.00E-87	Identity = 49.58%
	3		gi|88796571|ref|ZP_01112235.1|	Alteromonas macleodii 'Deep ecotype'	E-value = 1.00E-86	Identity = 49.41%
	4		gi|85712482|ref|ZP_01043531.1|	Idiomarina baltica OS145	E-value = 2.00E-86	Identity = 49.00%
	5		gi|116220881|ref|ZP_01486301.1|	Vibrio cholerae V51	E-value = 4.00E-86	Identity = 44.65%
AB0530	1		gi|34556576|ref|NP_906391.1|	Wolinella succinogenes DSM 1740	E-value = 2.00E-53	Identity = 47.78%
	2		gi|24213214|ref|NP_710695.1|	Leptospira interrogans serovar Lai str. 56601	E-value = 2.00E-34	Identity = 33.92%
	3		gi|78778144|ref|YP_394459.1|	Sulfuromonas denitrificans ATCC 33889	E-value = 1.00E-32	Identity = 32.52%
	4		gi|110640174|ref|YP_680384.1|	Cytophaga hutchinsonii ATCC 33406	E-value = 2.00E-32	Identity = 32.87%
	5		gi|116330189|ref|YP_799907.1|	Leptospira borgpetersenii serovar Hardjo-bovis JB197	E-value = 2.00E-32	Identity = 33.10%
AB0531	1	*moeA2*	gi|152991708|ref|YP_001357429.1|	Sulfurovum sp. NBC37-1	E-value = 6.00E-116	Identity = 56.53%
	2		gi|78776456|ref|YP_392771.1|	Sulfuromonas denitrificans ATCC 33889	E-value = 9.00E-110	Identity = 52.61%
	3		gi|152991374|ref|YP_001357096.1|	Nitratiruptor sp. SB155-2	E-value = 7.00E-109	Identity = 51.75%
	4		gi|86152476|ref|ZP_01070681.1|	Campylobacter jejuni subsp. jejuni HB93-13	E-value = 3.00E-82	Identity = 43.78%

	5		gi|57238543|ref|YP_179674.1|	Campylobacter jejuni subsp. jejuni RM1221	E-value = 4.00E-82	Identity = 43.03%
AB0532	1			***** No matches found *****		
AB0533	1		gi|154173805|ref|YP_001408798.1|	Campylobacter curvus 525.92	E-value = 8.00E-94	Identity = 65.98%
	2		gi|157165487|ref|YP_001466356.1|	Campylobacter concisus 13826	E-value = 5.00E-92	Identity = 66.39%
	3		gi|118474467|ref|YP_892648.1|	Campylobacter fetus subsp. fetus 82-40	E-value = 4.00E-87	Identity = 62.70%
	4		gi|78777588|ref|YP_393903.1|	Sulfuromonas denitrificans ATCC 33889	E-value = 7.00E-85	Identity = 62.10%
	5		gi|57240739|ref|ZP_00368687.1|	Campylobacter lari RM2100	E-value = 2.00E-82	Identity = 59.51%
AB0534	1		gi|114045828|ref|YP_736378.1|	Shewanella sp. MR-7	E-value = 2.00E-30	Identity = 46.79%
	2		gi|146294725|ref|YP_001185149.1|	Shewanella putrefaciens CN-32	E-value = 5.00E-30	Identity = 46.58%
	3		gi|113971955|ref|YP_735748.1|	Shewanella sp. MR-4	E-value = 6.00E-30	Identity = 46.79%
	4		gi|126176231|ref|YP_001052380.1|	Shewanella baltica OS155	E-value = 7.00E-30	Identity = 47.20%
	5		gi|149114283|ref|ZP_01841037.1|	Shewanella baltica OS223	E-value = 8.00E-30	Identity = 48.72%
AB0535	1			***** No matches found *****		
AB0536	1		gi|34557209|ref|NP_907024.1|	Wolinella succinogenes DSM 1740	E-value = 8.00E-10	Identity = 32.61%
AB0537	1	*rpsB*	gi|34556640|ref|NP_906455.1|	Wolinella succinogenes DSM 1740	E-value = 3.00E-104	Identity = 75.69%
	2		gi|118474889|ref|YP_892581.1|	Campylobacter fetus subsp. fetus 82-40	E-value = 3.00E-103	Identity = 72.05%
	3		gi|157164805|ref|YP_001467386.1|	Campylobacter concisus 13826	E-value = 5.00E-102	Identity = 70.72%
	4		gi|154174229|ref|YP_001407659.1|	Campylobacter curvus 525.92	E-value = 9.00E-101	Identity = 70.54%
	5		gi|57168060|ref|ZP_00367199.1|	Campylobacter coli RM2228	E-value = 1.00E-100	Identity = 71.32%
AB0538	1	*tsf*	gi|57168059|ref|ZP_00367198.1|	Campylobacter coli RM2228	E-value = 2.00E-98	Identity = 58.54%
	2		gi|153952055|ref|YP_001397723.1|	Campylobacter jejuni subsp. doylei 269.97	E-value = 2.00E-97	Identity = 58.54%
	3		gi|57240504|ref|ZP_00368453.1|	Campylobacter lari RM2100	E-value = 2.00E-96	Identity = 56.98%
	4		gi|86150416|ref|ZP_01068642.1|	Campylobacter jejuni subsp. jejuni CF93-6	E-value = 3.00E-96	Identity = 58.26%
	5		gi|86152846|ref|ZP_01071051.1|	Campylobacter jejuni subsp. jejuni HB93-13	E-value = 3.00E-96	Identity = 58.26%
AB0539	1		gi|154173986|ref|YP_001407661.1|	Campylobacter curvus 525.92	E-value = 3.00E-70	Identity = 65.88%
	2		gi|157164016|ref|YP_001467384.1|	Campylobacter concisus 13826	E-value = 5.00E-69	Identity = 68.27%
	3		gi|118474231|ref|YP_892579.1|	Campylobacter fetus subsp. fetus 82-40	E-value = 3.00E-68	Identity = 62.98%
	4		gi|78777993|ref|YP_394308.1|	Sulfuromonas denitrificans ATCC 33889	E-value = 1.00E-65	Identity = 63.64%
	5		gi|154148688|ref|YP_001406402.1|	Campylobacter hominis ATCC BAA-381	E-value = 1.00E-63	Identity = 60.85%
AB0540	1			***** No matches found *****		
AB0541	1	*gmk*	gi|152993786|ref|YP_001359507.1|	Sulfurovum sp. NBC37-1	E-value = 2.00E-64	Identity = 62.00%
	2		gi|152991239|ref|YP_001356961.1|	Nitratiruptor sp. SB155-2	E-value = 1.00E-52	Identity = 52.24%
	3		gi|78777990|ref|YP_394305.1|	Sulfuromonas denitrificans ATCC 33889	E-value = 4.00E-52	Identity = 54.19%
	4		gi|154173927|ref|YP_001407665.1|	Campylobacter curvus 525.92	E-value = 2.00E-51	Identity = 51.74%
	5		gi|157164565|ref|YP_001467381.1|	Campylobacter concisus 13826	E-value = 6.00E-51	Identity = 52.24%
AB0542	1		gi|34557353|ref|NP_907168.1|	Wolinella succinogenes DSM 1740	E-value = 1.00E-61	Identity = 35.58%
	2		gi|78777182|ref|YP_393497.1|	Sulfuromonas denitrificans ATCC 33889	E-value = 4.00E-54	Identity = 29.66%
	3		gi|152990819|ref|YP_001356541.1|	Nitratiruptor sp. SB155-2	E-value = 7.00E-48	Identity = 29.40%
	4		gi|85711301|ref|ZP_01042360.1|	Idiomarina baltica OS145	E-value = 3.00E-46	Identity = 31.62%
	5		gi|56460645|ref|YP_155926.1|	Idiomarina loihiensis L2TR	E-value = 1.00E-45	Identity = 30.50%
AB0543	1		gi|152991194|ref|YP_001356916.1|	Nitratiruptor sp. SB155-2	E-value = 0	Identity = 50.86%
	2		gi|152993513|ref|YP_001359234.1|	Sulfurovum sp. NBC37-1	E-value = 0	Identity = 49.57%
	3		gi|78776775|ref|YP_393090.1|	Sulfuromonas denitrificans ATCC 33889	E-value = 0	Identity = 51.52%
	4		gi|34558447|ref|NP_908262.1|	Wolinella succinogenes DSM 1740	E-value = 0	Identity = 49.01%
	5		gi|118475762|ref|YP_892559.1|	Campylobacter fetus subsp. fetus 82-40	E-value = 0	Identity = 48.59%
AB0544	1		gi|15612155|ref|NP_223807.1|	Helicobacter pylori J99	E-value = 3.00E-06	Identity = 45.16%
	2		gi|109947840|ref|YP_665068.1|	Helicobacter acinonychis str. Sheeba	E-value = 4.00E-06	Identity = 44.62%
AB0545	1		gi|78776777|ref|YP_393092.1|	Sulfuromonas denitrificans ATCC 33889	E-value = 8.00E-10	Identity = 47.92%
	2		gi|120601385|ref|YP_965785.1|	Desulfovibrio vulgaris subsp. vulgaris DP4	E-value = 3.00E-07	Identity = 42.86%

	3		gi|32267016|ref|NP_861048.1|	Helicobacter hepaticus ATCC 51449	E-value = 4.00E-07	Identity = 46.51%
	4		gi|46581444|ref|YP_012252.1|	Desulfovibrio vulgaris subsp. vulgaris str. Hildenborough	E-value = 9.00E-07	Identity = 41.76%
	5		gi|1705528|sp|P00120|CY553_DESVM	Desulfovibrio vulgaris	E-value = 2.00E-06	Identity = 37.86%
AB0546	1		gi|152990613|ref|YP_001356335.1|	Nitratiruptor sp. SB155-2	E-value = 3.00E-57	Identity = 61.38%
	2		gi|78776685|ref|YP_393000.1|	Sulfuromonas denitrificans ATCC 33889	E-value = 7.00E-48	Identity = 52.91%
	3		gi|82523980|emb|CAI78791.1|	uncultured epsilon proteobacterium	E-value = 1.00E-44	Identity = 51.34%
	4		gi|121533977|ref|ZP_01665803.1|	Thermosinus carboxydivorans Nor1	E-value = 3.00E-25	Identity = 35.44%
	5		gi|77979678|ref|ZP_00835095.1|	Yersinia intermedia ATCC 29909	E-value = 8.00E-25	Identity = 33.01%
AB0547	1	*hemH*	gi|152990483|ref|YP_001356205.1|	Nitratiruptor sp. SB155-2	E-value = 3.00E-77	Identity = 49.84%
	2		gi|78777116|ref|YP_393431.1|	Sulfuromonas denitrificans ATCC 33889	E-value = 2.00E-76	Identity = 48.56%
	3		gi|34558436|ref|NP_908251.1|	Wolinella succinogenes DSM 1740	E-value = 3.00E-71	Identity = 47.13%
	4		gi|154148514|ref|YP_001406549.1|	Campylobacter hominis ATCC BAA-381	E-value = 2.00E-69	Identity = 49.52%
	5		gi|157164099|ref|YP_001467068.1|	Campylobacter concisus 13826	E-value = 4.00E-68	Identity = 48.69%
AB0548	1	*feoB1*	gi|152992399|ref|YP_001358120.1|	Sulfurovum sp. NBC37-1	E-value = 0	Identity = 63.75%
	2		gi|78777955|ref|YP_394270.1|	Sulfuromonas denitrificans ATCC 33889	E-value = 0	Identity = 61.35%
	3		gi|34557344|ref|NP_907159.1|	Wolinella succinogenes DSM 1740	E-value = 0	Identity = 58.91%
	4		gi|154149133|ref|YP_001406037.1|	Campylobacter hominis ATCC BAA-381	E-value = 0	Identity = 54.35%
	5		gi|57240816|ref|ZP_00368764.1|	Campylobacter lari RM2100	E-value = 0	Identity = 53.8%
AB0549	1			***** No matches found *****		
AB0550	1		gi|152992958|ref|YP_001358679.1|	Sulfurovum sp. NBC37-1	E-value = 2.00E-54	Identity = 46.00%
	2		gi|152993138|ref|YP_001358859.1|	Sulfurovum sp. NBC37-1	E-value = 4.00E-42	Identity = 38.31%
	3		gi|90579519|ref|ZP_01235328.1|	Vibrio angustum S14	E-value = 1.00E-41	Identity = 40.78%
	4		gi|149911645|ref|ZP_01900255.1|	Moritella sp. PE36	E-value = 3.00E-40	Identity = 40.00%
	5		gi|116190004|ref|ZP_01479747.1|	Vibrio cholerae MO10	E-value = 6.00E-40	Identity = 37.70%
AB0551	1		gi|152992655|ref|YP_001358376.1|	Sulfurovum sp. NBC37-1	E-value = 1.00E-75	Identity = 59.18%
	2		gi|34557677|ref|NP_907492.1|	Wolinella succinogenes DSM 1740	E-value = 5.00E-61	Identity = 50.00%
	3		gi|154174193|ref|YP_001407418.1|	Campylobacter curvus 525.92	E-value = 2.00E-59	Identity = 51.76%
	4		gi|154148800|ref|YP_001406105.1|	Campylobacter hominis ATCC BAA-381	E-value = 5.00E-59	Identity = 53.09%
	5		gi|157164968|ref|YP_001465982.1|	Campylobacter concisus 13826	E-value = 2.00E-56	Identity = 54.62%
AB0552	1		gi|152992654|ref|YP_001358375.1|	Sulfurovum sp. NBC37-1	E-value = 4.00E-61	Identity = 62.60%
	2		gi|118474931|ref|YP_892846.1|	Campylobacter fetus subsp. fetus 82-40	E-value = 5.00E-60	Identity = 58.27%
	3		gi|157164381|ref|YP_001465983.1|	Campylobacter concisus 13826	E-value = 1.00E-56	Identity = 59.52%
	4		gi|154174616|ref|YP_001407419.1|	Campylobacter curvus 525.92	E-value = 1.00E-56	Identity = 57.94%
	5		gi|116750161|ref|YP_846848.1|	Syntrophobacter fumaroxidans MPOB	E-value = 3.00E-53	Identity = 49.24%
AB0553	1		gi|78776513|ref|YP_392828.1|	Sulfuromonas denitrificans ATCC 33889	E-value = 6.00E-65	Identity = 51.08%
	2		gi|152993436|ref|YP_001359157.1|	Sulfurovum sp. NBC37-1	E-value = 7.00E-65	Identity = 49.10%
	3		gi|156862505|gb|EDO55936.1|	Bacteroides uniformis ATCC 8492	E-value = 9.00E-51	Identity = 42.55%
	4		gi|53711833|ref|YP_097825.1|	Bacteroides fragilis YCH46	E-value = 1.00E-48	Identity = 40.88%
	5		gi|29347915|ref|NP_811418.1|	Bacteroides thetaiotaomicron VPI-5482	E-value = 2.00E-48	Identity = 40.88%
AB0554	1	*tpx*	gi|34557922|ref|NP_907737.1|	Wolinella succinogenes DSM 1740	E-value = 2.00E-52	Identity = 63.64%
	2		gi|154174915|ref|YP_001409037.1|	Campylobacter curvus 525.92	E-value = 3.00E-51	Identity = 63.84%
	3		gi|149194723|ref|ZP_01871818.1|	Caminibacter mediatlanticus TB-2	E-value = 2.00E-46	Identity = 63.06%
	4		gi|118475243|ref|YP_892893.1|	Campylobacter fetus subsp. fetus 82-40	E-value = 2.00E-46	Identity = 58.18%
	5		gi|154148995|ref|YP_001407293.1|	Campylobacter hominis ATCC BAA-381	E-value = 4.00E-46	Identity = 59.52%
AB0555	1		gi|34556817|ref|NP_906632.1|	Wolinella succinogenes DSM 1740	E-value = 3.00E-33	Identity = 37.55%
	2		gi|108563461|ref|YP_627777.1|	Helicobacter pylori HPAG1	E-value = 2.00E-29	Identity = 35.71%
	3		gi|15612089|ref|NP_223741.1|	Helicobacter pylori J99	E-value = 2.00E-29	Identity = 35.71%
	4		gi|109947046|ref|YP_664274.1|	Helicobacter acinonychis str. Sheeba	E-value = 3.00E-29	Identity = 34.45%
	5		gi|15645712|ref|NP_207889.1|	Helicobacter pylori 26695	E-value = 2.00E-28	Identity = 34.45%

AB0556	1		gi|55977160|gb|AAV68375.1|	Herminiimonas arsenicoxydans	E-value = 9.00E-80	Identity = 55.56%
	2		gi|134094807|ref|YP_001099882.1|	Herminiimonas arsenicoxydans	E-value = 2.00E-78	Identity = 55.49%
	3		gi|134093767|ref|YP_001098842.1|	Herminiimonas arsenicoxydans	E-value = 1.00E-75	Identity = 52.94%
	4		gi|146293062|ref|YP_001183486.1|	Shewanella putrefaciens CN-32	E-value = 2.00E-75	Identity = 59.29%
	5		gi|58039157|ref|YP_191121.1|	Gluconobacter oxydans 621H	E-value = 1.00E-73	Identity = 49.21%
AB0558	1		gi|154174485|ref|YP_001409036.1|	Campylobacter curvus 525.92	E-value = 5.00E-67	Identity = 48.85%
	2		gi|152992825|ref|YP_001358546.1|	Sulfurovum sp. NBC37-1	E-value = 1.00E-42	Identity = 32.65%
	3		gi|152989874|ref|YP_001355596.1|	Nitratiruptor sp. SB155-2	E-value = 3.00E-30	Identity = 34.78%
AB0559	1		gi|34558055|ref|NP_907870.1|	Wolinella succinogenes DSM 1740	E-value = 6.00E-98	Identity = 57.05%
	2		gi|57242529|ref|ZP_00370467.1|	Campylobacter upsaliensis RM3195	E-value = 9.00E-91	Identity = 57.72%
	3		gi|57237434|ref|YP_178447.1|	Campylobacter jejuni subsp. jejuni RM1221	E-value = 2.00E-90	Identity = 57.05%
	4		gi|15791746|ref|NP_281569.1|	Campylobacter jejuni subsp. jejuni NCTC 11168	E-value = 6.00E-90	Identity = 56.71%
	5		gi|86153952|ref|ZP_01072155.1|	Campylobacter jejuni subsp. jejuni HB93-13	E-value = 7.00E-90	Identity = 56.71%
AB0560	1		gi|149910401|ref|ZP_01899043.1|	Moritella sp. PE36	E-value = 5.00E-16	Identity = 36.63%
	2		gi|87118570|ref|ZP_01074469.1|	Marinomonas sp. MED121	E-value = 1.00E-14	Identity = 29.79%
	3		gi|116626574|ref|YP_828730.1|	Solibacter usitatus Ellin6076	E-value = 4.00E-14	Identity = 28.00%
	4		gi|90413960|ref|ZP_01221945.1|	Photobacterium profundum 3TCK	E-value = 7.00E-14	Identity = 32.56%
	5		gi|157375119|ref|YP_001473719.1|	Shewanella sediminis HAW-EB3	E-value = 7.00E-14	Identity = 35.80%
AB0561	1	*rep*	gi|78776939|ref|YP_393254.1|	Sulfuromonas denitrificans ATCC 33889	E-value = 0	Identity = 59.88%
	2		gi|34557966|ref|NP_907781.1|	Wolinella succinogenes DSM 1740	E-value = 0	Identity = 57.96%
	3		gi|152990757|ref|YP_001356479.1|	Nitratiruptor sp. SB155-2	E-value = 0	Identity = 56.3%
	4		gi|154175325|ref|YP_001408248.1|	Campylobacter curvus 525.92	E-value = 0	Identity = 56.74%
	5		gi|157163928|ref|YP_001466843.1|	Campylobacter concisus 13826	E-value = 0	Identity = 55.6%
AB0562	1		gi|34557699|ref|NP_907514.1|	Wolinella succinogenes DSM 1740	E-value = 1.00E-66	Identity = 42.52%
	2		gi|78778138|ref|YP_394453.1|	Sulfuromonas denitrificans ATCC 33889	E-value = 4.00E-63	Identity = 42.78%
	3		gi|152991395|ref|YP_001357117.1|	Nitratiruptor sp. SB155-2	E-value = 2.00E-57	Identity = 41.58%
	4		gi|154175252|ref|YP_001408977.1|	Campylobacter curvus 525.92	E-value = 2.00E-56	Identity = 37.53%
	5		gi|78776369|ref|YP_392684.1|	Sulfuromonas denitrificans ATCC 33889	E-value = 1.00E-55	Identity = 41.10%
AB0563	1	*soxC*	gi|78778254|ref|YP_394569.1|	Sulfuromonas denitrificans ATCC 33889	E-value = 2.00E-175	Identity = 68.32%
	2		gi|152991645|ref|YP_001357366.1|	Sulfurovum sp. NBC37-1	E-value = 7.00E-167	Identity = 66.21%
	3		gi|89094214|ref|ZP_01167156.1|	Oceanospirillum sp. MED92	E-value = 8.00E-119	Identity = 51.62%
	4		gi|88704850|ref|ZP_01102563.1|	gamma proteobacterium KT 71	E-value = 8.00E-112	Identity = 52.51%
	5		gi|149120191|ref|ZP_01846649.1|	Methylobacterium sp. 4-46	E-value = 8.00E-110	Identity = 50.00%
AB0564	1	*soxD*	gi|152991646|ref|YP_001357367.1|	Sulfurovum sp. NBC37-1	E-value = 5.00E-86	Identity = 47.12%
	2		gi|78778253|ref|YP_394568.1|	Sulfuromonas denitrificans ATCC 33889	E-value = 3.00E-80	Identity = 44.65%
	3		gi|89094213|ref|ZP_01167155.1|	Oceanospirillum sp. MED92	E-value = 4.00E-47	Identity = 34.42%
	4		gi|88704849|ref|ZP_01102562.1|	gamma proteobacterium KT 71	E-value = 1.00E-42	Identity = 34.62%
	5		gi|83943683|ref|ZP_00956141.1|	Sulfitobacter sp. EE-36	E-value = 2.00E-27	Identity = 31.46%
AB0565	1	*soxX*	gi|78484949|ref|YP_390874.1|	Thiomicrospira crunogena XCL-2	E-value = 3.00E-12	Identity = 36.36%
	2		gi|146343608|ref|YP_001208656.1|	Bradyrhizobium sp. ORS278	E-value = 1.00E-10	Identity = 35.58%
	3		gi|145218965|ref|YP_001129674.1|	Prosthecochloris vibrioformis DSM 265	E-value = 1.00E-10	Identity = 35.83%
	4		gi|21673843|ref|NP_661908.1|	Chlorobium tepidum TLS	E-value = 4.00E-10	Identity = 40.17%
	5		gi|149120189|ref|ZP_01846647.1|	Methylobacterium sp. 4-46	E-value = 2.00E-09	Identity = 37.14%
AB0566	1	*soxY*	gi|152991647|ref|YP_001357368.1|	Sulfurovum sp. NBC37-1	E-value = 1.00E-31	Identity = 50.98%
	2		gi|78778252|ref|YP_394567.1|	Sulfuromonas denitrificans ATCC 33889	E-value = 1.00E-31	Identity = 52.67%
	3		gi|21673844|ref|NP_661909.1|	Chlorobium tepidum TLS	E-value = 6.00E-21	Identity = 43.23%
	4		gi|27376124|ref|NP_767653.1|	Bradyrhizobium japonicum USDA 110	E-value = 1.00E-19	Identity = 43.66%
	5		gi|149120188|ref|ZP_01846646.1|	Methylobacterium sp. 4-46	E-value = 1.00E-18	Identity = 44.52%
AB0567	1	*soxZ*	gi|78778251|ref|YP_394566.1|	Sulfuromonas denitrificans ATCC 33889	E-value = 7.00E-14	Identity = 52.08%

	2		gi|153873204|ref|ZP_02001863.1|	Beggiatoa sp. PS	E-value = 1.00E-12	Identity = 45.54%
	3		gi|71908744|ref|YP_286331.1|	Dechloromonas aromatica RCB	E-value = 6.00E-11	Identity = 45.26%
	4		gi|152991648|ref|YP_001357369.1|	Sulfurovum sp. NBC37-1	E-value = 6.00E-11	Identity = 47.67%
	5		gi|21673845|ref|NP_661910.1|	Chlorobium tepidum TLS	E-value = 9.00E-11	Identity = 40.21%
AB0568	1	*soxA*	gi|78189878|ref|YP_380216.1|	Chlorobium chlorochromatii CaD3	E-value = 4.00E-54	Identity = 45.78%
	2		gi|21673846|ref|NP_661911.1|	Chlorobium tepidum TLS	E-value = 2.00E-53	Identity = 39.00%
	3		gi|145218962|ref|YP_001129671.1|	Prosthecochloris vibrioformis DSM 265	E-value = 6.00E-52	Identity = 38.91%
	4		gi|19879578|gb|AAL68886.1|	Chlorobium limicola	E-value = 1.00E-51	Identity = 42.80%
	5		gi|68549173|ref|ZP_00588640.1|	Pelodictyon phaeoclathratiforme BU-1	E-value = 8.00E-51	Identity = 39.37%
AB0569	1			***** No matches found *****		
AB0570	1	*soxB*	gi|21673848|ref|NP_661913.1|	Chlorobium tepidum TLS	E-value = 0	Identity = 57.53%
	2		gi|153871310|ref|ZP_02000515.1|	Beggiatoa sp. PS	E-value = 0	Identity = 55.65%
	3		gi|145218960|ref|YP_001129669.1|	Prosthecochloris vibrioformis DSM 265	E-value = 0	Identity = 56.68%
	4		gi|68549175|ref|ZP_00588642.1|	Pelodictyon phaeoclathratiforme BU-1	E-value = 0	Identity = 56.29%
	5		gi|19879580|gb|AAL68888.1|	Chlorobium limicola	E-value = 0	Identity = 56.34%
AB0571	1		gi|152992100|ref|YP_001357821.1|	Sulfurovum sp. NBC37-1	E-value = 5.00E-37	Identity = 35.77%
	2		gi|152991565|ref|YP_001357287.1|	Nitratiruptor sp. SB155-2	E-value = 8.00E-35	Identity = 33.97%
	3		gi|152992101|ref|YP_001357822.1|	Sulfurovum sp. NBC37-1	E-value = 7.00E-32	Identity = 34.21%
	4		gi|152993435|ref|YP_001359156.1|	Sulfurovum sp. NBC37-1	E-value = 1.00E-25	Identity = 33.08%
AB0572	1		gi|55981296|ref|YP_144593.1|	Thermus thermophilus HB8	E-value = 8.00E-26	Identity = 43.33%
	2		gi|46199265|ref|YP_004932.1|	Thermus thermophilus HB27	E-value = 3.00E-25	Identity = 41.80%
	3		gi|78778255|ref|YP_394570.1|	Sulfuromonas denitrificans ATCC 33889	E-value = 9.00E-21	Identity = 40.35%
	4		gi|153811137|ref|ZP_01963805.1|	Ruminococcus obeum ATCC 29174	E-value = 1.00E-09	Identity = 29.01%
	5		gi|154504510|ref|ZP_02041248.1|	Ruminococcus gnavus ATCC 29149	E-value = 5.00E-09	Identity = 30.15%
AB0573	1		gi|152992102|ref|YP_001357823.1|	Sulfurovum sp. NBC37-1	E-value = 4.00E-29	Identity = 43.36%
	2		gi|152991564|ref|YP_001357286.1|	Nitratiruptor sp. SB155-2	E-value = 8.00E-27	Identity = 43.17%
	3		gi|144899374|emb|CAM76238.1|	Magnetospirillum gryphiswaldense MSR-1	E-value = 7.00E-23	Identity = 42.74%
	4		gi|78776468|ref|YP_392783.1|	Sulfuromonas denitrificans ATCC 33889	E-value = 4.00E-18	Identity = 36.69%
	5		gi|78484892|ref|YP_390817.1|	Thiomicrospira crunogena XCL-2	E-value = 5.00E-09	Identity = 28.57%
AB0574	1			***** No matches found *****		
AB0576	1	*pfpI*	gi|94309255|ref|YP_582465.1|	Ralstonia metallidurans CH34	E-value = 6.00E-74	Identity = 67.20%
	2		gi|73540068|ref|YP_294588.1|	Ralstonia eutropha JMP134	E-value = 1.00E-73	Identity = 67.20%
	3		gi|118038911|ref|ZP_01510314.1|	Burkholderia phytofirmans PsJN	E-value = 4.00E-71	Identity = 66.49%
	4		gi|119510731|ref|ZP_01629858.1|	Nodularia spumigena CCY9414	E-value = 4.00E-71	Identity = 62.70%
	5		gi|120555856|ref|YP_960207.1|	Marinobacter aquaeolei VT8	E-value = 8.00E-71	Identity = 65.41%
AB0578	1		gi|152991649|ref|YP_001357370.1|	Sulfurovum sp. NBC37-1	E-value = 3.00E-77	Identity = 48.99%
	2		gi|78778250|ref|YP_394565.1|	Sulfuromonas denitrificans ATCC 33889	E-value = 6.00E-76	Identity = 51.76%
	3		gi|152992099|ref|YP_001357820.1|	Sulfurovum sp. NBC37-1	E-value = 3.00E-70	Identity = 45.72%
	4		gi|152990188|ref|YP_001355910.1|	Nitratiruptor sp. SB155-2	E-value = 6.00E-45	Identity = 34.45%
	5		gi|68549368|ref|ZP_00588833.1|	Pelodictyon phaeoclathratiforme BU-1	E-value = 3.00E-23	Identity = 28.43%
AB0579	1		gi|150017891|ref|YP_001310145.1|	Clostridium beijerinckii NCIMB 8052	E-value = 5.00E-37	Identity = 29.78%
	2		gi|15895797|ref|NP_349146.1|	Clostridium acetobutylicum ATCC 824	E-value = 3.00E-35	Identity = 28.47%
	3		gi|118444989|ref|YP_878971.1|	Clostridium novyi NT	E-value = 5.00E-21	Identity = 28.61%
	4		gi|20806580|ref|NP_621751.1|	Thermoanaerobacter tengcongensis MB4	E-value = 6.00E-19	Identity = 25.63%
	5		gi|78043440|ref|YP_359007.1|	Carboxydothermus hydrogenoformans Z-2901	E-value = 1.00E-18	Identity = 25.64%
AB0580	1		gi|152992706|ref|YP_001358427.1|	Sulfurovum sp. NBC37-1	E-value = 5.00E-50	Identity = 40.53%
	2		gi|152992760|ref|YP_001358481.1|	Sulfurovum sp. NBC37-1	E-value = 1.00E-47	Identity = 45.70%
AB0581	1	*valS*	gi|78776936|ref|YP_393251.1|	Sulfuromonas denitrificans ATCC 33889	E-value = 0	Identity = 70.22%
	2		gi|152992964|ref|YP_001358685.1|	Sulfurovum sp. NBC37-1	E-value = 0	Identity = 67.59%

	3		gi|152990758|ref|YP_001356480.1|	Nitratiruptor sp. SB155-2	E-value = 0	Identity = 66.36%
	4		gi|157164498|ref|YP_001466839.1|	Campylobacter concisus 13826	E-value = 0	Identity = 62.38%
	5		gi|154174900|ref|YP_001408244.1|	Campylobacter curvus 525.92	E-value = 0	Identity = 64.9%
AB0582	1			***** No matches found *****		
AB0583	1		gi|150400259|ref|YP_001324026.1|	Methanococcus vannielii SB	E-value = 1.00E-15	Identity = 77.78%
	2		gi|157400869|gb|EDO67132.1|	Methanococcus maripaludis C6	E-value = 2.00E-15	Identity = 77.78%
	3		gi|89205320|ref|ZP_01183891.1|	Bacillus weihenstephanensis KBAB4	E-value = 3.00E-15	Identity = 76.19%
	4		gi|30022616|ref|NP_834247.1|	Bacillus cereus ATCC 14579	E-value = 4.00E-15	Identity = 76.19%
	5		gi|45358234|ref|NP_987791.1|	Methanococcus maripaludis S2	E-value = 5.00E-15	Identity = 76.19%
AB0584	1			***** No matches found *****		
AB0585	1		gi|152991372|ref|YP_001357094.1|	Nitratiruptor sp. SB155-2	E-value = 3.00E-113	Identity = 50.68%
	2		gi|34558097|ref|NP_907912.1|	Wolinella succinogenes DSM 1740	E-value = 2.00E-110	Identity = 51.60%
	3		gi|78776271|ref|YP_392586.1|	Sulfuromonas denitrificans ATCC 33889	E-value = 3.00E-107	Identity = 52.29%
	4		gi|118474240|ref|YP_891389.1|	Campylobacter fetus subsp. fetus 82-40	E-value = 1.00E-103	Identity = 49.54%
	5		gi|32267113|ref|NP_861145.1|	Helicobacter hepaticus ATCC 51449	E-value = 6.00E-103	Identity = 47.06%
AB0586	1		gi|152991764|ref|YP_001357485.1|	Sulfurovum sp. NBC37-1	E-value = 2.00E-53	Identity = 56.78%
	2		gi|78776272|ref|YP_392587.1|	Sulfuromonas denitrificans ATCC 33889	E-value = 2.00E-50	Identity = 55.28%
	3		gi|152991371|ref|YP_001357093.1|	Nitratiruptor sp. SB155-2	E-value = 3.00E-50	Identity = 59.18%
	4		gi|34558098|ref|NP_907913.1|	Wolinella succinogenes DSM 1740	E-value = 5.00E-50	Identity = 48.47%
	5		gi|149195344|ref|ZP_01872431.1|	Caminibacter mediatlanticus TB-2	E-value = 9.00E-50	Identity = 54.82%
AB0587	1			***** No matches found *****		
AB0588	1			***** No matches found *****		
AB0589	1			***** No matches found *****		
AB0590	1	*proC*	gi|152993344|ref|YP_001359065.1|	Sulfurovum sp. NBC37-1	E-value = 1.00E-60	Identity = 50.60%
	2		gi|78776990|ref|YP_393305.1|	Sulfuromonas denitrificans ATCC 33889	E-value = 2.00E-59	Identity = 50.20%
	3		gi|152991093|ref|YP_001356815.1|	Nitratiruptor sp. SB155-2	E-value = 1.00E-51	Identity = 49.80%
	4		gi|149193816|ref|ZP_01870914.1|	Caminibacter mediatlanticus TB-2	E-value = 3.00E-49	Identity = 45.42%
	5		gi|34558263|ref|NP_908078.1|	Wolinella succinogenes DSM 1740	E-value = 5.00E-49	Identity = 46.22%
AB0591	1		gi|152991092|ref|YP_001356814.1|	Nitratiruptor sp. SB155-2	E-value = 2.00E-43	Identity = 42.79%
	2		gi|152993490|ref|YP_001359211.1|	Sulfurovum sp. NBC37-1	E-value = 2.00E-42	Identity = 43.87%
	3		gi|34558261|ref|NP_908076.1|	Wolinella succinogenes DSM 1740	E-value = 4.00E-40	Identity = 41.71%
	4		gi|109946693|ref|YP_663921.1|	Helicobacter acinonychis str. Sheeba	E-value = 8.00E-39	Identity = 37.32%
	5		gi|15645988|ref|NP_208169.1|	Helicobacter pylori 26695	E-value = 3.00E-37	Identity = 36.92%
AB0592	1	*lon*	gi|152991091|ref|YP_001356813.1|	Nitratiruptor sp. SB155-2	E-value = 0	Identity = 62.11%
	2		gi|34558260|ref|NP_908075.1|	Wolinella succinogenes DSM 1740	E-value = 0	Identity = 61.49%
	3		gi|157164570|ref|YP_001467201.1|	Campylobacter concisus 13826	E-value = 0	Identity = 60.5%
	4		gi|78776615|ref|YP_392930.1|	Sulfuromonas denitrificans ATCC 33889	E-value = 0	Identity = 60.62%
	5		gi|154173725|ref|YP_001407992.1|	Campylobacter curvus 525.92	E-value = 0	Identity = 59.5%
AB0593	1		gi|154147965|ref|YP_001406479.1|	Campylobacter hominis ATCC BAA-381	E-value = 2.00E-25	Identity = 46.47%
	2		gi|118474503|ref|YP_892056.1|	Campylobacter fetus subsp. fetus 82-40	E-value = 2.00E-24	Identity = 47.65%
	3		gi|154174587|ref|YP_001408255.1|	Campylobacter curvus 525.92	E-value = 6.00E-24	Identity = 52.00%
	4		gi|157165578|ref|YP_001466851.1|	Campylobacter concisus 13826	E-value = 3.00E-22	Identity = 42.26%
	5		gi|57168869|ref|ZP_00367999.1|	Campylobacter coli RM2228	E-value = 2.00E-20	Identity = 46.67%
AB0594	1		gi|34557684|ref|NP_907499.1|	Wolinella succinogenes DSM 1740	E-value = 8.00E-40	Identity = 53.10%
	2		gi|32267262|ref|NP_861294.1|	Helicobacter hepaticus ATCC 51449	E-value = 6.00E-34	Identity = 51.05%
	3		gi|86132703|ref|ZP_01051295.1|	Cellulophaga sp. MED134	E-value = 4.00E-27	Identity = 46.05%
	4		gi|118476160|ref|YP_893311.1|	Bacillus thuringiensis str. Al Hakam	E-value = 5.00E-27	Identity = 46.21%
	5		gi|52144839|ref|YP_081990.1|	Bacillus cereus E33L	E-value = 6.00E-27	Identity = 46.21%
AB0595	1		gi|68550325|ref|ZP_00589776.1|	Pelodictyon phaeoclathratiforme BU-1	E-value = 2.00E-66	Identity = 37.58%

	2		gi|78188842|ref|YP_379180.1|	Chlorobium chlorochromatii CaD3	E-value = 5.00E-63	Identity = 35.59%
	3		gi|23128792|ref|ZP_00110631.1|	Nostoc punctiforme PCC 73102	E-value = 2.00E-17	Identity = 25.77%
	4		gi|113477368|ref|YP_723429.1|	Trichodesmium erythraeum IMS101	E-value = 7.00E-14	Identity = 25.74%
	5		gi|52548589|gb|AAU82438.1|	uncultured archaeon GZfos17F1	E-value = 5.00E-07	Identity = 25.07%
AB0596	1			***** No matches found *****		
AB0597	1		gi|91223048|ref|ZP_01258314.1|	Vibrio alginolyticus 12G01	E-value = 4.00E-27	Identity = 47.69%
	2		gi|91794509|ref|YP_564160.1|	Shewanella denitrificans OS217	E-value = 8.00E-27	Identity = 44.88%
	3		gi|153832557|ref|ZP_01985224.1|	Vibrio harveyi HY01	E-value = 1.00E-26	Identity = 46.92%
	4		gi|117619751|ref|YP_858079.1|	Aeromonas hydrophila subsp. hydrophila ATCC 7966	E-value = 2.00E-26	Identity = 42.03%
	5		gi|116187079|ref|ZP_01476969.1|	Vibrio sp. Ex25	E-value = 2.00E-26	Identity = 47.69%
AB0598	1		gi|84358333|ref|ZP_00983116.1|	Burkholderia dolosa AUO158	E-value = 5.00E-09	Identity = 32.26%
AB0599	1		gi|152993881|ref|YP_001359602.1|	Sulfurovum sp. NBC37-1	E-value = 6.00E-09	Identity = 47.22%
AB0602	1		gi|87311198|ref|ZP_01093321.1|	Blastopirellula marina DSM 3645	E-value = 2.00E-97	Identity = 41.53%
	2		gi|118592320|ref|ZP_01549712.1|	Stappia aggregata IAM 12614	E-value = 5.00E-93	Identity = 38.46%
	3		gi|85713882|ref|ZP_01044871.1|	Nitrobacter sp. Nb-311A	E-value = 2.00E-89	Identity = 39.03%
	4		gi|91974708|ref|YP_567367.1|	Rhodopseudomonas palustris BisB5	E-value = 8.00E-89	Identity = 42.26%
	5		gi|39933508|ref|NP_945784.1|	Rhodopseudomonas palustris CGA009	E-value = 9.00E-89	Identity = 42.48%
AB0603	1		gi|88859208|ref|ZP_01133848.1|	Pseudoalteromonas tunicata D2	E-value = 4.00E-06	Identity = 45.95%
	2		gi|157165452|ref|YP_001467803.1|	Campylobacter concisus 13826	E-value = 9.00E-06	Identity = 47.76%
AB0604	1		gi|85703820|ref|ZP_01034923.1|	Roseovarius sp. 217	E-value = 2.00E-33	Identity = 32.71%
	2		gi|127514037|ref|YP_001095234.1|	Shewanella loihica PV-4	E-value = 5.00E-33	Identity = 33.57%
	3		gi|149203310|ref|ZP_01880280.1|	Roseovarius sp. TM1035	E-value = 2.00E-32	Identity = 33.45%
	4		gi|99080441|ref|YP_612595.1|	Silicibacter sp. TM1040	E-value = 1.00E-31	Identity = 31.02%
	5		gi|28871406|ref|NP_794025.1|	Pseudomonas syringae pv. tomato str. DC3000	E-value = 3.00E-31	Identity = 34.52%
AB0605	1	*accC2*	gi|78776821|ref|YP_393136.1|	Sulfuromonas denitrificans ATCC 33889	E-value = 0	Identity = 77.80%
	2		gi|149194561|ref|ZP_01871657.1|	Caminibacter mediatlanticus TB-2	E-value = 0	Identity = 66.67%
	3		gi|15606634|ref|NP_214014.1|	Aquifex aeolicus VF5	E-value = 3.00E-161	Identity = 60.27%
	4		gi|156719254|ref|ZP_02060897.1|	Hydrogenobaculum sp. Y04AAS1	E-value = 7.00E-158	Identity = 60.40%
	5		gi|116234998|dbj|BAF34937.1|	Hydrogenobacter thermophilus	E-value = 8.00E-154	Identity = 56.71%
AB0606	1		gi|150009188|ref|YP_001303931.1|	Parabacteroides distasonis ATCC 8503	E-value = 4.00E-19	Identity = 56.12%
	2		gi|60680005|ref|YP_210149.1|	Bacteroides fragilis NCTC 9343	E-value = 2.00E-18	Identity = 51.52%
	3		gi|85714910|ref|ZP_01045896.1|	Nitrobacter sp. Nb-311A	E-value = 2.00E-18	Identity = 48.08%
	4		gi|154494084|ref|ZP_02033404.1|	Parabacteroides merdae ATCC 43184	E-value = 2.00E-18	Identity = 56.12%
	5		gi|156862636|gb|EDO56067.1|	Bacteroides uniformis ATCC 8492	E-value = 3.00E-18	Identity = 50.51%
AB0607	1	*sufE*	gi|150010126|ref|YP_001304869.1|	Parabacteroides distasonis ATCC 8503	E-value = 8.00E-24	Identity = 40.15%
	2		gi|110637384|ref|YP_677591.1|	Cytophaga hutchinsonii ATCC 33406	E-value = 2.00E-23	Identity = 42.54%
	3		gi|150026417|ref|YP_001297243.1|	Flavobacterium psychrophilum JIP02/86	E-value = 3.00E-23	Identity = 43.94%
	4		gi|120437452|ref|YP_863138.1|	Gramella forsetii KT0803	E-value = 5.00E-23	Identity = 41.67%
	5		gi|88804367|ref|ZP_01119887.1|	Robiginitalea biformata HTCC2501	E-value = 6.00E-23	Identity = 39.39%
AB0608	1	*sufS*	gi|88799897|ref|ZP_01115469.1|	Reinekea sp. MED297	E-value = 5.00E-88	Identity = 43.51%
	2		gi|116695456|ref|YP_841032.1|	Ralstonia eutropha H16	E-value = 2.00E-87	Identity = 41.12%
	3		gi|88804366|ref|ZP_01119886.1|	Robiginitalea biformata HTCC2501	E-value = 3.00E-87	Identity = 42.32%
	4		gi|90021064|ref|YP_526891.1|	Saccharophagus degradans 2-40	E-value = 3.00E-87	Identity = 43.50%
	5		gi|116626730|ref|YP_828886.1|	Solibacter usitatus Ellin6076	E-value = 3.00E-87	Identity = 41.37%
AB0609	1	*sufD*	gi|152992590|ref|YP_001358311.1|	Sulfurovum sp. NBC37-1	E-value = 8.00E-27	Identity = 27.32%
	2		gi|90407346|ref|ZP_01215531.1|	Psychromonas sp. CNPT3	E-value = 1.00E-13	Identity = 27.60%
	3		gi|89256547|ref|YP_513909.1|	Francisella tularensis subsp. holarctica	E-value = 3.00E-12	Identity = 25.17%
	4		gi|134253681|gb|EBA52775.1|	Francisella tularensis subsp. holarctica 257	E-value = 3.00E-12	Identity = 25.17%
AB0610	1	*sufC*	gi|152992591|ref|YP_001358312.1|	Sulfurovum sp. NBC37-1	E-value = 6.00E-70	Identity = 55.14%

	2		gi|114776743|ref|ZP_01451786.1|	Mariprofundus ferrooxydans PV-1	E-value = 7.00E-70	Identity = 53.31%
	3		gi|116626728|ref|YP_828884.1|	Solibacter usitatus Ellin6076	E-value = 7.00E-69	Identity = 51.81%
	4		gi|146329287|ref|YP_001209735.1|	Dichelobacter nodosus VCS1703A	E-value = 2.00E-68	Identity = 51.19%
	5		gi|50120791|ref|YP_049958.1|	Erwinia carotovora subsp. atroseptica SCRI1043	E-value = 3.00E-68	Identity = 52.28%
AB0611	1	*sufB*	gi|152990761|ref|YP_001356483.1|	Nitratiruptor sp. SB155-2	E-value = 0	Identity = 72.36%
	2		gi|120556061|ref|YP_960412.1|	Marinobacter aquaeolei VT8	E-value = 0	Identity = 70.63%
	3		gi|126665742|ref|ZP_01736723.1|	Marinobacter sp. ELB17	E-value = 0	Identity = 70%
	4		gi|152992592|ref|YP_001358313.1|	Sulfurovum sp. NBC37-1	E-value = 0	Identity = 73.32%
	5		gi|149376032|ref|ZP_01893798.1|	Marinobacter algicola DG893	E-value = 0	Identity = 70.63%
AB0612	1	*iscS*	gi|34558481|ref|NP_908296.1|	Wolinella succinogenes DSM 1740	E-value = 4.00E-153	Identity = 66.67%
	2		gi|118475402|ref|YP_892816.1|	Campylobacter fetus subsp. fetus 82-40	E-value = 1.00E-151	Identity = 66.83%
	3		gi|32266063|ref|NP_860095.1|	Helicobacter hepaticus ATCC 51449	E-value = 9.00E-149	Identity = 64.77%
	4		gi|157164887|ref|YP_001466052.1|	Campylobacter concisus 13826	E-value = 1.00E-148	Identity = 63.57%
	5		gi|154173772|ref|YP_001407486.1|	Campylobacter curvus 525.92	E-value = 2.00E-148	Identity = 63.82%
AB0613	1	*iscU*	gi|157164931|ref|YP_001466051.1|	Campylobacter concisus 13826	E-value = 8.00E-137	Identity = 73.33%
	2		gi|154173662|ref|YP_001407485.1|	Campylobacter curvus 525.92	E-value = 1.00E-134	Identity = 73.64%
	3		gi|152991603|ref|YP_001357324.1|	Sulfurovum sp. NBC37-1	E-value = 5.00E-134	Identity = 74.39%
	4		gi|34558480|ref|NP_908295.1|	Wolinella succinogenes DSM 1740	E-value = 1.00E-133	Identity = 70.34%
	5		gi|154148924|ref|YP_001405665.1|	Campylobacter hominis ATCC BAA-381	E-value = 1.00E-132	Identity = 70.91%
AB0614	1		gi|89900422|ref|YP_522893.1|	Rhodoferax ferrireducens T118	E-value = 3.00E-14	Identity = 27.38%
AB0615	1			***** No matches found *****		
AB0616	1	*argH*	gi|152990406|ref|YP_001356128.1|	Nitratiruptor sp. SB155-2	E-value = 4.00E-167	Identity = 67.04%
	2		gi|78777064|ref|YP_393379.1|	Sulfuromonas denitrificans ATCC 33889	E-value = 4.00E-164	Identity = 65.11%
	3		gi|149194544|ref|ZP_01871640.1|	Caminibacter mediatlanticus TB-2	E-value = 1.00E-159	Identity = 64.96%
	4		gi|34557584|ref|NP_907399.1|	Wolinella succinogenes DSM 1740	E-value = 9.00E-158	Identity = 62.53%
	5		gi|152992140|ref|YP_001357861.1|	Sulfurovum sp. NBC37-1	E-value = 3.00E-154	Identity = 61.32%
AB0617	1	*cheV*	gi|34558164|ref|NP_907979.1|	Wolinella succinogenes DSM 1740	E-value = 8.00E-34	Identity = 28.57%
	2		gi|109947387|ref|YP_664615.1|	Helicobacter acinonychis str. Sheeba	E-value = 5.00E-27	Identity = 29.49%
	3		gi|108563022|ref|YP_627338.1|	Helicobacter pylori HPAG1	E-value = 7.00E-26	Identity = 27.88%
	4		gi|32267301|ref|NP_861333.1|	Helicobacter hepaticus ATCC 51449	E-value = 8.00E-26	Identity = 26.98%
	5		gi|15645241|ref|NP_207411.1|	Helicobacter pylori 26695	E-value = 3.00E-25	Identity = 27.24%
AB0618	1		gi|152991062|ref|YP_001356784.1|	Nitratiruptor sp. SB155-2	E-value = 6.00E-55	Identity = 50.23%
	2		gi|152992896|ref|YP_001358617.1|	Sulfurovum sp. NBC37-1	E-value = 5.00E-46	Identity = 44.08%
	3		gi|157164942|ref|YP_001466446.1|	Campylobacter concisus 13826	E-value = 1.00E-45	Identity = 44.55%
	4		gi|149193774|ref|ZP_01870872.1|	Caminibacter mediatlanticus TB-2	E-value = 7.00E-44	Identity = 48.82%
	5		gi|118474392|ref|YP_891526.1|	Campylobacter fetus subsp. fetus 82-40	E-value = 9.00E-44	Identity = 45.45%
AB0619	1	*greA*	gi|108563274|ref|YP_627590.1|	Helicobacter pylori HPAG1	E-value = 5.00E-44	Identity = 55.97%
	2		gi|15611867|ref|NP_223518.1|	Helicobacter pylori J99	E-value = 5.00E-44	Identity = 55.97%
	3		gi|109947754|ref|YP_664982.1|	Helicobacter acinonychis str. Sheeba	E-value = 1.00E-43	Identity = 56.60%
	4		gi|149194430|ref|ZP_01871527.1|	Caminibacter mediatlanticus TB-2	E-value = 2.00E-42	Identity = 57.32%
	5		gi|32265653|ref|NP_859685.1|	Helicobacter hepaticus ATCC 51449	E-value = 2.00E-42	Identity = 54.72%
AB0620	1		gi|152991054|ref|YP_001356776.1|	Nitratiruptor sp. SB155-2	E-value = 1.00E-42	Identity = 43.44%
	2		gi|152992889|ref|YP_001358610.1|	Sulfurovum sp. NBC37-1	E-value = 4.00E-39	Identity = 44.50%
	3		gi|57241433|ref|ZP_00369379.1|	Campylobacter lari RM2100	E-value = 2.00E-32	Identity = 42.98%
	4		gi|78777729|ref|YP_394044.1|	Sulfuromonas denitrificans ATCC 33889	E-value = 3.00E-32	Identity = 37.55%
	5		gi|118474912|ref|YP_891531.1|	Campylobacter fetus subsp. fetus 82-40	E-value = 6.00E-32	Identity = 40.60%
AB0621	1	*uvrB*	gi|152990079|ref|YP_001355801.1|	Nitratiruptor sp. SB155-2	E-value = 0	Identity = 71.22%
	2		gi|152993729|ref|YP_001359450.1|	Sulfurovum sp. NBC37-1	E-value = 0	Identity = 69.86%
	3		gi|78777891|ref|YP_394206.1|	Sulfuromonas denitrificans ATCC 33889	E-value = 0	Identity = 70.18%

	4		gi|154174543|ref|YP_001408491.1|	Campylobacter curvus 525.92	E-value = 0	Identity = 69.35%
	5		gi|34558250|ref|NP_908065.1|	Wolinella succinogenes DSM 1740	E-value = 0	Identity = 69.51%
AB0622	1		gi|34557060|ref|NP_906875.1|	Wolinella succinogenes DSM 1740	E-value = 5.00E-38	Identity = 25.86%
AB0623	1	*nth*	gi|152993389|ref|YP_001359110.1|	Sulfurovum sp. NBC37-1	E-value = 1.00E-73	Identity = 66.04%
	2		gi|78776716|ref|YP_393031.1|	Sulfuromonas denitrificans ATCC 33889	E-value = 2.00E-73	Identity = 65.09%
	3		gi|149194290|ref|ZP_01871387.1|	Caminibacter mediatlanticus TB-2	E-value = 5.00E-70	Identity = 63.33%
	4		gi|154149294|ref|YP_001406776.1|	Campylobacter hominis ATCC BAA-381	E-value = 6.00E-68	Identity = 61.76%
	5		gi|152990308|ref|YP_001356030.1|	Nitratiruptor sp. SB155-2	E-value = 1.00E-67	Identity = 63.46%
AB0624	1			***** No matches found *****		
AB0625	1		gi|78776578|ref|YP_392893.1|	Sulfuromonas denitrificans ATCC 33889	E-value = 2.00E-155	Identity = 46.95%
	2		gi|152993756|ref|YP_001359477.1|	Sulfurovum sp. NBC37-1	E-value = 5.00E-153	Identity = 46.29%
	3		gi|34558795|gb|AAQ75140.1|	Alvinella pompejana epibiont 6C6	E-value = 1.00E-139	Identity = 45.35%
	4		gi|152991362|ref|YP_001357084.1|	Nitratiruptor sp. SB155-2	E-value = 1.00E-103	Identity = 34.43%
	5		gi|95931381|ref|ZP_01314092.1|	Desulfuromonas acetoxidans DSM 684	E-value = 3.00E-74	Identity = 29.69%
AB0626	1		gi|70734150|ref|YP_257790.1|	Pseudomonas fluorescens Pf-5	E-value = 1.00E-150	Identity = 42.34%
	2		gi|126195859|gb|EAZ59922.1|	Pseudomonas aeruginosa 2192	E-value = 3.00E-144	Identity = 41.99%
	3		gi|84325518|ref|ZP_00973553.1|	Pseudomonas aeruginosa 2192	E-value = 3.00E-144	Identity = 41.99%
	4		gi|107103310|ref|ZP_01367228.1|	Pseudomonas aeruginosa PACS2	E-value = 3.00E-144	Identity = 41.99%
	5		gi|15598985|ref|NP_252479.1|	Pseudomonas aeruginosa PAO1	E-value = 3.00E-144	Identity = 41.99%
AB0627	1			***** No matches found *****		
AB0628	1			***** No matches found *****		
AB0629	1			***** No matches found *****		
AB0630	1		gi|118475245|ref|YP_891579.1|	Campylobacter fetus subsp. fetus 82-40	E-value = 4.00E-30	Identity = 33.18%
	2		gi|157164839|ref|YP_001466181.1|	Campylobacter concisus 13826	E-value = 4.00E-29	Identity = 34.09%
	3		gi|152993952|ref|YP_001359673.1|	Sulfurovum sp. NBC37-1	E-value = 1.00E-27	Identity = 33.04%
	4		gi|57169075|ref|ZP_00368202.1|	Campylobacter coli RM2228	E-value = 1.00E-26	Identity = 31.22%
	5		gi|154173869|ref|YP_001407525.1|	Campylobacter curvus 525.92	E-value = 6.00E-26	Identity = 34.39%
AB0631	1			***** No matches found *****		
AB0632	1		gi|95928655|ref|ZP_01311402.1|	Desulfuromonas acetoxidans DSM 684	E-value = 2.00E-59	Identity = 27.81%
AB0633	1		gi|34557328|ref|NP_907143.1|	Wolinella succinogenes DSM 1740	E-value = 3.00E-58	Identity = 32.54%
	2		gi|117619052|ref|YP_855577.1|	Aeromonas hydrophila subsp. hydrophila ATCC 7966	E-value = 1.00E-52	Identity = 30.90%
	3		gi|144897509|emb|CAM74373.1|	Magnetospirillum gryphiswaldense MSR-1	E-value = 6.00E-47	Identity = 27.44%
	4		gi|30249816|ref|NP_841886.1|	Nitrosomonas europaea ATCC 19718	E-value = 5.00E-43	Identity = 27.93%
	5		gi|34557239|ref|NP_907054.1|	Wolinella succinogenes DSM 1740	E-value = 5.00E-42	Identity = 30.98%
AB0634	1		gi|154174828|ref|YP_001407308.1|	Campylobacter curvus 525.92	E-value = 3.00E-132	Identity = 62.22%
	2		gi|157164264|ref|YP_001465930.1|	Campylobacter concisus 13826	E-value = 3.00E-131	Identity = 63.12%
	3		gi|118474660|ref|YP_891216.1|	Campylobacter fetus subsp. fetus 82-40	E-value = 2.00E-130	Identity = 61.77%
	4		gi|57505253|ref|ZP_00371182.1|	Campylobacter upsaliensis RM3195	E-value = 2.00E-129	Identity = 61.67%
	5		gi|57241729|ref|ZP_00369674.1|	Campylobacter lari RM2100	E-value = 3.00E-128	Identity = 62.41%
AB0636	1	*rpiB*	gi|78777314|ref|YP_393629.1|	Sulfuromonas denitrificans ATCC 33889	E-value = 6.00E-48	Identity = 61.54%
	2		gi|149194498|ref|ZP_01871594.1|	Caminibacter mediatlanticus TB-2	E-value = 2.00E-45	Identity = 62.41%
	3		gi|34557704|ref|NP_907519.1|	Wolinella succinogenes DSM 1740	E-value = 4.00E-44	Identity = 58.57%
	4		gi|152992257|ref|YP_001357978.1|	Sulfurovum sp. NBC37-1	E-value = 1.00E-43	Identity = 57.14%
	5		gi|152990457|ref|YP_001356179.1|	Nitratiruptor sp. SB155-2	E-value = 8.00E-43	Identity = 59.15%
AB0637	1	*lepP*	gi|154175097|ref|YP_001407943.1|	Campylobacter curvus 525.92	E-value = 3.00E-86	Identity = 58.58%
	2		gi|152990459|ref|YP_001356181.1|	Nitratiruptor sp. SB155-2	E-value = 3.00E-83	Identity = 57.04%
	3		gi|57242455|ref|ZP_00370393.1|	Campylobacter upsaliensis RM3195	E-value = 4.00E-79	Identity = 56.59%
	4		gi|34557702|ref|NP_907517.1|	Wolinella succinogenes DSM 1740	E-value = 4.00E-79	Identity = 54.92%
	5		gi|57241057|ref|ZP_00369004.1|	Campylobacter lari RM2100	E-value = 3.00E-77	Identity = 56.09%

AB0638	1	*folD*	gi|152992260|ref|YP_001357981.1|	Sulfurovum sp. NBC37-1	E-value = 2.00E-96	Identity = 65.36%
	2		gi|78777311|ref|YP_393626.1|	Sulfuromonas denitrificans ATCC 33889	E-value = 8.00E-95	Identity = 62.41%
	3		gi|149193973|ref|ZP_01871071.1|	Caminibacter mediatlanticus TB-2	E-value = 3.00E-94	Identity = 65.11%
	4		gi|34557701|ref|NP_907516.1|	Wolinella succinogenes DSM 1740	E-value = 3.00E-94	Identity = 62.41%
	5		gi|152990460|ref|YP_001356182.1|	Nitratiruptor sp. SB155-2	E-value = 8.00E-92	Identity = 63.48%
AB0639	1	*rplY*	gi|149194451|ref|ZP_01871548.1|	Caminibacter mediatlanticus TB-2	E-value = 5.00E-60	Identity = 65.17%
	2		gi|152990133|ref|YP_001355855.1|	Nitratiruptor sp. SB155-2	E-value = 7.00E-50	Identity = 62.28%
	3		gi|57241439|ref|ZP_00369385.1|	Campylobacter lari RM2100	E-value = 2.00E-49	Identity = 62.36%
	4		gi|154174063|ref|YP_001408881.1|	Campylobacter curvus 525.92	E-value = 3.00E-48	Identity = 60.67%
	5		gi|57168447|ref|ZP_00367581.1|	Campylobacter coli RM2228	E-value = 4.00E-48	Identity = 60.67%
AB0640	1	*pth*	gi|152990134|ref|YP_001355856.1|	Nitratiruptor sp. SB155-2	E-value = 3.00E-49	Identity = 50.27%
	2		gi|118474844|ref|YP_891539.1|	Campylobacter fetus subsp. fetus 82-40	E-value = 1.00E-42	Identity = 52.17%
	3		gi|78776667|ref|YP_392982.1|	Sulfuromonas denitrificans ATCC 33889	E-value = 8.00E-42	Identity = 55.80%
	4		gi|154173637|ref|YP_001408880.1|	Campylobacter curvus 525.92	E-value = 7.00E-41	Identity = 52.46%
	5		gi|82523965|emb|CAI78776.1|	uncultured epsilon proteobacterium	E-value = 1.00E-39	Identity = 51.93%
AB0641	1		gi|34558361|ref|NP_908176.1|	Wolinella succinogenes DSM 1740	E-value = 2.00E-60	Identity = 35.33%
	2		gi|82523966|emb|CAI78777.1|	uncultured epsilon proteobacterium	E-value = 2.00E-54	Identity = 36.42%
	3		gi|118474677|ref|YP_891476.1|	Campylobacter fetus subsp. fetus 82-40	E-value = 3.00E-51	Identity = 33.62%
	4		gi|78776668|ref|YP_392983.1|	Sulfuromonas denitrificans ATCC 33889	E-value = 3.00E-51	Identity = 34.50%
	5		gi|157165310|ref|YP_001466458.1|	Campylobacter concisus 13826	E-value = 8.00E-48	Identity = 30.95%
AB0642	1			***** No matches found *****		
AB0643	1	*amiA*	gi|34556684|ref|NP_906499.1|	Wolinella succinogenes DSM 1740	E-value = 1.00E-78	Identity = 46.30%
	2		gi|78776921|ref|YP_393236.1|	Sulfuromonas denitrificans ATCC 33889	E-value = 3.00E-78	Identity = 39.08%
	3		gi|154174999|ref|YP_001407882.1|	Campylobacter curvus 525.92	E-value = 2.00E-71	Identity = 40.86%
	4		gi|157165276|ref|YP_001467337.1|	Campylobacter concisus 13826	E-value = 3.00E-67	Identity = 39.96%
	5		gi|118474687|ref|YP_892367.1|	Campylobacter fetus subsp. fetus 82-40	E-value = 6.00E-67	Identity = 40.35%
AB0644	1	*npd*	gi|152990509|ref|YP_001356231.1|	Nitratiruptor sp. SB155-2	E-value = 9.00E-147	Identity = 73.26%
	2		gi|78776920|ref|YP_393235.1|	Sulfuromonas denitrificans ATCC 33889	E-value = 5.00E-140	Identity = 69.77%
	3		gi|34556683|ref|NP_906498.1|	Wolinella succinogenes DSM 1740	E-value = 5.00E-138	Identity = 65.60%
	4		gi|15611777|ref|NP_223428.1|	Helicobacter pylori J99	E-value = 2.00E-137	Identity = 69.39%
	5		gi|149194047|ref|ZP_01871145.1|	Caminibacter mediatlanticus TB-2	E-value = 1.00E-136	Identity = 70.85%
AB0645	1	*tyrS*	gi|78776919|ref|YP_393234.1|	Sulfuromonas denitrificans ATCC 33889	E-value = 9.00E-150	Identity = 68.61%
	2		gi|152990508|ref|YP_001356230.1|	Nitratiruptor sp. SB155-2	E-value = 3.00E-146	Identity = 67.85%
	3		gi|34556682|ref|NP_906497.1|	Wolinella succinogenes DSM 1740	E-value = 1.00E-145	Identity = 64.66%
	4		gi|118474811|ref|YP_892369.1|	Campylobacter fetus subsp. fetus 82-40	E-value = 2.00E-143	Identity = 64.09%
	5		gi|157164866|ref|YP_001467339.1|	Campylobacter concisus 13826	E-value = 3.00E-143	Identity = 65.40%
AB0646	1	*spoT*	gi|34556681|ref|NP_906496.1|	Wolinella succinogenes DSM 1740	E-value = 0	Identity = 51.26%
	2		gi|152990507|ref|YP_001356229.1|	Nitratiruptor sp. SB155-2	E-value = 0	Identity = 49.79%
	3		gi|152993328|ref|YP_001359049.1|	Sulfurovum sp. NBC37-1	E-value = 0	Identity = 50.48%
	4		gi|32266330|ref|NP_860362.1|	Helicobacter hepaticus ATCC 51449	E-value = 0	Identity = 47.07%
	5		gi|154174887|ref|YP_001407885.1|	Campylobacter curvus 525.92	E-value = 0	Identity = 46.9%
AB0647	1	*rpoZ*	gi|152990506|ref|YP_001356228.1|	Nitratiruptor sp. SB155-2	E-value = 6.00E-08	Identity = 56.92%
	2		gi|57505446|ref|ZP_00371374.1|	Campylobacter upsaliensis RM3195	E-value = 3.00E-07	Identity = 45.71%
	3		gi|118475016|ref|YP_892371.1|	Campylobacter fetus subsp. fetus 82-40	E-value = 4.00E-07	Identity = 50.00%
	4		gi|154148131|ref|YP_001406833.1|	Campylobacter hominis ATCC BAA-381	E-value = 5.00E-07	Identity = 47.06%
	5		gi|34556680|ref|NP_906495.1|	Wolinella succinogenes DSM 1740	E-value = 5.00E-07	Identity = 55.74%
AB0648	1	*pyrH*	gi|152993330|ref|YP_001359051.1|	Sulfurovum sp. NBC37-1	E-value = 4.00E-88	Identity = 74.89%
	2		gi|57505445|ref|ZP_00371373.1|	Campylobacter upsaliensis RM3195	E-value = 1.00E-81	Identity = 70.82%
	3		gi|152990505|ref|YP_001356227.1|	Nitratiruptor sp. SB155-2	E-value = 1.00E-81	Identity = 71.06%

	4		gi|34556679|ref|NP_906494.1|	Wolinella succinogenes DSM 1740	E-value = 2.00E-81	Identity = 74.46%
	5		gi|78776916|ref|YP_393231.1|	Sulfuromonas denitrificans ATCC 33889	E-value = 6.00E-81	Identity = 75.32%
AB0649	1		gi|34558028|ref|NP_907843.1|	Wolinella succinogenes DSM 1740	E-value = 9.00E-49	Identity = 32.87%
	2		gi|157164688|ref|YP_001467722.1|	Campylobacter concisus 13826	E-value = 3.00E-40	Identity = 33.33%
	3		gi|152989970|ref|YP_001355692.1|	Nitratiruptor sp. SB155-2	E-value = 4.00E-39	Identity = 33.71%
	4		gi|149194773|ref|ZP_01871868.1|	Caminibacter mediatlanticus TB-2	E-value = 2.00E-34	Identity = 34.42%
	5		gi|154148312|ref|YP_001406027.1|	Campylobacter hominis ATCC BAA-381	E-value = 2.00E-34	Identity = 34.24%
AB0650	1	*pdxA*	gi|78777093|ref|YP_393408.1|	Sulfuromonas denitrificans ATCC 33889	E-value = 6.00E-104	Identity = 62.33%
	2		gi|149194479|ref|ZP_01871575.1|	Caminibacter mediatlanticus TB-2	E-value = 7.00E-102	Identity = 67.23%
	3		gi|152990831|ref|YP_001356553.1|	Nitratiruptor sp. SB155-2	E-value = 7.00E-100	Identity = 61.13%
	4		gi|118475415|ref|YP_891856.1|	Campylobacter fetus subsp. fetus 82-40	E-value = 3.00E-97	Identity = 61.06%
	5		gi|152993274|ref|YP_001358995.1|	Sulfurovum sp. NBC37-1	E-value = 1.00E-95	Identity = 57.14%
AB0651	1	*pdxJ*	gi|152993273|ref|YP_001358994.1|	Sulfurovum sp. NBC37-1	E-value = 2.00E-87	Identity = 65.89%
	2		gi|152990830|ref|YP_001356552.1|	Nitratiruptor sp. SB155-2	E-value = 9.00E-85	Identity = 66.67%
	3		gi|34556557|ref|NP_906372.1|	Wolinella succinogenes DSM 1740	E-value = 1.00E-77	Identity = 58.91%
	4		gi|78777094|ref|YP_393409.1|	Sulfuromonas denitrificans ATCC 33889	E-value = 4.00E-77	Identity = 62.65%
	5		gi|154147967|ref|YP_001407226.1|	Campylobacter hominis ATCC BAA-381	E-value = 7.00E-73	Identity = 59.07%
AB0652	1	*trpE*	gi|152990924|ref|YP_001356646.1|	Nitratiruptor sp. SB155-2	E-value = 1.00E-164	Identity = 63.75%
	2		gi|152992067|ref|YP_001357788.1|	Sulfurovum sp. NBC37-1	E-value = 4.00E-156	Identity = 59.14%
	3		gi|78777475|ref|YP_393790.1|	Sulfuromonas denitrificans ATCC 33889	E-value = 1.00E-152	Identity = 58.89%
	4		gi|149193726|ref|ZP_01870824.1|	Caminibacter mediatlanticus TB-2	E-value = 5.00E-150	Identity = 60.73%
	5		gi|34556541|ref|NP_906356.1|	Wolinella succinogenes DSM 1740	E-value = 4.00E-138	Identity = 54.85%
AB0653	1			***** No matches found *****		
AB0654	1	*glyA1*	gi|78778141|ref|YP_394456.1|	Sulfuromonas denitrificans ATCC 33889	E-value = 0	Identity = 84.29%
	2		gi|78777472|ref|YP_393787.1|	Sulfuromonas denitrificans ATCC 33889	E-value = 0	Identity = 83.01%
	3		gi|152992073|ref|YP_001357794.1|	Sulfurovum sp. NBC37-1	E-value = 0	Identity = 83.82%
	4		gi|149193729|ref|ZP_01870827.1|	Caminibacter mediatlanticus TB-2	E-value = 0	Identity = 77.24%
	5		gi|118475761|ref|YP_892299.1|	Campylobacter fetus subsp. fetus 82-40	E-value = 0	Identity = 75.49%
AB0655	1	*lysS*	gi|78777471|ref|YP_393786.1|	Sulfuromonas denitrificans ATCC 33889	E-value = 0	Identity = 78.77%
	2		gi|152990921|ref|YP_001356643.1|	Nitratiruptor sp. SB155-2	E-value = 0	Identity = 66.73%
	3		gi|152992078|ref|YP_001357799.1|	Sulfurovum sp. NBC37-1	E-value = 0	Identity = 63.91%
	4		gi|34556537|ref|NP_906352.1|	Wolinella succinogenes DSM 1740	E-value = 0	Identity = 64.62%
	5		gi|149193730|ref|ZP_01870828.1|	Caminibacter mediatlanticus TB-2	E-value = 6.00E-177	Identity = 62.50%
AB0656	1		gi|152990920|ref|YP_001356642.1|	Nitratiruptor sp. SB155-2	E-value = 8.00E-28	Identity = 39.42%
	2		gi|153951399|ref|YP_001398559.1|	Campylobacter jejuni subsp. doylei 269.97	E-value = 1.00E-27	Identity = 39.78%
	3		gi|86151287|ref|ZP_01069502.1|	Campylobacter jejuni subsp. jejuni 260.94	E-value = 1.00E-27	Identity = 40.32%
	4		gi|157414694|ref|YP_001481950.1|	Campylobacter jejuni subsp. jejuni 81116	E-value = 1.00E-27	Identity = 40.32%
	5		gi|86153823|ref|ZP_01072026.1|	Campylobacter jejuni subsp. jejuni HB93-13	E-value = 2.00E-27	Identity = 40.32%
AB0657	1	*ispG*	gi|78776577|ref|YP_392892.1|	Sulfuromonas denitrificans ATCC 33889	E-value = 3.00E-151	Identity = 77.21%
	2		gi|152993710|ref|YP_001359431.1|	Sulfurovum sp. NBC37-1	E-value = 1.00E-148	Identity = 76.59%
	3		gi|152990086|ref|YP_001355808.1|	Nitratiruptor sp. SB155-2	E-value = 3.00E-144	Identity = 75.21%
	4		gi|149193897|ref|ZP_01870995.1|	Caminibacter mediatlanticus TB-2	E-value = 1.00E-132	Identity = 69.36%
	5		gi|57506053|ref|ZP_00371976.1|	Campylobacter upsaliensis RM3195	E-value = 2.00E-128	Identity = 63.82%
AB0658	1		gi|78778156|ref|YP_394471.1|	Sulfuromonas denitrificans ATCC 33889	E-value = 2.00E-78	Identity = 42.46%
AB0659	1	*dnaB*	gi|78776576|ref|YP_392891.1|	Sulfuromonas denitrificans ATCC 33889	E-value = 8.00E-135	Identity = 56.56%
	2		gi|34556788|ref|NP_906603.1|	Wolinella succinogenes DSM 1740	E-value = 2.00E-128	Identity = 56.72%
	3		gi|152990087|ref|YP_001355809.1|	Nitratiruptor sp. SB155-2	E-value = 1.00E-125	Identity = 54.28%
	4		gi|149193895|ref|ZP_01870993.1|	Caminibacter mediatlanticus TB-2	E-value = 2.00E-115	Identity = 50.42%
	5		gi|118474379|ref|YP_892150.1|	Campylobacter fetus subsp. fetus 82-40	E-value = 8.00E-103	Identity = 48.11%

AB0660	1	*ugd*	gi|78778162|ref|YP_394477.1|	Sulfuromonas denitrificans ATCC 33889	E-value = 4.00E-176	Identity = 81.96%
	2		gi|152994834|ref|YP_001339669.1|	Marinomonas sp. MWYL1	E-value = 5.00E-171	Identity = 78.81%
	3		gi|88795903|ref|ZP_01111592.1|	Alteromonas macleodii 'Deep ecotype'	E-value = 1.00E-168	Identity = 78.55%
	4		gi|116220093|ref|ZP_01485541.1|	Vibrio cholerae V51	E-value = 4.00E-167	Identity = 75.00%
	5		gi|116190104|ref|ZP_01479846.1|	Vibrio cholerae MO10	E-value = 8.00E-167	Identity = 74.74%
AB0661	1		gi|34556483|ref|NP_906298.1|	Wolinella succinogenes DSM 1740	E-value = 2.00E-152	Identity = 74.50%
	2		gi|21673069|ref|NP_661134.1|	Chlorobium tepidum TLS	E-value = 3.00E-141	Identity = 68.84%
	3		gi|56459673|ref|YP_154954.1|	Idiomarina loihiensis L2TR	E-value = 1.00E-140	Identity = 67.23%
	4		gi|152991158|ref|YP_001356880.1|	Nitratiruptor sp. SB155-2	E-value = 3.00E-137	Identity = 66.67%
	5		gi|157164041|ref|YP_001467309.1|	Campylobacter concisus 13826	E-value = 9.00E-135	Identity = 67.51%
AB0662	1		gi|157165142|ref|YP_001467302.1|	Campylobacter concisus 13826	E-value = 6.00E-178	Identity = 74.51%
	2		gi|149195132|ref|ZP_01872223.1|	Caminibacter mediatlanticus TB-2	E-value = 2.00E-174	Identity = 74.41%
	3		gi|154175455|ref|YP_001407699.1|	Campylobacter curvus 525.92	E-value = 2.00E-174	Identity = 72.33%
	4		gi|113475907|ref|YP_721968.1|	Trichodesmium erythraeum IMS101	E-value = 5.00E-168	Identity = 69.64%
	5		gi|34556508|ref|NP_906323.1|	Wolinella succinogenes DSM 1740	E-value = 2.00E-166	Identity = 70.78%
AB0663	1		gi|157165229|ref|YP_001467378.1|	Campylobacter concisus 13826	E-value = 2.00E-162	Identity = 84.81%
	2		gi|78777619|ref|YP_393934.1|	Sulfuromonas denitrificans ATCC 33889	E-value = 5.00E-151	Identity = 79.13%
	3		gi|152991689|ref|YP_001357410.1|	Sulfurovum sp. NBC37-1	E-value = 1.00E-146	Identity = 77.67%
	4		gi|34558464|ref|NP_908279.1|	Wolinella succinogenes DSM 1740	E-value = 1.00E-131	Identity = 69.94%
	5		gi|124004200|ref|ZP_01689046.1|	Microscilla marina ATCC 23134	E-value = 2.00E-117	Identity = 65.40%
AB0664	1		gi|78777618|ref|YP_393933.1|	Sulfuromonas denitrificans ATCC 33889	E-value = 1.00E-87	Identity = 81.77%
	2		gi|157164668|ref|YP_001467377.1|	Campylobacter concisus 13826	E-value = 1.00E-82	Identity = 79.37%
	3		gi|34558463|ref|NP_908278.1|	Wolinella succinogenes DSM 1740	E-value = 9.00E-73	Identity = 71.51%
	4		gi|152991690|ref|YP_001357411.1|	Sulfurovum sp. NBC37-1	E-value = 2.00E-72	Identity = 72.73%
	5		gi|32267160|ref|NP_861192.1|	Helicobacter hepaticus ATCC 51449	E-value = 1.00E-61	Identity = 60.11%
AB0665	1		gi|157164115|ref|YP_001467376.1|	Campylobacter concisus 13826	E-value = 3.00E-160	Identity = 75.14%
	2		gi|152991691|ref|YP_001357412.1|	Sulfurovum sp. NBC37-1	E-value = 8.00E-156	Identity = 74.31%
	3		gi|34558462|ref|NP_908277.1|	Wolinella succinogenes DSM 1740	E-value = 5.00E-142	Identity = 70.11%
	4		gi|152991058|ref|YP_001356780.1|	Nitratiruptor sp. SB155-2	E-value = 6.00E-125	Identity = 60.77%
	5		gi|154175323|ref|YP_001407552.1|	Campylobacter curvus 525.92	E-value = 3.00E-115	Identity = 57.82%
AB0666	1		gi|118745620|ref|ZP_01593590.1|	Geobacter lovleyi SZ	E-value = 2.00E-117	Identity = 53.69%
	2		gi|124515336|gb|EAY56846.1|	Leptospirillum sp. Group II UBA	E-value = 3.00E-103	Identity = 46.58%
	3		gi|91201285|emb|CAJ74345.1|	Candidatus Kuenenia stuttgartiensis	E-value = 9.00E-100	Identity = 50.00%
	4		gi|117926941|ref|YP_867558.1|	Magnetococcus sp. MC-1	E-value = 6.00E-88	Identity = 42.96%
	5		gi|154707773|ref|YP_001423503.1|	Coxiella burnetii Dugway 7E9-12	E-value = 3.00E-79	Identity = 42.49%
AB0667	1		gi|157164630|ref|YP_001467375.1|	Campylobacter concisus 13826	E-value = 2.00E-171	Identity = 87.42%
	2		gi|124004199|ref|ZP_01689045.1|	Microscilla marina ATCC 23134	E-value = 5.00E-138	Identity = 71.34%
	3		gi|33594840|ref|NP_882483.1|	Bordetella parapertussis 12822	E-value = 8.00E-137	Identity = 69.16%
	4		gi|114566251|ref|YP_753405.1|	Syntrophomonas wolfei subsp. wolfei str. Goettingen	E-value = 8.00E-98	Identity = 55.94%
	5		gi|153208020|ref|ZP_01946554.1|	Coxiella burnetii 'MSU Goat Q177'	E-value = 1.00E-95	Identity = 53.16%
AB0668	1		gi|157163982|ref|YP_001467373.1|	Campylobacter concisus 13826	E-value = 5.00E-179	Identity = 75.68%
	2		gi|78776391|ref|YP_392706.1|	Sulfuromonas denitrificans ATCC 33889	E-value = 2.00E-151	Identity = 65.90%
	3		gi|114566252|ref|YP_753406.1|	Syntrophomonas wolfei subsp. wolfei str. Goettingen	E-value = 2.00E-99	Identity = 47.45%
	4		gi|124004198|ref|ZP_01689044.1|	Microscilla marina ATCC 23134	E-value = 3.00E-92	Identity = 45.32%
	5		gi|154706674|ref|YP_001424278.1|	Coxiella burnetii Dugway 7E9-12	E-value = 1.00E-81	Identity = 42.09%
AB0669	1	*asnB2*	gi|157165663|ref|YP_001467372.1|	Campylobacter concisus 13826	E-value = 0	Identity = 74.17%
	2		gi|78776401|ref|YP_392716.1|	Sulfuromonas denitrificans ATCC 33889	E-value = 0	Identity = 68.5%
	3		gi|33594842|ref|NP_882485.1|	Bordetella parapertussis 12822	E-value = 0	Identity = 55.46%
	4		gi|104304766|gb|ABF72471.1|	Bordetella parapertussis	E-value = 0	Identity = 55.46%

	5		gi|114566253|ref|YP_753407.1|	Syntrophomonas wolfei subsp. wolfei str. Goettingen	E-value = 0	Identity = 57.55%
AB0670	1		gi|153208027|ref|ZP_01946561.1|	Coxiella burnetii 'MSU Goat Q177'	E-value = 4.00E-42	Identity = 52.63%
	2		gi|154707691|ref|YP_001424280.1|	Coxiella burnetii Dugway 7E9-12	E-value = 1.00E-41	Identity = 51.46%
	3		gi|29654160|ref|NP_819852.1|	Coxiella burnetii RSA 493	E-value = 2.00E-41	Identity = 52.05%
	4		gi|93005449|ref|YP_579886.1|	Psychrobacter cryohalolentis K5	E-value = 2.00E-39	Identity = 59.59%
	5		gi|144899767|emb|CAM76631.1|	Magnetospirillum gryphiswaldense MSR-1	E-value = 5.00E-32	Identity = 40.83%
AB0671	1	*wbpG1*	gi|124004195|ref|ZP_01689041.1|	Microscilla marina ATCC 23134	E-value = 8.00E-137	Identity = 64.42%
	2		gi|152982955|ref|YP_001353959.1|	Janthinobacterium sp. Marseille	E-value = 1.00E-107	Identity = 51.74%
	3		gi|147919832|ref|YP_686419.1|	uncultured methanogenic archaeon RC-I	E-value = 3.00E-86	Identity = 41.62%
	4		gi|45358644|ref|NP_988201.1|	Methanococcus maripaludis S2	E-value = 2.00E-65	Identity = 40.33%
	5		gi|88810769|ref|ZP_01126026.1|	Nitrococcus mobilis Nb-231	E-value = 3.00E-63	Identity = 39.18%
AB0672	1	*hisH2*	gi|124004194|ref|ZP_01689040.1|	Microscilla marina ATCC 23134	E-value = 3.00E-66	Identity = 60.49%
	2		gi|147919831|ref|YP_686420.1|	uncultured methanogenic archaeon RC-I	E-value = 3.00E-56	Identity = 50.49%
	3		gi|45358645|ref|NP_988202.1|	Methanococcus maripaludis S2	E-value = 1.00E-54	Identity = 53.43%
	4		gi|88810767|ref|ZP_01126024.1|	Nitrococcus mobilis Nb-231	E-value = 5.00E-50	Identity = 48.02%
	5		gi|37678539|ref|NP_933148.1|	Vibrio vulnificus YJ016	E-value = 6.00E-50	Identity = 44.23%
AB0673	1	*hisF2*	gi|124004193|ref|ZP_01689039.1|	Microscilla marina ATCC 23134	E-value = 4.00E-88	Identity = 61.75%
	2		gi|124483504|emb|CAM32625.1|	Herbaspirillum seropedicae	E-value = 1.00E-85	Identity = 56.92%
	3		gi|20559897|gb|AAM27665.1|AF498408_13	Pseudomonas aeruginosa	E-value = 3.00E-68	Identity = 50.00%
	4		gi|15598347|ref|NP_251841.1|	Pseudomonas aeruginosa PAO1	E-value = 3.00E-68	Identity = 50.40%
	5		gi|49082270|gb|AAT50535.1|	synthetic construct	E-value = 3.00E-68	Identity = 50.40%
AB0674	1		gi|114566261|ref|YP_753415.1|	Syntrophomonas wolfei subsp. wolfei str. Goettingen	E-value = 6.00E-11	Identity = 26.07%
AB0675	1			***** No matches found *****		
AB0676	1		gi|34556507|ref|NP_906322.1|	Wolinella succinogenes DSM 1740	E-value = 9.00E-158	Identity = 55.22%
	2		gi|153951013|ref|YP_001397760.1|	Campylobacter jejuni subsp. doylei 269.97	E-value = 1.00E-137	Identity = 48.58%
	3		gi|57241552|ref|ZP_00369498.1|	Campylobacter lari RM2100	E-value = 1.00E-137	Identity = 49.46%
	4		gi|86152730|ref|ZP_01070935.1|	Campylobacter jejuni subsp. jejuni HB93-13	E-value = 3.00E-135	Identity = 48.22%
	5		gi|15792455|ref|NP_282278.1|	Campylobacter jejuni subsp. jejuni NCTC 11168	E-value = 7.00E-135	Identity = 48.22%
AB0677	1		gi|78189231|ref|YP_379569.1|	Chlorobium chlorochromatii CaD3	E-value = 1.00E-73	Identity = 32.66%
	2		gi|126462117|ref|YP_001043231.1|	Rhodobacter sphaeroides ATCC 17029	E-value = 8.00E-68	Identity = 29.33%
	3		gi|109897409|ref|YP_660664.1|	Pseudoalteromonas atlantica T6c	E-value = 6.00E-67	Identity = 30.31%
	4		gi|77463243|ref|YP_352747.1|	Rhodobacter sphaeroides 2.4.1	E-value = 3.00E-66	Identity = 29.25%
	5		gi|116620311|ref|YP_822467.1|	Solibacter usitatus Ellin6076	E-value = 4.00E-66	Identity = 28.76%
AB0678	1	*pglJ*	gi|88712954|ref|ZP_01107039.1|	Flavobacteriales bacterium HTCC2170	E-value = 4.00E-64	Identity = 40.05%
	2		gi|34556500|ref|NP_906315.1|	Wolinella succinogenes DSM 1740	E-value = 2.00E-63	Identity = 41.84%
	3		gi|154173620|ref|YP_001407706.1|	Campylobacter curvus 525.92	E-value = 2.00E-59	Identity = 37.76%
	4		gi|154148421|ref|YP_001406516.1|	Campylobacter hominis ATCC BAA-381	E-value = 1.00E-58	Identity = 39.68%
	5		gi|57168011|ref|ZP_00367150.1|	Campylobacter coli RM2228	E-value = 1.00E-56	Identity = 41.05%
AB0679	1	*asnB*	gi|149195369|ref|ZP_01872455.1|	Caminibacter mediatlanticus TB-2	E-value = 0	Identity = 60.19%
	2		gi|125621795|gb|EAZ50122.1|	Vibrio cholerae V51	E-value = 3.00E-155	Identity = 45.54%
	3		gi|116220099|ref|ZP_01485547.1|	Vibrio cholerae V51	E-value = 4.00E-150	Identity = 45.63%
	4		gi|23128937|ref|ZP_00110773.1|	Nostoc punctiforme PCC 73102	E-value = 5.00E-142	Identity = 40.71%
	5		gi|71065231|ref|YP_263958.1|	Psychrobacter arcticus 273-4	E-value = 5.00E-140	Identity = 45.10%
AB0680	1		gi|30250198|ref|NP_842268.1|	Nitrosomonas europaea ATCC 19718	E-value = 5.00E-102	Identity = 48.37%
	2		gi|114332077|ref|YP_748299.1|	Nitrosomonas eutropha C91	E-value = 6.00E-102	Identity = 49.86%
	3		gi|149195368|ref|ZP_01872454.1|	Caminibacter mediatlanticus TB-2	E-value = 7.00E-100	Identity = 54.62%
	4		gi|124005067|ref|ZP_01689909.1|	Microscilla marina ATCC 23134	E-value = 3.00E-95	Identity = 47.41%
	5		gi|152993103|ref|YP_001358824.1|	Sulfurovum sp. NBC37-1	E-value = 4.00E-95	Identity = 45.11%
AB0681	1		gi|153201654|ref|ZP_01943463.1|	Listeria monocytogenes HPB2262	E-value = 5.00E-22	Identity = 32.14%

	2		gi|76798981|ref|ZP_00781181.1|	Streptococcus agalactiae 18RS21	E-value = 8.00E-21	Identity = 31.60%
	3		gi|121534916|ref|ZP_01666735.1|	Thermosinus carboxydivorans Nor1	E-value = 5.00E-20	Identity = 31.13%
	4		gi|127512347|ref|YP_001093544.1|	Shewanella loihica PV-4	E-value = 2.00E-19	Identity = 30.57%
	5		gi|153956291|ref|YP_001397056.1|	Clostridium kluyveri DSM 555	E-value = 3.00E-19	Identity = 35.47%
AB0682	1			***** No matches found *****		
AB0683	1		gi|118744670|ref|ZP_01592660.1|	Geobacter lovleyi SZ	E-value = 4.00E-90	Identity = 46.14%
	2		gi|78777617|ref|YP_393932.1|	Sulfuromonas denitrificans ATCC 33889	E-value = 5.00E-60	Identity = 39.10%
	3		gi|152991692|ref|YP_001357413.1|	Sulfurovum sp. NBC37-1	E-value = 2.00E-59	Identity = 38.73%
	4		gi|121606015|ref|YP_983344.1|	Polaromonas naphthalenivorans CJ2	E-value = 1.00E-52	Identity = 32.20%
	5		gi|73668674|ref|YP_304689.1|	Methanosarcina barkeri str. Fusaro	E-value = 5.00E-52	Identity = 34.47%
AB0684	1	*wbpG2*	gi|1545856|gb|AAC45861.1|	Pseudomonas aeruginosa	E-value = 3.00E-95	Identity = 46.03%
	2		gi|15598346|ref|NP_251840.1|	Pseudomonas aeruginosa PAO1	E-value = 3.00E-95	Identity = 46.03%
	3		gi|121606012|ref|YP_983341.1|	Polaromonas naphthalenivorans CJ2	E-value = 4.00E-95	Identity = 49.13%
	4		gi|117926371|ref|YP_866988.1|	Magnetococcus sp. MC-1	E-value = 2.00E-92	Identity = 46.34%
	5		gi|20559898|gb|AAM27666.1|AF498408_14	Pseudomonas aeruginosa	E-value = 3.00E-92	Identity = 46.11%
AB0685	1		gi|109897502|ref|YP_660757.1|	Pseudoalteromonas atlantica T6c	E-value = 3.00E-32	Identity = 41.76%
	2		gi|126667443|ref|ZP_01738414.1|	Marinobacter sp. ELB17	E-value = 3.00E-29	Identity = 42.68%
	3		gi|88704010|ref|ZP_01101725.1|	gamma proteobacterium KT 71	E-value = 1.00E-28	Identity = 37.58%
	4		gi|118580867|ref|YP_902117.1|	Pelobacter propionicus DSM 2379	E-value = 3.00E-25	Identity = 36.52%
	5		gi|113367016|gb|ABI34563.1|	Escherichia coli	E-value = 1.00E-24	Identity = 34.66%
AB0686	1		gi|88802433|ref|ZP_01117960.1|	Polaribacter irgensii 23-P	E-value = 4.00E-09	Identity = 25.41%
AB0687	1			***** No matches found *****		
AB0688	1		gi|148652056|ref|YP_001279149.1|	Psychrobacter sp. PRwf-1	E-value = 3.00E-56	Identity = 38.60%
	2		gi|52425551|ref|YP_088688.1|	Mannheimia succiniciproducens MBEL55E	E-value = 2.00E-52	Identity = 32.73%
	3		gi|117926370|ref|YP_866987.1|	Magnetococcus sp. MC-1	E-value = 4.00E-52	Identity = 30.95%
	4		gi|152977792|ref|YP_001343421.1|	Actinobacillus succinogenes 130Z	E-value = 3.00E-51	Identity = 31.79%
	5		gi|150015874|ref|YP_001308128.1|	Clostridium beijerinckii NCIMB 8052	E-value = 7.00E-48	Identity = 37.11%
AB0689	1	*wbpG3*	gi|121606012|ref|YP_983341.1|	Polaromonas naphthalenivorans CJ2	E-value = 6.00E-144	Identity = 60.42%
	2		gi|1545856|gb|AAC45861.1|	Pseudomonas aeruginosa	E-value = 1.00E-138	Identity = 61.07%
	3		gi|15598346|ref|NP_251840.1|	Pseudomonas aeruginosa PAO1	E-value = 1.00E-138	Identity = 61.07%
	4		gi|20559898|gb|AAM27666.1|AF498408_14	Pseudomonas aeruginosa	E-value = 4.00E-134	Identity = 60.87%
	5		gi|85714648|ref|ZP_01045635.1|	Nitrobacter sp. Nb-311A	E-value = 8.00E-97	Identity = 45.20%
AB0690	1	*hisH3*	gi|147919831|ref|YP_686420.1|	uncultured methanogenic archaeon RC-I	E-value = 8.00E-60	Identity = 53.27%
	2		gi|124004194|ref|ZP_01689040.1|	Microscilla marina ATCC 23134	E-value = 4.00E-58	Identity = 55.28%
	3		gi|45358645|ref|NP_988202.1|	Methanococcus maripaludis S2	E-value = 5.00E-58	Identity = 59.30%
	4		gi|15598348|ref|NP_251842.1|	Pseudomonas aeruginosa PAO1	E-value = 3.00E-55	Identity = 46.53%
	5		gi|49079444|gb|AAT49893.1|	synthetic construct	E-value = 3.00E-55	Identity = 46.53%
AB0691	1	*hisF3*	gi|20559897|gb|AAM27665.1|AF498408_13	Pseudomonas aeruginosa	E-value = 8.00E-76	Identity = 52.42%
	2		gi|15598347|ref|NP_251841.1|	Pseudomonas aeruginosa PAO1	E-value = 9.00E-76	Identity = 52.82%
	3		gi|49082270|gb|AAT50535.1|	synthetic construct	E-value = 9.00E-76	Identity = 52.82%
	4		gi|42523189|ref|NP_968569.1|	Bdellovibrio bacteriovorus HD100	E-value = 5.00E-73	Identity = 54.12%
	5		gi|85714650|ref|ZP_01045637.1|	Nitrobacter sp. Nb-311A	E-value = 5.00E-73	Identity = 55.51%
AB0692	1		gi|121606010|ref|YP_983339.1|	Polaromonas naphthalenivorans CJ2	E-value = 2.00E-129	Identity = 58.70%
	2		gi|116693989|ref|YP_728200.1|	Ralstonia eutropha H16	E-value = 6.00E-127	Identity = 56.01%
	3		gi|20559975|gb|AAM27733.1|AF498412_15	Pseudomonas aeruginosa	E-value = 2.00E-126	Identity = 56.40%
	4		gi|20559899|gb|AAM27667.1|AF498408_15	Pseudomonas aeruginosa	E-value = 3.00E-126	Identity = 56.13%
	5		gi|20559932|gb|AAM27698.1|AF498410_15	Pseudomonas aeruginosa	E-value = 7.00E-126	Identity = 56.13%
AB0693	1		gi|78777615|ref|YP_393930.1|	Sulfuromonas denitrificans ATCC 33889	E-value = 2.00E-169	Identity = 82.87%
	2		gi|157164094|ref|YP_001467359.1|	Campylobacter concisus 13826	E-value = 5.00E-162	Identity = 78.47%

	3		gi|152994867|ref|YP_001339702.1|	Marinomonas sp. MWYL1	E-value = 2.00E-143	Identity = 70.19%
	4		gi|156858319|gb|EDO51750.1|	Bacteroides uniformis ATCC 8492	E-value = 1.00E-131	Identity = 63.74%
	5		gi|15598344|ref|NP_251838.1|	Pseudomonas aeruginosa PAO1	E-value = 1.00E-120	Identity = 60.34%
AB0694	1		gi|152990994|ref|YP_001356716.1|	Nitratiruptor sp. SB155-2	E-value = 6.00E-90	Identity = 54.98%
	2		gi|152991698|ref|YP_001357419.1|	Sulfurovum sp. NBC37-1	E-value = 8.00E-62	Identity = 42.30%
	3		gi|37528619|ref|NP_931964.1|	Photorhabdus luminescens subsp. laumondii TTO1	E-value = 1.00E-61	Identity = 42.94%
	4		gi|157364739|ref|YP_001471506.1|	Thermotoga lettingae TMO	E-value = 2.00E-58	Identity = 39.04%
	5		gi|154248927|ref|YP_001409752.1|	Fervidobacterium nodosum Rt17-B1	E-value = 3.00E-51	Identity = 37.35%
AB0695	1		gi|114566271|ref|YP_753425.1|	Syntrophomonas wolfei subsp. wolfei str. Goettingen	E-value = 7.00E-47	Identity = 45.00%
	2		gi|148549143|ref|YP_001269245.1|	Pseudomonas putida F1	E-value = 1.00E-43	Identity = 36.54%
	3		gi|28868961|ref|NP_791580.1|	Pseudomonas syringae pv. tomato str. DC3000	E-value = 1.00E-41	Identity = 38.83%
	4		gi|116051138|ref|YP_790031.1|	Pseudomonas aeruginosa UCBPP-PA14	E-value = 4.00E-40	Identity = 36.79%
	5		gi|59803193|gb|AAX07740.1|	Escherichia coli	E-value = 5.00E-40	Identity = 35.60%
AB0696	1		gi|151572529|gb|EDN38183.1|	Francisella tularensis subsp. novicida GA99-3548	E-value = 8.00E-44	Identity = 44.21%
	2		gi|86144024|ref|ZP_01062362.1|	Flavobacterium sp. MED217	E-value = 9.00E-40	Identity = 49.40%
	3		gi|88802242|ref|ZP_01117769.1|	Polaribacter irgensii 23-P	E-value = 9.00E-40	Identity = 47.57%
	4		gi|29346238|ref|NP_809741.1|	Bacteroides thetaiotaomicron VPI-5482	E-value = 1.00E-39	Identity = 50.00%
	5		gi|150004940|ref|YP_001299684.1|	Bacteroides vulgatus ATCC 8482	E-value = 2.00E-39	Identity = 45.79%
AB0697	1	*pglF*	gi|151572542|gb|EDN38196.1|	Francisella tularensis subsp. novicida GA99-3548	E-value = 1.00E-166	Identity = 53.04%
	2		gi|151571086|gb|EDN36740.1|	Francisella tularensis subsp. novicida GA99-3549	E-value = 3.00E-162	Identity = 53.04%
	3		gi|89255990|ref|YP_513352.1|	Francisella tularensis subsp. holarctica	E-value = 9.00E-161	Identity = 52.70%
	4		gi|56708505|ref|YP_170401.1|	Francisella tularensis subsp. tularensis SCHU S4	E-value = 9.00E-161	Identity = 52.70%
	5		gi|156501993|ref|YP_001428058.1|	Francisella tularensis subsp. holarctica FTA	E-value = 2.00E-159	Identity = 52.82%
AB0698	1		gi|152993712|ref|YP_001359433.1|	Sulfurovum sp. NBC37-1	E-value = 5.00E-06	Identity = 32.06%
AB0699	1	*ligA*	gi|78776783|ref|YP_393098.1|	Sulfuromonas denitrificans ATCC 33889	E-value = 1.00E-64	Identity = 50.18%
	2		gi|52424994|ref|YP_088131.1|	Mannheimia succiniciproducens MBEL55E	E-value = 1.00E-55	Identity = 43.21%
	3		gi|113461467|ref|YP_719536.1|	Haemophilus somnus 129PT	E-value = 4.00E-55	Identity = 43.66%
	4		gi|53728451|ref|ZP_00132618.2|	Haemophilus somnus 2336	E-value = 6.00E-55	Identity = 45.68%
	5		gi|154148219|ref|YP_001406356.1|	Campylobacter hominis ATCC BAA-381	E-value = 2.00E-54	Identity = 46.86%
AB0700	1	*apt*	gi|34557706|ref|NP_907521.1|	Wolinella succinogenes DSM 1740	E-value = 1.00E-59	Identity = 60.77%
	2		gi|78777316|ref|YP_393631.1|	Sulfuromonas denitrificans ATCC 33889	E-value = 3.00E-55	Identity = 58.10%
	3		gi|57241150|ref|ZP_00369097.1|	Campylobacter lari RM2100	E-value = 4.00E-55	Identity = 60.00%
	4		gi|118474996|ref|YP_892327.1|	Campylobacter fetus subsp. fetus 82-40	E-value = 1.00E-54	Identity = 59.22%
	5		gi|157165725|ref|YP_001466475.1|	Campylobacter concisus 13826	E-value = 3.00E-54	Identity = 55.00%
AB0701	1	*trpB1*	gi|78777317|ref|YP_393632.1|	Sulfuromonas denitrificans ATCC 33889	E-value = 0	Identity = 78.95%
	2		gi|34557707|ref|NP_907522.1|	Wolinella succinogenes DSM 1740	E-value = 0	Identity = 73.88%
	3		gi|152990455|ref|YP_001356177.1|	Nitratiruptor sp. SB155-2	E-value = 0	Identity = 75.19%
	4		gi|152992248|ref|YP_001357969.1|	Sulfurovum sp. NBC37-1	E-value = 5.00E-175	Identity = 74.31%
	5		gi|149193711|ref|ZP_01870809.1|	Caminibacter mediatlanticus TB-2	E-value = 4.00E-173	Identity = 75.31%
AB0702	1		gi|152990454|ref|YP_001356176.1|	Nitratiruptor sp. SB155-2	E-value = 7.00E-69	Identity = 59.82%
	2		gi|32266870|ref|NP_860902.1|	Helicobacter hepaticus ATCC 51449	E-value = 4.00E-62	Identity = 62.18%
	3		gi|157164178|ref|YP_001466474.1|	Campylobacter concisus 13826	E-value = 4.00E-58	Identity = 57.22%
	4		gi|34557708|ref|NP_907523.1|	Wolinella succinogenes DSM 1740	E-value = 8.00E-54	Identity = 56.45%
	5		gi|118474234|ref|YP_892326.1|	Campylobacter fetus subsp. fetus 82-40	E-value = 9.00E-54	Identity = 54.77%
AB0703	1	*pepA*	gi|149193709|ref|ZP_01870807.1|	Caminibacter mediatlanticus TB-2	E-value = 3.00E-130	Identity = 56.28%
	2		gi|34557709|ref|NP_907524.1|	Wolinella succinogenes DSM 1740	E-value = 4.00E-126	Identity = 51.06%
	3		gi|152990453|ref|YP_001356175.1|	Nitratiruptor sp. SB155-2	E-value = 4.00E-125	Identity = 56.56%
	4		gi|157164548|ref|YP_001466473.1|	Campylobacter concisus 13826	E-value = 2.00E-124	Identity = 54.91%
	5		gi|78777318|ref|YP_393633.1|	Sulfuromonas denitrificans ATCC 33889	E-value = 1.00E-123	Identity = 52.95%

AB0705	1		gi|78777741|ref|YP_394056.1|	Sulfuromonas denitrificans ATCC 33889	E-value = 1.00E-06	Identity = 31.18%
	2		gi|126668448|ref|ZP_01739404.1|	Marinobacter sp. ELB17	E-value = 2.00E-06	Identity = 26.37%
AB0706	1	*exbD2*	gi|78776478|ref|YP_392793.1|	Sulfuromonas denitrificans ATCC 33889	E-value = 2.00E-20	Identity = 42.40%
	2		gi|109946886|ref|YP_664114.1|	Helicobacter acinonychis str. Sheeba	E-value = 4.00E-20	Identity = 44.35%
	3		gi|15645953|ref|NP_208132.1|	Helicobacter pylori 26695	E-value = 5.00E-20	Identity = 44.35%
	4		gi|15612324|ref|NP_223977.1|	Helicobacter pylori J99	E-value = 5.00E-20	Identity = 44.35%
	5		gi|108563712|ref|YP_628028.1|	Helicobacter pylori HPAG1	E-value = 6.00E-20	Identity = 44.35%
AB0707	1	*exbB2*	gi|78776479|ref|YP_392794.1|	Sulfuromonas denitrificans ATCC 33889	E-value = 1.00E-31	Identity = 57.86%
	2		gi|46156301|ref|ZP_00133119.2|	Haemophilus somnus 2336	E-value = 1.00E-28	Identity = 56.30%
	3		gi|118744579|ref|ZP_01592570.1|	Geobacter lovleyi SZ	E-value = 8.00E-28	Identity = 55.47%
	4		gi|52424570|ref|YP_087707.1|	Mannheimia succiniciproducens MBEL55E	E-value = 6.00E-27	Identity = 54.81%
	5		gi|152979664|ref|YP_001345293.1|	Actinobacillus succinogenes 130Z	E-value = 9.00E-27	Identity = 54.74%
AB0708	1		gi|78778234|ref|YP_394549.1|	Sulfuromonas denitrificans ATCC 33889	E-value = 3.00E-38	Identity = 31.35%
	2		gi|152990809|ref|YP_001356531.1|	Nitratiruptor sp. SB155-2	E-value = 7.00E-25	Identity = 27.32%
	3		gi|34556739|ref|NP_906554.1|	Wolinella succinogenes DSM 1740	E-value = 9.00E-25	Identity = 25.96%
	4		gi|78777451|ref|YP_393766.1|	Sulfuromonas denitrificans ATCC 33889	E-value = 7.00E-23	Identity = 27.70%
	5		gi|149193919|ref|ZP_01871017.1|	Caminibacter mediatlanticus TB-2	E-value = 3.00E-15	Identity = 25.16%
AB0709	1		gi|110600581|ref|ZP_01388798.1|	Geobacter sp. FRC-32	E-value = 2.00E-70	Identity = 41.08%
	2		gi|78778235|ref|YP_394550.1|	Sulfuromonas denitrificans ATCC 33889	E-value = 7.00E-69	Identity = 42.35%
	3		gi|15889118|ref|NP_354799.1|	Agrobacterium tumefaciens str. C58	E-value = 2.00E-68	Identity = 38.90%
	4		gi|17935705|ref|NP_532495.1|	Agrobacterium tumefaciens str. C58	E-value = 2.00E-68	Identity = 38.90%
	5		gi|116748526|ref|YP_845213.1|	Syntrophobacter fumaroxidans MPOB	E-value = 6.00E-66	Identity = 37.66%
AB0710	1		gi|78778236|ref|YP_394551.1|	Sulfuromonas denitrificans ATCC 33889	E-value = 3.00E-81	Identity = 64.19%
	2		gi|89901955|ref|YP_524426.1|	Rhodoferax ferrireducens T118	E-value = 5.00E-79	Identity = 66.96%
	3		gi|114562988|ref|YP_750501.1|	Shewanella frigidimarina NCIMB 400	E-value = 1.00E-76	Identity = 62.13%
	4		gi|71909528|ref|YP_287115.1|	Dechloromonas aromatica RCB	E-value = 2.00E-76	Identity = 69.55%
	5		gi|148555065|ref|YP_001262647.1|	Sphingomonas wittichii RW1	E-value = 3.00E-76	Identity = 66.22%
AB0711	1		gi|78778237|ref|YP_394552.1|	Sulfuromonas denitrificans ATCC 33889	E-value = 4.00E-126	Identity = 57.82%
	2		gi|114562987|ref|YP_750500.1|	Shewanella frigidimarina NCIMB 400	E-value = 3.00E-109	Identity = 52.11%
	3		gi|15889120|ref|NP_354801.1|	Agrobacterium tumefaciens str. C58	E-value = 8.00E-109	Identity = 53.40%
	4		gi|17935707|ref|NP_532497.1|	Agrobacterium tumefaciens str. C58	E-value = 7.00E-108	Identity = 53.40%
	5		gi|85860349|ref|YP_462551.1|	Syntrophus aciditrophicus SB	E-value = 9.00E-106	Identity = 51.36%
AB0712	1		gi|72090049|ref|XP_788280.1|	Strongylocentrotus purpuratus	E-value = 2.00E-06	Identity = 28.00%
AB0714	1		gi|34557896|ref|NP_907711.1|	Wolinella succinogenes DSM 1740	E-value = 0	Identity = 57.22%
	2		gi|157165391|ref|YP_001466946.1|	Campylobacter concisus 13826	E-value = 0	Identity = 61.88%
	3		gi|154175545|ref|YP_001408094.1|	Campylobacter curvus 525.92	E-value = 0	Identity = 59.71%
	4		gi|118475026|ref|YP_892006.1|	Campylobacter fetus subsp. fetus 82-40	E-value = 0	Identity = 59.46%
	5		gi|154149333|ref|YP_001405955.1|	Campylobacter hominis ATCC BAA-381	E-value = 0	Identity = 53.24%
AB0715	1		gi|34557897|ref|NP_907712.1|	Wolinella succinogenes DSM 1740	E-value = 7.00E-16	Identity = 51.58%
	2		gi|154174983|ref|YP_001408095.1|	Campylobacter curvus 525.92	E-value = 1.00E-11	Identity = 47.92%
	3		gi|118474798|ref|YP_892007.1|	Campylobacter fetus subsp. fetus 82-40	E-value = 9.00E-09	Identity = 47.87%
AB0716	1		gi|34557898|ref|NP_907713.1|	Wolinella succinogenes DSM 1740	E-value = 3.00E-16	Identity = 54.95%
AB0717	1			***** No matches found *****		
AB0718	1		gi|67159171|ref|ZP_00419877.1|	Azotobacter vinelandii AvOP	E-value = 7.00E-22	Identity = 30.09%
AB0719	1		gi|157165427|ref|YP_001466941.1|	Campylobacter concisus 13826	E-value = 3.00E-22	Identity = 50.98%
	2		gi|154174615|ref|YP_001408099.1|	Campylobacter curvus 525.92	E-value = 4.00E-20	Identity = 50.00%
	3		gi|34557901|ref|NP_907716.1|	Wolinella succinogenes DSM 1740	E-value = 4.00E-18	Identity = 42.72%
	4		gi|118475457|ref|YP_892011.1|	Campylobacter fetus subsp. fetus 82-40	E-value = 4.00E-13	Identity = 40.86%
	5		gi|154149520|ref|YP_001405959.1|	Campylobacter hominis ATCC BAA-381	E-value = 8.00E-11	Identity = 35.87%

AB0720	1	*fur1*	gi|57168539|ref|ZP_00367672.1|	Campylobacter coli RM2228	E-value = 6.00E-26	Identity = 40.14%
	2		gi|15791767|ref|NP_281590.1|	Campylobacter jejuni subsp. jejuni NCTC 11168	E-value = 7.00E-26	Identity = 40.69%
	3		gi|57237455|ref|YP_178468.1|	Campylobacter jejuni subsp. jejuni RM1221	E-value = 8.00E-26	Identity = 41.33%
	4		gi|511113|emb|CAA84528.1|	Campylobacter jejuni	E-value = 1.00E-25	Identity = 40.69%
	5		gi|57241807|ref|ZP_00369751.1|	Campylobacter lari RM2100	E-value = 1.00E-25	Identity = 40.14%
AB0721	1			***** No matches found *****		
AB0722	1	*fumC*	gi|118474555|ref|YP_892133.1|	Campylobacter fetus subsp. fetus 82-40	E-value = 0	Identity = 77.11%
	2		gi|154173867|ref|YP_001407615.1|	Campylobacter curvus 525.92	E-value = 0	Identity = 76.89%
	3		gi|152992171|ref|YP_001357892.1|	Sulfurovum sp. NBC37-1	E-value = 0	Identity = 71.27%
	4		gi|78777247|ref|YP_393562.1|	Sulfuromonas denitrificans ATCC 33889	E-value = 0	Identity = 67.60%
	5		gi|148926719|ref|ZP_01810399.1|	Campylobacter jejuni subsp. jejuni CG8486	E-value = 4.00E-174	Identity = 66.52%
AB0723	1			***** No matches found *****		
AB0724	1			***** No matches found *****		
AB0725	1		gi|34558244|ref|NP_908059.1|	Wolinella succinogenes DSM 1740	E-value = 5.00E-43	Identity = 37.66%
	2		gi|152990261|ref|YP_001355983.1|	Nitratiruptor sp. SB155-2	E-value = 3.00E-25	Identity = 32.72%
	3		gi|152993617|ref|YP_001359338.1|	Sulfurovum sp. NBC37-1	E-value = 1.00E-24	Identity = 31.88%
	4		gi|78776815|ref|YP_393130.1|	Sulfuromonas denitrificans ATCC 33889	E-value = 2.00E-22	Identity = 26.75%
	5		gi|13472734|ref|NP_104301.1|	Mesorhizobium loti MAFF303099	E-value = 2.00E-12	Identity = 27.04%
AB0726	1	*bfrE*	gi|114798931|ref|YP_760813.1|	Hyphomonas neptunium ATCC 15444	E-value = 5.00E-142	Identity = 44.70%
	2		gi|134096547|ref|YP_001101622.1|	Herminiimonas arsenicoxydans	E-value = 3.00E-138	Identity = 41.34%
	3		gi|152980420|ref|YP_001355311.1|	Janthinobacterium sp. Marseille	E-value = 5.00E-135	Identity = 42.95%
	4		gi|152980708|ref|YP_001355309.1|	Janthinobacterium sp. Marseille	E-value = 4.00E-130	Identity = 41.38%
	5		gi|124268908|ref|YP_001022912.1|	Methylibium petroleiphilum PM1	E-value = 1.00E-129	Identity = 40.33%
AB0727	1		gi|124483394|emb|CAM32570.1|	Herbaspirillum seropedicae	E-value = 1.00E-73	Identity = 57.52%
	2		gi|152979871|ref|YP_001355310.1|	Janthinobacterium sp. Marseille	E-value = 6.00E-69	Identity = 52.23%
	3		gi|118049576|ref|ZP_01518127.1|	Comamonas testosteroni KF-1	E-value = 7.00E-69	Identity = 52.00%
	4		gi|134096546|ref|YP_001101621.1|	Herminiimonas arsenicoxydans	E-value = 4.00E-68	Identity = 52.47%
	5		gi|124268909|ref|YP_001022913.1|	Methylibium petroleiphilum PM1	E-value = 2.00E-67	Identity = 50.88%
AB0728	1		gi|146305839|ref|YP_001186304.1|	Pseudomonas mendocina ymp	E-value = 5.00E-103	Identity = 48.61%
	2		gi|67156232|ref|ZP_00417858.1|	Azotobacter vinelandii AvOP	E-value = 2.00E-102	Identity = 48.89%
	3		gi|154250834|ref|YP_001411658.1|	Parvibaculum lavamentivorans DS-1	E-value = 3.00E-97	Identity = 46.70%
	4		gi|75674899|ref|YP_317320.1|	Nitrobacter winogradskyi Nb-255	E-value = 3.00E-95	Identity = 49.30%
	5		gi|121605455|ref|YP_982784.1|	Polaromonas naphthalenivorans CJ2	E-value = 4.00E-89	Identity = 44.38%
AB0729	1	*irgA*	gi|120556201|ref|YP_960552.1|	Marinobacter aquaeolei VT8	E-value = 6.00E-117	Identity = 39.24%
	2		gi|37526740|ref|NP_930084.1|	Photorhabdus luminescens subsp. laumondii TTO1	E-value = 3.00E-112	Identity = 36.35%
	3		gi|92114796|ref|YP_574724.1|	Chromohalobacter salexigens DSM 3043	E-value = 2.00E-110	Identity = 37.41%
	4		gi|153002619|ref|YP_001368300.1|	Shewanella baltica OS185	E-value = 1.00E-109	Identity = 37.56%
	5		gi|113950813|ref|ZP_01436425.1|	Shewanella baltica OS195	E-value = 2.00E-109	Identity = 37.56%
AB0730	1		gi|152976121|ref|YP_001375638.1|	Bacillus cereus subsp. cytotoxis NVH 391-98	E-value = 5.00E-32	Identity = 37.80%
	2		gi|154687307|ref|YP_001422468.1|	Bacillus amyloliquefaciens FZB42	E-value = 1.00E-31	Identity = 37.44%
	3		gi|49479432|ref|YP_037799.1|	Bacillus thuringiensis serovar konkukian str. 97-27	E-value = 1.00E-30	Identity = 35.87%
	4		gi|118478955|ref|YP_896106.1|	Bacillus thuringiensis str. Al Hakam	E-value = 2.00E-30	Identity = 35.87%
	5		gi|30263736|ref|NP_846113.1|	Bacillus anthracis str. Ames	E-value = 5.00E-30	Identity = 35.87%
AB0731	1		gi|46241706|gb|AAS83091.1|	Azospirillum brasilense	E-value = 5.00E-40	Identity = 29.08%
	2		gi|34763057|ref|ZP_00144031.1|	Fusobacterium nucleatum subsp. vincentii ATCC 49256	E-value = 2.00E-19	Identity = 27.34%
AB0732	1		gi|34557763|ref|NP_907578.1|	Wolinella succinogenes DSM 1740	E-value = 2.00E-32	Identity = 33.33%
	2		gi|34557758|ref|NP_907573.1|	Wolinella succinogenes DSM 1740	E-value = 6.00E-27	Identity = 29.09%
	3		gi|95928256|ref|ZP_01311004.1|	Desulfuromonas acetoxidans DSM 684	E-value = 2.00E-17	Identity = 27.80%
	4		gi|21241046|ref|NP_640628.1|	Xanthomonas axonopodis pv. citri str. 306	E-value = 4.00E-17	Identity = 29.25%

	5		gi|78045836|ref|YP_362011.1|	Xanthomonas campestris pv. vesicatoria str. 85-10	E-value = 4.00E-17	Identity = 28.77%
AB0733	1	*cysJ*	gi|121529643|ref|ZP_01662254.1|	Ralstonia pickettii 12J	E-value = 1.00E-41	Identity = 27.45%
	2		gi|86751344|ref|YP_487840.1|	Rhodopseudomonas palustris HaA2	E-value = 1.00E-38	Identity = 27.45%
	3		gi|154173846|ref|YP_001407610.1|	Campylobacter curvus 525.92	E-value = 1.00E-36	Identity = 28.37%
	4		gi|27383078|ref|NP_774607.1|	Bradyrhizobium japonicum USDA 110	E-value = 4.00E-34	Identity = 25.88%
	5		gi|154173992|ref|YP_001409200.1|	Campylobacter curvus 525.92	E-value = 9.00E-33	Identity = 27.05%
AB0734	1		gi|57242216|ref|ZP_00370155.1|	Campylobacter upsaliensis RM3195	E-value = 3.00E-16	Identity = 68.75%
	2		gi|121612197|ref|YP_001000590.1|	Campylobacter jejuni subsp. jejuni 81-176	E-value = 4.00E-15	Identity = 69.84%
	3		gi|15792245|ref|NP_282068.1|	Campylobacter jejuni subsp. jejuni NCTC 11168	E-value = 5.00E-15	Identity = 69.84%
	4		gi|57504974|ref|ZP_00370926.1|	Campylobacter coli RM2228	E-value = 7.00E-15	Identity = 69.84%
	5		gi|153951104|ref|YP_001398014.1|	Campylobacter jejuni subsp. doylei 269.97	E-value = 4.00E-14	Identity = 68.25%
AB0735	1	*cstA*	gi|57242215|ref|ZP_00370154.1|	Campylobacter upsaliensis RM3195	E-value = 0	Identity = 85.9%
	2		gi|57504975|ref|ZP_00370927.1|	Campylobacter coli RM2228	E-value = 0	Identity = 83.91%
	3		gi|121613419|ref|YP_001000591.1|	Campylobacter jejuni subsp. jejuni 81-176	E-value = 0	Identity = 84.48%
	4		gi|148926598|ref|ZP_01810280.1|	Campylobacter jejuni subsp. jejuni CG8486	E-value = 0	Identity = 84.2%
	5		gi|57237745|ref|YP_178993.1|	Campylobacter jejuni subsp. jejuni RM1221	E-value = 0	Identity = 84.48%
AB0736	1		gi|152990452|ref|YP_001356174.1|	Nitratiruptor sp. SB155-2	E-value = 3.00E-152	Identity = 75.75%
	2		gi|152992243|ref|YP_001357964.1|	Sulfurovum sp. NBC37-1	E-value = 1.00E-143	Identity = 71.47%
	3		gi|154175179|ref|YP_001407931.1|	Campylobacter curvus 525.92	E-value = 5.00E-142	Identity = 71.47%
	4		gi|149193707|ref|ZP_01870805.1|	Caminibacter mediatlanticus TB-2	E-value = 2.00E-141	Identity = 70.68%
	5		gi|157164621|ref|YP_001466472.1|	Campylobacter concisus 13826	E-value = 4.00E-140	Identity = 70.11%
AB0737	1		gi|78777397|ref|YP_393712.1|	Sulfuromonas denitrificans ATCC 33889	E-value = 2.00E-56	Identity = 45.32%
	2		gi|78776743|ref|YP_393058.1|	Sulfuromonas denitrificans ATCC 33889	E-value = 8.00E-34	Identity = 32.95%
	3		gi|154175137|ref|YP_001407999.1|	Campylobacter curvus 525.92	E-value = 5.00E-30	Identity = 31.23%
	4		gi|157165151|ref|YP_001467161.1|	Campylobacter concisus 13826	E-value = 5.00E-28	Identity = 30.86%
	5		gi|34558171|ref|NP_907986.1|	Wolinella succinogenes DSM 1740	E-value = 1.00E-26	Identity = 27.88%
AB0738	1		gi|157165659|ref|YP_001466364.1|	Campylobacter concisus 13826	E-value = 1.00E-51	Identity = 50.24%
	2		gi|154148067|ref|YP_001406809.1|	Campylobacter hominis ATCC BAA-381	E-value = 8.00E-51	Identity = 47.80%
	3		gi|34556607|ref|NP_906422.1|	Wolinella succinogenes DSM 1740	E-value = 4.00E-50	Identity = 47.80%
	4		gi|15791508|ref|NP_281331.1|	Campylobacter jejuni subsp. jejuni NCTC 11168	E-value = 7.00E-49	Identity = 50.24%
	5		gi|86151188|ref|ZP_01069403.1|	Campylobacter jejuni subsp. jejuni 260.94	E-value = 8.00E-49	Identity = 50.24%
AB0739	1			***** No matches found *****		
AB0740	1		gi|78188262|ref|YP_378600.1|	Chlorobium chlorochromatii CaD3	E-value = 1.00E-30	Identity = 59.80%
	2		gi|149927986|ref|ZP_01916235.1|	Limnobacter sp. MED105	E-value = 2.00E-29	Identity = 55.34%
	3		gi|78778296|ref|YP_394611.1|	Sulfuromonas denitrificans ATCC 33889	E-value = 6.00E-28	Identity = 51.46%
	4		gi|77918942|ref|YP_356757.1|	Pelobacter carbinolicus DSM 2380	E-value = 9.00E-27	Identity = 50.96%
	5		gi|148264277|ref|YP_001230983.1|	Geobacter uraniumreducens Rf4	E-value = 1.00E-26	Identity = 52.43%
AB0741	1		gi|34556998|ref|NP_906813.1|	Wolinella succinogenes DSM 1740	E-value = 2.00E-35	Identity = 40.69%
AB0742	1			***** No matches found *****		
AB0743	1	*thiL*	gi|152990440|ref|YP_001356162.1|	Nitratiruptor sp. SB155-2	E-value = 4.00E-75	Identity = 51.65%
	2		gi|78777229|ref|YP_393544.1|	Sulfuromonas denitrificans ATCC 33889	E-value = 7.00E-73	Identity = 51.65%
	3		gi|57168997|ref|ZP_00368126.1|	Campylobacter coli RM2228	E-value = 3.00E-72	Identity = 54.74%
	4		gi|86152891|ref|ZP_01071096.1|	Campylobacter jejuni subsp. jejuni HB93-13	E-value = 1.00E-71	Identity = 55.11%
	5		gi|148925630|ref|ZP_01809318.1|	Campylobacter jejuni subsp. jejuni CG8486	E-value = 9.00E-71	Identity = 54.38%
AB0744	1	*truD*	gi|78777230|ref|YP_393545.1|	Sulfuromonas denitrificans ATCC 33889	E-value = 3.00E-104	Identity = 54.78%
	2		gi|152990441|ref|YP_001356163.1|	Nitratiruptor sp. SB155-2	E-value = 3.00E-103	Identity = 54.73%
	3		gi|34557593|ref|NP_907408.1|	Wolinella succinogenes DSM 1740	E-value = 1.00E-95	Identity = 51.97%
	4		gi|152992784|ref|YP_001358505.1|	Sulfurovum sp. NBC37-1	E-value = 1.00E-91	Identity = 51.18%
	5		gi|32267014|ref|NP_861046.1|	Helicobacter hepaticus ATCC 51449	E-value = 4.00E-87	Identity = 44.82%

AB0745	1			***** No matches found *****		
AB0746	1			***** No matches found *****		
AB0747	1	*ruvA*	gi|152992417|ref|YP_001358138.1|	Sulfurovum sp. NBC37-1	E-value = 4.00E-55	Identity = 63.98%
	2		gi|152990842|ref|YP_001356564.1|	Nitratiruptor sp. SB155-2	E-value = 2.00E-54	Identity = 60.00%
	3		gi|34556867|ref|NP_906682.1|	Wolinella succinogenes DSM 1740	E-value = 2.00E-48	Identity = 53.76%
	4		gi|78777407|ref|YP_393722.1|	Sulfuromonas denitrificans ATCC 33889	E-value = 2.00E-46	Identity = 56.68%
	5		gi|32266013|ref|NP_860045.1|	Helicobacter hepaticus ATCC 51449	E-value = 2.00E-37	Identity = 47.18%
AB0748	1	*ddlA*	gi|34556865|ref|NP_906680.1|	Wolinella succinogenes DSM 1740	E-value = 5.00E-105	Identity = 57.47%
	2		gi|152991773|ref|YP_001357494.1|	Sulfurovum sp. NBC37-1	E-value = 5.00E-101	Identity = 56.73%
	3		gi|32265630|ref|NP_859662.1|	Helicobacter hepaticus ATCC 51449	E-value = 1.00E-96	Identity = 54.26%
	4		gi|153952610|ref|YP_001398281.1|	Campylobacter jejuni subsp. doylei 269.97	E-value = 2.00E-91	Identity = 51.72%
	5		gi|86152116|ref|ZP_01070328.1|	Campylobacter jejuni subsp. jejuni 260.94	E-value = 7.00E-91	Identity = 52.01%
AB0749	1		gi|152990838|ref|YP_001356560.1|	Nitratiruptor sp. SB155-2	E-value = 1.00E-67	Identity = 54.04%
	2		gi|154174769|ref|YP_001408217.1|	Campylobacter curvus 525.92	E-value = 3.00E-64	Identity = 53.36%
	3		gi|78777405|ref|YP_393720.1|	Sulfuromonas denitrificans ATCC 33889	E-value = 1.00E-62	Identity = 52.54%
	4		gi|152992425|ref|YP_001358146.1|	Sulfurovum sp. NBC37-1	E-value = 2.00E-60	Identity = 50.42%
	5		gi|118475498|ref|YP_891985.1|	Campylobacter fetus subsp. fetus 82-40	E-value = 3.00E-57	Identity = 50.21%
AB0750	1	*murF*	gi|78777404|ref|YP_393719.1|	Sulfuromonas denitrificans ATCC 33889	E-value = 3.00E-129	Identity = 50.95%
	2		gi|34556863|ref|NP_906678.1|	Wolinella succinogenes DSM 1740	E-value = 5.00E-121	Identity = 46.56%
	3		gi|157165282|ref|YP_001466906.1|	Campylobacter concisus 13826	E-value = 1.00E-118	Identity = 49.79%
	4		gi|152992426|ref|YP_001358147.1|	Sulfurovum sp. NBC37-1	E-value = 3.00E-118	Identity = 45.77%
	5		gi|152990837|ref|YP_001356559.1|	Nitratiruptor sp. SB155-2	E-value = 1.00E-117	Identity = 46.86%
AB0751	1		gi|78777401|ref|YP_393716.1|	Sulfuromonas denitrificans ATCC 33889	E-value = 5.00E-45	Identity = 50.63%
	2		gi|57505406|ref|ZP_00371334.1|	Campylobacter upsaliensis RM3195	E-value = 5.00E-44	Identity = 55.19%
	3		gi|157164691|ref|YP_001466732.1|	Campylobacter concisus 13826	E-value = 6.00E-43	Identity = 54.19%
	4		gi|57240337|ref|ZP_00368286.1|	Campylobacter lari RM2100	E-value = 7.00E-43	Identity = 56.49%
	5		gi|152990835|ref|YP_001356557.1|	Nitratiruptor sp. SB155-2	E-value = 4.00E-42	Identity = 55.97%
AB0752	1			***** No matches found *****		
AB0753	1		gi|119510183|ref|ZP_01629321.1|	Nodularia spumigena CCY9414	E-value = 1.00E-07	Identity = 25.00%
AB0754	1	*rpsJ*	gi|154174951|ref|YP_001409154.1|	Campylobacter curvus 525.92	E-value = 7.00E-38	Identity = 80.20%
	2		gi|152989971|ref|YP_001355693.1|	Nitratiruptor sp. SB155-2	E-value = 1.00E-37	Identity = 85.44%
	3		gi|154148252|ref|YP_001405696.1|	Campylobacter hominis ATCC BAA-381	E-value = 1.00E-36	Identity = 77.67%
	4		gi|153952248|ref|YP_001398987.1|	Campylobacter jejuni subsp. doylei 269.97	E-value = 1.00E-36	Identity = 81.55%
	5		gi|78776486|ref|YP_392801.1|	Sulfuromonas denitrificans ATCC 33889	E-value = 1.00E-36	Identity = 90.10%
AB0755	1	*rplC*	gi|78776487|ref|YP_392802.1|	Sulfuromonas denitrificans ATCC 33889	E-value = 3.00E-70	Identity = 73.30%
	2		gi|34558027|ref|NP_907842.1|	Wolinella succinogenes DSM 1740	E-value = 3.00E-66	Identity = 71.35%
	3		gi|57238730|ref|YP_179845.1|	Campylobacter jejuni subsp. jejuni RM1221	E-value = 4.00E-63	Identity = 66.49%
	4		gi|15793010|ref|NP_282833.1|	Campylobacter jejuni subsp. jejuni NCTC 11168	E-value = 4.00E-63	Identity = 65.97%
	5		gi|152989972|ref|YP_001355694.1|	Nitratiruptor sp. SB155-2	E-value = 5.00E-63	Identity = 64.58%
AB0756	1	*rplD*	gi|154174036|ref|YP_001409152.1|	Campylobacter curvus 525.92	E-value = 3.00E-61	Identity = 63.24%
	2		gi|34558026|ref|NP_907841.1|	Wolinella succinogenes DSM 1740	E-value = 3.00E-60	Identity = 61.76%
	3		gi|157164387|ref|YP_001467719.1|	Campylobacter concisus 13826	E-value = 4.00E-59	Identity = 61.27%
	4		gi|15793009|ref|NP_282832.1|	Campylobacter jejuni subsp. jejuni NCTC 11168	E-value = 4.00E-59	Identity = 62.75%
	5		gi|121612191|ref|YP_001001350.1|	Campylobacter jejuni subsp. jejuni 81-176	E-value = 5.00E-59	Identity = 62.75%
AB0757	1	*rplW*	gi|118474455|ref|YP_891246.1|	Campylobacter fetus subsp. fetus 82-40	E-value = 1.00E-30	Identity = 73.12%
	2		gi|57504696|ref|ZP_00370774.1|	Campylobacter coli RM2228	E-value = 6.00E-30	Identity = 70.97%
	3		gi|86152332|ref|ZP_01070543.1|	Campylobacter jejuni subsp. jejuni 260.94	E-value = 7.00E-30	Identity = 70.97%
	4		gi|78776489|ref|YP_392804.1|	Sulfuromonas denitrificans ATCC 33889	E-value = 7.00E-30	Identity = 70.65%
	5		gi|15793008|ref|NP_282831.1|	Campylobacter jejuni subsp. jejuni NCTC 11168	E-value = 3.00E-29	Identity = 68.82%

AB0758	1	*rplB*	gi|152989975|ref|YP_001355697.1|	Nitratiruptor sp. SB155-2	E-value = 4.00E-122	Identity = 81.45%
	2		gi|152993896|ref|YP_001359617.1|	Sulfurovum sp. NBC37-1	E-value = 2.00E-121	Identity = 79.64%
	3		gi|118474857|ref|YP_891247.1|	Campylobacter fetus subsp. fetus 82-40	E-value = 2.00E-115	Identity = 77.74%
	4		gi|57241623|ref|ZP_00369568.1|	Campylobacter lari RM2100	E-value = 5.00E-112	Identity = 75.55%
	5		gi|78776490|ref|YP_392805.1|	Sulfuromonas denitrificans ATCC 33889	E-value = 6.00E-112	Identity = 75.91%
AB0759	1	*rpsS*	gi|118474223|ref|YP_891248.1|	Campylobacter fetus subsp. fetus 82-40	E-value = 1.00E-35	Identity = 80.00%
	2		gi|78776491|ref|YP_392806.1|	Sulfuromonas denitrificans ATCC 33889	E-value = 1.00E-35	Identity = 82.42%
	3		gi|57504694|ref|ZP_00370772.1|	Campylobacter coli RM2228	E-value = 2.00E-35	Identity = 80.00%
	4		gi|157165068|ref|YP_001467716.1|	Campylobacter concisus 13826	E-value = 2.00E-35	Identity = 80.00%
	5		gi|152989976|ref|YP_001355698.1|	Nitratiruptor sp. SB155-2	E-value = 2.00E-35	Identity = 84.44%
AB0760	1	*rplV*	gi|78776492|ref|YP_392807.1|	Sulfuromonas denitrificans ATCC 33889	E-value = 6.00E-29	Identity = 73.53%
	2		gi|152989977|ref|YP_001355699.1|	Nitratiruptor sp. SB155-2	E-value = 5.00E-28	Identity = 69.90%
	3		gi|152993898|ref|YP_001359619.1|	Sulfurovum sp. NBC37-1	E-value = 1.00E-27	Identity = 70.09%
	4		gi|34558022|ref|NP_907837.1|	Wolinella succinogenes DSM 1740	E-value = 1.00E-27	Identity = 67.29%
	5		gi|154173740|ref|YP_001409149.1|	Campylobacter curvus 525.92	E-value = 5.00E-27	Identity = 65.42%
AB0761	1	*rpsC*	gi|78776493|ref|YP_392808.1|	Sulfuromonas denitrificans ATCC 33889	E-value = 5.00E-91	Identity = 78.70%
	2		gi|152989978|ref|YP_001355700.1|	Nitratiruptor sp. SB155-2	E-value = 2.00E-86	Identity = 79.82%
	3		gi|154174323|ref|YP_001409148.1|	Campylobacter curvus 525.92	E-value = 3.00E-86	Identity = 76.50%
	4		gi|152993899|ref|YP_001359620.1|	Sulfurovum sp. NBC37-1	E-value = 4.00E-86	Identity = 75.21%
	5		gi|118474301|ref|YP_891250.1|	Campylobacter fetus subsp. fetus 82-40	E-value = 1.00E-85	Identity = 76.07%
AB0762	1	*rplP*	gi|34558020|ref|NP_907835.1|	Wolinella succinogenes DSM 1740	E-value = 2.00E-56	Identity = 82.98%
	2		gi|109946766|ref|YP_663994.1|	Helicobacter acinonychis str. Sheeba	E-value = 7.00E-55	Identity = 80.85%
	3		gi|15645925|ref|NP_208104.1|	Helicobacter pylori 26695	E-value = 8.00E-55	Identity = 80.14%
	4		gi|15612297|ref|NP_223950.1|	Helicobacter pylori J99	E-value = 2.00E-54	Identity = 79.43%
	5		gi|152989979|ref|YP_001355701.1|	Nitratiruptor sp. SB155-2	E-value = 3.00E-53	Identity = 79.43%
AB0763	1	*rpmC*	gi|152993901|ref|YP_001359622.1|	Sulfurovum sp. NBC37-1	E-value = 2.00E-05	Identity = 72.58%
	2		gi|157164401|ref|YP_001467712.1|	Campylobacter concisus 13826	E-value = 2.00E-05	Identity = 60.66%
	3		gi|118474438|ref|YP_891252.1|	Campylobacter fetus subsp. fetus 82-40	E-value = 3.00E-05	Identity = 62.30%
AB0764	1	*rpsQ*	gi|154174249|ref|YP_001409145.1|	Campylobacter curvus 525.92	E-value = 4.00E-24	Identity = 71.08%
	2		gi|57241617|ref|ZP_00369562.1|	Campylobacter lari RM2100	E-value = 3.00E-23	Identity = 75.90%
	3		gi|157165212|ref|YP_001467711.1|	Campylobacter concisus 13826	E-value = 1.00E-22	Identity = 67.47%
	4		gi|152993902|ref|YP_001359623.1|	Sulfurovum sp. NBC37-1	E-value = 1.00E-22	Identity = 79.49%
	5		gi|78776496|ref|YP_392811.1|	Sulfuromonas denitrificans ATCC 33889	E-value = 1.00E-22	Identity = 76.92%
AB0765	1	*rplN*	gi|34558018|ref|NP_907833.1|	Wolinella succinogenes DSM 1740	E-value = 6.00E-50	Identity = 90.98%
	2		gi|57241616|ref|ZP_00369561.1|	Campylobacter lari RM2100	E-value = 9.00E-50	Identity = 88.52%
	3		gi|57505346|ref|ZP_00371275.1|	Campylobacter upsaliensis RM3195	E-value = 1.00E-49	Identity = 89.34%
	4		gi|149195487|ref|ZP_01872565.1|	Caminibacter mediatlanticus TB-2	E-value = 2.00E-49	Identity = 88.52%
	5		gi|15793000|ref|NP_282823.1|	Campylobacter jejuni subsp. jejuni NCTC 11168	E-value = 2.00E-49	Identity = 87.70%
AB0766	1	*rplX*	gi|57505345|ref|ZP_00371274.1|	Campylobacter upsaliensis RM3195	E-value = 4.00E-15	Identity = 77.63%
	2		gi|152989983|ref|YP_001355705.1|	Nitratiruptor sp. SB155-2	E-value = 7.00E-15	Identity = 76.00%
	3		gi|15792999|ref|NP_282822.1|	Campylobacter jejuni subsp. jejuni NCTC 11168	E-value = 8.00E-15	Identity = 74.03%
	4		gi|107784838|gb|ABF83910.1|	Campylobacter jejuni subsp. doylei	E-value = 1.00E-14	Identity = 74.03%
	5		gi|153951584|ref|YP_001398975.1|	Campylobacter jejuni subsp. doylei 269.97	E-value = 2.00E-14	Identity = 72.73%
AB0767	1	*rplE*	gi|157164844|ref|YP_001467708.1|	Campylobacter concisus 13826	E-value = 1.00E-63	Identity = 71.67%
	2		gi|118474581|ref|YP_891256.1|	Campylobacter fetus subsp. fetus 82-40	E-value = 1.00E-63	Identity = 70.95%
	3		gi|15792998|ref|NP_282821.1|	Campylobacter jejuni subsp. jejuni NCTC 11168	E-value = 3.00E-63	Identity = 71.51%
	4		gi|152989984|ref|YP_001355706.1|	Nitratiruptor sp. SB155-2	E-value = 3.00E-63	Identity = 68.51%
	5		gi|57504686|ref|ZP_00370764.1|	Campylobacter coli RM2228	E-value = 4.00E-63	Identity = 70.95%
AB0768	1	*rpsN*	gi|152993906|ref|YP_001359627.1|	Sulfurovum sp. NBC37-1	E-value = 3.00E-20	Identity = 85.25%

	2		gi|157165464|ref|YP_001467707.1|	Campylobacter concisus 13826	E-value = 8.00E-20	Identity = 81.97%
	3		gi|57505343|ref|ZP_00371272.1|	Campylobacter upsaliensis RM3195	E-value = 8.00E-20	Identity = 80.33%
	4		gi|57241613|ref|ZP_00369558.1|	Campylobacter lari RM2100	E-value = 9.00E-20	Identity = 81.97%
	5		gi|149195484|ref|ZP_01872562.1|	Caminibacter mediatlanticus TB-2	E-value = 2.00E-19	Identity = 85.25%
AB0769	1	*rpsH*	gi|57241612|ref|ZP_00369557.1|	Campylobacter lari RM2100	E-value = 3.00E-39	Identity = 65.91%
	2		gi|152993907|ref|YP_001359628.1|	Sulfurovum sp. NBC37-1	E-value = 4.00E-39	Identity = 62.88%
	3		gi|148926784|ref|ZP_01810463.1|	Campylobacter jejuni subsp. jejuni CG8486	E-value = 4.00E-38	Identity = 62.88%
	4		gi|32266891|ref|NP_860923.1|	Helicobacter hepaticus ATCC 51449	E-value = 4.00E-38	Identity = 63.64%
	5		gi|57504684|ref|ZP_00370762.1|	Campylobacter coli RM2228	E-value = 7.00E-38	Identity = 63.64%
AB0770	1	*rplF*	gi|118475079|ref|YP_891259.1|	Campylobacter fetus subsp. fetus 82-40	E-value = 1.00E-57	Identity = 64.61%
	2		gi|34558014|ref|NP_907829.1|	Wolinella succinogenes DSM 1740	E-value = 1.00E-56	Identity = 63.48%
	3		gi|15792995|ref|NP_282818.1|	Campylobacter jejuni subsp. jejuni NCTC 11168	E-value = 2.00E-55	Identity = 61.24%
	4		gi|154148630|ref|YP_001405712.1|	Campylobacter hominis ATCC BAA-381	E-value = 4.00E-55	Identity = 61.80%
	5		gi|57241611|ref|ZP_00369556.1|	Campylobacter lari RM2100	E-value = 4.00E-55	Identity = 62.92%
AB0771	1	*rplR*	gi|34558013|ref|NP_907828.1|	Wolinella succinogenes DSM 1740	E-value = 2.00E-30	Identity = 67.24%
	2		gi|32266893|ref|NP_860925.1|	Helicobacter hepaticus ATCC 51449	E-value = 5.00E-29	Identity = 64.66%
	3		gi|152989988|ref|YP_001355710.1|	Nitratiruptor sp. SB155-2	E-value = 2.00E-27	Identity = 61.54%
	4		gi|15645916|ref|NP_208095.1|	Helicobacter pylori 26695	E-value = 6.00E-25	Identity = 55.46%
	5		gi|152993909|ref|YP_001359630.1|	Sulfurovum sp. NBC37-1	E-value = 6.00E-25	Identity = 57.66%
AB0772	1	*rpsE*	gi|109946775|ref|YP_664003.1|	Helicobacter acinonychis str. Sheeba	E-value = 5.00E-54	Identity = 77.78%
	2		gi|15612287|ref|NP_223940.1|	Helicobacter pylori J99	E-value = 5.00E-54	Identity = 78.47%
	3		gi|78776504|ref|YP_392819.1|	Sulfuromonas denitrificans ATCC 33889	E-value = 1.00E-53	Identity = 79.45%
	4		gi|32266894|ref|NP_860926.1|	Helicobacter hepaticus ATCC 51449	E-value = 2.00E-53	Identity = 76.71%
	5		gi|152989989|ref|YP_001355711.1|	Nitratiruptor sp. SB155-2	E-value = 5.00E-53	Identity = 78.32%
AB0773	1	*rplO*	gi|152993911|ref|YP_001359632.1|	Sulfurovum sp. NBC37-1	E-value = 2.00E-37	Identity = 71.21%
	2		gi|118474217|ref|YP_891262.1|	Campylobacter fetus subsp. fetus 82-40	E-value = 2.00E-35	Identity = 70.99%
	3		gi|152989990|ref|YP_001355712.1|	Nitratiruptor sp. SB155-2	E-value = 2.00E-35	Identity = 62.88%
	4		gi|154173917|ref|YP_001409136.1|	Campylobacter curvus 525.92	E-value = 3.00E-35	Identity = 68.70%
	5		gi|157165477|ref|YP_001467702.1|	Campylobacter concisus 13826	E-value = 2.00E-34	Identity = 67.94%
AB0774	1	*secY*	gi|57241607|ref|ZP_00369552.1|	Campylobacter lari RM2100	E-value = 4.00E-169	Identity = 78.57%
	2		gi|152989991|ref|YP_001355713.1|	Nitratiruptor sp. SB155-2	E-value = 4.00E-169	Identity = 78.81%
	3		gi|118474381|ref|YP_891263.1|	Campylobacter fetus subsp. fetus 82-40	E-value = 3.00E-167	Identity = 79.76%
	4		gi|121612558|ref|YP_001001332.1|	Campylobacter jejuni subsp. jejuni 81-176	E-value = 3.00E-163	Identity = 74.58%
	5		gi|57504679|ref|ZP_00370757.1|	Campylobacter coli RM2228	E-value = 4.00E-163	Identity = 75.53%
AB0775	1	*map*	gi|152989992|ref|YP_001355714.1|	Nitratiruptor sp. SB155-2	E-value = 8.00E-104	Identity = 70.36%
	2		gi|78776507|ref|YP_392822.1|	Sulfuromonas denitrificans ATCC 33889	E-value = 5.00E-99	Identity = 68.80%
	3		gi|152993913|ref|YP_001359634.1|	Sulfurovum sp. NBC37-1	E-value = 2.00E-94	Identity = 65.22%
	4		gi|121612289|ref|YP_001001294.1|	Campylobacter jejuni subsp. jejuni 81-176	E-value = 2.00E-92	Identity = 65.86%
	5		gi|86152260|ref|ZP_01070471.1|	Campylobacter jejuni subsp. jejuni 260.94	E-value = 3.00E-92	Identity = 65.86%
AB0776	1	*infA*	gi|152993914|ref|YP_001359635.1|	Sulfurovum sp. NBC37-1	E-value = 1.00E-27	Identity = 86.11%
	2		gi|78776508|ref|YP_392823.1|	Sulfuromonas denitrificans ATCC 33889	E-value = 5.00E-27	Identity = 81.94%
	3		gi|152989993|ref|YP_001355715.1|	Nitratiruptor sp. SB155-2	E-value = 9.00E-27	Identity = 87.50%
	4		gi|34558008|ref|NP_907823.1|	Wolinella succinogenes DSM 1740	E-value = 1.00E-26	Identity = 84.72%
	5		gi|32266898|ref|NP_860930.1|	Helicobacter hepaticus ATCC 51449	E-value = 2.00E-26	Identity = 83.33%
AB0777	1		gi|152990436|ref|YP_001356158.1|	Nitratiruptor sp. SB155-2	E-value = 2.00E-43	Identity = 46.20%
	2		gi|149194079|ref|ZP_01871177.1|	Caminibacter mediatlanticus TB-2	E-value = 4.00E-42	Identity = 45.41%
	3		gi|152992742|ref|YP_001358463.1|	Sulfurovum sp. NBC37-1	E-value = 4.00E-42	Identity = 44.33%
	4		gi|78777161|ref|YP_393476.1|	Sulfuromonas denitrificans ATCC 33889	E-value = 7.00E-38	Identity = 43.63%
	5		gi|154248846|ref|YP_001409671.1|	Fervidobacterium nodosum Rt17-B1	E-value = 2.00E-28	Identity = 37.77%

AB0778	1		gi|118474499|ref|YP_891736.1|	Campylobacter fetus subsp. fetus 82-40	E-value = 3.00E-37	Identity = 33.44%
	2		gi|34557600|ref|NP_907415.1|	Wolinella succinogenes DSM 1740	E-value = 7.00E-35	Identity = 30.60%
	3		gi|57241028|ref|ZP_00368975.1|	Campylobacter lari RM2100	E-value = 1.00E-31	Identity = 32.37%
	4		gi|57167871|ref|ZP_00367011.1|	Campylobacter coli RM2228	E-value = 8.00E-30	Identity = 31.49%
	5		gi|86151094|ref|ZP_01069310.1|	Campylobacter jejuni subsp. jejuni 260.94	E-value = 9.00E-30	Identity = 27.72%
AB0779	1	*aspS*	gi|152993093|ref|YP_001358814.1|	Sulfurovum sp. NBC37-1	E-value = 0	Identity = 76.37%
	2		gi|78777284|ref|YP_393599.1|	Sulfuromonas denitrificans ATCC 33889	E-value = 0	Identity = 68.55%
	3		gi|57241119|ref|ZP_00369066.1|	Campylobacter lari RM2100	E-value = 0	Identity = 68.43%
	4		gi|121612173|ref|YP_001000341.1|	Campylobacter jejuni subsp. jejuni 81-176	E-value = 0	Identity = 67.69%
	5		gi|157414919|ref|YP_001482175.1|	Campylobacter jejuni subsp. jejuni 81116	E-value = 0	Identity = 67.69%
AB0780	1		gi|94263847|ref|ZP_01287652.1|	delta proteobacterium MLMS-1	E-value = 3.00E-171	Identity = 38.09%
	2		gi|94266908|ref|ZP_01290563.1|	delta proteobacterium MLMS-1	E-value = 5.00E-171	Identity = 38.41%
	3		gi|119898573|ref|YP_933786.1|	Azoarcus sp. BH72	E-value = 7.00E-157	Identity = 36.71%
	4		gi|91776113|ref|YP_545869.1|	Methylobacillus flagellatus KT	E-value = 3.00E-152	Identity = 36.74%
	5		gi|71906710|ref|YP_284297.1|	Dechloromonas aromatica RCB	E-value = 1.00E-143	Identity = 35.65%
AB0781	1		gi|148323922|gb|EDK89172.1|	Fusobacterium nucleatum subsp. polymorphum ATCC 10953	E-value = 0	Identity = 59.72%
	2		gi|157404678|gb|EDO70714.1|	Dehalococcoides sp. VS	E-value = 0	Identity = 57.04%
	3		gi|34763472|ref|ZP_00144417.1|	Fusobacterium nucleatum subsp. vincentii ATCC 49256	E-value = 0	Identity = 59.54%
	4		gi|19704069|ref|NP_603631.1|	Fusobacterium nucleatum subsp. nucleatum ATCC 25586	E-value = 0	Identity = 59.54%
	5		gi|152993218|ref|YP_001358939.1|	Sulfurovum sp. NBC37-1	E-value = 0	Identity = 57.29%
AB0782	1	*adk1*	gi|28212159|ref|NP_783103.1|	Clostridium tetani E88	E-value = 2.00E-68	Identity = 57.28%
	2		gi|121534654|ref|ZP_01666475.1|	Thermosinus carboxydivorans Nor1	E-value = 6.00E-67	Identity = 54.25%
	3		gi|20089966|ref|NP_616041.1|	Methanosarcina acetivorans C2A	E-value = 9.00E-67	Identity = 56.81%
	4		gi|124522588|ref|ZP_01697117.1|	Bacillus coagulans 36D1	E-value = 1.00E-66	Identity = 55.40%
	5		gi|153940386|ref|YP_001392813.1|	Clostridium botulinum F str. Langeland	E-value = 4.00E-66	Identity = 55.40%
AB0783	1	*adk2*	gi|78777285|ref|YP_393600.1|	Sulfuromonas denitrificans ATCC 33889	E-value = 3.00E-61	Identity = 66.14%
	2		gi|57241118|ref|ZP_00369065.1|	Campylobacter lari RM2100	E-value = 2.00E-59	Identity = 63.78%
	3		gi|34558089|ref|NP_907904.1|	Wolinella succinogenes DSM 1740	E-value = 2.00E-59	Identity = 64.21%
	4		gi|32267270|ref|NP_861302.1|	Helicobacter hepaticus ATCC 51449	E-value = 5.00E-58	Identity = 62.63%
	5		gi|57167665|ref|ZP_00366805.1|	Campylobacter coli RM2228	E-value = 1.00E-56	Identity = 63.44%
AB0784	1		gi|152990515|ref|YP_001356237.1|	Nitratiruptor sp. SB155-2	E-value = 1.00E-70	Identity = 57.33%
	2		gi|34558090|ref|NP_907905.1|	Wolinella succinogenes DSM 1740	E-value = 2.00E-58	Identity = 53.27%
	3		gi|78777288|ref|YP_393603.1|	Sulfuromonas denitrificans ATCC 33889	E-value = 2.00E-57	Identity = 50.00%
	4		gi|152993294|ref|YP_001359015.1|	Sulfurovum sp. NBC37-1	E-value = 1.00E-55	Identity = 46.28%
	5		gi|77164862|ref|YP_343387.1|	Nitrosococcus oceani ATCC 19707	E-value = 6.00E-28	Identity = 38.76%
AB0785	1	*cat*	gi|37526342|ref|NP_929686.1|	Photorhabdus luminescens subsp. laumondii TTO1	E-value = 9.00E-59	Identity = 52.86%
	2		gi|115686|sp|P00484|CAT3_ECOLI	Plasmid R387 (Escherichia coli)	E-value = 9.00E-57	Identity = 49.77%
	3		gi|115683|sp|P22616|CAT2_HAEIN	Haemophilus influenzae	E-value = 2.00E-56	Identity = 46.89%
	4		gi|12084929|ref|NP_073222.1|	Mannheimia haemolytica	E-value = 2.00E-56	Identity = 49.30%
	5		gi|119633061|ref|YP_918951.1|	Vibrio sp. TC68	E-value = 5.00E-56	Identity = 46.41%
AB0786	1		gi|86142577|ref|ZP_01061016.1|	Flavobacterium sp. MED217	E-value = 1.00E-135	Identity = 59.95%
	2		gi|73668313|ref|YP_304328.1|	Methanosarcina barkeri str. Fusaro	E-value = 2.00E-124	Identity = 61.43%
	3		gi|20089298|ref|NP_615373.1|	Methanosarcina acetivorans C2A	E-value = 7.00E-123	Identity = 61.43%
	4		gi|119963254|ref|YP_947785.1|	Arthrobacter aurescens TC1	E-value = 1.00E-82	Identity = 45.64%
	5		gi|116668823|ref|YP_829756.1|	Arthrobacter sp. FB24	E-value = 2.00E-81	Identity = 44.38%
AB0787	1		gi|14626715|gb|AAK71638.1|AF388671_1	Aeromonas hydrophila	E-value = 7.00E-133	Identity = 61.16%
	2		gi|118475707|ref|YP_892440.1|	Campylobacter fetus subsp. fetus 82-40	E-value = 2.00E-127	Identity = 60.58%
	3		gi|154174869|ref|YP_001407341.1|	Campylobacter curvus 525.92	E-value = 5.00E-125	Identity = 62.61%
	4		gi|157163944|ref|YP_001467811.1|	Campylobacter concisus 13826	E-value = 9.00E-122	Identity = 57.41%

	5		gi|118746108|ref|ZP_01594062.1|	Geobacter lovleyi SZ	E-value = 5.00E-115	Identity = 54.65%
AB0788	1		gi|34557576|ref|NP_907391.1|	Wolinella succinogenes DSM 1740	E-value = 1.00E-79	Identity = 32.43%
	2		gi|149926851|ref|ZP_01915110.1|	Limnobacter sp. MED105	E-value = 3.00E-49	Identity = 25.66%
	3		gi|77918940|ref|YP_356755.1|	Pelobacter carbinolicus DSM 2380	E-value = 2.00E-48	Identity = 28.18%
	4		gi|157376246|ref|YP_001474846.1|	Shewanella sediminis HAW-EB3	E-value = 3.00E-48	Identity = 27.92%
	5		gi|114562324|ref|YP_749837.1|	Shewanella frigidimarina NCIMB 400	E-value = 1.00E-47	Identity = 25.51%
AB0789	1	*alr*	gi|152993699|ref|YP_001359420.1|	Sulfurovum sp. NBC37-1	E-value = 6.00E-86	Identity = 48.82%
	2		gi|152990104|ref|YP_001355826.1|	Nitratiruptor sp. SB155-2	E-value = 1.00E-82	Identity = 46.76%
	3		gi|78776580|ref|YP_392895.1|	Sulfuromonas denitrificans ATCC 33889	E-value = 3.00E-81	Identity = 48.67%
	4		gi|154148940|ref|YP_001406018.1|	Campylobacter hominis ATCC BAA-381	E-value = 3.00E-80	Identity = 45.43%
	5		gi|154174920|ref|YP_001408670.1|	Campylobacter curvus 525.92	E-value = 1.00E-78	Identity = 43.53%
AB0790	1	*uvrC*	gi|152991086|ref|YP_001356808.1|	Nitratiruptor sp. SB155-2	E-value = 0	Identity = 55.41%
	2		gi|78776619|ref|YP_392934.1|	Sulfuromonas denitrificans ATCC 33889	E-value = 1.00E-176	Identity = 56.41%
	3		gi|149194567|ref|ZP_01871663.1|	Caminibacter mediatlanticus TB-2	E-value = 2.00E-174	Identity = 55.48%
	4		gi|34557927|ref|NP_907742.1|	Wolinella succinogenes DSM 1740	E-value = 6.00E-173	Identity = 55.92%
	5		gi|152993484|ref|YP_001359205.1|	Sulfurovum sp. NBC37-1	E-value = 9.00E-165	Identity = 51.41%
AB0791	1		gi|34557258|ref|NP_907073.1|	Wolinella succinogenes DSM 1740	E-value = 2.00E-88	Identity = 32.26%
	2		gi|125975509|ref|YP_001039419.1|	Clostridium thermocellum ATCC 27405	E-value = 2.00E-63	Identity = 29.01%
	3		gi|116251086|ref|YP_766924.1|	Rhizobium leguminosarum bv. viciae 3841	E-value = 9.00E-62	Identity = 26.95%
	4		gi|57241752|ref|ZP_00369697.1|	Campylobacter lari RM2100	E-value = 2.00E-58	Identity = 28.08%
	5		gi|32266836|ref|NP_860868.1|	Helicobacter hepaticus ATCC 51449	E-value = 2.00E-57	Identity = 26.60%
AB0792	1		gi|157165318|ref|YP_001466951.1|	Campylobacter concisus 13826	E-value = 2.00E-43	Identity = 32.69%
	2		gi|34558241|ref|NP_908056.1|	Wolinella succinogenes DSM 1740	E-value = 2.00E-40	Identity = 30.25%
	3		gi|34557328|ref|NP_907143.1|	Wolinella succinogenes DSM 1740	E-value = 2.00E-39	Identity = 31.25%
	4		gi|34558440|ref|NP_908255.1|	Wolinella succinogenes DSM 1740	E-value = 2.00E-38	Identity = 27.79%
AB0793	1		gi|34557239|ref|NP_907054.1|	Wolinella succinogenes DSM 1740	E-value = 9.00E-55	Identity = 31.50%
	2		gi|34557328|ref|NP_907143.1|	Wolinella succinogenes DSM 1740	E-value = 2.00E-54	Identity = 31.45%
	3		gi|34558241|ref|NP_908056.1|	Wolinella succinogenes DSM 1740	E-value = 5.00E-54	Identity = 31.29%
	4		gi|21492865|ref|NP_659940.1|	Rhizobium etli CFN 42	E-value = 3.00E-48	Identity = 25.96%
	5		gi|34558248|ref|NP_908063.1|	Wolinella succinogenes DSM 1740	E-value = 4.00E-43	Identity = 32.25%
AB0794	1		gi|34557239|ref|NP_907054.1|	Wolinella succinogenes DSM 1740	E-value = 1.00E-80	Identity = 36.89%
	2		gi|34557328|ref|NP_907143.1|	Wolinella succinogenes DSM 1740	E-value = 3.00E-46	Identity = 30.29%
	3		gi|34558241|ref|NP_908056.1|	Wolinella succinogenes DSM 1740	E-value = 9.00E-45	Identity = 31.09%
	4		gi|57505516|ref|ZP_00371443.1|	Campylobacter upsaliensis RM3195	E-value = 5.00E-43	Identity = 29.66%
AB0795	1		gi|157164104|ref|YP_001467231.1|	Campylobacter concisus 13826	E-value = 7.00E-33	Identity = 36.70%
	2		gi|154174591|ref|YP_001407606.1|	Campylobacter curvus 525.92	E-value = 6.00E-32	Identity = 37.73%
	3		gi|78776459|ref|YP_392774.1|	Sulfuromonas denitrificans ATCC 33889	E-value = 9.00E-32	Identity = 40.28%
	4		gi|34556470|ref|NP_906285.1|	Wolinella succinogenes DSM 1740	E-value = 5.00E-31	Identity = 36.00%
	5		gi|154174786|ref|YP_001408696.1|	Campylobacter curvus 525.92	E-value = 6.00E-31	Identity = 36.70%
AB0796	1		gi|119483261|ref|ZP_01618675.1|	Lyngbya sp. PCC 8106	E-value = 8.00E-40	Identity = 34.62%
	2		gi|67924013|ref|ZP_00517465.1|	Crocosphaera watsonii WH 8501	E-value = 3.00E-34	Identity = 32.87%
	3		gi|126659636|ref|ZP_01730766.1|	Cyanothece sp. CCY0110	E-value = 5.00E-33	Identity = 35.12%
	4		gi|150018233|ref|YP_001310487.1|	Clostridium beijerinckii NCIMB 8052	E-value = 6.00E-33	Identity = 32.34%
	5		gi|16330677|ref|NP_441405.1|	Synechocystis sp. PCC 6803	E-value = 1.00E-32	Identity = 29.97%
AB0797	1		gi|82702320|ref|YP_411886.1|	Nitrosospira multiformis ATCC 25196	E-value = 9.00E-69	Identity = 31.36%
	2		gi|111223594|ref|YP_714388.1|	Frankia alni ACN14a	E-value = 2.00E-67	Identity = 30.09%
AB0798	1		gi|152993037|ref|YP_001358758.1|	Sulfurovum sp. NBC37-1	E-value = 4.00E-47	Identity = 69.17%
	2		gi|149195428|ref|ZP_01872511.1|	Caminibacter mediatlanticus TB-2	E-value = 1.00E-40	Identity = 62.60%
	3		gi|15606620|ref|NP_214000.1|	Aquifex aeolicus VF5	E-value = 2.00E-39	Identity = 62.41%

	4		gi|152991029|ref|YP_001356751.1|	Nitratiruptor sp. SB155-2	E-value = 3.00E-36	Identity = 58.33%
	5		gi|77166101|ref|YP_344626.1|	Nitrosococcus oceani ATCC 19707	E-value = 1.00E-32	Identity = 57.36%
AB0799	1		gi|94498997|ref|ZP_01305535.1|	Oceanobacter sp. RED65	E-value = 1.00E-22	Identity = 30.47%
	2		gi|149276082|ref|ZP_01882227.1|	Pedobacter sp. BAL39	E-value = 3.00E-12	Identity = 27.27%
AB0800	1		gi|152992407|ref|YP_001358128.1|	Sulfurovum sp. NBC37-1	E-value = 2.00E-10	Identity = 42.98%
AB0801	1		gi|154149012|ref|YP_001405757.1|	Campylobacter hominis ATCC BAA-381	E-value = 6.00E-06	Identity = 36.84%
AB0802	1		gi|152994979|ref|YP_001339814.1|	Marinomonas sp. MWYL1	E-value = 0	Identity = 79.76%
	2		gi|88798002|ref|ZP_01113589.1|	Reinekea sp. MED297	E-value = 0	Identity = 81.37%
	3		gi|110835370|ref|YP_694229.1|	Alcanivorax borkumensis SK2	E-value = 0	Identity = 79.38%
	4		gi|119946607|ref|YP_944287.1|	Psychromonas ingrahamii 37	E-value = 0	Identity = 78.2%
	5		gi|88793946|ref|ZP_01109658.1|	Alteromonas macleodii 'Deep ecotype'	E-value = 0	Identity = 77.43%
AB0803	1		gi|90414544|ref|ZP_01222518.1|	Photobacterium profundum 3TCK	E-value = 4.00E-135	Identity = 52.73%
	2		gi|83647208|ref|YP_435643.1|	Hahella chejuensis KCTC 2396	E-value = 6.00E-135	Identity = 52.53%
	3		gi|149191434|ref|ZP_01869684.1|	Vibrio shilonii AK1	E-value = 6.00E-135	Identity = 53.16%
	4		gi|84387635|ref|ZP_00990652.1|	Vibrio splendidus 12B01	E-value = 7.00E-135	Identity = 52.23%
	5		gi|148979616|ref|ZP_01815621.1|	Vibrionales bacterium SWAT-3	E-value = 3.00E-133	Identity = 55.51%
AB0804	1		gi|83647207|ref|YP_435642.1|	Hahella chejuensis KCTC 2396	E-value = 3.00E-135	Identity = 70.11%
	2		gi|94501625|ref|ZP_01308141.1|	Oceanobacter sp. RED65	E-value = 2.00E-126	Identity = 68.91%
	3		gi|118589403|ref|ZP_01546809.1|	Stappia aggregata IAM 12614	E-value = 2.00E-126	Identity = 67.44%
	4		gi|120555909|ref|YP_960260.1|	Marinobacter aquaeolei VT8	E-value = 3.00E-125	Identity = 66.48%
	5		gi|110835372|ref|YP_694231.1|	Alcanivorax borkumensis SK2	E-value = 6.00E-125	Identity = 67.70%
AB0805	1		gi|89093715|ref|ZP_01166662.1|	Oceanospirillum sp. MED92	E-value = 2.00E-88	Identity = 66.53%
	2		gi|120555908|ref|YP_960259.1|	Marinobacter aquaeolei VT8	E-value = 5.00E-88	Identity = 65.86%
	3		gi|152994982|ref|YP_001339817.1|	Marinomonas sp. MWYL1	E-value = 1.00E-87	Identity = 68.18%
	4		gi|126668638|ref|ZP_01739590.1|	Marinobacter sp. ELB17	E-value = 3.00E-87	Identity = 66.27%
	5		gi|114319353|ref|YP_741036.1|	Alkalilimnicola ehrlichei MLHE-1	E-value = 5.00E-87	Identity = 59.35%
AB0806	1		gi|110835374|ref|YP_694233.1|	Alcanivorax borkumensis SK2	E-value = 3.00E-78	Identity = 60.61%
	2		gi|92114430|ref|YP_574358.1|	Chromohalobacter salexigens DSM 3043	E-value = 4.00E-75	Identity = 58.05%
	3		gi|149378300|ref|ZP_01896009.1|	Marinobacter algicola DG893	E-value = 3.00E-74	Identity = 60.34%
	4		gi|149909703|ref|ZP_01898355.1|	Moritella sp. PE36	E-value = 6.00E-74	Identity = 59.31%
	5		gi|120555907|ref|YP_960258.1|	Marinobacter aquaeolei VT8	E-value = 2.00E-73	Identity = 59.48%
AB0807	1		gi|149277418|ref|ZP_01883559.1|	Pedobacter sp. BAL39	E-value = 6.00E-30	Identity = 28.69%
AB0808	1	*ureD*	gi|586169|sp|Q07400|URED_BACSB	Bacillus sp. TB-90	E-value = 1.00E-10	Identity = 25.65%
AB0809	1	*ureAB*	gi|118776872|ref|XP_306932.3|	Anopheles gambiae str. PEST	E-value = 1.00E-62	Identity = 55.56%
	2		gi|15807979|ref|NP_285642.1|	Deinococcus radiodurans R1	E-value = 3.00E-62	Identity = 54.38%
	3		gi|157407679|gb|EDO73680.1|	Methylobacterium populi BJ001	E-value = 3.00E-62	Identity = 57.82%
	4		gi|156451974|ref|ZP_02058347.1|	Methylobacterium chloromethanicum CM4	E-value = 1.00E-61	Identity = 57.62%
	5		gi|85705528|ref|ZP_01036626.1|	Roseovarius sp. 217	E-value = 1.00E-60	Identity = 54.42%
AB0810	1	*ureC*	gi|42782712|ref|NP_979959.1|	Bacillus cereus ATCC 10987	E-value = 0	Identity = 64.5%
	2		gi|125974320|ref|YP_001038230.1|	Clostridium thermocellum ATCC 27405	E-value = 0	Identity = 65.09%
	3		gi|86607525|ref|YP_476287.1|	Synechococcus sp. JA-2-3B'a(2-13)	E-value = 0	Identity = 62.46%
	4		gi|88793954|ref|ZP_01109666.1|	Alteromonas macleodii 'Deep ecotype'	E-value = 0	Identity = 64.49%
	5		gi|148979625|ref|ZP_01815630.1|	Vibrionales bacterium SWAT-3	E-value = 0	Identity = 65.61%
AB0811	1	*ureE*	gi|119946616|ref|YP_944296.1|	Psychromonas ingrahamii 37	E-value = 4.00E-06	Identity = 29.93%
	2		gi|86609467|ref|YP_478229.1|	Synechococcus sp. JA-2-3B'a(2-13)	E-value = 8.00E-06	Identity = 27.13%
	3		gi|15612818|ref|NP_241121.1|	Bacillus halodurans C-125	E-value = 9.00E-06	Identity = 27.74%
AB0812	1	*ureF*	gi|586171|sp|Q07402|UREF_BACSB	Bacillus sp. TB-90	E-value = 9.00E-10	Identity = 25.44%
	2		gi|4545297|gb|AAD22482.1|AF120718_6	Lactobacillus fermentum	E-value = 2.00E-09	Identity = 25.42%
	3		gi|92089298|ref|ZP_01274246.1|	Lactobacillus reuteri 100-23	E-value = 6.00E-09	Identity = 25.23%

	4		gi|72004425|ref|XP_787021.1|	Strongylocentrotus purpuratus	E-value = 3.00E-06	Identity = 25.42%
AB0813	1	*ureG*	gi|15612820|ref|NP_241123.1|	Bacillus halodurans C-125	E-value = 2.00E-58	Identity = 59.16%
	2		gi|86608358|ref|YP_477120.1|	Synechococcus sp. JA-2-3B'a(2-13)	E-value = 5.00E-58	Identity = 58.12%
	3		gi|75910832|ref|YP_325128.1|	Anabaena variabilis ATCC 29413	E-value = 7.00E-58	Identity = 57.07%
	4		gi|32265911|ref|NP_859943.1|	Helicobacter hepaticus ATCC 51449	E-value = 7.00E-58	Identity = 60.42%
	5		gi|17228230|ref|NP_484778.1|	Nostoc sp. PCC 7120	E-value = 1.00E-57	Identity = 57.07%
AB0814	1		gi|71908778|ref|YP_286365.1|	Dechloromonas aromatica RCB	E-value = 4.00E-23	Identity = 32.97%
AB0815	1		gi|39995315|ref|NP_951266.1|	Geobacter sulfurreducens PCA	E-value = 1.00E-21	Identity = 31.72%
	2		gi|95929697|ref|ZP_01312439.1|	Desulfuromonas acetoxidans DSM 684	E-value = 4.00E-19	Identity = 29.89%
	3		gi|113941432|ref|ZP_01427231.1|	Herpetosiphon aurantiacus ATCC 23779	E-value = 5.00E-15	Identity = 31.25%
	4		gi|149376391|ref|ZP_01894153.1|	Marinobacter algicola DG893	E-value = 6.00E-15	Identity = 31.58%
	5		gi|71737186|ref|YP_276888.1|	Pseudomonas syringae pv. phaseolicola 1448A	E-value = 2.00E-14	Identity = 32.76%
AB0816	1		gi|78776735|ref|YP_393050.1|	Sulfuromonas denitrificans ATCC 33889	E-value = 0	Identity = 65.63%
	2		gi|34557170|ref|NP_906985.1|	Wolinella succinogenes DSM 1740	E-value = 0	Identity = 55.17%
	3		gi|146305861|ref|YP_001186326.1|	Pseudomonas mendocina ymp	E-value = 0	Identity = 54.63%
	4		gi|34495889|ref|NP_900104.1|	Chromobacterium violaceum ATCC 12472	E-value = 0	Identity = 53.64%
	5		gi|153007721|ref|YP_001368936.1|	Ochrobactrum anthropi ATCC 49188	E-value = 0	Identity = 52.92%
AB0817	1		gi|78776734|ref|YP_393049.1|	Sulfuromonas denitrificans ATCC 33889	E-value = 2.00E-63	Identity = 43.30%
	2		gi|34557169|ref|NP_906984.1|	Wolinella succinogenes DSM 1740	E-value = 2.00E-48	Identity = 32.01%
	3		gi|32265674|ref|NP_859706.1|	Helicobacter hepaticus ATCC 51449	E-value = 6.00E-42	Identity = 32.10%
	4		gi|149190753|ref|ZP_01869019.1|	Vibrio shilonii AK1	E-value = 2.00E-41	Identity = 33.72%
	5		gi|91775946|ref|YP_545702.1|	Methylobacillus flagellatus KT	E-value = 4.00E-41	Identity = 35.52%
AB0818	1		gi|78776733|ref|YP_393048.1|	Sulfuromonas denitrificans ATCC 33889	E-value = 1.00E-69	Identity = 40.00%
	2		gi|78777639|ref|YP_393954.1|	Sulfuromonas denitrificans ATCC 33889	E-value = 2.00E-33	Identity = 28.88%
	3		gi|152991869|ref|YP_001357590.1|	Sulfurovum sp. NBC37-1	E-value = 4.00E-33	Identity = 29.38%
	4		gi|15645230|ref|NP_207400.1|	Helicobacter pylori 26695	E-value = 7.00E-26	Identity = 25.13%
	5		gi|3068785|gb|AAC14432.1|	Helicobacter pylori	E-value = 8.00E-26	Identity = 25.13%
AB0819	1		gi|78776732|ref|YP_393047.1|	Sulfuromonas denitrificans ATCC 33889	E-value = 7.00E-26	Identity = 45.00%
	2		gi|149910873|ref|ZP_01899505.1|	Moritella sp. PE36	E-value = 5.00E-15	Identity = 37.25%
	3		gi|113939473|ref|ZP_01425327.1|	Herpetosiphon aurantiacus ATCC 23779	E-value = 1.00E-11	Identity = 34.59%
	4		gi|115423564|emb|CAJ50100.1|	Bordetella avium 197N	E-value = 4.00E-10	Identity = 29.08%
	5		gi|146339402|ref|YP_001204450.1|	Bradyrhizobium sp. ORS278	E-value = 6.00E-10	Identity = 31.68%
AB0820	1			***** No matches found *****		
AB0821	1		gi|78777553|ref|YP_393868.1|	Sulfuromonas denitrificans ATCC 33889	E-value = 4.00E-17	Identity = 80.65%
	2		gi|34557799|ref|NP_907614.1|	Wolinella succinogenes DSM 1740	E-value = 4.00E-08	Identity = 52.46%
AB0822	1			***** No matches found *****		
AB0823	1			***** No matches found *****		
AB0824	1	*xth*	gi|152992239|ref|YP_001357960.1|	Sulfurovum sp. NBC37-1	E-value = 3.00E-85	Identity = 61.07%
	2		gi|157164085|ref|YP_001465973.1|	Campylobacter concisus 13826	E-value = 7.00E-75	Identity = 54.09%
	3		gi|118475279|ref|YP_892840.1|	Campylobacter fetus subsp. fetus 82-40	E-value = 4.00E-73	Identity = 54.09%
	4		gi|57168378|ref|ZP_00367512.1|	Campylobacter coli RM2228	E-value = 9.00E-72	Identity = 53.31%
	5		gi|57237312|ref|YP_178325.1|	Campylobacter jejuni subsp. jejuni RM1221	E-value = 7.00E-71	Identity = 51.91%
AB0825	1			***** No matches found *****		
AB0826	1		gi|90577398|ref|ZP_01233209.1|	Vibrio angustum S14	E-value = 1.00E-44	Identity = 48.88%
	2		gi|89072261|ref|ZP_01158840.1|	Photobacterium sp. SKA34	E-value = 1.00E-43	Identity = 48.43%
	3		gi|149909876|ref|ZP_01898526.1|	Moritella sp. PE36	E-value = 2.00E-42	Identity = 47.30%
	4		gi|89091888|ref|ZP_01164843.1|	Oceanospirillum sp. MED92	E-value = 7.00E-28	Identity = 36.82%
	5		gi|119503962|ref|ZP_01626043.1|	marine gamma proteobacterium HTCC2080	E-value = 1.00E-25	Identity = 32.34%
AB0827	1		gi|90417182|ref|ZP_01225109.1|	marine gamma proteobacterium HTCC2207	E-value = 2.00E-09	Identity = 45.33%

	2		gi|83856923|ref|ZP_00950452.1|	Croceibacter atlanticus HTCC2559	E-value = 1.00E-08	Identity = 46.67%
	3		gi|86140976|ref|ZP_01059535.1|	Flavobacterium sp. MED217	E-value = 1.00E-08	Identity = 44.59%
	4		gi|126663897|ref|ZP_01734892.1|	Flavobacteria bacterium BAL38	E-value = 9.00E-08	Identity = 43.75%
	5		gi|42522477|ref|NP_967857.1|	Bdellovibrio bacteriovorus HD100	E-value = 9.00E-07	Identity = 40.74%
AB0828	1		gi|153006041|ref|YP_001380366.1|	Anaeromyxobacter sp. Fw109-5	E-value = 2.00E-37	Identity = 45.14%
	2		gi|39997424|ref|NP_953375.1|	Geobacter sulfurreducens PCA	E-value = 5.00E-37	Identity = 50.00%
	3		gi|114773662|ref|ZP_01450697.1|	alpha proteobacterium HTCC2255	E-value = 2.00E-32	Identity = 44.85%
	4		gi|118035152|ref|ZP_01506560.1|	Burkholderia phytofirmans PsJN	E-value = 2.00E-32	Identity = 47.65%
	5		gi|91778750|ref|YP_553958.1|	Burkholderia xenovorans LB400	E-value = 2.00E-32	Identity = 46.06%
AB0829	1		gi|125972928|ref|YP_001036838.1|	Clostridium thermocellum ATCC 27405	E-value = 2.00E-127	Identity = 59.58%
	2		gi|156718437|ref|ZP_02060103.1|	Hydrogenobaculum sp. Y04AAS1	E-value = 3.00E-126	Identity = 63.14%
	3		gi|78221351|ref|YP_383098.1|	Geobacter metallireducens GS-15	E-value = 2.00E-124	Identity = 51.64%
	4		gi|39998418|ref|NP_954369.1|	Geobacter sulfurreducens PCA	E-value = 5.00E-124	Identity = 51.76%
	5		gi|156866715|gb|EDO60087.1|	Clostridium leptum DSM 753	E-value = 3.00E-123	Identity = 51.40%
AB0830	1		gi|106895521|ref|ZP_01362611.1|	Clostridium sp. OhILAs	E-value = 2.00E-28	Identity = 29.29%
	2		gi|134299146|ref|YP_001112642.1|	Desulfotomaculum reducens MI-1	E-value = 8.00E-26	Identity = 28.75%
	3		gi|125972927|ref|YP_001036837.1|	Clostridium thermocellum ATCC 27405	E-value = 3.00E-25	Identity = 29.63%
	4		gi|78221352|ref|YP_383099.1|	Geobacter metallireducens GS-15	E-value = 2.00E-24	Identity = 28.98%
	5		gi|118727500|ref|ZP_01576099.1|	Clostridium cellulolyticum H10	E-value = 3.00E-24	Identity = 30.58%
AB0831	1		gi|78777130|ref|YP_393445.1|	Sulfuromonas denitrificans ATCC 33889	E-value = 5.00E-149	Identity = 63.72%
	2		gi|152990898|ref|YP_001356620.1|	Nitratiruptor sp. SB155-2	E-value = 5.00E-134	Identity = 56.33%
	3		gi|152993698|ref|YP_001359419.1|	Sulfurovum sp. NBC37-1	E-value = 6.00E-128	Identity = 53.85%
	4		gi|34556836|ref|NP_906651.1|	Wolinella succinogenes DSM 1740	E-value = 5.00E-120	Identity = 53.46%
	5		gi|118580482|ref|YP_901732.1|	Pelobacter propionicus DSM 2379	E-value = 3.00E-108	Identity = 47.26%
AB0832	1			***** No matches found *****		
AB0833	1		gi|95930967|ref|ZP_01313696.1|	Desulfuromonas acetoxidans DSM 684	E-value = 2.00E-115	Identity = 57.43%
	2		gi|115377841|ref|ZP_01465029.1|	Stigmatella aurantiaca DW4/3-1	E-value = 2.00E-56	Identity = 33.90%
	3		gi|78356120|ref|YP_387569.1|	Desulfovibrio desulfuricans G20	E-value = 8.00E-56	Identity = 33.05%
	4		gi|86605819|ref|YP_474582.1|	Synechococcus sp. JA-3-3Ab	E-value = 9.00E-49	Identity = 33.71%
	5		gi|124522565|ref|ZP_01697101.1|	Bacillus coagulans 36D1	E-value = 3.00E-45	Identity = 35.87%
AB0834	1		gi|78777133|ref|YP_393448.1|	Sulfuromonas denitrificans ATCC 33889	E-value = 1.00E-37	Identity = 44.74%
	2		gi|148322813|gb|EDK88063.1|	Fusobacterium nucleatum subsp. polymorphum ATCC 10953	E-value = 6.00E-26	Identity = 36.17%
	3		gi|34763624|ref|ZP_00144554.1|	Fusobacterium nucleatum subsp. vincentii ATCC 49256	E-value = 2.00E-25	Identity = 34.89%
	4		gi|77408045|ref|ZP_00784793.1|	Streptococcus agalactiae COH1	E-value = 2.00E-25	Identity = 37.62%
	5		gi|76788683|ref|YP_329732.1|	Streptococcus agalactiae A909	E-value = 3.00E-25	Identity = 37.62%
AB0835	1		gi|78777132|ref|YP_393447.1|	Sulfuromonas denitrificans ATCC 33889	E-value = 2.00E-57	Identity = 44.14%
	2		gi|83814064|ref|YP_444560.1|	Salinibacter ruber DSM 13855	E-value = 2.00E-19	Identity = 26.06%
	3		gi|124009718|ref|ZP_01694389.1|	Microscilla marina ATCC 23134	E-value = 2.00E-17	Identity = 28.12%
	4		gi|29349375|ref|NP_812878.1|	Bacteroides thetaiotaomicron VPI-5482	E-value = 3.00E-17	Identity = 30.45%
	5		gi|126646794|ref|ZP_01719304.1|	Algoriphagus sp. PR1	E-value = 1.00E-16	Identity = 31.14%
AB0836	1		gi|78777131|ref|YP_393446.1|	Sulfuromonas denitrificans ATCC 33889	E-value = 3.00E-79	Identity = 39.74%
	2		gi|124515770|gb|EAY57279.1|	Leptospirillum sp. Group II UBA	E-value = 2.00E-36	Identity = 28.35%
	3		gi|68552563|ref|ZP_00591951.1|	Prosthecochloris aestuarii DSM 271	E-value = 7.00E-28	Identity = 28.05%
	4		gi|68549638|ref|ZP_00589099.1|	Pelodictyon phaeoclathratiforme BU-1	E-value = 3.00E-21	Identity = 25.91%
	5		gi|67939520|ref|ZP_00532019.1|	Chlorobium phaeobacteroides BS1	E-value = 9.00E-21	Identity = 26.82%
AB0837	1	*phrB*	gi|78777808|ref|YP_394123.1|	Sulfuromonas denitrificans ATCC 33889	E-value = 9.00E-97	Identity = 47.26%
	2		gi|34557872|ref|NP_907687.1|	Wolinella succinogenes DSM 1740	E-value = 3.00E-96	Identity = 41.20%
	3		gi|126663929|ref|ZP_01734924.1|	Flavobacteria bacterium BAL38	E-value = 2.00E-80	Identity = 41.76%
	4		gi|86134119|ref|ZP_01052701.1|	Tenacibaculum sp. MED152	E-value = 4.00E-80	Identity = 40.17%

	5		gi|150025093|ref|YP_001295919.1|	Flavobacterium psychrophilum JIP02/86	E-value = 6.00E-79	Identity = 41.32%
AB0838	1		gi|78486202|ref|YP_392127.1|	Thiomicrospira crunogena XCL-2	E-value = 9.00E-65	Identity = 38.99%
	2		gi|34557873|ref|NP_907688.1|	Wolinella succinogenes DSM 1740	E-value = 8.00E-64	Identity = 39.12%
	3		gi|51246429|ref|YP_066313.1|	Desulfotalea psychrophila LSv54	E-value = 2.00E-61	Identity = 40.69%
	4		gi|118578564|ref|YP_899814.1|	Pelobacter propionicus DSM 2379	E-value = 9.00E-59	Identity = 37.70%
	5		gi|46581379|ref|YP_012187.1|	Desulfovibrio vulgaris subsp. vulgaris str. Hildenborough	E-value = 1.00E-57	Identity = 37.11%
AB0839	1		gi|118474211|ref|YP_892191.1|	Campylobacter fetus subsp. fetus 82-40	E-value = 2.00E-53	Identity = 49.00%
	2		gi|152988816|ref|YP_001351575.1|	Pseudomonas aeruginosa PA7	E-value = 2.00E-45	Identity = 37.04%
	3		gi|117923982|ref|YP_864599.1|	Magnetococcus sp. MC-1	E-value = 4.00E-45	Identity = 36.49%
	4		gi|15600659|ref|NP_254153.1|	Pseudomonas aeruginosa PAO1	E-value = 9.00E-45	Identity = 36.73%
	5		gi|149376565|ref|ZP_01894325.1|	Marinobacter algicola DG893	E-value = 4.00E-44	Identity = 38.70%
AB0840	1		gi|57168961|ref|ZP_00368090.1|	Campylobacter coli RM2228	E-value = 6.00E-19	Identity = 39.26%
	2		gi|34557099|ref|NP_906914.1|	Wolinella succinogenes DSM 1740	E-value = 9.00E-14	Identity = 29.70%
	3		gi|153893617|ref|ZP_02014292.1|	Opitutaceae bacterium TAV2	E-value = 2.00E-13	Identity = 29.24%
	4		gi|15894123|ref|NP_347472.1|	Clostridium acetobutylicum ATCC 824	E-value = 4.00E-10	Identity = 33.33%
	5		gi|15611463|ref|NP_223114.1|	Helicobacter pylori J99	E-value = 1.00E-09	Identity = 30.18%
AB0841	1	*pgi*	gi|78777669|ref|YP_393984.1|	Sulfuromonas denitrificans ATCC 33889	E-value = 5.00E-127	Identity = 60.20%
	2		gi|152993518|ref|YP_001359239.1|	Sulfurovum sp. NBC37-1	E-value = 2.00E-102	Identity = 51.59%
	3		gi|152992865|ref|YP_001358586.1|	Sulfurovum sp. NBC37-1	E-value = 3.00E-89	Identity = 46.31%
	4		gi|148244946|ref|YP_001219640.1|	Candidatus Vesicomyosocius okutanii HA	E-value = 5.00E-89	Identity = 48.89%
	5		gi|152990690|ref|YP_001356412.1|	Nitratiruptor sp. SB155-2	E-value = 2.00E-86	Identity = 47.72%
AB0842	1	*galU*	gi|34556775|ref|NP_906590.1|	Wolinella succinogenes DSM 1740	E-value = 2.00E-117	Identity = 76.30%
	2		gi|78777670|ref|YP_393985.1|	Sulfuromonas denitrificans ATCC 33889	E-value = 2.00E-116	Identity = 75.37%
	3		gi|152993519|ref|YP_001359240.1|	Sulfurovum sp. NBC37-1	E-value = 3.00E-116	Identity = 75.93%
	4		gi|146307378|ref|YP_001187843.1|	Pseudomonas mendocina ymp	E-value = 6.00E-115	Identity = 72.10%
	5		gi|119478090|ref|ZP_01618169.1|	marine gamma proteobacterium HTCC2143	E-value = 3.00E-113	Identity = 72.01%
AB0843	1		gi|152991170|ref|YP_001356892.1|	Nitratiruptor sp. SB155-2	E-value = 4.00E-165	Identity = 64.77%
	2		gi|78776822|ref|YP_393137.1|	Sulfuromonas denitrificans ATCC 33889	E-value = 7.00E-151	Identity = 59.69%
	3		gi|152993517|ref|YP_001359238.1|	Sulfurovum sp. NBC37-1	E-value = 1.00E-148	Identity = 59.70%
	4		gi|149195311|ref|ZP_01872399.1|	Caminibacter mediatlanticus TB-2	E-value = 2.00E-145	Identity = 59.74%
	5		gi|32266112|ref|NP_860144.1|	Helicobacter hepaticus ATCC 51449	E-value = 9.00E-140	Identity = 54.87%
AB0844	1		gi|153869834|ref|ZP_01999355.1|	Beggiatoa sp. PS	E-value = 2.00E-39	Identity = 32.75%
AB0845	1		gi|78777849|ref|YP_394164.1|	Sulfuromonas denitrificans ATCC 33889	E-value = 5.00E-39	Identity = 64.80%
	2		gi|121605706|ref|YP_983035.1|	Polaromonas naphthalenivorans CJ2	E-value = 9.00E-30	Identity = 49.23%
	3		gi|152993485|ref|YP_001359206.1|	Sulfurovum sp. NBC37-1	E-value = 1.00E-28	Identity = 54.17%
	4		gi|89094324|ref|ZP_01167265.1|	Oceanospirillum sp. MED92	E-value = 5.00E-27	Identity = 50.00%
	5		gi|57505415|ref|ZP_00371343.1|	Campylobacter upsaliensis RM3195	E-value = 3.00E-26	Identity = 45.86%
AB0846	1		gi|154174478|ref|YP_001408754.1|	Campylobacter curvus 525.92	E-value = 2.00E-10	Identity = 51.43%
	2		gi|57168158|ref|ZP_00367297.1|	Campylobacter coli RM2228	E-value = 1.00E-09	Identity = 54.41%
	3		gi|86152979|ref|ZP_01071184.1|	Campylobacter jejuni subsp. jejuni HB93-13	E-value = 3.00E-09	Identity = 51.47%
	4		gi|57505648|ref|ZP_00371575.1|	Campylobacter upsaliensis RM3195	E-value = 5.00E-09	Identity = 56.72%
	5		gi|56962362|ref|YP_174087.1|	Bacillus clausii KSM-K16	E-value = 7.00E-09	Identity = 52.86%
AB0847	1		gi|95929033|ref|ZP_01311778.1|	Desulfuromonas acetoxidans DSM 684	E-value = 4.00E-39	Identity = 35.69%
	2		gi|51244145|ref|YP_064029.1|	Desulfotalea psychrophila LSv54	E-value = 2.00E-38	Identity = 35.21%
	3		gi|89206591|ref|ZP_01185147.1|	Bacillus weihenstephanensis KBAB4	E-value = 7.00E-35	Identity = 38.60%
	4		gi|1710375|gb|AAB47963.1|	Bacillus subtilis	E-value = 3.00E-34	Identity = 33.45%
	5		gi|16079720|ref|NP_390544.1|	Bacillus subtilis subsp. subtilis str. 168	E-value = 6.00E-34	Identity = 33.10%
AB0848	1	*trpC*	gi|34558206|ref|NP_908021.1|	Wolinella succinogenes DSM 1740	E-value = 9.00E-81	Identity = 62.75%
	2		gi|154174456|ref|YP_001408349.1|	Campylobacter curvus 525.92	E-value = 5.00E-79	Identity = 61.39%

	3		gi|152993281|ref|YP_001359002.1|	Sulfurovum sp. NBC37-1	E-value = 8.00E-79	Identity = 62.40%
	4		gi|152990734|ref|YP_001356456.1|	Nitratiruptor sp. SB155-2	E-value = 1.00E-78	Identity = 62.55%
	5		gi|121612469|ref|YP_001000194.1|	Campylobacter jejuni subsp. jejuni 81-176	E-value = 2.00E-73	Identity = 64.37%
AB0849	1		gi|152990735|ref|YP_001356457.1|	Nitratiruptor sp. SB155-2	E-value = 2.00E-36	Identity = 28.70%
	2		gi|78776929|ref|YP_393244.1|	Sulfuromonas denitrificans ATCC 33889	E-value = 5.00E-36	Identity = 30.63%
	3		gi|152993282|ref|YP_001359003.1|	Sulfurovum sp. NBC37-1	E-value = 4.00E-32	Identity = 28.97%
	4		gi|34558207|ref|NP_908022.1|	Wolinella succinogenes DSM 1740	E-value = 4.00E-31	Identity = 27.46%
	5		gi|154148793|ref|YP_001406872.1|	Campylobacter hominis ATCC BAA-381	E-value = 7.00E-29	Identity = 29.68%
AB0850	1		gi|152993283|ref|YP_001359004.1|	Sulfurovum sp. NBC37-1	E-value = 5.00E-33	Identity = 53.72%
	2		gi|78776928|ref|YP_393243.1|	Sulfuromonas denitrificans ATCC 33889	E-value = 6.00E-29	Identity = 48.39%
	3		gi|152990736|ref|YP_001356458.1|	Nitratiruptor sp. SB155-2	E-value = 1.00E-27	Identity = 49.21%
	4		gi|149194483|ref|ZP_01871579.1|	Caminibacter mediatlanticus TB-2	E-value = 2.00E-26	Identity = 52.46%
	5		gi|157165139|ref|YP_001466730.1|	Campylobacter concisus 13826	E-value = 6.00E-26	Identity = 46.28%
AB0851	1		gi|152990737|ref|YP_001356459.1|	Nitratiruptor sp. SB155-2	E-value = 1.00E-56	Identity = 48.10%
	2		gi|78776927|ref|YP_393242.1|	Sulfuromonas denitrificans ATCC 33889	E-value = 4.00E-49	Identity = 48.29%
	3		gi|34558209|ref|NP_908024.1|	Wolinella succinogenes DSM 1740	E-value = 2.00E-42	Identity = 39.83%
	4		gi|152993284|ref|YP_001359005.1|	Sulfurovum sp. NBC37-1	E-value = 7.00E-42	Identity = 39.66%
	5		gi|154175164|ref|YP_001408352.1|	Campylobacter curvus 525.92	E-value = 3.00E-40	Identity = 41.38%
AB0852	1	*oorD*	gi|34557450|ref|NP_907265.1|	Wolinella succinogenes DSM 1740	E-value = 1.00E-32	Identity = 65.42%
	2		gi|78777250|ref|YP_393565.1|	Sulfuromonas denitrificans ATCC 33889	E-value = 4.00E-31	Identity = 63.73%
	3		gi|152992174|ref|YP_001357895.1|	Sulfurovum sp. NBC37-1	E-value = 5.00E-31	Identity = 62.62%
	4		gi|57241287|ref|ZP_00369234.1|	Campylobacter lari RM2100	E-value = 2.00E-30	Identity = 69.70%
	5		gi|57167580|ref|ZP_00366720.1|	Campylobacter coli RM2228	E-value = 5.00E-30	Identity = 67.33%
AB0853	1	*oorA*	gi|152992175|ref|YP_001357896.1|	Sulfurovum sp. NBC37-1	E-value = 2.00E-147	Identity = 68.82%
	2		gi|118474959|ref|YP_892078.1|	Campylobacter fetus subsp. fetus 82-40	E-value = 1.00E-144	Identity = 66.13%
	3		gi|149194475|ref|ZP_01871571.1|	Caminibacter mediatlanticus TB-2	E-value = 1.00E-143	Identity = 65.44%
	4		gi|86153533|ref|ZP_01071737.1|	Campylobacter jejuni subsp. jejuni HB93-13	E-value = 3.00E-140	Identity = 63.98%
	5		gi|57241286|ref|ZP_00369233.1|	Campylobacter lari RM2100	E-value = 5.00E-140	Identity = 64.52%
AB0854	1	*oorB*	gi|152990590|ref|YP_001356312.1|	Nitratiruptor sp. SB155-2	E-value = 1.00E-127	Identity = 73.29%
	2		gi|152992176|ref|YP_001357897.1|	Sulfurovum sp. NBC37-1	E-value = 2.00E-122	Identity = 72.96%
	3		gi|34557452|ref|NP_907267.1|	Wolinella succinogenes DSM 1740	E-value = 2.00E-122	Identity = 72.16%
	4		gi|57241285|ref|ZP_00369232.1|	Campylobacter lari RM2100	E-value = 2.00E-121	Identity = 71.84%
	5		gi|32267067|ref|NP_861099.1|	Helicobacter hepaticus ATCC 51449	E-value = 1.00E-120	Identity = 72.45%
AB0855	1	*oorC*	gi|152992177|ref|YP_001357898.1|	Sulfurovum sp. NBC37-1	E-value = 1.00E-67	Identity = 74.18%
	2		gi|118475141|ref|YP_892076.1|	Campylobacter fetus subsp. fetus 82-40	E-value = 5.00E-62	Identity = 70.06%
	3		gi|78777253|ref|YP_393568.1|	Sulfuromonas denitrificans ATCC 33889	E-value = 8.00E-62	Identity = 75.14%
	4		gi|32267066|ref|NP_861098.1|	Helicobacter hepaticus ATCC 51449	E-value = 1.00E-60	Identity = 68.54%
	5		gi|152990591|ref|YP_001356313.1|	Nitratiruptor sp. SB155-2	E-value = 4.00E-60	Identity = 65.03%
AB0856	1	*dnaQ3*	gi|152990071|ref|YP_001355793.1|	Nitratiruptor sp. SB155-2	E-value = 2.00E-46	Identity = 45.61%
	2		gi|154148582|ref|YP_001406342.1|	Campylobacter hominis ATCC BAA-381	E-value = 2.00E-44	Identity = 44.98%
	3		gi|157165763|ref|YP_001466765.1|	Campylobacter concisus 13826	E-value = 3.00E-43	Identity = 45.86%
	4		gi|152993730|ref|YP_001359451.1|	Sulfurovum sp. NBC37-1	E-value = 8.00E-42	Identity = 43.58%
	5		gi|78777804|ref|YP_394119.1|	Sulfuromonas denitrificans ATCC 33889	E-value = 4.00E-41	Identity = 45.81%
AB0857	1	*trpF*	gi|152990070|ref|YP_001355792.1|	Nitratiruptor sp. SB155-2	E-value = 4.00E-53	Identity = 55.44%
	2		gi|152993731|ref|YP_001359452.1|	Sulfurovum sp. NBC37-1	E-value = 8.00E-53	Identity = 50.51%
	3		gi|78777803|ref|YP_394118.1|	Sulfuromonas denitrificans ATCC 33889	E-value = 8.00E-51	Identity = 51.30%
	4		gi|34556663|ref|NP_906478.1|	Wolinella succinogenes DSM 1740	E-value = 2.00E-43	Identity = 45.69%
	5		gi|149195071|ref|ZP_01872163.1|	Caminibacter mediatlanticus TB-2	E-value = 5.00E-43	Identity = 52.33%
AB0858	1	*rpe*	gi|78777802|ref|YP_394117.1|	Sulfuromonas denitrificans ATCC 33889	E-value = 1.00E-85	Identity = 76.06%

	2	gi|34556662|ref|NP_906477.1|	Wolinella succinogenes DSM 1740	E-value = 2.00E-84	Identity = 69.48%
	3	gi|152993732|ref|YP_001359453.1|	Sulfurovum sp. NBC37-1	E-value = 3.00E-78	Identity = 77.46%
	4	gi|154149308|ref|YP_001406343.1|	Campylobacter hominis ATCC BAA-381	E-value = 1.00E-77	Identity = 67.14%
	5	gi|149195070|ref|ZP_01872162.1|	Caminibacter mediatlanticus TB-2	E-value = 3.00E-77	Identity = 66.51%
AB0859	1 *pldA*	gi|154173970|ref|YP_001409012.1|	Campylobacter curvus 525.92	E-value = 4.00E-56	Identity = 40.53%
	2	gi|157164974|ref|YP_001467595.1|	Campylobacter concisus 13826	E-value = 1.00E-54	Identity = 40.06%
	3	gi|118474551|ref|YP_891402.1|	Campylobacter fetus subsp. fetus 82-40	E-value = 2.00E-52	Identity = 38.86%
	4	gi|154149331|ref|YP_001405739.1|	Campylobacter hominis ATCC BAA-381	E-value = 3.00E-51	Identity = 39.18%
	5	gi|88596271|ref|ZP_01099508.1|	Campylobacter jejuni subsp. jejuni 84-25	E-value = 3.00E-44	Identity = 37.83%
AB0860	1	gi|34557700|ref|NP_907515.1|	Wolinella succinogenes DSM 1740	E-value = 3.00E-15	Identity = 39.17%
	2	gi|152990461|ref|YP_001356183.1|	Nitratiruptor sp. SB155-2	E-value = 1.00E-14	Identity = 36.84%
	3	gi|152992261|ref|YP_001357982.1|	Sulfurovum sp. NBC37-1	E-value = 6.00E-14	Identity = 36.28%
	4	gi|154149148|ref|YP_001406594.1|	Campylobacter hominis ATCC BAA-381	E-value = 7.00E-14	Identity = 36.17%
	5	gi|149193974|ref|ZP_01871072.1|	Caminibacter mediatlanticus TB-2	E-value = 1.00E-12	Identity = 38.39%
AB0861	1	gi|152993703|ref|YP_001359424.1|	Sulfurovum sp. NBC37-1	E-value = 3.00E-53	Identity = 54.89%
	2	gi|119947278|ref|YP_944958.1|	Psychromonas ingrahamii 37	E-value = 4.00E-48	Identity = 50.00%
	3	gi|119947263|ref|YP_944943.1|	Psychromonas ingrahamii 37	E-value = 9.00E-48	Identity = 50.00%
	4	gi|78776570|ref|YP_392885.1|	Sulfuromonas denitrificans ATCC 33889	E-value = 2.00E-45	Identity = 46.70%
	5	gi|152990094|ref|YP_001355816.1|	Nitratiruptor sp. SB155-2	E-value = 3.00E-45	Identity = 49.72%
AB0862	1	gi|152991822|ref|YP_001357543.1|	Sulfurovum sp. NBC37-1	E-value = 3.00E-37	Identity = 64.02%
	2	gi|78776240|ref|YP_392555.1|	Sulfuromonas denitrificans ATCC 33889	E-value = 1.00E-33	Identity = 55.83%
	3	gi|34556535|ref|NP_906350.1|	Wolinella succinogenes DSM 1740	E-value = 1.00E-30	Identity = 53.29%
	4	gi|154174327|ref|YP_001407469.1|	Campylobacter curvus 525.92	E-value = 1.00E-28	Identity = 47.53%
	5	gi|157164961|ref|YP_001466097.1|	Campylobacter concisus 13826	E-value = 3.00E-28	Identity = 47.53%
AB0863	1	gi|24374390|ref|NP_718433.1|	Shewanella oneidensis MR-1	E-value = 1.00E-30	Identity = 32.96%
	2	gi|118073501|ref|ZP_01541681.1|	Shewanella woodyi ATCC 51908	E-value = 4.00E-30	Identity = 33.85%
	3	gi|113969853|ref|YP_733646.1|	Shewanella sp. MR-4	E-value = 2.00E-28	Identity = 30.83%
	4	gi|37525518|ref|NP_928862.1|	Photorhabdus luminescens subsp. laumondii TTO1	E-value = 8.00E-28	Identity = 33.33%
	5	gi|114563628|ref|YP_751141.1|	Shewanella frigidimarina NCIMB 400	E-value = 8.00E-28	Identity = 34.23%
AB0864	1	gi|82702160|ref|YP_411726.1|	Nitrosospira multiformis ATCC 25196	E-value = 2.00E-16	Identity = 48.72%
	2	gi|45659279|ref|YP_003365.1|	Leptospira interrogans serovar Copenhageni str. Fiocruz L1-130	E-value = 5.00E-15	Identity = 42.98%
	3	gi|24217019|ref|NP_714500.1|	Leptospira interrogans serovar Lai str. 56601	E-value = 9.00E-15	Identity = 42.98%
	4	gi|77165544|ref|YP_344069.1|	Nitrosococcus oceani ATCC 19707	E-value = 2.00E-14	Identity = 46.28%
	5	gi|115422966|emb|CAJ49496.1|	Bordetella avium 197N	E-value = 4.00E-14	Identity = 38.98%
AB0865	1	gi|34558451|ref|NP_908266.1|	Wolinella succinogenes DSM 1740	E-value = 1.00E-37	Identity = 52.08%
	2	gi|86150785|ref|ZP_01069001.1|	Campylobacter jejuni subsp. jejuni 260.94	E-value = 4.00E-37	Identity = 53.10%
	3	gi|152993092|ref|YP_001358813.1|	Sulfurovum sp. NBC37-1	E-value = 5.00E-37	Identity = 53.15%
	4	gi|15792693|ref|NP_282516.1|	Campylobacter jejuni subsp. jejuni NCTC 11168	E-value = 1.00E-36	Identity = 52.41%
	5	gi|157415608|ref|YP_001482864.1|	Campylobacter jejuni subsp. jejuni 81116	E-value = 3.00E-36	Identity = 52.41%
AB0866	1	gi|116187134|ref|ZP_01477023.1|	Vibrio sp. Ex25	E-value = 7.00E-32	Identity = 44.50%
	2	gi|91224209|ref|ZP_01259472.1|	Vibrio alginolyticus 12G01	E-value = 9.00E-32	Identity = 43.46%
	3	gi|153831614|ref|ZP_01984281.1|	Vibrio harveyi HY01	E-value = 6.00E-31	Identity = 41.88%
	4	gi|148975673|ref|ZP_01812504.1|	Vibrionales bacterium SWAT-3	E-value = 4.00E-30	Identity = 43.98%
	5	gi|84390407|ref|ZP_00991418.1|	Vibrio splendidus 12B01	E-value = 5.00E-30	Identity = 43.98%
AB0867	1		***** No matches found *****		
AB0868	1	gi|34558498|ref|NP_908313.1|	Wolinella succinogenes DSM 1740	E-value = 2.00E-70	Identity = 61.01%
	2	gi|152992349|ref|YP_001358070.1|	Sulfurovum sp. NBC37-1	E-value = 3.00E-70	Identity = 60.00%
	3	gi|78777433|ref|YP_393748.1|	Sulfuromonas denitrificans ATCC 33889	E-value = 4.00E-68	Identity = 60.18%
	4	gi|152990444|ref|YP_001356166.1|	Nitratiruptor sp. SB155-2	E-value = 9.00E-68	Identity = 58.22%

	5		gi|32267181|ref|NP_861213.1|	Helicobacter hepaticus ATCC 51449	E-value = 3.00E-65	Identity = 55.96%
AB0869	1		gi|152992350|ref|YP_001358071.1|	Sulfurovum sp. NBC37-1	E-value = 5.00E-99	Identity = 53.16%
	2		gi|78777432|ref|YP_393747.1|	Sulfuromonas denitrificans ATCC 33889	E-value = 1.00E-96	Identity = 53.74%
	3		gi|152990445|ref|YP_001356167.1|	Nitratiruptor sp. SB155-2	E-value = 7.00E-95	Identity = 53.30%
	4		gi|34558497|ref|NP_908312.1|	Wolinella succinogenes DSM 1740	E-value = 3.00E-90	Identity = 53.74%
	5		gi|149194614|ref|ZP_01871710.1|	Caminibacter mediatlanticus TB-2	E-value = 1.00E-83	Identity = 50.00%
AB0870	1	*pgsA*	gi|152992401|ref|YP_001358122.1|	Sulfurovum sp. NBC37-1	E-value = 5.00E-51	Identity = 62.86%
	2		gi|157164350|ref|YP_001466915.1|	Campylobacter concisus 13826	E-value = 6.00E-51	Identity = 65.34%
	3		gi|152990446|ref|YP_001356168.1|	Nitratiruptor sp. SB155-2	E-value = 9.00E-51	Identity = 58.86%
	4		gi|154174159|ref|YP_001408200.1|	Campylobacter curvus 525.92	E-value = 1.00E-50	Identity = 65.91%
	5		gi|154148964|ref|YP_001406173.1|	Campylobacter hominis ATCC BAA-381	E-value = 5.00E-49	Identity = 62.92%
AB0871	1		gi|57505465|ref|ZP_00371393.1|	Campylobacter upsaliensis RM3195	E-value = 7.00E-107	Identity = 73.36%
	2		gi|86152197|ref|ZP_01070409.1|	Campylobacter jejuni subsp. jejuni 260.94	E-value = 1.00E-106	Identity = 74.13%
	3		gi|57241095|ref|ZP_00369042.1|	Campylobacter lari RM2100	E-value = 5.00E-106	Identity = 74.13%
	4		gi|157163926|ref|YP_001466914.1|	Campylobacter concisus 13826	E-value = 1.00E-104	Identity = 73.54%
	5		gi|154173801|ref|YP_001408201.1|	Campylobacter curvus 525.92	E-value = 7.00E-104	Identity = 73.93%
AB0872	1	*dapA*	gi|152992403|ref|YP_001358124.1|	Sulfurovum sp. NBC37-1	E-value = 3.00E-110	Identity = 64.95%
	2		gi|152990448|ref|YP_001356170.1|	Nitratiruptor sp. SB155-2	E-value = 5.00E-110	Identity = 64.29%
	3		gi|157165445|ref|YP_001466913.1|	Campylobacter concisus 13826	E-value = 2.00E-107	Identity = 63.79%
	4		gi|57167805|ref|ZP_00366945.1|	Campylobacter coli RM2228	E-value = 1.00E-106	Identity = 61.64%
	5		gi|154174259|ref|YP_001408202.1|	Campylobacter curvus 525.92	E-value = 1.00E-106	Identity = 61.99%
AB0873	1		gi|34558493|ref|NP_908308.1|	Wolinella succinogenes DSM 1740	E-value = 8.00E-163	Identity = 66.91%
	2		gi|152990449|ref|YP_001356171.1|	Nitratiruptor sp. SB155-2	E-value = 2.00E-160	Identity = 68.37%
	3		gi|78777423|ref|YP_393738.1|	Sulfuromonas denitrificans ATCC 33889	E-value = 9.00E-156	Identity = 64.73%
	4		gi|157164188|ref|YP_001466912.1|	Campylobacter concisus 13826	E-value = 7.00E-149	Identity = 64.08%
	5		gi|154174701|ref|YP_001408203.1|	Campylobacter curvus 525.92	E-value = 4.00E-148	Identity = 63.83%
AB0874	1	*pyrD*	gi|152992405|ref|YP_001358126.1|	Sulfurovum sp. NBC37-1	E-value = 3.00E-118	Identity = 59.71%
	2		gi|152990450|ref|YP_001356172.1|	Nitratiruptor sp. SB155-2	E-value = 8.00E-114	Identity = 56.41%
	3		gi|78777422|ref|YP_393737.1|	Sulfuromonas denitrificans ATCC 33889	E-value = 2.00E-110	Identity = 57.67%
	4		gi|57241098|ref|ZP_00369045.1|	Campylobacter lari RM2100	E-value = 6.00E-109	Identity = 56.53%
	5		gi|118474237|ref|YP_891961.1|	Campylobacter fetus subsp. fetus 82-40	E-value = 3.00E-108	Identity = 57.39%
AB0875	1	*msbA*	gi|34556696|ref|NP_906511.1|	Wolinella succinogenes DSM 1740	E-value = 5.00E-171	Identity = 56.48%
	2		gi|154174697|ref|YP_001408205.1|	Campylobacter curvus 525.92	E-value = 2.00E-170	Identity = 54.67%
	3		gi|118475187|ref|YP_891962.1|	Campylobacter fetus subsp. fetus 82-40	E-value = 3.00E-166	Identity = 53.82%
	4		gi|149194654|ref|ZP_01871749.1|	Caminibacter mediatlanticus TB-2	E-value = 2.00E-158	Identity = 57.02%
	5		gi|157165032|ref|YP_001466910.1|	Campylobacter concisus 13826	E-value = 3.00E-158	Identity = 54.32%
AB0876	1	*mviN*	gi|78777409|ref|YP_393724.1|	Sulfuromonas denitrificans ATCC 33889	E-value = 2.00E-108	Identity = 57.05%
	2		gi|34556869|ref|NP_906684.1|	Wolinella succinogenes DSM 1740	E-value = 4.00E-108	Identity = 52.64%
	3		gi|152990844|ref|YP_001356566.1|	Nitratiruptor sp. SB155-2	E-value = 4.00E-103	Identity = 56.16%
	4		gi|154173819|ref|YP_001408207.1|	Campylobacter curvus 525.92	E-value = 1.00E-99	Identity = 54.92%
	5		gi|154148088|ref|YP_001406166.1|	Campylobacter hominis ATCC BAA-381	E-value = 8.00E-99	Identity = 53.00%
AB0877	1		gi|152993433|ref|YP_001359154.1|	Sulfurovum sp. NBC37-1	E-value = 2.00E-77	Identity = 36.26%
	2		gi|154149223|ref|YP_001406798.1|	Campylobacter hominis ATCC BAA-381	E-value = 2.00E-34	Identity = 29.88%
	3		gi|157164312|ref|YP_001467079.1|	Campylobacter concisus 13826	E-value = 1.00E-32	Identity = 26.92%
	4		gi|118475423|ref|YP_891999.1|	Campylobacter fetus subsp. fetus 82-40	E-value = 3.00E-27	Identity = 29.43%
	5		gi|154174202|ref|YP_001408089.1|	Campylobacter curvus 525.92	E-value = 2.00E-26	Identity = 28.06%
AB0878	1	*perR*	gi|34556965|ref|NP_906780.1|	Wolinella succinogenes DSM 1740	E-value = 3.00E-25	Identity = 42.31%
	2		gi|32266441|ref|NP_860473.1|	Helicobacter hepaticus ATCC 51449	E-value = 3.00E-24	Identity = 40.15%
	3		gi|152992347|ref|YP_001358068.1|	Sulfurovum sp. NBC37-1	E-value = 3.00E-22	Identity = 44.03%

	4		gi|152990894|ref|YP_001356616.1|	Nitratiruptor sp. SB155-2	E-value = 3.00E-20	Identity = 40.31%
	5		gi|149194236|ref|ZP_01871333.1|	Caminibacter mediatlanticus TB-2	E-value = 6.00E-19	Identity = 44.03%
AB0879	1		gi|149194504|ref|ZP_01871600.1|	Caminibacter mediatlanticus TB-2	E-value = 4.00E-32	Identity = 60.43%
	2		gi|152992855|ref|YP_001358576.1|	Sulfurovum sp. NBC37-1	E-value = 4.00E-32	Identity = 57.64%
	3		gi|78777269|ref|YP_393584.1|	Sulfuromonas denitrificans ATCC 33889	E-value = 5.00E-31	Identity = 57.45%
	4		gi|34556974|ref|NP_906789.1|	Wolinella succinogenes DSM 1740	E-value = 6.00E-28	Identity = 52.90%
	5		gi|57505296|ref|ZP_00371225.1|	Campylobacter upsaliensis RM3195	E-value = 1.00E-27	Identity = 58.70%
AB0880	1		gi|118027356|ref|ZP_01498820.1|	Burkholderia phymatum STM815	E-value = 2.00E-20	Identity = 41.98%
	2		gi|83592073|ref|YP_425825.1|	Rhodospirillum rubrum ATCC 11170	E-value = 2.00E-12	Identity = 33.61%
	3		gi|51894172|ref|YP_076863.1|	Symbiobacterium thermophilum IAM 14863	E-value = 6.00E-12	Identity = 36.64%
	4		gi|145620473|ref|ZP_01776503.1|	Geobacter bemidjiensis Bem	E-value = 7.00E-12	Identity = 36.29%
	5		gi|147678002|ref|YP_001212217.1|	Pelotomaculum thermopropionicum SI	E-value = 7.00E-11	Identity = 35.71%
AB0881	1		gi|34557918|ref|NP_907733.1|	Wolinella succinogenes DSM 1740	E-value = 6.00E-64	Identity = 63.05%
	2		gi|16799459|ref|NP_469727.1|	Listeria innocua Clip11262	E-value = 3.00E-63	Identity = 62.81%
	3		gi|46906601|ref|YP_012990.1|	Listeria monocytogenes str. 4b F2365	E-value = 9.00E-62	Identity = 62.31%
	4		gi|16802408|ref|NP_463893.1|	Listeria monocytogenes EGD-e	E-value = 2.00E-61	Identity = 62.37%
	5		gi|47091501|ref|ZP_00229298.1|	Listeria monocytogenes str. 4b H7858	E-value = 2.00E-61	Identity = 62.31%
AB0882	1	*radA*	gi|78777040|ref|YP_393355.1|	Sulfuromonas denitrificans ATCC 33889	E-value = 1.00E-167	Identity = 67.26%
	2		gi|86151058|ref|ZP_01069274.1|	Campylobacter jejuni subsp. jejuni 260.94	E-value = 3.00E-162	Identity = 64.65%
	3		gi|148926208|ref|ZP_01809893.1|	Campylobacter jejuni subsp. jejuni CG8486	E-value = 4.00E-162	Identity = 64.65%
	4		gi|153951878|ref|YP_001397703.1|	Campylobacter jejuni subsp. doylei 269.97	E-value = 5.00E-162	Identity = 64.21%
	5		gi|15792529|ref|NP_282352.1|	Campylobacter jejuni subsp. jejuni NCTC 11168	E-value = 5.00E-162	Identity = 64.43%
AB0883	1		gi|152990982|ref|YP_001356704.1|	Nitratiruptor sp. SB155-2	E-value = 1.00E-31	Identity = 45.11%
	2		gi|118400429|ref|XP_001032537.1|	Tetrahymena thermophila SB210	E-value = 2.00E-31	Identity = 37.84%
	3		gi|151936884|gb|EDN55780.1|	Vibrio sp. Ex25	E-value = 1.00E-29	Identity = 39.58%
	4		gi|153835671|ref|ZP_01988338.1|	Vibrio harveyi HY01	E-value = 3.00E-29	Identity = 39.06%
	5		gi|91228379|ref|ZP_01262306.1|	Vibrio alginolyticus 12G01	E-value = 1.00E-28	Identity = 39.06%
AB0885	1		gi|152993213|ref|YP_001358934.1|	Sulfurovum sp. NBC37-1	E-value = 1.00E-85	Identity = 52.03%
	2		gi|86153977|ref|ZP_01072179.1|	Campylobacter jejuni subsp. jejuni HB93-13	E-value = 8.00E-76	Identity = 49.01%
	3		gi|57236932|ref|YP_178047.1|	Campylobacter jejuni subsp. jejuni RM1221	E-value = 9.00E-76	Identity = 49.01%
	4		gi|86150541|ref|ZP_01068765.1|	Campylobacter jejuni subsp. jejuni CF93-6	E-value = 1.00E-75	Identity = 48.68%
	5		gi|88597641|ref|ZP_01100874.1|	Campylobacter jejuni subsp. jejuni 84-25	E-value = 2.00E-75	Identity = 48.36%
AB0886	1		gi|87119415|ref|ZP_01075312.1|	Marinomonas sp. MED121	E-value = 2.00E-91	Identity = 32.04%
AB0887	1			***** No matches found *****		
AB0888	1	*ftsY*	gi|152990801|ref|YP_001356523.1|	Nitratiruptor sp. SB155-2	E-value = 3.00E-90	Identity = 63.16%
	2		gi|154173993|ref|YP_001408112.1|	Campylobacter curvus 525.92	E-value = 5.00E-89	Identity = 60.28%
	3		gi|157164288|ref|YP_001466926.1|	Campylobacter concisus 13826	E-value = 2.00E-88	Identity = 59.09%
	4		gi|152992192|ref|YP_001357913.1|	Sulfurovum sp. NBC37-1	E-value = 8.00E-88	Identity = 58.39%
	5		gi|149194174|ref|ZP_01871272.1|	Caminibacter mediatlanticus TB-2	E-value = 2.00E-85	Identity = 58.95%
AB0889	1		gi|34556527|ref|NP_906342.1|	Wolinella succinogenes DSM 1740	E-value = 5.00E-40	Identity = 49.40%
	2		gi|15792531|ref|NP_282354.1|	Campylobacter jejuni subsp. jejuni NCTC 11168	E-value = 2.00E-30	Identity = 42.25%
	3		gi|86150395|ref|ZP_01068621.1|	Campylobacter jejuni subsp. jejuni CF93-6	E-value = 3.00E-30	Identity = 42.78%
	4		gi|57238079|ref|YP_179328.1|	Campylobacter jejuni subsp. jejuni RM1221	E-value = 4.00E-30	Identity = 42.25%
	5		gi|157415470|ref|YP_001482726.1|	Campylobacter jejuni subsp. jejuni 81116	E-value = 4.00E-30	Identity = 42.25%
AB0890	1		gi|152992194|ref|YP_001357915.1|	Sulfurovum sp. NBC37-1	E-value = 2.00E-36	Identity = 46.89%
	2		gi|149194172|ref|ZP_01871270.1|	Caminibacter mediatlanticus TB-2	E-value = 4.00E-32	Identity = 51.14%
	3		gi|78777043|ref|YP_393358.1|	Sulfuromonas denitrificans ATCC 33889	E-value = 3.00E-30	Identity = 47.93%
	4		gi|152990799|ref|YP_001356521.1|	Nitratiruptor sp. SB155-2	E-value = 2.00E-28	Identity = 39.64%
	5		gi|57242521|ref|ZP_00370459.1|	Campylobacter upsaliensis RM3195	E-value = 1.00E-23	Identity = 41.88%

AB0891	1	gi|152990798|ref|YP_001356520.1|	Nitratiruptor sp. SB155-2	E-value = 9.00E-148	Identity = 60.89%
	2	gi|15792533|ref|NP_282356.1|	Campylobacter jejuni subsp. jejuni NCTC 11168	E-value = 9.00E-145	Identity = 58.61%
	3	gi|86150310|ref|ZP_01068536.1|	Campylobacter jejuni subsp. jejuni CF93-6	E-value = 1.00E-144	Identity = 58.73%
	4	gi|57238081|ref|YP_179330.1|	Campylobacter jejuni subsp. jejuni RM1221	E-value = 3.00E-144	Identity = 58.54%
	5	gi|57242520|ref|ZP_00370458.1|	Campylobacter upsaliensis RM3195	E-value = 3.00E-144	Identity = 58.03%
AB0892	1	gi|152992772|ref|YP_001358493.1|	Sulfurovum sp. NBC37-1	E-value = 7.00E-42	Identity = 44.39%
	2	gi|152990742|ref|YP_001356464.1|	Nitratiruptor sp. SB155-2	E-value = 3.00E-39	Identity = 48.13%
	3	gi|57240934|ref|ZP_00368882.1|	Campylobacter lari RM2100	E-value = 2.00E-38	Identity = 47.40%
	4	gi|121612964|ref|YP_001001110.1|	Campylobacter jejuni subsp. jejuni 81-176	E-value = 2.00E-37	Identity = 47.85%
	5	gi|148925633|ref|ZP_01809321.1|	Campylobacter jejuni subsp. jejuni CG8486	E-value = 2.00E-37	Identity = 47.06%
AB0893	1	gi|152990741|ref|YP_001356463.1|	Nitratiruptor sp. SB155-2	E-value = 3.00E-81	Identity = 65.77%
	2	gi|78776766|ref|YP_393081.1|	Sulfuromonas denitrificans ATCC 33889	E-value = 4.00E-77	Identity = 63.12%
	3	gi|118580429|ref|YP_901679.1|	Pelobacter propionicus DSM 2379	E-value = 1.00E-48	Identity = 42.15%
	4	gi|118744146|ref|ZP_01592143.1|	Geobacter lovleyi SZ	E-value = 6.00E-46	Identity = 43.05%
	5	gi|145619727|ref|ZP_01775773.1|	Geobacter bemidjiensis Bem	E-value = 1.00E-45	Identity = 41.06%
AB0894	1		***** No matches found *****		
AB0895	1		***** No matches found *****		
AB0896	1		***** No matches found *****		
AB0897	1	gi|126654730|ref|ZP_01726264.1|	Cyanothece sp. CCY0110	E-value = 2.00E-07	Identity = 33.78%
	2	gi|16329523|ref|NP_440251.1|	Synechocystis sp. PCC 6803	E-value = 4.00E-07	Identity = 33.78%
	3	gi|17229316|ref|NP_485864.1|	Nostoc sp. PCC 7120	E-value = 2.00E-06	Identity = 35.14%
AB0898	1	gi|152992763|ref|YP_001358484.1|	Sulfurovum sp. NBC37-1	E-value = 4.00E-76	Identity = 53.11%
	2	gi|151572635|gb|EDN38289.1|	Francisella tularensis subsp. novicida GA99-3548	E-value = 4.00E-75	Identity = 56.41%
	3	gi|62261996|gb|AAX78036.1|	synthetic construct	E-value = 5.00E-75	Identity = 56.04%
	4	gi|89256603|ref|YP_513965.1|	Francisella tularensis subsp. holarctica	E-value = 4.00E-74	Identity = 56.04%
	5	gi|118498104|ref|YP_899154.1|	Francisella tularensis subsp. novicida U112	E-value = 4.00E-74	Identity = 56.04%
AB0899	1	gi|152993169|ref|YP_001358890.1|	Sulfurovum sp. NBC37-1	E-value = 6.00E-72	Identity = 55.73%
	2	gi|78777345|ref|YP_393660.1|	Sulfuromonas denitrificans ATCC 33889	E-value = 9.00E-70	Identity = 53.75%
	3	gi|157165731|ref|YP_001466717.1|	Campylobacter concisus 13826	E-value = 1.00E-60	Identity = 50.20%
	4	gi|152990599|ref|YP_001356321.1|	Nitratiruptor sp. SB155-2	E-value = 9.00E-59	Identity = 49.01%
	5	gi|57240928|ref|ZP_00368876.1|	Campylobacter lari RM2100	E-value = 5.00E-58	Identity = 54.58%
AB0900	1		***** No matches found *****		
AB0901	1	gi|34556468|ref|NP_906283.1|	Wolinella succinogenes DSM 1740	E-value = 5.00E-83	Identity = 46.77%
	2	gi|121605719|ref|YP_983048.1|	Polaromonas naphthalenivorans CJ2	E-value = 9.00E-77	Identity = 51.79%
	3	gi|86152084|ref|ZP_01070296.1|	Campylobacter jejuni subsp. jejuni 260.94	E-value = 2.00E-73	Identity = 44.87%
	4	gi|157415135|ref|YP_001482391.1|	Campylobacter jejuni subsp. jejuni 81116	E-value = 2.00E-73	Identity = 44.87%
	5	gi|119898960|ref|YP_934173.1|	Azoarcus sp. BH72	E-value = 4.00E-73	Identity = 47.72%
AB0903	1	gi|34556469|ref|NP_906284.1|	Wolinella succinogenes DSM 1740	E-value = 2.00E-27	Identity = 32.44%
	2	gi|154173672|ref|YP_001408694.1|	Campylobacter curvus 525.92	E-value = 1.00E-12	Identity = 27.12%
AB0904	1	gi|34556470|ref|NP_906285.1|	Wolinella succinogenes DSM 1740	E-value = 6.00E-51	Identity = 50.45%
	2	gi|154174786|ref|YP_001408696.1|	Campylobacter curvus 525.92	E-value = 6.00E-44	Identity = 43.64%
	3	gi|157164104|ref|YP_001467231.1|	Campylobacter concisus 13826	E-value = 1.00E-42	Identity = 44.09%
	4	gi|154149275|ref|YP_001407283.1|	Campylobacter hominis ATCC BAA-381	E-value = 2.00E-42	Identity = 43.38%
	5	gi|78776459|ref|YP_392774.1|	Sulfuromonas denitrificans ATCC 33889	E-value = 2.00E-39	Identity = 43.58%
AB0905	1	gi|34556471|ref|NP_906286.1|	Wolinella succinogenes DSM 1740	E-value = 2.00E-64	Identity = 39.79%
	2	gi|152991572|ref|YP_001357294.1|	Nitratiruptor sp. SB155-2	E-value = 2.00E-53	Identity = 35.81%
	3	gi|78776460|ref|YP_392775.1|	Sulfuromonas denitrificans ATCC 33889	E-value = 5.00E-52	Identity = 35.42%
	4	gi|152992092|ref|YP_001357813.1|	Sulfurovum sp. NBC37-1	E-value = 8.00E-51	Identity = 32.45%
	5	gi|154174203|ref|YP_001408695.1|	Campylobacter curvus 525.92	E-value = 1.00E-41	Identity = 36.33%

AB0906	1	gi|157164151|ref|YP_001466949.1|	Campylobacter concisus 13826	E-value = 4.00E-63	Identity = 45.71%
	2	gi|118474208|ref|YP_891522.1|	Campylobacter fetus subsp. fetus 82-40	E-value = 5.00E-59	Identity = 43.77%
	3	gi|154175132|ref|YP_001408092.1|	Campylobacter curvus 525.92	E-value = 8.00E-59	Identity = 43.53%
	4	gi|156719903|ref|ZP_02061505.1|	Hydrogenobaculum sp. Y04AAS1	E-value = 1.00E-48	Identity = 42.09%
	5	gi|118745821|ref|ZP_01593785.1|	Geobacter lovleyi SZ	E-value = 1.00E-47	Identity = 37.99%
AB0907	1	gi|114799344|ref|YP_759497.1|	Hyphomonas neptunium ATCC 15444	E-value = 5.00E-22	Identity = 29.83%
	2	gi|87307282|ref|ZP_01089427.1|	Blastopirellula marina DSM 3645	E-value = 6.00E-21	Identity = 31.07%
	3	gi|87200494|ref|YP_497751.1|	Novosphingobium aromaticivorans DSM 12444	E-value = 1.00E-20	Identity = 28.15%
AB0908	1	gi|152990733|ref|YP_001356455.1|	Nitratiruptor sp. SB155-2	E-value = 9.00E-30	Identity = 57.82%
	2	gi|78776931|ref|YP_393246.1|	Sulfuromonas denitrificans ATCC 33889	E-value = 3.00E-26	Identity = 48.30%
	3	gi|152993280|ref|YP_001359001.1|	Sulfurovum sp. NBC37-1	E-value = 8.00E-26	Identity = 51.02%
	4	gi|34558387|ref|NP_908202.1|	Wolinella succinogenes DSM 1740	E-value = 1.00E-25	Identity = 52.08%
	5	gi|74318404|ref|YP_316144.1|	Thiobacillus denitrificans ATCC 25259	E-value = 1.00E-23	Identity = 44.90%
AB0909	1	gi|152992199|ref|YP_001357920.1|	Sulfurovum sp. NBC37-1	E-value = 0	Identity = 59.48%
	2	gi|78777466|ref|YP_393781.1|	Sulfuromonas denitrificans ATCC 33889	E-value = 0	Identity = 58.4%
	3	gi|152990783|ref|YP_001356505.1|	Nitratiruptor sp. SB155-2	E-value = 0	Identity = 60.22%
	4	gi|149194563|ref|ZP_01871659.1|	Caminibacter mediatlanticus TB-2	E-value = 0	Identity = 59.87%
	5	gi|34556688|ref|NP_906503.1|	Wolinella succinogenes DSM 1740	E-value = 0	Identity = 58.69%
AB0910	1	gi|157165200|ref|YP_001466550.1|	Campylobacter concisus 13826	E-value = 8.00E-26	Identity = 40.59%
	2	gi|78777700|ref|YP_394015.1|	Sulfuromonas denitrificans ATCC 33889	E-value = 3.00E-21	Identity = 40.22%
	3	gi|34557920|ref|NP_907735.1|	Wolinella succinogenes DSM 1740	E-value = 3.00E-20	Identity = 40.68%
	4	gi|86151344|ref|ZP_01069559.1|	Campylobacter jejuni subsp. jejuni 260.94	E-value = 3.00E-19	Identity = 34.80%
	5	gi|86153694|ref|ZP_01071897.1|	Campylobacter jejuni subsp. jejuni HB93-13	E-value = 1.00E-18	Identity = 34.31%
AB0911	1	gi|15605907|ref|NP_213284.1|	Aquifex aeolicus VF5	E-value = 2.00E-42	Identity = 46.73%
	2	gi|156719004|ref|ZP_02060656.1|	Hydrogenobaculum sp. Y04AAS1	E-value = 4.00E-36	Identity = 43.14%
	3	gi|34558330|ref|NP_908145.1|	Wolinella succinogenes DSM 1740	E-value = 5.00E-30	Identity = 37.63%
	4	gi|126662519|ref|ZP_01733518.1|	Flavobacteria bacterium BAL38	E-value = 2.00E-17	Identity = 32.99%
	5	gi|153951006|ref|YP_001398999.1|	Campylobacter jejuni subsp. doylei 269.97	E-value = 5.00E-17	Identity = 32.99%
AB0912	1	gi|91792468|ref|YP_562119.1|	Shewanella denitrificans OS217	E-value = 1.00E-37	Identity = 37.39%
	2	gi|88797395|ref|ZP_01112985.1|	Reinekea sp. MED297	E-value = 1.00E-33	Identity = 35.06%
	3	gi|87121429|ref|ZP_01077318.1|	Marinomonas sp. MED121	E-value = 5.00E-31	Identity = 35.96%
	4	gi|152996390|ref|YP_001341225.1|	Marinomonas sp. MWYL1	E-value = 6.00E-25	Identity = 34.56%
	5	gi|42522480|ref|NP_967860.1|	Bdellovibrio bacteriovorus HD100	E-value = 1.00E-21	Identity = 27.51%
AB0913	1	gi|34557182|ref|NP_906997.1|	Wolinella succinogenes DSM 1740	E-value = 1.00E-114	Identity = 49.19%
	2	gi|78777577|ref|YP_393892.1|	Sulfuromonas denitrificans ATCC 33889	E-value = 1.00E-97	Identity = 50.00%
	3	gi|78355110|ref|YP_386559.1|	Desulfovibrio desulfuricans G20	E-value = 7.00E-79	Identity = 36.41%
	4	gi|34556672|ref|NP_906487.1|	Wolinella succinogenes DSM 1740	E-value = 1.00E-77	Identity = 38.30%
	5	gi|154175227|ref|YP_001408994.1|	Campylobacter curvus 525.92	E-value = 1.00E-74	Identity = 40.27%
AB0914	1		***** No matches found *****		
AB0915	1		***** No matches found *****		
AB0916	1	gi|114332167|ref|YP_748389.1|	Nitrosomonas eutropha C91	E-value = 0	Identity = 62.81%
	2	gi|149374841|ref|ZP_01892614.1|	Marinobacter algicola DG893	E-value = 0	Identity = 60.85%
	3	gi|149911249|ref|ZP_01899872.1|	Moritella sp. PE36	E-value = 0	Identity = 59.36%
	4	gi|114563022|ref|YP_750535.1|	Shewanella frigidimarina NCIMB 400	E-value = 0	Identity = 59.62%
	5	gi|126668355|ref|ZP_01739313.1|	Marinobacter sp. ELB17	E-value = 0	Identity = 61.25%
AB0917	1	gi|91794373|ref|YP_564024.1|	Shewanella denitrificans OS217	E-value = 2.00E-38	Identity = 68.14%
	2	gi|95930121|ref|ZP_01312860.1|	Desulfuromonas acetoxidans DSM 684	E-value = 2.00E-34	Identity = 66.36%
	3	gi|95930113|ref|ZP_01312852.1|	Desulfuromonas acetoxidans DSM 684	E-value = 4.00E-33	Identity = 61.82%
	4	gi|113938812|ref|ZP_01424675.1|	Herpetosiphon aurantiacus ATCC 23779	E-value = 8.00E-33	Identity = 56.64%

	5		gi|15612940|ref|NP_241243.1|	Bacillus halodurans C-125	E-value = 1.00E-30	Identity = 56.88%
AB0918	1			***** No matches found *****		
AB0919	1			***** No matches found *****		
AB0920	1			***** No matches found *****		
AB0921	1	*guaA*	gi|152993480|ref|YP_001359201.1|	Sulfurovum sp. NBC37-1	E-value = 0	Identity = 77%
	2		gi|34557924|ref|NP_907739.1|	Wolinella succinogenes DSM 1740	E-value = 0	Identity = 74.07%
	3		gi|57238122|ref|YP_179372.1|	Campylobacter jejuni subsp. jejuni RM1221	E-value = 0	Identity = 71.68%
	4		gi|57168146|ref|ZP_00367285.1|	Campylobacter coli RM2228	E-value = 0	Identity = 71.29%
	5		gi|148926245|ref|ZP_01809930.1|	Campylobacter jejuni subsp. jejuni CG8486	E-value = 0	Identity = 71.48%
AB0922	1	*nadB*	gi|152991084|ref|YP_001356806.1|	Nitratiruptor sp. SB155-2	E-value = 3.00E-146	Identity = 53.96%
	2		gi|34557925|ref|NP_907740.1|	Wolinella succinogenes DSM 1740	E-value = 4.00E-140	Identity = 51.67%
	3		gi|152993482|ref|YP_001359203.1|	Sulfurovum sp. NBC37-1	E-value = 1.00E-137	Identity = 50.83%
	4		gi|78776621|ref|YP_392936.1|	Sulfuromonas denitrificans ATCC 33889	E-value = 1.00E-135	Identity = 52.25%
	5		gi|32265516|ref|NP_859548.1|	Helicobacter hepaticus ATCC 51449	E-value = 2.00E-130	Identity = 50.52%
AB0923	1		gi|152993483|ref|YP_001359204.1|	Sulfurovum sp. NBC37-1	E-value = 3.00E-13	Identity = 34.25%
	2		gi|34557926|ref|NP_907741.1|	Wolinella succinogenes DSM 1740	E-value = 8.00E-10	Identity = 32.09%
	3		gi|78776620|ref|YP_392935.1|	Sulfuromonas denitrificans ATCC 33889	E-value = 2.00E-08	Identity = 33.56%
	4		gi|154174897|ref|YP_001407805.1|	Campylobacter curvus 525.92	E-value = 2.00E-07	Identity = 30.56%
AB0924	1		gi|57639937|ref|YP_182415.1|	Thermococcus kodakarensis KOD1	E-value = 7.00E-19	Identity = 30.96%
	2		gi|149194442|ref|ZP_01871539.1|	Caminibacter mediatlanticus TB-2	E-value = 1.00E-17	Identity = 39.29%
	3		gi|21310089|gb|AAM46140.1|AF380136_1	Helicobacter pylori	E-value = 3.00E-17	Identity = 34.19%
	4		gi|108563216|ref|YP_627532.1|	Helicobacter pylori HPAG1	E-value = 3.00E-17	Identity = 33.55%
	5		gi|15611809|ref|NP_223460.1|	Helicobacter pylori J99	E-value = 8.00E-17	Identity = 34.19%
AB0925	1	*aspB3*	gi|152990562|ref|YP_001356284.1|	Nitratiruptor sp. SB155-2	E-value = 4.00E-142	Identity = 65.80%
	2		gi|78777332|ref|YP_393647.1|	Sulfuromonas denitrificans ATCC 33889	E-value = 1.00E-141	Identity = 67.36%
	3		gi|152992951|ref|YP_001358672.1|	Sulfurovum sp. NBC37-1	E-value = 1.00E-139	Identity = 65.19%
	4		gi|57167758|ref|ZP_00366898.1|	Campylobacter coli RM2228	E-value = 1.00E-131	Identity = 62.34%
	5		gi|86152185|ref|ZP_01070397.1|	Campylobacter jejuni subsp. jejuni 260.94	E-value = 7.00E-131	Identity = 63.12%
AB0926	1		gi|34557976|ref|NP_907791.1|	Wolinella succinogenes DSM 1740	E-value = 2.00E-77	Identity = 57.14%
	2		gi|152990797|ref|YP_001356519.1|	Nitratiruptor sp. SB155-2	E-value = 3.00E-74	Identity = 56.45%
	3		gi|152993476|ref|YP_001359197.1|	Sulfurovum sp. NBC37-1	E-value = 4.00E-72	Identity = 52.92%
	4		gi|78777045|ref|YP_393360.1|	Sulfuromonas denitrificans ATCC 33889	E-value = 1.00E-69	Identity = 53.95%
	5		gi|157415211|ref|YP_001482467.1|	Campylobacter jejuni subsp. jejuni 81116	E-value = 4.00E-64	Identity = 47.95%
AB0927	1		gi|78777128|ref|YP_393443.1|	Sulfuromonas denitrificans ATCC 33889	E-value = 1.00E-161	Identity = 42.75%
AB0928	1	*cyaA*	gi|78777127|ref|YP_393442.1|	Sulfuromonas denitrificans ATCC 33889	E-value = 0	Identity = 48.66%
	2		gi|34557949|ref|NP_907764.1|	Wolinella succinogenes DSM 1740	E-value = 5.00E-175	Identity = 44.73%
	3		gi|152993438|ref|YP_001359159.1|	Sulfurovum sp. NBC37-1	E-value = 1.00E-156	Identity = 43.12%
	4		gi|94266538|ref|ZP_01290225.1|	delta proteobacterium MLMS-1	E-value = 1.00E-125	Identity = 36.51%
	5		gi|94264878|ref|ZP_01288652.1|	delta proteobacterium MLMS-1	E-value = 6.00E-125	Identity = 36.68%
AB0929	1		gi|34557948|ref|NP_907763.1|	Wolinella succinogenes DSM 1740	E-value = 4.00E-23	Identity = 25.35%
	2		gi|94265133|ref|ZP_01288897.1|	delta proteobacterium MLMS-1	E-value = 7.00E-23	Identity = 28.28%
	3		gi|94264704|ref|ZP_01288485.1|	delta proteobacterium MLMS-1	E-value = 2.00E-21	Identity = 27.38%
AB0930	1			***** No matches found *****		
AB0931	1			***** No matches found *****		
AB0932	1		gi|78777412|ref|YP_393727.1|	Sulfuromonas denitrificans ATCC 33889	E-value = 9.00E-78	Identity = 50.66%
	2		gi|90412161|ref|ZP_01220167.1|	Photobacterium profundum 3TCK	E-value = 4.00E-67	Identity = 42.81%
	3		gi|84391791|ref|ZP_00991693.1|	Vibrio splendidus 12B01	E-value = 9.00E-67	Identity = 43.05%
	4		gi|90581197|ref|ZP_01236995.1|	Vibrio angustum S14	E-value = 1.00E-66	Identity = 43.60%
	5		gi|116184950|ref|ZP_01474871.1|	Vibrio sp. Ex25	E-value = 2.00E-66	Identity = 43.77%

AB0933	1		gi|15615091|ref|NP_243394.1|	Bacillus halodurans C-125	E-value = 1.00E-127	Identity = 63.11%
	2		gi|56418997|ref|YP_146315.1|	Geobacillus kaustophilus HTA426	E-value = 3.00E-119	Identity = 60.49%
	3		gi|52082204|ref|YP_080995.1|	Bacillus licheniformis ATCC 14580	E-value = 3.00E-118	Identity = 59.17%
	4		gi|89097967|ref|ZP_01170854.1|	Bacillus sp. NRRL B-14911	E-value = 3.00E-116	Identity = 61.31%
	5		gi|138894141|ref|YP_001124594.1|	Geobacillus thermodenitrificans NG80-2	E-value = 3.00E-116	Identity = 59.75%
AB0934	1		gi|34557061|ref|NP_906876.1|	Wolinella succinogenes DSM 1740	E-value = 2.00E-59	Identity = 51.52%
	2		gi|154174730|ref|YP_001408961.1|	Campylobacter curvus 525.92	E-value = 3.00E-59	Identity = 47.33%
	3		gi|118475653|ref|YP_891375.1|	Campylobacter fetus subsp. fetus 82-40	E-value = 2.00E-58	Identity = 49.37%
	4		gi|154147896|ref|YP_001406133.1|	Campylobacter hominis ATCC BAA-381	E-value = 1.00E-57	Identity = 47.54%
	5		gi|68553688|ref|ZP_00593054.1|	Prosthecochloris aestuarii DSM 271	E-value = 2.00E-56	Identity = 48.12%
AB0935	1	*cbiM*	gi|121543259|ref|ZP_01674947.1|	Candidatus Desulfococcus oleovorans Hxd3	E-value = 7.00E-25	Identity = 39.70%
	2		gi|51244612|ref|YP_064496.1|	Desulfotalea psychrophila LSv54	E-value = 4.00E-23	Identity = 37.56%
	3		gi|154148148|ref|YP_001406131.1|	Campylobacter hominis ATCC BAA-381	E-value = 1.00E-22	Identity = 43.15%
	4		gi|94987305|ref|YP_595238.1|	Lawsonia intracellularis PHE/MN1-00	E-value = 3.00E-22	Identity = 37.31%
	5		gi|83594829|ref|YP_428581.1|	Rhodospirillum rubrum ATCC 11170	E-value = 6.00E-21	Identity = 36.55%
AB0936	1			***** No matches found *****		
AB0938	1		gi|118475061|ref|YP_891378.1|	Campylobacter fetus subsp. fetus 82-40	E-value = 4.00E-19	Identity = 35.68%
	2		gi|154175425|ref|YP_001408958.1|	Campylobacter curvus 525.92	E-value = 6.00E-17	Identity = 32.18%
	3		gi|154148342|ref|YP_001406129.1|	Campylobacter hominis ATCC BAA-381	E-value = 3.00E-16	Identity = 35.71%
AB0939	1		gi|154175272|ref|YP_001408957.1|	Campylobacter curvus 525.92	E-value = 1.00E-55	Identity = 53.05%
	2		gi|118474444|ref|YP_891379.1|	Campylobacter fetus subsp. fetus 82-40	E-value = 2.00E-52	Identity = 50.23%
	3		gi|154149357|ref|YP_001406128.1|	Campylobacter hominis ATCC BAA-381	E-value = 4.00E-52	Identity = 49.77%
	4		gi|126738176|ref|ZP_01753897.1|	Roseobacter sp. SK209-2-6	E-value = 2.00E-38	Identity = 39.41%
	5		gi|157375233|ref|YP_001473833.1|	Shewanella sediminis HAW-EB3	E-value = 7.00E-36	Identity = 38.10%
AB0940	1		gi|157372636|ref|YP_001480625.1|	Serratia proteamaculans 568	E-value = 1.00E-52	Identity = 32.93%
	2		gi|77978573|ref|ZP_00834000.1|	Yersinia intermedia ATCC 29909	E-value = 7.00E-50	Identity = 29.64%
	3		gi|146309719|ref|YP_001174793.1|	Enterobacter sp. 638	E-value = 4.00E-48	Identity = 29.27%
	4		gi|50121044|ref|YP_050211.1|	Erwinia carotovora subsp. atroseptica SCRI1043	E-value = 5.00E-47	Identity = 28.30%
	5		gi|15597659|ref|NP_251153.1|	Pseudomonas aeruginosa PAO1	E-value = 2.00E-45	Identity = 27.02%
AB0941	1			***** No matches found *****		
AB0942	1			***** No matches found *****		
AB0943	1			***** No matches found *****		
AB0944	1			***** No matches found *****		
AB0945	1			***** No matches found *****		
AB0946	1			***** No matches found *****		
AB0947	1			***** No matches found *****		
AB0948	1		gi|78066185|ref|YP_368954.1|	Burkholderia sp. 383	E-value = 3.00E-11	Identity = 26.79%
AB0949	1			***** No matches found *****		
AB0950	1		gi|126734333|ref|ZP_01750080.1|	Roseobacter sp. CCS2	E-value = 1.00E-07	Identity = 29.70%
	2		gi|89056233|ref|YP_511684.1|	Jannaschia sp. CCS1	E-value = 9.00E-07	Identity = 25.00%
AB0951	1		gi|119945466|ref|YP_943146.1|	Psychromonas ingrahamii 37	E-value = 4.00E-24	Identity = 32.23%
	2		gi|121583243|ref|YP_973679.1|	Polaromonas naphthalenivorans CJ2	E-value = 5.00E-21	Identity = 30.21%
	3		gi|77164826|ref|YP_343351.1|	Nitrosococcus oceani ATCC 19707	E-value = 1.00E-16	Identity = 27.78%
	4		gi|92114915|ref|YP_574843.1|	Chromohalobacter salexigens DSM 3043	E-value = 2.00E-14	Identity = 28.45%
	5		gi|153872680|ref|ZP_02001500.1|	Beggiatoa sp. PS	E-value = 5.00E-10	Identity = 26.27%
AB0953	1		gi|154174361|ref|YP_001408609.1|	Campylobacter curvus 525.92	E-value = 1.00E-51	Identity = 49.77%
	2		gi|125718980|ref|YP_001036113.1|	Streptococcus sanguinis SK36	E-value = 8.00E-50	Identity = 50.68%
	3		gi|42526038|ref|NP_971136.1|	Treponema denticola ATCC 35405	E-value = 2.00E-39	Identity = 42.79%
	4		gi|149173397|ref|ZP_01852027.1|	Planctomyces maris DSM 8797	E-value = 1.00E-37	Identity = 41.52%

	5	gi|116249363|ref|YP_765204.1|	Rhizobium leguminosarum bv. viciae 3841	E-value = 5.00E-34	Identity = 39.19%
AB0954	1	gi|78777366|ref|YP_393681.1|	Sulfuromonas denitrificans ATCC 33889	E-value = 5.00E-48	Identity = 36.93%
AB0955	1	gi|118590999|ref|ZP_01548399.1|	Stappia aggregata IAM 12614	E-value = 2.00E-44	Identity = 33.23%
	2	gi|52842249|ref|YP_096048.1|	Legionella pneumophila subsp. pneumophila str. Philadelphia 1	E-value = 6.00E-44	Identity = 34.26%
	3	gi|148359604|ref|YP_001250811.1|	Legionella pneumophila str. Corby	E-value = 8.00E-44	Identity = 34.26%
	4	gi|54294930|ref|YP_127345.1|	Legionella pneumophila str. Lens	E-value = 1.00E-43	Identity = 34.26%
	5	gi|113931933|ref|ZP_01417835.1|	Caulobacter sp. K31	E-value = 9.00E-43	Identity = 31.69%
AB0956	1		***** No matches found *****		
AB0957	1		***** No matches found *****		
AB0958	1	gi|157165103|ref|YP_001466306.1|	Campylobacter concisus 13826	E-value = 0	Identity = 51.73%
	2	gi|154174490|ref|YP_001408849.1|	Campylobacter curvus 525.92	E-value = 0	Identity = 50.96%
	3	gi|154148573|ref|YP_001407040.1|	Campylobacter hominis ATCC BAA-381	E-value = 0	Identity = 46.61%
	4	gi|32474356|ref|NP_867350.1|	Rhodopirellula baltica SH 1	E-value = 0	Identity = 40.81%
	5	gi|152991630|ref|YP_001357351.1|	Sulfurovum sp. NBC37-1	E-value = 2.00E-177	Identity = 44.32%
AB0959	1		***** No matches found *****		
AB0960	1 *uvrA*	gi|152990935|ref|YP_001356657.1|	Nitratiruptor sp. SB155-2	E-value = 0	Identity = 73.69%
	2	gi|152992905|ref|YP_001358626.1|	Sulfurovum sp. NBC37-1	E-value = 0	Identity = 74.04%
	3	gi|154174446|ref|YP_001408842.1|	Campylobacter curvus 525.92	E-value = 0	Identity = 72.97%
	4	gi|78777361|ref|YP_393676.1|	Sulfuromonas denitrificans ATCC 33889	E-value = 0	Identity = 75.05%
	5	gi|57168481|ref|ZP_00367615.1|	Campylobacter coli RM2228	E-value = 0	Identity = 73.11%
AB0961	1	gi|78223485|ref|YP_385232.1|	Geobacter metallireducens GS-15	E-value = 1.00E-29	Identity = 38.46%
	2	gi|76803928|gb|ABA55871.1|	Vibrio sp. DAT722	E-value = 2.00E-13	Identity = 31.03%
AB0962	1	gi|78777849|ref|YP_394164.1|	Sulfuromonas denitrificans ATCC 33889	E-value = 3.00E-41	Identity = 66.13%
	2	gi|152993485|ref|YP_001359206.1|	Sulfurovum sp. NBC37-1	E-value = 2.00E-27	Identity = 47.15%
	3	gi|89094324|ref|ZP_01167265.1|	Oceanospirillum sp. MED92	E-value = 9.00E-27	Identity = 50.86%
	4	gi|154174054|ref|YP_001408339.1|	Campylobacter curvus 525.92	E-value = 4.00E-26	Identity = 46.27%
	5	gi|86151085|ref|ZP_01069301.1|	Campylobacter jejuni subsp. jejuni 260.94	E-value = 3.00E-25	Identity = 44.27%
AB0963	1 *ggt*	gi|50083726|ref|YP_045236.1|	Acinetobacter sp. ADP1	E-value = 0	Identity = 86.79%
	2	gi|152981219|ref|YP_001352291.1|	Janthinobacterium sp. Marseille	E-value = 0	Identity = 65.20%
	3	gi|16080663|ref|NP_391491.1|	Bacillus subtilis subsp. subtilis str. 168	E-value = 4.00E-131	Identity = 45.91%
	4	gi|76801570|ref|YP_326578.1|	Natronomonas pharaonis DSM 2160	E-value = 7.00E-130	Identity = 46.34%
	5	gi|154687727|ref|YP_001422888.1|	Bacillus amyloliquefaciens FZB42	E-value = 5.00E-125	Identity = 44.81%
AB0964	1	gi|152990936|ref|YP_001356658.1|	Nitratiruptor sp. SB155-2	E-value = 1.00E-27	Identity = 43.43%
	2	gi|157165283|ref|YP_001466468.1|	Campylobacter concisus 13826	E-value = 8.00E-27	Identity = 40.20%
	3	gi|154175043|ref|YP_001408863.1|	Campylobacter curvus 525.92	E-value = 1.00E-24	Identity = 39.29%
	4	gi|34557943|ref|NP_907758.1|	Wolinella succinogenes DSM 1740	E-value = 7.00E-24	Identity = 38.89%
	5	gi|149194015|ref|ZP_01871113.1|	Caminibacter mediatlanticus TB-2	E-value = 8.00E-24	Identity = 40.50%
AB0966	1 *cysE*	gi|152990563|ref|YP_001356285.1|	Nitratiruptor sp. SB155-2	E-value = 1.00E-73	Identity = 59.83%
	2	gi|154148205|ref|YP_001406488.1|	Campylobacter hominis ATCC BAA-381	E-value = 1.00E-72	Identity = 59.13%
	3	gi|78777331|ref|YP_393646.1|	Sulfuromonas denitrificans ATCC 33889	E-value = 1.00E-71	Identity = 56.84%
	4	gi|152992950|ref|YP_001358671.1|	Sulfurovum sp. NBC37-1	E-value = 2.00E-71	Identity = 57.76%
	5	gi|32266733|ref|NP_860765.1|	Helicobacter hepaticus ATCC 51449	E-value = 4.00E-70	Identity = 58.19%
AB0967	1 *speA*	gi|152990564|ref|YP_001356286.1|	Nitratiruptor sp. SB155-2	E-value = 0	Identity = 63.17%
	2	gi|152992949|ref|YP_001358670.1|	Sulfurovum sp. NBC37-1	E-value = 0	Identity = 59.32%
	3	gi|118474532|ref|YP_892067.1|	Campylobacter fetus subsp. fetus 82-40	E-value = 0	Identity = 59.64%
	4	gi|57167760|ref|ZP_00366900.1|	Campylobacter coli RM2228	E-value = 0	Identity = 60.23%
	5	gi|57241218|ref|ZP_00369165.1|	Campylobacter lari RM2100	E-value = 0	Identity = 59.8%
AB0968	1 *hisS*	gi|78777328|ref|YP_393643.1|	Sulfuromonas denitrificans ATCC 33889	E-value = 4.00E-149	Identity = 63.84%
	2	gi|152992944|ref|YP_001358665.1|	Sulfurovum sp. NBC37-1	E-value = 1.00E-144	Identity = 61.60%

	3		gi|152990565|ref|YP_001356287.1|	Nitratiruptor sp. SB155-2	E-value = 3.00E-132	Identity = 57.46%
	4		gi|149194438|ref|ZP_01871535.1|	Caminibacter mediatlanticus TB-2	E-value = 2.00E-130	Identity = 60.93%
	5		gi|118474445|ref|YP_892066.1|	Campylobacter fetus subsp. fetus 82-40	E-value = 2.00E-129	Identity = 57.71%
AB0969	1	*tmk*	gi|34557029|ref|NP_906844.1|	Wolinella succinogenes DSM 1740	E-value = 9.00E-50	Identity = 56.74%
	2		gi|152992943|ref|YP_001358664.1|	Sulfurovum sp. NBC37-1	E-value = 2.00E-42	Identity = 49.21%
	3		gi|149193824|ref|ZP_01870922.1|	Caminibacter mediatlanticus TB-2	E-value = 5.00E-40	Identity = 55.87%
	4		gi|78777324|ref|YP_393639.1|	Sulfuromonas denitrificans ATCC 33889	E-value = 5.00E-40	Identity = 52.88%
	5		gi|152990566|ref|YP_001356288.1|	Nitratiruptor sp. SB155-2	E-value = 2.00E-39	Identity = 50.79%
AB0970	1	*coaD*	gi|157165743|ref|YP_001466857.1|	Campylobacter concisus 13826	E-value = 5.00E-49	Identity = 61.33%
	2		gi|57167763|ref|ZP_00366903.1|	Campylobacter coli RM2228	E-value = 2.00E-46	Identity = 60.96%
	3		gi|154174638|ref|YP_001408261.1|	Campylobacter curvus 525.92	E-value = 3.00E-46	Identity = 60.14%
	4		gi|15792105|ref|NP_281928.1|	Campylobacter jejuni subsp. jejuni NCTC 11168	E-value = 2.00E-45	Identity = 59.59%
	5		gi|32266860|ref|NP_860892.1|	Helicobacter hepaticus ATCC 51449	E-value = 2.00E-45	Identity = 62.24%
AB0971	1	*ubiD*	gi|78777322|ref|YP_393637.1|	Sulfuromonas denitrificans ATCC 33889	E-value = 7.00E-49	Identity = 53.55%
	2		gi|34557027|ref|NP_906842.1|	Wolinella succinogenes DSM 1740	E-value = 6.00E-47	Identity = 52.46%
	3		gi|152992941|ref|YP_001358662.1|	Sulfurovum sp. NBC37-1	E-value = 6.00E-46	Identity = 51.63%
	4		gi|157165444|ref|YP_001466856.1|	Campylobacter concisus 13826	E-value = 2.00E-45	Identity = 51.91%
	5		gi|152990568|ref|YP_001356290.1|	Nitratiruptor sp. SB155-2	E-value = 2.00E-45	Identity = 50.27%
AB0972	1		gi|24376378|ref|NP_720486.1|	Shewanella oneidensis MR-1	E-value = 1.00E-17	Identity = 31.72%
	2		gi|149197831|ref|ZP_01874880.1|	Lentisphaera araneosa HTCC2155	E-value = 7.00E-17	Identity = 32.42%
	3		gi|110601859|ref|ZP_01390024.1|	Geobacter sp. FRC-32	E-value = 4.00E-16	Identity = 29.76%
	4		gi|34558220|ref|NP_908035.1|	Wolinella succinogenes DSM 1740	E-value = 1.00E-15	Identity = 32.21%
	5		gi|149196761|ref|ZP_01873814.1|	Lentisphaera araneosa HTCC2155	E-value = 4.00E-15	Identity = 30.77%
AB0973	1	*rplI*	gi|34557658|ref|NP_907473.1|	Wolinella succinogenes DSM 1740	E-value = 2.00E-30	Identity = 57.24%
	2		gi|152990744|ref|YP_001356466.1|	Nitratiruptor sp. SB155-2	E-value = 2.00E-29	Identity = 60.81%
	3		gi|157164966|ref|YP_001466811.1|	Campylobacter concisus 13826	E-value = 2.00E-28	Identity = 54.05%
	4		gi|78777572|ref|YP_393887.1|	Sulfuromonas denitrificans ATCC 33889	E-value = 2.00E-28	Identity = 52.70%
	5		gi|154175047|ref|YP_001408327.1|	Campylobacter curvus 525.92	E-value = 4.00E-27	Identity = 53.69%
AB0974	1	*hslV*	gi|78777573|ref|YP_393888.1|	Sulfuromonas denitrificans ATCC 33889	E-value = 3.00E-75	Identity = 85.31%
	2		gi|152990745|ref|YP_001356467.1|	Nitratiruptor sp. SB155-2	E-value = 1.00E-70	Identity = 81.46%
	3		gi|86151772|ref|ZP_01069986.1|	Campylobacter jejuni subsp. jejuni 260.94	E-value = 2.00E-69	Identity = 77.53%
	4		gi|57167684|ref|ZP_00366824.1|	Campylobacter coli RM2228	E-value = 5.00E-69	Identity = 76.97%
	5		gi|15792018|ref|NP_281841.1|	Campylobacter jejuni subsp. jejuni NCTC 11168	E-value = 5.00E-69	Identity = 76.97%
AB0975	1	*hslU*	gi|78777574|ref|YP_393889.1|	Sulfuromonas denitrificans ATCC 33889	E-value = 4.00E-144	Identity = 65.46%
	2		gi|34557656|ref|NP_907471.1|	Wolinella succinogenes DSM 1740	E-value = 7.00E-143	Identity = 67.12%
	3		gi|157165582|ref|YP_001466813.1|	Campylobacter concisus 13826	E-value = 2.00E-141	Identity = 65.91%
	4		gi|152990746|ref|YP_001356468.1|	Nitratiruptor sp. SB155-2	E-value = 7.00E-140	Identity = 65.77%
	5		gi|32265808|ref|NP_859840.1|	Helicobacter hepaticus ATCC 51449	E-value = 1.00E-138	Identity = 62.22%
AB0976	1			***** No matches found *****		
AB0977	1		gi|152992820|ref|YP_001358541.1|	Sulfurovum sp. NBC37-1	E-value = 2.00E-74	Identity = 67.15%
	2		gi|78777281|ref|YP_393596.1|	Sulfuromonas denitrificans ATCC 33889	E-value = 2.00E-68	Identity = 63.29%
	3		gi|154174378|ref|YP_001407901.1|	Campylobacter curvus 525.92	E-value = 7.00E-68	Identity = 59.62%
	4		gi|157414923|ref|YP_001482179.1|	Campylobacter jejuni subsp. jejuni 81116	E-value = 1.00E-67	Identity = 61.54%
	5		gi|86153267|ref|ZP_01071471.1|	Campylobacter jejuni subsp. jejuni HB93-13	E-value = 1.00E-67	Identity = 61.54%
AB0978	1		gi|34558356|ref|NP_908171.1|	Wolinella succinogenes DSM 1740	E-value = 2.00E-36	Identity = 28.76%
	2		gi|152990522|ref|YP_001356244.1|	Nitratiruptor sp. SB155-2	E-value = 3.00E-34	Identity = 31.59%
	3		gi|78777280|ref|YP_393595.1|	Sulfuromonas denitrificans ATCC 33889	E-value = 2.00E-33	Identity = 30.33%
	4		gi|57167671|ref|ZP_00366811.1|	Campylobacter coli RM2228	E-value = 2.00E-31	Identity = 28.04%
	5		gi|15792005|ref|NP_281828.1|	Campylobacter jejuni subsp. jejuni NCTC 11168	E-value = 2.00E-28	Identity = 27.80%

AB0979	1	*rlpA*	gi|32266823|ref|NP_860855.1|	Helicobacter hepaticus ATCC 51449	E-value = 1.00E-56	Identity = 44.48%
	2		gi|152992818|ref|YP_001358539.1|	Sulfurovum sp. NBC37-1	E-value = 1.00E-54	Identity = 42.26%
	3		gi|15646178|ref|NP_208362.1|	Helicobacter pylori 26695	E-value = 4.00E-51	Identity = 44.80%
	4		gi|108563945|ref|YP_628261.1|	Helicobacter pylori HPAG1	E-value = 8.00E-51	Identity = 45.80%
	5		gi|154175501|ref|YP_001407903.1|	Campylobacter curvus 525.92	E-value = 7.00E-50	Identity = 41.97%
AB0980	1	*hisB*	gi|152992817|ref|YP_001358538.1|	Sulfurovum sp. NBC37-1	E-value = 1.00E-69	Identity = 66.84%
	2		gi|118475282|ref|YP_892347.1|	Campylobacter fetus subsp. fetus 82-40	E-value = 1.00E-67	Identity = 65.26%
	3		gi|152990524|ref|YP_001356246.1|	Nitratiruptor sp. SB155-2	E-value = 3.00E-66	Identity = 61.05%
	4		gi|154149199|ref|YP_001405905.1|	Campylobacter hominis ATCC BAA-381	E-value = 1.00E-65	Identity = 63.68%
	5		gi|34558354|ref|NP_908169.1|	Wolinella succinogenes DSM 1740	E-value = 1.00E-65	Identity = 58.95%
AB0981	1		gi|152992816|ref|YP_001358537.1|	Sulfurovum sp. NBC37-1	E-value = 1.00E-48	Identity = 61.35%
	2		gi|152990525|ref|YP_001356247.1|	Nitratiruptor sp. SB155-2	E-value = 3.00E-44	Identity = 56.10%
	3		gi|149194643|ref|ZP_01871739.1|	Caminibacter mediatlanticus TB-2	E-value = 1.00E-42	Identity = 57.32%
	4		gi|34558353|ref|NP_908168.1|	Wolinella succinogenes DSM 1740	E-value = 4.00E-41	Identity = 56.36%
	5		gi|78777277|ref|YP_393592.1|	Sulfuromonas denitrificans ATCC 33889	E-value = 1.00E-39	Identity = 51.57%
AB0982	1			***** No matches found *****		
AB0983	1		gi|157165382|ref|YP_001467170.1|	Campylobacter concisus 13826	E-value = 7.00E-23	Identity = 46.94%
	2		gi|154174256|ref|YP_001407907.1|	Campylobacter curvus 525.92	E-value = 1.00E-22	Identity = 45.39%
	3		gi|57242608|ref|ZP_00370545.1|	Campylobacter upsaliensis RM3195	E-value = 7.00E-22	Identity = 39.61%
	4		gi|57167675|ref|ZP_00366815.1|	Campylobacter coli RM2228	E-value = 7.00E-19	Identity = 42.22%
	5		gi|118474366|ref|YP_892344.1|	Campylobacter fetus subsp. fetus 82-40	E-value = 1.00E-18	Identity = 37.33%
AB0984	1		gi|152990528|ref|YP_001356250.1|	Nitratiruptor sp. SB155-2	E-value = 1.00E-52	Identity = 52.55%
	2		gi|152992813|ref|YP_001358534.1|	Sulfurovum sp. NBC37-1	E-value = 1.00E-50	Identity = 53.81%
	3		gi|78777274|ref|YP_393589.1|	Sulfuromonas denitrificans ATCC 33889	E-value = 8.00E-49	Identity = 53.72%
	4		gi|149194640|ref|ZP_01871736.1|	Caminibacter mediatlanticus TB-2	E-value = 2.00E-48	Identity = 56.19%
	5		gi|34558350|ref|NP_908165.1|	Wolinella succinogenes DSM 1740	E-value = 5.00E-48	Identity = 47.24%
AB0985	1		gi|152990529|ref|YP_001356251.1|	Nitratiruptor sp. SB155-2	E-value = 1.00E-41	Identity = 53.95%
	2		gi|154148667|ref|YP_001405910.1|	Campylobacter hominis ATCC BAA-381	E-value = 2.00E-40	Identity = 52.32%
	3		gi|118475358|ref|YP_892342.1|	Campylobacter fetus subsp. fetus 82-40	E-value = 3.00E-39	Identity = 52.70%
	4		gi|152992812|ref|YP_001358533.1|	Sulfurovum sp. NBC37-1	E-value = 1.00E-38	Identity = 55.03%
	5		gi|78777273|ref|YP_393588.1|	Sulfuromonas denitrificans ATCC 33889	E-value = 2.00E-38	Identity = 52.00%
AB0986	1		gi|34557006|ref|NP_906821.1|	Wolinella succinogenes DSM 1740	E-value = 4.00E-16	Identity = 32.91%
	2		gi|30248569|ref|NP_840639.1|	Nitrosomonas europaea ATCC 19718	E-value = 8.00E-15	Identity = 31.61%
	3		gi|34557770|ref|NP_907585.1|	Wolinella succinogenes DSM 1740	E-value = 2.00E-13	Identity = 33.33%
	4		gi|83592774|ref|YP_426526.1|	Rhodospirillum rubrum ATCC 11170	E-value = 2.00E-12	Identity = 30.52%
	5		gi|30249178|ref|NP_841248.1|	Nitrosomonas europaea ATCC 19718	E-value = 3.00E-12	Identity = 29.80%
AB0987	1		gi|34557007|ref|NP_906822.1|	Wolinella succinogenes DSM 1740	E-value = 1.00E-35	Identity = 30.06%
	2		gi|34557769|ref|NP_907584.1|	Wolinella succinogenes DSM 1740	E-value = 2.00E-23	Identity = 25.73%
	3		gi|70730270|ref|YP_260009.1|	Pseudomonas fluorescens Pf-5	E-value = 1.00E-18	Identity = 25.31%
	4		gi|90021090|ref|YP_526917.1|	Saccharophagus degradans 2-40	E-value = 1.00E-14	Identity = 25.62%
	5		gi|71280943|ref|YP_267011.1|	Colwellia psychrerythraea 34H	E-value = 8.00E-14	Identity = 26.42%
AB0988	1		gi|28871270|ref|NP_793889.1|	Pseudomonas syringae pv. tomato str. DC3000	E-value = 7.00E-121	Identity = 36.17%
	2		gi|66047091|ref|YP_236932.1|	Pseudomonas syringae pv. syringae B728a	E-value = 5.00E-119	Identity = 36.29%
	3		gi|152982618|ref|YP_001352446.1|	Janthinobacterium sp. Marseille	E-value = 2.00E-115	Identity = 34.30%
	4		gi|30248820|ref|NP_840890.1|	Nitrosomonas europaea ATCC 19718	E-value = 2.00E-109	Identity = 34.38%
	5		gi|30248981|ref|NP_841051.1|	Nitrosomonas europaea ATCC 19718	E-value = 6.00E-109	Identity = 35.43%
AB0989	1	*argC*	gi|124871586|gb|EAY63302.1|	Burkholderia cenocepacia PC184	E-value = 7.00E-90	Identity = 50.31%
	2		gi|84356216|ref|ZP_00981072.1|	Burkholderia cenocepacia PC184	E-value = 7.00E-90	Identity = 50.31%
	3		gi|107024336|ref|YP_622663.1|	Burkholderia cenocepacia AU 1054	E-value = 1.00E-89	Identity = 50.31%

	4		gi|116688338|ref|YP_833961.1|	Burkholderia cenocepacia HI2424	E-value = 1.00E-89	Identity = 50.31%
	5		gi|134294409|ref|YP_001118144.1|	Burkholderia vietnamiensis G4	E-value = 1.00E-89	Identity = 50.31%
AB0990	1		gi|152992516|ref|YP_001358237.1|	Sulfurovum sp. NBC37-1	E-value = 9.00E-113	Identity = 45.00%
	2		gi|149194355|ref|ZP_01871452.1|	Caminibacter mediatlanticus TB-2	E-value = 3.00E-95	Identity = 43.70%
	3		gi|145620794|ref|ZP_01776811.1|	Geobacter bemidjiensis Bem	E-value = 7.00E-87	Identity = 37.93%
	4		gi|145617707|ref|ZP_01773771.1|	Geobacter bemidjiensis Bem	E-value = 9.00E-86	Identity = 38.95%
	5		gi|95930308|ref|ZP_01313045.1|	Desulfuromonas acetoxidans DSM 684	E-value = 3.00E-85	Identity = 37.79%
AB0991	1		gi|78223846|ref|YP_385593.1|	Geobacter metallireducens GS-15	E-value = 1.00E-21	Identity = 38.98%
	2		gi|91203156|emb|CAJ72795.1|	Candidatus Kuenenia stuttgartiensis	E-value = 7.00E-21	Identity = 40.68%
	3		gi|73535416|pdb|1WLI|A	Desulfovibrio vulgaris	E-value = 5.00E-20	Identity = 41.23%
	4		gi|145620795|ref|ZP_01776812.1|	Geobacter bemidjiensis Bem	E-value = 2.00E-18	Identity = 35%
	5		gi|119356604|ref|YP_911248.1|	Chlorobium phaeobacteroides DSM 266	E-value = 2.00E-17	Identity = 37.29%
AB0992	1		gi|34556834|ref|NP_906649.1|	Wolinella succinogenes DSM 1740	E-value = 2.00E-39	Identity = 43.88%
	2		gi|152992543|ref|YP_001358264.1|	Sulfurovum sp. NBC37-1	E-value = 1.00E-35	Identity = 41.67%
	3		gi|152992517|ref|YP_001358238.1|	Sulfurovum sp. NBC37-1	E-value = 2.00E-30	Identity = 38.54%
	4		gi|32266973|ref|NP_861005.1|	Helicobacter hepaticus ATCC 51449	E-value = 2.00E-24	Identity = 38.04%
	5		gi|154242519|ref|ZP_02024081.1|	Candidatus Nitrosopumilus maritimus SCM1	E-value = 1.00E-19	Identity = 34.03%
AB0993	1		gi|152990417|ref|YP_001356139.1|	Nitratiruptor sp. SB155-2	E-value = 5.00E-134	Identity = 47.99%
	2		gi|78777074|ref|YP_393389.1|	Sulfuromonas denitrificans ATCC 33889	E-value = 7.00E-131	Identity = 48.72%
	3		gi|152992151|ref|YP_001357872.1|	Sulfurovum sp. NBC37-1	E-value = 3.00E-123	Identity = 49.45%
	4		gi|152993097|ref|YP_001358818.1|	Sulfurovum sp. NBC37-1	E-value = 4.00E-118	Identity = 43.99%
	5		gi|124547499|ref|ZP_01706384.1|	Shewanella putrefaciens 200	E-value = 1.00E-50	Identity = 29.13%
AB0994	1		gi|78777075|ref|YP_393390.1|	Sulfuromonas denitrificans ATCC 33889	E-value = 5.00E-142	Identity = 55.17%
	2		gi|152992150|ref|YP_001357871.1|	Sulfurovum sp. NBC37-1	E-value = 3.00E-141	Identity = 50.49%
	3		gi|152990416|ref|YP_001356138.1|	Nitratiruptor sp. SB155-2	E-value = 1.00E-140	Identity = 51.17%
	4		gi|152993098|ref|YP_001358819.1|	Sulfurovum sp. NBC37-1	E-value = 1.00E-130	Identity = 48.89%
	5		gi|124547500|ref|ZP_01706385.1|	Shewanella putrefaciens 200	E-value = 2.00E-33	Identity = 28.19%
AB0995	1		gi|152990415|ref|YP_001356137.1|	Nitratiruptor sp. SB155-2	E-value = 7.00E-44	Identity = 42.21%
	2		gi|152992149|ref|YP_001357870.1|	Sulfurovum sp. NBC37-1	E-value = 4.00E-39	Identity = 41.67%
	3		gi|78777076|ref|YP_393391.1|	Sulfuromonas denitrificans ATCC 33889	E-value = 5.00E-39	Identity = 41.06%
	4		gi|34558839|gb|AAQ75183.1|	Alvinella pompejana epibiont 7G3	E-value = 8.00E-33	Identity = 37.64%
	5		gi|152993096|ref|YP_001358817.1|	Sulfurovum sp. NBC37-1	E-value = 1.00E-30	Identity = 34.36%
AB0996	1		gi|149195340|ref|ZP_01872427.1|	Caminibacter mediatlanticus TB-2	E-value = 2.00E-74	Identity = 41.11%
	2		gi|78777077|ref|YP_393392.1|	Sulfuromonas denitrificans ATCC 33889	E-value = 1.00E-31	Identity = 28.27%
	3		gi|152990414|ref|YP_001356136.1|	Nitratiruptor sp. SB155-2	E-value = 1.00E-27	Identity = 25.74%
	4		gi|152992148|ref|YP_001357869.1|	Sulfurovum sp. NBC37-1	E-value = 4.00E-22	Identity = 25.74%
	5		gi|126662106|ref|ZP_01733105.1|	Flavobacteria bacterium BAL38	E-value = 3.00E-12	Identity = 25.24%
AB0997	1			***** No matches found *****		
AB0998	1	*fliP*	gi|118474799|ref|YP_891929.1|	Campylobacter fetus subsp. fetus 82-40	E-value = 6.00E-64	Identity = 59.41%
	2		gi|57167820|ref|ZP_00366960.1|	Campylobacter coli RM2228	E-value = 3.00E-63	Identity = 59.59%
	3		gi|66735095|gb|AAY53788.1|	Campylobacter jejuni	E-value = 4.00E-63	Identity = 60.87%
	4		gi|57242347|ref|ZP_00370286.1|	Campylobacter upsaliensis RM3195	E-value = 9.00E-63	Identity = 58.26%
	5		gi|86152107|ref|ZP_01070319.1|	Campylobacter jejuni subsp. jejuni 260.94	E-value = 2.00E-62	Identity = 61.23%
AB0999	1	*pbpC*	gi|152990531|ref|YP_001356253.1|	Nitratiruptor sp. SB155-2	E-value = 2.00E-172	Identity = 51.76%
	2		gi|78777271|ref|YP_393586.1|	Sulfuromonas denitrificans ATCC 33889	E-value = 2.00E-166	Identity = 50.67%
	3		gi|154174019|ref|YP_001407911.1|	Campylobacter curvus 525.92	E-value = 8.00E-164	Identity = 49.42%
	4		gi|157164806|ref|YP_001467167.1|	Campylobacter concisus 13826	E-value = 4.00E-163	Identity = 48.34%
	5		gi|57241131|ref|ZP_00369078.1|	Campylobacter lari RM2100	E-value = 3.00E-162	Identity = 48.91%
AB1000	1			***** No matches found *****		

AB1001	1		gi|154173726|ref|YP_001407888.1|	Campylobacter curvus 525.92	E-value = 4.00E-47	Identity = 33.82%
	2		gi|157165489|ref|YP_001467343.1|	Campylobacter concisus 13826	E-value = 3.00E-46	Identity = 33.80%
	3		gi|78776987|ref|YP_393302.1|	Sulfuromonas denitrificans ATCC 33889	E-value = 6.00E-46	Identity = 33.41%
	4		gi|34556596|ref|NP_906411.1|	Wolinella succinogenes DSM 1740	E-value = 2.00E-45	Identity = 30.71%
	5		gi|32266287|ref|NP_860319.1|	Helicobacter hepaticus ATCC 51449	E-value = 2.00E-43	Identity = 30.47%
AB1002	1	*ftsX*	gi|57167611|ref|ZP_00366751.1|	Campylobacter coli RM2228	E-value = 6.00E-26	Identity = 30.71%
	2		gi|57238148|ref|YP_179398.1|	Campylobacter jejuni subsp. jejuni RM1221	E-value = 1.00E-22	Identity = 29.21%
	3		gi|86152534|ref|ZP_01070739.1|	Campylobacter jejuni subsp. jejuni HB93-13	E-value = 1.00E-22	Identity = 29.21%
	4		gi|15792600|ref|NP_282423.1|	Campylobacter jejuni subsp. jejuni NCTC 11168	E-value = 1.00E-22	Identity = 29.21%
	5		gi|157164680|ref|YP_001467344.1|	Campylobacter concisus 13826	E-value = 4.00E-22	Identity = 28.10%
AB1003	1	*ftsE*	gi|34556594|ref|NP_906409.1|	Wolinella succinogenes DSM 1740	E-value = 7.00E-74	Identity = 60.45%
	2		gi|32266285|ref|NP_860317.1|	Helicobacter hepaticus ATCC 51449	E-value = 1.00E-66	Identity = 59.11%
	3		gi|78776985|ref|YP_393300.1|	Sulfuromonas denitrificans ATCC 33889	E-value = 1.00E-66	Identity = 57.53%
	4		gi|152990502|ref|YP_001356224.1|	Nitratiruptor sp. SB155-2	E-value = 3.00E-63	Identity = 55.45%
	5		gi|57167610|ref|ZP_00366750.1|	Campylobacter coli RM2228	E-value = 6.00E-63	Identity = 54.79%
AB1004	1		gi|152990501|ref|YP_001356223.1|	Nitratiruptor sp. SB155-2	E-value = 2.00E-87	Identity = 44.33%
	2		gi|78776984|ref|YP_393299.1|	Sulfuromonas denitrificans ATCC 33889	E-value = 2.00E-83	Identity = 45.73%
	3		gi|152993334|ref|YP_001359055.1|	Sulfurovum sp. NBC37-1	E-value = 4.00E-80	Identity = 45.00%
	4		gi|34556593|ref|NP_906408.1|	Wolinella succinogenes DSM 1740	E-value = 3.00E-75	Identity = 41.75%
	5		gi|118475337|ref|YP_892376.1|	Campylobacter fetus subsp. fetus 82-40	E-value = 2.00E-64	Identity = 40.40%
AB1005	1		gi|152990500|ref|YP_001356222.1|	Nitratiruptor sp. SB155-2	E-value = 7.00E-78	Identity = 40.93%
	2		gi|118474336|ref|YP_892377.1|	Campylobacter fetus subsp. fetus 82-40	E-value = 3.00E-66	Identity = 38.56%
	3		gi|34556592|ref|NP_906407.1|	Wolinella succinogenes DSM 1740	E-value = 4.00E-65	Identity = 36.34%
	4		gi|157415543|ref|YP_001482799.1|	Campylobacter jejuni subsp. jejuni 81116	E-value = 3.00E-58	Identity = 35.15%
	5		gi|121613534|ref|YP_001000955.1|	Campylobacter jejuni subsp. jejuni 81-176	E-value = 3.00E-58	Identity = 35.15%
AB1006	1	*rluD*	gi|152990499|ref|YP_001356221.1|	Nitratiruptor sp. SB155-2	E-value = 2.00E-95	Identity = 60.44%
	2		gi|152993336|ref|YP_001359057.1|	Sulfurovum sp. NBC37-1	E-value = 6.00E-86	Identity = 55.59%
	3		gi|78776982|ref|YP_393297.1|	Sulfuromonas denitrificans ATCC 33889	E-value = 1.00E-85	Identity = 53.25%
	4		gi|34556591|ref|NP_906406.1|	Wolinella succinogenes DSM 1740	E-value = 9.00E-80	Identity = 52.01%
	5		gi|118475501|ref|YP_892378.1|	Campylobacter fetus subsp. fetus 82-40	E-value = 1.00E-79	Identity = 54.06%
AB1007	1	*mrdB*	gi|154174319|ref|YP_001407892.1|	Campylobacter curvus 525.92	E-value = 1.00E-101	Identity = 58.06%
	2		gi|118475772|ref|YP_892379.1|	Campylobacter fetus subsp. fetus 82-40	E-value = 8.00E-101	Identity = 58.36%
	3		gi|152990498|ref|YP_001356220.1|	Nitratiruptor sp. SB155-2	E-value = 6.00E-99	Identity = 58.61%
	4		gi|57238153|ref|YP_179403.1|	Campylobacter jejuni subsp. jejuni RM1221	E-value = 3.00E-98	Identity = 59.05%
	5		gi|86150838|ref|ZP_01069054.1|	Campylobacter jejuni subsp. jejuni 260.94	E-value = 3.00E-98	Identity = 59.05%
AB1008	1		gi|78777308|ref|YP_393623.1|	Sulfuromonas denitrificans ATCC 33889	E-value = 1.00E-14	Identity = 30.77%
	2		gi|57505503|ref|ZP_00371431.1|	Campylobacter upsaliensis RM3195	E-value = 3.00E-06	Identity = 27.57%
AB1009	1	*hemL*	gi|152990470|ref|YP_001356192.1|	Nitratiruptor sp. SB155-2	E-value = 2.00E-171	Identity = 67.61%
	2		gi|152992844|ref|YP_001358565.1|	Sulfurovum sp. NBC37-1	E-value = 8.00E-170	Identity = 69.48%
	3		gi|78777307|ref|YP_393622.1|	Sulfuromonas denitrificans ATCC 33889	E-value = 3.00E-164	Identity = 67.54%
	4		gi|149193936|ref|ZP_01871034.1|	Caminibacter mediatlanticus TB-2	E-value = 2.00E-156	Identity = 63.40%
	5		gi|34558181|ref|NP_907996.1|	Wolinella succinogenes DSM 1740	E-value = 1.00E-153	Identity = 60.76%
AB1010	1		gi|126699264|ref|YP_001088161.1|	Clostridium difficile 630	E-value = 1.00E-27	Identity = 59.22%
	2		gi|77918942|ref|YP_356757.1|	Pelobacter carbinolicus DSM 2380	E-value = 1.00E-27	Identity = 58.65%
	3		gi|88859639|ref|ZP_01134279.1|	Pseudoalteromonas tunicata D2	E-value = 5.00E-27	Identity = 59.62%
	4		gi|78485875|ref|YP_391800.1|	Thiomicrospira crunogena XCL-2	E-value = 6.00E-27	Identity = 56.31%
	5		gi|110601129|ref|ZP_01389329.1|	Geobacter sp. FRC-32	E-value = 7.00E-27	Identity = 60.19%
AB1011	1		gi|109947556|ref|YP_664784.1|	Helicobacter acinonychis str. Sheeba	E-value = 4.00E-19	Identity = 54.12%
	2		gi|152992836|ref|YP_001358557.1|	Sulfurovum sp. NBC37-1	E-value = 1.00E-18	Identity = 51.11%

	3		gi|15611361|ref|NP_223012.1|	Helicobacter pylori J99	E-value = 3.00E-18	Identity = 54.12%
	4		gi|15644935|ref|NP_207105.1|	Helicobacter pylori 26695	E-value = 6.00E-18	Identity = 54.88%
	5		gi|152990471|ref|YP_001356193.1|	Nitratiruptor sp. SB155-2	E-value = 9.00E-18	Identity = 52.63%
AB1012	1		gi|152990472|ref|YP_001356194.1|	Nitratiruptor sp. SB155-2	E-value = 2.00E-16	Identity = 34.10%
	2		gi|154175390|ref|YP_001407948.1|	Campylobacter curvus 525.92	E-value = 2.00E-12	Identity = 30.77%
	3		gi|149193738|ref|ZP_01870836.1|	Caminibacter mediatlanticus TB-2	E-value = 4.00E-11	Identity = 35.23%
	4		gi|152992835|ref|YP_001358556.1|	Sulfurovum sp. NBC37-1	E-value = 4.00E-10	Identity = 30.99%
AB1013	1		gi|78777304|ref|YP_393619.1|	Sulfuromonas denitrificans ATCC 33889	E-value = 1.00E-34	Identity = 39.74%
	2		gi|34558184|ref|NP_907999.1|	Wolinella succinogenes DSM 1740	E-value = 8.00E-29	Identity = 31.28%
	3		gi|152993223|ref|YP_001358944.1|	Sulfurovum sp. NBC37-1	E-value = 1.00E-25	Identity = 34.07%
	4		gi|152990240|ref|YP_001355962.1|	Nitratiruptor sp. SB155-2	E-value = 6.00E-23	Identity = 33.04%
	5		gi|149195053|ref|ZP_01872145.1|	Caminibacter mediatlanticus TB-2	E-value = 2.00E-17	Identity = 32.59%
AB1014	1	*rpoD*	gi|78777303|ref|YP_393618.1|	Sulfuromonas denitrificans ATCC 33889	E-value = 0	Identity = 66.77%
	2		gi|118475308|ref|YP_891711.1|	Campylobacter fetus subsp. fetus 82-40	E-value = 0	Identity = 69.33%
	3		gi|34558185|ref|NP_908000.1|	Wolinella succinogenes DSM 1740	E-value = 0	Identity = 67.94%
	4		gi|152990473|ref|YP_001356195.1|	Nitratiruptor sp. SB155-2	E-value = 0	Identity = 68.69%
	5		gi|157164653|ref|YP_001467098.1|	Campylobacter concisus 13826	E-value = 0	Identity = 68.11%
AB1015	1	*leuD*	gi|78777300|ref|YP_393615.1|	Sulfuromonas denitrificans ATCC 33889	E-value = 3.00E-64	Identity = 75.00%
	2		gi|149194276|ref|ZP_01871373.1|	Caminibacter mediatlanticus TB-2	E-value = 1.00E-63	Identity = 76.22%
	3		gi|152993292|ref|YP_001359013.1|	Sulfurovum sp. NBC37-1	E-value = 2.00E-62	Identity = 72.22%
	4		gi|154174978|ref|YP_001408082.1|	Campylobacter curvus 525.92	E-value = 6.00E-59	Identity = 69.38%
	5		gi|157164743|ref|YP_001467085.1|	Campylobacter concisus 13826	E-value = 2.00E-57	Identity = 71.34%
AB1016	1	*leuB*	gi|34558114|ref|NP_907929.1|	Wolinella succinogenes DSM 1740	E-value = 2.00E-144	Identity = 71.59%
	2		gi|152993291|ref|YP_001359012.1|	Sulfurovum sp. NBC37-1	E-value = 5.00E-143	Identity = 71.88%
	3		gi|78777299|ref|YP_393614.1|	Sulfuromonas denitrificans ATCC 33889	E-value = 2.00E-142	Identity = 70.17%
	4		gi|118475010|ref|YP_892184.1|	Campylobacter fetus subsp. fetus 82-40	E-value = 4.00E-142	Identity = 69.41%
	5		gi|154149000|ref|YP_001406815.1|	Campylobacter hominis ATCC BAA-381	E-value = 6.00E-142	Identity = 70.74%
AB1017	1		gi|83313054|ref|YP_423318.1|	Magnetospirillum magneticum AMB-1	E-value = 3.00E-121	Identity = 64.82%
	2		gi|119947027|ref|YP_944707.1|	Psychromonas ingrahamii 37	E-value = 4.00E-120	Identity = 65.03%
	3		gi|46201869|ref|ZP_00054193.2|	Magnetospirillum magnetotacticum MS-1	E-value = 6.00E-119	Identity = 63.52%
	4		gi|149911504|ref|ZP_01900119.1|	Moritella sp. PE36	E-value = 2.00E-117	Identity = 62.90%
	5		gi|144898842|emb|CAM75706.1|	Magnetospirillum gryphiswaldense MSR-1	E-value = 1.00E-116	Identity = 62.21%
AB1018	1		gi|152992696|ref|YP_001358417.1|	Sulfurovum sp. NBC37-1	E-value = 2.00E-32	Identity = 63.16%
	2		gi|152990279|ref|YP_001356001.1|	Nitratiruptor sp. SB155-2	E-value = 5.00E-32	Identity = 59.83%
	3		gi|154149367|ref|YP_001406842.1|	Campylobacter hominis ATCC BAA-381	E-value = 3.00E-30	Identity = 57.63%
	4		gi|154174144|ref|YP_001408693.1|	Campylobacter curvus 525.92	E-value = 3.00E-28	Identity = 51.75%
	5		gi|57242403|ref|ZP_00370341.1|	Campylobacter upsaliensis RM3195	E-value = 4.00E-28	Identity = 59.66%
AB1019	1	*rpmJ*	gi|94968278|ref|YP_590326.1|	Acidobacteria bacterium Ellin345	E-value = 3.50E-02	Identity = 86.49%
	2		gi|15792896|ref|NP_282719.1|	Campylobacter jejuni subsp. jejuni NCTC 11168	E-value = 4.20E-02	Identity = 81.08%
	3		gi|57238604|ref|YP_179735.1|	Campylobacter jejuni subsp. jejuni RM1221	E-value = 4.20E-02	Identity = 81.08%
	4		gi|121612597|ref|ZP_01067621.1|	Campylobacter jejuni subsp. jejuni CF93-6	E-value = 4.20E-02	Identity = 81.08%
	5		gi|148925735|ref|ZP_01099680.1|	Campylobacter jejuni subsp. jejuni 84-25	E-value = 4.20E-02	Identity = 81.08%
AB1020	1	*rpsM*	gi|154173907|ref|YP_001409134.1|	Campylobacter curvus 525.92	E-value = 1.00E-45	Identity = 83.61%
	2		gi|118475454|ref|YP_891266.1|	Campylobacter fetus subsp. fetus 82-40	E-value = 1.00E-45	Identity = 85.25%
	3		gi|157164203|ref|YP_001467698.1|	Campylobacter concisus 13826	E-value = 4.00E-45	Identity = 82.79%
	4		gi|86152866|ref|ZP_01071071.1|	Campylobacter jejuni subsp. jejuni HB93-13	E-value = 6.00E-44	Identity = 83.47%
	5		gi|57241594|ref|ZP_00369539.1|	Campylobacter lari RM2100	E-value = 1.00E-43	Identity = 82.64%
AB1021	1	*rpsK*	gi|152989996|ref|YP_001355718.1|	Nitratiruptor sp. SB155-2	E-value = 1.00E-46	Identity = 78.46%
	2		gi|34558006|ref|NP_907821.1|	Wolinella succinogenes DSM 1740	E-value = 1.00E-45	Identity = 74.05%

	3		gi|57241593|ref|ZP_00369538.1|	Campylobacter lari RM2100	E-value = 9.00E-45	Identity = 80.77%
	4		gi|118474117|ref|YP_891267.1|	Campylobacter fetus subsp. fetus 82-40	E-value = 4.00E-44	Identity = 80.77%
	5		gi|153951410|ref|YP_001398873.1|	Campylobacter jejuni subsp. doylei 269.97	E-value = 4.00E-44	Identity = 82.14%
AB1022	1	*rpsD*	gi|152993918|ref|YP_001359639.1|	Sulfurovum sp. NBC37-1	E-value = 9.00E-86	Identity = 80.77%
	2		gi|118474712|ref|YP_891268.1|	Campylobacter fetus subsp. fetus 82-40	E-value = 2.00E-83	Identity = 79.33%
	3		gi|78776527|ref|YP_392842.1|	Sulfuromonas denitrificans ATCC 33889	E-value = 9.00E-83	Identity = 78.85%
	4		gi|152989997|ref|YP_001355719.1|	Nitratiruptor sp. SB155-2	E-value = 1.00E-82	Identity = 80.29%
	5		gi|154174170|ref|YP_001409132.1|	Campylobacter curvus 525.92	E-value = 2.00E-82	Identity = 77.40%
AB1023	1	*rpoA*	gi|34558004|ref|NP_907819.1|	Wolinella succinogenes DSM 1740	E-value = 3.00E-108	Identity = 60.12%
	2		gi|32266902|ref|NP_860934.1|	Helicobacter hepaticus ATCC 51449	E-value = 4.00E-103	Identity = 57.98%
	3		gi|78776528|ref|YP_392843.1|	Sulfuromonas denitrificans ATCC 33889	E-value = 5.00E-98	Identity = 54.85%
	4		gi|118475267|ref|YP_891269.1|	Campylobacter fetus subsp. fetus 82-40	E-value = 6.00E-98	Identity = 55.56%
	5		gi|154173639|ref|YP_001409131.1|	Campylobacter curvus 525.92	E-value = 4.00E-97	Identity = 56.33%
AB1024	1	*rplQ*	gi|15792901|ref|NP_282724.1|	Campylobacter jejuni subsp. jejuni NCTC 11168	E-value = 4.00E-35	Identity = 70.69%
	2		gi|153951800|ref|YP_001398876.1|	Campylobacter jejuni subsp. doylei 269.97	E-value = 6.00E-35	Identity = 70.69%
	3		gi|57504892|ref|ZP_00370867.1|	Campylobacter coli RM2228	E-value = 7.00E-35	Identity = 70.69%
	4		gi|57241590|ref|ZP_00369535.1|	Campylobacter lari RM2100	E-value = 7.00E-35	Identity = 71.55%
	5		gi|57242508|ref|ZP_00370446.1|	Campylobacter upsaliensis RM3195	E-value = 2.00E-34	Identity = 69.83%
AB1025	1	*gatA*	gi|149195256|ref|ZP_01872345.1|	Caminibacter mediatlanticus TB-2	E-value = 6.00E-161	Identity = 64.10%
	2		gi|78776972|ref|YP_393287.1|	Sulfuromonas denitrificans ATCC 33889	E-value = 1.00E-155	Identity = 66.15%
	3		gi|154149337|ref|YP_001406469.1|	Campylobacter hominis ATCC BAA-381	E-value = 6.00E-152	Identity = 64.67%
	4		gi|118474307|ref|YP_891787.1|	Campylobacter fetus subsp. fetus 82-40	E-value = 2.00E-151	Identity = 63.33%
	5		gi|152991024|ref|YP_001356746.1|	Nitratiruptor sp. SB155-2	E-value = 5.00E-151	Identity = 64.67%
AB1026	1	*guaB*	gi|152991023|ref|YP_001356745.1|	Nitratiruptor sp. SB155-2	E-value = 0	Identity = 76.72%
	2		gi|149195257|ref|ZP_01872346.1|	Caminibacter mediatlanticus TB-2	E-value = 0	Identity = 75.88%
	3		gi|152993377|ref|YP_001359098.1|	Sulfurovum sp. NBC37-1	E-value = 0	Identity = 73.96%
	4		gi|78776969|ref|YP_393284.1|	Sulfuromonas denitrificans ATCC 33889	E-value = 0	Identity = 74.64%
	5		gi|32266201|ref|NP_860233.1|	Helicobacter hepaticus ATCC 51449	E-value = 0	Identity = 70.83%
AB1027	1		gi|89207496|ref|ZP_01186036.1|	Bacillus weihenstephanensis KBAB4	E-value = 5.00E-25	Identity = 40.82%
	2		gi|118743698|ref|ZP_01591700.1|	Geobacter lovleyi SZ	E-value = 5.00E-22	Identity = 41.43%
	3		gi|149175943|ref|ZP_01854560.1|	Planctomyces maris DSM 8797	E-value = 4.00E-19	Identity = 33.33%
	4		gi|123458083|ref|XP_001316524.1|	Trichomonas vaginalis G3	E-value = 1.00E-15	Identity = 35.04%
	5		gi|157165692|ref|YP_001466497.1|	Campylobacter concisus 13826	E-value = 3.00E-14	Identity = 38.02%
AB1028	1			***** No matches found *****		
AB1029	1		gi|152993951|ref|YP_001359672.1|	Sulfurovum sp. NBC37-1	E-value = 2.00E-33	Identity = 25.14%
	2		gi|152994030|ref|YP_001359751.1|	Sulfurovum sp. NBC37-1	E-value = 5.00E-33	Identity = 25.58%
AB1030	1		gi|78776448|ref|YP_392763.1|	Sulfuromonas denitrificans ATCC 33889	E-value = 4.00E-46	Identity = 47.06%
	2		gi|34557248|ref|NP_907063.1|	Wolinella succinogenes DSM 1740	E-value = 1.00E-29	Identity = 35.59%
	3		gi|154173869|ref|YP_001407525.1|	Campylobacter curvus 525.92	E-value = 7.00E-22	Identity = 29.86%
	4		gi|154148783|ref|YP_001406911.1|	Campylobacter hominis ATCC BAA-381	E-value = 2.00E-20	Identity = 28.38%
	5		gi|152993952|ref|YP_001359673.1|	Sulfurovum sp. NBC37-1	E-value = 2.00E-19	Identity = 27.40%
AB1031	1		gi|119946791|ref|YP_944471.1|	Psychromonas ingrahamii 37	E-value = 2.00E-119	Identity = 51.40%
	2		gi|152995696|ref|YP_001340531.1|	Marinomonas sp. MWYL1	E-value = 4.00E-113	Identity = 46.34%
	3		gi|34495920|ref|NP_900135.1|	Chromobacterium violaceum ATCC 12472	E-value = 1.00E-86	Identity = 37.58%
	4		gi|119859815|ref|ZP_01641225.1|	Pseudomonas putida W619	E-value = 1.00E-83	Identity = 36.79%
	5		gi|104779987|ref|YP_606485.1|	Pseudomonas entomophila L48	E-value = 1.00E-80	Identity = 36.73%
AB1032	1		gi|91763334|ref|ZP_01265298.1|	Candidatus Pelagibacter ubique HTCC1002	E-value = 3.00E-42	Identity = 40.93%
	2		gi|71083629|ref|YP_266349.1|	Candidatus Pelagibacter ubique HTCC1062	E-value = 4.00E-42	Identity = 41.74%
	3		gi|119946790|ref|YP_944470.1|	Psychromonas ingrahamii 37	E-value = 7.00E-41	Identity = 38.43%

	4	gi|152995697|ref|YP_001340532.1|	Marinomonas sp. MWYL1	E-value = 1.00E-38	Identity = 39.44%
	5	gi|34495921|ref|NP_900136.1|	Chromobacterium violaceum ATCC 12472	E-value = 6.00E-35	Identity = 35.85%
AB1033	1		***** No matches found *****		
AB1034	1	gi|119946788|ref|YP_944468.1|	Psychromonas ingrahamii 37	E-value = 8.00E-92	Identity = 40.22%
	2	gi|152995699|ref|YP_001340534.1|	Marinomonas sp. MWYL1	E-value = 1.00E-84	Identity = 38.62%
	3	gi|71083630|ref|YP_266350.1|	Candidatus Pelagibacter ubique HTCC1062	E-value = 7.00E-77	Identity = 37.39%
	4	gi|117924454|ref|YP_865071.1|	Magnetococcus sp. MC-1	E-value = 2.00E-64	Identity = 32.55%
	5	gi|145219243|ref|YP_001129952.1|	Prosthecochloris vibrioformis DSM 265	E-value = 6.00E-63	Identity = 33.70%
AB1035	1	gi|152995700|ref|YP_001340535.1|	Marinomonas sp. MWYL1	E-value = 6.00E-147	Identity = 40.09%
	2	gi|119946787|ref|YP_944467.1|	Psychromonas ingrahamii 37	E-value = 2.00E-142	Identity = 39.97%
	3	gi|91763332|ref|ZP_01265296.1|	Candidatus Pelagibacter ubique HTCC1002	E-value = 3.00E-138	Identity = 39.14%
	4	gi|71083631|ref|YP_266351.1|	Candidatus Pelagibacter ubique HTCC1062	E-value = 3.00E-138	Identity = 39.14%
	5	gi|145219244|ref|YP_001129953.1|	Prosthecochloris vibrioformis DSM 265	E-value = 4.00E-126	Identity = 34.24%
AB1036	1		***** No matches found *****		
AB1037	1	gi|78777428|ref|YP_393743.1|	Sulfuromonas denitrificans ATCC 33889	E-value = 7.00E-10	Identity = 41.86%
	2	gi|94266013|ref|ZP_01289735.1|	delta proteobacterium MLMS-1	E-value = 2.00E-07	Identity = 34.48%
	3	gi|77918125|ref|YP_355940.1|	Pelobacter carbinolicus DSM 2380	E-value = 2.00E-07	Identity = 40.51%
	4	gi|152991077|ref|YP_001356799.1|	Nitratiruptor sp. SB155-2	E-value = 1.00E-06	Identity = 42.25%
	5	gi|78223140|ref|YP_384887.1|	Geobacter metallireducens GS-15	E-value = 2.00E-06	Identity = 35.00%
AB1038	1	gi|78778263|ref|YP_394578.1|	Sulfuromonas denitrificans ATCC 33889	E-value = 1.00E-93	Identity = 56.25%
	2	gi|152992043|ref|YP_001357764.1|	Sulfurovum sp. NBC37-1	E-value = 5.00E-76	Identity = 46.07%
	3	gi|95929642|ref|ZP_01312384.1|	Desulfuromonas acetoxidans DSM 684	E-value = 2.00E-71	Identity = 43.02%
	4	gi|67939140|ref|ZP_00531651.1|	Chlorobium phaeobacteroides BS1	E-value = 2.00E-69	Identity = 41.06%
	5	gi|78189925|ref|YP_380263.1|	Chlorobium chlorochromatii CaD3	E-value = 9.00E-68	Identity = 41.19%
AB1039	1	gi|78776949|ref|YP_393264.1|	Sulfuromonas denitrificans ATCC 33889	E-value = 5.00E-147	Identity = 49.43%
	2	gi|95928674|ref|ZP_01311421.1|	Desulfuromonas acetoxidans DSM 684	E-value = 5.00E-49	Identity = 26.76%
AB1040	1	gi|78776948|ref|YP_393263.1|	Sulfuromonas denitrificans ATCC 33889	E-value = 8.00E-54	Identity = 53.57%
	2	gi|34557323|ref|NP_907138.1|	Wolinella succinogenes DSM 1740	E-value = 9.00E-25	Identity = 32.58%
	3	gi|157165169|ref|YP_001467840.1|	Campylobacter concisus 13826	E-value = 7.00E-24	Identity = 32.57%
	4	gi|157164839|ref|YP_001466181.1|	Campylobacter concisus 13826	E-value = 2.00E-23	Identity = 34.08%
	5	gi|86152014|ref|ZP_01070227.1|	Campylobacter jejuni subsp. jejuni 260.94	E-value = 2.00E-23	Identity = 32.88%
AB1041	1		***** No matches found *****		
AB1042	1	gi|119356774|ref|YP_911418.1|	Chlorobium phaeobacteroides DSM 266	E-value = 4.00E-51	Identity = 38.13%
	2	gi|110598478|ref|ZP_01386749.1|	Chlorobium ferrooxidans DSM 13031	E-value = 1.00E-50	Identity = 43.65%
	3	gi|21673583|ref|NP_661648.1|	Chlorobium tepidum TLS	E-value = 2.00E-50	Identity = 40.23%
	4	gi|21226672|ref|NP_632594.1|	Methanosarcina mazei Go1	E-value = 4.00E-48	Identity = 41.41%
	5	gi|116620929|ref|YP_823085.1|	Solibacter usitatus Ellin6076	E-value = 5.00E-48	Identity = 39.06%
AB1044	1	gi|34557006|ref|NP_906821.1|	Wolinella succinogenes DSM 1740	E-value = 4.00E-23	Identity = 40.13%
	2	gi|34557770|ref|NP_907585.1|	Wolinella succinogenes DSM 1740	E-value = 2.00E-18	Identity = 40.13%
	3	gi|33598198|ref|NP_885841.1|	Bordetella parapertussis 12822	E-value = 7.00E-18	Identity = 32.28%
	4	gi|30249178|ref|NP_841248.1|	Nitrosomonas europaea ATCC 19718	E-value = 2.00E-17	Identity = 32.89%
	5	gi|70731436|ref|YP_261177.1|	Pseudomonas fluorescens Pf-5	E-value = 8.00E-17	Identity = 32.48%
AB1045	1	gi|34557007|ref|NP_906822.1|	Wolinella succinogenes DSM 1740	E-value = 3.00E-33	Identity = 30.42%
	2	gi|34557769|ref|NP_907584.1|	Wolinella succinogenes DSM 1740	E-value = 8.00E-32	Identity = 32.18%
	3	gi|70731396|ref|YP_261137.1|	Pseudomonas fluorescens Pf-5	E-value = 2.00E-25	Identity = 28.71%
	4	gi|123440705|ref|YP_001004697.1|	Yersinia enterocolitica subsp. enterocolitica 8081	E-value = 2.00E-24	Identity = 26.52%
	5	gi|107100752|ref|ZP_01364670.1|	Pseudomonas aeruginosa PACS2	E-value = 5.00E-23	Identity = 28.24%
AB1046	1	gi|154503261|ref|ZP_02040321.1|	Ruminococcus gnavus ATCC 29149	E-value = 2.00E-142	Identity = 68.77%
	2	gi|67475633|ref|XP_653507.1|	Entamoeba histolytica HM-1:IMSS	E-value = 1.00E-139	Identity = 69.05%

	3		gi|123456594|ref|XP_001316031.1|	Trichomonas vaginalis G3	E-value = 3.00E-139	Identity = 67.62%
	4		gi|124485586|ref|YP_001030202.1|	Methanocorpusculum labreanum Z	E-value = 2.00E-137	Identity = 68.77%
	5		gi|153940566|ref|YP_001392185.1|	Clostridium botulinum F str. Langeland	E-value = 9.00E-137	Identity = 67.52%
AB1047	1		gi|119475255|ref|ZP_01615608.1|	marine gamma proteobacterium HTCC2143	E-value = 2.00E-41	Identity = 40.49%
	2		gi|54296584|ref|YP_122953.1|	Legionella pneumophila str. Paris	E-value = 2.00E-37	Identity = 41.46%
	3		gi|52840798|ref|YP_094597.1|	Legionella pneumophila subsp. pneumophila str. Philadelphia 1	E-value = 2.00E-37	Identity = 41.95%
	4		gi|54293546|ref|YP_125961.1|	Legionella pneumophila str. Lens	E-value = 4.00E-37	Identity = 41.95%
	5		gi|90021159|ref|YP_526986.1|	Saccharophagus degradans 2-40	E-value = 3.00E-35	Identity = 40.20%
AB1048	1		gi|90023635|ref|YP_529462.1|	Saccharophagus degradans 2-40	E-value = 5.00E-40	Identity = 42.49%
	2		gi|88859375|ref|ZP_01134015.1|	Pseudoalteromonas tunicata D2	E-value = 8.00E-37	Identity = 40.96%
	3		gi|56461176|ref|YP_156457.1|	Idiomarina loihiensis L2TR	E-value = 1.00E-35	Identity = 39.18%
	4		gi|109899083|ref|YP_662338.1|	Pseudoalteromonas atlantica T6c	E-value = 1.00E-35	Identity = 39.89%
	5		gi|146302039|ref|YP_001196630.1|	Flavobacterium johnsoniae UW101	E-value = 1.00E-29	Identity = 35.38%
AB1049	1		gi|149909936|ref|ZP_01898585.1|	Moritella sp. PE36	E-value = 1.00E-60	Identity = 50.00%
	2		gi|95928960|ref|ZP_01311705.1|	Desulfuromonas acetoxidans DSM 684	E-value = 2.00E-59	Identity = 43.49%
	3		gi|71281249|ref|YP_270708.1|	Colwellia psychrerythraea 34H	E-value = 6.00E-58	Identity = 50.00%
	4		gi|54302016|ref|YP_132009.1|	Photobacterium profundum SS9	E-value = 3.00E-56	Identity = 47.04%
	5		gi|90411431|ref|ZP_01219442.1|	Photobacterium profundum 3TCK	E-value = 9.00E-55	Identity = 47.39%
AB1050	1		gi|15896187|ref|NP_349536.1|	Clostridium acetobutylicum ATCC 824	E-value = 5.00E-19	Identity = 41.07%
	2		gi|154149562|ref|YP_001403180.1|	Candidatus Methanoregula boonei 6A8	E-value = 8.00E-19	Identity = 40.00%
	3		gi|118579542|ref|YP_900792.1|	Pelobacter propionicus DSM 2379	E-value = 3.00E-18	Identity = 38.61%
	4		gi|134046172|ref|YP_001097657.1|	Methanococcus maripaludis C5	E-value = 5.00E-18	Identity = 41.12%
	5		gi|118444543|ref|YP_879205.1|	Clostridium novyi NT	E-value = 9.00E-18	Identity = 39.45%
AB1051	1		gi|37520911|ref|NP_924288.1|	Gloeobacter violaceus PCC 7421	E-value = 1.00E-36	Identity = 45.81%
	2		gi|154687715|ref|YP_001422876.1|	Bacillus amyloliquefaciens FZB42	E-value = 1.00E-34	Identity = 44.44%
	3		gi|16079700|ref|NP_390524.1|	Bacillus subtilis subsp. subtilis str. 168	E-value = 3.00E-34	Identity = 45.03%
	4		gi|16080652|ref|NP_391480.1|	Bacillus subtilis subsp. subtilis str. 168	E-value = 4.00E-34	Identity = 45.03%
	5		gi|154684981|ref|YP_001420142.1|	Bacillus amyloliquefaciens FZB42	E-value = 7.00E-34	Identity = 43.53%
AB1052	1		gi|89093655|ref|ZP_01166602.1|	Oceanospirillum sp. MED92	E-value = 1.00E-66	Identity = 38.59%
	2		gi|94314188|ref|YP_587397.1|	Ralstonia metallidurans CH34	E-value = 8.00E-57	Identity = 29.29%
	3		gi|124546735|ref|ZP_01705770.1|	Shewanella putrefaciens 200	E-value = 2.00E-55	Identity = 33.73%
	4		gi|120598907|ref|YP_963481.1|	Shewanella sp. W3-18-1	E-value = 3.00E-55	Identity = 33.09%
	5		gi|114047606|ref|YP_738156.1|	Shewanella sp. MR-7	E-value = 4.00E-55	Identity = 34.52%
AB1053	1	*sugE*	gi|75239117|ref|ZP_00723097.1|	Escherichia coli F11	E-value = 3.00E-22	Identity = 58.65%
	2		gi|91213700|ref|YP_543686.1|	Escherichia coli UTI89	E-value = 3.00E-22	Identity = 58.65%
	3		gi|39995813|ref|NP_951764.1|	Geobacter sulfurreducens PCA	E-value = 2.00E-21	Identity = 59.62%
	4		gi|32476724|ref|NP_869718.1|	Rhodopirellula baltica SH 1	E-value = 4.00E-21	Identity = 57.14%
	5		gi|84319845|ref|ZP_00968232.1|	Pseudomonas aeruginosa C3719	E-value = 6.00E-21	Identity = 59.22%
AB1054	1		gi|149909936|ref|ZP_01898585.1|	Moritella sp. PE36	E-value = 3.00E-56	Identity = 48.23%
	2		gi|95928960|ref|ZP_01311705.1|	Desulfuromonas acetoxidans DSM 684	E-value = 1.00E-55	Identity = 43.15%
	3		gi|54302016|ref|YP_132009.1|	Photobacterium profundum SS9	E-value = 2.00E-53	Identity = 48.07%
	4		gi|90411431|ref|ZP_01219442.1|	Photobacterium profundum 3TCK	E-value = 1.00E-51	Identity = 47.37%
	5		gi|71281249|ref|YP_270708.1|	Colwellia psychrerythraea 34H	E-value = 6.00E-47	Identity = 42.11%
AB1055	1		gi|57168912|ref|ZP_00368042.1|	Campylobacter coli RM2228	E-value = 3.00E-24	Identity = 32.59%
	2		gi|15792369|ref|NP_282192.1|	Campylobacter jejuni subsp. jejuni NCTC 11168	E-value = 6.00E-23	Identity = 32.12%
	3		gi|57240657|ref|ZP_00368605.1|	Campylobacter lari RM2100	E-value = 9.00E-21	Identity = 29.63%
	4		gi|124009367|ref|ZP_01694045.1|	Microscilla marina ATCC 23134	E-value = 1.00E-20	Identity = 28.27%
	5		gi|88713586|ref|ZP_01107668.1|	Flavobacteriales bacterium HTCC2170	E-value = 2.00E-20	Identity = 30.80%
AB1056	1		gi|152992554|ref|YP_001358275.1|	Sulfurovum sp. NBC37-1	E-value = 3.00E-55	Identity = 60.57%

	2	gi|146299752|ref|YP_001194343.1|	Flavobacterium johnsoniae UW101	E-value = 8.00E-53	Identity = 59.88%
	3	gi|150025085|ref|YP_001295911.1|	Flavobacterium psychrophilum JIP02/86	E-value = 3.00E-51	Identity = 57.80%
	4	gi|88803113|ref|ZP_01118639.1|	Polaribacter irgensii 23-P	E-value = 1.00E-50	Identity = 56.59%
	5	gi|124009366|ref|ZP_01694044.1|	Microscilla marina ATCC 23134	E-value = 5.00E-49	Identity = 52.97%
AB1057	1	gi|149194707|ref|ZP_01871802.1|	Caminibacter mediatlanticus TB-2	E-value = 6.00E-56	Identity = 49.30%
	2	gi|152991613|ref|YP_001357334.1|	Sulfurovum sp. NBC37-1	E-value = 2.00E-48	Identity = 38.73%
	3	gi|152989918|ref|YP_001355640.1|	Nitratiruptor sp. SB155-2	E-value = 3.00E-40	Identity = 34.02%
	4	gi|37526745|ref|NP_930089.1|	Photorhabdus luminescens subsp. laumondii TTO1	E-value = 8.00E-36	Identity = 31.38%
	5	gi|50121648|ref|YP_050815.1|	Erwinia carotovora subsp. atroseptica SCRI1043	E-value = 4.00E-34	Identity = 30.45%
AB1058	1	gi|150008437|ref|YP_001303180.1|	Parabacteroides distasonis ATCC 8503	E-value = 5.00E-79	Identity = 49.85%
	2	gi|154490143|ref|ZP_02030404.1|	Parabacteroides merdae ATCC 43184	E-value = 6.00E-79	Identity = 50.44%
	3	gi|29347329|ref|NP_810832.1|	Bacteroides thetaiotaomicron VPI-5482	E-value = 3.00E-76	Identity = 51.21%
	4	gi|153809401|ref|ZP_01962069.1|	Bacteroides caccae ATCC 43185	E-value = 3.00E-75	Identity = 49.70%
	5	gi|60680800|ref|YP_210944.1|	Bacteroides fragilis NCTC 9343	E-value = 4.00E-74	Identity = 50.45%
AB1059	1	gi|149194763|ref|ZP_01871858.1|	Caminibacter mediatlanticus TB-2	E-value = 8.00E-26	Identity = 35.80%
	2	gi|118577386|ref|YP_899626.1|	Pelobacter propionicus DSM 2379	E-value = 6.00E-12	Identity = 28.29%
	3	gi|145618143|ref|ZP_01774204.1|	Geobacter bemidjiensis Bem	E-value = 4.00E-11	Identity = 28.64%
	4	gi|78222366|ref|YP_384113.1|	Geobacter metallireducens GS-15	E-value = 9.00E-11	Identity = 27.91%
	5	gi|15607050|ref|NP_214432.1|	Aquifex aeolicus VF5	E-value = 3.00E-10	Identity = 28.40%
AB1060	1	gi|78777357|ref|YP_393672.1|	Sulfuromonas denitrificans ATCC 33889	E-value = 7.00E-70	Identity = 60.27%
	2	gi|145621268|ref|ZP_01777245.1|	Geobacter bemidjiensis Bem	E-value = 7.00E-46	Identity = 39.33%
	3	gi|56461212|ref|YP_156493.1|	Idiomarina loihiensis L2TR	E-value = 1.00E-45	Identity = 38.85%
	4	gi|117923887|ref|YP_864504.1|	Magnetococcus sp. MC-1	E-value = 9.00E-43	Identity = 40.48%
	5	gi|85711171|ref|ZP_01042231.1|	Idiomarina baltica OS145	E-value = 1.00E-41	Identity = 40.73%
AB1061	1	gi|15896187|ref|NP_349536.1|	Clostridium acetobutylicum ATCC 824	E-value = 3.00E-23	Identity = 47.66%
	2	gi|34557539|ref|NP_907354.1|	Wolinella succinogenes DSM 1740	E-value = 3.00E-22	Identity = 44.04%
	3	gi|150017921|ref|YP_001310175.1|	Clostridium beijerinckii NCIMB 8052	E-value = 6.00E-22	Identity = 47.52%
	4	gi|68056146|ref|ZP_00540278.1|	Exiguobacterium sibiricum 255-15	E-value = 3.00E-21	Identity = 45.79%
	5	gi|89098840|ref|ZP_01171721.1|	Bacillus sp. NRRL B-14911	E-value = 4.00E-21	Identity = 46.08%
AB1062	1	gi|148652985|ref|YP_001280078.1|	Psychrobacter sp. PRwf-1	E-value = 3.00E-45	Identity = 45.36%
	2	gi|123443842|ref|YP_001007813.1|	Yersinia enterocolitica subsp. enterocolitica 8081	E-value = 1.00E-43	Identity = 45.69%
	3	gi|77978324|ref|ZP_00833755.1|	Yersinia intermedia ATCC 29909	E-value = 1.00E-43	Identity = 45.69%
	4	gi|16120995|ref|NP_404308.1|	Yersinia pestis CO92	E-value = 3.00E-43	Identity = 45.41%
	5	gi|77974144|ref|ZP_00829686.1|	Yersinia frederiksenii ATCC 33641	E-value = 4.00E-43	Identity = 48.62%
AB1063	1	gi|39995367|ref|NP_951318.1|	Geobacter sulfurreducens PCA	E-value = 2.00E-10	Identity = 32.29%
	2	gi|148262648|ref|YP_001229354.1|	Geobacter uraniumreducens Rf4	E-value = 1.00E-09	Identity = 38.30%
	3	gi|157370139|ref|YP_001478128.1|	Serratia proteamaculans 568	E-value = 1.00E-09	Identity = 36.56%
	4	gi|121635232|ref|YP_975477.1|	Neisseria meningitidis FAM18	E-value = 5.00E-09	Identity = 38.89%
	5	gi|15794657|ref|NP_284479.1|	Neisseria meningitidis Z2491	E-value = 6.00E-09	Identity = 38.89%
AB1064	1	gi|95931414|ref|ZP_01314122.1|	Desulfuromonas acetoxidans DSM 684	E-value = 9.00E-123	Identity = 61.11%
	2	gi|154173753|ref|YP_001407677.1|	Campylobacter curvus 525.92	E-value = 1.00E-101	Identity = 54.71%
	3	gi|118475694|ref|YP_891794.1|	Campylobacter fetus subsp. fetus 82-40	E-value = 3.00E-87	Identity = 50.29%
	4	gi|68054616|ref|ZP_00538773.1|	Exiguobacterium sibiricum 255-15	E-value = 9.00E-77	Identity = 44.05%
	5	gi|30262063|ref|NP_844440.1|	Bacillus anthracis str. Ames	E-value = 2.00E-74	Identity = 44.84%
AB1065	1	gi|78778144|ref|YP_394459.1|	Sulfuromonas denitrificans ATCC 33889	E-value = 9.00E-93	Identity = 64.71%
	2	gi|24213214|ref|NP_710695.1|	Leptospira interrogans serovar Lai str. 56601	E-value = 4.00E-34	Identity = 33.22%
	3	gi|110640174|ref|YP_680384.1|	Cytophaga hutchinsonii ATCC 33406	E-value = 5.00E-32	Identity = 32.65%
	4	gi|116330189|ref|YP_799907.1|	Leptospira borgpetersenii serovar Hardjo-bovis JB197	E-value = 1.00E-30	Identity = 30.00%
	5	gi|116329204|ref|YP_798924.1|	Leptospira borgpetersenii serovar Hardjo-bovis L550	E-value = 1.00E-30	Identity = 30.00%

AB1066	1		gi|78777356|ref|YP_393671.1|	Sulfuromonas denitrificans ATCC 33889	E-value = 4.00E-81	Identity = 68.57%
	2		gi|152992515|ref|YP_001358236.1|	Sulfurovum sp. NBC37-1	E-value = 9.00E-74	Identity = 65.05%
	3		gi|34558820|gb|AAQ75164.1|	Alvinella pompejana epibiont 7G3	E-value = 8.00E-66	Identity = 58.45%
	4		gi|152991588|ref|YP_001357310.1|	Nitratiruptor sp. SB155-2	E-value = 1.00E-56	Identity = 52.88%
	5		gi|154173699|ref|YP_001408677.1|	Campylobacter curvus 525.92	E-value = 3.00E-47	Identity = 51.92%
AB1067	1			***** No matches found *****		
AB1068	1		gi|34558488|ref|NP_908303.1|	Wolinella succinogenes DSM 1740	E-value = 0	Identity = 68.98%
	2		gi|152993062|ref|YP_001358783.1|	Sulfurovum sp. NBC37-1	E-value = 0	Identity = 68.5%
	3		gi|78777704|ref|YP_394019.1|	Sulfuromonas denitrificans ATCC 33889	E-value = 0	Identity = 70.06%
	4		gi|15645472|ref|NP_207647.1|	Helicobacter pylori 26695	E-value = 0	Identity = 67.29%
	5		gi|108563262|ref|YP_627578.1|	Helicobacter pylori HPAG1	E-value = 0	Identity = 66.73%
AB1069	1		gi|118050897|ref|ZP_01519446.1|	Comamonas testosteroni KF-1	E-value = 4.00E-48	Identity = 65.00%
	2		gi|146283890|ref|YP_001174043.1|	Pseudomonas stutzeri A1501	E-value = 1.00E-47	Identity = 63.64%
	3		gi|119897558|ref|YP_932771.1|	Azoarcus sp. BH72	E-value = 3.00E-47	Identity = 64.29%
	4		gi|119877843|ref|ZP_01644822.1|	Stenotrophomonas maltophilia R551-3	E-value = 3.00E-47	Identity = 58.28%
	5		gi|121532736|ref|ZP_01664580.1|	Ralstonia pickettii 12J	E-value = 9.00E-47	Identity = 60.93%
AB1070	1		gi|34557239|ref|NP_907054.1|	Wolinella succinogenes DSM 1740	E-value = 2.00E-45	Identity = 32.27%
	2		gi|153952029|ref|YP_001398846.1|	Campylobacter jejuni subsp. doylei 269.97	E-value = 1.00E-40	Identity = 28.76%
AB1071	1	*lig*	gi|152992057|ref|YP_001357778.1|	Sulfurovum sp. NBC37-1	E-value = 0	Identity = 64.51%
	2		gi|78777542|ref|YP_393857.1|	Sulfuromonas denitrificans ATCC 33889	E-value = 0	Identity = 59.35%
	3		gi|157164612|ref|YP_001467146.1|	Campylobacter concisus 13826	E-value = 0	Identity = 56.72%
	4		gi|34558095|ref|NP_907910.1|	Wolinella succinogenes DSM 1740	E-value = 0	Identity = 54.48%
	5		gi|149195296|ref|ZP_01872384.1|	Caminibacter mediatlanticus TB-2	E-value = 0	Identity = 54.65%
AB1072	1		gi|152992208|ref|YP_001357929.1|	Sulfurovum sp. NBC37-1	E-value = 7.00E-156	Identity = 67.86%
	2		gi|157163954|ref|YP_001466095.1|	Campylobacter concisus 13826	E-value = 2.00E-151	Identity = 69.21%
	3		gi|154173717|ref|YP_001407471.1|	Campylobacter curvus 525.92	E-value = 3.00E-148	Identity = 66.41%
	4		gi|57241703|ref|ZP_00369648.1|	Campylobacter lari RM2100	E-value = 3.00E-147	Identity = 66.84%
	5		gi|118475672|ref|YP_892788.1|	Campylobacter fetus subsp. fetus 82-40	E-value = 5.00E-143	Identity = 64.89%
AB1073	1		gi|154175293|ref|YP_001407472.1|	Campylobacter curvus 525.92	E-value = 3.00E-46	Identity = 54.69%
	2		gi|152992209|ref|YP_001357930.1|	Sulfurovum sp. NBC37-1	E-value = 1.00E-42	Identity = 54.59%
	3		gi|34556994|ref|NP_906809.1|	Wolinella succinogenes DSM 1740	E-value = 5.00E-37	Identity = 49.51%
	4		gi|118474118|ref|YP_892786.1|	Campylobacter fetus subsp. fetus 82-40	E-value = 2.00E-36	Identity = 48.28%
	5		gi|157164431|ref|YP_001466094.1|	Campylobacter concisus 13826	E-value = 6.00E-36	Identity = 47.28%
AB1074	1	*dgt*	gi|119471009|ref|ZP_01613568.1|	Alteromonadales bacterium TW-7	E-value = 2.00E-105	Identity = 42.97%
	2		gi|77362030|ref|YP_341604.1|	Pseudoalteromonas haloplanktis TAC125	E-value = 1.00E-101	Identity = 42.91%
	3		gi|146310364|ref|YP_001175438.1|	Enterobacter sp. 638	E-value = 2.00E-101	Identity = 40.71%
	4		gi|157147408|ref|YP_001454727.1|	Citrobacter koseri ATCC BAA-895	E-value = 2.00E-100	Identity = 40.94%
	5		gi|6685388|sp|Q59827|DGTP_SHIBO	Shigella boydii	E-value = 4.00E-100	Identity = 41.37%
AB1076	1	*mloA*	gi|19881268|gb|AAM00873.1|AF486555_4	Campylobacter jejuni	E-value = 3.00E-96	Identity = 57.06%
	2		gi|19881225|gb|AAM00837.1|AF486548_4	Campylobacter jejuni	E-value = 3.00E-96	Identity = 57.06%
	3		gi|19881251|gb|AAM00859.1|AF486552_5	Campylobacter jejuni	E-value = 5.00E-96	Identity = 56.78%
	4		gi|86153169|ref|ZP_01071374.1|	Campylobacter jejuni subsp. jejuni HB93-13	E-value = 6.00E-96	Identity = 56.50%
	5		gi|19881229|gb|AAM00840.1|AF486549_2	Campylobacter jejuni	E-value = 1.00E-95	Identity = 56.78%
AB1077	1		gi|15677601|ref|NP_274759.1|	Neisseria meningitidis MC58	E-value = 7.00E-30	Identity = 29.35%
	2		gi|145956009|ref|ZP_01805007.1|	Clostridium difficile QCD-32g58	E-value = 1.00E-29	Identity = 30.79%
	3		gi|126180058|ref|YP_001048023.1|	Methanoculleus marisnigri JR1	E-value = 1.00E-22	Identity = 30.22%
	4		gi|149196785|ref|ZP_01873838.1|	Lentisphaera araneosa HTCC2155	E-value = 7.00E-21	Identity = 26.02%
	5		gi|150401094|ref|YP_001324860.1|	Methanococcus aeolicus Nankai-3	E-value = 9.00E-20	Identity = 30.28%
AB1080	1	*aroE*	gi|78777476|ref|YP_393791.1|	Sulfuromonas denitrificans ATCC 33889	E-value = 1.00E-68	Identity = 53.54%

	2		gi|152992066|ref|YP_001357787.1|	Sulfurovum sp. NBC37-1	E-value = 8.00E-64	Identity = 50.79%
	3		gi|152990925|ref|YP_001356647.1|	Nitratiruptor sp. SB155-2	E-value = 5.00E-63	Identity = 51.53%
	4		gi|154175195|ref|YP_001407793.1|	Campylobacter curvus 525.92	E-value = 1.00E-60	Identity = 47.33%
	5		gi|157165583|ref|YP_001467465.1|	Campylobacter concisus 13826	E-value = 1.00E-59	Identity = 48.28%
AB1081	1		gi|152991048|ref|YP_001356770.1|	Nitratiruptor sp. SB155-2	E-value = 1.00E-30	Identity = 39.90%
	2		gi|74317073|ref|YP_314813.1|	Thiobacillus denitrificans ATCC 25259	E-value = 2.00E-28	Identity = 33.01%
	3		gi|118579301|ref|YP_900551.1|	Pelobacter propionicus DSM 2379	E-value = 4.00E-27	Identity = 32.64%
	4		gi|34557883|ref|NP_907698.1|	Wolinella succinogenes DSM 1740	E-value = 1.00E-26	Identity = 40.00%
	5		gi|67939622|ref|ZP_00532117.1|	Chlorobium phaeobacteroides BS1	E-value = 9.00E-26	Identity = 35.89%
AB1082	1		gi|78776677|ref|YP_392992.1|	Sulfuromonas denitrificans ATCC 33889	E-value = 2.00E-37	Identity = 43.75%
	2		gi|82523974|emb|CAI78785.1|	uncultured epsilon proteobacterium	E-value = 1.00E-34	Identity = 40.79%
	3		gi|91201670|emb|CAJ74730.1|	Candidatus Kuenenia stuttgartiensis	E-value = 4.00E-29	Identity = 35.54%
	4		gi|68055464|ref|ZP_00539609.1|	Exiguobacterium sibiricum 255-15	E-value = 9.00E-29	Identity = 34.93%
	5		gi|154174292|ref|YP_001407656.1|	Campylobacter curvus 525.92	E-value = 1.00E-28	Identity = 37.67%
AB1083	1	*maeA*	gi|152992111|ref|YP_001357832.1|	Sulfurovum sp. NBC37-1	E-value = 3.00E-134	Identity = 60.67%
	2		gi|154149335|ref|YP_001405725.1|	Campylobacter hominis ATCC BAA-381	E-value = 1.00E-132	Identity = 61.99%
	3		gi|154175276|ref|YP_001408952.1|	Campylobacter curvus 525.92	E-value = 2.00E-131	Identity = 61.74%
	4		gi|152991381|ref|YP_001357103.1|	Nitratiruptor sp. SB155-2	E-value = 8.00E-130	Identity = 61.22%
	5		gi|66043668|ref|YP_233509.1|	Pseudomonas syringae pv. syringae B728a	E-value = 8.00E-126	Identity = 57.39%
AB1084	1	*purU*	gi|151570319|gb|EDN35973.1|	Francisella tularensis subsp. novicida GA99-3549	E-value = 4.00E-95	Identity = 62.82%
	2		gi|151571770|gb|EDN37424.1|	Francisella tularensis subsp. novicida GA99-3548	E-value = 9.00E-95	Identity = 62.82%
	3		gi|118497226|ref|YP_898276.1|	Francisella tularensis subsp. novicida U112	E-value = 2.00E-94	Identity = 62.45%
	4		gi|34557815|ref|NP_907630.1|	Wolinella succinogenes DSM 1740	E-value = 2.00E-92	Identity = 58.48%
	5		gi|152993290|ref|YP_001359011.1|	Sulfurovum sp. NBC37-1	E-value = 3.00E-92	Identity = 62.50%
AB1085	1		gi|57241070|ref|ZP_00369017.1|	Campylobacter lari RM2100	E-value = 2.00E-41	Identity = 56.77%
	2		gi|152993289|ref|YP_001359010.1|	Sulfurovum sp. NBC37-1	E-value = 3.00E-41	Identity = 51.95%
	3		gi|34557814|ref|NP_907629.1|	Wolinella succinogenes DSM 1740	E-value = 4.00E-41	Identity = 51.32%
	4		gi|118474338|ref|YP_891998.1|	Campylobacter fetus subsp. fetus 82-40	E-value = 5.00E-41	Identity = 51.95%
	5		gi|86152124|ref|ZP_01070336.1|	Campylobacter jejuni subsp. jejuni 260.94	E-value = 2.00E-40	Identity = 53.90%
AB1086	1			***** No matches found *****		
AB1087	1		gi|78777160|ref|YP_393475.1|	Sulfuromonas denitrificans ATCC 33889	E-value = 0	Identity = 68.98%
	2		gi|78778137|ref|YP_394452.1|	Sulfuromonas denitrificans ATCC 33889	E-value = 2.00E-134	Identity = 49.19%
	3		gi|89093328|ref|ZP_01166277.1|	Oceanospirillum sp. MED92	E-value = 1.00E-125	Identity = 42.91%
	4		gi|88711423|ref|ZP_01105511.1|	Flavobacteriales bacterium HTCC2170	E-value = 1.00E-110	Identity = 40.67%
	5		gi|117923542|ref|YP_864159.1|	Magnetococcus sp. MC-1	E-value = 1.00E-110	Identity = 40.75%
AB1088	1			***** No matches found *****		
AB1089	1			***** No matches found *****		
AB1090	1		gi|152993763|ref|YP_001359484.1|	Sulfurovum sp. NBC37-1	E-value = 1.00E-46	Identity = 47.93%
	2		gi|152991168|ref|YP_001356890.1|	Nitratiruptor sp. SB155-2	E-value = 3.00E-39	Identity = 45.16%
	3		gi|78776824|ref|YP_393139.1|	Sulfuromonas denitrificans ATCC 33889	E-value = 4.00E-37	Identity = 43.58%
	4		gi|157164687|ref|YP_001466781.1|	Campylobacter concisus 13826	E-value = 1.00E-36	Identity = 40.83%
	5		gi|34556531|ref|NP_906346.1|	Wolinella succinogenes DSM 1740	E-value = 1.00E-36	Identity = 41.20%
AB1091	1		gi|152993762|ref|YP_001359483.1|	Sulfurovum sp. NBC37-1	E-value = 6.00E-36	Identity = 37.12%
	2		gi|152991167|ref|YP_001356889.1|	Nitratiruptor sp. SB155-2	E-value = 2.00E-33	Identity = 40.74%
	3		gi|4104349|gb|AAD02004.1|	Campylobacter rectus	E-value = 3.00E-14	Identity = 29.60%
AB1092	1		gi|157165096|ref|YP_001466403.1|	Campylobacter concisus 13826	E-value = 2.00E-89	Identity = 64.29%
	2		gi|32266355|ref|NP_860387.1|	Helicobacter hepaticus ATCC 51449	E-value = 8.00E-83	Identity = 61.68%
	3		gi|154149444|ref|YP_001406590.1|	Campylobacter hominis ATCC BAA-381	E-value = 3.00E-79	Identity = 63.18%
	4		gi|34557241|ref|NP_907056.1|	Wolinella succinogenes DSM 1740	E-value = 1.00E-78	Identity = 53.20%

	5		gi|118588415|ref|ZP_01545824.1|	Stappia aggregata IAM 12614	E-value = 4.00E-50	Identity = 39.66%
AB1093	1		gi|32266354|ref|NP_860386.1|	Helicobacter hepaticus ATCC 51449	E-value = 3.00E-11	Identity = 33.57%
	2		gi|154148669|ref|YP_001406589.1|	Campylobacter hominis ATCC BAA-381	E-value = 2.00E-10	Identity = 42.11%
	3		gi|157165675|ref|YP_001466404.1|	Campylobacter concisus 13826	E-value = 8.00E-10	Identity = 33.82%
	4		gi|154174670|ref|YP_001407959.1|	Campylobacter curvus 525.92	E-value = 2.00E-06	Identity = 30.22%
AB1094	1		gi|156719420|ref|ZP_02061056.1|	Hydrogenobaculum sp. Y04AAS1	E-value = 1.00E-09	Identity = 32.76%
AB1095	1	*cydB*	gi|154173945|ref|YP_001409214.1|	Campylobacter curvus 525.92	E-value = 2.00E-111	Identity = 60.05%
	2		gi|32266601|ref|NP_860633.1|	Helicobacter hepaticus ATCC 51449	E-value = 6.00E-110	Identity = 59.63%
	3		gi|57168761|ref|ZP_00367892.1|	Campylobacter coli RM2228	E-value = 6.00E-107	Identity = 61.66%
	4		gi|86154030|ref|ZP_01072231.1|	Campylobacter jejuni subsp. jejuni HB93-13	E-value = 5.00E-105	Identity = 63.13%
	5		gi|15791472|ref|NP_281295.1|	Campylobacter jejuni subsp. jejuni NCTC 11168	E-value = 5.00E-105	Identity = 62.60%
AB1096	1	*cydA*	gi|86154010|ref|ZP_01072211.1|	Campylobacter jejuni subsp. jejuni HB93-13	E-value = 0	Identity = 69.88%
	2		gi|157414395|ref|YP_001481651.1|	Campylobacter jejuni subsp. jejuni 81116	E-value = 0	Identity = 69.88%
	3		gi|57237089|ref|YP_178101.1|	Campylobacter jejuni subsp. jejuni RM1221	E-value = 0	Identity = 69.88%
	4		gi|15791471|ref|NP_281294.1|	Campylobacter jejuni subsp. jejuni NCTC 11168	E-value = 0	Identity = 69.68%
	5		gi|86149602|ref|ZP_01067832.1|	Campylobacter jejuni subsp. jejuni CF93-6	E-value = 0	Identity = 69.88%
AB1097	1			***** No matches found *****		
AB1098	1		gi|68552744|ref|ZP_00592130.1|	Prosthecochloris aestuarii DSM 271	E-value = 1.00E-27	Identity = 49.28%
	2		gi|67937922|ref|ZP_00530452.1|	Chlorobium phaeobacteroides BS1	E-value = 1.00E-26	Identity = 52.76%
	3		gi|88792732|ref|ZP_01108450.1|	Alteromonas macleodii 'Deep ecotype'	E-value = 2.00E-26	Identity = 48.98%
	4		gi|21673835|ref|NP_661900.1|	Chlorobium tepidum TLS	E-value = 4.00E-23	Identity = 47.06%
	5		gi|149927292|ref|ZP_01915548.1|	Limnobacter sp. MED105	E-value = 2.00E-18	Identity = 43.80%
AB1099	1		gi|34557380|ref|NP_907195.1|	Wolinella succinogenes DSM 1740	E-value = 3.00E-92	Identity = 52.86%
	2		gi|78189564|ref|YP_379902.1|	Chlorobium chlorochromatii CaD3	E-value = 5.00E-92	Identity = 50.00%
	3		gi|68551751|ref|ZP_00591145.1|	Prosthecochloris aestuarii DSM 271	E-value = 2.00E-84	Identity = 46.34%
	4		gi|88797524|ref|ZP_01113113.1|	Reinekea sp. MED297	E-value = 2.00E-76	Identity = 44.79%
	5		gi|118048683|ref|ZP_01517274.1|	Chloroflexus aggregans DSM 9485	E-value = 5.00E-71	Identity = 40.99%
AB1100	1		gi|110599668|ref|ZP_01387903.1|	Geobacter sp. FRC-32	E-value = 6.00E-20	Identity = 37.91%
	2		gi|145637539|ref|ZP_01793196.1|	Haemophilus influenzae PittHH	E-value = 2.00E-19	Identity = 37.43%
	3		gi|68249923|ref|YP_249035.1|	Haemophilus influenzae 86-028NP	E-value = 1.00E-18	Identity = 36.77%
	4		gi|126640192|ref|YP_001083176.1|	Acinetobacter baumannii ATCC 17978	E-value = 5.00E-18	Identity = 38.13%
	5		gi|148264055|ref|YP_001230761.1|	Geobacter uraniumreducens Rf4	E-value = 1.00E-17	Identity = 33.33%
AB1101	1			***** No matches found *****		
AB1102	1	*aat*	gi|126663053|ref|ZP_01734051.1|	Flavobacteria bacterium BAL38	E-value = 8.00E-54	Identity = 50.94%
	2		gi|86135051|ref|ZP_01053633.1|	Tenacibaculum sp. MED152	E-value = 2.00E-52	Identity = 51.69%
	3		gi|88804827|ref|ZP_01120347.1|	Robiginitalea biformata HTCC2501	E-value = 4.00E-52	Identity = 47.50%
	4		gi|149910268|ref|ZP_01898912.1|	Moritella sp. PE36	E-value = 7.00E-52	Identity = 49.77%
	5		gi|88860140|ref|ZP_01134779.1|	Pseudoalteromonas tunicata D2	E-value = 1.00E-51	Identity = 48.83%
AB1103	1	*rsuA*	gi|78778204|ref|YP_394519.1|	Sulfuromonas denitrificans ATCC 33889	E-value = 6.00E-86	Identity = 64.47%
	2		gi|83311576|ref|YP_421840.1|	Magnetospirillum magneticum AMB-1	E-value = 2.00E-58	Identity = 48.13%
	3		gi|114800237|ref|YP_759483.1|	Hyphomonas neptunium ATCC 15444	E-value = 7.00E-58	Identity = 49.55%
	4		gi|144900938|emb|CAM77802.1|	Magnetospirillum gryphiswaldense MSR-1	E-value = 4.00E-57	Identity = 48.61%
	5		gi|46202419|ref|ZP_00053237.2|	Magnetospirillum magnetotacticum MS-1	E-value = 5.00E-56	Identity = 46.76%
AB1104	1		gi|152992674|ref|YP_001358395.1|	Sulfurovum sp. NBC37-1	E-value = 1.00E-46	Identity = 49.50%
	2		gi|152991591|ref|YP_001357313.1|	Nitratiruptor sp. SB155-2	E-value = 2.00E-37	Identity = 42.64%
	3		gi|78777117|ref|YP_393432.1|	Sulfuromonas denitrificans ATCC 33889	E-value = 8.00E-32	Identity = 39.68%
	4		gi|154148415|ref|YP_001406455.1|	Campylobacter hominis ATCC BAA-381	E-value = 7.00E-27	Identity = 37.70%
	5		gi|154173736|ref|YP_001408393.1|	Campylobacter curvus 525.92	E-value = 3.00E-24	Identity = 35.89%
AB1105	1		gi|78777444|ref|YP_393759.1|	Sulfuromonas denitrificans ATCC 33889	E-value = 7.00E-47	Identity = 50.90%

	2	gi|34556823|ref|NP_906638.1|	Wolinella succinogenes DSM 1740	E-value = 2.00E-24	Identity = 40.37%
	3	gi|78777504|ref|YP_393819.1|	Sulfuromonas denitrificans ATCC 33889	E-value = 3.00E-17	Identity = 35.57%
	4	gi|23015450|ref|ZP_00055226.1|	Magnetospirillum magnetotacticum MS-1	E-value = 3.00E-11	Identity = 31.82%
AB1106	1	gi|78777443|ref|YP_393758.1|	Sulfuromonas denitrificans ATCC 33889	E-value = 2.00E-60	Identity = 60.18%
	2	gi|152992851|ref|YP_001358572.1|	Sulfurovum sp. NBC37-1	E-value = 1.00E-59	Identity = 55.90%
	3	gi|152991484|ref|YP_001357206.1|	Nitratiruptor sp. SB155-2	E-value = 9.00E-52	Identity = 50.66%
	4	gi|34557090|ref|NP_906905.1|	Wolinella succinogenes DSM 1740	E-value = 3.00E-49	Identity = 55.61%
	5	gi|32267034|ref|NP_861066.1|	Helicobacter hepaticus ATCC 51449	E-value = 9.00E-27	Identity = 38.84%
AB1107	1		***** No matches found *****		
AB1108	1	gi|56477715|ref|YP_159304.1|	Azoarcus sp. EbN1	E-value = 6.00E-132	Identity = 42.44%
	2	gi|119899845|ref|YP_935058.1|	Azoarcus sp. BH72	E-value = 9.00E-129	Identity = 40.03%
	3	gi|78776319|ref|YP_392634.1|	Sulfuromonas denitrificans ATCC 33889	E-value = 7.00E-108	Identity = 39.01%
	4	gi|34557598|ref|NP_907413.1|	Wolinella succinogenes DSM 1740	E-value = 1.00E-71	Identity = 31.90%
	5	gi|110833489|ref|YP_692348.1|	Alcanivorax borkumensis SK2	E-value = 7.00E-37	Identity = 26.47%
AB1109	1	gi|78776318|ref|YP_392633.1|	Sulfuromonas denitrificans ATCC 33889	E-value = 4.00E-36	Identity = 38.06%
	2	gi|149200637|ref|ZP_01877639.1|	Lentisphaera araneosa HTCC2155	E-value = 1.00E-20	Identity = 31.32%
	3	gi|91794204|ref|YP_563855.1|	Shewanella denitrificans OS217	E-value = 4.00E-19	Identity = 25.77%
	4	gi|88860274|ref|ZP_01134912.1|	Pseudoalteromonas tunicata D2	E-value = 6.00E-19	Identity = 27.60%
	5	gi|85858072|ref|YP_460274.1|	Syntrophus aciditrophicus SB	E-value = 2.00E-18	Identity = 27.97%
AB1110	1	gi|78776317|ref|YP_392632.1|	Sulfuromonas denitrificans ATCC 33889	E-value = 2.00E-21	Identity = 37.34%
	2	gi|124485972|ref|YP_001030588.1|	Methanocorpusculum labreanum Z	E-value = 9.00E-18	Identity = 27.90%
	3	gi|14521214|ref|NP_126689.1|	Pyrococcus abyssi GE5	E-value = 1.00E-17	Identity = 30.13%
	4	gi|14591054|ref|NP_143129.1|	Pyrococcus horikoshii OT3	E-value = 8.00E-16	Identity = 29.44%
	5	gi|88601601|ref|YP_501779.1|	Methanospirillum hungatei JF-1	E-value = 1.00E-15	Identity = 27.90%
AB1111	1	gi|78776316|ref|YP_392631.1|	Sulfuromonas denitrificans ATCC 33889	E-value = 8.00E-50	Identity = 42.81%
	2	gi|77164356|ref|YP_342881.1|	Nitrosococcus oceani ATCC 19707	E-value = 2.00E-34	Identity = 38.31%
	3	gi|116750166|ref|YP_846853.1|	Syntrophobacter fumaroxidans MPOB	E-value = 2.00E-33	Identity = 33.45%
	4	gi|154150529|ref|YP_001404147.1|	Candidatus Methanoregula boonei 6A8	E-value = 6.00E-33	Identity = 34.04%
	5	gi|52141138|ref|YP_085691.1|	Bacillus cereus E33L	E-value = 1.00E-31	Identity = 29.06%
AB1112	1	gi|110638733|ref|YP_678942.1|	Cytophaga hutchinsonii ATCC 33406	E-value = 3.00E-36	Identity = 46.30%
	2	gi|124002665|ref|ZP_01687517.1|	Microscilla marina ATCC 23134	E-value = 1.00E-35	Identity = 45.06%
	3	gi|67939139|ref|ZP_00531650.1|	Chlorobium phaeobacteroides BS1	E-value = 5.00E-35	Identity = 41.46%
	4	gi|152993478|ref|YP_001359199.1|	Sulfurovum sp. NBC37-1	E-value = 5.00E-34	Identity = 43.21%
	5	gi|119358347|ref|YP_912991.1|	Chlorobium phaeobacteroides DSM 266	E-value = 9.00E-34	Identity = 43.83%
AB1113	1	gi|78776270|ref|YP_392585.1|	Sulfuromonas denitrificans ATCC 33889	E-value = 0	Identity = 65.14%
	2	gi|152993024|ref|YP_001358745.1|	Sulfurovum sp. NBC37-1	E-value = 1.00E-140	Identity = 49.31%
	3	gi|89094030|ref|ZP_01166974.1|	Oceanospirillum sp. MED92	E-value = 2.00E-136	Identity = 43.08%
	4	gi|156974631|ref|YP_001445538.1|	Vibrio harveyi ATCC BAA-1116	E-value = 1.00E-135	Identity = 43.49%
	5	gi|152990485|ref|YP_001356207.1|	Nitratiruptor sp. SB155-2	E-value = 4.00E-135	Identity = 45.47%
AB1114	1		***** No matches found *****		
AB1115	1	gi|152991031|ref|YP_001356753.1|	Nitratiruptor sp. SB155-2	E-value = 6.00E-61	Identity = 59.63%
	2	gi|152992275|ref|YP_001357996.1|	Sulfurovum sp. NBC37-1	E-value = 2.00E-60	Identity = 58.72%
	3	gi|78777562|ref|YP_393877.1|	Sulfuromonas denitrificans ATCC 33889	E-value = 5.00E-52	Identity = 53.67%
	4	gi|152992978|ref|YP_001358699.1|	Sulfurovum sp. NBC37-1	E-value = 6.00E-47	Identity = 49.77%
	5	gi|57242734|ref|ZP_00370671.1|	Campylobacter upsaliensis RM3195	E-value = 1.00E-46	Identity = 47.71%
AB1116	1	gi|152991032|ref|YP_001356754.1|	Nitratiruptor sp. SB155-2	E-value = 9.00E-88	Identity = 46.60%
	2	gi|152992276|ref|YP_001357997.1|	Sulfurovum sp. NBC37-1	E-value = 2.00E-82	Identity = 43.94%
	3	gi|78777563|ref|YP_393878.1|	Sulfuromonas denitrificans ATCC 33889	E-value = 1.00E-70	Identity = 42.05%
	4	gi|152992979|ref|YP_001358700.1|	Sulfurovum sp. NBC37-1	E-value = 5.00E-62	Identity = 34.72%

	5	gi|32267156|ref|NP_861188.1|	Helicobacter hepaticus ATCC 51449	E-value = 1.00E-42	Identity = 28.77%
AB1117	1	gi|152992277|ref|YP_001357998.1|	Sulfurovum sp. NBC37-1	E-value = 5.00E-19	Identity = 46.85%
	2	gi|152992980|ref|YP_001358701.1|	Sulfurovum sp. NBC37-1	E-value = 1.00E-12	Identity = 39.78%
	3	gi|78777564|ref|YP_393879.1|	Sulfuromonas denitrificans ATCC 33889	E-value = 2.00E-10	Identity = 33.04%
	4	gi|152991033|ref|YP_001356755.1|	Nitratiruptor sp. SB155-2	E-value = 1.00E-07	Identity = 28.32%
AB1118	1		***** No matches found *****		
AB1119	1	gi|20807004|ref|NP_622175.1|	Thermoanaerobacter tengcongensis MB4	E-value = 5.00E-07	Identity = 26.11%
	2	gi|77918718|ref|YP_356533.1|	Pelobacter carbinolicus DSM 2380	E-value = 5.00E-06	Identity = 25.62%
AB1120	1	gi|150400976|ref|YP_001324742.1|	Methanococcus aeolicus Nankai-3	E-value = 2.00E-41	Identity = 52.05%
AB1121	1	gi|114778329|ref|ZP_01453188.1|	Mariprofundus ferrooxydans PV-1	E-value = 1.00E-26	Identity = 27.74%
	2	gi|56461165|ref|YP_156446.1|	Idiomarina loihiensis L2TR	E-value = 2.00E-26	Identity = 28.78%
	3	gi|152994439|ref|YP_001339274.1|	Marinomonas sp. MWYL1	E-value = 6.00E-26	Identity = 27.95%
AB1122	1		***** No matches found *****		
AB1123	1	gi|56460652|ref|YP_155933.1|	Idiomarina loihiensis L2TR	E-value = 3.00E-78	Identity = 47.23%
	2	gi|37525462|ref|NP_928806.1|	Photorhabdus luminescens subsp. laumondii TTO1	E-value = 4.00E-78	Identity = 46.13%
	3	gi|134095351|ref|YP_001100426.1|	Herminiimonas arsenicoxydans	E-value = 6.00E-77	Identity = 45.43%
	4	gi|119468751|ref|ZP_01611803.1|	Alteromonadales bacterium TW-7	E-value = 9.00E-76	Identity = 44.44%
	5	gi|77362320|ref|YP_341894.1|	Pseudoalteromonas haloplanktis TAC125	E-value = 3.00E-74	Identity = 43.30%
AB1124	1	gi|118743958|ref|ZP_01591957.1|	Geobacter lovleyi SZ	E-value = 6.00E-52	Identity = 32.16%
	2	gi|152991361|ref|YP_001357083.1|	Nitratiruptor sp. SB155-2	E-value = 3.00E-48	Identity = 38.21%
	3	gi|118745838|ref|ZP_01593802.1|	Geobacter lovleyi SZ	E-value = 5.00E-44	Identity = 29.85%
	4	gi|110599469|ref|ZP_01387707.1|	Geobacter sp. FRC-32	E-value = 6.00E-43	Identity = 32.31%
	5	gi|78777441|ref|YP_393756.1|	Sulfuromonas denitrificans ATCC 33889	E-value = 1.00E-41	Identity = 33.51%
AB1125	1	gi|152993952|ref|YP_001359673.1|	Sulfurovum sp. NBC37-1	E-value = 4.00E-37	Identity = 41.82%
	2	gi|152993408|ref|YP_001359129.1|	Sulfurovum sp. NBC37-1	E-value = 6.00E-32	Identity = 37.61%
	3	gi|34558806|gb|AAQ75151.1|	Alvinella pompejana epibiont 6C6	E-value = 8.00E-32	Identity = 40.36%
	4	gi|78778185|ref|YP_394500.1|	Sulfuromonas denitrificans ATCC 33889	E-value = 4.00E-31	Identity = 35.96%
	5	gi|34557323|ref|NP_907138.1|	Wolinella succinogenes DSM 1740	E-value = 2.00E-30	Identity = 34.40%
AB1126	1		***** No matches found *****		
AB1127	1	gi|154173935|ref|YP_001408654.1|	Campylobacter curvus 525.92	E-value = 0	Identity = 52.79%
	2	gi|157164509|ref|YP_001466532.1|	Campylobacter concisus 13826	E-value = 0	Identity = 53.75%
	3	gi|78484552|ref|YP_390477.1|	Thiomicrospira crunogena XCL-2	E-value = 0	Identity = 47.52%
	4	gi|51244365|ref|YP_064249.1|	Desulfotalea psychrophila LSv54	E-value = 0	Identity = 46.27%
	5	gi|89094686|ref|ZP_01167622.1|	Oceanospirillum sp. MED92	E-value = 3.00E-178	Identity = 46.52%
AB1128	1	gi|50122191|ref|YP_051358.1|	Erwinia carotovora subsp. atroseptica SCRI1043	E-value = 1.00E-87	Identity = 41.75%
	2	gi|157165040|ref|YP_001466533.1|	Campylobacter concisus 13826	E-value = 9.00E-86	Identity = 43.26%
	3	gi|154174251|ref|YP_001408653.1|	Campylobacter curvus 525.92	E-value = 7.00E-85	Identity = 42.33%
	4	gi|119856615|ref|ZP_01638047.1|	Pseudomonas putida W619	E-value = 5.00E-84	Identity = 39.85%
	5	gi|146306833|ref|YP_001187298.1|	Pseudomonas mendocina ymp	E-value = 4.00E-83	Identity = 40.00%
AB1129	1	gi|51244367|ref|YP_064251.1|	Desulfotalea psychrophila LSv54	E-value = 4.00E-15	Identity = 27.08%
	2	gi|157164514|ref|YP_001466535.1|	Campylobacter concisus 13826	E-value = 8.00E-15	Identity = 29.56%
	3	gi|154175194|ref|YP_001408651.1|	Campylobacter curvus 525.92	E-value = 1.00E-13	Identity = 28.43%
	4	gi|78484550|ref|YP_390475.1|	Thiomicrospira crunogena XCL-2	E-value = 1.00E-12	Identity = 28.14%
	5	gi|37679701|ref|NP_934310.1|	Vibrio vulnificus YJ016	E-value = 6.00E-08	Identity = 26.26%
AB1130	1	gi|154174597|ref|YP_001408656.1|	Campylobacter curvus 525.92	E-value = 5.00E-42	Identity = 45.69%
	2	gi|157164834|ref|YP_001466530.1|	Campylobacter concisus 13826	E-value = 4.00E-38	Identity = 45.25%
	3	gi|109897405|ref|YP_660660.1|	Pseudoalteromonas atlantica T6c	E-value = 3.00E-36	Identity = 45.56%
	4	gi|89093183|ref|ZP_01166133.1|	Oceanospirillum sp. MED92	E-value = 7.00E-36	Identity = 46.63%
	5	gi|78484554|ref|YP_390479.1|	Thiomicrospira crunogena XCL-2	E-value = 9.00E-36	Identity = 43.43%

AB1131	1		gi|157164294|ref|YP_001466531.1|	Campylobacter concisus 13826	E-value = 8.00E-47	Identity = 26.83%
	2		gi|154175034|ref|YP_001408655.1|	Campylobacter curvus 525.92	E-value = 9.00E-47	Identity = 26.66%
	3		gi|78484553|ref|YP_390478.1|	Thiomicrospira crunogena XCL-2	E-value = 3.00E-46	Identity = 25.51%
	4		gi|152996838|ref|YP_001341673.1|	Marinomonas sp. MWYL1	E-value = 4.00E-44	Identity = 25.19%
	5		gi|51244369|ref|YP_064253.1|	Desulfotalea psychrophila LSv54	E-value = 1.00E-42	Identity = 26.75%
AB1132	1	*aroB*	gi|152992603|ref|YP_001358324.1|	Sulfurovum sp. NBC37-1	E-value = 2.00E-123	Identity = 64.16%
	2		gi|78777218|ref|YP_393533.1|	Sulfuromonas denitrificans ATCC 33889	E-value = 2.00E-123	Identity = 65.32%
	3		gi|152990827|ref|YP_001356549.1|	Nitratiruptor sp. SB155-2	E-value = 6.00E-122	Identity = 61.79%
	4		gi|32266381|ref|NP_860413.1|	Helicobacter hepaticus ATCC 51449	E-value = 7.00E-117	Identity = 62.09%
	5		gi|34557850|ref|NP_907665.1|	Wolinella succinogenes DSM 1740	E-value = 2.00E-115	Identity = 61.19%
AB1133	1		gi|78777219|ref|YP_393534.1|	Sulfuromonas denitrificans ATCC 33889	E-value = 3.00E-115	Identity = 45.28%
	2		gi|154174819|ref|YP_001408234.1|	Campylobacter curvus 525.92	E-value = 9.00E-113	Identity = 44.89%
	3		gi|152992604|ref|YP_001358325.1|	Sulfurovum sp. NBC37-1	E-value = 4.00E-112	Identity = 45.60%
	4		gi|118475324|ref|YP_892030.1|	Campylobacter fetus subsp. fetus 82-40	E-value = 3.00E-109	Identity = 45.19%
	5		gi|157164128|ref|YP_001466754.1|	Campylobacter concisus 13826	E-value = 8.00E-108	Identity = 42.71%
AB1134	1		gi|152990825|ref|YP_001356547.1|	Nitratiruptor sp. SB155-2	E-value = 1.00E-157	Identity = 66.59%
	2		gi|152992605|ref|YP_001358326.1|	Sulfurovum sp. NBC37-1	E-value = 2.00E-156	Identity = 63.29%
	3		gi|78777220|ref|YP_393535.1|	Sulfuromonas denitrificans ATCC 33889	E-value = 2.00E-151	Identity = 60.87%
	4		gi|34557848|ref|NP_907663.1|	Wolinella succinogenes DSM 1740	E-value = 2.00E-140	Identity = 59.47%
	5		gi|154175462|ref|YP_001408235.1|	Campylobacter curvus 525.92	E-value = 2.00E-132	Identity = 55.42%
AB1135	1	*ftsH2*	gi|149193778|ref|ZP_01870876.1|	Caminibacter mediatlanticus TB-2	E-value = 2.00E-98	Identity = 45.88%
	2		gi|32266378|ref|NP_860410.1|	Helicobacter hepaticus ATCC 51449	E-value = 4.00E-98	Identity = 46.83%
	3		gi|157164271|ref|YP_001466756.1|	Campylobacter concisus 13826	E-value = 2.00E-96	Identity = 45.20%
	4		gi|154148871|ref|YP_001406629.1|	Campylobacter hominis ATCC BAA-381	E-value = 2.00E-93	Identity = 48.71%
	5		gi|57242029|ref|ZP_00369969.1|	Campylobacter upsaliensis RM3195	E-value = 1.00E-92	Identity = 44.93%
AB1136	1		gi|152992607|ref|YP_001358328.1|	Sulfurovum sp. NBC37-1	E-value = 1.00E-37	Identity = 50.60%
	2		gi|152990823|ref|YP_001356545.1|	Nitratiruptor sp. SB155-2	E-value = 5.00E-31	Identity = 46.75%
	3		gi|34557846|ref|NP_907661.1|	Wolinella succinogenes DSM 1740	E-value = 5.00E-29	Identity = 43.20%
	4		gi|78777222|ref|YP_393537.1|	Sulfuromonas denitrificans ATCC 33889	E-value = 2.00E-28	Identity = 48.52%
	5		gi|15644915|ref|NP_207085.1|	Helicobacter pylori 26695	E-value = 2.00E-24	Identity = 43.20%
AB1137	1	*mog*	gi|113947990|ref|ZP_01433653.1|	Shewanella baltica OS195	E-value = 2.00E-74	Identity = 81.40%
	2		gi|91795033|ref|YP_564684.1|	Shewanella denitrificans OS217	E-value = 2.00E-74	Identity = 80.81%
	3		gi|24371665|ref|NP_715707.1|	Shewanella oneidensis MR-1	E-value = 2.00E-74	Identity = 81.40%
	4		gi|59713748|ref|YP_206523.1|	Vibrio fischeri ES114	E-value = 6.00E-74	Identity = 80.79%
	5		gi|90414003|ref|ZP_01221987.1|	Photobacterium profundum 3TCK	E-value = 2.00E-73	Identity = 79.19%
AB1138	1	*ribB*	gi|118578754|ref|YP_900004.1|	Pelobacter propionicus DSM 2379	E-value = 3.00E-64	Identity = 55.61%
	2		gi|78356097|ref|YP_387546.1|	Desulfovibrio desulfuricans G20	E-value = 1.00E-62	Identity = 53.23%
	3		gi|95929160|ref|ZP_01311904.1|	Desulfuromonas acetoxidans DSM 684	E-value = 5.00E-62	Identity = 57.00%
	4		gi|146301799|ref|YP_001196390.1|	Flavobacterium johnsoniae UW101	E-value = 6.00E-62	Identity = 57.21%
	5		gi|156977451|ref|YP_001448357.1|	Vibrio harveyi ATCC BAA-1116	E-value = 7.00E-62	Identity = 57.14%
AB1139	1		gi|152992512|ref|YP_001358233.1|	Sulfurovum sp. NBC37-1	E-value = 2.00E-64	Identity = 67.55%
	2		gi|126640101|ref|YP_001083088.1|	Acinetobacter baumannii ATCC 17978	E-value = 2.00E-60	Identity = 57.67%
	3		gi|126659953|ref|ZP_01731076.1|	Cyanothece sp. CCY0110	E-value = 5.00E-55	Identity = 55.56%
	4		gi|39996649|ref|NP_952600.1|	Geobacter sulfurreducens PCA	E-value = 1.00E-54	Identity = 52.38%
	5		gi|89092916|ref|ZP_01165868.1|	Oceanospirillum sp. MED92	E-value = 1.00E-53	Identity = 51.85%
AB1140	1		gi|24380445|ref|NP_722400.1|	Streptococcus mutans UA159	E-value = 4.00E-11	Identity = 34.29%
	2		gi|150016702|ref|YP_001308956.1|	Clostridium beijerinckii NCIMB 8052	E-value = 2.00E-10	Identity = 34.44%
	3		gi|68053910|ref|ZP_00538075.1|	Exiguobacterium sibiricum 255-15	E-value = 2.00E-08	Identity = 28.02%
	4		gi|51894351|ref|YP_077042.1|	Symbiobacterium thermophilum IAM 14863	E-value = 1.00E-07	Identity = 26.16%

	5	gi|15613299|ref|NP_241602.1|	Bacillus halodurans C-125	E-value = 3.00E-06	Identity = 30.56%
AB1141	1	gi|126172472|ref|YP_001048621.1|	Shewanella baltica OS155	E-value = 7.00E-16	Identity = 52.00%
	2	gi|149117081|ref|ZP_01843788.1|	Shewanella baltica OS223	E-value = 2.00E-15	Identity = 52.00%
	3	gi|24375998|ref|NP_720041.1|	Shewanella oneidensis MR-1	E-value = 2.00E-15	Identity = 51.95%
	4	gi|113950814|ref|ZP_01436426.1|	Shewanella baltica OS195	E-value = 4.00E-15	Identity = 50.65%
	5	gi|120597232|ref|YP_961806.1|	Shewanella sp. W3-18-1	E-value = 6.00E-15	Identity = 50.65%
AB1142	1		***** No matches found *****		
AB1143	1	gi|124010463|ref|ZP_01695102.1|	Microscilla marina ATCC 23134	E-value = 1.00E-15	Identity = 36.84%
	2	gi|37520282|ref|NP_923659.1|	Gloeobacter violaceus PCC 7421	E-value = 3.00E-15	Identity = 33.33%
AB1144	1	gi|32266448|ref|NP_860480.1|	Helicobacter hepaticus ATCC 51449	E-value = 1.00E-09	Identity = 56.92%
	2	gi|152994012|ref|YP_001359733.1|	Sulfurovum sp. NBC37-1	E-value = 2.00E-07	Identity = 43.59%
	3	gi|118474627|ref|YP_892564.1|	Campylobacter fetus subsp. fetus 82-40	E-value = 3.00E-07	Identity = 35.90%
	4	gi|57168434|ref|ZP_00367568.1|	Campylobacter coli RM2228	E-value = 5.00E-07	Identity = 42.19%
AB1145	1		***** No matches found *****		
AB1146	1	gi|71738045|ref|YP_273514.1|	Pseudomonas syringae pv. phaseolicola 1448A	E-value = 2.00E-36	Identity = 45.22%
	2	gi|66044431|ref|YP_234272.1|	Pseudomonas syringae pv. syringae B728a	E-value = 3.00E-35	Identity = 44.81%
	3	gi|89092565|ref|ZP_01165518.1|	Oceanospirillum sp. MED92	E-value = 2.00E-34	Identity = 44.03%
	4	gi|94310685|ref|YP_583895.1|	Ralstonia metallidurans CH34	E-value = 5.00E-34	Identity = 46.10%
	5	gi|149114586|ref|ZP_01841338.1|	Shewanella baltica OS223	E-value = 1.00E-33	Identity = 45.10%
AB1147	1	gi|118745541|ref|ZP_01593513.1|	Geobacter lovleyi SZ	E-value = 1.00E-83	Identity = 36.20%
	2	gi|85859546|ref|YP_461747.1|	Syntrophus aciditrophicus SB	E-value = 5.00E-81	Identity = 36.01%
	3	gi|108763409|ref|YP_632166.1|	Myxococcus xanthus DK 1622	E-value = 6.00E-59	Identity = 29.90%
	4	gi|115380096|ref|ZP_01467135.1|	Stigmatella aurantiaca DW4/3-1	E-value = 2.00E-57	Identity = 31.17%
	5	gi|153005614|ref|YP_001379939.1|	Anaeromyxobacter sp. Fw109-5	E-value = 9.00E-47	Identity = 28.75%
AB1149	1	gi|153000086|ref|YP_001365767.1|	Shewanella baltica OS185	E-value = 3.00E-24	Identity = 39.22%
	2	gi|119470649|ref|ZP_01613317.1|	Alteromonadales bacterium TW-7	E-value = 2.00E-23	Identity = 40.79%
	3	gi|126173797|ref|YP_001049946.1|	Shewanella baltica OS155	E-value = 1.00E-22	Identity = 37.91%
	4	gi|113947251|ref|ZP_01432917.1|	Shewanella baltica OS195	E-value = 2.00E-22	Identity = 37.25%
	5	gi|114048098|ref|YP_738648.1|	Shewanella sp. MR-7	E-value = 3.00E-22	Identity = 39.01%
AB1150	1 *torC*	gi|154173943|ref|YP_001408191.1|	Campylobacter curvus 525.92	E-value = 5.00E-37	Identity = 38.07%
	2	gi|157165180|ref|YP_001466955.1|	Campylobacter concisus 13826	E-value = 2.00E-34	Identity = 36.98%
	3	gi|32266450|ref|NP_860482.1|	Helicobacter hepaticus ATCC 51449	E-value = 3.00E-31	Identity = 38.58%
	4	gi|57168334|ref|ZP_00367468.1|	Campylobacter coli RM2228	E-value = 4.00E-31	Identity = 35.60%
	5	gi|154174167|ref|YP_001408194.1|	Campylobacter curvus 525.92	E-value = 6.00E-31	Identity = 36.60%
AB1151	1 *bisC*	gi|154148302|ref|YP_001407047.1|	Campylobacter hominis ATCC BAA-381	E-value = 0	Identity = 63.72%
	2	gi|154174303|ref|YP_001408193.1|	Campylobacter curvus 525.92	E-value = 0	Identity = 61.77%
	3	gi|32266449|ref|NP_860481.1|	Helicobacter hepaticus ATCC 51449	E-value = 0	Identity = 62.73%
	4	gi|57168333|ref|ZP_00367467.1|	Campylobacter coli RM2228	E-value = 0	Identity = 61.98%
	5	gi|88597385|ref|ZP_01100620.1|	Campylobacter jejuni subsp. jejuni 84-25	E-value = 0	Identity = 62.46%
AB1152	1	gi|145235651|ref|XP_001390474.1|	Aspergillus niger	E-value = 2.00E-51	Identity = 38.02%
	2	gi|146338954|ref|YP_001204002.1|	Bradyrhizobium sp. ORS278	E-value = 1.00E-49	Identity = 36.17%
	3	gi|115398624|ref|XP_001214901.1|	Aspergillus terreus NIH2624	E-value = 2.00E-49	Identity = 37.12%
	4	gi|16330234|ref|NP_440962.1|	Synechocystis sp. PCC 6803	E-value = 1.00E-48	Identity = 37.84%
	5	gi|148253712|ref|YP_001238297.1|	Bradyrhizobium sp. BTAi1	E-value = 1.00E-47	Identity = 36.23%
AB1153	1	gi|117923855|ref|YP_864472.1|	Magnetococcus sp. MC-1	E-value = 6.00E-21	Identity = 31.82%
	2	gi|152992524|ref|YP_001358245.1|	Sulfurovum sp. NBC37-1	E-value = 4.00E-18	Identity = 33.33%
	3	gi|74316500|ref|YP_314240.1|	Thiobacillus denitrificans ATCC 25259	E-value = 1.00E-16	Identity = 27.60%
	4	gi|120556316|ref|YP_960667.1|	Marinobacter aquaeolei VT8	E-value = 1.00E-15	Identity = 30.77%
	5	gi|118743704|ref|ZP_01591706.1|	Geobacter lovleyi SZ	E-value = 2.00E-15	Identity = 30.11%

AB1154	1	*mutS2*	gi|152991004|ref|YP_001356726.1|	Nitratiruptor sp. SB155-2	E-value = 0	Identity = 55.68%
	2		gi|34557825|ref|NP_907640.1|	Wolinella succinogenes DSM 1740	E-value = 0	Identity = 50.41%
	3		gi|157165178|ref|YP_001467481.1|	Campylobacter concisus 13826	E-value = 0	Identity = 52.11%
	4		gi|118475577|ref|YP_891800.1|	Campylobacter fetus subsp. fetus 82-40	E-value = 0	Identity = 50.61%
	5		gi|154174500|ref|YP_001407778.1|	Campylobacter curvus 525.92	E-value = 0	Identity = 50.41%
AB1155	1		gi|157165241|ref|YP_001467655.1|	Campylobacter concisus 13826	E-value = 5.00E-144	Identity = 60.97%
	2		gi|154173896|ref|YP_001409039.1|	Campylobacter curvus 525.92	E-value = 5.00E-143	Identity = 60.51%
	3		gi|57505732|ref|ZP_00371658.1|	Campylobacter upsaliensis RM3195	E-value = 1.00E-141	Identity = 58.26%
	4		gi|109948217|ref|YP_665445.1|	Helicobacter acinonychis str. Sheeba	E-value = 3.00E-139	Identity = 57.66%
	5		gi|108563848|ref|YP_628164.1|	Helicobacter pylori HPAG1	E-value = 2.00E-138	Identity = 58.03%
AB1156	1			***** No matches found *****		
AB1157	1		gi|154148646|ref|YP_001406906.1|	Campylobacter hominis ATCC BAA-381	E-value = 2.00E-53	Identity = 26.27%
	2		gi|118474146|ref|YP_891514.1|	Campylobacter fetus subsp. fetus 82-40	E-value = 1.00E-49	Identity = 27.85%
AB1158	1	*dapE*	gi|78777642|ref|YP_393957.1|	Sulfuromonas denitrificans ATCC 33889	E-value = 1.00E-130	Identity = 63.11%
	2		gi|152990981|ref|YP_001356703.1|	Nitratiruptor sp. SB155-2	E-value = 3.00E-126	Identity = 60.38%
	3		gi|149195290|ref|ZP_01872378.1|	Caminibacter mediatlanticus TB-2	E-value = 1.00E-123	Identity = 61.48%
	4		gi|152992085|ref|YP_001357806.1|	Sulfurovum sp. NBC37-1	E-value = 4.00E-121	Identity = 58.47%
	5		gi|57240564|ref|ZP_00368513.1|	Campylobacter lari RM2100	E-value = 4.00E-117	Identity = 55.74%
AB1159	1	*amtB*	gi|152993224|ref|YP_001358945.1|	Sulfurovum sp. NBC37-1	E-value = 7.00E-134	Identity = 69.23%
	2		gi|149195056|ref|ZP_01872148.1|	Caminibacter mediatlanticus TB-2	E-value = 1.00E-127	Identity = 62.09%
	3		gi|152990241|ref|YP_001355963.1|	Nitratiruptor sp. SB155-2	E-value = 4.00E-124	Identity = 61.69%
	4		gi|121542175|ref|ZP_01673911.1|	Candidatus Desulfococcus oleovorans Hxd3	E-value = 1.00E-102	Identity = 49.66%
	5		gi|78776840|ref|YP_393155.1|	Sulfuromonas denitrificans ATCC 33889	E-value = 3.00E-96	Identity = 49.20%
AB1160	1	*glnB2*	gi|152990242|ref|YP_001355964.1|	Nitratiruptor sp. SB155-2	E-value = 5.00E-35	Identity = 75.00%
	2		gi|52424481|ref|YP_087618.1|	Mannheimia succiniciproducens MBEL55E	E-value = 1.00E-34	Identity = 64.29%
	3		gi|42629799|ref|ZP_00155344.1|	Haemophilus influenzae R2846	E-value = 3.00E-34	Identity = 66.07%
	4		gi|152991172|ref|YP_001356894.1|	Nitratiruptor sp. SB155-2	E-value = 4.00E-34	Identity = 73.21%
	5		gi|124871885|gb|EAY63601.1|	Burkholderia cenocepacia PC184	E-value = 5.00E-34	Identity = 66.07%
AB1161	1	*pssA*	gi|78777958|ref|YP_394273.1|	Sulfuromonas denitrificans ATCC 33889	E-value = 5.00E-44	Identity = 55.98%
	2		gi|152993792|ref|YP_001359513.1|	Sulfurovum sp. NBC37-1	E-value = 2.00E-40	Identity = 50.55%
	3		gi|30019131|ref|NP_830762.1|	Bacillus cereus ATCC 14579	E-value = 2.00E-13	Identity = 31.21%
	4		gi|75761825|ref|ZP_00741756.1|	Bacillus thuringiensis serovar israelensis ATCC 35646	E-value = 3.00E-13	Identity = 30.06%
	5		gi|52144363|ref|YP_082466.1|	Bacillus cereus E33L	E-value = 6.00E-13	Identity = 29.48%
AB1162	1		gi|152993409|ref|YP_001359130.1|	Sulfurovum sp. NBC37-1	E-value = 2.00E-50	Identity = 39.66%
	2		gi|149193841|ref|ZP_01870939.1|	Caminibacter mediatlanticus TB-2	E-value = 8.00E-49	Identity = 40.68%
	3		gi|34556556|ref|NP_906371.1|	Wolinella succinogenes DSM 1740	E-value = 1.00E-39	Identity = 37.21%
	4		gi|152990556|ref|YP_001356278.1|	Nitratiruptor sp. SB155-2	E-value = 4.00E-38	Identity = 36.18%
	5		gi|154175174|ref|YP_001408343.1|	Campylobacter curvus 525.92	E-value = 3.00E-32	Identity = 33.33%
AB1163	1			***** No matches found *****		
AB1164	1	*ilvI*	gi|152993406|ref|YP_001359127.1|	Sulfurovum sp. NBC37-1	E-value = 0	Identity = 67.32%
	2		gi|78777339|ref|YP_393654.1|	Sulfuromonas denitrificans ATCC 33889	E-value = 0	Identity = 66.37%
	3		gi|152990557|ref|YP_001356279.1|	Nitratiruptor sp. SB155-2	E-value = 0	Identity = 66.61%
	4		gi|32266363|ref|NP_860395.1|	Helicobacter hepaticus ATCC 51449	E-value = 0	Identity = 63.59%
	5		gi|157163882|ref|YP_001466735.1|	Campylobacter concisus 13826	E-value = 0	Identity = 63.41%
AB1165	1	*ilvH*	gi|152990558|ref|YP_001356280.1|	Nitratiruptor sp. SB155-2	E-value = 6.00E-42	Identity = 56.58%
	2		gi|34556554|ref|NP_906369.1|	Wolinella succinogenes DSM 1740	E-value = 1.00E-40	Identity = 52.29%
	3		gi|118475062|ref|YP_891992.1|	Campylobacter fetus subsp. fetus 82-40	E-value = 2.00E-40	Identity = 58.94%
	4		gi|149193839|ref|ZP_01870937.1|	Caminibacter mediatlanticus TB-2	E-value = 2.00E-39	Identity = 56.58%
	5		gi|78777338|ref|YP_393653.1|	Sulfuromonas denitrificans ATCC 33889	E-value = 2.00E-38	Identity = 55.48%

AB1166	1	*lpxD*	gi|152990559|ref|YP_001356281.1|	Nitratiruptor sp. SB155-2	E-value = 1.00E-97	Identity = 57.73%
	2		gi|78777336|ref|YP_393651.1|	Sulfuromonas denitrificans ATCC 33889	E-value = 4.00E-90	Identity = 55.06%
	3		gi|149193838|ref|ZP_01870936.1|	Caminibacter mediatlanticus TB-2	E-value = 8.00E-90	Identity = 57.01%
	4		gi|152993404|ref|YP_001359125.1|	Sulfurovum sp. NBC37-1	E-value = 8.00E-87	Identity = 52.87%
	5		gi|34556553|ref|NP_906368.1|	Wolinella succinogenes DSM 1740	E-value = 1.00E-81	Identity = 49.05%
AB1167	1	*ftsK*	gi|152990573|ref|YP_001356295.1|	Nitratiruptor sp. SB155-2	E-value = 0	Identity = 59.3%
	2		gi|154175516|ref|YP_001408168.1|	Campylobacter curvus 525.92	E-value = 0	Identity = 61.3%
	3		gi|118474833|ref|YP_891859.1|	Campylobacter fetus subsp. fetus 82-40	E-value = 0	Identity = 58.89%
	4		gi|78777236|ref|YP_393551.1|	Sulfuromonas denitrificans ATCC 33889	E-value = 0	Identity = 58%
	5		gi|149194323|ref|ZP_01871420.1|	Caminibacter mediatlanticus TB-2	E-value = 0	Identity = 61.78%
AB1168	1		gi|118474546|ref|YP_891938.1|	Campylobacter fetus subsp. fetus 82-40	E-value = 1.00E-57	Identity = 55.56%
	2		gi|154149077|ref|YP_001406703.1|	Campylobacter hominis ATCC BAA-381	E-value = 1.00E-57	Identity = 52.65%
	3		gi|154174499|ref|YP_001407800.1|	Campylobacter curvus 525.92	E-value = 1.00E-55	Identity = 52.65%
	4		gi|157165656|ref|YP_001466290.1|	Campylobacter concisus 13826	E-value = 2.00E-55	Identity = 52.65%
	5		gi|154174385|ref|YP_001408399.1|	Campylobacter curvus 525.92	E-value = 2.00E-55	Identity = 52.65%
AB1169	1		gi|157163989|ref|YP_001466291.1|	Campylobacter concisus 13826	E-value = 5.00E-44	Identity = 37.26%
	2		gi|121612247|ref|YP_001000938.1|	Campylobacter jejuni subsp. jejuni 81-176	E-value = 4.00E-41	Identity = 34.55%
	3		gi|153951530|ref|YP_001397648.1|	Campylobacter jejuni subsp. doylei 269.97	E-value = 8.00E-41	Identity = 34.88%
	4		gi|89096578|ref|ZP_01169470.1|	Bacillus sp. NRRL B-14911	E-value = 1.00E-11	Identity = 25.75%
	5		gi|118757043|ref|ZP_01604810.1|	Shewanella pealeana ATCC 700345	E-value = 1.00E-11	Identity = 25.65%
AB1170	1			***** No matches found *****		
AB1171	1		gi|152990263|ref|YP_001355985.1|	Nitratiruptor sp. SB155-2	E-value = 1.00E-79	Identity = 33.77%
	2		gi|152993616|ref|YP_001359337.1|	Sulfurovum sp. NBC37-1	E-value = 2.00E-70	Identity = 31.22%
	3		gi|95930805|ref|ZP_01313537.1|	Desulfuromonas acetoxidans DSM 684	E-value = 5.00E-42	Identity = 26.79%
AB1172	1		gi|152990261|ref|YP_001355983.1|	Nitratiruptor sp. SB155-2	E-value = 2.00E-32	Identity = 38.01%
	2		gi|152993617|ref|YP_001359338.1|	Sulfurovum sp. NBC37-1	E-value = 1.00E-31	Identity = 38.22%
	3		gi|78776815|ref|YP_393130.1|	Sulfuromonas denitrificans ATCC 33889	E-value = 1.00E-29	Identity = 36.18%
	4		gi|95930806|ref|ZP_01313538.1|	Desulfuromonas acetoxidans DSM 684	E-value = 2.00E-29	Identity = 34.51%
	5		gi|34558244|ref|NP_908059.1|	Wolinella succinogenes DSM 1740	E-value = 5.00E-24	Identity = 31.25%
AB1173	1		gi|153873017|ref|ZP_02001742.1|	Beggiatoa sp. PS	E-value = 6.00E-14	Identity = 27.75%
AB1174	1		gi|152990261|ref|YP_001355983.1|	Nitratiruptor sp. SB155-2	E-value = 4.00E-34	Identity = 36.70%
	2		gi|34558244|ref|NP_908059.1|	Wolinella succinogenes DSM 1740	E-value = 6.00E-30	Identity = 35.56%
	3		gi|152993617|ref|YP_001359338.1|	Sulfurovum sp. NBC37-1	E-value = 5.00E-27	Identity = 36.16%
	4		gi|78776815|ref|YP_393130.1|	Sulfuromonas denitrificans ATCC 33889	E-value = 1.00E-23	Identity = 28.63%
	5		gi|95930806|ref|ZP_01313538.1|	Desulfuromonas acetoxidans DSM 684	E-value = 2.00E-18	Identity = 27.88%
AB1175	1			***** No matches found *****		
AB1176	1			***** No matches found *****		
AB1177	1			***** No matches found *****		
AB1178	1		gi|149193704|ref|ZP_01870802.1|	Caminibacter mediatlanticus TB-2	E-value = 4.00E-21	Identity = 51.49%
	2		gi|152990602|ref|YP_001356324.1|	Nitratiruptor sp. SB155-2	E-value = 1.00E-17	Identity = 43.48%
	3		gi|51893022|ref|YP_075713.1|	Symbiobacterium thermophilum IAM 14863	E-value = 2.00E-16	Identity = 35.25%
	4		gi|108760292|ref|YP_634638.1|	Myxococcus xanthus DK 1622	E-value = 3.00E-16	Identity = 33.33%
	5		gi|153835180|ref|ZP_01987847.1|	Vibrio harveyi HY01	E-value = 8.00E-14	Identity = 33.82%
AB1179	1			***** No matches found *****		
AB1180	1		gi|19704777|ref|NP_604339.1|	Fusobacterium nucleatum subsp. nucleatum ATCC 25586	E-value = 4.00E-172	Identity = 45.58%
	2		gi|156863268|gb|EDO56699.1|	Clostridium sp. L2-50	E-value = 4.00E-171	Identity = 40.83%
	3		gi|34557480|ref|NP_907295.1|	Wolinella succinogenes DSM 1740	E-value = 1.00E-163	Identity = 47.13%
	4		gi|119356141|ref|YP_910785.1|	Chlorobium phaeobacteroides DSM 266	E-value = 8.00E-160	Identity = 36.58%
	5		gi|67919198|ref|ZP_00512783.1|	Chlorobium limicola DSM 245	E-value = 4.00E-157	Identity = 36.69%

AB1181	1			***** No matches found *****		
AB1182	1		gi|118475196|ref|YP_892233.1|	Campylobacter fetus subsp. fetus 82-40	E-value = 2.00E-41	Identity = 57.78%
	2		gi|154175401|ref|YP_001407984.1|	Campylobacter curvus 525.92	E-value = 2.00E-39	Identity = 53.33%
	3		gi|57240447|ref|ZP_00368396.1|	Campylobacter lari RM2100	E-value = 5.00E-39	Identity = 55.56%
	4		gi|34558446|ref|NP_908261.1|	Wolinella succinogenes DSM 1740	E-value = 9.00E-38	Identity = 49.44%
	5		gi|157165597|ref|YP_001467194.1|	Campylobacter concisus 13826	E-value = 1.00E-36	Identity = 51.67%
AB1183	1	*engA*	gi|78776593|ref|YP_392908.1|	Sulfuromonas denitrificans ATCC 33889	E-value = 7.00E-139	Identity = 55.71%
	2		gi|34558325|ref|NP_908140.1|	Wolinella succinogenes DSM 1740	E-value = 3.00E-131	Identity = 54.79%
	3		gi|152991112|ref|YP_001356834.1|	Nitratiruptor sp. SB155-2	E-value = 1.00E-130	Identity = 54.98%
	4		gi|118474391|ref|YP_892485.1|	Campylobacter fetus subsp. fetus 82-40	E-value = 2.00E-127	Identity = 52.51%
	5		gi|86151224|ref|ZP_01069439.1|	Campylobacter jejuni subsp. jejuni 260.94	E-value = 3.00E-127	Identity = 51.99%
AB1184	1		gi|109692194|gb|ABG37985.1|	Alkalimonas amylolytica	E-value = 9.00E-103	Identity = 47.25%
	2		gi|88704869|ref|ZP_01102582.1|	gamma proteobacterium KT 71	E-value = 8.00E-100	Identity = 44.47%
	3		gi|16331770|ref|NP_442498.1|	Synechocystis sp. PCC 6803	E-value = 2.00E-99	Identity = 43.60%
	4		gi|119489620|ref|ZP_01622380.1|	Lyngbya sp. PCC 8106	E-value = 9.00E-98	Identity = 44.91%
	5		gi|78778602|ref|YP_396714.1|	Prochlorococcus marinus str. MIT 9312	E-value = 3.00E-97	Identity = 43.54%
AB1185	1	*hemD*	gi|152991081|ref|YP_001356803.1|	Nitratiruptor sp. SB155-2	E-value = 5.00E-38	Identity = 39.90%
	2		gi|34557923|ref|NP_907738.1|	Wolinella succinogenes DSM 1740	E-value = 8.00E-31	Identity = 34.12%
	3		gi|118474698|ref|YP_892418.1|	Campylobacter fetus subsp. fetus 82-40	E-value = 1.00E-29	Identity = 41.26%
	4		gi|149194573|ref|ZP_01871669.1|	Caminibacter mediatlanticus TB-2	E-value = 7.00E-29	Identity = 41.63%
	5		gi|154174614|ref|YP_001407816.1|	Campylobacter curvus 525.92	E-value = 2.00E-28	Identity = 38.03%
AB1186	1			***** No matches found *****		
AB1187	1	*purD*	gi|78776625|ref|YP_392940.1|	Sulfuromonas denitrificans ATCC 33889	E-value = 2.00E-168	Identity = 68.88%
	2		gi|152991080|ref|YP_001356802.1|	Nitratiruptor sp. SB155-2	E-value = 9.00E-163	Identity = 67.30%
	3		gi|152993474|ref|YP_001359195.1|	Sulfurovum sp. NBC37-1	E-value = 9.00E-162	Identity = 67.54%
	4		gi|149193799|ref|ZP_01870897.1|	Caminibacter mediatlanticus TB-2	E-value = 2.00E-157	Identity = 66.83%
	5		gi|157163965|ref|YP_001467332.1|	Campylobacter concisus 13826	E-value = 1.00E-144	Identity = 62.86%
AB1188	1		gi|118474986|ref|YP_892416.1|	Campylobacter fetus subsp. fetus 82-40	E-value = 1.00E-23	Identity = 41.55%
	2		gi|154174671|ref|YP_001407818.1|	Campylobacter curvus 525.92	E-value = 1.00E-22	Identity = 42.75%
	3		gi|78776626|ref|YP_392941.1|	Sulfuromonas denitrificans ATCC 33889	E-value = 1.00E-17	Identity = 36.23%
	4		gi|34557285|ref|NP_907100.1|	Wolinella succinogenes DSM 1740	E-value = 4.00E-17	Identity = 35.51%
	5		gi|57168153|ref|ZP_00367292.1|	Campylobacter coli RM2228	E-value = 7.00E-16	Identity = 34.06%
AB1189	1		gi|154147997|ref|YP_001405841.1|	Campylobacter hominis ATCC BAA-381	E-value = 2.00E-85	Identity = 31.95%
	2		gi|154175010|ref|YP_001407819.1|	Campylobacter curvus 525.92	E-value = 4.00E-82	Identity = 31.93%
	3		gi|118474176|ref|YP_892415.1|	Campylobacter fetus subsp. fetus 82-40	E-value = 5.00E-81	Identity = 30.28%
	4		gi|121612176|ref|YP_001000928.1|	Campylobacter jejuni subsp. jejuni 81-176	E-value = 4.00E-80	Identity = 32.31%
	5		gi|57168154|ref|ZP_00367293.1|	Campylobacter coli RM2228	E-value = 7.00E-80	Identity = 32.50%
AB1190	1		gi|34557288|ref|NP_907103.1|	Wolinella succinogenes DSM 1740	E-value = 1.00E-41	Identity = 40.74%
	2		gi|118475165|ref|YP_892414.1|	Campylobacter fetus subsp. fetus 82-40	E-value = 1.00E-39	Identity = 41.78%
	3		gi|78776628|ref|YP_392943.1|	Sulfuromonas denitrificans ATCC 33889	E-value = 7.00E-39	Identity = 39.91%
	4		gi|154175508|ref|YP_001407820.1|	Campylobacter curvus 525.92	E-value = 7.00E-34	Identity = 38.76%
	5		gi|152993470|ref|YP_001359191.1|	Sulfurovum sp. NBC37-1	E-value = 1.00E-33	Identity = 35.38%
AB1191	1	*pnp*	gi|34557289|ref|NP_907104.1|	Wolinella succinogenes DSM 1740	E-value = 0	Identity = 56.77%
	2		gi|152991076|ref|YP_001356798.1|	Nitratiruptor sp. SB155-2	E-value = 0	Identity = 57.28%
	3		gi|154174457|ref|YP_001407821.1|	Campylobacter curvus 525.92	E-value = 0	Identity = 55.03%
	4		gi|157164996|ref|YP_001467328.1|	Campylobacter concisus 13826	E-value = 0	Identity = 54.84%
	5		gi|15792577|ref|NP_282400.1|	Campylobacter jejuni subsp. jejuni NCTC 11168	E-value = 0	Identity = 55.54%
AB1192	1	*cheY1*	gi|121998947|ref|YP_001003734.1|	Halorhodospira halophila SL1	E-value = 1.00E-30	Identity = 60.00%
	2		gi|152982726|ref|YP_001353786.1|	Janthinobacterium sp. Marseille	E-value = 2.00E-29	Identity = 61.54%

	3		gi|149908621|ref|ZP_01897283.1|	Moritella sp. PE36	E-value = 4.00E-29	Identity = 58.33%
	4		gi|89094274|ref|ZP_01167216.1|	Oceanospirillum sp. MED92	E-value = 5.00E-29	Identity = 55.00%
	5		gi|134094525|ref|YP_001099600.1|	Herminiimonas arsenicoxydans	E-value = 1.00E-28	Identity = 59.83%
AB1193	1	*cheA*	gi|37525780|ref|NP_929124.1|	Photorhabdus luminescens subsp. laumondii TTO1	E-value = 1.00E-104	Identity = 35.38%
	2		gi|78485953|ref|YP_391878.1|	Thiomicrospira crunogena XCL-2	E-value = 6.00E-104	Identity = 36.30%
	3		gi|145529|gb|AAA23573.1|	Escherichia coli	E-value = 2.00E-103	Identity = 35.21%
	4		gi|16129840|ref|NP_416402.1|	Escherichia coli K12	E-value = 3.00E-103	Identity = 35.21%
	5		gi|157161360|ref|YP_001458678.1|	Escherichia coli HS	E-value = 5.00E-103	Identity = 35.21%
AB1194	1	*cheR*	gi|34557579|ref|NP_907394.1|	Wolinella succinogenes DSM 1740	E-value = 2.00E-68	Identity = 49.82%
	2		gi|32265954|ref|NP_859986.1|	Helicobacter hepaticus ATCC 51449	E-value = 2.00E-54	Identity = 48.13%
	3		gi|91776284|ref|YP_546040.1|	Methylobacillus flagellatus KT	E-value = 7.00E-41	Identity = 40.24%
	4		gi|118054234|ref|ZP_01522775.1|	Comamonas testosteroni KF-1	E-value = 3.00E-39	Identity = 36.82%
	5		gi|34498892|ref|NP_903107.1|	Chromobacterium violaceum ATCC 12472	E-value = 5.00E-39	Identity = 38.25%
AB1195	1	*cheD*	gi|118054235|ref|ZP_01522776.1|	Comamonas testosteroni KF-1	E-value = 2.00E-20	Identity = 35.26%
	2		gi|121596074|ref|YP_987970.1|	Acidovorax sp. JS42	E-value = 2.00E-18	Identity = 32.63%
	3		gi|110602187|ref|ZP_01390322.1|	Geobacter sp. FRC-32	E-value = 1.00E-17	Identity = 31.61%
	4		gi|121609132|ref|YP_996939.1|	Verminephrobacter eiseniae EF01-2	E-value = 3.00E-17	Identity = 29.85%
	5		gi|21242633|ref|NP_642215.1|	Xanthomonas axonopodis pv. citri str. 306	E-value = 3.00E-17	Identity = 31.82%
AB1196	1	*cheB*	gi|78485103|ref|YP_391028.1|	Thiomicrospira crunogena XCL-2	E-value = 1.00E-72	Identity = 42.74%
	2		gi|127511043|ref|YP_001092240.1|	Shewanella loihica PV-4	E-value = 2.00E-71	Identity = 44.00%
	3		gi|50120627|ref|YP_049794.1|	Erwinia carotovora subsp. atroseptica SCRI1043	E-value = 7.00E-71	Identity = 42.09%
	4		gi|157371220|ref|YP_001479209.1|	Serratia proteamaculans 568	E-value = 8.00E-71	Identity = 40.91%
	5		gi|118072309|ref|ZP_01540499.1|	Shewanella woodyi ATCC 51908	E-value = 7.00E-70	Identity = 41.50%
AB1197	1		gi|46580049|ref|YP_010857.1|	Desulfovibrio vulgaris subsp. vulgaris str. Hildenborough	E-value = 6.00E-14	Identity = 28.83%
	2		gi|120602541|ref|YP_966941.1|	Desulfovibrio vulgaris subsp. vulgaris DP4	E-value = 7.00E-14	Identity = 28.22%
	3		gi|119944262|ref|YP_941942.1|	Psychromonas ingrahamii 37	E-value = 3.00E-12	Identity = 29.09%
AB1198	1	*fsr*	gi|83718758|ref|YP_443145.1|	Burkholderia thailandensis E264	E-value = 2.00E-101	Identity = 52.53%
	2		gi|124384439|ref|YP_001026418.1|	Burkholderia mallei NCTC 10229	E-value = 5.00E-101	Identity = 52.53%
	3		gi|76808590|ref|YP_333250.1|	Burkholderia pseudomallei 1710b	E-value = 9.00E-101	Identity = 52.27%
	4		gi|53719589|ref|YP_108575.1|	Burkholderia pseudomallei K96243	E-value = 9.00E-101	Identity = 52.27%
	5		gi|37527689|ref|NP_931033.1|	Photorhabdus luminescens subsp. laumondii TTO1	E-value = 8.00E-98	Identity = 53.25%
AB1199	1	*xerD*	gi|34557587|ref|NP_907402.1|	Wolinella succinogenes DSM 1740	E-value = 3.00E-125	Identity = 64.77%
	2		gi|152990726|ref|YP_001356448.1|	Nitratiruptor sp. SB155-2	E-value = 4.00E-117	Identity = 62.75%
	3		gi|32267344|ref|NP_861376.1|	Helicobacter hepaticus ATCC 51449	E-value = 4.00E-107	Identity = 59.49%
	4		gi|118474094|ref|YP_892018.1|	Campylobacter fetus subsp. fetus 82-40	E-value = 4.00E-102	Identity = 57.59%
	5		gi|154175082|ref|YP_001408312.1|	Campylobacter curvus 525.92	E-value = 9.00E-100	Identity = 56.73%
AB1200	1		gi|119477635|ref|ZP_01617785.1|	marine gamma proteobacterium HTCC2143	E-value = 1.00E-16	Identity = 35.96%
	2		gi|94501283|ref|ZP_01307804.1|	Oceanobacter sp. RED65	E-value = 2.00E-15	Identity = 35.96%
	3		gi|89093277|ref|ZP_01166227.1|	Oceanospirillum sp. MED92	E-value = 3.00E-14	Identity = 38.10%
	4		gi|78778026|ref|YP_394341.1|	Sulfuromonas denitrificans ATCC 33889	E-value = 1.00E-13	Identity = 39.09%
	5		gi|110833113|ref|YP_691972.1|	Alcanivorax borkumensis SK2	E-value = 2.00E-11	Identity = 29.41%
AB1201	1			***** No matches found *****		
AB1202	1			***** No matches found *****		
AB1203	1	*prlC*	gi|152993269|ref|YP_001358990.1|	Sulfurovum sp. NBC37-1	E-value = 0	Identity = 52.62%
	2		gi|78776911|ref|YP_393226.1|	Sulfuromonas denitrificans ATCC 33889	E-value = 0	Identity = 55.31%
	3		gi|152990282|ref|YP_001356004.1|	Nitratiruptor sp. SB155-2	E-value = 0	Identity = 51.67%
	4		gi|116331236|ref|YP_800954.1|	Leptospira borgpetersenii serovar Hardjo-bovis JB197	E-value = 3.00E-174	Identity = 50.08%
	5		gi|116328508|ref|YP_798228.1|	Leptospira borgpetersenii serovar Hardjo-bovis L550	E-value = 8.00E-174	Identity = 50.08%
AB1204	1	*hemK*	gi|78777517|ref|YP_393832.1|	Sulfuromonas denitrificans ATCC 33889	E-value = 7.00E-58	Identity = 43.38%

	2		gi|154173807|ref|YP_001408445.1|	Campylobacter curvus 525.92	E-value = 2.00E-53	Identity = 45.79%
	3		gi|34556665|ref|NP_906480.1|	Wolinella succinogenes DSM 1740	E-value = 5.00E-52	Identity = 41.52%
	4		gi|152992202|ref|YP_001357923.1|	Sulfurovum sp. NBC37-1	E-value = 9.00E-49	Identity = 42.12%
	5		gi|109947073|ref|YP_664301.1|	Helicobacter acinonychis str. Sheeba	E-value = 5.00E-45	Identity = 44.76%
AB1205	1	*hemN1*	gi|78777851|ref|YP_394166.1|	Sulfuromonas denitrificans ATCC 33889	E-value = 1.00E-92	Identity = 52.38%
	2		gi|152992050|ref|YP_001357771.1|	Sulfurovum sp. NBC37-1	E-value = 3.00E-88	Identity = 47.47%
	3		gi|118475057|ref|YP_892276.1|	Campylobacter fetus subsp. fetus 82-40	E-value = 4.00E-82	Identity = 49.01%
	4		gi|152990886|ref|YP_001356608.1|	Nitratiruptor sp. SB155-2	E-value = 5.00E-82	Identity = 48.18%
	5		gi|154148889|ref|YP_001406558.1|	Campylobacter hominis ATCC BAA-381	E-value = 2.00E-80	Identity = 48.60%
AB1206	1	*nudH*	gi|153952382|ref|YP_001398179.1|	Campylobacter jejuni subsp. doylei 269.97	E-value = 3.00E-51	Identity = 66.03%
	2		gi|15791941|ref|NP_281764.1|	Campylobacter jejuni subsp. jejuni NCTC 11168	E-value = 3.00E-51	Identity = 66.03%
	3		gi|57238223|ref|YP_178696.1|	Campylobacter jejuni subsp. jejuni RM1221	E-value = 6.00E-51	Identity = 66.03%
	4		gi|152990885|ref|YP_001356607.1|	Nitratiruptor sp. SB155-2	E-value = 9.00E-50	Identity = 69.28%
	5		gi|57168183|ref|ZP_00367322.1|	Campylobacter coli RM2228	E-value = 3.00E-48	Identity = 61.54%
AB1207	1	*lysC*	gi|78777847|ref|YP_394162.1|	Sulfuromonas denitrificans ATCC 33889	E-value = 6.00E-147	Identity = 72.10%
	2		gi|152990884|ref|YP_001356606.1|	Nitratiruptor sp. SB155-2	E-value = 1.00E-143	Identity = 69.73%
	3		gi|149194491|ref|ZP_01871587.1|	Caminibacter mediatlanticus TB-2	E-value = 2.00E-140	Identity = 68.24%
	4		gi|34558039|ref|NP_907854.1|	Wolinella succinogenes DSM 1740	E-value = 1.00E-136	Identity = 67.99%
	5		gi|152992052|ref|YP_001357773.1|	Sulfurovum sp. NBC37-1	E-value = 4.00E-133	Identity = 67.49%
AB1208	1		gi|34558040|ref|NP_907855.1|	Wolinella succinogenes DSM 1740	E-value = 7.00E-43	Identity = 47.25%
	2		gi|157164563|ref|YP_001467150.1|	Campylobacter concisus 13826	E-value = 2.00E-41	Identity = 47.49%
	3		gi|154174018|ref|YP_001408012.1|	Campylobacter curvus 525.92	E-value = 2.00E-40	Identity = 46.37%
	4		gi|152992053|ref|YP_001357774.1|	Sulfurovum sp. NBC37-1	E-value = 6.00E-37	Identity = 41.99%
	5		gi|118474073|ref|YP_892273.1|	Campylobacter fetus subsp. fetus 82-40	E-value = 1.00E-36	Identity = 41.99%
AB1209	1	*holB*	gi|152990882|ref|YP_001356604.1|	Nitratiruptor sp. SB155-2	E-value = 2.00E-24	Identity = 45.29%
	2		gi|78777845|ref|YP_394160.1|	Sulfuromonas denitrificans ATCC 33889	E-value = 6.00E-23	Identity = 40.56%
	3		gi|57241141|ref|ZP_00369088.1|	Campylobacter lari RM2100	E-value = 1.00E-22	Identity = 39.63%
	4		gi|157164770|ref|YP_001467149.1|	Campylobacter concisus 13826	E-value = 5.00E-22	Identity = 43.37%
	5		gi|57168180|ref|ZP_00367319.1|	Campylobacter coli RM2228	E-value = 2.00E-21	Identity = 39.30%
AB1210	1	*folP*	gi|152992055|ref|YP_001357776.1|	Sulfurovum sp. NBC37-1	E-value = 9.00E-122	Identity = 57.94%
	2		gi|149194494|ref|ZP_01871590.1|	Caminibacter mediatlanticus TB-2	E-value = 4.00E-120	Identity = 58.96%
	3		gi|78777844|ref|YP_394159.1|	Sulfuromonas denitrificans ATCC 33889	E-value = 8.00E-116	Identity = 57.68%
	4		gi|152990881|ref|YP_001356603.1|	Nitratiruptor sp. SB155-2	E-value = 1.00E-114	Identity = 56.91%
	5		gi|34558042|ref|NP_907857.1|	Wolinella succinogenes DSM 1740	E-value = 3.00E-98	Identity = 48.94%
AB1211	1		gi|88704979|ref|ZP_01102691.1|	gamma proteobacterium KT 71	E-value = 8.00E-39	Identity = 44.38%
	2		gi|120556794|ref|YP_961145.1|	Marinobacter aquaeolei VT8	E-value = 6.00E-38	Identity = 41.42%
	3		gi|119504628|ref|ZP_01626707.1|	marine gamma proteobacterium HTCC2080	E-value = 1.00E-35	Identity = 42.24%
	4		gi|90022480|ref|YP_528307.1|	Saccharophagus degradans 2-40	E-value = 1.00E-35	Identity = 41.57%
	5		gi|149375649|ref|ZP_01893418.1|	Marinobacter algicola DG893	E-value = 3.00E-32	Identity = 40.74%
AB1212	1	*cfa*	gi|90577748|ref|ZP_01233559.1|	Vibrio angustum S14	E-value = 2.00E-89	Identity = 43.32%
	2		gi|37679521|ref|NP_934130.1|	Vibrio vulnificus YJ016	E-value = 2.00E-89	Identity = 44.92%
	3		gi|89073737|ref|ZP_01160251.1|	Photobacterium sp. SKA34	E-value = 5.00E-89	Identity = 43.05%
	4		gi|27366209|ref|NP_761737.1|	Vibrio vulnificus CMCP6	E-value = 9.00E-89	Identity = 44.63%
	5		gi|146306100|ref|YP_001186565.1|	Pseudomonas mendocina ymp	E-value = 3.00E-88	Identity = 42.29%
AB1213	1		gi|89094237|ref|ZP_01167179.1|	Oceanospirillum sp. MED92	E-value = 2.00E-21	Identity = 30.86%
	2		gi|71083374|ref|YP_266093.1|	Candidatus Pelagibacter ubique HTCC1062	E-value = 1.00E-20	Identity = 32.79%
	3		gi|113934535|ref|ZP_01420435.1|	Caulobacter sp. K31	E-value = 3.00E-20	Identity = 28.27%
	4		gi|91762202|ref|ZP_01264167.1|	Candidatus Pelagibacter ubique HTCC1002	E-value = 5.00E-20	Identity = 33.05%
	5		gi|154252319|ref|YP_001413143.1|	Parvibaculum lavamentivorans DS-1	E-value = 6.00E-20	Identity = 26.86%

AB1214	1		gi|149374752|ref|ZP_01892525.1|	Marinobacter algicola DG893	E-value = 8.00E-92	Identity = 43.03%
	2		gi|89094236|ref|ZP_01167178.1|	Oceanospirillum sp. MED92	E-value = 1.00E-91	Identity = 43.60%
	3		gi|77460960|ref|YP_350467.1|	Pseudomonas fluorescens PfO-1	E-value = 1.00E-91	Identity = 40.68%
	4		gi|28868333|ref|NP_790952.1|	Pseudomonas syringae pv. tomato str. DC3000	E-value = 2.00E-91	Identity = 40.68%
	5		gi|66044211|ref|YP_234052.1|	Pseudomonas syringae pv. syringae B728a	E-value = 4.00E-91	Identity = 41.79%
AB1215	1		gi|134096563|ref|YP_001101638.1|	Herminiimonas arsenicoxydans	E-value = 1.00E-16	Identity = 36.14%
	2		gi|107104203|ref|ZP_01368121.1|	Pseudomonas aeruginosa PACS2	E-value = 2.00E-15	Identity = 32.39%
	3		gi|77919624|ref|YP_357439.1|	Pelobacter carbinolicus DSM 2380	E-value = 3.00E-15	Identity = 33.57%
	4		gi|146305548|ref|YP_001186013.1|	Pseudomonas mendocina ymp	E-value = 4.00E-15	Identity = 31.54%
	5		gi|15600300|ref|NP_253794.1|	Pseudomonas aeruginosa PAO1	E-value = 5.00E-15	Identity = 32.39%
AB1216	1		gi|83592065|ref|YP_425817.1|	Rhodospirillum rubrum ATCC 11170	E-value = 2.00E-47	Identity = 30.56%
	2		gi|71906134|ref|YP_283721.1|	Dechloromonas aromatica RCB	E-value = 9.00E-47	Identity = 34.25%
	3		gi|121604359|ref|YP_981688.1|	Polaromonas naphthalenivorans CJ2	E-value = 8.00E-39	Identity = 30.52%
	4		gi|148260617|ref|YP_001234744.1|	Acidiphilium cryptum JF-5	E-value = 1.00E-37	Identity = 27.00%
	5		gi|115423157|emb|CAJ49688.1|	Bordetella avium 197N	E-value = 4.00E-37	Identity = 26.60%
AB1217	1		gi|145590153|ref|YP_001156750.1|	Polynucleobacter sp. QLW-P1DMWA-1	E-value = 1.00E-36	Identity = 37.60%
	2		gi|91762199|ref|ZP_01264164.1|	Candidatus Pelagibacter ubique HTCC1002	E-value = 6.00E-36	Identity = 34.80%
	3		gi|71083377|ref|YP_266096.1|	Candidatus Pelagibacter ubique HTCC1062	E-value = 6.00E-36	Identity = 34.80%
	4		gi|118589051|ref|ZP_01546458.1|	Stappia aggregata IAM 12614	E-value = 5.00E-34	Identity = 37.30%
	5		gi|116251744|ref|YP_767582.1|	Rhizobium leguminosarum bv. viciae 3841	E-value = 9.00E-34	Identity = 37.90%
AB1218	1	*tyrA*	gi|152990155|ref|YP_001355877.1|	Nitratiruptor sp. SB155-2	E-value = 2.00E-87	Identity = 60.14%
	2		gi|78776858|ref|YP_393173.1|	Sulfuromonas denitrificans ATCC 33889	E-value = 2.00E-76	Identity = 53.99%
	3		gi|152993577|ref|YP_001359298.1|	Sulfurovum sp. NBC37-1	E-value = 1.00E-75	Identity = 54.17%
	4		gi|149194900|ref|ZP_01871994.1|	Caminibacter mediatlanticus TB-2	E-value = 2.00E-75	Identity = 51.08%
	5		gi|34556783|ref|NP_906598.1|	Wolinella succinogenes DSM 1740	E-value = 4.00E-69	Identity = 52.12%
AB1219	1		gi|152990156|ref|YP_001355878.1|	Nitratiruptor sp. SB155-2	E-value = 2.00E-174	Identity = 45.01%
	2		gi|34556784|ref|NP_906599.1|	Wolinella succinogenes DSM 1740	E-value = 9.00E-167	Identity = 42.67%
	3		gi|32266491|ref|NP_860523.1|	Helicobacter hepaticus ATCC 51449	E-value = 9.00E-149	Identity = 39.66%
	4		gi|57168700|ref|ZP_00367832.1|	Campylobacter coli RM2228	E-value = 5.00E-141	Identity = 38.74%
	5		gi|121612878|ref|YP_999853.1|	Campylobacter jejuni subsp. jejuni 81-176	E-value = 6.00E-141	Identity = 38.78%
AB1220	1		gi|118474940|ref|YP_891976.1|	Campylobacter fetus subsp. fetus 82-40	E-value = 8.00E-159	Identity = 77.75%
	2		gi|157165027|ref|YP_001466406.1|	Campylobacter concisus 13826	E-value = 6.00E-158	Identity = 76.95%
	3		gi|15791826|ref|NP_281649.1|	Campylobacter jejuni subsp. jejuni NCTC 11168	E-value = 3.00E-157	Identity = 78.10%
	4		gi|154175395|ref|YP_001407962.1|	Campylobacter curvus 525.92	E-value = 5.00E-157	Identity = 76.95%
	5		gi|152991101|ref|YP_001356823.1|	Nitratiruptor sp. SB155-2	E-value = 2.00E-156	Identity = 76.88%
AB1221	1		gi|78776606|ref|YP_392921.1|	Sulfuromonas denitrificans ATCC 33889	E-value = 6.00E-103	Identity = 48.94%
	2		gi|152993499|ref|YP_001359220.1|	Sulfurovum sp. NBC37-1	E-value = 8.00E-100	Identity = 47.57%
	3		gi|152991100|ref|YP_001356822.1|	Nitratiruptor sp. SB155-2	E-value = 9.00E-97	Identity = 48.67%
	4		gi|34556786|ref|NP_906601.1|	Wolinella succinogenes DSM 1740	E-value = 6.00E-95	Identity = 48.05%
	5		gi|32266489|ref|NP_860521.1|	Helicobacter hepaticus ATCC 51449	E-value = 4.00E-91	Identity = 46.88%
AB1222	1	*gltX2*	gi|78777257|ref|YP_393572.1|	Sulfuromonas denitrificans ATCC 33889	E-value = 5.00E-155	Identity = 63.51%
	2		gi|148926546|ref|ZP_01810228.1|	Campylobacter jejuni subsp. jejuni CG8486	E-value = 6.00E-139	Identity = 59.58%
	3		gi|57167845|ref|ZP_00366985.1|	Campylobacter coli RM2228	E-value = 9.00E-139	Identity = 58.66%
	4		gi|57237686|ref|YP_178934.1|	Campylobacter jejuni subsp. jejuni RM1221	E-value = 2.00E-138	Identity = 59.12%
	5		gi|86150270|ref|ZP_01068497.1|	Campylobacter jejuni subsp. jejuni CF93-6	E-value = 2.00E-138	Identity = 59.35%
AB1223	1		gi|152990547|ref|YP_001356269.1|	Nitratiruptor sp. SB155-2	E-value = 2.00E-15	Identity = 60.67%
	2		gi|149193751|ref|ZP_01870849.1|	Caminibacter mediatlanticus TB-2	E-value = 2.00E-14	Identity = 58.62%
	3		gi|154174516|ref|YP_001407922.1|	Campylobacter curvus 525.92	E-value = 1.00E-13	Identity = 55.56%
	4		gi|34556711|ref|NP_906526.1|	Wolinella succinogenes DSM 1740	E-value = 2.00E-13	Identity = 55.56%

	5		gi|152992728|ref|YP_001358449.1|	Sulfurovum sp. NBC37-1	E-value = 5.00E-13	Identity = 58.82%
AB1225	1	*slt*	gi|32266079|ref|NP_860111.1|	Helicobacter hepaticus ATCC 51449	E-value = 3.00E-67	Identity = 32.89%
	2		gi|34556710|ref|NP_906525.1|	Wolinella succinogenes DSM 1740	E-value = 1.00E-66	Identity = 33.08%
	3		gi|154174437|ref|YP_001407921.1|	Campylobacter curvus 525.92	E-value = 9.00E-59	Identity = 32.83%
	4		gi|15792181|ref|NP_282004.1|	Campylobacter jejuni subsp. jejuni NCTC 11168	E-value = 3.00E-56	Identity = 33.93%
	5		gi|118475611|ref|YP_891317.1|	Campylobacter fetus subsp. fetus 82-40	E-value = 6.00E-56	Identity = 30.80%
AB1226	1		gi|152992726|ref|YP_001358447.1|	Sulfurovum sp. NBC37-1	E-value = 6.00E-06	Identity = 27.11%
AB1227	1	*mobB*	gi|152990544|ref|YP_001356266.1|	Nitratiruptor sp. SB155-2	E-value = 4.00E-46	Identity = 60.25%
	2		gi|149195306|ref|ZP_01872394.1|	Caminibacter mediatlanticus TB-2	E-value = 9.00E-45	Identity = 59.63%
	3		gi|78777260|ref|YP_393575.1|	Sulfuromonas denitrificans ATCC 33889	E-value = 1.00E-44	Identity = 59.04%
	4		gi|157164895|ref|YP_001466421.1|	Campylobacter concisus 13826	E-value = 3.00E-42	Identity = 58.02%
	5		gi|152992725|ref|YP_001358446.1|	Sulfurovum sp. NBC37-1	E-value = 4.00E-42	Identity = 56.10%
AB1228	1	*fbp*	gi|78777261|ref|YP_393576.1|	Sulfuromonas denitrificans ATCC 33889	E-value = 1.00E-101	Identity = 63.44%
	2		gi|152990543|ref|YP_001356265.1|	Nitratiruptor sp. SB155-2	E-value = 3.00E-94	Identity = 60.57%
	3		gi|152992724|ref|YP_001358445.1|	Sulfurovum sp. NBC37-1	E-value = 6.00E-94	Identity = 60.42%
	4		gi|34556661|ref|NP_906476.1|	Wolinella succinogenes DSM 1740	E-value = 2.00E-92	Identity = 57.86%
	5		gi|118475247|ref|YP_891969.1|	Campylobacter fetus subsp. fetus 82-40	E-value = 2.00E-88	Identity = 57.19%
AB1229	1		gi|78777262|ref|YP_393577.1|	Sulfuromonas denitrificans ATCC 33889	E-value = 3.00E-09	Identity = 55.00%
	2		gi|57241193|ref|ZP_00369140.1|	Campylobacter lari RM2100	E-value = 8.00E-09	Identity = 57.63%
	3		gi|152992723|ref|YP_001358444.1|	Sulfurovum sp. NBC37-1	E-value = 1.00E-08	Identity = 49.21%
	4		gi|152990542|ref|YP_001356264.1|	Nitratiruptor sp. SB155-2	E-value = 2.00E-07	Identity = 50.00%
	5		gi|154173673|ref|YP_001407917.1|	Campylobacter curvus 525.92	E-value = 8.00E-07	Identity = 47.46%
AB1230	1	*metS*	gi|152992719|ref|YP_001358440.1|	Sulfurovum sp. NBC37-1	E-value = 0	Identity = 67.38%
	2		gi|34556660|ref|NP_906475.1|	Wolinella succinogenes DSM 1740	E-value = 0	Identity = 65.22%
	3		gi|78777263|ref|YP_393578.1|	Sulfuromonas denitrificans ATCC 33889	E-value = 0	Identity = 64.74%
	4		gi|152990541|ref|YP_001356263.1|	Nitratiruptor sp. SB155-2	E-value = 0	Identity = 67.39%
	5		gi|149195308|ref|ZP_01872396.1|	Caminibacter mediatlanticus TB-2	E-value = 0	Identity = 62.42%
AB1231	1		gi|152990540|ref|YP_001356262.1|	Nitratiruptor sp. SB155-2	E-value = 5.00E-27	Identity = 30.99%
	2		gi|78777264|ref|YP_393579.1|	Sulfuromonas denitrificans ATCC 33889	E-value = 2.00E-24	Identity = 29.91%
	3		gi|118474938|ref|YP_891972.1|	Campylobacter fetus subsp. fetus 82-40	E-value = 1.00E-23	Identity = 29.41%
	4		gi|154174953|ref|YP_001407915.1|	Campylobacter curvus 525.92	E-value = 6.00E-23	Identity = 25.48%
	5		gi|152992718|ref|YP_001358439.1|	Sulfurovum sp. NBC37-1	E-value = 2.00E-20	Identity = 27.59%
AB1232	1		gi|34556743|ref|NP_906558.1|	Wolinella succinogenes DSM 1740	E-value = 1.00E-57	Identity = 49.53%
	2		gi|78777294|ref|YP_393609.1|	Sulfuromonas denitrificans ATCC 33889	E-value = 2.00E-57	Identity = 52.58%
	3		gi|152990029|ref|YP_001355751.1|	Nitratiruptor sp. SB155-2	E-value = 4.00E-52	Identity = 46.26%
	4		gi|152991709|ref|YP_001357430.1|	Sulfurovum sp. NBC37-1	E-value = 4.00E-47	Identity = 46.76%
	5		gi|149195301|ref|ZP_01872389.1|	Caminibacter mediatlanticus TB-2	E-value = 2.00E-38	Identity = 42.86%
AB1233	1			***** No matches found *****		
AB1234	1			***** No matches found *****		
AB1235	1	*pycB2*	gi|152991046|ref|YP_001356768.1|	Nitratiruptor sp. SB155-2	E-value = 0	Identity = 72.49%
	2		gi|152993020|ref|YP_001358741.1|	Sulfurovum sp. NBC37-1	E-value = 0	Identity = 72.41%
	3		gi|149194559|ref|ZP_01871655.1|	Caminibacter mediatlanticus TB-2	E-value = 0	Identity = 69.46%
	4		gi|154174885|ref|YP_001407832.1|	Campylobacter curvus 525.92	E-value = 0	Identity = 69.44%
	5		gi|157165713|ref|YP_001466387.1|	Campylobacter concisus 13826	E-value = 0	Identity = 69.46%
AB1236	1	*pckA*	gi|152993018|ref|YP_001358739.1|	Sulfurovum sp. NBC37-1	E-value = 0	Identity = 71.54%
	2		gi|57240611|ref|ZP_00368560.1|	Campylobacter lari RM2100	E-value = 0	Identity = 69.62%
	3		gi|118474992|ref|YP_892449.1|	Campylobacter fetus subsp. fetus 82-40	E-value = 0	Identity = 69.94%
	4		gi|157165286|ref|YP_001466388.1|	Campylobacter concisus 13826	E-value = 0	Identity = 68.64%
	5		gi|154174492|ref|YP_001407833.1|	Campylobacter curvus 525.92	E-value = 0	Identity = 69.22%

AB1237	1	*ribAB*	gi|78777387|ref|YP_393702.1|	Sulfuromonas denitrificans ATCC 33889	E-value = 7.00E-112	Identity = 61.40%
	2		gi|152990431|ref|YP_001356153.1|	Nitratiruptor sp. SB155-2	E-value = 4.00E-109	Identity = 58.58%
	3		gi|8928299|sp|O68249|RIBB_DEHMU	Dehalospirillum multivorans	E-value = 4.00E-105	Identity = 56.64%
	4		gi|152992732|ref|YP_001358453.1|	Sulfurovum sp. NBC37-1	E-value = 1.00E-103	Identity = 55.29%
	5		gi|34557588|ref|NP_907403.1|	Wolinella succinogenes DSM 1740	E-value = 1.00E-94	Identity = 52.35%
AB1238	1	*glyS*	gi|152990481|ref|YP_001356203.1|	Nitratiruptor sp. SB155-2	E-value = 0	Identity = 56.57%
	2		gi|78777189|ref|YP_393504.1|	Sulfuromonas denitrificans ATCC 33889	E-value = 0	Identity = 54.98%
	3		gi|152993287|ref|YP_001359008.1|	Sulfurovum sp. NBC37-1	E-value = 0	Identity = 53.23%
	4		gi|34556652|ref|NP_906467.1|	Wolinella succinogenes DSM 1740	E-value = 0	Identity = 51.32%
	5		gi|154173823|ref|YP_001408091.1|	Campylobacter curvus 525.92	E-value = 0	Identity = 51.1%
AB1239	1			***** No matches found *****		
AB1240	1		gi|152990105|ref|YP_001355827.1|	Nitratiruptor sp. SB155-2	E-value = 6.00E-13	Identity = 38.26%
	2		gi|154175457|ref|YP_001408665.1|	Campylobacter curvus 525.92	E-value = 3.00E-12	Identity = 38.98%
	3		gi|157165160|ref|YP_001466521.1|	Campylobacter concisus 13826	E-value = 4.00E-11	Identity = 38.46%
	4		gi|78776582|ref|YP_392897.1|	Sulfuromonas denitrificans ATCC 33889	E-value = 5.00E-10	Identity = 33.06%
	5		gi|153951374|ref|YP_001398236.1|	Campylobacter jejuni subsp. doylei 269.97	E-value = 1.00E-09	Identity = 38.14%
AB1241	1		gi|78776583|ref|YP_392898.1|	Sulfuromonas denitrificans ATCC 33889	E-value = 4.00E-08	Identity = 50.00%
	2		gi|152990106|ref|YP_001355828.1|	Nitratiruptor sp. SB155-2	E-value = 5.00E-07	Identity = 50.75%
AB1242	1	*gidB*	gi|152993652|ref|YP_001359373.1|	Sulfurovum sp. NBC37-1	E-value = 3.00E-38	Identity = 47.62%
	2		gi|34557261|ref|NP_907076.1|	Wolinella succinogenes DSM 1740	E-value = 8.00E-35	Identity = 41.67%
	3		gi|152990107|ref|YP_001355829.1|	Nitratiruptor sp. SB155-2	E-value = 1.00E-32	Identity = 43.43%
	4		gi|78776584|ref|YP_392899.1|	Sulfuromonas denitrificans ATCC 33889	E-value = 2.00E-31	Identity = 41.40%
	5		gi|149194230|ref|ZP_01871327.1|	Caminibacter mediatlanticus TB-2	E-value = 2.00E-30	Identity = 47.37%
AB1243	1	*ribA*	gi|152993665|ref|YP_001359386.1|	Sulfurovum sp. NBC37-1	E-value = 2.00E-57	Identity = 59.49%
	2		gi|78776585|ref|YP_392900.1|	Sulfuromonas denitrificans ATCC 33889	E-value = 1.00E-56	Identity = 57.07%
	3		gi|152990108|ref|YP_001355830.1|	Nitratiruptor sp. SB155-2	E-value = 5.00E-56	Identity = 57.07%
	4		gi|154175373|ref|YP_001408662.1|	Campylobacter curvus 525.92	E-value = 6.00E-56	Identity = 58.12%
	5		gi|118474145|ref|YP_892708.1|	Campylobacter fetus subsp. fetus 82-40	E-value = 3.00E-55	Identity = 57.81%
AB1244	1	*hemB*	gi|154173852|ref|YP_001408661.1|	Campylobacter curvus 525.92	E-value = 4.00E-143	Identity = 74.92%
	2		gi|154148310|ref|YP_001407210.1|	Campylobacter hominis ATCC BAA-381	E-value = 2.00E-142	Identity = 74.30%
	3		gi|152990109|ref|YP_001355831.1|	Nitratiruptor sp. SB155-2	E-value = 5.00E-142	Identity = 74.77%
	4		gi|157164906|ref|YP_001466525.1|	Campylobacter concisus 13826	E-value = 4.00E-141	Identity = 73.60%
	5		gi|78776586|ref|YP_392901.1|	Sulfuromonas denitrificans ATCC 33889	E-value = 4.00E-141	Identity = 73.07%
AB1245	1		gi|78777441|ref|YP_393756.1|	Sulfuromonas denitrificans ATCC 33889	E-value = 3.00E-87	Identity = 49.25%
	2		gi|154173641|ref|YP_001407572.1|	Campylobacter curvus 525.92	E-value = 1.00E-82	Identity = 46.70%
	3		gi|152991361|ref|YP_001357083.1|	Nitratiruptor sp. SB155-2	E-value = 6.00E-80	Identity = 43.69%
	4		gi|118475391|ref|YP_891578.1|	Campylobacter fetus subsp. fetus 82-40	E-value = 2.00E-79	Identity = 46.52%
	5		gi|154147923|ref|YP_001406912.1|	Campylobacter hominis ATCC BAA-381	E-value = 4.00E-71	Identity = 42.68%
AB1246	1		gi|78777440|ref|YP_393755.1|	Sulfuromonas denitrificans ATCC 33889	E-value = 2.00E-42	Identity = 51.58%
	2		gi|118475245|ref|YP_891579.1|	Campylobacter fetus subsp. fetus 82-40	E-value = 5.00E-38	Identity = 45.41%
	3		gi|86152014|ref|ZP_01070227.1|	Campylobacter jejuni subsp. jejuni 260.94	E-value = 1.00E-36	Identity = 46.58%
	4		gi|86152639|ref|ZP_01070844.1|	Campylobacter jejuni subsp. jejuni HB93-13	E-value = 1.00E-36	Identity = 46.58%
	5		gi|15792806|ref|NP_282629.1|	Campylobacter jejuni subsp. jejuni NCTC 11168	E-value = 1.00E-36	Identity = 46.12%
AB1247	1		gi|154173832|ref|YP_001407349.1|	Campylobacter curvus 525.92	E-value = 5.00E-53	Identity = 46.22%
	2		gi|78777438|ref|YP_393753.1|	Sulfuromonas denitrificans ATCC 33889	E-value = 5.00E-49	Identity = 53.93%
	3		gi|152991853|ref|YP_001357574.1|	Sulfurovum sp. NBC37-1	E-value = 3.00E-48	Identity = 46.33%
	4		gi|149194702|ref|ZP_01871797.1|	Caminibacter mediatlanticus TB-2	E-value = 3.00E-46	Identity = 46.46%
	5		gi|157163946|ref|YP_001467812.1|	Campylobacter concisus 13826	E-value = 6.00E-45	Identity = 43.81%
AB1248	1		gi|118474776|ref|YP_891233.1|	Campylobacter fetus subsp. fetus 82-40	E-value = 3.00E-10	Identity = 69.39%

	2		gi|152991852|ref|YP_001357573.1|	Sulfurovum sp. NBC37-1	E-value = 2.00E-09	Identity = 69.23%
	3		gi|146301795|ref|YP_001196386.1|	Flavobacterium johnsoniae UW101	E-value = 1.00E-08	Identity = 80.95%
	4		gi|154173656|ref|YP_001409072.1|	Campylobacter curvus 525.92	E-value = 4.00E-08	Identity = 63.27%
	5		gi|152991510|ref|YP_001357232.1|	Nitratiruptor sp. SB155-2	E-value = 6.00E-08	Identity = 66.67%
AB1249	1	*nrdD*	gi|78777437|ref|YP_393752.1|	Sulfuromonas denitrificans ATCC 33889	E-value = 0	Identity = 79.08%
	2		gi|34557096|ref|NP_906911.1|	Wolinella succinogenes DSM 1740	E-value = 0	Identity = 73.88%
	3		gi|152991850|ref|YP_001357571.1|	Sulfurovum sp. NBC37-1	E-value = 0	Identity = 74.57%
	4		gi|152991511|ref|YP_001357233.1|	Nitratiruptor sp. SB155-2	E-value = 0	Identity = 73.39%
	5		gi|154175399|ref|YP_001407350.1|	Campylobacter curvus 525.92	E-value = 0	Identity = 71.53%
AB1250	1	*argF*	gi|152993667|ref|YP_001359388.1|	Sulfurovum sp. NBC37-1	E-value = 1.00E-127	Identity = 74.01%
	2		gi|152990110|ref|YP_001355832.1|	Nitratiruptor sp. SB155-2	E-value = 2.00E-127	Identity = 74.03%
	3		gi|34558060|ref|NP_907875.1|	Wolinella succinogenes DSM 1740	E-value = 2.00E-120	Identity = 69.61%
	4		gi|78776587|ref|YP_392902.1|	Sulfuromonas denitrificans ATCC 33889	E-value = 7.00E-117	Identity = 68.08%
	5		gi|118474198|ref|YP_892706.1|	Campylobacter fetus subsp. fetus 82-40	E-value = 4.00E-114	Identity = 66.99%
AB1251	1	*hemN2*	gi|152990113|ref|YP_001355835.1|	Nitratiruptor sp. SB155-2	E-value = 0	Identity = 67.25%
	2		gi|78776805|ref|YP_393120.1|	Sulfuromonas denitrificans ATCC 33889	E-value = 1.00E-173	Identity = 63.96%
	3		gi|152993668|ref|YP_001359389.1|	Sulfurovum sp. NBC37-1	E-value = 6.00E-172	Identity = 64.98%
	4		gi|157164719|ref|YP_001466528.1|	Campylobacter concisus 13826	E-value = 2.00E-170	Identity = 63.96%
	5		gi|154174680|ref|YP_001408658.1|	Campylobacter curvus 525.92	E-value = 5.00E-170	Identity = 62.86%
AB1252	1			***** No matches found *****		
AB1253	1			***** No matches found *****		
AB1254	1	*lpxK*	gi|32266508|ref|NP_860540.1|	Helicobacter hepaticus ATCC 51449	E-value = 9.00E-64	Identity = 46.10%
	2		gi|154174985|ref|YP_001408053.1|	Campylobacter curvus 525.92	E-value = 2.00E-60	Identity = 42.96%
	3		gi|109947533|ref|YP_664761.1|	Helicobacter acinonychis str. Sheeba	E-value = 9.00E-59	Identity = 46.43%
	4		gi|78777051|ref|YP_393366.1|	Sulfuromonas denitrificans ATCC 33889	E-value = 5.00E-58	Identity = 46.83%
	5		gi|15644956|ref|NP_207126.1|	Helicobacter pylori 26695	E-value = 6.00E-58	Identity = 45.45%
AB1255	1		gi|152990789|ref|YP_001356511.1|	Nitratiruptor sp. SB155-2	E-value = 2.00E-106	Identity = 53.46%
	2		gi|34556648|ref|NP_906463.1|	Wolinella succinogenes DSM 1740	E-value = 3.00E-102	Identity = 50.40%
	3		gi|118474790|ref|YP_892240.1|	Campylobacter fetus subsp. fetus 82-40	E-value = 8.00E-99	Identity = 48.94%
	4		gi|157164603|ref|YP_001467121.1|	Campylobacter concisus 13826	E-value = 5.00E-94	Identity = 49.33%
	5		gi|154175374|ref|YP_001408054.1|	Campylobacter curvus 525.92	E-value = 2.00E-93	Identity = 48.94%
AB1256	1	*nadE*	gi|149194281|ref|ZP_01871378.1|	Caminibacter mediatlanticus TB-2	E-value = 3.00E-70	Identity = 56.22%
	2		gi|78777049|ref|YP_393364.1|	Sulfuromonas denitrificans ATCC 33889	E-value = 2.00E-69	Identity = 53.39%
	3		gi|34556649|ref|NP_906464.1|	Wolinella succinogenes DSM 1740	E-value = 2.00E-64	Identity = 52.70%
	4		gi|39995758|ref|NP_951709.1|	Geobacter sulfurreducens PCA	E-value = 1.00E-63	Identity = 48.37%
	5		gi|148265774|ref|YP_001232480.1|	Geobacter uraniumreducens Rf4	E-value = 3.00E-63	Identity = 48.78%
AB1257	1			***** No matches found *****		
AB1258	1			***** No matches found *****		
AB1259	1		gi|78776598|ref|YP_392913.1|	Sulfuromonas denitrificans ATCC 33889	E-value = 7.00E-36	Identity = 39.11%
	2		gi|154173810|ref|YP_001408429.1|	Campylobacter curvus 525.92	E-value = 2.00E-29	Identity = 37.56%
	3		gi|157165606|ref|YP_001466653.1|	Campylobacter concisus 13826	E-value = 9.00E-27	Identity = 35.38%
	4		gi|154148447|ref|YP_001406249.1|	Campylobacter hominis ATCC BAA-381	E-value = 2.00E-26	Identity = 34.96%
	5		gi|118474971|ref|YP_891747.1|	Campylobacter fetus subsp. fetus 82-40	E-value = 1.00E-20	Identity = 35.75%
AB1260	1		gi|54297010|ref|YP_123379.1|	Legionella pneumophila str. Paris	E-value = 3.00E-06	Identity = 33.65%
AB1261	1		gi|154174852|ref|YP_001408007.1|	Campylobacter curvus 525.92	E-value = 2.00E-14	Identity = 45.53%
	2		gi|154148643|ref|YP_001406651.1|	Campylobacter hominis ATCC BAA-381	E-value = 1.00E-09	Identity = 42.86%
	3		gi|152990888|ref|YP_001356610.1|	Nitratiruptor sp. SB155-2	E-value = 1.00E-09	Identity = 37.70%
	4		gi|78777852|ref|YP_394167.1|	Sulfuromonas denitrificans ATCC 33889	E-value = 1.00E-07	Identity = 42.24%
	5		gi|85711923|ref|ZP_01042978.1|	Idiomarina baltica OS145	E-value = 2.00E-06	Identity = 32.41%

AB1262	1	*tatC*	gi|118475065|ref|YP_892278.1|	Campylobacter fetus subsp. fetus 82-40	E-value = 4.00E-76	Identity = 65.31%
	2		gi|152990889|ref|YP_001356611.1|	Nitratiruptor sp. SB155-2	E-value = 7.00E-74	Identity = 69.79%
	3		gi|154174804|ref|YP_001408006.1|	Campylobacter curvus 525.92	E-value = 5.00E-73	Identity = 63.27%
	4		gi|34557263|ref|NP_907078.1|	Wolinella succinogenes DSM 1740	E-value = 1.00E-64	Identity = 65.15%
	5		gi|154148770|ref|YP_001406650.1|	Campylobacter hominis ATCC BAA-381	E-value = 2.00E-63	Identity = 59.83%
AB1263	1	*queA*	gi|152992047|ref|YP_001357768.1|	Sulfurovum sp. NBC37-1	E-value = 3.00E-119	Identity = 62.76%
	2		gi|152990890|ref|YP_001356612.1|	Nitratiruptor sp. SB155-2	E-value = 1.00E-116	Identity = 62.94%
	3		gi|149193933|ref|ZP_01871031.1|	Caminibacter mediatlanticus TB-2	E-value = 4.00E-112	Identity = 63.48%
	4		gi|154149256|ref|YP_001406649.1|	Campylobacter hominis ATCC BAA-381	E-value = 2.00E-107	Identity = 58.53%
	5		gi|57168187|ref|ZP_00367326.1|	Campylobacter coli RM2228	E-value = 1.00E-104	Identity = 57.23%
AB1264	1	*dnaQ4*	gi|152992669|ref|YP_001358390.1|	Sulfurovum sp. NBC37-1	E-value = 4.00E-85	Identity = 66.94%
	2		gi|78777984|ref|YP_394299.1|	Sulfuromonas denitrificans ATCC 33889	E-value = 6.00E-64	Identity = 52.87%
	3		gi|149195052|ref|ZP_01872144.1|	Caminibacter mediatlanticus TB-2	E-value = 8.00E-45	Identity = 44.26%
	4		gi|78776758|ref|YP_393073.1|	Sulfuromonas denitrificans ATCC 33889	E-value = 7.00E-36	Identity = 36.93%
	5		gi|77980074|ref|ZP_00835489.1|	Yersinia intermedia ATCC 29909	E-value = 8.00E-25	Identity = 32.24%
AB1265	1		gi|126175882|ref|YP_001052031.1|	Shewanella baltica OS155	E-value = 5.00E-41	Identity = 45.37%
	2		gi|113947099|ref|ZP_01432765.1|	Shewanella baltica OS195	E-value = 3.00E-40	Identity = 44.91%
	3		gi|152999209|ref|YP_001364890.1|	Shewanella baltica OS185	E-value = 4.00E-40	Identity = 43.52%
	4		gi|149115184|ref|ZP_01841930.1|	Shewanella baltica OS223	E-value = 8.00E-40	Identity = 40.24%
	5		gi|53711450|ref|YP_097442.1|	Bacteroides fragilis YCH46	E-value = 4.00E-37	Identity = 43.83%
AB1266	1	*comE*	gi|154175464|ref|YP_001408498.1|	Campylobacter curvus 525.92	E-value = 3.00E-45	Identity = 34.21%
	2		gi|34556789|ref|NP_906604.1|	Wolinella succinogenes DSM 1740	E-value = 2.00E-44	Identity = 32.17%
	3		gi|57240631|ref|ZP_00368580.1|	Campylobacter lari RM2100	E-value = 8.00E-44	Identity = 38.11%
	4		gi|152990088|ref|YP_001355810.1|	Nitratiruptor sp. SB155-2	E-value = 1.00E-43	Identity = 34.00%
	5		gi|152993708|ref|YP_001359429.1|	Sulfurovum sp. NBC37-1	E-value = 4.00E-41	Identity = 33.60%
AB1267	1			***** No matches found *****		
AB1268	1		gi|148262102|ref|YP_001228808.1|	Geobacter uraniumreducens Rf4	E-value = 4.00E-174	Identity = 46.60%
	2		gi|50083707|ref|YP_045217.1|	Acinetobacter sp. ADP1	E-value = 4.00E-173	Identity = 47.70%
	3		gi|152991993|ref|YP_001357714.1|	Sulfurovum sp. NBC37-1	E-value = 1.00E-170	Identity = 45.53%
	4		gi|120553327|ref|YP_957678.1|	Marinobacter aquaeolei VT8	E-value = 2.00E-170	Identity = 47.86%
	5		gi|145620400|ref|ZP_01776432.1|	Geobacter bemidjiensis Bem	E-value = 6.00E-170	Identity = 45.38%
AB1269	1			***** No matches found *****		
AB1270	1			***** No matches found *****		
AB1271	1			***** No matches found *****		
AB1272	1	*glpC*	gi|152993669|ref|YP_001359390.1|	Sulfurovum sp. NBC37-1	E-value = 5.00E-170	Identity = 64.57%
	2		gi|152990116|ref|YP_001355838.1|	Nitratiruptor sp. SB155-2	E-value = 1.00E-165	Identity = 63.64%
	3		gi|78776806|ref|YP_393121.1|	Sulfuromonas denitrificans ATCC 33889	E-value = 4.00E-157	Identity = 62.53%
	4		gi|34558057|ref|NP_907872.1|	Wolinella succinogenes DSM 1740	E-value = 6.00E-150	Identity = 60.09%
	5		gi|118475651|ref|YP_892701.1|	Campylobacter fetus subsp. fetus 82-40	E-value = 3.00E-148	Identity = 59.91%
AB1273	1	*lgt*	gi|152990119|ref|YP_001355841.1|	Nitratiruptor sp. SB155-2	E-value = 3.00E-79	Identity = 57.89%
	2		gi|34558394|ref|NP_908209.1|	Wolinella succinogenes DSM 1740	E-value = 9.00E-79	Identity = 60.55%
	3		gi|57240863|ref|ZP_00368811.1|	Campylobacter lari RM2100	E-value = 2.00E-74	Identity = 59.83%
	4		gi|154149140|ref|YP_001406419.1|	Campylobacter hominis ATCC BAA-381	E-value = 3.00E-74	Identity = 55.13%
	5		gi|118475330|ref|YP_891619.1|	Campylobacter fetus subsp. fetus 82-40	E-value = 7.00E-74	Identity = 55.38%
AB1274	1			***** No matches found *****		
AB1275	1		gi|154173869|ref|YP_001407525.1|	Campylobacter curvus 525.92	E-value = 9.00E-22	Identity = 32.90%
	2		gi|118474558|ref|YP_891350.1|	Campylobacter fetus subsp. fetus 82-40	E-value = 5.00E-18	Identity = 28.96%
	3		gi|149194353|ref|ZP_01871450.1|	Caminibacter mediatlanticus TB-2	E-value = 4.00E-14	Identity = 29.77%
	4		gi|34558332|ref|NP_908147.1|	Wolinella succinogenes DSM 1740	E-value = 4.00E-13	Identity = 27.06%

	5		gi|157164839|ref|YP_001466181.1|	Campylobacter concisus 13826	E-value = 4.00E-11	Identity = 26.52%
AB1276	1	*polA*	gi|152990932|ref|YP_001356654.1|	Nitratiruptor sp. SB155-2	E-value = 0	Identity = 54.13%
	2		gi|34556857|ref|NP_906672.1|	Wolinella succinogenes DSM 1740	E-value = 0	Identity = 52.22%
	3		gi|78777442|ref|YP_393757.1|	Sulfuromonas denitrificans ATCC 33889	E-value = 0	Identity = 53.49%
	4		gi|152992904|ref|YP_001358625.1|	Sulfurovum sp. NBC37-1	E-value = 0	Identity = 51.39%
	5		gi|32266816|ref|NP_860848.1|	Helicobacter hepaticus ATCC 51449	E-value = 0	Identity = 48.47%
AB1277	1		gi|52345267|emb|CAG30571.1|	Streptococcus pyogenes	E-value = 1.00E-51	Identity = 56.90%
	2		gi|75760144|ref|ZP_00740204.1|	Bacillus thuringiensis serovar israelensis ATCC 35646	E-value = 4.00E-49	Identity = 55.49%
	3		gi|49478854|ref|YP_039043.1|	Bacillus thuringiensis serovar konkukian str. 97-27	E-value = 5.00E-49	Identity = 53.76%
	4		gi|47567067|ref|ZP_00237784.1|	Bacillus cereus G9241	E-value = 7.00E-49	Identity = 54.91%
	5		gi|52140509|ref|YP_086320.1|	Bacillus cereus E33L	E-value = 1.00E-48	Identity = 54.91%
AB1278	1	*kdsB*	gi|78776926|ref|YP_393241.1|	Sulfuromonas denitrificans ATCC 33889	E-value = 6.00E-74	Identity = 58.58%
	2		gi|152993256|ref|YP_001358977.1|	Sulfurovum sp. NBC37-1	E-value = 3.00E-71	Identity = 60.08%
	3		gi|34558211|ref|NP_908026.1|	Wolinella succinogenes DSM 1740	E-value = 2.00E-65	Identity = 50.21%
	4		gi|86152189|ref|ZP_01070401.1|	Campylobacter jejuni subsp. jejuni 260.94	E-value = 2.00E-63	Identity = 54.36%
	5		gi|157415084|ref|YP_001482340.1|	Campylobacter jejuni subsp. jejuni 81116	E-value = 3.00E-63	Identity = 54.36%
AB1279	1		gi|34556560|ref|NP_906375.1|	Wolinella succinogenes DSM 1740	E-value = 2.00E-87	Identity = 34.41%
	2		gi|150017350|ref|YP_001309604.1|	Clostridium beijerinckii NCIMB 8052	E-value = 2.00E-51	Identity = 29.76%
	3		gi|146311190|ref|YP_001176264.1|	Enterobacter sp. 638	E-value = 8.00E-45	Identity = 28.97%
	4		gi|78223826|ref|YP_385573.1|	Geobacter metallireducens GS-15	E-value = 3.00E-39	Identity = 25.32%
AB1280	1		gi|118474771|ref|YP_892027.1|	Campylobacter fetus subsp. fetus 82-40	E-value = 4.00E-94	Identity = 72.69%
	2		gi|78777347|ref|YP_393662.1|	Sulfuromonas denitrificans ATCC 33889	E-value = 4.00E-94	Identity = 71.25%
	3		gi|157164499|ref|YP_001466828.1|	Campylobacter concisus 13826	E-value = 9.00E-94	Identity = 71.25%
	4		gi|154174132|ref|YP_001408320.1|	Campylobacter curvus 525.92	E-value = 6.00E-92	Identity = 70.00%
	5		gi|34558382|ref|NP_908197.1|	Wolinella succinogenes DSM 1740	E-value = 1.00E-88	Identity = 67.65%
AB1281	1		gi|78777348|ref|YP_393663.1|	Sulfuromonas denitrificans ATCC 33889	E-value = 4.00E-28	Identity = 50.37%
	2		gi|118475620|ref|YP_892026.1|	Campylobacter fetus subsp. fetus 82-40	E-value = 3.00E-24	Identity = 46.72%
	3		gi|109947486|ref|YP_664714.1|	Helicobacter acinonychis str. Sheeba	E-value = 5.00E-24	Identity = 47.33%
	4		gi|108563126|ref|YP_627442.1|	Helicobacter pylori HPAG1	E-value = 6.00E-24	Identity = 48.06%
	5		gi|15611721|ref|NP_223372.1|	Helicobacter pylori J99	E-value = 7.00E-24	Identity = 48.06%
AB1282	1	*trpD*	gi|152990596|ref|YP_001356318.1|	Nitratiruptor sp. SB155-2	E-value = 5.00E-93	Identity = 55.80%
	2		gi|152993172|ref|YP_001358893.1|	Sulfurovum sp. NBC37-1	E-value = 2.00E-91	Identity = 55.45%
	3		gi|149194532|ref|ZP_01871628.1|	Caminibacter mediatlanticus TB-2	E-value = 3.00E-91	Identity = 57.01%
	4		gi|34558380|ref|NP_908195.1|	Wolinella succinogenes DSM 1740	E-value = 5.00E-84	Identity = 53.73%
	5		gi|78777349|ref|YP_393664.1|	Sulfuromonas denitrificans ATCC 33889	E-value = 3.00E-81	Identity = 50.92%
AB1283	1		gi|78776337|ref|YP_392652.1|	Sulfuromonas denitrificans ATCC 33889	E-value = 1.00E-52	Identity = 39.86%
	2		gi|78778035|ref|YP_394350.1|	Sulfuromonas denitrificans ATCC 33889	E-value = 3.00E-40	Identity = 34.33%
	3		gi|152992341|ref|YP_001358062.1|	Sulfurovum sp. NBC37-1	E-value = 1.00E-37	Identity = 32.97%
	4		gi|152993221|ref|YP_001358942.1|	Sulfurovum sp. NBC37-1	E-value = 4.00E-25	Identity = 27.82%
	5		gi|154173719|ref|YP_001407872.1|	Campylobacter curvus 525.92	E-value = 2.00E-15	Identity = 28.40%
AB1284	1		gi|157414942|ref|YP_001482198.1|	Campylobacter jejuni subsp. jejuni 81116	E-value = 7.00E-16	Identity = 60.49%
	2		gi|86151691|ref|ZP_01069905.1|	Campylobacter jejuni subsp. jejuni 260.94	E-value = 9.00E-16	Identity = 60.49%
	3		gi|88597127|ref|ZP_01100363.1|	Campylobacter jejuni subsp. jejuni 84-25	E-value = 1.00E-15	Identity = 59.26%
	4		gi|34558439|ref|NP_908254.1|	Wolinella succinogenes DSM 1740	E-value = 1.00E-15	Identity = 57.69%
	5		gi|57236976|ref|YP_178777.1|	Campylobacter jejuni subsp. jejuni RM1221	E-value = 2.00E-15	Identity = 59.26%
AB1285	1	*argG*	gi|152993271|ref|YP_001358992.1|	Sulfurovum sp. NBC37-1	E-value = 0	Identity = 78.69%
	2		gi|118474439|ref|YP_892017.1|	Campylobacter fetus subsp. fetus 82-40	E-value = 3.00E-179	Identity = 74.82%
	3		gi|78777570|ref|YP_393885.1|	Sulfuromonas denitrificans ATCC 33889	E-value = 4.00E-178	Identity = 75.12%
	4		gi|57236975|ref|YP_178776.1|	Campylobacter jejuni subsp. jejuni RM1221	E-value = 1.00E-175	Identity = 73.97%

	5		gi|148926641|ref|ZP_01810322.1|	Campylobacter jejuni subsp. jejuni CG8486	E-value = 1.00E-175	Identity = 73.97%
AB1286	1	*dltA*	gi|115424044|emb|CAJ50597.1|	Bordetella avium 197N	E-value = 3.00E-98	Identity = 39.59%
	2		gi|33598414|ref|NP_886057.1|	Bordetella parapertussis 12822	E-value = 3.00E-93	Identity = 38.68%
	3		gi|33593919|ref|NP_881563.1|	Bordetella pertussis Tohama I	E-value = 4.00E-93	Identity = 38.68%
	4		gi|50122051|ref|YP_051218.1|	Erwinia carotovora subsp. atroseptica SCRI1043	E-value = 3.00E-91	Identity = 40.79%
	5		gi|37528212|ref|NP_931557.1|	Photorhabdus luminescens subsp. laumondii TTO1	E-value = 8.00E-89	Identity = 40.46%
AB1287	1	*dltB*	gi|115424045|emb|CAJ50598.1|	Bordetella avium 197N	E-value = 5.00E-48	Identity = 38.24%
	2		gi|37528211|ref|NP_931556.1|	Photorhabdus luminescens subsp. laumondii TTO1	E-value = 7.00E-42	Identity = 38.53%
	3		gi|50122052|ref|YP_051219.1|	Erwinia carotovora subsp. atroseptica SCRI1043	E-value = 8.00E-39	Identity = 30.65%
	4		gi|116630358|ref|YP_815642.1|	Lactobacillus gasseri ATCC 33323	E-value = 8.00E-33	Identity = 31.89%
	5		gi|42519832|ref|NP_965762.1|	Lactobacillus johnsonii NCC 533	E-value = 4.00E-32	Identity = 32.16%
AB1288	1			***** No matches found *****		
AB1289	1	*dltD*	gi|37528210|ref|NP_931555.1|	Photorhabdus luminescens subsp. laumondii TTO1	E-value = 4.00E-18	Identity = 27.21%
AB1290	1	*dltC*		***** No matches found *****		
AB1291	1		gi|152990076|ref|YP_001355798.1|	Nitratiruptor sp. SB155-2	E-value = 2.00E-56	Identity = 61.27%
	2		gi|149193930|ref|ZP_01871028.1|	Caminibacter mediatlanticus TB-2	E-value = 4.00E-56	Identity = 62.64%
	3		gi|152993727|ref|YP_001359448.1|	Sulfurovum sp. NBC37-1	E-value = 2.00E-55	Identity = 61.49%
	4		gi|78777776|ref|YP_394091.1|	Sulfuromonas denitrificans ATCC 33889	E-value = 2.00E-55	Identity = 60.92%
	5		gi|15791601|ref|NP_281424.1|	Campylobacter jejuni subsp. jejuni NCTC 11168	E-value = 2.00E-45	Identity = 50.85%
AB1292	1		gi|78776632|ref|YP_392947.1|	Sulfuromonas denitrificans ATCC 33889	E-value = 0	Identity = 78.54%
	2		gi|83645302|ref|YP_433737.1|	Hahella chejuensis KCTC 2396	E-value = 0	Identity = 77.44%
	3		gi|152995877|ref|YP_001340712.1|	Marinomonas sp. MWYL1	E-value = 0	Identity = 78.21%
	4		gi|119857761|ref|ZP_01639188.1|	Pseudomonas putida W619	E-value = 0	Identity = 74.8%
	5		gi|126667859|ref|ZP_01738825.1|	Marinobacter sp. ELB17	E-value = 0	Identity = 74.6%
AB1293	1	*uspA*	gi|83645303|ref|YP_433738.1|	Hahella chejuensis KCTC 2396	E-value = 3.00E-54	Identity = 38.57%
	2		gi|57240793|ref|ZP_00368741.1|	Campylobacter lari RM2100	E-value = 2.00E-52	Identity = 42.14%
	3		gi|88799888|ref|ZP_01115460.1|	Reinekea sp. MED297	E-value = 2.00E-51	Identity = 37.14%
	4		gi|149374529|ref|ZP_01892303.1|	Marinobacter algicola DG893	E-value = 8.00E-47	Identity = 34.98%
	5		gi|110834372|ref|YP_693231.1|	Alcanivorax borkumensis SK2	E-value = 2.00E-46	Identity = 35.34%
AB1295	1	*ktrA*	gi|34557730|ref|NP_907545.1|	Wolinella succinogenes DSM 1740	E-value = 2.00E-49	Identity = 50.24%
	2		gi|78776729|ref|YP_393044.1|	Sulfuromonas denitrificans ATCC 33889	E-value = 1.00E-38	Identity = 44.86%
	3		gi|15606658|ref|NP_214038.1|	Aquifex aeolicus VF5	E-value = 2.00E-32	Identity = 38.97%
	4		gi|57240669|ref|ZP_00368617.1|	Campylobacter lari RM2100	E-value = 5.00E-31	Identity = 42.65%
	5		gi|118474169|ref|YP_892770.1|	Campylobacter fetus subsp. fetus 82-40	E-value = 7.00E-31	Identity = 40.28%
AB1296	1	*ktrB*	gi|34557731|ref|NP_907546.1|	Wolinella succinogenes DSM 1740	E-value = 3.00E-85	Identity = 45.52%
	2		gi|118475510|ref|YP_892769.1|	Campylobacter fetus subsp. fetus 82-40	E-value = 1.00E-84	Identity = 47.40%
	3		gi|57240670|ref|ZP_00368618.1|	Campylobacter lari RM2100	E-value = 1.00E-81	Identity = 45.72%
	4		gi|57238333|ref|YP_179461.1|	Campylobacter jejuni subsp. jejuni RM1221	E-value = 2.00E-80	Identity = 42.99%
	5		gi|153952224|ref|YP_001397629.1|	Campylobacter jejuni subsp. doylei 269.97	E-value = 5.00E-80	Identity = 42.99%
AB1297	1	*kdpE*	gi|78776730|ref|YP_393045.1|	Sulfuromonas denitrificans ATCC 33889	E-value = 3.00E-86	Identity = 73.89%
	2		gi|153811679|ref|ZP_01964347.1|	Ruminococcus obeum ATCC 29174	E-value = 7.00E-51	Identity = 46.05%
	3		gi|83623310|ref|ZP_00933585.1|	Burkholderia mallei JHU	E-value = 2.00E-49	Identity = 42.08%
	4		gi|153854044|ref|ZP_01995377.1|	Dorea longicatena DSM 13814	E-value = 2.00E-49	Identity = 44.30%
	5		gi|154483201|ref|ZP_02025649.1|	Eubacterium ventriosum ATCC 27560	E-value = 2.00E-49	Identity = 44.30%
AB1298	1	*kdpD*	gi|145619557|ref|ZP_01775605.1|	Geobacter bemidjiensis Bem	E-value = 5.00E-41	Identity = 31.96%
	2		gi|124546147|ref|ZP_01705263.1|	Shewanella putrefaciens 200	E-value = 7.00E-28	Identity = 30.09%
	3		gi|120600811|ref|YP_965385.1|	Shewanella sp. W3-18-1	E-value = 3.00E-27	Identity = 30.09%
	4		gi|149114035|ref|ZP_01840791.1|	Shewanella baltica OS223	E-value = 4.00E-27	Identity = 30.84%
	5		gi|126176487|ref|YP_001052636.1|	Shewanella baltica OS155	E-value = 7.00E-27	Identity = 30.52%

AB1299	1	*dut*	gi|148925624|ref|ZP_01809312.1|	Campylobacter jejuni subsp. jejuni CG8486	E-value = 4.00E-37	Identity = 40.34%
	2		gi|121612582|ref|YP_001001100.1|	Campylobacter jejuni subsp. jejuni 81-176	E-value = 6.00E-37	Identity = 40.34%
	3		gi|15792768|ref|NP_282591.1|	Campylobacter jejuni subsp. jejuni NCTC 11168	E-value = 8.00E-37	Identity = 40.34%
	4		gi|86151902|ref|ZP_01070115.1|	Campylobacter jejuni subsp. jejuni 260.94	E-value = 1.00E-36	Identity = 40.34%
	5		gi|57240945|ref|ZP_00368893.1|	Campylobacter lari RM2100	E-value = 3.00E-36	Identity = 38.75%
AB1300	1	*recR*	gi|78777482|ref|YP_393797.1|	Sulfuromonas denitrificans ATCC 33889	E-value = 4.00E-58	Identity = 61.05%
	2		gi|152990929|ref|YP_001356651.1|	Nitratiruptor sp. SB155-2	E-value = 2.00E-56	Identity = 55.79%
	3		gi|152993460|ref|YP_001359181.1|	Sulfurovum sp. NBC37-1	E-value = 2.00E-54	Identity = 58.20%
	4		gi|118475618|ref|YP_892295.1|	Campylobacter fetus subsp. fetus 82-40	E-value = 3.00E-54	Identity = 57.53%
	5		gi|34557110|ref|NP_906925.1|	Wolinella succinogenes DSM 1740	E-value = 5.00E-52	Identity = 49.47%
AB1301	1	*dnaJ*	gi|78777484|ref|YP_393799.1|	Sulfuromonas denitrificans ATCC 33889	E-value = 2.00E-125	Identity = 61.66%
	2		gi|34557109|ref|NP_906924.1|	Wolinella succinogenes DSM 1740	E-value = 2.00E-122	Identity = 60.00%
	3		gi|152990930|ref|YP_001356652.1|	Nitratiruptor sp. SB155-2	E-value = 2.00E-117	Identity = 56.84%
	4		gi|32267018|ref|NP_861050.1|	Helicobacter hepaticus ATCC 51449	E-value = 2.00E-114	Identity = 57.18%
	5		gi|118474865|ref|YP_892292.1|	Campylobacter fetus subsp. fetus 82-40	E-value = 9.00E-114	Identity = 57.99%
AB1302	1	*trpB2*	gi|78776941|ref|YP_393256.1|	Sulfuromonas denitrificans ATCC 33889	E-value = 0	Identity = 77.81%
	2		gi|66356326|ref|XP_625304.1|	Cryptosporidium parvum Iowa II	E-value = 2.00E-159	Identity = 68.81%
	3		gi|34557127|ref|NP_906942.1|	Wolinella succinogenes DSM 1740	E-value = 1.00E-158	Identity = 70.60%
	4		gi|67583616|ref|XP_665003.1|	Cryptosporidium hominis TU502	E-value = 2.00E-156	Identity = 68.86%
	5		gi|153892496|ref|ZP_02013395.1|	Opitutaceae bacterium TAV2	E-value = 1.00E-154	Identity = 70.84%
AB1303	1			***** No matches found *****		
AB1304	1			***** No matches found *****		
AB1305	1		gi|152990790|ref|YP_001356512.1|	Nitratiruptor sp. SB155-2	E-value = 2.00E-71	Identity = 47.01%
	2		gi|152993216|ref|YP_001358937.1|	Sulfurovum sp. NBC37-1	E-value = 1.00E-54	Identity = 46.86%
	3		gi|154173968|ref|YP_001408254.1|	Campylobacter curvus 525.92	E-value = 3.00E-50	Identity = 45.93%
	4		gi|154148227|ref|YP_001406478.1|	Campylobacter hominis ATCC BAA-381	E-value = 8.00E-49	Identity = 44.85%
	5		gi|149195423|ref|ZP_01872506.1|	Caminibacter mediatlanticus TB-2	E-value = 2.00E-46	Identity = 50.24%
AB1306	1		gi|152993217|ref|YP_001358938.1|	Sulfurovum sp. NBC37-1	E-value = 8.00E-61	Identity = 55.44%
	2		gi|78777048|ref|YP_393363.1|	Sulfuromonas denitrificans ATCC 33889	E-value = 2.00E-56	Identity = 54.84%
	3		gi|152990791|ref|YP_001356513.1|	Nitratiruptor sp. SB155-2	E-value = 5.00E-56	Identity = 54.92%
	4		gi|57167808|ref|ZP_00366948.1|	Campylobacter coli RM2228	E-value = 1.00E-55	Identity = 52.53%
	5		gi|157415080|ref|YP_001482336.1|	Campylobacter jejuni subsp. jejuni 81116	E-value = 4.00E-53	Identity = 51.01%
AB1307	1		gi|157375319|ref|YP_001473919.1|	Shewanella sediminis HAW-EB3	E-value = 2.00E-18	Identity = 30.96%
AB1308	1		gi|94499383|ref|ZP_01305920.1|	Oceanobacter sp. RED65	E-value = 1.00E-14	Identity = 25.00%
AB1309	1		gi|152992198|ref|YP_001357919.1|	Sulfurovum sp. NBC37-1	E-value = 4.00E-102	Identity = 61.84%
	2		gi|78777046|ref|YP_393361.1|	Sulfuromonas denitrificans ATCC 33889	E-value = 1.00E-99	Identity = 59.41%
	3		gi|152990795|ref|YP_001356517.1|	Nitratiruptor sp. SB155-2	E-value = 6.00E-89	Identity = 56.45%
	4		gi|154173800|ref|YP_001408059.1|	Campylobacter curvus 525.92	E-value = 1.00E-85	Identity = 60.80%
	5		gi|34557081|ref|NP_906896.1|	Wolinella succinogenes DSM 1740	E-value = 1.00E-84	Identity = 59.52%
AB1310	1			***** No matches found *****		
AB1311	1		gi|34558438|ref|NP_908253.1|	Wolinella succinogenes DSM 1740	E-value = 0	Identity = 46.04%
	2		gi|34557356|ref|NP_907171.1|	Wolinella succinogenes DSM 1740	E-value = 0	Identity = 44.08%
	3		gi|34556815|ref|NP_906630.1|	Wolinella succinogenes DSM 1740	E-value = 0	Identity = 43.55%
	4		gi|57241279|ref|ZP_00369226.1|	Campylobacter lari RM2100	E-value = 0	Identity = 42.76%
	5		gi|121612515|ref|YP_999754.1|	Campylobacter jejuni subsp. jejuni 81-176	E-value = 0	Identity = 42.02%
AB1312	1	*pepD*	gi|152990306|ref|YP_001356028.1|	Nitratiruptor sp. SB155-2	E-value = 2.00E-83	Identity = 39.43%
	2		gi|152993391|ref|YP_001359112.1|	Sulfurovum sp. NBC37-1	E-value = 2.00E-83	Identity = 42.92%
	3		gi|154174402|ref|YP_001408064.1|	Campylobacter curvus 525.92	E-value = 3.00E-72	Identity = 38.15%
	4		gi|118474505|ref|YP_892234.1|	Campylobacter fetus subsp. fetus 82-40	E-value = 3.00E-71	Identity = 38.82%

	5		gi|57505466|ref|ZP_00371394.1|	Campylobacter upsaliensis RM3195	E-value = 1.00E-65	Identity = 36.85%
AB1313	1			***** No matches found *****		
AB1314	1		gi|57241710|ref|ZP_00369655.1|	Campylobacter lari RM2100	E-value = 2.00E-45	Identity = 29.17%
	2		gi|154175470|ref|YP_001408196.1|	Campylobacter curvus 525.92	E-value = 1.00E-44	Identity = 27.75%
	3		gi|118474107|ref|YP_892458.1|	Campylobacter fetus subsp. fetus 82-40	E-value = 5.00E-42	Identity = 27.91%
	4		gi|78485122|ref|YP_391047.1|	Thiomicrospira crunogena XCL-2	E-value = 3.00E-40	Identity = 27.72%
	5		gi|15889452|ref|NP_355133.1|	Agrobacterium tumefaciens str. C58	E-value = 5.00E-39	Identity = 25.80%
AB1315	1			***** No matches found *****		
AB1316	1	*lolA*	gi|152990576|ref|YP_001356298.1|	Nitratiruptor sp. SB155-2	E-value = 2.00E-18	Identity = 34.34%
	2		gi|118475445|ref|YP_891765.1|	Campylobacter fetus subsp. fetus 82-40	E-value = 1.00E-17	Identity = 36.84%
	3		gi|32265815|ref|NP_859847.1|	Helicobacter hepaticus ATCC 51449	E-value = 6.00E-17	Identity = 31.79%
	4		gi|34557621|ref|NP_907436.1|	Wolinella succinogenes DSM 1740	E-value = 3.00E-16	Identity = 32.35%
	5		gi|57167892|ref|ZP_00367032.1|	Campylobacter coli RM2228	E-value = 6.00E-16	Identity = 32.56%
AB1317	1	*secA*	gi|154175377|ref|YP_001408406.1|	Campylobacter curvus 525.92	E-value = 0	Identity = 66.24%
	2		gi|157165105|ref|YP_001466674.1|	Campylobacter concisus 13826	E-value = 0	Identity = 66.02%
	3		gi|154147932|ref|YP_001406499.1|	Campylobacter hominis ATCC BAA-381	E-value = 0	Identity = 65.72%
	4		gi|34557622|ref|NP_907437.1|	Wolinella succinogenes DSM 1740	E-value = 0	Identity = 66.13%
	5		gi|118475233|ref|YP_891766.1|	Campylobacter fetus subsp. fetus 82-40	E-value = 0	Identity = 65.17%
AB1318	1		gi|34557623|ref|NP_907438.1|	Wolinella succinogenes DSM 1740	E-value = 2.00E-123	Identity = 56.28%
	2		gi|154148848|ref|YP_001406500.1|	Campylobacter hominis ATCC BAA-381	E-value = 2.00E-121	Identity = 56.42%
	3		gi|154174987|ref|YP_001408405.1|	Campylobacter curvus 525.92	E-value = 6.00E-121	Identity = 57.04%
	4		gi|152990578|ref|YP_001356300.1|	Nitratiruptor sp. SB155-2	E-value = 2.00E-119	Identity = 55.11%
	5		gi|57240551|ref|ZP_00368500.1|	Campylobacter lari RM2100	E-value = 7.00E-118	Identity = 54.61%
AB1319	1			***** No matches found *****		
AB1320	1		gi|118475244|ref|YP_892097.1|	Campylobacter fetus subsp. fetus 82-40	E-value = 3.00E-83	Identity = 50.16%
	2		gi|34557446|ref|NP_907261.1|	Wolinella succinogenes DSM 1740	E-value = 1.00E-81	Identity = 48.04%
	3		gi|78777244|ref|YP_393559.1|	Sulfuromonas denitrificans ATCC 33889	E-value = 2.00E-80	Identity = 45.82%
	4		gi|57167574|ref|ZP_00366714.1|	Campylobacter coli RM2228	E-value = 5.00E-80	Identity = 48.01%
	5		gi|157414810|ref|YP_001482066.1|	Campylobacter jejuni subsp. jejuni 81116	E-value = 1.00E-79	Identity = 48.48%
AB1321	1	*icd*	gi|83309706|ref|YP_419970.1|	Magnetospirillum magneticum AMB-1	E-value = 0	Identity = 69.7%
	2		gi|119470169|ref|ZP_01612935.1|	Alteromonadales bacterium TW-7	E-value = 0	Identity = 66.39%
	3		gi|23016634|ref|ZP_00056388.1|	Magnetospirillum magnetotacticum MS-1	E-value = 0	Identity = 68.75%
	4		gi|110596828|ref|ZP_01385118.1|	Chlorobium ferrooxidans DSM 13031	E-value = 0	Identity = 66.76%
	5		gi|21396513|dbj|BAC00856.1|	Chlorobium limicola	E-value = 0	Identity = 66.49%
AB1322	1	*mdh*	gi|149194469|ref|ZP_01871565.1|	Caminibacter mediatlanticus TB-2	E-value = 3.00E-82	Identity = 48.24%
	2		gi|78777246|ref|YP_393561.1|	Sulfuromonas denitrificans ATCC 33889	E-value = 3.00E-82	Identity = 50.97%
	3		gi|152992167|ref|YP_001357888.1|	Sulfurovum sp. NBC37-1	E-value = 5.00E-81	Identity = 48.72%
	4		gi|152990582|ref|YP_001356304.1|	Nitratiruptor sp. SB155-2	E-value = 3.00E-78	Identity = 48.06%
	5		gi|34557447|ref|NP_907262.1|	Wolinella succinogenes DSM 1740	E-value = 2.00E-74	Identity = 47.40%
AB1323	1		gi|78776777|ref|YP_393092.1|	Sulfuromonas denitrificans ATCC 33889	E-value = 7.00E-12	Identity = 51.06%
	2		gi|152992280|ref|YP_001358001.1|	Sulfurovum sp. NBC37-1	E-value = 1.00E-08	Identity = 50.00%
	3		gi|40890030|pdb|1DWL|B	Desulfovibrio vulgaris	E-value = 7.00E-08	Identity = 41.67%
AB1324	1			***** No matches found *****		
AB1325	1		gi|87118708|ref|ZP_01074607.1|	Marinomonas sp. MED121	E-value = 6.00E-96	Identity = 44.33%
	2		gi|152992771|ref|YP_001358492.1|	Sulfurovum sp. NBC37-1	E-value = 5.00E-77	Identity = 40.79%
	3		gi|85858632|ref|YP_460834.1|	Syntrophus aciditrophicus SB	E-value = 2.00E-70	Identity = 37.05%
	4		gi|116748434|ref|YP_845121.1|	Syntrophobacter fumaroxidans MPOB	E-value = 2.00E-66	Identity = 34.75%
	5		gi|78777293|ref|YP_393608.1|	Sulfuromonas denitrificans ATCC 33889	E-value = 6.00E-61	Identity = 34.68%
AB1326	1		gi|118474753|ref|YP_891756.1|	Campylobacter fetus subsp. fetus 82-40	E-value = 3.00E-06	Identity = 45.95%

AB1327	1	*mraW*	gi|78776996|ref|YP_393311.1|	Sulfuromonas denitrificans ATCC 33889	E-value = 2.00E-93	Identity = 59.14%
	2		gi|152990774|ref|YP_001356496.1|	Nitratiruptor sp. SB155-2	E-value = 3.00E-90	Identity = 56.81%
	3		gi|152992227|ref|YP_001357948.1|	Sulfurovum sp. NBC37-1	E-value = 4.00E-89	Identity = 54.46%
	4		gi|34556618|ref|NP_906433.1|	Wolinella succinogenes DSM 1740	E-value = 6.00E-86	Identity = 54.61%
	5		gi|154174311|ref|YP_001408418.1|	Campylobacter curvus 525.92	E-value = 4.00E-84	Identity = 54.75%
AB1328	1		gi|34556920|ref|NP_906735.1|	Wolinella succinogenes DSM 1740	E-value = 5.00E-48	Identity = 49.47%
	2		gi|152992226|ref|YP_001357947.1|	Sulfurovum sp. NBC37-1	E-value = 4.00E-45	Identity = 45.79%
	3		gi|78777487|ref|YP_393802.1|	Sulfuromonas denitrificans ATCC 33889	E-value = 5.00E-43	Identity = 49.72%
	4		gi|154174628|ref|YP_001408419.1|	Campylobacter curvus 525.92	E-value = 2.00E-40	Identity = 46.81%
	5		gi|118475124|ref|YP_891758.1|	Campylobacter fetus subsp. fetus 82-40	E-value = 2.00E-40	Identity = 48.63%
AB1329	1	*hup*	gi|34556922|ref|NP_906737.1|	Wolinella succinogenes DSM 1740	E-value = 2.00E-17	Identity = 69.23%
	2		gi|152990572|ref|YP_001356294.1|	Nitratiruptor sp. SB155-2	E-value = 1.00E-14	Identity = 66.67%
	3		gi|157164945|ref|YP_001466788.1|	Campylobacter concisus 13826	E-value = 5.00E-14	Identity = 66.67%
	4		gi|58979367|gb|AAW83348.1|	Campylobacter jejuni	E-value = 8.00E-14	Identity = 67.42%
	5		gi|58979591|gb|AAW83460.1|	Campylobacter upsaliensis	E-value = 2.00E-13	Identity = 65.88%
AB1330	1		gi|152993984|ref|YP_001359705.1|	Sulfurovum sp. NBC37-1	E-value = 4.00E-69	Identity = 40.83%
	2		gi|145617646|ref|ZP_01773710.1|	Geobacter bemidjiensis Bem	E-value = 7.00E-67	Identity = 36.05%
	3		gi|124515059|gb|EAY56570.1|	Leptospirillum sp. Group II UBA	E-value = 3.00E-65	Identity = 35.91%
	4		gi|77919419|ref|YP_357234.1|	Pelobacter carbinolicus DSM 2380	E-value = 5.00E-64	Identity = 34.38%
	5		gi|154173928|ref|YP_001409117.1|	Campylobacter curvus 525.92	E-value = 8.00E-63	Identity = 38.65%
AB1331	1			***** No matches found *****		
AB1332	1			***** No matches found *****		
AB1333	1			***** No matches found *****		
AB1334	1			***** No matches found *****		
AB1335	1		gi|84385563|ref|ZP_00988594.1|	Vibrio splendidus 12B01	E-value = 1.00E-63	Identity = 40.88%
	2		gi|149190701|ref|ZP_01868968.1|	Vibrio shilonii AK1	E-value = 5.00E-57	Identity = 38.74%
	3		gi|26991137|ref|NP_746562.1|	Pseudomonas putida KT2440	E-value = 1.00E-37	Identity = 29.26%
	4		gi|66047741|ref|YP_237582.1|	Pseudomonas syringae pv. syringae B728a	E-value = 4.00E-34	Identity = 27.13%
AB1336	1			***** No matches found *****		
AB1337	1			***** No matches found *****		
AB1338	1			***** No matches found *****		
AB1339	1			***** No matches found *****		
AB1340	1			***** No matches found *****		
AB1341	1		gi|52425039|ref|YP_088176.1|	Mannheimia succiniciproducens MBEL55E	E-value = 9.00E-17	Identity = 25.64%
AB1342	1			***** No matches found *****		
AB1343	1			***** No matches found *****		
AB1344	1			***** No matches found *****		
AB1345	1			***** No matches found *****		
AB1346	1			***** No matches found *****		
AB1347	1			***** No matches found *****		
AB1348	1			***** No matches found *****		
AB1349	1			***** No matches found *****		
AB1350	1			***** No matches found *****		
AB1351	1			***** No matches found *****		
AB1352	1		gi|152991425|ref|YP_001357147.1|	Nitratiruptor sp. SB155-2	E-value = 2.00E-17	Identity = 35.85%
AB1353	1			***** No matches found *****		
AB1354	1			***** No matches found *****		
AB1355	1			***** No matches found *****		
AB1356	1			***** No matches found *****		

AB1357	1			***** No matches found *****		
AB1358	1			***** No matches found *****		
AB1359	1		gi|67941489|ref|ZP_00533613.1|	Chlorobium phaeobacteroides BS1	E-value = 3.00E-16	Identity = 35.34%
	2		gi|74316354|ref|YP_314094.1|	Thiobacillus denitrificans ATCC 25259	E-value = 1.00E-15	Identity = 39.23%
	3		gi|82702897|ref|YP_412463.1|	Nitrosospira multiformis ATCC 25196	E-value = 4.00E-14	Identity = 37.69%
	4		gi|88811720|ref|ZP_01126974.1|	Nitrococcus mobilis Nb-231	E-value = 7.00E-13	Identity = 29.69%
	5		gi|118579599|ref|YP_900849.1|	Pelobacter propionicus DSM 2379	E-value = 1.00E-12	Identity = 30.00%
AB1360	1	*norB*	gi|156719025|ref|ZP_02060676.1|	Hydrogenobaculum sp. Y04AAS1	E-value = 0	Identity = 47.26%
	2		gi|138894315|ref|YP_001124768.1|	Geobacillus thermodenitrificans NG80-2	E-value = 5.00E-112	Identity = 33.99%
	3		gi|153894661|ref|ZP_02015245.1|	Halorubrum lacusprofundi ATCC 49239	E-value = 2.00E-111	Identity = 35.51%
	4		gi|126442657|ref|YP_001063055.1|	Burkholderia pseudomallei 668	E-value = 2.00E-110	Identity = 33.69%
	5		gi|100232639|ref|ZP_01333831.1|	Burkholderia pseudomallei 406e	E-value = 3.00E-110	Identity = 33.69%
AB1361	1			***** No matches found *****		
AB1362	1		gi|78777053|ref|YP_393368.1|	Sulfuromonas denitrificans ATCC 33889	E-value = 2.00E-47	Identity = 34.25%
	2		gi|149195049|ref|ZP_01872141.1|	Caminibacter mediatlanticus TB-2	E-value = 1.00E-42	Identity = 36.48%
	3		gi|152993286|ref|YP_001359007.1|	Sulfurovum sp. NBC37-1	E-value = 1.00E-37	Identity = 28.61%
	4		gi|152990482|ref|YP_001356204.1|	Nitratiruptor sp. SB155-2	E-value = 3.00E-29	Identity = 28.72%
AB1363	1		gi|78776250|ref|YP_392565.1|	Sulfuromonas denitrificans ATCC 33889	E-value = 1.00E-25	Identity = 52.43%
AB1364	1		gi|15641095|ref|NP_230727.1|	Vibrio cholerae O1 biovar eltor str. N16961	E-value = 5.00E-21	Identity = 47.86%
	2		gi|116217592|ref|ZP_01483388.1|	Vibrio cholerae RC385	E-value = 3.00E-20	Identity = 47.01%
	3		gi|53717484|ref|YP_105425.1|	Burkholderia mallei ATCC 23344	E-value = 2.00E-19	Identity = 43.97%
	4		gi|83716053|ref|YP_439782.1|	Burkholderia thailandensis E264	E-value = 4.00E-19	Identity = 43.10%
	5		gi|76818754|ref|YP_337554.1|	Burkholderia pseudomallei 1710b	E-value = 7.00E-19	Identity = 43.97%
AB1365	1		gi|78777233|ref|YP_393548.1|	Sulfuromonas denitrificans ATCC 33889	E-value = 1.00E-23	Identity = 35.18%
	2		gi|51245405|ref|YP_065289.1|	Desulfotalea psychrophila LSv54	E-value = 2.00E-23	Identity = 38.97%
	3		gi|121541499|ref|ZP_01673250.1|	Candidatus Desulfococcus oleovorans Hxd3	E-value = 2.00E-16	Identity = 26.64%
	4		gi|90020042|ref|YP_525869.1|	Saccharophagus degradans 2-40	E-value = 2.00E-16	Identity = 27.78%
	5		gi|95930997|ref|ZP_01313726.1|	Desulfuromonas acetoxidans DSM 684	E-value = 9.00E-16	Identity = 28.50%
AB1366	1		gi|78778075|ref|YP_394390.1|	Sulfuromonas denitrificans ATCC 33889	E-value = 2.00E-33	Identity = 32.34%
	2		gi|148265594|ref|YP_001232300.1|	Geobacter uraniumreducens Rf4	E-value = 8.00E-16	Identity = 25.32%
AB1367	1	*cbpA*	gi|32267141|ref|NP_861173.1|	Helicobacter hepaticus ATCC 51449	E-value = 3.00E-87	Identity = 59.93%
	2		gi|78776753|ref|YP_393068.1|	Sulfuromonas denitrificans ATCC 33889	E-value = 1.00E-86	Identity = 63.79%
	3		gi|34557617|ref|NP_907432.1|	Wolinella succinogenes DSM 1740	E-value = 4.00E-86	Identity = 63.14%
	4		gi|152990700|ref|YP_001356422.1|	Nitratiruptor sp. SB155-2	E-value = 1.00E-85	Identity = 62.24%
	5		gi|152993148|ref|YP_001358869.1|	Sulfurovum sp. NBC37-1	E-value = 1.00E-84	Identity = 62.89%
AB1368	1		gi|154173851|ref|YP_001408403.1|	Campylobacter curvus 525.92	E-value = 4.00E-28	Identity = 56.78%
	2		gi|152990701|ref|YP_001356423.1|	Nitratiruptor sp. SB155-2	E-value = 4.00E-28	Identity = 57.63%
	3		gi|34557618|ref|NP_907433.1|	Wolinella succinogenes DSM 1740	E-value = 4.00E-28	Identity = 54.92%
	4		gi|78776754|ref|YP_393069.1|	Sulfuromonas denitrificans ATCC 33889	E-value = 1.00E-27	Identity = 56.91%
	5		gi|152993147|ref|YP_001358868.1|	Sulfurovum sp. NBC37-1	E-value = 2.00E-27	Identity = 57.89%
AB1369	1		gi|118475276|ref|YP_891463.1|	Campylobacter fetus subsp. fetus 82-40	E-value = 1.00E-71	Identity = 48.75%
	2		gi|154149454|ref|YP_001405953.1|	Campylobacter hominis ATCC BAA-381	E-value = 1.00E-59	Identity = 45.48%
	3		gi|146310944|ref|YP_001176018.1|	Enterobacter sp. 638	E-value = 3.00E-57	Identity = 39.53%
	4		gi|56414082|ref|YP_151157.1|	Salmonella enterica subsp. enterica serovar Paratyphi A str. ATCC 9150	E-value = 1.00E-55	Identity = 40.20%
	5		gi|16759739|ref|NP_455356.1|	Salmonella enterica subsp. enterica serovar Typhi str. CT18	E-value = 1.00E-55	Identity = 40.20%
AB1370	1		gi|116253422|ref|YP_769260.1|	Rhizobium leguminosarum bv. viciae 3841	E-value = 3.00E-178	Identity = 54.39%
	2		gi|51595555|ref|YP_069746.1|	Yersinia pseudotuberculosis IP 32953	E-value = 8.00E-176	Identity = 54.47%
	3		gi|86358850|ref|YP_470742.1|	Rhizobium etli CFN 42	E-value = 2.00E-175	Identity = 54.64%
	4		gi|45440812|ref|NP_992351.1|	Yersinia pestis biovar Microtus str. 91001	E-value = 2.00E-175	Identity = 54.47%

	5		gi|37525459|ref|NP_928803.1|	Photorhabdus luminescens subsp. laumondii TTO1	E-value = 4.00E-175	Identity = 54.56%
AB1371	1		gi|118475223|ref|YP_891465.1|	Campylobacter fetus subsp. fetus 82-40	E-value = 5.00E-94	Identity = 59.67%
	2		gi|116253423|ref|YP_769261.1|	Rhizobium leguminosarum bv. viciae 3841	E-value = 2.00E-93	Identity = 49.86%
	3		gi|86358851|ref|YP_470743.1|	Rhizobium etli CFN 42	E-value = 3.00E-92	Identity = 49.86%
	4		gi|110632493|ref|YP_672701.1|	Mesorhizobium sp. BNC1	E-value = 8.00E-88	Identity = 47.81%
	5		gi|83593126|ref|YP_426878.1|	Rhodospirillum rubrum ATCC 11170	E-value = 5.00E-87	Identity = 47.54%
AB1372	1		gi|118474271|ref|YP_891466.1|	Campylobacter fetus subsp. fetus 82-40	E-value = 8.00E-92	Identity = 55.74%
	2		gi|16264618|ref|NP_437410.1|	Sinorhizobium meliloti 1021	E-value = 3.00E-87	Identity = 45.90%
	3		gi|116253424|ref|YP_769262.1|	Rhizobium leguminosarum bv. viciae 3841	E-value = 3.00E-86	Identity = 45.23%
	4		gi|150376829|ref|YP_001313425.1|	Sinorhizobium medicae WSM419	E-value = 7.00E-85	Identity = 45.08%
	5		gi|86358852|ref|YP_470744.1|	Rhizobium etli CFN 42	E-value = 1.00E-84	Identity = 45.90%
AB1373	1		gi|126462873|ref|YP_001043987.1|	Rhodobacter sphaeroides ATCC 17029	E-value = 4.00E-62	Identity = 39.95%
	2		gi|77464028|ref|YP_353532.1|	Rhodobacter sphaeroides 2.4.1	E-value = 5.00E-62	Identity = 39.95%
	3		gi|146278415|ref|YP_001168574.1|	Rhodobacter sphaeroides ATCC 17025	E-value = 2.00E-61	Identity = 42.44%
	4		gi|150376529|ref|YP_001313125.1|	Sinorhizobium medicae WSM419	E-value = 8.00E-59	Identity = 44.19%
	5		gi|86359171|ref|YP_471063.1|	Rhizobium etli CFN 42	E-value = 2.00E-58	Identity = 39.03%
AB1374	1	*brnQ*	gi|145954983|ref|ZP_01803987.1|	Clostridium difficile QCD-32g58	E-value = 5.00E-83	Identity = 43.33%
	2		gi|126698856|ref|YP_001087753.1|	Clostridium difficile 630	E-value = 3.00E-82	Identity = 43.10%
	3		gi|145954984|ref|ZP_01803988.1|	Clostridium difficile QCD-32g58	E-value = 5.00E-79	Identity = 42.76%
	4		gi|126698857|ref|YP_001087754.1|	Clostridium difficile 630	E-value = 6.00E-79	Identity = 42.52%
	5		gi|110800785|ref|YP_694516.1|	Clostridium perfringens ATCC 13124	E-value = 4.00E-71	Identity = 39.14%
AB1376	1		gi|15602475|ref|NP_245547.1|	Pasteurella multocida subsp. multocida str. Pm70	E-value = 3.00E-33	Identity = 39.65%
	2		gi|145636087|ref|ZP_01791757.1|	Haemophilus influenzae PittHH	E-value = 1.00E-32	Identity = 40.07%
	3		gi|68250107|ref|YP_249219.1|	Haemophilus influenzae 86-028NP	E-value = 2.00E-32	Identity = 40.42%
	4		gi|148825914|ref|YP_001290667.1|	Haemophilus influenzae PittEE	E-value = 2.00E-32	Identity = 40.07%
	5		gi|46133444|ref|ZP_00157214.2|	Haemophilus influenzae R2866	E-value = 2.00E-32	Identity = 40.42%
AB1377	1		gi|152990295|ref|YP_001356017.1|	Nitratiruptor sp. SB155-2	E-value = 1.00E-26	Identity = 48.30%
	2		gi|34558425|ref|NP_908240.1|	Wolinella succinogenes DSM 1740	E-value = 3.00E-25	Identity = 42.86%
	3		gi|149195151|ref|ZP_01872242.1|	Caminibacter mediatlanticus TB-2	E-value = 9.00E-24	Identity = 52.38%
	4		gi|86151299|ref|ZP_01069514.1|	Campylobacter jejuni subsp. jejuni 260.94	E-value = 4.00E-23	Identity = 47.55%
	5		gi|15791650|ref|NP_281473.1|	Campylobacter jejuni subsp. jejuni NCTC 11168	E-value = 6.00E-23	Identity = 47.55%
AB1378	1	*carB*	gi|78777664|ref|YP_393979.1|	Sulfuromonas denitrificans ATCC 33889	E-value = 0	Identity = 74.13%
	2		gi|152990294|ref|YP_001356016.1|	Nitratiruptor sp. SB155-2	E-value = 0	Identity = 73.20%
	3		gi|34558426|ref|NP_908241.1|	Wolinella succinogenes DSM 1740	E-value = 0	Identity = 73.46%
	4		gi|149195024|ref|ZP_01872117.1|	Caminibacter mediatlanticus TB-2	E-value = 0	Identity = 73.44%
	5		gi|152992698|ref|YP_001358419.1|	Sulfurovum sp. NBC37-1	E-value = 0	Identity = 72.89%
AB1379	1		gi|118744760|ref|ZP_01592748.1|	Geobacter lovleyi SZ	E-value = 7.00E-83	Identity = 56.98%
	2		gi|118579415|ref|YP_900665.1|	Pelobacter propionicus DSM 2379	E-value = 3.00E-76	Identity = 50.96%
	3		gi|78224046|ref|YP_385793.1|	Geobacter metallireducens GS-15	E-value = 8.00E-74	Identity = 54.89%
	4		gi|39995765|ref|NP_951716.1|	Geobacter sulfurreducens PCA	E-value = 7.00E-69	Identity = 51.50%
	5		gi|15613420|ref|NP_241723.1|	Bacillus halodurans C-125	E-value = 4.00E-66	Identity = 53.18%
AB1380	1	*nhaA1*	gi|34557615|ref|NP_907430.1|	Wolinella succinogenes DSM 1740	E-value = 2.00E-97	Identity = 47.53%
	2		gi|32267144|ref|NP_861176.1|	Helicobacter hepaticus ATCC 51449	E-value = 3.00E-90	Identity = 45.67%
	3		gi|108563926|ref|YP_628242.1|	Helicobacter pylori HPAG1	E-value = 7.00E-88	Identity = 47.97%
	4		gi|15612512|ref|NP_224165.1|	Helicobacter pylori J99	E-value = 3.00E-86	Identity = 47.02%
	5		gi|15646159|ref|NP_208343.1|	Helicobacter pylori 26695	E-value = 1.00E-85	Identity = 47.49%
AB1381	1			***** No matches found *****		
AB1382	1	*metX*	gi|118474380|ref|YP_891789.1|	Campylobacter fetus subsp. fetus 82-40	E-value = 2.00E-131	Identity = 60.93%
	2		gi|157165371|ref|YP_001467502.1|	Campylobacter concisus 13826	E-value = 1.00E-129	Identity = 59.56%

	3		gi|152993373|ref|YP_001359094.1|	Sulfurovum sp. NBC37-1	E-value = 7.00E-128	Identity = 58.74%
	4		gi|152991019|ref|YP_001356741.1|	Nitratiruptor sp. SB155-2	E-value = 8.00E-127	Identity = 60.22%
	5		gi|154148628|ref|YP_001406471.1|	Campylobacter hominis ATCC BAA-381	E-value = 1.00E-125	Identity = 61.06%
AB1383	1		gi|34557008|ref|NP_906823.1|	Wolinella succinogenes DSM 1740	E-value = 1.00E-157	Identity = 44.17%
	2		gi|34557768|ref|NP_907583.1|	Wolinella succinogenes DSM 1740	E-value = 2.00E-140	Identity = 39.75%
	3		gi|119899284|ref|YP_934497.1|	Azoarcus sp. BH72	E-value = 3.00E-137	Identity = 39.01%
	4		gi|21244223|ref|NP_643805.1|	Xanthomonas axonopodis pv. citri str. 306	E-value = 2.00E-126	Identity = 38.32%
	5		gi|154174913|ref|YP_001408304.1|	Campylobacter curvus 525.92	E-value = 7.00E-124	Identity = 40.15%
AB1384	1			***** No matches found *****		
AB1385	1	*ftsZ*	gi|34556673|ref|NP_906488.1|	Wolinella succinogenes DSM 1740	E-value = 3.00E-82	Identity = 56.97%
	2		gi|154174794|ref|YP_001408422.1|	Campylobacter curvus 525.92	E-value = 2.00E-81	Identity = 53.85%
	3		gi|152990778|ref|YP_001356500.1|	Nitratiruptor sp. SB155-2	E-value = 2.00E-80	Identity = 54.65%
	4		gi|57242102|ref|ZP_00370042.1|	Campylobacter upsaliensis RM3195	E-value = 1.00E-78	Identity = 56.92%
	5		gi|152992210|ref|YP_001357931.1|	Sulfurovum sp. NBC37-1	E-value = 5.00E-78	Identity = 59.05%
AB1386	1	*ftsA*	gi|34556674|ref|NP_906489.1|	Wolinella succinogenes DSM 1740	E-value = 1.00E-80	Identity = 42.86%
	2		gi|154174212|ref|YP_001408421.1|	Campylobacter curvus 525.92	E-value = 5.00E-74	Identity = 39.55%
	3		gi|157165758|ref|YP_001466660.1|	Campylobacter concisus 13826	E-value = 2.00E-73	Identity = 40.85%
	4		gi|152990777|ref|YP_001356499.1|	Nitratiruptor sp. SB155-2	E-value = 2.00E-72	Identity = 39.35%
	5		gi|152992211|ref|YP_001357932.1|	Sulfurovum sp. NBC37-1	E-value = 2.00E-72	Identity = 40.25%
AB1387	1		gi|152990776|ref|YP_001356498.1|	Nitratiruptor sp. SB155-2	E-value = 7.00E-77	Identity = 37.94%
	2		gi|157164574|ref|YP_001466661.1|	Campylobacter concisus 13826	E-value = 2.00E-73	Identity = 34.09%
	3		gi|118475291|ref|YP_891759.1|	Campylobacter fetus subsp. fetus 82-40	E-value = 1.00E-70	Identity = 36.01%
	4		gi|154175271|ref|YP_001408420.1|	Campylobacter curvus 525.92	E-value = 6.00E-70	Identity = 33.06%
	5		gi|78777490|ref|YP_393805.1|	Sulfuromonas denitrificans ATCC 33889	E-value = 1.00E-68	Identity = 36.68%
AB1388	1	*recD*	gi|78778046|ref|YP_394361.1|	Sulfuromonas denitrificans ATCC 33889	E-value = 0	Identity = 47.73%
	2		gi|118577405|ref|YP_899645.1|	Pelobacter propionicus DSM 2379	E-value = 1.00E-112	Identity = 35.94%
	3		gi|46446323|ref|YP_007688.1|	Candidatus Protochlamydia amoebophila UWE25	E-value = 2.00E-74	Identity = 28.86%
	4		gi|68553218|ref|ZP_00592597.1|	Prosthecochloris aestuarii DSM 271	E-value = 3.00E-74	Identity = 29.00%
	5		gi|125974734|ref|YP_001038644.1|	Clostridium thermocellum ATCC 27405	E-value = 1.00E-72	Identity = 29.77%
AB1389	1		gi|154149139|ref|YP_001406454.1|	Campylobacter hominis ATCC BAA-381	E-value = 5.00E-06	Identity = 42.20%
AB1391	1		gi|18645103|gb|AAL76404.1|	uncultured proteobacterium	E-value = 7.00E-24	Identity = 53.04%
	2		gi|119504070|ref|ZP_01626151.1|	marine gamma proteobacterium HTCC2080	E-value = 1.00E-23	Identity = 52.17%
	3		gi|146308011|ref|YP_001188476.1|	Pseudomonas mendocina ymp	E-value = 1.00E-23	Identity = 54.31%
	4		gi|78776350|ref|YP_392665.1|	Sulfuromonas denitrificans ATCC 33889	E-value = 2.00E-23	Identity = 53.45%
	5		gi|40063507|gb|AAR38307.1|	uncultured bacterium 581	E-value = 2.00E-23	Identity = 52.17%
AB1392	1			***** No matches found *****		
AB1393	1		gi|57505939|ref|ZP_00371863.1|	Campylobacter upsaliensis RM3195	E-value = 3.00E-12	Identity = 48.91%
	2		gi|78777011|ref|YP_393326.1|	Sulfuromonas denitrificans ATCC 33889	E-value = 3.00E-07	Identity = 39.78%
AB1394	1		gi|57505940|ref|ZP_00371864.1|	Campylobacter upsaliensis RM3195	E-value = 1.00E-07	Identity = 47.14%
AB1395	1		gi|57241050|ref|ZP_00368997.1|	Campylobacter lari RM2100	E-value = 1.00E-14	Identity = 25.59%
	2		gi|57237690|ref|YP_178938.1|	Campylobacter jejuni subsp. jejuni RM1221	E-value = 5.00E-11	Identity = 25.56%
	3		gi|86150273|ref|ZP_01068500.1|	Campylobacter jejuni subsp. jejuni CF93-6	E-value = 1.00E-10	Identity = 25.69%
	4		gi|15792187|ref|NP_282010.1|	Campylobacter jejuni subsp. jejuni NCTC 11168	E-value = 1.00E-10	Identity = 25.69%
	5		gi|86152182|ref|ZP_01070394.1|	Campylobacter jejuni subsp. jejuni 260.94	E-value = 2.00E-10	Identity = 25.75%
AB1396	1		gi|78776339|ref|YP_392654.1|	Sulfuromonas denitrificans ATCC 33889	E-value = 5.00E-18	Identity = 59.76%
	2		gi|15792186|ref|NP_282009.1|	Campylobacter jejuni subsp. jejuni NCTC 11168	E-value = 9.00E-18	Identity = 59.09%
	3		gi|57167848|ref|ZP_00366988.1|	Campylobacter coli RM2228	E-value = 1.00E-17	Identity = 59.76%
	4		gi|153952619|ref|YP_001398106.1|	Campylobacter jejuni subsp. doylei 269.97	E-value = 2.00E-17	Identity = 59.09%
	5		gi|57505499|ref|ZP_00371427.1|	Campylobacter upsaliensis RM3195	E-value = 4.00E-16	Identity = 59.30%

AB1397	1		gi|78776344|ref|YP_392659.1|	Sulfuromonas denitrificans ATCC 33889	E-value = 0	Identity = 61.97%
	2		gi|152993399|ref|YP_001359120.1|	Sulfurovum sp. NBC37-1	E-value = 2.00E-176	Identity = 60.33%
	3		gi|121542511|ref|ZP_01674237.1|	Candidatus Desulfococcus oleovorans Hxd3	E-value = 2.00E-142	Identity = 46.79%
	4		gi|116327458|ref|YP_797178.1|	Leptospira borgpetersenii serovar Hardjo-bovis L550	E-value = 2.00E-136	Identity = 51.51%
	5		gi|24215872|ref|NP_713353.1|	Leptospira interrogans serovar Lai str. 56601	E-value = 2.00E-136	Identity = 52.42%
AB1398	1		gi|117621019|ref|YP_858283.1|	Aeromonas hydrophila subsp. hydrophila ATCC 7966	E-value = 2.00E-44	Identity = 53.25%
	2		gi|145297538|ref|YP_001140379.1|	Aeromonas salmonicida subsp. salmonicida A449	E-value = 3.00E-44	Identity = 53.25%
	3		gi|145297526|ref|YP_001140367.1|	Aeromonas salmonicida subsp. salmonicida A449	E-value = 1.00E-43	Identity = 52.66%
	4		gi|91206311|ref|YP_538665.1|	Escherichia coli UTI89	E-value = 1.00E-42	Identity = 53.85%
	5		gi|149276822|ref|ZP_01882965.1|	Pedobacter sp. BAL39	E-value = 2.00E-42	Identity = 53.33%
AB1399	1	*dapD*	gi|152993174|ref|YP_001358895.1|	Sulfurovum sp. NBC37-1	E-value = 1.00E-142	Identity = 65.23%
	2		gi|78777291|ref|YP_393606.1|	Sulfuromonas denitrificans ATCC 33889	E-value = 7.00E-140	Identity = 61.83%
	3		gi|149194807|ref|ZP_01871901.1|	Caminibacter mediatlanticus TB-2	E-value = 3.00E-136	Identity = 61.89%
	4		gi|152991384|ref|YP_001357106.1|	Nitratiruptor sp. SB155-2	E-value = 4.00E-136	Identity = 61.71%
	5		gi|57504883|ref|ZP_00370858.1|	Campylobacter coli RM2228	E-value = 5.00E-116	Identity = 54.96%
AB1400	1		gi|153954815|ref|YP_001395580.1|	Clostridium kluyveri DSM 555	E-value = 0	Identity = 54.02%
	2		gi|91776082|ref|YP_545838.1|	Methylobacillus flagellatus KT	E-value = 0	Identity = 50.99%
	3		gi|126356759|ref|ZP_01713763.1|	Pseudomonas putida GB-1	E-value = 8.00E-179	Identity = 44.97%
	4		gi|119857090|ref|ZP_01638520.1|	Pseudomonas putida W619	E-value = 9.00E-178	Identity = 44.99%
	5		gi|26988777|ref|NP_744202.1|	Pseudomonas putida KT2440	E-value = 5.00E-175	Identity = 44.19%
AB1401	1			***** No matches found *****		
AB1403	1	*priA*	gi|152990085|ref|YP_001355807.1|	Nitratiruptor sp. SB155-2	E-value = 3.00E-167	Identity = 46.74%
	2		gi|78776569|ref|YP_392884.1|	Sulfuromonas denitrificans ATCC 33889	E-value = 3.00E-160	Identity = 46.67%
	3		gi|154175169|ref|YP_001408495.1|	Campylobacter curvus 525.92	E-value = 4.00E-153	Identity = 43.96%
	4		gi|57236987|ref|YP_178788.1|	Campylobacter jejuni subsp. jejuni RM1221	E-value = 7.00E-151	Identity = 46.00%
	5		gi|15792033|ref|NP_281856.1|	Campylobacter jejuni subsp. jejuni NCTC 11168	E-value = 9.00E-151	Identity = 46.17%
AB1404	1			***** No matches found *****		
AB1405	1			***** No matches found *****		
AB1406	1			***** No matches found *****		
AB1407	1			***** No matches found *****		
AB1408	1	*hypA*	gi|78777621|ref|YP_393936.1|	Sulfuromonas denitrificans ATCC 33889	E-value = 2.00E-32	Identity = 65.49%
	2		gi|149194372|ref|ZP_01871469.1|	Caminibacter mediatlanticus TB-2	E-value = 1.00E-30	Identity = 61.06%
	3		gi|113949958|ref|ZP_01435598.1|	Shewanella baltica OS195	E-value = 2.00E-28	Identity = 59.29%
	4		gi|149116397|ref|ZP_01843123.1|	Shewanella baltica OS223	E-value = 5.00E-28	Identity = 58.41%
	5		gi|126174105|ref|YP_001050254.1|	Shewanella baltica OS155	E-value = 9.00E-28	Identity = 58.41%
AB1409	1	*hypE*	gi|78777623|ref|YP_393938.1|	Sulfuromonas denitrificans ATCC 33889	E-value = 2.00E-145	Identity = 77.11%
	2		gi|152993232|ref|YP_001358953.1|	Sulfurovum sp. NBC37-1	E-value = 2.00E-130	Identity = 69.88%
	3		gi|90578808|ref|ZP_01234618.1|	Vibrio angustum S14	E-value = 2.00E-109	Identity = 60.84%
	4		gi|114563283|ref|YP_750796.1|	Shewanella frigidimarina NCIMB 400	E-value = 5.00E-109	Identity = 58.01%
	5		gi|118756768|ref|ZP_01604540.1|	Shewanella pealeana ATCC 700345	E-value = 6.00E-108	Identity = 58.01%
AB1410	1		gi|90411066|ref|ZP_01219079.1|	Photobacterium profundum 3TCK	E-value = 6.00E-25	Identity = 49.11%
	2		gi|54303177|ref|YP_133170.1|	Photobacterium profundum SS9	E-value = 9.00E-25	Identity = 49.11%
	3		gi|119859817|ref|ZP_01641227.1|	Pseudomonas putida W619	E-value = 2.00E-23	Identity = 43.48%
	4		gi|115423581|emb|CAJ50117.1|	Bordetella avium 197N	E-value = 4.00E-22	Identity = 45.13%
	5		gi|89075725|ref|ZP_01162120.1|	Photobacterium sp. SKA34	E-value = 6.00E-22	Identity = 44.64%
AB1411	1		gi|150016193|ref|YP_001308447.1|	Clostridium beijerinckii NCIMB 8052	E-value = 1.00E-89	Identity = 60.84%
	2		gi|110800693|ref|YP_694838.1|	Clostridium perfringens ATCC 13124	E-value = 3.00E-87	Identity = 60.35%
	3		gi|18309375|ref|NP_561309.1|	Clostridium perfringens str. 13	E-value = 5.00E-86	Identity = 59.65%
	4		gi|153814319|ref|ZP_01966987.1|	Ruminococcus torques ATCC 27756	E-value = 7.00E-85	Identity = 55.94%

	5		gi|153854863|ref|ZP_01996086.1|	Dorea longicatena DSM 13814	E-value = 2.00E-83	Identity = 57.34%
AB1412	1		gi|66046953|ref|YP_236794.1|	Pseudomonas syringae pv. syringae B728a	E-value = 3.00E-43	Identity = 34.34%
	2		gi|28868859|ref|NP_791478.1|	Pseudomonas syringae pv. tomato str. DC3000	E-value = 4.00E-43	Identity = 32.33%
	3		gi|124004104|ref|ZP_01688951.1|	Microscilla marina ATCC 23134	E-value = 2.00E-40	Identity = 36.67%
	4		gi|34540720|ref|NP_905199.1|	Porphyromonas gingivalis W83	E-value = 9.00E-37	Identity = 31.44%
	5		gi|86149519|ref|ZP_01067749.1|	Campylobacter jejuni subsp. jejuni CF93-6	E-value = 8.00E-29	Identity = 30.83%
AB1413	1		gi|86748716|ref|YP_485212.1|	Rhodopseudomonas palustris HaA2	E-value = 5.00E-25	Identity = 27.84%
	2		gi|156449125|ref|ZP_02055508.1|	Methylobacterium chloromethanicum CM4	E-value = 3.00E-24	Identity = 26.43%
	3		gi|77974093|ref|ZP_00829636.1|	Yersinia frederiksenii ATCC 33641	E-value = 4.00E-24	Identity = 27.08%
	4		gi|118443173|ref|YP_877507.1|	Clostridium novyi NT	E-value = 2.00E-22	Identity = 29.16%
	5		gi|16120722|ref|NP_404035.1|	Yersinia pestis CO92	E-value = 2.00E-22	Identity = 26.49%
AB1414	1	*hypD*	gi|152993233|ref|YP_001358954.1|	Sulfurovum sp. NBC37-1	E-value = 4.00E-154	Identity = 68.60%
	2		gi|78777625|ref|YP_393940.1|	Sulfuromonas denitrificans ATCC 33889	E-value = 8.00E-154	Identity = 69.13%
	3		gi|152990721|ref|YP_001356443.1|	Nitratiruptor sp. SB155-2	E-value = 2.00E-139	Identity = 63.76%
	4		gi|34557191|ref|NP_907006.1|	Wolinella succinogenes DSM 1740	E-value = 1.00E-129	Identity = 59.23%
	5		gi|32265821|ref|NP_859853.1|	Helicobacter hepaticus ATCC 51449	E-value = 5.00E-127	Identity = 56.38%
AB1415	1	*hypC*	gi|152990720|ref|YP_001356442.1|	Nitratiruptor sp. SB155-2	E-value = 8.00E-23	Identity = 63.74%
	2		gi|78777626|ref|YP_393941.1|	Sulfuromonas denitrificans ATCC 33889	E-value = 3.00E-20	Identity = 59.57%
	3		gi|78486381|ref|YP_392306.1|	Thiomicrospira crunogena XCL-2	E-value = 2.00E-17	Identity = 62.82%
	4		gi|118475307|ref|YP_892102.1|	Campylobacter fetus subsp. fetus 82-40	E-value = 3.00E-17	Identity = 58.23%
	5		gi|154148697|ref|YP_001406436.1|	Campylobacter hominis ATCC BAA-381	E-value = 7.00E-17	Identity = 61.33%
AB1416	1	*hypB*	gi|152993235|ref|YP_001358956.1|	Sulfurovum sp. NBC37-1	E-value = 7.00E-99	Identity = 73.99%
	2		gi|78777627|ref|YP_393942.1|	Sulfuromonas denitrificans ATCC 33889	E-value = 1.00E-94	Identity = 72.16%
	3		gi|152990719|ref|YP_001356441.1|	Nitratiruptor sp. SB155-2	E-value = 1.00E-92	Identity = 68.13%
	4		gi|78486382|ref|YP_392307.1|	Thiomicrospira crunogena XCL-2	E-value = 4.00E-86	Identity = 70.40%
	5		gi|90578811|ref|ZP_01234621.1|	Vibrio angustum S14	E-value = 3.00E-85	Identity = 62.92%
AB1417	1		gi|152990717|ref|YP_001356439.1|	Nitratiruptor sp. SB155-2	E-value = 7.00E-22	Identity = 37.38%
	2		gi|152993237|ref|YP_001358958.1|	Sulfurovum sp. NBC37-1	E-value = 2.00E-18	Identity = 28.57%
	3		gi|78776259|ref|YP_392574.1|	Sulfuromonas denitrificans ATCC 33889	E-value = 1.00E-16	Identity = 31.46%
	4		gi|149194392|ref|ZP_01871489.1|	Caminibacter mediatlanticus TB-2	E-value = 1.00E-10	Identity = 33.50%
	5		gi|31335179|gb|AAP44503.1|	Neisseria meningitidis	E-value = 2.00E-06	Identity = 25.71%
AB1418	1			***** No matches found *****		
AB1419	1		gi|154686498|ref|YP_001421659.1|	Bacillus amyloliquefaciens FZB42	E-value = 6.00E-34	Identity = 30.58%
	2		gi|15894135|ref|NP_347484.1|	Clostridium acetobutylicum ATCC 824	E-value = 5.00E-33	Identity = 33.70%
	3		gi|78042996|ref|YP_359036.1|	Carboxydothermus hydrogenoformans Z-2901	E-value = 2.00E-30	Identity = 31.25%
	4		gi|149183114|ref|ZP_01861565.1|	Bacillus sp. SG-1	E-value = 4.00E-30	Identity = 30.51%
	5		gi|138895765|ref|YP_001126218.1|	Geobacillus thermodenitrificans NG80-2	E-value = 4.00E-30	Identity = 28.78%
AB1420	1		gi|78777435|ref|YP_393750.1|	Sulfuromonas denitrificans ATCC 33889	E-value = 9.00E-136	Identity = 61.22%
	2		gi|152992758|ref|YP_001358479.1|	Sulfurovum sp. NBC37-1	E-value = 2.00E-134	Identity = 56.16%
	3		gi|91201475|emb|CAJ74535.1|	Candidatus Kuenenia stuttgartiensis	E-value = 2.00E-63	Identity = 35.61%
	4		gi|150383881|ref|ZP_01922593.1|	Victivallis vadensis ATCC BAA-548	E-value = 7.00E-63	Identity = 35.29%
	5		gi|126180327|ref|YP_001048292.1|	Methanoculleus marisnigri JR1	E-value = 2.00E-59	Identity = 34.87%
AB1421	1		gi|78776631|ref|YP_392946.1|	Sulfuromonas denitrificans ATCC 33889	E-value = 1.00E-131	Identity = 70.77%
	2		gi|157369598|ref|YP_001477587.1|	Serratia proteamaculans 568	E-value = 8.00E-51	Identity = 36.36%
	3		gi|153091046|gb|EDN73051.1|	Mannheimia haemolytica PHL213	E-value = 4.00E-49	Identity = 37.50%
	4		gi|50085910|ref|YP_047420.1|	Acinetobacter sp. ADP1	E-value = 1.00E-48	Identity = 35.31%
	5		gi|46143772|ref|ZP_00134234.2|	Actinobacillus pleuropneumoniae serovar 1 str. 4074	E-value = 4.00E-48	Identity = 38.87%
AB1422	1			***** No matches found *****		
AB1423	1			***** No matches found *****		

AB1424	1		gi|95930514|ref|ZP_01313249.1|	Desulfuromonas acetoxidans DSM 684	E-value = 1.00E-19	Identity = 32.54%
	2		gi|78776247|ref|YP_392562.1|	Sulfuromonas denitrificans ATCC 33889	E-value = 6.00E-19	Identity = 31.63%
	3		gi|152995597|ref|YP_001340432.1|	Marinomonas sp. MWYL1	E-value = 6.00E-11	Identity = 25.56%
	4		gi|114561837|ref|YP_749350.1|	Shewanella frigidimarina NCIMB 400	E-value = 1.00E-10	Identity = 25.93%
	5		gi|88796766|ref|ZP_01112417.1|	Alteromonas macleodii 'Deep ecotype'	E-value = 3.00E-10	Identity = 25.78%
AB1425	1		gi|78777459|ref|YP_393774.1|	Sulfuromonas denitrificans ATCC 33889	E-value = 9.00E-15	Identity = 53.57%
	2		gi|149197841|ref|ZP_01874890.1|	Lentisphaera araneosa HTCC2155	E-value = 1.00E-12	Identity = 46.51%
	3		gi|16077491|ref|NP_388305.1|	Bacillus subtilis subsp. subtilis str. 168	E-value = 1.00E-11	Identity = 53.25%
	4		gi|49481297|ref|YP_039388.1|	Bacillus thuringiensis serovar konkukian str. 97-27	E-value = 2.00E-11	Identity = 54.55%
	5		gi|126645126|ref|ZP_01717670.1|	Algoriphagus sp. PR1	E-value = 3.00E-11	Identity = 42.53%
AB1426	1			***** No matches found *****		
AB1427	1	*thiI*	gi|152993069|ref|YP_001358790.1|	Sulfurovum sp. NBC37-1	E-value = 0	Identity = 64.34%
	2		gi|78485834|ref|YP_391759.1|	Thiomicrospira crunogena XCL-2	E-value = 1.00E-154	Identity = 58.71%
	3		gi|94499692|ref|ZP_01306229.1|	Oceanobacter sp. RED65	E-value = 9.00E-117	Identity = 47.22%
	4		gi|89092122|ref|ZP_01165077.1|	Oceanospirillum sp. MED92	E-value = 1.00E-116	Identity = 45.59%
	5		gi|119856413|ref|ZP_01637846.1|	Pseudomonas putida W619	E-value = 1.00E-116	Identity = 45.40%
AB1428	1			***** No matches found *****		
AB1429	1			***** No matches found *****		
AB1430	1		gi|82703408|ref|YP_412974.1|	Nitrosospira multiformis ATCC 25196	E-value = 4.00E-123	Identity = 57.14%
	2		gi|114331308|ref|YP_747530.1|	Nitrosomonas eutropha C91	E-value = 1.00E-118	Identity = 56.43%
	3		gi|89901851|ref|YP_524322.1|	Rhodoferax ferrireducens T118	E-value = 3.00E-118	Identity = 55.07%
	4		gi|30249211|ref|NP_841281.1|	Nitrosomonas europaea ATCC 19718	E-value = 5.00E-118	Identity = 55.85%
	5		gi|119898591|ref|YP_933804.1|	Azoarcus sp. BH72	E-value = 1.00E-117	Identity = 52.98%
AB1431	1	*hypF*	gi|152993244|ref|YP_001358965.1|	Sulfurovum sp. NBC37-1	E-value = 0	Identity = 48.45%
	2		gi|78777628|ref|YP_393943.1|	Sulfuromonas denitrificans ATCC 33889	E-value = 0	Identity = 50.07%
	3		gi|152061261|dbj|BAF73680.1|	Hydrogenobacter thermophilus	E-value = 0	Identity = 42.65%
	4		gi|15606085|ref|NP_213462.1|	Aquifex aeolicus VF5	E-value = 0	Identity = 42.91%
	5		gi|34557188|ref|NP_907003.1|	Wolinella succinogenes DSM 1740	E-value = 0	Identity = 44.94%
AB1432	1		gi|149194380|ref|ZP_01871477.1|	Caminibacter mediatlanticus TB-2	E-value = 3.00E-49	Identity = 30.38%
	2		gi|78777629|ref|YP_393944.1|	Sulfuromonas denitrificans ATCC 33889	E-value = 1.00E-43	Identity = 30.58%
	3		gi|118475232|ref|YP_892106.1|	Campylobacter fetus subsp. fetus 82-40	E-value = 1.00E-31	Identity = 26.75%
	4		gi|154148724|ref|YP_001406429.1|	Campylobacter hominis ATCC BAA-381	E-value = 2.00E-27	Identity = 25.28%
	5		gi|154174343|ref|YP_001408292.1|	Campylobacter curvus 525.92	E-value = 4.00E-23	Identity = 25.32%
AB1433	1	*hydD*	gi|78777630|ref|YP_393945.1|	Sulfuromonas denitrificans ATCC 33889	E-value = 7.00E-46	Identity = 50.56%
	2		gi|149194379|ref|ZP_01871476.1|	Caminibacter mediatlanticus TB-2	E-value = 2.00E-44	Identity = 53.07%
	3		gi|34557996|ref|NP_907811.1|	Wolinella succinogenes DSM 1740	E-value = 4.00E-43	Identity = 51.98%
	4		gi|32265558|ref|NP_859590.1|	Helicobacter hepaticus ATCC 51449	E-value = 6.00E-43	Identity = 49.15%
	5		gi|118474372|ref|YP_892107.1|	Campylobacter fetus subsp. fetus 82-40	E-value = 3.00E-42	Identity = 50.58%
AB1434	1	*hydC*	gi|154173991|ref|YP_001408294.1|	Campylobacter curvus 525.92	E-value = 2.00E-60	Identity = 58.49%
	2		gi|57167623|ref|ZP_00366763.1|	Campylobacter coli RM2228	E-value = 3.00E-60	Identity = 55.35%
	3		gi|118474674|ref|YP_892108.1|	Campylobacter fetus subsp. fetus 82-40	E-value = 4.00E-60	Identity = 56.88%
	4		gi|154148495|ref|YP_001406427.1|	Campylobacter hominis ATCC BAA-381	E-value = 2.00E-59	Identity = 56.60%
	5		gi|57241184|ref|ZP_00369131.1|	Campylobacter lari RM2100	E-value = 6.00E-58	Identity = 57.21%
AB1435	1	*hydB*	gi|1333732|emb|CAA46303.1|	Wolinella succinogenes	E-value = 0	Identity = 68.66%
	2		gi|34557998|ref|NP_907813.1|	Wolinella succinogenes DSM 1740	E-value = 0	Identity = 68.66%
	3		gi|78777632|ref|YP_393947.1|	Sulfuromonas denitrificans ATCC 33889	E-value = 0	Identity = 68.36%
	4		gi|32265556|ref|NP_859588.1|	Helicobacter hepaticus ATCC 51449	E-value = 0	Identity = 69.88%
	5		gi|118475447|ref|YP_892109.1|	Campylobacter fetus subsp. fetus 82-40	E-value = 0	Identity = 66.14%
AB1436	1	*hydA*	gi|15792591|ref|NP_282414.1|	Campylobacter jejuni subsp. jejuni NCTC 11168	E-value = 7.00E-179	Identity = 76.92%

	2		gi|86151084|ref|ZP_01069300.1|	Campylobacter jejuni subsp. jejuni 260.94	E-value = 1.00E-178	Identity = 76.66%
	3		gi|157415531|ref|YP_001482787.1|	Campylobacter jejuni subsp. jejuni 81116	E-value = 2.00E-178	Identity = 76.66%
	4		gi|86153079|ref|ZP_01071284.1|	Campylobacter jejuni subsp. jejuni HB93-13	E-value = 2.00E-178	Identity = 76.66%
	5		gi|118474859|ref|YP_892110.1|	Campylobacter fetus subsp. fetus 82-40	E-value = 5.00E-178	Identity = 73.63%
AB1437	1		gi|34557007|ref|NP_906822.1|	Wolinella succinogenes DSM 1740	E-value = 1.00E-33	Identity = 30.34%
	2		gi|34557769|ref|NP_907584.1|	Wolinella succinogenes DSM 1740	E-value = 2.00E-31	Identity = 28.97%
	3		gi|84328238|ref|ZP_00976245.1|	Pseudomonas aeruginosa 2192	E-value = 1.00E-26	Identity = 26.69%
	4		gi|107100752|ref|ZP_01364670.1|	Pseudomonas aeruginosa PACS2	E-value = 3.00E-26	Identity = 26.69%
	5		gi|15596498|ref|NP_249992.1|	Pseudomonas aeruginosa PAO1	E-value = 4.00E-26	Identity = 26.69%
AB1438	1		gi|34557006|ref|NP_906821.1|	Wolinella succinogenes DSM 1740	E-value = 6.00E-16	Identity = 31.41%
	2		gi|70731436|ref|YP_261177.1|	Pseudomonas fluorescens Pf-5	E-value = 1.00E-14	Identity = 31.03%
	3		gi|30248569|ref|NP_840639.1|	Nitrosomonas europaea ATCC 19718	E-value = 1.00E-13	Identity = 31.17%
	4		gi|34557770|ref|NP_907585.1|	Wolinella succinogenes DSM 1740	E-value = 5.00E-13	Identity = 33.77%
	5		gi|30249178|ref|NP_841248.1|	Nitrosomonas europaea ATCC 19718	E-value = 4.00E-12	Identity = 26.39%
AB1439	1		gi|149194380|ref|ZP_01871477.1|	Caminibacter mediatlanticus TB-2	E-value = 2.00E-20	Identity = 26.89%
	2		gi|57242240|ref|ZP_00370179.1|	Campylobacter upsaliensis RM3195	E-value = 2.00E-09	Identity = 26.27%
AB1440	1	*hyaD*	gi|152990711|ref|YP_001356433.1|	Nitratiruptor sp. SB155-2	E-value = 5.00E-47	Identity = 62.28%
	2		gi|78486375|ref|YP_392300.1|	Thiomicrospira crunogena XCL-2	E-value = 5.00E-33	Identity = 42.77%
	3		gi|152993246|ref|YP_001358967.1|	Sulfurovum sp. NBC37-1	E-value = 8.00E-25	Identity = 40.35%
	4		gi|152993228|ref|YP_001358949.1|	Sulfurovum sp. NBC37-1	E-value = 4.00E-21	Identity = 36.05%
	5		gi|116750373|ref|YP_847060.1|	Syntrophobacter fumaroxidans MPOB	E-value = 4.00E-19	Identity = 33.13%
AB1441	1	*hyaC*	gi|152990710|ref|YP_001356432.1|	Nitratiruptor sp. SB155-2	E-value = 1.00E-78	Identity = 67.70%
	2		gi|152993247|ref|YP_001358968.1|	Sulfurovum sp. NBC37-1	E-value = 2.00E-77	Identity = 67.22%
	3		gi|126732740|ref|ZP_01748535.1|	Sagittula stellata E-37	E-value = 3.00E-12	Identity = 27.80%
	4		gi|154246118|ref|YP_001417076.1|	Xanthobacter autotrophicus Py2	E-value = 4.00E-12	Identity = 29.52%
	5		gi|71909597|ref|YP_287184.1|	Dechloromonas aromatica RCB	E-value = 8.00E-12	Identity = 28.51%
AB1442	1	*hyaB*	gi|152990709|ref|YP_001356431.1|	Nitratiruptor sp. SB155-2	E-value = 0	Identity = 74.48%
	2		gi|152993248|ref|YP_001358969.1|	Sulfurovum sp. NBC37-1	E-value = 0	Identity = 72.04%
	3		gi|74356071|dbj|BAE44407.1|	Sulfurimonas paralvinella	E-value = 0	Identity = 73.59%
	4		gi|74356068|dbj|BAE44405.1|	Nitratifractor salsuginis	E-value = 0	Identity = 70.41%
	5		gi|74356062|dbj|BAE44401.1|	Hydrogenimonas thermophila	E-value = 0	Identity = 71.09%
AB1443	1	*hyaA*	gi|152990708|ref|YP_001356430.1|	Nitratiruptor sp. SB155-2	E-value = 0	Identity = 76.24%
	2		gi|152993249|ref|YP_001358970.1|	Sulfurovum sp. NBC37-1	E-value = 0	Identity = 76.96%
	3		gi|149194554|ref|ZP_01871650.1|	Caminibacter mediatlanticus TB-2	E-value = 1.00E-117	Identity = 57.69%
	4		gi|117920325|ref|YP_869517.1|	Shewanella sp. ANA-3	E-value = 6.00E-114	Identity = 51.73%
	5		gi|126174115|ref|YP_001050264.1|	Shewanella baltica OS155	E-value = 2.00E-113	Identity = 51.73%
AB1444	1	*hupL*	gi|152061264|dbj|BAF73683.1|	Hydrogenobacter thermophilus	E-value = 1.00E-97	Identity = 43.68%
	2		gi|152993250|ref|YP_001358971.1|	Sulfurovum sp. NBC37-1	E-value = 2.00E-93	Identity = 40.00%
	3		gi|78777634|ref|YP_393949.1|	Sulfuromonas denitrificans ATCC 33889	E-value = 1.00E-88	Identity = 40.31%
	4		gi|15606172|ref|NP_213549.1|	Aquifex aeolicus VF5	E-value = 1.00E-77	Identity = 38.51%
	5		gi|156718554|ref|ZP_02060218.1|	Hydrogenobaculum sp. Y04AAS1	E-value = 5.00E-72	Identity = 35.18%
AB1445	1	*hupS*	gi|156718553|ref|ZP_02060217.1|	Hydrogenobaculum sp. Y04AAS1	E-value = 7.00E-78	Identity = 49.01%
	2		gi|78777635|ref|YP_393950.1|	Sulfuromonas denitrificans ATCC 33889	E-value = 7.00E-76	Identity = 46.80%
	3		gi|152993251|ref|YP_001358972.1|	Sulfurovum sp. NBC37-1	E-value = 2.00E-73	Identity = 46.03%
	4		gi|152990706|ref|YP_001356428.1|	Nitratiruptor sp. SB155-2	E-value = 5.00E-71	Identity = 45.15%
	5		gi|149194374|ref|ZP_01871471.1|	Caminibacter mediatlanticus TB-2	E-value = 1.00E-68	Identity = 44.86%
AB1446	1		gi|152993252|ref|YP_001358973.1|	Sulfurovum sp. NBC37-1	E-value = 3.00E-39	Identity = 55.63%
	2		gi|152990705|ref|YP_001356427.1|	Nitratiruptor sp. SB155-2	E-value = 9.00E-38	Identity = 54.25%
	3		gi|149194373|ref|ZP_01871470.1|	Caminibacter mediatlanticus TB-2	E-value = 3.00E-37	Identity = 52.32%

	4		gi|78777636|ref|YP_393951.1|	Sulfuromonas denitrificans ATCC 33889	E-value = 7.00E-37	Identity = 53.59%
	5		gi|156718552|ref|ZP_02060216.1|	Hydrogenobaculum sp. Y04AAS1	E-value = 2.00E-35	Identity = 50.99%
AB1447	1	*acnB*	gi|152992939|ref|YP_001358660.1|	Sulfurovum sp. NBC37-1	E-value = 0	Identity = 76.08%
	2		gi|21673378|ref|NP_661443.1|	Chlorobium tepidum TLS	E-value = 0	Identity = 71.65%
	3		gi|145219413|ref|YP_001130122.1|	Prosthecochloris vibrioformis DSM 265	E-value = 0	Identity = 69.89%
	4		gi|149194800|ref|ZP_01871894.1|	Caminibacter mediatlanticus TB-2	E-value = 0	Identity = 70.35%
	5		gi|110597959|ref|ZP_01386240.1|	Chlorobium ferrooxidans DSM 13031	E-value = 0	Identity = 69.31%
AB1448	1		gi|83644037|ref|YP_432472.1|	Hahella chejuensis KCTC 2396	E-value = 2.00E-16	Identity = 35.00%
	2		gi|17233198|ref|NP_490288.1|	Nostoc sp. PCC 7120	E-value = 1.00E-15	Identity = 34.51%
	3		gi|21673295|ref|NP_661360.1|	Chlorobium tepidum TLS	E-value = 2.00E-15	Identity = 34.72%
	4		gi|89092965|ref|ZP_01165916.1|	Oceanospirillum sp. MED92	E-value = 2.00E-15	Identity = 35.29%
	5		gi|114778304|ref|ZP_01453163.1|	Mariprofundus ferrooxydans PV-1	E-value = 2.00E-14	Identity = 31.39%
AB1449	1		gi|149910133|ref|ZP_01898780.1|	Moritella sp. PE36	E-value = 4.00E-58	Identity = 42.36%
	2		gi|119945951|ref|YP_943631.1|	Psychromonas ingrahamii 37	E-value = 5.00E-55	Identity = 38.10%
	3		gi|85858780|ref|YP_460982.1|	Syntrophus aciditrophicus SB	E-value = 4.00E-34	Identity = 35.28%
	4		gi|114778031|ref|ZP_01452931.1|	Mariprofundus ferrooxydans PV-1	E-value = 4.00E-33	Identity = 32.24%
	5		gi|118591123|ref|ZP_01548522.1|	Stappia aggregata IAM 12614	E-value = 1.00E-32	Identity = 33.11%
AB1450	1		gi|34557403|ref|NP_907218.1|	Wolinella succinogenes DSM 1740	E-value = 1.00E-75	Identity = 62.87%
	2		gi|78777586|ref|YP_393901.1|	Sulfuromonas denitrificans ATCC 33889	E-value = 3.00E-68	Identity = 60.59%
	3		gi|67462064|sp|Q7VFS1|Y1604_HELHP	Helicobacter pylori	E-value = 7.00E-67	Identity = 60.83%
	4		gi|49036523|sp|Q7MXR6|Y097_PORGI	Porphyromonas gingivalis	E-value = 4.00E-62	Identity = 52.77%
	5		gi|118474323|ref|YP_891625.1|	Campylobacter fetus subsp. fetus 82-40	E-value = 1.00E-61	Identity = 57.45%
AB1451	1		gi|157165770|ref|YP_001467606.1|	Campylobacter concisus 13826	E-value = 1.00E-60	Identity = 53.63%
	2		gi|154175259|ref|YP_001408988.1|	Campylobacter curvus 525.92	E-value = 4.00E-59	Identity = 54.25%
	3		gi|118474953|ref|YP_891566.1|	Campylobacter fetus subsp. fetus 82-40	E-value = 8.00E-53	Identity = 46.56%
	4		gi|78777481|ref|YP_393796.1|	Sulfuromonas denitrificans ATCC 33889	E-value = 7.00E-52	Identity = 50.00%
	5		gi|154148618|ref|YP_001405921.1|	Campylobacter hominis ATCC BAA-381	E-value = 4.00E-48	Identity = 44.53%
AB1452	1		gi|15792695|ref|NP_282518.1|	Campylobacter jejuni subsp. jejuni NCTC 11168	E-value = 2.00E-27	Identity = 43.09%
	2		gi|57238416|ref|YP_179547.1|	Campylobacter jejuni subsp. jejuni RM1221	E-value = 4.00E-27	Identity = 41.49%
	3		gi|157415610|ref|YP_001482866.1|	Campylobacter jejuni subsp. jejuni 81116	E-value = 4.00E-27	Identity = 43.09%
	4		gi|121613470|ref|YP_001001031.1|	Campylobacter jejuni subsp. jejuni 81-176	E-value = 6.00E-27	Identity = 42.02%
	5		gi|154174115|ref|YP_001408989.1|	Campylobacter curvus 525.92	E-value = 1.00E-25	Identity = 40.22%
AB1453	1		gi|118475581|ref|YP_891568.1|	Campylobacter fetus subsp. fetus 82-40	E-value = 0	Identity = 49.88%
	2		gi|78777479|ref|YP_393794.1|	Sulfuromonas denitrificans ATCC 33889	E-value = 0	Identity = 47.92%
	3		gi|121541285|ref|ZP_01673039.1|	Candidatus Desulfococcus oleovorans Hxd3	E-value = 0	Identity = 46.26%
	4		gi|157164665|ref|YP_001467604.1|	Campylobacter concisus 13826	E-value = 0	Identity = 46.94%
	5		gi|154175484|ref|YP_001408990.1|	Campylobacter curvus 525.92	E-value = 0	Identity = 46.05%
AB1454	1		gi|83644827|ref|YP_433262.1|	Hahella chejuensis KCTC 2396	E-value = 2.00E-47	Identity = 46.49%
	2		gi|152998118|ref|YP_001342953.1|	Marinomonas sp. MWYL1	E-value = 2.00E-46	Identity = 46.49%
	3		gi|149375693|ref|ZP_01893462.1|	Marinobacter algicola DG893	E-value = 6.00E-45	Identity = 42.70%
	4		gi|120555885|ref|YP_960236.1|	Marinobacter aquaeolei VT8	E-value = 9.00E-44	Identity = 44.32%
	5		gi|23097794|ref|NP_691260.1|	Oceanobacillus iheyensis HTE831	E-value = 3.00E-43	Identity = 47.03%
AB1455	1		gi|39995295|ref|NP_951246.1|	Geobacter sulfurreducens PCA	E-value = 1.00E-30	Identity = 36.71%
	2		gi|78221449|ref|YP_383196.1|	Geobacter metallireducens GS-15	E-value = 2.00E-30	Identity = 35.44%
	3		gi|118581293|ref|YP_902543.1|	Pelobacter propionicus DSM 2379	E-value = 5.00E-30	Identity = 35.24%
	4		gi|67919842|ref|ZP_00513401.1|	Chlorobium limicola DSM 245	E-value = 1.00E-26	Identity = 35.53%
	5		gi|67938042|ref|ZP_00530572.1|	Chlorobium phaeobacteroides BS1	E-value = 8.00E-25	Identity = 32.66%
AB1456	1		gi|34556527|ref|NP_906342.1|	Wolinella succinogenes DSM 1740	E-value = 4.00E-23	Identity = 39.77%
	2		gi|157415470|ref|YP_001482726.1|	Campylobacter jejuni subsp. jejuni 81116	E-value = 3.00E-19	Identity = 35.29%

	3		gi|86150820|ref|ZP_01069036.1|	Campylobacter jejuni subsp. jejuni 260.94	E-value = 4.00E-19	Identity = 34.76%
	4		gi|148926210|ref|ZP_01809895.1|	Campylobacter jejuni subsp. jejuni CG8486	E-value = 4.00E-19	Identity = 35.29%
	5		gi|57238079|ref|YP_179328.1|	Campylobacter jejuni subsp. jejuni RM1221	E-value = 1.00E-18	Identity = 34.76%
AB1457	1	*dsbA*	gi|110346947|ref|YP_665765.1|	Mesorhizobium sp. BNC1	E-value = 2.00E-31	Identity = 32.86%
	2		gi|120556184|ref|YP_960535.1|	Marinobacter aquaeolei VT8	E-value = 3.00E-31	Identity = 34.27%
	3		gi|149374352|ref|ZP_01892126.1|	Marinobacter algicola DG893	E-value = 1.00E-30	Identity = 32.86%
	4		gi|85706988|ref|ZP_01038077.1|	Roseovarius sp. 217	E-value = 5.00E-29	Identity = 35.45%
	5		gi|86139682|ref|ZP_01058249.1|	Roseobacter sp. MED193	E-value = 1.00E-28	Identity = 33.95%
AB1458	1	*dsbB*	gi|113940134|ref|ZP_01425975.1|	Herpetosiphon aurantiacus ATCC 23779	E-value = 5.00E-26	Identity = 46.09%
	2		gi|89899229|ref|YP_521700.1|	Rhodoferax ferrireducens T118	E-value = 1.00E-25	Identity = 54.84%
	3		gi|52081838|ref|YP_080629.1|	Bacillus licheniformis ATCC 14580	E-value = 4.00E-23	Identity = 52.25%
	4		gi|154687466|ref|YP_001422627.1|	Bacillus amyloliquefaciens FZB42	E-value = 2.00E-22	Identity = 46.46%
	5		gi|149180934|ref|ZP_01859436.1|	Bacillus sp. SG-1	E-value = 4.00E-22	Identity = 44.96%
AB1459	1		gi|78777478|ref|YP_393793.1|	Sulfuromonas denitrificans ATCC 33889	E-value = 0	Identity = 58.60%
	2		gi|27379725|ref|NP_771254.1|	Bradyrhizobium japonicum USDA 110	E-value = 0	Identity = 43.57%
	3		gi|148255555|ref|YP_001240140.1|	Bradyrhizobium sp. BTAi1	E-value = 0	Identity = 43.38%
	4		gi|146340703|ref|YP_001205751.1|	Bradyrhizobium sp. ORS278	E-value = 0	Identity = 43.08%
	5		gi|92117324|ref|YP_577053.1|	Nitrobacter hamburgensis X14	E-value = 0	Identity = 42.68%
AB1460	1		gi|34557006|ref|NP_906821.1|	Wolinella succinogenes DSM 1740	E-value = 6.00E-33	Identity = 52.56%
	2		gi|118729457|ref|ZP_01577974.1|	Delftia acidovorans SPH-1	E-value = 5.00E-16	Identity = 33.13%
	3		gi|34557770|ref|NP_907585.1|	Wolinella succinogenes DSM 1740	E-value = 8.00E-16	Identity = 38.31%
	4		gi|116496542|gb|ABJ99054.1|	uncultured bacterium	E-value = 2.00E-15	Identity = 27.67%
	5		gi|33598198|ref|NP_885841.1|	Bordetella parapertussis 12822	E-value = 3.00E-15	Identity = 31.85%
AB1461	1		gi|34557007|ref|NP_906822.1|	Wolinella succinogenes DSM 1740	E-value = 4.00E-60	Identity = 42.34%
	2		gi|34557769|ref|NP_907584.1|	Wolinella succinogenes DSM 1740	E-value = 4.00E-29	Identity = 30.56%
	3		gi|30248821|ref|NP_840891.1|	Nitrosomonas europaea ATCC 19718	E-value = 2.00E-20	Identity = 27.49%
	4		gi|83747516|ref|ZP_00944554.1|	Ralstonia solanacearum UW551	E-value = 1.00E-18	Identity = 27.21%
	5		gi|17547638|ref|NP_521040.1|	Ralstonia solanacearum GMI1000	E-value = 4.00E-18	Identity = 26.84%
AB1462	1		gi|30248981|ref|NP_841051.1|	Nitrosomonas europaea ATCC 19718	E-value = 9.00E-178	Identity = 47.15%
	2		gi|30249066|ref|NP_841136.1|	Nitrosomonas europaea ATCC 19718	E-value = 5.00E-175	Identity = 45.62%
	3		gi|30248820|ref|NP_840890.1|	Nitrosomonas europaea ATCC 19718	E-value = 1.00E-173	Identity = 46.65%
	4		gi|66047091|ref|YP_236932.1|	Pseudomonas syringae pv. syringae B728a	E-value = 2.00E-122	Identity = 35.79%
	5		gi|28871270|ref|NP_793889.1|	Pseudomonas syringae pv. tomato str. DC3000	E-value = 5.00E-121	Identity = 35.78%
AB1463	1		gi|119945575|ref|YP_943255.1|	Psychromonas ingrahamii 37	E-value = 3.00E-120	Identity = 47.17%
	2		gi|71280812|ref|YP_270895.1|	Colwellia psychrerythraea 34H	E-value = 3.00E-115	Identity = 44.73%
	3		gi|134096466|ref|YP_001101541.1|	Herminiimonas arsenicoxydans	E-value = 2.00E-81	Identity = 32.85%
	4		gi|95928328|ref|ZP_01311076.1|	Desulfuromonas acetoxidans DSM 684	E-value = 2.00E-80	Identity = 32.98%
	5		gi|51244314|ref|YP_064198.1|	Desulfotalea psychrophila LSv54	E-value = 1.00E-79	Identity = 33.11%
AB1464	1		gi|154174424|ref|YP_001407383.1|	Campylobacter curvus 525.92	E-value = 5.00E-92	Identity = 66.19%
	2		gi|152989873|ref|YP_001355595.1|	Nitratiruptor sp. SB155-2	E-value = 2.00E-80	Identity = 57.89%
	3		gi|125974025|ref|YP_001037935.1|	Clostridium thermocellum ATCC 27405	E-value = 9.00E-58	Identity = 44.44%
	4		gi|95929109|ref|ZP_01311854.1|	Desulfuromonas acetoxidans DSM 684	E-value = 1.00E-56	Identity = 40.79%
	5		gi|15643263|ref|NP_228307.1|	Thermotoga maritima MSB8	E-value = 1.00E-54	Identity = 44.56%
AB1465	1	*betT*	gi|15600484|ref|NP_253978.1|	Pseudomonas aeruginosa PAO1	E-value = 0	Identity = 52.60%
	2		gi|84324003|ref|ZP_00972068.1|	Pseudomonas aeruginosa 2192	E-value = 0	Identity = 52.60%
	3		gi|26990662|ref|NP_746087.1|	Pseudomonas putida KT2440	E-value = 0	Identity = 51.54%
	4		gi|56461488|ref|YP_156769.1|	Idiomarina loihiensis L2TR	E-value = 0	Identity = 51.52%
	5		gi|152986479|ref|YP_001351380.1|	Pseudomonas aeruginosa PA7	E-value = 0	Identity = 52.52%
AB1466	1	*kefB*	gi|109898580|ref|YP_661835.1|	Pseudoalteromonas atlantica T6c	E-value = 3.00E-156	Identity = 58.30%

	2		gi|71281756|ref|YP_268492.1|	Colwellia psychrerythraea 34H	E-value = 2.00E-148	Identity = 55.06%
	3		gi|119946263|ref|YP_943943.1|	Psychromonas ingrahamii 37	E-value = 9.00E-147	Identity = 57.92%
	4		gi|78778273|ref|YP_394588.1|	Sulfuromonas denitrificans ATCC 33889	E-value = 4.00E-116	Identity = 48.59%
	5		gi|154174318|ref|YP_001408404.1|	Campylobacter curvus 525.92	E-value = 1.00E-91	Identity = 38.94%
AB1467	1		gi|114775673|ref|ZP_01451241.1|	Mariprofundus ferrooxydans PV-1	E-value = 4.00E-17	Identity = 48.96%
	2		gi|91788227|ref|YP_549179.1|	Polaromonas sp. JS666	E-value = 3.00E-13	Identity = 40.00%
	3		gi|149907978|ref|ZP_01896646.1|	Moritella sp. PE36	E-value = 7.00E-13	Identity = 44.44%
	4		gi|90412832|ref|ZP_01220832.1|	Photobacterium profundum 3TCK	E-value = 8.00E-13	Identity = 52.94%
	5		gi|54309950|ref|YP_130970.1|	Photobacterium profundum SS9	E-value = 9.00E-13	Identity = 52.94%
AB1468	1	*glyA2*	gi|78778141|ref|YP_394456.1|	Sulfuromonas denitrificans ATCC 33889	E-value = 0	Identity = 84.52%
	2		gi|78777472|ref|YP_393787.1|	Sulfuromonas denitrificans ATCC 33889	E-value = 0	Identity = 83.01%
	3		gi|152992073|ref|YP_001357794.1|	Sulfurovum sp. NBC37-1	E-value = 0	Identity = 83.58%
	4		gi|149193729|ref|ZP_01870827.1|	Caminibacter mediatlanticus TB-2	E-value = 0	Identity = 76.76%
	5		gi|118475761|ref|YP_892299.1|	Campylobacter fetus subsp. fetus 82-40	E-value = 0	Identity = 75.49%
AB1469	1	*ada*	gi|78778140|ref|YP_394455.1|	Sulfuromonas denitrificans ATCC 33889	E-value = 7.00E-138	Identity = 87.59%
	2		gi|34557931|ref|NP_907746.1|	Wolinella succinogenes DSM 1740	E-value = 6.00E-91	Identity = 60.44%
	3		gi|116326974|ref|YP_796694.1|	Leptospira borgpetersenii serovar Hardjo-bovis L550	E-value = 8.00E-62	Identity = 43.22%
	4		gi|71281861|ref|YP_270029.1|	Colwellia psychrerythraea 34H	E-value = 7.00E-61	Identity = 41.89%
	5		gi|108760938|ref|YP_634036.1|	Myxococcus xanthus DK 1622	E-value = 1.00E-60	Identity = 39.42%
AB1470	1		gi|78776541|ref|YP_392856.1|	Sulfuromonas denitrificans ATCC 33889	E-value = 2.00E-68	Identity = 58.05%
	2		gi|157374449|ref|YP_001473049.1|	Shewanella sediminis HAW-EB3	E-value = 1.00E-62	Identity = 54.74%
	3		gi|148643535|ref|YP_001274048.1|	Methanobrevibacter smithii ATCC 35061	E-value = 9.00E-52	Identity = 51.09%
	4		gi|45358149|ref|NP_987706.1|	Methanococcus maripaludis S2	E-value = 7.00E-49	Identity = 51.83%
	5		gi|21228990|ref|NP_634912.1|	Methanosarcina mazei Go1	E-value = 1.00E-48	Identity = 46.55%
AB1471	1		gi|77919867|ref|YP_357682.1|	Pelobacter carbinolicus DSM 2380	E-value = 1.00E-35	Identity = 48.41%
	2		gi|109899705|ref|YP_662960.1|	Pseudoalteromonas atlantica T6c	E-value = 1.00E-31	Identity = 45.68%
	3		gi|90023082|ref|YP_528909.1|	Saccharophagus degradans 2-40	E-value = 4.00E-27	Identity = 46.25%
	4		gi|78777319|ref|YP_393634.1|	Sulfuromonas denitrificans ATCC 33889	E-value = 2.00E-22	Identity = 44.37%
	5		gi|91201967|emb|CAJ75027.1|	Candidatus Kuenenia stuttgartiensis	E-value = 5.00E-14	Identity = 31.21%
AB1472	1		gi|78778128|ref|YP_394443.1|	Sulfuromonas denitrificans ATCC 33889	E-value = 2.00E-24	Identity = 63.92%
	2		gi|148265234|ref|YP_001231940.1|	Geobacter uraniumreducens Rf4	E-value = 5.00E-19	Identity = 53.06%
	3		gi|110597931|ref|ZP_01386213.1|	Chlorobium ferrooxidans DSM 13031	E-value = 6.00E-19	Identity = 52.27%
	4		gi|67154185|ref|ZP_00415930.1|	Azotobacter vinelandii AvOP	E-value = 3.00E-18	Identity = 48.45%
	5		gi|94264326|ref|ZP_01288119.1|	delta proteobacterium MLMS-1	E-value = 1.00E-17	Identity = 46.32%
AB1473	1			***** No matches found *****		
AB1474	1		gi|78778155|ref|YP_394470.1|	Sulfuromonas denitrificans ATCC 33889	E-value = 8.00E-68	Identity = 76.43%
	2		gi|78778142|ref|YP_394457.1|	Sulfuromonas denitrificans ATCC 33889	E-value = 2.00E-63	Identity = 71.34%
	3		gi|34557930|ref|NP_907745.1|	Wolinella succinogenes DSM 1740	E-value = 2.00E-61	Identity = 68.79%
	4		gi|106886469|ref|ZP_01353808.1|	Clostridium phytofermentans ISDg	E-value = 2.00E-49	Identity = 57.32%
	5		gi|106894095|ref|ZP_01361217.1|	Clostridium sp. OhILAs	E-value = 6.00E-43	Identity = 52.23%
AB1475	1	*benE*	gi|118733230|ref|ZP_01581742.1|	Delftia acidovorans SPH-1	E-value = 1.00E-132	Identity = 69.82%
	2		gi|118051873|ref|ZP_01520421.1|	Comamonas testosteroni KF-1	E-value = 1.00E-128	Identity = 71.28%
	3		gi|152981116|ref|YP_001353696.1|	Janthinobacterium sp. Marseille	E-value = 5.00E-128	Identity = 70.45%
	4		gi|91976777|ref|YP_569436.1|	Rhodopseudomonas palustris BisB5	E-value = 5.00E-127	Identity = 69.95%
	5		gi|153011455|ref|YP_001372669.1|	Ochrobactrum anthropi ATCC 49188	E-value = 1.00E-124	Identity = 70.98%
AB1476	1	*argD2*	gi|78777452|ref|YP_393767.1|	Sulfuromonas denitrificans ATCC 33889	E-value = 2.00E-117	Identity = 54.45%
	2		gi|152990810|ref|YP_001356532.1|	Nitratiruptor sp. SB155-2	E-value = 2.00E-115	Identity = 53.81%
	3		gi|34556738|ref|NP_906553.1|	Wolinella succinogenes DSM 1740	E-value = 4.00E-112	Identity = 51.44%
	4		gi|154148678|ref|YP_001406297.1|	Campylobacter hominis ATCC BAA-381	E-value = 7.00E-110	Identity = 55.99%

	5		gi|154174095|ref|YP_001408104.1|	Campylobacter curvus 525.92	E-value = 2.00E-109	Identity = 55.06%
AB1477	1		gi|34556825|ref|NP_906640.1|	Wolinella succinogenes DSM 1740	E-value = 1.00E-40	Identity = 44.50%
	2		gi|52425939|ref|YP_089076.1|	Mannheimia succiniciproducens MBEL55E	E-value = 4.00E-30	Identity = 33.19%
	3		gi|145632522|ref|ZP_01788256.1|	Haemophilus influenzae 3655	E-value = 2.00E-29	Identity = 33.81%
	4		gi|152979425|ref|YP_001345054.1|	Actinobacillus succinogenes 130Z	E-value = 3.00E-29	Identity = 31.47%
	5		gi|145638816|ref|ZP_01794425.1|	Haemophilus influenzae PittII	E-value = 4.00E-29	Identity = 33.81%
AB1478	1	*lipA*	gi|78224341|ref|YP_386088.1|	Geobacter metallireducens GS-15	E-value = 7.00E-82	Identity = 54.51%
	2		gi|148262482|ref|YP_001229188.1|	Geobacter uraniumreducens Rf4	E-value = 2.00E-81	Identity = 54.15%
	3		gi|152992558|ref|YP_001358279.1|	Sulfurovum sp. NBC37-1	E-value = 1.00E-78	Identity = 52.82%
	4		gi|110600043|ref|ZP_01388272.1|	Geobacter sp. FRC-32	E-value = 1.00E-77	Identity = 51.61%
	5		gi|145618036|ref|ZP_01774098.1|	Geobacter bemidjiensis Bem	E-value = 9.00E-77	Identity = 50.72%
AB1479	1		gi|152992559|ref|YP_001358280.1|	Sulfurovum sp. NBC37-1	E-value = 5.00E-36	Identity = 36.86%
	2		gi|148262479|ref|YP_001229185.1|	Geobacter uraniumreducens Rf4	E-value = 3.00E-31	Identity = 35.39%
	3		gi|118580653|ref|YP_901903.1|	Pelobacter propionicus DSM 2379	E-value = 2.00E-30	Identity = 32.37%
	4		gi|110600042|ref|ZP_01388271.1|	Geobacter sp. FRC-32	E-value = 3.00E-29	Identity = 32.02%
	5		gi|145618035|ref|ZP_01774097.1|	Geobacter bemidjiensis Bem	E-value = 2.00E-27	Identity = 32.37%
AB1480	1	*aceE*	gi|117918883|ref|YP_868075.1|	Shewanella sp. ANA-3	E-value = 0	Identity = 70.22%
	2		gi|24372019|ref|NP_716061.1|	Shewanella oneidensis MR-1	E-value = 0	Identity = 70.00%
	3		gi|120597357|ref|YP_961931.1|	Shewanella sp. W3-18-1	E-value = 0	Identity = 70.11%
	4		gi|113968772|ref|YP_732565.1|	Shewanella sp. MR-4	E-value = 0	Identity = 70.00%
	5		gi|126176103|ref|YP_001052252.1|	Shewanella baltica OS155	E-value = 0	Identity = 69.78%
AB1481	1	*aceF*	gi|113461237|ref|YP_719306.1|	Haemophilus somnus 129PT	E-value = 5.00E-137	Identity = 50.62%
	2		gi|114564928|ref|YP_752442.1|	Shewanella frigidimarina NCIMB 400	E-value = 1.00E-135	Identity = 50.27%
	3		gi|53728561|ref|ZP_00132374.2|	Haemophilus somnus 2336	E-value = 2.00E-135	Identity = 50.44%
	4		gi|113950875|ref|ZP_01436484.1|	Shewanella baltica OS195	E-value = 2.00E-135	Identity = 50.45%
	5		gi|149114198|ref|ZP_01840953.1|	Shewanella baltica OS223	E-value = 3.00E-135	Identity = 51.09%
AB1482	1	*lpdA*	gi|149191281|ref|ZP_01869536.1|	Vibrio shilonii AK1	E-value = 1.00E-176	Identity = 66.32%
	2		gi|15642409|ref|NP_232042.1|	Vibrio cholerae O1 biovar eltor str. N16961	E-value = 2.00E-176	Identity = 66.11%
	3		gi|116216869|ref|ZP_01482696.1|	Vibrio cholerae RC385	E-value = 2.00E-176	Identity = 66.11%
	4		gi|37680955|ref|NP_935564.1|	Vibrio vulnificus YJ016	E-value = 9.00E-176	Identity = 65.68%
	5		gi|52425389|ref|YP_088526.1|	Mannheimia succiniciproducens MBEL55E	E-value = 2.00E-175	Identity = 64.77%
AB1483	1		gi|116748043|ref|YP_844730.1|	Syntrophobacter fumaroxidans MPOB	E-value = 1.00E-32	Identity = 37.95%
	2		gi|152981489|ref|YP_001355336.1|	Janthinobacterium sp. Marseille	E-value = 3.00E-32	Identity = 42.13%
	3		gi|77165701|ref|YP_344226.1|	Nitrosococcus oceani ATCC 19707	E-value = 7.00E-32	Identity = 38.29%
	4		gi|118745022|ref|ZP_01593006.1|	Geobacter lovleyi SZ	E-value = 4.00E-30	Identity = 43.35%
	5		gi|20091784|ref|NP_617859.1|	Methanosarcina acetivorans C2A	E-value = 1.00E-29	Identity = 38.74%
AB1484	1		gi|119899284|ref|YP_934497.1|	Azoarcus sp. BH72	E-value = 0	Identity = 47.27%
	2		gi|34557768|ref|NP_907583.1|	Wolinella succinogenes DSM 1740	E-value = 0	Identity = 45.51%
	3		gi|34557008|ref|NP_906823.1|	Wolinella succinogenes DSM 1740	E-value = 1.00E-170	Identity = 43.81%
	4		gi|21244223|ref|NP_643805.1|	Xanthomonas axonopodis pv. citri str. 306	E-value = 3.00E-151	Identity = 40.63%
	5		gi|114046494|ref|YP_737044.1|	Shewanella sp. MR-7	E-value = 8.00E-149	Identity = 43.15%
AB1485	1		gi|15896283|ref|NP_349632.1|	Clostridium acetobutylicum ATCC 824	E-value = 2.00E-58	Identity = 43.92%
	2		gi|75759687|ref|ZP_00739770.1|	Bacillus thuringiensis serovar israelensis ATCC 35646	E-value = 5.00E-58	Identity = 41.05%
	3		gi|116872716|ref|YP_849497.1|	Listeria welshimeri serovar 6b str. SLCC5334	E-value = 5.00E-58	Identity = 42.32%
	4		gi|47097699|ref|ZP_00235208.1|	Listeria monocytogenes str. 1/2a F6854	E-value = 7.00E-58	Identity = 41.30%
	5		gi|16803323|ref|NP_464808.1|	Listeria monocytogenes EGD-e	E-value = 9.00E-58	Identity = 41.30%
AB1486	1		gi|118743697|ref|ZP_01591699.1|	Geobacter lovleyi SZ	E-value = 1.00E-58	Identity = 46.89%
	2		gi|148265527|ref|YP_001232233.1|	Geobacter uraniumreducens Rf4	E-value = 5.00E-57	Identity = 43.68%
	3		gi|67917703|ref|ZP_00511308.1|	Chlorobium limicola DSM 245	E-value = 7.00E-57	Identity = 47.22%

	4		gi|146283038|ref|YP_001173191.1|	Pseudomonas stutzeri A1501	E-value = 5.00E-56	Identity = 43.10%
	5		gi|55418027|ref|YP_133927.1|	Plasmid pB3	E-value = 3.00E-54	Identity = 43.19%
AB1487	1	*rhlE*	gi|78776226|ref|YP_392541.1|	Sulfuromonas denitrificans ATCC 33889	E-value = 2.00E-149	Identity = 69.54%
	2		gi|152992886|ref|YP_001358607.1|	Sulfurovum sp. NBC37-1	E-value = 8.00E-147	Identity = 63.66%
	3		gi|37525462|ref|NP_928806.1|	Photorhabdus luminescens subsp. laumondii TTO1	E-value = 1.00E-146	Identity = 63.74%
	4		gi|145589523|ref|YP_001156120.1|	Polynucleobacter sp. QLW-P1DMWA-1	E-value = 4.00E-144	Identity = 64.71%
	5		gi|157369574|ref|YP_001477563.1|	Serratia proteamaculans 568	E-value = 4.00E-142	Identity = 64.99%
AB1488	1		gi|150003156|ref|YP_001297900.1|	Bacteroides vulgatus ATCC 8482	E-value = 6.00E-67	Identity = 38.48%
	2		gi|148266154|ref|YP_001232860.1|	Geobacter uraniumreducens Rf4	E-value = 6.00E-66	Identity = 36.07%
	3		gi|91216664|ref|ZP_01253629.1|	Psychroflexus torquis ATCC 700755	E-value = 4.00E-61	Identity = 37.95%
	4		gi|150010028|ref|YP_001304771.1|	Parabacteroides distasonis ATCC 8503	E-value = 2.00E-60	Identity = 39.78%
	5		gi|152992178|ref|YP_001357899.1|	Sulfurovum sp. NBC37-1	E-value = 3.00E-60	Identity = 41.90%
AB1489	1		gi|157414453|ref|YP_001481709.1|	Campylobacter jejuni subsp. jejuni 81116	E-value = 8.00E-125	Identity = 73.27%
	2		gi|78777762|ref|YP_394077.1|	Sulfuromonas denitrificans ATCC 33889	E-value = 9.00E-119	Identity = 66.88%
	3		gi|462657|sp|P34877|MTSA_LACLC	Lactococcus lactis subsp. cremoris	E-value = 5.00E-105	Identity = 61.59%
	4		gi|50914506|ref|YP_060478.1|	Streptococcus pyogenes MGAS10394	E-value = 5.00E-105	Identity = 60.12%
	5		gi|127471|sp|P24581|MTNX_NEILA	Neisseria lactamica	E-value = 9.00E-103	Identity = 60.47%
AB1490	1		gi|157414452|ref|YP_001481708.1|	Campylobacter jejuni subsp. jejuni 81116	E-value = 5.00E-35	Identity = 36.61%
	2		gi|78777760|ref|YP_394075.1|	Sulfuromonas denitrificans ATCC 33889	E-value = 1.00E-31	Identity = 33.53%
AB1492	1		gi|152993198|ref|YP_001358919.1|	Sulfurovum sp. NBC37-1	E-value = 5.00E-15	Identity = 50.55%
	2		gi|19705293|ref|NP_602788.1|	Fusobacterium nucleatum subsp. nucleatum ATCC 25586	E-value = 2.00E-12	Identity = 49.43%
	3		gi|34763122|ref|ZP_00144092.1|	Fusobacterium nucleatum subsp. vincentii ATCC 49256	E-value = 8.00E-12	Identity = 49.43%
	4		gi|148322442|gb|EDK87692.1|	Fusobacterium nucleatum subsp. polymorphum ATCC 10953	E-value = 1.00E-11	Identity = 49.43%
	5		gi|34540741|ref|NP_905220.1|	Porphyromonas gingivalis W83	E-value = 6.00E-09	Identity = 38.82%
AB1493	1		gi|51246875|ref|YP_066759.1|	Desulfotalea psychrophila LSv54	E-value = 0	Identity = 62.94%
	2		gi|78776315|ref|YP_392630.1|	Sulfuromonas denitrificans ATCC 33889	E-value = 3.00E-174	Identity = 60.15%
	3		gi|119469669|ref|ZP_01612538.1|	Alteromonadales bacterium TW-7	E-value = 1.00E-93	Identity = 34.86%
	4		gi|77359055|ref|YP_338630.1|	Pseudoalteromonas haloplanktis TAC125	E-value = 3.00E-92	Identity = 34.51%
	5		gi|71908545|ref|YP_286132.1|	Dechloromonas aromatica RCB	E-value = 7.00E-92	Identity = 35.17%
AB1494	1		gi|78486195|ref|YP_392120.1|	Thiomicrospira crunogena XCL-2	E-value = 3.00E-15	Identity = 38.28%
	2		gi|119946529|ref|YP_944209.1|	Psychromonas ingrahamii 37	E-value = 4.00E-15	Identity = 33.07%
	3		gi|78486408|ref|YP_392333.1|	Thiomicrospira crunogena XCL-2	E-value = 2.00E-14	Identity = 32.28%
	4		gi|88797679|ref|ZP_01113267.1|	Reinekea sp. MED297	E-value = 3.00E-13	Identity = 30.71%
	5		gi|121533905|ref|ZP_01665731.1|	Thermosinus carboxydivorans Nor1	E-value = 4.00E-13	Identity = 38.10%
AB1495	1		gi|78485876|ref|YP_391801.1|	Thiomicrospira crunogena XCL-2	E-value = 2.00E-22	Identity = 53.19%
	2		gi|118746413|ref|ZP_01594354.1|	Geobacter lovleyi SZ	E-value = 2.00E-15	Identity = 41.13%
	3		gi|145618245|ref|ZP_01774305.1|	Geobacter bemidjiensis Bem	E-value = 1.00E-13	Identity = 41.30%
	4		gi|87302654|ref|ZP_01085471.1|	Synechococcus sp. WH 5701	E-value = 2.00E-13	Identity = 41.18%
	5		gi|118580064|ref|YP_901314.1|	Pelobacter propionicus DSM 2379	E-value = 1.00E-12	Identity = 36.17%
AB1496	1		gi|34557389|ref|NP_907204.1|	Wolinella succinogenes DSM 1740	E-value = 5.00E-69	Identity = 31.06%
	2		gi|46200953|ref|ZP_00056091.2|	Magnetospirillum magnetotacticum MS-1	E-value = 6.00E-63	Identity = 28.40%
	3		gi|83309953|ref|YP_420217.1|	Magnetospirillum magneticum AMB-1	E-value = 1.00E-62	Identity = 28.23%
	4		gi|78777187|ref|YP_393502.1|	Sulfuromonas denitrificans ATCC 33889	E-value = 3.00E-61	Identity = 30.87%
	5		gi|83312482|ref|YP_422746.1|	Magnetospirillum magneticum AMB-1	E-value = 7.00E-59	Identity = 26.44%
AB1497	1	*rnhA*	gi|88857533|ref|ZP_01132176.1|	Pseudoalteromonas tunicata D2	E-value = 1.00E-39	Identity = 46.77%
AB1498	1		gi|156863454|gb|EDO56885.1|	Clostridium sp. L2-50	E-value = 5.00E-105	Identity = 58.36%
	2		gi|104780196|ref|YP_606694.1|	Pseudomonas entomophila L48	E-value = 5.00E-32	Identity = 31.67%
	3		gi|148358583|ref|YP_001249790.1|	Legionella pneumophila str. Corby	E-value = 2.00E-28	Identity = 34.12%
	4		gi|54295521|ref|YP_127936.1|	Legionella pneumophila str. Lens	E-value = 4.00E-28	Identity = 33.73%

	5		gi|54298671|ref|YP_125040.1|	Legionella pneumophila str. Paris	E-value = 6.00E-28	Identity = 33.73%
AB1499	1		gi|148652374|ref|YP_001279467.1|	Psychrobacter sp. PRwf-1	E-value = 9.00E-27	Identity = 61.05%
	2		gi|16120955|ref|NP_404268.1|	Yersinia pestis CO92	E-value = 4.00E-26	Identity = 61.05%
	3		gi|119899155|ref|YP_934368.1|	Azoarcus sp. BH72	E-value = 3.00E-24	Identity = 54.64%
	4		gi|47564352|ref|ZP_00235397.1|	Bacillus cereus G9241	E-value = 4.00E-24	Identity = 58.95%
	5		gi|73663500|ref|YP_302281.1|	Staphylococcus saprophyticus subsp. saprophyticus ATCC 15305	E-value = 4.00E-23	Identity = 54.64%
AB1500	1		gi|88711273|ref|ZP_01105361.1|	Flavobacteriales bacterium HTCC2170	E-value = 2.00E-19	Identity = 42.74%
	2		gi|88712111|ref|ZP_01106198.1|	Flavobacteriales bacterium HTCC2170	E-value = 1.00E-14	Identity = 42.86%
	3		gi|83815994|ref|YP_445270.1|	Salinibacter ruber DSM 13855	E-value = 1.00E-10	Identity = 37.50%
	4		gi|126646652|ref|ZP_01719162.1|	Algoriphagus sp. PR1	E-value = 7.00E-07	Identity = 34.88%
AB1501	1	*dinP*	gi|152992967|ref|YP_001358688.1|	Sulfurovum sp. NBC37-1	E-value = 4.00E-101	Identity = 43.76%
	2		gi|152990028|ref|YP_001355750.1|	Nitratiruptor sp. SB155-2	E-value = 8.00E-91	Identity = 43.74%
	3		gi|78778034|ref|YP_394349.1|	Sulfuromonas denitrificans ATCC 33889	E-value = 2.00E-87	Identity = 42.29%
	4		gi|149195302|ref|ZP_01872390.1|	Caminibacter mediatlanticus TB-2	E-value = 1.00E-84	Identity = 45.73%
	5		gi|118474273|ref|YP_891621.1|	Campylobacter fetus subsp. fetus 82-40	E-value = 2.00E-54	Identity = 34.20%
AB1502	1	*moeA1*	gi|149194391|ref|ZP_01871488.1|	Caminibacter mediatlanticus TB-2	E-value = 3.00E-93	Identity = 46.80%
	2		gi|34557231|ref|NP_907046.1|	Wolinella succinogenes DSM 1740	E-value = 1.00E-91	Identity = 42.29%
	3		gi|154175348|ref|YP_001408258.1|	Campylobacter curvus 525.92	E-value = 4.00E-81	Identity = 43.72%
	4		gi|157164837|ref|YP_001466854.1|	Campylobacter concisus 13826	E-value = 2.00E-79	Identity = 44.30%
	5		gi|118475083|ref|YP_892062.1|	Campylobacter fetus subsp. fetus 82-40	E-value = 5.00E-73	Identity = 40.51%
AB1503	1		gi|78777015|ref|YP_393330.1|	Sulfuromonas denitrificans ATCC 33889	E-value = 1.00E-81	Identity = 59.26%
AB1504	1	*fdhD*	gi|78777016|ref|YP_393331.1|	Sulfuromonas denitrificans ATCC 33889	E-value = 6.00E-98	Identity = 67.15%
	2		gi|34557521|ref|NP_907336.1|	Wolinella succinogenes DSM 1740	E-value = 2.00E-71	Identity = 55.86%
	3		gi|34556484|ref|NP_906299.1|	Wolinella succinogenes DSM 1740	E-value = 2.00E-67	Identity = 53.94%
	4		gi|34557234|ref|NP_907049.1|	Wolinella succinogenes DSM 1740	E-value = 1.00E-65	Identity = 50.99%
	5		gi|32265724|ref|NP_859756.1|	Helicobacter hepaticus ATCC 51449	E-value = 9.00E-60	Identity = 48.80%
AB1505	1	*fdhC*	gi|78777017|ref|YP_393332.1|	Sulfuromonas denitrificans ATCC 33889	E-value = 3.00E-122	Identity = 68.47%
AB1506	1	*fdhB1*	gi|78777018|ref|YP_393333.1|	Sulfuromonas denitrificans ATCC 33889	E-value = 1.00E-90	Identity = 86.98%
	2		gi|34733215|gb|AAQ81583.1|	Sulfurospirillum multivorans	E-value = 7.00E-75	Identity = 70.98%
	3		gi|34556486|ref|NP_906301.1|	Wolinella succinogenes DSM 1740	E-value = 2.00E-74	Identity = 71.05%
	4		gi|149194120|ref|ZP_01871218.1|	Caminibacter mediatlanticus TB-2	E-value = 4.00E-74	Identity = 71.88%
	5		gi|157164783|ref|YP_001467446.1|	Campylobacter concisus 13826	E-value = 5.00E-71	Identity = 70.37%
AB1507	1	*fdhA1*	gi|78777019|ref|YP_393334.1|	Sulfuromonas denitrificans ATCC 33889	E-value = 0	Identity = 79.17%
	2		gi|34733214|gb|AAQ81582.1|	Sulfurospirillum multivorans	E-value = 0	Identity = 60.38%
	3		gi|34557518|ref|NP_907333.1|	Wolinella succinogenes DSM 1740	E-value = 0	Identity = 57.63%
	4		gi|34556487|ref|NP_906302.1|	Wolinella succinogenes DSM 1740	E-value = 0	Identity = 57.63%
	5		gi|48507|emb|CAA37989.1|	Wolinella succinogenes	E-value = 0	Identity = 57.63%
AB1509	1		gi|78777021|ref|YP_393336.1|	Sulfuromonas denitrificans ATCC 33889	E-value = 7.00E-15	Identity = 66.22%
AB1510	1		gi|78777022|ref|YP_393337.1|	Sulfuromonas denitrificans ATCC 33889	E-value = 3.00E-58	Identity = 57.71%
	2		gi|34557516|ref|NP_907331.1|	Wolinella succinogenes DSM 1740	E-value = 4.00E-14	Identity = 28.50%
	3		gi|57504778|ref|ZP_00370810.1|	Campylobacter coli RM2228	E-value = 1.00E-12	Identity = 31.96%
	4		gi|148925676|ref|ZP_01809364.1|	Campylobacter jejuni subsp. jejuni CG8486	E-value = 2.00E-12	Identity = 31.12%
	5		gi|51246836|ref|YP_066720.1|	Desulfotalea psychrophila LSv54	E-value = 4.00E-12	Identity = 26.53%
AB1511	1		gi|78777023|ref|YP_393338.1|	Sulfuromonas denitrificans ATCC 33889	E-value = 1.00E-164	Identity = 51.88%
	2		gi|154174150|ref|YP_001408944.1|	Campylobacter curvus 525.92	E-value = 5.00E-123	Identity = 42.93%
	3		gi|57168293|ref|ZP_00367432.1|	Campylobacter coli RM2228	E-value = 1.00E-117	Identity = 40.97%
	4		gi|57238420|ref|YP_179551.1|	Campylobacter jejuni subsp. jejuni RM1221	E-value = 3.00E-117	Identity = 40.79%
	5		gi|153952390|ref|YP_001397493.1|	Campylobacter jejuni subsp. doylei 269.97	E-value = 6.00E-117	Identity = 40.97%
AB1512	1	*livJ*	gi|78777024|ref|YP_393339.1|	Sulfuromonas denitrificans ATCC 33889	E-value = 7.00E-99	Identity = 51.75%

	2		gi|34557420|ref|NP_907235.1|	Wolinella succinogenes DSM 1740	E-value = 3.00E-56	Identity = 37.46%
	3		gi|88809249|ref|ZP_01124758.1|	Synechococcus sp. WH 7805	E-value = 2.00E-49	Identity = 34.01%
	4		gi|51245627|ref|YP_065511.1|	Desulfotalea psychrophila LSv54	E-value = 4.00E-47	Identity = 33.33%
	5		gi|113954052|ref|YP_729852.1|	Synechococcus sp. CC9311	E-value = 2.00E-44	Identity = 31.32%
AB1513	1		gi|78777025|ref|YP_393340.1|	Sulfuromonas denitrificans ATCC 33889	E-value = 2.00E-125	Identity = 53.83%
	2		gi|34557419|ref|NP_907234.1|	Wolinella succinogenes DSM 1740	E-value = 2.00E-83	Identity = 37.97%
	3		gi|32265520|ref|NP_859552.1|	Helicobacter hepaticus ATCC 51449	E-value = 4.00E-49	Identity = 31.63%
AB1514	1		gi|78777026|ref|YP_393341.1|	Sulfuromonas denitrificans ATCC 33889	E-value = 4.00E-61	Identity = 68.49%
	2		gi|34557418|ref|NP_907233.1|	Wolinella succinogenes DSM 1740	E-value = 3.00E-39	Identity = 42.58%
	3		gi|32265521|ref|NP_859553.1|	Helicobacter hepaticus ATCC 51449	E-value = 1.00E-33	Identity = 40.85%
	4		gi|78222590|ref|YP_384337.1|	Geobacter metallireducens GS-15	E-value = 1.00E-26	Identity = 29.17%
	5		gi|110601676|ref|ZP_01389851.1|	Geobacter sp. FRC-32	E-value = 1.00E-26	Identity = 30.37%
AB1515	1		gi|149189827|ref|ZP_01868107.1|	Vibrio shilonii AK1	E-value = 2.00E-111	Identity = 57.49%
	2		gi|156975909|ref|YP_001446816.1|	Vibrio harveyi ATCC BAA-1116	E-value = 4.00E-110	Identity = 55.12%
	3		gi|153835570|ref|ZP_01988237.1|	Vibrio harveyi HY01	E-value = 4.00E-110	Identity = 55.61%
	4		gi|28899455|ref|NP_799060.1|	Vibrio parahaemolyticus RIMD 2210633	E-value = 1.00E-109	Identity = 55.37%
	5		gi|153839606|ref|ZP_01992273.1|	Vibrio parahaemolyticus AQ3810	E-value = 5.00E-109	Identity = 55.37%
AB1516	1		gi|149194144|ref|ZP_01871242.1|	Caminibacter mediatlanticus TB-2	E-value = 2.00E-07	Identity = 39.74%
	2		gi|154174362|ref|YP_001408063.1|	Campylobacter curvus 525.92	E-value = 1.00E-06	Identity = 38.10%
AB1517	1			***** No matches found *****		
AB1518	1		gi|154175018|ref|YP_001408061.1|	Campylobacter curvus 525.92	E-value = 1.00E-83	Identity = 45.08%
	2		gi|113971125|ref|YP_734918.1|	Shewanella sp. MR-4	E-value = 2.00E-83	Identity = 42.78%
	3		gi|113949655|ref|ZP_01435300.1|	Shewanella baltica OS195	E-value = 1.00E-82	Identity = 41.58%
	4		gi|126175262|ref|YP_001051411.1|	Shewanella baltica OS155	E-value = 2.00E-82	Identity = 41.58%
	5		gi|153001587|ref|YP_001367268.1|	Shewanella baltica OS185	E-value = 2.00E-82	Identity = 41.58%
AB1519	1		gi|78777017|ref|YP_393332.1|	Sulfuromonas denitrificans ATCC 33889	E-value = 1.00E-121	Identity = 68.15%
AB1520	1	*fdhB2*	gi|78777018|ref|YP_393333.1|	Sulfuromonas denitrificans ATCC 33889	E-value = 1.00E-90	Identity = 86.98%
	2		gi|34733215|gb|AAQ81583.1|	Sulfurospirillum multivorans	E-value = 7.00E-75	Identity = 70.98%
	3		gi|34556486|ref|NP_906301.1|	Wolinella succinogenes DSM 1740	E-value = 2.00E-74	Identity = 71.05%
	4		gi|149194120|ref|ZP_01871218.1|	Caminibacter mediatlanticus TB-2	E-value = 4.00E-74	Identity = 71.88%
	5		gi|157164783|ref|YP_001467446.1|	Campylobacter concisus 13826	E-value = 5.00E-71	Identity = 70.37%
AB1521	1	*fdhA2*	gi|78777019|ref|YP_393334.1|	Sulfuromonas denitrificans ATCC 33889	E-value = 0	Identity = 75.37%
	2		gi|34557518|ref|NP_907333.1|	Wolinella succinogenes DSM 1740	E-value = 0	Identity = 56.30%
	3		gi|34556487|ref|NP_906302.1|	Wolinella succinogenes DSM 1740	E-value = 0	Identity = 56.19%
	4		gi|48507|emb|CAA37989.1|	Wolinella succinogenes	E-value = 0	Identity = 56.19%
	5		gi|34733214|gb|AAQ81582.1|	Sulfurospirillum multivorans	E-value = 0	Identity = 57.78%
AB1522	1		gi|78777021|ref|YP_393336.1|	Sulfuromonas denitrificans ATCC 33889	E-value = 2.00E-15	Identity = 69.86%
AB1523	1	*selD*	gi|78777029|ref|YP_393344.1|	Sulfuromonas denitrificans ATCC 33889	E-value = 4.00E-125	Identity = 65.85%
	2		gi|149194146|ref|ZP_01871244.1|	Caminibacter mediatlanticus TB-2	E-value = 2.00E-92	Identity = 54.77%
	3		gi|34557385|ref|NP_907200.1|	Wolinella succinogenes DSM 1740	E-value = 2.00E-88	Identity = 48.41%
	4		gi|29336986|sp|Q9PMF9|SELD_CAMJE	Campylobacter jejuni subsp. jejuni NCTC 11168	E-value = 2.00E-79	Identity = 47.79%
	5		gi|57238527|ref|YP_179658.1|	Campylobacter jejuni subsp. jejuni RM1221	E-value = 4.00E-78	Identity = 47.20%
AB1524	1			***** No matches found *****		
AB1525	1		gi|110599704|ref|ZP_01387939.1|	Geobacter sp. FRC-32	E-value = 2.00E-07	Identity = 25.99%
AB1526	1		gi|21242357|ref|NP_641939.1|	Xanthomonas axonopodis pv. citri str. 306	E-value = 3.00E-43	Identity = 52.33%
	2		gi|110599707|ref|ZP_01387942.1|	Geobacter sp. FRC-32	E-value = 4.00E-42	Identity = 48.53%
	3		gi|87121962|ref|ZP_01077847.1|	Marinomonas sp. MED121	E-value = 1.00E-37	Identity = 46.84%
	4		gi|53715126|ref|YP_101118.1|	Bacteroides fragilis YCH46	E-value = 5.00E-27	Identity = 40.80%
	5		gi|90022547|ref|YP_528374.1|	Saccharophagus degradans 2-40	E-value = 4.00E-24	Identity = 41.27%

AB1527	1		gi|78047206|ref|YP_363381.1|	Xanthomonas campestris pv. vesicatoria str. 85-10	E-value = 7.00E-22	Identity = 51.81%
	2		gi|21242358|ref|NP_641940.1|	Xanthomonas axonopodis pv. citri str. 306	E-value = 1.00E-21	Identity = 51.81%
	3		gi|110599706|ref|ZP_01387941.1|	Geobacter sp. FRC-32	E-value = 3.00E-20	Identity = 45.24%
	4		gi|59714164|ref|YP_206939.1|	Vibrio fischeri ES114	E-value = 4.00E-09	Identity = 34.94%
AB1528	1			***** No matches found *****		
AB1529	1		gi|37525649|ref|NP_928993.1|	Photorhabdus luminescens subsp. laumondii TTO1	E-value = 3.00E-16	Identity = 29.43%
AB1530	1		gi|153206714|ref|ZP_01945555.1|	Coxiella burnetii 'MSU Goat Q177'	E-value = 1.00E-14	Identity = 34.00%
	2		gi|95926610|ref|ZP_01309390.1|	Coxiella burnetii RSA 331	E-value = 2.00E-14	Identity = 34.00%
	3		gi|29654343|ref|NP_820035.1|	Coxiella burnetii RSA 493	E-value = 2.00E-14	Identity = 34.00%
	4		gi|32453612|ref|NP_861818.1|	Enterobacteria phage RB69	E-value = 2.00E-14	Identity = 31.79%
	5		gi|56750786|ref|YP_171487.1|	Synechococcus elongatus PCC 6301	E-value = 3.00E-14	Identity = 35.46%
AB1531	1			***** No matches found *****		
AB1532	1			***** No matches found *****		
AB1533	1	*selA*	gi|78777030|ref|YP_393345.1|	Sulfuromonas denitrificans ATCC 33889	E-value = 3.00E-148	Identity = 63.45%
	2		gi|149194139|ref|ZP_01871237.1|	Caminibacter mediatlanticus TB-2	E-value = 4.00E-122	Identity = 58.52%
	3		gi|34557236|ref|NP_907051.1|	Wolinella succinogenes DSM 1740	E-value = 1.00E-119	Identity = 50.23%
	4		gi|154148567|ref|YP_001406339.1|	Campylobacter hominis ATCC BAA-381	E-value = 8.00E-111	Identity = 51.02%
	5		gi|154175332|ref|YP_001408945.1|	Campylobacter curvus 525.92	E-value = 6.00E-108	Identity = 49.44%
AB1534	1	*selB*	gi|78777031|ref|YP_393346.1|	Sulfuromonas denitrificans ATCC 33889	E-value = 0	Identity = 60.76%
	2		gi|34557235|ref|NP_907050.1|	Wolinella succinogenes DSM 1740	E-value = 7.00E-152	Identity = 49.84%
	3		gi|154174928|ref|YP_001408946.1|	Campylobacter curvus 525.92	E-value = 8.00E-141	Identity = 45.89%
	4		gi|32266240|ref|NP_860272.1|	Helicobacter hepaticus ATCC 51449	E-value = 1.00E-140	Identity = 44.18%
	5		gi|157165277|ref|YP_001466209.1|	Campylobacter concisus 13826	E-value = 7.00E-139	Identity = 47.05%
AB1535	1		gi|110639876|ref|YP_680086.1|	Cytophaga hutchinsonii ATCC 33406	E-value = 7.00E-57	Identity = 33.73%
	2		gi|124008233|ref|ZP_01692930.1|	Microscilla marina ATCC 23134	E-value = 4.00E-54	Identity = 32.81%
	3		gi|118577360|ref|YP_899600.1|	Pelobacter propionicus DSM 2379	E-value = 8.00E-49	Identity = 31.34%
	4		gi|134288383|ref|YP_001110546.1|	Burkholderia vietnamiensis G4	E-value = 5.00E-45	Identity = 28.86%
	5		gi|157158381|ref|YP_001464360.1|	Escherichia coli E24377A	E-value = 8.00E-43	Identity = 29.33%
AB1536	1		gi|124008100|ref|ZP_01692798.1|	Microscilla marina ATCC 23134	E-value = 7.00E-36	Identity = 37.65%
	2		gi|149279572|ref|ZP_01885701.1|	Pedobacter sp. BAL39	E-value = 1.00E-33	Identity = 32.77%
	3		gi|29348519|ref|NP_812022.1|	Bacteroides thetaiotaomicron VPI-5482	E-value = 4.00E-30	Identity = 32.20%
	4		gi|146298424|ref|YP_001193015.1|	Flavobacterium johnsoniae UW101	E-value = 4.00E-29	Identity = 34.87%
AB1537	1			***** No matches found *****		
AB1538	1		gi|149279572|ref|ZP_01885701.1|	Pedobacter sp. BAL39	E-value = 2.00E-34	Identity = 31.76%
	2		gi|124008100|ref|ZP_01692798.1|	Microscilla marina ATCC 23134	E-value = 6.00E-34	Identity = 36.76%
	3		gi|146298424|ref|YP_001193015.1|	Flavobacterium johnsoniae UW101	E-value = 2.00E-31	Identity = 34.84%
	4		gi|29348519|ref|NP_812022.1|	Bacteroides thetaiotaomicron VPI-5482	E-value = 8.00E-30	Identity = 30.79%
	5		gi|110639215|ref|YP_679424.1|	Cytophaga hutchinsonii ATCC 33406	E-value = 1.00E-21	Identity = 29.97%
AB1539	1			***** No matches found *****		
AB1540	1		gi|78777190|ref|YP_393505.1|	Sulfuromonas denitrificans ATCC 33889	E-value = 4.00E-32	Identity = 33.88%
AB1541	1			***** No matches found *****		
AB1542	1		gi|87123470|ref|ZP_01079321.1|	Synechococcus sp. RS9917	E-value = 2.00E-25	Identity = 36.88%
	2		gi|33239495|ref|NP_874437.1|	Prochlorococcus marinus subsp. marinus str. CCMP1375	E-value = 1.00E-24	Identity = 35.76%
	3		gi|123967579|ref|YP_001008437.1|	Prochlorococcus marinus str. AS9601	E-value = 3.00E-24	Identity = 32.52%
	4		gi|126695382|ref|YP_001090268.1|	Prochlorococcus marinus str. MIT 9301	E-value = 3.00E-24	Identity = 33.13%
	5		gi|123965283|ref|YP_001010364.1|	Prochlorococcus marinus str. MIT 9515	E-value = 3.00E-24	Identity = 32.52%
AB1543	1		gi|34558072|ref|NP_907887.1|	Wolinella succinogenes DSM 1740	E-value = 5.00E-38	Identity = 28.81%
	2		gi|149194867|ref|ZP_01871961.1|	Caminibacter mediatlanticus TB-2	E-value = 2.00E-34	Identity = 33.12%
	3		gi|32267318|ref|NP_861350.1|	Helicobacter hepaticus ATCC 51449	E-value = 8.00E-32	Identity = 29.18%

	4		gi|18310647|ref|NP_562581.1|	Clostridium perfringens str. 13	E-value = 8.00E-28	Identity = 28.45%
	5		gi|46446118|ref|YP_007483.1|	Candidatus Protochlamydia amoebophila UWE25	E-value = 5.00E-27	Identity = 29.43%
AB1544	1			***** No matches found *****		
AB1545	1		gi|34557884|ref|NP_907699.1|	Wolinella succinogenes DSM 1740	E-value = 1.00E-103	Identity = 39.20%
	2		gi|89095243|ref|ZP_01168165.1|	Oceanospirillum sp. MED92	E-value = 3.00E-94	Identity = 36.53%
	3		gi|95931153|ref|ZP_01313877.1|	Desulfuromonas acetoxidans DSM 684	E-value = 9.00E-93	Identity = 35.26%
	4		gi|42779774|ref|NP_977021.1|	Bacillus cereus ATCC 10987	E-value = 3.00E-56	Identity = 29.53%
	5		gi|30018813|ref|NP_830444.1|	Bacillus cereus ATCC 14579	E-value = 5.00E-56	Identity = 29.19%
AB1546	1	*pdxH*	gi|67922765|ref|ZP_00516266.1|	Crocosphaera watsonii WH 8501	E-value = 3.00E-72	Identity = 66.51%
	2		gi|126659758|ref|ZP_01730885.1|	Cyanothece sp. CCY0110	E-value = 8.00E-72	Identity = 64.62%
	3		gi|120553098|ref|YP_957449.1|	Marinobacter aquaeolei VT8	E-value = 1.00E-69	Identity = 58.96%
	4		gi|149377548|ref|ZP_01895288.1|	Marinobacter algicola DG893	E-value = 4.00E-68	Identity = 57.55%
	5		gi|126665506|ref|ZP_01736488.1|	Marinobacter sp. ELB17	E-value = 6.00E-68	Identity = 58.14%
AB1547	1			***** No matches found *****		
AB1548	1	*dbpA*	gi|89075877|ref|ZP_01162256.1|	Photobacterium sp. SKA34	E-value = 2.00E-115	Identity = 47.35%
	2		gi|90578403|ref|ZP_01234214.1|	Vibrio angustum S14	E-value = 2.00E-114	Identity = 47.12%
	3		gi|118746345|ref|ZP_01594289.1|	Geobacter lovleyi SZ	E-value = 5.00E-113	Identity = 44.59%
	4		gi|90417199|ref|ZP_01225126.1|	marine gamma proteobacterium HTCC2207	E-value = 7.00E-112	Identity = 45.47%
	5		gi|109896916|ref|YP_660171.1|	Pseudoalteromonas atlantica T6c	E-value = 8.00E-112	Identity = 43.78%
AB1549	1		gi|154174817|ref|YP_001408117.1|	Campylobacter curvus 525.92	E-value = 2.00E-29	Identity = 43.03%
	2		gi|118474701|ref|YP_891698.1|	Campylobacter fetus subsp. fetus 82-40	E-value = 2.00E-29	Identity = 51.70%
	3		gi|34558102|ref|NP_907917.1|	Wolinella succinogenes DSM 1740	E-value = 2.00E-29	Identity = 49.10%
	4		gi|78777892|ref|YP_394207.1|	Sulfuromonas denitrificans ATCC 33889	E-value = 4.00E-29	Identity = 45.52%
	5		gi|157164921|ref|YP_001466922.1|	Campylobacter concisus 13826	E-value = 2.00E-28	Identity = 42.17%
AB1550	1		gi|118581328|ref|YP_902578.1|	Pelobacter propionicus DSM 2379	E-value = 2.00E-35	Identity = 39.82%
	2		gi|89093264|ref|ZP_01166214.1|	Oceanospirillum sp. MED92	E-value = 4.00E-35	Identity = 35.51%
	3		gi|145619338|ref|ZP_01775389.1|	Geobacter bemidjiensis Bem	E-value = 2.00E-34	Identity = 39.01%
	4		gi|118744752|ref|ZP_01592740.1|	Geobacter lovleyi SZ	E-value = 2.00E-33	Identity = 41.59%
	5		gi|78224174|ref|YP_385921.1|	Geobacter metallireducens GS-15	E-value = 4.00E-33	Identity = 41.33%
AB1551	1			***** No matches found *****		
AB1552	1	*htpG*	gi|152993477|ref|YP_001359198.1|	Sulfurovum sp. NBC37-1	E-value = 0	Identity = 65.57%
	2		gi|34558046|ref|NP_907861.1|	Wolinella succinogenes DSM 1740	E-value = 3.00E-180	Identity = 56.34%
	3		gi|42527980|ref|NP_973078.1|	Treponema denticola ATCC 35405	E-value = 1.00E-167	Identity = 52.39%
	4		gi|118475345|ref|YP_892126.1|	Campylobacter fetus subsp. fetus 82-40	E-value = 4.00E-165	Identity = 53.29%
	5		gi|109948000|ref|YP_665228.1|	Helicobacter acinonychis str. Sheeba	E-value = 3.00E-164	Identity = 52.52%
AB1553	1	*katG*	gi|152995613|ref|YP_001340448.1|	Marinomonas sp. MWYL1	E-value = 0	Identity = 67.21%
	2		gi|86139782|ref|ZP_01058348.1|	Roseobacter sp. MED193	E-value = 0	Identity = 68.26%
	3		gi|88800396|ref|ZP_01115961.1|	Reinekea sp. MED297	E-value = 0	Identity = 68.49%
	4		gi|109646677|ref|ZP_01370581.1|	Desulfitobacterium hafniense DCB-2	E-value = 0	Identity = 67.17%
	5		gi|110597767|ref|ZP_01386051.1|	Chlorobium ferrooxidans DSM 13031	E-value = 0	Identity = 68.67%
AB1555	1	*ciaB*	gi|152991765|ref|YP_001357486.1|	Sulfurovum sp. NBC37-1	E-value = 9.00E-148	Identity = 46.19%
	2		gi|152991370|ref|YP_001357092.1|	Nitratiruptor sp. SB155-2	E-value = 6.00E-144	Identity = 46.89%
	3		gi|34557578|ref|NP_907393.1|	Wolinella succinogenes DSM 1740	E-value = 1.00E-138	Identity = 43.54%
	4		gi|149195062|ref|ZP_01872154.1|	Caminibacter mediatlanticus TB-2	E-value = 2.00E-118	Identity = 43.42%
	5		gi|14547127|emb|CAC42493.1|	Campylobacter fetus	E-value = 1.00E-113	Identity = 40.33%
AB1557	1		gi|57240807|ref|ZP_00368755.1|	Campylobacter lari RM2100	E-value = 5.00E-36	Identity = 53.06%
	2		gi|118475074|ref|YP_892219.1|	Campylobacter fetus subsp. fetus 82-40	E-value = 1.00E-33	Identity = 49.02%
	3		gi|15792709|ref|NP_282532.1|	Campylobacter jejuni subsp. jejuni NCTC 11168	E-value = 9.00E-32	Identity = 47.95%
	4		gi|148926740|ref|ZP_01810420.1|	Campylobacter jejuni subsp. jejuni CG8486	E-value = 2.00E-31	Identity = 47.26%

	5		gi|86152414|ref|ZP_01070622.1|	Campylobacter jejuni subsp. jejuni 260.94	E-value = 3.00E-31	Identity = 47.95%
AB1558	1	*pqiB*	gi|91227973|ref|ZP_01262083.1|	Vibrio alginolyticus 12G01	E-value = 2.00E-94	Identity = 28.17%
	2		gi|28898385|ref|NP_797990.1|	Vibrio parahaemolyticus RIMD 2210633	E-value = 5.00E-94	Identity = 28.30%
	3		gi|149191345|ref|ZP_01869598.1|	Vibrio shilonii AK1	E-value = 2.00E-93	Identity = 28.51%
	4		gi|153835226|ref|ZP_01987893.1|	Vibrio harveyi HY01	E-value = 3.00E-93	Identity = 27.70%
	5		gi|37679826|ref|NP_934435.1|	Vibrio vulnificus YJ016	E-value = 3.00E-93	Identity = 27.42%
AB1561	1		gi|152994033|ref|YP_001359754.1|	Sulfurovum sp. NBC37-1	E-value = 9.00E-31	Identity = 53.08%
	2		gi|149197336|ref|ZP_01874387.1|	Lentisphaera araneosa HTCC2155	E-value = 4.00E-22	Identity = 42.31%
	3		gi|78777191|ref|YP_393506.1|	Sulfuromonas denitrificans ATCC 33889	E-value = 3.00E-20	Identity = 38.19%
	4		gi|24216675|ref|NP_714156.1|	Leptospira interrogans serovar Lai str. 56601	E-value = 7.00E-20	Identity = 44.74%
	5		gi|46446613|ref|YP_007978.1|	Candidatus Protochlamydia amoebophila UWE25	E-value = 2.00E-19	Identity = 39.26%
AB1562	1		gi|78777107|ref|YP_393422.1|	Sulfuromonas denitrificans ATCC 33889	E-value = 0	Identity = 58.14%
	2		gi|117923718|ref|YP_864335.1|	Magnetococcus sp. MC-1	E-value = 0	Identity = 42.19%
	3		gi|152989945|ref|YP_001355667.1|	Nitratiruptor sp. SB155-2	E-value = 0	Identity = 45.57%
	4		gi|94263998|ref|ZP_01287799.1|	delta proteobacterium MLMS-1	E-value = 0	Identity = 40.90%
	5		gi|94267023|ref|ZP_01290667.1|	delta proteobacterium MLMS-1	E-value = 0	Identity = 40.79%
AB1563	1			***** No matches found *****		
AB1564	1		gi|156109447|gb|EDO11192.1|	Bacteroides ovatus ATCC 8483	E-value = 3.00E-48	Identity = 49.32%
	2		gi|150005751|ref|YP_001300495.1|	Bacteroides vulgatus ATCC 8482	E-value = 8.00E-47	Identity = 48.11%
	3		gi|154493707|ref|ZP_02033027.1|	Parabacteroides merdae ATCC 43184	E-value = 1.00E-46	Identity = 42.66%
	4		gi|150010031|ref|YP_001304774.1|	Parabacteroides distasonis ATCC 8503	E-value = 3.00E-45	Identity = 45.73%
	5		gi|53714374|ref|YP_100366.1|	Bacteroides fragilis YCH46	E-value = 7.00E-45	Identity = 45.21%
AB1565	1		gi|32035604|ref|ZP_00135525.1|	Actinobacillus pleuropneumoniae serovar 1 str. 4074	E-value = 4.00E-44	Identity = 62.58%
	2		gi|50120130|ref|YP_049297.1|	Erwinia carotovora subsp. atroseptica SCRI1043	E-value = 3.00E-43	Identity = 61.94%
	3		gi|152969034|ref|YP_001334143.1|	Klebsiella pneumoniae subsp. pneumoniae MGH 78578	E-value = 3.00E-42	Identity = 60.65%
	4		gi|33151593|ref|NP_872946.1|	Haemophilus ducreyi 35000HP	E-value = 5.00E-42	Identity = 60.65%
	5		gi|146310621|ref|YP_001175695.1|	Enterobacter sp. 638	E-value = 2.00E-41	Identity = 60.00%
AB1566	1		gi|91776543|ref|YP_546299.1|	Methylobacillus flagellatus KT	E-value = 4.00E-31	Identity = 47.13%
	2		gi|152990897|ref|YP_001356619.1|	Nitratiruptor sp. SB155-2	E-value = 6.00E-13	Identity = 28.95%
	3		gi|15644933|ref|NP_207103.1|	Helicobacter pylori 26695	E-value = 3.00E-07	Identity = 27.46%
	4		gi|108562732|ref|YP_627048.1|	Helicobacter pylori HPAG1	E-value = 3.00E-07	Identity = 26.94%
	5		gi|15611359|ref|NP_223010.1|	Helicobacter pylori J99	E-value = 1.00E-06	Identity = 26.94%
AB1567	1		gi|149176496|ref|ZP_01855109.1|	Planctomyces maris DSM 8797	E-value = 8.00E-83	Identity = 44.38%
	2		gi|119945081|ref|YP_942761.1|	Psychromonas ingrahamii 37	E-value = 5.00E-80	Identity = 44.26%
	3		gi|124007271|ref|ZP_01691979.1|	Microscilla marina ATCC 23134	E-value = 3.00E-79	Identity = 44.70%
	4		gi|126651592|ref|ZP_01723795.1|	Bacillus sp. B14905	E-value = 1.00E-75	Identity = 42.46%
	5		gi|16078046|ref|NP_388862.1|	Bacillus subtilis subsp. subtilis str. 168	E-value = 6.00E-75	Identity = 43.48%
AB1568	1		gi|152993197|ref|YP_001358918.1|	Sulfurovum sp. NBC37-1	E-value = 9.00E-68	Identity = 51.94%
	2		gi|29611533|ref|NP_818986.1|	Bacteroides thetaiotaomicron VPI-5482	E-value = 2.00E-62	Identity = 49.08%
	3		gi|78188491|ref|YP_378829.1|	Chlorobium chlorochromatii CaD3	E-value = 1.00E-53	Identity = 43.68%
	4		gi|53711692|ref|YP_097684.1|	Bacteroides fragilis YCH46	E-value = 5.00E-53	Identity = 42.50%
	5		gi|60679934|ref|YP_210078.1|	Bacteroides fragilis NCTC 9343	E-value = 2.00E-35	Identity = 39.38%
AB1569	1		gi|28371733|gb|AAO38240.1|	Vibrio fischeri	E-value = 7.00E-15	Identity = 25.90%
AB1570	1		gi|152980668|ref|YP_001352035.1|	Janthinobacterium sp. Marseille	E-value = 1.00E-65	Identity = 56.31%
	2		gi|148265552|ref|YP_001232258.1|	Geobacter uraniumreducens Rf4	E-value = 1.00E-59	Identity = 54.90%
	3		gi|52142658|ref|YP_084170.1|	Bacillus cereus E33L	E-value = 1.00E-42	Identity = 43.75%
	4		gi|75761540|ref|ZP_00741499.1|	Bacillus thuringiensis serovar israelensis ATCC 35646	E-value = 1.00E-42	Identity = 48.96%
	5		gi|119486018|ref|ZP_01620080.1|	Lyngbya sp. PCC 8106	E-value = 2.00E-42	Identity = 43.30%
AB1572	1		gi|95929661|ref|ZP_01312403.1|	Desulfuromonas acetoxidans DSM 684	E-value = 2.00E-76	Identity = 61.29%

	2		gi|114775476|ref|ZP_01451044.1|	Mariprofundus ferrooxydans PV-1	E-value = 2.00E-76	Identity = 62.84%
	3		gi|113949368|ref|ZP_01435017.1|	Shewanella baltica OS195	E-value = 3.00E-72	Identity = 60.37%
	4		gi|146293955|ref|YP_001184379.1|	Shewanella putrefaciens CN-32	E-value = 5.00E-72	Identity = 60.37%
	5		gi|145300809|ref|YP_001143650.1|	Aeromonas salmonicida subsp. salmonicida A449	E-value = 1.00E-71	Identity = 58.99%
AB1573	1		gi|116052210|ref|YP_788946.1|	Pseudomonas aeruginosa UCBPP-PA14	E-value = 5.00E-144	Identity = 39.33%
	2		gi|126168979|gb|EAZ54490.1|	Pseudomonas aeruginosa C3719	E-value = 1.00E-143	Identity = 41.56%
	3		gi|84319024|ref|ZP_00967431.1|	Pseudomonas aeruginosa C3719	E-value = 1.00E-143	Identity = 41.56%
	4		gi|15599363|ref|NP_252857.1|	Pseudomonas aeruginosa PAO1	E-value = 1.00E-143	Identity = 41.56%
	5		gi|126196230|gb|EAZ60293.1|	Pseudomonas aeruginosa 2192	E-value = 3.00E-143	Identity = 41.41%
AB1574	1	*hicA*	gi|120555578|ref|YP_959929.1|	Marinobacter aquaeolei VT8	E-value = 1.00E-10	Identity = 54.05%
	2		gi|26990605|ref|NP_746030.1|	Pseudomonas putida KT2440	E-value = 1.00E-09	Identity = 50.00%
	3		gi|33152053|ref|NP_873406.1|	Haemophilus ducreyi 35000HP	E-value = 4.00E-09	Identity = 48.65%
	4		gi|149115898|ref|ZP_01842634.1|	Shewanella baltica OS223	E-value = 4.00E-09	Identity = 48.65%
	5		gi|113948537|ref|ZP_01434196.1|	Shewanella baltica OS195	E-value = 3.00E-08	Identity = 48.65%
AB1575	1	*hicB*	gi|146298195|ref|YP_001192786.1|	Flavobacterium johnsoniae UW101	E-value = 1.00E-25	Identity = 59.80%
	2		gi|91203238|emb|CAJ72877.1|	Candidatus Kuenenia stuttgartiensis	E-value = 7.00E-21	Identity = 41.67%
	3		gi|92113802|ref|YP_573730.1|	Chromohalobacter salexigens DSM 3043	E-value = 2.00E-20	Identity = 44.90%
	4		gi|119510524|ref|ZP_01629655.1|	Nodularia spumigena CCY9414	E-value = 2.00E-19	Identity = 43.69%
	5		gi|26990604|ref|NP_746029.1|	Pseudomonas putida KT2440	E-value = 2.00E-19	Identity = 45.26%
AB1576	1		gi|34557007|ref|NP_906822.1|	Wolinella succinogenes DSM 1740	E-value = 1.00E-32	Identity = 30.06%
	2		gi|34557769|ref|NP_907584.1|	Wolinella succinogenes DSM 1740	E-value = 2.00E-25	Identity = 26.81%
	3		gi|90021090|ref|YP_526917.1|	Saccharophagus degradans 2-40	E-value = 2.00E-18	Identity = 25.16%
	4		gi|156112456|gb|EDO14201.1|	Bacteroides ovatus ATCC 8483	E-value = 2.00E-14	Identity = 25.00%
	5		gi|154493462|ref|ZP_02032782.1|	Parabacteroides merdae ATCC 43184	E-value = 4.00E-12	Identity = 25.00%
AB1577	1		gi|34557006|ref|NP_906821.1|	Wolinella succinogenes DSM 1740	E-value = 7.00E-22	Identity = 39.47%
	2		gi|34557770|ref|NP_907585.1|	Wolinella succinogenes DSM 1740	E-value = 8.00E-15	Identity = 40.67%
	3		gi|30248569|ref|NP_840639.1|	Nitrosomonas europaea ATCC 19718	E-value = 4.00E-14	Identity = 32.26%
	4		gi|30250072|ref|NP_842142.1|	Nitrosomonas europaea ATCC 19718	E-value = 5.00E-13	Identity = 30.72%
	5		gi|70731436|ref|YP_261177.1|	Pseudomonas fluorescens Pf-5	E-value = 6.00E-13	Identity = 31.03%
AB1578	1	*speB*	gi|42521974|ref|NP_967354.1|	Bdellovibrio bacteriovorus HD100	E-value = 9.00E-70	Identity = 43.11%
	2		gi|150026245|ref|YP_001297071.1|	Flavobacterium psychrophilum JIP02/86	E-value = 4.00E-69	Identity = 43.96%
	3		gi|108761940|ref|YP_632602.1|	Myxococcus xanthus DK 1622	E-value = 7.00E-68	Identity = 42.64%
	4		gi|23128506|ref|ZP_00110351.1|	Nostoc punctiforme PCC 73102	E-value = 2.00E-67	Identity = 43.98%
	5		gi|119510848|ref|ZP_01629973.1|	Nodularia spumigena CCY9414	E-value = 7.00E-66	Identity = 41.34%
AB1579	1	*leuA2*	gi|15893565|ref|NP_346914.1|	Clostridium acetobutylicum ATCC 824	E-value = 1.00E-135	Identity = 53.58%
	2		gi|21674916|ref|NP_662981.1|	Chlorobium tepidum TLS	E-value = 2.00E-135	Identity = 50.93%
	3		gi|13472480|ref|NP_104047.1|	Mesorhizobium loti MAFF303099	E-value = 3.00E-134	Identity = 51.53%
	4		gi|22001756|sp|Q8UD63|LEU1_AGRT5	Agrobacterium tumefaciens str. C58	E-value = 2.00E-133	Identity = 50.94%
	5		gi|38258153|sp|Q8FZC4|LEU1_BRUSU	Brucella suis	E-value = 4.00E-133	Identity = 51.29%
AB1580	1		gi|149194303|ref|ZP_01871400.1|	Caminibacter mediatlanticus TB-2	E-value = 1.00E-33	Identity = 37.83%
	2		gi|88799777|ref|ZP_01115351.1|	Reinekea sp. MED297	E-value = 4.00E-28	Identity = 27.41%
	3		gi|120556759|ref|YP_961110.1|	Marinobacter aquaeolei VT8	E-value = 2.00E-25	Identity = 27.78%
	4		gi|149377021|ref|ZP_01894772.1|	Marinobacter algicola DG893	E-value = 3.00E-22	Identity = 25.64%
	5		gi|95930609|ref|ZP_01313343.1|	Desulfuromonas acetoxidans DSM 684	E-value = 8.00E-22	Identity = 26.89%
AB1581	1		gi|121540582|ref|ZP_01672343.1|	Candidatus Desulfococcus oleovorans Hxd3	E-value = 2.00E-27	Identity = 54.13%
	2		gi|78223703|ref|YP_385450.1|	Geobacter metallireducens GS-15	E-value = 6.00E-26	Identity = 57.28%
	3		gi|148265402|ref|YP_001232108.1|	Geobacter uraniumreducens Rf4	E-value = 3.00E-24	Identity = 56.31%
	4		gi|119511253|ref|ZP_01630369.1|	Nodularia spumigena CCY9414	E-value = 1.00E-23	Identity = 53.40%
	5		gi|78773873|gb|ABB51222.1|	Arthrospira platensis	E-value = 2.00E-23	Identity = 54.90%

AB1582	1		gi|121540581|ref|ZP_01672342.1|	Candidatus Desulfococcus oleovorans Hxd3	E-value = 1.00E-07	Identity = 40.91%
	2		gi|116329237|ref|YP_798957.1|	Leptospira borgpetersenii serovar Hardjo-bovis L550	E-value = 9.00E-06	Identity = 42.86%
	3		gi|119511254|ref|ZP_01630370.1|	Nodularia spumigena CCY9414	E-value = 9.00E-06	Identity = 40.00%
AB1583	1		gi|34557928|ref|NP_907743.1|	Wolinella succinogenes DSM 1740	E-value = 3.00E-19	Identity = 26.06%
	2		gi|118475050|ref|YP_892365.1|	Campylobacter fetus subsp. fetus 82-40	E-value = 8.00E-19	Identity = 28.47%
	3		gi|17230117|ref|NP_486665.1|	Nostoc sp. PCC 7120	E-value = 1.00E-15	Identity = 25.76%
	4		gi|146301060|ref|YP_001195651.1|	Flavobacterium johnsoniae UW101	E-value = 7.00E-12	Identity = 26.07%
	5		gi|150024940|ref|YP_001295766.1|	Flavobacterium psychrophilum JIP02/86	E-value = 2.00E-11	Identity = 26.56%
AB1584	1		gi|34557929|ref|NP_907744.1|	Wolinella succinogenes DSM 1740	E-value = 3.00E-74	Identity = 28.91%
	2		gi|78356643|ref|YP_388092.1|	Desulfovibrio desulfuricans G20	E-value = 7.00E-69	Identity = 30.47%
	3		gi|28192385|gb|AAL65282.1|	Pseudomonas fluorescens	E-value = 1.00E-59	Identity = 25.34%
	4		gi|17231518|ref|NP_488066.1|	Nostoc sp. PCC 7120	E-value = 6.00E-58	Identity = 28.81%
	5		gi|83592201|ref|YP_425953.1|	Rhodospirillum rubrum ATCC 11170	E-value = 1.00E-57	Identity = 28.42%
AB1585	1	*sodB*	gi|153885654|ref|ZP_02006810.1|	Ralstonia pickettii 12D	E-value = 7.00E-73	Identity = 69.79%
	2		gi|121527722|ref|ZP_01660336.1|	Ralstonia pickettii 12J	E-value = 7.00E-73	Identity = 69.79%
	3		gi|134095671|ref|YP_001100746.1|	Herminiimonas arsenicoxydans	E-value = 1.00E-72	Identity = 68.75%
	4		gi|83749054|ref|ZP_00946060.1|	Ralstonia solanacearum UW551	E-value = 1.00E-72	Identity = 70.31%
	5		gi|152981017|ref|YP_001354279.1|	Janthinobacterium sp. Marseille	E-value = 2.00E-71	Identity = 67.71%
AB1586	1		gi|153801520|ref|ZP_01956106.1|	Vibrio cholerae MZO-3	E-value = 1.00E-112	Identity = 52.66%
	2		gi|15640294|ref|NP_229921.1|	Vibrio cholerae O1 biovar eltor str. N16961	E-value = 2.00E-112	Identity = 52.66%
	3		gi|121727056|ref|ZP_01680247.1|	Vibrio cholerae V52	E-value = 2.00E-112	Identity = 52.66%
	4		gi|150423676|gb|EDN15618.1|	Vibrio cholerae AM-19226	E-value = 3.00E-112	Identity = 52.66%
	5		gi|153818301|ref|ZP_01970968.1|	Vibrio cholerae NCTC 8457	E-value = 4.00E-112	Identity = 52.39%
AB1587	1	*ppa*	gi|91791842|ref|YP_561493.1|	Shewanella denitrificans OS217	E-value = 4.00E-119	Identity = 68.30%
	2		gi|157375310|ref|YP_001473910.1|	Shewanella sediminis HAW-EB3	E-value = 4.00E-119	Identity = 68.30%
	3		gi|114564713|ref|YP_752227.1|	Shewanella frigidimarina NCIMB 400	E-value = 3.00E-118	Identity = 67.97%
	4		gi|127512903|ref|YP_001094100.1|	Shewanella loihica PV-4	E-value = 3.00E-118	Identity = 66.67%
	5		gi|24375675|ref|NP_719718.1|	Shewanella oneidensis MR-1	E-value = 4.00E-118	Identity = 67.32%
AB1588	1		gi|154148744|ref|YP_001406896.1|	Campylobacter hominis ATCC BAA-381	E-value = 8.00E-48	Identity = 54.27%
	2		gi|106894504|ref|ZP_01361623.1|	Clostridium sp. OhILAs	E-value = 7.00E-26	Identity = 39.47%
	3		gi|150400151|ref|YP_001323918.1|	Methanococcus vannielii SB	E-value = 8.00E-26	Identity = 37.13%
	4		gi|121534575|ref|ZP_01666397.1|	Thermosinus carboxydivorans Nor1	E-value = 9.00E-26	Identity = 32.72%
	5		gi|154503451|ref|ZP_02040511.1|	Ruminococcus gnavus ATCC 29149	E-value = 1.00E-24	Identity = 36.92%
AB1589	1		gi|152991195|ref|YP_001356917.1|	Nitratiruptor sp. SB155-2	E-value = 1.00E-33	Identity = 46.20%
	2		gi|78776774|ref|YP_393089.1|	Sulfuromonas denitrificans ATCC 33889	E-value = 4.00E-30	Identity = 42.95%
	3		gi|152992311|ref|YP_001358032.1|	Sulfurovum sp. NBC37-1	E-value = 2.00E-29	Identity = 44.38%
	4		gi|34557131|ref|NP_906946.1|	Wolinella succinogenes DSM 1740	E-value = 9.00E-28	Identity = 40.96%
	5		gi|86158564|ref|YP_465349.1|	Anaeromyxobacter dehalogenans 2CP-C	E-value = 2.00E-19	Identity = 34.57%
AB1590	1	*aspA*	gi|2644959|emb|CAA05764.1|	Wolinella succinogenes	E-value = 1.00E-172	Identity = 68.12%
	2		gi|34557077|ref|NP_906892.1|	Wolinella succinogenes DSM 1740	E-value = 2.00E-172	Identity = 68.12%
	3		gi|32266221|ref|NP_860253.1|	Helicobacter hepaticus ATCC 51449	E-value = 2.00E-164	Identity = 62.14%
	4		gi|118474906|ref|YP_892771.1|	Campylobacter fetus subsp. fetus 82-40	E-value = 3.00E-163	Identity = 61.71%
	5		gi|109947600|ref|YP_664828.1|	Helicobacter acinonychis str. Sheeba	E-value = 1.00E-162	Identity = 61.93%
AB1591	1		gi|34557780|ref|NP_907595.1|	Wolinella succinogenes DSM 1740	E-value = 4.00E-39	Identity = 40.99%
	2		gi|118475245|ref|YP_891579.1|	Campylobacter fetus subsp. fetus 82-40	E-value = 8.00E-31	Identity = 35.00%
	3		gi|15792806|ref|NP_282629.1|	Campylobacter jejuni subsp. jejuni NCTC 11168	E-value = 1.00E-30	Identity = 35.75%
	4		gi|57238514|ref|YP_179645.1|	Campylobacter jejuni subsp. jejuni RM1221	E-value = 2.00E-30	Identity = 35.75%
	5		gi|78778185|ref|YP_394500.1|	Sulfuromonas denitrificans ATCC 33889	E-value = 4.00E-30	Identity = 39.11%
AB1592	1		gi|90023443|ref|YP_529270.1|	Saccharophagus degradans 2-40	E-value = 2.00E-14	Identity = 27.72%

AB1593	1		gi|88704151|ref|ZP_01101866.1|	gamma proteobacterium KT 71	E-value = 9.00E-148	Identity = 59.13%
	2		gi|146305176|ref|YP_001185641.1|	Pseudomonas mendocina ymp	E-value = 7.00E-139	Identity = 57.39%
	3		gi|152996664|ref|YP_001341499.1|	Marinomonas sp. MWYL1	E-value = 2.00E-133	Identity = 54.37%
	4		gi|146280590|ref|YP_001170743.1|	Pseudomonas stutzeri A1501	E-value = 6.00E-130	Identity = 58.32%
	5		gi|28872605|ref|NP_795224.1|	Pseudomonas syringae pv. tomato str. DC3000	E-value = 3.00E-126	Identity = 50.96%
AB1594	1	*pfs*	gi|152993338|ref|YP_001359059.1|	Sulfurovum sp. NBC37-1	E-value = 2.00E-80	Identity = 64.98%
	2		gi|152990953|ref|YP_001356675.1|	Nitratiruptor sp. SB155-2	E-value = 2.00E-75	Identity = 59.76%
	3		gi|78777589|ref|YP_393904.1|	Sulfuromonas denitrificans ATCC 33889	E-value = 2.00E-74	Identity = 63.60%
	4		gi|34556949|ref|NP_906764.1|	Wolinella succinogenes DSM 1740	E-value = 8.00E-74	Identity = 61.67%
	5		gi|157164087|ref|YP_001466355.1|	Campylobacter concisus 13826	E-value = 1.00E-68	Identity = 58.41%
AB1595	1	*fabD*	gi|152993339|ref|YP_001359060.1|	Sulfurovum sp. NBC37-1	E-value = 4.00E-99	Identity = 60.13%
	2		gi|152990955|ref|YP_001356677.1|	Nitratiruptor sp. SB155-2	E-value = 1.00E-94	Identity = 54.22%
	3		gi|78777590|ref|YP_393905.1|	Sulfuromonas denitrificans ATCC 33889	E-value = 1.00E-93	Identity = 57.56%
	4		gi|118474647|ref|YP_892650.1|	Campylobacter fetus subsp. fetus 82-40	E-value = 8.00E-87	Identity = 56.03%
	5		gi|157164479|ref|YP_001466354.1|	Campylobacter concisus 13826	E-value = 3.00E-86	Identity = 54.69%
AB1596	1	*slyD*	gi|152990956|ref|YP_001356678.1|	Nitratiruptor sp. SB155-2	E-value = 8.00E-51	Identity = 66.88%
	2		gi|152993342|ref|YP_001359063.1|	Sulfurovum sp. NBC37-1	E-value = 1.00E-41	Identity = 58.54%
	3		gi|57505316|ref|ZP_00371245.1|	Campylobacter upsaliensis RM3195	E-value = 2.00E-38	Identity = 52.35%
	4		gi|57168716|ref|ZP_00367848.1|	Campylobacter coli RM2228	E-value = 2.00E-37	Identity = 52.35%
	5		gi|153951916|ref|YP_001397369.1|	Campylobacter jejuni subsp. doylei 269.97	E-value = 2.00E-37	Identity = 52.35%
AB1597	1		gi|152990957|ref|YP_001356679.1|	Nitratiruptor sp. SB155-2	E-value = 2.00E-24	Identity = 31.92%
	2		gi|152993345|ref|YP_001359066.1|	Sulfurovum sp. NBC37-1	E-value = 1.00E-23	Identity = 33.33%
	3		gi|57168717|ref|ZP_00367849.1|	Campylobacter coli RM2228	E-value = 5.00E-22	Identity = 31.94%
	4		gi|78777593|ref|YP_393908.1|	Sulfuromonas denitrificans ATCC 33889	E-value = 3.00E-21	Identity = 30.95%
	5		gi|57240735|ref|ZP_00368683.1|	Campylobacter lari RM2100	E-value = 6.00E-21	Identity = 27.63%
AB1598	1		gi|78777594|ref|YP_393909.1|	Sulfuromonas denitrificans ATCC 33889	E-value = 5.00E-30	Identity = 46.67%
	2		gi|157164304|ref|YP_001466351.1|	Campylobacter concisus 13826	E-value = 4.00E-28	Identity = 46.55%
	3		gi|152990958|ref|YP_001356680.1|	Nitratiruptor sp. SB155-2	E-value = 9.00E-27	Identity = 45.00%
	4		gi|34556944|ref|NP_906759.1|	Wolinella succinogenes DSM 1740	E-value = 2.00E-26	Identity = 44.51%
	5		gi|154174275|ref|YP_001408803.1|	Campylobacter curvus 525.92	E-value = 2.00E-25	Identity = 44.87%
AB1599	1	*tolB*	gi|34556943|ref|NP_906758.1|	Wolinella succinogenes DSM 1740	E-value = 3.00E-97	Identity = 45.39%
	2		gi|152990959|ref|YP_001356681.1|	Nitratiruptor sp. SB155-2	E-value = 9.00E-90	Identity = 43.85%
	3		gi|154173809|ref|YP_001408804.1|	Campylobacter curvus 525.92	E-value = 1.00E-89	Identity = 43.19%
	4		gi|157164610|ref|YP_001466350.1|	Campylobacter concisus 13826	E-value = 3.00E-88	Identity = 42.73%
	5		gi|118474869|ref|YP_892654.1|	Campylobacter fetus subsp. fetus 82-40	E-value = 8.00E-87	Identity = 44.44%
AB1600	1		gi|34556942|ref|NP_906757.1|	Wolinella succinogenes DSM 1740	E-value = 4.00E-10	Identity = 31.33%
AB1601	1	*exbD3*	gi|152990961|ref|YP_001356683.1|	Nitratiruptor sp. SB155-2	E-value = 3.00E-35	Identity = 59.69%
	2		gi|34556941|ref|NP_906756.1|	Wolinella succinogenes DSM 1740	E-value = 2.00E-34	Identity = 58.59%
	3		gi|32265931|ref|NP_859963.1|	Helicobacter hepaticus ATCC 51449	E-value = 3.00E-28	Identity = 52.38%
	4		gi|154148539|ref|YP_001406271.1|	Campylobacter hominis ATCC BAA-381	E-value = 6.00E-27	Identity = 47.73%
	5		gi|118474578|ref|YP_892656.1|	Campylobacter fetus subsp. fetus 82-40	E-value = 2.00E-26	Identity = 51.54%
AB1602	1	*exbB3*	gi|34556940|ref|NP_906755.1|	Wolinella succinogenes DSM 1740	E-value = 2.00E-34	Identity = 45.41%
	2		gi|152993352|ref|YP_001359073.1|	Sulfurovum sp. NBC37-1	E-value = 8.00E-34	Identity = 52.43%
	3		gi|109947171|ref|YP_664399.1|	Helicobacter acinonychis str. Sheeba	E-value = 2.00E-29	Identity = 43.09%
	4		gi|57240730|ref|ZP_00368678.1|	Campylobacter lari RM2100	E-value = 2.00E-28	Identity = 48.07%
	5		gi|108563493|ref|YP_627809.1|	Helicobacter pylori HPAG1	E-value = 2.00E-28	Identity = 42.02%
AB1603	1	*atpC*	gi|118475189|ref|YP_892658.1|	Campylobacter fetus subsp. fetus 82-40	E-value = 3.00E-25	Identity = 56.35%
	2		gi|34556939|ref|NP_906754.1|	Wolinella succinogenes DSM 1740	E-value = 4.00E-24	Identity = 54.76%
	3		gi|152993353|ref|YP_001359074.1|	Sulfurovum sp. NBC37-1	E-value = 7.00E-24	Identity = 58.20%

	4		gi|152990963|ref|YP_001356685.1|	Nitratiruptor sp. SB155-2	E-value = 8.00E-24	Identity = 53.97%
	5		gi|157163910|ref|YP_001466346.1|	Campylobacter concisus 13826	E-value = 4.00E-23	Identity = 50.00%
AB1605	1	*atpD*	gi|153951038|ref|YP_001397361.1|	Campylobacter jejuni subsp. doylei 269.97	E-value = 0	Identity = 82.80%
	2		gi|154173677|ref|YP_001408809.1|	Campylobacter curvus 525.92	E-value = 0	Identity = 83.23%
	3		gi|157164647|ref|YP_001466345.1|	Campylobacter concisus 13826	E-value = 0	Identity = 83.01%
	4		gi|34556938|ref|NP_906753.1|	Wolinella succinogenes DSM 1740	E-value = 0	Identity = 83.23%
	5		gi|57240728|ref|ZP_00368676.1|	Campylobacter lari RM2100	E-value = 0	Identity = 83.23%
AB1606	1	*atpG*	gi|34556937|ref|NP_906752.1|	Wolinella succinogenes DSM 1740	E-value = 2.00E-81	Identity = 57.09%
	2		gi|152990965|ref|YP_001356687.1|	Nitratiruptor sp. SB155-2	E-value = 3.00E-81	Identity = 57.29%
	3		gi|154174108|ref|YP_001408810.1|	Campylobacter curvus 525.92	E-value = 2.00E-77	Identity = 54.24%
	4		gi|157164986|ref|YP_001466344.1|	Campylobacter concisus 13826	E-value = 7.00E-77	Identity = 53.22%
	5		gi|118474696|ref|YP_892660.1|	Campylobacter fetus subsp. fetus 82-40	E-value = 6.00E-76	Identity = 54.39%
AB1607	1	*atpA*	gi|78777602|ref|YP_393917.1|	Sulfuromonas denitrificans ATCC 33889	E-value = 0	Identity = 78.09%
	2		gi|34556936|ref|NP_906751.1|	Wolinella succinogenes DSM 1740	E-value = 0	Identity = 77.80%
	3		gi|152990966|ref|YP_001356688.1|	Nitratiruptor sp. SB155-2	E-value = 0	Identity = 76.59%
	4		gi|32265926|ref|NP_859958.1|	Helicobacter hepaticus ATCC 51449	E-value = 0	Identity = 76.19%
	5		gi|118474260|ref|YP_892661.1|	Campylobacter fetus subsp. fetus 82-40	E-value = 0	Identity = 76.59%
AB1608	1	*atpH*	gi|157163953|ref|YP_001466342.1|	Campylobacter concisus 13826	E-value = 7.00E-31	Identity = 43.18%
	2		gi|154175315|ref|YP_001408812.1|	Campylobacter curvus 525.92	E-value = 1.00E-30	Identity = 42.61%
	3		gi|118474956|ref|YP_892662.1|	Campylobacter fetus subsp. fetus 82-40	E-value = 2.00E-29	Identity = 42.05%
	4		gi|34556935|ref|NP_906750.1|	Wolinella succinogenes DSM 1740	E-value = 8.00E-26	Identity = 36.57%
	5		gi|154148856|ref|YP_001406265.1|	Campylobacter hominis ATCC BAA-381	E-value = 6.00E-25	Identity = 40.34%
AB1609	1	*atpF*	gi|34556934|ref|NP_906749.1|	Wolinella succinogenes DSM 1740	E-value = 1.00E-24	Identity = 44.12%
	2		gi|32265924|ref|NP_859956.1|	Helicobacter hepaticus ATCC 51449	E-value = 7.00E-20	Identity = 35.09%
	3		gi|157164079|ref|YP_001466341.1|	Campylobacter concisus 13826	E-value = 3.00E-19	Identity = 38.24%
	4		gi|154175540|ref|YP_001408813.1|	Campylobacter curvus 525.92	E-value = 4.00E-19	Identity = 33.93%
	5		gi|57504653|ref|ZP_00370731.1|	Campylobacter coli RM2228	E-value = 2.00E-18	Identity = 39.41%
AB1610	1	*atpF'*	gi|34556933|ref|NP_906748.1|	Wolinella succinogenes DSM 1740	E-value = 1.00E-18	Identity = 43.88%
	2		gi|78777605|ref|YP_393920.1|	Sulfuromonas denitrificans ATCC 33889	E-value = 3.00E-16	Identity = 40.71%
	3		gi|152990969|ref|YP_001356691.1|	Nitratiruptor sp. SB155-2	E-value = 3.00E-15	Identity = 38.57%
	4		gi|32265923|ref|NP_859955.1|	Helicobacter hepaticus ATCC 51449	E-value = 1.00E-13	Identity = 35.97%
	5		gi|157165108|ref|YP_001466340.1|	Campylobacter concisus 13826	E-value = 3.00E-12	Identity = 37.86%
AB1611	1	*parB*	gi|34556932|ref|NP_906747.1|	Wolinella succinogenes DSM 1740	E-value = 3.00E-60	Identity = 50.18%
	2		gi|32265922|ref|NP_859954.1|	Helicobacter hepaticus ATCC 51449	E-value = 1.00E-59	Identity = 56.90%
	3		gi|152993360|ref|YP_001359081.1|	Sulfurovum sp. NBC37-1	E-value = 7.00E-58	Identity = 52.05%
	4		gi|152990970|ref|YP_001356692.1|	Nitratiruptor sp. SB155-2	E-value = 1.00E-53	Identity = 49.45%
	5		gi|57240722|ref|ZP_00368670.1|	Campylobacter lari RM2100	E-value = 2.00E-53	Identity = 46.98%
AB1612	1	*parA*	gi|78777607|ref|YP_393922.1|	Sulfuromonas denitrificans ATCC 33889	E-value = 1.00E-100	Identity = 73.26%
	2		gi|152990971|ref|YP_001356693.1|	Nitratiruptor sp. SB155-2	E-value = 8.00E-99	Identity = 72.76%
	3		gi|34556931|ref|NP_906746.1|	Wolinella succinogenes DSM 1740	E-value = 6.00E-98	Identity = 70.93%
	4		gi|118474892|ref|YP_892666.1|	Campylobacter fetus subsp. fetus 82-40	E-value = 4.00E-92	Identity = 66.15%
	5		gi|157164247|ref|YP_001466338.1|	Campylobacter concisus 13826	E-value = 2.00E-91	Identity = 68.99%
AB1613	1	*birA*	gi|152990972|ref|YP_001356694.1|	Nitratiruptor sp. SB155-2	E-value = 2.00E-53	Identity = 49.29%
	2		gi|154174735|ref|YP_001408817.1|	Campylobacter curvus 525.92	E-value = 2.00E-51	Identity = 52.43%
	3		gi|78777608|ref|YP_393923.1|	Sulfuromonas denitrificans ATCC 33889	E-value = 2.00E-51	Identity = 50.71%
	4		gi|157164230|ref|YP_001466337.1|	Campylobacter concisus 13826	E-value = 3.00E-47	Identity = 45.02%
	5		gi|118474785|ref|YP_892667.1|	Campylobacter fetus subsp. fetus 82-40	E-value = 7.00E-46	Identity = 45.50%
AB1614	1			***** No matches found *****		
AB1615	1		gi|56461212|ref|YP_156493.1|	Idiomarina loihiensis L2TR	E-value = 7.00E-28	Identity = 31.49%

	2		gi|85711171|ref|ZP_01042231.1|	Idiomarina baltica OS145	E-value = 2.00E-27	Identity = 32.78%
	3		gi|68551939|ref|ZP_00591332.1|	Prosthecochloris aestuarii DSM 271	E-value = 7.00E-27	Identity = 28.93%
	4		gi|15606582|ref|NP_213962.1|	Aquifex aeolicus VF5	E-value = 1.00E-26	Identity = 29.96%
	5		gi|34557125|ref|NP_906940.1|	Wolinella succinogenes DSM 1740	E-value = 4.00E-26	Identity = 29.90%
AB1616	1	*fmt*	gi|152990973|ref|YP_001356695.1|	Nitratiruptor sp. SB155-2	E-value = 3.00E-93	Identity = 55.67%
	2		gi|34556929|ref|NP_906744.1|	Wolinella succinogenes DSM 1740	E-value = 3.00E-83	Identity = 51.82%
	3		gi|78777609|ref|YP_393924.1|	Sulfuromonas denitrificans ATCC 33889	E-value = 2.00E-82	Identity = 52.63%
	4		gi|57240719|ref|ZP_00368667.1|	Campylobacter lari RM2100	E-value = 5.00E-79	Identity = 52.81%
	5		gi|149194001|ref|ZP_01871099.1|	Caminibacter mediatlanticus TB-2	E-value = 4.00E-78	Identity = 48.49%
AB1617	1	*proB*	gi|152990975|ref|YP_001356697.1|	Nitratiruptor sp. SB155-2	E-value = 8.00E-75	Identity = 60.64%
	2		gi|34556928|ref|NP_906743.1|	Wolinella succinogenes DSM 1740	E-value = 3.00E-73	Identity = 56.42%
	3		gi|154339603|ref|XP_001562493.1|	Leishmania braziliensis	E-value = 7.00E-68	Identity = 53.36%
	4		gi|154339609|ref|XP_001562496.1|	Leishmania braziliensis	E-value = 7.00E-68	Identity = 53.36%
	5		gi|68127346|emb|CAJ05678.1|	Leishmania major	E-value = 3.00E-66	Identity = 52.57%
AB1618	1	*obg*	gi|152990976|ref|YP_001356698.1|	Nitratiruptor sp. SB155-2	E-value = 5.00E-118	Identity = 62.53%
	2		gi|118474188|ref|YP_892670.1|	Campylobacter fetus subsp. fetus 82-40	E-value = 5.00E-110	Identity = 63.23%
	3		gi|34556927|ref|NP_906742.1|	Wolinella succinogenes DSM 1740	E-value = 6.00E-109	Identity = 60.78%
	4		gi|78777611|ref|YP_393926.1|	Sulfuromonas denitrificans ATCC 33889	E-value = 9.00E-106	Identity = 56.16%
	5		gi|154174924|ref|YP_001408821.1|	Campylobacter curvus 525.92	E-value = 6.00E-102	Identity = 60.93%
AB1619	1	*rpmA*	gi|34556926|ref|NP_906741.1|	Wolinella succinogenes DSM 1740	E-value = 3.00E-27	Identity = 84.71%
	2		gi|149194856|ref|ZP_01871950.1|	Caminibacter mediatlanticus TB-2	E-value = 2.00E-26	Identity = 87.80%
	3		gi|154173918|ref|YP_001408822.1|	Campylobacter curvus 525.92	E-value = 3.00E-26	Identity = 83.53%
	4		gi|57240717|ref|ZP_00368665.1|	Campylobacter lari RM2100	E-value = 3.00E-26	Identity = 85.71%
	5		gi|15791483|ref|NP_281306.1|	Campylobacter jejuni subsp. jejuni NCTC 11168	E-value = 5.00E-26	Identity = 84.52%
AB1620	1	*rplU*	gi|118474453|ref|YP_892672.1|	Campylobacter fetus subsp. fetus 82-40	E-value = 4.00E-20	Identity = 60.00%
	2		gi|154148578|ref|YP_001406889.1|	Campylobacter hominis ATCC BAA-381	E-value = 3.00E-19	Identity = 58.10%
	3		gi|152993367|ref|YP_001359088.1|	Sulfurovum sp. NBC37-1	E-value = 3.00E-19	Identity = 60.00%
	4		gi|78777613|ref|YP_393928.1|	Sulfuromonas denitrificans ATCC 33889	E-value = 3.00E-19	Identity = 61.90%
	5		gi|157165737|ref|YP_001466320.1|	Campylobacter concisus 13826	E-value = 6.00E-19	Identity = 57.69%
AB1621	1	*dnaG*	gi|152989961|ref|YP_001355683.1|	Nitratiruptor sp. SB155-2	E-value = 5.00E-161	Identity = 53.14%
	2		gi|78776224|ref|YP_392539.1|	Sulfuromonas denitrificans ATCC 33889	E-value = 4.00E-157	Identity = 52.18%
	3		gi|152993832|ref|YP_001359553.1|	Sulfurovum sp. NBC37-1	E-value = 6.00E-152	Identity = 53.02%
	4		gi|34558196|ref|NP_908011.1|	Wolinella succinogenes DSM 1740	E-value = 4.00E-150	Identity = 48.91%
	5		gi|32266698|ref|NP_860730.1|	Helicobacter hepaticus ATCC 51449	E-value = 3.00E-144	Identity = 51.17%
AB1622	1		gi|78778220|ref|YP_394535.1|	Sulfuromonas denitrificans ATCC 33889	E-value = 2.00E-43	Identity = 37.39%
	2		gi|34558195|ref|NP_908010.1|	Wolinella succinogenes DSM 1740	E-value = 3.00E-40	Identity = 35.51%
	3		gi|32266196|ref|NP_860228.1|	Helicobacter hepaticus ATCC 51449	E-value = 2.00E-37	Identity = 30.14%
	4		gi|157165736|ref|YP_001466238.1|	Campylobacter concisus 13826	E-value = 3.00E-36	Identity = 29.71%
	5		gi|154174836|ref|YP_001409226.1|	Campylobacter curvus 525.92	E-value = 9.00E-36	Identity = 29.71%
AB1623	1	*rnc*	gi|34558193|ref|NP_908008.1|	Wolinella succinogenes DSM 1740	E-value = 2.00E-71	Identity = 64.84%
	2		gi|152993835|ref|YP_001359556.1|	Sulfurovum sp. NBC37-1	E-value = 3.00E-71	Identity = 67.26%
	3		gi|78778218|ref|YP_394533.1|	Sulfuromonas denitrificans ATCC 33889	E-value = 4.00E-71	Identity = 67.13%
	4		gi|57241757|ref|ZP_00369702.1|	Campylobacter lari RM2100	E-value = 2.00E-63	Identity = 61.11%
	5		gi|57238646|ref|YP_179777.1|	Campylobacter jejuni subsp. jejuni RM1221	E-value = 4.00E-63	Identity = 57.92%
AB1624	1	*aroC*	gi|152993836|ref|YP_001359557.1|	Sulfurovum sp. NBC37-1	E-value = 4.00E-135	Identity = 67.13%
	2		gi|78778217|ref|YP_394532.1|	Sulfuromonas denitrificans ATCC 33889	E-value = 7.00E-133	Identity = 66.48%
	3		gi|152989965|ref|YP_001355687.1|	Nitratiruptor sp. SB155-2	E-value = 5.00E-131	Identity = 66.85%
	4		gi|149194958|ref|ZP_01872051.1|	Caminibacter mediatlanticus TB-2	E-value = 6.00E-129	Identity = 66.11%
	5		gi|32266199|ref|NP_860231.1|	Helicobacter hepaticus ATCC 51449	E-value = 2.00E-125	Identity = 62.01%

AB1625	1		gi|148253338|ref|YP_001237923.1|	Bradyrhizobium sp. BTAi1	E-value = 2.00E-54	Identity = 43.29%
	2		gi|34557984|ref|NP_907799.1|	Wolinella succinogenes DSM 1740	E-value = 5.00E-53	Identity = 43.81%
	3		gi|13472673|ref|NP_104240.1|	Mesorhizobium loti MAFF303099	E-value = 8.00E-52	Identity = 43.50%
	4		gi|146300635|ref|YP_001195226.1|	Flavobacterium johnsoniae UW101	E-value = 1.00E-51	Identity = 47.22%
	5		gi|32265720|ref|NP_859752.1|	Helicobacter hepaticus ATCC 51449	E-value = 2.00E-43	Identity = 40.32%
AB1626	1		gi|157165575|ref|YP_001466520.1|	Campylobacter concisus 13826	E-value = 2.00E-88	Identity = 56.79%
	2		gi|152992029|ref|YP_001357750.1|	Sulfurovum sp. NBC37-1	E-value = 1.00E-86	Identity = 61.29%
	3		gi|154175155|ref|YP_001408666.1|	Campylobacter curvus 525.92	E-value = 6.00E-82	Identity = 57.19%
	4		gi|154148635|ref|YP_001406000.1|	Campylobacter hominis ATCC BAA-381	E-value = 7.00E-80	Identity = 59.01%
	5		gi|118474137|ref|YP_892712.1|	Campylobacter fetus subsp. fetus 82-40	E-value = 7.00E-73	Identity = 54.68%
AB1627	1		gi|146300223|ref|YP_001194814.1|	Flavobacterium johnsoniae UW101	E-value = 1.00E-41	Identity = 42.17%
	2		gi|110637732|ref|YP_677939.1|	Cytophaga hutchinsonii ATCC 33406	E-value = 6.00E-41	Identity = 37.33%
	3		gi|149184720|ref|ZP_01863038.1|	Erythrobacter sp. SD-21	E-value = 3.00E-24	Identity = 29.14%
	4		gi|85708853|ref|ZP_01039919.1|	Erythrobacter sp. NAP1	E-value = 4.00E-24	Identity = 31.49%
	5		gi|124005388|ref|ZP_01690229.1|	Microscilla marina ATCC 23134	E-value = 1.00E-22	Identity = 32.96%
AB1628	1		gi|67158560|ref|ZP_00419466.1|	Azotobacter vinelandii AvOP	E-value = 1.00E-19	Identity = 35.22%
	2		gi|152993478|ref|YP_001359199.1|	Sulfurovum sp. NBC37-1	E-value = 6.00E-17	Identity = 33.33%
	3		gi|67918015|ref|ZP_00511617.1|	Chlorobium limicola DSM 245	E-value = 2.00E-16	Identity = 35.63%
	4		gi|57237827|ref|YP_179075.1|	Campylobacter jejuni subsp. jejuni RM1221	E-value = 4.00E-16	Identity = 33.54%
	5		gi|15792329|ref|NP_282152.1|	Campylobacter jejuni subsp. jejuni NCTC 11168	E-value = 5.00E-16	Identity = 33.54%
AB1629	1			***** No matches found *****		
AB1630	1	*trmE*	gi|152990630|ref|YP_001356352.1|	Nitratiruptor sp. SB155-2	E-value = 3.00E-139	Identity = 61.52%
	2		gi|78776726|ref|YP_393041.1|	Sulfuromonas denitrificans ATCC 33889	E-value = 1.00E-137	Identity = 60.95%
	3		gi|152993155|ref|YP_001358876.1|	Sulfurovum sp. NBC37-1	E-value = 8.00E-134	Identity = 58.78%
	4		gi|149193806|ref|ZP_01870904.1|	Caminibacter mediatlanticus TB-2	E-value = 1.00E-128	Identity = 59.01%
	5		gi|34557648|ref|NP_907463.1|	Wolinella succinogenes DSM 1740	E-value = 1.00E-114	Identity = 49.31%
AB1631	1		gi|152990629|ref|YP_001356351.1|	Nitratiruptor sp. SB155-2	E-value = 8.00E-56	Identity = 49.47%
	2		gi|34557649|ref|NP_907464.1|	Wolinella succinogenes DSM 1740	E-value = 2.00E-53	Identity = 43.04%
	3		gi|118475440|ref|YP_891744.1|	Campylobacter fetus subsp. fetus 82-40	E-value = 5.00E-50	Identity = 41.72%
	4		gi|152993156|ref|YP_001358877.1|	Sulfurovum sp. NBC37-1	E-value = 2.00E-47	Identity = 41.98%
	5		gi|57167878|ref|ZP_00367018.1|	Campylobacter coli RM2228	E-value = 3.00E-45	Identity = 39.60%
AB1632	1	*oxaA*	gi|118474690|ref|YP_891743.1|	Campylobacter fetus subsp. fetus 82-40	E-value = 2.00E-155	Identity = 53.26%
	2		gi|152990628|ref|YP_001356350.1|	Nitratiruptor sp. SB155-2	E-value = 1.00E-150	Identity = 51.82%
	3		gi|157164729|ref|YP_001466647.1|	Campylobacter concisus 13826	E-value = 4.00E-150	Identity = 50.00%
	4		gi|154174780|ref|YP_001408435.1|	Campylobacter curvus 525.92	E-value = 7.00E-147	Identity = 49.52%
	5		gi|34557650|ref|NP_907465.1|	Wolinella succinogenes DSM 1740	E-value = 3.00E-142	Identity = 48.39%
AB1633	1		gi|152990627|ref|YP_001356349.1|	Nitratiruptor sp. SB155-2	E-value = 1.00E-20	Identity = 50.53%
	2		gi|154148175|ref|YP_001406233.1|	Campylobacter hominis ATCC BAA-381	E-value = 3.00E-18	Identity = 43.64%
	3		gi|154175264|ref|YP_001408436.1|	Campylobacter curvus 525.92	E-value = 4.00E-17	Identity = 46.88%
	4		gi|78776723|ref|YP_393038.1|	Sulfuromonas denitrificans ATCC 33889	E-value = 3.00E-15	Identity = 46.53%
	5		gi|157165305|ref|YP_001466646.1|	Campylobacter concisus 13826	E-value = 6.00E-15	Identity = 50.00%
AB1634	1	*rpmH*	gi|32265615|ref|NP_859647.1|	Helicobacter hepaticus ATCC 51449	E-value = 4.00E-05	Identity = 93.18%
	2		gi|15792290|ref|NP_282113.1|	Campylobacter jejuni subsp. jejuni NCTC 11168	E-value = 5.00E-05	Identity = 93.18%
	3		gi|57167874|ref|ZP_00367014.1|	Campylobacter coli	E-value = 5.00E-05	Identity = 93.18%
	4		gi|57237789|ref|YP_179037.1|	Campylobacter jejuni subsp. jejuni RM1221	E-value = 5.00E-05	Identity = 93.18%
	5		gi|86150683|ref|ZP_01068904.1|	Campylobacter jejuni subsp. jejuni CF93-6	E-value = 5.00E-05	Identity = 93.18%
AB1635	1	*clpB*	gi|152993046|ref|YP_001358767.1|	Sulfurovum sp. NBC37-1	E-value = 0	Identity = 74.18%
	2		gi|152990623|ref|YP_001356345.1|	Nitratiruptor sp. SB155-2	E-value = 0	Identity = 73.28%
	3		gi|34557025|ref|NP_906840.1|	Wolinella succinogenes DSM 1740	E-value = 0	Identity = 70.21%

	4		gi|78776720|ref|YP_393035.1|	Sulfuromonas denitrificans ATCC 33889	E-value = 0	Identity = 69.43%
	5		gi|118475028|ref|YP_892113.1|	Campylobacter fetus subsp. fetus 82-40	E-value = 0	Identity = 68.42%
AB1636	1	*rnhB*	gi|78776481|ref|YP_392796.1|	Sulfuromonas denitrificans ATCC 33889	E-value = 5.00E-49	Identity = 58.99%
	2		gi|152993838|ref|YP_001359559.1|	Sulfurovum sp. NBC37-1	E-value = 4.00E-45	Identity = 53.59%
	3		gi|34558029|ref|NP_907844.1|	Wolinella succinogenes DSM 1740	E-value = 1.00E-44	Identity = 53.11%
	4		gi|149194772|ref|ZP_01871867.1|	Caminibacter mediatlanticus TB-2	E-value = 2.00E-44	Identity = 57.45%
	5		gi|152989967|ref|YP_001355689.1|	Nitratiruptor sp. SB155-2	E-value = 3.00E-44	Identity = 52.54%
AB1637	1		gi|152990832|ref|YP_001356554.1|	Nitratiruptor sp. SB155-2	E-value = 4.00E-23	Identity = 59.18%
	2		gi|149194534|ref|ZP_01871630.1|	Caminibacter mediatlanticus TB-2	E-value = 4.00E-17	Identity = 49.51%
	3		gi|152993930|ref|YP_001359651.1|	Sulfurovum sp. NBC37-1	E-value = 3.00E-13	Identity = 42.42%
	4		gi|124516764|gb|EAY58272.1|	Leptospirillum sp. Group II UBA	E-value = 1.00E-12	Identity = 41.58%
	5		gi|117925472|ref|YP_866089.1|	Magnetococcus sp. MC-1	E-value = 1.00E-10	Identity = 39.00%
AB1638	1		gi|149193695|ref|ZP_01870793.1|	Caminibacter mediatlanticus TB-2	E-value = 3.00E-89	Identity = 66.40%
	2		gi|152993180|ref|YP_001358901.1|	Sulfurovum sp. NBC37-1	E-value = 2.00E-88	Identity = 57.75%
	3		gi|117926643|ref|YP_867260.1|	Magnetococcus sp. MC-1	E-value = 2.00E-82	Identity = 56.98%
	4		gi|152992285|ref|YP_001358006.1|	Sulfurovum sp. NBC37-1	E-value = 6.00E-69	Identity = 52.65%
	5		gi|78778078|ref|YP_394393.1|	Sulfuromonas denitrificans ATCC 33889	E-value = 4.00E-66	Identity = 51.41%
AB1639	1		gi|152992779|ref|YP_001358500.1|	Sulfurovum sp. NBC37-1	E-value = 3.00E-21	Identity = 48.18%
	2		gi|78776744|ref|YP_393059.1|	Sulfuromonas denitrificans ATCC 33889	E-value = 1.00E-16	Identity = 46.23%
	3		gi|152990187|ref|YP_001355909.1|	Nitratiruptor sp. SB155-2	E-value = 2.00E-14	Identity = 44.44%
	4		gi|118474533|ref|YP_892509.1|	Campylobacter fetus subsp. fetus 82-40	E-value = 3.00E-11	Identity = 37.14%
	5		gi|154148829|ref|YP_001406087.1|	Campylobacter hominis ATCC BAA-381	E-value = 3.00E-10	Identity = 37.61%
AB1640	1	*msrA*	gi|145622518|ref|ZP_01778476.1|	Petrotoga mobilis SJ95	E-value = 1.00E-48	Identity = 62.42%
	2		gi|94986564|ref|YP_594497.1|	Lawsonia intracellularis PHE/MN1-00	E-value = 3.00E-39	Identity = 55.56%
	3		gi|123430810|ref|XP_001307970.1|	Trichomonas vaginalis G3	E-value = 4.00E-39	Identity = 50.63%
	4		gi|149194462|ref|ZP_01871559.1|	Caminibacter mediatlanticus TB-2	E-value = 5.00E-37	Identity = 48.70%
	5		gi|123396703|ref|XP_001300955.1|	Trichomonas vaginalis G3	E-value = 1.00E-36	Identity = 52.20%
AB1641	1	*msrB*	gi|88857491|ref|ZP_01132134.1|	Pseudoalteromonas tunicata D2	E-value = 4.00E-43	Identity = 68.10%
	2		gi|153951037|ref|YP_001397776.1|	Campylobacter jejuni subsp. doylei 269.97	E-value = 2.00E-41	Identity = 73.68%
	3		gi|15792437|ref|NP_282260.1|	Campylobacter jejuni subsp. jejuni NCTC 11168	E-value = 3.00E-41	Identity = 73.68%
	4		gi|57237994|ref|YP_179243.1|	Campylobacter jejuni subsp. jejuni RM1221	E-value = 4.00E-41	Identity = 72.81%
	5		gi|90416627|ref|ZP_01224558.1|	marine gamma proteobacterium HTCC2207	E-value = 4.00E-41	Identity = 67.54%
AB1642	1		gi|90406948|ref|ZP_01215139.1|	Psychromonas sp. CNPT3	E-value = 2.00E-58	Identity = 58.13%
	2		gi|119945138|ref|YP_942818.1|	Psychromonas ingrahamii 37	E-value = 3.00E-58	Identity = 56.86%
	3		gi|90413984|ref|ZP_01221968.1|	Photobacterium profundum 3TCK	E-value = 1.00E-56	Identity = 50.75%
	4		gi|127512464|ref|YP_001093661.1|	Shewanella loihica PV-4	E-value = 2.00E-56	Identity = 52.58%
	5		gi|157375004|ref|YP_001473604.1|	Shewanella sediminis HAW-EB3	E-value = 1.00E-54	Identity = 52.45%
AB1643	1		gi|78358249|ref|YP_389698.1|	Desulfovibrio desulfuricans G20	E-value = 5.00E-11	Identity = 28.43%
	2		gi|29346832|ref|NP_810335.1|	Bacteroides thetaiotaomicron VPI-5482	E-value = 2.00E-07	Identity = 28.16%
	3		gi|88192935|pdb|2FB6|A	Bacteroides thetaiotaomicron VPI-5482	E-value = 7.00E-07	Identity = 28.16%
	4		gi|150021579|ref|YP_001306933.1|	Thermosipho melanesiensis BI429	E-value = 6.00E-06	Identity = 26.85%
AB1644	1		gi|134299425|ref|YP_001112921.1|	Desulfotomaculum reducens MI-1	E-value = 2.00E-44	Identity = 43.81%
	2		gi|34763836|ref|ZP_00144745.1|	Fusobacterium nucleatum subsp. vincentii ATCC 49256	E-value = 6.00E-28	Identity = 39.50%
	3		gi|149180516|ref|ZP_01859020.1|	Bacillus sp. SG-1	E-value = 7.00E-25	Identity = 35.29%
	4		gi|147678158|ref|YP_001212373.1|	Pelotomaculum thermopropionicum SI	E-value = 1.00E-24	Identity = 36.27%
	5		gi|114566563|ref|YP_753717.1|	Syntrophomonas wolfei subsp. wolfei str. Goettingen	E-value = 1.00E-24	Identity = 32.56%
AB1645	1	*bioA*	gi|110833117|ref|YP_691976.1|	Alcanivorax borkumensis SK2	E-value = 8.00E-132	Identity = 54.96%
	2		gi|6137239|gb|AAF04396.1|AF191556_4	Xenorhabdus nematophila	E-value = 9.00E-130	Identity = 52.52%
	3		gi|67940204|ref|ZP_00532661.1|	Chlorobium phaeobacteroides BS1	E-value = 1.00E-129	Identity = 54.39%

	4		gi|77960830|ref|ZP_00824685.1|	Yersinia mollaretii ATCC 43969	E-value = 1.00E-129	Identity = 52.63%
	5		gi|37525435|ref|NP_928779.1|	Photorhabdus luminescens subsp. laumondii TTO1	E-value = 2.00E-129	Identity = 52.28%
AB1646	1		gi|119946331|ref|YP_944011.1|	Psychromonas ingrahamii 37	E-value = 9.00E-148	Identity = 43.02%
	2		gi|78776237|ref|YP_392552.1|	Sulfuromonas denitrificans ATCC 33889	E-value = 3.00E-138	Identity = 35.42%
	3		gi|95931350|ref|ZP_01314063.1|	Desulfuromonas acetoxidans DSM 684	E-value = 3.00E-77	Identity = 30.24%
AB1647	1	*purH*	gi|78776749|ref|YP_393064.1|	Sulfuromonas denitrificans ATCC 33889	E-value = 0	Identity = 77.60%
	2		gi|152993150|ref|YP_001358871.1|	Sulfurovum sp. NBC37-1	E-value = 0	Identity = 73.33%
	3		gi|154174806|ref|YP_001408428.1|	Campylobacter curvus 525.92	E-value = 0	Identity = 68.24%
	4		gi|157165620|ref|YP_001466655.1|	Campylobacter concisus 13826	E-value = 0	Identity = 69.02%
	5		gi|34558180|ref|NP_907995.1|	Wolinella succinogenes DSM 1740	E-value = 0	Identity = 66.08%
AB1648	1	*purL*	gi|78776746|ref|YP_393061.1|	Sulfuromonas denitrificans ATCC 33889	E-value = 0	Identity = 71.78%
	2		gi|152990642|ref|YP_001356364.1|	Nitratiruptor sp. SB155-2	E-value = 0	Identity = 70.45%
	3		gi|152993151|ref|YP_001358872.1|	Sulfurovum sp. NBC37-1	E-value = 0	Identity = 69.78%
	4		gi|34557223|ref|NP_907038.1|	Wolinella succinogenes DSM 1740	E-value = 0	Identity = 66.94%
	5		gi|149194633|ref|ZP_01871729.1|	Caminibacter mediatlanticus TB-2	E-value = 0	Identity = 67.77%
AB1649	1		gi|34557654|ref|NP_907469.1|	Wolinella succinogenes DSM 1740	E-value = 2.00E-64	Identity = 40.44%
	2		gi|147667732|gb|ABQ45832.1|	Helicobacter pylori	E-value = 5.00E-60	Identity = 38.41%
	3		gi|109947439|ref|YP_664667.1|	Helicobacter acinonychis str. Sheeba	E-value = 8.00E-60	Identity = 38.11%
	4		gi|147667728|gb|ABQ45830.1|	Helicobacter pylori	E-value = 1.00E-59	Identity = 37.80%
	5		gi|15611534|ref|NP_223185.1|	Helicobacter pylori J99	E-value = 1.00E-59	Identity = 37.50%
AB1650	1		gi|118475048|ref|YP_891871.1|	Campylobacter fetus subsp. fetus 82-40	E-value = 4.00E-94	Identity = 48.94%
	2		gi|157164560|ref|YP_001466774.1|	Campylobacter concisus 13826	E-value = 2.00E-89	Identity = 44.57%
	3		gi|34556703|ref|NP_906518.1|	Wolinella succinogenes DSM 1740	E-value = 5.00E-88	Identity = 47.51%
	4		gi|57240635|ref|ZP_00368584.1|	Campylobacter lari RM2100	E-value = 8.00E-87	Identity = 46.05%
	5		gi|154149492|ref|YP_001407150.1|	Campylobacter hominis ATCC BAA-381	E-value = 3.00E-84	Identity = 44.66%
AB1651	1	*folE*	gi|152991606|ref|YP_001357327.1|	Sulfurovum sp. NBC37-1	E-value = 2.00E-71	Identity = 68.48%
	2		gi|152989913|ref|YP_001355635.1|	Nitratiruptor sp. SB155-2	E-value = 6.00E-71	Identity = 67.93%
	3		gi|157164234|ref|YP_001465994.1|	Campylobacter concisus 13826	E-value = 2.00E-68	Identity = 67.78%
	4		gi|154174139|ref|YP_001407434.1|	Campylobacter curvus 525.92	E-value = 3.00E-68	Identity = 67.78%
	5		gi|78778280|ref|YP_394595.1|	Sulfuromonas denitrificans ATCC 33889	E-value = 1.00E-67	Identity = 68.79%
AB1652	1	*corA*	gi|78221361|ref|YP_383108.1|	Geobacter metallireducens GS-15	E-value = 3.00E-78	Identity = 48.75%
	2		gi|39998411|ref|NP_954362.1|	Geobacter sulfurreducens PCA	E-value = 8.00E-76	Identity = 47.81%
	3		gi|118743828|ref|ZP_01591829.1|	Geobacter lovleyi SZ	E-value = 6.00E-73	Identity = 48.46%
	4		gi|34557417|ref|NP_907232.1|	Wolinella succinogenes DSM 1740	E-value = 5.00E-68	Identity = 43.03%
	5		gi|32267178|ref|NP_861210.1|	Helicobacter hepaticus ATCC 51449	E-value = 2.00E-63	Identity = 39.75%
AB1653	1	*ctsF*	gi|152990756|ref|YP_001356478.1|	Nitratiruptor sp. SB155-2	E-value = 3.00E-38	Identity = 31.04%
	2		gi|118475095|ref|YP_892177.1|	Campylobacter fetus subsp. fetus 82-40	E-value = 2.00E-36	Identity = 28.68%
	3		gi|152991304|ref|YP_001357026.1|	Nitratiruptor sp. SB155-2	E-value = 5.00E-35	Identity = 27.45%
	4		gi|154174517|ref|YP_001408338.1|	Campylobacter curvus 525.92	E-value = 1.00E-34	Identity = 26.09%
	5		gi|157164422|ref|YP_001466823.1|	Campylobacter concisus 13826	E-value = 1.00E-33	Identity = 25.49%
AB1654	1	*ctsE*	gi|17388924|gb|AAL38651.1|	Burkholderia cenocepacia	E-value = 7.00E-71	Identity = 40.47%
	2		gi|84356363|ref|ZP_00981211.1|	Burkholderia cenocepacia PC184	E-value = 2.00E-70	Identity = 40.18%
	3		gi|153839991|ref|ZP_01992658.1|	Vibrio parahaemolyticus AQ3810	E-value = 2.00E-70	Identity = 39.27%
	4		gi|1657238|emb|CAA70319.1|	Acinetobacter sp. ADP1	E-value = 1.00E-67	Identity = 39.08%
	5		gi|94267867|ref|ZP_01291018.1|	delta proteobacterium MLMS-1	E-value = 1.00E-67	Identity = 37.82%
AB1655	1		gi|134288517|ref|YP_001110680.1|	Burkholderia vietnamiensis G4	E-value = 3.00E-10	Identity = 32.37%
AB1656	1		gi|152990029|ref|YP_001355751.1|	Nitratiruptor sp. SB155-2	E-value = 4.00E-40	Identity = 41.78%
	2		gi|152991709|ref|YP_001357430.1|	Sulfurovum sp. NBC37-1	E-value = 2.00E-36	Identity = 40.09%
	3		gi|34556743|ref|NP_906558.1|	Wolinella succinogenes DSM 1740	E-value = 2.00E-36	Identity = 41.31%

	4	gi|149195301|ref|ZP_01872389.1|	Caminibacter mediatlanticus TB-2	E-value = 3.00E-32	Identity = 42.03%
	5	gi|57237226|ref|YP_178238.1|	Campylobacter jejuni subsp. jejuni RM1221	E-value = 6.00E-30	Identity = 43.75%
AB1657	1	gi|115359529|ref|YP_776667.1|	Burkholderia cepacia AMMD	E-value = 6.00E-33	Identity = 39.64%
	2	gi|91783393|ref|YP_558599.1|	Burkholderia xenovorans LB400	E-value = 5.00E-32	Identity = 39.64%
	3	gi|118746918|ref|ZP_01594822.1|	Geobacter lovleyi SZ	E-value = 4.00E-20	Identity = 32.71%
	4	gi|119509502|ref|ZP_01628650.1|	Nodularia spumigena CCY9414	E-value = 6.00E-20	Identity = 33.33%
	5	gi|148262486|ref|YP_001229192.1|	Geobacter uraniumreducens Rf4	E-value = 2.00E-17	Identity = 30.99%
AB1658	1	gi|118072798|ref|ZP_01540985.1|	Shewanella woodyi ATCC 51908	E-value = 5.00E-63	Identity = 62.57%
AB1659	1	gi|86149184|ref|ZP_01067416.1|	Campylobacter jejuni subsp. jejuni CF93-6	E-value = 2.00E-74	Identity = 58.91%
	2	gi|156147244|gb|ABU53822.1|	Campylobacter phage CGC-2007	E-value = 5.00E-74	Identity = 58.91%
	3	gi|156147278|gb|ABU53854.1|	Campylobacter phage CGC-2007	E-value = 6.00E-74	Identity = 58.91%
	4	gi|156147207|gb|ABU53787.1|	Campylobacter phage CGC-2007	E-value = 9.00E-74	Identity = 58.91%
	5	gi|156147139|gb|ABU53723.1|	Campylobacter phage CGC-2007	E-value = 2.00E-73	Identity = 58.53%
AB1660	1	gi|86151696|ref|ZP_01069910.1|	Campylobacter jejuni subsp. jejuni 260.94	E-value = 4.00E-36	Identity = 33.23%
	2	gi|57237264|ref|YP_178277.1|	Campylobacter jejuni subsp. jejuni RM1221	E-value = 1.00E-35	Identity = 32.62%
	3	gi|86149765|ref|ZP_01067994.1|	Campylobacter jejuni subsp. jejuni CF93-6	E-value = 1.00E-35	Identity = 32.62%
	4	gi|83586159|ref|ZP_00924796.1|	Escherichia coli 101-1	E-value = 5.00E-22	Identity = 26.67%
	5	gi|16761548|ref|NP_457165.1|	Salmonella enterica subsp. enterica serovar Typhi str. CT18	E-value = 9.00E-22	Identity = 26.67%
AB1661	1		***** No matches found *****		
AB1662	1		***** No matches found *****		
AB1663	1	gi|42520427|ref|NP_966342.1|	Wolbachia endosymbiont of Drosophila melanogaster	E-value = 1.00E-90	Identity = 40.19%
	2	gi|99034521|ref|ZP_01314502.1|	Wolbachia endosymbiont of Drosophila willistoni TSC#14030-0811.24	E-value = 4.00E-90	Identity = 40.34%
	3	gi|16517105|gb|AAL24514.1|	Wolbachia endosymbiont of Drosophila melanogaster	E-value = 2.00E-89	Identity = 39.62%
	4	gi|58698944|ref|ZP_00373803.1|	Wolbachia endosymbiont of Drosophila ananassae	E-value = 1.00E-88	Identity = 38.68%
	5	gi|71901663|ref|ZP_00683740.1|	Xylella fastidiosa Ann-1	E-value = 1.00E-50	Identity = 26.48%
AB1664	1		***** No matches found *****		
AB1665	1	gi|86151682|ref|ZP_01069896.1|	Campylobacter jejuni subsp. jejuni 260.94	E-value = 1.00E-11	Identity = 31.90%
	2	gi|156147197|gb|ABU53778.1|	Campylobacter phage CGC-2007	E-value = 2.00E-11	Identity = 32.30%
	3	gi|57237237|ref|YP_178249.1|	Campylobacter jejuni subsp. jejuni RM1221	E-value = 3.00E-11	Identity = 31.45%
AB1666	1	gi|28870560|ref|NP_793179.1|	Pseudomonas syringae pv. tomato str. DC3000	E-value = 2.00E-13	Identity = 26.51%
	2	gi|46133068|ref|ZP_00156540.2|	Haemophilus influenzae R2866	E-value = 4.00E-06	Identity = 26.13%
AB1667	1		***** No matches found *****		
AB1668	1	gi|77975670|ref|ZP_00831204.1|	Yersinia frederiksenii ATCC 33641	E-value = 7.00E-10	Identity = 32.26%
	2	gi|75240415|ref|ZP_00724349.1|	Escherichia coli F11	E-value = 4.00E-07	Identity = 28.48%
	3	gi|15799958|ref|NP_285970.1|	Escherichia coli O157:H7 EDL933	E-value = 6.00E-07	Identity = 27.85%
	4	gi|83586328|ref|ZP_00924963.1|	Escherichia coli 101-1	E-value = 1.00E-06	Identity = 27.85%
	5	gi|16129119|ref|NP_415674.1|	Escherichia coli K12	E-value = 1.00E-06	Identity = 26.75%
AB1669	1	gi|146313120|ref|YP_001178194.1|	Enterobacter sp. 638	E-value = 4.00E-21	Identity = 28.19%
	2	gi|156147202|gb|ABU53783.1|	Campylobacter phage CGC-2007	E-value = 2.00E-19	Identity = 27.62%
	3	gi|156147236|gb|ABU53815.1|	Campylobacter phage CGC-2007	E-value = 3.00E-19	Identity = 26.92%
	4	gi|156147133|gb|ABU53718.1|	Campylobacter phage CGC-2007	E-value = 7.00E-19	Identity = 26.92%
	5	gi|78358433|ref|YP_389882.1|	Desulfovibrio desulfuricans G20	E-value = 3.00E-11	Identity = 26.79%
AB1670	1	gi|86149092|ref|ZP_01067324.1|	Campylobacter jejuni subsp. jejuni CF93-6	E-value = 2.00E-17	Identity = 35.54%
	2	gi|57237243|ref|YP_178255.1|	Campylobacter jejuni subsp. jejuni RM1221	E-value = 4.00E-17	Identity = 35.54%
	3	gi|86151762|ref|ZP_01069976.1|	Campylobacter jejuni subsp. jejuni 260.94	E-value = 9.00E-17	Identity = 34.94%
	4	gi|75228033|ref|ZP_00714713.1|	Escherichia coli B7A	E-value = 4.00E-12	Identity = 28.11%
	5	gi|28870563|ref|NP_793182.1|	Pseudomonas syringae pv. tomato str. DC3000	E-value = 8.00E-12	Identity = 29.26%
AB1671	1	gi|8670758|emb|CAB94939.1|	Campylobacter coli	E-value = 1.00E-24	Identity = 30.77%
	2	gi|57237244|ref|YP_178256.1|	Campylobacter jejuni subsp. jejuni RM1221	E-value = 1.00E-24	Identity = 31.09%

	3	gi|86151542|ref|ZP_01069756.1|	Campylobacter jejuni subsp. jejuni 260.94	E-value = 9.00E-24	Identity = 30.45%
	4	gi|86148960|ref|ZP_01067192.1|	Campylobacter jejuni subsp. jejuni CF93-6	E-value = 1.00E-23	Identity = 30.13%
	5	gi|145633950|ref|ZP_01789669.1|	Haemophilus influenzae 3655	E-value = 4.00E-12	Identity = 26.99%
AB1672	1		***** No matches found *****		
AB1673	1	gi|86151824|ref|ZP_01070038.1|	Campylobacter jejuni subsp. jejuni 260.94	E-value = 4.00E-22	Identity = 40.21%
	2	gi|57237247|ref|YP_178259.1|	Campylobacter jejuni subsp. jejuni RM1221	E-value = 5.00E-22	Identity = 40.21%
	3	gi|86149197|ref|ZP_01067429.1|	Campylobacter jejuni subsp. jejuni CF93-6	E-value = 7.00E-22	Identity = 39.44%
	4	gi|72537705|ref|YP_293735.1|	Burkholderia phage phi52237	E-value = 3.00E-12	Identity = 26.98%
	5	gi|53722105|ref|YP_111090.1|	Burkholderia pseudomallei K96243	E-value = 3.00E-12	Identity = 28.11%
AB1674	1		***** No matches found *****		
AB1675	1	gi|154149417|ref|YP_001405877.1|	Campylobacter hominis ATCC BAA-381	E-value = 2.00E-08	Identity = 45.16%
AB1676	1	gi|154148430|ref|YP_001405876.1|	Campylobacter hominis ATCC BAA-381	E-value = 5.00E-07	Identity = 36.27%
AB1677	1		***** No matches found *****		
AB1678	1	gi|57237254|ref|YP_178267.1|	Campylobacter jejuni subsp. jejuni RM1221	E-value = 2.00E-29	Identity = 35.86%
	2	gi|86151555|ref|ZP_01069769.1|	Campylobacter jejuni subsp. jejuni 260.94	E-value = 5.00E-29	Identity = 35.46%
	3	gi|153092842|gb|EDN73879.1|	Mannheimia haemolytica PHL213	E-value = 8.00E-10	Identity = 30.14%
	4	gi|33151720|ref|NP_873073.1|	Haemophilus ducreyi 35000HP	E-value = 4.00E-08	Identity = 25.54%
AB1679	1		***** No matches found *****		
AB1680	1	gi|86148938|ref|ZP_01067170.1|	Campylobacter jejuni subsp. jejuni CF93-6	E-value = 4.00E-30	Identity = 31.45%
	2	gi|57237256|ref|YP_178269.1|	Campylobacter jejuni subsp. jejuni RM1221	E-value = 5.00E-30	Identity = 31.23%
	3	gi|86151743|ref|ZP_01069957.1|	Campylobacter jejuni subsp. jejuni 260.94	E-value = 6.00E-30	Identity = 31.45%
	4	gi|53802937|ref|YP_115315.1|	Methylococcus capsulatus str. Bath	E-value = 1.00E-07	Identity = 26.59%
AB1681	1	gi|57237257|ref|YP_178270.1|	Campylobacter jejuni subsp. jejuni RM1221	E-value = 7.00E-16	Identity = 46.23%
AB1682	1		***** No matches found *****		
AB1683	1	gi|86149227|ref|ZP_01067459.1|	Campylobacter jejuni subsp. jejuni CF93-6	E-value = 2.00E-122	Identity = 44.04%
	2	gi|86151825|ref|ZP_01070039.1|	Campylobacter jejuni subsp. jejuni 260.94	E-value = 6.00E-122	Identity = 43.67%
	3	gi|57237259|ref|YP_178272.1|	Campylobacter jejuni subsp. jejuni RM1221	E-value = 1.00E-121	Identity = 44.66%
	4	gi|46581109|ref|YP_011917.1|	Desulfovibrio vulgaris subsp. vulgaris str. Hildenborough	E-value = 1.00E-82	Identity = 36.19%
	5	gi|38229138|ref|NP_938233.1|	Pseudomonas phage D3112	E-value = 2.00E-77	Identity = 37.00%
AB1684	1	gi|57237260|ref|YP_178273.1|	Campylobacter jejuni subsp. jejuni RM1221	E-value = 3.00E-61	Identity = 34.95%
	2	gi|86149035|ref|ZP_01067267.1|	Campylobacter jejuni subsp. jejuni CF93-6	E-value = 4.00E-61	Identity = 34.95%
	3	gi|145635818|ref|ZP_01791510.1|	Haemophilus influenzae PittAA	E-value = 1.00E-25	Identity = 26.21%
	4	gi|54654574|gb|AAV37152.1|	Haemophilus influenzae biotype aegyptius	E-value = 9.00E-25	Identity = 25.46%
	5	gi|145637185|ref|ZP_01792847.1|	Haemophilus influenzae PittHH	E-value = 2.00E-24	Identity = 25.35%
AB1685	1	gi|134295415|ref|YP_001119150.1|	Burkholderia vietnamiensis G4	E-value = 2.00E-32	Identity = 29.17%
	2	gi|114778722|ref|ZP_01453532.1|	Mariprofundus ferrooxydans PV-1	E-value = 1.00E-27	Identity = 26.53%
AB1686	1	gi|53802934|ref|YP_115318.1|	Methylococcus capsulatus str. Bath	E-value = 6.00E-11	Identity = 29.33%
	2	gi|85058816|ref|YP_454518.1|	Sodalis glossinidius str. 'morsitans'	E-value = 3.00E-08	Identity = 29.41%
	3	gi|86149132|ref|ZP_01067364.1|	Campylobacter jejuni subsp. jejuni CF93-6	E-value = 5.00E-08	Identity = 29.34%
	4	gi|156147226|gb|ABU53805.1|	Campylobacter phage CGC-2007	E-value = 6.00E-08	Identity = 29.34%
	5	gi|15794245|ref|NP_284067.1|	Neisseria meningitidis Z2491	E-value = 7.00E-08	Identity = 32.64%
AB1687	1	gi|57237265|ref|YP_178278.1|	Campylobacter jejuni subsp. jejuni RM1221	E-value = 3.00E-08	Identity = 41.05%
AB1688	1		***** No matches found *****		
AB1690	1	gi|51245417|ref|YP_065301.1|	Desulfotalea psychrophila LSv54	E-value = 3.00E-22	Identity = 28.44%
	2	gi|153215749|ref|ZP_01950118.1|	Vibrio cholerae 1587	E-value = 1.00E-19	Identity = 30.95%
AB1692	1	gi|86149931|ref|ZP_01068160.1|	Campylobacter jejuni subsp. jejuni CF93-6	E-value = 8.00E-38	Identity = 34.51%
	2	gi|57237271|ref|YP_178284.1|	Campylobacter jejuni subsp. jejuni RM1221	E-value = 1.00E-37	Identity = 34.51%
	3	gi|86151593|ref|ZP_01069807.1|	Campylobacter jejuni subsp. jejuni 260.94	E-value = 2.00E-37	Identity = 34.51%
AB1693	1		***** No matches found *****		

AB1694	1			***** No matches found *****		
AB1696	1		gi|154174211|ref|YP_001409071.1|	Campylobacter curvus 525.92	E-value = 2.00E-37	Identity = 49.73%
	2		gi|152990232|ref|YP_001355954.1|	Nitratiruptor sp. SB155-2	E-value = 3.00E-34	Identity = 42.08%
	3		gi|154148627|ref|YP_001405879.1|	Campylobacter hominis ATCC BAA-381	E-value = 4.00E-28	Identity = 44.51%
	4		gi|154148945|ref|YP_001407114.1|	Campylobacter hominis ATCC BAA-381	E-value = 2.00E-27	Identity = 44.19%
	5		gi|118732540|ref|ZP_01581054.1|	Delftia acidovorans SPH-1	E-value = 8.00E-20	Identity = 36.84%
AB1697	1		gi|114707500|ref|ZP_01440396.1|	Fulvimarina pelagi HTCC2506	E-value = 7.00E-27	Identity = 36.63%
	2		gi|118593663|ref|ZP_01551039.1|	Stappia aggregata IAM 12614	E-value = 4.00E-24	Identity = 34.16%
	3		gi|85058798|ref|YP_454500.1|	Sodalis glossinidius str. 'morsitans'	E-value = 6.00E-24	Identity = 32.37%
	4		gi|77957251|ref|ZP_00821311.1|	Yersinia bercovieri ATCC 43970	E-value = 2.00E-23	Identity = 34.33%
	5		gi|153000597|ref|YP_001366278.1|	Shewanella baltica OS185	E-value = 1.00E-22	Identity = 32.84%
AB1698	1			***** No matches found *****		
AB1700	1			***** No matches found *****		
AB1701	1			***** No matches found *****		
AB1702	1		gi|120599731|ref|YP_964305.1|	Shewanella sp. W3-18-1	E-value = 1.00E-24	Identity = 35.08%
	2		gi|46581093|ref|YP_011901.1|	Desulfovibrio vulgaris subsp. vulgaris str. Hildenborough	E-value = 6.00E-20	Identity = 27.03%
	3		gi|120612757|ref|YP_972435.1|	Acidovorax avenae subsp. citrulli AAC00-1	E-value = 2.00E-19	Identity = 28.70%
	4		gi|85858365|ref|YP_460567.1|	Syntrophus aciditrophicus SB	E-value = 2.00E-19	Identity = 28.83%
	5		gi|114778747|ref|ZP_01453557.1|	Mariprofundus ferrooxydans PV-1	E-value = 3.00E-19	Identity = 29.67%
AB1703	1			***** No matches found *****		
AB1704	1			***** No matches found *****		
AB1705	1		gi|154148443|ref|YP_001405854.1|	Campylobacter hominis ATCC BAA-381	E-value = 1.00E-15	Identity = 33.33%
	2		gi|32266247|ref|NP_860279.1|	Helicobacter hepaticus ATCC 51449	E-value = 3.00E-14	Identity = 28.15%
	3		gi|149195301|ref|ZP_01872389.1|	Caminibacter mediatlanticus TB-2	E-value = 2.00E-10	Identity = 33.33%
	4		gi|34556743|ref|NP_906558.1|	Wolinella succinogenes DSM 1740	E-value = 2.00E-10	Identity = 30.64%
	5		gi|9635582|ref|NP_061565.1|	Pseudomonas phage D3	E-value = 2.00E-09	Identity = 27.11%
AB1706	1			***** No matches found *****		
AB1707	1			***** No matches found *****		
AB1708	1			***** No matches found *****		
AB1709	1	*era*	gi|152990747|ref|YP_001356469.1|	Nitratiruptor sp. SB155-2	E-value = 6.00E-99	Identity = 61.94%
	2		gi|152993265|ref|YP_001358986.1|	Sulfurovum sp. NBC37-1	E-value = 6.00E-97	Identity = 62.41%
	3		gi|34557655|ref|NP_907470.1|	Wolinella succinogenes DSM 1740	E-value = 8.00E-97	Identity = 59.11%
	4		gi|78777575|ref|YP_393890.1|	Sulfuromonas denitrificans ATCC 33889	E-value = 4.00E-90	Identity = 60.55%
	5		gi|149194255|ref|ZP_01871352.1|	Caminibacter mediatlanticus TB-2	E-value = 2.00E-86	Identity = 57.53%
AB1710	1		gi|154149407|ref|YP_001406183.1|	Campylobacter hominis ATCC BAA-381	E-value = 2.00E-07	Identity = 25.45%
AB1711	1		gi|78778227|ref|YP_394542.1|	Sulfuromonas denitrificans ATCC 33889	E-value = 5.00E-146	Identity = 53.56%
	2		gi|152991611|ref|YP_001357332.1|	Sulfurovum sp. NBC37-1	E-value = 2.00E-143	Identity = 53.31%
	3		gi|57241774|ref|ZP_00369719.1|	Campylobacter lari RM2100	E-value = 3.00E-139	Identity = 52.18%
	4		gi|154173654|ref|YP_001407440.1|	Campylobacter curvus 525.92	E-value = 6.00E-139	Identity = 52.77%
	5		gi|157164155|ref|YP_001465988.1|	Campylobacter concisus 13826	E-value = 4.00E-135	Identity = 52.36%
AB1712	1			***** No matches found *****		
AB1713	1	*def*	gi|152989916|ref|YP_001355638.1|	Nitratiruptor sp. SB155-2	E-value = 5.00E-52	Identity = 67.55%
	2		gi|152991610|ref|YP_001357331.1|	Sulfurovum sp. NBC37-1	E-value = 4.00E-48	Identity = 59.88%
	3		gi|154175184|ref|YP_001407439.1|	Campylobacter curvus 525.92	E-value = 2.00E-45	Identity = 59.75%
	4		gi|154149172|ref|YP_001405640.1|	Campylobacter hominis ATCC BAA-381	E-value = 2.00E-45	Identity = 57.65%
	5		gi|118475317|ref|YP_892810.1|	Campylobacter fetus subsp. fetus 82-40	E-value = 5.00E-45	Identity = 58.97%
AB1714	1	*clpP*	gi|34558484|ref|NP_908299.1|	Wolinella succinogenes DSM 1740	E-value = 4.00E-87	Identity = 82.29%
	2		gi|152991609|ref|YP_001357330.1|	Sulfurovum sp. NBC37-1	E-value = 7.00E-86	Identity = 81.25%
	3		gi|118474160|ref|YP_892812.1|	Campylobacter fetus subsp. fetus 82-40	E-value = 1.00E-85	Identity = 83.16%

	4		gi|78778230|ref|YP_394545.1|	Sulfuromonas denitrificans ATCC 33889	E-value = 6.00E-85	Identity = 80.21%
	5		gi|152989915|ref|YP_001355637.1|	Nitratiruptor sp. SB155-2	E-value = 2.00E-84	Identity = 83.16%
AB1715	1	*tig*	gi|152991608|ref|YP_001357329.1|	Sulfurovum sp. NBC37-1	E-value = 9.00E-104	Identity = 54.57%
	2		gi|78778231|ref|YP_394546.1|	Sulfuromonas denitrificans ATCC 33889	E-value = 5.00E-102	Identity = 51.04%
	3		gi|157165093|ref|YP_001465992.1|	Campylobacter concisus 13826	E-value = 6.00E-97	Identity = 49.18%
	4		gi|152989914|ref|YP_001355636.1|	Nitratiruptor sp. SB155-2	E-value = 2.00E-90	Identity = 48.26%
	5		gi|154175478|ref|YP_001407436.1|	Campylobacter curvus 525.92	E-value = 5.00E-90	Identity = 46.73%
AB1716	1		gi|117926643|ref|YP_867260.1|	Magnetococcus sp. MC-1	E-value = 2.00E-82	Identity = 52.46%
	2		gi|51246595|ref|YP_066479.1|	Desulfotalea psychrophila LSv54	E-value = 7.00E-62	Identity = 47.46%
	3		gi|33600251|ref|NP_887811.1|	Bordetella bronchiseptica RB50	E-value = 7.00E-61	Identity = 40.13%
	4		gi|33595727|ref|NP_883370.1|	Bordetella parapertussis 12822	E-value = 6.00E-60	Identity = 39.80%
	5		gi|118729738|ref|ZP_01578255.1|	Delftia acidovorans SPH-1	E-value = 1.00E-56	Identity = 39.10%
AB1718	1		gi|78778078|ref|YP_394393.1|	Sulfuromonas denitrificans ATCC 33889	E-value = 6.00E-91	Identity = 67.42%
	2		gi|152992285|ref|YP_001358006.1|	Sulfurovum sp. NBC37-1	E-value = 8.00E-90	Identity = 63.37%
	3		gi|118474779|ref|YP_892249.1|	Campylobacter fetus subsp. fetus 82-40	E-value = 3.00E-89	Identity = 66.42%
	4		gi|57238200|ref|YP_178719.1|	Campylobacter jejuni subsp. jejuni RM1221	E-value = 5.00E-88	Identity = 66.42%
	5		gi|86151711|ref|ZP_01069925.1|	Campylobacter jejuni subsp. jejuni 260.94	E-value = 1.00E-87	Identity = 66.06%
AB1719	1	*nspC*	gi|78777378|ref|YP_393693.1|	Sulfuromonas denitrificans ATCC 33889	E-value = 2.00E-140	Identity = 63.35%
	2		gi|152993160|ref|YP_001358881.1|	Sulfurovum sp. NBC37-1	E-value = 8.00E-138	Identity = 59.52%
	3		gi|152991040|ref|YP_001356762.1|	Nitratiruptor sp. SB155-2	E-value = 2.00E-133	Identity = 58.84%
	4		gi|149194966|ref|ZP_01872059.1|	Caminibacter mediatlanticus TB-2	E-value = 1.00E-128	Identity = 61.79%
	5		gi|34556614|ref|NP_906429.1|	Wolinella succinogenes DSM 1740	E-value = 6.00E-127	Identity = 56.15%
AB1720	1		gi|57168656|ref|ZP_00367788.1|	Campylobacter coli RM2228	E-value = 0	Identity = 77.41%
	2		gi|121612631|ref|YP_999896.1|	Campylobacter jejuni subsp. jejuni 81-176	E-value = 0	Identity = 76.65%
	3		gi|157414486|ref|YP_001481742.1|	Campylobacter jejuni subsp. jejuni 81116	E-value = 0	Identity = 76.65%
	4		gi|15791559|ref|NP_281382.1|	Campylobacter jejuni subsp. jejuni NCTC 11168	E-value = 0	Identity = 76.65%
	5		gi|148926338|ref|ZP_01810022.1|	Campylobacter jejuni subsp. jejuni CG8486	E-value = 0	Identity = 76.40%
AB1721	1		gi|152991432|ref|YP_001357154.1|	Nitratiruptor sp. SB155-2	E-value = 1.00E-77	Identity = 49.48%
	2		gi|152994022|ref|YP_001359743.1|	Sulfurovum sp. NBC37-1	E-value = 2.00E-63	Identity = 42.86%
	3		gi|78776442|ref|YP_392757.1|	Sulfuromonas denitrificans ATCC 33889	E-value = 1.00E-56	Identity = 38.13%
	4		gi|154175228|ref|YP_001408727.1|	Campylobacter curvus 525.92	E-value = 2.00E-46	Identity = 37.66%
	5		gi|152993870|ref|YP_001359591.1|	Sulfurovum sp. NBC37-1	E-value = 6.00E-38	Identity = 34.76%
AB1722	1		gi|95929204|ref|ZP_01311948.1|	Desulfuromonas acetoxidans DSM 684	E-value = 2.00E-71	Identity = 52.85%
	2		gi|94499772|ref|ZP_01306308.1|	Oceanobacter sp. RED65	E-value = 2.00E-70	Identity = 55.02%
	3		gi|153885239|ref|ZP_02006396.1|	Ralstonia pickettii 12D	E-value = 2.00E-70	Identity = 54.03%
	4		gi|21233401|ref|NP_639318.1|	Xanthomonas campestris pv. campestris str. ATCC 33913	E-value = 8.00E-63	Identity = 45.20%
	5		gi|58580001|ref|YP_199017.1|	Xanthomonas oryzae pv. oryzae KACC10331	E-value = 1.00E-62	Identity = 44.94%
AB1723	1		gi|152991431|ref|YP_001357153.1|	Nitratiruptor sp. SB155-2	E-value = 2.00E-15	Identity = 64.47%
AB1724	1			***** No matches found *****		
AB1725	1			***** No matches found *****		
AB1726	1			***** No matches found *****		
AB1727	1			***** No matches found *****		
AB1728	1		gi|110637901|ref|YP_678108.1|	Cytophaga hutchinsonii ATCC 33406	E-value = 3.00E-27	Identity = 27.30%
	2		gi|34541111|ref|NP_905590.1|	Porphyromonas gingivalis W83	E-value = 8.00E-22	Identity = 25.18%
	3		gi|147678832|ref|YP_001213047.1|	Pelotomaculum thermopropionicum SI	E-value = 7.00E-19	Identity = 25.37%
	4		gi|42525747|ref|NP_970845.1|	Treponema denticola ATCC 35405	E-value = 7.00E-17	Identity = 25.98%
AB1729	1		gi|151570667|gb|EDN36321.1|	Francisella tularensis subsp. novicida GA99-3549	E-value = 6.00E-78	Identity = 37.25%
AB1730	1	*hsdS*	gi|151570668|gb|EDN36322.1|	Francisella tularensis subsp. novicida GA99-3549	E-value = 1.00E-48	Identity = 31.49%
	2		gi|56707658|ref|YP_169554.1|	Francisella tularensis subsp. tularensis SCHU S4	E-value = 6.00E-35	Identity = 28.06%

	3		gi|88856339|ref|ZP_01130998.1|	marine actinobacterium PHSC20C1	E-value = 1.00E-30	Identity = 28.98%
	4		gi|86153318|ref|ZP_01071522.1|	Campylobacter jejuni subsp. jejuni HB93-13	E-value = 2.00E-30	Identity = 32.70%
	5		gi|75674466|ref|YP_316887.1|	Nitrobacter winogradskyi Nb-255	E-value = 2.00E-29	Identity = 26.83%
AB1731	1	*hsdM*	gi|151570669|gb|EDN36323.1|	Francisella tularensis subsp. novicida GA99-3549	E-value = 0	Identity = 56.56%
	2		gi|156502396|ref|YP_001428461.1|	Francisella tularensis subsp. holarctica FTA	E-value = 0	Identity = 61.90%
	3		gi|157122252|gb|EDO66392.1|	Francisella tularensis subsp. holarctica FSC022	E-value = 0	Identity = 61.90%
	4		gi|89256323|ref|YP_513685.1|	Francisella tularensis subsp. holarctica	E-value = 0	Identity = 61.90%
	5		gi|57242478|ref|ZP_00370416.1|	Campylobacter upsaliensis RM3195	E-value = 2.00E-52	Identity = 28.65%
AB1732	1		gi|71558959|ref|YP_271693.1|	Haemophilus influenzae biotype aegyptius	E-value = 2.00E-13	Identity = 37.04%
	2		gi|21628957|ref|NP_660216.1|	Haemophilus influenzae biotype aegyptius	E-value = 5.00E-13	Identity = 35.56%
	3		gi|29375136|ref|NP_814289.1|	Enterococcus faecalis V583	E-value = 1.00E-12	Identity = 32.08%
	4		gi|124008716|ref|ZP_01693406.1|	Microscilla marina ATCC 23134	E-value = 3.00E-12	Identity = 37.33%
	5		gi|75214512|ref|ZP_00713084.1|	Escherichia coli E110019	E-value = 1.00E-11	Identity = 34.75%
AB1733	1			***** No matches found *****		
AB1734	1			***** No matches found *****		
AB1735	1			***** No matches found *****		
AB1736	1			***** No matches found *****		
AB1737	1	*metC1*	gi|110598546|ref|ZP_01386815.1|	Chlorobium ferrooxidans DSM 13031	E-value = 6.00E-72	Identity = 38.90%
	2		gi|68551294|ref|ZP_00590711.1|	Pelodictyon phaeoclathratiforme BU-1	E-value = 3.00E-71	Identity = 38.80%
	3		gi|78187405|ref|YP_375448.1|	Pelodictyon luteolum DSM 273	E-value = 3.00E-69	Identity = 37.31%
	4		gi|126662648|ref|ZP_01733647.1|	Flavobacteria bacterium BAL38	E-value = 2.00E-68	Identity = 38.75%
	5		gi|150024459|ref|YP_001295285.1|	Flavobacterium psychrophilum JIP02/86	E-value = 5.00E-68	Identity = 40.44%
AB1738	1	*metC2*	gi|45656012|ref|YP_000098.1|	Leptospira interrogans serovar Copenhageni str. Fiocruz L1-130	E-value = 9.00E-103	Identity = 43.56%
	2		gi|24212813|ref|NP_710294.1|	Leptospira interrogans serovar Lai str. 56601	E-value = 1.00E-102	Identity = 43.56%
	3		gi|116329878|ref|YP_799596.1|	Leptospira borgpetersenii serovar Hardjo-bovis JB197	E-value = 2.00E-98	Identity = 41.67%
	4		gi|116326890|ref|YP_796610.1|	Leptospira borgpetersenii serovar Hardjo-bovis L550	E-value = 2.00E-98	Identity = 41.67%
	5		gi|153890172|ref|ZP_02011265.1|	Opitutaceae bacterium TAV2	E-value = 1.00E-73	Identity = 31.20%
AB1739	1		gi|57242328|ref|ZP_00370267.1|	Campylobacter upsaliensis RM3195	E-value = 9.00E-33	Identity = 39.09%
	2		gi|86152679|ref|ZP_01070884.1|	Campylobacter jejuni subsp. jejuni HB93-13	E-value = 1.00E-32	Identity = 39.55%
	3		gi|15792551|ref|NP_282374.1|	Campylobacter jejuni subsp. jejuni NCTC 11168	E-value = 3.00E-32	Identity = 39.09%
	4		gi|57168123|ref|ZP_00367262.1|	Campylobacter coli RM2228	E-value = 4.00E-32	Identity = 38.64%
	5		gi|86150781|ref|ZP_01068997.1|	Campylobacter jejuni subsp. jejuni 260.94	E-value = 1.00E-31	Identity = 38.64%
AB1740	1		gi|34557328|ref|NP_907143.1|	Wolinella succinogenes DSM 1740	E-value = 3.00E-58	Identity = 32.54%
	2		gi|117619052|ref|YP_855577.1|	Aeromonas hydrophila subsp. hydrophila ATCC 7966	E-value = 1.00E-52	Identity = 30.90%
	3		gi|144897509|emb|CAM74373.1|	Magnetospirillum gryphiswaldense MSR-1	E-value = 6.00E-47	Identity = 27.44%
	4		gi|30249816|ref|NP_841886.1|	Nitrosomonas europaea ATCC 19718	E-value = 5.00E-43	Identity = 27.93%
	5		gi|34557239|ref|NP_907054.1|	Wolinella succinogenes DSM 1740	E-value = 5.00E-42	Identity = 30.98%
AB1741	1			***** No matches found *****		
AB1742	1		gi|76260085|ref|ZP_00767726.1|	Chloroflexus aurantiacus J-10-fl	E-value = 2.00E-49	Identity = 25.18%
AB1743	1		gi|86139783|ref|ZP_01058349.1|	Roseobacter sp. MED193	E-value = 5.00E-50	Identity = 27.16%
	2		gi|34557239|ref|NP_907054.1|	Wolinella succinogenes DSM 1740	E-value = 2.00E-47	Identity = 32.09%
AB1744	1			***** No matches found *****		
AB1745	1		gi|84318419|ref|ZP_00966843.1|	Pseudomonas aeruginosa C3719	E-value = 1.00E-34	Identity = 33.58%
	2		gi|15595386|ref|NP_248878.1|	Pseudomonas aeruginosa PAO1	E-value = 6.00E-34	Identity = 33.21%
	3		gi|49083068|gb|AAT50934.1|	synthetic construct	E-value = 4.00E-33	Identity = 32.84%
	4		gi|76819292|ref|YP_335259.1|	Burkholderia pseudomallei 1710b	E-value = 4.00E-30	Identity = 30.11%
	5		gi|100235459|ref|ZP_01336193.1|	Burkholderia pseudomallei 406e	E-value = 5.00E-30	Identity = 30.32%
AB1746	1			***** No matches found *****		
AB1747	1			***** No matches found *****		

AB1748	1			***** No matches found *****		
AB1749	1		gi|118746165|ref|ZP_01594117.1|	Geobacter lovleyi SZ	E-value = 3.00E-49	Identity = 32.31%
	2		gi|77920163|ref|YP_357978.1|	Pelobacter carbinolicus DSM 2380	E-value = 4.00E-37	Identity = 29.78%
	3		gi|120601809|ref|YP_966209.1|	Desulfovibrio vulgaris subsp. vulgaris DP4	E-value = 1.00E-36	Identity = 28.11%
	4		gi|46580888|ref|YP_011696.1|	Desulfovibrio vulgaris subsp. vulgaris str. Hildenborough	E-value = 1.00E-36	Identity = 28.11%
	5		gi|34557150|ref|NP_906965.1|	Wolinella succinogenes DSM 1740	E-value = 2.00E-31	Identity = 26.38%
AB1750	1		gi|57240662|ref|ZP_00368610.1|	Campylobacter lari RM2100	E-value = 4.00E-14	Identity = 32.00%
	2		gi|57242734|ref|ZP_00370671.1|	Campylobacter upsaliensis RM3195	E-value = 2.00E-13	Identity = 27.35%
	3		gi|34556531|ref|NP_906346.1|	Wolinella succinogenes DSM 1740	E-value = 2.00E-13	Identity = 25.23%
	4		gi|154149275|ref|YP_001407283.1|	Campylobacter hominis ATCC BAA-381	E-value = 1.00E-12	Identity = 27.27%
	5		gi|32267155|ref|NP_861187.1|	Helicobacter hepaticus ATCC 51449	E-value = 4.00E-12	Identity = 29.33%
AB1751	1		gi|154174035|ref|YP_001407840.1|	Campylobacter curvus 525.92	E-value = 2.00E-31	Identity = 43.55%
	2		gi|42780539|ref|NP_977786.1|	Bacillus cereus ATCC 10987	E-value = 1.00E-29	Identity = 36.70%
	3		gi|30019499|ref|NP_831130.1|	Bacillus cereus ATCC 14579	E-value = 4.00E-29	Identity = 37.23%
	4		gi|52143988|ref|YP_082837.1|	Bacillus cereus E33L	E-value = 2.00E-28	Identity = 36.70%
	5		gi|152974874|ref|YP_001374391.1|	Bacillus cereus subsp. cytotoxis NVH 391-98	E-value = 2.00E-28	Identity = 35.29%
AB1752	1		gi|157164262|ref|YP_001465981.1|	Campylobacter concisus 13826	E-value = 2.00E-51	Identity = 31.87%
	2		gi|154174366|ref|YP_001407417.1|	Campylobacter curvus 525.92	E-value = 2.00E-50	Identity = 29.92%
	3		gi|154148592|ref|YP_001407034.1|	Campylobacter hominis ATCC BAA-381	E-value = 2.00E-50	Identity = 29.07%
	4		gi|118475351|ref|YP_892848.1|	Campylobacter fetus subsp. fetus 82-40	E-value = 2.00E-44	Identity = 28.71%
	5		gi|34556709|ref|NP_906524.1|	Wolinella succinogenes DSM 1740	E-value = 4.00E-43	Identity = 26.50%
AB1753	1		gi|154173905|ref|YP_001407416.1|	Campylobacter curvus 525.92	E-value = 4.00E-64	Identity = 47.71%
	2		gi|157165776|ref|YP_001465980.1|	Campylobacter concisus 13826	E-value = 1.00E-61	Identity = 47.06%
	3		gi|78357251|ref|YP_388700.1|	Desulfovibrio desulfuricans G20	E-value = 1.00E-60	Identity = 40.07%
	4		gi|152992656|ref|YP_001358377.1|	Sulfurovum sp. NBC37-1	E-value = 1.00E-59	Identity = 41.04%
	5		gi|116747475|ref|YP_844162.1|	Syntrophobacter fumaroxidans MPOB	E-value = 7.00E-59	Identity = 42.66%
AB1754	1		gi|106879748|emb|CAJ90443.1|	Leptospira biflexa serovar Patoc	E-value = 2.00E-11	Identity = 34.13%
	2		gi|154148068|ref|YP_001406103.1|	Campylobacter hominis ATCC BAA-381	E-value = 2.00E-09	Identity = 33.33%
	3		gi|149176351|ref|ZP_01854965.1|	Planctomyces maris DSM 8797	E-value = 2.00E-09	Identity = 29.84%
	4		gi|154685336|ref|YP_001420497.1|	Bacillus amyloliquefaciens FZB42	E-value = 3.00E-09	Identity = 32.31%
	5		gi|157164717|ref|YP_001465979.1|	Campylobacter concisus 13826	E-value = 3.00E-09	Identity = 35.29%
AB1755	1	*nhaA2*	gi|34557615|ref|NP_907430.1|	Wolinella succinogenes DSM 1740	E-value = 2.00E-92	Identity = 48.46%
	2		gi|108563926|ref|YP_628242.1|	Helicobacter pylori HPAG1	E-value = 5.00E-88	Identity = 47.86%
	3		gi|15612512|ref|NP_224165.1|	Helicobacter pylori J99	E-value = 1.00E-86	Identity = 47.14%
	4		gi|109946902|ref|YP_664130.1|	Helicobacter acinonychis str. Sheeba	E-value = 6.00E-86	Identity = 48.33%
	5		gi|15646159|ref|NP_208343.1|	Helicobacter pylori 26695	E-value = 2.00E-85	Identity = 47.07%
AB1756	1		gi|89093535|ref|ZP_01166483.1|	Oceanospirillum sp. MED92	E-value = 1.00E-117	Identity = 60.22%
	2		gi|15890039|ref|NP_355720.1|	Agrobacterium tumefaciens str. C58	E-value = 2.00E-107	Identity = 58.10%
	3		gi|86138431|ref|ZP_01057005.1|	Roseobacter sp. MED193	E-value = 2.00E-107	Identity = 54.55%
	4		gi|114764278|ref|ZP_01443506.1|	Roseovarius sp. HTCC2601	E-value = 9.00E-103	Identity = 54.52%
	5		gi|87121394|ref|ZP_01077283.1|	Marinomonas sp. MED121	E-value = 3.00E-101	Identity = 52.54%
AB1757	1		gi|148244802|ref|YP_001219496.1|	Candidatus Vesicomyosocius okutanii HA	E-value = 2.00E-74	Identity = 53.08%
	2		gi|88860485|ref|ZP_01135123.1|	Pseudoalteromonas tunicata D2	E-value = 4.00E-52	Identity = 39.73%
	3		gi|87122422|ref|ZP_01078302.1|	Marinomonas sp. MED121	E-value = 9.00E-49	Identity = 40.07%
	4		gi|119505418|ref|ZP_01627491.1|	marine gamma proteobacterium HTCC2080	E-value = 2.00E-46	Identity = 36.63%
	5		gi|88799344|ref|ZP_01114922.1|	Reinekea sp. MED297	E-value = 4.00E-45	Identity = 37.21%
AB1758	1		gi|148244803|ref|YP_001219497.1|	Candidatus Vesicomyosocius okutanii HA	E-value = 6.00E-53	Identity = 40.68%
AB1759	1		gi|118602701|ref|YP_903916.1|	Candidatus Ruthia magnifica str. Cm (Calyptogena magnifica)	E-value = 3.00E-117	Identity = 47.66%
	2		gi|148244804|ref|YP_001219498.1|	Candidatus Vesicomyosocius okutanii HA	E-value = 4.00E-112	Identity = 48.96%

	3		gi|66047290|ref|YP_237131.1|	Pseudomonas syringae pv. syringae B728a	E-value = 7.00E-92	Identity = 42.26%
	4		gi|88799523|ref|ZP_01115100.1|	Reinekea sp. MED297	E-value = 2.00E-91	Identity = 41.69%
	5		gi|84316919|ref|ZP_00965377.1|	Pseudomonas aeruginosa C3719	E-value = 1.00E-90	Identity = 41.10%
AB1760	1		gi|148244805|ref|YP_001219499.1|	Candidatus Vesicomyosocius okutanii HA	E-value = 1.00E-126	Identity = 54.42%
	2		gi|118602702|ref|YP_903917.1|	Candidatus Ruthia magnifica str. Cm (Calyptogena magnifica)	E-value = 4.00E-123	Identity = 55.09%
	3		gi|149921138|ref|ZP_01909596.1|	Plesiocystis pacifica SIR-1	E-value = 2.00E-54	Identity = 35.33%
	4		gi|124007535|ref|ZP_01692240.1|	Microscilla marina ATCC 23134	E-value = 4.00E-54	Identity = 39.42%
	5		gi|149927624|ref|ZP_01915877.1|	Limnobacter sp. MED105	E-value = 3.00E-52	Identity = 39.10%
AB1761	1		gi|34556620|ref|NP_906435.1|	Wolinella succinogenes DSM 1740	E-value = 1.00E-61	Identity = 26.96%
	2		gi|78777839|ref|YP_394154.1|	Sulfuromonas denitrificans ATCC 33889	E-value = 7.00E-59	Identity = 27.27%
	3		gi|32266498|ref|NP_860530.1|	Helicobacter hepaticus ATCC 51449	E-value = 2.00E-53	Identity = 26.91%
	4		gi|154175302|ref|YP_001408487.1|	Campylobacter curvus 525.92	E-value = 4.00E-48	Identity = 26.88%
AB1763	1	*atpE*	gi|118474883|ref|YP_892437.1|	Campylobacter fetus subsp. fetus 82-40	E-value = 2.00E-18	Identity = 77.88%
	2		gi|15792265|ref|NP_282088.1|	Campylobacter jejuni subsp. jejuni NCTC 11168	E-value = 4.00E-17	Identity = 72.48%
	3		gi|57242302|ref|ZP_00370241.1|	Campylobacter upsaliensis RM3195	E-value = 6.00E-17	Identity = 71.30%
	4		gi|57240616|ref|ZP_00368565.1|	Campylobacter lari RM2100	E-value = 8.00E-17	Identity = 81.37%
	5		gi|153951147|ref|YP_001397995.1|	Campylobacter jejuni subsp. doylei 269.97	E-value = 4.00E-16	Identity = 81.18%
AB1764	1		gi|57241710|ref|ZP_00369655.1|	Campylobacter lari RM2100	E-value = 1.00E-63	Identity = 30.40%
	2		gi|34557692|ref|NP_907507.1|	Wolinella succinogenes DSM 1740	E-value = 1.00E-63	Identity = 29.97%
	3		gi|154175075|ref|YP_001408197.1|	Campylobacter curvus 525.92	E-value = 8.00E-63	Identity = 27.91%
	4		gi|153951071|ref|YP_001398694.1|	Campylobacter jejuni subsp. doylei 269.97	E-value = 2.00E-62	Identity = 29.85%
	5		gi|118474782|ref|YP_892818.1|	Campylobacter fetus subsp. fetus 82-40	E-value = 3.00E-61	Identity = 30.23%
AB1765	1		gi|146293482|ref|YP_001183906.1|	Shewanella putrefaciens CN-32	E-value = 3.00E-55	Identity = 38.39%
	2		gi|24374447|ref|NP_718490.1|	Shewanella oneidensis MR-1	E-value = 4.00E-55	Identity = 38.44%
	3		gi|120598439|ref|YP_963013.1|	Shewanella sp. W3-18-1	E-value = 5.00E-55	Identity = 38.39%
	4		gi|118072429|ref|ZP_01540618.1|	Shewanella woodyi ATCC 51908	E-value = 1.00E-54	Identity = 37.61%
	5		gi|117919992|ref|YP_869184.1|	Shewanella sp. ANA-3	E-value = 3.00E-54	Identity = 38.14%
AB1766	1		gi|146284065|ref|YP_001174218.1|	Pseudomonas stutzeri A1501	E-value = 2.00E-72	Identity = 52.47%
	2		gi|118589326|ref|ZP_01546732.1|	Stappia aggregata IAM 12614	E-value = 2.00E-66	Identity = 49.61%
	3		gi|114707297|ref|ZP_01440194.1|	Fulvimarina pelagi HTCC2506	E-value = 7.00E-63	Identity = 48.85%
	4		gi|86136791|ref|ZP_01055369.1|	Roseobacter sp. MED193	E-value = 3.00E-59	Identity = 45.88%
	5		gi|146291693|ref|YP_001182117.1|	Shewanella putrefaciens CN-32	E-value = 7.00E-59	Identity = 49.79%
AB1767	1			***** No matches found *****		
AB1768	1			***** No matches found *****		
AB1769	1		gi|34558044|ref|NP_907859.1|	Wolinella succinogenes DSM 1740	E-value = 4.00E-11	Identity = 43.66%
	2		gi|152993990|ref|YP_001359711.1|	Sulfurovum sp. NBC37-1	E-value = 2.00E-08	Identity = 39.71%
	3		gi|154147962|ref|YP_001407002.1|	Campylobacter hominis ATCC BAA-381	E-value = 4.00E-06	Identity = 41.79%
AB1770	1	*thiD*	gi|34557186|ref|NP_907001.1|	Wolinella succinogenes DSM 1740	E-value = 6.00E-53	Identity = 48.06%
	2		gi|85374878|ref|YP_458940.1|	Erythrobacter litoralis HTCC2594	E-value = 2.00E-45	Identity = 38.04%
	3		gi|85709356|ref|ZP_01040421.1|	Erythrobacter sp. NAP1	E-value = 1.00E-44	Identity = 40.47%
	4		gi|154503371|ref|ZP_02040431.1|	Ruminococcus gnavus ATCC 29149	E-value = 4.00E-44	Identity = 40.54%
	5		gi|83592438|ref|YP_426190.1|	Rhodospirillum rubrum ATCC 11170	E-value = 1.00E-42	Identity = 37.65%
AB1771	1	*proA*	gi|152993343|ref|YP_001359064.1|	Sulfurovum sp. NBC37-1	E-value = 4.00E-150	Identity = 64.88%
	2		gi|152990608|ref|YP_001356330.1|	Nitratiruptor sp. SB155-2	E-value = 3.00E-142	Identity = 61.89%
	3		gi|78776991|ref|YP_393306.1|	Sulfuromonas denitrificans ATCC 33889	E-value = 1.00E-139	Identity = 61.46%
	4		gi|149193842|ref|ZP_01870940.1|	Caminibacter mediatlanticus TB-2	E-value = 1.00E-137	Identity = 59.12%
	5		gi|34557662|ref|NP_907477.1|	Wolinella succinogenes DSM 1740	E-value = 2.00E-134	Identity = 58.64%
AB1772	1		gi|78776269|ref|YP_392584.1|	Sulfuromonas denitrificans ATCC 33889	E-value = 9.00E-176	Identity = 53.46%
	2		gi|91775726|ref|YP_545482.1|	Methylobacillus flagellatus KT	E-value = 1.00E-72	Identity = 32.19%

	3		gi|56478932|ref|YP_160521.1|	Azoarcus sp. EbN1	E-value = 4.00E-72	Identity = 33.73%
	4		gi|27379723|ref|NP_771252.1|	Bradyrhizobium japonicum USDA 110	E-value = 9.00E-67	Identity = 31.61%
	5		gi|146340705|ref|YP_001205753.1|	Bradyrhizobium sp. ORS278	E-value = 1.00E-65	Identity = 31.02%
AB1773	1			***** No matches found *****		
AB1774	1		gi|152991668|ref|YP_001357389.1|	Sulfurovum sp. NBC37-1	E-value = 3.00E-114	Identity = 48.67%
	2		gi|118474306|ref|YP_891331.1|	Campylobacter fetus subsp. fetus 82-40	E-value = 9.00E-110	Identity = 47.63%
	3		gi|34557050|ref|NP_906865.1|	Wolinella succinogenes DSM 1740	E-value = 8.00E-109	Identity = 48.76%
	4		gi|152991354|ref|YP_001357076.1|	Nitratiruptor sp. SB155-2	E-value = 2.00E-108	Identity = 45.19%
	5		gi|152989894|ref|YP_001355616.1|	Nitratiruptor sp. SB155-2	E-value = 5.00E-108	Identity = 45.19%
AB1775	1			***** No matches found *****		
AB1776	1		gi|34557780|ref|NP_907595.1|	Wolinella succinogenes DSM 1740	E-value = 6.00E-35	Identity = 40.28%
	2		gi|118475245|ref|YP_891579.1|	Campylobacter fetus subsp. fetus 82-40	E-value = 6.00E-28	Identity = 32.74%
	3		gi|152993408|ref|YP_001359129.1|	Sulfurovum sp. NBC37-1	E-value = 2.00E-27	Identity = 35.11%
	4		gi|34558806|gb|AAQ75151.1|	Alvinella pompejana epibiont 6C6	E-value = 1.00E-26	Identity = 35.75%
	5		gi|157164839|ref|YP_001466181.1|	Campylobacter concisus 13826	E-value = 4.00E-26	Identity = 33.63%
AB1777	1	*ogt*	gi|151572627|gb|EDN38281.1|	Francisella tularensis subsp. novicida GA99-3548	E-value = 2.00E-32	Identity = 46.75%
	2		gi|151571175|gb|EDN36829.1|	Francisella tularensis subsp. novicida GA99-3549	E-value = 7.00E-32	Identity = 46.15%
	3		gi|56708554|ref|YP_170450.1|	Francisella tularensis subsp. tularensis SCHU S4	E-value = 2.00E-31	Identity = 45.51%
	4		gi|54113435|gb|AAV29351.1|	synthetic construct	E-value = 3.00E-31	Identity = 45.51%
	5		gi|89255707|ref|YP_513068.1|	Francisella tularensis subsp. holarctica	E-value = 1.00E-30	Identity = 44.91%
AB1778	1		gi|152993517|ref|YP_001359238.1|	Sulfurovum sp. NBC37-1	E-value = 1.00E-130	Identity = 50.32%
	2		gi|152991170|ref|YP_001356892.1|	Nitratiruptor sp. SB155-2	E-value = 4.00E-130	Identity = 50.11%
	3		gi|78776822|ref|YP_393137.1|	Sulfuromonas denitrificans ATCC 33889	E-value = 4.00E-126	Identity = 50.43%
	4		gi|34558288|ref|NP_908103.1|	Wolinella succinogenes DSM 1740	E-value = 7.00E-116	Identity = 47.00%
	5		gi|118475128|ref|YP_891312.1|	Campylobacter fetus subsp. fetus 82-40	E-value = 1.00E-113	Identity = 49.44%
AB1779	1		gi|34556848|ref|NP_906663.1|	Wolinella succinogenes DSM 1740	E-value = 1.00E-63	Identity = 51.49%
	2		gi|152989799|ref|YP_001355521.1|	Nitratiruptor sp. SB155-2	E-value = 8.00E-58	Identity = 48.52%
	3		gi|32266104|ref|NP_860136.1|	Helicobacter hepaticus ATCC 51449	E-value = 1.00E-55	Identity = 50.21%
	4		gi|152993971|ref|YP_001359692.1|	Sulfurovum sp. NBC37-1	E-value = 8.00E-52	Identity = 43.35%
	5		gi|78776215|ref|YP_392530.1|	Sulfuromonas denitrificans ATCC 33889	E-value = 3.00E-50	Identity = 41.78%
AB1780	1		gi|34419513|ref|NP_899526.1|	Vibrio phage KVP40	E-value = 9.00E-17	Identity = 35.20%
	2		gi|38707641|ref|NP_944779.1|	Bacteriophage Felix 01	E-value = 1.00E-15	Identity = 36.08%
	3		gi|38707794|ref|NP_944780.1|	Bacteriophage Felix 01	E-value = 3.00E-14	Identity = 37.32%
	4		gi|38640206|ref|NP_944162.1|	Bacteriophage Aeh1	E-value = 9.00E-13	Identity = 29.41%
AB1781	1		gi|57240940|ref|ZP_00368888.1|	Campylobacter lari RM2100	E-value = 5.00E-30	Identity = 63.37%
	2		gi|118474607|ref|YP_891318.1|	Campylobacter fetus subsp. fetus 82-40	E-value = 1.00E-27	Identity = 60.00%
	3		gi|15792707|ref|NP_282530.1|	Campylobacter jejuni subsp. jejuni NCTC 11168	E-value = 1.00E-27	Identity = 61.76%
	4		gi|154175460|ref|YP_001407590.1|	Campylobacter curvus 525.92	E-value = 2.00E-27	Identity = 57.28%
	5		gi|157165497|ref|YP_001466149.1|	Campylobacter concisus 13826	E-value = 3.00E-27	Identity = 60.00%
AB1782	1		gi|157165012|ref|YP_001466150.1|	Campylobacter concisus 13826	E-value = 2.00E-06	Identity = 26.70%
AB1783	1	*fldA*	gi|156108607|gb|EDO10352.1|	Bacteroides ovatus ATCC 8483	E-value = 5.00E-35	Identity = 49.69%
	2		gi|399495|sp|P31158|FLAV_SYNP2	Synechococcus sp. PCC 7002	E-value = 3.00E-34	Identity = 47.83%
	3		gi|153806151|ref|ZP_01958819.1|	Bacteroides caccae ATCC 43185	E-value = 8.00E-34	Identity = 47.24%
	4		gi|15792705|ref|NP_282528.1|	Campylobacter jejuni subsp. jejuni NCTC 11168	E-value = 8.00E-34	Identity = 49.69%
	5		gi|150005724|ref|YP_001300468.1|	Bacteroides vulgatus ATCC 8482	E-value = 1.00E-33	Identity = 49.08%
AB1784	1	*fur2*	gi|157164982|ref|YP_001467460.1|	Campylobacter concisus 13826	E-value = 5.00E-37	Identity = 51.68%
	2		gi|154174593|ref|YP_001407788.1|	Campylobacter curvus 525.92	E-value = 5.00E-37	Identity = 52.35%
	3		gi|57168539|ref|ZP_00367672.1|	Campylobacter coli RM2228	E-value = 6.00E-37	Identity = 54.00%
	4		gi|118474524|ref|YP_892301.1|	Campylobacter fetus subsp. fetus 82-40	E-value = 2.00E-36	Identity = 51.75%

	5		gi|15791767|ref|NP_281590.1|	Campylobacter jejuni subsp. jejuni NCTC 11168	E-value = 2.00E-36	Identity = 53.38%
AB1785	1			***** No matches found *****		
AB1786	1	*ate*	gi|34557640|ref|NP_907455.1|	Wolinella succinogenes DSM 1740	E-value = 9.00E-67	Identity = 52.79%
	2		gi|32267328|ref|NP_861360.1|	Helicobacter hepaticus ATCC 51449	E-value = 2.00E-60	Identity = 45.73%
	3		gi|152989910|ref|YP_001355632.1|	Nitratiruptor sp. SB155-2	E-value = 1.00E-56	Identity = 47.35%
	4		gi|152993285|ref|YP_001359006.1|	Sulfurovum sp. NBC37-1	E-value = 4.00E-55	Identity = 46.02%
	5		gi|153951214|ref|YP_001397883.1|	Campylobacter jejuni subsp. doylei 269.97	E-value = 2.00E-53	Identity = 49.56%
AB1787	1	*trpA*	gi|152993675|ref|YP_001359396.1|	Sulfurovum sp. NBC37-1	E-value = 4.00E-83	Identity = 61.89%
	2		gi|152990123|ref|YP_001355845.1|	Nitratiruptor sp. SB155-2	E-value = 5.00E-79	Identity = 58.37%
	3		gi|78777878|ref|YP_394193.1|	Sulfuromonas denitrificans ATCC 33889	E-value = 6.00E-74	Identity = 57.96%
	4		gi|34558127|ref|NP_907942.1|	Wolinella succinogenes DSM 1740	E-value = 4.00E-71	Identity = 57.32%
	5		gi|149195241|ref|ZP_01872330.1|	Caminibacter mediatlanticus TB-2	E-value = 9.00E-68	Identity = 56.10%
AB1788	1		gi|152991483|ref|YP_001357205.1|	Nitratiruptor sp. SB155-2	E-value = 3.00E-132	Identity = 59.00%
	2		gi|152991861|ref|YP_001357582.1|	Sulfurovum sp. NBC37-1	E-value = 7.00E-126	Identity = 58.28%
	3		gi|145617940|ref|ZP_01774002.1|	Geobacter bemidjiensis Bem	E-value = 3.00E-84	Identity = 44.50%
	4		gi|118581673|ref|YP_902923.1|	Pelobacter propionicus DSM 2379	E-value = 1.00E-76	Identity = 40.75%
	5		gi|83748726|ref|ZP_00945742.1|	Ralstonia solanacearum UW551	E-value = 5.00E-63	Identity = 35.82%
AB1789	1	*panB*	gi|78777876|ref|YP_394191.1|	Sulfuromonas denitrificans ATCC 33889	E-value = 7.00E-97	Identity = 67.43%
	2		gi|152990122|ref|YP_001355844.1|	Nitratiruptor sp. SB155-2	E-value = 6.00E-94	Identity = 64.37%
	3		gi|149195242|ref|ZP_01872331.1|	Caminibacter mediatlanticus TB-2	E-value = 9.00E-88	Identity = 65.59%
	4		gi|152993674|ref|YP_001359395.1|	Sulfurovum sp. NBC37-1	E-value = 8.00E-86	Identity = 60.15%
	5		gi|109947699|ref|YP_664927.1|	Helicobacter acinonychis str. Sheeba	E-value = 4.00E-83	Identity = 54.65%
AB1790	1	*ruvB*	gi|152993673|ref|YP_001359394.1|	Sulfurovum sp. NBC37-1	E-value = 3.00E-139	Identity = 76.45%
	2		gi|78777875|ref|YP_394190.1|	Sulfuromonas denitrificans ATCC 33889	E-value = 2.00E-135	Identity = 74.24%
	3		gi|157165204|ref|YP_001467229.1|	Campylobacter concisus 13826	E-value = 5.00E-134	Identity = 72.78%
	4		gi|154174013|ref|YP_001408691.1|	Campylobacter curvus 525.92	E-value = 3.00E-132	Identity = 71.56%
	5		gi|152990121|ref|YP_001355843.1|	Nitratiruptor sp. SB155-2	E-value = 7.00E-131	Identity = 74.09%
AB1791	1	*amaA*	gi|152990120|ref|YP_001355842.1|	Nitratiruptor sp. SB155-2	E-value = 4.00E-54	Identity = 41.23%
	2		gi|154173718|ref|YP_001408692.1|	Campylobacter curvus 525.92	E-value = 6.00E-53	Identity = 41.61%
	3		gi|152993672|ref|YP_001359393.1|	Sulfurovum sp. NBC37-1	E-value = 1.00E-52	Identity = 38.87%
	4		gi|157164430|ref|YP_001467226.1|	Campylobacter concisus 13826	E-value = 2.00E-52	Identity = 41.37%
	5		gi|154149249|ref|YP_001406317.1|	Campylobacter hominis ATCC BAA-381	E-value = 3.00E-52	Identity = 37.09%
AB1792	1		gi|152993921|ref|YP_001359642.1|	Sulfurovum sp. NBC37-1	E-value = 8.00E-69	Identity = 54.73%
	2		gi|78777571|ref|YP_393886.1|	Sulfuromonas denitrificans ATCC 33889	E-value = 3.00E-64	Identity = 50.00%
	3		gi|149195059|ref|ZP_01872151.1|	Caminibacter mediatlanticus TB-2	E-value = 1.00E-46	Identity = 43.50%
AB1795	1		gi|152993193|ref|YP_001358914.1|	Sulfurovum sp. NBC37-1	E-value = 1.00E-78	Identity = 53.95%
	2		gi|157164348|ref|YP_001467204.1|	Campylobacter concisus 13826	E-value = 6.00E-75	Identity = 49.16%
	3		gi|34556807|ref|NP_906622.1|	Wolinella succinogenes DSM 1740	E-value = 7.00E-69	Identity = 46.31%
	4		gi|152990234|ref|YP_001355956.1|	Nitratiruptor sp. SB155-2	E-value = 2.00E-67	Identity = 48.82%
	5		gi|57241003|ref|ZP_00368950.1|	Campylobacter lari RM2100	E-value = 2.00E-64	Identity = 45.18%
AB1796	1	*hemE*	gi|152993199|ref|YP_001358920.1|	Sulfurovum sp. NBC37-1	E-value = 1.00E-175	Identity = 83.77%
	2		gi|78776833|ref|YP_393148.1|	Sulfuromonas denitrificans ATCC 33889	E-value = 3.00E-171	Identity = 82.90%
	3		gi|152990235|ref|YP_001355957.1|	Nitratiruptor sp. SB155-2	E-value = 2.00E-134	Identity = 66.76%
	4		gi|154149184|ref|YP_001406819.1|	Campylobacter hominis ATCC BAA-381	E-value = 8.00E-131	Identity = 62.65%
	5		gi|57241002|ref|ZP_00368949.1|	Campylobacter lari RM2100	E-value = 5.00E-129	Identity = 64.91%
AB1797	1		gi|78776834|ref|YP_393149.1|	Sulfuromonas denitrificans ATCC 33889	E-value = 2.00E-49	Identity = 65.29%
	2		gi|152993200|ref|YP_001358921.1|	Sulfurovum sp. NBC37-1	E-value = 8.00E-46	Identity = 69.01%
	3		gi|114777342|ref|ZP_01452339.1|	Mariprofundus ferrooxydans PV-1	E-value = 1.00E-45	Identity = 61.76%
	4		gi|152990236|ref|YP_001355958.1|	Nitratiruptor sp. SB155-2	E-value = 3.00E-41	Identity = 61.82%

	5		gi|149193740|ref|ZP_01870838.1|	Caminibacter mediatlanticus TB-2	E-value = 4.00E-38	Identity = 56.36%
AB1798	1	*asd*	gi|152993201|ref|YP_001358922.1|	Sulfurovum sp. NBC37-1	E-value = 4.00E-147	Identity = 77.33%
	2		gi|154175060|ref|YP_001408746.1|	Campylobacter curvus 525.92	E-value = 1.00E-128	Identity = 69.12%
	3		gi|118474950|ref|YP_892594.1|	Campylobacter fetus subsp. fetus 82-40	E-value = 3.00E-128	Identity = 67.25%
	4		gi|57241000|ref|ZP_00368947.1|	Campylobacter lari RM2100	E-value = 2.00E-126	Identity = 67.86%
	5		gi|57168893|ref|ZP_00368023.1|	Campylobacter coli RM2228	E-value = 4.00E-126	Identity = 66.67%
AB1799	1	*gyrA*	gi|118574930|gb|ABL07042.1|	Arcobacter cryaerophilus	E-value = 0	Identity = 92.67%
	2		gi|118574932|gb|ABL07043.1|	Arcobacter cibarius	E-value = 0	Identity = 91.93%
	3		gi|118574934|gb|ABL07044.1|	Arcobacter skirrowii	E-value = 0	Identity = 87.24%
	4		gi|118647919|gb|ABL09947.1|	Arcobacter nitrofigilis	E-value = 0	Identity = 83.04%
	5		gi|152993202|ref|YP_001358923.1|	Sulfurovum sp. NBC37-1	E-value = 0	Identity = 73.18%
AB1800	1		gi|118030969|ref|ZP_01502425.1|	Burkholderia phymatum STM815	E-value = 1.00E-08	Identity = 56.06%
	2		gi|91777490|ref|YP_552698.1|	Burkholderia xenovorans LB400	E-value = 3.00E-08	Identity = 51.47%
	3		gi|121583416|ref|YP_973847.1|	Polaromonas naphthalenivorans CJ2	E-value = 3.00E-07	Identity = 50.00%
	4		gi|94313780|ref|YP_586989.1|	Ralstonia metallidurans CH34	E-value = 7.00E-07	Identity = 50.00%
	5		gi|73540816|ref|YP_295336.1|	Ralstonia eutropha JMP134	E-value = 9.00E-07	Identity = 53.03%
AB1801	1	*argJ*	gi|152990067|ref|YP_001355789.1|	Nitratiruptor sp. SB155-2	E-value = 5.00E-125	Identity = 59.90%
	2		gi|118475747|ref|YP_892141.1|	Campylobacter fetus subsp. fetus 82-40	E-value = 4.00E-122	Identity = 58.73%
	3		gi|78777797|ref|YP_394112.1|	Sulfuromonas denitrificans ATCC 33889	E-value = 1.00E-120	Identity = 58.99%
	4		gi|154174867|ref|YP_001408513.1|	Campylobacter curvus 525.92	E-value = 7.00E-120	Identity = 56.68%
	5		gi|152993736|ref|YP_001359457.1|	Sulfurovum sp. NBC37-1	E-value = 1.00E-117	Identity = 51.90%
AB1802	1		gi|34556764|ref|NP_906579.1|	Wolinella succinogenes DSM 1740	E-value = 2.00E-111	Identity = 55.47%
	2		gi|32266789|ref|NP_860821.1|	Helicobacter hepaticus ATCC 51449	E-value = 5.00E-102	Identity = 48.26%
	3		gi|152993735|ref|YP_001359456.1|	Sulfurovum sp. NBC37-1	E-value = 1.00E-98	Identity = 49.20%
	4		gi|78777798|ref|YP_394113.1|	Sulfuromonas denitrificans ATCC 33889	E-value = 3.00E-97	Identity = 49.34%
	5		gi|157165610|ref|YP_001466761.1|	Campylobacter concisus 13826	E-value = 2.00E-95	Identity = 47.61%
AB1803	1	*rpmB*	gi|148925829|ref|ZP_01809516.1|	Campylobacter jejuni subsp. jejuni CG8486	E-value = 3.00E-13	Identity = 75.81%
	2		gi|78777799|ref|YP_394114.1|	Sulfuromonas denitrificans ATCC 33889	E-value = 8.00E-13	Identity = 77.42%
	3		gi|153951942|ref|YP_001398512.1|	Campylobacter jejuni subsp. doylei 269.97	E-value = 9.00E-13	Identity = 75.81%
	4		gi|154173798|ref|YP_001408511.1|	Campylobacter curvus 525.92	E-value = 1.00E-12	Identity = 74.19%
	5		gi|118475728|ref|YP_892139.1|	Campylobacter fetus subsp. fetus 82-40	E-value = 1.00E-12	Identity = 74.19%
AB1804	1			***** No matches found *****		
AB1805	1		gi|100064556|ref|ZP_01325947.1|	Burkholderia pseudomallei Pasteur	E-value = 2.00E-20	Identity = 43.36%
	2		gi|303564|dbj|BAA03661.1|	Escherichia coli W3110	E-value = 1.00E-18	Identity = 33.33%
AB1806	1	*waaD*	gi|78776778|ref|YP_393093.1|	Sulfuromonas denitrificans ATCC 33889	E-value = 7.00E-126	Identity = 69.94%
	2		gi|152991191|ref|YP_001356913.1|	Nitratiruptor sp. SB155-2	E-value = 4.00E-109	Identity = 62.80%
	3		gi|154175499|ref|YP_001408599.1|	Campylobacter curvus 525.92	E-value = 2.00E-102	Identity = 60.42%
	4		gi|157164747|ref|YP_001467035.1|	Campylobacter concisus 13826	E-value = 6.00E-99	Identity = 59.82%
	5		gi|118474525|ref|YP_892553.1|	Campylobacter fetus subsp. fetus 82-40	E-value = 5.00E-98	Identity = 62.04%
AB1807	1	*waaE*	gi|78776779|ref|YP_393094.1|	Sulfuromonas denitrificans ATCC 33889	E-value = 2.00E-158	Identity = 62.92%
	2		gi|34557093|ref|NP_906908.1|	Wolinella succinogenes DSM 1740	E-value = 1.00E-142	Identity = 56.10%
	3		gi|152991190|ref|YP_001356912.1|	Nitratiruptor sp. SB155-2	E-value = 2.00E-140	Identity = 56.99%
	4		gi|154174008|ref|YP_001408598.1|	Campylobacter curvus 525.92	E-value = 4.00E-129	Identity = 53.81%
	5		gi|118475685|ref|YP_892552.1|	Campylobacter fetus subsp. fetus 82-40	E-value = 2.00E-127	Identity = 54.64%
AB1808	1	*gmhA*	gi|34557094|ref|NP_906909.1|	Wolinella succinogenes DSM 1740	E-value = 6.00E-64	Identity = 66.30%
	2		gi|32267030|ref|NP_861062.1|	Helicobacter hepaticus ATCC 51449	E-value = 3.00E-63	Identity = 63.98%
	3		gi|154173847|ref|YP_001408597.1|	Campylobacter curvus 525.92	E-value = 5.00E-63	Identity = 68.33%
	4		gi|108563265|ref|YP_627581.1|	Helicobacter pylori HPAG1	E-value = 1.00E-61	Identity = 63.78%
	5		gi|118475208|ref|YP_892550.1|	Campylobacter fetus subsp. fetus 82-40	E-value = 4.00E-61	Identity = 67.03%

AB1809	1		gi|78776520|ref|YP_392835.1|	Sulfuromonas denitrificans ATCC 33889	E-value = 6.00E-169	Identity = 51.64%
	2		gi|153093040|gb|EDN74056.1|	Mannheimia haemolytica PHL213	E-value = 8.00E-115	Identity = 39.06%
	3		gi|146302750|ref|YP_001197341.1|	Flavobacterium johnsoniae UW101	E-value = 4.00E-114	Identity = 40.06%
	4		gi|126208280|ref|YP_001053505.1|	Actinobacillus pleuropneumoniae L20	E-value = 7.00E-112	Identity = 39.60%
	5		gi|46143507|ref|ZP_00135083.2|	Actinobacillus pleuropneumoniae serovar 1 str. 4074	E-value = 7.00E-112	Identity = 39.60%
AB1810	1	*waaF*	gi|157163923|ref|YP_001467320.1|	Campylobacter concisus 13826	E-value = 6.00E-82	Identity = 49.52%
	2		gi|154173877|ref|YP_001407683.1|	Campylobacter curvus 525.92	E-value = 2.00E-81	Identity = 46.28%
	3		gi|118474261|ref|YP_892549.1|	Campylobacter fetus subsp. fetus 82-40	E-value = 4.00E-77	Identity = 50.51%
	4		gi|78776781|ref|YP_393096.1|	Sulfuromonas denitrificans ATCC 33889	E-value = 9.00E-75	Identity = 43.96%
	5		gi|57168029|ref|ZP_00367168.1|	Campylobacter coli RM2228	E-value = 4.00E-74	Identity = 46.50%
AB1811	1		gi|78776782|ref|YP_393097.1|	Sulfuromonas denitrificans ATCC 33889	E-value = 6.00E-80	Identity = 47.28%
	2		gi|54298941|ref|YP_125310.1|	Legionella pneumophila str. Paris	E-value = 2.00E-68	Identity = 41.46%
AB1812	1		gi|78776784|ref|YP_393099.1|	Sulfuromonas denitrificans ATCC 33889	E-value = 3.00E-52	Identity = 39.06%
	2		gi|149189222|ref|ZP_01867509.1|	Vibrio shilonii AK1	E-value = 5.00E-42	Identity = 34.98%
	3		gi|37678477|ref|NP_933086.1|	Vibrio vulnificus YJ016	E-value = 9.00E-42	Identity = 34.54%
	4		gi|126207905|ref|YP_001053130.1|	Actinobacillus pleuropneumoniae L20	E-value = 9.00E-40	Identity = 34.42%
	5		gi|15603159|ref|NP_246231.1|	Pasteurella multocida subsp. multocida str. Pm70	E-value = 1.00E-39	Identity = 35.00%
AB1813	1		gi|78776786|ref|YP_393101.1|	Sulfuromonas denitrificans ATCC 33889	E-value = 6.00E-48	Identity = 48.28%
	2		gi|54298940|ref|YP_125309.1|	Legionella pneumophila str. Paris	E-value = 5.00E-37	Identity = 40.83%
	3		gi|60679778|ref|YP_209922.1|	Bacteroides fragilis NCTC 9343	E-value = 1.00E-33	Identity = 32.60%
	4		gi|53711515|ref|YP_097507.1|	Bacteroides fragilis YCH46	E-value = 2.00E-33	Identity = 32.60%
	5		gi|149370606|ref|ZP_01890295.1|	unidentified eubacterium SCB49	E-value = 3.00E-32	Identity = 37.44%
AB1814	1		gi|152991157|ref|YP_001356879.1|	Nitratiruptor sp. SB155-2	E-value = 2.00E-48	Identity = 31.84%
	2		gi|149189229|ref|ZP_01867516.1|	Vibrio shilonii AK1	E-value = 2.00E-48	Identity = 27.22%
	3		gi|94501600|ref|ZP_01308117.1|	Oceanobacter sp. RED65	E-value = 7.00E-42	Identity = 30.91%
	4		gi|15677836|ref|NP_275002.1|	Neisseria meningitidis MC58	E-value = 3.00E-39	Identity = 26.77%
	5		gi|121635666|ref|YP_975911.1|	Neisseria meningitidis FAM18	E-value = 4.00E-39	Identity = 27.51%
AB1815	1		gi|157148778|ref|YP_001456097.1|	Citrobacter koseri ATCC BAA-895	E-value = 2.00E-22	Identity = 31.55%
	2		gi|68552600|ref|ZP_00591988.1|	Prosthecochloris aestuarii DSM 271	E-value = 3.00E-22	Identity = 32.79%
	3		gi|74313744|ref|YP_312163.1|	Shigella sonnei Ss046	E-value = 4.00E-22	Identity = 31.55%
	4		gi|75235393|ref|ZP_00719606.1|	Escherichia coli F11	E-value = 5.00E-22	Identity = 31.55%
	5		gi|145219499|ref|YP_001130208.1|	Prosthecochloris vibrioformis DSM 265	E-value = 5.00E-22	Identity = 33.16%
AB1816	1			***** No matches found *****		
AB1817	1		gi|118602180|ref|YP_903395.1|	Candidatus Ruthia magnifica str. Cm (Calyptogena magnifica)	E-value = 2.00E-157	Identity = 64.96%
	2		gi|152993121|ref|YP_001358842.1|	Sulfurovum sp. NBC37-1	E-value = 2.00E-142	Identity = 61.02%
	3		gi|154174890|ref|YP_001407538.1|	Campylobacter curvus 525.92	E-value = 8.00E-140	Identity = 61.52%
	4		gi|114565689|ref|YP_752843.1|	Syntrophomonas wolfei subsp. wolfei str. Goettingen	E-value = 1.00E-136	Identity = 58.02%
	5		gi|52144432|ref|YP_082397.1|	Bacillus cereus E33L	E-value = 5.00E-131	Identity = 56.10%
AB1818	1		gi|94264214|ref|ZP_01288010.1|	delta proteobacterium MLMS-1	E-value = 6.00E-62	Identity = 34.53%
	2		gi|119944121|ref|YP_941801.1|	Psychromonas ingrahamii 37	E-value = 2.00E-41	Identity = 32.05%
	3		gi|92112149|ref|YP_572077.1|	Chromohalobacter salexigens DSM 3043	E-value = 3.00E-27	Identity = 26.10%
	4		gi|124006976|ref|ZP_01691805.1|	Microscilla marina ATCC 23134	E-value = 3.00E-26	Identity = 27.10%
	5		gi|20807430|ref|NP_622601.1|	Thermoanaerobacter tengcongensis MB4	E-value = 8.00E-22	Identity = 32.30%
AB1819	1		gi|152997483|ref|YP_001342318.1|	Marinomonas sp. MWYL1	E-value = 8.00E-60	Identity = 47.18%
	2		gi|94264206|ref|ZP_01288002.1|	delta proteobacterium MLMS-1	E-value = 4.00E-59	Identity = 48.74%
	3		gi|92112147|ref|YP_572075.1|	Chromohalobacter salexigens DSM 3043	E-value = 3.00E-58	Identity = 44.98%
	4		gi|87119522|ref|ZP_01075419.1|	Marinomonas sp. MED121	E-value = 6.00E-57	Identity = 46.37%
	5		gi|126662046|ref|ZP_01733045.1|	Flavobacteria bacterium BAL38	E-value = 6.00E-50	Identity = 41.77%
AB1820	1		gi|118474603|ref|YP_892527.1|	Campylobacter fetus subsp. fetus 82-40	E-value = 2.00E-08	Identity = 26.28%

	2		gi|729025|sp|P39857|CAPH_STAAU	Staphylococcus aureus	E-value = 5.00E-08	Identity = 26.32%
AB1821	1		gi|84489253|ref|YP_447485.1|	Methanosphaera stadtmanae DSM 3091	E-value = 1.00E-27	Identity = 33.33%
	2		gi|156869426|gb|EDO62798.1|	Clostridium leptum DSM 753	E-value = 2.00E-25	Identity = 32.97%
	3		gi|150008821|ref|YP_001303564.1|	Parabacteroides distasonis ATCC 8503	E-value = 2.00E-25	Identity = 32.45%
	4		gi|14389021|gb|AAK61903.1|AF373595_12	Streptococcus thermophilus	E-value = 3.00E-25	Identity = 32.35%
	5		gi|60683259|ref|YP_213403.1|	Bacteroides fragilis NCTC 9343	E-value = 3.00E-24	Identity = 31.56%
AB1822	1		gi|152995829|ref|YP_001340664.1|	Marinomonas sp. MWYL1	E-value = 3.00E-20	Identity = 35.48%
	2		gi|75235393|ref|ZP_00719606.1|	Escherichia coli F11	E-value = 1.00E-18	Identity = 30.27%
	3		gi|26249793|ref|NP_755833.1|	Escherichia coli CFT073	E-value = 2.00E-18	Identity = 29.73%
	4		gi|15803747|ref|NP_289781.1|	Escherichia coli O157:H7 EDL933	E-value = 5.00E-18	Identity = 29.73%
	5		gi|82545556|ref|YP_409503.1|	Shigella boydii Sb227	E-value = 5.00E-18	Identity = 29.73%
AB1823	1		gi|119356194|ref|YP_910838.1|	Chlorobium phaeobacteroides DSM 266	E-value = 2.00E-53	Identity = 35.84%
	2		gi|121997570|ref|YP_001002357.1|	Halorhodospira halophila SL1	E-value = 3.00E-43	Identity = 29.18%
	3		gi|157413793|ref|YP_001484659.1|	Prochlorococcus marinus str. MIT 9215	E-value = 3.00E-29	Identity = 30.49%
	4		gi|117923487|ref|YP_864104.1|	Magnetococcus sp. MC-1	E-value = 1.00E-24	Identity = 29.24%
	5		gi|20807153|ref|NP_622324.1|	Thermoanaerobacter tengcongensis MB4	E-value = 8.00E-07	Identity = 26.40%
AB1824	1		gi|67940520|ref|ZP_00532922.1|	Chlorobium phaeobacteroides BS1	E-value = 2.00E-25	Identity = 27.30%
	2		gi|152993528|ref|YP_001359249.1|	Sulfurovum sp. NBC37-1	E-value = 3.00E-20	Identity = 27.78%
	3		gi|149195184|ref|ZP_01872275.1|	Caminibacter mediatlanticus TB-2	E-value = 2.00E-17	Identity = 26.43%
	4		gi|78777895|ref|YP_394210.1|	Sulfuromonas denitrificans ATCC 33889	E-value = 9.00E-17	Identity = 25.84%
AB1825	1		gi|56708496|ref|YP_170392.1|	Francisella tularensis subsp. tularensis SCHU S4	E-value = 1.00E-130	Identity = 68.51%
	2		gi|157121752|gb|EDO65922.1|	Francisella tularensis subsp. holarctica FSC022	E-value = 3.00E-130	Identity = 68.51%
	3		gi|146282129|ref|YP_001172282.1|	Pseudomonas stutzeri A1501	E-value = 2.00E-113	Identity = 55.81%
	4		gi|71735150|ref|YP_275305.1|	Pseudomonas syringae pv. phaseolicola 1448A	E-value = 8.00E-110	Identity = 52.97%
	5		gi|66046450|ref|YP_236291.1|	Pseudomonas syringae pv. syringae B728a	E-value = 1.00E-109	Identity = 51.84%
AB1826	1		gi|149195143|ref|ZP_01872234.1|	Caminibacter mediatlanticus TB-2	E-value = 1.00E-155	Identity = 78.74%
	2		gi|152993139|ref|YP_001358860.1|	Sulfurovum sp. NBC37-1	E-value = 1.00E-143	Identity = 60.22%
	3		gi|45250013|gb|AAS55726.1|	Aneurinibacillus thermoaerophilus	E-value = 2.00E-131	Identity = 69.85%
	4		gi|126662747|ref|ZP_01733746.1|	Flavobacteria bacterium BAL38	E-value = 7.00E-131	Identity = 67.95%
	5		gi|86135155|ref|ZP_01053737.1|	Tenacibaculum sp. MED152	E-value = 7.00E-131	Identity = 68.66%
AB1827	1		gi|152993140|ref|YP_001358861.1|	Sulfurovum sp. NBC37-1	E-value = 4.00E-126	Identity = 73.70%
	2		gi|29725990|gb|AAO88922.1|	Vibrio cholerae	E-value = 9.00E-124	Identity = 72.57%
	3		gi|86147260|ref|ZP_01065575.1|	Vibrio sp. MED222	E-value = 1.00E-123	Identity = 72.92%
	4		gi|148976931|ref|ZP_01813586.1|	Vibrionales bacterium SWAT-3	E-value = 1.00E-123	Identity = 72.57%
	5		gi|126175092|ref|YP_001051241.1|	Shewanella baltica OS155	E-value = 2.00E-123	Identity = 74.13%
AB1829	1		gi|42523997|ref|NP_969377.1|	Bdellovibrio bacteriovorus HD100	E-value = 2.00E-86	Identity = 32.17%
	2		gi|27364269|ref|NP_759797.1|	Vibrio vulnificus CMCP6	E-value = 4.00E-53	Identity = 26.90%
	3		gi|37678475|ref|NP_933084.1|	Vibrio vulnificus YJ016	E-value = 2.00E-52	Identity = 27.09%
	4		gi|77978553|ref|ZP_00833980.1|	Yersinia intermedia ATCC 29909	E-value = 1.00E-51	Identity = 28.85%
	5		gi|77956456|ref|ZP_00820553.1|	Yersinia bercovieri ATCC 43970	E-value = 2.00E-51	Identity = 26.81%
AB1830	1	*dgkA*	gi|78776519|ref|YP_392834.1|	Sulfuromonas denitrificans ATCC 33889	E-value = 8.00E-22	Identity = 53.91%
	2		gi|154174822|ref|YP_001407409.1|	Campylobacter curvus 525.92	E-value = 2.00E-19	Identity = 47.83%
	3		gi|34557058|ref|NP_906873.1|	Wolinella succinogenes DSM 1740	E-value = 4.00E-16	Identity = 40.18%
	4		gi|148926402|ref|ZP_01810086.1|	Campylobacter jejuni subsp. jejuni CG8486	E-value = 2.00E-15	Identity = 46.43%
	5		gi|126174077|ref|YP_001050226.1|	Shewanella baltica OS155	E-value = 2.00E-15	Identity = 43.86%
AB1831	1		gi|52425503|ref|YP_088640.1|	Mannheimia succiniciproducens MBEL55E	E-value = 8.00E-13	Identity = 28.99%
	2		gi|68249995|ref|YP_249107.1|	Haemophilus influenzae 86-028NP	E-value = 2.00E-11	Identity = 26.77%
	3		gi|148826017|ref|YP_001290770.1|	Haemophilus influenzae PittEE	E-value = 2.00E-11	Identity = 26.77%
	4		gi|157165010|ref|YP_001467761.1|	Campylobacter concisus 13826	E-value = 2.00E-10	Identity = 30.00%

	5		gi|32029690|ref|ZP_00132673.1|	Haemophilus somnus 2336	E-value = 2.00E-10	Identity = 27.47%
AB1832	1	*waaM*	gi|34558202|ref|NP_908017.1|	Wolinella succinogenes DSM 1740	E-value = 1.00E-40	Identity = 34.80%
	2		gi|118475340|ref|YP_892541.1|	Campylobacter fetus subsp. fetus 82-40	E-value = 3.00E-37	Identity = 34.33%
	3		gi|78777873|ref|YP_394188.1|	Sulfuromonas denitrificans ATCC 33889	E-value = 1.00E-35	Identity = 34.40%
	4		gi|154174903|ref|YP_001407691.1|	Campylobacter curvus 525.92	E-value = 7.00E-33	Identity = 31.99%
	5		gi|157165600|ref|YP_001467312.1|	Campylobacter concisus 13826	E-value = 6.00E-31	Identity = 31.77%
AB1833	1	*waaC*	gi|78776788|ref|YP_393103.1|	Sulfuromonas denitrificans ATCC 33889	E-value = 3.00E-81	Identity = 54.08%
	2		gi|118474382|ref|YP_892540.1|	Campylobacter fetus subsp. fetus 82-40	E-value = 3.00E-59	Identity = 43.33%
	3		gi|57241555|ref|ZP_00369501.1|	Campylobacter lari RM2100	E-value = 4.00E-59	Identity = 42.82%
	4		gi|157164075|ref|YP_001467311.1|	Campylobacter concisus 13826	E-value = 7.00E-57	Identity = 41.49%
	5		gi|118744273|ref|ZP_01592268.1|	Geobacter lovleyi SZ	E-value = 2.00E-56	Identity = 37.65%
AB1834	1	*gppA*	gi|32266483|ref|NP_860515.1|	Helicobacter hepaticus ATCC 51449	E-value = 2.00E-119	Identity = 46.46%
	2		gi|152990157|ref|YP_001355879.1|	Nitratiruptor sp. SB155-2	E-value = 8.00E-119	Identity = 47.63%
	3		gi|149194897|ref|ZP_01871991.1|	Caminibacter mediatlanticus TB-2	E-value = 9.00E-114	Identity = 50.00%
	4		gi|109947129|ref|YP_664357.1|	Helicobacter acinonychis str. Sheeba	E-value = 3.00E-112	Identity = 45.21%
	5		gi|108562705|ref|YP_627021.1|	Helicobacter pylori HPAG1	E-value = 6.00E-112	Identity = 44.60%
AB1835	1	*fdxB*	gi|152990158|ref|YP_001355880.1|	Nitratiruptor sp. SB155-2	E-value = 1.00E-21	Identity = 78.31%
	2		gi|152993563|ref|YP_001359284.1|	Sulfurovum sp. NBC37-1	E-value = 3.00E-21	Identity = 79.52%
	3		gi|34558205|ref|NP_908020.1|	Wolinella succinogenes DSM 1740	E-value = 5.00E-21	Identity = 78.31%
	4		gi|109947128|ref|YP_664356.1|	Helicobacter acinonychis str. Sheeba	E-value = 2.00E-20	Identity = 73.17%
	5		gi|78776861|ref|YP_393176.1|	Sulfuromonas denitrificans ATCC 33889	E-value = 2.00E-20	Identity = 74.70%
AB1836	1		gi|32266738|ref|NP_860770.1|	Helicobacter hepaticus ATCC 51449	E-value = 1.00E-37	Identity = 40.24%
	2		gi|57168701|ref|ZP_00367833.1|	Campylobacter coli RM2228	E-value = 3.00E-36	Identity = 40.17%
	3		gi|86153692|ref|ZP_01071895.1|	Campylobacter jejuni subsp. jejuni HB93-13	E-value = 8.00E-36	Identity = 38.59%
	4		gi|15791516|ref|NP_281339.1|	Campylobacter jejuni subsp. jejuni NCTC 11168	E-value = 8.00E-36	Identity = 39.00%
	5		gi|121613474|ref|YP_999852.1|	Campylobacter jejuni subsp. jejuni 81-176	E-value = 9.00E-36	Identity = 38.59%
AB1837	1	*gltD*	gi|152990944|ref|YP_001356666.1|	Nitratiruptor sp. SB155-2	E-value = 2.00E-155	Identity = 57.61%
	2		gi|34557570|ref|NP_907385.1|	Wolinella succinogenes DSM 1740	E-value = 3.00E-151	Identity = 58.39%
	3		gi|78777373|ref|YP_393688.1|	Sulfuromonas denitrificans ATCC 33889	E-value = 8.00E-145	Identity = 53.48%
	4		gi|77919084|ref|YP_356899.1|	Pelobacter carbinolicus DSM 2380	E-value = 2.00E-131	Identity = 52.60%
	5		gi|95928931|ref|ZP_01311676.1|	Desulfuromonas acetoxidans DSM 684	E-value = 8.00E-131	Identity = 50.98%
AB1838	1	*gltB*	gi|152990945|ref|YP_001356667.1|	Nitratiruptor sp. SB155-2	E-value = 0	Identity = 65.74%
	2		gi|34557571|ref|NP_907386.1|	Wolinella succinogenes DSM 1740	E-value = 0	Identity = 65.51%
	3		gi|78777374|ref|YP_393689.1|	Sulfuromonas denitrificans ATCC 33889	E-value = 0	Identity = 64.41%
	4		gi|152992501|ref|YP_001358222.1|	Sulfurovum sp. NBC37-1	E-value = 0	Identity = 61.13%
	5		gi|77919083|ref|YP_356898.1|	Pelobacter carbinolicus DSM 2380	E-value = 0	Identity = 59.24%
AB1839	1		gi|78776943|ref|YP_393258.1|	Sulfuromonas denitrificans ATCC 33889	E-value = 7.00E-34	Identity = 43.41%
	2		gi|154149146|ref|YP_001405924.1|	Campylobacter hominis ATCC BAA-381	E-value = 4.00E-19	Identity = 29.63%
	3		gi|152990987|ref|YP_001356709.1|	Nitratiruptor sp. SB155-2	E-value = 3.00E-16	Identity = 27.85%
	4		gi|157165130|ref|YP_001467532.1|	Campylobacter concisus 13826	E-value = 3.00E-13	Identity = 27.64%
	5		gi|154174455|ref|YP_001407637.1|	Campylobacter curvus 525.92	E-value = 4.00E-13	Identity = 27.16%
AB1840	1		gi|152992962|ref|YP_001358683.1|	Sulfurovum sp. NBC37-1	E-value = 3.00E-66	Identity = 47.29%
	2		gi|78777889|ref|YP_394204.1|	Sulfuromonas denitrificans ATCC 33889	E-value = 1.00E-60	Identity = 47.29%
	3		gi|65317439|ref|ZP_00390398.1|	Bacillus anthracis str. A2012	E-value = 6.00E-45	Identity = 36.74%
	4		gi|30265476|ref|NP_847853.1|	Bacillus anthracis str. Ames	E-value = 1.00E-44	Identity = 36.74%
	5		gi|30023483|ref|NP_835114.1|	Bacillus cereus ATCC 14579	E-value = 1.00E-44	Identity = 37.12%
AB1841	1		gi|34557258|ref|NP_907073.1|	Wolinella succinogenes DSM 1740	E-value = 8.00E-73	Identity = 28.93%
	2		gi|57242486|ref|ZP_00370424.1|	Campylobacter upsaliensis RM3195	E-value = 2.00E-57	Identity = 27.55%
	3		gi|153952201|ref|YP_001398807.1|	Campylobacter jejuni subsp. doylei 269.97	E-value = 4.00E-57	Identity = 27.85%

	4		gi|57242629|ref|ZP_00370566.1|	Campylobacter upsaliensis RM3195	E-value = 4.00E-55	Identity = 27.72%
	5		gi|157415728|ref|YP_001482984.1|	Campylobacter jejuni subsp. jejuni 81116	E-value = 6.00E-55	Identity = 27.83%
AB1842	1		gi|34558144|ref|NP_907959.1|	Wolinella succinogenes DSM 1740	E-value = 3.00E-46	Identity = 30.69%
	2		gi|34557328|ref|NP_907143.1|	Wolinella succinogenes DSM 1740	E-value = 1.00E-42	Identity = 30.82%
	3		gi|34558241|ref|NP_908056.1|	Wolinella succinogenes DSM 1740	E-value = 3.00E-42	Identity = 32.01%
	4		gi|34558248|ref|NP_908063.1|	Wolinella succinogenes DSM 1740	E-value = 2.00E-41	Identity = 31.90%
AB1843	1			***** No matches found *****		
AB1844	1	*gspD*		***** No matches found *****		
AB1845	1		gi|57240784|ref|ZP_00368732.1|	Campylobacter lari RM2100	E-value = 9.00E-40	Identity = 51.11%
	2		gi|152993253|ref|YP_001358974.1|	Sulfurovum sp. NBC37-1	E-value = 2.00E-39	Identity = 50.00%
	3		gi|15791796|ref|NP_281619.1|	Campylobacter jejuni subsp. jejuni NCTC 11168	E-value = 7.00E-38	Identity = 50.00%
	4		gi|153952063|ref|YP_001398535.1|	Campylobacter jejuni subsp. doylei 269.97	E-value = 2.00E-37	Identity = 50.00%
	5		gi|86153740|ref|ZP_01071943.1|	Campylobacter jejuni subsp. jejuni HB93-13	E-value = 2.00E-37	Identity = 50.57%
AB1846	1	*tlyA*	gi|149193898|ref|ZP_01870996.1|	Caminibacter mediatlanticus TB-2	E-value = 3.00E-53	Identity = 55.80%
	2		gi|152990876|ref|YP_001356598.1|	Nitratiruptor sp. SB155-2	E-value = 5.00E-52	Identity = 52.59%
	3		gi|152992058|ref|YP_001357779.1|	Sulfurovum sp. NBC37-1	E-value = 7.00E-50	Identity = 46.78%
	4		gi|57241144|ref|ZP_00369091.1|	Campylobacter lari RM2100	E-value = 1.00E-49	Identity = 50.84%
	5		gi|57241998|ref|ZP_00369938.1|	Campylobacter upsaliensis RM3195	E-value = 8.00E-49	Identity = 48.89%
AB1847	1	*ribF*	gi|118475078|ref|YP_892268.1|	Campylobacter fetus subsp. fetus 82-40	E-value = 2.00E-70	Identity = 47.94%
	2		gi|157165726|ref|YP_001467144.1|	Campylobacter concisus 13826	E-value = 2.00E-66	Identity = 49.81%
	3		gi|154175170|ref|YP_001408018.1|	Campylobacter curvus 525.92	E-value = 1.00E-65	Identity = 49.07%
	4		gi|152990875|ref|YP_001356597.1|	Nitratiruptor sp. SB155-2	E-value = 1.00E-64	Identity = 47.67%
	5		gi|152992059|ref|YP_001357780.1|	Sulfurovum sp. NBC37-1	E-value = 3.00E-63	Identity = 48.66%
AB1848	1		gi|78776711|ref|YP_393026.1|	Sulfuromonas denitrificans ATCC 33889	E-value = 2.00E-73	Identity = 64.26%
	2		gi|152990873|ref|YP_001356595.1|	Nitratiruptor sp. SB155-2	E-value = 1.00E-72	Identity = 60.94%
	3		gi|149193761|ref|ZP_01870859.1|	Caminibacter mediatlanticus TB-2	E-value = 4.00E-72	Identity = 65.11%
	4		gi|34557004|ref|NP_906819.1|	Wolinella succinogenes DSM 1740	E-value = 3.00E-68	Identity = 56.12%
	5		gi|152992061|ref|YP_001357782.1|	Sulfurovum sp. NBC37-1	E-value = 2.00E-66	Identity = 57.02%
AB1849	1	*bcp*	gi|152991909|ref|YP_001357630.1|	Sulfurovum sp. NBC37-1	E-value = 1.00E-54	Identity = 62.28%
	2		gi|78777997|ref|YP_394312.1|	Sulfuromonas denitrificans ATCC 33889	E-value = 5.00E-50	Identity = 59.74%
	3		gi|152991280|ref|YP_001357002.1|	Nitratiruptor sp. SB155-2	E-value = 2.00E-46	Identity = 53.61%
	4		gi|126658834|ref|ZP_01729978.1|	Cyanothece sp. CCY0110	E-value = 6.00E-43	Identity = 51.20%
	5		gi|34556641|ref|NP_906456.1|	Wolinella succinogenes DSM 1740	E-value = 4.00E-42	Identity = 51.53%
AB1850	1			***** No matches found *****		
AB1851	1	*glcD*	gi|152993706|ref|YP_001359427.1|	Sulfurovum sp. NBC37-1	E-value = 0	Identity = 75.97%
	2		gi|118474967|ref|YP_891868.1|	Campylobacter fetus subsp. fetus 82-40	E-value = 0	Identity = 70.72%
	3		gi|78776573|ref|YP_392888.1|	Sulfuromonas denitrificans ATCC 33889	E-value = 0	Identity = 68.40%
	4		gi|34556706|ref|NP_906521.1|	Wolinella succinogenes DSM 1740	E-value = 0	Identity = 68.40%
	5		gi|154148549|ref|YP_001407138.1|	Campylobacter hominis ATCC BAA-381	E-value = 0	Identity = 67.46%
AB1852	1	*rbn*	gi|152993707|ref|YP_001359428.1|	Sulfurovum sp. NBC37-1	E-value = 4.00E-33	Identity = 39.18%
	2		gi|152990089|ref|YP_001355811.1|	Nitratiruptor sp. SB155-2	E-value = 6.00E-32	Identity = 38.19%
	3		gi|32267229|ref|NP_861261.1|	Helicobacter hepaticus ATCC 51449	E-value = 3.00E-29	Identity = 34.44%
	4		gi|78776574|ref|YP_392889.1|	Sulfuromonas denitrificans ATCC 33889	E-value = 1.00E-26	Identity = 34.80%
	5		gi|154174119|ref|YP_001408500.1|	Campylobacter curvus 525.92	E-value = 2.00E-26	Identity = 33.58%
AB1853	1			***** No matches found *****		
AB1854	1	*murA*	gi|157164419|ref|YP_001466853.1|	Campylobacter concisus 13826	E-value = 2.00E-156	Identity = 67.77%
	2		gi|154174374|ref|YP_001408257.1|	Campylobacter curvus 525.92	E-value = 2.00E-153	Identity = 67.54%
	3		gi|78776755|ref|YP_393070.1|	Sulfuromonas denitrificans ATCC 33889	E-value = 6.00E-151	Identity = 68.09%
	4		gi|152990702|ref|YP_001356424.1|	Nitratiruptor sp. SB155-2	E-value = 7.00E-151	Identity = 65.80%

	5		gi|152992781|ref|YP_001358502.1|	Sulfurovum sp. NBC37-1	E-value = 1.00E-148	Identity = 66.82%
AB1855	1		gi|152991109|ref|YP_001356831.1|	Nitratiruptor sp. SB155-2	E-value = 5.00E-78	Identity = 61.03%
	2		gi|152993504|ref|YP_001359225.1|	Sulfurovum sp. NBC37-1	E-value = 9.00E-75	Identity = 57.59%
	3		gi|78776601|ref|YP_392916.1|	Sulfuromonas denitrificans ATCC 33889	E-value = 9.00E-71	Identity = 54.30%
	4		gi|118475229|ref|YP_892484.1|	Campylobacter fetus subsp. fetus 82-40	E-value = 2.00E-68	Identity = 56.79%
	5		gi|57505515|ref|ZP_00371442.1|	Campylobacter upsaliensis RM3195	E-value = 1.00E-59	Identity = 48.12%
AB1856	1	*kdsA*	gi|154174222|ref|YP_001407735.1|	Campylobacter curvus 525.92	E-value = 1.00E-102	Identity = 66.29%
	2		gi|118474288|ref|YP_892483.1|	Campylobacter fetus subsp. fetus 82-40	E-value = 4.00E-101	Identity = 65.66%
	3		gi|157164076|ref|YP_001466231.1|	Campylobacter concisus 13826	E-value = 1.00E-100	Identity = 64.39%
	4		gi|154148818|ref|YP_001405676.1|	Campylobacter hominis ATCC BAA-381	E-value = 3.00E-98	Identity = 63.02%
	5		gi|78776602|ref|YP_392917.1|	Sulfuromonas denitrificans ATCC 33889	E-value = 2.00E-94	Identity = 61.07%
AB1857	1	*ribH*	gi|118474152|ref|YP_892480.1|	Campylobacter fetus subsp. fetus 82-40	E-value = 3.00E-63	Identity = 77.27%
	2		gi|157164346|ref|YP_001466234.1|	Campylobacter concisus 13826	E-value = 7.00E-63	Identity = 78.21%
	3		gi|154174152|ref|YP_001407738.1|	Campylobacter curvus 525.92	E-value = 8.00E-63	Identity = 79.35%
	4		gi|152991104|ref|YP_001356826.1|	Nitratiruptor sp. SB155-2	E-value = 2.00E-61	Identity = 75.50%
	5		gi|57168522|ref|ZP_00367655.1|	Campylobacter coli RM2228	E-value = 4.00E-60	Identity = 75.82%
AB1858	1	*nusB*	gi|152991103|ref|YP_001356825.1|	Nitratiruptor sp. SB155-2	E-value = 1.00E-35	Identity = 58.46%
	2		gi|152993501|ref|YP_001359222.1|	Sulfurovum sp. NBC37-1	E-value = 5.00E-33	Identity = 60.00%
	3		gi|34556732|ref|NP_906547.1|	Wolinella succinogenes DSM 1740	E-value = 1.00E-32	Identity = 53.91%
	4		gi|78776604|ref|YP_392919.1|	Sulfuromonas denitrificans ATCC 33889	E-value = 2.00E-32	Identity = 54.62%
	5		gi|15791749|ref|NP_281572.1|	Campylobacter jejuni subsp. jejuni NCTC 11168	E-value = 3.00E-31	Identity = 59.54%
AB1859	1	*pyrF*	gi|152992271|ref|YP_001357992.1|	Sulfurovum sp. NBC37-1	E-value = 5.00E-84	Identity = 68.42%
	2		gi|152990265|ref|YP_001355987.1|	Nitratiruptor sp. SB155-2	E-value = 4.00E-83	Identity = 66.67%
	3		gi|78777644|ref|YP_393959.1|	Sulfuromonas denitrificans ATCC 33889	E-value = 7.00E-80	Identity = 65.35%
	4		gi|154174269|ref|YP_001407740.1|	Campylobacter curvus 525.92	E-value = 5.00E-75	Identity = 60.09%
	5		gi|153951680|ref|YP_001398576.1|	Campylobacter jejuni subsp. doylei 269.97	E-value = 7.00E-74	Identity = 63.16%
AB1860	1		gi|78355492|ref|YP_386941.1|	Desulfovibrio desulfuricans G20	E-value = 3.00E-72	Identity = 42.66%
	2		gi|154174977|ref|YP_001408723.1|	Campylobacter curvus 525.92	E-value = 3.00E-70	Identity = 49.29%
	3		gi|67919809|ref|ZP_00513370.1|	Chlorobium limicola DSM 245	E-value = 3.00E-66	Identity = 43.15%
	4		gi|46580785|ref|YP_011593.1|	Desulfovibrio vulgaris subsp. vulgaris str. Hildenborough	E-value = 3.00E-61	Identity = 45.22%
	5		gi|53714511|ref|YP_100503.1|	Bacteroides fragilis YCH46	E-value = 4.00E-61	Identity = 42.32%
AB1861	1			***** No matches found *****		
AB1862	1	*feoA*	gi|67938802|ref|ZP_00531321.1|	Chlorobium phaeobacteroides BS1	E-value = 1.00E-09	Identity = 48.65%
	2		gi|91772791|ref|YP_565483.1|	Methanococcoides burtonii DSM 6242	E-value = 1.00E-09	Identity = 47.83%
	3		gi|34557762|ref|NP_907577.1|	Wolinella succinogenes DSM 1740	E-value = 1.00E-09	Identity = 55.17%
	4		gi|156744177|ref|YP_001434306.1|	Roseiflexus castenholzii DSM 13941	E-value = 4.00E-09	Identity = 45.83%
	5		gi|149922628|ref|ZP_01911056.1|	Plesiocystis pacifica SIR-1	E-value = 1.00E-08	Identity = 40.30%
AB1863	1	*feoB2*	gi|95931046|ref|ZP_01313774.1|	Desulfuromonas acetoxidans DSM 684	E-value = 0	Identity = 56.07%
	2		gi|146329115|ref|YP_001209067.1|	Dichelobacter nodosus VCS1703A	E-value = 0	Identity = 45.01%
	3		gi|116749920|ref|YP_846607.1|	Syntrophobacter fumaroxidans MPOB	E-value = 1.00E-137	Identity = 33.38%
	4		gi|77918492|ref|YP_356307.1|	Pelobacter carbinolicus DSM 2380	E-value = 1.00E-128	Identity = 34.52%
	5		gi|95928507|ref|ZP_01311254.1|	Desulfuromonas acetoxidans DSM 684	E-value = 2.00E-127	Identity = 32.42%
AB1864	1		gi|95931045|ref|ZP_01313773.1|	Desulfuromonas acetoxidans DSM 684	E-value = 2.00E-23	Identity = 63.64%
	2		gi|34557760|ref|NP_907575.1|	Wolinella succinogenes DSM 1740	E-value = 5.00E-20	Identity = 51.61%
	3		gi|146328963|ref|YP_001209064.1|	Dichelobacter nodosus VCS1703A	E-value = 5.00E-18	Identity = 45.45%
	4		gi|42527558|ref|NP_972656.1|	Treponema denticola ATCC 35405	E-value = 5.00E-16	Identity = 48.51%
	5		gi|26990504|ref|NP_745929.1|	Pseudomonas putida KT2440	E-value = 7.00E-13	Identity = 38.24%
AB1865	1		gi|34557011|ref|NP_906826.1|	Wolinella succinogenes DSM 1740	E-value = 1.00E-41	Identity = 56.69%
AB1866	1		gi|34557010|ref|NP_906825.1|	Wolinella succinogenes DSM 1740	E-value = 0	Identity = 62.50%

AB1867	1		gi|34557009|ref|NP_906824.1|	Wolinella succinogenes DSM 1740	E-value = 3.00E-16	Identity = 56.84%
AB1868	1		gi|150387979|ref|YP_001318028.1|	Alkaliphilus metalliredigens QYMF	E-value = 5.00E-172	Identity = 49.35%
	2		gi|52081840|ref|YP_080631.1|	Bacillus licheniformis ATCC 14580	E-value = 6.00E-172	Identity = 48.22%
	3		gi|152974343|ref|YP_001373860.1|	Bacillus cereus subsp. cytotoxis NVH 391-98	E-value = 5.00E-171	Identity = 47.41%
	4		gi|89893575|ref|YP_517062.1|	Desulfitobacterium hafniense Y51	E-value = 2.00E-170	Identity = 47.59%
	5		gi|30018782|ref|NP_830413.1|	Bacillus cereus ATCC 14579	E-value = 6.00E-170	Identity = 47.78%
AB1869	1		gi|34557496|ref|NP_907311.1|	Wolinella succinogenes DSM 1740	E-value = 1.00E-26	Identity = 56.31%
	2		gi|118443001|ref|YP_877281.1|	Clostridium novyi NT	E-value = 2.00E-26	Identity = 55.24%
	3		gi|154483372|ref|ZP_02025820.1|	Eubacterium ventriosum ATCC 27560	E-value = 5.00E-26	Identity = 58.25%
	4		gi|146295534|ref|YP_001179305.1|	Caldicellulosiruptor saccharolyticus DSM 8903	E-value = 3.00E-25	Identity = 56.19%
	5		gi|114844658|ref|ZP_01455100.1|	Thermoanaerobacter ethanolicus X514	E-value = 3.00E-25	Identity = 56.19%
AB1870	1		gi|34557008|ref|NP_906823.1|	Wolinella succinogenes DSM 1740	E-value = 0	Identity = 55.78%
	2		gi|154174913|ref|YP_001408304.1|	Campylobacter curvus 525.92	E-value = 1.00E-173	Identity = 47.28%
	3		gi|118475112|ref|YP_892048.1|	Campylobacter fetus subsp. fetus 82-40	E-value = 3.00E-165	Identity = 47.31%
	4		gi|154174559|ref|YP_001408303.1|	Campylobacter curvus 525.92	E-value = 5.00E-154	Identity = 45.68%
	5		gi|119899284|ref|YP_934497.1|	Azoarcus sp. BH72	E-value = 4.00E-128	Identity = 38.83%
AB1871	1			***** No matches found *****		
AB1872	1		gi|78777696|ref|YP_394011.1|	Sulfuromonas denitrificans ATCC 33889	E-value = 0	Identity = 42.36%
	2		gi|34556616|ref|NP_906431.1|	Wolinella succinogenes DSM 1740	E-value = 0	Identity = 41.12%
	3		gi|152992069|ref|YP_001357790.1|	Sulfurovum sp. NBC37-1	E-value = 0	Identity = 40.37%
	4		gi|152990772|ref|YP_001356494.1|	Nitratiruptor sp. SB155-2	E-value = 0	Identity = 38.33%
	5		gi|78776998|ref|YP_393313.1|	Sulfuromonas denitrificans ATCC 33889	E-value = 0	Identity = 37.42%
AB1873	1		gi|152992181|ref|YP_001357902.1|	Sulfurovum sp. NBC37-1	E-value = 3.00E-23	Identity = 31.56%
	2		gi|152990814|ref|YP_001356536.1|	Nitratiruptor sp. SB155-2	E-value = 1.00E-18	Identity = 29.77%
	3		gi|34556737|ref|NP_906552.1|	Wolinella succinogenes DSM 1740	E-value = 5.00E-18	Identity = 31.71%
	4		gi|149195288|ref|ZP_01872376.1|	Caminibacter mediatlanticus TB-2	E-value = 1.00E-16	Identity = 35.22%
	5		gi|78777454|ref|YP_393769.1|	Sulfuromonas denitrificans ATCC 33889	E-value = 9.00E-12	Identity = 30.68%
AB1874	1		gi|78778234|ref|YP_394549.1|	Sulfuromonas denitrificans ATCC 33889	E-value = 3.00E-30	Identity = 28.80%
	2		gi|78777451|ref|YP_393766.1|	Sulfuromonas denitrificans ATCC 33889	E-value = 6.00E-24	Identity = 27.37%
	3		gi|34558828|gb|AAQ75172.1|	Alvinella pompejana epibiont 7G3	E-value = 2.00E-22	Identity = 25.85%
	4		gi|152992187|ref|YP_001357908.1|	Sulfurovum sp. NBC37-1	E-value = 5.00E-21	Identity = 25.13%
	5		gi|149193919|ref|ZP_01871017.1|	Caminibacter mediatlanticus TB-2	E-value = 1.00E-19	Identity = 25.77%
AB1875	1	*cspA*	gi|149911304|ref|ZP_01899925.1|	Moritella sp. PE36	E-value = 3.00E-13	Identity = 62.12%
	2		gi|109900034|ref|YP_663289.1|	Pseudoalteromonas atlantica T6c	E-value = 4.00E-13	Identity = 63.49%
	3		gi|78777701|ref|YP_394016.1|	Sulfuromonas denitrificans ATCC 33889	E-value = 7.00E-13	Identity = 55.07%
	4		gi|152992263|ref|YP_001357984.1|	Sulfurovum sp. NBC37-1	E-value = 1.00E-12	Identity = 59.42%
	5		gi|119944855|ref|YP_942535.1|	Psychromonas ingrahamii 37	E-value = 3.00E-12	Identity = 58.46%
AB1876	1			***** No matches found *****		
AB1877	1		gi|34557780|ref|NP_907595.1|	Wolinella succinogenes DSM 1740	E-value = 1.00E-40	Identity = 42.01%
	2		gi|152993408|ref|YP_001359129.1|	Sulfurovum sp. NBC37-1	E-value = 3.00E-32	Identity = 37.12%
	3		gi|78778185|ref|YP_394500.1|	Sulfuromonas denitrificans ATCC 33889	E-value = 4.00E-29	Identity = 37.55%
	4		gi|118475245|ref|YP_891579.1|	Campylobacter fetus subsp. fetus 82-40	E-value = 8.00E-27	Identity = 32.73%
	5		gi|152993952|ref|YP_001359673.1|	Sulfurovum sp. NBC37-1	E-value = 6.00E-26	Identity = 33.48%
AB1878	1		gi|121542733|ref|ZP_01674450.1|	Candidatus Desulfococcus oleovorans Hxd3	E-value = 3.00E-14	Identity = 25.51%
AB1879	1			***** No matches found *****		
AB1880	1		gi|110803274|ref|YP_697644.1|	Clostridium perfringens SM101	E-value = 2.00E-11	Identity = 32.10%
	2		gi|10955835|ref|NP_053154.1|	Yersinia pestis	E-value = 2.00E-11	Identity = 29.11%
	3		gi|1808662|emb|CAA71788.1|	Pseudomonas alcaligenes	E-value = 5.00E-10	Identity = 25.56%
	4		gi|83816884|ref|YP_446960.1|	Salinibacter ruber DSM 13855	E-value = 1.00E-09	Identity = 25.00%

	5		gi|84180838|gb|ABC54843.1|	Escherichia coli	E-value = 5.00E-09	Identity = 25.58%
AB1881	1			***** No matches found *****		
AB1882	1			***** No matches found *****		
AB1883	1	*ahpC*	gi|152992346|ref|YP_001358067.1|	Sulfurovum sp. NBC37-1	E-value = 1.00E-91	Identity = 80.81%
	2		gi|78777972|ref|YP_394287.1|	Sulfuromonas denitrificans ATCC 33889	E-value = 6.00E-90	Identity = 78.28%
	3		gi|34558282|ref|NP_908097.1|	Wolinella succinogenes DSM 1740	E-value = 5.00E-88	Identity = 76.77%
	4		gi|57241472|ref|ZP_00369418.1|	Campylobacter lari RM2100	E-value = 5.00E-86	Identity = 76.26%
	5		gi|152990141|ref|YP_001355863.1|	Nitratiruptor sp. SB155-2	E-value = 5.00E-86	Identity = 75.38%
AB1884	1	*trxA1*	gi|152991292|ref|YP_001357014.1|	Nitratiruptor sp. SB155-2	E-value = 1.00E-36	Identity = 65.71%
	2		gi|57168680|ref|ZP_00367812.1|	Campylobacter coli RM2228	E-value = 2.00E-36	Identity = 65.71%
	3		gi|57241392|ref|ZP_00369338.1|	Campylobacter lari RM2100	E-value = 2.00E-36	Identity = 67.62%
	4		gi|153951052|ref|YP_001397405.1|	Campylobacter jejuni subsp. doylei 269.97	E-value = 3.00E-36	Identity = 64.76%
	5		gi|15791535|ref|NP_281358.1|	Campylobacter jejuni subsp. jejuni NCTC 11168	E-value = 4.00E-36	Identity = 64.76%
AB1885	1		gi|150391952|ref|YP_001322001.1|	Alkaliphilus metalliredigens QYMF	E-value = 4.00E-12	Identity = 45.65%
	2		gi|92112915|ref|YP_572843.1|	Chromohalobacter salexigens DSM 3043	E-value = 2.00E-10	Identity = 43.21%
	3		gi|126642945|ref|YP_001085929.1|	Acinetobacter baumannii ATCC 17978	E-value = 2.00E-09	Identity = 39.53%
	4		gi|50086136|ref|YP_047646.1|	Acinetobacter sp. ADP1	E-value = 4.00E-09	Identity = 39.02%
	5		gi|119501168|ref|XP_001267341.1|	Neosartorya fischeri NRRL 181	E-value = 2.00E-08	Identity = 32.63%
AB1886	1		gi|152991196|ref|YP_001356918.1|	Nitratiruptor sp. SB155-2	E-value = 2.00E-08	Identity = 33.87%
AB1887	1	*rpoC*	gi|152990018|ref|YP_001355740.1|	Nitratiruptor sp. SB155-2	E-value = 0	Identity = 74.18%
	2		gi|157164541|ref|YP_001466545.1|	Campylobacter concisus 13826	E-value = 0	Identity = 72.65%
	3		gi|118474969|ref|YP_892467.1|	Campylobacter fetus subsp. fetus 82-40	E-value = 0	Identity = 71.23%
	4		gi|152991728|ref|YP_001357449.1|	Sulfurovum sp. NBC37-1	E-value = 0	Identity = 70.31%
	5		gi|157414772|ref|YP_001482028.1|	Campylobacter jejuni subsp. jejuni 81116	E-value = 0	Identity = 70.62%
AB1888	1	*rpoB*	gi|152990017|ref|YP_001355739.1|	Nitratiruptor sp. SB155-2	E-value = 0	Identity = 72.21%
	2		gi|152991727|ref|YP_001357448.1|	Sulfurovum sp. NBC37-1	E-value = 0	Identity = 72.92%
	3		gi|57237532|ref|YP_178546.1|	Campylobacter jejuni subsp. jejuni RM1221	E-value = 0	Identity = 72.46%
	4		gi|121613040|ref|YP_001000185.1|	Campylobacter jejuni subsp. jejuni 81-176	E-value = 0	Identity = 72.46%
	5		gi|157414771|ref|YP_001482027.1|	Campylobacter jejuni subsp. jejuni 81116	E-value = 0	Identity = 72.53%
AB1889	1	*rplL*	gi|78776553|ref|YP_392868.1|	Sulfuromonas denitrificans ATCC 33889	E-value = 3.00E-25	Identity = 76.42%
	2		gi|57504728|ref|ZP_00370782.1|	Campylobacter coli RM2228	E-value = 6.00E-25	Identity = 76.80%
	3		gi|157165629|ref|YP_001466544.1|	Campylobacter concisus 13826	E-value = 6.00E-25	Identity = 72.00%
	4		gi|154174384|ref|YP_001408642.1|	Campylobacter curvus 525.92	E-value = 8.00E-25	Identity = 74.40%
	5		gi|32265861|ref|NP_859893.1|	Helicobacter hepaticus ATCC 51449	E-value = 2.00E-24	Identity = 67.46%
AB1890	1	*rplJ*	gi|32265862|ref|NP_859894.1|	Helicobacter hepaticus ATCC 51449	E-value = 3.00E-40	Identity = 51.57%
	2		gi|34556890|ref|NP_906705.1|	Wolinella succinogenes DSM 1740	E-value = 2.00E-38	Identity = 52.83%
	3		gi|118475150|ref|YP_892470.1|	Campylobacter fetus subsp. fetus 82-40	E-value = 6.00E-38	Identity = 53.42%
	4		gi|78776552|ref|YP_392867.1|	Sulfuromonas denitrificans ATCC 33889	E-value = 8.00E-38	Identity = 55.97%
	5		gi|152991725|ref|YP_001357446.1|	Sulfurovum sp. NBC37-1	E-value = 1.00E-37	Identity = 49.69%
AB1891	1	*rplA*	gi|152990014|ref|YP_001355736.1|	Nitratiruptor sp. SB155-2	E-value = 8.00E-81	Identity = 70.13%
	2		gi|57504726|ref|ZP_00370780.1|	Campylobacter coli RM2228	E-value = 4.00E-79	Identity = 68.67%
	3		gi|148925854|ref|ZP_01809541.1|	Campylobacter jejuni subsp. jejuni CG8486	E-value = 5.00E-79	Identity = 69.10%
	4		gi|15791839|ref|NP_281662.1|	Campylobacter jejuni subsp. jejuni NCTC 11168	E-value = 7.00E-79	Identity = 69.10%
	5		gi|57240974|ref|ZP_00368922.1|	Campylobacter lari RM2100	E-value = 3.00E-78	Identity = 68.67%
AB1892	1	*rplK*	gi|15791838|ref|NP_281661.1|	Campylobacter jejuni subsp. jejuni NCTC 11168	E-value = 5.00E-48	Identity = 73.91%
	2		gi|57242442|ref|ZP_00370380.1|	Campylobacter upsaliensis RM3195	E-value = 2.00E-47	Identity = 73.19%
	3		gi|57240973|ref|ZP_00368921.1|	Campylobacter lari RM2100	E-value = 3.00E-47	Identity = 73.91%
	4		gi|152991723|ref|YP_001357444.1|	Sulfurovum sp. NBC37-1	E-value = 5.00E-47	Identity = 70.29%
	5		gi|118474174|ref|YP_892472.1|	Campylobacter fetus subsp. fetus 82-40	E-value = 7.00E-47	Identity = 74.45%

AB1893	1	*nusG*	gi|118475193|ref|YP_892473.1|	Campylobacter fetus subsp. fetus 82-40	E-value = 2.00E-66	Identity = 71.59%
	2		gi|154175492|ref|YP_001408646.1|	Campylobacter curvus 525.92	E-value = 4.00E-64	Identity = 68.18%
	3		gi|157165039|ref|YP_001466540.1|	Campylobacter concisus 13826	E-value = 2.00E-62	Identity = 65.34%
	4		gi|57504724|ref|ZP_00370778.1|	Campylobacter coli RM2228	E-value = 2.00E-61	Identity = 66.67%
	5		gi|57240972|ref|ZP_00368920.1|	Campylobacter lari RM2100	E-value = 2.00E-61	Identity = 68.75%
AB1894	1	*secE*	gi|154148329|ref|YP_001407186.1|	Campylobacter hominis ATCC BAA-381	E-value = 4.00E-06	Identity = 59.32%
	2		gi|148925851|ref|ZP_01809538.1|	Campylobacter jejuni subsp. jejuni CG8486	E-value = 8.00E-06	Identity = 61.02%
	3		gi|15791836|ref|NP_281659.1|	Campylobacter jejuni subsp. jejuni NCTC 11168	E-value = 8.00E-06	Identity = 61.02%
	4		gi|57237526|ref|YP_178540.1|	Campylobacter jejuni subsp. jejuni RM1221	E-value = 8.00E-06	Identity = 61.02%
	5		gi|86151679|ref|ZP_01067198.1|	Campylobacter jejuni subsp. jejuni CF93-6	E-value = 8.00E-06	Identity = 61.02%
AB1895	1	*rpmG*	gi|57504722|ref|ZP_00370776.1|	Campylobacter coli RM2228	E-value = 8.00E-10	Identity = 76.92%
	2		gi|153952140|ref|YP_001398492.1|	Campylobacter jejuni subsp. doylei 269.97	E-value = 1.00E-09	Identity = 76.92%
	3		gi|15791835|ref|NP_281658.1|	Campylobacter jejuni subsp. jejuni NCTC 11168	E-value = 1.00E-09	Identity = 76.92%
	4		gi|57240991|ref|ZP_00368939.1|	Campylobacter lari RM2100	E-value = 1.00E-09	Identity = 76.92%
	5		gi|57242445|ref|ZP_00370383.1|	Campylobacter upsaliensis RM3195	E-value = 2.00E-09	Identity = 75.00%
AB1896	1	*tufA*	gi|37538296|sp|P42482|EFTU_WOLSU	Wolinella succinogenes DSM 1740	E-value = 0	Identity = 79.75%
	2		gi|152990009|ref|YP_001355731.1|	Nitratiruptor sp. SB155-2	E-value = 1.00E-180	Identity = 80.05%
	3		gi|118474729|ref|YP_892476.1|	Campylobacter fetus subsp. fetus 82-40	E-value = 2.00E-180	Identity = 80.60%
	4		gi|78776546|ref|YP_392861.1|	Sulfuromonas denitrificans ATCC 33889	E-value = 9.00E-180	Identity = 81.34%
	5		gi|154174158|ref|YP_001408648.1|	Campylobacter curvus 525.92	E-value = 1.00E-179	Identity = 80.10%
AB1897	1	*murD*	gi|34558322|ref|NP_908137.1|	Wolinella succinogenes DSM 1740	E-value = 1.00E-113	Identity = 50.90%
	2		gi|78777813|ref|YP_394128.1|	Sulfuromonas denitrificans ATCC 33889	E-value = 7.00E-106	Identity = 49.12%
	3		gi|57240799|ref|ZP_00368747.1|	Campylobacter lari RM2100	E-value = 7.00E-103	Identity = 48.49%
	4		gi|57168569|ref|ZP_00367702.1|	Campylobacter coli RM2228	E-value = 1.00E-102	Identity = 48.49%
	5		gi|154174445|ref|YP_001407648.1|	Campylobacter curvus 525.92	E-value = 1.00E-102	Identity = 49.10%
AB1898	1	*mraY*	gi|152990061|ref|YP_001355783.1|	Nitratiruptor sp. SB155-2	E-value = 2.00E-113	Identity = 62.99%
	2		gi|154174657|ref|YP_001407649.1|	Campylobacter curvus 525.92	E-value = 2.00E-109	Identity = 59.89%
	3		gi|78777812|ref|YP_394127.1|	Sulfuromonas denitrificans ATCC 33889	E-value = 7.00E-108	Identity = 62.71%
	4		gi|57240800|ref|ZP_00368748.1|	Campylobacter lari RM2100	E-value = 3.00E-107	Identity = 58.86%
	5		gi|118475561|ref|YP_892502.1|	Campylobacter fetus subsp. fetus 82-40	E-value = 2.00E-105	Identity = 60.45%
AB1899	1	*pgm*	gi|152993767|ref|YP_001359488.1|	Sulfurovum sp. NBC37-1	E-value = 0	Identity = 64.42%
	2		gi|34558324|ref|NP_908139.1|	Wolinella succinogenes DSM 1740	E-value = 2.00E-173	Identity = 59.63%
	3		gi|78777811|ref|YP_394126.1|	Sulfuromonas denitrificans ATCC 33889	E-value = 3.00E-173	Identity = 60.29%
	4		gi|32266668|ref|NP_860700.1|	Helicobacter hepaticus ATCC 51449	E-value = 2.00E-165	Identity = 58.81%
	5		gi|109947594|ref|YP_664822.1|	Helicobacter acinonychis str. Sheeba	E-value = 3.00E-163	Identity = 59.76%
AB1900	1		gi|78778087|ref|YP_394402.1|	Sulfuromonas denitrificans ATCC 33889	E-value = 3.00E-57	Identity = 54.15%
	2		gi|152991264|ref|YP_001356986.1|	Nitratiruptor sp. SB155-2	E-value = 8.00E-51	Identity = 51.98%
	3		gi|118591239|ref|ZP_01548638.1|	Stappia aggregata IAM 12614	E-value = 2.00E-31	Identity = 33.59%
	4		gi|118594982|ref|ZP_01552329.1|	Methylophilales bacterium HTCC2181	E-value = 1.00E-29	Identity = 35.74%
	5		gi|148323160|gb|EDK88410.1|	Fusobacterium nucleatum subsp. polymorphum ATCC 10953	E-value = 2.00E-29	Identity = 37.05%
AB1901	1	*pbpA*	gi|34558334|ref|NP_908149.1|	Wolinella succinogenes DSM 1740	E-value = 0	Identity = 55.28%
	2		gi|157165718|ref|YP_001467073.1|	Campylobacter concisus 13826	E-value = 0	Identity = 51.99%
	3		gi|118474121|ref|YP_892196.1|	Campylobacter fetus subsp. fetus 82-40	E-value = 0	Identity = 54.30%
	4		gi|154173954|ref|YP_001408549.1|	Campylobacter curvus 525.92	E-value = 0	Identity = 51.07%
	5		gi|152991263|ref|YP_001356985.1|	Nitratiruptor sp. SB155-2	E-value = 0	Identity = 51.75%
AB1902	1	*glnA*	gi|152991949|ref|YP_001357670.1|	Sulfurovum sp. NBC37-1	E-value = 0	Identity = 77.10%
	2		gi|118475736|ref|YP_892217.1|	Campylobacter fetus subsp. fetus 82-40	E-value = 0	Identity = 77.31%
	3		gi|78778083|ref|YP_394398.1|	Sulfuromonas denitrificans ATCC 33889	E-value = 0	Identity = 76.89%
	4		gi|157165609|ref|YP_001466999.1|	Campylobacter concisus 13826	E-value = 0	Identity = 72.90%

	5		gi|154147867|ref|YP_001407222.1|	Campylobacter hominis ATCC BAA-381	E-value = 0	Identity = 73.53%
AB1903	1	*hisJ*	gi|152991260|ref|YP_001356982.1|	Nitratiruptor sp. SB155-2	E-value = 4.00E-91	Identity = 61.03%
	2		gi|152991950|ref|YP_001357671.1|	Sulfurovum sp. NBC37-1	E-value = 2.00E-87	Identity = 60.69%
	3		gi|154174464|ref|YP_001408568.1|	Campylobacter curvus 525.92	E-value = 2.00E-81	Identity = 56.27%
	4		gi|154148198|ref|YP_001407037.1|	Campylobacter hominis ATCC BAA-381	E-value = 3.00E-79	Identity = 57.79%
	5		gi|78778060|ref|YP_394375.1|	Sulfuromonas denitrificans ATCC 33889	E-value = 5.00E-77	Identity = 55.13%
AB1904	1	*moaE*	gi|118475007|ref|YP_891397.1|	Campylobacter fetus subsp. fetus 82-40	E-value = 5.00E-52	Identity = 67.36%
	2		gi|78776455|ref|YP_392770.1|	Sulfuromonas denitrificans ATCC 33889	E-value = 6.00E-51	Identity = 66.67%
	3		gi|157165158|ref|YP_001467542.1|	Campylobacter concisus 13826	E-value = 6.00E-50	Identity = 65.73%
	4		gi|152991376|ref|YP_001357098.1|	Nitratiruptor sp. SB155-2	E-value = 4.00E-47	Identity = 62.76%
	5		gi|152991707|ref|YP_001357428.1|	Sulfurovum sp. NBC37-1	E-value = 7.00E-45	Identity = 58.74%
AB1905	1	*moaD*	gi|78776454|ref|YP_392769.1|	Sulfuromonas denitrificans ATCC 33889	E-value = 6.00E-20	Identity = 64.38%
	2		gi|34557565|ref|NP_907380.1|	Wolinella succinogenes DSM 1740	E-value = 3.00E-19	Identity = 61.64%
	3		gi|154148751|ref|YP_001405730.1|	Campylobacter hominis ATCC BAA-381	E-value = 8.00E-19	Identity = 60.27%
	4		gi|86149491|ref|ZP_01067722.1|	Campylobacter jejuni subsp. jejuni CF93-6	E-value = 1.00E-17	Identity = 61.64%
	5		gi|15792830|ref|NP_282653.1|	Campylobacter jejuni subsp. jejuni NCTC 11168	E-value = 2.00E-17	Identity = 61.64%
AB1906	1		gi|152991705|ref|YP_001357426.1|	Sulfurovum sp. NBC37-1	E-value = 1.00E-48	Identity = 49.53%
	2		gi|78776453|ref|YP_392768.1|	Sulfuromonas denitrificans ATCC 33889	E-value = 1.00E-44	Identity = 49.31%
	3		gi|152991378|ref|YP_001357100.1|	Nitratiruptor sp. SB155-2	E-value = 1.00E-42	Identity = 47.57%
	4		gi|34556686|ref|NP_906501.1|	Wolinella succinogenes DSM 1740	E-value = 6.00E-42	Identity = 41.90%
	5		gi|154147991|ref|YP_001405948.1|	Campylobacter hominis ATCC BAA-381	E-value = 8.00E-40	Identity = 46.58%
AB1907	1	*uppP*	gi|114776784|ref|ZP_01451827.1|	Mariprofundus ferrooxydans PV-1	E-value = 1.00E-72	Identity = 59.13%
	2		gi|77362182|ref|YP_341756.1|	Pseudoalteromonas haloplanktis TAC125	E-value = 3.00E-67	Identity = 57.87%
	3		gi|78486157|ref|YP_392082.1|	Thiomicrospira crunogena XCL-2	E-value = 2.00E-66	Identity = 56.08%
	4		gi|78776273|ref|YP_392588.1|	Sulfuromonas denitrificans ATCC 33889	E-value = 2.00E-66	Identity = 62.89%
	5		gi|51246236|ref|YP_066120.1|	Desulfotalea psychrophila LSv54	E-value = 3.00E-65	Identity = 58.33%
AB1908	1	*murG*	gi|86151053|ref|ZP_01069269.1|	Campylobacter jejuni subsp. jejuni 260.94	E-value = 3.00E-84	Identity = 50.91%
	2		gi|157415296|ref|YP_001482552.1|	Campylobacter jejuni subsp. jejuni 81116	E-value = 3.00E-84	Identity = 50.91%
	3		gi|86150455|ref|ZP_01068680.1|	Campylobacter jejuni subsp. jejuni CF93-6	E-value = 3.00E-84	Identity = 50.91%
	4		gi|15792366|ref|NP_282189.1|	Campylobacter jejuni subsp. jejuni NCTC 11168	E-value = 3.00E-84	Identity = 50.91%
	5		gi|88596600|ref|ZP_01099837.1|	Campylobacter jejuni subsp. jejuni 84-25	E-value = 4.00E-84	Identity = 50.91%
AB1909	1	*pbpB*	gi|154175193|ref|YP_001408313.1|	Campylobacter curvus 525.92	E-value = 1.00E-99	Identity = 54.81%
	2		gi|78778002|ref|YP_394317.1|	Sulfuromonas denitrificans ATCC 33889	E-value = 5.00E-99	Identity = 53.13%
	3		gi|34557982|ref|NP_907797.1|	Wolinella succinogenes DSM 1740	E-value = 4.00E-98	Identity = 51.36%
	4		gi|157164714|ref|YP_001466833.1|	Campylobacter concisus 13826	E-value = 5.00E-98	Identity = 52.48%
	5		gi|118475699|ref|YP_892019.1|	Campylobacter fetus subsp. fetus 82-40	E-value = 3.00E-97	Identity = 53.33%
AB1910	1	*ftsW*	gi|152991271|ref|YP_001356993.1|	Nitratiruptor sp. SB155-2	E-value = 2.00E-134	Identity = 43.59%
	2		gi|154174173|ref|YP_001408543.1|	Campylobacter curvus 525.92	E-value = 1.00E-126	Identity = 43.54%
	3		gi|34557978|ref|NP_907793.1|	Wolinella succinogenes DSM 1740	E-value = 3.00E-126	Identity = 43.99%
	4		gi|157164916|ref|YP_001467066.1|	Campylobacter concisus 13826	E-value = 4.00E-126	Identity = 44.44%
	5		gi|118475499|ref|YP_891878.1|	Campylobacter fetus subsp. fetus 82-40	E-value = 1.00E-121	Identity = 43.56%
AB1911	1	*ppi*	gi|78777657|ref|YP_393972.1|	Sulfuromonas denitrificans ATCC 33889	E-value = 5.00E-58	Identity = 68.10%
	2		gi|152990286|ref|YP_001356008.1|	Nitratiruptor sp. SB155-2	E-value = 2.00E-52	Identity = 61.35%
	3		gi|152992270|ref|YP_001357991.1|	Sulfurovum sp. NBC37-1	E-value = 4.00E-52	Identity = 57.89%
	4		gi|15612399|ref|NP_224052.1|	Helicobacter pylori J99	E-value = 2.00E-48	Identity = 59.87%
	5		gi|154148315|ref|YP_001406969.1|	Campylobacter hominis ATCC BAA-381	E-value = 1.00E-46	Identity = 56.79%
AB1912	1		gi|78776940|ref|YP_393255.1|	Sulfuromonas denitrificans ATCC 33889	E-value = 4.00E-16	Identity = 37.78%
	2		gi|152991321|ref|YP_001357043.1|	Nitratiruptor sp. SB155-2	E-value = 7.00E-09	Identity = 31.93%
	3		gi|78485296|ref|YP_391221.1|	Thiomicrospira crunogena XCL-2	E-value = 1.00E-06	Identity = 28.48%

AB1913	1	*clpA*	gi|152990129|ref|YP_001355851.1|	Nitratiruptor sp. SB155-2	E-value = 0	Identity = 58.13%
	2		gi|34556772|ref|NP_906587.1|	Wolinella succinogenes DSM 1740	E-value = 0	Identity = 56.68%
	3		gi|152993642|ref|YP_001359363.1|	Sulfurovum sp. NBC37-1	E-value = 0	Identity = 55.15%
	4		gi|82523954|emb|CAI78765.1|	uncultured epsilon proteobacterium	E-value = 0	Identity = 55.30%
	5		gi|78776658|ref|YP_392973.1|	Sulfuromonas denitrificans ATCC 33889	E-value = 0	Identity = 54.82%
AB1914	1	*clpS*	gi|152990128|ref|YP_001355850.1|	Nitratiruptor sp. SB155-2	E-value = 1.00E-24	Identity = 64.89%
	2		gi|82523955|emb|CAI78766.1|	uncultured epsilon proteobacterium	E-value = 5.00E-24	Identity = 58.59%
	3		gi|34556771|ref|NP_906586.1|	Wolinella succinogenes DSM 1740	E-value = 2.00E-22	Identity = 52.08%
	4		gi|78776659|ref|YP_392974.1|	Sulfuromonas denitrificans ATCC 33889	E-value = 3.00E-22	Identity = 55.56%
	5		gi|121540453|ref|ZP_01672214.1|	Candidatus Desulfococcus oleovorans Hxd3	E-value = 8.00E-22	Identity = 56.47%
AB1915	1	*bioD*	gi|152993644|ref|YP_001359365.1|	Sulfurovum sp. NBC37-1	E-value = 3.00E-51	Identity = 52.94%
	2		gi|152990127|ref|YP_001355849.1|	Nitratiruptor sp. SB155-2	E-value = 8.00E-50	Identity = 50.50%
	3		gi|82523957|emb|CAI78768.1|	uncultured epsilon proteobacterium	E-value = 5.00E-44	Identity = 47.64%
	4		gi|78776661|ref|YP_392976.1|	Sulfuromonas denitrificans ATCC 33889	E-value = 2.00E-41	Identity = 46.60%
	5		gi|149194830|ref|ZP_01871924.1|	Caminibacter mediatlanticus TB-2	E-value = 4.00E-36	Identity = 47.64%
AB1916	1			***** No matches found *****		
AB1917	1		gi|118594803|ref|ZP_01552150.1|	Methylophilales bacterium HTCC2181	E-value = 2.00E-129	Identity = 59.04%
	2		gi|17981051|gb|AAL50820.1|AF453714_2	Rhodococcus erythropolis	E-value = 5.00E-10	Identity = 26.01%
	3		gi|134294069|ref|YP_001117805.1|	Burkholderia vietnamiensis G4	E-value = 1.00E-09	Identity = 26.21%
	4		gi|118473647|ref|YP_890852.1|	Mycobacterium smegmatis str. MC2 155	E-value = 1.00E-08	Identity = 25.95%
	5		gi|118467078|ref|YP_879629.1|	Mycobacterium avium 104	E-value = 2.00E-08	Identity = 26.26%
AB1918	1			***** No matches found *****		
AB1919	1		gi|156740917|ref|YP_001431046.1|	Roseiflexus castenholzii DSM 13941	E-value = 2.00E-105	Identity = 52.86%
	2		gi|148658261|ref|YP_001278466.1|	Roseiflexus sp. RS-1	E-value = 1.00E-102	Identity = 52.00%
	3		gi|118047898|ref|ZP_01516523.1|	Chloroflexus aggregans DSM 9485	E-value = 2.00E-102	Identity = 50.85%
	4		gi|76260205|ref|ZP_00767844.1|	Chloroflexus aurantiacus J-10-fl	E-value = 2.00E-100	Identity = 51.14%
	5		gi|83592537|ref|YP_426289.1|	Rhodospirillum rubrum ATCC 11170	E-value = 1.00E-99	Identity = 51.14%
AB1920	1		gi|116327769|ref|YP_797489.1|	Leptospira borgpetersenii serovar Hardjo-bovis L550	E-value = 1.00E-129	Identity = 68.40%
	2		gi|24215541|ref|NP_713022.1|	Leptospira interrogans serovar Lai str. 56601	E-value = 6.00E-129	Identity = 67.89%
	3		gi|45657078|ref|YP_001164.1|	Leptospira interrogans serovar Copenhageni str. Fiocruz L1-130	E-value = 7.00E-129	Identity = 67.89%
	4		gi|118594813|ref|ZP_01552160.1|	Methylophilales bacterium HTCC2181	E-value = 9.00E-125	Identity = 64.11%
	5		gi|73537979|ref|YP_298346.1|	Ralstonia eutropha JMP134	E-value = 8.00E-120	Identity = 62.46%
AB1921	1	*fumA*	gi|67156353|ref|ZP_00417946.1|	Azotobacter vinelandii AvOP	E-value = 0	Identity = 64.98%
	2		gi|89093995|ref|ZP_01166939.1|	Oceanospirillum sp. MED92	E-value = 0	Identity = 64.44%
	3		gi|152996356|ref|YP_001341191.1|	Marinomonas sp. MWYL1	E-value = 0	Identity = 64.50%
	4		gi|120554952|ref|YP_959303.1|	Marinobacter aquaeolei VT8	E-value = 0	Identity = 62.90%
	5		gi|15838453|ref|NP_299141.1|	Xylella fastidiosa 9a5c	E-value = 0	Identity = 61.74%
AB1922	1		gi|90412611|ref|ZP_01220613.1|	Photobacterium profundum 3TCK	E-value = 3.00E-63	Identity = 47.01%
	2		gi|152993464|ref|YP_001359185.1|	Sulfurovum sp. NBC37-1	E-value = 7.00E-63	Identity = 47.54%
	3		gi|78777705|ref|YP_394020.1|	Sulfuromonas denitrificans ATCC 33889	E-value = 2.00E-62	Identity = 45.59%
	4		gi|126208838|ref|YP_001054063.1|	Actinobacillus pleuropneumoniae L20	E-value = 3.00E-62	Identity = 45.79%
	5		gi|90577729|ref|ZP_01233540.1|	Vibrio angustum S14	E-value = 7.00E-62	Identity = 46.51%
AB1923	1		gi|149193826|ref|ZP_01870924.1|	Caminibacter mediatlanticus TB-2	E-value = 2.00E-38	Identity = 26.49%
AB1924	1	*arsB*	gi|78776514|ref|YP_392829.1|	Sulfuromonas denitrificans ATCC 33889	E-value = 5.00E-139	Identity = 73.15%
	2		gi|152992971|ref|YP_001358692.1|	Sulfurovum sp. NBC37-1	E-value = 8.00E-128	Identity = 66.59%
	3		gi|152989779|ref|YP_001355501.1|	Nitratiruptor sp. SB155-2	E-value = 2.00E-121	Identity = 65.36%
	4		gi|34557153|ref|NP_906968.1|	Wolinella succinogenes DSM 1740	E-value = 5.00E-116	Identity = 60.82%
	5		gi|118474803|ref|YP_892784.1|	Campylobacter fetus subsp. fetus 82-40	E-value = 8.00E-116	Identity = 62.50%
AB1925	1	*arsR*	gi|78776516|ref|YP_392831.1|	Sulfuromonas denitrificans ATCC 33889	E-value = 5.00E-35	Identity = 69.61%

	2		gi|152992970|ref|YP_001358691.1|	Sulfurovum sp. NBC37-1	E-value = 2.00E-30	Identity = 62.24%
	3		gi|57241136|ref|ZP_00369083.1|	Campylobacter lari RM2100	E-value = 2.00E-22	Identity = 51.96%
	4		gi|152989783|ref|YP_001355505.1|	Nitratiruptor sp. SB155-2	E-value = 2.00E-20	Identity = 49.02%
	5		gi|34557167|ref|NP_906982.1|	Wolinella succinogenes DSM 1740	E-value = 3.00E-20	Identity = 47.42%
AB1926	1		gi|152993005|ref|YP_001358726.1|	Sulfurovum sp. NBC37-1	E-value = 1.00E-17	Identity = 67.11%
	2		gi|34557156|ref|NP_906971.1|	Wolinella succinogenes DSM 1740	E-value = 8.00E-17	Identity = 74.65%
	3		gi|152989781|ref|YP_001355503.1|	Nitratiruptor sp. SB155-2	E-value = 2.00E-14	Identity = 62.67%
	4		gi|86157455|ref|YP_464240.1|	Anaeromyxobacter dehalogenans 2CP-C	E-value = 2.00E-13	Identity = 61.33%
	5		gi|110600603|ref|ZP_01388820.1|	Geobacter sp. FRC-32	E-value = 6.00E-13	Identity = 62.67%
AB1927	1		gi|152993006|ref|YP_001358727.1|	Sulfurovum sp. NBC37-1	E-value = 4.00E-94	Identity = 62.16%
	2		gi|152989780|ref|YP_001355502.1|	Nitratiruptor sp. SB155-2	E-value = 3.00E-92	Identity = 58.92%
	3		gi|118746248|ref|ZP_01594197.1|	Geobacter lovleyi SZ	E-value = 7.00E-80	Identity = 51.78%
	4		gi|148262521|ref|YP_001229227.1|	Geobacter uraniumreducens Rf4	E-value = 2.00E-78	Identity = 52.37%
	5		gi|39998046|ref|NP_953997.1|	Geobacter sulfurreducens PCA	E-value = 1.00E-76	Identity = 53.72%
AB1928	1	*arsC*	gi|34557154|ref|NP_906969.1|	Wolinella succinogenes DSM 1740	E-value = 4.00E-43	Identity = 63.64%
	2		gi|152992969|ref|YP_001358690.1|	Sulfurovum sp. NBC37-1	E-value = 5.00E-42	Identity = 72.44%
	3		gi|152989782|ref|YP_001355504.1|	Nitratiruptor sp. SB155-2	E-value = 2.00E-39	Identity = 62.20%
	4		gi|78776515|ref|YP_392830.1|	Sulfuromonas denitrificans ATCC 33889	E-value = 4.00E-38	Identity = 64.29%
	5		gi|149194612|ref|ZP_01871708.1|	Caminibacter mediatlanticus TB-2	E-value = 9.00E-37	Identity = 61.07%
AB1929	1		gi|152991488|ref|YP_001357210.1|	Nitratiruptor sp. SB155-2	E-value = 2.00E-36	Identity = 43.72%
	2		gi|149194880|ref|ZP_01871974.1|	Caminibacter mediatlanticus TB-2	E-value = 8.00E-30	Identity = 44.72%
	3		gi|152993061|ref|YP_001358782.1|	Sulfurovum sp. NBC37-1	E-value = 9.00E-29	Identity = 37.43%
	4		gi|87119381|ref|ZP_01075279.1|	Marinomonas sp. MED121	E-value = 3.00E-22	Identity = 32.77%
	5		gi|32267306|ref|NP_861338.1|	Helicobacter hepaticus ATCC 51449	E-value = 4.00E-22	Identity = 36.07%
AB1930	1		gi|152993295|ref|YP_001359016.1|	Sulfurovum sp. NBC37-1	E-value = 4.00E-85	Identity = 48.81%
	2		gi|78777568|ref|YP_393883.1|	Sulfuromonas denitrificans ATCC 33889	E-value = 4.00E-77	Identity = 45.80%
	3		gi|57168597|ref|ZP_00367730.1|	Campylobacter coli RM2228	E-value = 4.00E-75	Identity = 42.08%
	4		gi|15791825|ref|NP_281648.1|	Campylobacter jejuni subsp. jejuni NCTC 11168	E-value = 6.00E-75	Identity = 42.60%
	5		gi|148925840|ref|ZP_01809527.1|	Campylobacter jejuni subsp. jejuni CG8486	E-value = 8.00E-75	Identity = 42.60%
AB1931	1	*flhA*	gi|78776923|ref|YP_393238.1|	Sulfuromonas denitrificans ATCC 33889	E-value = 0	Identity = 52.21%
	2		gi|149193762|ref|ZP_01870860.1|	Caminibacter mediatlanticus TB-2	E-value = 8.00E-173	Identity = 52.03%
	3		gi|154174693|ref|YP_001408026.1|	Campylobacter curvus 525.92	E-value = 3.00E-172	Identity = 49.72%
	4		gi|57240352|ref|ZP_00368301.1|	Campylobacter lari RM2100	E-value = 6.00E-171	Identity = 50.37%
	5		gi|153951195|ref|YP_001398127.1|	Campylobacter jejuni subsp. doylei 269.97	E-value = 4.00E-170	Identity = 49.79%
AB1932	1	*flgL*		***** No matches found *****		
AB1933	1	*fliI*	gi|149194683|ref|ZP_01871778.1|	Caminibacter mediatlanticus TB-2	E-value = 1.00E-141	Identity = 60.28%
	2		gi|78778279|ref|YP_394594.1|	Sulfuromonas denitrificans ATCC 33889	E-value = 1.00E-141	Identity = 60.19%
	3		gi|34558482|ref|NP_908297.1|	Wolinella succinogenes DSM 1740	E-value = 9.00E-141	Identity = 60.71%
	4		gi|154174001|ref|YP_001407433.1|	Campylobacter curvus 525.92	E-value = 1.00E-135	Identity = 59.35%
	5		gi|157163914|ref|YP_001465995.1|	Campylobacter concisus 13826	E-value = 5.00E-135	Identity = 59.76%
AB1934	1			***** No matches found *****		
AB1935	1	*flhB*	gi|149194192|ref|ZP_01871290.1|	Caminibacter mediatlanticus TB-2	E-value = 1.00E-76	Identity = 47.56%
	2		gi|86151337|ref|ZP_01069552.1|	Campylobacter jejuni subsp. jejuni 260.94	E-value = 3.00E-75	Identity = 45.71%
	3		gi|86153620|ref|ZP_01071823.1|	Campylobacter jejuni subsp. jejuni HB93-13	E-value = 8.00E-75	Identity = 45.43%
	4		gi|148926430|ref|ZP_01810113.1|	Campylobacter jejuni subsp. jejuni CG8486	E-value = 9.00E-75	Identity = 45.43%
	5		gi|15791703|ref|NP_281526.1|	Campylobacter jejuni subsp. jejuni NCTC 11168	E-value = 9.00E-75	Identity = 45.43%
AB1936	1	*fliR*	gi|57240502|ref|ZP_00368451.1|	Campylobacter lari RM2100	E-value = 2.00E-18	Identity = 28.23%
	2		gi|153951433|ref|YP_001397725.1|	Campylobacter jejuni subsp. doylei 269.97	E-value = 1.00E-17	Identity = 28.00%
	3		gi|57238051|ref|YP_179300.1|	Campylobacter jejuni subsp. jejuni RM1221	E-value = 2.00E-17	Identity = 27.13%

	4		gi|86152607|ref|ZP_01070812.1|	Campylobacter jejuni subsp. jejuni HB93-13	E-value = 3.00E-17	Identity = 26.72%
	5		gi|15792503|ref|NP_282326.1|	Campylobacter jejuni subsp. jejuni NCTC 11168	E-value = 5.00E-17	Identity = 26.72%
AB1937	1			***** No matches found *****		
AB1938	1	*flgC*	gi|149195179|ref|ZP_01872270.1|	Caminibacter mediatlanticus TB-2	E-value = 2.00E-32	Identity = 46.67%
	2		gi|34557980|ref|NP_907795.1|	Wolinella succinogenes DSM 1740	E-value = 8.00E-31	Identity = 42.68%
	3		gi|118474843|ref|YP_891880.1|	Campylobacter fetus subsp. fetus 82-40	E-value = 1.00E-30	Identity = 45.68%
	4		gi|157165285|ref|YP_001467064.1|	Campylobacter concisus 13826	E-value = 2.00E-29	Identity = 42.94%
	5		gi|32266907|ref|NP_860939.1|	Helicobacter hepaticus ATCC 51449	E-value = 3.00E-29	Identity = 42.59%
AB1939	1	*fliE*	gi|153951288|ref|YP_001398446.1|	Campylobacter jejuni subsp. doylei 269.97	E-value = 2.00E-08	Identity = 39.39%
	2		gi|15791887|ref|NP_281710.1|	Campylobacter jejuni subsp. jejuni NCTC 11168	E-value = 2.00E-08	Identity = 40.40%
	3		gi|57242335|ref|ZP_00370274.1|	Campylobacter upsaliensis RM3195	E-value = 2.00E-08	Identity = 40.40%
	4		gi|91202524|emb|CAJ72163.1|	Candidatus Kuenenia stuttgartiensis	E-value = 2.00E-08	Identity = 34.74%
	5		gi|57238274|ref|YP_178645.1|	Campylobacter jejuni subsp. jejuni RM1221	E-value = 3.00E-08	Identity = 39.39%
AB1940	1			***** No matches found *****		
AB1941	1		gi|152990182|ref|YP_001355904.1|	Nitratiruptor sp. SB155-2	E-value = 2.00E-33	Identity = 36.10%
	2		gi|34557957|ref|NP_907772.1|	Wolinella succinogenes DSM 1740	E-value = 1.00E-32	Identity = 31.56%
	3		gi|154174453|ref|YP_001408772.1|	Campylobacter curvus 525.92	E-value = 2.00E-32	Identity = 31.06%
	4		gi|149194330|ref|ZP_01871427.1|	Caminibacter mediatlanticus TB-2	E-value = 4.00E-32	Identity = 30.80%
	5		gi|118474588|ref|YP_892643.1|	Campylobacter fetus subsp. fetus 82-40	E-value = 4.00E-32	Identity = 33.33%
AB1942	1	*flhF*	gi|149194329|ref|ZP_01871426.1|	Caminibacter mediatlanticus TB-2	E-value = 2.00E-73	Identity = 42.05%
	2		gi|78776902|ref|YP_393217.1|	Sulfuromonas denitrificans ATCC 33889	E-value = 4.00E-68	Identity = 37.92%
	3		gi|118475040|ref|YP_892642.1|	Campylobacter fetus subsp. fetus 82-40	E-value = 8.00E-66	Identity = 37.47%
	4		gi|15611457|ref|NP_223108.1|	Helicobacter pylori J99	E-value = 6.00E-55	Identity = 36.81%
	5		gi|108562837|ref|YP_627153.1|	Helicobacter pylori HPAG1	E-value = 2.00E-54	Identity = 36.81%
AB1943	1			***** No matches found *****		
AB1944	1			***** No matches found *****		
AB1945	1			***** No matches found *****		
AB1946	1	*fliY*	gi|34557953|ref|NP_907768.1|	Wolinella succinogenes DSM 1740	E-value = 2.00E-23	Identity = 33.18%
	2		gi|149194334|ref|ZP_01871431.1|	Caminibacter mediatlanticus TB-2	E-value = 2.00E-22	Identity = 32.25%
	3		gi|32266647|ref|NP_860679.1|	Helicobacter hepaticus ATCC 51449	E-value = 3.00E-22	Identity = 35.27%
	4		gi|15611462|ref|NP_223113.1|	Helicobacter pylori J99	E-value = 1.00E-18	Identity = 28.78%
	5		gi|154175006|ref|YP_001408768.1|	Campylobacter curvus 525.92	E-value = 4.00E-18	Identity = 29.82%
AB1947	1	*flaG*		***** No matches found *****		
AB1948	1			***** No matches found *****		
AB1949	1			***** No matches found *****		
AB1950	1	*flgE1*		***** No matches found *****		
AB1951	1	*flgE2*	gi|152990377|ref|YP_001356099.1|	Nitratiruptor sp. SB155-2	E-value = 2.00E-17	Identity = 26.12%
AB1952	1	*flgD*	gi|149195337|ref|ZP_01872424.1|	Caminibacter mediatlanticus TB-2	E-value = 5.00E-08	Identity = 30.49%
AB1953	1	*fliN*	gi|157164107|ref|YP_001466298.1|	Campylobacter concisus 13826	E-value = 9.00E-12	Identity = 40.23%
	2		gi|154174584|ref|YP_001408856.1|	Campylobacter curvus 525.92	E-value = 9.00E-12	Identity = 41.38%
	3		gi|118475399|ref|YP_891558.1|	Campylobacter fetus subsp. fetus 82-40	E-value = 1.00E-11	Identity = 43.53%
	4		gi|78777364|ref|YP_393679.1|	Sulfuromonas denitrificans ATCC 33889	E-value = 3.00E-11	Identity = 42.86%
	5		gi|149194013|ref|ZP_01871111.1|	Caminibacter mediatlanticus TB-2	E-value = 5.00E-11	Identity = 39.53%
AB1954	1	*fliH*	gi|109947512|ref|YP_664740.1|	Helicobacter acinonychis str. Sheeba	E-value = 1.00E-06	Identity = 26.78%
AB1955	1	*fliG*	gi|149194459|ref|ZP_01871556.1|	Caminibacter mediatlanticus TB-2	E-value = 6.00E-64	Identity = 43.75%
	2		gi|34558285|ref|NP_908100.1|	Wolinella succinogenes DSM 1740	E-value = 3.00E-63	Identity = 43.26%
	3		gi|82523971|emb|CAI78782.1|	uncultured epsilon proteobacterium	E-value = 6.00E-63	Identity = 43.79%
	4		gi|157164732|ref|YP_001466463.1|	Campylobacter concisus 13826	E-value = 1.00E-61	Identity = 43.65%
	5		gi|154175354|ref|YP_001408874.1|	Campylobacter curvus 525.92	E-value = 1.00E-61	Identity = 43.26%

AB1956	1	*fliF*	gi|149194458|ref|ZP_01871555.1|	Caminibacter mediatlanticus TB-2	E-value = 2.00E-64	Identity = 34.31%
	2		gi|157165014|ref|YP_001466462.1|	Campylobacter concisus 13826	E-value = 1.00E-59	Identity = 31.95%
	3		gi|34558286|ref|NP_908101.1|	Wolinella succinogenes DSM 1740	E-value = 3.00E-57	Identity = 30.07%
	4		gi|108562771|ref|YP_627087.1|	Helicobacter pylori HPAG1	E-value = 5.00E-57	Identity = 30.98%
	5		gi|154174065|ref|YP_001408875.1|	Campylobacter curvus 525.92	E-value = 6.00E-57	Identity = 31.28%
AB1957	1	*flgB*	gi|78776566|ref|YP_392881.1|	Sulfuromonas denitrificans ATCC 33889	E-value = 3.00E-13	Identity = 38.10%
	2		gi|157164065|ref|YP_001467063.1|	Campylobacter concisus 13826	E-value = 5.00E-13	Identity = 34.75%
	3		gi|154174839|ref|YP_001408540.1|	Campylobacter curvus 525.92	E-value = 9.00E-13	Identity = 38.71%
	4		gi|149195180|ref|ZP_01872271.1|	Caminibacter mediatlanticus TB-2	E-value = 8.00E-12	Identity = 35.29%
	5		gi|57242333|ref|ZP_00370272.1|	Campylobacter upsaliensis RM3195	E-value = 2.00E-11	Identity = 34.68%
AB1958	1	*flgG1*	gi|149194279|ref|ZP_01871376.1|	Caminibacter mediatlanticus TB-2	E-value = 2.00E-20	Identity = 34.75%
	2		gi|153952418|ref|YP_001398358.1|	Campylobacter jejuni subsp. doylei 269.97	E-value = 6.00E-20	Identity = 30.26%
	3		gi|86151576|ref|ZP_01069790.1|	Campylobacter jejuni subsp. jejuni 260.94	E-value = 1.00E-19	Identity = 29.89%
	4		gi|121612923|ref|YP_001000393.1|	Campylobacter jejuni subsp. jejuni 81-176	E-value = 2.00E-19	Identity = 30.00%
	5		gi|86153287|ref|ZP_01071491.1|	Campylobacter jejuni subsp. jejuni HB93-13	E-value = 2.00E-19	Identity = 29.52%
AB1960	1	*cheY2*	gi|154174704|ref|YP_001407722.1|	Campylobacter curvus 525.92	E-value = 8.00E-35	Identity = 66.39%
	2		gi|157164984|ref|YP_001467285.1|	Campylobacter concisus 13826	E-value = 7.00E-34	Identity = 66.39%
	3		gi|15792443|ref|NP_282266.1|	Campylobacter jejuni subsp. jejuni NCTC 11168	E-value = 2.00E-33	Identity = 65.57%
	4		gi|109947708|ref|YP_664936.1|	Helicobacter acinonychis str. Sheeba	E-value = 3.00E-33	Identity = 65.57%
	5		gi|15611426|ref|NP_223077.1|	Helicobacter pylori J99	E-value = 6.00E-33	Identity = 65.57%
AB1961	1	*flgG2*	gi|157165544|ref|YP_001467096.1|	Campylobacter concisus 13826	E-value = 1.00E-73	Identity = 60.15%
	2		gi|154174905|ref|YP_001408076.1|	Campylobacter curvus 525.92	E-value = 1.00E-72	Identity = 59.00%
	3		gi|34558104|ref|NP_907919.1|	Wolinella succinogenes DSM 1740	E-value = 4.00E-71	Identity = 58.62%
	4		gi|32266580|ref|NP_860612.1|	Helicobacter hepaticus ATCC 51449	E-value = 3.00E-70	Identity = 57.85%
	5		gi|109946869|ref|YP_664097.1|	Helicobacter acinonychis str. Sheeba	E-value = 2.00E-69	Identity = 56.32%
AB1962	1			***** No matches found *****		
AB1963	1			***** No matches found *****		
AB1964	1	*rimM*	gi|78776654|ref|YP_392969.1|	Sulfuromonas denitrificans ATCC 33889	E-value = 6.00E-38	Identity = 49.44%
	2		gi|152991966|ref|YP_001357687.1|	Sulfurovum sp. NBC37-1	E-value = 8.00E-36	Identity = 44.89%
	3		gi|152991244|ref|YP_001356966.1|	Nitratiruptor sp. SB155-2	E-value = 2.00E-33	Identity = 45.88%
	4		gi|157164206|ref|YP_001466986.1|	Campylobacter concisus 13826	E-value = 7.00E-32	Identity = 47.46%
	5		gi|153951260|ref|YP_001398343.1|	Campylobacter jejuni subsp. doylei 269.97	E-value = 9.00E-32	Identity = 49.44%
AB1965	1		gi|57167723|ref|ZP_00366863.1|	Campylobacter coli RM2228	E-value = 1.00E-12	Identity = 50.00%
	2		gi|152991245|ref|YP_001356967.1|	Nitratiruptor sp. SB155-2	E-value = 1.00E-12	Identity = 51.32%
	3		gi|34556968|ref|NP_906783.1|	Wolinella succinogenes DSM 1740	E-value = 2.00E-12	Identity = 52.63%
	4		gi|152991965|ref|YP_001357686.1|	Sulfurovum sp. NBC37-1	E-value = 3.00E-12	Identity = 51.32%
	5		gi|157164994|ref|YP_001466987.1|	Campylobacter concisus 13826	E-value = 5.00E-12	Identity = 51.32%
AB1966	1	*rpsP*	gi|15792059|ref|NP_281882.1|	Campylobacter jejuni subsp. jejuni NCTC 11168	E-value = 2.00E-22	Identity = 76.00%
	2		gi|152991246|ref|YP_001356968.1|	Nitratiruptor sp. SB155-2	E-value = 2.00E-22	Identity = 76.00%
	3		gi|57167722|ref|ZP_00366862.1|	Campylobacter coli RM2228	E-value = 3.00E-22	Identity = 76.00%
	4		gi|57242692|ref|ZP_00370629.1|	Campylobacter upsaliensis RM3195	E-value = 3.00E-22	Identity = 76.00%
	5		gi|152991964|ref|YP_001357685.1|	Sulfurovum sp. NBC37-1	E-value = 6.00E-22	Identity = 74.67%
AB1967	1	*ffh*	gi|78776651|ref|YP_392966.1|	Sulfuromonas denitrificans ATCC 33889	E-value = 1.00E-148	Identity = 64.41%
	2		gi|157165369|ref|YP_001466989.1|	Campylobacter concisus 13826	E-value = 1.00E-147	Identity = 63.78%
	3		gi|34556966|ref|NP_906781.1|	Wolinella succinogenes DSM 1740	E-value = 1.00E-145	Identity = 61.40%
	4		gi|154174570|ref|YP_001408556.1|	Campylobacter curvus 525.92	E-value = 3.00E-144	Identity = 62.64%
	5		gi|86151630|ref|ZP_01069844.1|	Campylobacter jejuni subsp. jejuni 260.94	E-value = 3.00E-144	Identity = 62.73%
AB1968	1		gi|152991248|ref|YP_001356970.1|	Nitratiruptor sp. SB155-2	E-value = 6.00E-63	Identity = 58.68%
	2		gi|78776648|ref|YP_392963.1|	Sulfuromonas denitrificans ATCC 33889	E-value = 1.00E-60	Identity = 51.64%

	3		gi|118474954|ref|YP_892206.1|	Campylobacter fetus subsp. fetus 82-40	E-value = 2.00E-55	Identity = 52.65%
	4		gi|152991961|ref|YP_001357682.1|	Sulfurovum sp. NBC37-1	E-value = 5.00E-55	Identity = 51.85%
	5		gi|34558398|ref|NP_908213.1|	Wolinella succinogenes DSM 1740	E-value = 4.00E-52	Identity = 49.80%
AB1969	1	*kdtA*	gi|152991958|ref|YP_001357679.1|	Sulfurovum sp. NBC37-1	E-value = 7.00E-107	Identity = 50.90%
	2		gi|78776647|ref|YP_392962.1|	Sulfuromonas denitrificans ATCC 33889	E-value = 1.00E-93	Identity = 47.86%
	3		gi|154173747|ref|YP_001408558.1|	Campylobacter curvus 525.92	E-value = 1.00E-91	Identity = 48.15%
	4		gi|157164650|ref|YP_001466991.1|	Campylobacter concisus 13826	E-value = 2.00E-91	Identity = 47.76%
	5		gi|118475256|ref|YP_892207.1|	Campylobacter fetus subsp. fetus 82-40	E-value = 3.00E-89	Identity = 47.51%
AB1970	1		gi|57167718|ref|ZP_00366858.1|	Campylobacter coli RM2228	E-value = 3.00E-44	Identity = 50.42%
	2		gi|57242696|ref|ZP_00370633.1|	Campylobacter upsaliensis RM3195	E-value = 8.00E-43	Identity = 49.37%
	3		gi|154174062|ref|YP_001408559.1|	Campylobacter curvus 525.92	E-value = 3.00E-42	Identity = 47.88%
	4		gi|148926671|ref|ZP_01810352.1|	Campylobacter jejuni subsp. jejuni CG8486	E-value = 8.00E-42	Identity = 50.42%
	5		gi|152991251|ref|YP_001356973.1|	Nitratiruptor sp. SB155-2	E-value = 8.00E-42	Identity = 53.16%
AB1971	1		gi|149195328|ref|ZP_01872415.1|	Caminibacter mediatlanticus TB-2	E-value = 1.00E-62	Identity = 55.56%
	2		gi|57167717|ref|ZP_00366857.1|	Campylobacter coli RM2228	E-value = 8.00E-62	Identity = 51.02%
	3		gi|78776645|ref|YP_392960.1|	Sulfuromonas denitrificans ATCC 33889	E-value = 2.00E-61	Identity = 49.38%
	4		gi|152991252|ref|YP_001356974.1|	Nitratiruptor sp. SB155-2	E-value = 4.00E-60	Identity = 51.03%
	5		gi|157414992|ref|YP_001482248.1|	Campylobacter jejuni subsp. jejuni 81116	E-value = 3.00E-59	Identity = 52.24%
AB1972	1	*glyQ*	gi|152991955|ref|YP_001357676.1|	Sulfurovum sp. NBC37-1	E-value = 8.00E-147	Identity = 83.51%
	2		gi|78778051|ref|YP_394366.1|	Sulfuromonas denitrificans ATCC 33889	E-value = 8.00E-145	Identity = 82.82%
	3		gi|152991253|ref|YP_001356975.1|	Nitratiruptor sp. SB155-2	E-value = 2.00E-139	Identity = 82.27%
	4		gi|34558401|ref|NP_908216.1|	Wolinella succinogenes DSM 1740	E-value = 1.00E-133	Identity = 77.13%
	5		gi|32266030|ref|NP_860062.1|	Helicobacter hepaticus ATCC 51449	E-value = 4.00E-131	Identity = 74.57%
AB1973	1		gi|152991254|ref|YP_001356976.1|	Nitratiruptor sp. SB155-2	E-value = 3.00E-15	Identity = 49.33%
	2		gi|149195324|ref|ZP_01872411.1|	Caminibacter mediatlanticus TB-2	E-value = 8.00E-14	Identity = 48.68%
	3		gi|152993541|ref|YP_001359262.1|	Sulfurovum sp. NBC37-1	E-value = 3.00E-13	Identity = 49.33%
	4		gi|145621400|ref|ZP_01777369.1|	Petrotoga mobilis SJ95	E-value = 1.00E-09	Identity = 37.84%
	5		gi|108804446|ref|YP_644383.1|	Rubrobacter xylanophilus DSM 9941	E-value = 8.00E-08	Identity = 31.58%
AB1974	1	*purE*	gi|152991256|ref|YP_001356978.1|	Nitratiruptor sp. SB155-2	E-value = 5.00E-55	Identity = 75.00%
	2		gi|32266027|ref|NP_860059.1|	Helicobacter hepaticus ATCC 51449	E-value = 2.00E-51	Identity = 67.28%
	3		gi|149195322|ref|ZP_01872409.1|	Caminibacter mediatlanticus TB-2	E-value = 4.00E-51	Identity = 66.46%
	4		gi|78778057|ref|YP_394372.1|	Sulfuromonas denitrificans ATCC 33889	E-value = 8.00E-49	Identity = 72.56%
	5		gi|34558404|ref|NP_908219.1|	Wolinella succinogenes DSM 1740	E-value = 1.00E-48	Identity = 73.17%
AB1975	1			***** No matches found *****		
AB1976	1		gi|34558405|ref|NP_908220.1|	Wolinella succinogenes DSM 1740	E-value = 5.00E-164	Identity = 64.44%
	2		gi|152991258|ref|YP_001356980.1|	Nitratiruptor sp. SB155-2	E-value = 2.00E-161	Identity = 66.27%
	3		gi|78778058|ref|YP_394373.1|	Sulfuromonas denitrificans ATCC 33889	E-value = 7.00E-160	Identity = 66.19%
	4		gi|152991951|ref|YP_001357672.1|	Sulfurovum sp. NBC37-1	E-value = 1.00E-157	Identity = 63.40%
	5		gi|109946960|ref|YP_664188.1|	Helicobacter acinonychis str. Sheeba	E-value = 1.00E-156	Identity = 64.66%
AB1977	1		gi|95930248|ref|ZP_01312986.1|	Desulfuromonas acetoxidans DSM 684	E-value = 5.00E-66	Identity = 29.73%
	2		gi|95929898|ref|ZP_01312639.1|	Desulfuromonas acetoxidans DSM 684	E-value = 3.00E-64	Identity = 30.87%
	3		gi|15889206|ref|NP_354887.1|	Agrobacterium tumefaciens str. C58	E-value = 2.00E-62	Identity = 31.40%
	4		gi|116252678|ref|YP_768516.1|	Rhizobium leguminosarum bv. viciae 3841	E-value = 8.00E-61	Identity = 30.52%
	5		gi|116255096|ref|YP_770930.1|	Rhizobium leguminosarum bv. viciae 3841	E-value = 3.00E-52	Identity = 27.57%
AB1978	1	*recN*	gi|152990520|ref|YP_001356242.1|	Nitratiruptor sp. SB155-2	E-value = 5.00E-102	Identity = 50.30%
	2		gi|78777282|ref|YP_393597.1|	Sulfuromonas denitrificans ATCC 33889	E-value = 8.00E-99	Identity = 47.15%
	3		gi|157163888|ref|YP_001467179.1|	Campylobacter concisus 13826	E-value = 3.00E-93	Identity = 47.21%
	4		gi|154147949|ref|YP_001405901.1|	Campylobacter hominis ATCC BAA-381	E-value = 7.00E-93	Identity = 48.23%
	5		gi|118475107|ref|YP_892352.1|	Campylobacter fetus subsp. fetus 82-40	E-value = 7.00E-89	Identity = 44.79%

AB1979	1		gi|152992823|ref|YP_001358544.1|	Sulfurovum sp. NBC37-1	E-value = 6.00E-82	Identity = 51.57%
	2		gi|78777283|ref|YP_393598.1|	Sulfuromonas denitrificans ATCC 33889	E-value = 8.00E-82	Identity = 51.60%
	3		gi|34557940|ref|NP_907755.1|	Wolinella succinogenes DSM 1740	E-value = 1.00E-77	Identity = 45.23%
	4		gi|152990519|ref|YP_001356241.1|	Nitratiruptor sp. SB155-2	E-value = 1.00E-73	Identity = 49.64%
	5		gi|149194530|ref|ZP_01871626.1|	Caminibacter mediatlanticus TB-2	E-value = 6.00E-72	Identity = 51.10%
AB1980	1	*fus*	gi|57240981|ref|ZP_00368929.1|	Campylobacter lari RM2100	E-value = 0	Identity = 76.67%
	2		gi|154149329|ref|YP_001407177.1|	Campylobacter hominis ATCC BAA-381	E-value = 0	Identity = 75.78%
	3		gi|148925869|ref|ZP_01809556.1|	Campylobacter jejuni subsp. jejuni CG8486	E-value = 0	Identity = 75.78%
	4		gi|153952626|ref|YP_001398475.1|	Campylobacter jejuni subsp. doylei 269.97	E-value = 0	Identity = 75.93%
	5		gi|15791857|ref|NP_281680.1|	Campylobacter jejuni subsp. jejuni NCTC 11168	E-value = 0	Identity = 75.93%
AB1981	1	*rpsG*	gi|149195225|ref|ZP_01872315.1|	Caminibacter mediatlanticus TB-2	E-value = 3.00E-55	Identity = 74.19%
	2		gi|78776557|ref|YP_392872.1|	Sulfuromonas denitrificans ATCC 33889	E-value = 2.00E-54	Identity = 72.26%
	3		gi|57242430|ref|ZP_00370368.1|	Campylobacter upsaliensis RM3195	E-value = 4.00E-54	Identity = 69.87%
	4		gi|154174218|ref|YP_001408637.1|	Campylobacter curvus 525.92	E-value = 1.00E-53	Identity = 70.51%
	5		gi|32265858|ref|NP_859890.1|	Helicobacter hepaticus ATCC 51449	E-value = 3.00E-53	Identity = 72.90%
AB1982	1	*rpsL*	gi|57504742|ref|ZP_00370796.1|	Campylobacter coli RM2228	E-value = 2.00E-50	Identity = 94.40%
	2		gi|57242431|ref|ZP_00370369.1|	Campylobacter upsaliensis RM3195	E-value = 2.00E-50	Identity = 92.13%
	3		gi|57240979|ref|ZP_00368927.1|	Campylobacter lari RM2100	E-value = 3.00E-50	Identity = 92.91%
	4		gi|118474757|ref|YP_892465.1|	Campylobacter fetus subsp. fetus 82-40	E-value = 5.00E-50	Identity = 93.70%
	5		gi|15791855|ref|NP_281678.1|	Campylobacter jejuni subsp. jejuni NCTC 11168	E-value = 5.00E-50	Identity = 93.60%
AB1983	1		gi|114327835|ref|YP_744992.1|	Granulibacter bethesdensis CGDNIH1	E-value = 6.00E-24	Identity = 30.71%
	2		gi|124870207|gb|EAY61923.1|	Burkholderia cenocepacia PC184	E-value = 1.00E-23	Identity = 27.20%
	3		gi|134293411|ref|YP_001117147.1|	Burkholderia vietnamiensis G4	E-value = 2.00E-22	Identity = 25.60%
	4		gi|78062301|ref|YP_372209.1|	Burkholderia sp. 383	E-value = 4.00E-21	Identity = 26.00%
	5		gi|157370545|ref|YP_001478534.1|	Serratia proteamaculans 568	E-value = 3.00E-19	Identity = 26.27%
AB1984	1	*araJ*	gi|146297951|ref|YP_001192542.1|	Flavobacterium johnsoniae UW101	E-value = 2.00E-100	Identity = 55.00%
	2		gi|149277701|ref|ZP_01883841.1|	Pedobacter sp. BAL39	E-value = 3.00E-92	Identity = 52.45%
	3		gi|86140318|ref|ZP_01058877.1|	Flavobacterium sp. MED217	E-value = 3.00E-86	Identity = 49.19%
	4		gi|120435108|ref|YP_860794.1|	Gramella forsetii KT0803	E-value = 8.00E-82	Identity = 48.52%
	5		gi|29348522|ref|NP_812025.1|	Bacteroides thetaiotaomicron VPI-5482	E-value = 3.00E-78	Identity = 44.74%
AB1985	1		gi|78777203|ref|YP_393518.1|	Sulfuromonas denitrificans ATCC 33889	E-value = 6.00E-123	Identity = 39.09%
	2		gi|34557301|ref|NP_907116.1|	Wolinella succinogenes DSM 1740	E-value = 2.00E-112	Identity = 37.77%
	3		gi|34557772|ref|NP_907587.1|	Wolinella succinogenes DSM 1740	E-value = 1.00E-79	Identity = 30.72%
	4		gi|78187396|ref|YP_375439.1|	Pelodictyon luteolum DSM 273	E-value = 2.00E-56	Identity = 29.55%
	5		gi|68552057|ref|ZP_00591450.1|	Prosthecochloris aestuarii DSM 271	E-value = 3.00E-56	Identity = 29.42%
AB1986	1		gi|78777544|ref|YP_393859.1|	Sulfuromonas denitrificans ATCC 33889	E-value = 3.00E-104	Identity = 62.58%
	2		gi|34557300|ref|NP_907115.1|	Wolinella succinogenes DSM 1740	E-value = 2.00E-93	Identity = 52.20%
	3		gi|152991332|ref|YP_001357054.1|	Nitratiruptor sp. SB155-2	E-value = 7.00E-89	Identity = 53.92%
	4		gi|56476605|ref|YP_158194.1|	Azoarcus sp. EbN1	E-value = 6.00E-87	Identity = 49.67%
	5		gi|30249182|ref|NP_841252.1|	Nitrosomonas europaea ATCC 19718	E-value = 2.00E-84	Identity = 47.40%
AB1987	1		gi|78777547|ref|YP_393862.1|	Sulfuromonas denitrificans ATCC 33889	E-value = 7.00E-98	Identity = 49.88%
	2		gi|152991335|ref|YP_001357057.1|	Nitratiruptor sp. SB155-2	E-value = 8.00E-84	Identity = 46.19%
	3		gi|34557296|ref|NP_907111.1|	Wolinella succinogenes DSM 1740	E-value = 1.00E-60	Identity = 41.38%
	4		gi|30249179|ref|NP_841249.1|	Nitrosomonas europaea ATCC 19718	E-value = 3.00E-25	Identity = 29.12%
	5		gi|56476602|ref|YP_158191.1|	Azoarcus sp. EbN1	E-value = 2.00E-23	Identity = 27.68%
AB1988	1		gi|152991334|ref|YP_001357056.1|	Nitratiruptor sp. SB155-2	E-value = 8.00E-82	Identity = 67.21%
	2		gi|78777546|ref|YP_393861.1|	Sulfuromonas denitrificans ATCC 33889	E-value = 5.00E-81	Identity = 67.48%
	3		gi|34557297|ref|NP_907112.1|	Wolinella succinogenes DSM 1740	E-value = 3.00E-64	Identity = 51.63%
	4		gi|30249180|ref|NP_841250.1|	Nitrosomonas europaea ATCC 19718	E-value = 9.00E-64	Identity = 53.68%

	5		gi|56476603|ref|YP_158192.1|	Azoarcus sp. EbN1	E-value = 4.00E-57	Identity = 47.08%
AB1989	1		gi|78777545|ref|YP_393860.1|	Sulfuromonas denitrificans ATCC 33889	E-value = 3.00E-96	Identity = 73.08%
	2		gi|152991333|ref|YP_001357055.1|	Nitratiruptor sp. SB155-2	E-value = 8.00E-87	Identity = 65.00%
	3		gi|34557298|ref|NP_907113.1|	Wolinella succinogenes DSM 1740	E-value = 3.00E-82	Identity = 64.62%
	4		gi|56476604|ref|YP_158193.1|	Azoarcus sp. EbN1	E-value = 6.00E-74	Identity = 59.84%
	5		gi|30249181|ref|NP_841251.1|	Nitrosomonas europaea ATCC 19718	E-value = 1.00E-66	Identity = 55.56%
AB1990	1		gi|154148250|ref|YP_001406400.1|	Campylobacter hominis ATCC BAA-381	E-value = 2.00E-12	Identity = 26.42%
	2		gi|152993495|ref|YP_001359216.1|	Sulfurovum sp. NBC37-1	E-value = 2.00E-12	Identity = 28.84%
	3		gi|154174416|ref|YP_001408602.1|	Campylobacter curvus 525.92	E-value = 4.00E-12	Identity = 26.55%
	4		gi|78776771|ref|YP_393086.1|	Sulfuromonas denitrificans ATCC 33889	E-value = 3.00E-10	Identity = 30.23%
	5		gi|34558779|gb|AAQ75124.1|	Alvinella pompejana epibiont 6C6	E-value = 3.00E-09	Identity = 25.00%
AB1991	1		gi|157165779|ref|YP_001466577.1|	Campylobacter concisus 13826	E-value = 1.00E-64	Identity = 43.06%
	2		gi|154173952|ref|YP_001408603.1|	Campylobacter curvus 525.92	E-value = 3.00E-63	Identity = 41.62%
	3		gi|154148550|ref|YP_001406399.1|	Campylobacter hominis ATCC BAA-381	E-value = 1.00E-59	Identity = 40.37%
	4		gi|57240966|ref|ZP_00368914.1|	Campylobacter lari RM2100	E-value = 1.00E-59	Identity = 42.49%
	5		gi|118474965|ref|YP_891769.1|	Campylobacter fetus subsp. fetus 82-40	E-value = 2.00E-59	Identity = 42.08%
AB1992	1	*trpG*	gi|152990399|ref|YP_001356121.1|	Nitratiruptor sp. SB155-2	E-value = 9.00E-78	Identity = 74.60%
	2		gi|78776767|ref|YP_393082.1|	Sulfuromonas denitrificans ATCC 33889	E-value = 9.00E-77	Identity = 75.13%
	3		gi|152992135|ref|YP_001357856.1|	Sulfurovum sp. NBC37-1	E-value = 5.00E-76	Identity = 70.37%
	4		gi|34558448|ref|NP_908263.1|	Wolinella succinogenes DSM 1740	E-value = 9.00E-72	Identity = 69.15%
	5		gi|149195268|ref|ZP_01872357.1|	Caminibacter mediatlanticus TB-2	E-value = 3.00E-69	Identity = 69.31%
AB1993	1	*fbpA*	gi|134299387|ref|YP_001112883.1|	Desulfotomaculum reducens MI-1	E-value = 3.00E-87	Identity = 53.50%
	2		gi|149180748|ref|ZP_01859251.1|	Bacillus sp. SG-1	E-value = 5.00E-85	Identity = 50.47%
	3		gi|91217373|ref|ZP_01254333.1|	Psychroflexus torquis ATCC 700755	E-value = 2.00E-84	Identity = 50.16%
	4		gi|86606749|ref|YP_475512.1|	Synechococcus sp. JA-3-3Ab	E-value = 1.00E-82	Identity = 46.71%
	5		gi|86610096|ref|YP_478858.1|	Synechococcus sp. JA-2-3B'a(2-13)	E-value = 6.00E-82	Identity = 45.35%
AB1994	1	*fbpB*	gi|152997754|ref|YP_001342589.1|	Marinomonas sp. MWYL1	E-value = 3.00E-71	Identity = 32.62%
	2		gi|118474264|ref|YP_891326.1|	Campylobacter fetus subsp. fetus 82-40	E-value = 5.00E-71	Identity = 36.63%
	3		gi|89095388|ref|ZP_01168303.1|	Oceanospirillum sp. MED92	E-value = 5.00E-71	Identity = 33.78%
	4		gi|22299255|ref|NP_682502.1|	Thermosynechococcus elongatus BP-1	E-value = 7.00E-69	Identity = 32.70%
	5		gi|85713063|ref|ZP_01044099.1|	Idiomarina baltica OS145	E-value = 8.00E-69	Identity = 31.53%
AB1996	1		gi|22299035|ref|NP_682282.1|	Thermosynechococcus elongatus BP-1	E-value = 1.00E-58	Identity = 40.39%
	2		gi|4514346|dbj|BAA75384.1|	Bacillus halodurans	E-value = 1.00E-57	Identity = 38.76%
	3		gi|15613075|ref|NP_241378.1|	Bacillus halodurans C-125	E-value = 1.00E-57	Identity = 38.76%
	4		gi|42780882|ref|NP_978129.1|	Bacillus cereus ATCC 10987	E-value = 5.00E-57	Identity = 37.97%
	5		gi|16330805|ref|NP_441533.1|	Synechocystis sp. PCC 6803	E-value = 6.00E-57	Identity = 36.34%
AB1997	1		gi|34556724|ref|NP_906539.1|	Wolinella succinogenes DSM 1740	E-value = 9.00E-77	Identity = 47.38%
	2		gi|17229700|ref|NP_486248.1|	Nostoc sp. PCC 7120	E-value = 4.00E-13	Identity = 25.74%
	3		gi|146298703|ref|YP_001193294.1|	Flavobacterium johnsoniae UW101	E-value = 9.00E-08	Identity = 27.24%
AB1998	1		gi|34556725|ref|NP_906540.1|	Wolinella succinogenes DSM 1740	E-value = 0	Identity = 53.60%
	2		gi|17229677|ref|NP_486225.1|	Nostoc sp. PCC 7120	E-value = 1.00E-83	Identity = 30.72%
	3		gi|37519930|ref|NP_923307.1|	Gloeobacter violaceus PCC 7421	E-value = 2.00E-77	Identity = 27.88%
	4		gi|16329191|ref|NP_439919.1|	Synechocystis sp. PCC 6803	E-value = 5.00E-76	Identity = 30.90%
	5		gi|126658812|ref|ZP_01729956.1|	Cyanothece sp. CCY0110	E-value = 2.00E-69	Identity = 27.34%
AB1999	1		gi|34557009|ref|NP_906824.1|	Wolinella succinogenes DSM 1740	E-value = 1.00E-12	Identity = 51.14%
AB2000	1		gi|34557010|ref|NP_906825.1|	Wolinella succinogenes DSM 1740	E-value = 2.00E-127	Identity = 44.36%
	2		gi|118071667|ref|ZP_01539861.1|	Shewanella woodyi ATCC 51908	E-value = 1.00E-06	Identity = 25.80%
AB2001	1		gi|34557011|ref|NP_906826.1|	Wolinella succinogenes DSM 1740	E-value = 4.00E-24	Identity = 42.07%
	2		gi|42527566|ref|NP_972664.1|	Treponema denticola ATCC 35405	E-value = 2.00E-15	Identity = 34.34%

AB2002	1	*nirA*	gi|78776360|ref|YP_392675.1|	Sulfuromonas denitrificans ATCC 33889	E-value = 6.00E-119	Identity = 42.47%
	2		gi|111220484|ref|YP_711278.1|	Frankia alni ACN14a	E-value = 1.00E-90	Identity = 37.38%
	3		gi|86739238|ref|YP_479638.1|	Frankia sp. CcI3	E-value = 4.00E-88	Identity = 36.17%
	4		gi|29828669|ref|NP_823303.1|	Streptomyces avermitilis MA-4680	E-value = 6.00E-88	Identity = 36.23%
	5		gi|68232797|ref|ZP_00571935.1|	Frankia sp. EAN1pec	E-value = 7.00E-88	Identity = 35.42%
AB2003	1		gi|78777886|ref|YP_394201.1|	Sulfuromonas denitrificans ATCC 33889	E-value = 4.00E-114	Identity = 56.85%
	2		gi|152994429|ref|YP_001339264.1|	Marinomonas sp. MWYL1	E-value = 2.00E-112	Identity = 57.10%
	3		gi|90022558|ref|YP_528385.1|	Saccharophagus degradans 2-40	E-value = 2.00E-111	Identity = 55.72%
	4		gi|78484621|ref|YP_390546.1|	Thiomicrospira crunogena XCL-2	E-value = 8.00E-110	Identity = 55.94%
	5		gi|109896554|ref|YP_659809.1|	Pseudoalteromonas atlantica T6c	E-value = 5.00E-108	Identity = 53.96%
AB2004	1			***** No matches found *****		
AB2005	1		gi|126734957|ref|ZP_01750703.1|	Roseobacter sp. CCS2	E-value = 2.00E-12	Identity = 31.50%
	2		gi|116623373|ref|YP_825529.1|	Solibacter usitatus Ellin6076	E-value = 1.00E-11	Identity = 28.46%
	3		gi|148658298|ref|YP_001278503.1|	Roseiflexus sp. RS-1	E-value = 5.00E-11	Identity = 25.78%
	4		gi|94971000|ref|YP_593048.1|	Acidobacteria bacterium Ellin345	E-value = 1.00E-10	Identity = 29.01%
	5		gi|156740461|ref|YP_001430590.1|	Roseiflexus castenholzii DSM 13941	E-value = 1.00E-10	Identity = 25.56%
AB2006	1		gi|152992317|ref|YP_001358038.1|	Sulfurovum sp. NBC37-1	E-value = 0	Identity = 53.65%
	2		gi|78776336|ref|YP_392651.1|	Sulfuromonas denitrificans ATCC 33889	E-value = 0	Identity = 56.16%
	3		gi|76797414|ref|ZP_00779734.1|	Thermoanaerobacter ethanolicus ATCC 33223	E-value = 2.00E-163	Identity = 46.38%
	4		gi|125974456|ref|YP_001038366.1|	Clostridium thermocellum ATCC 27405	E-value = 2.00E-160	Identity = 44.62%
	5		gi|106893073|ref|ZP_01360199.1|	Clostridium sp. OhILAs	E-value = 2.00E-156	Identity = 46.64%
AB2007	1		gi|78776523|ref|YP_392838.1|	Sulfuromonas denitrificans ATCC 33889	E-value = 3.00E-25	Identity = 53.45%
AB2008	1		gi|27363650|ref|NP_759178.1|	Vibrio vulnificus CMCP6	E-value = 4.00E-35	Identity = 57.94%
	2		gi|118072364|ref|ZP_01540554.1|	Shewanella woodyi ATCC 51908	E-value = 9.00E-35	Identity = 60.00%
	3		gi|117619955|ref|YP_858453.1|	Aeromonas hydrophila subsp. hydrophila ATCC 7966	E-value = 1.00E-34	Identity = 62.10%
	4		gi|106885988|ref|ZP_01353338.1|	Clostridium phytofermentans ISDg	E-value = 2.00E-34	Identity = 63.93%
	5		gi|90409711|ref|ZP_01217728.1|	Photobacterium profundum 3TCK	E-value = 3.00E-34	Identity = 58.40%
AB2009	1		gi|87121405|ref|ZP_01077294.1|	Marinomonas sp. MED121	E-value = 3.00E-36	Identity = 34.30%
	2		gi|84394198|ref|ZP_00992928.1|	Vibrio splendidus 12B01	E-value = 3.00E-35	Identity = 34.16%
	3		gi|148976266|ref|ZP_01812990.1|	Vibrionales bacterium SWAT-3	E-value = 3.00E-35	Identity = 34.16%
	4		gi|86144986|ref|ZP_01063318.1|	Vibrio sp. MED222	E-value = 6.00E-35	Identity = 33.74%
	5		gi|71736796|ref|YP_274967.1|	Pseudomonas syringae pv. phaseolicola 1448A	E-value = 8.00E-34	Identity = 34.08%
AB2010	1		gi|78776820|ref|YP_393135.1|	Sulfuromonas denitrificans ATCC 33889	E-value = 3.00E-91	Identity = 58.22%
	2		gi|34557184|ref|NP_906999.1|	Wolinella succinogenes DSM 1740	E-value = 3.00E-74	Identity = 46.13%
	3		gi|59714034|ref|YP_206809.1|	Vibrio fischeri ES114	E-value = 4.00E-45	Identity = 34.52%
	4		gi|116187268|ref|ZP_01477157.1|	Vibrio sp. Ex25	E-value = 4.00E-45	Identity = 34.97%
	5		gi|28900255|ref|NP_799910.1|	Vibrio parahaemolyticus RIMD 2210633	E-value = 6.00E-45	Identity = 34.62%
AB2011	1		gi|34557183|ref|NP_906998.1|	Wolinella succinogenes DSM 1740	E-value = 3.00E-84	Identity = 43.21%
	2		gi|52632015|gb|AAU85415.1|	uncultured archaeon GZfos12E1	E-value = 3.00E-67	Identity = 39.73%
	3		gi|126652114|ref|ZP_01724296.1|	Bacillus sp. B14905	E-value = 1.00E-66	Identity = 39.37%
	4		gi|51245462|ref|YP_065346.1|	Desulfotalea psychrophila LSv54	E-value = 3.00E-66	Identity = 39.62%
	5		gi|145622093|ref|ZP_01778055.1|	Petrotoga mobilis SJ95	E-value = 2.00E-64	Identity = 39.84%
AB2012	1			***** No matches found *****		
AB2013	1		gi|34556727|ref|NP_906542.1|	Wolinella succinogenes DSM 1740	E-value = 2.00E-59	Identity = 66.84%
	2		gi|74318099|ref|YP_315839.1|	Thiobacillus denitrificans ATCC 25259	E-value = 1.00E-58	Identity = 65.83%
	3		gi|152993739|ref|YP_001359460.1|	Sulfurovum sp. NBC37-1	E-value = 1.00E-55	Identity = 59.30%
	4		gi|118474566|ref|YP_891995.1|	Campylobacter fetus subsp. fetus 82-40	E-value = 2.00E-54	Identity = 61.19%
	5		gi|78778000|ref|YP_394315.1|	Sulfuromonas denitrificans ATCC 33889	E-value = 2.00E-51	Identity = 57.00%
AB2014	1	*rplS*	gi|32266434|ref|NP_860466.1|	Helicobacter hepaticus ATCC 51449	E-value = 9.00E-33	Identity = 66.95%

	2		gi|15612139|ref|NP_223791.1|	Helicobacter pylori J99	E-value = 3.00E-32	Identity = 64.96%
	3		gi|34556971|ref|NP_906786.1|	Wolinella succinogenes DSM 1740	E-value = 3.00E-32	Identity = 65.25%
	4		gi|15645761|ref|NP_207938.1|	Helicobacter pylori 26695	E-value = 4.00E-32	Identity = 64.96%
	5		gi|109947824|ref|YP_665052.1|	Helicobacter acinonychis str. Sheeba	E-value = 7.00E-32	Identity = 65.81%
AB2015	1	*trmD*	gi|152991967|ref|YP_001357688.1|	Sulfurovum sp. NBC37-1	E-value = 1.00E-81	Identity = 65.49%
	2		gi|78776655|ref|YP_392970.1|	Sulfuromonas denitrificans ATCC 33889	E-value = 3.00E-80	Identity = 67.27%
	3		gi|157164613|ref|YP_001466985.1|	Campylobacter concisus 13826	E-value = 3.00E-73	Identity = 60.81%
	4		gi|154174128|ref|YP_001408553.1|	Campylobacter curvus 525.92	E-value = 7.00E-73	Identity = 61.26%
	5		gi|118475610|ref|YP_892199.1|	Campylobacter fetus subsp. fetus 82-40	E-value = 4.00E-69	Identity = 57.21%
AB2016	1			***** No matches found *****		
AB2017	1	*metE*	gi|152992121|ref|YP_001357842.1|	Sulfurovum sp. NBC37-1	E-value = 0	Identity = 65.56%
	2		gi|78777097|ref|YP_393412.1|	Sulfuromonas denitrificans ATCC 33889	E-value = 0	Identity = 65.96%
	3		gi|154174273|ref|YP_001407376.1|	Campylobacter curvus 525.92	E-value = 0	Identity = 65.52%
	4		gi|118474569|ref|YP_892052.1|	Campylobacter fetus subsp. fetus 82-40	E-value = 0	Identity = 63.93%
	5		gi|134299605|ref|YP_001113101.1|	Desulfotomaculum reducens MI-1	E-value = 0	Identity = 58.43%
AB2018	1	*ilvA*	gi|152993410|ref|YP_001359131.1|	Sulfurovum sp. NBC37-1	E-value = 2.00E-128	Identity = 59.60%
	2		gi|149193905|ref|ZP_01871003.1|	Caminibacter mediatlanticus TB-2	E-value = 5.00E-126	Identity = 60.15%
	3		gi|57167828|ref|ZP_00366968.1|	Campylobacter coli RM2228	E-value = 4.00E-120	Identity = 58.35%
	4		gi|78777341|ref|YP_393656.1|	Sulfuromonas denitrificans ATCC 33889	E-value = 5.00E-120	Identity = 56.17%
	5		gi|118474294|ref|YP_891918.1|	Campylobacter fetus subsp. fetus 82-40	E-value = 2.00E-118	Identity = 58.10%
AB2019	1	*atpB*	gi|152993168|ref|YP_001358889.1|	Sulfurovum sp. NBC37-1	E-value = 5.00E-64	Identity = 59.47%
	2		gi|34556754|ref|NP_906569.1|	Wolinella succinogenes DSM 1740	E-value = 6.00E-62	Identity = 59.01%
	3		gi|152990600|ref|YP_001356322.1|	Nitratiruptor sp. SB155-2	E-value = 8.00E-62	Identity = 57.52%
	4		gi|118475487|ref|YP_891946.1|	Campylobacter fetus subsp. fetus 82-40	E-value = 5.00E-60	Identity = 59.62%
	5		gi|154175380|ref|YP_001408364.1|	Campylobacter curvus 525.92	E-value = 9.00E-60	Identity = 60.19%
AB2020	1		gi|78777436|ref|YP_393751.1|	Sulfuromonas denitrificans ATCC 33889	E-value = 7.00E-40	Identity = 50.54%
	2		gi|152990601|ref|YP_001356323.1|	Nitratiruptor sp. SB155-2	E-value = 4.00E-28	Identity = 40.33%
	3		gi|34556756|ref|NP_906571.1|	Wolinella succinogenes DSM 1740	E-value = 3.00E-27	Identity = 37.84%
	4		gi|118474768|ref|YP_891948.1|	Campylobacter fetus subsp. fetus 82-40	E-value = 2.00E-18	Identity = 37.30%
	5		gi|149194901|ref|ZP_01871995.1|	Caminibacter mediatlanticus TB-2	E-value = 3.00E-13	Identity = 38.38%
AB2021	1	*porA*	gi|34558087|ref|NP_907902.1|	Wolinella succinogenes DSM 1740	E-value = 1.00E-15	Identity = 30.08%
	2		gi|154174725|ref|YP_001408057.1|	Campylobacter curvus 525.92	E-value = 3.00E-09	Identity = 25.61%
	3		gi|149195106|ref|ZP_01872198.1|	Caminibacter mediatlanticus TB-2	E-value = 9.00E-06	Identity = 26.20%
AB2022	1	*folB*	gi|78776864|ref|YP_393179.1|	Sulfuromonas denitrificans ATCC 33889	E-value = 1.00E-15	Identity = 53.85%
	2		gi|152993561|ref|YP_001359282.1|	Sulfurovum sp. NBC37-1	E-value = 3.00E-10	Identity = 44.66%
	3		gi|34556464|ref|NP_906279.1|	Wolinella succinogenes DSM 1740	E-value = 2.00E-09	Identity = 37.86%
	4		gi|57242731|ref|ZP_00370668.1|	Campylobacter upsaliensis RM3195	E-value = 4.00E-08	Identity = 42.71%
	5		gi|109946706|ref|YP_663934.1|	Helicobacter acinonychis str. Sheeba	E-value = 1.00E-07	Identity = 42.16%
AB2023	1		gi|78776865|ref|YP_393180.1|	Sulfuromonas denitrificans ATCC 33889	E-value = 1.00E-56	Identity = 61.35%
	2		gi|152990162|ref|YP_001355884.1|	Nitratiruptor sp. SB155-2	E-value = 4.00E-53	Identity = 59.90%
	3		gi|57240710|ref|ZP_00368658.1|	Campylobacter lari RM2100	E-value = 8.00E-50	Identity = 57.43%
	4		gi|57242730|ref|ZP_00370667.1|	Campylobacter upsaliensis RM3195	E-value = 5.00E-49	Identity = 56.93%
	5		gi|157164453|ref|YP_001466305.1|	Campylobacter concisus 13826	E-value = 4.00E-48	Identity = 57.43%
AB2024	1	*nadA*	gi|78776866|ref|YP_393181.1|	Sulfuromonas denitrificans ATCC 33889	E-value = 5.00E-134	Identity = 71.60%
	2		gi|152993559|ref|YP_001359280.1|	Sulfurovum sp. NBC37-1	E-value = 1.00E-128	Identity = 68.18%
	3		gi|152990163|ref|YP_001355885.1|	Nitratiruptor sp. SB155-2	E-value = 2.00E-127	Identity = 66.77%
	4		gi|34557638|ref|NP_907453.1|	Wolinella succinogenes DSM 1740	E-value = 1.00E-121	Identity = 64.35%
	5		gi|32267338|ref|NP_861370.1|	Helicobacter hepaticus ATCC 51449	E-value = 1.00E-105	Identity = 56.25%
AB2025	1	*nadC*	gi|78776867|ref|YP_393182.1|	Sulfuromonas denitrificans ATCC 33889	E-value = 1.00E-86	Identity = 58.15%

	2		gi|152993558|ref|YP_001359279.1|	Sulfurovum sp. NBC37-1	E-value = 5.00E-81	Identity = 55.02%
	3		gi|152990164|ref|YP_001355886.1|	Nitratiruptor sp. SB155-2	E-value = 1.00E-73	Identity = 52.19%
	4		gi|149194891|ref|ZP_01871985.1|	Caminibacter mediatlanticus TB-2	E-value = 8.00E-71	Identity = 54.28%
	5		gi|32267332|ref|NP_861364.1|	Helicobacter hepaticus ATCC 51449	E-value = 2.00E-70	Identity = 49.82%
AB2026	1		gi|152990165|ref|YP_001355887.1|	Nitratiruptor sp. SB155-2	E-value = 4.00E-54	Identity = 40.58%
	2		gi|152993557|ref|YP_001359278.1|	Sulfurovum sp. NBC37-1	E-value = 2.00E-51	Identity = 39.29%
	3		gi|149194191|ref|ZP_01871289.1|	Caminibacter mediatlanticus TB-2	E-value = 3.00E-50	Identity = 38.61%
	4		gi|94263054|ref|ZP_01286873.1|	delta proteobacterium MLMS-1	E-value = 3.00E-43	Identity = 35.81%
	5		gi|15606737|ref|NP_214117.1|	Aquifex aeolicus VF5	E-value = 8.00E-38	Identity = 34.98%
AB2027	1		gi|149194190|ref|ZP_01871288.1|	Caminibacter mediatlanticus TB-2	E-value = 7.00E-85	Identity = 42.73%
	2		gi|152990166|ref|YP_001355888.1|	Nitratiruptor sp. SB155-2	E-value = 1.00E-84	Identity = 36.64%
	3		gi|78776870|ref|YP_393185.1|	Sulfuromonas denitrificans ATCC 33889	E-value = 1.00E-80	Identity = 38.78%
	4		gi|118475260|ref|YP_892633.1|	Campylobacter fetus subsp. fetus 82-40	E-value = 2.00E-79	Identity = 38.02%
	5		gi|154174642|ref|YP_001408788.1|	Campylobacter curvus 525.92	E-value = 7.00E-78	Identity = 35.85%
AB2028	1	*lpxC*	gi|152990167|ref|YP_001355889.1|	Nitratiruptor sp. SB155-2	E-value = 2.00E-103	Identity = 62.75%
	2		gi|149194188|ref|ZP_01871286.1|	Caminibacter mediatlanticus TB-2	E-value = 4.00E-97	Identity = 64.08%
	3		gi|78776871|ref|YP_393186.1|	Sulfuromonas denitrificans ATCC 33889	E-value = 3.00E-96	Identity = 63.30%
	4		gi|32266514|ref|NP_860546.1|	Helicobacter hepaticus ATCC 51449	E-value = 7.00E-95	Identity = 62.54%
	5		gi|34558300|ref|NP_908115.1|	Wolinella succinogenes DSM 1740	E-value = 2.00E-93	Identity = 62.54%
AB2029	1		gi|152990168|ref|YP_001355890.1|	Nitratiruptor sp. SB155-2	E-value = 2.00E-28	Identity = 41.33%
	2		gi|78776872|ref|YP_393187.1|	Sulfuromonas denitrificans ATCC 33889	E-value = 8.00E-27	Identity = 44.97%
	3		gi|149194187|ref|ZP_01871285.1|	Caminibacter mediatlanticus TB-2	E-value = 1.00E-26	Identity = 50.67%
	4		gi|152993553|ref|YP_001359274.1|	Sulfurovum sp. NBC37-1	E-value = 5.00E-23	Identity = 39.86%
	5		gi|57240752|ref|ZP_00368700.1|	Campylobacter lari RM2100	E-value = 3.00E-19	Identity = 40.58%
AB2030	1	*thrB*	gi|152993552|ref|YP_001359273.1|	Sulfurovum sp. NBC37-1	E-value = 6.00E-98	Identity = 60.55%
	2		gi|78776873|ref|YP_393188.1|	Sulfuromonas denitrificans ATCC 33889	E-value = 1.00E-93	Identity = 61.64%
	3		gi|34558302|ref|NP_908117.1|	Wolinella succinogenes DSM 1740	E-value = 7.00E-93	Identity = 57.44%
	4		gi|152990169|ref|YP_001355891.1|	Nitratiruptor sp. SB155-2	E-value = 2.00E-90	Identity = 63.70%
	5		gi|57168695|ref|ZP_00367827.1|	Campylobacter coli RM2228	E-value = 6.00E-87	Identity = 56.01%
AB2031	1			***** No matches found *****		
AB2032	1	*infB*	gi|152993550|ref|YP_001359271.1|	Sulfurovum sp. NBC37-1	E-value = 0	Identity = 51.52%
	2		gi|78776875|ref|YP_393190.1|	Sulfuromonas denitrificans ATCC 33889	E-value = 0	Identity = 50.72%
	3		gi|152990170|ref|YP_001355892.1|	Nitratiruptor sp. SB155-2	E-value = 0	Identity = 52.30%
	4		gi|157164914|ref|YP_001466377.1|	Campylobacter concisus 13826	E-value = 0	Identity = 48.53%
	5		gi|154174465|ref|YP_001408785.1|	Campylobacter curvus 525.92	E-value = 0	Identity = 46.88%
AB2033	1	*rbfA*	gi|34558304|ref|NP_908119.1|	Wolinella succinogenes DSM 1740	E-value = 4.00E-21	Identity = 45.69%
	2		gi|154173886|ref|YP_001408784.1|	Campylobacter curvus 525.92	E-value = 5.00E-20	Identity = 43.64%
	3		gi|153951532|ref|YP_001397396.1|	Campylobacter jejuni subsp. doylei 269.97	E-value = 2.00E-19	Identity = 45.87%
	4		gi|121612306|ref|YP_999861.1|	Campylobacter jejuni subsp. jejuni 81-176	E-value = 2.00E-19	Identity = 45.87%
	5		gi|15791525|ref|NP_281348.1|	Campylobacter jejuni subsp. jejuni NCTC 11168	E-value = 2.00E-19	Identity = 45.87%
AB2034	1		gi|152993548|ref|YP_001359269.1|	Sulfurovum sp. NBC37-1	E-value = 1.00E-33	Identity = 55.63%
	2		gi|152990172|ref|YP_001355894.1|	Nitratiruptor sp. SB155-2	E-value = 8.00E-31	Identity = 51.41%
	3		gi|157164808|ref|YP_001466379.1|	Campylobacter concisus 13826	E-value = 2.00E-26	Identity = 51.52%
	4		gi|154174423|ref|YP_001408783.1|	Campylobacter curvus 525.92	E-value = 5.00E-25	Identity = 47.73%
	5		gi|34558305|ref|NP_908120.1|	Wolinella succinogenes DSM 1740	E-value = 5.00E-25	Identity = 43.97%
AB2035	1	*ribD*	gi|149194180|ref|ZP_01871278.1|	Caminibacter mediatlanticus TB-2	E-value = 3.00E-85	Identity = 54.74%
	2		gi|152990173|ref|YP_001355895.1|	Nitratiruptor sp. SB155-2	E-value = 4.00E-84	Identity = 44.34%
	3		gi|78776878|ref|YP_393193.1|	Sulfuromonas denitrificans ATCC 33889	E-value = 4.00E-84	Identity = 47.92%
	4		gi|152993547|ref|YP_001359268.1|	Sulfurovum sp. NBC37-1	E-value = 4.00E-75	Identity = 43.98%

	5		gi|148925766|ref|ZP_01809454.1|	Campylobacter jejuni subsp. jejuni CG8486	E-value = 2.00E-73	Identity = 46.15%
AB2036	1	*efp*	gi|32265607|ref|NP_859639.1|	Helicobacter hepaticus ATCC 51449	E-value = 5.00E-74	Identity = 74.87%
	2		gi|154175266|ref|YP_001407861.1|	Campylobacter curvus 525.92	E-value = 4.00E-73	Identity = 72.83%
	3		gi|157164695|ref|YP_001466442.1|	Campylobacter concisus 13826	E-value = 1.00E-72	Identity = 73.33%
	4		gi|34557643|ref|NP_907458.1|	Wolinella succinogenes DSM 1740	E-value = 1.00E-69	Identity = 67.38%
	5		gi|118474233|ref|YP_892403.1|	Campylobacter fetus subsp. fetus 82-40	E-value = 2.00E-69	Identity = 71.82%
AB2037	1	*serA*	gi|152990413|ref|YP_001356135.1|	Nitratiruptor sp. SB155-2	E-value = 0	Identity = 65.09%
	2		gi|34558837|gb|AAQ75181.1|	Alvinella pompejana epibiont 7G3	E-value = 0	Identity = 62.19%
	3		gi|152992147|ref|YP_001357868.1|	Sulfurovum sp. NBC37-1	E-value = 0	Identity = 64.08%
	4		gi|34557674|ref|NP_907489.1|	Wolinella succinogenes DSM 1740	E-value = 0	Identity = 62.33%
	5		gi|157165087|ref|YP_001466441.1|	Campylobacter concisus 13826	E-value = 0	Identity = 61.64%
AB2038	1	*rpsA*	gi|34557672|ref|NP_907487.1|	Wolinella succinogenes DSM 1740	E-value = 3.00E-154	Identity = 55.70%
	2		gi|157164057|ref|YP_001466439.1|	Campylobacter concisus 13826	E-value = 5.00E-150	Identity = 56.33%
	3		gi|118475172|ref|YP_892406.1|	Campylobacter fetus subsp. fetus 82-40	E-value = 1.00E-148	Identity = 56.39%
	4		gi|154175267|ref|YP_001407858.1|	Campylobacter curvus 525.92	E-value = 7.00E-148	Identity = 57.17%
	5		gi|154147885|ref|YP_001406069.1|	Campylobacter hominis ATCC BAA-381	E-value = 4.00E-144	Identity = 54.81%
AB2039	1	*ispH*	gi|34558835|gb|AAQ75179.1|	Alvinella pompejana epibiont 7G3	E-value = 3.00E-101	Identity = 67.51%
	2		gi|152992144|ref|YP_001357865.1|	Sulfurovum sp. NBC37-1	E-value = 5.00E-100	Identity = 66.43%
	3		gi|152990411|ref|YP_001356133.1|	Nitratiruptor sp. SB155-2	E-value = 8.00E-93	Identity = 64.26%
	4		gi|157164476|ref|YP_001466438.1|	Campylobacter concisus 13826	E-value = 3.00E-89	Identity = 61.01%
	5		gi|154173692|ref|YP_001407857.1|	Campylobacter curvus 525.92	E-value = 1.00E-86	Identity = 59.27%
AB2040	1	*aroA*	gi|34558834|gb|AAQ75178.1|	Alvinella pompejana epibiont 7G3	E-value = 5.00E-144	Identity = 62.09%
	2		gi|152990410|ref|YP_001356132.1|	Nitratiruptor sp. SB155-2	E-value = 3.00E-141	Identity = 63.37%
	3		gi|78777069|ref|YP_393384.1|	Sulfuromonas denitrificans ATCC 33889	E-value = 9.00E-130	Identity = 60.53%
	4		gi|34557670|ref|NP_907485.1|	Wolinella succinogenes DSM 1740	E-value = 1.00E-128	Identity = 59.25%
	5		gi|154174161|ref|YP_001407856.1|	Campylobacter curvus 525.92	E-value = 2.00E-128	Identity = 57.93%
AB2041	1	*pheT*	gi|152992143|ref|YP_001357864.1|	Sulfurovum sp. NBC37-1	E-value = 4.00E-177	Identity = 44.03%
	2		gi|78777068|ref|YP_393383.1|	Sulfuromonas denitrificans ATCC 33889	E-value = 5.00E-171	Identity = 43.85%
	3		gi|34558833|gb|AAQ75177.1|	Alvinella pompejana epibiont 7G3	E-value = 1.00E-170	Identity = 44.29%
	4		gi|152990409|ref|YP_001356131.1|	Nitratiruptor sp. SB155-2	E-value = 9.00E-167	Identity = 42.97%
	5		gi|34557669|ref|NP_907484.1|	Wolinella succinogenes DSM 1740	E-value = 6.00E-162	Identity = 42.95%
AB2042	1	*pheS*	gi|152992142|ref|YP_001357863.1|	Sulfurovum sp. NBC37-1	E-value = 2.00E-134	Identity = 70.68%
	2		gi|118475664|ref|YP_892410.1|	Campylobacter fetus subsp. fetus 82-40	E-value = 1.00E-133	Identity = 67.27%
	3		gi|154174528|ref|YP_001407854.1|	Campylobacter curvus 525.92	E-value = 3.00E-132	Identity = 66.97%
	4		gi|78777067|ref|YP_393382.1|	Sulfuromonas denitrificans ATCC 33889	E-value = 4.00E-130	Identity = 66.06%
	5		gi|157165614|ref|YP_001466437.1|	Campylobacter concisus 13826	E-value = 1.00E-128	Identity = 67.07%
AB2043	1		gi|34558830|gb|AAQ75174.1|	Alvinella pompejana epibiont 7G3	E-value = 2.00E-33	Identity = 63.55%
	2		gi|152992141|ref|YP_001357862.1|	Sulfurovum sp. NBC37-1	E-value = 2.00E-31	Identity = 64.36%
	3		gi|152990407|ref|YP_001356129.1|	Nitratiruptor sp. SB155-2	E-value = 5.00E-31	Identity = 57.02%
	4		gi|118474817|ref|YP_892411.1|	Campylobacter fetus subsp. fetus 82-40	E-value = 1.00E-29	Identity = 58.00%
	5		gi|78777066|ref|YP_393381.1|	Sulfuromonas denitrificans ATCC 33889	E-value = 5.00E-28	Identity = 57.84%
AB2044	1	*accA*	gi|152990065|ref|YP_001355787.1|	Nitratiruptor sp. SB155-2	E-value = 2.00E-124	Identity = 71.06%
	2		gi|78777805|ref|YP_394120.1|	Sulfuromonas denitrificans ATCC 33889	E-value = 2.00E-120	Identity = 66.56%
	3		gi|32266225|ref|NP_860257.1|	Helicobacter hepaticus ATCC 51449	E-value = 4.00E-117	Identity = 68.06%
	4		gi|157165124|ref|YP_001467393.1|	Campylobacter concisus 13826	E-value = 5.00E-117	Identity = 66.99%
	5		gi|34557044|ref|NP_906859.1|	Wolinella succinogenes DSM 1740	E-value = 7.00E-116	Identity = 66.56%
AB2045	1	*fabF*	gi|152993741|ref|YP_001359462.1|	Sulfurovum sp. NBC37-1	E-value = 1.00E-151	Identity = 67.39%
	2		gi|152990064|ref|YP_001355786.1|	Nitratiruptor sp. SB155-2	E-value = 2.00E-151	Identity = 68.42%
	3		gi|78777806|ref|YP_394121.1|	Sulfuromonas denitrificans ATCC 33889	E-value = 2.00E-149	Identity = 68.03%

	4		gi|157414737|ref|YP_001481993.1|	Campylobacter jejuni subsp. jejuni 81116	E-value = 1.00E-146	Identity = 64.90%
	5		gi|57237499|ref|YP_178513.1|	Campylobacter jejuni subsp. jejuni RM1221	E-value = 2.00E-146	Identity = 64.90%
AB2046	1	*acpP*	gi|78777807|ref|YP_394122.1|	Sulfuromonas denitrificans ATCC 33889	E-value = 3.00E-15	Identity = 79.73%
	2		gi|34557046|ref|NP_906861.1|	Wolinella succinogenes DSM 1740	E-value = 2.00E-14	Identity = 76.00%
	3		gi|152993742|ref|YP_001359463.1|	Sulfurovum sp. NBC37-1	E-value = 1.00E-13	Identity = 82.43%
	4		gi|113954189|ref|YP_729369.1|	Synechococcus sp. CC9311	E-value = 3.00E-13	Identity = 65.75%
	5		gi|109947341|ref|YP_664569.1|	Helicobacter acinonychis str. Sheeba	E-value = 2.00E-12	Identity = 65.33%
AB2047	1	*fabG*	gi|157164484|ref|YP_001467396.1|	Campylobacter concisus 13826	E-value = 9.00E-93	Identity = 72.87%
	2		gi|57242189|ref|ZP_00370128.1|	Campylobacter upsaliensis RM3195	E-value = 3.00E-92	Identity = 69.64%
	3		gi|154149516|ref|YP_001405837.1|	Campylobacter hominis ATCC BAA-381	E-value = 9.00E-92	Identity = 71.66%
	4		gi|152990062|ref|YP_001355784.1|	Nitratiruptor sp. SB155-2	E-value = 1.00E-91	Identity = 74.49%
	5		gi|57240802|ref|ZP_00368750.1|	Campylobacter lari RM2100	E-value = 2.00E-91	Identity = 73.68%
AB2048	1		gi|152991808|ref|YP_001357529.1|	Sulfurovum sp. NBC37-1	E-value = 7.00E-46	Identity = 40.49%
	2		gi|152991323|ref|YP_001357045.1|	Nitratiruptor sp. SB155-2	E-value = 2.00E-45	Identity = 41.46%
	3		gi|15611935|ref|NP_223586.1|	Helicobacter pylori J99	E-value = 2.00E-41	Identity = 41.06%
	4		gi|108563339|ref|YP_627655.1|	Helicobacter pylori HPAG1	E-value = 4.00E-41	Identity = 40.24%
	5		gi|57241379|ref|ZP_00369325.1|	Campylobacter lari RM2100	E-value = 5.00E-41	Identity = 45.14%
AB2049	1		gi|34557974|ref|NP_907789.1|	Wolinella succinogenes DSM 1740	E-value = 4.00E-59	Identity = 61.02%
	2		gi|15605809|ref|NP_213186.1|	Aquifex aeolicus VF5	E-value = 6.00E-13	Identity = 32.79%
	3		gi|156719953|ref|ZP_02061549.1|	Hydrogenobaculum sp. Y04AAS1	E-value = 5.00E-12	Identity = 35.56%
	4		gi|83593166|ref|YP_426918.1|	Rhodospirillum rubrum ATCC 11170	E-value = 3.00E-06	Identity = 28.02%
AB2050	1	*exsB*	gi|152992854|ref|YP_001358575.1|	Sulfurovum sp. NBC37-1	E-value = 1.00E-86	Identity = 70.00%
	2		gi|149194505|ref|ZP_01871601.1|	Caminibacter mediatlanticus TB-2	E-value = 2.00E-86	Identity = 68.64%
	3		gi|152990536|ref|YP_001356258.1|	Nitratiruptor sp. SB155-2	E-value = 1.00E-82	Identity = 67.89%
	4		gi|78777268|ref|YP_393583.1|	Sulfuromonas denitrificans ATCC 33889	E-value = 7.00E-82	Identity = 66.52%
	5		gi|154174066|ref|YP_001407913.1|	Campylobacter curvus 525.92	E-value = 1.00E-76	Identity = 62.84%
AB2051	1		gi|34558435|ref|NP_908250.1|	Wolinella succinogenes DSM 1740	E-value = 2.00E-08	Identity = 29.57%
AB2052	1		gi|152991814|ref|YP_001357535.1|	Sulfurovum sp. NBC37-1	E-value = 8.00E-50	Identity = 46.22%
	2		gi|152991316|ref|YP_001357038.1|	Nitratiruptor sp. SB155-2	E-value = 1.00E-48	Identity = 46.19%
	3		gi|78778105|ref|YP_394420.1|	Sulfuromonas denitrificans ATCC 33889	E-value = 1.00E-43	Identity = 42.41%
	4		gi|118475053|ref|YP_891431.1|	Campylobacter fetus subsp. fetus 82-40	E-value = 6.00E-39	Identity = 44.39%
	5		gi|149195029|ref|ZP_01872121.1|	Caminibacter mediatlanticus TB-2	E-value = 6.00E-38	Identity = 48.43%
AB2053	1	*petC*	gi|154175339|ref|YP_001407598.1|	Campylobacter curvus 525.92	E-value = 2.00E-82	Identity = 44.63%
	2		gi|157164244|ref|YP_001466161.1|	Campylobacter concisus 13826	E-value = 4.00E-81	Identity = 45.33%
	3		gi|118474682|ref|YP_892589.1|	Campylobacter fetus subsp. fetus 82-40	E-value = 6.00E-80	Identity = 46.29%
	4		gi|34558433|ref|NP_908248.1|	Wolinella succinogenes DSM 1740	E-value = 4.00E-78	Identity = 44.87%
	5		gi|57505419|ref|ZP_00371347.1|	Campylobacter upsaliensis RM3195	E-value = 1.00E-73	Identity = 42.60%
AB2054	1	*petB*	gi|152991326|ref|YP_001357048.1|	Nitratiruptor sp. SB155-2	E-value = 2.00E-158	Identity = 73.65%
	2		gi|34558432|ref|NP_908247.1|	Wolinella succinogenes DSM 1740	E-value = 3.00E-158	Identity = 70.77%
	3		gi|57240507|ref|ZP_00368456.1|	Campylobacter lari RM2100	E-value = 1.00E-157	Identity = 72.15%
	4		gi|57505418|ref|ZP_00371346.1|	Campylobacter upsaliensis RM3195	E-value = 3.00E-156	Identity = 72.09%
	5		gi|153952423|ref|YP_001397719.1|	Campylobacter jejuni subsp. doylei 269.97	E-value = 1.00E-155	Identity = 72.70%
AB2055	1	*petA*	gi|57505417|ref|ZP_00371345.1|	Campylobacter upsaliensis RM3195	E-value = 6.00E-48	Identity = 63.64%
	2		gi|118475313|ref|YP_892587.1|	Campylobacter fetus subsp. fetus 82-40	E-value = 2.00E-47	Identity = 63.98%
	3		gi|57240508|ref|ZP_00368457.1|	Campylobacter lari RM2100	E-value = 5.00E-47	Identity = 61.21%
	4		gi|15792510|ref|NP_282333.1|	Campylobacter jejuni subsp. jejuni NCTC 11168	E-value = 2.00E-46	Identity = 59.39%
	5		gi|57168065|ref|ZP_00367204.1|	Campylobacter coli RM2228	E-value = 3.00E-46	Identity = 59.51%
AB2056	1	*thrC*	gi|152993210|ref|YP_001358931.1|	Sulfurovum sp. NBC37-1	E-value = 8.00E-173	Identity = 62.24%
	2		gi|78776988|ref|YP_393303.1|	Sulfuromonas denitrificans ATCC 33889	E-value = 4.00E-169	Identity = 62.73%

	3		gi|152990785|ref|YP_001356507.1|	Nitratiruptor sp. SB155-2	E-value = 4.00E-168	Identity = 62.32%
	4		gi|78485593|ref|YP_391518.1|	Thiomicrospira crunogena XCL-2	E-value = 1.00E-164	Identity = 58.25%
	5		gi|34558393|ref|NP_908208.1|	Wolinella succinogenes DSM 1740	E-value = 1.00E-142	Identity = 55.71%
AB2057	1	*argB*	gi|34558420|ref|NP_908235.1|	Wolinella succinogenes DSM 1740	E-value = 4.00E-96	Identity = 62.54%
	2		gi|152990787|ref|YP_001356509.1|	Nitratiruptor sp. SB155-2	E-value = 2.00E-95	Identity = 65.45%
	3		gi|78777052|ref|YP_393367.1|	Sulfuromonas denitrificans ATCC 33889	E-value = 5.00E-95	Identity = 65.36%
	4		gi|152993214|ref|YP_001358935.1|	Sulfurovum sp. NBC37-1	E-value = 2.00E-89	Identity = 62.87%
	5		gi|32265678|ref|NP_859710.1|	Helicobacter hepaticus ATCC 51449	E-value = 5.00E-87	Identity = 56.78%
AB2058	1		gi|34557616|ref|NP_907431.1|	Wolinella succinogenes DSM 1740	E-value = 7.00E-131	Identity = 35.28%
	2		gi|78776291|ref|YP_392606.1|	Sulfuromonas denitrificans ATCC 33889	E-value = 1.00E-126	Identity = 37.61%
	3		gi|152991783|ref|YP_001357504.1|	Sulfurovum sp. NBC37-1	E-value = 2.00E-123	Identity = 35.29%
	4		gi|152990115|ref|YP_001355837.1|	Nitratiruptor sp. SB155-2	E-value = 2.00E-112	Identity = 35.09%
	5		gi|154174808|ref|YP_001407581.1|	Campylobacter curvus 525.92	E-value = 2.00E-110	Identity = 32.46%
AB2059	1			***** No matches found *****		
AB2060	1	*prfB*	gi|152991276|ref|YP_001356998.1|	Nitratiruptor sp. SB155-2	E-value = 2.00E-142	Identity = 75.07%
	2		gi|152991913|ref|YP_001357634.1|	Sulfurovum sp. NBC37-1	E-value = 9.00E-134	Identity = 72.30%
	3		gi|78776561|ref|YP_392876.1|	Sulfuromonas denitrificans ATCC 33889	E-value = 6.00E-132	Identity = 68.87%
	4		gi|34556562|ref|NP_906377.1|	Wolinella succinogenes DSM 1740	E-value = 9.00E-132	Identity = 69.81%
	5		gi|118475480|ref|YP_892510.1|	Campylobacter fetus subsp. fetus 82-40	E-value = 4.00E-131	Identity = 67.67%
AB2061	1		gi|24373301|ref|NP_717344.1|	Shewanella oneidensis MR-1	E-value = 1.00E-36	Identity = 57.94%
	2		gi|117921162|ref|YP_870354.1|	Shewanella sp. ANA-3	E-value = 1.00E-36	Identity = 57.94%
	3		gi|113970889|ref|YP_734682.1|	Shewanella sp. MR-4	E-value = 1.00E-36	Identity = 57.94%
	4		gi|126173778|ref|YP_001049927.1|	Shewanella baltica OS155	E-value = 2.00E-36	Identity = 57.14%
	5		gi|146292539|ref|YP_001182963.1|	Shewanella putrefaciens CN-32	E-value = 2.00E-36	Identity = 59.52%
AB2062	1		gi|78776289|ref|YP_392604.1|	Sulfuromonas denitrificans ATCC 33889	E-value = 9.00E-102	Identity = 34.60%
	2		gi|152991781|ref|YP_001357502.1|	Sulfurovum sp. NBC37-1	E-value = 3.00E-90	Identity = 30.43%
	3		gi|118475773|ref|YP_891587.1|	Campylobacter fetus subsp. fetus 82-40	E-value = 1.00E-78	Identity = 31.50%
	4		gi|157164411|ref|YP_001466191.1|	Campylobacter concisus 13826	E-value = 5.00E-77	Identity = 30.35%
	5		gi|34556981|ref|NP_906796.1|	Wolinella succinogenes DSM 1740	E-value = 7.00E-76	Identity = 29.56%
AB2063	1			***** No matches found *****		
AB2064	1		gi|154148411|ref|YP_001406943.1|	Campylobacter hominis ATCC BAA-381	E-value = 3.00E-19	Identity = 28.44%
	2		gi|154173773|ref|YP_001407578.1|	Campylobacter curvus 525.92	E-value = 1.00E-17	Identity = 31.00%
	3		gi|152991353|ref|YP_001357075.1|	Nitratiruptor sp. SB155-2	E-value = 8.00E-17	Identity = 33.99%
	4		gi|157164464|ref|YP_001466185.1|	Campylobacter concisus 13826	E-value = 1.00E-16	Identity = 31.03%
	5		gi|34556630|ref|NP_906445.1|	Wolinella succinogenes DSM 1740	E-value = 1.00E-13	Identity = 25.41%
AB2065	1			***** No matches found *****		
AB2066	1			***** No matches found *****		
AB2067	1	*ccoP*	gi|152991777|ref|YP_001357498.1|	Sulfurovum sp. NBC37-1	E-value = 9.00E-83	Identity = 51.19%
	2		gi|78776285|ref|YP_392600.1|	Sulfuromonas denitrificans ATCC 33889	E-value = 2.00E-71	Identity = 44.66%
	3		gi|152991356|ref|YP_001357078.1|	Nitratiruptor sp. SB155-2	E-value = 5.00E-71	Identity = 51.57%
	4		gi|157164062|ref|YP_001466183.1|	Campylobacter concisus 13826	E-value = 3.00E-62	Identity = 45.42%
	5		gi|34556628|ref|NP_906443.1|	Wolinella succinogenes DSM 1740	E-value = 3.00E-62	Identity = 43.97%
AB2068	1	*ccoQ*		***** No matches found *****		
AB2069	1	*ccoO*	gi|57240906|ref|ZP_00368854.1|	Campylobacter lari RM2100	E-value = 4.00E-97	Identity = 73.76%
	2		gi|57169073|ref|ZP_00368200.1|	Campylobacter coli RM2228	E-value = 3.00E-96	Identity = 74.66%
	3		gi|152991775|ref|YP_001357496.1|	Sulfurovum sp. NBC37-1	E-value = 7.00E-96	Identity = 75.00%
	4		gi|57242569|ref|ZP_00370507.1|	Campylobacter upsaliensis RM3195	E-value = 1.00E-95	Identity = 74.21%
	5		gi|34556626|ref|NP_906441.1|	Wolinella succinogenes DSM 1740	E-value = 3.00E-92	Identity = 72.73%
AB2070	1	*ccoN*	gi|153951418|ref|YP_001398790.1|	Campylobacter jejuni subsp. doylei 269.97	E-value = 0	Identity = 79.10%

	2		gi|57240905|ref|ZP_00368853.1|	Campylobacter lari RM2100	E-value = 0	Identity = 78.48%
	3		gi|118475616|ref|YP_891580.1|	Campylobacter fetus subsp. fetus 82-40	E-value = 0	Identity = 77.71%
	4		gi|57169074|ref|ZP_00368201.1|	Campylobacter coli RM2228	E-value = 0	Identity = 77.87%
	5		gi|148925657|ref|ZP_01809345.1|	Campylobacter jejuni subsp. jejuni CG8486	E-value = 0	Identity = 78.89%
AB2071	1	*smpB*	gi|154174845|ref|YP_001408538.1|	Campylobacter curvus 525.92	E-value = 3.00E-43	Identity = 69.44%
	2		gi|118474780|ref|YP_891887.1|	Campylobacter fetus subsp. fetus 82-40	E-value = 7.00E-42	Identity = 64.58%
	3		gi|34557274|ref|NP_907089.1|	Wolinella succinogenes DSM 1740	E-value = 2.00E-39	Identity = 63.95%
	4		gi|78776642|ref|YP_392957.1|	Sulfuromonas denitrificans ATCC 33889	E-value = 1.00E-38	Identity = 60.96%
	5		gi|32265620|ref|NP_859652.1|	Helicobacter hepaticus ATCC 51449	E-value = 6.00E-38	Identity = 57.82%
AB2072	1	*ispE*	gi|78776641|ref|YP_392956.1|	Sulfuromonas denitrificans ATCC 33889	E-value = 2.00E-64	Identity = 51.18%
	2		gi|152991217|ref|YP_001356939.1|	Nitratiruptor sp. SB155-2	E-value = 5.00E-56	Identity = 45.63%
	3		gi|152991977|ref|YP_001357698.1|	Sulfurovum sp. NBC37-1	E-value = 6.00E-53	Identity = 44.80%
	4		gi|154174184|ref|YP_001408537.1|	Campylobacter curvus 525.92	E-value = 2.00E-48	Identity = 43.60%
	5		gi|149194065|ref|ZP_01871163.1|	Caminibacter mediatlanticus TB-2	E-value = 3.00E-46	Identity = 41.67%
AB2073	1	*truB*	gi|152991979|ref|YP_001357700.1|	Sulfurovum sp. NBC37-1	E-value = 2.00E-80	Identity = 55.68%
	2		gi|118474138|ref|YP_891890.1|	Campylobacter fetus subsp. fetus 82-40	E-value = 1.00E-71	Identity = 53.16%
	3		gi|152991216|ref|YP_001356938.1|	Nitratiruptor sp. SB155-2	E-value = 1.00E-68	Identity = 51.10%
	4		gi|78776639|ref|YP_392954.1|	Sulfuromonas denitrificans ATCC 33889	E-value = 8.00E-66	Identity = 47.97%
	5		gi|57167989|ref|ZP_00367128.1|	Campylobacter coli RM2228	E-value = 2.00E-65	Identity = 52.94%
AB2074	1		gi|152991215|ref|YP_001356937.1|	Nitratiruptor sp. SB155-2	E-value = 0	Identity = 61.88%
	2		gi|152991980|ref|YP_001357701.1|	Sulfurovum sp. NBC37-1	E-value = 0	Identity = 59.48%
	3		gi|154174762|ref|YP_001408534.1|	Campylobacter curvus 525.92	E-value = 0	Identity = 60.64%
	4		gi|157164973|ref|YP_001467057.1|	Campylobacter concisus 13826	E-value = 0	Identity = 60.20%
	5		gi|78776638|ref|YP_392953.1|	Sulfuromonas denitrificans ATCC 33889	E-value = 0	Identity = 58.65%
AB2075	1		gi|78776637|ref|YP_392952.1|	Sulfuromonas denitrificans ATCC 33889	E-value = 1.00E-81	Identity = 54.70%
	2		gi|152991214|ref|YP_001356936.1|	Nitratiruptor sp. SB155-2	E-value = 5.00E-79	Identity = 52.33%
	3		gi|34557268|ref|NP_907083.1|	Wolinella succinogenes DSM 1740	E-value = 1.00E-77	Identity = 53.18%
	4		gi|152991981|ref|YP_001357702.1|	Sulfurovum sp. NBC37-1	E-value = 1.00E-74	Identity = 50.84%
	5		gi|149194061|ref|ZP_01871159.1|	Caminibacter mediatlanticus TB-2	E-value = 2.00E-39	Identity = 35.05%
AB2076	1		gi|78778066|ref|YP_394381.1|	Sulfuromonas denitrificans ATCC 33889	E-value = 1.00E-109	Identity = 62.18%
	2		gi|152991288|ref|YP_001357010.1|	Nitratiruptor sp. SB155-2	E-value = 5.00E-107	Identity = 57.10%
	3		gi|34556884|ref|NP_906699.1|	Wolinella succinogenes DSM 1740	E-value = 1.00E-99	Identity = 56.19%
	4		gi|149194160|ref|ZP_01871258.1|	Caminibacter mediatlanticus TB-2	E-value = 2.00E-96	Identity = 61.99%
	5		gi|32265988|ref|NP_860020.1|	Helicobacter hepaticus ATCC 51449	E-value = 1.00E-95	Identity = 51.88%
AB2077	1	*purF*	gi|152991832|ref|YP_001357553.1|	Sulfurovum sp. NBC37-1	E-value = 1.00E-176	Identity = 66.97%
	2		gi|118475248|ref|YP_891501.1|	Campylobacter fetus subsp. fetus 82-40	E-value = 2.00E-175	Identity = 67.71%
	3		gi|157164900|ref|YP_001466027.1|	Campylobacter concisus 13826	E-value = 1.00E-172	Identity = 66.74%
	4		gi|154174756|ref|YP_001407520.1|	Campylobacter curvus 525.92	E-value = 3.00E-171	Identity = 67.41%
	5		gi|154147906|ref|YP_001406908.1|	Campylobacter hominis ATCC BAA-381	E-value = 3.00E-169	Identity = 64.81%
AB2078	1	*dapB*	gi|149194158|ref|ZP_01871256.1|	Caminibacter mediatlanticus TB-2	E-value = 2.00E-85	Identity = 63.67%
	2		gi|152991831|ref|YP_001357552.1|	Sulfurovum sp. NBC37-1	E-value = 4.00E-85	Identity = 62.65%
	3		gi|152991290|ref|YP_001357012.1|	Nitratiruptor sp. SB155-2	E-value = 5.00E-84	Identity = 63.04%
	4		gi|78778064|ref|YP_394379.1|	Sulfuromonas denitrificans ATCC 33889	E-value = 5.00E-83	Identity = 63.92%
	5		gi|34556882|ref|NP_906697.1|	Wolinella succinogenes DSM 1740	E-value = 6.00E-82	Identity = 59.92%
AB2079	1	*trxB*	gi|78778063|ref|YP_394378.1|	Sulfuromonas denitrificans ATCC 33889	E-value = 1.00E-117	Identity = 68.18%
	2		gi|157414463|ref|YP_001481719.1|	Campylobacter jejuni subsp. jejuni 81116	E-value = 1.00E-112	Identity = 66.99%
	3		gi|86151191|ref|ZP_01069406.1|	Campylobacter jejuni subsp. jejuni 260.94	E-value = 2.00E-112	Identity = 66.67%
	4		gi|86153593|ref|ZP_01071796.1|	Campylobacter jejuni subsp. jejuni HB93-13	E-value = 2.00E-112	Identity = 66.67%
	5		gi|15611831|ref|NP_223482.1|	Helicobacter pylori J99	E-value = 2.00E-112	Identity = 65.26%

AB2080	1	*trxA2*	gi|34556879|ref|NP_906694.1|	Wolinella succinogenes DSM 1740	E-value = 4.00E-46	Identity = 80.00%
	2		gi|78778061|ref|YP_394376.1|	Sulfuromonas denitrificans ATCC 33889	E-value = 9.00E-42	Identity = 76.19%
	3		gi|157165021|ref|YP_001466032.1|	Campylobacter concisus 13826	E-value = 1.00E-41	Identity = 76.19%
	4		gi|154174395|ref|YP_001407510.1|	Campylobacter curvus 525.92	E-value = 3.00E-41	Identity = 76.19%
	5		gi|154149269|ref|YP_001405822.1|	Campylobacter hominis ATCC BAA-381	E-value = 4.00E-41	Identity = 74.29%
AB2081	1	*alaS*	gi|152991270|ref|YP_001356992.1|	Nitratiruptor sp. SB155-2	E-value = 0	Identity = 62.30%
	2		gi|78778092|ref|YP_394407.1|	Sulfuromonas denitrificans ATCC 33889	E-value = 0	Identity = 62.24%
	3		gi|152991942|ref|YP_001357663.1|	Sulfurovum sp. NBC37-1	E-value = 0	Identity = 61.77%
	4		gi|154174082|ref|YP_001408546.1|	Campylobacter curvus 525.92	E-value = 0	Identity = 60.56%
	5		gi|57240482|ref|ZP_00368431.1|	Campylobacter lari RM2100	E-value = 0	Identity = 60.38%
AB2082	1		gi|28897148|ref|NP_796753.1|	Vibrio parahaemolyticus RIMD 2210633	E-value = 7.00E-59	Identity = 49.37%
	2		gi|151937737|gb|EDN56587.1|	Vibrio sp. Ex25	E-value = 1.00E-58	Identity = 47.70%
	3		gi|156973158|ref|YP_001444065.1|	Vibrio harveyi ATCC BAA-1116	E-value = 5.00E-58	Identity = 48.54%
	4		gi|153834236|ref|ZP_01986903.1|	Vibrio harveyi HY01	E-value = 1.00E-57	Identity = 48.54%
	5		gi|109898727|ref|YP_661982.1|	Pseudoalteromonas atlantica T6c	E-value = 3.00E-57	Identity = 47.84%
AB2083	1		gi|34557718|ref|NP_907533.1|	Wolinella succinogenes DSM 1740	E-value = 5.00E-65	Identity = 39.41%
	2		gi|114319908|ref|YP_741591.1|	Alkalilimnicola ehrlichei MLHE-1	E-value = 1.00E-54	Identity = 38.14%
	3		gi|118474173|ref|YP_891770.1|	Campylobacter fetus subsp. fetus 82-40	E-value = 4.00E-51	Identity = 34.50%
	4		gi|154149505|ref|YP_001406398.1|	Campylobacter hominis ATCC BAA-381	E-value = 9.00E-50	Identity = 34.14%
	5		gi|121999073|ref|YP_001003860.1|	Halorhodospira halophila SL1	E-value = 1.00E-48	Identity = 33.61%
AB2084	1			***** No matches found *****		
AB2085	1		gi|152993177|ref|YP_001358898.1|	Sulfurovum sp. NBC37-1	E-value = 2.00E-41	Identity = 60.43%
	2		gi|78776314|ref|YP_392629.1|	Sulfuromonas denitrificans ATCC 33889	E-value = 1.00E-34	Identity = 56.03%
AB2086	1		gi|152993670|ref|YP_001359391.1|	Sulfurovum sp. NBC37-1	E-value = 1.00E-72	Identity = 42.72%
	2		gi|157163896|ref|YP_001466683.1|	Campylobacter concisus 13826	E-value = 2.00E-49	Identity = 33.08%
	3		gi|152991456|ref|YP_001357178.1|	Nitratiruptor sp. SB155-2	E-value = 2.00E-49	Identity = 33.99%
	4		gi|154174937|ref|YP_001408398.1|	Campylobacter curvus 525.92	E-value = 9.00E-49	Identity = 33.17%
	5		gi|118474309|ref|YP_891937.1|	Campylobacter fetus subsp. fetus 82-40	E-value = 2.00E-47	Identity = 33.25%
AB2087	1		gi|152993671|ref|YP_001359392.1|	Sulfurovum sp. NBC37-1	E-value = 5.00E-56	Identity = 59.82%
	2		gi|157164458|ref|YP_001466682.1|	Campylobacter concisus 13826	E-value = 7.00E-53	Identity = 54.09%
	3		gi|154174385|ref|YP_001408399.1|	Campylobacter curvus 525.92	E-value = 1.00E-52	Identity = 54.05%
	4		gi|118474546|ref|YP_891938.1|	Campylobacter fetus subsp. fetus 82-40	E-value = 3.00E-51	Identity = 54.55%
	5		gi|154149383|ref|YP_001406539.1|	Campylobacter hominis ATCC BAA-381	E-value = 8.00E-50	Identity = 51.80%
AB2088	1	*htrA*	gi|152991454|ref|YP_001357176.1|	Nitratiruptor sp. SB155-2	E-value = 5.00E-116	Identity = 50.95%
	2		gi|15611473|ref|NP_223124.1|	Helicobacter pylori J99	E-value = 4.00E-112	Identity = 49.68%
	3		gi|108562853|ref|YP_627169.1|	Helicobacter pylori HPAG1	E-value = 6.00E-112	Identity = 50.11%
	4		gi|34558499|ref|NP_908314.1|	Wolinella succinogenes DSM 1740	E-value = 1.00E-111	Identity = 48.12%
	5		gi|109947655|ref|YP_664883.1|	Helicobacter acinonychis str. Sheeba	E-value = 2.00E-110	Identity = 49.46%
AB2089	1	*ilvD*	gi|146300963|ref|YP_001195554.1|	Flavobacterium johnsoniae UW101	E-value = 0	Identity = 87.72%
	2		gi|152993997|ref|YP_001359718.1|	Sulfurovum sp. NBC37-1	E-value = 0	Identity = 82.74%
	3		gi|149194931|ref|ZP_01872024.1|	Caminibacter mediatlanticus TB-2	E-value = 0	Identity = 76.16%
	4		gi|152989876|ref|YP_001355598.1|	Nitratiruptor sp. SB155-2	E-value = 0	Identity = 76.91%
	5		gi|157165359|ref|YP_001467789.1|	Campylobacter concisus 13826	E-value = 0	Identity = 75.36%
AB2090	1	*int*	gi|119357428|ref|YP_912072.1|	Chlorobium phaeobacteroides DSM 266	E-value = 1.00E-51	Identity = 31.19%
	2		gi|85859572|ref|YP_461774.1|	Syntrophus aciditrophicus SB	E-value = 7.00E-48	Identity = 30.79%
	3		gi|118745460|ref|ZP_01593434.1|	Geobacter lovleyi SZ	E-value = 5.00E-47	Identity = 29.76%
	4		gi|118707336|ref|ZP_01559922.1|	Burkholderia cenocepacia MC0-3	E-value = 2.00E-46	Identity = 30.05%
	5		gi|83718523|ref|YP_441483.1|	Burkholderia thailandensis E264	E-value = 4.00E-46	Identity = 28.64%
AB2091	1		gi|14194406|gb|AAK56382.1|AF378539_1	unidentified bacterium	E-value = 1.00E-10	Identity = 39.39%

AB2092	1			***** No matches found *****		
AB2093	1			***** No matches found *****		
AB2094	1		gi|118475596|ref|YP_892827.1|	Campylobacter fetus subsp. fetus 82-40	E-value = 5.00E-14	Identity = 31.39%
	2		gi|157164949|ref|YP_001467751.1|	Campylobacter concisus 13826	E-value = 4.00E-11	Identity = 30.74%
	3		gi|154148951|ref|YP_001407001.1|	Campylobacter hominis ATCC BAA-381	E-value = 4.00E-09	Identity = 27.95%
	4		gi|34558319|ref|NP_908134.1|	Wolinella succinogenes DSM 1740	E-value = 2.00E-07	Identity = 28.77%
AB2095	1			***** No matches found *****		
AB2096	1			***** No matches found *****		
AB2097	1			***** No matches found *****		
AB2098	1			***** No matches found *****		
AB2099	1		gi|34556822|ref|NP_906637.1|	Wolinella succinogenes DSM 1740	E-value = 3.00E-58	Identity = 44.38%
	2		gi|157163875|ref|YP_001467827.1|	Campylobacter concisus 13826	E-value = 1.00E-53	Identity = 41.32%
	3		gi|90407986|ref|ZP_01216159.1|	Psychromonas sp. CNPT3	E-value = 4.00E-42	Identity = 37.70%
	4		gi|78777512|ref|YP_393827.1|	Sulfuromonas denitrificans ATCC 33889	E-value = 1.00E-37	Identity = 41.43%
	5		gi|154148650|ref|YP_001406101.1|	Campylobacter hominis ATCC BAA-381	E-value = 8.00E-32	Identity = 38.24%
AB2100	1		gi|157163867|ref|YP_001467828.1|	Campylobacter concisus 13826	E-value = 8.00E-57	Identity = 52.94%
	2		gi|34556821|ref|NP_906636.1|	Wolinella succinogenes DSM 1740	E-value = 8.00E-57	Identity = 50.22%
	3		gi|78777513|ref|YP_393828.1|	Sulfuromonas denitrificans ATCC 33889	E-value = 3.00E-55	Identity = 56.11%
	4		gi|83312015|ref|YP_422279.1|	Magnetospirillum magneticum AMB-1	E-value = 7.00E-47	Identity = 39.46%
	5		gi|154149411|ref|YP_001406100.1|	Campylobacter hominis ATCC BAA-381	E-value = 1.00E-45	Identity = 54.90%
AB2101	1		gi|78777514|ref|YP_393829.1|	Sulfuromonas denitrificans ATCC 33889	E-value = 7.00E-60	Identity = 38.79%
	2		gi|90407988|ref|ZP_01216161.1|	Psychromonas sp. CNPT3	E-value = 1.00E-55	Identity = 31.52%
	3		gi|124003207|ref|ZP_01688057.1|	Microscilla marina ATCC 23134	E-value = 8.00E-51	Identity = 29.61%
	4		gi|154148489|ref|YP_001406099.1|	Campylobacter hominis ATCC BAA-381	E-value = 4.00E-45	Identity = 36.01%
	5		gi|157165309|ref|YP_001467829.1|	Campylobacter concisus 13826	E-value = 5.00E-44	Identity = 32.56%
AB2103	1	*dksA*	gi|34556603|ref|NP_906418.1|	Wolinella succinogenes DSM 1740	E-value = 3.00E-15	Identity = 39.64%
	2		gi|152990732|ref|YP_001356454.1|	Nitratiruptor sp. SB155-2	E-value = 1.00E-14	Identity = 50.00%
	3		gi|154149522|ref|YP_001406123.1|	Campylobacter hominis ATCC BAA-381	E-value = 2.00E-14	Identity = 41.59%
	4		gi|157165088|ref|YP_001466367.1|	Campylobacter concisus 13826	E-value = 1.00E-13	Identity = 39.29%
	5		gi|118475537|ref|YP_891453.1|	Campylobacter fetus subsp. fetus 82-40	E-value = 3.00E-13	Identity = 38.60%
AB2104	1		gi|78777538|ref|YP_393853.1|	Sulfuromonas denitrificans ATCC 33889	E-value = 7.00E-66	Identity = 48.41%
	2		gi|152990974|ref|YP_001356696.1|	Nitratiruptor sp. SB155-2	E-value = 1.00E-59	Identity = 44.10%
	3		gi|34557593|ref|NP_907408.1|	Wolinella succinogenes DSM 1740	E-value = 4.00E-56	Identity = 37.43%
	4		gi|149193912|ref|ZP_01871010.1|	Caminibacter mediatlanticus TB-2	E-value = 2.00E-52	Identity = 39.30%
	5		gi|152990441|ref|YP_001356163.1|	Nitratiruptor sp. SB155-2	E-value = 2.00E-52	Identity = 37.54%
AB2105	1		gi|152990916|ref|YP_001356638.1|	Nitratiruptor sp. SB155-2	E-value = 1.00E-42	Identity = 28.96%
	2		gi|34557200|ref|NP_907015.1|	Wolinella succinogenes DSM 1740	E-value = 1.00E-41	Identity = 30.98%
	3		gi|157164066|ref|YP_001467456.1|	Campylobacter concisus 13826	E-value = 1.00E-34	Identity = 31.08%
	4		gi|152992016|ref|YP_001357737.1|	Sulfurovum sp. NBC37-1	E-value = 3.00E-31	Identity = 30.25%
	5		gi|154175383|ref|YP_001407783.1|	Campylobacter curvus 525.92	E-value = 4.00E-31	Identity = 32.37%
AB2106	1		gi|152990917|ref|YP_001356639.1|	Nitratiruptor sp. SB155-2	E-value = 7.00E-16	Identity = 29.74%
	2		gi|157164673|ref|YP_001467457.1|	Campylobacter concisus 13826	E-value = 3.00E-15	Identity = 33.67%
	3		gi|153951242|ref|YP_001398561.1|	Campylobacter jejuni subsp. doylei 269.97	E-value = 6.00E-14	Identity = 37.57%
	4		gi|157414692|ref|YP_001481948.1|	Campylobacter jejuni subsp. jejuni 81116	E-value = 7.00E-14	Identity = 37.57%
	5		gi|15791764|ref|NP_281587.1|	Campylobacter jejuni subsp. jejuni NCTC 11168	E-value = 7.00E-14	Identity = 37.57%
AB2107	1	*ctpA*	gi|118474694|ref|YP_892114.1|	Campylobacter fetus subsp. fetus 82-40	E-value = 4.00E-127	Identity = 60.19%
	2		gi|157164709|ref|YP_001466744.1|	Campylobacter concisus 13826	E-value = 1.00E-125	Identity = 60.23%
	3		gi|154174324|ref|YP_001408298.1|	Campylobacter curvus 525.92	E-value = 1.00E-125	Identity = 60.14%
	4		gi|152990622|ref|YP_001356344.1|	Nitratiruptor sp. SB155-2	E-value = 5.00E-120	Identity = 56.55%

	5		gi|57238286|ref|YP_178633.1|	Campylobacter jejuni subsp. jejuni RM1221	E-value = 8.00E-119	Identity = 58.14%
AB2108	1	*purC*	gi|51246354|ref|YP_066238.1|	Desulfotalea psychrophila LSv54	E-value = 2.00E-26	Identity = 32.71%
	2		gi|59800779|ref|YP_207491.1|	Neisseria gonorrhoeae FA 1090	E-value = 2.00E-25	Identity = 31.37%
	3		gi|91776594|ref|YP_546350.1|	Methylobacillus flagellatus KT	E-value = 1.00E-24	Identity = 31.97%
	4		gi|94264336|ref|ZP_01288128.1|	delta proteobacterium MLMS-1	E-value = 6.00E-24	Identity = 32.62%
	5		gi|153095129|gb|EDN75676.1|	Mannheimia haemolytica PHL213	E-value = 7.00E-24	Identity = 34.01%
AB2109	1	*purS*	gi|152993049|ref|YP_001358770.1|	Sulfurovum sp. NBC37-1	E-value = 2.00E-17	Identity = 72.73%
	2		gi|152990620|ref|YP_001356342.1|	Nitratiruptor sp. SB155-2	E-value = 1.00E-16	Identity = 64.20%
	3		gi|78776690|ref|YP_393005.1|	Sulfuromonas denitrificans ATCC 33889	E-value = 8.00E-15	Identity = 61.25%
	4		gi|154174281|ref|YP_001408300.1|	Campylobacter curvus 525.92	E-value = 1.00E-14	Identity = 57.14%
	5		gi|82523985|emb|CAI78796.1|	uncultured epsilon proteobacterium	E-value = 1.00E-14	Identity = 58.75%
AB2110	1	*purQ*	gi|82523984|emb|CAI78795.1|	uncultured epsilon proteobacterium	E-value = 4.00E-92	Identity = 71.17%
	2		gi|78776689|ref|YP_393004.1|	Sulfuromonas denitrificans ATCC 33889	E-value = 3.00E-91	Identity = 68.61%
	3		gi|152993050|ref|YP_001358771.1|	Sulfurovum sp. NBC37-1	E-value = 3.00E-90	Identity = 68.33%
	4		gi|152990619|ref|YP_001356341.1|	Nitratiruptor sp. SB155-2	E-value = 5.00E-87	Identity = 64.57%
	5		gi|157165019|ref|YP_001466741.1|	Campylobacter concisus 13826	E-value = 5.00E-81	Identity = 63.96%
AB2111	1		gi|57167563|ref|ZP_00366703.1|	Campylobacter coli RM2228	E-value = 6.00E-35	Identity = 30.63%
	2		gi|57241176|ref|ZP_00369123.1|	Campylobacter lari RM2100	E-value = 6.00E-35	Identity = 29.50%
	3		gi|157414799|ref|YP_001482055.1|	Campylobacter jejuni subsp. jejuni 81116	E-value = 2.00E-34	Identity = 27.98%
	4		gi|15791877|ref|NP_281700.1|	Campylobacter jejuni subsp. jejuni NCTC 11168	E-value = 4.00E-34	Identity = 28.27%
	5		gi|57238282|ref|YP_178637.1|	Campylobacter jejuni subsp. jejuni RM1221	E-value = 5.00E-34	Identity = 27.98%
AB2112	1	*plsC*	gi|34557019|ref|NP_906834.1|	Wolinella succinogenes DSM 1740	E-value = 8.00E-42	Identity = 39.38%
	2		gi|118474516|ref|YP_892119.1|	Campylobacter fetus subsp. fetus 82-40	E-value = 1.00E-41	Identity = 40.45%
	3		gi|154173980|ref|YP_001408302.1|	Campylobacter curvus 525.92	E-value = 2.00E-40	Identity = 38.29%
	4		gi|154148369|ref|YP_001406407.1|	Campylobacter hominis ATCC BAA-381	E-value = 3.00E-38	Identity = 43.24%
	5		gi|15645961|ref|NP_208140.1|	Helicobacter pylori 26695	E-value = 4.00E-37	Identity = 36.68%
AB2113	1	*crcB*	gi|82523981|emb|CAI78792.1|	uncultured epsilon proteobacterium	E-value = 7.00E-16	Identity = 47.66%
	2		gi|78776686|ref|YP_393001.1|	Sulfuromonas denitrificans ATCC 33889	E-value = 1.00E-15	Identity = 47.66%
	3		gi|57238280|ref|YP_178639.1|	Campylobacter jejuni subsp. jejuni RM1221	E-value = 8.00E-12	Identity = 46.96%
	4		gi|86151783|ref|ZP_01069997.1|	Campylobacter jejuni subsp. jejuni 260.94	E-value = 1.00E-11	Identity = 46.96%
	5		gi|152992868|ref|YP_001358589.1|	Sulfurovum sp. NBC37-1	E-value = 3.00E-11	Identity = 44.92%
AB2114	1		gi|34556633|ref|NP_906448.1|	Wolinella succinogenes DSM 1740	E-value = 1.00E-08	Identity = 55.00%
	2		gi|78777989|ref|YP_394304.1|	Sulfuromonas denitrificans ATCC 33889	E-value = 5.00E-07	Identity = 47.30%
	3		gi|156719078|ref|ZP_02060727.1|	Hydrogenobaculum sp. Y04AAS1	E-value = 2.00E-06	Identity = 47.54%
	4		gi|124002667|ref|ZP_01687519.1|	Microscilla marina ATCC 23134	E-value = 4.00E-06	Identity = 48.53%
AB2115	1		gi|34556633|ref|NP_906448.1|	Wolinella succinogenes DSM 1740	E-value = 7.00E-10	Identity = 56.76%
	2		gi|78777989|ref|YP_394304.1|	Sulfuromonas denitrificans ATCC 33889	E-value = 7.00E-08	Identity = 50.00%
	3		gi|124002667|ref|ZP_01687519.1|	Microscilla marina ATCC 23134	E-value = 4.00E-06	Identity = 43.66%
AB2116	1	*argS*	gi|152991237|ref|YP_001356959.1|	Nitratiruptor sp. SB155-2	E-value = 2.00E-172	Identity = 57.47%
	2		gi|154173779|ref|YP_001407667.1|	Campylobacter curvus 525.92	E-value = 1.00E-169	Identity = 56.79%
	3		gi|34556632|ref|NP_906447.1|	Wolinella succinogenes DSM 1740	E-value = 2.00E-167	Identity = 56.14%
	4		gi|57240498|ref|ZP_00368447.1|	Campylobacter lari RM2100	E-value = 8.00E-167	Identity = 56.14%
	5		gi|15792499|ref|NP_282322.1|	Campylobacter jejuni subsp. jejuni NCTC 11168	E-value = 6.00E-166	Identity = 55.20%
AB2117	1		gi|118474789|ref|YP_892570.1|	Campylobacter fetus subsp. fetus 82-40	E-value = 6.00E-24	Identity = 60.00%
	2		gi|57240953|ref|ZP_00368901.1|	Campylobacter lari RM2100	E-value = 1.00E-23	Identity = 55.56%
	3		gi|78777950|ref|YP_394265.1|	Sulfuromonas denitrificans ATCC 33889	E-value = 1.00E-23	Identity = 54.63%
	4		gi|32265849|ref|NP_859881.1|	Helicobacter hepaticus ATCC 51449	E-value = 2.00E-21	Identity = 54.46%
	5		gi|152991236|ref|YP_001356958.1|	Nitratiruptor sp. SB155-2	E-value = 3.00E-21	Identity = 53.00%
AB2118	1	*nadD*	gi|152991235|ref|YP_001356957.1|	Nitratiruptor sp. SB155-2	E-value = 8.00E-40	Identity = 48.85%

	2		gi|57505271|ref|ZP_00371200.1|	Campylobacter upsaliensis RM3195	E-value = 2.00E-35	Identity = 46.59%
	3		gi|118475033|ref|YP_892569.1|	Campylobacter fetus subsp. fetus 82-40	E-value = 1.00E-34	Identity = 46.11%
	4		gi|57168944|ref|ZP_00368073.1|	Campylobacter coli RM2228	E-value = 4.00E-34	Identity = 44.81%
	5		gi|149195101|ref|ZP_01872193.1|	Caminibacter mediatlanticus TB-2	E-value = 1.00E-33	Identity = 49.43%
AB2119	1	*gapA*	gi|152993780|ref|YP_001359501.1|	Sulfurovum sp. NBC37-1	E-value = 1.00E-140	Identity = 75.53%
	2		gi|78777948|ref|YP_394263.1|	Sulfuromonas denitrificans ATCC 33889	E-value = 2.00E-114	Identity = 62.24%
	3		gi|157164277|ref|YP_001467354.1|	Campylobacter concisus 13826	E-value = 6.00E-110	Identity = 62.46%
	4		gi|154175057|ref|YP_001407672.1|	Campylobacter curvus 525.92	E-value = 4.00E-107	Identity = 61.89%
	5		gi|57505272|ref|ZP_00371201.1|	Campylobacter upsaliensis RM3195	E-value = 1.00E-105	Identity = 59.57%
AB2120	1	*pgk*	gi|78777947|ref|YP_394262.1|	Sulfuromonas denitrificans ATCC 33889	E-value = 3.00E-159	Identity = 68.35%
	2		gi|57240950|ref|ZP_00368898.1|	Campylobacter lari RM2100	E-value = 8.00E-152	Identity = 67.59%
	3		gi|152991233|ref|YP_001356955.1|	Nitratiruptor sp. SB155-2	E-value = 2.00E-150	Identity = 65.74%
	4		gi|154173771|ref|YP_001407673.1|	Campylobacter curvus 525.92	E-value = 4.00E-150	Identity = 66.32%
	5		gi|149195103|ref|ZP_01872195.1|	Caminibacter mediatlanticus TB-2	E-value = 3.00E-149	Identity = 65.24%
AB2121	1	*tpiA*	gi|78777946|ref|YP_394261.1|	Sulfuromonas denitrificans ATCC 33889	E-value = 6.00E-70	Identity = 55.56%
	2		gi|34556853|ref|NP_906668.1|	Wolinella succinogenes DSM 1740	E-value = 6.00E-69	Identity = 51.97%
	3		gi|152993778|ref|YP_001359499.1|	Sulfurovum sp. NBC37-1	E-value = 9.00E-65	Identity = 51.32%
	4		gi|152991232|ref|YP_001356954.1|	Nitratiruptor sp. SB155-2	E-value = 1.00E-61	Identity = 50.00%
	5		gi|149195104|ref|ZP_01872196.1|	Caminibacter mediatlanticus TB-2	E-value = 1.00E-57	Identity = 51.28%
AB2122	1	*fabI*	gi|152992312|ref|YP_001358033.1|	Sulfurovum sp. NBC37-1	E-value = 2.00E-122	Identity = 81.32%
	2		gi|34556852|ref|NP_906667.1|	Wolinella succinogenes DSM 1740	E-value = 6.00E-101	Identity = 67.78%
	3		gi|152991231|ref|YP_001356953.1|	Nitratiruptor sp. SB155-2	E-value = 5.00E-98	Identity = 65.43%
	4		gi|153951593|ref|YP_001398703.1|	Campylobacter jejuni subsp. doylei 269.97	E-value = 1.00E-97	Identity = 66.79%
	5		gi|157415633|ref|YP_001482889.1|	Campylobacter jejuni subsp. jejuni 81116	E-value = 1.00E-97	Identity = 66.79%
AB2123	1	*lysA*	gi|82523967|emb|CAI78778.1|	uncultured epsilon proteobacterium	E-value = 1.00E-164	Identity = 70.07%
	2		gi|78776669|ref|YP_392984.1|	Sulfuromonas denitrificans ATCC 33889	E-value = 6.00E-164	Identity = 70.50%
	3		gi|149194454|ref|ZP_01871551.1|	Caminibacter mediatlanticus TB-2	E-value = 6.00E-163	Identity = 71.07%
	4		gi|152993635|ref|YP_001359356.1|	Sulfurovum sp. NBC37-1	E-value = 2.00E-158	Identity = 66.17%
	5		gi|152990136|ref|YP_001355858.1|	Nitratiruptor sp. SB155-2	E-value = 5.00E-156	Identity = 67.67%
AB2124	1	*pheA*	gi|152993634|ref|YP_001359355.1|	Sulfurovum sp. NBC37-1	E-value = 6.00E-122	Identity = 62.32%
	2		gi|152990138|ref|YP_001355860.1|	Nitratiruptor sp. SB155-2	E-value = 1.00E-109	Identity = 60.73%
	3		gi|82523968|emb|CAI78779.1|	uncultured epsilon proteobacterium	E-value = 1.00E-108	Identity = 58.76%
	4		gi|34556767|ref|NP_906582.1|	Wolinella succinogenes DSM 1740	E-value = 8.00E-105	Identity = 54.37%
	5		gi|78776671|ref|YP_392986.1|	Sulfuromonas denitrificans ATCC 33889	E-value = 1.00E-104	Identity = 57.63%
AB2125	1	*hisC*	gi|34558287|ref|NP_908102.1|	Wolinella succinogenes DSM 1740	E-value = 3.00E-132	Identity = 62.13%
	2		gi|82523969|emb|CAI78780.1|	uncultured epsilon proteobacterium	E-value = 7.00E-122	Identity = 59.34%
	3		gi|152990139|ref|YP_001355861.1|	Nitratiruptor sp. SB155-2	E-value = 9.00E-121	Identity = 59.34%
	4		gi|157165462|ref|YP_001466461.1|	Campylobacter concisus 13826	E-value = 5.00E-120	Identity = 60.22%
	5		gi|78776672|ref|YP_392987.1|	Sulfuromonas denitrificans ATCC 33889	E-value = 1.00E-119	Identity = 59.89%
AB2126	1	*dxs*	gi|152993632|ref|YP_001359353.1|	Sulfurovum sp. NBC37-1	E-value = 0	Identity = 65.28%
	2		gi|82523973|emb|CAI78784.1|	uncultured epsilon proteobacterium	E-value = 0	Identity = 65.07%
	3		gi|152990140|ref|YP_001355862.1|	Nitratiruptor sp. SB155-2	E-value = 0	Identity = 64.95%
	4		gi|78776676|ref|YP_392991.1|	Sulfuromonas denitrificans ATCC 33889	E-value = 0	Identity = 63.74%
	5		gi|118475513|ref|YP_891468.1|	Campylobacter fetus subsp. fetus 82-40	E-value = 0	Identity = 61.98%
AB2127	1		gi|152992824|ref|YP_001358545.1|	Sulfurovum sp. NBC37-1	E-value = 1.00E-135	Identity = 73.58%
	2		gi|34557828|ref|NP_907643.1|	Wolinella succinogenes DSM 1740	E-value = 7.00E-134	Identity = 68.34%
	3		gi|78776415|ref|YP_392730.1|	Sulfuromonas denitrificans ATCC 33889	E-value = 5.00E-124	Identity = 65.18%
	4		gi|157414655|ref|YP_001481911.1|	Campylobacter jejuni subsp. jejuni 81116	E-value = 2.00E-121	Identity = 63.91%
	5		gi|57237413|ref|YP_178426.1|	Campylobacter jejuni subsp. jejuni RM1221	E-value = 4.00E-121	Identity = 63.61%

AB2128	1		gi|78778090|ref|YP_394405.1|	Sulfuromonas denitrificans ATCC 33889	E-value = 2.00E-50	Identity = 59.67%
	2		gi|152991267|ref|YP_001356989.1|	Nitratiruptor sp. SB155-2	E-value = 6.00E-45	Identity = 55.31%
	3		gi|34556760|ref|NP_906575.1|	Wolinella succinogenes DSM 1740	E-value = 6.00E-38	Identity = 54.48%
	4		gi|149194978|ref|ZP_01872071.1|	Caminibacter mediatlanticus TB-2	E-value = 1.00E-35	Identity = 50.00%
	5		gi|152991944|ref|YP_001357665.1|	Sulfurovum sp. NBC37-1	E-value = 4.00E-35	Identity = 52.81%
AB2129	1	*panE*	gi|149375503|ref|ZP_01893273.1|	Marinobacter algicola DG893	E-value = 1.00E-50	Identity = 38.41%
	2		gi|149927298|ref|ZP_01915554.1|	Limnobacter sp. MED105	E-value = 1.00E-44	Identity = 34.84%
	3		gi|78223840|ref|YP_385587.1|	Geobacter metallireducens GS-15	E-value = 7.00E-23	Identity = 27.16%
	4		gi|39997777|ref|NP_953728.1|	Geobacter sulfurreducens PCA	E-value = 7.00E-21	Identity = 25.64%
	5		gi|18977768|ref|NP_579125.1|	Pyrococcus furiosus DSM 3638	E-value = 8.00E-21	Identity = 28.38%
AB2130	1		gi|89092478|ref|ZP_01165431.1|	Oceanospirillum sp. MED92	E-value = 1.00E-40	Identity = 29.48%
	2		gi|56479463|ref|YP_161052.1|	Azoarcus sp. EbN1	E-value = 5.00E-40	Identity = 29.77%
	3		gi|95930322|ref|ZP_01313059.1|	Desulfuromonas acetoxidans DSM 684	E-value = 3.00E-38	Identity = 30.87%
	4		gi|92114022|ref|YP_573950.1|	Chromohalobacter salexigens DSM 3043	E-value = 5.00E-38	Identity = 32.41%
	5		gi|152995886|ref|YP_001340721.1|	Marinomonas sp. MWYL1	E-value = 8.00E-38	Identity = 31.70%
AB2131	1		gi|52425388|ref|YP_088525.1|	Mannheimia succiniciproducens MBEL55E	E-value = 1.00E-28	Identity = 41.05%
	2		gi|85058930|ref|YP_454632.1|	Sodalis glossinidius str. 'morsitans'	E-value = 3.00E-27	Identity = 38.61%
	3		gi|156933250|ref|YP_001437166.1|	Enterobacter sakazakii ATCC BAA-894	E-value = 1.00E-26	Identity = 36.20%
	4		gi|77974755|ref|ZP_00830293.1|	Yersinia frederiksenii ATCC 33641	E-value = 1.00E-26	Identity = 37.65%
	5		gi|123441757|ref|YP_001005741.1|	Yersinia enterocolitica subsp. enterocolitica 8081	E-value = 8.00E-26	Identity = 37.04%
AB2132	1		gi|34557562|ref|NP_907377.1|	Wolinella succinogenes DSM 1740	E-value = 3.00E-37	Identity = 44.02%
	2		gi|152991538|ref|YP_001357260.1|	Nitratiruptor sp. SB155-2	E-value = 4.00E-35	Identity = 43.56%
	3		gi|118474814|ref|YP_891973.1|	Campylobacter fetus subsp. fetus 82-40	E-value = 3.00E-31	Identity = 40.53%
	4		gi|157164227|ref|YP_001466426.1|	Campylobacter concisus 13826	E-value = 4.00E-30	Identity = 39.47%
	5		gi|156719026|ref|ZP_02060677.1|	Hydrogenobaculum sp. Y04AAS1	E-value = 3.00E-29	Identity = 38.28%
AB2133	1		gi|149194267|ref|ZP_01871364.1|	Caminibacter mediatlanticus TB-2	E-value = 1.00E-22	Identity = 50.46%
	2		gi|34558386|ref|NP_908201.1|	Wolinella succinogenes DSM 1740	E-value = 3.00E-20	Identity = 44.07%
	3		gi|118474666|ref|YP_892245.1|	Campylobacter fetus subsp. fetus 82-40	E-value = 2.00E-13	Identity = 41.07%
	4		gi|154174906|ref|YP_001408049.1|	Campylobacter curvus 525.92	E-value = 4.00E-13	Identity = 41.74%
	5		gi|148262376|ref|YP_001229082.1|	Geobacter uraniumreducens Rf4	E-value = 4.00E-07	Identity = 31.68%
AB2134	1	*ung*	gi|59712720|ref|YP_205496.1|	Vibrio fischeri ES114	E-value = 1.00E-64	Identity = 56.02%
	2		gi|124523398|ref|ZP_01697522.1|	Bacillus coagulans 36D1	E-value = 5.00E-64	Identity = 55.87%
	3		gi|113460626|ref|YP_718692.1|	Haemophilus somnus 129PT	E-value = 1.00E-63	Identity = 50.70%
	4		gi|149192226|ref|ZP_01870441.1|	Vibrio shilonii AK1	E-value = 1.00E-62	Identity = 50.00%
	5		gi|89072524|ref|ZP_01159096.1|	Photobacterium sp. SKA34	E-value = 2.00E-62	Identity = 51.63%
AB2135	1		gi|124005320|ref|ZP_01690161.1|	Microscilla marina ATCC 23134	E-value = 3.00E-158	Identity = 59.30%
	2		gi|71279133|ref|YP_270035.1|	Colwellia psychrerythraea 34H	E-value = 1.00E-144	Identity = 54.49%
	3		gi|149925663|ref|ZP_01913927.1|	Limnobacter sp. MED105	E-value = 8.00E-141	Identity = 53.71%
	4		gi|126739065|ref|ZP_01754759.1|	Roseobacter sp. SK209-2-6	E-value = 9.00E-141	Identity = 54.17%
	5		gi|85709162|ref|ZP_01040228.1|	Erythrobacter sp. NAP1	E-value = 4.00E-138	Identity = 52.19%
AB2136	1	*ilvB*	gi|124005319|ref|ZP_01690160.1|	Microscilla marina ATCC 23134	E-value = 0	Identity = 71.72%
	2		gi|126665546|ref|ZP_01736528.1|	Marinobacter sp. ELB17	E-value = 0	Identity = 67.03%
	3		gi|149925662|ref|ZP_01913926.1|	Limnobacter sp. MED105	E-value = 0	Identity = 61.90%
	4		gi|88795734|ref|ZP_01111426.1|	Alteromonas macleodii 'Deep ecotype'	E-value = 0	Identity = 64.51%
	5		gi|109900149|ref|YP_663404.1|	Pseudoalteromonas atlantica T6c	E-value = 0	Identity = 62.57%
AB2137	1	*lemA*	gi|23502675|ref|NP_698802.1|	Brucella suis 1330	E-value = 4.00E-53	Identity = 58.97%
	2		gi|17986512|ref|NP_539146.1|	Brucella melitensis 16M	E-value = 1.00E-52	Identity = 58.97%
	3		gi|153008415|ref|YP_001369630.1|	Ochrobactrum anthropi ATCC 49188	E-value = 1.00E-52	Identity = 56.92%
	4		gi|70728732|ref|YP_258481.1|	Pseudomonas fluorescens Pf-5	E-value = 5.00E-52	Identity = 61.49%

	5		gi|110834743|ref|YP_693602.1|	Alcanivorax borkumensis SK2	E-value = 1.00E-51	Identity = 58.20%
AB2138	1		gi|146280843|ref|YP_001170996.1|	Pseudomonas stutzeri A1501	E-value = 5.00E-24	Identity = 32.35%
	2		gi|89072216|ref|ZP_01158795.1|	Photobacterium sp. SKA34	E-value = 8.00E-24	Identity = 35.18%
	3		gi|90577423|ref|ZP_01233234.1|	Vibrio angustum S14	E-value = 8.00E-24	Identity = 35.18%
	4		gi|117619000|ref|YP_856321.1|	Aeromonas hydrophila subsp. hydrophila ATCC 7966	E-value = 2.00E-20	Identity = 30.26%
	5		gi|70728735|ref|YP_258484.1|	Pseudomonas fluorescens Pf-5	E-value = 3.00E-20	Identity = 29.41%
AB2139	1		gi|68549041|ref|ZP_00588509.1|	Pelodictyon phaeoclathratiforme BU-1	E-value = 2.00E-27	Identity = 33.46%
	2		gi|90422845|ref|YP_531215.1|	Rhodopseudomonas palustris BisB18	E-value = 4.00E-23	Identity = 30.29%
	3		gi|146338498|ref|YP_001203546.1|	Bradyrhizobium sp. ORS278	E-value = 1.00E-22	Identity = 30.80%
	4		gi|39934644|ref|NP_946920.1|	Rhodopseudomonas palustris CGA009	E-value = 4.00E-22	Identity = 29.79%
	5		gi|114331384|ref|YP_747606.1|	Nitrosomonas eutropha C91	E-value = 3.00E-21	Identity = 30.57%
AB2140	1	*gph*	gi|51245986|ref|YP_065870.1|	Desulfotalea psychrophila LSv54	E-value = 3.00E-50	Identity = 46.41%
	2		gi|91203839|emb|CAJ71492.1|	Candidatus Kuenenia stuttgartiensis	E-value = 7.00E-45	Identity = 44.60%
	3		gi|116750803|ref|YP_847490.1|	Syntrophobacter fumaroxidans MPOB	E-value = 1.00E-43	Identity = 42.06%
	4		gi|120603872|ref|YP_968272.1|	Desulfovibrio vulgaris subsp. vulgaris DP4	E-value = 7.00E-41	Identity = 41.98%
	5		gi|46578547|ref|YP_009355.1|	Desulfovibrio vulgaris subsp. vulgaris str. Hildenborough	E-value = 1.00E-40	Identity = 41.51%
AB2141	1		gi|32265709|ref|NP_859741.1|	Helicobacter hepaticus ATCC 51449	E-value = 2.00E-36	Identity = 55.00%
	2		gi|34557913|ref|NP_907728.1|	Wolinella succinogenes DSM 1740	E-value = 5.00E-35	Identity = 55.00%
	3		gi|33316782|gb|AAQ04636.1|AF444005_1	Helicobacter bilis	E-value = 6.00E-33	Identity = 54.41%
	4		gi|15792842|ref|NP_282665.1|	Campylobacter jejuni subsp. jejuni NCTC 11168	E-value = 2.00E-31	Identity = 52.52%
	5		gi|157415752|ref|YP_001483008.1|	Campylobacter jejuni subsp. jejuni 81116	E-value = 4.00E-31	Identity = 52.52%
AB2142	1		gi|88861263|ref|ZP_01135895.1|	Pseudoalteromonas tunicata D2	E-value = 3.00E-55	Identity = 29.56%
AB2143	1		gi|88861264|ref|ZP_01135896.1|	Pseudoalteromonas tunicata D2	E-value = 2.00E-07	Identity = 33.63%
AB2144	1		gi|92113599|ref|YP_573527.1|	Chromohalobacter salexigens DSM 3043	E-value = 3.00E-49	Identity = 34.34%
	2		gi|83646919|ref|YP_435354.1|	Hahella chejuensis KCTC 2396	E-value = 8.00E-44	Identity = 32.31%
	3		gi|110834264|ref|YP_693123.1|	Alcanivorax borkumensis SK2	E-value = 4.00E-43	Identity = 34.44%
	4		gi|78485221|ref|YP_391146.1|	Thiomicrospira crunogena XCL-2	E-value = 2.00E-42	Identity = 33.93%
	5		gi|107022043|ref|YP_620370.1|	Burkholderia cenocepacia AU 1054	E-value = 8.00E-40	Identity = 28.77%
AB2145	1			***** No matches found *****		
AB2146	1		gi|149908639|ref|ZP_01897300.1|	Moritella sp. PE36	E-value = 2.00E-31	Identity = 28.23%
	2		gi|82701771|ref|YP_411337.1|	Nitrosospira multiformis ATCC 25196	E-value = 2.00E-31	Identity = 27.03%
AB2147	1		gi|123440822|ref|YP_001004813.1|	Yersinia enterocolitica subsp. enterocolitica 8081	E-value = 9.00E-88	Identity = 48.77%
	2		gi|16123629|ref|NP_406942.1|	Yersinia pestis CO92	E-value = 6.00E-87	Identity = 48.63%
	3		gi|153947901|ref|YP_001402540.1|	Yersinia pseudotuberculosis IP 31758	E-value = 6.00E-87	Identity = 48.36%
	4		gi|109900099|ref|YP_663354.1|	Pseudoalteromonas atlantica T6c	E-value = 6.00E-85	Identity = 47.22%
	5		gi|120601547|ref|YP_965947.1|	Desulfovibrio vulgaris subsp. vulgaris DP4	E-value = 3.00E-77	Identity = 44.14%
AB2148	1		gi|153948753|ref|YP_001402539.1|	Yersinia pseudotuberculosis IP 31758	E-value = 0	Identity = 63.11%
	2		gi|51594844|ref|YP_069035.1|	Yersinia pseudotuberculosis IP 32953	E-value = 0	Identity = 63.02%
	3		gi|16123628|ref|NP_406941.1|	Yersinia pestis CO92	E-value = 0	Identity = 63.02%
	4		gi|123440823|ref|YP_001004814.1|	Yersinia enterocolitica subsp. enterocolitica 8081	E-value = 0	Identity = 62.12%
	5		gi|152995028|ref|YP_001339863.1|	Marinomonas sp. MWYL1	E-value = 0	Identity = 60.19%
AB2149	1		gi|83748930|ref|ZP_00945940.1|	Ralstonia solanacearum UW551	E-value = 5.00E-96	Identity = 41.74%
	2		gi|17549038|ref|NP_522378.1|	Ralstonia solanacearum GMI1000	E-value = 2.00E-95	Identity = 44.20%
	3		gi|148265519|ref|YP_001232225.1|	Geobacter uraniumreducens Rf4	E-value = 5.00E-93	Identity = 44.15%
	4		gi|118744030|ref|ZP_01592028.1|	Geobacter lovleyi SZ	E-value = 6.00E-92	Identity = 41.41%
	5		gi|118578771|ref|YP_900021.1|	Pelobacter propionicus DSM 2379	E-value = 1.00E-89	Identity = 41.81%
AB2150	1		gi|30248820|ref|NP_840890.1|	Nitrosomonas europaea ATCC 19718	E-value = 3.00E-139	Identity = 40.08%
	2		gi|30249066|ref|NP_841136.1|	Nitrosomonas europaea ATCC 19718	E-value = 1.00E-133	Identity = 39.14%
	3		gi|30248981|ref|NP_841051.1|	Nitrosomonas europaea ATCC 19718	E-value = 4.00E-133	Identity = 38.88%

	4		gi|66047091|ref|YP_236932.1|	Pseudomonas syringae pv. syringae B728a	E-value = 1.00E-122	Identity = 36.08%
	5		gi|28871270|ref|NP_793889.1|	Pseudomonas syringae pv. tomato str. DC3000	E-value = 4.00E-121	Identity = 35.63%
AB2151	1		gi|34557769|ref|NP_907584.1|	Wolinella succinogenes DSM 1740	E-value = 4.00E-35	Identity = 32.51%
	2		gi|34557007|ref|NP_906822.1|	Wolinella succinogenes DSM 1740	E-value = 5.00E-35	Identity = 28.88%
	3		gi|70731396|ref|YP_261137.1|	Pseudomonas fluorescens Pf-5	E-value = 8.00E-31	Identity = 25.82%
	4		gi|77460007|ref|YP_349514.1|	Pseudomonas fluorescens PfO-1	E-value = 4.00E-29	Identity = 26.47%
	5		gi|30248821|ref|NP_840891.1|	Nitrosomonas europaea ATCC 19718	E-value = 1.00E-27	Identity = 26.20%
AB2152	1		gi|34557006|ref|NP_906821.1|	Wolinella succinogenes DSM 1740	E-value = 7.00E-22	Identity = 39.35%
	2		gi|30249178|ref|NP_841248.1|	Nitrosomonas europaea ATCC 19718	E-value = 4.00E-19	Identity = 34.69%
	3		gi|34557770|ref|NP_907585.1|	Wolinella succinogenes DSM 1740	E-value = 6.00E-19	Identity = 36.36%
	4		gi|30248569|ref|NP_840639.1|	Nitrosomonas europaea ATCC 19718	E-value = 6.00E-17	Identity = 33.55%
	5		gi|104782680|ref|YP_609178.1|	Pseudomonas entomophila L48	E-value = 8.00E-17	Identity = 32.47%
AB2153	1			***** No matches found *****		
AB2154	1		gi|78776953|ref|YP_393268.1|	Sulfuromonas denitrificans ATCC 33889	E-value = 3.00E-94	Identity = 46.91%
	2		gi|34557963|ref|NP_907778.1|	Wolinella succinogenes DSM 1740	E-value = 6.00E-92	Identity = 45.08%
	3		gi|28209891|ref|NP_780835.1|	Clostridium tetani E88	E-value = 3.00E-69	Identity = 38.02%
	4		gi|152993929|ref|YP_001359650.1|	Sulfurovum sp. NBC37-1	E-value = 7.00E-69	Identity = 37.69%
	5		gi|15896824|ref|NP_350173.1|	Clostridium acetobutylicum ATCC 824	E-value = 1.00E-66	Identity = 36.12%
AB2155	1		gi|119946479|ref|YP_944159.1|	Psychromonas ingrahamii 37	E-value = 2.00E-21	Identity = 49.50%
	2		gi|59711949|ref|YP_204725.1|	Vibrio fischeri ES114	E-value = 1.00E-19	Identity = 47.52%
	3		gi|67939411|ref|ZP_00531913.1|	Chlorobium phaeobacteroides BS1	E-value = 2.00E-19	Identity = 46.53%
	4		gi|145219387|ref|YP_001130096.1|	Prosthecochloris vibrioformis DSM 265	E-value = 6.00E-19	Identity = 45.54%
	5		gi|148975530|ref|ZP_01812401.1|	Vibrionales bacterium SWAT-3	E-value = 6.00E-19	Identity = 47.52%
AB2156	1	*metY*	gi|152991021|ref|YP_001356743.1|	Nitratiruptor sp. SB155-2	E-value = 4.00E-146	Identity = 63.90%
	2		gi|118474795|ref|YP_892089.1|	Campylobacter fetus subsp. fetus 82-40	E-value = 2.00E-144	Identity = 63.83%
	3		gi|157164414|ref|YP_001466687.1|	Campylobacter concisus 13826	E-value = 5.00E-142	Identity = 60.14%
	4		gi|152993374|ref|YP_001359095.1|	Sulfurovum sp. NBC37-1	E-value = 1.00E-141	Identity = 62.47%
	5		gi|78776964|ref|YP_393279.1|	Sulfuromonas denitrificans ATCC 33889	E-value = 9.00E-141	Identity = 59.00%
AB2157	1	*iscR*	gi|78776353|ref|YP_392668.1|	Sulfuromonas denitrificans ATCC 33889	E-value = 7.00E-39	Identity = 62.04%
	2		gi|34557102|ref|NP_906917.1|	Wolinella succinogenes DSM 1740	E-value = 3.00E-23	Identity = 44.09%
	3		gi|118475649|ref|YP_892090.1|	Campylobacter fetus subsp. fetus 82-40	E-value = 3.00E-18	Identity = 41.13%
	4		gi|83590489|ref|YP_430498.1|	Moorella thermoacetica ATCC 39073	E-value = 4.00E-18	Identity = 36.59%
	5		gi|152990511|ref|YP_001356233.1|	Nitratiruptor sp. SB155-2	E-value = 6.00E-18	Identity = 34.78%
AB2158	1	*cysK1*	gi|78776355|ref|YP_392670.1|	Sulfuromonas denitrificans ATCC 33889	E-value = 3.00E-111	Identity = 68.61%
	2		gi|78776634|ref|YP_392949.1|	Sulfuromonas denitrificans ATCC 33889	E-value = 2.00E-105	Identity = 66.67%
	3		gi|118474133|ref|YP_892091.1|	Campylobacter fetus subsp. fetus 82-40	E-value = 2.00E-102	Identity = 63.73%
	4		gi|125974344|ref|YP_001038254.1|	Clostridium thermocellum ATCC 27405	E-value = 2.00E-102	Identity = 66.12%
	5		gi|34557557|ref|NP_907372.1|	Wolinella succinogenes DSM 1740	E-value = 3.00E-101	Identity = 67.53%
AB2159	1		gi|78776356|ref|YP_392671.1|	Sulfuromonas denitrificans ATCC 33889	E-value = 1.00E-23	Identity = 72.37%
	2		gi|86134274|ref|ZP_01052856.1|	Tenacibaculum sp. MED152	E-value = 1.00E-16	Identity = 62.12%
	3		gi|126646383|ref|ZP_01718900.1|	Algoriphagus sp. PR1	E-value = 5.00E-14	Identity = 51.47%
	4		gi|120434689|ref|YP_860376.1|	Gramella forsetii KT0803	E-value = 8.00E-14	Identity = 53.13%
	5		gi|149186184|ref|ZP_01864498.1|	Erythrobacter sp. SD-21	E-value = 6.00E-12	Identity = 52.17%
AB2160	1	*cysH*	gi|78776357|ref|YP_392672.1|	Sulfuromonas denitrificans ATCC 33889	E-value = 2.00E-81	Identity = 62.07%
	2		gi|110598811|ref|ZP_01387069.1|	Chlorobium ferrooxidans DSM 13031	E-value = 8.00E-70	Identity = 51.28%
	3		gi|42557692|emb|CAF28667.1|	uncultured crenarchaeote	E-value = 9.00E-70	Identity = 54.55%
	4		gi|119896721|ref|YP_931934.1|	Azoarcus sp. BH72	E-value = 3.00E-68	Identity = 56.93%
	5		gi|71908527|ref|YP_286114.1|	Dechloromonas aromatica RCB	E-value = 4.00E-67	Identity = 51.71%
AB2161	1	*cysD*	gi|78776358|ref|YP_392673.1|	Sulfuromonas denitrificans ATCC 33889	E-value = 9.00E-156	Identity = 88.45%

	2		gi|71280683|ref|YP_268867.1|	Colwellia psychrerythraea 34H	E-value = 2.00E-145	Identity = 83.16%
	3		gi|90021790|ref|YP_527617.1|	Saccharophagus degradans 2-40	E-value = 1.00E-141	Identity = 80.53%
	4		gi|145300398|ref|YP_001143239.1|	Aeromonas salmonicida subsp. salmonicida A449	E-value = 3.00E-141	Identity = 77.81%
	5		gi|117619880|ref|YP_858037.1|	Aeromonas hydrophila subsp. hydrophila ATCC 7966	E-value = 6.00E-141	Identity = 78.60%
AB2162	1	*cysNC*	gi|78776359|ref|YP_392674.1|	Sulfuromonas denitrificans ATCC 33889	E-value = 0	Identity = 79.36%
	2		gi|88795785|ref|ZP_01111476.1|	Alteromonas macleodii 'Deep ecotype'	E-value = 9.00E-167	Identity = 63.33%
	3		gi|119944561|ref|YP_942241.1|	Psychromonas ingrahamii 37	E-value = 3.00E-165	Identity = 63.46%
	4		gi|86147231|ref|ZP_01065546.1|	Vibrio sp. MED222	E-value = 3.00E-164	Identity = 62.55%
	5		gi|120555646|ref|YP_959997.1|	Marinobacter aquaeolei VT8	E-value = 5.00E-164	Identity = 63.17%
AB2163	1	*cysI*	gi|113474849|ref|YP_720910.1|	Trichodesmium erythraeum IMS101	E-value = 1.00E-26	Identity = 27.08%
	2		gi|119485484|ref|ZP_01619812.1|	Lyngbya sp. PCC 8106	E-value = 1.00E-24	Identity = 25.71%
	3		gi|119510725|ref|ZP_01629852.1|	Nodularia spumigena CCY9414	E-value = 2.00E-23	Identity = 25.06%
AB2164	1		gi|119720108|ref|YP_920603.1|	Thermofilum pendens Hrk 5	E-value = 6.00E-22	Identity = 25.71%
AB2165	1		gi|78776361|ref|YP_392676.1|	Sulfuromonas denitrificans ATCC 33889	E-value = 6.00E-158	Identity = 62.65%
	2		gi|34557558|ref|NP_907373.1|	Wolinella succinogenes DSM 1740	E-value = 3.00E-130	Identity = 52.91%
	3		gi|157164870|ref|YP_001467243.1|	Campylobacter concisus 13826	E-value = 4.00E-123	Identity = 52.28%
	4		gi|118474484|ref|YP_892393.1|	Campylobacter fetus subsp. fetus 82-40	E-value = 3.00E-119	Identity = 53.10%
	5		gi|57505524|ref|ZP_00371451.1|	Campylobacter upsaliensis RM3195	E-value = 3.00E-117	Identity = 48.94%
AB2166	1			***** No matches found *****		
AB2167	1			***** No matches found *****		
AB2168	1	*cobS*	gi|42527884|ref|NP_972982.1|	Treponema denticola ATCC 35405	E-value = 3.00E-10	Identity = 29.41%
	2		gi|118728569|ref|ZP_01577086.1|	Delftia acidovorans SPH-1	E-value = 7.00E-09	Identity = 25.94%
	3		gi|78776312|ref|YP_392627.1|	Sulfuromonas denitrificans ATCC 33889	E-value = 4.00E-07	Identity = 25.20%
	4		gi|110800171|ref|YP_695737.1|	Clostridium perfringens ATCC 13124	E-value = 4.00E-06	Identity = 27.95%
	5		gi|110801834|ref|YP_698432.1|	Clostridium perfringens SM101	E-value = 5.00E-06	Identity = 28.35%
AB2169	1	*cobP*	gi|153876063|ref|ZP_02003574.1|	Beggiatoa sp. PS	E-value = 1.00E-24	Identity = 37.13%
	2		gi|78776313|ref|YP_392628.1|	Sulfuromonas denitrificans ATCC 33889	E-value = 5.00E-21	Identity = 37.95%
	3		gi|78044997|ref|YP_359622.1|	Carboxydothermus hydrogenoformans Z-2901	E-value = 3.00E-20	Identity = 36.09%
	4		gi|90022846|ref|YP_528673.1|	Saccharophagus degradans 2-40	E-value = 9.00E-19	Identity = 35.12%
	5		gi|119476237|ref|ZP_01616588.1|	marine gamma proteobacterium HTCC2143	E-value = 1.00E-18	Identity = 32.12%
AB2170	1		gi|157370509|ref|YP_001478498.1|	Serratia proteamaculans 568	E-value = 1.00E-24	Identity = 39.78%
	2		gi|153954249|ref|YP_001395014.1|	Clostridium kluyveri DSM 555	E-value = 5.00E-24	Identity = 37.32%
	3		gi|68055681|ref|ZP_00539824.1|	Exiguobacterium sibiricum 255-15	E-value = 6.00E-21	Identity = 34.16%
	4		gi|53713020|ref|YP_099012.1|	Bacteroides fragilis YCH46	E-value = 6.00E-20	Identity = 34.90%
	5		gi|149369901|ref|ZP_01889752.1|	unidentified eubacterium SCB49	E-value = 6.00E-20	Identity = 36.73%
AB2171	1		gi|78776308|ref|YP_392623.1|	Sulfuromonas denitrificans ATCC 33889	E-value = 2.00E-81	Identity = 52.99%
	2		gi|15898988|ref|NP_343593.1|	Sulfolobus solfataricus P2	E-value = 1.00E-43	Identity = 34.88%
	3		gi|15679425|ref|NP_276542.1|	Methanothermobacter thermautotrophicus str. Delta H	E-value = 3.00E-42	Identity = 32.41%
	4		gi|147921629|ref|YP_684554.1|	uncultured methanogenic archaeon RC-I	E-value = 4.00E-42	Identity = 33.43%
	5		gi|15922470|ref|NP_378139.1|	Sulfolobus tokodaii str. 7	E-value = 4.00E-40	Identity = 33.62%
AB2172	1		gi|146312205|ref|YP_001177279.1|	Enterobacter sp. 638	E-value = 4.00E-103	Identity = 47.37%
	2		gi|152970099|ref|YP_001335208.1|	Klebsiella pneumoniae subsp. pneumoniae MGH 78578	E-value = 2.00E-101	Identity = 48.82%
	3		gi|108562566|ref|YP_626882.1|	Helicobacter pylori HPAG1	E-value = 4.00E-100	Identity = 45.30%
	4		gi|15803605|ref|NP_289638.1|	Escherichia coli O157:H7 EDL933	E-value = 5.00E-99	Identity = 46.27%
	5		gi|74313598|ref|YP_312017.1|	Shigella sonnei Ss046	E-value = 6.00E-99	Identity = 46.27%
AB2173	1		gi|114320249|ref|YP_741932.1|	Alkalilimnicola ehrlichei MLHE-1	E-value = 2.00E-28	Identity = 31.84%
	2		gi|154148756|ref|YP_001406972.1|	Campylobacter hominis ATCC BAA-381	E-value = 9.00E-20	Identity = 37.92%
	3		gi|93006941|ref|YP_581378.1|	Psychrobacter cryohalolentis K5	E-value = 9.00E-18	Identity = 30.58%
	4		gi|71066387|ref|YP_265114.1|	Psychrobacter arcticus 273-4	E-value = 1.00E-16	Identity = 28.63%

	5		gi|15676362|ref|NP_273498.1|	Neisseria meningitidis MC58	E-value = 9.00E-14	Identity = 29.55%
AB2174	1	*dsbD*	gi|152990860|ref|YP_001356582.1|	Nitratiruptor sp. SB155-2	E-value = 3.00E-152	Identity = 47.39%
	2		gi|78777096|ref|YP_393411.1|	Sulfuromonas denitrificans ATCC 33889	E-value = 6.00E-132	Identity = 47.00%
	3		gi|78484791|ref|YP_390716.1|	Thiomicrospira crunogena XCL-2	E-value = 3.00E-129	Identity = 44.92%
	4		gi|118475366|ref|YP_892248.1|	Campylobacter fetus subsp. fetus 82-40	E-value = 2.00E-128	Identity = 45.89%
	5		gi|152993441|ref|YP_001359162.1|	Sulfurovum sp. NBC37-1	E-value = 8.00E-128	Identity = 44.76%
AB2175	1	*rimK*	gi|78777884|ref|YP_394199.1|	Sulfuromonas denitrificans ATCC 33889	E-value = 4.00E-135	Identity = 81.61%
	2		gi|27367252|ref|NP_762779.1|	Vibrio vulnificus CMCP6	E-value = 1.00E-94	Identity = 60.00%
	3		gi|90021677|ref|YP_527504.1|	Saccharophagus degradans 2-40	E-value = 6.00E-94	Identity = 58.33%
	4		gi|88799473|ref|ZP_01115050.1|	Reinekea sp. MED297	E-value = 7.00E-94	Identity = 58.00%
	5		gi|91228093|ref|ZP_01262135.1|	Vibrio alginolyticus 12G01	E-value = 2.00E-93	Identity = 58.67%
AB2176	1		gi|78777883|ref|YP_394198.1|	Sulfuromonas denitrificans ATCC 33889	E-value = 1.00E-48	Identity = 73.19%
	2		gi|89889991|ref|ZP_01201502.1|	Flavobacteria bacterium BBFL7	E-value = 7.00E-24	Identity = 44.27%
	3		gi|86131051|ref|ZP_01049650.1|	Cellulophaga sp. MED134	E-value = 4.00E-23	Identity = 41.41%
	4		gi|86143736|ref|ZP_01062112.1|	Flavobacterium sp. MED217	E-value = 7.00E-22	Identity = 44.80%
	5		gi|149369505|ref|ZP_01889357.1|	unidentified eubacterium SCB49	E-value = 2.00E-21	Identity = 45.80%
AB2177	1		gi|118475767|ref|YP_891994.1|	Campylobacter fetus subsp. fetus 82-40	E-value = 8.00E-10	Identity = 53.85%
	2		gi|149194742|ref|ZP_01871837.1|	Caminibacter mediatlanticus TB-2	E-value = 3.00E-09	Identity = 52.31%
	3		gi|57241280|ref|ZP_00369227.1|	Campylobacter lari RM2100	E-value = 3.00E-09	Identity = 51.47%
	4		gi|152993403|ref|YP_001359124.1|	Sulfurovum sp. NBC37-1	E-value = 1.00E-07	Identity = 46.48%
	5		gi|78777335|ref|YP_393650.1|	Sulfuromonas denitrificans ATCC 33889	E-value = 2.00E-07	Identity = 50.82%
AB2178	1		gi|34557353|ref|NP_907168.1|	Wolinella succinogenes DSM 1740	E-value = 4.00E-57	Identity = 35.31%
	2		gi|85711301|ref|ZP_01042360.1|	Idiomarina baltica OS145	E-value = 8.00E-53	Identity = 33.17%
	3		gi|78777182|ref|YP_393497.1|	Sulfuromonas denitrificans ATCC 33889	E-value = 7.00E-50	Identity = 32.86%
	4		gi|56460645|ref|YP_155926.1|	Idiomarina loihiensis L2TR	E-value = 2.00E-49	Identity = 31.18%
	5		gi|34557884|ref|NP_907699.1|	Wolinella succinogenes DSM 1740	E-value = 7.00E-49	Identity = 33.74%
AB2179	1	*psd*	gi|152990549|ref|YP_001356271.1|	Nitratiruptor sp. SB155-2	E-value = 4.00E-69	Identity = 51.13%
	2		gi|78777231|ref|YP_393546.1|	Sulfuromonas denitrificans ATCC 33889	E-value = 4.00E-69	Identity = 50.37%
	3		gi|152992672|ref|YP_001358393.1|	Sulfurovum sp. NBC37-1	E-value = 6.00E-69	Identity = 49.44%
	4		gi|157164361|ref|YP_001466430.1|	Campylobacter concisus 13826	E-value = 2.00E-59	Identity = 47.76%
	5		gi|154174301|ref|YP_001407929.1|	Campylobacter curvus 525.92	E-value = 8.00E-57	Identity = 44.66%
AB2180	1		gi|152991823|ref|YP_001357544.1|	Sulfurovum sp. NBC37-1	E-value = 6.00E-09	Identity = 51.61%
AB2181	1	*mltA*	gi|134093493|ref|YP_001098568.1|	Herminiimonas arsenicoxydans	E-value = 4.00E-76	Identity = 41.43%
	2		gi|152979795|ref|YP_001351929.1|	Janthinobacterium sp. Marseille	E-value = 4.00E-75	Identity = 40.29%
	3		gi|95928224|ref|ZP_01310972.1|	Desulfuromonas acetoxidans DSM 684	E-value = 1.00E-74	Identity = 40.97%
	4		gi|114777321|ref|ZP_01452332.1|	Mariprofundus ferrooxydans PV-1	E-value = 2.00E-74	Identity = 37.87%
	5		gi|82703561|ref|YP_413127.1|	Nitrosospira multiformis ATCC 25196	E-value = 2.00E-74	Identity = 41.30%
AB2182	1	*dnaK*	gi|78777841|ref|YP_394156.1|	Sulfuromonas denitrificans ATCC 33889	E-value = 0	Identity = 75.83%
	2		gi|34556917|ref|NP_906732.1|	Wolinella succinogenes DSM 1740	E-value = 0	Identity = 73.69%
	3		gi|152990865|ref|YP_001356587.1|	Nitratiruptor sp. SB155-2	E-value = 0	Identity = 71.79%
	4		gi|32266162|ref|NP_860194.1|	Helicobacter hepaticus ATCC 51449	E-value = 0	Identity = 72.61%
	5		gi|154175431|ref|YP_001408036.1|	Campylobacter curvus 525.92	E-value = 0	Identity = 72.58%
AB2183	1	*grpE*	gi|154174607|ref|YP_001408034.1|	Campylobacter curvus 525.92	E-value = 9.00E-32	Identity = 48.11%
	2		gi|157165150|ref|YP_001467124.1|	Campylobacter concisus 13826	E-value = 2.00E-30	Identity = 49.73%
	3		gi|78777840|ref|YP_394155.1|	Sulfuromonas denitrificans ATCC 33889	E-value = 2.00E-30	Identity = 51.35%
	4		gi|34556918|ref|NP_906733.1|	Wolinella succinogenes DSM 1740	E-value = 5.00E-29	Identity = 48.60%
	5		gi|32266163|ref|NP_860195.1|	Helicobacter hepaticus ATCC 51449	E-value = 5.00E-25	Identity = 44.19%
AB2184	1	*hrcA*	gi|34556919|ref|NP_906734.1|	Wolinella succinogenes DSM 1740	E-value = 5.00E-38	Identity = 35.63%
	2		gi|109947965|ref|YP_665193.1|	Helicobacter acinonychis str. Sheeba	E-value = 4.00E-33	Identity = 33.59%

	3		gi|32266164|ref|NP_860196.1|	Helicobacter hepaticus ATCC 51449	E-value = 2.00E-32	Identity = 35.69%
	4		gi|15611173|ref|NP_222824.1|	Helicobacter pylori J99	E-value = 8.00E-32	Identity = 31.82%
	5		gi|108562536|ref|YP_626852.1|	Helicobacter pylori HPAG1	E-value = 7.00E-31	Identity = 31.06%
AB2185	1		gi|109897405|ref|YP_660660.1|	Pseudoalteromonas atlantica T6c	E-value = 3.00E-23	Identity = 36.76%
	2		gi|154174597|ref|YP_001408656.1|	Campylobacter curvus 525.92	E-value = 9.00E-22	Identity = 38.73%
	3		gi|83646649|ref|YP_435084.1|	Hahella chejuensis KCTC 2396	E-value = 1.00E-21	Identity = 34.55%
	4		gi|146306840|ref|YP_001187305.1|	Pseudomonas mendocina ymp	E-value = 2.00E-21	Identity = 37.21%
	5		gi|78484554|ref|YP_390479.1|	Thiomicrospira crunogena XCL-2	E-value = 1.00E-20	Identity = 35.85%
AB2186	1	*truA*	gi|78777192|ref|YP_393507.1|	Sulfuromonas denitrificans ATCC 33889	E-value = 3.00E-49	Identity = 46.06%
	2		gi|152990852|ref|YP_001356574.1|	Nitratiruptor sp. SB155-2	E-value = 2.00E-48	Identity = 41.91%
	3		gi|152993424|ref|YP_001359145.1|	Sulfurovum sp. NBC37-1	E-value = 1.00E-47	Identity = 43.98%
	4		gi|32266100|ref|NP_860132.1|	Helicobacter hepaticus ATCC 51449	E-value = 4.00E-41	Identity = 39.51%
	5		gi|57240386|ref|ZP_00368335.1|	Campylobacter lari RM2100	E-value = 2.00E-39	Identity = 38.75%
AB2187	1		gi|78777193|ref|YP_393508.1|	Sulfuromonas denitrificans ATCC 33889	E-value = 2.00E-49	Identity = 35.31%
	2		gi|32266101|ref|NP_860133.1|	Helicobacter hepaticus ATCC 51449	E-value = 8.00E-48	Identity = 35.69%
	3		gi|109947019|ref|YP_664247.1|	Helicobacter acinonychis str. Sheeba	E-value = 5.00E-46	Identity = 34.40%
	4		gi|34557088|ref|NP_906903.1|	Wolinella succinogenes DSM 1740	E-value = 3.00E-45	Identity = 35.10%
	5		gi|15644990|ref|NP_207160.1|	Helicobacter pylori 26695	E-value = 5.00E-45	Identity = 33.62%
AB2188	1			***** No matches found *****		
AB2189	1	*uppS*	gi|34558343|ref|NP_908158.1|	Wolinella succinogenes DSM 1740	E-value = 1.00E-77	Identity = 63.64%
	2		gi|78777195|ref|YP_393510.1|	Sulfuromonas denitrificans ATCC 33889	E-value = 7.00E-73	Identity = 64.25%
	3		gi|152990849|ref|YP_001356571.1|	Nitratiruptor sp. SB155-2	E-value = 3.00E-72	Identity = 60.99%
	4		gi|149193962|ref|ZP_01871060.1|	Caminibacter mediatlanticus TB-2	E-value = 7.00E-72	Identity = 65.16%
	5		gi|32266592|ref|NP_860624.1|	Helicobacter hepaticus ATCC 51449	E-value = 1.00E-68	Identity = 55.17%
AB2190	1	*dfp*	gi|78777196|ref|YP_393511.1|	Sulfuromonas denitrificans ATCC 33889	E-value = 3.00E-117	Identity = 55.26%
	2		gi|152990848|ref|YP_001356570.1|	Nitratiruptor sp. SB155-2	E-value = 2.00E-98	Identity = 49.76%
	3		gi|152993420|ref|YP_001359141.1|	Sulfurovum sp. NBC37-1	E-value = 2.00E-97	Identity = 48.07%
	4		gi|34558345|ref|NP_908160.1|	Wolinella succinogenes DSM 1740	E-value = 1.00E-89	Identity = 44.78%
	5		gi|149193960|ref|ZP_01871058.1|	Caminibacter mediatlanticus TB-2	E-value = 5.00E-82	Identity = 48.66%
AB2191	1	*glmU*	gi|34558373|ref|NP_908188.1|	Wolinella succinogenes DSM 1740	E-value = 3.00E-153	Identity = 64.49%
	2		gi|152990847|ref|YP_001356569.1|	Nitratiruptor sp. SB155-2	E-value = 6.00E-141	Identity = 60.61%
	3		gi|152993419|ref|YP_001359140.1|	Sulfurovum sp. NBC37-1	E-value = 9.00E-139	Identity = 59.48%
	4		gi|78777212|ref|YP_393527.1|	Sulfuromonas denitrificans ATCC 33889	E-value = 5.00E-135	Identity = 58.14%
	5		gi|149193959|ref|ZP_01871057.1|	Caminibacter mediatlanticus TB-2	E-value = 2.00E-128	Identity = 60.72%
AB2192	1		gi|123417454|ref|XP_001305115.1|	Trichomonas vaginalis G3	E-value = 3.00E-43	Identity = 28.49%
	2		gi|154419616|ref|XP_001582824.1|	Trichomonas vaginalis G3	E-value = 4.00E-37	Identity = 27.13%
	3		gi|123425100|ref|XP_001306730.1|	Trichomonas vaginalis G3	E-value = 8.00E-35	Identity = 26.24%
	4		gi|154417199|ref|XP_001581620.1|	Trichomonas vaginalis G3	E-value = 1.00E-34	Identity = 27.10%
	5		gi|123425100|ref|XP_001306730.1|	Trichomonas vaginalis G3	E-value = 3.00E-34	Identity = 25.61%
AB2193	1		gi|46203070|ref|ZP_00052136.2|	Magnetospirillum magnetotacticum MS-1	E-value = 3.00E-88	Identity = 41.23%
	2		gi|34497544|ref|NP_901759.1|	Chromobacterium violaceum ATCC 12472	E-value = 1.00E-78	Identity = 38.77%
	3		gi|116061803|emb|CAL52521.1|	Ostreococcus tauri	E-value = 2.00E-68	Identity = 34.15%
	4		gi|30682998|ref|NP_193215.2|	Arabidopsis thaliana	E-value = 5.00E-67	Identity = 34.71%
	5		gi|110739278|dbj|BAF01552.1|	Arabidopsis thaliana	E-value = 1.00E-66	Identity = 34.50%
AB2194	1	*trmA*	gi|78777216|ref|YP_393531.1|	Sulfuromonas denitrificans ATCC 33889	E-value = 4.00E-100	Identity = 50.13%
	2		gi|152993415|ref|YP_001359136.1|	Sulfurovum sp. NBC37-1	E-value = 5.00E-95	Identity = 48.53%
	3		gi|152990451|ref|YP_001356173.1|	Nitratiruptor sp. SB155-2	E-value = 8.00E-95	Identity = 47.72%
	4		gi|146305866|ref|YP_001186331.1|	Pseudomonas mendocina ymp	E-value = 3.00E-87	Identity = 43.80%
	5		gi|104783723|ref|YP_610221.1|	Pseudomonas entomophila L48	E-value = 5.00E-87	Identity = 44.97%

AB2195	1			***** No matches found *****		
AB2199	1	*gidA*	gi|149194011|ref|ZP_01871109.1|	Caminibacter mediatlanticus TB-2	E-value = 0	Identity = 63.96%
	2		gi|57168067|ref|ZP_00367206.1|	Campylobacter coli RM2228	E-value = 0	Identity = 62.29%
	3		gi|157164575|ref|YP_001466158.1|	Campylobacter concisus 13826	E-value = 0	Identity = 65.21%
	4		gi|154175126|ref|YP_001407617.1|	Campylobacter curvus 525.92	E-value = 0	Identity = 63.80%
	5		gi|118475109|ref|YP_892585.1|	Campylobacter fetus subsp. fetus 82-40	E-value = 0	Identity = 64.26%
AB2200	1	*ribE*	gi|78778124|ref|YP_394439.1|	Sulfuromonas denitrificans ATCC 33889	E-value = 2.00E-75	Identity = 67.00%
	2		gi|152991330|ref|YP_001357052.1|	Nitratiruptor sp. SB155-2	E-value = 6.00E-68	Identity = 61.08%
	3		gi|152991801|ref|YP_001357522.1|	Sulfurovum sp. NBC37-1	E-value = 2.00E-64	Identity = 57.56%
	4		gi|34558359|ref|NP_908174.1|	Wolinella succinogenes DSM 1740	E-value = 7.00E-64	Identity = 57.64%
	5		gi|157415481|ref|YP_001482737.1|	Campylobacter jejuni subsp. jejuni 81116	E-value = 4.00E-63	Identity = 56.65%
AB2201	1	*mreC*	gi|34556510|ref|NP_906325.1|	Wolinella succinogenes DSM 1740	E-value = 7.00E-36	Identity = 39.17%
	2		gi|57168397|ref|ZP_00367531.1|	Campylobacter coli RM2228	E-value = 1.00E-34	Identity = 38.83%
	3		gi|153952050|ref|YP_001398674.1|	Campylobacter jejuni subsp. doylei 269.97	E-value = 3.00E-32	Identity = 34.25%
	4		gi|157414574|ref|YP_001481830.1|	Campylobacter jejuni subsp. jejuni 81116	E-value = 4.00E-32	Identity = 33.86%
	5		gi|86153780|ref|ZP_01071983.1|	Campylobacter jejuni subsp. jejuni HB93-13	E-value = 4.00E-32	Identity = 34.54%
AB2202	1	*mreB*	gi|34556511|ref|NP_906326.1|	Wolinella succinogenes DSM 1740	E-value = 1.00E-125	Identity = 70.50%
	2		gi|78777662|ref|YP_393977.1|	Sulfuromonas denitrificans ATCC 33889	E-value = 3.00E-124	Identity = 71.09%
	3		gi|152990292|ref|YP_001356014.1|	Nitratiruptor sp. SB155-2	E-value = 1.00E-123	Identity = 69.10%
	4		gi|154174308|ref|YP_001407542.1|	Campylobacter curvus 525.92	E-value = 4.00E-122	Identity = 68.51%
	5		gi|57241407|ref|ZP_00369353.1|	Campylobacter lari RM2100	E-value = 2.00E-121	Identity = 69.82%
AB2203	1	*clpX*	gi|152990291|ref|YP_001356013.1|	Nitratiruptor sp. SB155-2	E-value = 2.00E-149	Identity = 68.24%
	2		gi|154174197|ref|YP_001407541.1|	Campylobacter curvus 525.92	E-value = 1.00E-146	Identity = 64.68%
	3		gi|157165299|ref|YP_001467559.1|	Campylobacter concisus 13826	E-value = 1.00E-144	Identity = 64.43%
	4		gi|154148896|ref|YP_001406902.1|	Campylobacter hominis ATCC BAA-381	E-value = 5.00E-142	Identity = 64.55%
	5		gi|152992701|ref|YP_001358422.1|	Sulfurovum sp. NBC37-1	E-value = 2.00E-140	Identity = 63.34%
AB2204	1	*lpxA*	gi|152992702|ref|YP_001358423.1|	Sulfurovum sp. NBC37-1	E-value = 6.00E-96	Identity = 67.31%
	2		gi|149194749|ref|ZP_01871844.1|	Caminibacter mediatlanticus TB-2	E-value = 3.00E-91	Identity = 69.39%
	3		gi|78777660|ref|YP_393975.1|	Sulfuromonas denitrificans ATCC 33889	E-value = 6.00E-91	Identity = 66.54%
	4		gi|152990290|ref|YP_001356012.1|	Nitratiruptor sp. SB155-2	E-value = 4.00E-79	Identity = 61.02%
	5		gi|34556513|ref|NP_906328.1|	Wolinella succinogenes DSM 1740	E-value = 2.00E-75	Identity = 54.41%
AB2205	1	*fabZ*	gi|152990289|ref|YP_001356011.1|	Nitratiruptor sp. SB155-2	E-value = 8.00E-54	Identity = 68.92%
	2		gi|86149614|ref|ZP_01067844.1|	Campylobacter jejuni subsp. jejuni CF93-6	E-value = 4.00E-52	Identity = 68.24%
	3		gi|154175396|ref|YP_001407539.1|	Campylobacter curvus 525.92	E-value = 9.00E-52	Identity = 65.54%
	4		gi|15791644|ref|NP_281467.1|	Campylobacter jejuni subsp. jejuni NCTC 11168	E-value = 2.00E-51	Identity = 68.24%
	5		gi|57505538|ref|ZP_00371465.1|	Campylobacter upsaliensis RM3195	E-value = 2.00E-51	Identity = 68.71%
AB2206	1	*lpxB*	gi|78777101|ref|YP_393416.1|	Sulfuromonas denitrificans ATCC 33889	E-value = 6.00E-106	Identity = 59.65%
	2		gi|152991057|ref|YP_001356779.1|	Nitratiruptor sp. SB155-2	E-value = 5.00E-105	Identity = 52.89%
	3		gi|152992705|ref|YP_001358426.1|	Sulfurovum sp. NBC37-1	E-value = 9.00E-99	Identity = 55.75%
	4		gi|34557196|ref|NP_907011.1|	Wolinella succinogenes DSM 1740	E-value = 8.00E-94	Identity = 49.86%
	5		gi|154148546|ref|YP_001406862.1|	Campylobacter hominis ATCC BAA-381	E-value = 1.00E-87	Identity = 48.99%
AB2207	1		gi|152992704|ref|YP_001358425.1|	Sulfurovum sp. NBC37-1	E-value = 4.00E-106	Identity = 51.82%
	2		gi|78777658|ref|YP_393973.1|	Sulfuromonas denitrificans ATCC 33889	E-value = 9.00E-106	Identity = 52.51%
	3		gi|152990288|ref|YP_001356010.1|	Nitratiruptor sp. SB155-2	E-value = 4.00E-105	Identity = 53.78%
	4		gi|149194747|ref|ZP_01871842.1|	Caminibacter mediatlanticus TB-2	E-value = 6.00E-86	Identity = 46.50%
	5		gi|57168392|ref|ZP_00367526.1|	Campylobacter coli RM2228	E-value = 5.00E-82	Identity = 46.69%
AB2208	1		gi|152993826|ref|YP_001359547.1|	Sulfurovum sp. NBC37-1	E-value = 4.00E-07	Identity = 26.45%
AB2209	1	*ndh*	gi|78777411|ref|YP_393726.1|	Sulfuromonas denitrificans ATCC 33889	E-value = 4.00E-64	Identity = 38.68%
	2		gi|152990716|ref|YP_001356438.1|	Nitratiruptor sp. SB155-2	E-value = 1.00E-50	Identity = 34.63%

	3		gi|15805974|ref|NP_294674.1|	Deinococcus radiodurans R1	E-value = 7.00E-29	Identity = 27.98%
	4		gi|90410170|ref|ZP_01218187.1|	Photobacterium profundum 3TCK	E-value = 1.00E-28	Identity = 25.32%
	5		gi|42521954|ref|NP_967334.1|	Bdellovibrio bacteriovorus HD100	E-value = 3.00E-28	Identity = 27.06%
AB2210	1	*nfo*	gi|78778029|ref|YP_394344.1|	Sulfuromonas denitrificans ATCC 33889	E-value = 3.00E-129	Identity = 78.42%
	2		gi|152993825|ref|YP_001359546.1|	Sulfurovum sp. NBC37-1	E-value = 4.00E-116	Identity = 69.15%
	3		gi|152990035|ref|YP_001355757.1|	Nitratiruptor sp. SB155-2	E-value = 2.00E-114	Identity = 71.33%
	4		gi|117925233|ref|YP_865850.1|	Magnetococcus sp. MC-1	E-value = 8.00E-110	Identity = 64.13%
	5		gi|34558061|ref|NP_907876.1|	Wolinella succinogenes DSM 1740	E-value = 5.00E-106	Identity = 65.59%
AB2211	1		gi|39997789|ref|NP_953740.1|	Geobacter sulfurreducens PCA	E-value = 1.00E-92	Identity = 41.45%
	2		gi|17549038|ref|NP_522378.1|	Ralstonia solanacearum GMI1000	E-value = 3.00E-92	Identity = 45.54%
	3		gi|83748930|ref|ZP_00945940.1|	Ralstonia solanacearum UW551	E-value = 1.00E-90	Identity = 41.28%
	4		gi|146284250|ref|YP_001174403.1|	Pseudomonas stutzeri A1501	E-value = 2.00E-90	Identity = 40.63%
	5		gi|56476525|ref|YP_158114.1|	Azoarcus sp. EbN1	E-value = 6.00E-90	Identity = 42.82%
AB2212	1		gi|51594844|ref|YP_069035.1|	Yersinia pseudotuberculosis IP 32953	E-value = 0	Identity = 56.22%
	2		gi|153948753|ref|YP_001402539.1|	Yersinia pseudotuberculosis IP 31758	E-value = 0	Identity = 56.22%
	3		gi|16123628|ref|NP_406941.1|	Yersinia pestis CO92	E-value = 0	Identity = 56.22%
	4		gi|123440823|ref|YP_001004814.1|	Yersinia enterocolitica subsp. enterocolitica 8081	E-value = 0	Identity = 56.41%
	5		gi|152995028|ref|YP_001339863.1|	Marinomonas sp. MWYL1	E-value = 0	Identity = 56.68%
AB2213	1		gi|109900099|ref|YP_663354.1|	Pseudoalteromonas atlantica T6c	E-value = 3.00E-75	Identity = 43.99%
	2		gi|123440822|ref|YP_001004813.1|	Yersinia enterocolitica subsp. enterocolitica 8081	E-value = 1.00E-73	Identity = 43.09%
	3		gi|16123629|ref|NP_406942.1|	Yersinia pestis CO92	E-value = 6.00E-73	Identity = 42.97%
	4		gi|153947901|ref|YP_001402540.1|	Yersinia pseudotuberculosis IP 31758	E-value = 6.00E-73	Identity = 42.70%
	5		gi|120601547|ref|YP_965947.1|	Desulfovibrio vulgaris subsp. vulgaris DP4	E-value = 2.00E-71	Identity = 43.58%
AB2214	1		gi|157370926|ref|YP_001478915.1|	Serratia proteamaculans 568	E-value = 2.00E-22	Identity = 30.73%
	2		gi|84321967|ref|ZP_00970317.1|	Pseudomonas aeruginosa C3719	E-value = 1.00E-20	Identity = 29.05%
	3		gi|152987615|ref|YP_001349461.1|	Pseudomonas aeruginosa PA7	E-value = 2.00E-20	Identity = 29.05%
	4		gi|107100734|ref|ZP_01364652.1|	Pseudomonas aeruginosa PACS2	E-value = 2.00E-20	Identity = 29.05%
	5		gi|15596480|ref|NP_249974.1|	Pseudomonas aeruginosa PAO1	E-value = 2.00E-20	Identity = 29.05%
AB2215	1		gi|104782149|ref|YP_608647.1|	Pseudomonas entomophila L48	E-value = 2.00E-62	Identity = 43.49%
	2		gi|126359256|ref|ZP_01716251.1|	Pseudomonas putida GB-1	E-value = 2.00E-62	Identity = 39.79%
	3		gi|148256052|ref|YP_001240637.1|	Bradyrhizobium sp. BTAi1	E-value = 6.00E-58	Identity = 37.83%
	4		gi|146300602|ref|YP_001195193.1|	Flavobacterium johnsoniae UW101	E-value = 6.00E-42	Identity = 30.16%
	5		gi|77918706|ref|YP_356521.1|	Pelobacter carbinolicus DSM 2380	E-value = 5.00E-41	Identity = 30.86%
AB2216	1		gi|88812623|ref|ZP_01127870.1|	Nitrococcus mobilis Nb-231	E-value = 4.00E-134	Identity = 51.25%
	2		gi|127511431|ref|YP_001092628.1|	Shewanella loihica PV-4	E-value = 1.00E-132	Identity = 55.38%
	3		gi|114776863|ref|ZP_01451906.1|	Mariprofundus ferrooxydans PV-1	E-value = 3.00E-132	Identity = 54.61%
	4		gi|146281486|ref|YP_001171639.1|	Pseudomonas stutzeri A1501	E-value = 1.00E-116	Identity = 46.54%
	5		gi|90581661|ref|ZP_01237450.1|	Vibrio angustum S14	E-value = 6.00E-116	Identity = 47.63%
AB2217	1			***** No matches found *****		
AB2218	1			***** No matches found *****		
AB2219	1			***** No matches found *****		
AB2220	1		gi|152990036|ref|YP_001355758.1|	Nitratiruptor sp. SB155-2	E-value = 0	Identity = 58.75%
	2		gi|78778027|ref|YP_394342.1|	Sulfuromonas denitrificans ATCC 33889	E-value = 4.00E-177	Identity = 57.71%
	3		gi|34557975|ref|NP_907790.1|	Wolinella succinogenes DSM 1740	E-value = 8.00E-175	Identity = 60.55%
	4		gi|152993812|ref|YP_001359533.1|	Sulfurovum sp. NBC37-1	E-value = 9.00E-175	Identity = 56.77%
	5		gi|32265973|ref|NP_860005.1|	Helicobacter hepaticus ATCC 51449	E-value = 8.00E-161	Identity = 55.29%
AB2221	1		gi|32267097|ref|NP_861129.1|	Helicobacter hepaticus ATCC 51449	E-value = 4.00E-15	Identity = 29.05%
	2		gi|154148054|ref|YP_001405794.1|	Campylobacter hominis ATCC BAA-381	E-value = 1.00E-11	Identity = 34.11%
	3		gi|57241364|ref|ZP_00369310.1|	Campylobacter lari RM2100	E-value = 4.00E-10	Identity = 31.12%

	4		gi|57168321|ref|ZP_00367455.1|	Campylobacter coli RM2228	E-value = 4.00E-09	Identity = 29.46%
	5		gi|149194369|ref|ZP_01871466.1|	Caminibacter mediatlanticus TB-2	E-value = 2.00E-08	Identity = 29.05%
AB2222	1		gi|154148760|ref|YP_001405942.1|	Campylobacter hominis ATCC BAA-381	E-value = 4.00E-106	Identity = 67.01%
	2		gi|118474559|ref|YP_891407.1|	Campylobacter fetus subsp. fetus 82-40	E-value = 2.00E-105	Identity = 66.43%
	3		gi|78778198|ref|YP_394513.1|	Sulfuromonas denitrificans ATCC 33889	E-value = 9.00E-105	Identity = 69.15%
	4		gi|34557829|ref|NP_907644.1|	Wolinella succinogenes DSM 1740	E-value = 1.00E-103	Identity = 66.08%
	5		gi|152991400|ref|YP_001357122.1|	Nitratiruptor sp. SB155-2	E-value = 7.00E-103	Identity = 69.15%
AB2223	1	*murB*	gi|152991402|ref|YP_001357124.1|	Nitratiruptor sp. SB155-2	E-value = 4.00E-67	Identity = 50.20%
	2		gi|152991676|ref|YP_001357397.1|	Sulfurovum sp. NBC37-1	E-value = 4.00E-63	Identity = 49.61%
	3		gi|78778200|ref|YP_394515.1|	Sulfuromonas denitrificans ATCC 33889	E-value = 1.00E-59	Identity = 49.02%
	4		gi|32265938|ref|NP_859970.1|	Helicobacter hepaticus ATCC 51449	E-value = 2.00E-59	Identity = 45.35%
	5		gi|154174461|ref|YP_001409020.1|	Campylobacter curvus 525.92	E-value = 1.00E-58	Identity = 47.27%
AB2224	1	*topA*	gi|114321779|ref|YP_743462.1|	Alkalilimnicola ehrlichei MLHE-1	E-value = 0	Identity = 47.15%
	2		gi|88811388|ref|ZP_01126643.1|	Nitrococcus mobilis Nb-231	E-value = 1.00E-178	Identity = 47.97%
	3		gi|77166457|ref|YP_344982.1|	Nitrosococcus oceani ATCC 19707	E-value = 6.00E-176	Identity = 46.11%
	4		gi|121999104|ref|YP_001003891.1|	Halorhodospira halophila SL1	E-value = 2.00E-174	Identity = 45.63%
	5		gi|28199638|ref|NP_779952.1|	Xylella fastidiosa Temecula1	E-value = 2.00E-173	Identity = 46.16%
AB2225	1		gi|78776371|ref|YP_392686.1|	Sulfuromonas denitrificans ATCC 33889	E-value = 3.00E-39	Identity = 44.71%
	2		gi|149194968|ref|ZP_01872061.1|	Caminibacter mediatlanticus TB-2	E-value = 4.00E-37	Identity = 47.24%
	3		gi|154174120|ref|YP_001408975.1|	Campylobacter curvus 525.92	E-value = 3.00E-32	Identity = 42.01%
	4		gi|152991393|ref|YP_001357115.1|	Nitratiruptor sp. SB155-2	E-value = 2.00E-31	Identity = 44.24%
	5		gi|154149295|ref|YP_001407081.1|	Campylobacter hominis ATCC BAA-381	E-value = 1.00E-27	Identity = 44.37%
AB2226	1	*bioB*	gi|152991394|ref|YP_001357116.1|	Nitratiruptor sp. SB155-2	E-value = 3.00E-100	Identity = 64.03%
	2		gi|78776370|ref|YP_392685.1|	Sulfuromonas denitrificans ATCC 33889	E-value = 1.00E-94	Identity = 57.97%
	3		gi|153951861|ref|YP_001398962.1|	Campylobacter jejuni subsp. doylei 269.97	E-value = 2.00E-91	Identity = 56.68%
	4		gi|157415900|ref|YP_001483156.1|	Campylobacter jejuni subsp. jejuni 81116	E-value = 5.00E-91	Identity = 56.68%
	5		gi|86152694|ref|ZP_01070899.1|	Campylobacter jejuni subsp. jejuni HB93-13	E-value = 5.00E-91	Identity = 56.68%
AB2227	1			***** No matches found *****		
AB2228	1		gi|34557832|ref|NP_907647.1|	Wolinella succinogenes DSM 1740	E-value = 3.00E-06	Identity = 42.86%
AB2229	1	*eno*	gi|152991679|ref|YP_001357400.1|	Sulfurovum sp. NBC37-1	E-value = 0	Identity = 78.12%
	2		gi|78778195|ref|YP_394510.1|	Sulfuromonas denitrificans ATCC 33889	E-value = 4.00E-180	Identity = 76.60%
	3		gi|34557831|ref|NP_907646.1|	Wolinella succinogenes DSM 1740	E-value = 1.00E-177	Identity = 72.81%
	4		gi|152991398|ref|YP_001357120.1|	Nitratiruptor sp. SB155-2	E-value = 2.00E-173	Identity = 73.29%
	5		gi|32266130|ref|NP_860162.1|	Helicobacter hepaticus ATCC 51449	E-value = 2.00E-167	Identity = 70.42%
AB2230	1	*recA*	gi|78778196|ref|YP_394511.1|	Sulfuromonas denitrificans ATCC 33889	E-value = 9.00E-142	Identity = 75.57%
	2		gi|157164266|ref|YP_001467588.1|	Campylobacter concisus 13826	E-value = 1.00E-139	Identity = 76.32%
	3		gi|154173783|ref|YP_001409018.1|	Campylobacter curvus 525.92	E-value = 1.00E-138	Identity = 75.37%
	4		gi|152991399|ref|YP_001357121.1|	Nitratiruptor sp. SB155-2	E-value = 4.00E-138	Identity = 74.55%
	5		gi|634052|emb|CAA84615.1|	Helicobacter pylori	E-value = 9.00E-138	Identity = 76.35%
AB2231	1			***** No matches found *****		
AB2232	1	*pseB*	gi|154174107|ref|YP_001408963.1|	Campylobacter curvus 525.92	E-value = 6.00E-144	Identity = 75.30%
	2		gi|86152657|ref|ZP_01070862.1|	Campylobacter jejuni subsp. jejuni HB93-13	E-value = 9.00E-142	Identity = 74.70%
	3		gi|37731984|gb|AAO73065.1|	Campylobacter coli	E-value = 2.00E-141	Identity = 74.70%
	4		gi|153951236|ref|YP_001397620.1|	Campylobacter jejuni subsp. doylei 269.97	E-value = 3.00E-141	Identity = 74.70%
	5		gi|157415556|ref|YP_001482812.1|	Campylobacter jejuni subsp. jejuni 81116	E-value = 4.00E-141	Identity = 74.09%
AB2233	1		gi|149194656|ref|ZP_01871751.1|	Caminibacter mediatlanticus TB-2	E-value = 7.00E-103	Identity = 57.92%
	2		gi|125974706|ref|YP_001038616.1|	Clostridium thermocellum ATCC 27405	E-value = 8.00E-90	Identity = 46.44%
	3		gi|148643596|ref|YP_001274109.1|	Methanobrevibacter smithii ATCC 35061	E-value = 4.00E-89	Identity = 45.93%
	4		gi|71065220|ref|YP_263947.1|	Psychrobacter arcticus 273-4	E-value = 7.00E-89	Identity = 42.75%

	5		gi|75761581|ref|ZP_00741536.1|	Bacillus thuringiensis serovar israelensis ATCC 35646	E-value = 4.00E-87	Identity = 46.05%
AB2234	1	*neuA*	gi|88604322|ref|YP_504500.1|	Methanospirillum hungatei JF-1	E-value = 2.00E-71	Identity = 58.48%
	2		gi|71065222|ref|YP_263949.1|	Psychrobacter arcticus 273-4	E-value = 2.00E-71	Identity = 57.78%
	3		gi|114321471|ref|YP_743154.1|	Alkalilimnicola ehrlichei MLHE-1	E-value = 8.00E-70	Identity = 56.58%
	4		gi|109899383|ref|YP_662638.1|	Pseudoalteromonas atlantica T6c	E-value = 1.00E-68	Identity = 60.70%
	5		gi|54309869|ref|YP_130889.1|	Photobacterium profundum SS9	E-value = 2.00E-67	Identity = 55.70%
AB2235	1	*neuB*	gi|154173860|ref|YP_001408968.1|	Campylobacter curvus 525.92	E-value = 2.00E-114	Identity = 61.29%
	2		gi|120436362|ref|YP_862048.1|	Gramella forsetii KT0803	E-value = 2.00E-113	Identity = 59.76%
	3		gi|106895373|ref|ZP_01362470.1|	Clostridium sp. OhILAs	E-value = 3.00E-112	Identity = 60.82%
	4		gi|149194662|ref|ZP_01871757.1|	Caminibacter mediatlanticus TB-2	E-value = 5.00E-111	Identity = 61.36%
	5		gi|34558392|ref|NP_908207.1|	Wolinella succinogenes DSM 1740	E-value = 3.00E-108	Identity = 58.28%
AB2236	1		gi|125974707|ref|YP_001038617.1|	Clostridium thermocellum ATCC 27405	E-value = 3.00E-21	Identity = 26.99%
	2		gi|114565781|ref|YP_752935.1|	Syntrophomonas wolfei subsp. wolfei str. Goettingen	E-value = 4.00E-18	Identity = 27.78%
	3		gi|15895455|ref|NP_348804.1|	Clostridium acetobutylicum ATCC 824	E-value = 4.00E-17	Identity = 27.38%
	4		gi|118444536|ref|YP_877963.1|	Clostridium novyi NT	E-value = 2.00E-15	Identity = 30.22%
	5		gi|148380657|ref|YP_001255198.1|	Clostridium botulinum A str. ATCC 3502	E-value = 3.00E-15	Identity = 31.47%
AB2237	1		gi|156860273|gb|EDO53704.1|	Bacteroides uniformis ATCC 8492	E-value = 1.00E-43	Identity = 34.49%
AB2238	1	*flmE*	gi|86151123|ref|ZP_01069339.1|	Campylobacter jejuni subsp. jejuni 260.94	E-value = 4.00E-28	Identity = 38.57%
	2		gi|153951192|ref|YP_001397602.1|	Campylobacter jejuni subsp. doylei 269.97	E-value = 5.00E-28	Identity = 38.12%
	3		gi|153952545|ref|YP_001397603.1|	Campylobacter jejuni subsp. doylei 269.97	E-value = 2.00E-24	Identity = 37.44%
	4		gi|86151072|ref|ZP_01069288.1|	Campylobacter jejuni subsp. jejuni 260.94	E-value = 2.00E-24	Identity = 37.62%
	5		gi|16127088|ref|NP_421652.1|	Caulobacter crescentus CB15	E-value = 5.00E-24	Identity = 34.40%
AB2239	1		gi|28211360|ref|NP_782304.1|	Clostridium tetani E88	E-value = 4.00E-39	Identity = 33.99%
	2		gi|156860272|gb|EDO53703.1|	Bacteroides uniformis ATCC 8492	E-value = 1.00E-30	Identity = 31.56%
	3		gi|29348783|ref|NP_812286.1|	Bacteroides thetaiotaomicron VPI-5482	E-value = 7.00E-28	Identity = 25.65%
	4		gi|120555506|ref|YP_959857.1|	Marinobacter aquaeolei VT8	E-value = 7.00E-27	Identity = 26.76%
	5		gi|114046894|ref|YP_737444.1|	Shewanella sp. MR-7	E-value = 4.00E-25	Identity = 27.91%
AB2240	1		gi|120598212|ref|YP_962786.1|	Shewanella sp. W3-18-1	E-value = 1.00E-16	Identity = 28.67%
	2		gi|146293714|ref|YP_001184138.1|	Shewanella putrefaciens CN-32	E-value = 2.00E-16	Identity = 28.67%
AB2241	1		gi|126652462|ref|ZP_01724634.1|	Bacillus sp. B14905	E-value = 3.00E-27	Identity = 38.03%
	2		gi|84488889|ref|YP_447121.1|	Methanosphaera stadtmanae DSM 3091	E-value = 6.00E-14	Identity = 28.91%
AB2242	1		gi|78776375|ref|YP_392690.1|	Sulfuromonas denitrificans ATCC 33889	E-value = 8.00E-42	Identity = 27.10%
AB2243	1	*flaA*	gi|2506429|sp|P21989|FLA2_TREPH	Treponema phagedenis	E-value = 8.00E-26	Identity = 30.29%
	2		gi|89210132|ref|ZP_01188524.1|	Halothermothrix orenii H 168	E-value = 9.00E-26	Identity = 32.68%
	3		gi|74273842|gb|ABA01569.1|	Aeromonas hydrophila	E-value = 1.00E-25	Identity = 33.75%
	4		gi|15214031|sp|Q9KWX0|FLAB2_TREMA	Treponema maltophilum	E-value = 2.00E-25	Identity = 28.80%
	5		gi|15639779|ref|NP_219229.1|	Treponema pallidum subsp. pallidum str. Nichols	E-value = 2.00E-25	Identity = 28.99%
AB2244	1	*flaB*	gi|89210132|ref|ZP_01188524.1|	Halothermothrix orenii H 168	E-value = 3.00E-26	Identity = 33.01%
	2		gi|2506429|sp|P21989|FLA2_TREPH	Treponema phagedenis	E-value = 4.00E-26	Identity = 30.94%
	3		gi|15639779|ref|NP_219229.1|	Treponema pallidum subsp. pallidum str. Nichols	E-value = 8.00E-26	Identity = 29.64%
	4		gi|15214031|sp|Q9KWX0|FLAB2_TREMA	Treponema maltophilum	E-value = 1.00E-25	Identity = 30.62%
	5		gi|15639853|ref|NP_219303.1|	Treponema pallidum subsp. pallidum str. Nichols	E-value = 1.00E-25	Identity = 31.60%
AB2245	1		gi|152991354|ref|YP_001357076.1|	Nitratiruptor sp. SB155-2	E-value = 2.00E-158	Identity = 60.26%
	2		gi|152989894|ref|YP_001355616.1|	Nitratiruptor sp. SB155-2	E-value = 8.00E-158	Identity = 60.26%
	3		gi|152991668|ref|YP_001357389.1|	Sulfurovum sp. NBC37-1	E-value = 2.00E-155	Identity = 61.96%
	4		gi|34557050|ref|NP_906865.1|	Wolinella succinogenes DSM 1740	E-value = 2.00E-147	Identity = 59.69%
	5		gi|78778224|ref|YP_394539.1|	Sulfuromonas denitrificans ATCC 33889	E-value = 6.00E-136	Identity = 54.87%
AB2246	1	*pyrG*	gi|78778274|ref|YP_394589.1|	Sulfuromonas denitrificans ATCC 33889	E-value = 0	Identity = 68.61%
	2		gi|152989906|ref|YP_001355628.1|	Nitratiruptor sp. SB155-2	E-value = 0	Identity = 69.59%

	3		gi|154174294|ref|YP_001409210.1|	Campylobacter curvus 525.92	E-value = 0	Identity = 67.98%
	4		gi|34556667|ref|NP_906482.1|	Wolinella succinogenes DSM 1740	E-value = 0	Identity = 69.66%
	5		gi|157164699|ref|YP_001467775.1|	Campylobacter concisus 13826	E-value = 0	Identity = 66.48%
AB2247	1	*recJ*	gi|57169020|ref|ZP_00368148.1|	Campylobacter coli RM2228	E-value = 3.00E-120	Identity = 45.80%
	2		gi|34556668|ref|NP_906483.1|	Wolinella succinogenes DSM 1740	E-value = 8.00E-119	Identity = 42.61%
	3		gi|153952383|ref|YP_001397298.1|	Campylobacter jejuni subsp. doylei 269.97	E-value = 1.00E-118	Identity = 45.42%
	4		gi|157165222|ref|YP_001467773.1|	Campylobacter concisus 13826	E-value = 2.00E-118	Identity = 45.14%
	5		gi|121612339|ref|YP_999748.1|	Campylobacter jejuni subsp. jejuni 81-176	E-value = 3.00E-118	Identity = 45.04%
AB2248	1			***** No matches found *****		
AB2249	1		gi|149909955|ref|ZP_01898604.1|	Moritella sp. PE36	E-value = 7.00E-14	Identity = 27.41%
	2		gi|153834452|ref|ZP_01987119.1|	Vibrio harveyi HY01	E-value = 2.00E-13	Identity = 25.14%
	3		gi|28897542|ref|NP_797147.1|	Vibrio parahaemolyticus RIMD 2210633	E-value = 6.00E-13	Identity = 25.14%
	4		gi|77359566|ref|YP_339141.1|	Pseudoalteromonas haloplanktis TAC125	E-value = 3.00E-09	Identity = 31.02%
	5		gi|118753972|ref|ZP_01601771.1|	Shewanella pealeana ATCC 700345	E-value = 1.00E-08	Identity = 26.63%
AB2250	1	*thiJ*	gi|149194370|ref|ZP_01871467.1|	Caminibacter mediatlanticus TB-2	E-value = 3.00E-47	Identity = 54.44%
	2		gi|78777413|ref|YP_393728.1|	Sulfuromonas denitrificans ATCC 33889	E-value = 3.00E-46	Identity = 56.35%
	3		gi|118474065|ref|YP_892402.1|	Campylobacter fetus subsp. fetus 82-40	E-value = 3.00E-43	Identity = 51.41%
	4		gi|154147989|ref|YP_001407156.1|	Campylobacter hominis ATCC BAA-381	E-value = 3.00E-42	Identity = 49.44%
	5		gi|154175067|ref|YP_001407863.1|	Campylobacter curvus 525.92	E-value = 1.00E-41	Identity = 48.07%
AB2251	1	*dnaE*	gi|152992163|ref|YP_001357884.1|	Sulfurovum sp. NBC37-1	E-value = 0	Identity = 74.72%
	2		gi|152990427|ref|YP_001356149.1|	Nitratiruptor sp. SB155-2	E-value = 0	Identity = 66.67%
	3		gi|34558271|ref|NP_908086.1|	Wolinella succinogenes DSM 1740	E-value = 0	Identity = 65.17%
	4		gi|78777108|ref|YP_393423.1|	Sulfuromonas denitrificans ATCC 33889	E-value = 0	Identity = 64.00%
	5		gi|157165237|ref|YP_001467325.1|	Campylobacter concisus 13826	E-value = 0	Identity = 64.54%
AB2252	1	*surE*	gi|152992895|ref|YP_001358616.1|	Sulfurovum sp. NBC37-1	E-value = 2.00E-91	Identity = 60.77%
	2		gi|152991061|ref|YP_001356783.1|	Nitratiruptor sp. SB155-2	E-value = 4.00E-86	Identity = 55.38%
	3		gi|32265834|ref|NP_859866.1|	Helicobacter hepaticus ATCC 51449	E-value = 7.00E-86	Identity = 57.58%
	4		gi|34557990|ref|NP_907805.1|	Wolinella succinogenes DSM 1740	E-value = 2.00E-85	Identity = 57.85%
	5		gi|149193772|ref|ZP_01870870.1|	Caminibacter mediatlanticus TB-2	E-value = 2.00E-70	Identity = 52.29%
AB2253	1			***** No matches found *****		
AB2254	1		gi|78778215|ref|YP_394530.1|	Sulfuromonas denitrificans ATCC 33889	E-value = 4.00E-15	Identity = 49.43%
	2		gi|34557624|ref|NP_907439.1|	Wolinella succinogenes DSM 1740	E-value = 6.00E-08	Identity = 37.50%
	3		gi|152991665|ref|YP_001357386.1|	Sulfurovum sp. NBC37-1	E-value = 9.00E-08	Identity = 32.10%
	4		gi|157165210|ref|YP_001467677.1|	Campylobacter concisus 13826	E-value = 1.00E-07	Identity = 45.33%
	5		gi|118474823|ref|YP_891352.1|	Campylobacter fetus subsp. fetus 82-40	E-value = 4.00E-07	Identity = 40.51%
AB2255	1	*moaC*	gi|78778214|ref|YP_394529.1|	Sulfuromonas denitrificans ATCC 33889	E-value = 2.00E-57	Identity = 75.00%
	2		gi|157165431|ref|YP_001467676.1|	Campylobacter concisus 13826	E-value = 6.00E-54	Identity = 70.78%
	3		gi|154173634|ref|YP_001409053.1|	Campylobacter curvus 525.92	E-value = 9.00E-54	Identity = 71.43%
	4		gi|152991442|ref|YP_001357164.1|	Nitratiruptor sp. SB155-2	E-value = 8.00E-53	Identity = 70.32%
	5		gi|152991666|ref|YP_001357387.1|	Sulfurovum sp. NBC37-1	E-value = 1.00E-52	Identity = 69.87%
AB2256	1	*rpsU*	gi|57168606|ref|ZP_00367739.1|	Campylobacter coli RM2228	E-value = 1.00E-16	Identity = 87.32%
	2		gi|148926462|ref|ZP_01810145.1|	Campylobacter jejuni subsp. jejuni CG8486	E-value = 2.00E-16	Identity = 88.57%
	3		gi|57241700|ref|ZP_00369645.1|	Campylobacter lari RM2100	E-value = 2.00E-16	Identity = 86.30%
	4		gi|154174430|ref|YP_001407474.1|	Campylobacter curvus 525.92	E-value = 5.00E-16	Identity = 88.57%
	5		gi|15791737|ref|NP_281560.1|	Campylobacter jejuni subsp. jejuni NCTC 11168	E-value = 6.00E-16	Identity = 87.14%
AB2257	1		gi|154175178|ref|YP_001409185.1|	Campylobacter curvus 525.92	E-value = 7.00E-52	Identity = 54.78%
	2		gi|157164800|ref|YP_001467730.1|	Campylobacter concisus 13826	E-value = 2.00E-51	Identity = 56.22%
	3		gi|57241745|ref|ZP_00369690.1|	Campylobacter lari RM2100	E-value = 2.00E-48	Identity = 51.72%
	4		gi|34556781|ref|NP_906596.1|	Wolinella succinogenes DSM 1740	E-value = 2.00E-47	Identity = 53.59%

	5		gi|152991576|ref|YP_001357298.1|	Nitratiruptor sp. SB155-2	E-value = 7.00E-47	Identity = 52.94%
AB2258	1		gi|78778249|ref|YP_394564.1|	Sulfuromonas denitrificans ATCC 33889	E-value = 8.00E-27	Identity = 70.09%
	2		gi|34556782|ref|NP_906597.1|	Wolinella succinogenes DSM 1740	E-value = 1.00E-25	Identity = 62.39%
	3		gi|152991577|ref|YP_001357299.1|	Nitratiruptor sp. SB155-2	E-value = 1.00E-24	Identity = 60.94%
	4		gi|149194936|ref|ZP_01872029.1|	Caminibacter mediatlanticus TB-2	E-value = 1.00E-23	Identity = 55.64%
	5		gi|154174496|ref|YP_001409186.1|	Campylobacter curvus 525.92	E-value = 2.00E-23	Identity = 58.54%
AB2259	1		gi|78778256|ref|YP_394571.1|	Sulfuromonas denitrificans ATCC 33889	E-value = 3.00E-45	Identity = 45.37%
	2		gi|152991579|ref|YP_001357301.1|	Nitratiruptor sp. SB155-2	E-value = 6.00E-39	Identity = 42.73%
	3		gi|152991635|ref|YP_001357356.1|	Sulfurovum sp. NBC37-1	E-value = 5.00E-37	Identity = 40.17%
	4		gi|34556748|ref|NP_906563.1|	Wolinella succinogenes DSM 1740	E-value = 2.00E-27	Identity = 31.65%
	5		gi|149194780|ref|ZP_01871875.1|	Caminibacter mediatlanticus TB-2	E-value = 9.00E-25	Identity = 35.56%
AB2260	1	*secG*	gi|157165673|ref|YP_001467728.1|	Campylobacter concisus 13826	E-value = 1.00E-23	Identity = 63.21%
	2		gi|34556749|ref|NP_906564.1|	Wolinella succinogenes DSM 1740	E-value = 3.00E-23	Identity = 62.14%
	3		gi|154174058|ref|YP_001409187.1|	Campylobacter curvus 525.92	E-value = 2.00E-22	Identity = 61.54%
	4		gi|149194779|ref|ZP_01871874.1|	Caminibacter mediatlanticus TB-2	E-value = 5.00E-22	Identity = 61.32%
	5		gi|152991580|ref|YP_001357302.1|	Nitratiruptor sp. SB155-2	E-value = 6.00E-20	Identity = 53.10%
AB2261	1	*frr*	gi|152991633|ref|YP_001357354.1|	Sulfurovum sp. NBC37-1	E-value = 1.00E-57	Identity = 69.73%
	2		gi|34556750|ref|NP_906565.1|	Wolinella succinogenes DSM 1740	E-value = 7.00E-55	Identity = 68.48%
	3		gi|154175389|ref|YP_001409189.1|	Campylobacter curvus 525.92	E-value = 2.00E-54	Identity = 63.24%
	4		gi|32265940|ref|NP_859972.1|	Helicobacter hepaticus ATCC 51449	E-value = 1.00E-52	Identity = 62.70%
	5		gi|86153726|ref|ZP_01071929.1|	Campylobacter jejuni subsp. jejuni HB93-13	E-value = 1.00E-50	Identity = 64.32%
AB2262	1	*pyrE*	gi|34556751|ref|NP_906566.1|	Wolinella succinogenes DSM 1740	E-value = 1.00E-76	Identity = 69.31%
	2		gi|78778262|ref|YP_394577.1|	Sulfuromonas denitrificans ATCC 33889	E-value = 6.00E-76	Identity = 72.28%
	3		gi|149194776|ref|ZP_01871871.1|	Caminibacter mediatlanticus TB-2	E-value = 2.00E-71	Identity = 66.50%
	4		gi|152991583|ref|YP_001357305.1|	Nitratiruptor sp. SB155-2	E-value = 4.00E-71	Identity = 67.82%
	5		gi|154148370|ref|YP_001405818.1|	Campylobacter hominis ATCC BAA-381	E-value = 2.00E-69	Identity = 64.36%
AB2263	1		gi|78778112|ref|YP_394427.1|	Sulfuromonas denitrificans ATCC 33889	E-value = 6.00E-25	Identity = 43.62%
	2		gi|152991584|ref|YP_001357306.1|	Nitratiruptor sp. SB155-2	E-value = 3.00E-21	Identity = 36.71%
	3		gi|154174187|ref|YP_001409192.1|	Campylobacter curvus 525.92	E-value = 1.00E-19	Identity = 38.52%
	4		gi|157165170|ref|YP_001465932.1|	Campylobacter concisus 13826	E-value = 3.00E-19	Identity = 41.73%
	5		gi|118474486|ref|YP_892894.1|	Campylobacter fetus subsp. fetus 82-40	E-value = 2.00E-17	Identity = 39.37%
AB2264	1		gi|152991615|ref|YP_001357336.1|	Sulfurovum sp. NBC37-1	E-value = 4.00E-76	Identity = 49.28%
	2		gi|152991585|ref|YP_001357307.1|	Nitratiruptor sp. SB155-2	E-value = 3.00E-74	Identity = 51.84%
	3		gi|78776246|ref|YP_392561.1|	Sulfuromonas denitrificans ATCC 33889	E-value = 2.00E-60	Identity = 46.72%
	4		gi|34556753|ref|NP_906568.1|	Wolinella succinogenes DSM 1740	E-value = 2.00E-57	Identity = 41.41%
	5		gi|149194774|ref|ZP_01871869.1|	Caminibacter mediatlanticus TB-2	E-value = 8.00E-36	Identity = 36.69%
AB2265	1		gi|114777071|ref|ZP_01452091.1|	Mariprofundus ferrooxydans PV-1	E-value = 2.00E-85	Identity = 47.62%
	2		gi|78485280|ref|YP_391205.1|	Thiomicrospira crunogena XCL-2	E-value = 1.00E-82	Identity = 49.68%
	3		gi|90019774|ref|YP_525601.1|	Saccharophagus degradans 2-40	E-value = 1.00E-81	Identity = 47.20%
	4		gi|119946455|ref|YP_944135.1|	Psychromonas ingrahamii 37	E-value = 3.00E-75	Identity = 49.31%
	5		gi|88703321|ref|ZP_01101037.1|	gamma proteobacterium KT 71	E-value = 2.00E-70	Identity = 40.13%
AB2266	1		gi|152991718|ref|YP_001357439.1|	Sulfurovum sp. NBC37-1	E-value = 5.00E-63	Identity = 51.43%
	2		gi|118475086|ref|YP_891818.1|	Campylobacter fetus subsp. fetus 82-40	E-value = 6.00E-63	Identity = 56.56%
	3		gi|154175413|ref|YP_001408156.1|	Campylobacter curvus 525.92	E-value = 2.00E-57	Identity = 53.06%
	4		gi|148926103|ref|ZP_01809789.1|	Campylobacter jejuni subsp. jejuni CG8486	E-value = 2.00E-55	Identity = 51.23%
	5		gi|157415243|ref|YP_001482499.1|	Campylobacter jejuni subsp. jejuni 81116	E-value = 3.00E-55	Identity = 51.23%
AB2267	1		gi|118474351|ref|YP_891700.1|	Campylobacter fetus subsp. fetus 82-40	E-value = 7.00E-07	Identity = 37.84%
AB2268	1		gi|118579675|ref|YP_900925.1|	Pelobacter propionicus DSM 2379	E-value = 3.00E-26	Identity = 34.96%
	2		gi|51244008|ref|YP_063892.1|	Desulfotalea psychrophila LSv54	E-value = 3.00E-22	Identity = 29.92%

	3		gi|85375738|ref|YP_459800.1|	Erythrobacter litoralis HTCC2594	E-value = 6.00E-13	Identity = 26.33%
	4		gi|157164909|ref|YP_001466115.1|	Campylobacter concisus 13826	E-value = 9.00E-13	Identity = 28.90%
	5		gi|121635158|ref|YP_975403.1|	Neisseria meningitidis FAM18	E-value = 4.00E-11	Identity = 25.36%
AB2270	1		gi|78776752|ref|YP_393067.1|	Sulfuromonas denitrificans ATCC 33889	E-value = 2.00E-100	Identity = 54.45%
	2		gi|154173625|ref|YP_001407928.1|	Campylobacter curvus 525.92	E-value = 3.00E-69	Identity = 42.45%
	3		gi|157164813|ref|YP_001466429.1|	Campylobacter concisus 13826	E-value = 4.00E-69	Identity = 41.32%
	4		gi|152992250|ref|YP_001357971.1|	Sulfurovum sp. NBC37-1	E-value = 8.00E-69	Identity = 43.23%
	5		gi|109947685|ref|YP_664913.1|	Helicobacter acinonychis str. Sheeba	E-value = 3.00E-64	Identity = 38.30%
AB2271	1			***** No matches found *****		
AB2272	1		gi|119875208|ref|ZP_01642330.1|	Stenotrophomonas maltophilia R551-3	E-value = 0	Identity = 46.22%
	2		gi|26989309|ref|NP_744734.1|	Pseudomonas putida KT2440	E-value = 1.00E-178	Identity = 46.84%
	3		gi|104781601|ref|YP_608099.1|	Pseudomonas entomophila L48	E-value = 2.00E-178	Identity = 45.88%
	4		gi|119875214|ref|ZP_01642336.1|	Stenotrophomonas maltophilia R551-3	E-value = 3.00E-170	Identity = 43.30%
	5		gi|118050189|ref|ZP_01518739.1|	Comamonas testosteroni KF-1	E-value = 1.00E-153	Identity = 43.76%
AB2273	1		gi|34557928|ref|NP_907743.1|	Wolinella succinogenes DSM 1740	E-value = 9.00E-28	Identity = 30.28%
	2		gi|118475050|ref|YP_892365.1|	Campylobacter fetus subsp. fetus 82-40	E-value = 6.00E-18	Identity = 30.45%
AB2274	1		gi|78486361|ref|YP_392286.1|	Thiomicrospira crunogena XCL-2	E-value = 2.00E-33	Identity = 56.30%
	2		gi|34557661|ref|NP_907476.1|	Wolinella succinogenes DSM 1740	E-value = 5.00E-31	Identity = 52.52%
	3		gi|78776713|ref|YP_393028.1|	Sulfuromonas denitrificans ATCC 33889	E-value = 6.00E-31	Identity = 60.33%
	4		gi|152991974|ref|YP_001357695.1|	Sulfurovum sp. NBC37-1	E-value = 1.00E-29	Identity = 58.59%
	5		gi|154149081|ref|YP_001406197.1|	Campylobacter hominis ATCC BAA-381	E-value = 4.00E-24	Identity = 46.72%
AB2275	1		gi|89093650|ref|ZP_01166597.1|	Oceanospirillum sp. MED92	E-value = 3.00E-18	Identity = 40.74%
	2		gi|156718720|ref|ZP_02060380.1|	Hydrogenobaculum sp. Y04AAS1	E-value = 2.00E-17	Identity = 36.76%
	3		gi|86156811|ref|YP_463596.1|	Anaeromyxobacter dehalogenans 2CP-C	E-value = 3.00E-14	Identity = 32.84%
	4		gi|153005908|ref|YP_001380233.1|	Anaeromyxobacter sp. Fw109-5	E-value = 2.00E-12	Identity = 33.57%
	5		gi|116234997|dbj|BAF34936.1|	Hydrogenobacter thermophilus	E-value = 3.00E-12	Identity = 33.08%
AB2276	1		gi|34557576|ref|NP_907391.1|	Wolinella succinogenes DSM 1740	E-value = 2.00E-75	Identity = 31.05%
	2		gi|121528909|ref|ZP_01661522.1|	Ralstonia pickettii 12J	E-value = 3.00E-57	Identity = 28.71%
	3		gi|153886064|ref|ZP_02007219.1|	Ralstonia pickettii 12D	E-value = 7.00E-52	Identity = 28.08%
	4		gi|57505516|ref|ZP_00371443.1|	Campylobacter upsaliensis RM3195	E-value = 3.00E-46	Identity = 29.45%
	5		gi|88797356|ref|ZP_01112946.1|	Reinekea sp. MED297	E-value = 8.00E-46	Identity = 25.86%
AB2277	1	*moeB*	gi|108803586|ref|YP_643523.1|	Rubrobacter xylanophilus DSM 9941	E-value = 2.00E-123	Identity = 59.19%
	2		gi|153006447|ref|YP_001380772.1|	Anaeromyxobacter sp. Fw109-5	E-value = 2.00E-117	Identity = 55.59%
	3		gi|116619883|ref|YP_822039.1|	Solibacter usitatus Ellin6076	E-value = 2.00E-117	Identity = 57.30%
	4		gi|156740340|ref|YP_001430469.1|	Roseiflexus castenholzii DSM 13941	E-value = 2.00E-115	Identity = 54.30%
	5		gi|148654884|ref|YP_001275089.1|	Roseiflexus sp. RS-1	E-value = 2.00E-115	Identity = 55.16%
AB2278	1		gi|118496649|ref|YP_897699.1|	Francisella tularensis subsp. novicida U112	E-value = 0	Identity = 82.03%
	2		gi|151569765|gb|EDN35419.1|	Francisella tularensis subsp. novicida GA99-3549	E-value = 0	Identity = 82.03%
	3		gi|151572900|gb|EDN38554.1|	Francisella tularensis subsp. novicida GA99-3548	E-value = 0	Identity = 81.94%
	4		gi|115314014|ref|YP_762737.1|	Francisella tularensis subsp. holarctica OSU18	E-value = 0	Identity = 81.14%
	5		gi|134252748|gb|EBA51842.1|	Francisella tularensis subsp. holarctica 257	E-value = 0	Identity = 80.96%
AB2279	1		gi|86141600|ref|ZP_01060146.1|	Flavobacterium sp. MED217	E-value = 0	Identity = 44.30%
	2		gi|126645228|ref|ZP_01717772.1|	Algoriphagus sp. PR1	E-value = 5.00E-168	Identity = 39.76%
	3		gi|152995018|ref|YP_001339853.1|	Marinomonas sp. MWYL1	E-value = 4.00E-138	Identity = 35.40%
	4		gi|90022740|ref|YP_528567.1|	Saccharophagus degradans 2-40	E-value = 1.00E-136	Identity = 34.30%
	5		gi|83643858|ref|YP_432293.1|	Hahella chejuensis KCTC 2396	E-value = 3.00E-133	Identity = 33.41%
AB2280	1		gi|86141601|ref|ZP_01060147.1|	Flavobacterium sp. MED217	E-value = 5.00E-79	Identity = 48.63%
	2		gi|152995017|ref|YP_001339852.1|	Marinomonas sp. MWYL1	E-value = 2.00E-57	Identity = 37.63%
	3		gi|91789021|ref|YP_549973.1|	Polaromonas sp. JS666	E-value = 5.00E-53	Identity = 35.92%

	4		gi|126645229|ref|ZP_01717773.1|	Algoriphagus sp. PR1	E-value = 7.00E-53	Identity = 40.27%
	5		gi|87308153|ref|ZP_01090295.1|	Blastopirellula marina DSM 3645	E-value = 7.00E-53	Identity = 38.05%
AB2281	1	*cysA*	gi|91774964|ref|YP_544720.1|	Methylobacillus flagellatus KT	E-value = 8.00E-111	Identity = 57.70%
	2		gi|118050244|ref|ZP_01518794.1|	Comamonas testosteroni KF-1	E-value = 3.00E-103	Identity = 52.24%
	3		gi|114331024|ref|YP_747246.1|	Nitrosomonas eutropha C91	E-value = 3.00E-103	Identity = 54.34%
	4		gi|148265907|ref|YP_001232613.1|	Geobacter uraniumreducens Rf4	E-value = 4.00E-103	Identity = 54.06%
	5		gi|71909302|ref|YP_286889.1|	Dechloromonas aromatica RCB	E-value = 2.00E-102	Identity = 55.65%
AB2282	1	*cysW*	gi|110600889|ref|ZP_01389097.1|	Geobacter sp. FRC-32	E-value = 2.00E-95	Identity = 66.79%
	2		gi|154252521|ref|YP_001413345.1|	Parvibaculum lavamentivorans DS-1	E-value = 1.00E-94	Identity = 72.24%
	3		gi|91788245|ref|YP_549197.1|	Polaromonas sp. JS666	E-value = 2.00E-94	Identity = 68.66%
	4		gi|152980950|ref|YP_001352503.1|	Janthinobacterium sp. Marseille	E-value = 5.00E-94	Identity = 68.13%
	5		gi|148265908|ref|YP_001232614.1|	Geobacter uraniumreducens Rf4	E-value = 5.00E-93	Identity = 65.94%
AB2283	1	*cysT*	gi|152980049|ref|YP_001352502.1|	Janthinobacterium sp. Marseille	E-value = 5.00E-92	Identity = 68.89%
	2		gi|37521642|ref|NP_925019.1|	Gloeobacter violaceus PCC 7421	E-value = 2.00E-91	Identity = 70.52%
	3		gi|73541658|ref|YP_296178.1|	Ralstonia eutropha JMP134	E-value = 2.00E-91	Identity = 66.67%
	4		gi|118729596|ref|ZP_01578113.1|	Delftia acidovorans SPH-1	E-value = 1.00E-90	Identity = 66.54%
	5		gi|145619608|ref|ZP_01775655.1|	Geobacter bemidjiensis Bem	E-value = 4.00E-89	Identity = 65.66%
AB2284	1	*cysK2*	gi|78776355|ref|YP_392670.1|	Sulfuromonas denitrificans ATCC 33889	E-value = 3.00E-111	Identity = 68.61%
	2		gi|78776634|ref|YP_392949.1|	Sulfuromonas denitrificans ATCC 33889	E-value = 2.00E-105	Identity = 66.67%
	3		gi|118474133|ref|YP_892091.1|	Campylobacter fetus subsp. fetus 82-40	E-value = 2.00E-102	Identity = 63.73%
	4		gi|125974344|ref|YP_001038254.1|	Clostridium thermocellum ATCC 27405	E-value = 2.00E-102	Identity = 66.12%
	5		gi|34557557|ref|NP_907372.1|	Wolinella succinogenes DSM 1740	E-value = 3.00E-101	Identity = 67.53%
AB2285	1		gi|34557353|ref|NP_907168.1|	Wolinella succinogenes DSM 1740	E-value = 8.00E-50	Identity = 32.67%
	2		gi|85711301|ref|ZP_01042360.1|	Idiomarina baltica OS145	E-value = 1.00E-47	Identity = 30.90%
	3		gi|56460645|ref|YP_155926.1|	Idiomarina loihiensis L2TR	E-value = 1.00E-41	Identity = 30.08%
	4		gi|78776934|ref|YP_393249.1|	Sulfuromonas denitrificans ATCC 33889	E-value = 3.00E-31	Identity = 30.85%
	5		gi|90418505|ref|ZP_01226417.1|	Aurantimonas sp. SI85-9A1	E-value = 2.00E-27	Identity = 25.64%
AB2286	1		gi|116619884|ref|YP_822040.1|	Solibacter usitatus Ellin6076	E-value = 2.00E-13	Identity = 45.98%
	2		gi|108803587|ref|YP_643524.1|	Rubrobacter xylanophilus DSM 9941	E-value = 3.00E-13	Identity = 40.23%
	3		gi|91204446|emb|CAJ70946.1|	Candidatus Kuenenia stuttgartiensis	E-value = 1.00E-11	Identity = 44.05%
	4		gi|108761704|ref|YP_632245.1|	Myxococcus xanthus DK 1622	E-value = 2.00E-10	Identity = 34.48%
	5		gi|37522982|ref|NP_926359.1|	Gloeobacter violaceus PCC 7421	E-value = 3.00E-10	Identity = 37.65%
AB2287	1	*sbp*	gi|78223113|ref|YP_384860.1|	Geobacter metallireducens GS-15	E-value = 4.00E-119	Identity = 66.15%
	2		gi|92119020|ref|YP_578749.1|	Nitrobacter hamburgensis X14	E-value = 2.00E-118	Identity = 66.16%
	3		gi|125975016|ref|YP_001038926.1|	Clostridium thermocellum ATCC 27405	E-value = 6.00E-118	Identity = 62.43%
	4		gi|148265910|ref|YP_001232616.1|	Geobacter uraniumreducens Rf4	E-value = 2.00E-117	Identity = 68.42%
	5		gi|91774975|ref|YP_544731.1|	Methylobacillus flagellatus KT	E-value = 5.00E-117	Identity = 69.97%
AB2288	1		gi|154252525|ref|YP_001413349.1|	Parvibaculum lavamentivorans DS-1	E-value = 1.00E-23	Identity = 42.96%
	2		gi|84702034|ref|ZP_01016609.1|	Parvularcula bermudensis HTCC2503	E-value = 2.00E-22	Identity = 41.91%
	3		gi|146299648|ref|YP_001194239.1|	Flavobacterium johnsoniae UW101	E-value = 1.00E-21	Identity = 41.67%
	4		gi|83592507|ref|YP_426259.1|	Rhodospirillum rubrum ATCC 11170	E-value = 2.00E-21	Identity = 40.69%
	5		gi|16126861|ref|NP_421425.1|	Caulobacter crescentus CB15	E-value = 9.00E-21	Identity = 40.31%
AB2289	1		gi|150019157|ref|YP_001311411.1|	Clostridium beijerinckii NCIMB 8052	E-value = 1.00E-49	Identity = 33.23%
	2		gi|106886953|ref|ZP_01354278.1|	Clostridium phytofermentans ISDg	E-value = 6.00E-47	Identity = 32.79%
	3		gi|156863129|gb|EDO56560.1|	Clostridium sp. L2-50	E-value = 6.00E-42	Identity = 31.86%
	4		gi|118746199|ref|ZP_01594149.1|	Geobacter lovleyi SZ	E-value = 3.00E-28	Identity = 30.29%
AB2290	1		gi|150019158|ref|YP_001311412.1|	Clostridium beijerinckii NCIMB 8052	E-value = 4.00E-78	Identity = 48.84%
	2		gi|106886954|ref|ZP_01354279.1|	Clostridium phytofermentans ISDg	E-value = 2.00E-77	Identity = 46.15%
	3		gi|118746200|ref|ZP_01594150.1|	Geobacter lovleyi SZ	E-value = 2.00E-77	Identity = 46.33%

	4		gi|156863130|gb|EDO56561.1|	Clostridium sp. L2-50	E-value = 3.00E-67	Identity = 37.86%
	5		gi|37523548|ref|NP_926925.1|	Gloeobacter violaceus PCC 7421	E-value = 1.00E-40	Identity = 32.27%
AB2291	1			***** No matches found *****		
AB2292	1		gi|118746203|ref|ZP_01594153.1|	Geobacter lovleyi SZ	E-value = 7.00E-108	Identity = 45.14%
	2		gi|116621617|ref|YP_823773.1|	Solibacter usitatus Ellin6076	E-value = 2.00E-105	Identity = 44.78%
	3		gi|126648767|ref|ZP_01721250.1|	Algoriphagus sp. PR1	E-value = 4.00E-105	Identity = 45.21%
	4		gi|151570847|gb|EDN36501.1|	Francisella tularensis subsp. novicida GA99-3549	E-value = 8.00E-105	Identity = 44.87%
	5		gi|118497762|ref|YP_898812.1|	Francisella tularensis subsp. novicida U112	E-value = 8.00E-105	Identity = 44.87%
AB2293	1	*typA*	gi|118474951|ref|YP_891223.1|	Campylobacter fetus subsp. fetus 82-40	E-value = 0	Identity = 76.08%
	2		gi|154175347|ref|YP_001407314.1|	Campylobacter curvus 525.92	E-value = 0	Identity = 76.46%
	3		gi|34558070|ref|NP_907885.1|	Wolinella succinogenes DSM 1740	E-value = 0	Identity = 75.67%
	4		gi|154148859|ref|YP_001407268.1|	Campylobacter hominis ATCC BAA-381	E-value = 0	Identity = 74.75%
	5		gi|157165061|ref|YP_001466014.1|	Campylobacter concisus 13826	E-value = 0	Identity = 75.79%
AB2294	1		gi|78777028|ref|YP_393343.1|	Sulfuromonas denitrificans ATCC 33889	E-value = 3.00E-150	Identity = 73.37%
	2		gi|146311916|ref|YP_001176990.1|	Enterobacter sp. 638	E-value = 2.00E-132	Identity = 60.97%
	3		gi|156933465|ref|YP_001437381.1|	Enterobacter sakazakii ATCC BAA-894	E-value = 3.00E-131	Identity = 60.56%
	4		gi|16765303|ref|NP_460918.1|	Salmonella typhimurium LT2	E-value = 7.00E-130	Identity = 60.71%
	5		gi|56413122|ref|YP_150197.1|	Salmonella enterica subsp. enterica serovar Paratyphi A str. ATCC 9150	E-value = 8.00E-130	Identity = 60.71%
AB2295	1		gi|20150407|pdb|1JE3|A	Escherichia coli	E-value = 5.00E-26	Identity = 78.38%
	2		gi|157368744|ref|YP_001476733.1|	Serratia proteamaculans 568	E-value = 6.00E-26	Identity = 75.95%
	3		gi|75208822|ref|ZP_00709106.1|	Escherichia coli B171	E-value = 4.00E-25	Identity = 79.73%
	4		gi|15802365|ref|NP_288391.1|	Escherichia coli O157:H7 EDL933	E-value = 4.00E-25	Identity = 78.38%
	5		gi|156933464|ref|YP_001437380.1|	Enterobacter sakazakii ATCC BAA-894	E-value = 5.00E-25	Identity = 76.32%
AB2296	1		gi|34557011|ref|NP_906826.1|	Wolinella succinogenes DSM 1740	E-value = 1.00E-41	Identity = 58.33%
AB2297	1		gi|34557009|ref|NP_906824.1|	Wolinella succinogenes DSM 1740	E-value = 7.00E-06	Identity = 36.08%
AB2298	1		gi|34557010|ref|NP_906825.1|	Wolinella succinogenes DSM 1740	E-value = 4.00E-153	Identity = 50.29%
AB2299	1		gi|104781634|ref|YP_608132.1|	Pseudomonas entomophila L48	E-value = 9.00E-121	Identity = 34.54%
	2		gi|53986775|gb|AAV27209.1|	Azospirillum brasilense	E-value = 2.00E-119	Identity = 34.75%
	3		gi|152981197|ref|YP_001355173.1|	Janthinobacterium sp. Marseille	E-value = 1.00E-117	Identity = 35.25%
	4		gi|148548404|ref|YP_001268506.1|	Pseudomonas putida F1	E-value = 8.00E-116	Identity = 34.18%
	5		gi|83592776|ref|YP_426528.1|	Rhodospirillum rubrum ATCC 11170	E-value = 6.00E-114	Identity = 36.19%
AB2300	1		gi|34557007|ref|NP_906822.1|	Wolinella succinogenes DSM 1740	E-value = 4.00E-47	Identity = 35.74%
	2		gi|34557769|ref|NP_907584.1|	Wolinella succinogenes DSM 1740	E-value = 2.00E-28	Identity = 30.23%
	3		gi|90415626|ref|ZP_01223560.1|	marine gamma proteobacterium HTCC2207	E-value = 5.00E-24	Identity = 26.75%
	4		gi|114332056|ref|YP_748278.1|	Nitrosomonas eutropha C91	E-value = 2.00E-21	Identity = 26.17%
	5		gi|30250073|ref|NP_842143.1|	Nitrosomonas europaea ATCC 19718	E-value = 4.00E-21	Identity = 25.15%
AB2301	1		gi|34557006|ref|NP_906821.1|	Wolinella succinogenes DSM 1740	E-value = 6.00E-22	Identity = 37.09%
	2		gi|116496542|gb|ABJ99054.1|	uncultured bacterium	E-value = 6.00E-16	Identity = 31.61%
	3		gi|15596497|ref|NP_249991.1|	Pseudomonas aeruginosa PAO1	E-value = 1.00E-15	Identity = 31.61%
	4		gi|49081662|gb|AAT50231.1|	synthetic construct	E-value = 1.00E-15	Identity = 31.61%
	5		gi|116496538|gb|ABJ99052.1|	uncultured bacterium	E-value = 1.00E-15	Identity = 31.61%
AB2302	1			***** No matches found *****		
AB2303	1		gi|118602906|ref|YP_904121.1|	Candidatus Ruthia magnifica str. Cm (Calyptogena magnifica)	E-value = 4.00E-147	Identity = 50.75%
	2		gi|149926525|ref|ZP_01914786.1|	Limnobacter sp. MED105	E-value = 2.00E-133	Identity = 47.35%
	3		gi|152994711|ref|YP_001339546.1|	Marinomonas sp. MWYL1	E-value = 6.00E-122	Identity = 42.67%
	4		gi|77459378|ref|YP_348885.1|	Pseudomonas fluorescens PfO-1	E-value = 6.00E-122	Identity = 41.30%
	5		gi|117923656|ref|YP_864273.1|	Magnetococcus sp. MC-1	E-value = 6.00E-119	Identity = 44.44%
AB2304	1		gi|78776522|ref|YP_392837.1|	Sulfuromonas denitrificans ATCC 33889	E-value = 1.00E-43	Identity = 45.25%
	2		gi|153893049|ref|ZP_02013856.1|	Opitutaceae bacterium TAV2	E-value = 9.00E-27	Identity = 33.80%

	3		gi|148652851|ref|YP_001279944.1|	Psychrobacter sp. PRwf-1	E-value = 4.00E-23	Identity = 31.78%
	4		gi|146328888|ref|YP_001209439.1|	Dichelobacter nodosus VCS1703A	E-value = 2.00E-22	Identity = 32.04%
	5		gi|71065723|ref|YP_264450.1|	Psychrobacter arcticus 273-4	E-value = 2.00E-21	Identity = 31.78%
AB2305	1		gi|28974231|gb|AAO64232.1|	Campylobacter fetus	E-value = 3.00E-35	Identity = 58.54%
	2		gi|118474342|ref|YP_891654.1|	Campylobacter fetus subsp. fetus 82-40	E-value = 3.00E-35	Identity = 55.81%
	3		gi|114776377|ref|ZP_01451422.1|	Mariprofundus ferrooxydans PV-1	E-value = 2.00E-17	Identity = 35.56%
	4		gi|89902577|ref|YP_525048.1|	Rhodoferax ferrireducens T118	E-value = 1.00E-16	Identity = 35.34%
	5		gi|118028610|ref|ZP_01500072.1|	Burkholderia phymatum STM815	E-value = 8.00E-16	Identity = 33.33%
AB2306	1		gi|118475626|ref|YP_891653.1|	Campylobacter fetus subsp. fetus 82-40	E-value = 3.00E-37	Identity = 46.99%
	2		gi|28974232|gb|AAO64233.1|	Campylobacter fetus	E-value = 5.00E-37	Identity = 46.39%
	3		gi|113968587|ref|YP_732380.1|	Shewanella sp. MR-4	E-value = 3.00E-27	Identity = 40.56%
	4		gi|114049265|ref|YP_739815.1|	Shewanella sp. MR-7	E-value = 3.00E-27	Identity = 40.56%
	5		gi|117918700|ref|YP_867892.1|	Shewanella sp. ANA-3	E-value = 4.00E-27	Identity = 40.56%
AB2307	1		gi|119775626|ref|YP_928366.1|	Shewanella amazonensis SB2B	E-value = 3.00E-18	Identity = 28.17%
	2		gi|85706266|ref|ZP_01037360.1|	Roseovarius sp. 217	E-value = 5.00E-17	Identity = 31.63%
	3		gi|149194871|ref|ZP_01871965.1|	Caminibacter mediatlanticus TB-2	E-value = 7.00E-17	Identity = 37.27%
	4		gi|88794682|ref|ZP_01110388.1|	Alteromonas macleodii 'Deep ecotype'	E-value = 8.00E-17	Identity = 27.64%
	5		gi|148243933|ref|YP_001220172.1|	Acidiphilium cryptum JF-5	E-value = 4.00E-16	Identity = 29.61%
AB2308	1		gi|109157726|pdb|2FWT|A	Rhodobacter sphaeroides	E-value = 6.00E-09	Identity = 34.74%
AB2309	1			***** No matches found *****		
AB2310	1		gi|149194607|ref|ZP_01871703.1|	Caminibacter mediatlanticus TB-2	E-value = 2.00E-66	Identity = 64.52%
	2		gi|118475242|ref|YP_891651.1|	Campylobacter fetus subsp. fetus 82-40	E-value = 3.00E-50	Identity = 50.00%
	3		gi|152993008|ref|YP_001358729.1|	Sulfurovum sp. NBC37-1	E-value = 2.00E-44	Identity = 42.60%
	4		gi|152991455|ref|YP_001357177.1|	Nitratiruptor sp. SB155-2	E-value = 3.00E-43	Identity = 42.86%
	5		gi|47566185|ref|ZP_00237213.1|	Bacillus cereus G9241	E-value = 5.00E-39	Identity = 35.56%
AB2311	1		gi|118474068|ref|YP_891650.1|	Campylobacter fetus subsp. fetus 82-40	E-value = 2.00E-51	Identity = 37.90%
	2		gi|28974235|gb|AAO64236.1|	Campylobacter fetus	E-value = 1.00E-47	Identity = 36.92%
	3		gi|157163989|ref|YP_001466291.1|	Campylobacter concisus 13826	E-value = 1.00E-36	Identity = 30.10%
	4		gi|154148119|ref|YP_001406702.1|	Campylobacter hominis ATCC BAA-381	E-value = 1.00E-36	Identity = 32.68%
	5		gi|154175055|ref|YP_001407799.1|	Campylobacter curvus 525.92	E-value = 3.00E-36	Identity = 30.46%
AB2312	1		gi|126657899|ref|ZP_01729052.1|	Cyanothece sp. CCY0110	E-value = 4.00E-58	Identity = 46.99%
	2		gi|119488863|ref|ZP_01621825.1|	Lyngbya sp. PCC 8106	E-value = 4.00E-42	Identity = 37.89%
	3		gi|17229480|ref|NP_486028.1|	Nostoc sp. PCC 7120	E-value = 9.00E-41	Identity = 40.39%
	4		gi|75907046|ref|YP_321342.1|	Anabaena variabilis ATCC 29413	E-value = 4.00E-40	Identity = 40.00%
	5		gi|77164767|ref|YP_343292.1|	Nitrosococcus oceani ATCC 19707	E-value = 4.00E-40	Identity = 37.30%
AB2313	1		gi|15792369|ref|NP_282192.1|	Campylobacter jejuni subsp. jejuni NCTC 11168	E-value = 2.00E-18	Identity = 31.82%
	2		gi|57168912|ref|ZP_00368042.1|	Campylobacter coli RM2228	E-value = 6.00E-18	Identity = 29.75%
	3		gi|88713586|ref|ZP_01107668.1|	Flavobacteriales bacterium HTCC2170	E-value = 6.00E-18	Identity = 31.28%
	4		gi|126648803|ref|ZP_01721286.1|	Algoriphagus sp. PR1	E-value = 4.00E-17	Identity = 26.22%
	5		gi|57240657|ref|ZP_00368605.1|	Campylobacter lari RM2100	E-value = 3.00E-16	Identity = 29.41%
AB2314	1			***** No matches found *****		
AB2315	1	*thyX*		***** No matches found *****		
AB2316	1	*glsA*	gi|78061601|ref|YP_371509.1|	Burkholderia sp. 383	E-value = 3.00E-88	Identity = 52.94%
	2		gi|91778970|ref|YP_554178.1|	Burkholderia xenovorans LB400	E-value = 3.00E-88	Identity = 52.46%
	3		gi|118035386|ref|ZP_01506794.1|	Burkholderia phytofirmans PsJN	E-value = 4.00E-88	Identity = 52.46%
	4		gi|116252369|ref|YP_768207.1|	Rhizobium leguminosarum bv. viciae 3841	E-value = 5.00E-88	Identity = 50.49%
	5		gi|34499613|ref|NP_903828.1|	Chromobacterium violaceum ATCC 12472	E-value = 8.00E-88	Identity = 50.16%
AB2317	1		gi|152990839|ref|YP_001356561.1|	Nitratiruptor sp. SB155-2	E-value = 6.00E-44	Identity = 52.26%
	2		gi|152992916|ref|YP_001358637.1|	Sulfurovum sp. NBC37-1	E-value = 5.00E-42	Identity = 54.59%

	3		gi|78777777|ref|YP_394092.1|	Sulfuromonas denitrificans ATCC 33889	E-value = 3.00E-41	Identity = 52.26%
	4		gi|118474290|ref|YP_892096.1|	Campylobacter fetus subsp. fetus 82-40	E-value = 1.00E-39	Identity = 44.33%
	5		gi|34558238|ref|NP_908053.1|	Wolinella succinogenes DSM 1740	E-value = 2.00E-39	Identity = 47.03%
AB2318	1		gi|152992667|ref|YP_001358388.1|	Sulfurovum sp. NBC37-1	E-value = 2.00E-17	Identity = 39.22%
	2		gi|118074974|ref|ZP_01543123.1|	Shewanella woodyi ATCC 51908	E-value = 2.00E-13	Identity = 34.48%
	3		gi|116052565|ref|YP_792880.1|	Pseudomonas aeruginosa UCBPP-PA14	E-value = 8.00E-13	Identity = 31.08%
	4		gi|157373599|ref|YP_001472199.1|	Shewanella sediminis HAW-EB3	E-value = 9.00E-13	Identity = 32.26%
	5		gi|15599729|ref|NP_253223.1|	Pseudomonas aeruginosa PAO1	E-value = 4.00E-12	Identity = 30.34%
AB2319	1	*purN*	gi|78777814|ref|YP_394129.1|	Sulfuromonas denitrificans ATCC 33889	E-value = 9.00E-45	Identity = 56.32%
	2		gi|20089214|ref|NP_615289.1|	Methanosarcina acetivorans C2A	E-value = 7.00E-33	Identity = 42.02%
	3		gi|68550499|ref|ZP_00589946.1|	Pelodictyon phaeoclathratiforme BU-1	E-value = 5.00E-31	Identity = 36.79%
	4		gi|150003621|ref|YP_001298365.1|	Bacteroides vulgatus ATCC 8482	E-value = 2.00E-30	Identity = 42.54%
	5		gi|73668823|ref|YP_304838.1|	Methanosarcina barkeri str. Fusaro	E-value = 2.00E-30	Identity = 41.49%
AB2320	1	*ruvC*	gi|154175236|ref|YP_001409235.1|	Campylobacter curvus 525.92	E-value = 1.00E-57	Identity = 74.68%
	2		gi|57504596|ref|ZP_00370708.1|	Campylobacter coli RM2228	E-value = 6.00E-57	Identity = 74.68%
	3		gi|157415948|ref|YP_001483204.1|	Campylobacter jejuni subsp. jejuni 81116	E-value = 7.00E-57	Identity = 74.68%
	4		gi|34558502|ref|NP_908317.1|	Wolinella succinogenes DSM 1740	E-value = 8.00E-57	Identity = 71.61%
	5		gi|57236914|ref|YP_179867.1|	Campylobacter jejuni subsp. jejuni RM1221	E-value = 1.00E-56	Identity = 74.03%
AB2321	1		gi|57167875|ref|ZP_00367015.1|	Campylobacter coli RM2228	E-value = 2.00E-14	Identity = 44.66%
	2		gi|57242372|ref|ZP_00370311.1|	Campylobacter upsaliensis RM3195	E-value = 5.00E-14	Identity = 44.34%
	3		gi|15792289|ref|NP_282112.1|	Campylobacter jejuni subsp. jejuni NCTC 11168	E-value = 3.00E-13	Identity = 43.69%
	4		gi|86153103|ref|ZP_01071308.1|	Campylobacter jejuni subsp. jejuni HB93-13	E-value = 4.00E-13	Identity = 42.72%
	5		gi|57237788|ref|YP_179036.1|	Campylobacter jejuni subsp. jejuni RM1221	E-value = 5.00E-13	Identity = 43.69%
