# Supplementary material for: The Complete Genome Sequence and Analysis of the Epsilonproteobacterium Arcobacter butzleri
Source: PLoS One. 2007 Dec 26;2(12):e1358. doi: 10.1371/journal.pone.0001358 (PMC2147049; doi:10.1371/journal.pone.0001358)
Supplement: Table S4 — Antibiotic resistance profiles of strain RM4018 and selected Campylobacters. Comparison of the antibiotic resistance profiles of A. butzleri strain RM4018, C. jejuni strain NCTC 11168, C. coli strain RM2228, C. lari strain RM2100 and C. upsaliensis strain RM3195. (0.02 MB PDF) [file pone.0001358.s006.pdf]

Table S4. Antibiotic resistance profiles of strain RM4018 and selected Campylobacters.

Antibiotic	Class[a]	Conc. (mg/L)	*Ab*	*Cj*	*Cc*	*Cl*	*Cu*
Novobiocin	AC	30	R	S	S	R	R
Amikacin	AG	30	S	S	I	S	S
Gentamicin	AG	10	S	S	S	S	S
Kanamycin	AG	30	S	S	R	S	S
Neomycin	AG	30	S	S	R	S	S
Netilmicin	AG	30	S	S	S	S	S
Streptomycin	AG	300	S	S	S	S	S
Tobramycin	AG	10	S	S	S	S	S
Cefaclor	C	30	R	S	S	S	S
Cefamandole	C	30	R	R	R	R	S
Cefazolin	C	30	R	R	R	R	S
Cefepime	C	30	R	S	S	S	S
Cefixime	C	5	R	R	R	R	S
Cefmetazole	C	30	R	R	R	I	S
Cefonicid	C	30	R	R	R	R	S
Cefoperazone	C	75	R	R	R	I	S
Cefotaxime	C	30	R	S	S	I	ND
Cefotetan	C	30	R	R	R	I	S
Cefoxitin	C	30	R	R	R	I	S
Cefpodoxime	C	10	R	R	R	R	S
Cefprozil	C	30	R	R	R	R	S
Ceftazidime	C	30	S	S	R	I	S
Ceftibuten	C	30	R	I	R	I	S
Ceftizoxime	C	30	I	S	S	S	S
Ceftriaxone	C	30	R	I	S	R	S
Cefuroxime	C	30	R	R	R	R	S
Cephalothin	C	30	R	R	R	R	S
Moxalactam	C	30	R	R	R	S	S
Loracarbef	CC	30	R	S	S	S	S
Imipenem	CP	10	S	S	S	S	S
Spectinomycin	CYC	100	I	S	S	S	S
Vancomycin	GP	30	R	R	R	R	R
Azithromycin	M	15	I	S	R	S	S
Clindamycin	M	2	R	S	R	S	S
Erythromycin	M	15	I	S	R	S	S
Tilmicosin	M	15	R	S	R	S	S
Aztreonam	MB	30	R	R	R	R	S
Amdinocillin	P	10	R	S	S	S	S
Ampicillin	P	10	R	S	S	I	S
Carbenicillin	P	100	R	S	S	I	S
Cloxacillin	P	1	R	R	R	R	R
Mezlocillin	P	75	R	R	S	I	S
Nafcillin	P	1	R	R	R	R	R
Oxacillin	P	1	R	R	R	R	R
Penicillin	P	10	R	R	I	R	S
Piperacillin	P	100	R	S	S	R	S
Ticarcillin	P	75	R	S	S	I	S

Cinoxacin	Q	100	I	S	S	R	S
Ciprofloxacin	Q	5	S	S	S	I	S
Enoxacin	Q	10	S	S	S	R	S
Enrofloxacin	Q	5	S	S	S	I	S
Lomefloxacin	Q	10	S	S	S	R	S
Nalidixic acid	Q	30	R	S	S	R	R
Norfloxacin	Q	10	S	S	S	R	S
Ofloxacin	Q	5	S	S	S	R	S
Oxolinic acid	Q	2	R	S	S	R	I
Sparfloxacin	Q	5	S	S	S	R	S
Rifampin	R	5	R	R	R	S	R
Sulfisoxazole	S	0.25	R	S	R	S	S
Triple Sulfa	S	1	R	S	R	S	R
Minocycline	T	30	S	S	R	S	S
Oxytetracycline	T	30	S	S	R	S	R
Tetracycline	T	30	S	S	R	S	I
Chloramphenicol	N	30	R	S	S	S	S
Trimethoprim	N	5	R	R	R	R	R

Strains tested represent five sequenced *Campylobacteraceae* genomes: *Ab*, *A. butzleri* RM4018; *Cj*, *Campylobacter jejuni* NCTC 11168; *Cc*, *Campylobacter coli* RM2228; *Cl*, *Campylobacter lari* RM2100; *Cu*, *Campylobacter upsaliensis* RM3195. Antibiotic resistance data for the *Campylobacter* species is in part from Fouts et al.(Fouts et al., *PLoS Biol* 2005, **3**(1):e15). R: resistant phenotype; S: susceptible phenotype; I: intermediate resistance/susceptibility. ND: not determined.

a. AC: aminocoumarin; AG: aminoglycoside; C: β-lactam cephalosporin; CC: β-lactam carbacephem; CP: carbopenem; CYC: aminocyclitol; GP: glycopeptide; M: macrolide; MB: β-lactam monobactam; P: β-lactam penicillin; Q: quinolone; R: rifampin; S: sulfonamide; T: tetracycline; N: not assigned.
