# Supplementary material for: The Complete Genome Sequence and Analysis of the Epsilonproteobacterium Arcobacter butzleri
Source: PLoS One. 2007 Dec 26;2(12):e1358. doi: 10.1371/journal.pone.0001358 (PMC2147049; doi:10.1371/journal.pone.0001358)
Supplement: Table S6 — CDS contained in all A. butzleri strains examined. Core set of 1676 genes identified in RM4018 and all 12 additional A. butzleri strains by comparative genomic indexing. The functional annotation of each gene is provided. (0.51 MB PDF) [file pone.0001358.s008.pdf]

Table S6. CDS contained in all *A. butzleri* strains examined

Gene Name	Common	Description
AB0001	*dnaA*	Chromosomal replication initiator protein DnaA
AB0002	*dnaN*	DNA polymerase III, beta subunit
AB0003	*gyrB*	DNA gyrase, subunit B
AB0004		Probable thioredoxin reductase
AB0005	*queF*	7-cyano-7-deazaguanine reductase
AB0006		Hypothetical protein
AB0007		Conserved hypothetical protein, putative integral membrane protein
AB0008		Conserved hypothetical protein, possible methyltransferase
AB0009	*modD*	Molybdenum ABC transporter, ATP-binding protein
AB0010	*modB*	Molybdenum ABC transporter, permease protein
AB0011		Conserved hypothetical protein
AB0012	*modA*	Molybdenum ABC transporter, periplasmic molybdate-binding protein
AB0013	*modE*	Molybdenum-binding protein, N-terminal:molybdenum-pterin binding domain
AB0014		Conserved hypothetical membrane protein
AB0015		Transcriptional regulator, LysR family
AB0016		Conserved hypothetical protein, putative MFS permease
AB0018		Ribosomal large subunit pseudouridine synthase
AB0019	*purB*	Adenylosuccinate lyase
AB0020	*nrdA*	Ribonucleoside-diphosphate reductase, alpha chain
AB0021	*nrdB*	Ribonucleoside-diphosphate reductase, beta chain
AB0023		Conserved hypothetical protein
AB0025		Hypothetical protein, OmpA domain
AB0026		Conserved hypothetical protein
AB0027		Zinc-containing alcohol dehydrogenase superfamily protein
AB0028		Conserved hypothetical protein (DUF24 domain protein)
AB0029	*gpsA*	Glycerol 3-phosphate dehydrogenase
AB0030	*gatB*	Glutamyl-tRNA(Gln) amidotransferase, subunit B
AB0031		Conserved hypothetical protein
AB0032		Oxidoreductase, FAD-binding/iron-sulfur cluster-binding protein
AB0033	*lctP*	L-lactate permease
AB0034		Two-component response regulator
AB0035		Two-component sensor histidine kinase
AB0036		Conserved hypothetical protein (DUF162 domain protein)
AB0037		Iron-sulfur cluster binding protein, putative
AB0038		Fe-S oxidoreductase
AB0039		dCMP deaminase, putative
AB0040	*accB*	Acetyl-CoA carboxylase, biotin carboxyl carrier protein
AB0041	*accC1*	Acetyl CoA carboxylase, biotin carboxylase subunit
AB0042		ATP-dependent RNA helicase, DEAD box family
AB0044		Phosphate permease, putative
AB0045		Conserved hypothetical protein, putative permease
AB0047		Hypothetical protein
AB0048		PhoH family protein
AB0049		Conserved hypothetical integral membrane protein
AB0050		ATPase, AAA family protein
AB0051	*rluB*	Ribosomal large subunit pseudouridine synthase B
AB0052		Carbohydrate isomerase, KpsF/GutQ family
AB0053		Conserved hypothetical protein, predicted hydrolase of the metallo-beta-lactamase
AB0054	*ksgA*	Dimethyladenosine transferase
AB0055	*hisF1*	Imidazoleglycerol phosphate synthase, cyclase subunit
AB0056		Conserved hypothetical protein
AB0057		Conserved hypothetical protein, possible purine nucleoside phosphorylase

AB0058		Conserved hypothetical protein, radical SAM enzyme, Cfr family
AB0060	*gltX1*	Glutamyl-tRNA synthetase
AB0061	*rbpA*	RNA-binding region RNP-1 (RNA recognition motif)
AB0062		Two-component response regulator
AB0063		Two-component sensor histidine kinase
AB0064		Conserved hypothetical protein
AB0065		Hypothetical protein
AB0066	*czcB*	Cation efflux system, membrane fusion protein
AB0067	*czcA*	Heavy metal efflux pump
AB0068		Conserved hypothetical periplasmic protein
AB0070		Fibronectin/fibrinogen-binding protein, putative
AB0071		Conserved hypothetical protein
AB0072		Conserved hypothetical protein
AB0073		Hypothetical protein
AB0074	*thrS*	Threonyl-tRNA synthetase
AB0075	*infC*	Translation initiation factor IF-3
AB0076	*rpmI*	50S ribosomal protein L35
AB0077	*rplT*	50S ribosomal protein L20
AB0078		Rhodanese-like sulfur transferase
AB0080		Conserved hypothetical protein, putative sulfide:quinone reductase
AB0081	*mobA*	Molybdopterin-guanine dinucleotide biosynthesis protein
AB0082	*leuC*	3-isopropylmalate dehydratase, large subunit
AB0083	*lspA*	Lipoprotein signal peptidase
AB0084	*glmM*	Phosphoglucosamine mutase
AB0085	*rpsT*	30S ribosomal protein S20
AB0086	*prfA*	Peptide chain release factor 1
AB0088	*pstS*	Phosphate ABC transporter, periplasmic phosphate-binding protein
AB0089		EAL/GGDEF domain protein
AB0090	*pstC*	Phosphate ABC transporter, permease protein
AB0091	*pstA*	Phosphate ABC transporter, permease protein
AB0092	*pstB*	Phosphate transporter, ATP-binding protein
AB0093		Phosphate transport system regulatory protein PhoU, putative
AB0094		Hypothetical protein
AB0095		Two-component response regulator
AB0096		Two-component sensor histidine kinase
AB0097	*rplM*	50S ribosomal protein L13
AB0098	*rpsI*	30S ribosomal protein S9
AB0099	*appA*	Oligopeptide ABC transporter, periplasmic substrate-binding protein
AB0100		Conserved hypothetical protein, HAD-superfamily hydrolase
AB0108		Conserved hypothetical protein, putative ammonia monooxygenase
AB0109		Hypothetical protein
AB0110		ABC transporter, periplasmic substrate-binding protein, putative
AB0111	*iamA*	ABC transporter, ATP-binding protein
AB0112	*iamB*	ABC transporter, permease protein
AB0115	*luxS*	Autoinducer-2 production protein LuxS
AB0116		Hypothetical protein
AB0117		Conserved hypothetical protein (DUF752 domain protein)
AB0118	*dacA*	Serine-type D-Ala-D-Ala carboxypeptidase
AB0119		Conserved hypothetical protein
AB0121	*metH*	5-methyltetrahydrofolate--homocysteine methyltransferase
AB0122	*ilvE*	Branched-chain amino-acid aminotransferase
AB0123		Conserved hypothetical protein, Band 7 family protein
AB0124		Hypothetical protein
AB0125	*hisI*	Phosphoribosyl-AMP cyclohydrolase/ phosphoribosyl-ATP pyrophosphohydrolase
AB0126		Hypothetical protein

AB0127	*mrp*	ATP/GTP-binding protein
AB0128	*thiC*	Thiamine biosynthesis protein ThiC
AB0129		Conserved hypothetical protein
AB0130	*ispDF*	2-C-methyl-D-erythritol 4-phosphate cytidylyl transferase/synthase
AB0131		Two-component response regulator
AB0132	*pgpA*	Phosphatidylglycerophosphatase A
AB0134	*carA*	Carbamoylphosphate synthase, small subunit
AB0135		Conserved hypothetical protein (DUF507 domain protein)
AB0136	*purA*	Adenylosuccinate synthetase
AB0137		Conserved hypothetical protein, putative tRNA synthetase
AB0138		Putative aminotransferase
AB0139		Conserved hypothetical periplasmic protein
AB0142		Conserved hypothetical protein, LigB family protein
AB0143	*pirA*	Pirin
AB0144	*gapB*	Glyceraldehyde-3-phosphate dehydrogenase B
AB0145	*purM*	Phosphoribosylaminoimidazole synthetase
AB0146	*speE*	Spermidine synthase
AB0147	*coaE*	Dephospho-CoA kinase
AB0148	*dapF*	Diaminopimelate epimerase
AB0149	*purT*	Phosphoribosylglycinamide formyltransferase 2
AB0150		Transcriptional regulator, MarR family
AB0151		Hypothetical protein
AB0153	*ubiA*	4-hydroxybenzoate octaprenyltranferase
AB0155	*miaA*	tRNA delta(2)-isopentenylpyrophosphate transferase
AB0156	*rpmE*	50S ribosomal protein L31
AB0157		Conserved hypothetical protein, putative methyltransferase
AB0158		tRNA methyltransferase, TrmH family
AB0159		Conserved hypothetical protein
AB0160	*aspB1*	Aspartate aminotransferase, aminotransferase, classes I and II
AB0161	*hom*	Homoserine dehydrogenase
AB0162	*traT*	TraT complement resistance protein precursor
AB0163	*cdsA*	CDP-diglyceride synthetase CdsA
AB0164	*dxr*	1-deoxy-D-xylulose 5-phosphate reductoisomerase
AB0166		Conserved hypothetical protein (DUF1234 domain protein)
AB0167	*gcp*	O-sialoglycoprotein endopeptidase
AB0168		Hypothetical protein
AB0169		Conserved hypothetical protein, putative carbohydrate kinase
AB0170		Conserved hypothetical periplasmic protein
AB0171	*thiG*	Thiazole biosynthesis protein ThiG
AB0172	*pycB1*	Pyruvate/oxaloacetate carboxyltransferase
AB0173		Hypothetical protein
AB0174		Transcriptional regulator, AraC family
AB0176		Methyl-accepting chemotaxis protein
AB0177		DNA/RNA helicase (DEAD/DEAH BOX family)
AB0178	*tkt*	Transketolase
AB0179	*lrgA*	LrgA family protein
AB0180	*lrgB*	LrgB-like protein
AB0181		Conserved hypothetical protein
AB0183		Conserved hypothetical protein (DUF350 domain protein)
AB0184	*dctA*	C4-dicarboxylate transport protein
AB0185		Hypothetical protein
AB0190	*aas*	2-acylglycerophosphoethanolamine acyltransferase / acyl-acyl carrier protein synthetase
AB0191	*glnD*	Protein-P-II uridylyltransferase
AB0192		Conserved hypothetical protein
AB0193		Conserved hypothetical protein

AB0194		Hypothetical protein, ankyrin repeat family
AB0195	*glnB1*	Nitrogen regulatory protein PII
AB0196	*pyrC*	Dihydroorotase
AB0197	*fliM*	Flagellar motor switch protein FliM
AB0202		Hypothetical protein
AB0203	*fliL*	Flagellar basal body-associated protein
AB0204		Hypothetical protein
AB0205	*flgH*	Flagellar basal body L-ring protein FlgH
AB0206	*flgK*	Flagellar hook-associated protein FlgK
AB0207	*fliD*	Flagellar hook-associated protein 2
AB0208	*fliS*	Flagellar protein FliS
AB0209		Hypothetical protein
AB0210	*tilS*	tRNA(Ile)-lysidine synthase
AB0211	*yliG*	MiaB-like tRNA modifying enzyme
AB0212	*panC*	Pantoate--beta-alanine ligase
AB0213		Oligopeptide ABC transporter, permease protein
AB0214	*acpS*	Holo-(acyl-carrier-protein) synthase
AB0215	*gltS*	Sodium:glutamate symporter
AB0216		Conserved hypothetical protein, putative Holliday junction resolvase
AB0217	*metF*	5,10-methylenetetrahydrofolate reductase
AB0218	*serB*	3-phosphoserine phosphatase
AB0219	*tal*	Transaldolase
AB0220	*dhs*	3-deoxy-D-arabinoheptulosonate 7-phosphate synthase (DAHP synthetase class II)
AB0221	*tsx*	Nucleoside-specific channel-forming protein
AB0224		Conserved hypothetical DNA binding protein
AB0225		Conserved hypothetical protein
AB0226	*gatC*	Glutamyl-tRNA(Gln) amidotransferase, subunit C
AB0227	*tgt*	Queuine tRNA-ribosyltransferase
AB0228		Conserved hypothetical protein
AB0229	*aspB2*	Aspartate aminotransferase, aminotransferase, classes I and II
AB0232	*dprA*	SMF protein, DNA processing chain A
AB0233		Conserved hypothetical protein
AB0234	*ilvC*	Ketol-acid reductoisomerase
AB0235	*rnr*	Ribonuclease R
AB0236		Hypothetical protein (DUF180 domain protein)
AB0237		Conserved hypothetical protein
AB0238	*rpsF*	30S ribosomal protein S6
AB0239	*ssb*	Single-strand DNA binding protein
AB0240	*rpsR*	30S ribosomal protein S18
AB0241		Hypothetical protein
AB0242	*cysS*	Cysteinyl-tRNA synthetase
AB0243	*nusA*	Transcription termination factor NusA
AB0244		Conserved hypothetical protein
AB0245	*miaB*	tRNA-methylthiotransferase
AB0246		Putative lipoprotein
AB0247		Hypothetical protein
AB0248		Conserved hypothetical protein
AB0249		Hypothetical protein
AB0250	*cynT2*	Carbonic anhydrase
AB0251	*bioF*	8-amino-7-oxononanoate synthase
AB0252		Conserved hypothetical protein
AB0253		Conserved hypothetical protein
AB0254		OmpA domain protein
AB0255	*pyk*	Pyruvate kinase
AB0256	*argD1*	N-acetylornithine aminotransferase

AB0257	*cti*	Fatty acid cis/trans isomerase
AB0258	*pyrB*	Aspartate carbamoyltransferase
AB0259	*pabB*	Para-aminobenzoate synthase, glutamine amidotransferase component I
AB0262		Conserved hypothetical protein, putative para-aminobenzoate synthase component I
AB0263	*phnA*	Phosphonoacetate hydrolase
AB0264		Conserved hypothetical protein, predicted metal-dependent hydrolase
AB0267		Hypothetical periplasmic protein
AB0268		Conserved hypothetical protein
AB0269		Conserved hypothetical protein, probable periplasmic protein
AB0271		D-isomer specific 2-hydroxyacid dehydrogenase, NAD-binding
AB0272		Conserved hypothetical protein
AB0273	*prpB*	Carboxyphosphoenolpyruvate phosphomutase PrpB
AB0274	*prpC*	2-methylcitrate synthase/citrate synthase 2
AB0275	*acnA*	Aconitate hydratase 1
AB0276		Conserved hypothetical protein (DUF453 domain protein)
AB0277		Hypothetical protein
AB0278		Isochorismatase hydrolase
AB0280	*hemC*	Porphobilinogen deaminase
AB0281		Conserved hypothetical protein, putative DSBA oxidoreductase
AB0282	*dnaX*	DNA polymerase III, gamma and tau subunits
AB0283	*murI*	Glutamate racemase
AB0284	*gdhA*	NADP-specific glutamate dehydrogenase
AB0285	*rho*	Transcription termination factor Rho
AB0286	*tsaA*	Alkyl hydroperoxide reductase/ Thiol specific antioxidant
AB0287	*fdxA*	Ferredoxin
AB0288	*ndk*	Nucleoside diphosphate kinase
AB0289		Conserved hypothetical protein
AB0290	*rpmF*	50S ribosomal protein L32
AB0291	*plsX*	Fatty acid/phospholipid synthesis protein
AB0292	*fabH*	3-oxoacyl-(acyl carrier protein) synthase III
AB0294		Conserved hypothetical protein, predicted ATP/GTP-binding protein
AB0295		Conserved hypothetical protein, predicted ATP/GTP-binding protein
AB0296	*frdB*	Fumarate reductase, iron-sulfur protein
AB0297	*frdA*	Fumarate reductase, flavoprotein subunit
AB0298	*frdC*	Fumarate reductase, cytochrome b subunit
AB0299	*nuoN*	NADH-quinone oxidoreductase, N subunit
AB0300	*nuoM*	NADH-quinone oxidoreductase, M subunit
AB0301	*nuoL*	NADH-quinone oxidoreductase, L subunit
AB0302	*nuoK*	NADH-quinone oxidoreductase, K subunit
AB0303	*nuoJ*	NADH-quinone oxidoreductase, J subunit
AB0304	*nuoI*	NADH-quinone oxidoreductase, I subunit
AB0305	*nuoH*	NADH-quinone oxidoreductase, H subunit
AB0306	*nuoG*	NADH-quinone oxidoreductase, G subunit
AB0307	*gltA*	Citrate synthase
AB0308	*nuoF*	NADH-quinone oxidoreductase, F subunit
AB0309	*nuoE*	NADH-quinone oxidoreductase, E subunit
AB0310	*nuoCD*	NADH-quinone oxidoreductase, C/D subunit
AB0312	*nuoA*	NADH-quinone oxidoreductase, A subunit
AB0313		Aminotransferase/L-cysteine desulfhydrase
AB0314		Carboxylyase-related protein
AB0315		Conserved hypothetical membrane protein (DUF1212 domain protein)
AB0316		Conserved hypothetical membrane protein (DUF1212 domain protein)
AB0317		Conserved hypothetical protein
AB0318		Transcriptional activator, putative, Baf family
AB0319	*hisG*	ATP phosphoribosyltransferase

AB0320		Conserved hypothetical protein, putative methyltransferase
AB0321		Methyl-accepting chemotaxis protein
AB0324	*trpS*	Tryptophanyl-tRNA synthetase
AB0325		TonB-dependent receptor protein
AB0326		Conserved hypothetical membrane protein
AB0333		Conserved hypothetical membrane protein (DUF204 domain protein)
AB0336		ABC transporter, ATP-binding/permease protein
AB0341		Hypothetical protein
AB0342		Cation efflux system, membrane protein
AB0343	*serS*	Seryl-tRNA synthetase
AB0344	*nrfI*	Cytochrome c biogenesis protein
AB0345	*nrfA*	Cytochrome c552 nitrite reductase catalytic subunit NrfA
AB0346	*nrfH*	Cytochrome c nitrite reductase, small subunit NrfH
AB0347		Hypothetical protein
AB0348		EAL/GGDEF/PAS domain protein
AB0349		Methyl-accepting chemotaxis protein
AB0350	*napD*	Putative periplasmic nitrate reductase assembly protein NapD
AB0351	*napL*	Putative periplasmic protein
AB0352	*napF*	Ferredoxin-type protein NapF
AB0353	*napB*	Periplasmic nitrate reductase, small subunit, cytochrome c-type protein NapB
AB0354	*napH*	Methylamine utilization ferredoxin-type protein NapH
AB0355	*napG*	Fe-S ferredoxin-type protein NapG
AB0356	*napA*	Periplasmic nitrate reductase, large subunit
AB0357	*dctP*	C4-dicarboxylate-binding periplasmic protein
AB0358	*dctQ*	C4-dicarboxylate transport system, permease small subunit
AB0359	*dctM*	C4-dicarboxylate transport protein
AB0360		Two-component sensor histidine kinase
AB0361		Two-component response regulator
AB0362	*menG*	S-adenosylmethionine:2-demethylmenaquinone methyltransferase
AB0363	*cysG*	Uroporphyrin-III C-methyltransferase
AB0364		Conserved hypothetical protein
AB0365		Putative transcriptional regulator
AB0366		Glycosyltransferase, putative
AB0367	*moaA*	Molybdopterin biosynthesis protein A
AB0368	*rpsO*	30S ribosomal protein S15
AB0369		Transcriptional regulator, BadM/Rrf2 family
AB0370		Conserved hypothetical protein
AB0371		CoA-binding domain protein
AB0372		Two-component sensor histidine kinase
AB0373		Two-component response regulator
AB0374	*ppiC*	Peptidyl-prolyl cis-trans isomerase
AB0375	*fba*	Fructose-bisphosphate aldolase
AB0376	*ald*	Aldehyde dehydrogenase
AB0377		Conserved hypothetical protein (DUF779 domain protein)
AB0378	*exbD1*	Biopolymer transport protein ExbD
AB0379	*exbB1*	Biopolymer transport protein ExbB
AB0380		Conserved hypothetical protein
AB0381	*aroK*	Shikimate kinase
AB0382	*hisD*	Histidinol dehydrogenase
AB0383		Conserved hypothetical protein
AB0384	*ispB*	Octaprenyl-diphosphate synthase
AB0385	*hemA*	Glutamyl-tRNA reductase
AB0386	*proS*	Prolyl-tRNA synthetase
AB0387	*mscS*	Mechanosensitive ion channel
AB0388		tRNA nucleotidyltransferase/poly(A) polymerase

AB0389		Conserved hypothetical protein
AB0390		Conserved hypothetical protein
AB0391	*lnt*	Apolipoprotein N-acyltransferase
AB0392	*yajC*	Preprotein translocase subunit
AB0393	*secD*	Protein-export membrane protein SecD
AB0394	*secF*	Protein-export membrane protein SecF
AB0395		Hypothetical protein
AB0396		Hypothetical protein
AB0397	*motB*	Flagellar motor component MotB
AB0398	*motA*	Flagellar motor component MotA
AB0399		Conserved hypothetical protein
AB0400	*leuS*	Leucyl-tRNA synthetase
AB0401		Conserved hypothetical protein
AB0402	*folC*	Folylpolyglutamate synthase/dihydrofolate synthase
AB0403		Probable helicase
AB0404		Phospholipid/glycerol acyltransferase, possible hemolysin
AB0405		Conserved hypothetical protein
AB0406		Hypothetical protein
AB0407		Hypothetical protein
AB0408	*mfd*	Transcription-repair coupling factor
AB0409		Putative acetyltransferase
AB0410		Conserved hypothetical protein
AB0411		Hypothetical periplasmic protein
AB0412	*ompR*	Two-component response regulator
AB0413		Two-component sensor histidine kinase
AB0414		Conserved hypothetical protein
AB0415		Methyl-accepting chemotaxis protein
AB0416		Two-component response regulator
AB0417		Two-component sensor histidine kinase
AB0418		Two-component sensor histidine kinase
AB0419		Conserved hypothetical protein
AB0420		Hypothetical protein
AB0421		Hypothetical protein
AB0422	*ubiE*	Ubiquinone\menaquinone biosynthesis methlytransferase
AB0423	*xseA*	Exodeoxyribonuclease VII, large subunit
AB0425	*cheW*	Chemotaxis protein CheW
AB0426		Peptidyl-prolyl cis-trans isomerase-like protein
AB0427	*panD*	Aspartate 1-decarboxylase
AB0428		Conserved hypothetical protein (DUF149 domain protein)
AB0429	*ispA*	Geranyltranstransferase
AB0431	*groEL*	60 kDa chaperonin
AB0432		Two-component sensor histidine kinase
AB0433		Two-component response regulator
AB0434		Ser/Thr protein phosphatase family protein
AB0435		Conserved hypothetical protein
AB0436		Hypothetical periplasmic protein
AB0437		Hypothetical protein
AB0438		Hypothetical protein
AB0439		ABC transporter, ATP-binding protein
AB0440		ABC transporter, permease protein
AB0442		Hypothetical protein
AB0443	*suhB*	Inositol-1-monophosphatase
AB0444	*glmS*	Glucosamine-fructose-6-phosphate aminotransferase
AB0445	*metK*	S-adenosylmethionine synthetase
AB0446	*accD*	Acetyl-CoA carboxylase, carboxyltransferase, beta subunit

AB0447	*thiE*	Thiamine-phosphate pyrophosphorylase
AB0448		Conserved hypothetical protein (DUF163 domain protein)
AB0449		Conserved hypothetical protein
AB0450		Probable tRNA-dihydrouridine synthase
AB0451	*prmA*	Ribosomal protein L11 methyltransferase
AB0452	*ftsH1*	Cell division protein FtsH
AB0456	*leuA1*	2-isopropylmalate synthase
AB0457		Conserved hypothetical protein
AB0458		Conserved hypothetical protein
AB0459	*mutS1*	Mismatch repair ATPase
AB0465		Quinohemoprotein amine dehydrogenase, 60 kDa (α) subunit
AB0466		Quinohemoprotein amine dehydrogenase, putative quinone cofactor formation protein
AB0467		Quinohemoprotein amine dehydrogenase, 9 kDa (γ) subunit
AB0468		Quinohemoprotein amine dehydrogenase, 40 kDa (β) subunit
AB0469		ABC transporter, ATP-binding protein
AB0470		Conserved hypothetical protein
AB0471		Conserved hypothetical protein
AB0472		GGDEF domain protein
AB0473	*hisA*	Phosphoribosylformimino-5-aminoimidazole carboxamide ribotide isomerase
AB0474		Conserved hypothetical protein (DUF1731 domain protein)
AB0475	*hisH1*	Glutamine amidotransferase HisH
AB0476	*ctsW*	Transformation system protein
AB0479	*lepA*	GTP-binding protein LepA
AB0480		Conserved hypothetical protein (DUF156 domain protein)
AB0481		Heavy-metal transporting P-type ATPase
AB0483	*cadF*	Outer membrane fibronectin-binding protein
AB0484	*prsA*	Ribose-phosphate pyrophosphokinase
AB0485		Conserved hypothetical protein
AB0486	*trmU1*	tRNA (5-methylaminomethyl-2-thiouridylate)-methyltransferase
AB0487	*trmU2*	tRNA (5-methylaminomethyl-2-thiouridylate)-methyltransferase
AB0488	*folK*	2-amino-4-hydroxy-6- hydroxymethyldihydropteridine pyrophosphokinase
AB0489	*pepQ*	Prolidase (Xaa-Pro dipeptidase) (pepQ)
AB0490	*aroQ*	3-dehydroquinate dehydratase
AB0491		Amidohydrolase family protein
AB0492	*pspA*	Protease IV (PspA)
AB0494	*ackA1*	Acetate kinase
AB0495	*pta*	Phosphate acetyltransferase
AB0496	*ackA2*	Acetate kinase
AB0497		Cation efflux protein
AB0498	*ackA3*	Acetate kinase
AB0499	*dnaQ1*	DNA polymerase III, epsilon subunit
AB0500		Conserved hypothetical protein (DUF294 domain protein)
AB0501		Sodium:solute symporter family protein
AB0502		Conserved hypothetical protein (DUF485 domain protein)
AB0504		Sodium:solute symporter family protein
AB0505		Conserved hypothetical protein (DUF485 domain protein)
AB0506	*dnaQ2*	DNA polymerase III, epsilon subunit
AB0507		Cyclic nucleotide-binding domain protein
AB0508		Sodium:solute symporter (Ssf family)
AB0509		Conserved hypothetical protein
AB0510		Two-component response regulator
AB0511		Two-component sensor histidine kinase
AB0512		CinA-like protein
AB0513	*mqo*	Malate:quinone oxidoreductase
AB0514	*ileS*	Isoleucyl-tRNA synthetase

AB0516		Conserved hypothetical NifU-like protein
AB0517		Conserved hypothetical protein
AB0518	*murE*	UDP-N-acetylmuramoylalanyl-D-glutamyl-2, 6-diaminopimelate ligase
AB0520		C4-dicarboxylate transporter/malic acid transport protein
AB0521	*tas*	Oxidoreductase Tas, aldo/keto reductase family
AB0522	*pitA*	Phosphate transporter family protein
AB0523		Methyl-accepting chemotaxis protein
AB0524		Hypothetical protein
AB0525		Methyl-accepting chemotaxis protein
AB0526		Conserved hypothetical protein
AB0527		Conserved hypothetical protein
AB0528		Peptidase, M48 family
AB0529		DNA recombination protein RmuC homolog
AB0530		Integral membrane domain protein (DUF6 domain protein)
AB0531	*moeA2*	Molybdenum cofactor biosynthesis protein A
AB0532		Hypothetical protein
AB0533		Conserved hypothetical protein
AB0534		Conserved hypothetical protein (DUF523 domain protein)
AB0535		Hypothetical protein
AB0536		Hypothetical protein
AB0537	*rpsB*	30S ribosomal protein S2
AB0538	*tsf*	Translation elongation factor EF-Ts
AB0539		ABC transporter, ATP-binding protein
AB0540		Hypothetical protein
AB0541	*gmk*	Guanylate kinase
AB0542		EAL/GGDEF domain protein
AB0543		Cation-transporting ATPase, P-type
AB0544		Hypothetical protein
AB0545		Conserved hypothetical protein, possible cytochrome c-553
AB0546		Oligopeptide ABC transporter, ATP-binding protein
AB0547	*hemH*	Ferrochelatase
AB0548	*feoB1*	Ferrous iron transport protein B
AB0549		Hypothetical protein
AB0550		Conserved hypothetical protein (DUF218 domain protein)
AB0551		ABC transporter, ATP-binding protein
AB0552		ABC transporter, permease protein
AB0553		Conserved hypothetical protein
AB0554	*tpx*	Thiol peroxidase
AB0555		Conserved hypothetical secreted protein
AB0556		Putative efflux protein, bile acid:sodium symporter family
AB0558		Hypothetical protein
AB0562		Probable sodium/hydrogen antiporter
AB0584		Hypothetical protein
AB0585		Major facilitator superfamily transporter
AB0586		HAM1 protein homolog
AB0588		Hypothetical protein
AB0589		Hypothetical protein
AB0590	*proC*	Pyrroline-5-carboxylate reductase
AB0591		Conserved hypothetical membrane protein
AB0592	*lon*	ATP-dependent protease La
AB0593		Rhomboid-like protein
AB0594		Protein tyrosine phosphatase
AB0595		Conserved hypothetical protein
AB0596		Hypothetical TPR repeat protein
AB0597		Transcriptional regulator, MarR family

AB0602		Methyl-accepting chemotaxis protein
AB0603		Conserved hypothetical protein
AB0604		Membrane protein, putative (DUF6 domain protein)
AB0609	*sufD*	Fe-S assembly protein
AB0610	*sufC*	Fe-S assembly ABC transporter, ATP-binding protein
AB0611	*sufB*	Fe-S assembly ABC transporter, permease protein
AB0612	*iscS*	Cysteine desulfurase/aminotransferase (IscS/NifS)
AB0613	*iscU*	NifU-like protein
AB0614		Conserved hypothetical protein
AB0615		GGDEF domain protein
AB0616	*argH*	Argininosuccinate lyase
AB0617	*cheV*	Chemotaxis signal transduction protein CheV
AB0626		TonB-dependent receptor protein
AB0627		Methyl-accepting chemotaxis protein
AB0628		Hypothetical protein
AB0630		Two-component response regulator
AB0631		Two-component sensor histidine kinase
AB0632		Methyl-accepting chemotaxis protein
AB0633		Methyl-accepting chemotaxis protein
AB0634		Conserved hypothetical protein
AB0636	*rpiB*	Ribose 5-phosphate isomerase
AB0637	*lepP*	Signal peptidase I
AB0638	*folD*	5,10-methylene-tetrahydrofolate dehydrogenase/5,10-methylene-tetrahydrofolate
AB0639	*rplY*	50S ribosomal protein L25
AB0640	*pth*	Peptidyl-tRNA hydrolase
AB0641		Conserved hypothetical membrane protein
AB0642		Hypothetical protein
AB0643	*amiA*	N-acetylmuramoyl-L-alanine amidase
AB0644	*npd*	2-nitropropane dioxygenase
AB0645	*tyrS*	Tyrosyl-tRNA synthetase
AB0646	*spoT*	ppGpp synthetase/guanosine-3',5'-bis(diphosphate) 3'-pyrophosphohydrolase
AB0647	*rpoZ*	DNA-directed RNA polymerase, omega chain
AB0648	*pyrH*	Uridylate kinase
AB0649		Conserved hypothetical protein
AB0650	*pdxA*	Pyridoxal phosphate biosynthetic protein A
AB0651	*pdxJ*	Pyridoxal phosphate biosynthesis protein
AB0652	*trpE*	Anthranilate synthase, component I
AB0653		Conserved hypothetical protein
AB0654	*glyA1*	Serine hydroxymethyltransferase
AB0655	*lysS*	Lysyl-tRNA synthetase
AB0656		Colicin V production protein
AB0657	*ispG*	4-hydroxy-3-methylbut-2-en-1-yl diphosphate synthase
AB0658		GGDEF domain protein
AB0659	*dnaB*	Replicative DNA helicase
AB0697	*pglF*	Sugar epimerase/dehydratase
AB0698		Hypothetical protein
AB0699	*ligA*	ATP-dependent DNA ligase
AB0700	*apt*	Adenine phosphoribosyltransferase
AB0701	*trpB1*	Tryptophan synthase, beta chain
AB0702		Conserved hypothetical membrane protein
AB0703	*pepA*	Aminopeptidase
AB0705		TonB-dependent receptor protein
AB0706	*exbD2*	Biopolymer transport protein ExbD
AB0707	*exbB2*	Biopolymer transport protein ExbB
AB0708		Conserved hypothetical membrane protein

AB0709		HlyD family secretion protein
AB0710		ABC transporter, ATP-binding protein
AB0711		ABC transporter, permease protein
AB0712		Hypothetical protein
AB0714		Heavy metal translocating P-type ATPase
AB0715		Conserved hypothetical protein
AB0716		Conserved hypothetical protein
AB0717		Hypothetical protein
AB0718		Conserved hypothetical protein
AB0719		Conserved hypothetical protein
AB0720	*fur1*	Ferric uptake regulation protein
AB0721		Hypothetical protein
AB0722	*fumC*	Fumarate hydratase, class II
AB0723		Hypothetical protein
AB0724		Two-component sensor histidine kinase
AB0725		Two-component response regulator
AB0732		Conserved hypothetical protein
AB0733	*cysJ*	Sulfite reductase, flavoprotein component
AB0734		Conserved hypothetical protein (DUF466 domain protein)
AB0735	*cstA*	Carbon starvation protein A
AB0736		GTP-binding protein, putative
AB0737		Conserved hypothetical protein
AB0738		Conserved hypothetical protein
AB0739		Conserved hypothetical protein
AB0740		Conserved hypothetical protein (DUF1255 domain protein)
AB0741		Conserved hypothetical protein
AB0743	*thiL*	Thiamine monophosphate kinase
AB0744	*truD*	tRNA pseudouridine synthase D
AB0746		Methyl-accepting chemotaxis protein
AB0747	*ruvA*	Holliday junction DNA helicase RuvA
AB0748	*ddlA*	D-alanine--D-alanine ligase
AB0749		2-hydroxy-6-oxohepta-2,4-dienoate hydrolase
AB0750	*murF*	UDP-N-acetylmuramoylalanyl-D-glutamyl-2,6-diaminopimelate ligase
AB0751		HIT family protein
AB0753		Hypothetical protein
AB0754	*rpsJ*	30S ribosomal protein S10
AB0755	*rplC*	50S ribosomal protein L3
AB0756	*rplD*	50S ribosomal protein L4
AB0757	*rplW*	50S ribosomal protein L23
AB0758	*rplB*	50S ribosomal protein L2
AB0759	*rpsS*	30S ribosomal protein S19
AB0760	*rplV*	50S ribosomal protein L22
AB0761	*rpsC*	30S ribosomal protein S3
AB0762	*rplP*	50S ribosomal protein L16
AB0763	*rpmC*	50S ribosomal protein L29
AB0764	*rpsQ*	30S ribosomal protein S17
AB0765	*rplN*	50S ribosomal protein L14
AB0766	*rplX*	50S ribosomal protein L24
AB0767	*rplE*	50S ribosomal protein L5
AB0768	*rpsN*	30S ribosomal protein S14
AB0769	*rpsH*	30S ribosomal protein S8
AB0770	*rplF*	50S ribosomal protein L6
AB0772	*rpsE*	30S ribosomal protein S5
AB0773	*rplO*	50S ribosomal protein L15
AB0774	*secY*	Preprotein translocase, SecY subunit

AB0775	*map*	Methionine aminopeptidase
AB0776	*infA*	Translation initiation factor IF-1
AB0777		Conserved hypothetical protein
AB0778		Conserved hypothetical protein
AB0779	*aspS*	Aspartyl-tRNA synthetase
AB0780		CheW-like chemotaxis protein
AB0782	*adk1*	Adenylate kinase
AB0783	*adk2*	Adenylate kinase
AB0784		Molybdopterin binding domain protein
AB0786		Zinc-containing NADP-dependent alcohol dehydrogenase
AB0787		Iron-containing NADP-dependent alcohol dehydrogenase
AB0788		Methyl-accepting chemotaxis protein
AB0789	*alr*	Alanine racemase
AB0790	*uvrC*	Excinuclease ABC, subunit C
AB0791		Methyl-accepting chemotaxis protein
AB0816		Hydrophobe/amphiphile efflux-1 family protein
AB0817		Multidrug efflux RND membrane fusion protein
AB0818		Outer membrane efflux protein
AB0820		Hypothetical protein
AB0824	*xth*	Exodeoxyribonuclease
AB0825		Hypothetical protein
AB0826		Conserved hypothetical protein (DUF81 domain protein)
AB0827		Conserved hypothetical protein
AB0828		Outer membrane lipoprotein
AB0829		Conserved hypothetical protein
AB0830		Conserved hypothetical protein
AB0831		ABC transporter protein
AB0832		Conserved hypothetical protein
AB0835		Two-component sensor histidine kinase
AB0836		Carotenoid isomerase, putative
AB0837	*phrB*	Deoxyribodipyrimidine photolyase
AB0838		Conserved hypothetical protein (DUF523/1722 domain protein)
AB0839		Auxin efflux carrier protein, putative
AB0840		Conserved hypothetical protein (DUF386 domain protein)
AB0841	*pgi*	Glucose-6-phosphate isomerase
AB0842	*galU*	UTP--glucose-1-phosphate uridylyltransferase
AB0843		Phosphohexosemutase
AB0844		Methyl-accepting chemotaxis protein
AB0847		Transcriptional regulator, LysR family
AB0848	*trpC*	Indole-3-glycerol phosphate synthase
AB0849		Conserved hypothetical protein
AB0850		Conserved hypothetical protein
AB0851		Conserved hypothetical protein
AB0852	*oorD*	OorD subunit of 2-oxoglutarate:acceptor oxidoreductase
AB0853	*oorA*	OorA subunit of 2-oxoglutarate:acceptor oxidoreductase
AB0854	*oorB*	OorB subunit of 2-oxoglutarate:acceptor oxidoreductase
AB0855	*oorC*	OorC subunit of 2-oxoglutarate:acceptor oxidoreductase
AB0856	*dnaQ3*	DNA polymerase III, epsilon subunit
AB0857	*trpF*	N-(5'phosphoribosyl)anthranilate isomerase
AB0858	*rpe*	Ribulose-phosphate 3-epimerase
AB0859	*pldA*	Outer membrane phospholipase A
AB0860		Conserved hypothetical protein
AB0861		Conserved hypothetical protein
AB0862		Conserved hypothetical protein (DUF520 domain protein)
AB0863		Conserved hypothetical protein

AB0864		Conserved hypothetical membrane protein (DUF423)
AB0865		Putative nucleotide phosphoribosyltransferase
AB0866		Conserved hypothetical protein
AB0867		Hypothetical protein
AB0868		Conserved hypothetical protein
AB0869		Membrane-associated zinc metalloprotease, putative
AB0870	*pgsA*	CDP-1,2-diacyl-sn-glycero-3-phosphate phosphatidyltransferase
AB0871		Oxidoreductase, short chain dehydrogenase/reductase family
AB0872	*dapA*	Dihydrodipicolinate synthase
AB0873		Putative zinc protease
AB0874	*pyrD*	Dihydroorotate dehydrogenase
AB0875	*msbA*	Multidrug resistance protein MsbA
AB0876	*mviN*	Virulence factor MviN protein
AB0877		Endonuclease/exonuclease/phosphatase
AB0878	*perR*	Peroxide stress regulator
AB0880		Conserved hypothetical protein
AB0882	*radA*	DNA repair protein RadA
AB0883		Conserved hypothetical protein
AB0887		Hypothetical protein
AB0888	*ftsY*	Signal recognition particle-docking GTPase FtsY
AB0889		Putative lipoprotein thiredoxin
AB0890		Putative 5-formyltetrahydrofolate cyclo-ligase
AB0891		HD/HDIG/KH domain protein
AB0892		Putative methyltransferase
AB0893		ABC transporter, permease protein
AB0898		Oxidoreductase, short-chain dehydrogenase/reductase family
AB0899		Conserved hypothetical protein
AB0901		Cytochrome c family protein
AB0905		Two-component sensor histidine kinase
AB0906		Molybdenum transport system protein ModD, putative
AB0907		GGDEF domain protein
AB0908		GatB/YqeY family protein
AB0909		ABC transporter, ATP-binding protein
AB0910		Conserved hypothetical protein
AB0911		Conserved hypothetical protein
AB0912		Conserved hypothetical protein
AB0913		Conserved hypothetical protein
AB0914		Hypothetical protein
AB0915		Hypothetical protein
AB0916		Conserved hypothetical protein
AB0921	*guaA*	GMP synthase
AB0922	*nadB*	L-aspartate oxidase
AB0923		Conserved hypothetical protein
AB0924		Conserved hypothetical protein (DUF45 domain protein)
AB0925	*aspB3*	Aspartate aminotransferase, aminotransferase, classes I and II
AB0926		Cation efflux protein
AB0931		Conserved hypothetical protein
AB0932		Conserved hypothetical protein
AB0933		Major facilitator superfamily protein, putative oxalate:formate antiporter
AB0934		Conserved hypothetical protein
AB0935	*cbiM*	Cobalamin (Vitamin B12) biosynthesis protein
AB0936		Hypothetical protein
AB0938		Cobalt ABC transporter, permease protein, putative
AB0939		Cobalt ABC transporter, ATP-binding protein, putative
AB0951		Conserved hypothetical protein

AB0954		Conserved hypothetical protein
AB0955		Putative permease
AB0956		Hypothetical protein
AB0957		Hypothetical protein
AB0958		Conserved hypothetical protein
AB0959		Hypothetical protein
AB0960	*uvrA*	Excinuclease ABC, subunit A
AB0961		Conserved hypothetical protein
AB0962		Methyl-accepting chemotaxis protein
AB0964		Conserved hypothetical protein (DUF81 domain protein)
AB0966	*cysE*	Serine acetyltransferase
AB0967	*speA*	Arginine decarboxylase
AB0968	*hisS*	Histidyl-tRNA synthetase
AB0969	*tmk*	Thymidylate kinase
AB0970	*coaD*	Phosphopantetheine adenylyltransferase
AB0971	*ubiD*	Phenylacrylic acid decarboxylase
AB0972		Site-specific recombinase, resolvase family
AB0973	*rplI*	50S ribosomal protein L9
AB0974	*hslV*	Heat shock protein HslVU, ATP-dependent protease subunit HslV
AB0975	*hslU*	Heat shock protein HslVU, ATP-dependent protease subunit HslU
AB0976		Hypothetical protein
AB0977		TatD-related deoxyribonuclease
AB0978		Putative lytic murein transglycosylase
AB0979	*rlpA*	Rare lipoprotein A
AB0980	*hisB*	Imidazoleglycerol-phosphate dehydratase
AB0981		HAD-superfamily hydrolase subfamily IIIA:Phosphatase
AB0982		Hypothetical protein
AB0983		OstA family protein
AB0984		Putative ATP /GTP binding protein
AB0985		Acetyltransferase, GNAT Family
AB0986		Sigma factor, ECF family
AB0987		Sigma factor regulatory protein, FecR/PupR family
AB0989	*argC*	N-acetyl-gamma-glutamyl-phosphate reductase
AB0990		Transcriptional regulator, GntR family
AB0991		FMN-binding protein
AB0992		Putative antibiotic resistance protein
AB0993		AcrB/AcrD/AcrF family protein
AB0994		AcrB/AcrD/AcrF family protein
AB0995		AcrA/AcrE family protein
AB0996		Outer membrane efflux protein
AB0997		Response regulator receiver:Metal-dependent phosphohydrolase, HD subdomain
AB0998	*fliP*	Flagellar biosynthetic protein FliP
AB0999	*pbpC*	Penicillin-binding protein
AB1000		Hypothetical protein
AB1001		Conserved hypothetical protein
AB1002	*ftsX*	Cell division protein FtsX
AB1011		Conserved hypothetical protein
AB1012		Conserved hypothetical protein
AB1013		Conserved hypothetical protein
AB1014	*rpoD*	RNA polymerase sigma 70 factor
AB1016	*leuB*	3-isopropylmalate dehydrogenase
AB1022	*rpsD*	30S ribosomal protein S4
AB1023	*rpoA*	DNA-directed RNA polymerase, alpha chain
AB1024	*rplQ*	50S ribosomal protein L17
AB1025	*gatA*	Glutamyl-tRNA(Gln) amidotransferase, subunit A

AB1026	*guaB*	Inosine-5-monophosphate dehydrogenase
AB1027		Conserved hypothetical protein
AB1028		Hypothetical protein
AB1029		Two-component sensor histidine kinase
AB1030		Two-component response regulator
AB1031		Outer membrane efflux protein, putative
AB1032		Conserved hypothetical protein
AB1033		Hypothetical membrane protein
AB1034		Putative membrane protein
AB1035		Peptidase, M50 family
AB1036		DnaJ domain protein
AB1037		DnaJ domain protein
AB1038		Glycosyl hydrolase
AB1042		ABC transporter, ATP-binding protein
AB1046		NADP-dependent alcohol dehydrogenase
AB1047		Conserved hypothetical protein
AB1048		Cyclic nucleotide-binding protein
AB1050		Conserved hypothetical protein (DUF24 domain protein)
AB1051		Conserved hypothetical protein
AB1052		Putative sodium:sulfate symporter
AB1053	*sugE*	Suppresses groEL, may be chaperone
AB1057		Transcriptional regulator, LysR family
AB1058		Putative integral membrane protein
AB1059		Conserved hypothetical periplasmic protein
AB1060		Auxin efflux carrier protein
AB1061		Conserved hypothetical protein (DUF24 domain protein)
AB1062		Flavodoxin-like fold domain protein, putative NADPH-quinone reductase
AB1063		Conserved hypothetical protein
AB1065		Conserved hypothetical integral membrane protein (DUF6 domain protein)
AB1066		NAD(P)H-flavin nitroreductase
AB1067		Hypothetical protein
AB1068		ABC transporter, ATP-binding protein
AB1069		Acyl-CoA thioester hydrolase family protein
AB1071	*lig*	DNA ligase
AB1072		Glutathionylspermidine synthase family protein
AB1073		Conserved hypothetical lipoprotein
AB1074	*dgt*	Deoxyguanosinetriphosphate triphosphohydrolase
AB1080	*aroE*	Shikimate 5-dehydrogenase
AB1081		Methyltransferase, putative
AB1082		Conserved hypothetical protein (DUF152 domain protein)
AB1083	*maeA*	NAD-dependent malic enzyme
AB1084	*purU*	Formyltetrahydrofolate deformylase
AB1085		RNA methylase, SpoU family
AB1087		Sodium:phosphate cotransporter
AB1088		Hypothetical protein
AB1089		Hypothetical protein
AB1090		Two-component response regulator
AB1091		Two-component sensor histidine kinase
AB1092		Putative protease
AB1093		Conserved hypothetical protein
AB1099		Conserved hypothetical protein
AB1100		TPR repeat protein, SEL1 subfamily
AB1102	*aat*	Leucyl/phenylalanyl-tRNA--protein transferase
AB1103	*rsuA*	Ribosomal small subunit pseudouridine synthase A
AB1104		HAD-superfamily hydrolase

AB1105		Conserved hypothetical protein
AB1106		Conserved hypothetical protein
AB1109		Putative iron compound ABC transporter, periplasmic substrate-binding protein
AB1110		Putative iron compound ABC transporter, ATP-binding protein
AB1111		Putative iron compound ABC transporter, permease protein
AB1112		Phosphohistidine phosphatase
AB1113		ABC transporter, ATP-binding protein
AB1114		Hypothetical protein
AB1115		Two-component response regulator
AB1116		Two-component sensor histidine kinase
AB1117		Conserved hypothetical protein
AB1118		Hypothetical protein
AB1119		Conserved hypothetical protein
AB1120		Hypothetical protein
AB1123		Putative ATP-dependent RNA helicase RhlE
AB1126		Hypothetical outer membrane protein
AB1127		ABC transporter, transmembrane region
AB1128		HlyD family secretion protein
AB1129		Two-component response regulator
AB1130		Conserved hypothetical protein
AB1131		EAL/GGDEF/HAMP domain protein
AB1132	*aroB*	3-dehydroquinate synthase
AB1133		Conserved hypothetical integral membrane protein
AB1134		MiaB-like tRNA modifying enzyme
AB1136		Conserved hypothetical protein
AB1137	*mog*	Molybdenum cofactor biosynthesis protein Mog
AB1139		Conserved hypothetical protein
AB1143		Conserved hypothetical protein
AB1153		Transcriptional regulator, TetR family
AB1154	*mutS2*	Mismatch repair ATPase
AB1157		Conserved hypothetical protein (DUF748 domain protein)
AB1158	*dapE*	Succinyl-diaminopimelate desuccinylase
AB1159	*amtB*	Ammonium transporter
AB1160	*glnB2*	Nitrogen regulatory protein PII
AB1161	*pssA*	CDP-diacylglycerol--serine O-phosphatidyltransferase
AB1162		Ppx/GppA phosphatase family protein
AB1163		Hypothetical protein
AB1164	*ilvI*	Acetolactate synthase, large subunit
AB1165	*ilvH*	Acetolactate synthase, small subunit
AB1166	*lpxD*	UDP-3-O-[3-hydroxymyristoyl] glucosamine N-acyltransferase
AB1167	*ftsK*	Cell division protein FtsK
AB1168		Two-component response regulator
AB1169		Two-component sensor histidine kinase
AB1170		Conserved hypothetical protein
AB1183	*engA*	GTP-binding protein
AB1184		Sulfate permease family protein
AB1185	*hemD*	Putative uroporphyrinogen III cosynthase HemD
AB1186		Hypothetical protein
AB1187	*purD*	Phosphoribosylamine-glycine ligase
AB1188		Conserved hypothetical protein
AB1189		Conserved hypothetical membrane protein
AB1190		Conserved hypothetical protein
AB1191	*pnp*	Polyribonucleotide nucleotidyltransferase
AB1192	*cheY1*	Chemotaxis protein CheY
AB1193	*cheA*	Chemotaxis protein CheA

AB1194	*cheR*	Chemotaxis protein methyltransferase
AB1195	*cheD*	Chemotaxis protein CheD
AB1196	*cheB*	Protein-glutamate methylesterase CheB
AB1197		Conserved hypothetical protein
AB1198	*fsr*	Fosmidomycin resistance protein
AB1199	*xerD*	Integrase/recombinase XerD
AB1200		Conserved hypothetical protein
AB1201		Hypothetical protein
AB1202		Hypothetical protein
AB1203	*prlC*	Oligopeptidase A
AB1204	*hemK*	Modification methylase
AB1205	*hemN1*	Oxygen-independent coproporphyrinogen III oxidase
AB1206	*nudH*	(Di)nucleoside polyphosphate hydrolase
AB1207	*lysC*	Aspartokinase
AB1208		Conserved hypothetical protein
AB1209	*holB*	Putative DNA polymerase III delta prime subunit HolB
AB1210	*folP*	Dihydropteroate synthase
AB1218	*tyrA*	Prephenate dehydrogenase
AB1219		Outer membrane surface antigen protein
AB1220		SAM domain protein
AB1221		Peptidase, M16 family
AB1222	*gltX2*	Glutamyl-tRNA synthetase
AB1223		Conserved hypothetical membrane protein
AB1225	*slt*	Soluble lytic murein transglycosylase (slt)
AB1226		Hypothetical protein
AB1227	*mobB*	Molybdopterin-guanine dinucleotide biosynthesis protein
AB1228	*fbp*	Fructose-1,6-biphosphatase
AB1229		Conserved hypothetical protein
AB1230	*metS*	Methionyl-tRNA synthetase
AB1231		Conserved hypothetical protein
AB1232		Peptidase, S24 family
AB1234		Hypothetical protein
AB1235	*pycB2*	Pyruvate/oxaloacetate carboxyltransferase
AB1236	*pckA*	Phosphoenolpyruvate carboxykinase
AB1237	*ribAB*	GTP cyclohydrolase II / 3,4-dihydroxy-2-butanone 4-phosphate synthase
AB1238	*glyS*	Glycyl-tRNA synthetase, beta chain
AB1240		Conserved hypothetical protein
AB1241		Conserved hypothetical protein
AB1242	*gidB*	Glucose inhibited division protein B
AB1243	*ribA*	GTP cyclohydrolase II
AB1244	*hemB*	Delta-aminolevulinic acid dehydratase
AB1245		Two-component sensor histidine kinase
AB1246		Two-component response regulator
AB1247		Radical SAM domain protein
AB1250	*argF*	Ornithine carbamoyltransferase
AB1251	*hemN2*	Oxygen-independent coproporphyrinogen III oxidase
AB1254	*lpxK*	Lipid A biosynthesis protein LpxK
AB1255		DegT/DnrJ/EryC1/StrS aminotransferase
AB1256	*nadE*	NH(3)-dependent NAD+ synthetase
AB1258		Hypothetical protein
AB1259		DnaJ domain protein
AB1261		Conserved hypothetical membrane protein
AB1262	*tatC*	Sec-independent protein secretion pathway component TatC
AB1263	*queA*	S-adenosylmethionine:tRNA ribosyltransferase-isomerase
AB1264	*dnaQ4*	DNA polymerase III, epsilon subunit

AB1266	*comE*	Competence locus E
AB1267		Hypothetical protein
AB1268		Conserved hypothetical protein
AB1269		Hypothetical protein
AB1270		Hypothetical protein
AB1271		Conserved hypothetical protein
AB1272	*glpC*	Anaerobic glycerol-3-phosphate dehydrogenase, subunit C (glpC)
AB1273	*lgt*	Phosphatidylglycerol-prolipoprotein diacylglyceryl transferase
AB1274		Hypothetical protein
AB1275		Two-component response regulator
AB1276	*polA*	DNA polymerase I
AB1278	*kdsB*	3-deoxy-manno-octulosonate cytidylyltransferase
AB1279		EAL/GGDEF domain protein
AB1280		ABC transporter, ATP-binding protein
AB1281		Conserved hypothetical protein
AB1282	*trpD*	Anthranilate phosphoribosyltransferase
AB1283		Conserved hypothetical protein
AB1284		S4 domain protein
AB1285	*argG*	Argininosuccinate synthase
AB1286	*dltA*	D-alanine activating enzyme
AB1287	*dltB*	D-alanyl transfer protein
AB1289	*dltD*	Poly D-alanine transfer protein
AB1290	*dltC*	D-alanyl carrier protein
AB1291		Conserved hypothetical protein
AB1295	*ktrA*	TRK system potassium uptake protein TrkA, putative
AB1296	*ktrB*	TRK system potassium uptake protein TrkB, putative
AB1297	*kdpE*	Two component system transcriptional regulatory protein
AB1298	*kdpD*	Two-component regulatory protein sensor kinase KdpD
AB1299	*dut*	Deoxyuridinetriphosphatase
AB1300	*recR*	Recombination protein RecR
AB1301	*dnaJ*	Co-chaperone and heat shock protein DnaJ
AB1302	*trpB2*	Tryptophan synthase, beta chain
AB1303		Hypothetical protein
AB1304		Hypothetical protein
AB1305		Conserved hypothetical protein (DUF455 domain protein)
AB1306		Beta-lactamase-like protein
AB1307		GGDEF domain protein
AB1308		GGDEF domain protein
AB1309		Conserved hypothetical protein
AB1311		Cytochrome c biogenesis protein
AB1312	*pepD*	Aminoacyl-histidine dipeptidase PepD
AB1313		Hypothetical protein
AB1314		Methyl-accepting chemotaxis protein
AB1315		Hypothetical protein
AB1316	*lolA*	Outer membrane lipoprotein carrier protein LolA
AB1317	*secA*	Protein translocase, SecA subunit
AB1318		Conserved hypothetical integral membrane protein
AB1319		Hypothetical protein
AB1320		4-amino-4-deoxychorismate lyase PabC
AB1321	*icd*	Isocitrate dehydrogenase
AB1323		Conserved hypothetical protein, putative cytochrome c
AB1324		Hypothetical protein
AB1325		Conserved hypothetical protein
AB1326		Hypothetical protein
AB1327	*mraW*	S-adenosyl-methyltransferase

AB1359		Conserved hypothetical protein
AB1360	*norB*	Nitric oxide reductase, cytochrome b subunit
AB1361		Hypothetical protein
AB1362		D-amino acid oxidase domain protein
AB1363		Conserved hypothetical protein
AB1364		Response regulator receiver domain protein, CheY-like
AB1366		Two-component sensor histidine kinase
AB1367	*cbpA*	Curved DNA-binding protein CbpA
AB1368		Transcriptional regulator (MerR family), putative heat shock regulator HspR
AB1369		HlyD-family secretion protein
AB1370		ABC transporter, ATP-binding protein
AB1371		ABC transporter, permease protein
AB1372		ABC transporter, permease protein
AB1373		Conserved hypothetical protein (DUF475 domain protein)
AB1374	*brnQ*	Branched-chain amino acid transport system II carrier protein
AB1376		Conserved hypothetical protein
AB1377		Conserved hypothetical protein
AB1379		Polysaccharide deacetylase
AB1380	*nhaA1*	Sodium:hydrogen antiporter
AB1381		Hypothetical protein
AB1382	*metX*	Homoserine O-acetyltransferase
AB1384		Hypothetical protein
AB1385	*ftsZ*	Cell division protein FtsZ
AB1386	*ftsA*	Cell division protein FtsA
AB1387		Conserved hypothetical membrane protein
AB1388	*recD*	Exodeoxyribonuclease V, alpha subunit
AB1389		Conserved hypothetical protein
AB1392		Hypothetical protein
AB1393		Conserved hypothetical protein
AB1394		Conserved hypothetical protein
AB1395		Conserved hypothetical membrane protein
AB1398		Conserved hypothetical protein (DUF1706 domain protein)
AB1399	*dapD*	2,3,4,5-tetrahydropyridine-2-carboxylate N-succinyltransferase DapD
AB1400		HAD-superfamily hydrolase
AB1401		Hypothetical protein
AB1403	*priA*	Primosomal protein N'
AB1404		Hypothetical protein
AB1405		Hypothetical protein
AB1407		Hypothetical protein
AB1408	*hypA*	Hydrogenase expression/formation protein HypA
AB1409	*hypE*	Hydrogenase expression/formation protein HypE
AB1410		Conserved hypothetical protein
AB1411		3-hydroxyisobutyrate dehydrogenase family protein
AB1412		McrBC endonuclease McrB, putative
AB1415	*hypC*	Hydrogenase expression/formation protein HypC
AB1416	*hypB*	Hydrogenase expression/formation protein HypB
AB1417		Conserved hypothetical protein
AB1421		Conserved hypothetical membrane protein (DUF808)
AB1422		Hypothetical protein
AB1423		Conserved hypothetical protein
AB1424		Conserved hypothetical membrane protein (DUF481)
AB1425		Conserved hypothetical protein
AB1427	*thiI*	Thiamine biosynthesis protein ThiI
AB1428		Hypothetical protein
AB1429		Hypothetical protein

AB1430		Conserved hypothetical protein (DUF1504 domain protein)
AB1431	*hypF*	Transcriptional regulatory protein HypF
AB1432		Conserved hypothetical protein
AB1433	*hydD*	Ni/Fe hydrogenase, expression/formation protein
AB1434	*hydC*	Ni/Fe hydrogenase, cytochrome b subunit
AB1435	*hydB*	Ni/Fe-hydrogenase, large subunit
AB1438		Sigma factor, ECF family
AB1439		Conserved hypothetical protein
AB1440	*hyaD*	Ni/Fe hydrogenase, expression/formation protein
AB1441	*hyaC*	Ni/Fe hydrogenase, cytochrome b subunit
AB1442	*hyaB*	Ni/Fe-hydrogenase, large subunit
AB1443	*hyaA*	Ni/Fe-hydrogenase, small subunit
AB1444	*hupL*	Ni/Fe-dependent hydrogenase, large subunit
AB1445	*hupS*	Ni/Fe-dependent hydrogenase, small subunit
AB1446		Transcriptional regulator, TetR family
AB1447	*acnB*	Aconitate hydratase 2
AB1449		GGDEF/PAS domain protein
AB1450		Conserved hypothetical protein (DUF28 domain protein)
AB1451		VacJ-like lipoprotein
AB1452		Putative periplasmic protein
AB1453		Conserved hypothetical integral membrane protein
AB1454		Lysine decarboxylase-like protein
AB1455		HAD-superfamily hydrolase
AB1456		Putative lipoprotein thiredoxin
AB1457	*dsbA*	DsbA-like thioredoxin domain protein
AB1458	*dsbB*	Disulfide bond formation protein, DsbB family
AB1459		Glycosyltransferase
AB1463		GGDEF domain protein
AB1464		Conserved hypothetical protein (DUF125 domain protein)
AB1466	*kefB*	Glutathione-regulated potassium-efflux system protein KefB
AB1467		Conserved hypothetical protein
AB1468	*glyA2*	Serine hydroxymethyltransferase
AB1469	*ada*	O6-methylguanine-DNA methyltransferase
AB1470		Endonuclease III
AB1471		Ribonuclease H-like protein
AB1474		Pyridoxamine 5'-phosphate oxidase-related, FMN-binding
AB1476	*argD2*	N-acetylornithine aminotransferase
AB1477		Conserved hypothetical protein
AB1478	*lipA*	Lipoic acid synthetase
AB1480	*aceE*	Pyruvate dehydrogenase E1 component
AB1481	*aceF*	Dihydrolipoamide acetyltransferase
AB1482	*lpdA*	Dihydrolipoamide dehydrogenase
AB1483		Glutamine amidotransferase, class I
AB1485		Aldose 1-epimerase family protein, LacX
AB1487	*rhlE*	ATP-dependent RNA helicase RhlE
AB1494		Conserved hypothetical protein (DUF302 domain protein)
AB1495		Conserved hypothetical protein
AB1497	*rnhA*	Ribonuclease HI
AB1498		HMGL family protein
AB1499		Conserved hypothetical protein
AB1500		Conserved hypothetical protein
AB1501	*dinP*	DNA polymerase IV
AB1502	*moeA1*	Molybdenum cofactor biosynthesis protein A
AB1503		Cysteine desulfurase, NifS homolog
AB1504	*fdhD*	FdhD/NarQ protein required for formate dehydrogenase activity

AB1505	*fdhC*	Putative FdhC protein
AB1506	*fdhB1*	Formate dehydrogenase, iron-sulfur subunit FdhB
AB1507	*fdhA1*	Formate dehydrogenase, large subunit FdhA (Selenocysteine containing)
AB1509		Conserved hypothetical protein
AB1510		Conserved hypothetical protein
AB1511		4Fe-4S ferredoxin, iron-sulfur binding
AB1512	*livJ*	Leucine/isoleucine/valine-binding protein
AB1513		Two-component sensor histidine kinase
AB1514		Two-component response regulator
AB1515		Mn2+ and Fe2+ transporter, NRAMP family
AB1516		Heavy-metal transporting ATPase
AB1517		Heavy-metal transport protein, MerT homolog
AB1518		Transglutaminase family protein
AB1519		Putative FdhC protein
AB1520	*fdhB2*	Formate dehydrogenase, iron-sulfur subunit FdhB
AB1521	*fdhA2*	Formate dehydrogenase, large subunit FdhA (Cysteine containing)
AB1522		Formate dehydrogenase subunit E, putative
AB1523	*selD*	Selenide, water dikinase
AB1533	*selA*	L-seryl-tRNA selenium transferase
AB1534	*selB*	Selenocysteine-specific elongation factor
AB1535		Putative DNA helicase
AB1536		Conserved hypothetical protein
AB1537		Hypothetical protein
AB1540		Conserved hypothetical protein
AB1541		Two-component sensor histidine kinase
AB1542		Putative FMN reductase
AB1543		PhoH family protein
AB1546	*pdxH*	Pyridoxamine 5'-phosphate oxidase
AB1547		GGDEF/PAS domain protein
AB1548	*dbpA*	ATP-dependent RNA helicase DbpA
AB1549		Conserved hypothetical integral membrane protein
AB1550		Conserved hypothetical protein
AB1551		Hypothetical protein
AB1552	*htpG*	Heat shock protein 90 HtpG
AB1555	*ciaB*	CiaB protein
AB1557		Ankyrin repeat protein
AB1558	*pqiB*	Paraquat-inducible protein B
AB1561		Protozoan/cyanobacterial globin homolog
AB1562		EAL/GGDEF domain protein
AB1563		Hypothetical protein
AB1564		Putative integral membrane protein (DUF6 domain protein)
AB1565		Conserved hypothetical membrane protein (DUF6)
AB1566		Conserved hypothetical protein
AB1567		Putative DNA alkylation repair enzyme
AB1570		Transcriptional regulator, ThiJ/PfpI family
AB1572		Conserved hypothetical protein
AB1578	*speB*	Arginase/agmatinase/formiminoglutamate hydrolase, arginase family
AB1580		Transcriptional regulator, AraC family
AB1585	*sodB*	Superoxide dismutase
AB1586		Conserved hypothetical membrane protein
AB1587	*ppa*	Inorganic pyrophosphatase, manganese-dependent
AB1588		Conserved hypothetical protein (DUF1121 domain protein)
AB1589		Conserved hypothetical protein, putative asparaginase
AB1590	*aspA*	Aspartate ammonia-lyase
AB1594	*pfs*	5'-methylthioadenosine\S-adenosylhomocysteine nucleosidase

AB1595	*fabD*	Malonyl coenzyme A-(acyl carrier protein) transacylase
AB1596	*slyD*	Peptidyl-prolyl cis-trans isomerase
AB1597		Conserved hypothetical protein
AB1598		OmpA/MotB precursor
AB1599	*tolB*	Colicin tolerance-like protein (tolB)
AB1600		Conserved hypothetical protein
AB1601	*exbD3*	Biopolymer transport protein ExbD
AB1602	*exbB3*	Biopolymer transport protein ExbB
AB1603	*atpC*	ATP synthase F1 sector, epsilon subunit
AB1605	*atpD*	ATP synthase F1 sector, beta subunit
AB1606	*atpG*	ATP synthase F1 sector, gamma subunit
AB1607	*atpA*	ATP synthase F1 sector, alpha subunit
AB1608	*atpH*	ATP synthase F1 sector, delta subunit
AB1609	*atpF*	ATP synthase F0 sector, subunit B
AB1610	*atpF'*	ATP synthase F0 sector, B' subunit
AB1611	*parB*	Transcriptional regulator involved in chromosome partitioning ParB
AB1612	*parA*	ATPases involved in chromosome partitioning ParA
AB1613	*birA*	Biotin--acetyl-CoA-carboxylase ligase
AB1615		Auxin efflux carrier protein
AB1616	*fmt*	10-formyltetrahydrofolate:L-methionyl-tRNA(fMet) N-formyltransferase
AB1617	*proB*	Glutamate 5-kinase
AB1618	*obg*	GTP-binding protein
AB1619	*rpmA*	50S ribosomal protein L27
AB1620	*rplU*	50S ribosomal protein L21
AB1621	*dnaG*	DNA primase
AB1622		Conserved hypothetical protein
AB1623	*rnc*	Ribonuclease III
AB1624	*aroC*	Chorismate synthase
AB1626		Peptidase, M48 family
AB1627		Conserved hypothetical protein
AB1628		Phosphoglycerate/bisphosphoglycerate mutase, putative
AB1629		Hypothetical protein
AB1630	*trmE*	tRNA modification GTPase
AB1631		Conserved hypothetical protein
AB1632	*oxaA*	Inner membrane protein, 60 kDa
AB1633		Conserved hypothetical protein (DUF37 domain protein)
AB1635	*clpB*	ATP-dependent Clp protease, ATP-binding subunit
AB1636	*rnhB*	Ribonuclease HII
AB1637		Hypothetical protein (DUF77 domain protein)
AB1638		Conserved hypothetical protein (DUF344 domain protein)
AB1639		Conserved hypothetical protein
AB1640	*msrA*	Peptide methionine sulfoxide reductase
AB1641	*msrB*	Peptide methionine sulfoxide reductase
AB1642		Fumarylacetoacetate (FAA) hydrolase
AB1643		Conserved hypothetical protein (DUF1291 domain protein)
AB1644		Phosphohydrolase (MUTT/NUDIX family protein)
AB1645	*bioA*	7,8-diaminopelargonic acid synthetase
AB1646		Two-component sensor histidine kinase
AB1647	*purH*	Phosphoribosylaminoimidazolecarboxamide formyltransferase / IMP cyclohydrolase
AB1648	*purL*	Phosphoribosylformylglycinamidine synthase II
AB1649		Conserved hypothetical protein
AB1650		Peptidase, M23/M37 family
AB1651	*folE*	GTP cyclohydrolase I
AB1652	*corA*	Magnesium and cobalt transport protein
AB1653	*ctsF*	Campylobacter transformation system protein CtsF

AB1654	*ctsE*	Campylobacter transformation system protein CtsE
AB1707		Hypothetical protein
AB1708		Hypothetical protein
AB1709	*era*	GTP-binding protein Era homolog
AB1711		Mg chelatase-related protein
AB1712		Hypothetical protein
AB1713	*def*	Polypeptide deformylase
AB1714	*clpP*	ATP-dependent Clp protease, proteolytic subunit
AB1715	*tig*	Trigger factor
AB1716		Conserved hypothetical protein
AB1718		Conserved hypothetical protein
AB1719	*nspC*	Carboxynorspermidine decarboxylase
AB1720		Saccharopine dehydrogenase (L-lysine-forming)
AB1737	*metC1*	Cystathionine gamma-synthase
AB1738	*metC2*	Cystathionine gamma-synthase
AB1745		Auxin efflux carrier protein
AB1746		Hypothetical protein
AB1747		Hypothetical protein
AB1748		Hypothetical protein
AB1749		Conserved hypothetical protein, putative cytochrome
AB1750		Two-component response regulator
AB1751		Acetyltransferase, GNAT family
AB1752		Putative nickel transporter
AB1753		Putative cation ABC transporter, periplasmic-binding protein
AB1754		Transcriptional regulator, Fur family
AB1755	*nhaA2*	Sodium:hydrogen antiporter
AB1756		Diheme cytochrome c peroxidase
AB1757		Sterol desaturase-related protein
AB1758		Hypothetical protein
AB1759		Conserved hypothetical protein (DUF1111 domain protein)
AB1760		Conserved hypothetical protein
AB1761		Conserved hypothetical protein
AB1763	*atpE*	ATP synthase F0 sector, C subunit
AB1767		Hypothetical protein
AB1768		Hypothetical protein
AB1769		Conserved hypothetical protein
AB1770	*thiD*	Phosphomethylpyrimidine kinase
AB1771	*proA*	Gamma-glutamyl phosphate reductase
AB1772		Glycosyl hydrolase, putative
AB1773		Hypothetical protein
AB1774		Ferredoxin-like protein
AB1775		Two-component sensor histidine kinase
AB1776		Two-component response regulator
AB1777	*ogt*	Putative methylated-DNA-protein-cysteine methyltransferase
AB1778		Phosphohexosemutase
AB1779		Conserved hypothetical protein, putative nitrilase/cyanide hydratase
AB1780		Conserved hypothetical protein
AB1781		Conserved hypothetical protein
AB1782		Conserved hypothetical protein
AB1783	*fldA*	Flavodoxin
AB1784	*fur2*	Ferric uptake regulation protein
AB1785		Hypothetical protein
AB1786	*ate*	Putative arginyl-tRNA--protein transferase
AB1787	*trpA*	Tryptophan synthase, alpha chain
AB1789	*panB*	3-methyl-2-oxobutanoate hydroxymethyltransferase

AB1790	*ruvB*	Holliday junction DNA helicase RuvB
AB1807	*waaE*	ADP-heptose synthase
AB1808	*gmhA*	D-sedoheptulose 7-phosphate isomerase
AB1809		Putative sulfatase
AB1810	*waaF*	Lipopolysaccharide heptosyltransferase II
AB1811		Conserved hypothetical protein
AB1812		Putative heptosyltransferase
AB1813		Conserved hypothetical protein
AB1814		Conserved hypothetical membrane protein
AB1817		Putative acetyltransferase
AB1818		Putative glycosyltransferase
AB1819		Putative glycosyltransferase
AB1820		Conserved hypothetical protein
AB1821		Probable glycosyltransferase
AB1822		Conserved hypothetical protein
AB1823		Glycosyltransferase
AB1824		Putative O-antigen polymerase
AB1825		Aminotransferase, DegT/DnrJ/EryC1/StrS family
AB1826		dTDP-glucose 4,6-dehydratase
AB1827		Glucose-1-phosphate thymidylyltransferase
AB1829		Phosphoglycerol transferase
AB1830	*dgkA*	Diacylglycerol kinase
AB1831		Conserved hypothetical protein
AB1832	*waaM*	Lipid A biosynthesis lauroyl acyltransferase
AB1833	*waaC*	Lipopolysaccharide heptosyltransferase I
AB1834	*gppA*	Guanosine pentaphosphate phosphohydrolase (gppA)
AB1835	*fdxB*	Ferredoxin
AB1836		Inositol monophosphatase family protein
AB1837	*gltD*	Glutamate synthase, small chain
AB1842		Methyl-accepting chemotaxis protein
AB1844	*gspD*	General secretion pathway protein D
AB1845		Conserved hypothetical protein
AB1846	*tlyA*	Hemolysin A
AB1847	*ribF*	Riboflavin kinase/FAD synthase RibF
AB1848		Methyltransferase, putative
AB1849	*bcp*	Bacterioferritin comigratory protein, alkyl hydroperoxide reductase/thiol specific antioxidant
AB1850		Hypothetical protein
AB1851	*glcD*	Glycolate oxidase
AB1852	*rbn*	tRNA-processing ribonuclease BN
AB1854	*murA*	UDP-N-acetylglucosamine 1-carboxyvinyltransferase
AB1855		Conserved hypothetical membrane protein (DUF6)
AB1856	*kdsA*	3-deoxy-D-manno-octulosonic acid 8-phosphate synthase
AB1857	*ribH*	Riboflavin synthase, beta subunit
AB1858	*nusB*	Transcription termination factor NusB
AB1859	*pyrF*	Orotidine 5'-phosphate decarboxylase
AB1860		ABC transporter, ATP-binding protein
AB1861		Hypothetical protein
AB1862	*feoA*	Ferrous iron transport protein A
AB1863	*feoB2*	Ferrous iron transport protein B
AB1864		Conserved hypothetical protein
AB1865		Conserved hypothetical protein
AB1866		Conserved hypothetical protein
AB1867		Conserved hypothetical protein
AB1868		Heavy metal-(Cd/Co/Hg/Pb/Zn)-translocating P-type ATPase
AB1869		Transcriptional regulator, ArsR family

AB1875	*cspA*	Cold-shock protein, DNA-binding
AB1877		Two-component response regulator
AB1878		Two-component sensor histidine kinase
AB1883	*ahpC*	Alkyl hydroperoxide reductase/ Thiol specific antioxidant
AB1885		Conserved hypothetical protein
AB1886		Hypothetical protein
AB1887	*rpoC*	DNA-directed RNA polymerase, beta' chain
AB1888	*rpoB*	DNA-directed RNA polymerase, beta chain
AB1889	*rplL*	50S ribosomal protein L7/L12
AB1890	*rplJ*	50S ribosomal protein L10
AB1891	*rplA*	50S ribosomal protein L1
AB1892	*rplK*	50S ribosomal protein L11
AB1893	*nusG*	Transcription termination factor NusG
AB1895	*rpmG*	50S ribosomal protein L33
AB1896	*tufA*	Elongation factor Tu
AB1897	*murD*	UDP-N-acetylmuramoylalanine--D-glutamate ligase
AB1898	*mraY*	Phospho-N-acetylmuramoyl-pentapeptide transferase
AB1899	*pgm*	Phosphoglycerate mutase, 2,3-bisphosphoglycerate-independent
AB1900		ABC transporter, ATP-binding protein
AB1901	*pbpA*	Penicillin-binding protein 1A
AB1902	*glnA*	Glutamine synthetase
AB1903	*hisJ*	Histidinol-phosphate phosphatase
AB1904	*moaE*	Molybdopterin converting factor, subunit 2
AB1905	*moaD*	Molybdopterin converting factor, subunit 1
AB1906		Conserved hypothetical protein (DUF178 domain protein)
AB1907	*uppP*	Undecaprenyl diphosphatase, putative
AB1908	*murG*	UDP-N-acetylglucosamine--N-acetylmuramyl-(pentapeptide) pyrophosphoryl-undecaprenol N-acetylglucosamine transferase
AB1909	*pbpB*	Penicillin-binding protein
AB1910	*ftsW*	Cell division protein FtsW
AB1911	*ppi*	Peptidyl-prolyl cis-trans isomerase
AB1912		Cytochrome c
AB1913	*clpA*	ATP-dependent Clp protease, ATP-binding subunit
AB1914	*clpS*	ATP-dependent Clp protease adaptor protein ClpS
AB1915	*bioD*	Dethiobiotin synthetase
AB1917		CitE domain protein
AB1918		AMP-dependent synthetase and ligase/CitE domain protein
AB1919		MaoC family protein, putative enoyl-CoA hydratase
AB1920		CitE domain protein
AB1921	*fumA*	Fumarate hydratase, class I
AB1922		Conserved hypothetical protein
AB1923		Methyl-accepting chemotaxis protein
AB1924	*arsB*	Arsenical pump membrane protein
AB1925	*arsR*	Transcriptional regulator, ArsR family
AB1926		Putative redox-active disulfide protein
AB1928	*arsC*	Arsenate reductase
AB1929		Conserved hypothetical protein
AB1930		Putative integral membrane protein
AB1931	*flhA*	Flagellar biosynthesis protein FlhA
AB1933	*fliI*	Flagellum-specific ATP synthase FliI
AB1934		Tetratricopeptide repeat domain protein
AB1935	*flhB*	Flagellar biosynthetic protein FlhB
AB1936	*fliR*	Flagellar biosynthetic protein FliR
AB1937		Hypothetical membrane protein
AB1938	*flgC*	Flagellar basal body rod protein FlgC

AB1939	*fliE*	Flagellar hook-basal body protein FliE
AB1940		Hypothetical protein
AB1941		ATP-binding protein
AB1942	*flhF*	Flagellar biosynthesis (GTP-binding) protein FlhF
AB1943		Hypothetical protein
AB1944		Hypothetical protein
AB1945		Hypothetical protein
AB1946	*fliY*	Flagellar motor switch protein FliY
AB1947	*flaG*	Polar flagellin
AB1948		TPR repeat protein
AB1949		Hypothetical protein
AB1953	*fliN*	Flagellar motor switch protein FliN
AB1954	*fliH*	Flagellar assembly protein FliH
AB1955	*fliG*	Flagellar motor switch protein FliG
AB1956	*fliF*	Flagellar M-ring protein FliF
AB1957	*flgB*	Flagellar basal body rod protein FlgB
AB1958	*flgG1*	Flagellar distal rod protein FlgG
AB1959		Conserved hypothetical protein
AB1960	*cheY2*	Chemotaxis protein CheY
AB1961	*flgG2*	Flagellar distal rod protein FlgG
AB1962		Hypothetical protein
AB1964	*rimM*	16S rRNA processing protein
AB1965		Conserved hypothetical protein
AB1966	*rpsP*	30S ribosomal protein S16
AB1967	*ffh*	Signal recognition particle protein
AB1968		Ribosomal large subunit pseudouridine synthase
AB1969	*kdtA*	3-deoxy-D-manno-octulosonic-acid transferase
AB1970		Conserved hypothetical protein (DUF164 domain protein)
AB1971		Conserved hypothetical protein
AB1972	*glyQ*	Glycyl-tRNA synthetase, alpha chain
AB1973		Glutaredoxin-like protein
AB1974	*purE*	Phosphoribosylaminoimidazole carboxylase, catalytic subunit
AB1975		Hypothetical protein
AB1976		Peptidase, U32 family
AB1977		Methyl-accepting chemotaxis protein
AB1978	*recN*	DNA repair protein RecN
AB1979		NAD(+) kinase
AB1980	*fus*	Translational elongation factor G
AB1982	*rpsL*	30S ribosomal protein S12
AB1983		Alpha/beta hydrolase
AB1984	*araJ*	Putative transport protein AraJ
AB1985		TonB-dependent receptor protein
AB1986		Putative sulfonate/nitrate transport system substrate-binding protein
AB1987		Nitrite/nitric oxide reductase-related protein NnrS
AB1988		ABC transporter, ATP-binding protein
AB1989		ABC transporter, permease protein
AB1990		Conserved hypothetical protein
AB1991		Conserved hypothetical membrane protein
AB1992	*trpG*	Anthranilate synthase, component II
AB1993	*fbpA*	Putative iron-uptake ABC transporter, periplasmic iron-binding protein
AB1994	*fbpB*	Putative iron-uptake ABC transporter, permease protein
AB1996		ABC transporter, ATP-binding protein
AB1997		Transcriptional regulator, AraC family
AB2000		Conserved hypothetical protein
AB2001		Conserved hypothetical protein

AB2002	*nirA*	Ferredoxin-nitrite reductase
AB2003		Conserved hypothetical protein
AB2004		Hypothetical protein
AB2005		Conserved hypothetical protein
AB2006		S1 RNA binding domain protein
AB2007		Conserved hypothetical protein
AB2008		Conserved hypothetical protein
AB2009		MutT/nudix family protein
AB2010		Transcriptional regulator, AraC family
AB2011		Aminotransferase, classes I and II
AB2014	*rplS*	50S ribosomal protein L19
AB2015	*trmD*	tRNA (guanine-N1)-methyltransferase
AB2016		Hypothetical protein
AB2017	*metE*	5-methyltetrahydropteroyltriglutamate--homocysteine methyltransferase
AB2018	*ilvA*	Threonine deaminase
AB2019	*atpB*	ATP synthase F0 sector, A subunit
AB2023		Conserved hypothetical membrane protein (DUF205 domain protein)
AB2024	*nadA*	Quinolinate synthetase A protein
AB2025	*nadC*	Nicotinate-nucleotide pyrophosphorylase
AB2026		Exopolyphosphatase-related protein
AB2027		Peptidase, M23/M37 family
AB2028	*lpxC*	UDP-3-O-acyl-N-acetylglucosamine deacetylase
AB2029		Conserved hypothetical protein
AB2030	*thrB*	Homoserine kinase
AB2031		Hypothetical protein (DUF448 domain protein)
AB2032	*infB*	Translation initiation factor IF-2
AB2033	*rbfA*	Ribosome binding factor A
AB2034		Conserved hypothetical protein (DUF150 domain protein)
AB2035	*ribD*	Riboflavin biosynthesis protein RibD
AB2036	*efp*	Translation elongation factor EF-P
AB2037	*serA*	D-3-phosphoglycerate dehydrogenase
AB2038	*rpsA*	30S ribosomal protein S1
AB2039	*ispH*	4-hydroxy-3-methylbut-2-enyl diphosphate reductase
AB2040	*aroA*	3-phosphoshikimate 1-carboxyvinyltransferase
AB2041	*pheT*	Phenylalanyl-tRNA synthetase, beta subunit
AB2042	*pheS*	Phenylalanyl-tRNA synthetase, alpha subunit
AB2043		HIT family protein
AB2044	*accA*	Acetyl-CoA carboxylase, carboxyltransferase, alpha subunit
AB2045	*fabF*	Beta ketoacyl-(acyl carrier protein) synthase II
AB2046	*acpP*	Acyl carrier protein, putative
AB2047	*fabG*	3-oxoacyl-(acyl carrier protein) reductase
AB2048		Radical SAM domain protein
AB2049		Conserved hypothetical protein
AB2050	*exsB*	Transcriptional regulator, ExsB family
AB2051		Conserved hypothetical protein
AB2052		Conserved hypothetical protein (DUF558 domain protein)
AB2053	*petC*	Ubiquinol cytochrome c oxidoreductase, cytochrome c1 subunit
AB2054	*petB*	Ubiquinol cytochrome c oxidoreductase, cytochrome b subunit
AB2055	*petA*	Ubiquinol cytochrome c oxidoreductase, 2Fe-2S subunit
AB2056	*thrC*	Threonine synthase
AB2057	*argB*	Acetylglutamate kinase
AB2058		ATP-dependent DNA helicase, UvrD/REP family
AB2059		Hypothetical protein
AB2060	*prfB*	Peptide chain release factor 2
AB2061		Conserved hypothetical protein

AB2062		Conserved hypothetical protein
AB2063		Hypothetical protein
AB2064		Conserved hypothetical protein
AB2066		Hypothetical protein
AB2067	*ccoP*	Cytochrome c oxidase, cbb3-type, subunit III
AB2068	*ccoQ*	Cytochrome c oxidase, cbb3-type, subunit IV
AB2069	*ccoO*	Cytochrome c oxidase, cbb3-type, subunit II
AB2070	*ccoN*	Cytochrome c oxidase, cbb3-type, subunit I
AB2071	*smpB*	tmRNA-binding protein SmpB
AB2072	*ispE*	4-diphosphocytidyl-2C-methyl-D-erythritol kinase
AB2073	*truB*	tRNA pseudouridine synthase B
AB2074		ATP-dependent DNA helicase, UvrD/PcrA family
AB2075		Transcriptional regulator, LysR family
AB2076		Conserved hypothetical protein, radical SAM domain protein
AB2077	*purF*	Amidophosphoribosyltransferase
AB2078	*dapB*	Dihydrodipicolinate reductase
AB2079	*trxB*	Thioredoxin reductase
AB2080	*trxA2*	Thioredoxin
AB2081	*alaS*	Alanyl-tRNA synthetase
AB2082		Conserved hypothetical protein (DUF541 domain protein)
AB2083		Major facilitator superfamily transporter
AB2085		Conserved hypothetical protein
AB2086		Two-component sensor histidine kinase
AB2087		Two-component response regulator
AB2088	*htrA*	Periplasmic serine protease DO; heat shock protein HtrA
AB2089	*ilvD*	Dihydroxyacid dehydratase
AB2098		Hypothetical protein
AB2099		Conserved hypothetical protein
AB2100		ABC transporter, ATP-binding protein
AB2103	*dksA*	DnaK suppressor protein DksA
AB2104		tRNA pseudouridine synthase
AB2105		Conserved hypothetical protein
AB2106		Conserved hypothetical protein
AB2107	*ctpA*	Carboxyl-terminal protease family protein
AB2108	*purC*	Phosphoribosylaminoimidazole-succinocarboxamide synthase
AB2109	*purS*	Phosphoribosylformylglycinamidine synthetase
AB2110	*purQ*	Phosphoribosylformylglycinamidine synthase I
AB2111		Conserved hypothetical protein
AB2112	*plsC*	1-acyl-sn-glycerol-3-phosphate acyltransferase PlsC
AB2113	*crcB*	Camphor resistance CrcB protein
AB2114		Twin-arginine translocation protein, TatA/E family
AB2115		Twin-arginine translocation protein, TatA/E family
AB2116	*argS*	Arginyl-tRNA synthetase
AB2117		Conserved hypothetical protein (DUF143 domain protein)
AB2118	*nadD*	Nicotinate (nicotinamide) nucleotide adenylyltransferase
AB2119	*gapA*	Glyceraldehyde 3-phosphate dehydrogenase A
AB2120	*pgk*	Phosphoglycerate kinase
AB2121	*tpiA*	Triosephosphate isomerase
AB2122	*fabI*	Enoyl-(acyl carrier protein) reductase
AB2123	*lysA*	Diaminopimelate decarboxylase
AB2124	*pheA*	Chorismate mutase\prephenate dehydratase
AB2125	*hisC*	Histidinol-phosphate aminotransferase
AB2126	*dxs*	1-deoxy-D-xylulose-5-phosphate synthase
AB2127		Cytochrome c551 peroxidase
AB2128		Septum formation protein Maf homolog

AB2129	*panE*	Ketopantoate reductase
AB2130		Conserved hypothetical protein
AB2131		Probable lipoprotein nlpC homolog precursor
AB2132		Putative transcriptional regulator, Crp/Fnr family
AB2133		Conserved hypothetical protein
AB2134	*ung*	Uracil-DNA glycosylase
AB2135		Aldehyde dehydrogenase family protein
AB2136	*ilvB*	Acetolactate synthase
AB2137	*lemA*	LemA protein
AB2138		Conserved hypothetical protein
AB2139		Conserved hypothetical membrane protein (DUF477)
AB2140	*gph*	Phosphoglycolate phosphatase
AB2141		DNA-binding ferritin-like protein (Dps/NapA)
AB2144		Major facilitator superfamily transporter
AB2145		Hypothetical protein
AB2146		GGDEF/HAMP domain protein
AB2147		Multidrug efflux protein, HlyD family
AB2148		Multidrug efflux protein, AcrB/AcrD/AcrF family
AB2149		RND efflux system, outer membrane lipoprotein
AB2151		Sigma factor regulatory protein, FecR/PupR family
AB2152		Sigma factor, ECF family
AB2155		Conserved hypothetical protein
AB2156	*metY*	O-acetylhomoserine sulfhydrylase
AB2157	*iscR*	Transcriptional regulator, BadM/Rrf2 family
AB2158	*cysK1*	Cysteine synthase
AB2159		Conserved hypothetical protein
AB2160	*cysH*	Adenosine phosphosulfate (APS) reductase
AB2161	*cysD*	ATP sulfurylase, small subunit
AB2162	*cysN*	ATP sulfurylase, large subunit
AB2163	*cysI*	Sulfite reductase, iron-sulfur subunit
AB2164		Hypothetical protein
AB2165		Aminotransferase, NifS-like protein
AB2167		Hypothetical protein
AB2168	*cobS*	Cobalamin (Vitamin B12) synthase
AB2169	*cobP*	Cobinamide kinase / Cobinamide phosphate guanylyltransferase
AB2170		Lipolytic enzyme, GDSL domain
AB2171		Conserved hypothetical protein
AB2172		Sodium:sulfate symporter family protein
AB2173		Conserved hypothetical protein (DUF81 domain protein)
AB2174	*dsbD*	Thiol:disulfide interchange protein DsbD
AB2175	*rimK*	Ribosomal protein S6 modification protein
AB2176		Conserved hypothetical protein (DUF785 domain protein)
AB2177		Conserved hypothetical protein
AB2178		EAL/GGDEF domain protein
AB2179	*psd*	Phosphatidylserine decarboxylase
AB2180		Hypothetical protein
AB2181	*mltA*	Peptidoglycan N-acetylmuramoylhydrolase
AB2182	*dnaK*	DnaK-type molecular chaperone
AB2183	*grpE*	Heat shock protein GrpE
AB2184	*hrcA*	Putative heat shock regulator
AB2185		Conserved hypothetical protein
AB2186	*truA*	tRNA pseudouridine synthase A
AB2191	*glmU*	UDP-N-acetylglucosamine pyrophosphorylase
AB2192		Ankyrin repeat protein
AB2193		Probable ATP-dependent RNA helicase

AB2194	*trmA*	tRNA (uracil-5-)-methyltransferase
AB2195		Hypothetical protein
AB2199	*gidA*	Glucose inhibited division protein A
AB2200	*ribE*	Riboflavin synthase, alpha subunit
AB2201	*mreC*	Rod shape-determining protein MreC
AB2202	*mreB*	Rod shape-determining protein MreB
AB2203	*clpX*	ATP-dependent Clp protease, ATP-binding subunit ClpX
AB2204	*lpxA*	UDP-N-acetylglucosamine acyltransferase
AB2205	*fabZ*	3-hydroxymyristoyl-(acyl carrier protein) dehydratase
AB2206	*lpxB*	Lipid A disaccharide synthase
AB2207		Conserved hypothetical protein (DUF208 domain protein)
AB2208		Hypothetical protein
AB2209	*ndh*	NADH dehydrogenase
AB2212		Multidrug efflux protein, Acr family
AB2213		Multidrug efflux protein, HlyD family
AB2214		Transcriptional regulator, TetR family
AB2215		Major facilitator superfamily transporter, Bcr/CflA subfamily
AB2216		Sulfate permease family protein
AB2217		Hypothetical protein
AB2218		Hypothetical protein
AB2219		Hypothetical protein
AB2223	*murB*	UDP-N-acetylenolpyruvoylglucosamine reductase
AB2224	*topA*	DNA topoisomerase I
AB2225		Conserved hypothetical protein
AB2226	*bioB*	Biotin synthetase
AB2227		Conserved hypothetical protein
AB2228		Hypothetical protein
AB2229	*eno*	Enolase
AB2230	*recA*	DNA-dependent ATPase, RecA
AB2231		Hypothetical protein
AB2232	*pseB*	UDP GlcNAc dehydratase/reductase PseB, putative
AB2233		DegT/DnrJ/EryC1/StrS aminotransferase
AB2234	*neuA*	Acylneuraminate cytidylyltransferase
AB2245		4Fe-4S ferredoxin, iron-sulfur binding
AB2246	*pyrG*	CTP synthetase
AB2247	*recJ*	Single-stranded DNA-specific exonuclease
AB2248		Hypothetical protein
AB2249		Conserved hypothetical protein
AB2250	*thiJ*	4-methyl-5(beta-hydroxyethyl)-thiazole monophosphate synthesis protein ThiJ
AB2251	*dnaE*	DNA polymerase III, alpha subunit
AB2252	*surE*	Stationary-phase survival protein SurE
AB2253		Hypothetical protein
AB2254		Conserved hypothetical protein
AB2255	*moaC*	Molybdenum cofactor biosynthesis protein C
AB2256	*rpsU*	30S ribosomal protein S21
AB2257		Conserved hypothetical integral membrane protein
AB2258		Conserved hypothetical protein
AB2259		Conserved hypothetical protein, putative methyltransferase
AB2260	*secG*	Protein-export membrane protein SecG
AB2261	*frr*	Ribosome releasing factor
AB2262	*pyrE*	Orotate phosphoribosyltransferase
AB2263		Conserved hypothetical protein, RDD family
AB2264		Putative major facilitator superfamily transporter
AB2265		CorA-like Mg2+ transporter protein
AB2266		Conserved hypothetical protein (DUF328 domain protein)

AB2270		Metallophosphoesterase
AB2275		Conserved hypothetical protein
AB2276		Methyl-accepting chemotaxis protein
AB2277	*moeB*	Molybdopterin biosynthesis protein
AB2281	*cysA*	Sulfate ABC transporter (ATP-binding protein)
AB2282	*cysW*	Sulfate transport system permease protein
AB2283	*cysT*	Sulfate transport system permease protein
AB2284	*cysK2*	Cysteine synthase
AB2285		EAL domain protein
AB2286		Conserved hypothetical protein
AB2287	*sbp*	Sulfate-binding protein precursor
AB2288		Transcriptional regulator, BadM/Rrf2 family
AB2293	*typA*	GTP-binding elongation factor family protein
AB2294		Conserved hypothetical protein, putative transport system permease protein
AB2295		Conserved hypothetical protein, SirA-like protein
AB2302		GGDEF/PAS domain protein
AB2303		Sulfatase
AB2305		Cytochrome c-type protein, putative
AB2306		Diheme cytochrome c precursor, putative
AB2307		Cytochrome b, putative
AB2308		Hypothetical protein
AB2310		Two-component response regulator
AB2311		Two-component sensor histidine kinase
AB2312		Methyltransferase
AB2313		Transcriptional regulator, AraC family
AB2314		HD_GYP domain response regulator
AB2315	*thyX*	Thymidylate synthase ThyX
AB2317		Conserved hypothetical membrane protein
AB2319	*purN*	Phosphoribosylglycinamide formyltransferase
AB2320	*ruvC*	Crossover junction endodeoxyribonuclease RuvC
