# Supplementary material for: The Complete Genome Sequence and Analysis of the Epsilonproteobacterium Arcobacter butzleri
Source: PLoS One. 2007 Dec 26;2(12):e1358. doi: 10.1371/journal.pone.0001358 (PMC2147049; doi:10.1371/journal.pone.0001358)
Supplement: Table S7 — CDS present only in A. butzleri strain RM4018. Set of 42 genes identified by comparative genomic indexing of 13 A. butzleri strains to be present only in strain RM4018. The functional annotation of each gene is provided. (0.02 MB PDF) [file pone.0001358.s009.pdf]

Table S7. CDS present only in *A. butzleri* strain RM4018

Gene	Common	Description
AB0104		Conserved hypothetical protein, putative tricarboxylic transport protein TctA
AB0198	*flgI*	Flagellar P-ring protein FlgI
AB0563	*soxC*	Sulfur oxidation protein SoxCD, sulfur dehydrogenase subunit
AB0564	*soxD*	Sulfur oxidation protein SoxCD, diheme cytochrome c subunit
AB0565	*soxX*	Sulfur oxidation protein SoxXA, monoheme cytochrome c subunit
AB0566	*soxY*	Sulfur oxidation protein SoxYZ, sulfur covalently binding protein
AB0567	*soxZ*	Sulfur oxidation protein SoxYZ, sulfur compound chelating protein
AB0568	*soxA*	Sulfur oxidation protein SoxXA, diheme cytochrome c subunit
AB0569		Hypothetical protein
AB0570	*soxB*	Sulfur oxidation protein, sulfate thiol esterase
AB0571		Hypothetical protein
AB0576	*pfpI*	Peptidase, ThiJ/PfpI family protein
AB0578		Conserved hypothetical protein, beta-lactamase-like protein
AB0941		Adhesin/haemagglutinin, HecA family
AB0942		Hypothetical protein
AB0944		Hypothetical protein
AB0945		Hypothetical protein
AB0946		Hypothetical protein
AB1214		Conserved hypothetical protein
AB1292		Sulfate permease
AB1332		Hypothetical protein
AB1335		Conserved hypothetical protein
AB1337		DEAD/DEAH box helicase domain protein
AB1338		Hypothetical protein
AB1340		Hypothetical protein
AB1342		Hypothetical protein
AB1344		Hypothetical protein
AB1345		Hypothetical protein
AB1346		Hypothetical protein
AB1347		Hypothetical protein
AB1348		Hypothetical protein
AB1349		Conserved hypothetical protein
AB1350		Hypothetical protein
AB1352		Hypothetical protein
AB1357		Hypothetical protein
AB1665		Phage major tail tube protein
AB1688		Hypothetical protein
AB1724		Conserved hypothetical protein
AB1729		Hypothetical protein
AB1730	*hsdS*	Type I restriction-modification system specificity determinant
AB1731	*hsdM*	Type I restriction-modification system, M subunit, putative
AB2272		TonB-dependent receptor protein
